# Supplementary material for: Single-nucleotide m⁶A mapping uncovers redundant YTHDF function in planarian progenitor fate selection
Source: EMBO J. 2026 Jan 3;45(3):749–88. doi: 10.1038/s44318-025-00662-3 (PMC12864844; doi:10.1038/s44318-025-00662-3)
Supplement: Supplementary file 1 — Appendix [file 44318_2025_662_MOESM1_ESM.pdf]

Appendix

Single-nucleotide m⁶A mapping uncovers redundant YTHDF function in planarian progenitor fate selection

Yarden Yesharim[1], Ophir Shwarzbard[1], Jenny Barboy-Smoliarenko[1], Prakash Varkey Cherian[1], Ran Shachar[2], Amrutha Palavalli[3], Hanh Thi-Kim Vu[3], Schraga Schwartz[2], Omri Wurtzel[1,4]

[1] The School of Neurobiology, Biochemistry & Biophysics, George S. Wise Faculty of Life Sciences, Tel Aviv University, Tel Aviv, Israel

[2] Department of Molecular Genetics, Weizmann Institute of Science, Rehovot, Israel

[3] European Molecular Biology Laboratory, Developmental Biology Unit, Meyerhofstraße 1, 69117 Heidelberg, Germany

[4] Sagol School of Neuroscience, Tel Aviv University, Tel Aviv, Israel

* Correspondence: owurtzel@tauex.tau.ac.il

Table of contents

Appendix Table S1. Primer sequences of genes cloned in this study.................................... 3

Appendix Table S2. qPCR primers used in this study... 4

Appendix Table S3. GLORI adapter sequences used in this study.. 5

Appendix Table S1. Primer sequences of genes cloned in this study

Gene	Contig	Forward primer	Reverse primer
ythdf-A	dd_Smed_v6_5578_0_1	TCGACGGATTTTAATTTATCGCA	AAGGCCTCCGCTTCATGA
ythdf-B	dd_Smed_v6_7162_0_1	TCCATTTGCACCAAATCAAA	ATGATGCCAATGATGTCGAA
ythdf-C	dd_Smed_v6_7891_0_1	TTTTCAATCATCCCCGAATC	CAGCCGGGATAAATTAACGA
ythdf-D	dd_Smed_v6_8450_0_1	AAGATCAGCCCAATGAATCG	ATAGCCGGAACCATTGACAG
ythdf-E	dd_Smed_v6_5316_0_1	TTGGCAACCTTTATGCTTCC	GTGGGTGGCTTGATTTCAGT
kiaa1429	dd_Smed_v6_4676_0_1	CCGCTATCCGTTGTTATTATGCG	TCCGTAATCGTGGCCAGC
ythdc-1	dd_Smed_v6_3491_0_1	CAGTCATCTCCCAATGTTGACG	ACAAACCGCAATAATTGTAACCA
dd_518	dd_Smed_v6_518_0_1	TTTTCAGAAGGAGCGACGAC	GTGACAAGAGAGCAGCACCA
dd_75	dd_Smed_v4_75_0_1	TGCCGTTATGAACATGATTTTCG	ACACAAAATATCGCATCCTGCC
dd_626	dd_Smed_v6_626_0_1	CAAATTGGAGACACGGCTTT	GATTTTATCGCCTCCGTTGA
dd_3451	dd_Smed_v6_3451_0_1	TGGAAAGGGAAGAATGGTTG	GGAAATCATCAGCAGGGAAA
dd_924	dd_Smed_v6_924_0_1	AGCAACGAACATGCAACAAA	CCGGAAGCCTCATTATACCA
dd_356	dd_Smed_v6_356_0_1	TCCAGTCCTCCTGCTTCCTA	CCTCCTCCGAAAACAGAGTG
dd_1837	dd_Smed_v6_1837_0_1	TTGGAACAGACCACTGGTGA	AACGACGACCTTTCCAACTG
dd_940	dd_Smed_v6_940_0_1	CAAAGTGCTCAAGGCGATCT	TGGGAAGTGTCACAGTTGGA

Appendix Table S2. qPCR primers used in this study

Gene	Contig	Forward primer	Reverse primer
ythdf-A	dd_Smed_v6_5578_0_1	TGCCTTCAGATCACGACAAC	CCGAGGTTTGACAACCCATA
ythdf-B	dd_Smed_v6_7162_0_1	CGGATTTGCGACTGGATATT	GCATAAGTGGCCTTCACGTT
ythdf-C	dd_Smed_v6_7891_0_1	AACAGGCGTCTCGATAATGC	GTCCTTTCCACTTGCCTTGA
gapdh	dd_smed_v6_78_0_ 1	TCTTCCCCAACCAATTTTCTGTTCTG	CCGAATATTTTATTTGGCTCTTCCTCCA

Appendix Table S3. GLORI adapter sequences used in this study

Ligated adapter ID	Complete 3’ adapter GLORI sequence	Barcode	Barcode reverse complement
1	/5Phos/rBrBrBrBrGrUrCrGrCrUr(m6A)rG r(m6A)rUrC rGrGr(m6A) r(m6A)rGr(m6A) rGrCr(m6A) rCr(m6A)rC rGrUrC rU/3SpC3/ 3SpC3/	GTCGCT	AGCGAC
2	/5Phos/rBrBrBrBrCrGrGrUrGrGr(m6A)rG r(m6A)rUrC rGrGr(m6A) r(m6A)rGr(m6A) rGrCr(m6A) rCr(m6A)rC rGrUrC rU/3SpC3/ 3SpC3/	CGGTGG	CCACCG
3	/5Phos/rBrBrBrBrUrUrGrGrCrCr(m6A)rG r(m6A)rUrC rGrGr(m6A) r(m6A)rGr(m6A) rGrCr(m6A) rCr(m6A)rC rGrUrC rU/3SpC3/ 3SpC3/	TTGGCC	GGCCAA
4	/5Phos/rBrBrBrBrUrUrCrGrUrGr(m6A)rG r(m6A)rUrC rGrGr(m6A) r(m6A)rGr(m6A) rGrCr(m6A) rCr(m6A)rC rGrUrC rU/3SpC3/ 3SpC3/	TTCGTG	CACGAA
5	/5Phos/rBrBrBrBrUrGrGrUrCrUr(m6A)rG r(m6A)rUrC rGrGr(m6A) r(m6A)rGr(m6A) rGrCr(m6A) rCr(m6A)rC rGrUrC rU/3SpC3/ 3SpC3/	TGGTCT	AGACCA
6	/5Phos/rBrBrBrBrCrUrUrGrUrCr(m6A)rG r(m6A)rUrC rGrGr(m6A) r(m6A)rGr(m6A) rGrCr(m6A) rCr(m6A)rC rGrUrC rU/3SpC3/ 3SpC3/	CTTGTC	GACAAG
