# Supplementary figures and images for: Single-nucleotide m⁶A mapping uncovers redundant YTHDF function in planarian progenitor fate selection (part 2 of 6)
Source: EMBO J. 2026 Jan 3;45(3):749–88. doi: 10.1038/s44318-025-00662-3 (PMC12864844; doi:10.1038/s44318-025-00662-3)

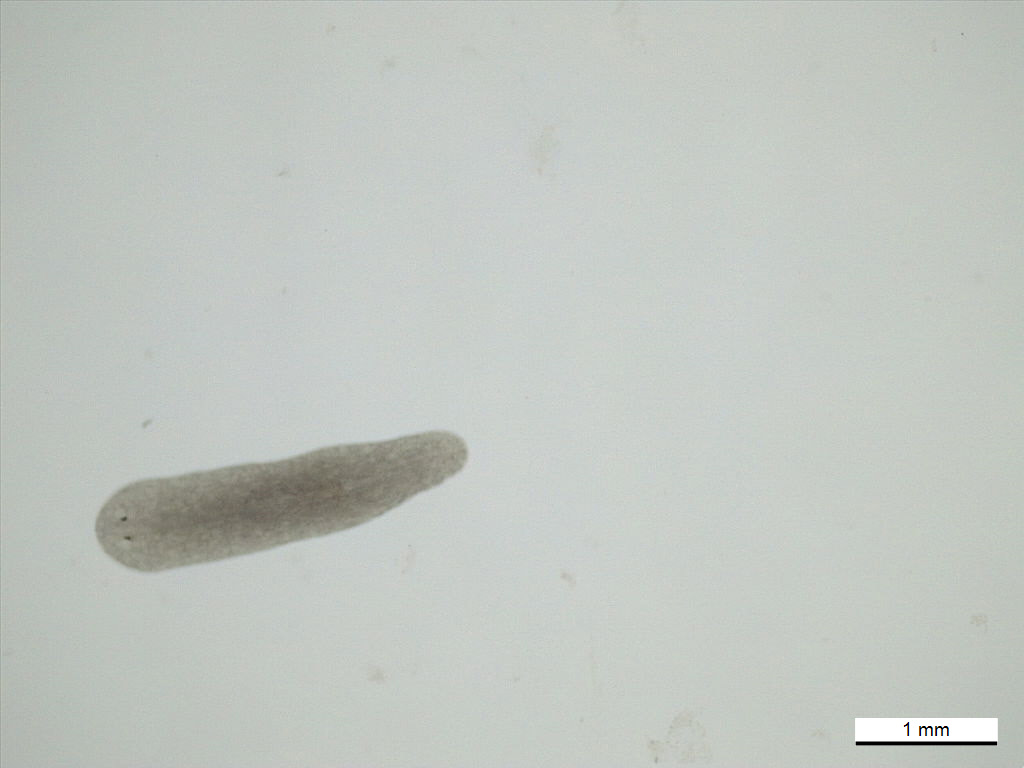

Supplement: Supplementary file 10 — Source data Fig. 3 [file 44318_2025_662_MOESM10_ESM.zip › Figure 3/3C-D/ythdf-a-b_RNAi_After_10_RNAi_feedings/ythdf_a-b_RNAi_After_10_RNAi_feedings_11.jpg]

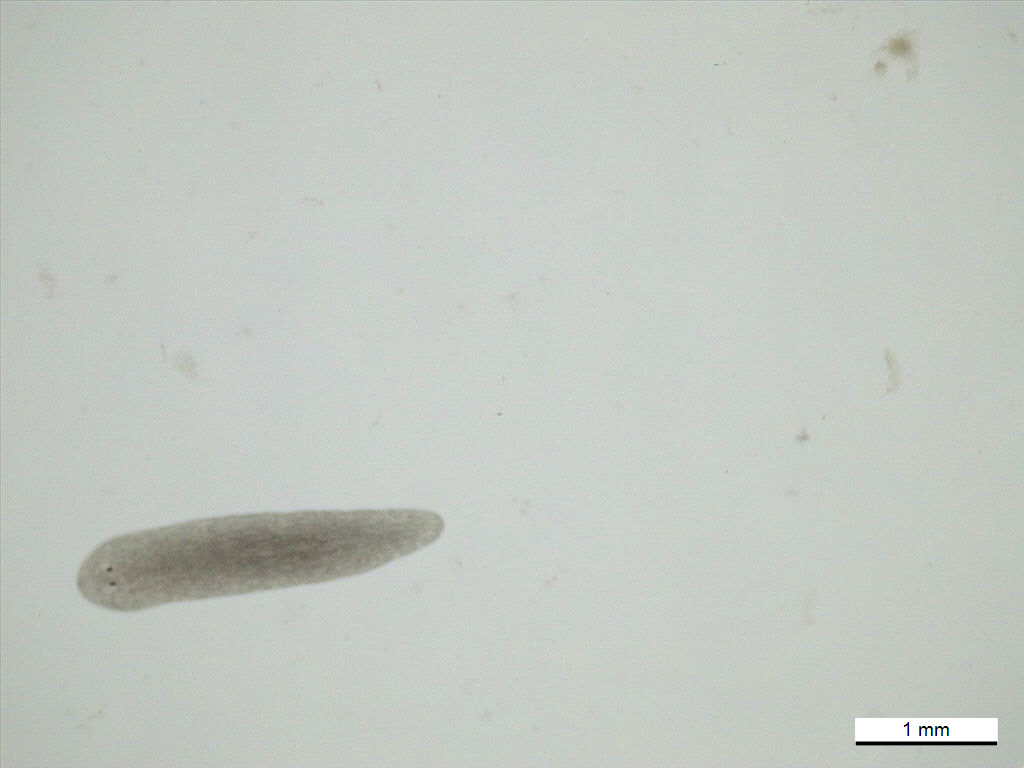

Supplement: Supplementary file 10 — Source data Fig. 3 [file 44318_2025_662_MOESM10_ESM.zip › Figure 3/3C-D/ythdf-a-b_RNAi_After_10_RNAi_feedings/ythdf_a-b_RNAi_After_10_RNAi_feedings_12.jpg]

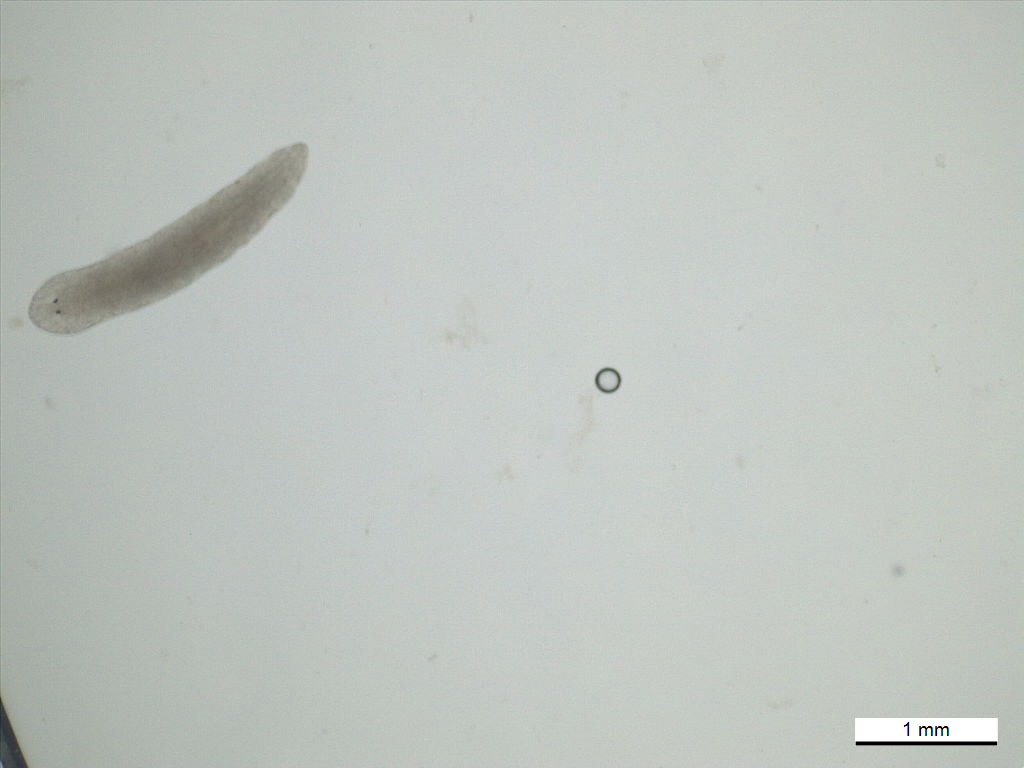

Supplement: Supplementary file 10 — Source data Fig. 3 [file 44318_2025_662_MOESM10_ESM.zip › Figure 3/3C-D/ythdf-a-b_RNAi_After_10_RNAi_feedings/ythdf_a-b_RNAi_After_10_RNAi_feedings_13.jpg]

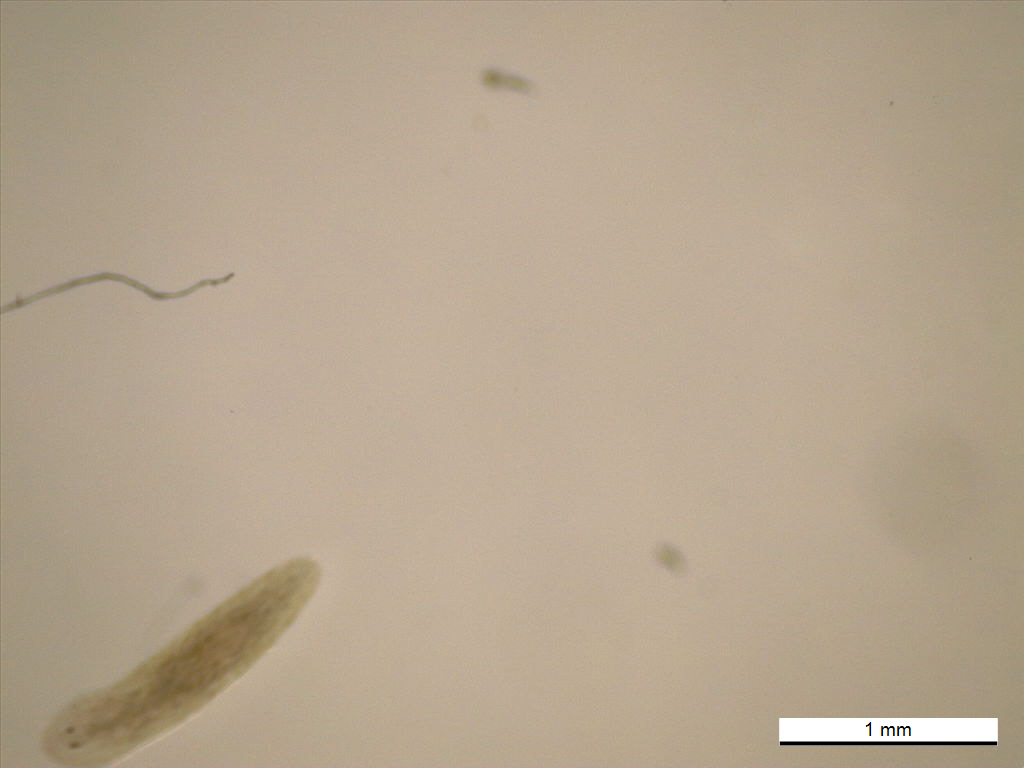

Supplement: Supplementary file 10 — Source data Fig. 3 [file 44318_2025_662_MOESM10_ESM.zip › Figure 3/3C-D/ythdf-a-b_RNAi_Before_RNAi_feedings/ythdf-a-b_RNAi_Before_RNAi_feedings_1.jpg]

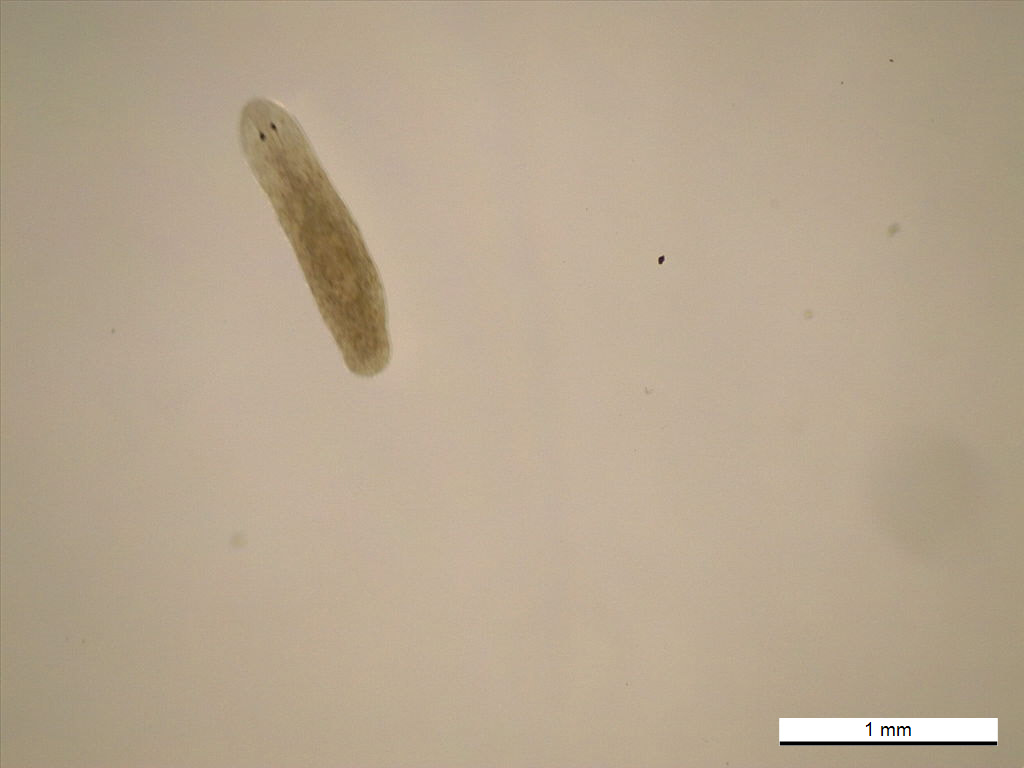

Supplement: Supplementary file 10 — Source data Fig. 3 [file 44318_2025_662_MOESM10_ESM.zip › Figure 3/3C-D/ythdf-a-b_RNAi_Before_RNAi_feedings/ythdf-a-b_RNAi_Before_RNAi_feedings_10.jpg]

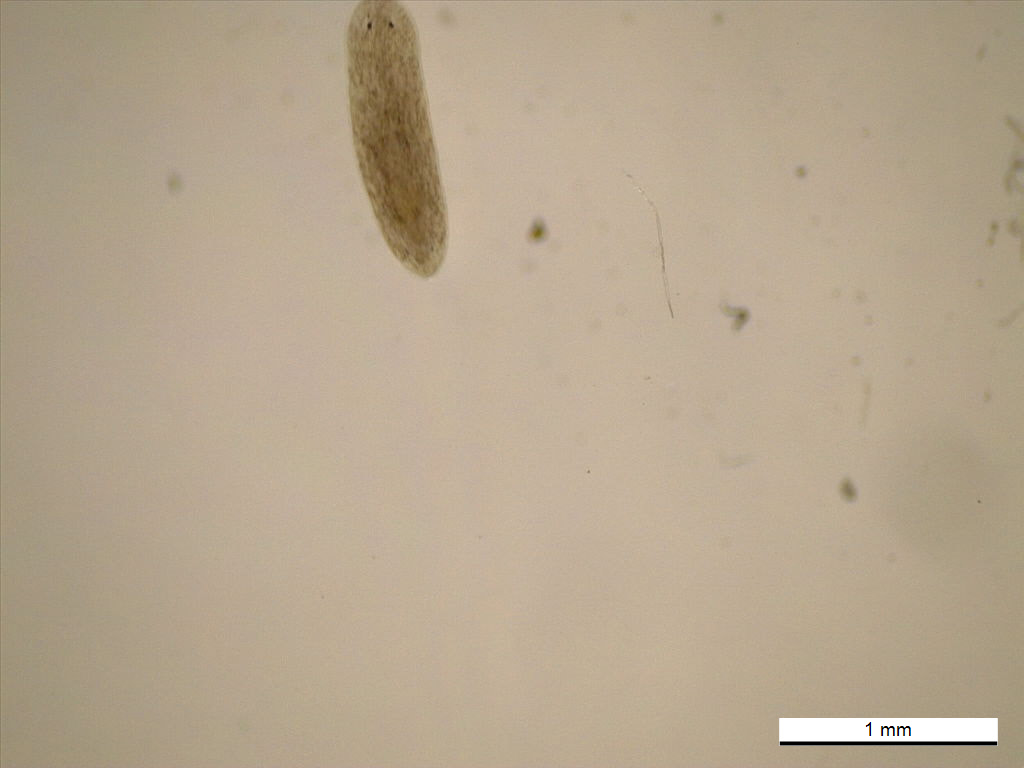

Supplement: Supplementary file 10 — Source data Fig. 3 [file 44318_2025_662_MOESM10_ESM.zip › Figure 3/3C-D/ythdf-a-b_RNAi_Before_RNAi_feedings/ythdf-a-b_RNAi_Before_RNAi_feedings_11.jpg]

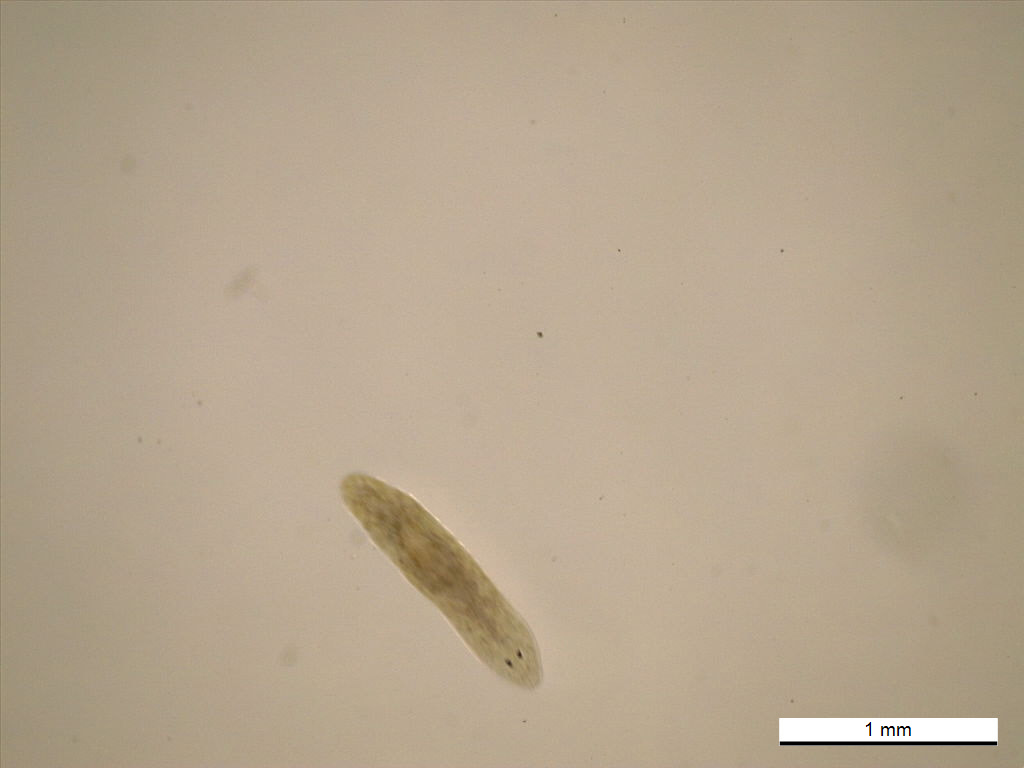

Supplement: Supplementary file 10 — Source data Fig. 3 [file 44318_2025_662_MOESM10_ESM.zip › Figure 3/3C-D/ythdf-a-b_RNAi_Before_RNAi_feedings/ythdf-a-b_RNAi_Before_RNAi_feedings_12.jpg]

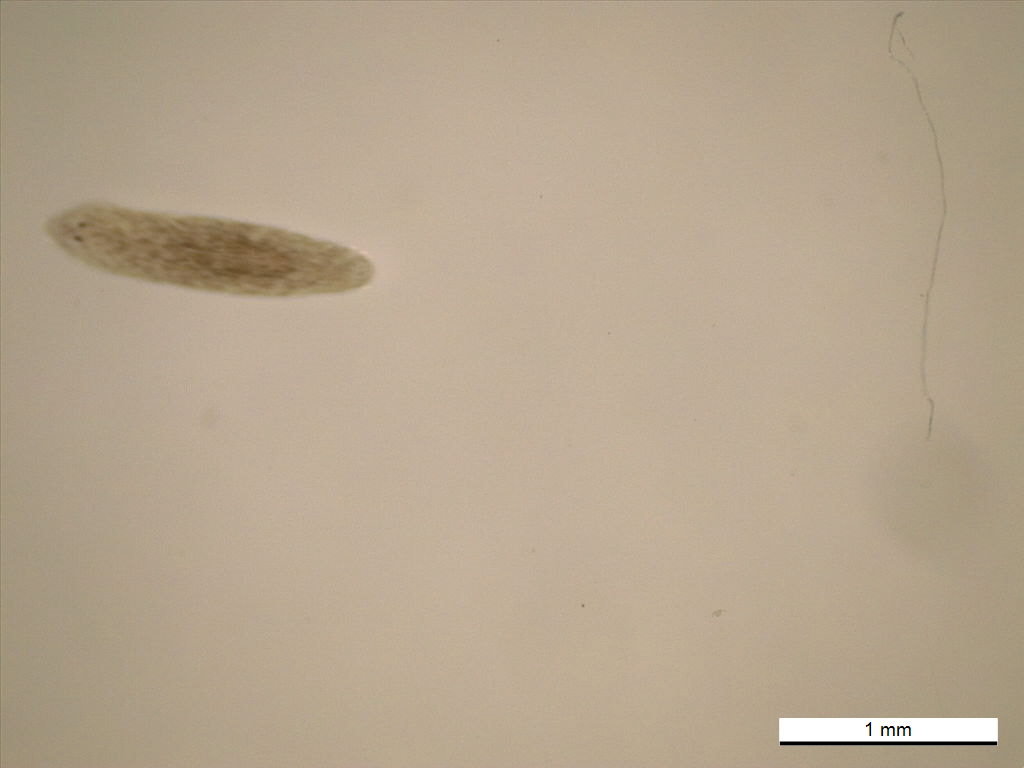

Supplement: Supplementary file 10 — Source data Fig. 3 [file 44318_2025_662_MOESM10_ESM.zip › Figure 3/3C-D/ythdf-a-b_RNAi_Before_RNAi_feedings/ythdf-a-b_RNAi_Before_RNAi_feedings_13.jpg]

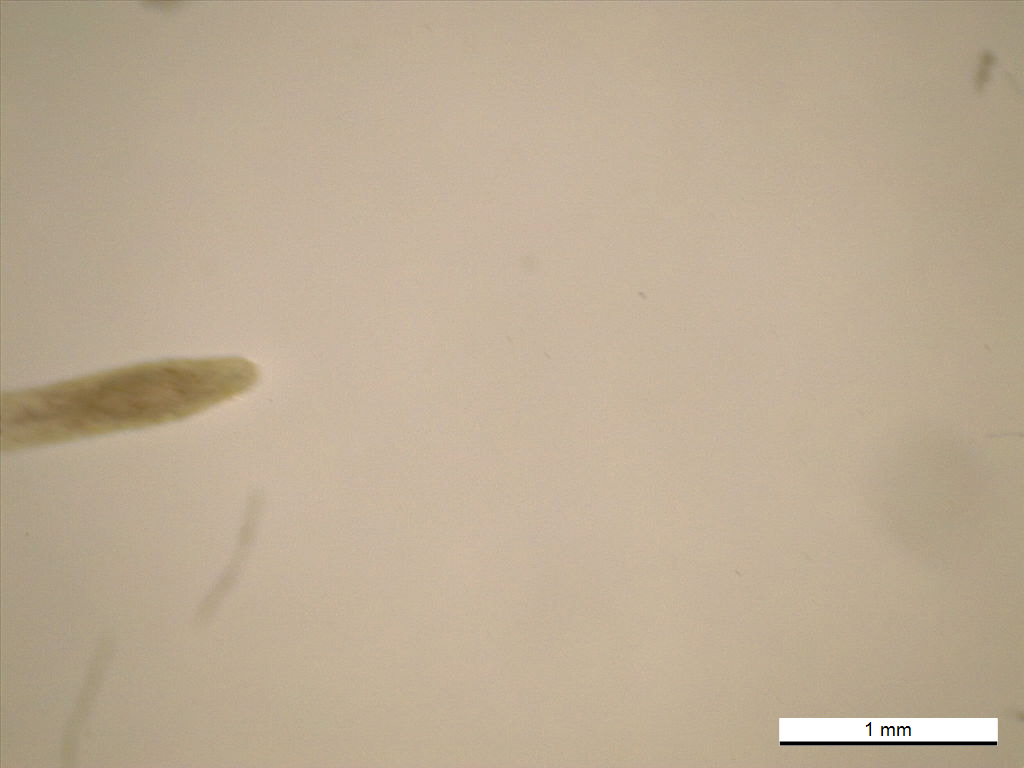

Supplement: Supplementary file 10 — Source data Fig. 3 [file 44318_2025_662_MOESM10_ESM.zip › Figure 3/3C-D/ythdf-a-b_RNAi_Before_RNAi_feedings/ythdf-a-b_RNAi_Before_RNAi_feedings_14.jpg]

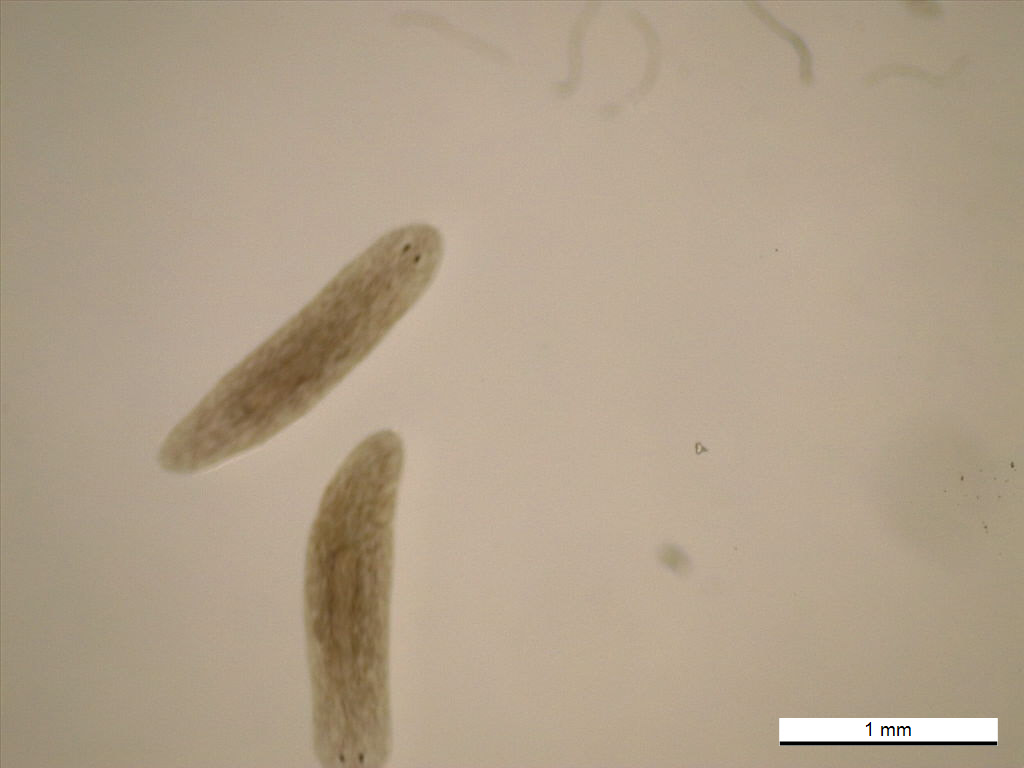

Supplement: Supplementary file 10 — Source data Fig. 3 [file 44318_2025_662_MOESM10_ESM.zip › Figure 3/3C-D/ythdf-a-b_RNAi_Before_RNAi_feedings/ythdf-a-b_RNAi_Before_RNAi_feedings_2.jpg]

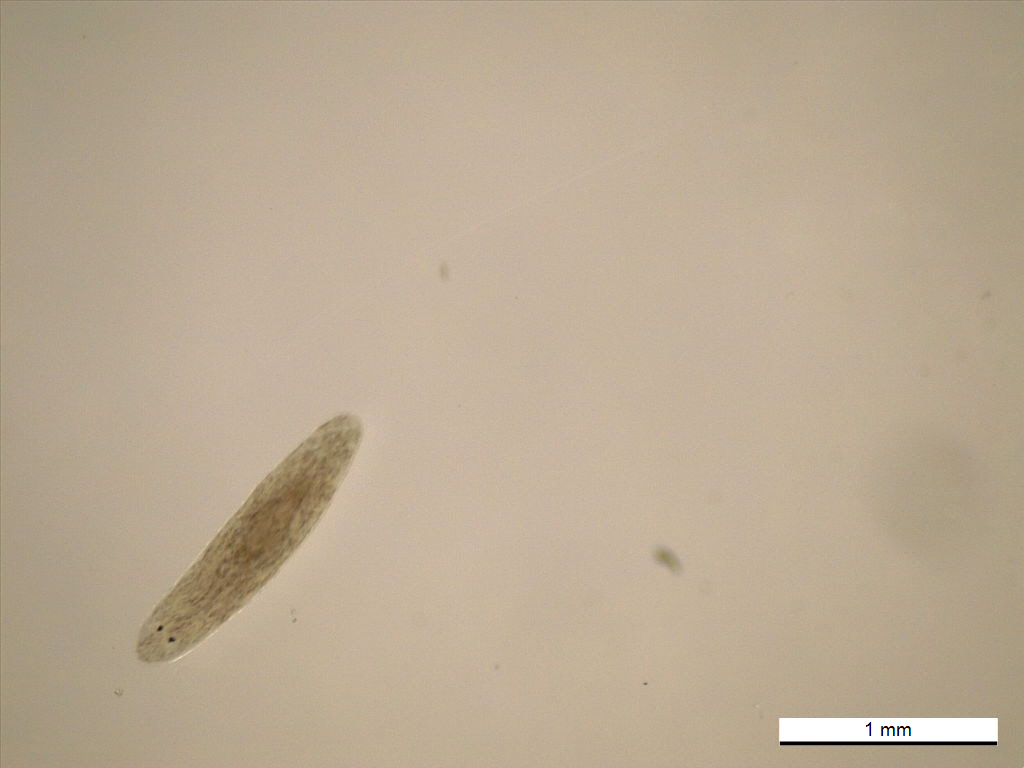

Supplement: Supplementary file 10 — Source data Fig. 3 [file 44318_2025_662_MOESM10_ESM.zip › Figure 3/3C-D/ythdf-a-b_RNAi_Before_RNAi_feedings/ythdf-a-b_RNAi_Before_RNAi_feedings_3.jpg]

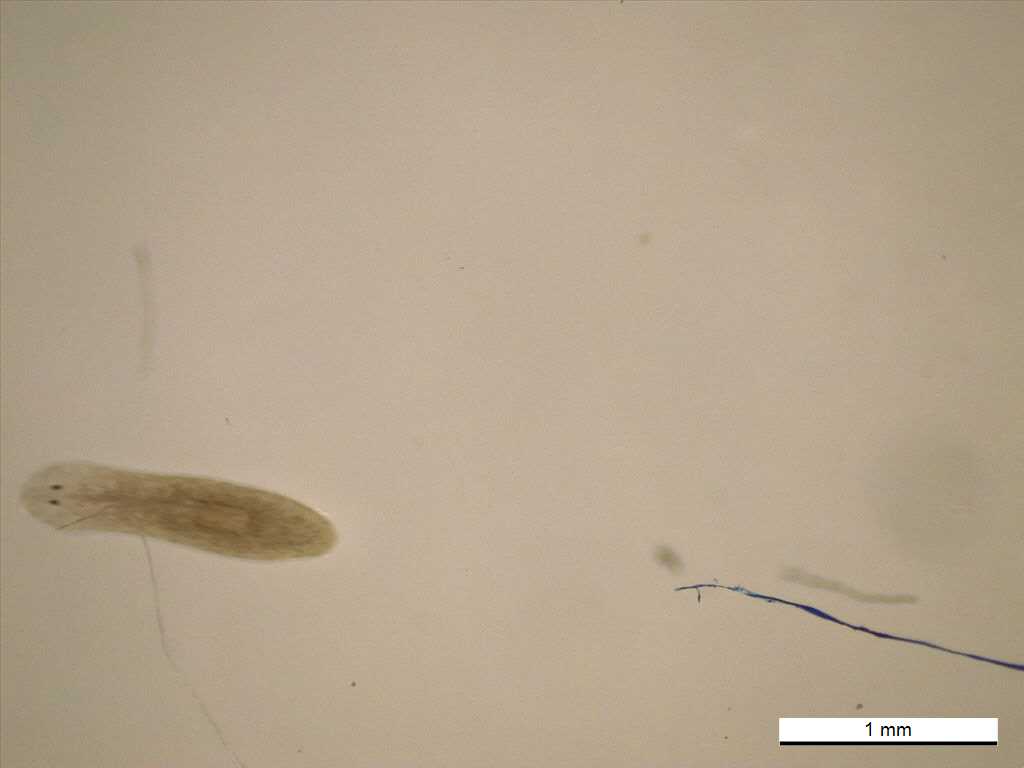

Supplement: Supplementary file 10 — Source data Fig. 3 [file 44318_2025_662_MOESM10_ESM.zip › Figure 3/3C-D/ythdf-a-b_RNAi_Before_RNAi_feedings/ythdf-a-b_RNAi_Before_RNAi_feedings_4.jpg]

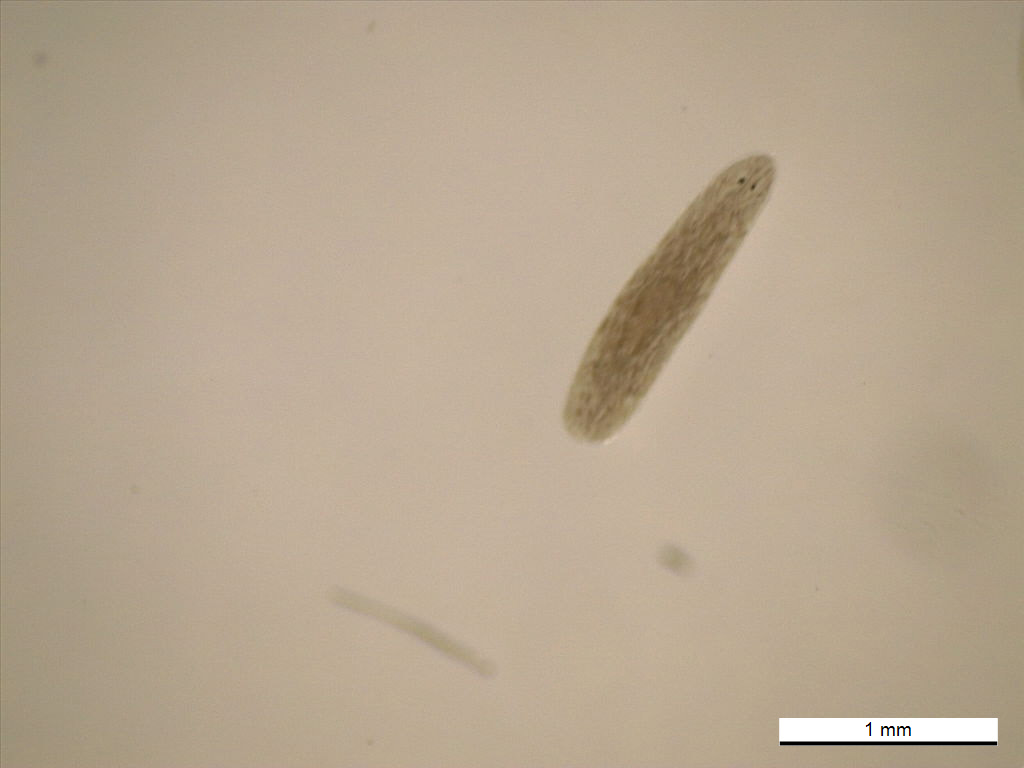

Supplement: Supplementary file 10 — Source data Fig. 3 [file 44318_2025_662_MOESM10_ESM.zip › Figure 3/3C-D/ythdf-a-b_RNAi_Before_RNAi_feedings/ythdf-a-b_RNAi_Before_RNAi_feedings_5.jpg]

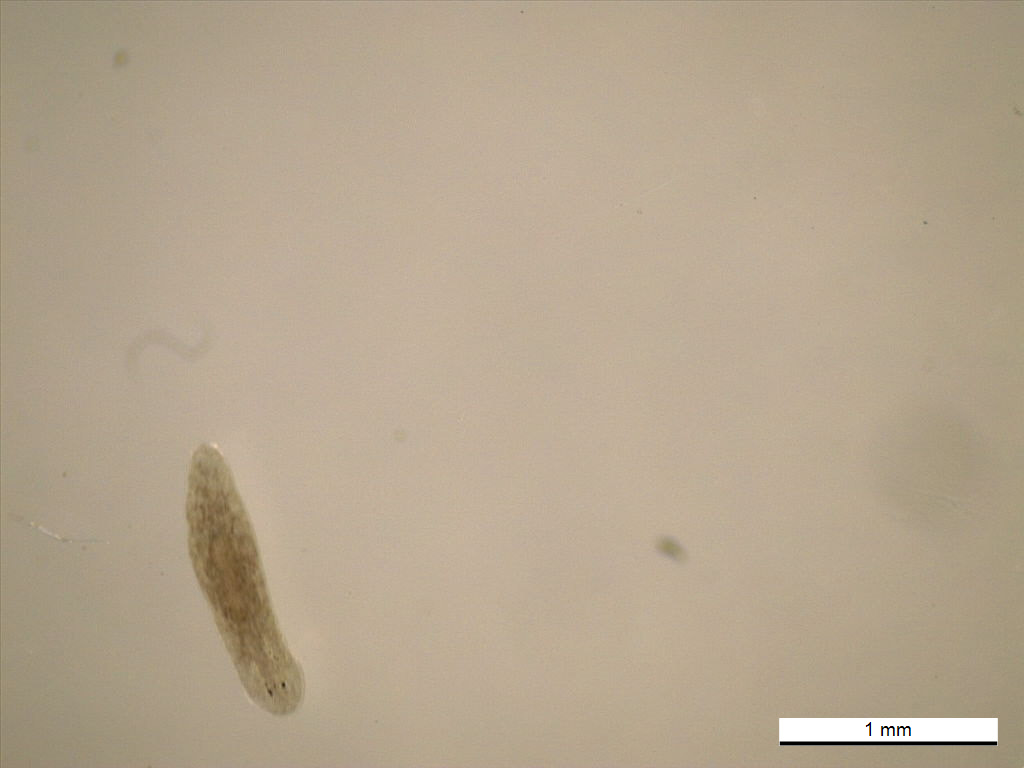

Supplement: Supplementary file 10 — Source data Fig. 3 [file 44318_2025_662_MOESM10_ESM.zip › Figure 3/3C-D/ythdf-a-b_RNAi_Before_RNAi_feedings/ythdf-a-b_RNAi_Before_RNAi_feedings_6.jpg]

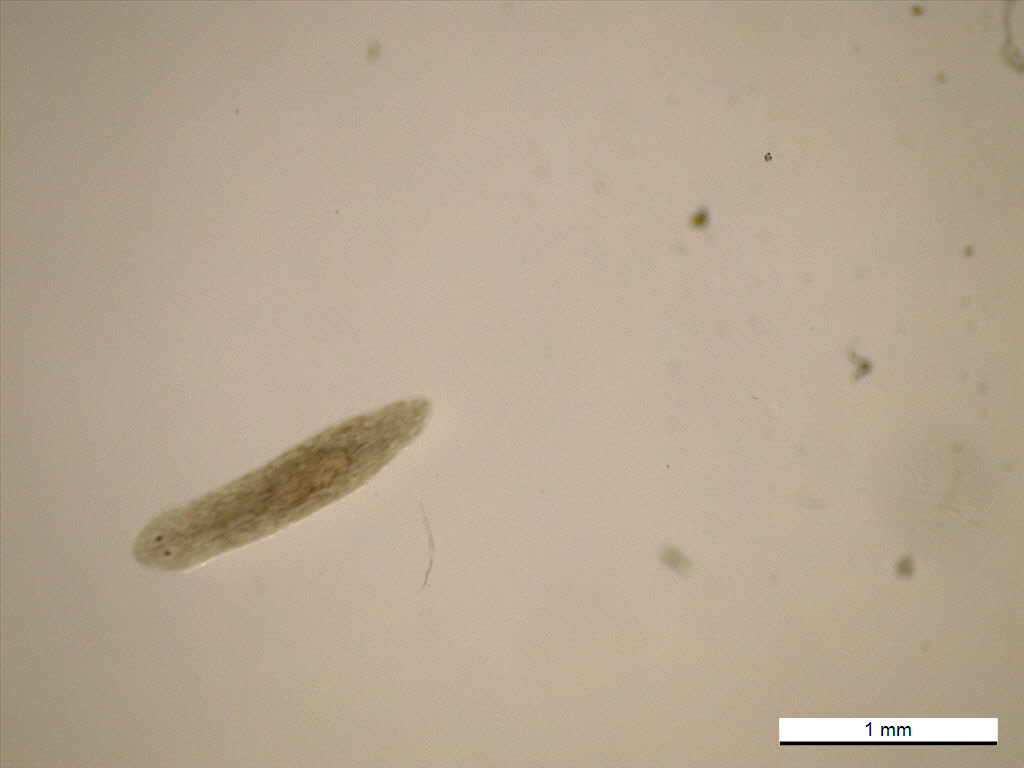

Supplement: Supplementary file 10 — Source data Fig. 3 [file 44318_2025_662_MOESM10_ESM.zip › Figure 3/3C-D/ythdf-a-b_RNAi_Before_RNAi_feedings/ythdf-a-b_RNAi_Before_RNAi_feedings_7.jpg]

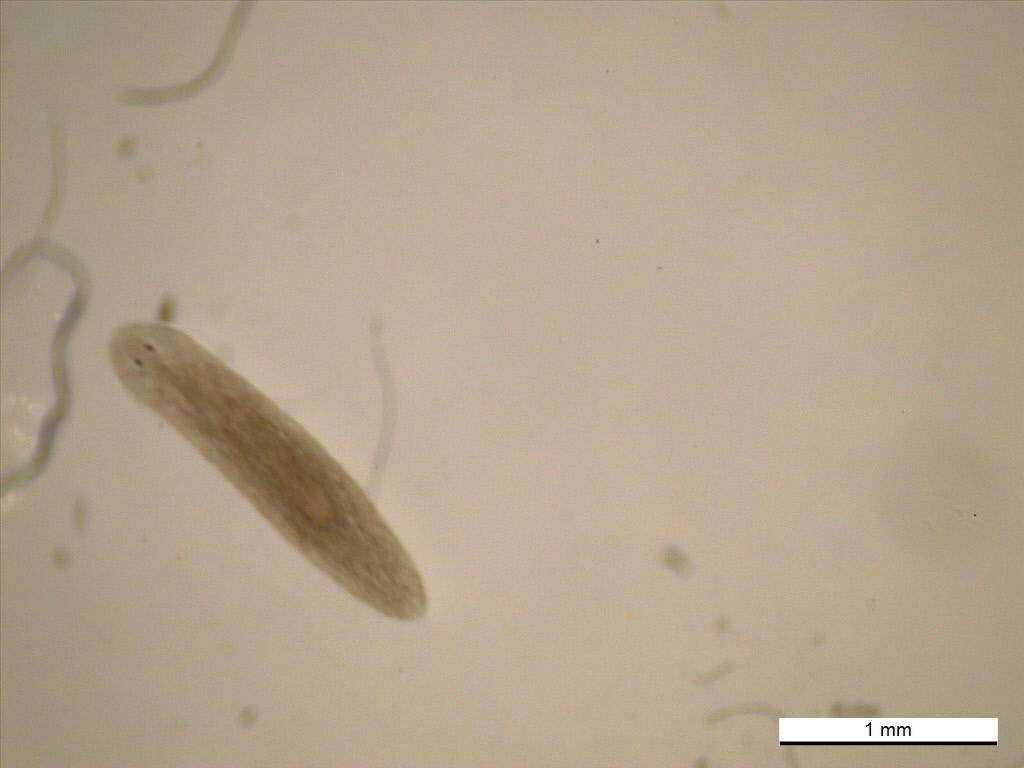

Supplement: Supplementary file 10 — Source data Fig. 3 [file 44318_2025_662_MOESM10_ESM.zip › Figure 3/3C-D/ythdf-a-b_RNAi_Before_RNAi_feedings/ythdf-a-b_RNAi_Before_RNAi_feedings_8.jpg]

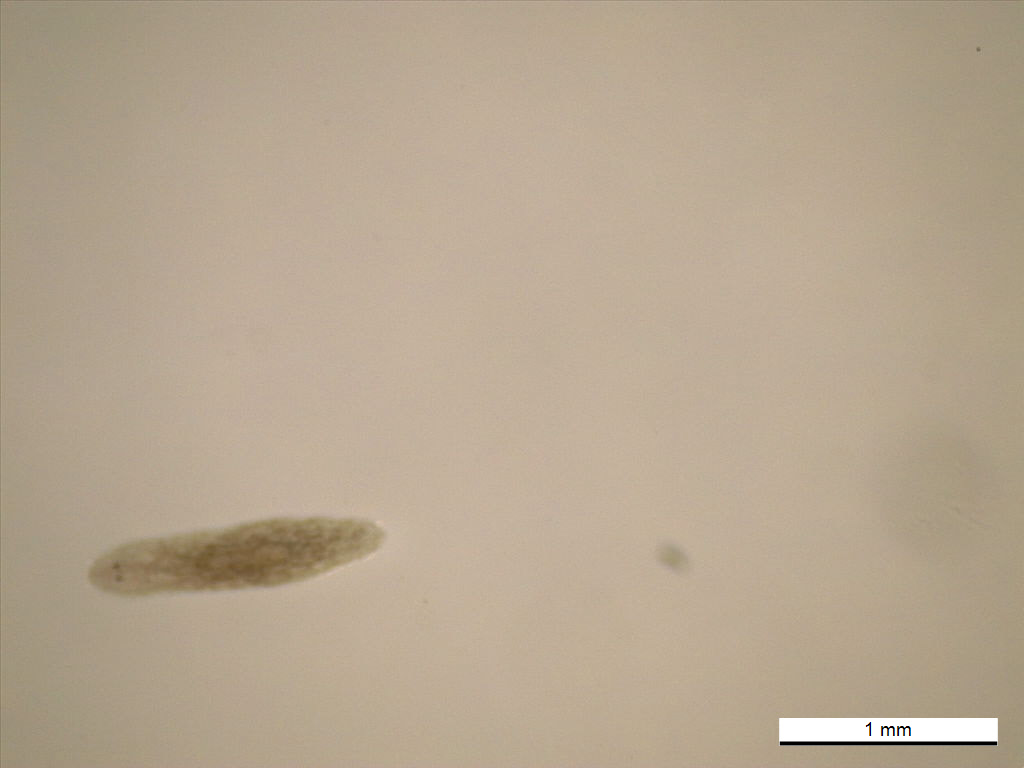

Supplement: Supplementary file 10 — Source data Fig. 3 [file 44318_2025_662_MOESM10_ESM.zip › Figure 3/3C-D/ythdf-a-b_RNAi_Before_RNAi_feedings/ythdf-a-b_RNAi_Before_RNAi_feedings_9.jpg]

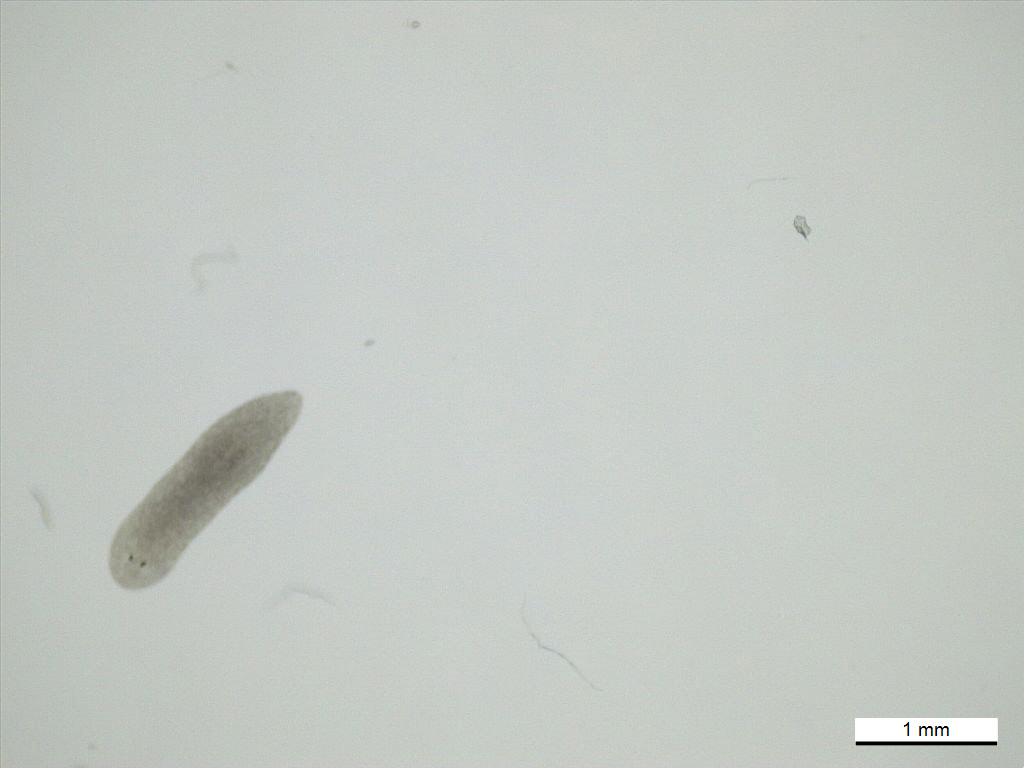

Supplement: Supplementary file 10 — Source data Fig. 3 [file 44318_2025_662_MOESM10_ESM.zip › Figure 3/3C-D/ythdf-a-c_RNAi_After_10_RNAi_feedings/ythdf_a-c_RNAi_After_10_RNAi_feedings_01.jpg]

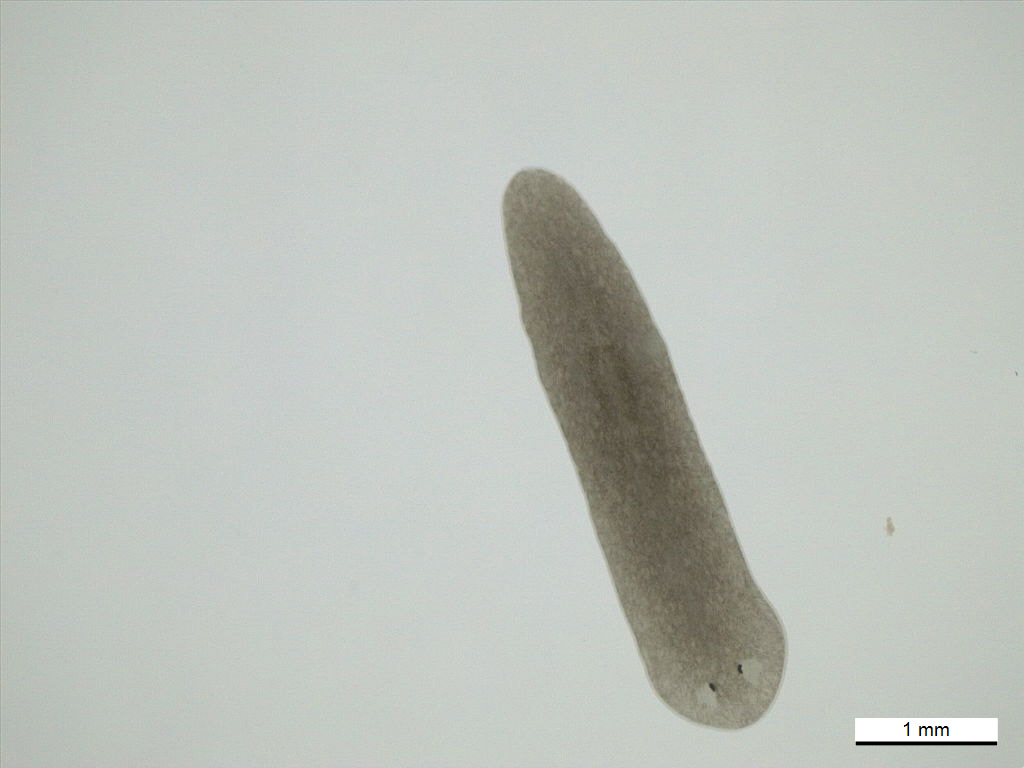

Supplement: Supplementary file 10 — Source data Fig. 3 [file 44318_2025_662_MOESM10_ESM.zip › Figure 3/3C-D/ythdf-a-c_RNAi_After_10_RNAi_feedings/ythdf_a-c_RNAi_After_10_RNAi_feedings_02.jpg]

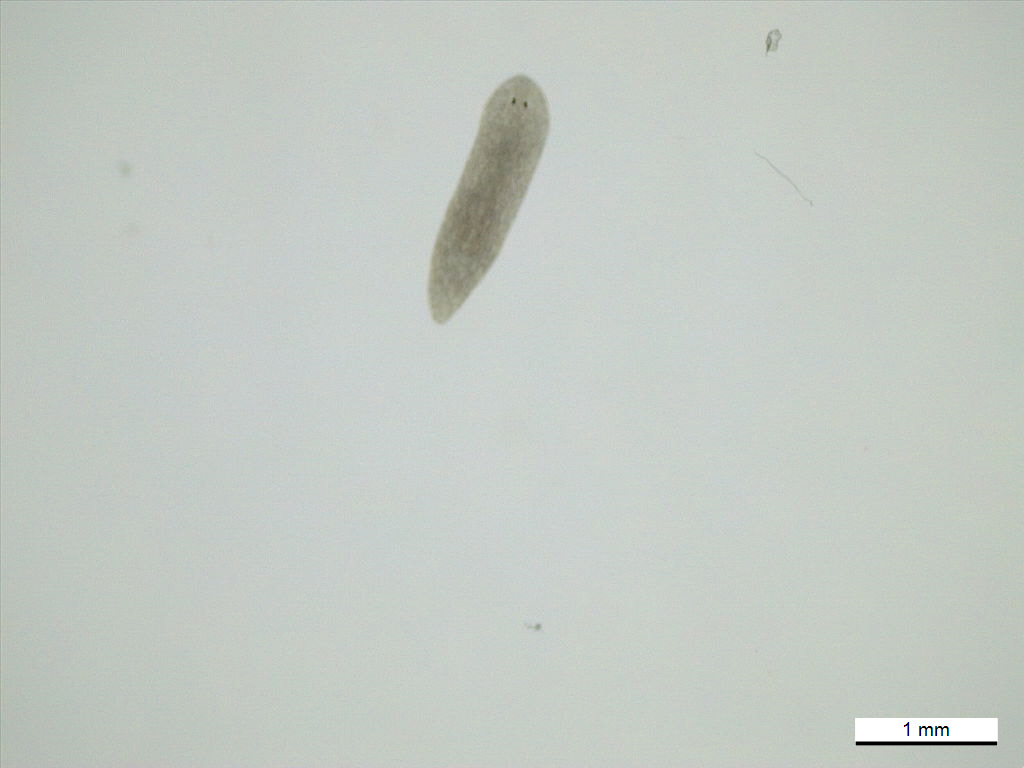

Supplement: Supplementary file 10 — Source data Fig. 3 [file 44318_2025_662_MOESM10_ESM.zip › Figure 3/3C-D/ythdf-a-c_RNAi_After_10_RNAi_feedings/ythdf_a-c_RNAi_After_10_RNAi_feedings_03.jpg]

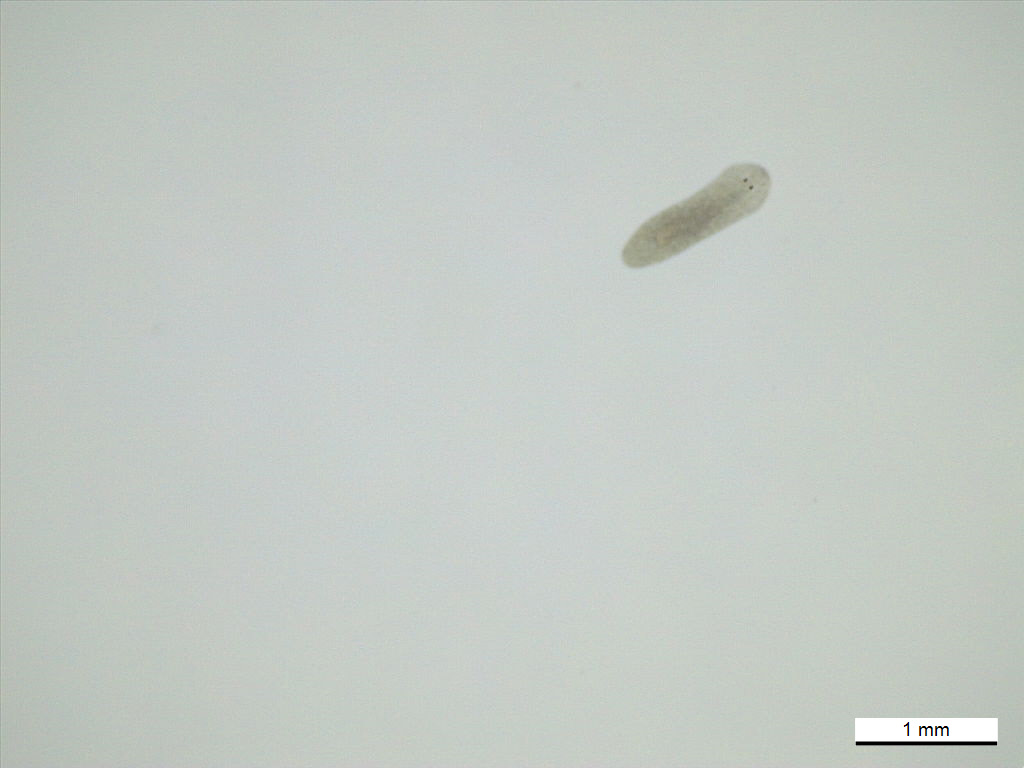

Supplement: Supplementary file 10 — Source data Fig. 3 [file 44318_2025_662_MOESM10_ESM.zip › Figure 3/3C-D/ythdf-a-c_RNAi_After_10_RNAi_feedings/ythdf_a-c_RNAi_After_10_RNAi_feedings_04.jpg]

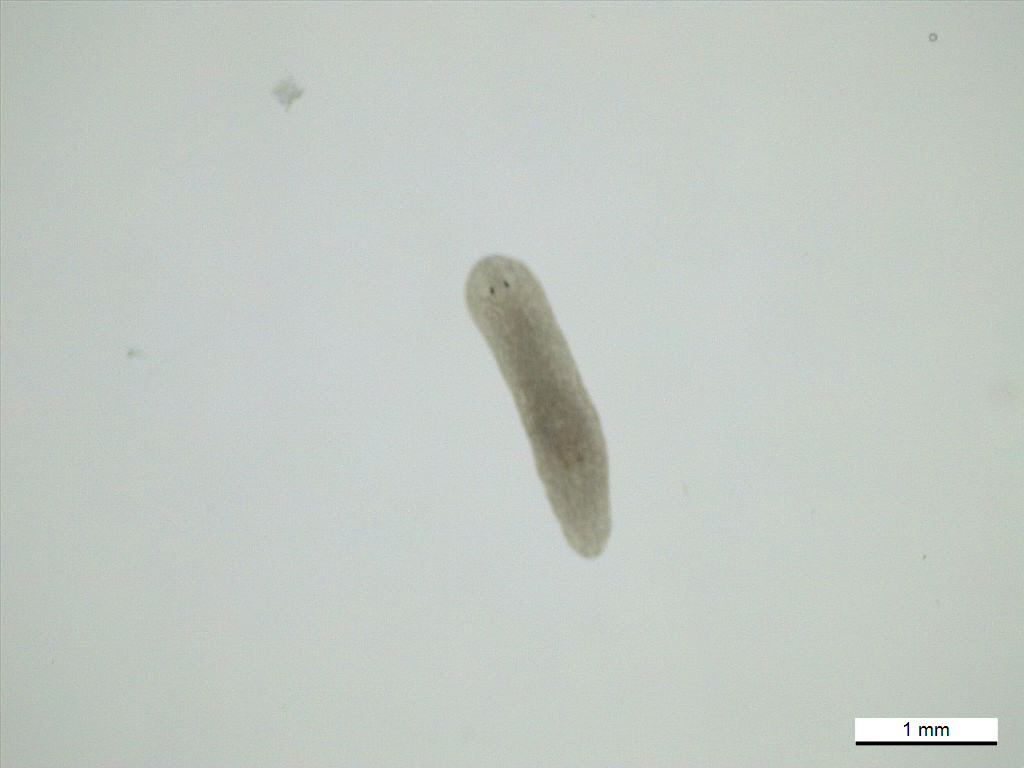

Supplement: Supplementary file 10 — Source data Fig. 3 [file 44318_2025_662_MOESM10_ESM.zip › Figure 3/3C-D/ythdf-a-c_RNAi_After_10_RNAi_feedings/ythdf_a-c_RNAi_After_10_RNAi_feedings_05.jpg]

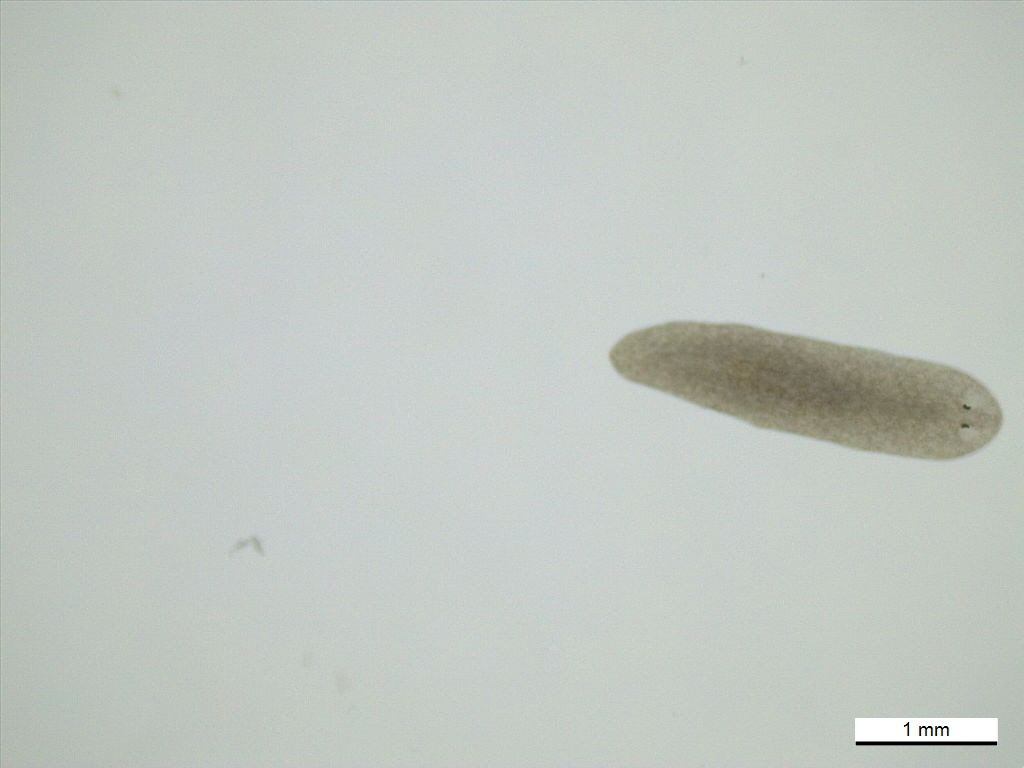

Supplement: Supplementary file 10 — Source data Fig. 3 [file 44318_2025_662_MOESM10_ESM.zip › Figure 3/3C-D/ythdf-a-c_RNAi_After_10_RNAi_feedings/ythdf_a-c_RNAi_After_10_RNAi_feedings_06.jpg]

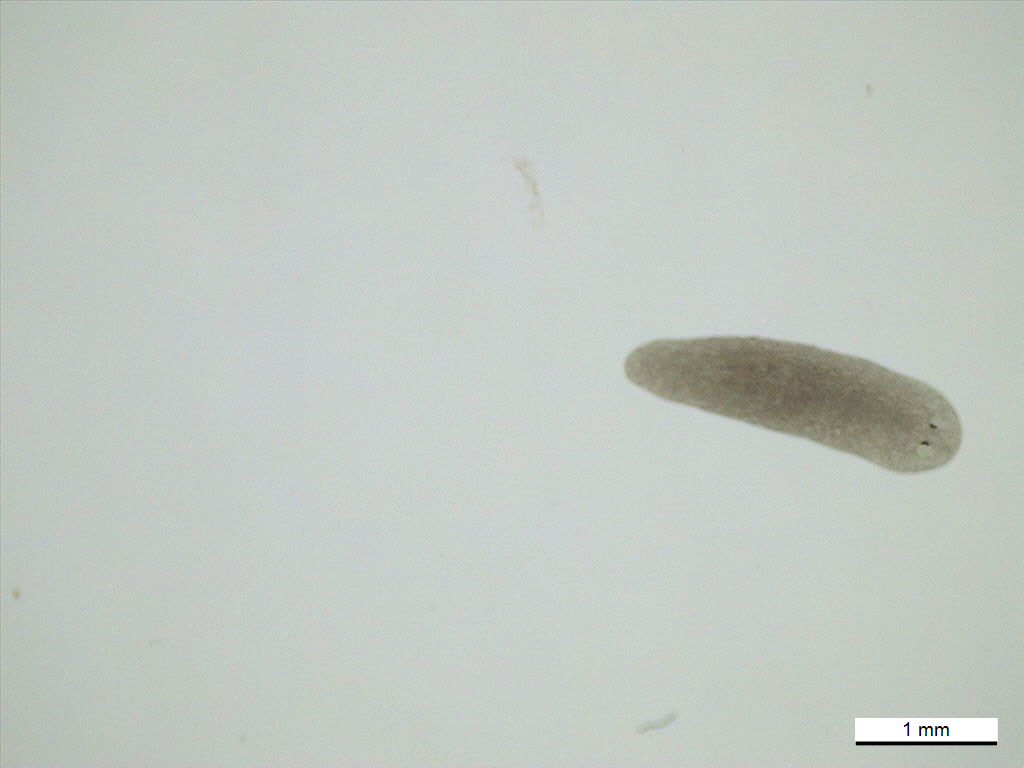

Supplement: Supplementary file 10 — Source data Fig. 3 [file 44318_2025_662_MOESM10_ESM.zip › Figure 3/3C-D/ythdf-a-c_RNAi_After_10_RNAi_feedings/ythdf_a-c_RNAi_After_10_RNAi_feedings_07.jpg]

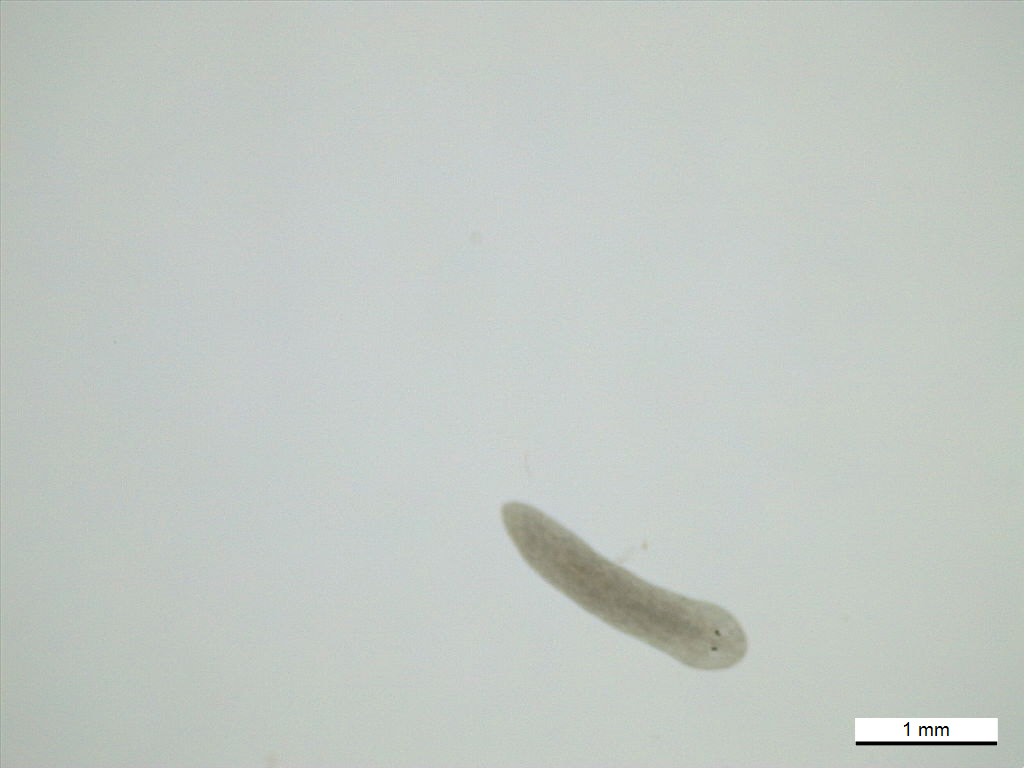

Supplement: Supplementary file 10 — Source data Fig. 3 [file 44318_2025_662_MOESM10_ESM.zip › Figure 3/3C-D/ythdf-a-c_RNAi_After_10_RNAi_feedings/ythdf_a-c_RNAi_After_10_RNAi_feedings_08.jpg]

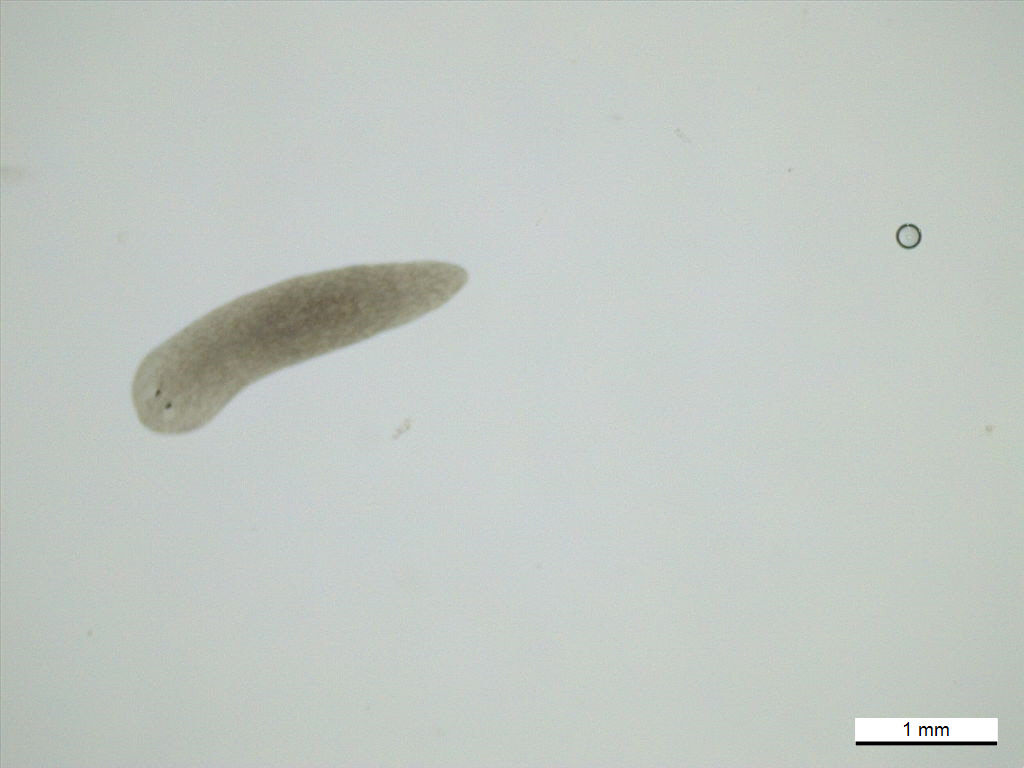

Supplement: Supplementary file 10 — Source data Fig. 3 [file 44318_2025_662_MOESM10_ESM.zip › Figure 3/3C-D/ythdf-a-c_RNAi_After_10_RNAi_feedings/ythdf_a-c_RNAi_After_10_RNAi_feedings_09.jpg]

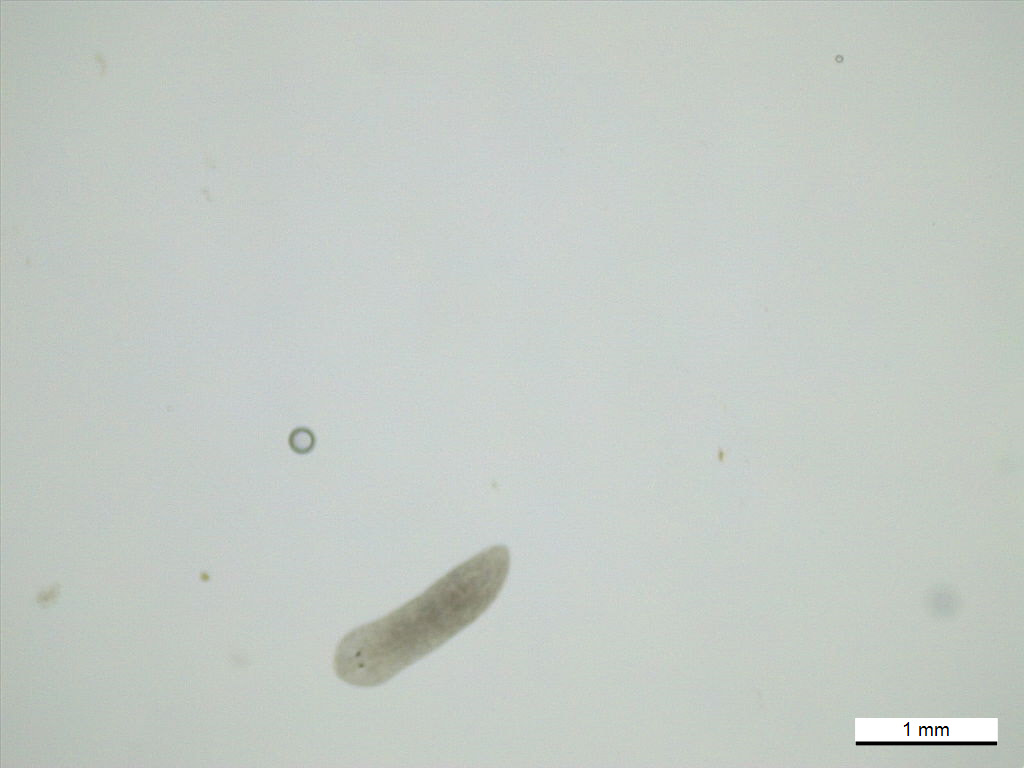

Supplement: Supplementary file 10 — Source data Fig. 3 [file 44318_2025_662_MOESM10_ESM.zip › Figure 3/3C-D/ythdf-a-c_RNAi_After_10_RNAi_feedings/ythdf_a-c_RNAi_After_10_RNAi_feedings_10.jpg]

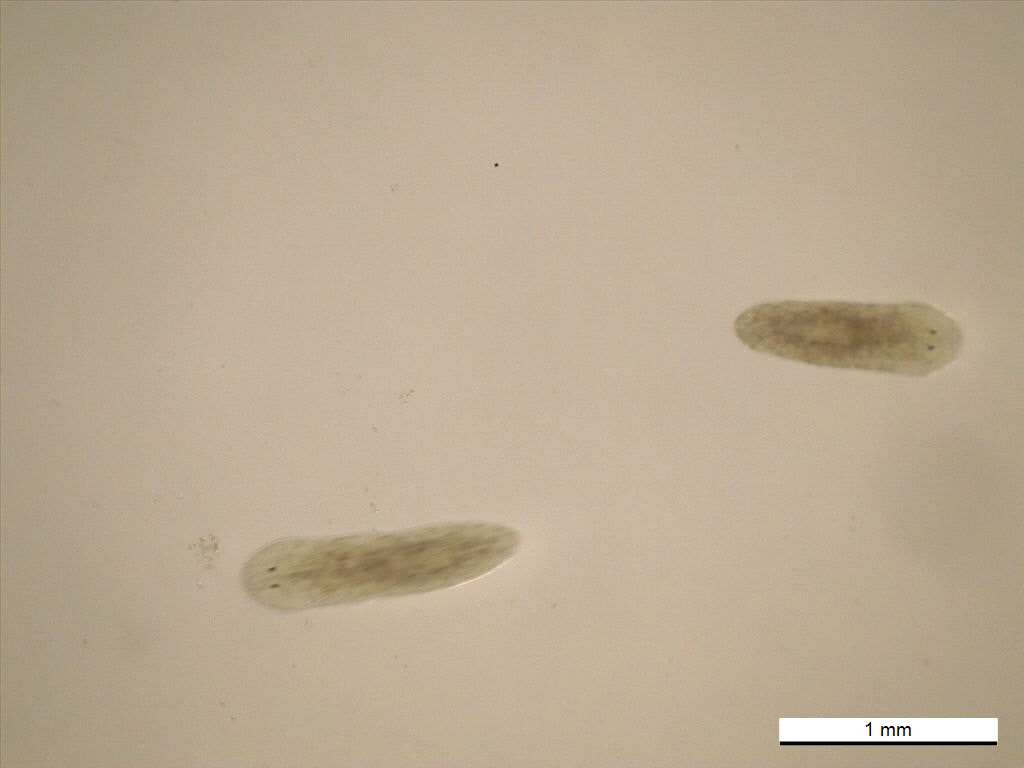

Supplement: Supplementary file 10 — Source data Fig. 3 [file 44318_2025_662_MOESM10_ESM.zip › Figure 3/3C-D/ythdf-a-c_RNAi_Before_RNAi_feedings/ythdf-a-c_RNAi_Before_RNAi_feedings_1.jpg]

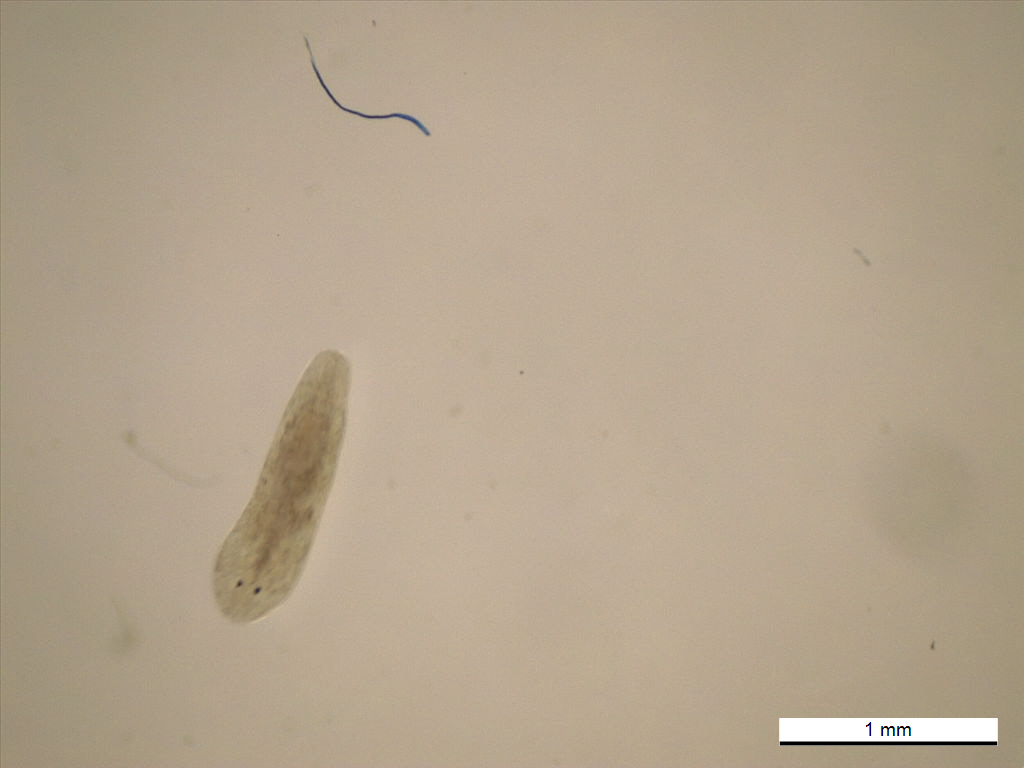

Supplement: Supplementary file 10 — Source data Fig. 3 [file 44318_2025_662_MOESM10_ESM.zip › Figure 3/3C-D/ythdf-a-c_RNAi_Before_RNAi_feedings/ythdf-a-c_RNAi_Before_RNAi_feedings_10.jpg]

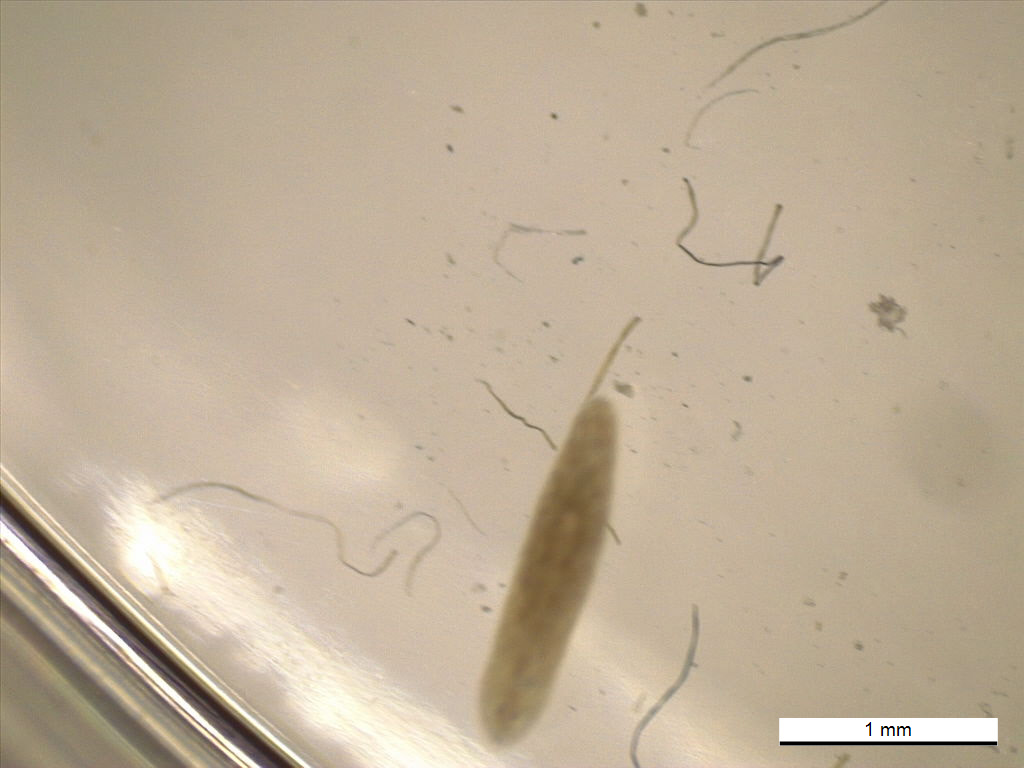

Supplement: Supplementary file 10 — Source data Fig. 3 [file 44318_2025_662_MOESM10_ESM.zip › Figure 3/3C-D/ythdf-a-c_RNAi_Before_RNAi_feedings/ythdf-a-c_RNAi_Before_RNAi_feedings_11.jpg]

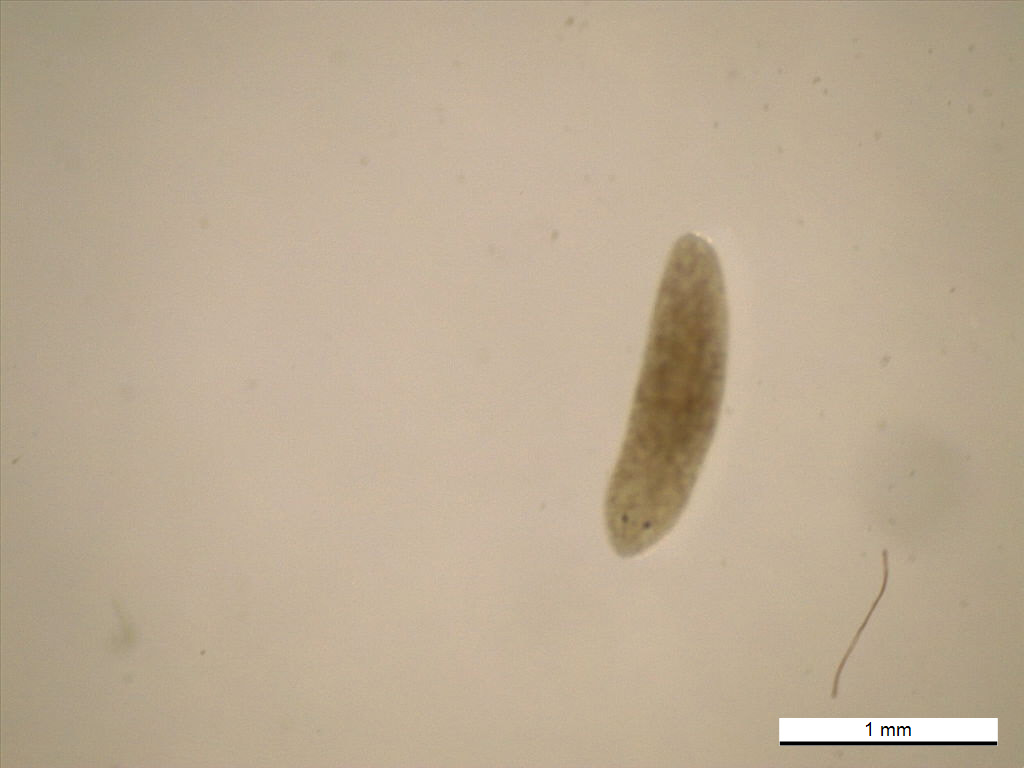

Supplement: Supplementary file 10 — Source data Fig. 3 [file 44318_2025_662_MOESM10_ESM.zip › Figure 3/3C-D/ythdf-a-c_RNAi_Before_RNAi_feedings/ythdf-a-c_RNAi_Before_RNAi_feedings_12.jpg]

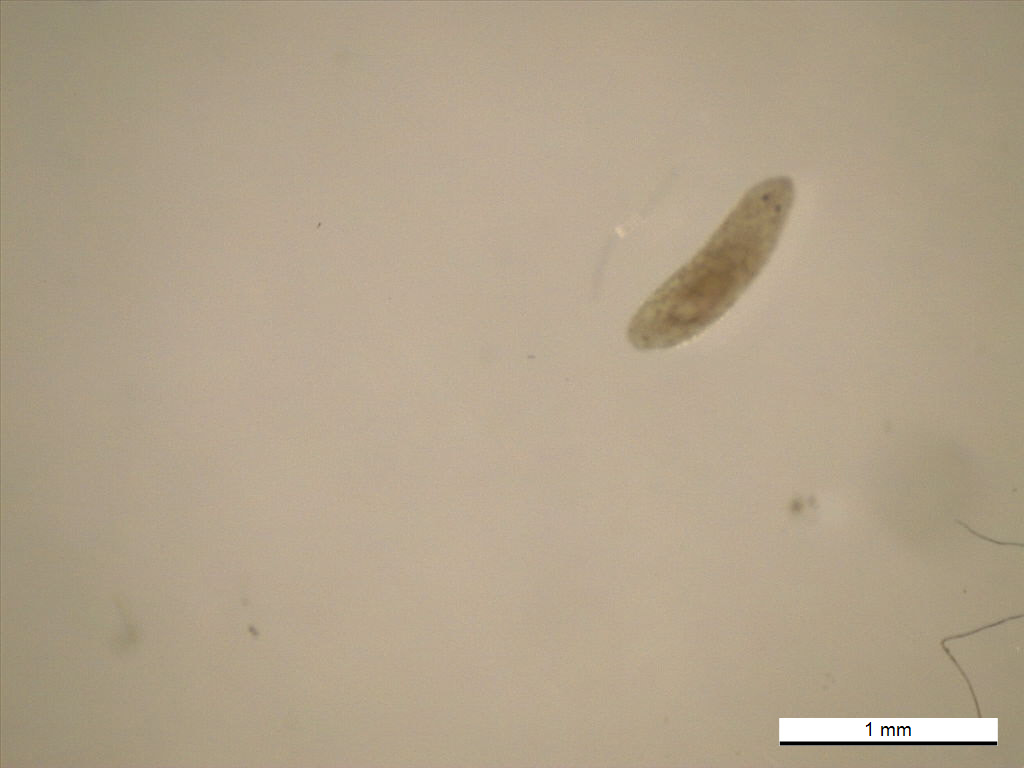

Supplement: Supplementary file 10 — Source data Fig. 3 [file 44318_2025_662_MOESM10_ESM.zip › Figure 3/3C-D/ythdf-a-c_RNAi_Before_RNAi_feedings/ythdf-a-c_RNAi_Before_RNAi_feedings_13.jpg]

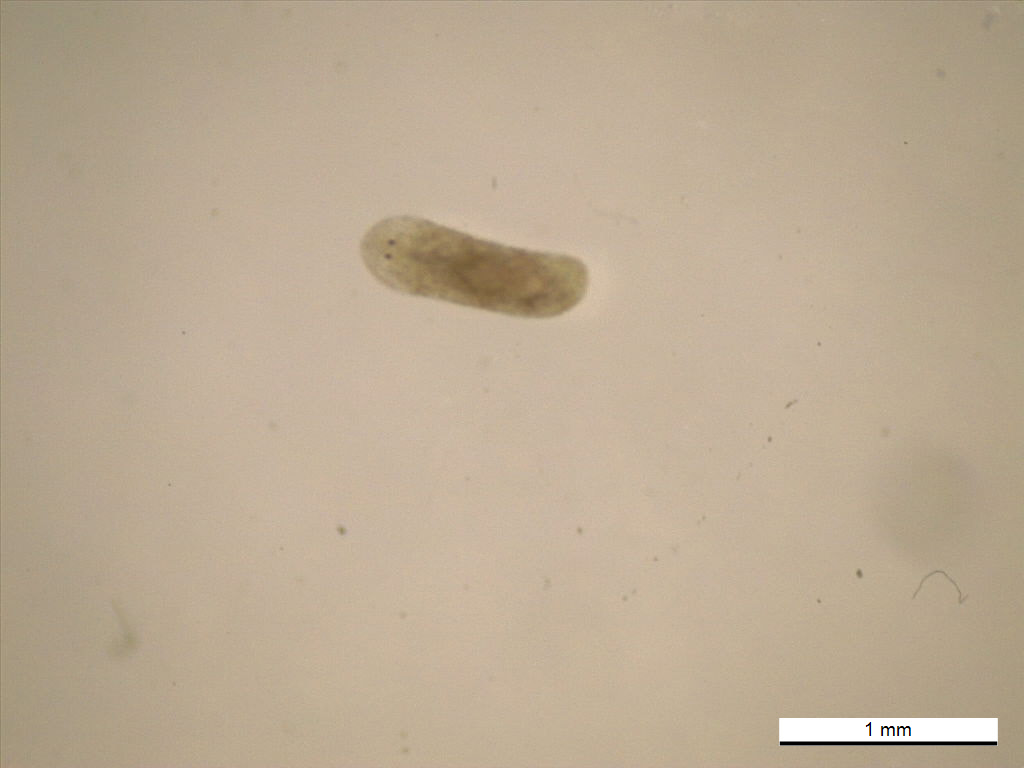

Supplement: Supplementary file 10 — Source data Fig. 3 [file 44318_2025_662_MOESM10_ESM.zip › Figure 3/3C-D/ythdf-a-c_RNAi_Before_RNAi_feedings/ythdf-a-c_RNAi_Before_RNAi_feedings_14.jpg]

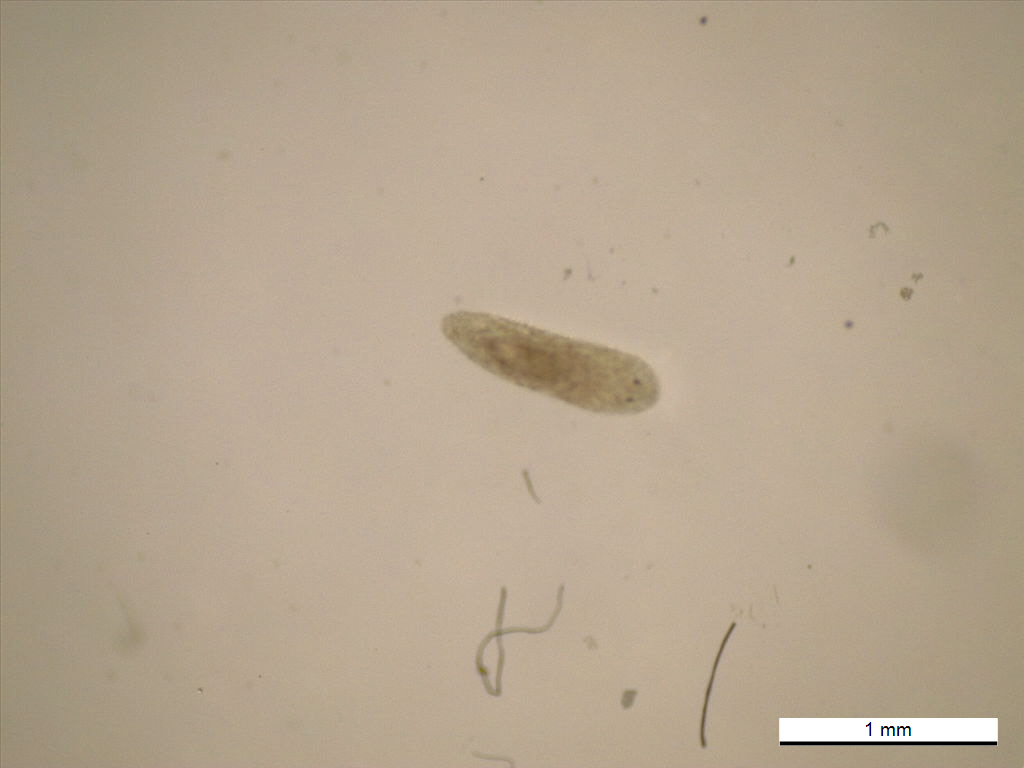

Supplement: Supplementary file 10 — Source data Fig. 3 [file 44318_2025_662_MOESM10_ESM.zip › Figure 3/3C-D/ythdf-a-c_RNAi_Before_RNAi_feedings/ythdf-a-c_RNAi_Before_RNAi_feedings_15.jpg]

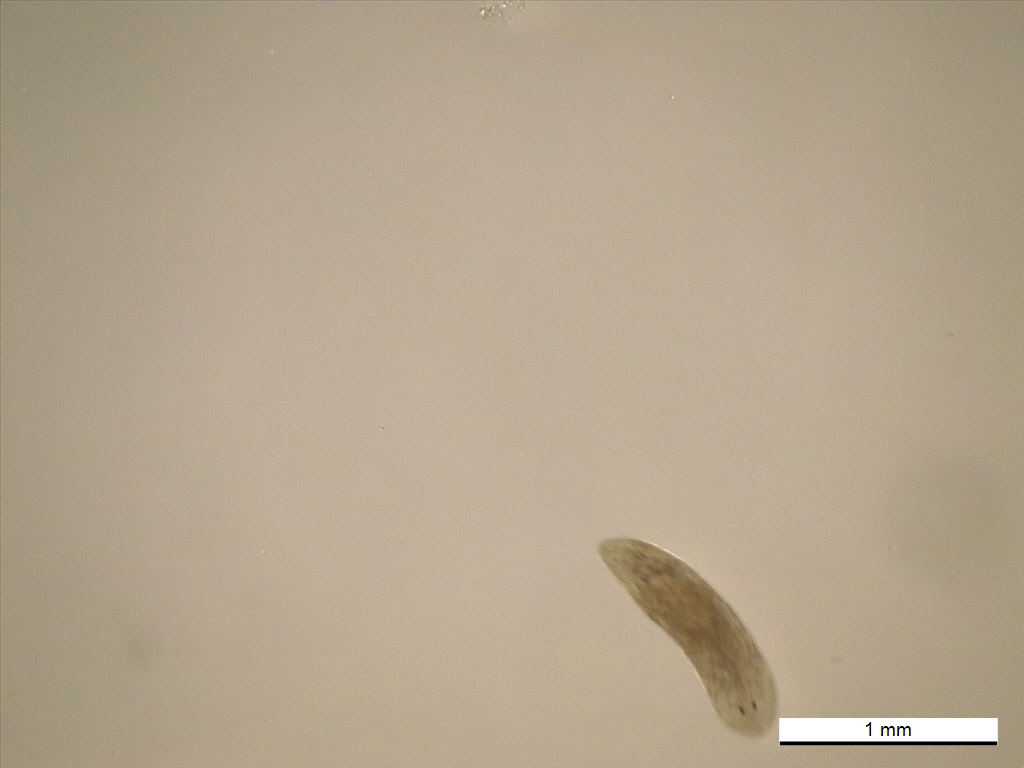

Supplement: Supplementary file 10 — Source data Fig. 3 [file 44318_2025_662_MOESM10_ESM.zip › Figure 3/3C-D/ythdf-a-c_RNAi_Before_RNAi_feedings/ythdf-a-c_RNAi_Before_RNAi_feedings_2.jpg]

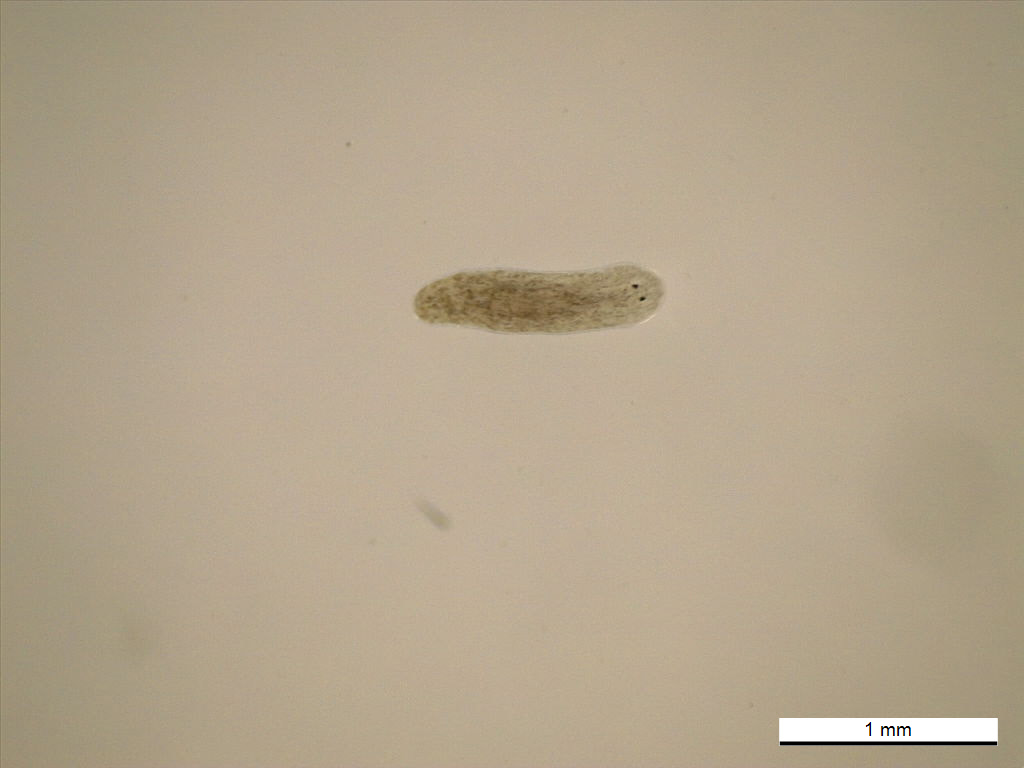

Supplement: Supplementary file 10 — Source data Fig. 3 [file 44318_2025_662_MOESM10_ESM.zip › Figure 3/3C-D/ythdf-a-c_RNAi_Before_RNAi_feedings/ythdf-a-c_RNAi_Before_RNAi_feedings_3.jpg]

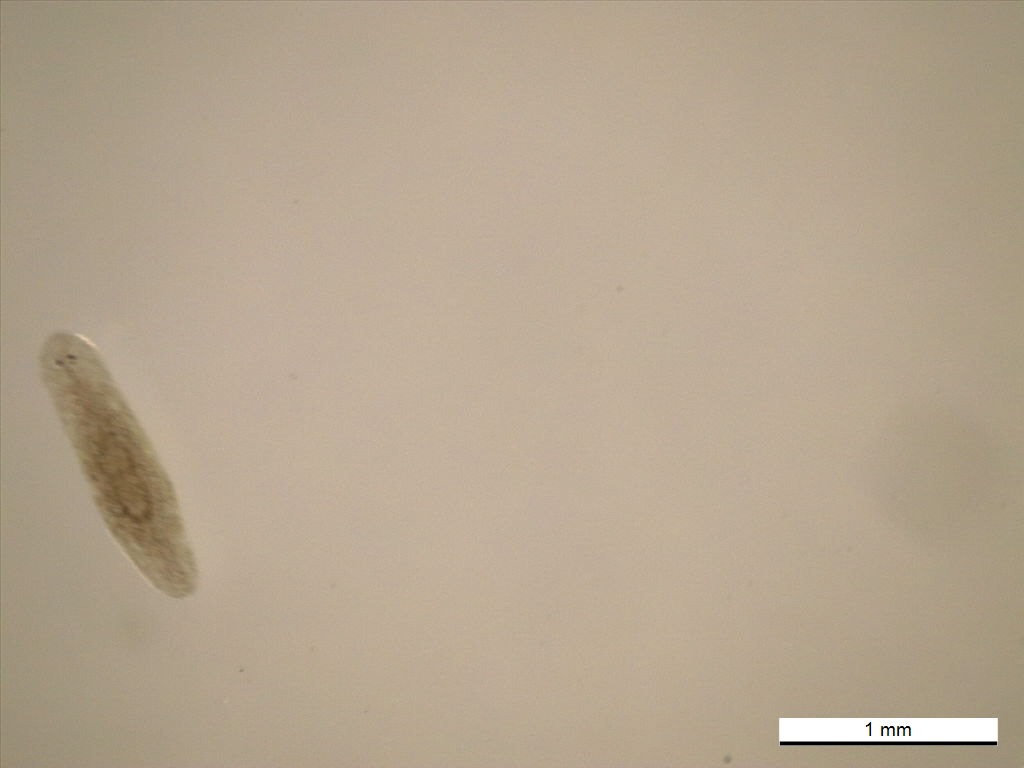

Supplement: Supplementary file 10 — Source data Fig. 3 [file 44318_2025_662_MOESM10_ESM.zip › Figure 3/3C-D/ythdf-a-c_RNAi_Before_RNAi_feedings/ythdf-a-c_RNAi_Before_RNAi_feedings_4.jpg]

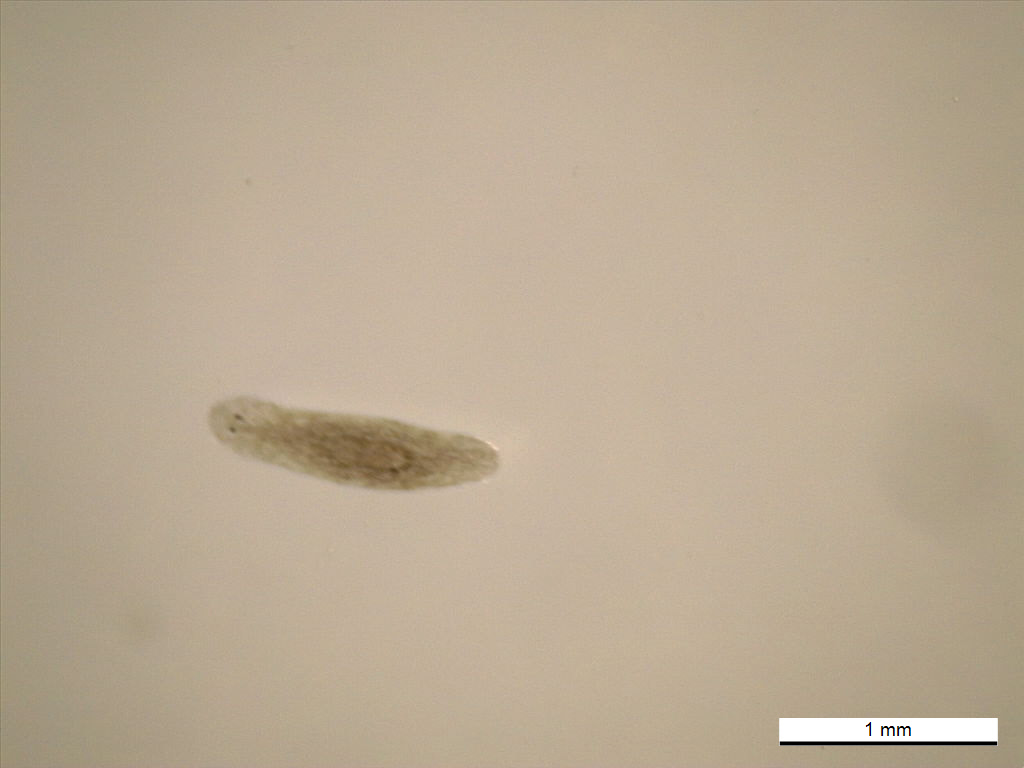

Supplement: Supplementary file 10 — Source data Fig. 3 [file 44318_2025_662_MOESM10_ESM.zip › Figure 3/3C-D/ythdf-a-c_RNAi_Before_RNAi_feedings/ythdf-a-c_RNAi_Before_RNAi_feedings_5.jpg]

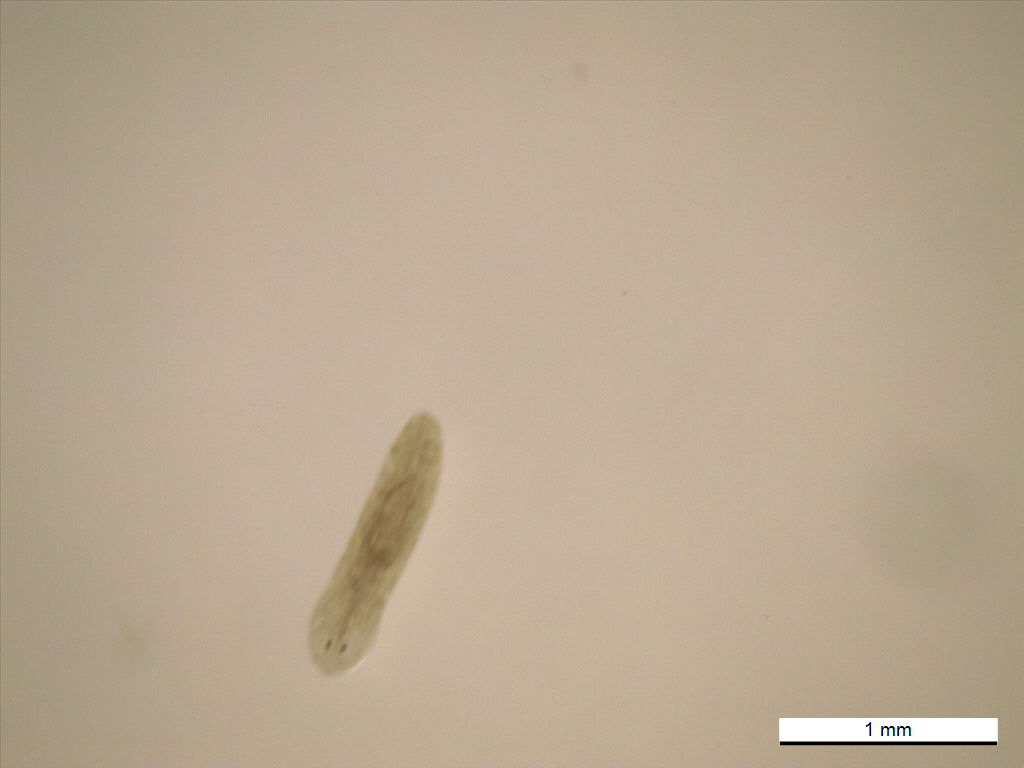

Supplement: Supplementary file 10 — Source data Fig. 3 [file 44318_2025_662_MOESM10_ESM.zip › Figure 3/3C-D/ythdf-a-c_RNAi_Before_RNAi_feedings/ythdf-a-c_RNAi_Before_RNAi_feedings_6.jpg]

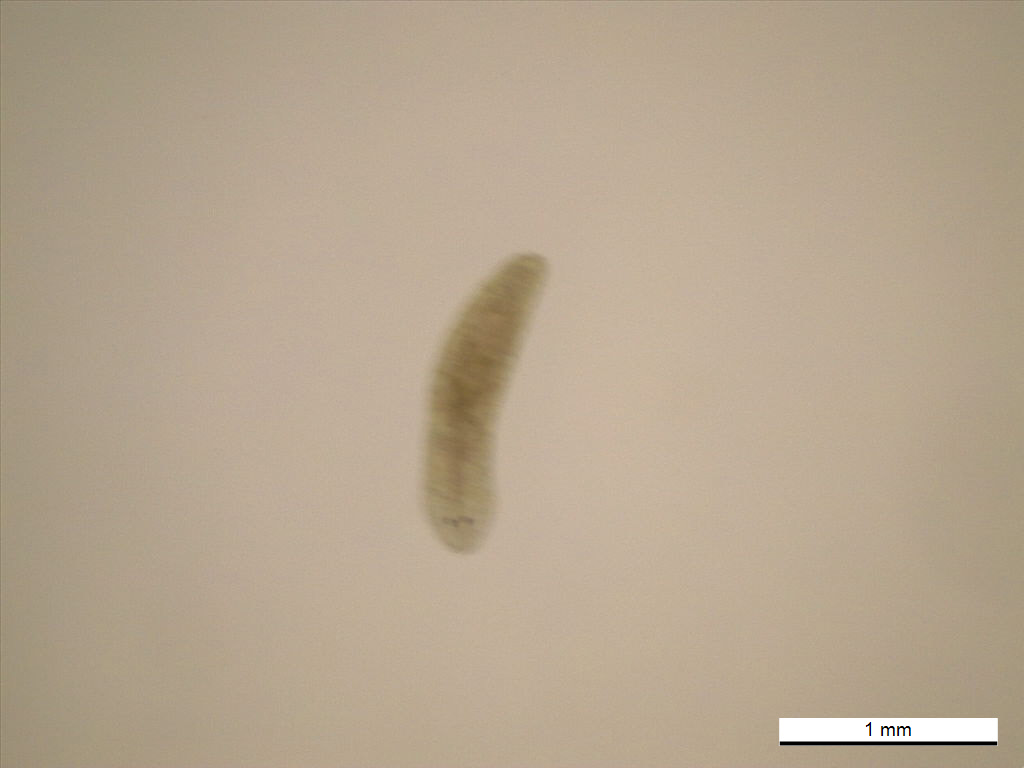

Supplement: Supplementary file 10 — Source data Fig. 3 [file 44318_2025_662_MOESM10_ESM.zip › Figure 3/3C-D/ythdf-a-c_RNAi_Before_RNAi_feedings/ythdf-a-c_RNAi_Before_RNAi_feedings_7.jpg]

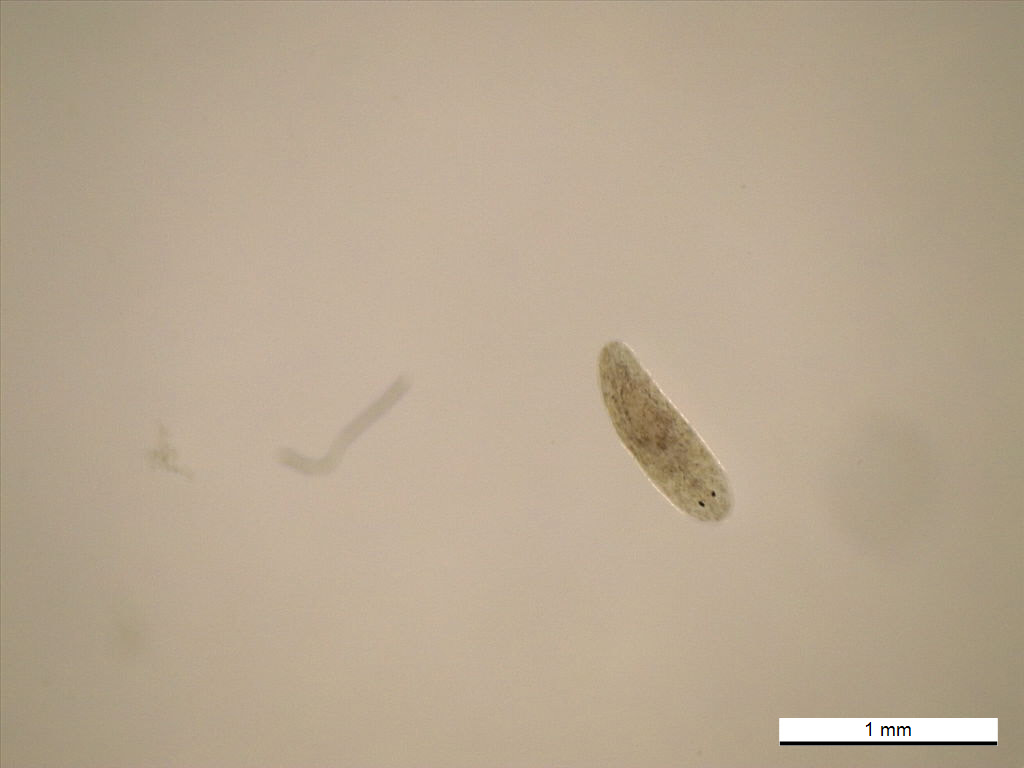

Supplement: Supplementary file 10 — Source data Fig. 3 [file 44318_2025_662_MOESM10_ESM.zip › Figure 3/3C-D/ythdf-a-c_RNAi_Before_RNAi_feedings/ythdf-a-c_RNAi_Before_RNAi_feedings_8.jpg]

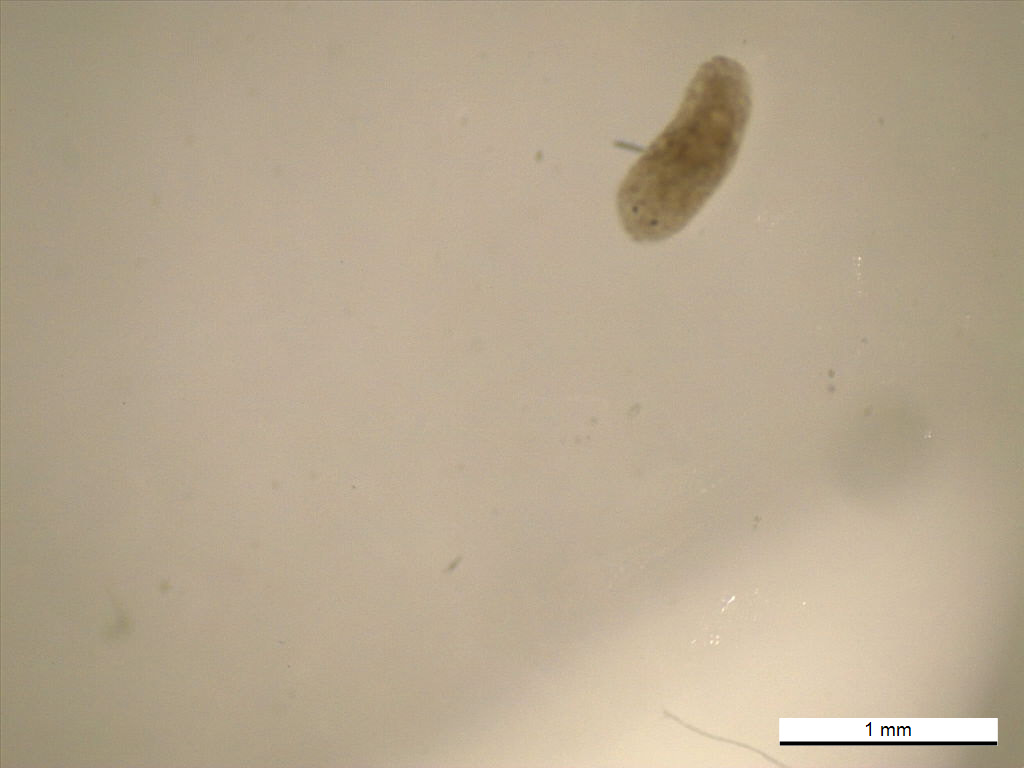

Supplement: Supplementary file 10 — Source data Fig. 3 [file 44318_2025_662_MOESM10_ESM.zip › Figure 3/3C-D/ythdf-a-c_RNAi_Before_RNAi_feedings/ythdf-a-c_RNAi_Before_RNAi_feedings_9.jpg]

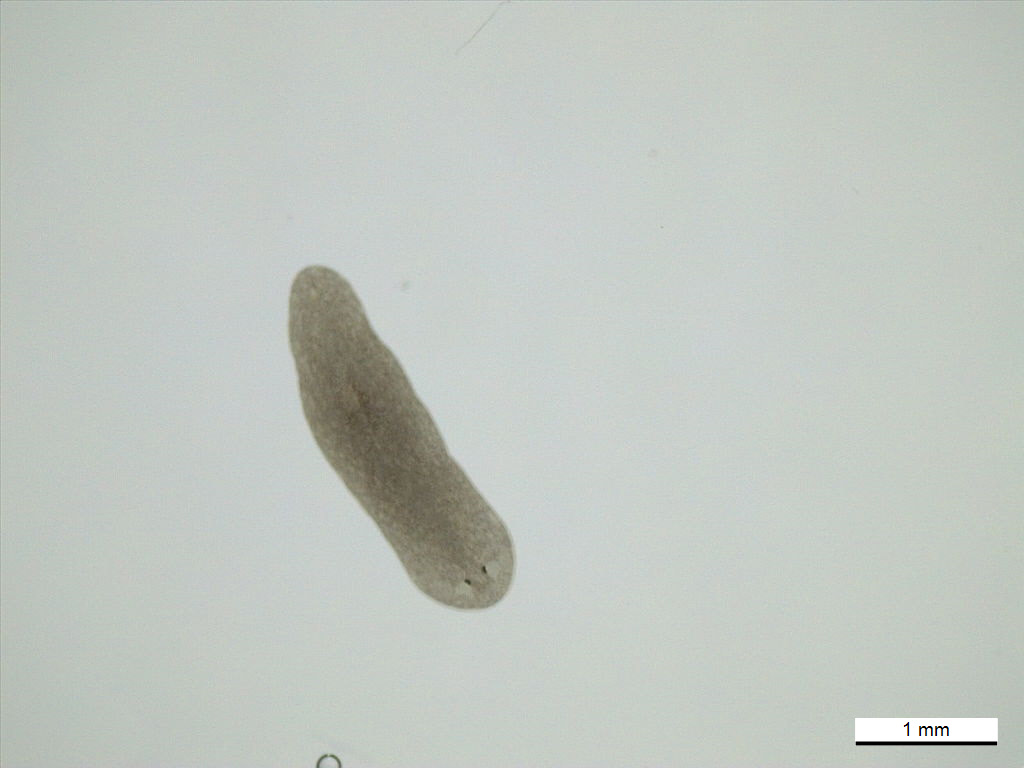

Supplement: Supplementary file 10 — Source data Fig. 3 [file 44318_2025_662_MOESM10_ESM.zip › Figure 3/3C-D/ythdf-a_RNAi_After_10_RNAi_feedings/ythdf-a_RNAi_After_10_RNAi_feedings_01.jpg]

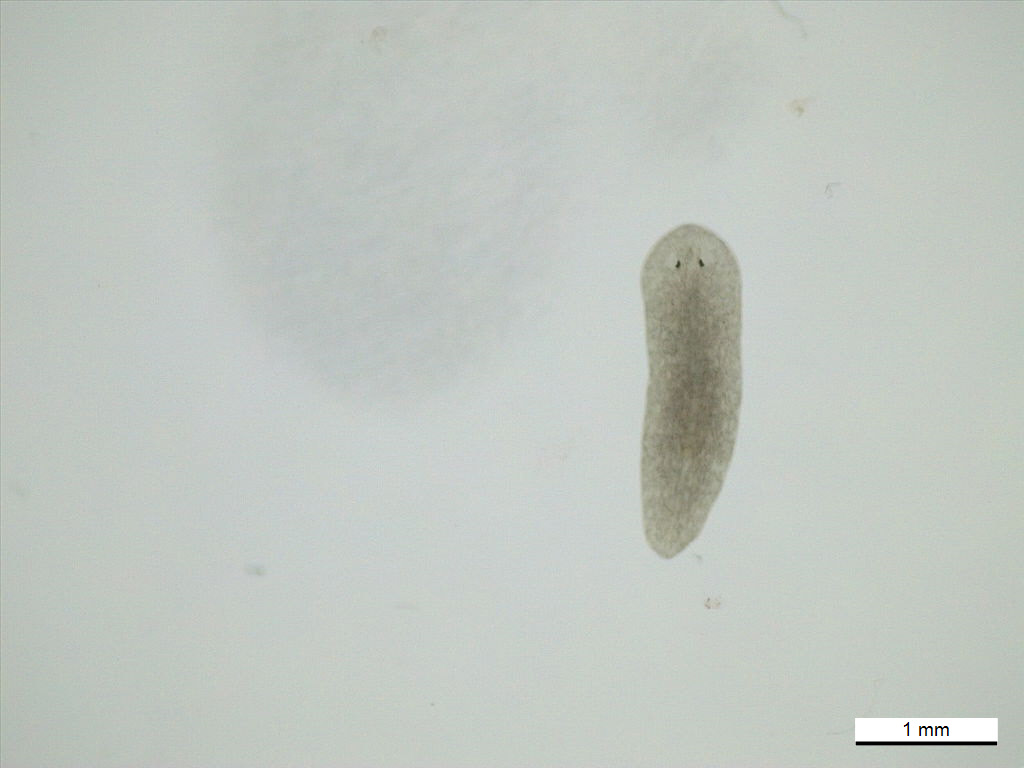

Supplement: Supplementary file 10 — Source data Fig. 3 [file 44318_2025_662_MOESM10_ESM.zip › Figure 3/3C-D/ythdf-a_RNAi_After_10_RNAi_feedings/ythdf-a_RNAi_After_10_RNAi_feedings_02.jpg]

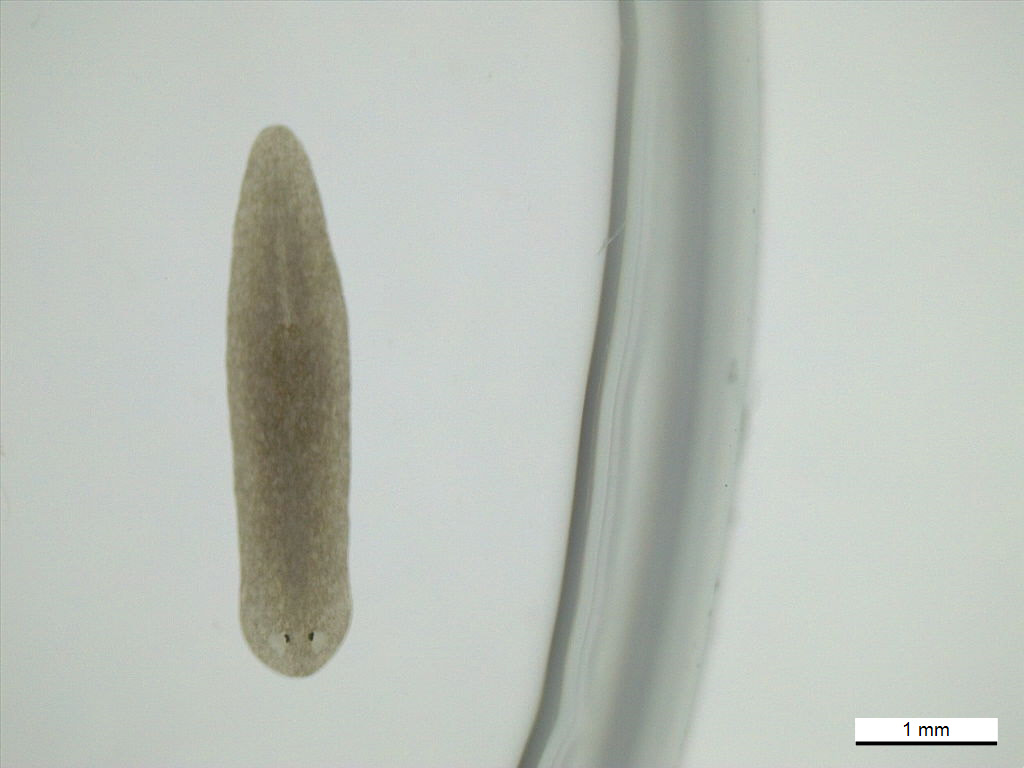

Supplement: Supplementary file 10 — Source data Fig. 3 [file 44318_2025_662_MOESM10_ESM.zip › Figure 3/3C-D/ythdf-a_RNAi_After_10_RNAi_feedings/ythdf-a_RNAi_After_10_RNAi_feedings_03.jpg]

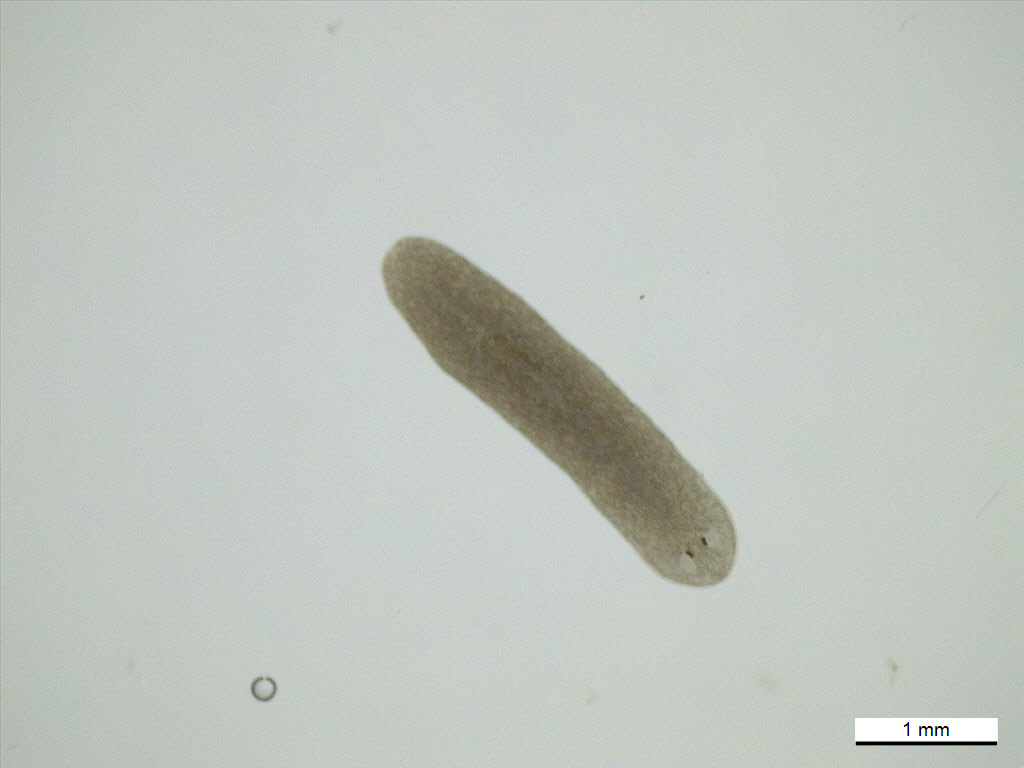

Supplement: Supplementary file 10 — Source data Fig. 3 [file 44318_2025_662_MOESM10_ESM.zip › Figure 3/3C-D/ythdf-a_RNAi_After_10_RNAi_feedings/ythdf-a_RNAi_After_10_RNAi_feedings_04.jpg]

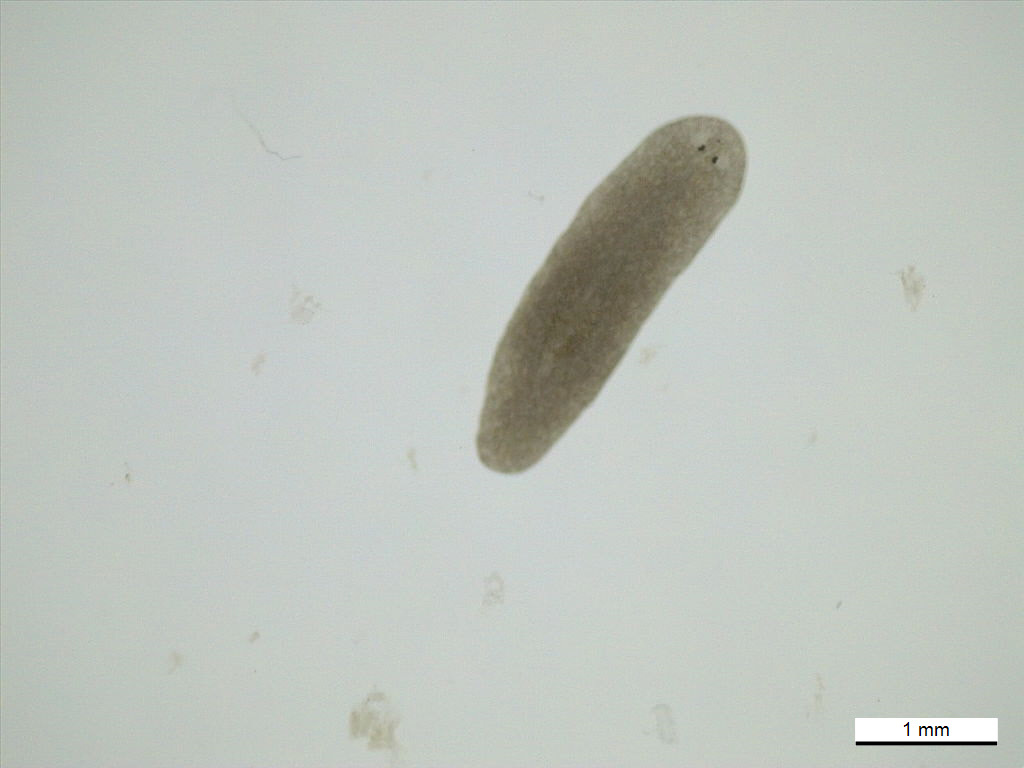

Supplement: Supplementary file 10 — Source data Fig. 3 [file 44318_2025_662_MOESM10_ESM.zip › Figure 3/3C-D/ythdf-a_RNAi_After_10_RNAi_feedings/ythdf-a_RNAi_After_10_RNAi_feedings_05.jpg]

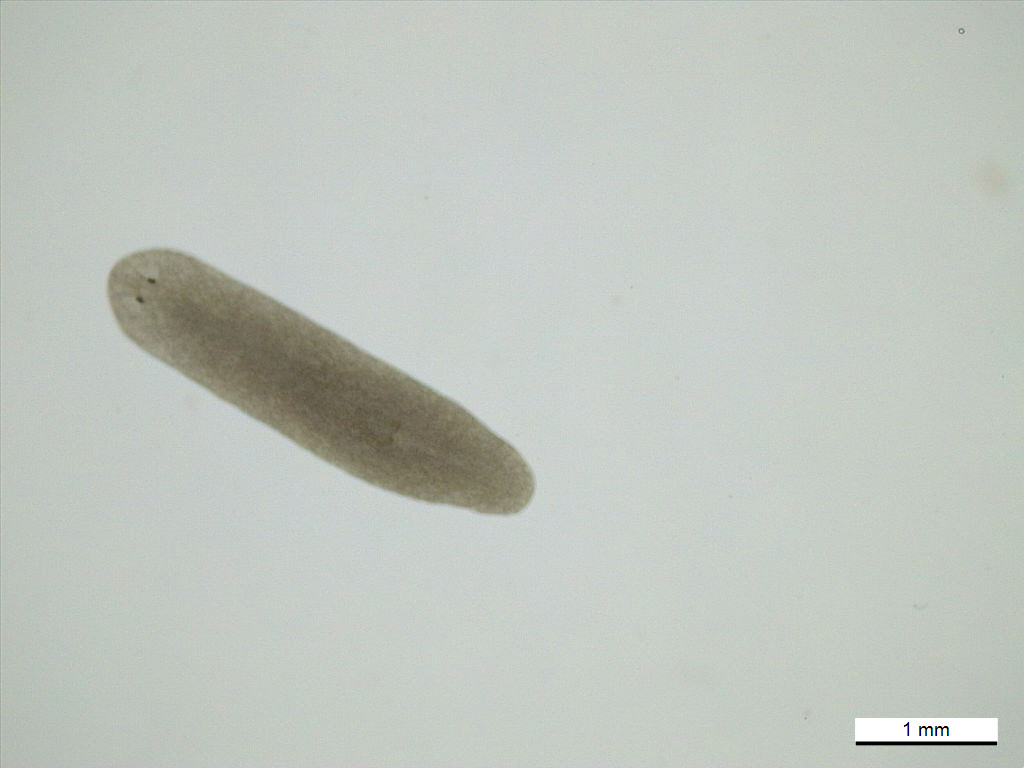

Supplement: Supplementary file 10 — Source data Fig. 3 [file 44318_2025_662_MOESM10_ESM.zip › Figure 3/3C-D/ythdf-a_RNAi_After_10_RNAi_feedings/ythdf-a_RNAi_After_10_RNAi_feedings_06.jpg]

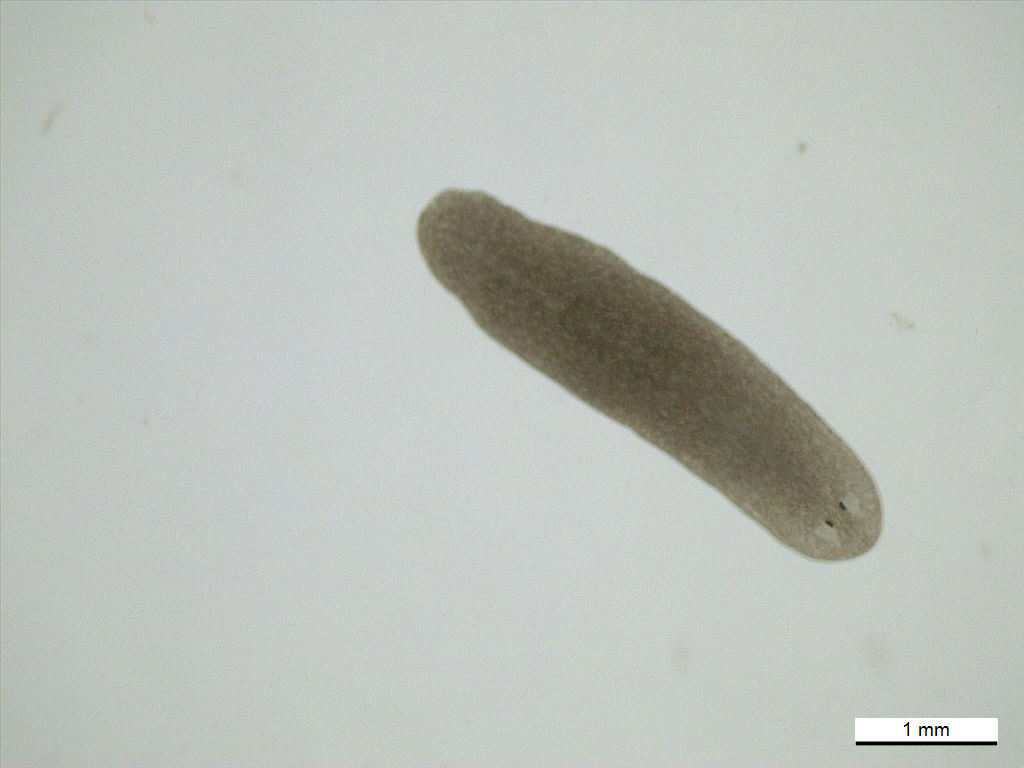

Supplement: Supplementary file 10 — Source data Fig. 3 [file 44318_2025_662_MOESM10_ESM.zip › Figure 3/3C-D/ythdf-a_RNAi_After_10_RNAi_feedings/ythdf-a_RNAi_After_10_RNAi_feedings_07.jpg]

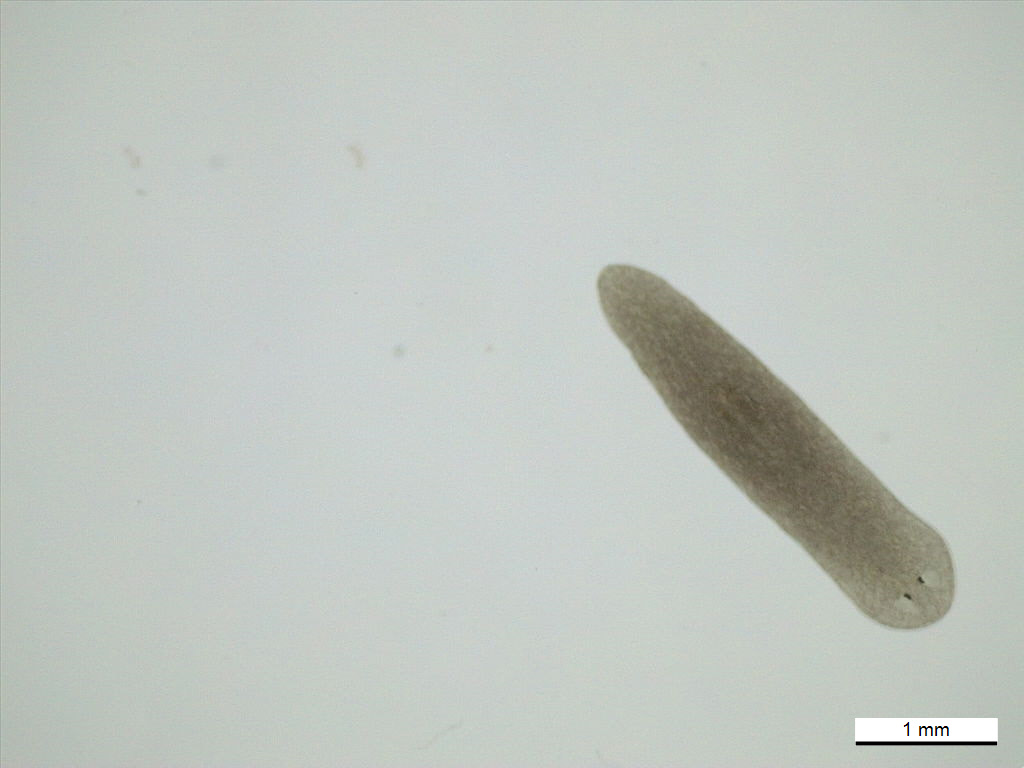

Supplement: Supplementary file 10 — Source data Fig. 3 [file 44318_2025_662_MOESM10_ESM.zip › Figure 3/3C-D/ythdf-a_RNAi_After_10_RNAi_feedings/ythdf-a_RNAi_After_10_RNAi_feedings_08.jpg]

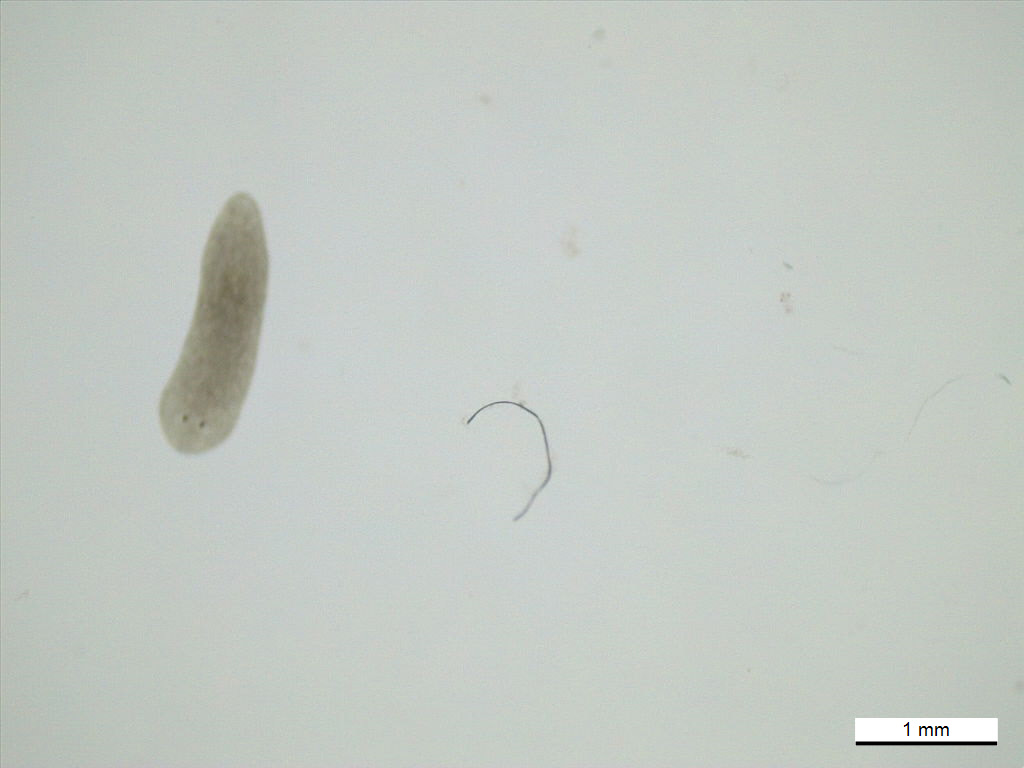

Supplement: Supplementary file 10 — Source data Fig. 3 [file 44318_2025_662_MOESM10_ESM.zip › Figure 3/3C-D/ythdf-a_RNAi_After_10_RNAi_feedings/ythdf-a_RNAi_After_10_RNAi_feedings_09.jpg]

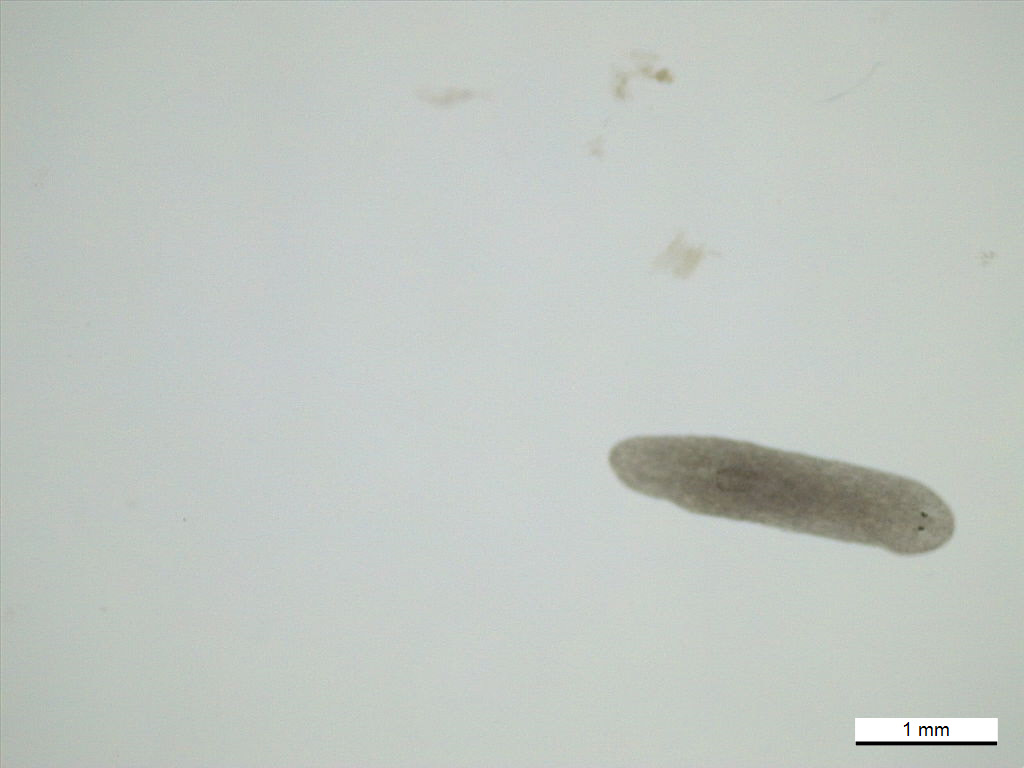

Supplement: Supplementary file 10 — Source data Fig. 3 [file 44318_2025_662_MOESM10_ESM.zip › Figure 3/3C-D/ythdf-a_RNAi_After_10_RNAi_feedings/ythdf-a_RNAi_After_10_RNAi_feedings_10.jpg]

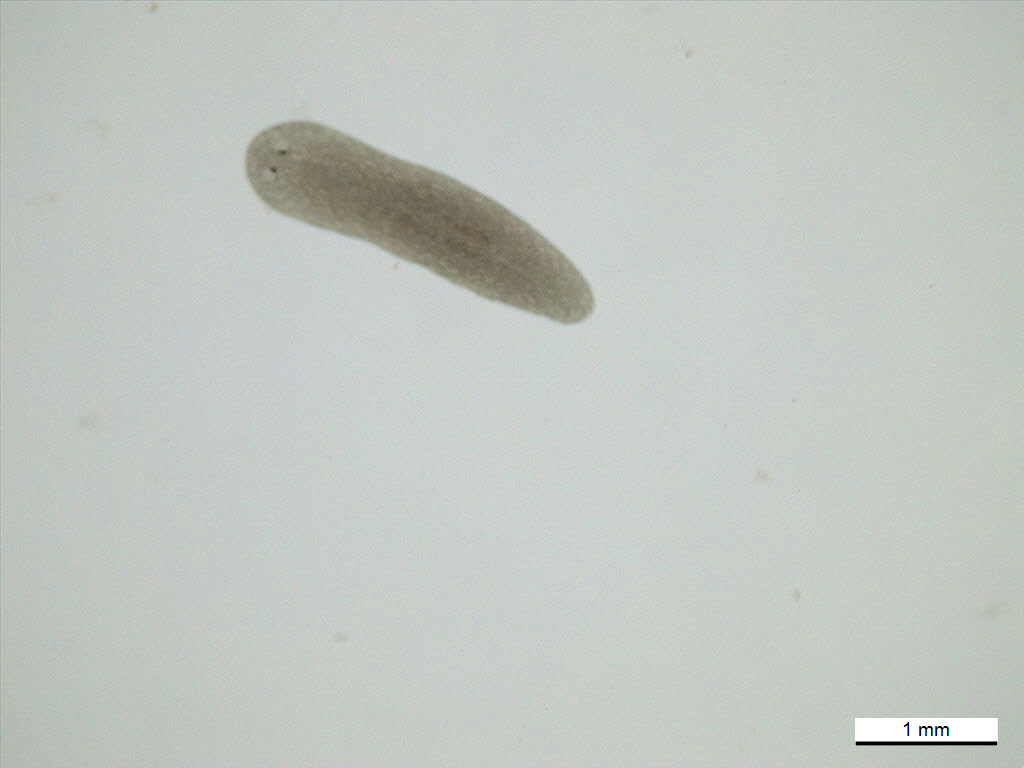

Supplement: Supplementary file 10 — Source data Fig. 3 [file 44318_2025_662_MOESM10_ESM.zip › Figure 3/3C-D/ythdf-a_RNAi_After_10_RNAi_feedings/ythdf-a_RNAi_After_10_RNAi_feedings_11.jpg]

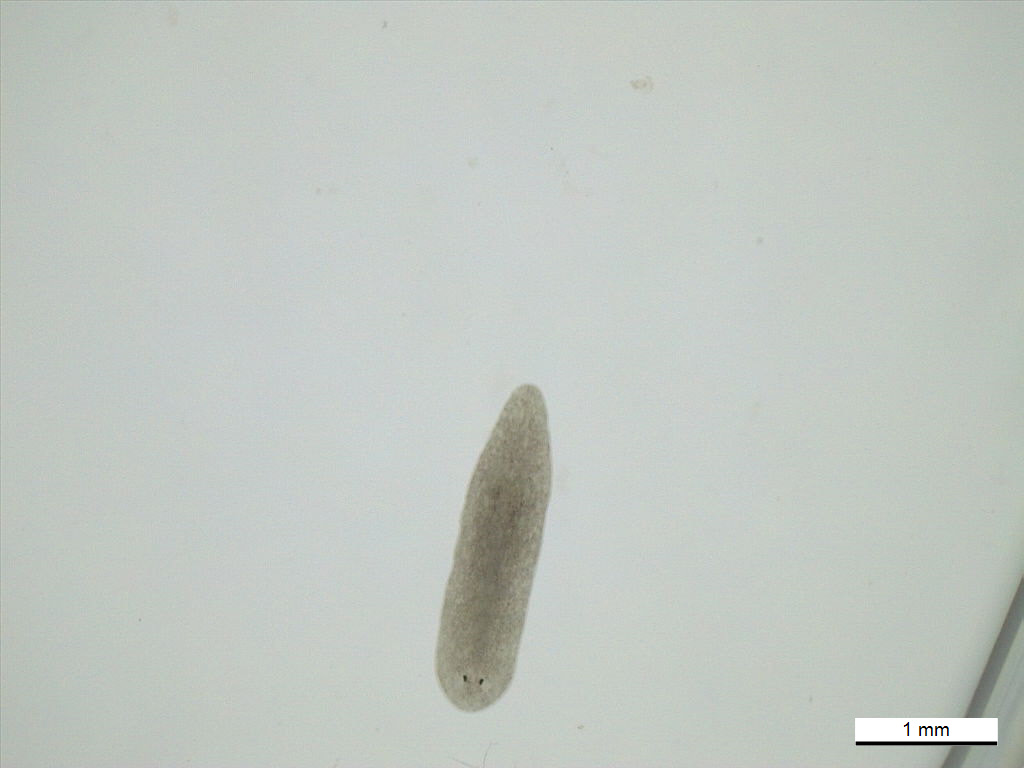

Supplement: Supplementary file 10 — Source data Fig. 3 [file 44318_2025_662_MOESM10_ESM.zip › Figure 3/3C-D/ythdf-a_RNAi_After_10_RNAi_feedings/ythdf-a_RNAi_After_10_RNAi_feedings_12.jpg]

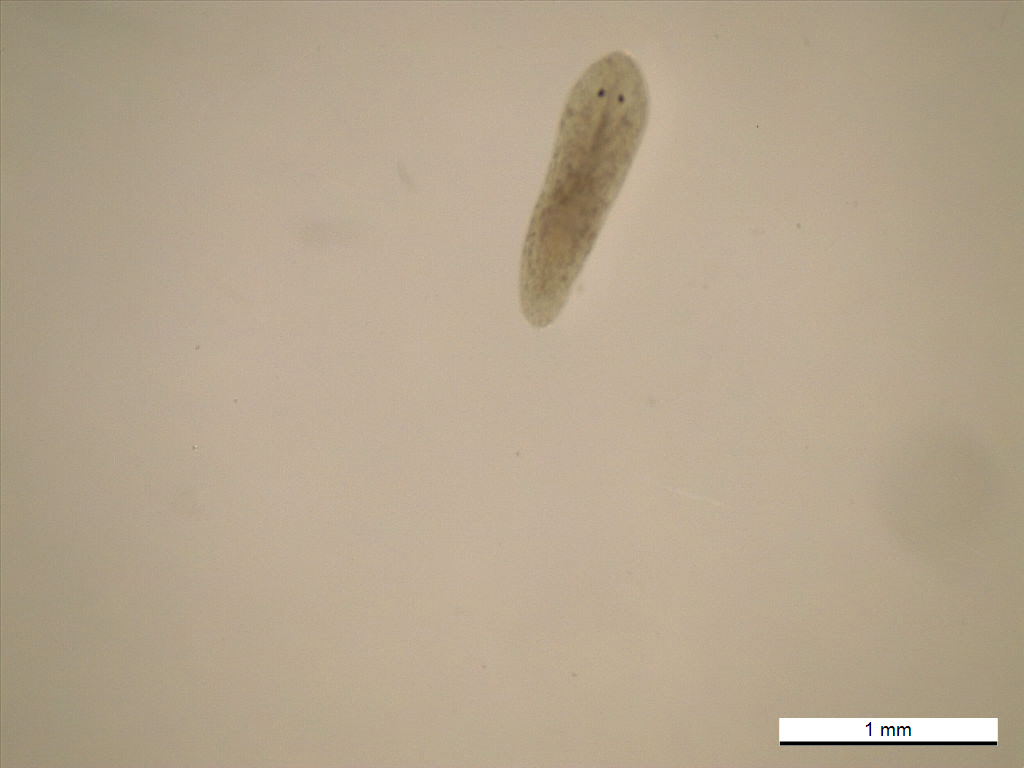

Supplement: Supplementary file 10 — Source data Fig. 3 [file 44318_2025_662_MOESM10_ESM.zip › Figure 3/3C-D/ythdf-a_RNAi_Before_RNAi_feedings/ythdf-a_RNAi_Before_RNAi_feedings_1.jpg]

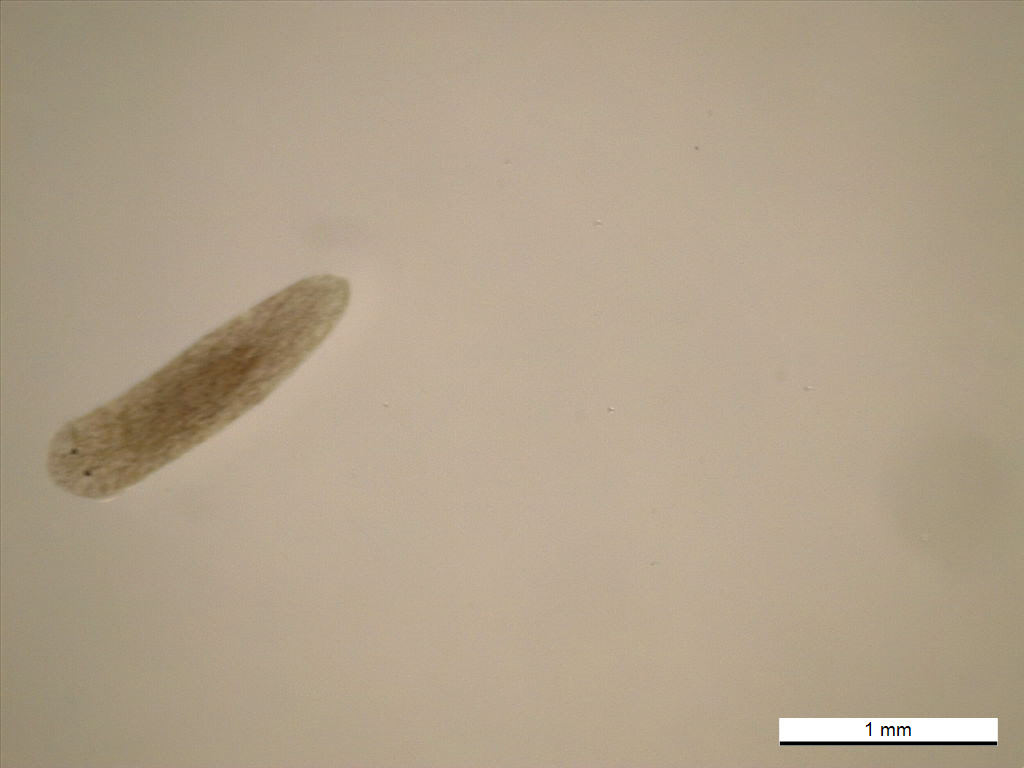

Supplement: Supplementary file 10 — Source data Fig. 3 [file 44318_2025_662_MOESM10_ESM.zip › Figure 3/3C-D/ythdf-a_RNAi_Before_RNAi_feedings/ythdf-a_RNAi_Before_RNAi_feedings_10.jpg]

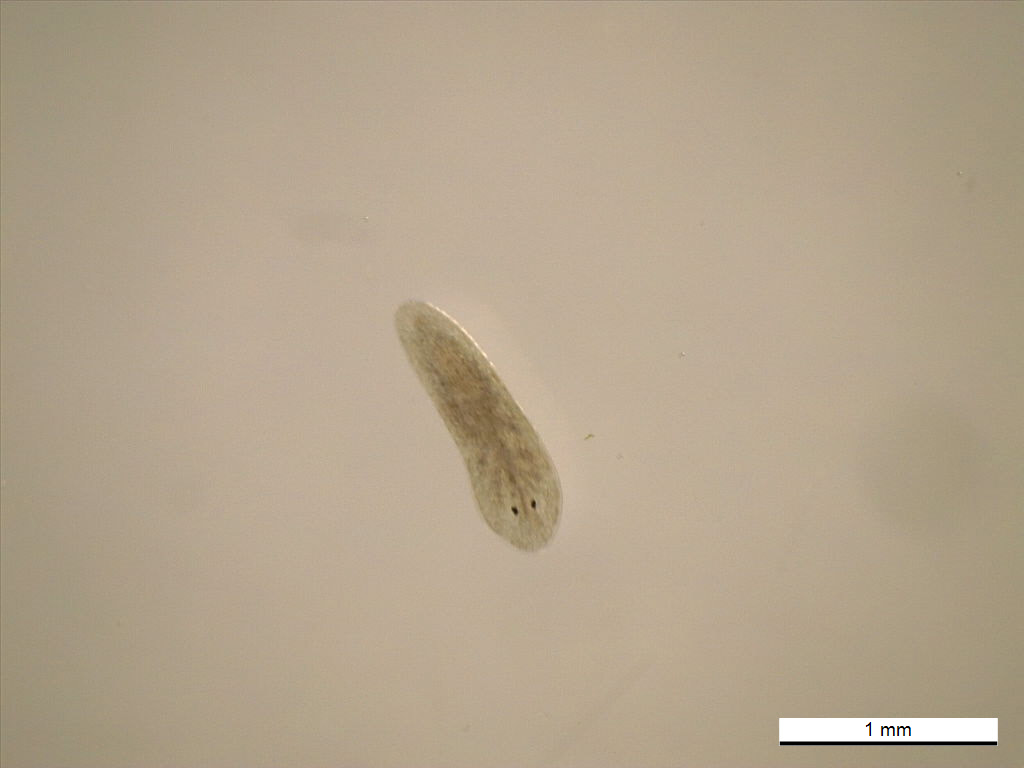

Supplement: Supplementary file 10 — Source data Fig. 3 [file 44318_2025_662_MOESM10_ESM.zip › Figure 3/3C-D/ythdf-a_RNAi_Before_RNAi_feedings/ythdf-a_RNAi_Before_RNAi_feedings_11.jpg]

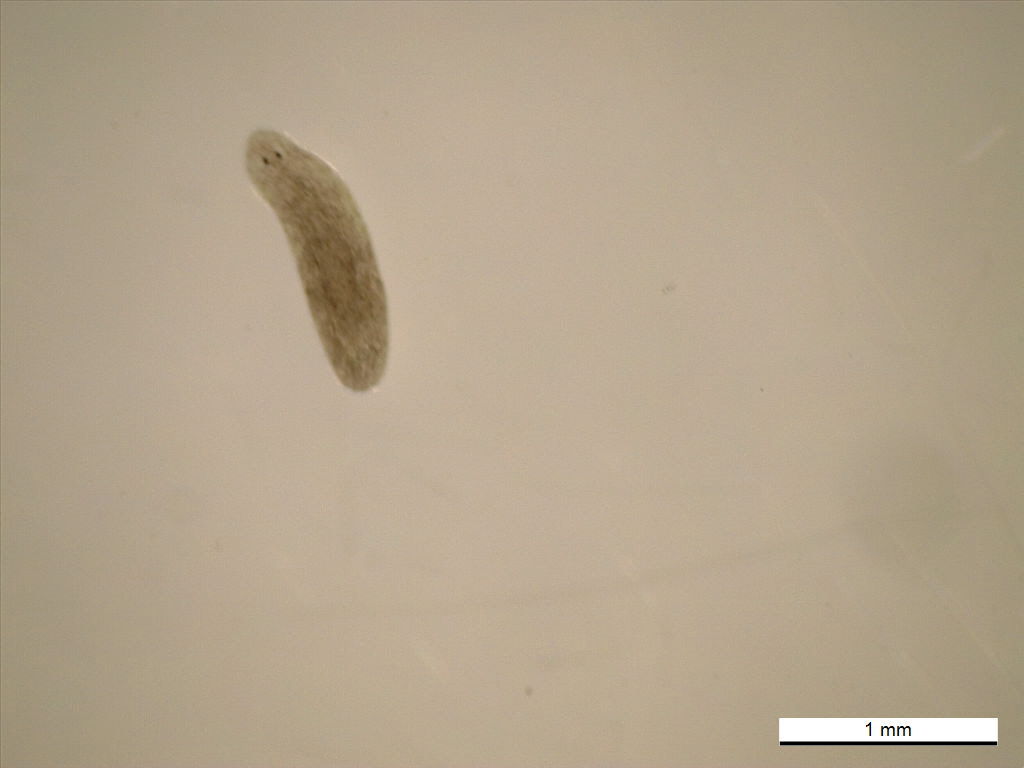

Supplement: Supplementary file 10 — Source data Fig. 3 [file 44318_2025_662_MOESM10_ESM.zip › Figure 3/3C-D/ythdf-a_RNAi_Before_RNAi_feedings/ythdf-a_RNAi_Before_RNAi_feedings_12.jpg]

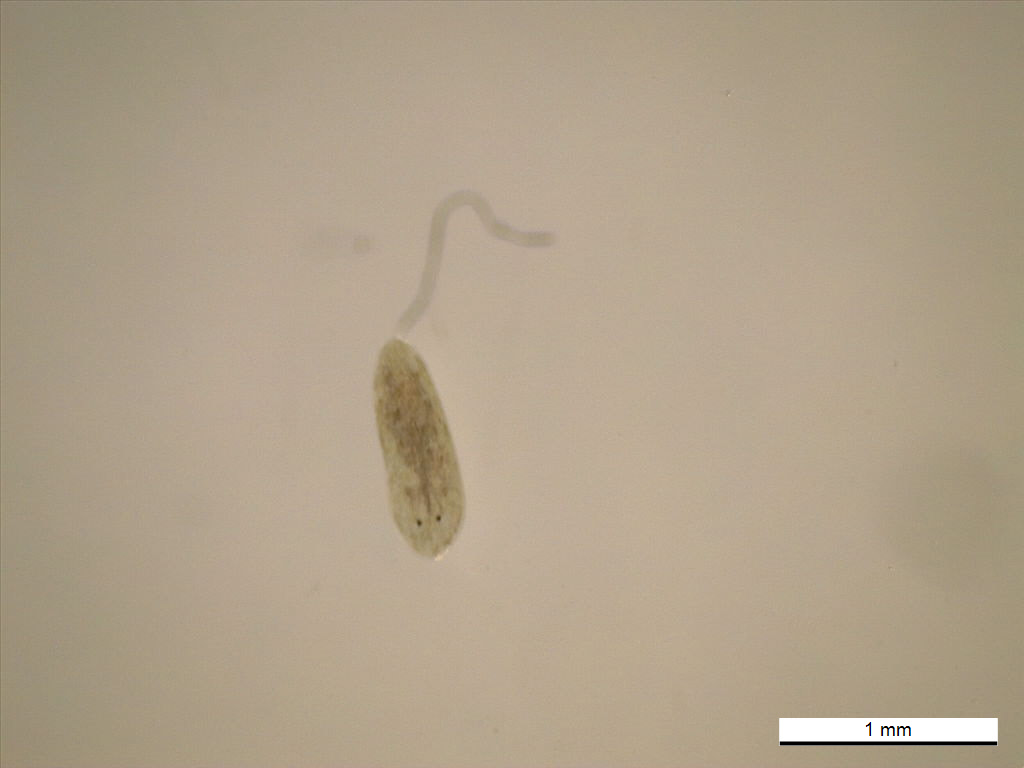

Supplement: Supplementary file 10 — Source data Fig. 3 [file 44318_2025_662_MOESM10_ESM.zip › Figure 3/3C-D/ythdf-a_RNAi_Before_RNAi_feedings/ythdf-a_RNAi_Before_RNAi_feedings_13.jpg]

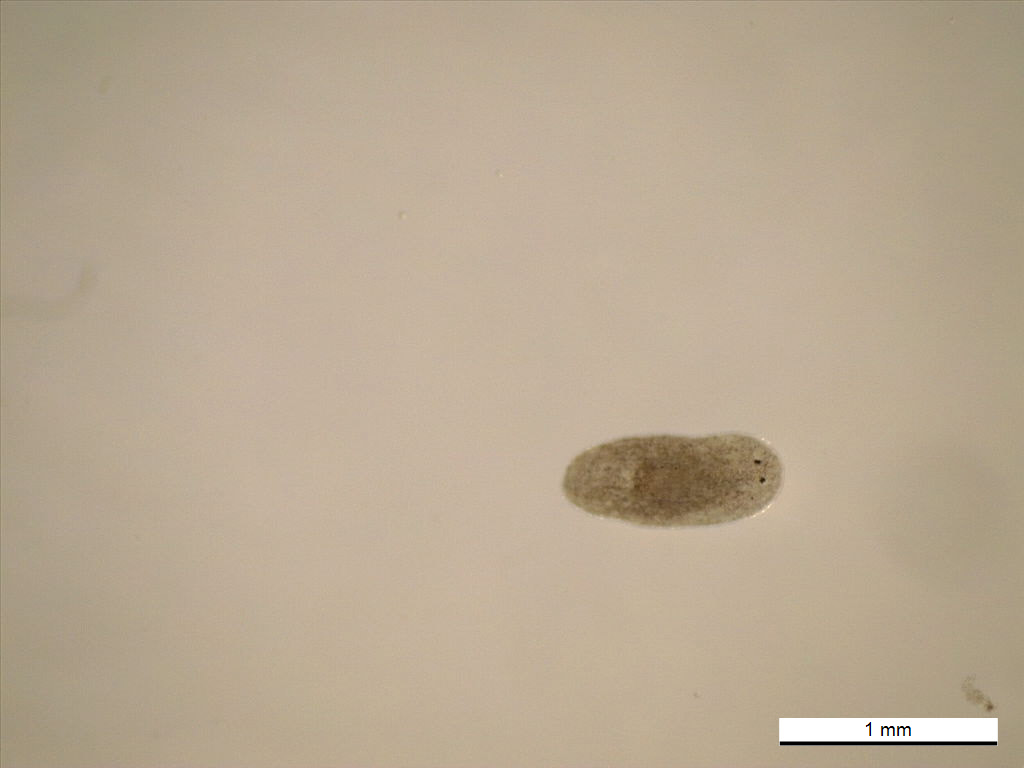

Supplement: Supplementary file 10 — Source data Fig. 3 [file 44318_2025_662_MOESM10_ESM.zip › Figure 3/3C-D/ythdf-a_RNAi_Before_RNAi_feedings/ythdf-a_RNAi_Before_RNAi_feedings_14.jpg]

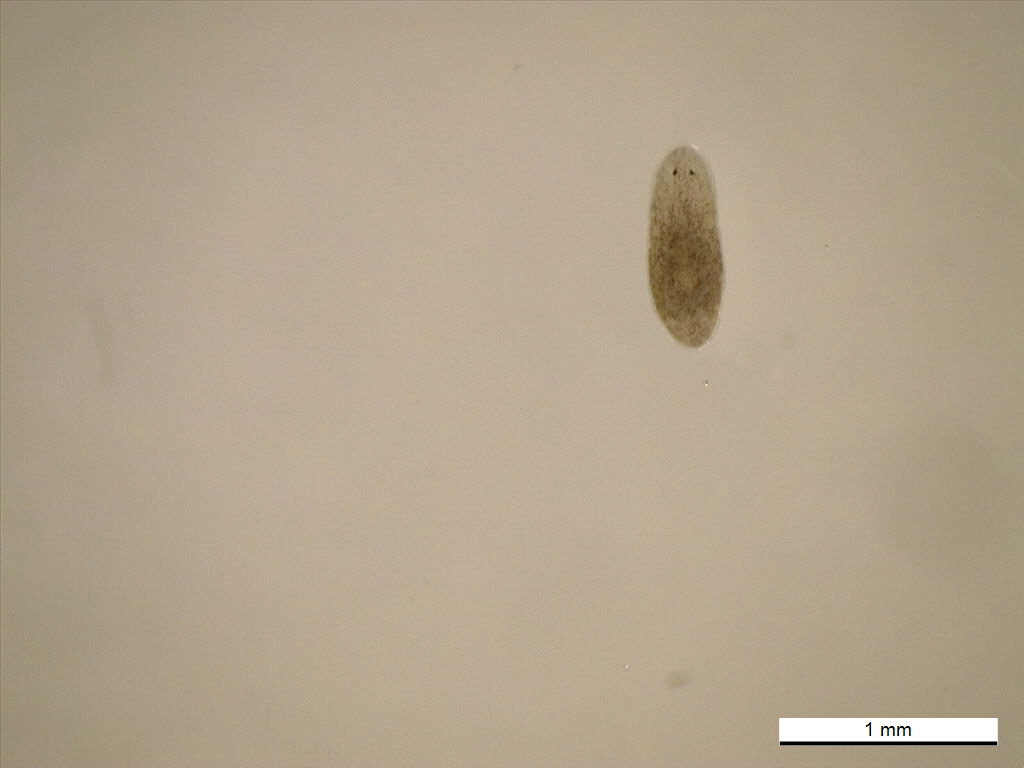

Supplement: Supplementary file 10 — Source data Fig. 3 [file 44318_2025_662_MOESM10_ESM.zip › Figure 3/3C-D/ythdf-a_RNAi_Before_RNAi_feedings/ythdf-a_RNAi_Before_RNAi_feedings_15.jpg]

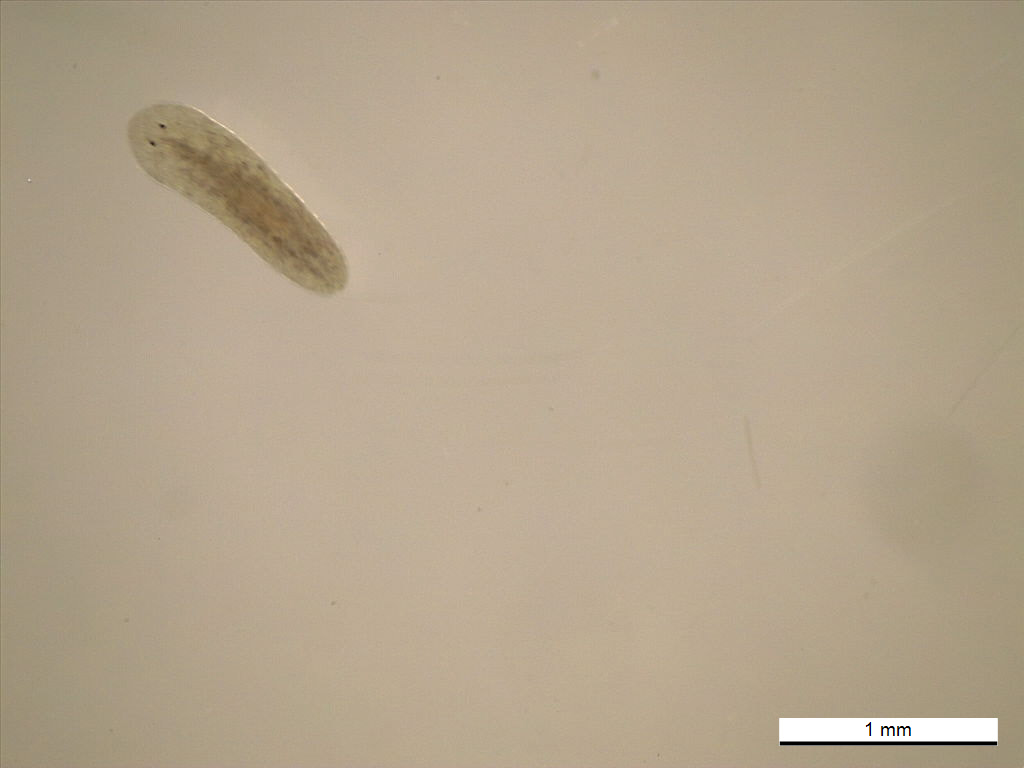

Supplement: Supplementary file 10 — Source data Fig. 3 [file 44318_2025_662_MOESM10_ESM.zip › Figure 3/3C-D/ythdf-a_RNAi_Before_RNAi_feedings/ythdf-a_RNAi_Before_RNAi_feedings_2.jpg]

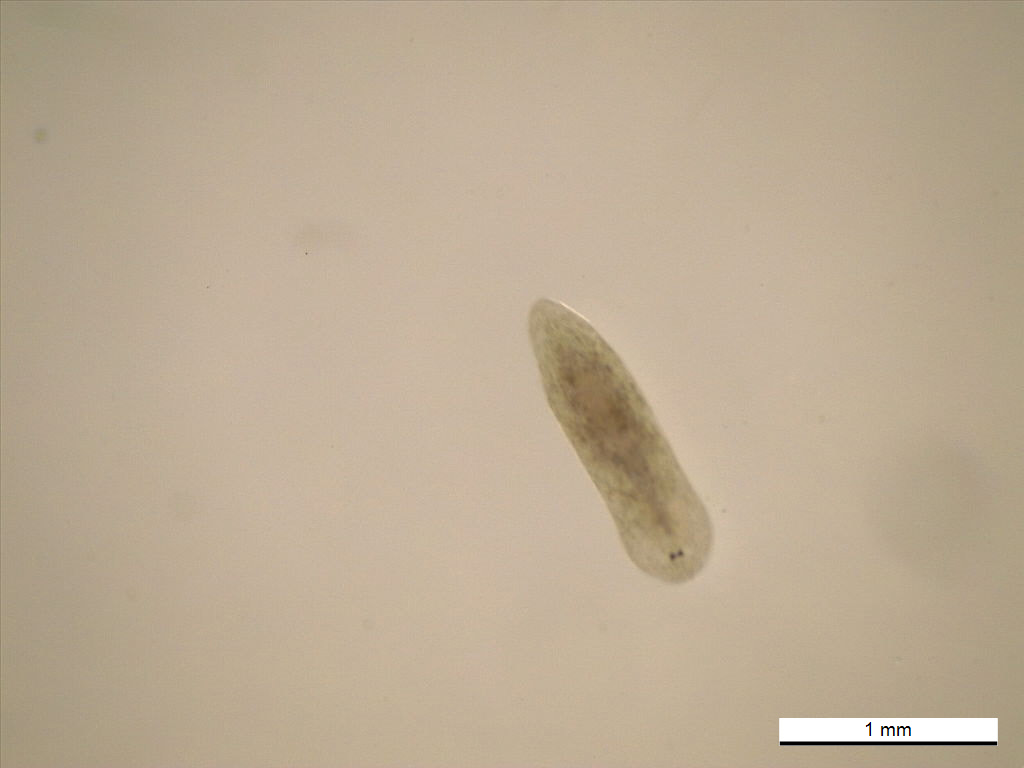

Supplement: Supplementary file 10 — Source data Fig. 3 [file 44318_2025_662_MOESM10_ESM.zip › Figure 3/3C-D/ythdf-a_RNAi_Before_RNAi_feedings/ythdf-a_RNAi_Before_RNAi_feedings_3.jpg]

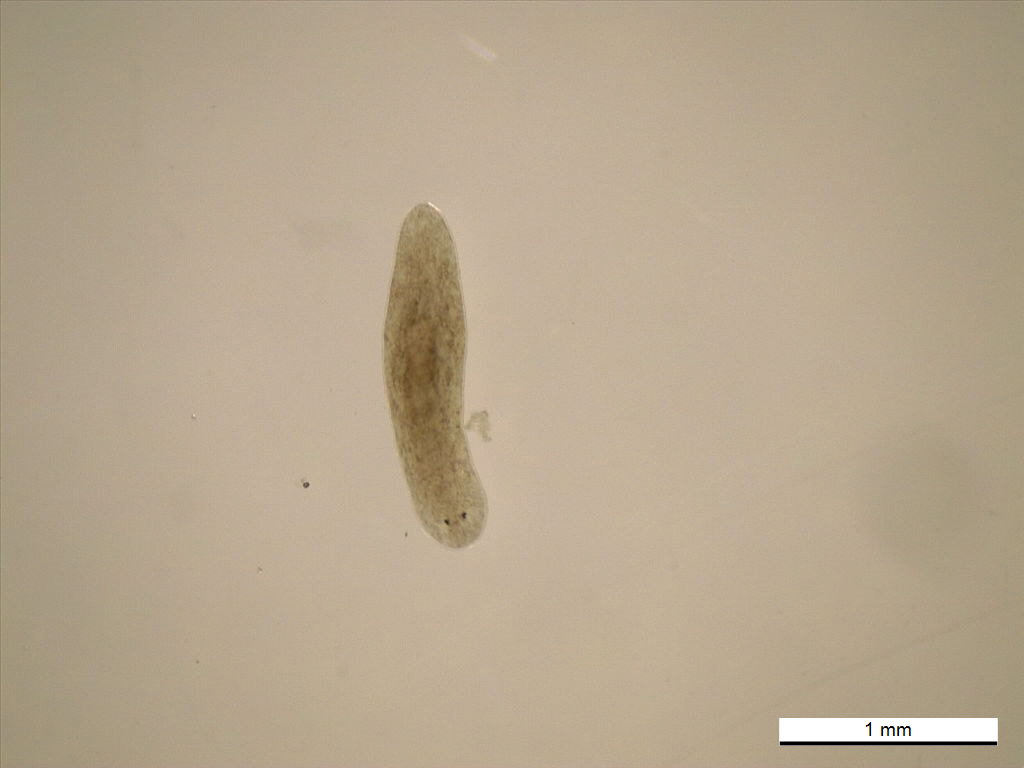

Supplement: Supplementary file 10 — Source data Fig. 3 [file 44318_2025_662_MOESM10_ESM.zip › Figure 3/3C-D/ythdf-a_RNAi_Before_RNAi_feedings/ythdf-a_RNAi_Before_RNAi_feedings_4.jpg]

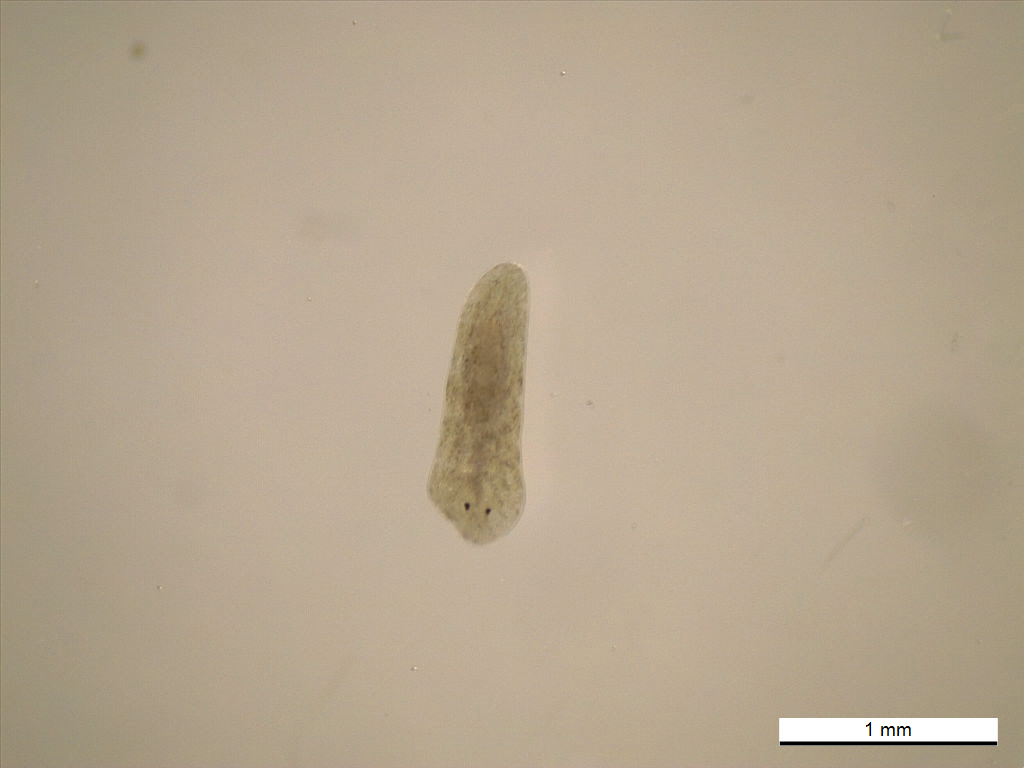

Supplement: Supplementary file 10 — Source data Fig. 3 [file 44318_2025_662_MOESM10_ESM.zip › Figure 3/3C-D/ythdf-a_RNAi_Before_RNAi_feedings/ythdf-a_RNAi_Before_RNAi_feedings_5.jpg]

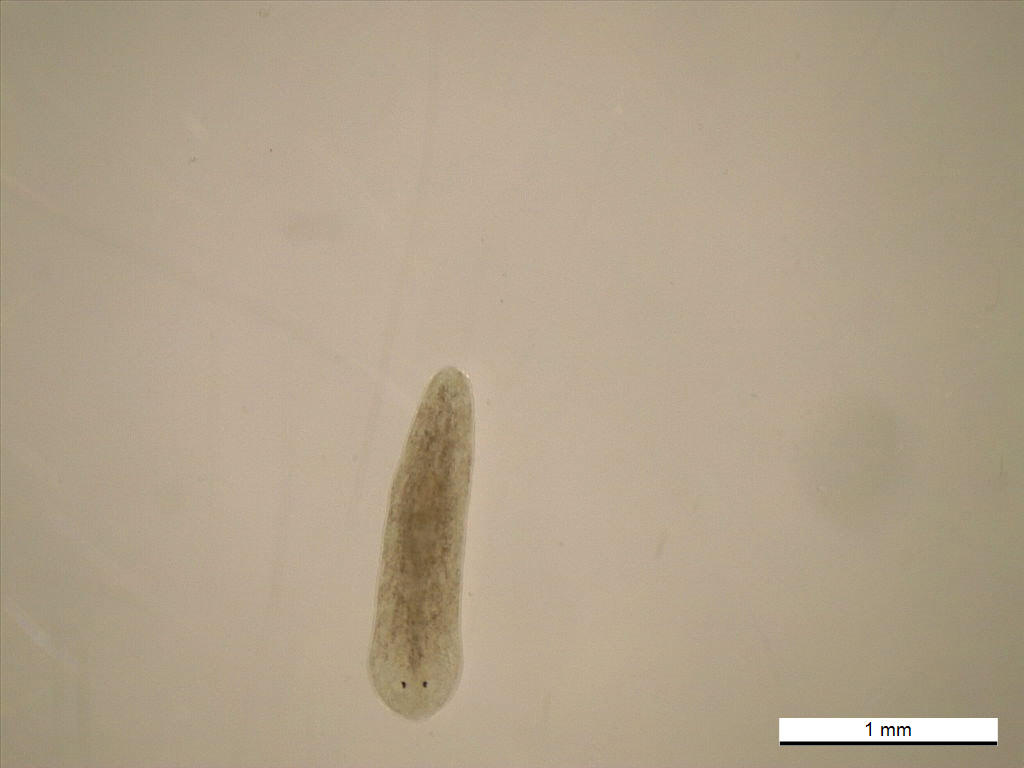

Supplement: Supplementary file 10 — Source data Fig. 3 [file 44318_2025_662_MOESM10_ESM.zip › Figure 3/3C-D/ythdf-a_RNAi_Before_RNAi_feedings/ythdf-a_RNAi_Before_RNAi_feedings_6.jpg]

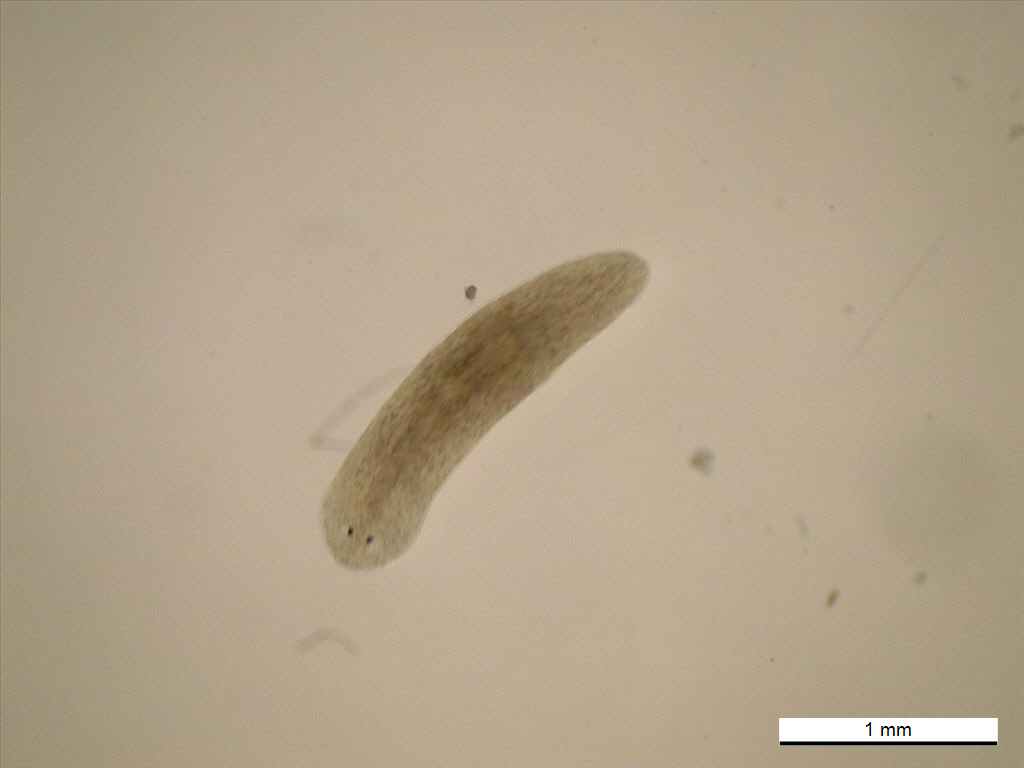

Supplement: Supplementary file 10 — Source data Fig. 3 [file 44318_2025_662_MOESM10_ESM.zip › Figure 3/3C-D/ythdf-a_RNAi_Before_RNAi_feedings/ythdf-a_RNAi_Before_RNAi_feedings_7.jpg]

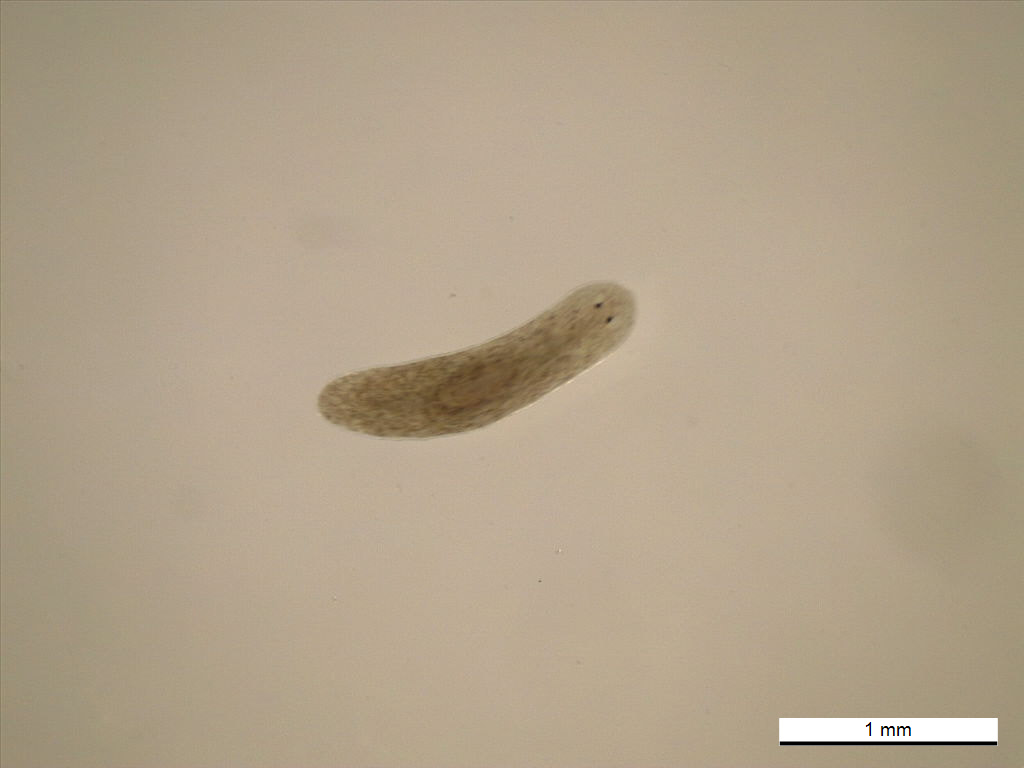

Supplement: Supplementary file 10 — Source data Fig. 3 [file 44318_2025_662_MOESM10_ESM.zip › Figure 3/3C-D/ythdf-a_RNAi_Before_RNAi_feedings/ythdf-a_RNAi_Before_RNAi_feedings_8.jpg]

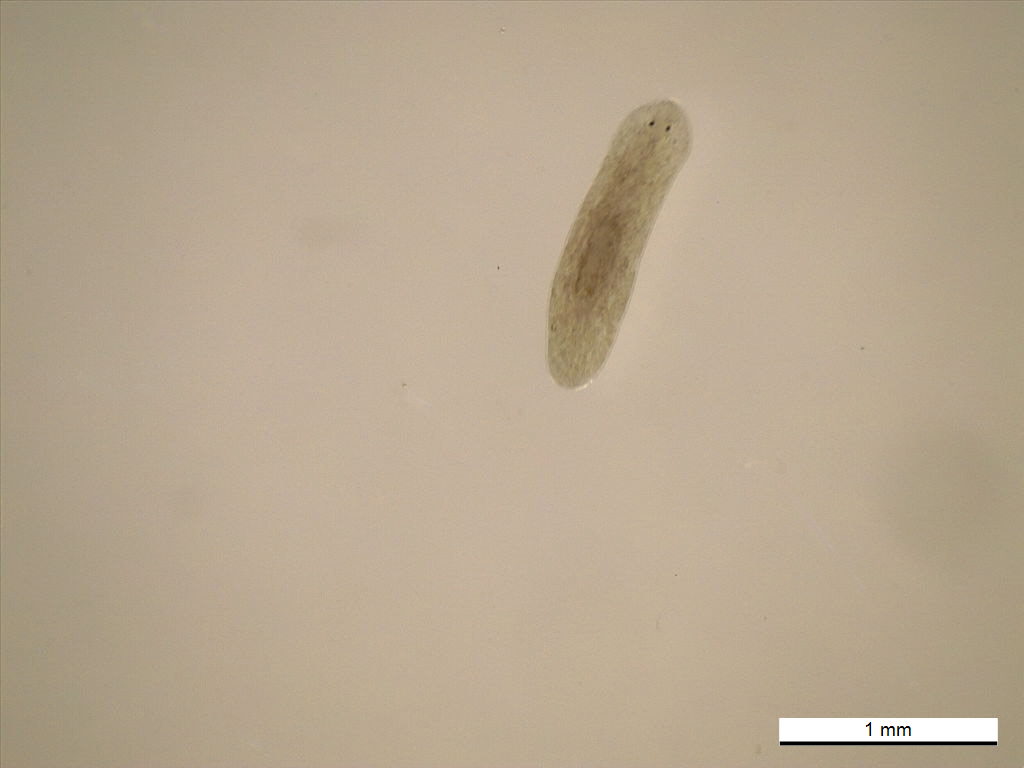

Supplement: Supplementary file 10 — Source data Fig. 3 [file 44318_2025_662_MOESM10_ESM.zip › Figure 3/3C-D/ythdf-a_RNAi_Before_RNAi_feedings/ythdf-a_RNAi_Before_RNAi_feedings_9.jpg]

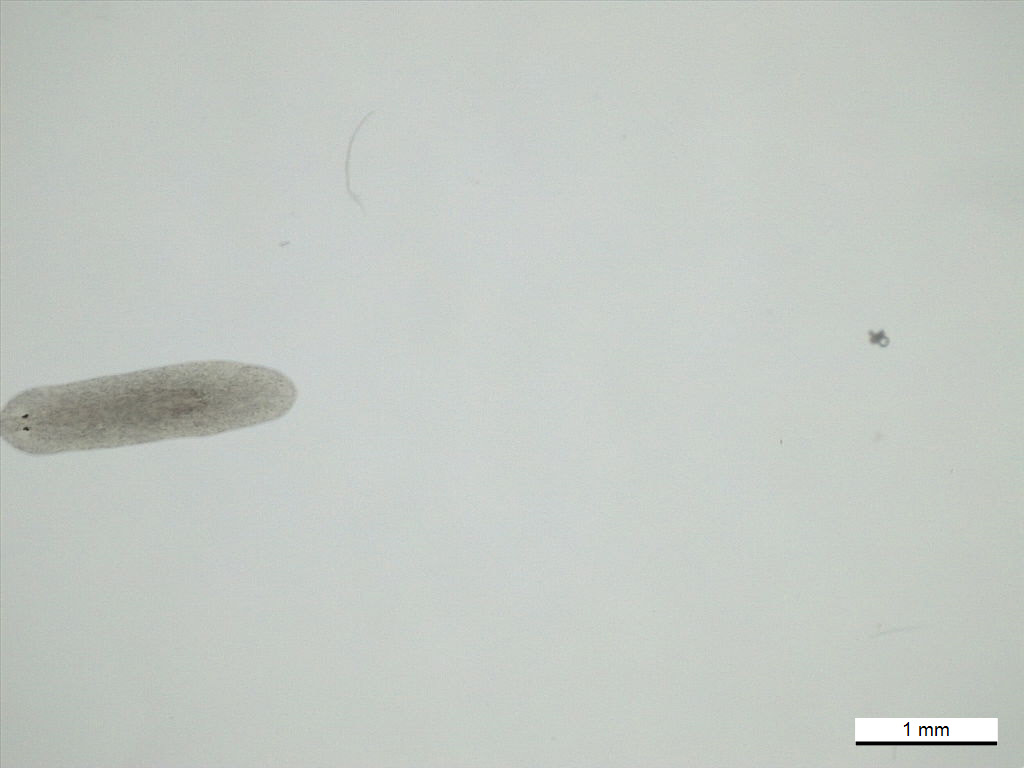

Supplement: Supplementary file 10 — Source data Fig. 3 [file 44318_2025_662_MOESM10_ESM.zip › Figure 3/3C-D/ythdf-b-c_RNAi_After_10_RNAi_feedings/ythdf_b-c_RNAi_After_10_RNAi_feedings_01.jpg]

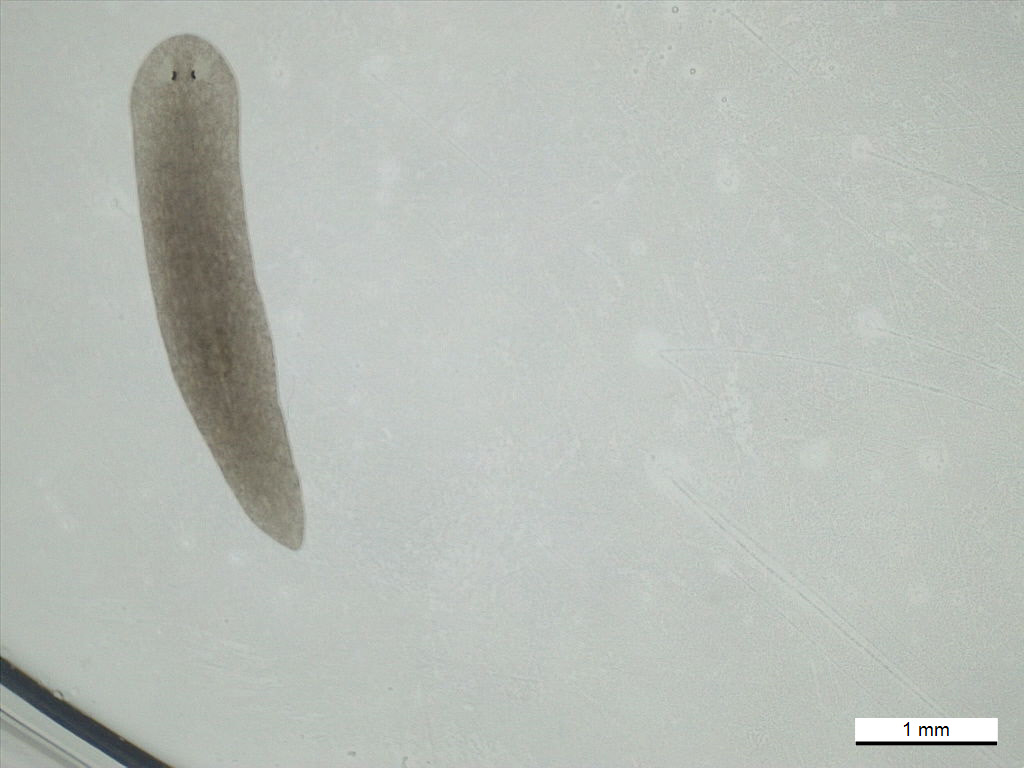

Supplement: Supplementary file 10 — Source data Fig. 3 [file 44318_2025_662_MOESM10_ESM.zip › Figure 3/3C-D/ythdf-b-c_RNAi_After_10_RNAi_feedings/ythdf_b-c_RNAi_After_10_RNAi_feedings_02.jpg]

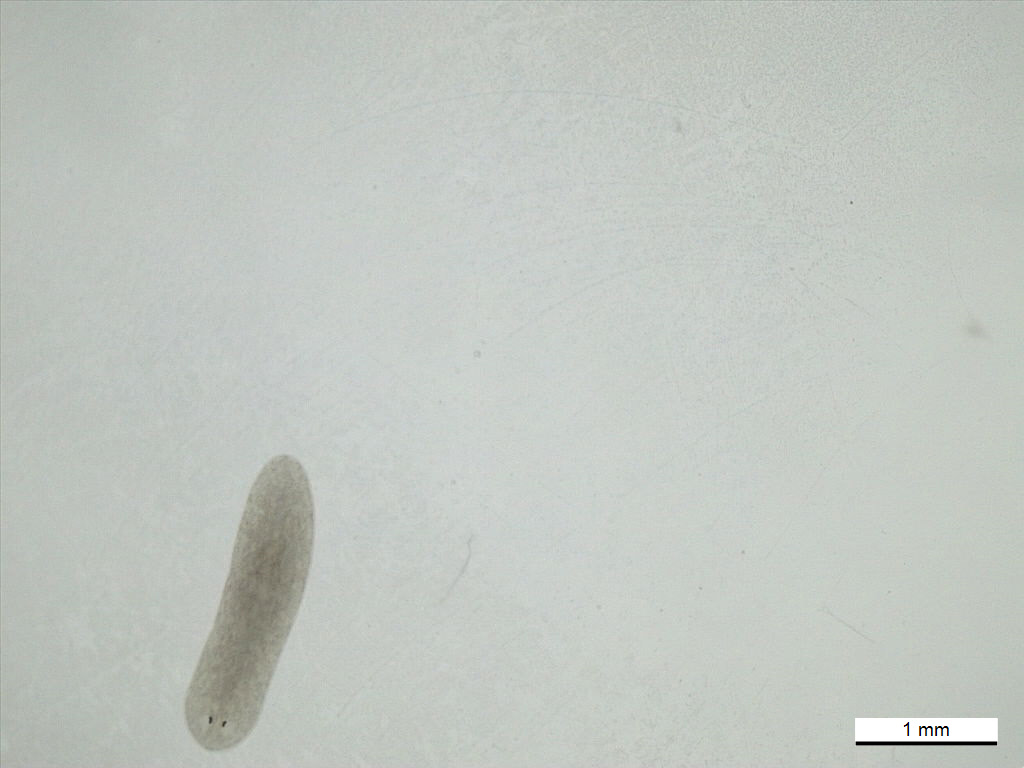

Supplement: Supplementary file 10 — Source data Fig. 3 [file 44318_2025_662_MOESM10_ESM.zip › Figure 3/3C-D/ythdf-b-c_RNAi_After_10_RNAi_feedings/ythdf_b-c_RNAi_After_10_RNAi_feedings_03.jpg]

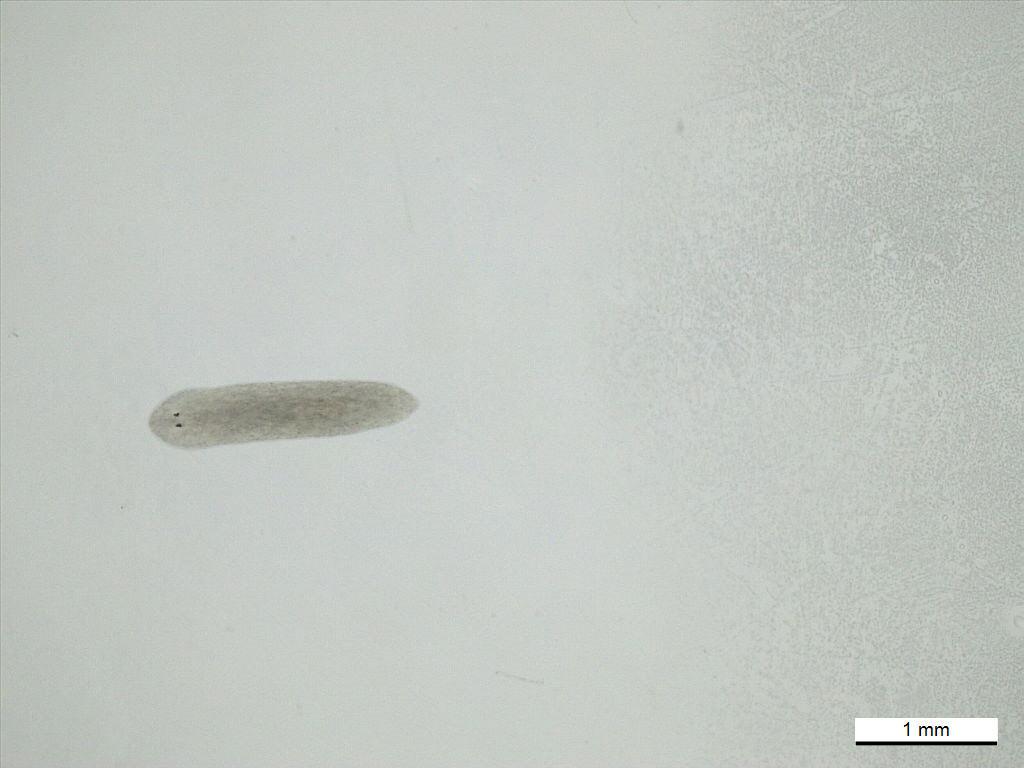

Supplement: Supplementary file 10 — Source data Fig. 3 [file 44318_2025_662_MOESM10_ESM.zip › Figure 3/3C-D/ythdf-b-c_RNAi_After_10_RNAi_feedings/ythdf_b-c_RNAi_After_10_RNAi_feedings_04.jpg]

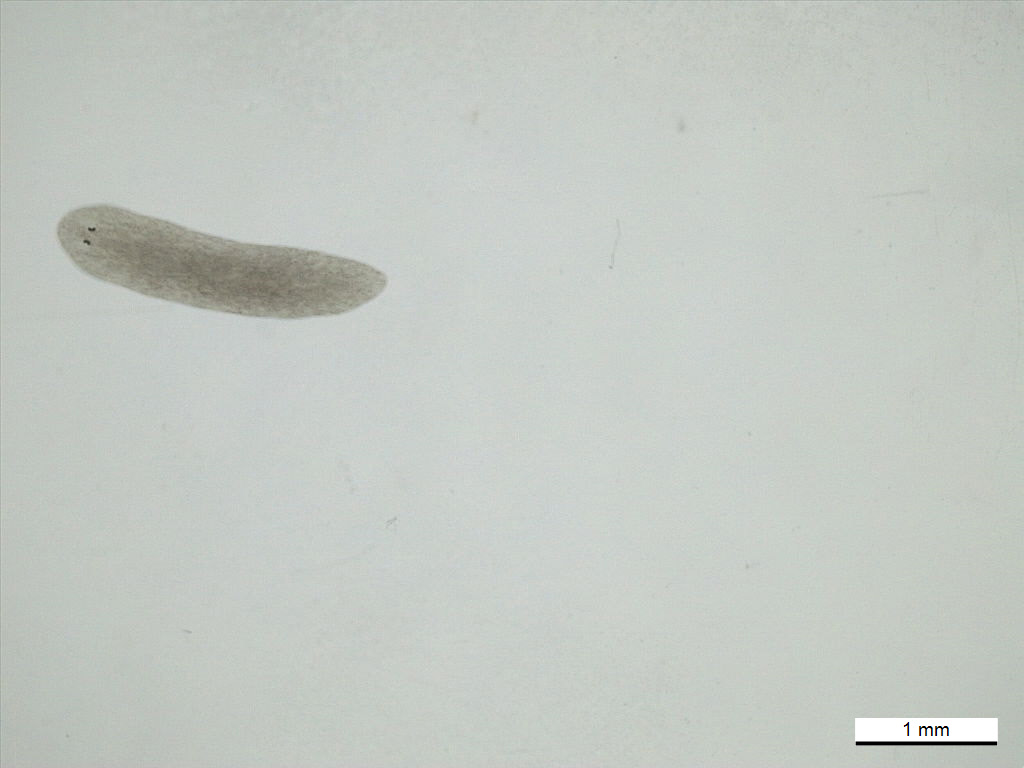

Supplement: Supplementary file 10 — Source data Fig. 3 [file 44318_2025_662_MOESM10_ESM.zip › Figure 3/3C-D/ythdf-b-c_RNAi_After_10_RNAi_feedings/ythdf_b-c_RNAi_After_10_RNAi_feedings_05.jpg]

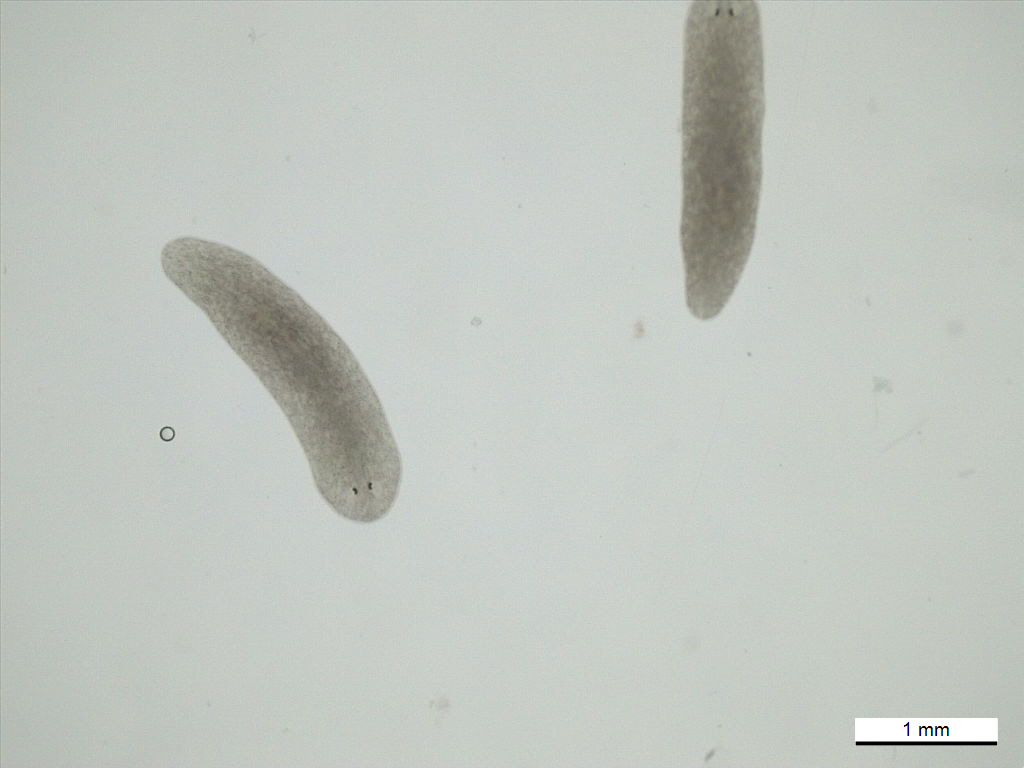

Supplement: Supplementary file 10 — Source data Fig. 3 [file 44318_2025_662_MOESM10_ESM.zip › Figure 3/3C-D/ythdf-b-c_RNAi_After_10_RNAi_feedings/ythdf_b-c_RNAi_After_10_RNAi_feedings_06-07.jpg]

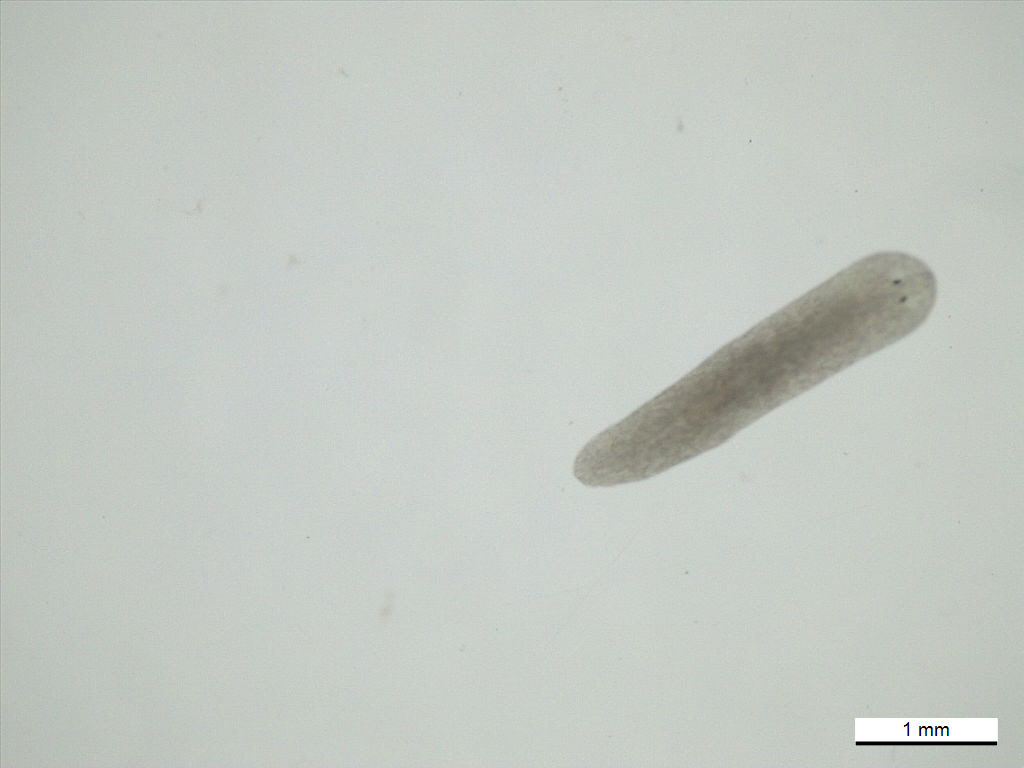

Supplement: Supplementary file 10 — Source data Fig. 3 [file 44318_2025_662_MOESM10_ESM.zip › Figure 3/3C-D/ythdf-b-c_RNAi_After_10_RNAi_feedings/ythdf_b-c_RNAi_After_10_RNAi_feedings_08.jpg]

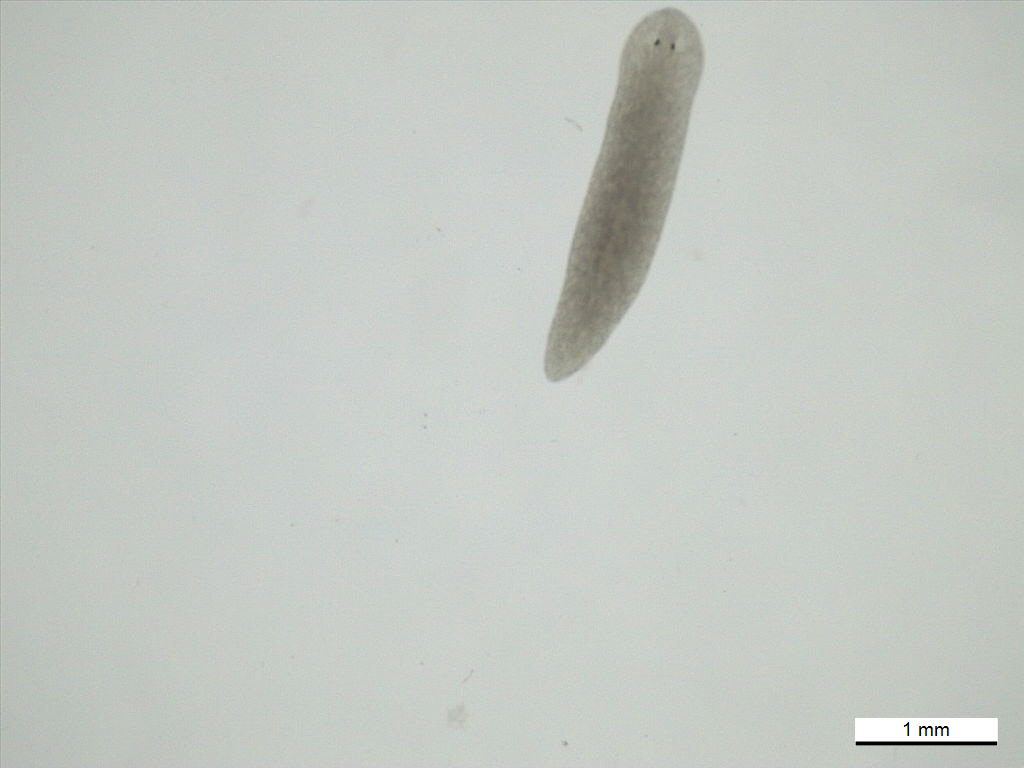

Supplement: Supplementary file 10 — Source data Fig. 3 [file 44318_2025_662_MOESM10_ESM.zip › Figure 3/3C-D/ythdf-b-c_RNAi_After_10_RNAi_feedings/ythdf_b-c_RNAi_After_10_RNAi_feedings_09.jpg]

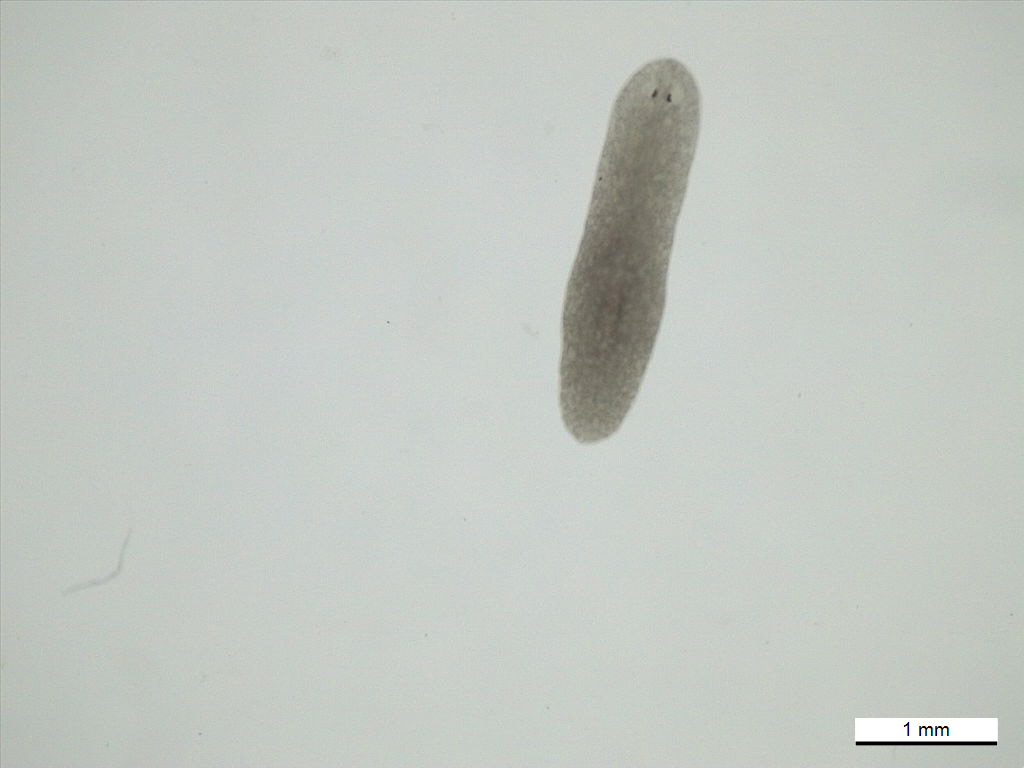

Supplement: Supplementary file 10 — Source data Fig. 3 [file 44318_2025_662_MOESM10_ESM.zip › Figure 3/3C-D/ythdf-b-c_RNAi_After_10_RNAi_feedings/ythdf_b-c_RNAi_After_10_RNAi_feedings_10.jpg]

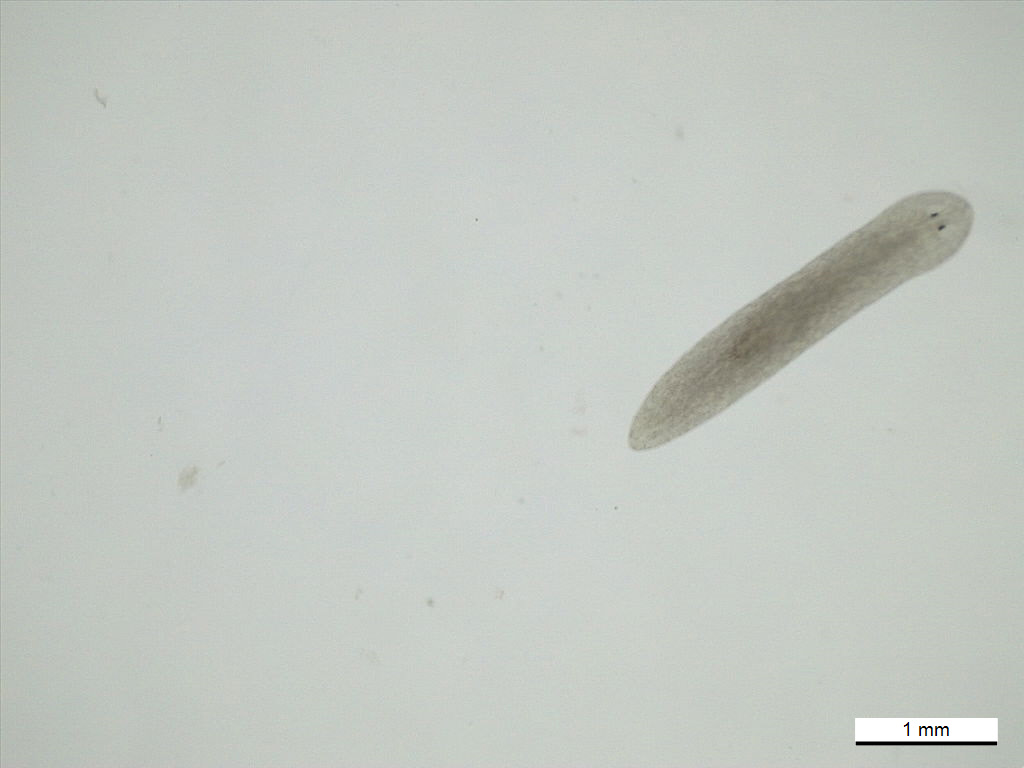

Supplement: Supplementary file 10 — Source data Fig. 3 [file 44318_2025_662_MOESM10_ESM.zip › Figure 3/3C-D/ythdf-b-c_RNAi_After_10_RNAi_feedings/ythdf_b-c_RNAi_After_10_RNAi_feedings_11.jpg]

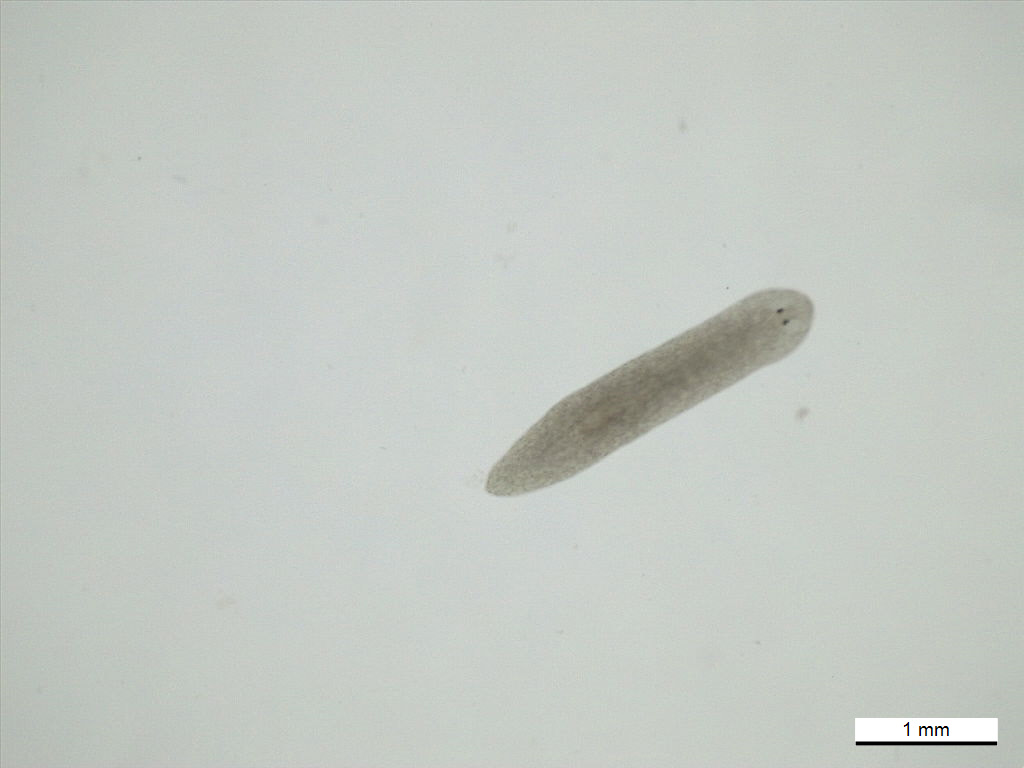

Supplement: Supplementary file 10 — Source data Fig. 3 [file 44318_2025_662_MOESM10_ESM.zip › Figure 3/3C-D/ythdf-b-c_RNAi_After_10_RNAi_feedings/ythdf_b-c_RNAi_After_10_RNAi_feedings_12.jpg]

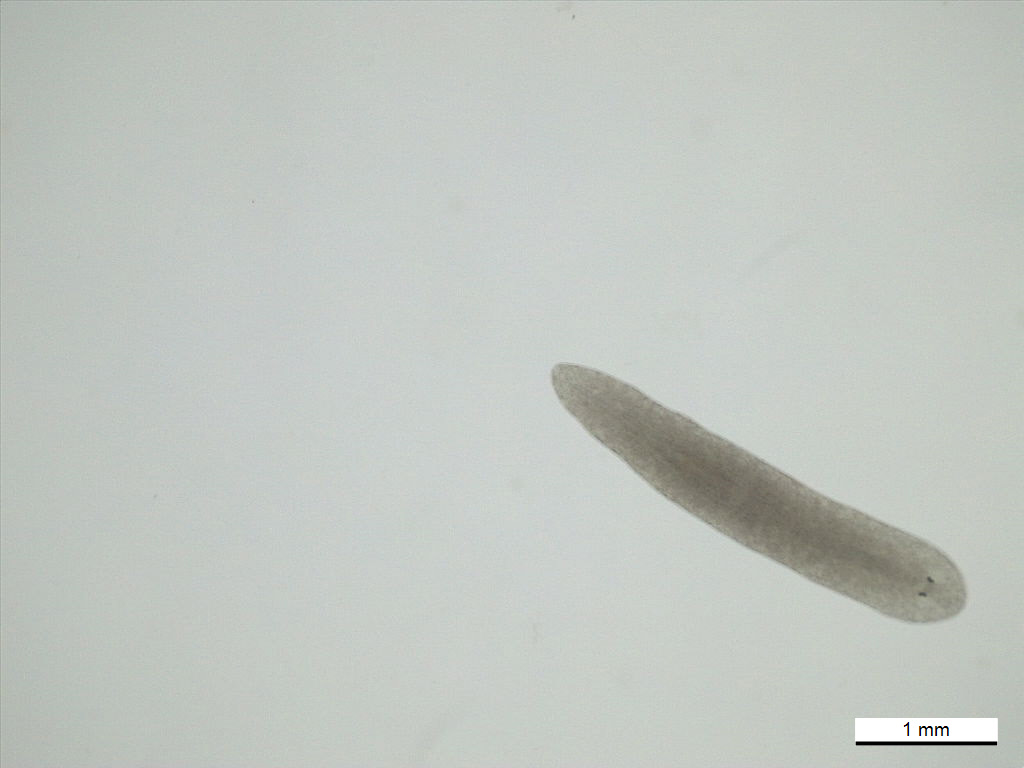

Supplement: Supplementary file 10 — Source data Fig. 3 [file 44318_2025_662_MOESM10_ESM.zip › Figure 3/3C-D/ythdf-b-c_RNAi_After_10_RNAi_feedings/ythdf_b-c_RNAi_After_10_RNAi_feedings_13.jpg]

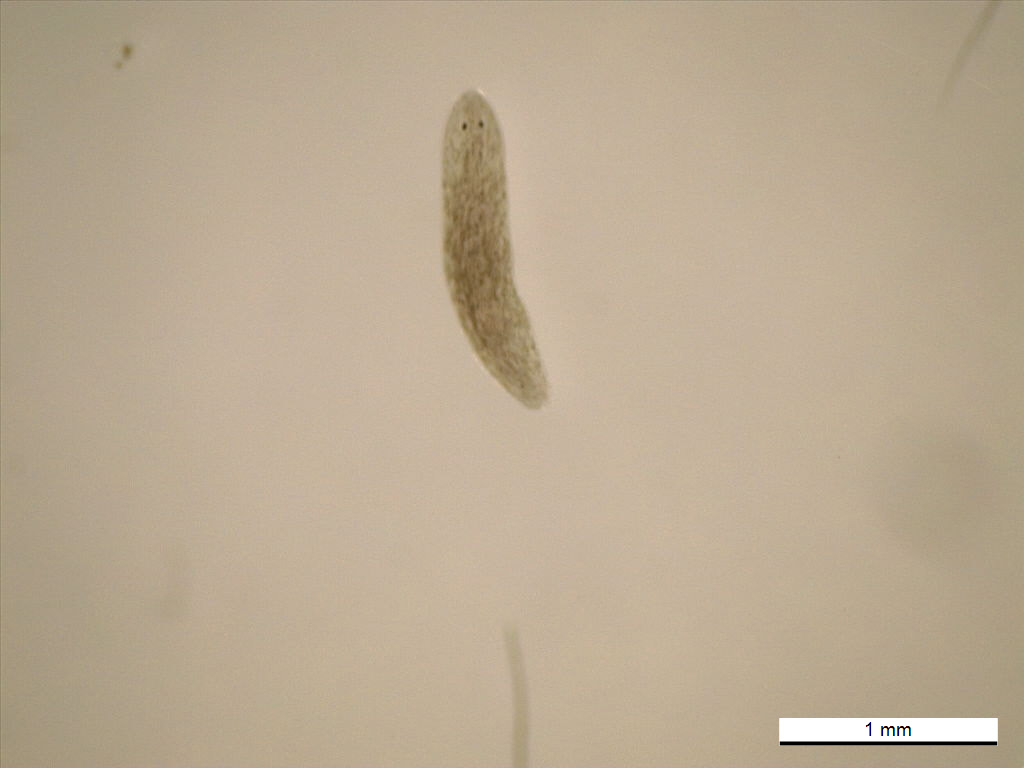

Supplement: Supplementary file 10 — Source data Fig. 3 [file 44318_2025_662_MOESM10_ESM.zip › Figure 3/3C-D/ythdf-b-c_RNAi_Before_RNAi_feedings/ythdf-b-c_RNAi_Before_RNAi_feedings_1.jpg]

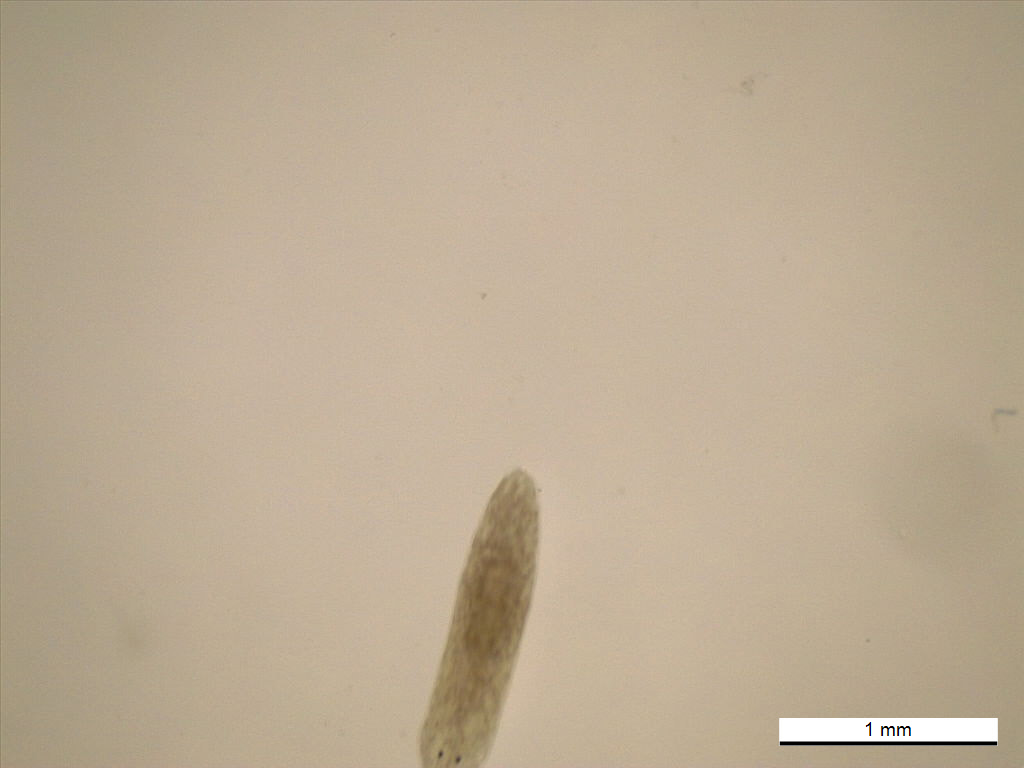

Supplement: Supplementary file 10 — Source data Fig. 3 [file 44318_2025_662_MOESM10_ESM.zip › Figure 3/3C-D/ythdf-b-c_RNAi_Before_RNAi_feedings/ythdf-b-c_RNAi_Before_RNAi_feedings_10.jpg]

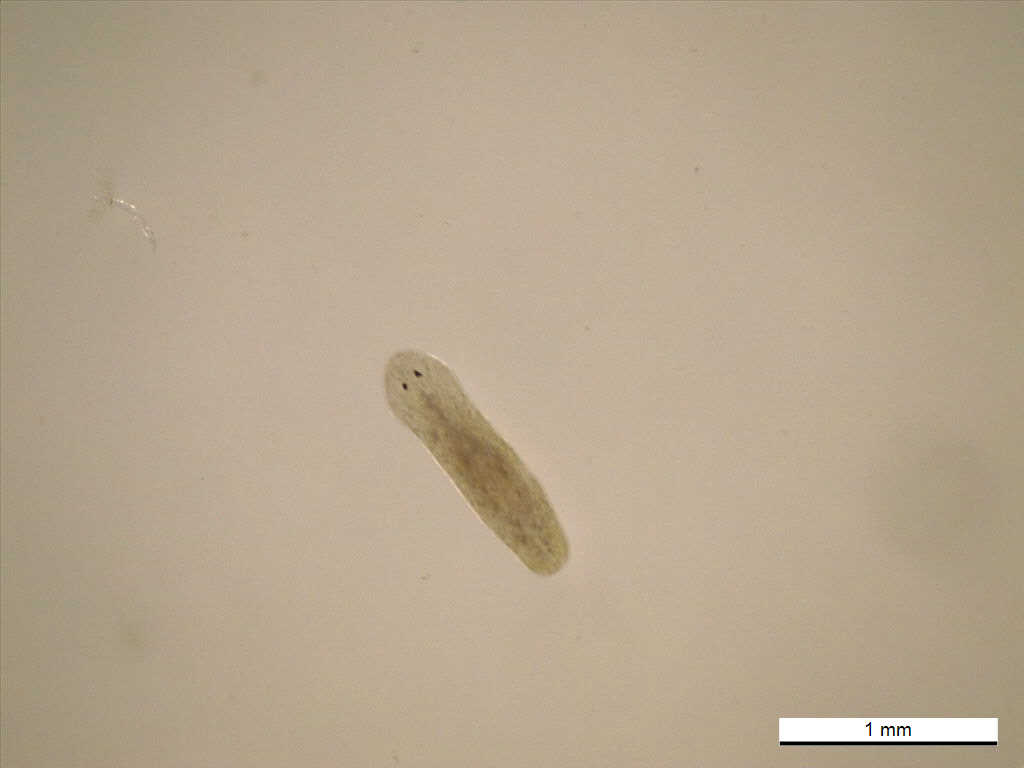

Supplement: Supplementary file 10 — Source data Fig. 3 [file 44318_2025_662_MOESM10_ESM.zip › Figure 3/3C-D/ythdf-b-c_RNAi_Before_RNAi_feedings/ythdf-b-c_RNAi_Before_RNAi_feedings_11.jpg]

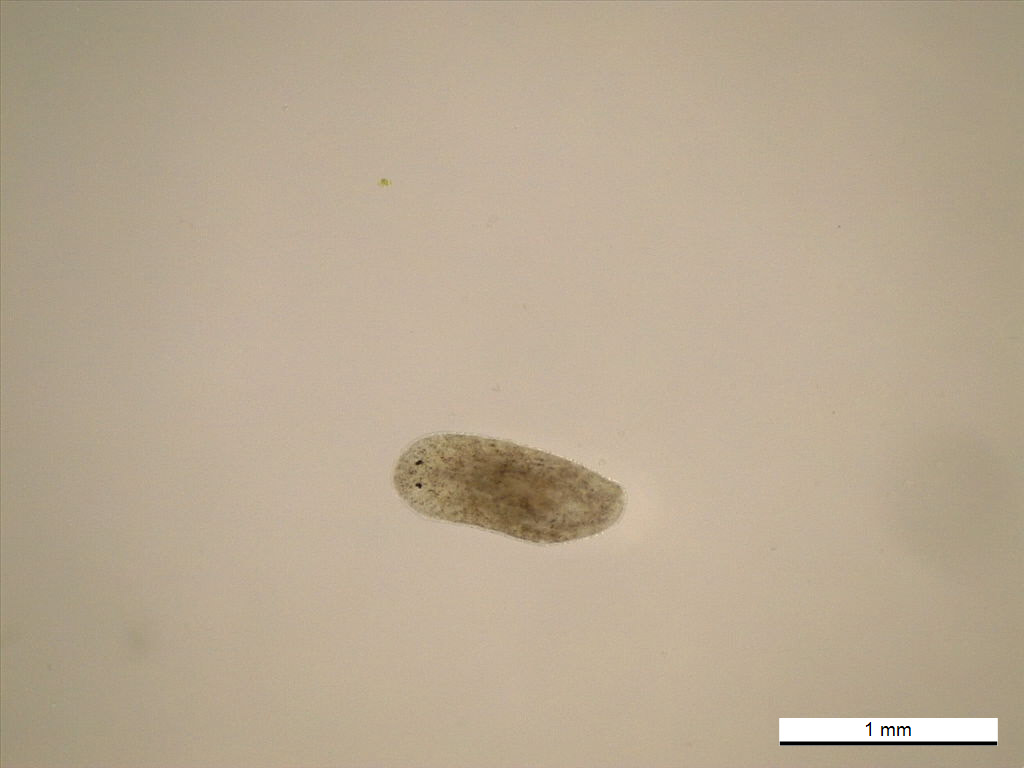

Supplement: Supplementary file 10 — Source data Fig. 3 [file 44318_2025_662_MOESM10_ESM.zip › Figure 3/3C-D/ythdf-b-c_RNAi_Before_RNAi_feedings/ythdf-b-c_RNAi_Before_RNAi_feedings_12.jpg]

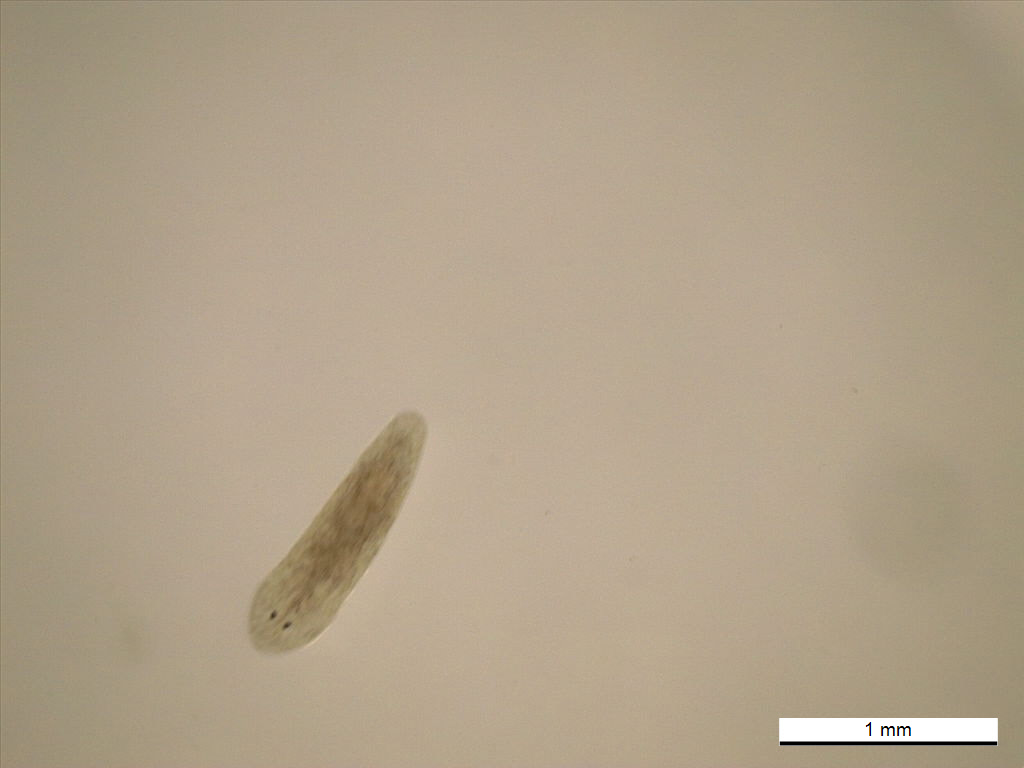

Supplement: Supplementary file 10 — Source data Fig. 3 [file 44318_2025_662_MOESM10_ESM.zip › Figure 3/3C-D/ythdf-b-c_RNAi_Before_RNAi_feedings/ythdf-b-c_RNAi_Before_RNAi_feedings_13.jpg]

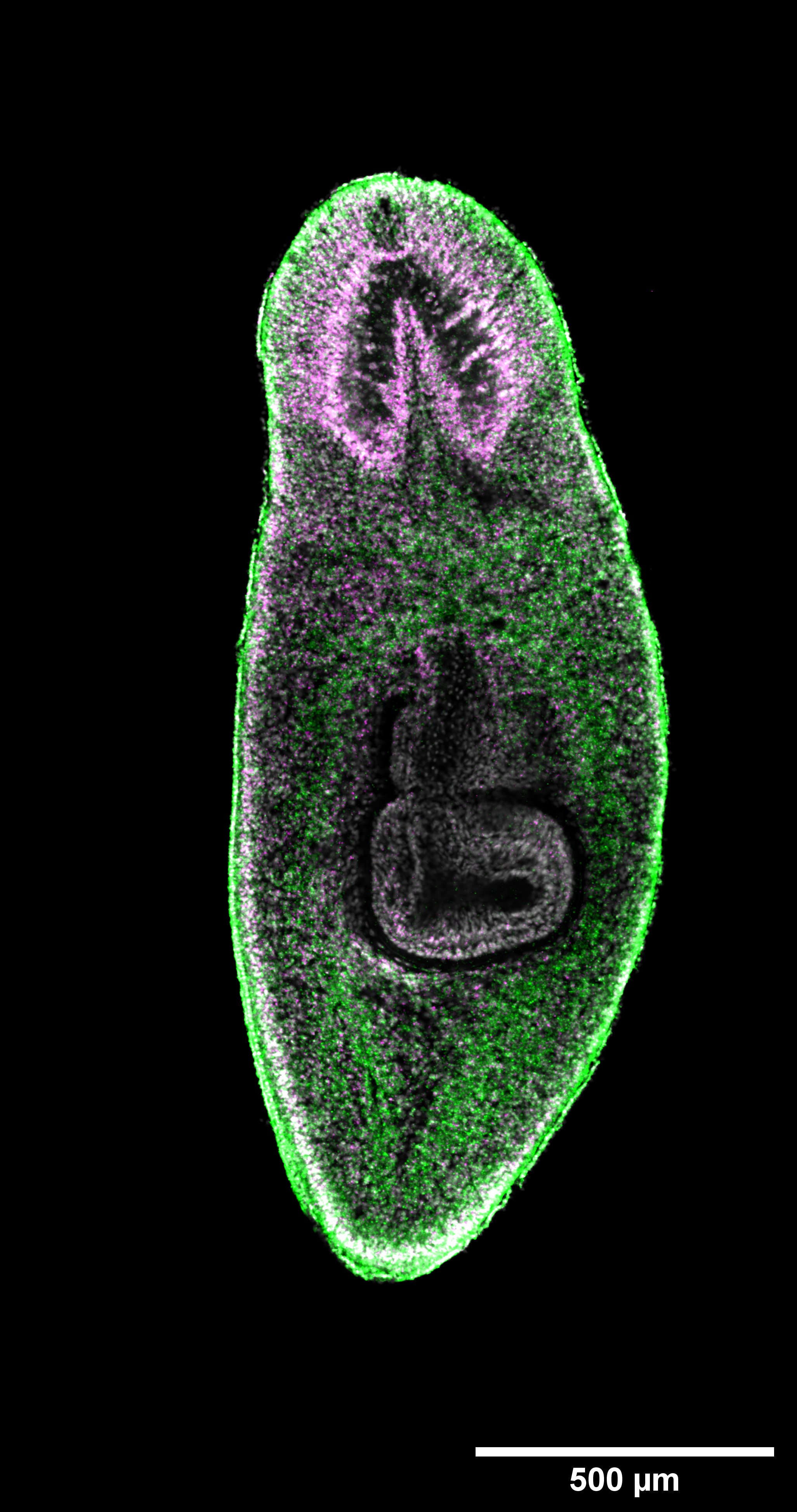

Supplement: Supplementary file 11 — Source data Fig. 4 [file 44318_2025_662_MOESM11_ESM.zip › Figure 4/4A/wildtype_ythdf-A_FITC-green_ythdf-b_Rhod-magenta_10x_stitched.jpg]

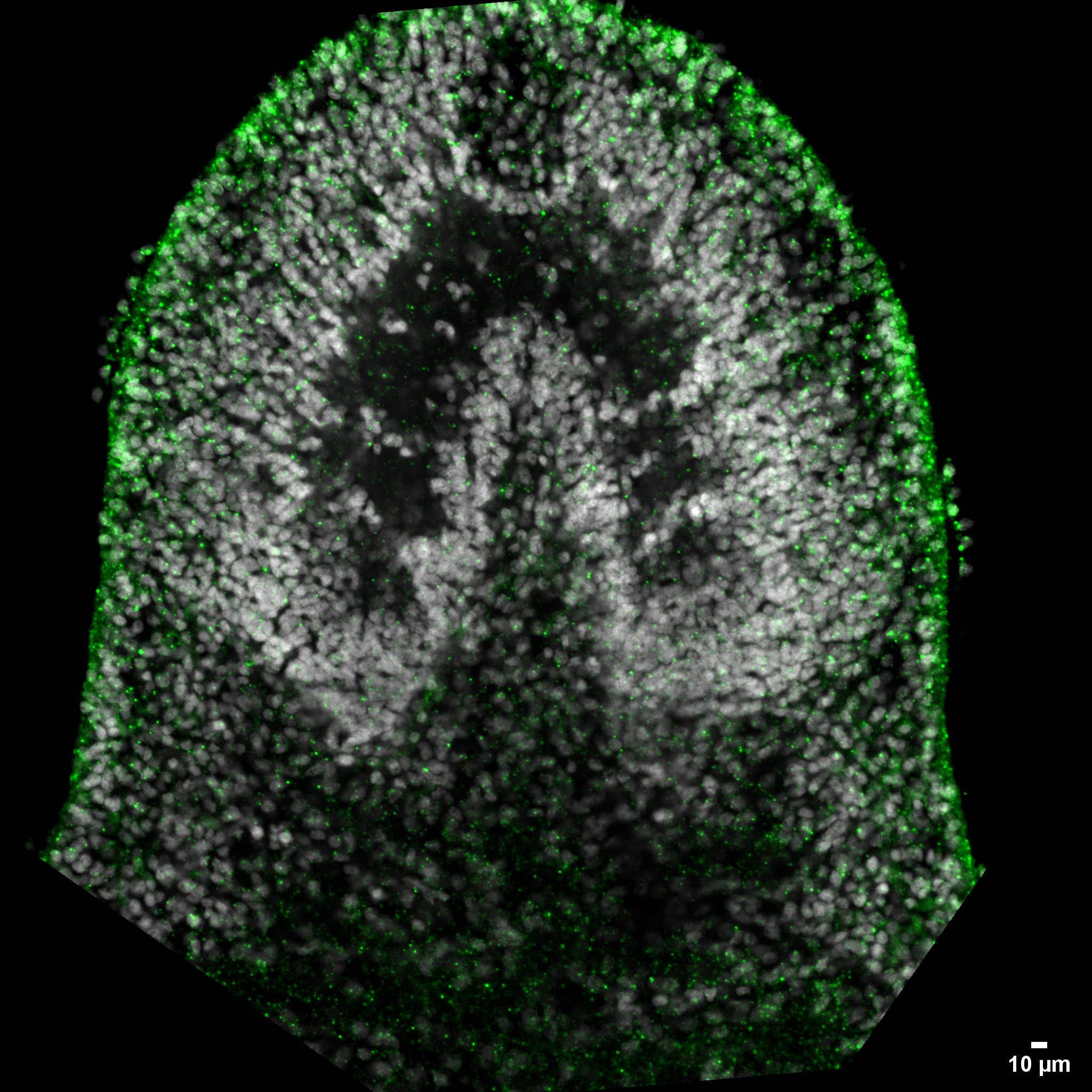

Supplement: Supplementary file 11 — Source data Fig. 4 [file 44318_2025_662_MOESM11_ESM.zip › Figure 4/4A/wildtype_ythdf-A_FITC-green_ythdf-b_Rhod-magenta_20x_Brain_FITC_channel.jpg]

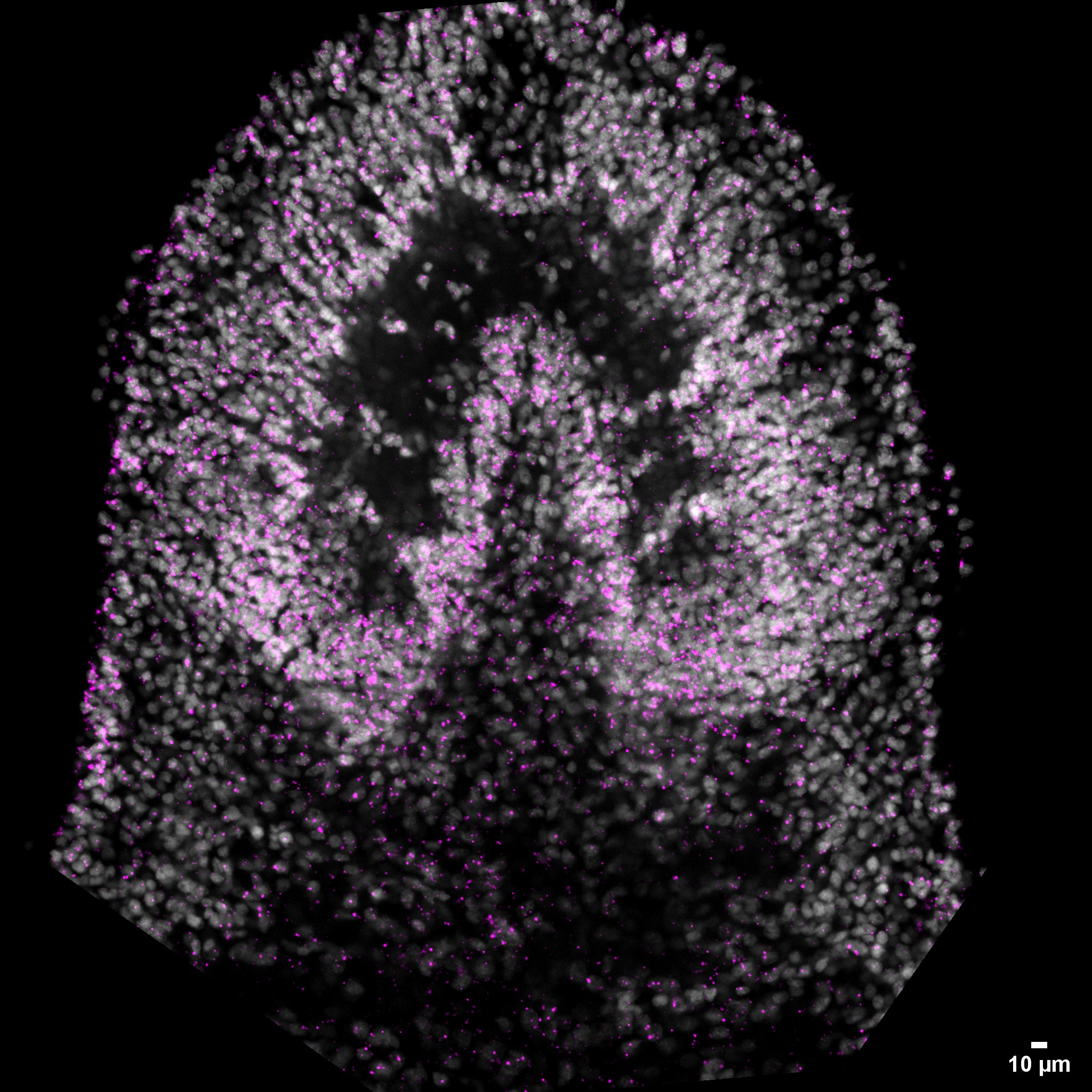

Supplement: Supplementary file 11 — Source data Fig. 4 [file 44318_2025_662_MOESM11_ESM.zip › Figure 4/4A/wildtype_ythdf-A_FITC-green_ythdf-b_Rhod-magenta_20x_Brain_Magenta_channel.jpg]

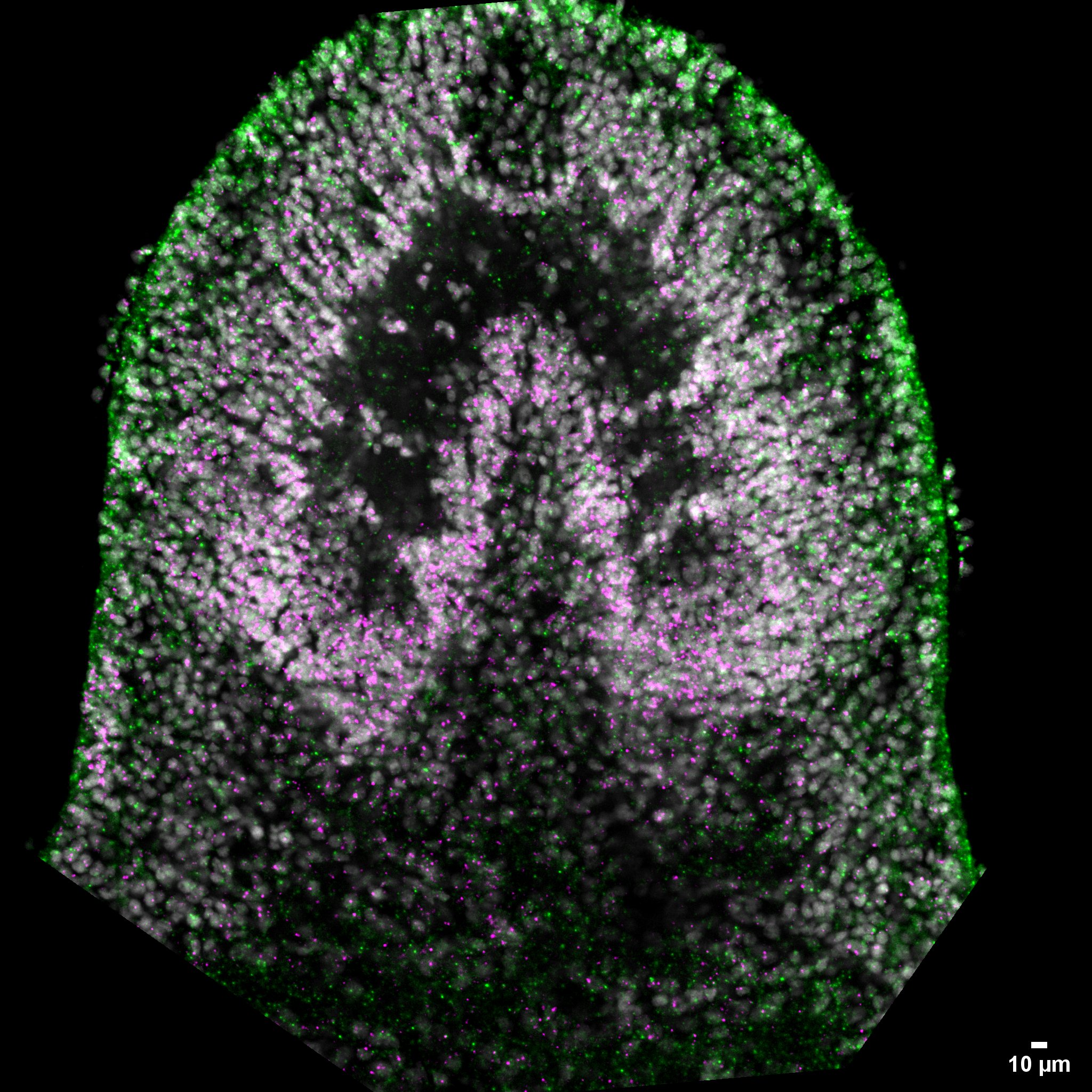

Supplement: Supplementary file 11 — Source data Fig. 4 [file 44318_2025_662_MOESM11_ESM.zip › Figure 4/4A/wildtype_ythdf-A_FITC-green_ythdf-b_Rhod-magenta_20x_Brain_Merged.jpg]

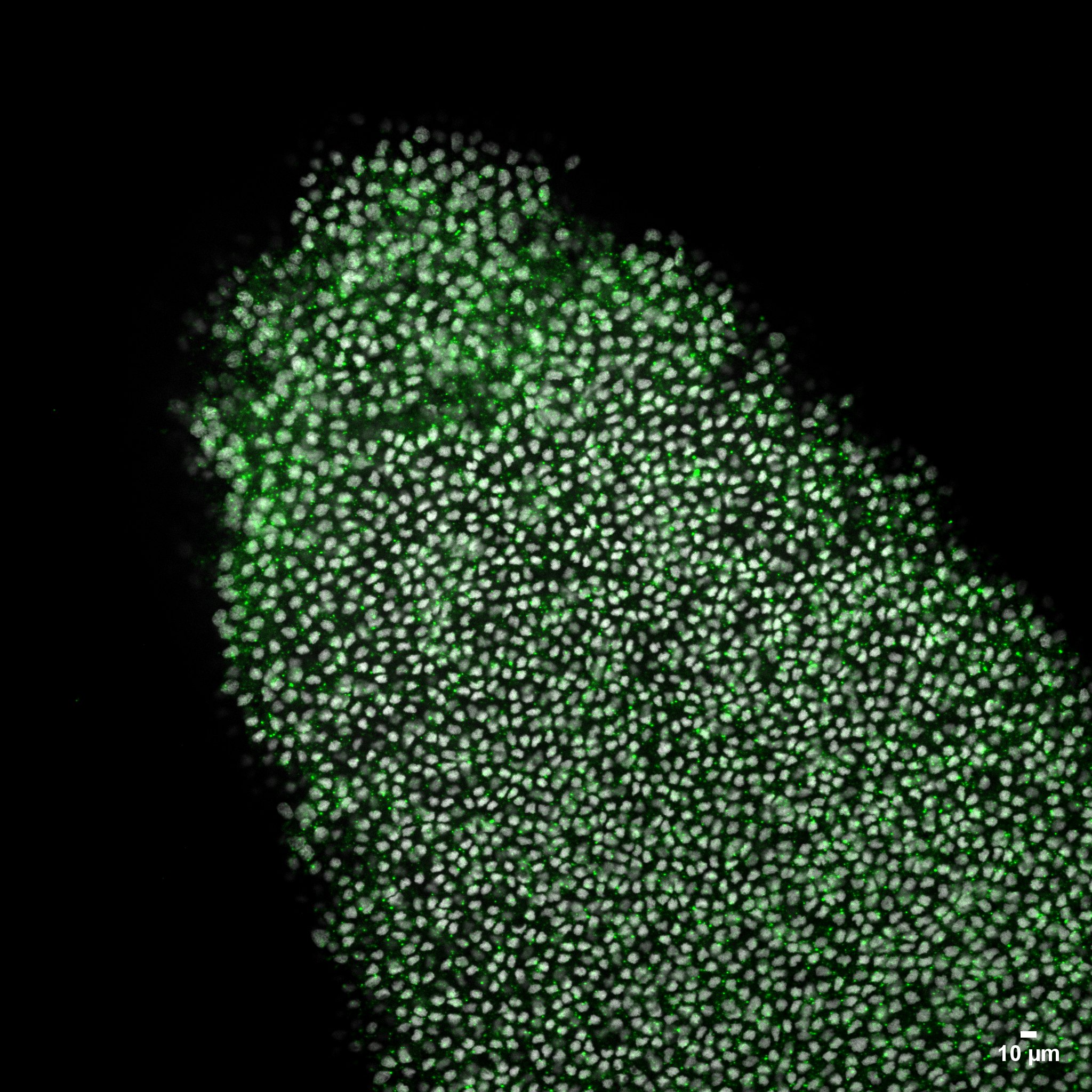

Supplement: Supplementary file 11 — Source data Fig. 4 [file 44318_2025_662_MOESM11_ESM.zip › Figure 4/4A/wildtype_ythdf-A_FITC-green_ythdf-b_Rhod-magenta_20x_Epidermis_FITC_channel.jpg]

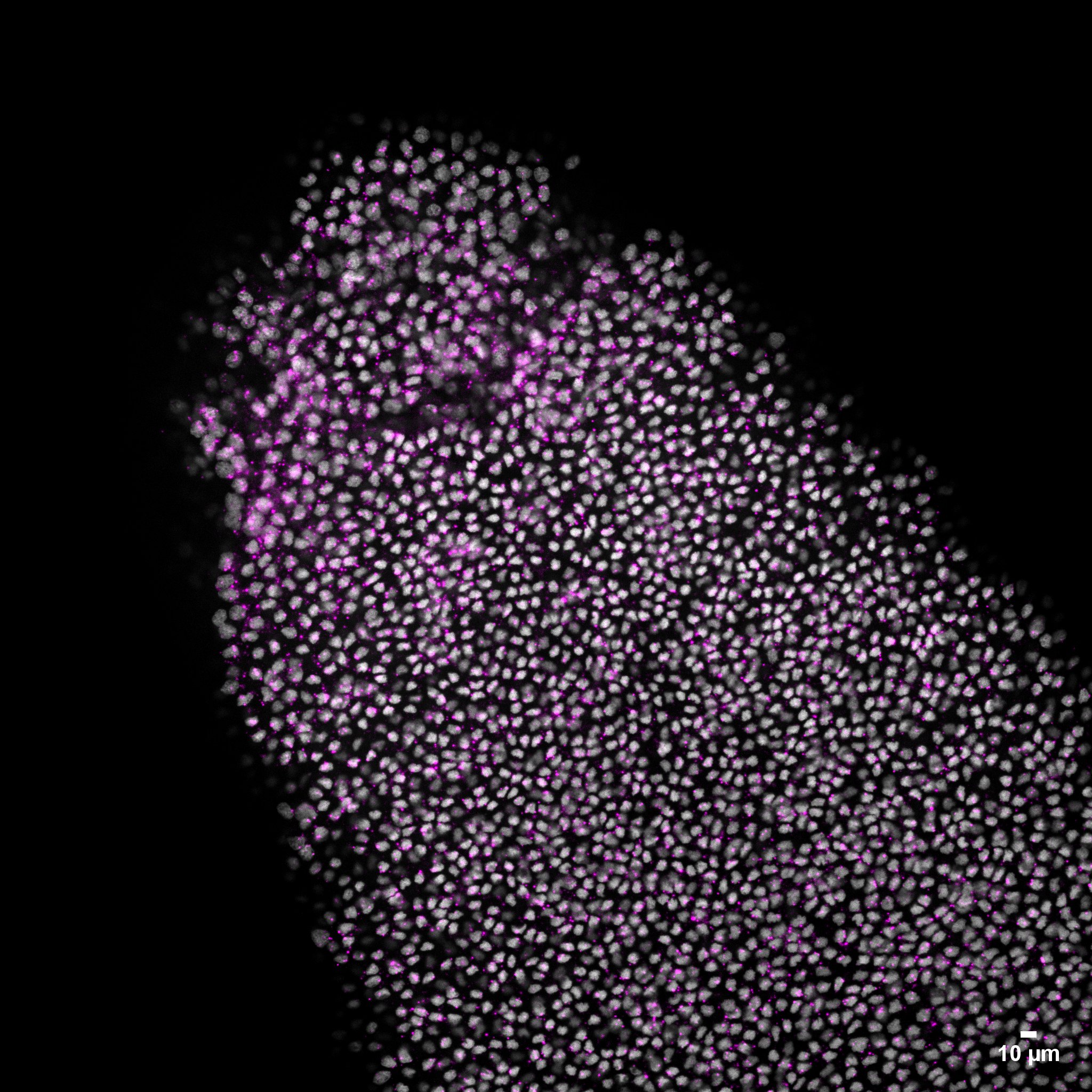

Supplement: Supplementary file 11 — Source data Fig. 4 [file 44318_2025_662_MOESM11_ESM.zip › Figure 4/4A/wildtype_ythdf-A_FITC-green_ythdf-b_Rhod-magenta_20x_Epidermis_Magenta_channel.jpg]

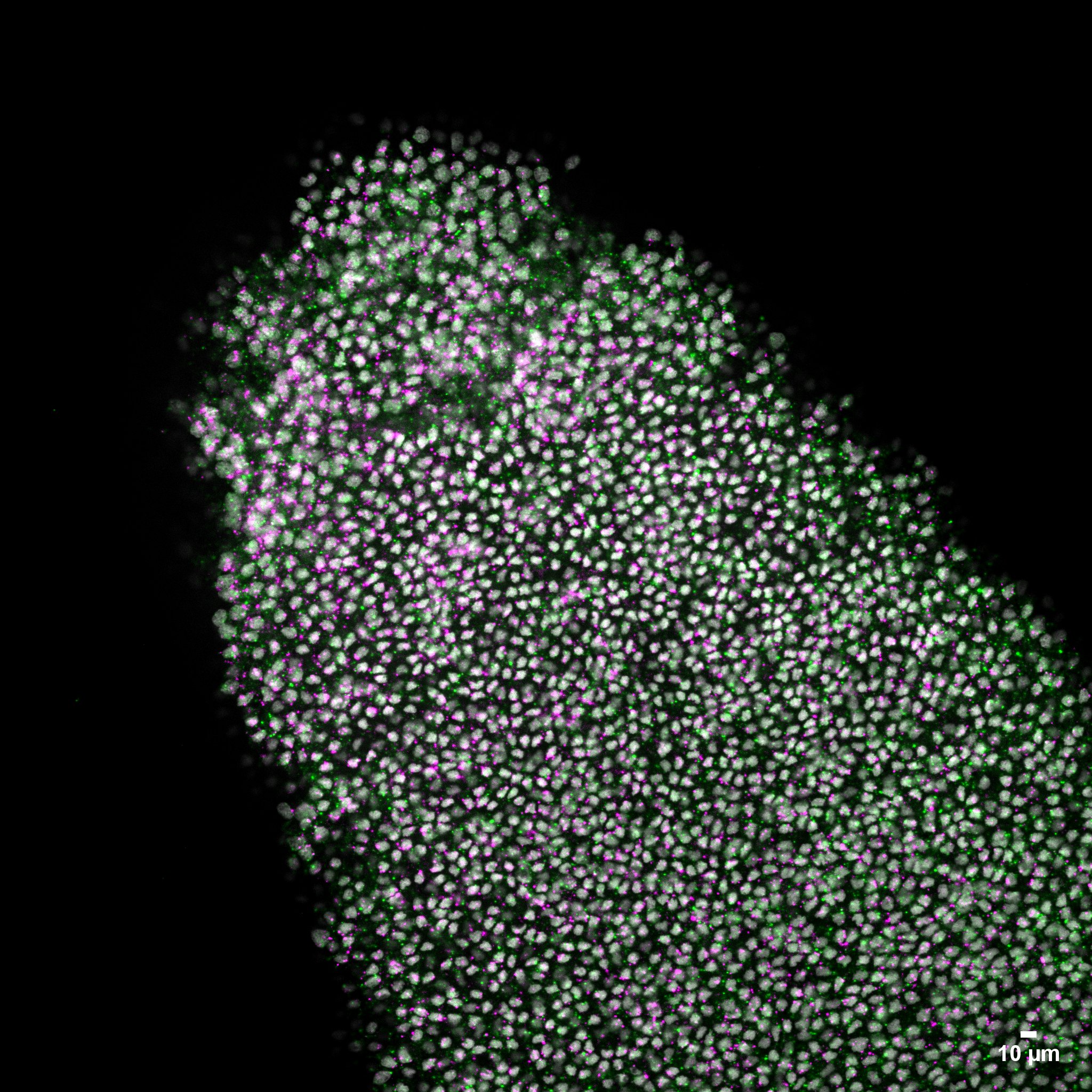

Supplement: Supplementary file 11 — Source data Fig. 4 [file 44318_2025_662_MOESM11_ESM.zip › Figure 4/4A/wildtype_ythdf-A_FITC-green_ythdf-b_Rhod-magenta_20x_Epidermis_Merged.jpg]

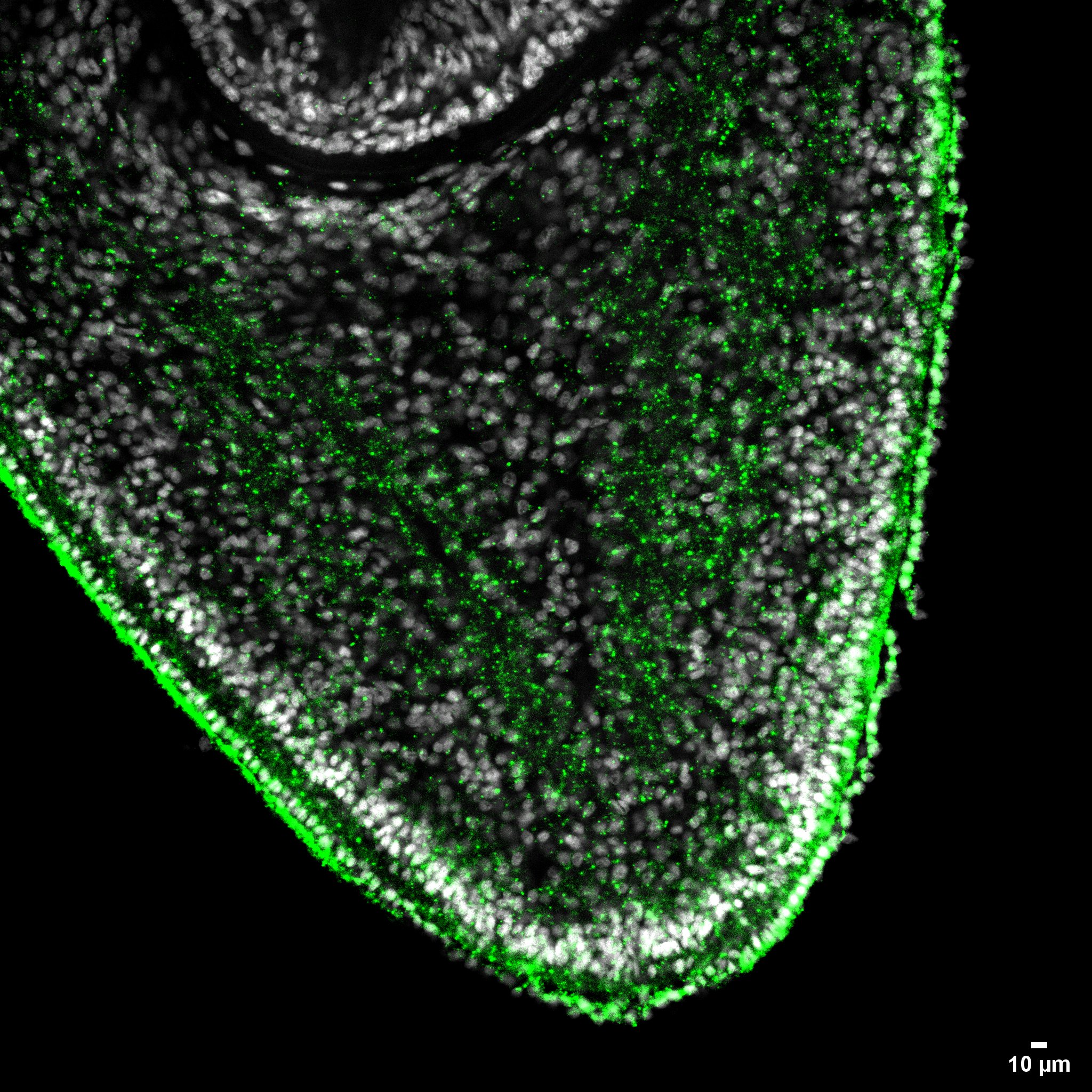

Supplement: Supplementary file 11 — Source data Fig. 4 [file 44318_2025_662_MOESM11_ESM.zip › Figure 4/4A/wildtype_ythdf-A_FITC-green_ythdf-b_Rhod-magenta_20x_Intestine_FITC_channel.jpg]

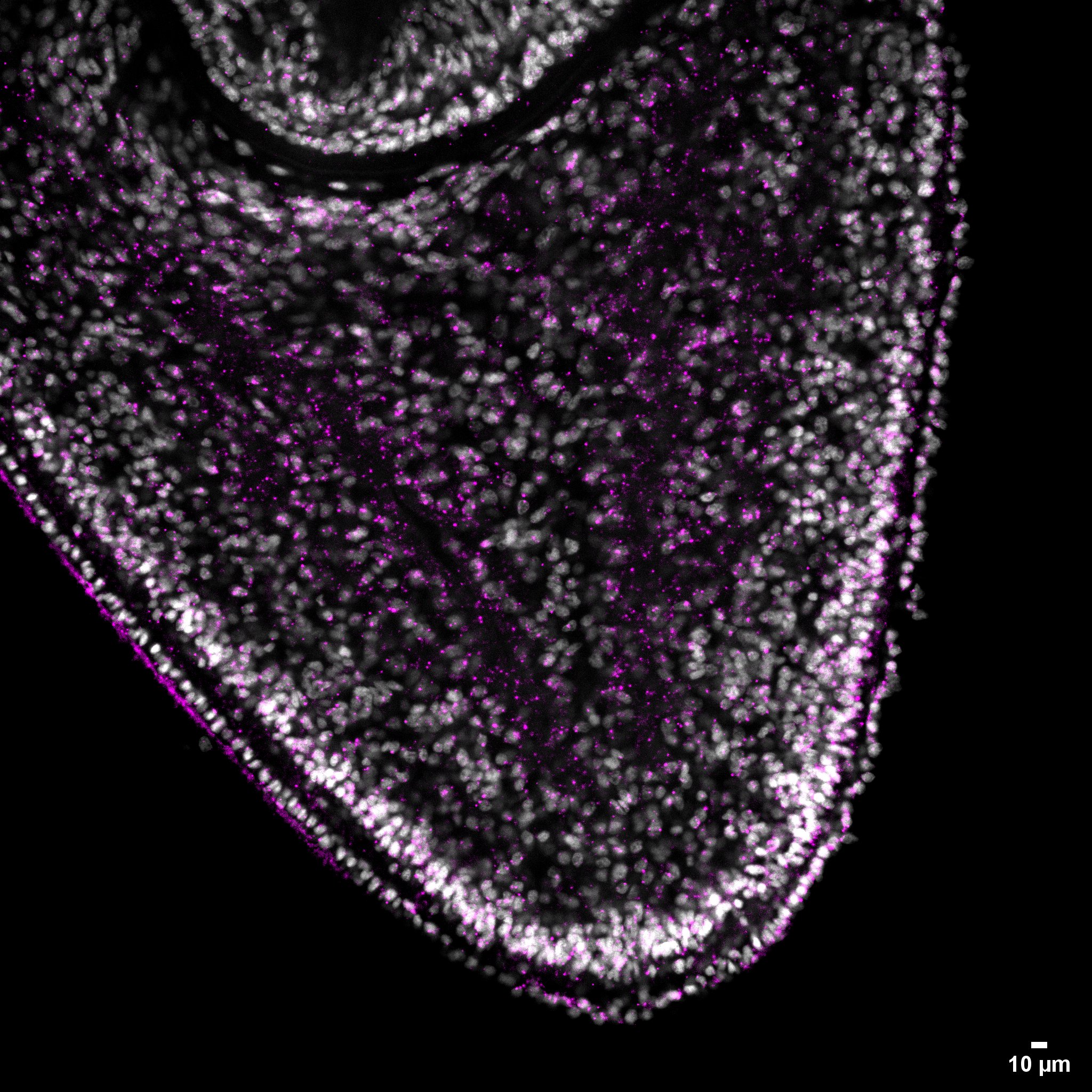

Supplement: Supplementary file 11 — Source data Fig. 4 [file 44318_2025_662_MOESM11_ESM.zip › Figure 4/4A/wildtype_ythdf-A_FITC-green_ythdf-b_Rhod-magenta_20x_Intestine_Magenta_channel.jpg]

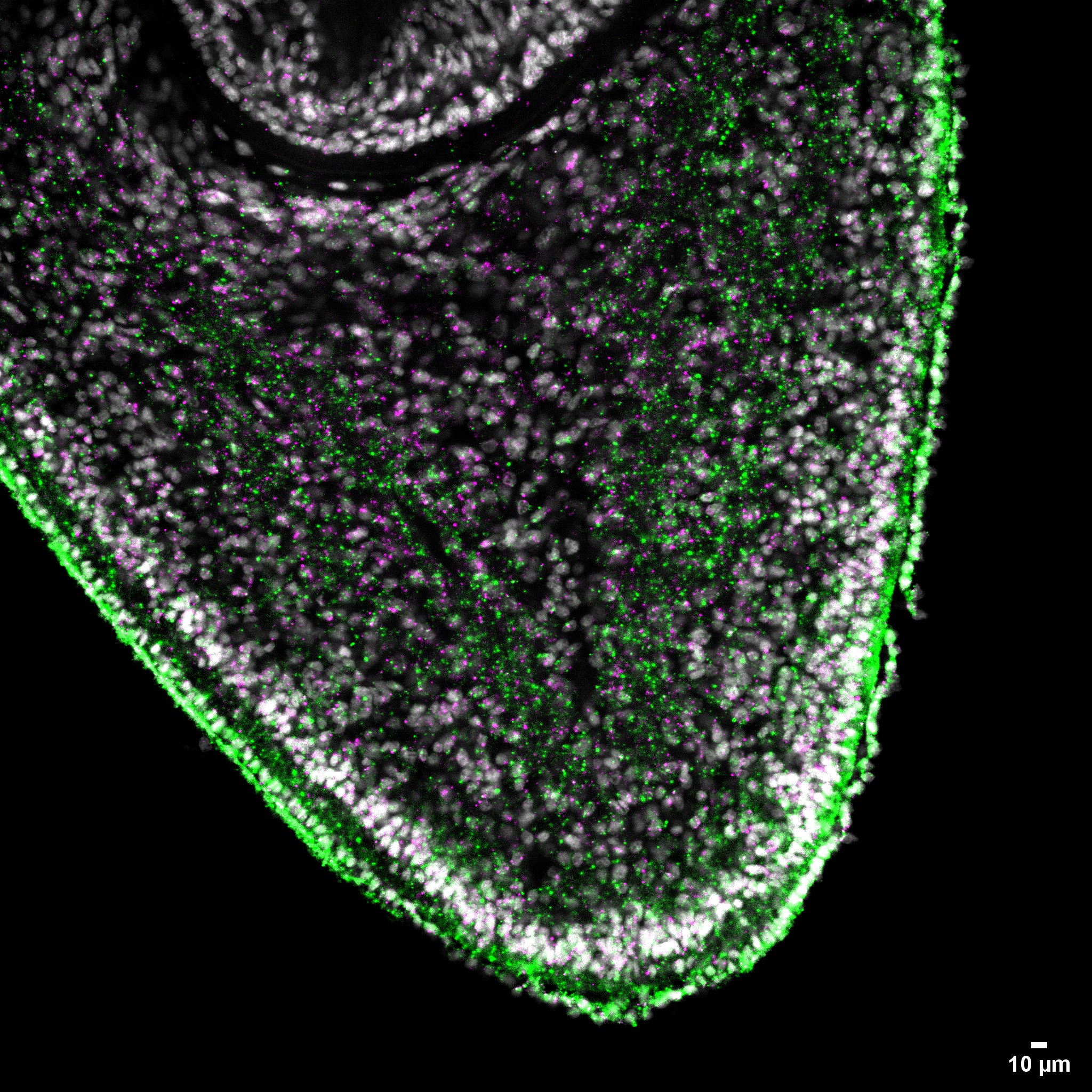

Supplement: Supplementary file 11 — Source data Fig. 4 [file 44318_2025_662_MOESM11_ESM.zip › Figure 4/4A/wildtype_ythdf-A_FITC-green_ythdf-b_Rhod-magenta_20x_Intestine_Merged.jpg]

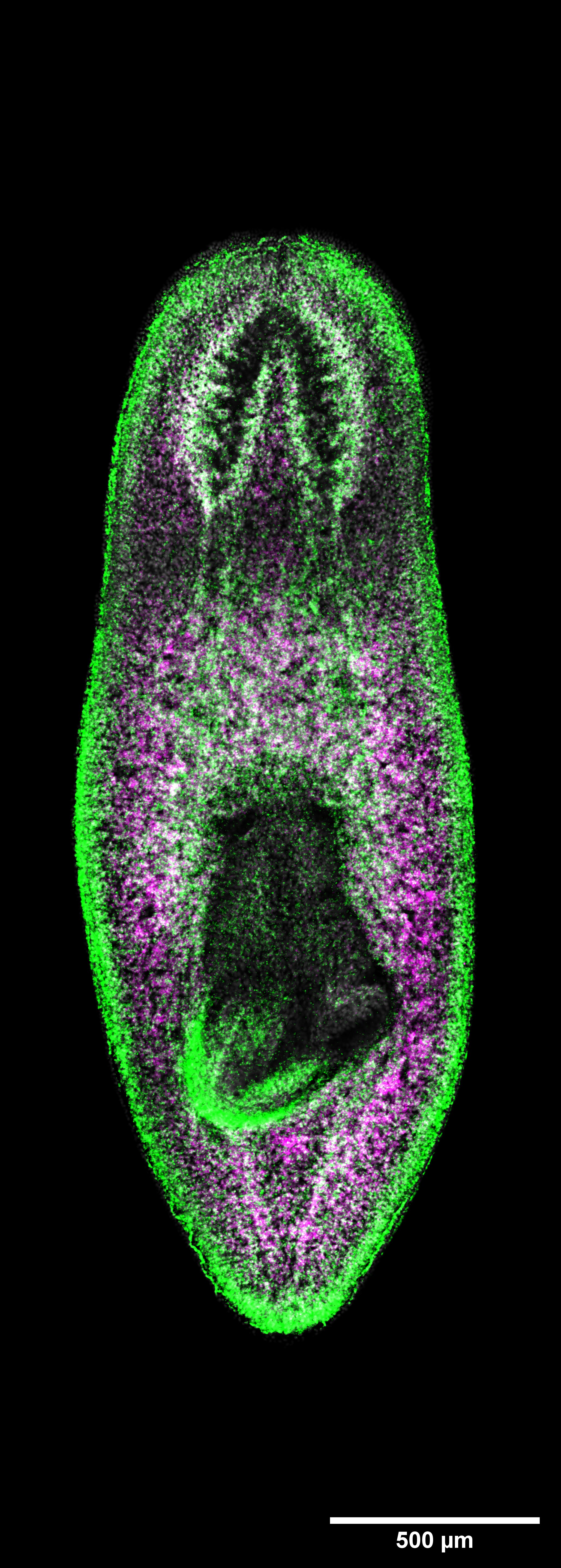

Supplement: Supplementary file 11 — Source data Fig. 4 [file 44318_2025_662_MOESM11_ESM.zip › Figure 4/4A/wildtype_ythdf-b_FITC-green_ythdf-c_Rhod-magenta_10x_stitched.jpg]

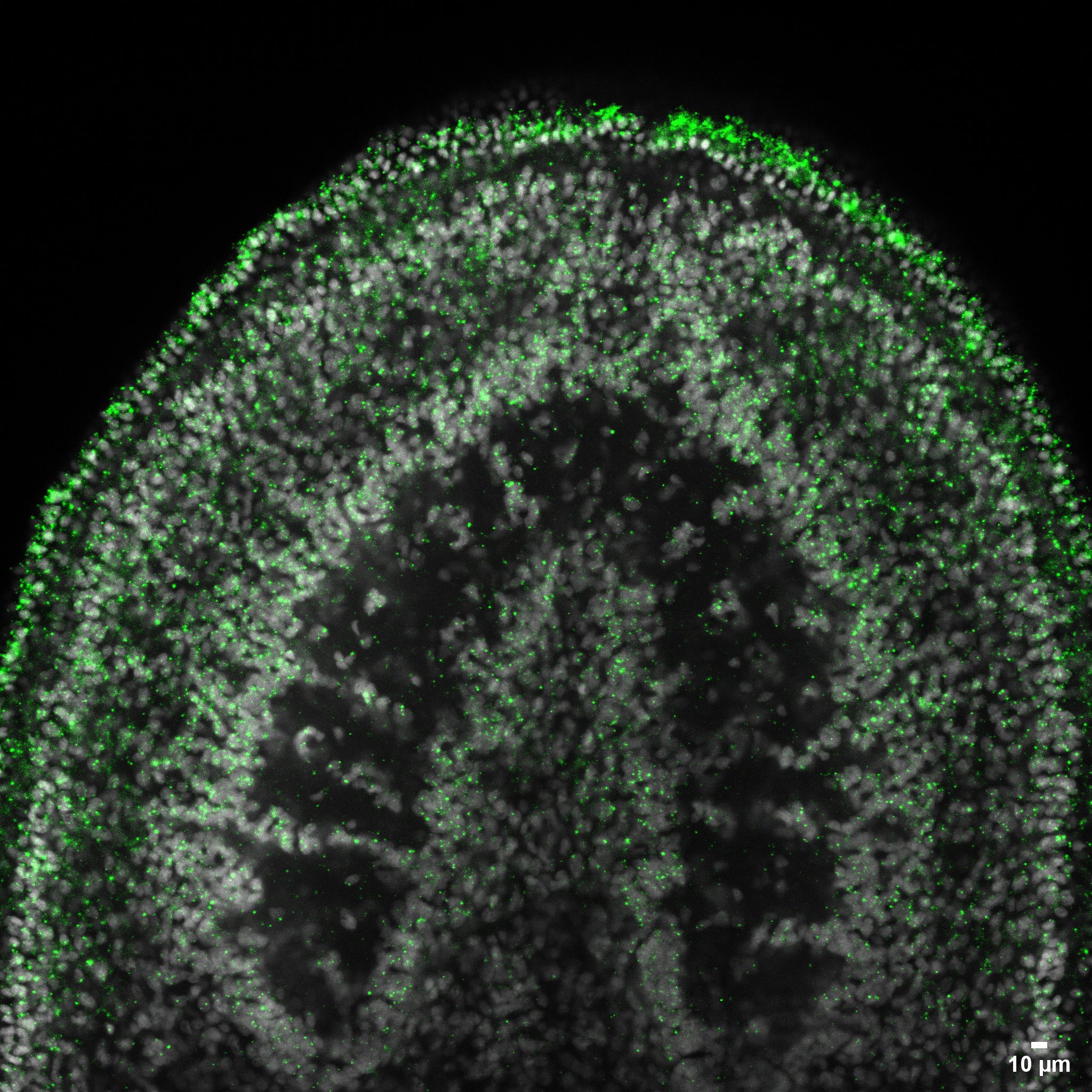

Supplement: Supplementary file 11 — Source data Fig. 4 [file 44318_2025_662_MOESM11_ESM.zip › Figure 4/4A/wildtype_ythdf-b_FITC-green_ythdf-c_Rhod-magenta_20x_Brain_FITC_channel.jpg]

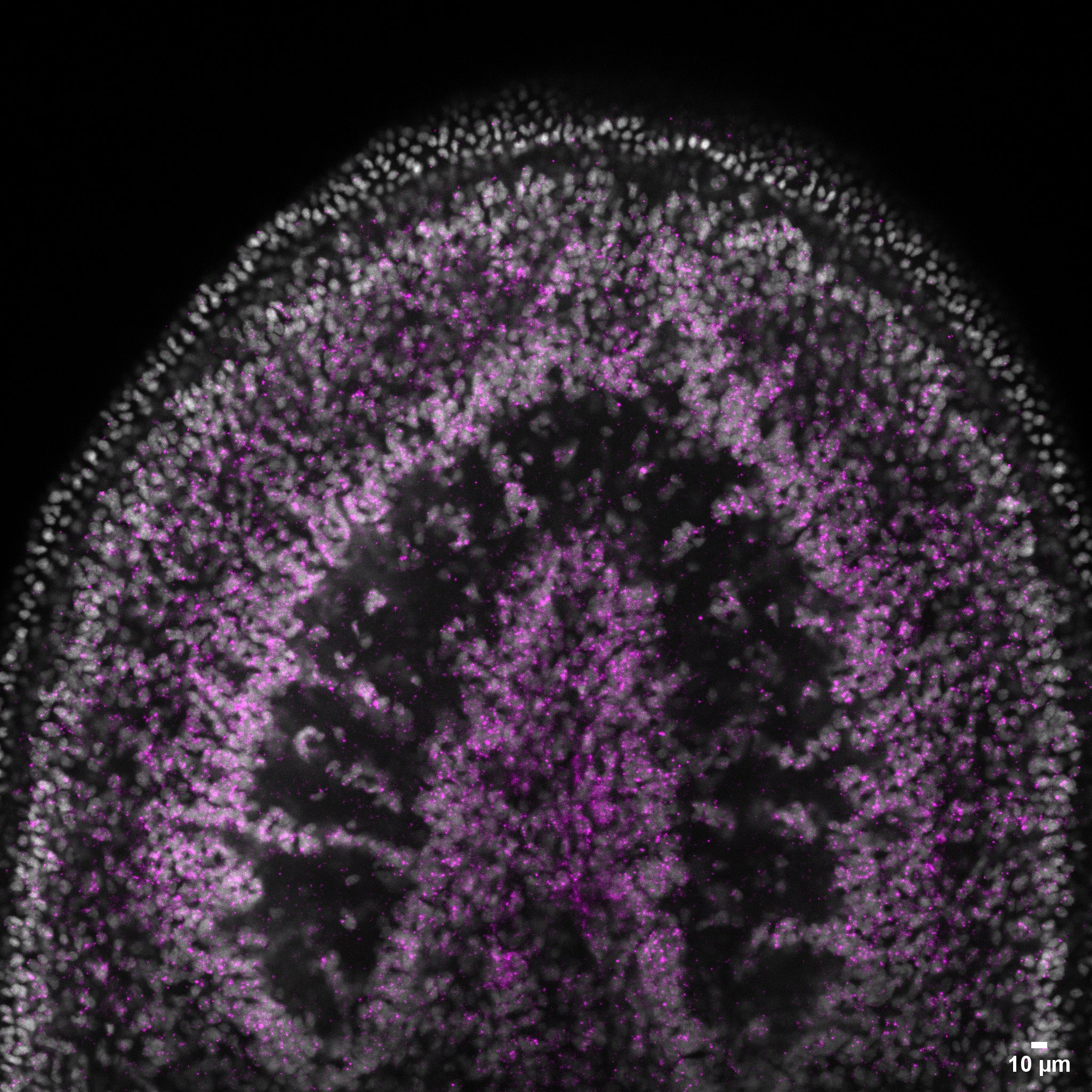

Supplement: Supplementary file 11 — Source data Fig. 4 [file 44318_2025_662_MOESM11_ESM.zip › Figure 4/4A/wildtype_ythdf-b_FITC-green_ythdf-c_Rhod-magenta_20x_Brain_Magenta_channel.jpg]

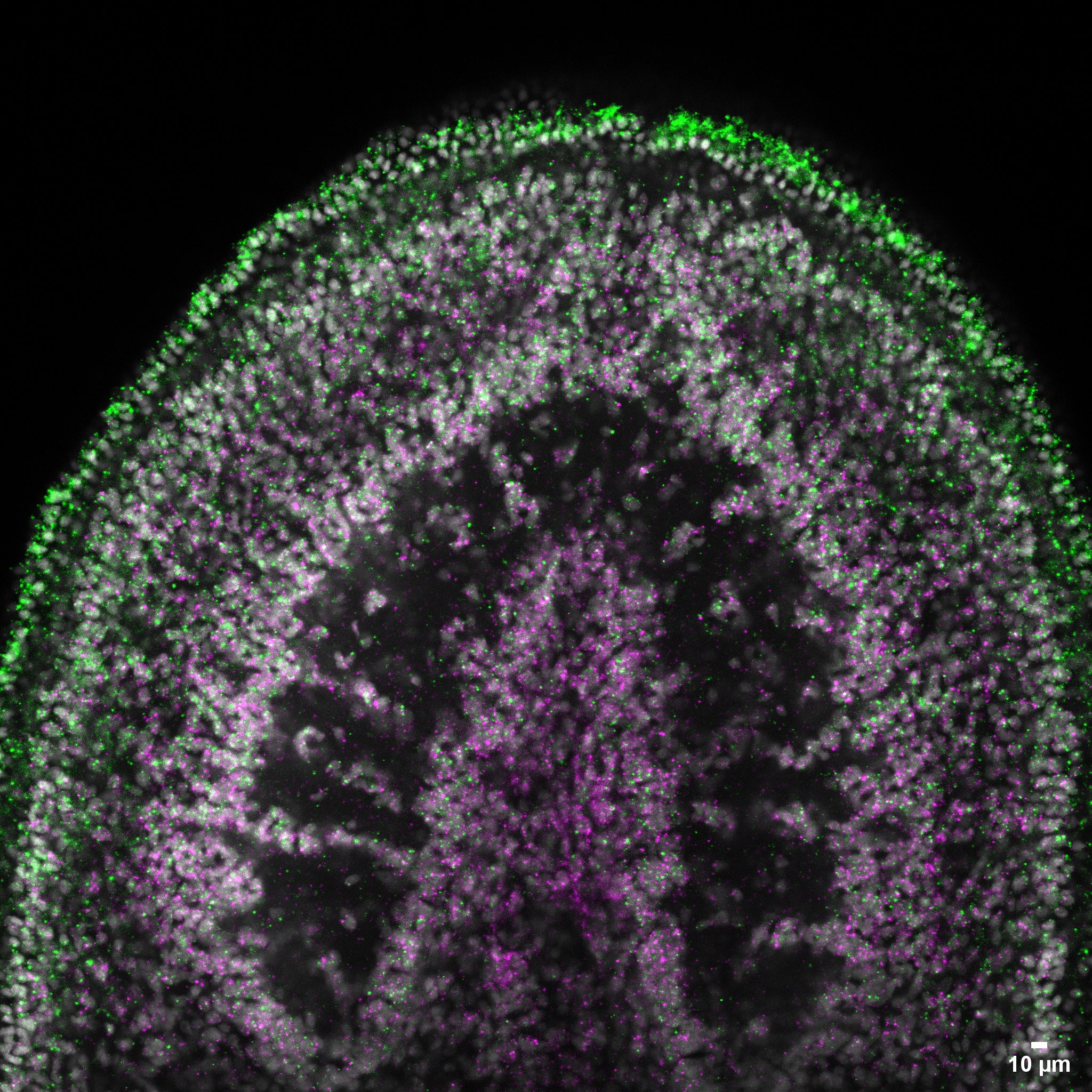

Supplement: Supplementary file 11 — Source data Fig. 4 [file 44318_2025_662_MOESM11_ESM.zip › Figure 4/4A/wildtype_ythdf-b_FITC-green_ythdf-c_Rhod-magenta_20x_Brain_Merged.jpg]
