# Supplementary figures and images for: Single-nucleotide m⁶A mapping uncovers redundant YTHDF function in planarian progenitor fate selection (part 1 of 6)
Source: EMBO J. 2026 Jan 3;45(3):749–88. doi: 10.1038/s44318-025-00662-3 (PMC12864844; doi:10.1038/s44318-025-00662-3)

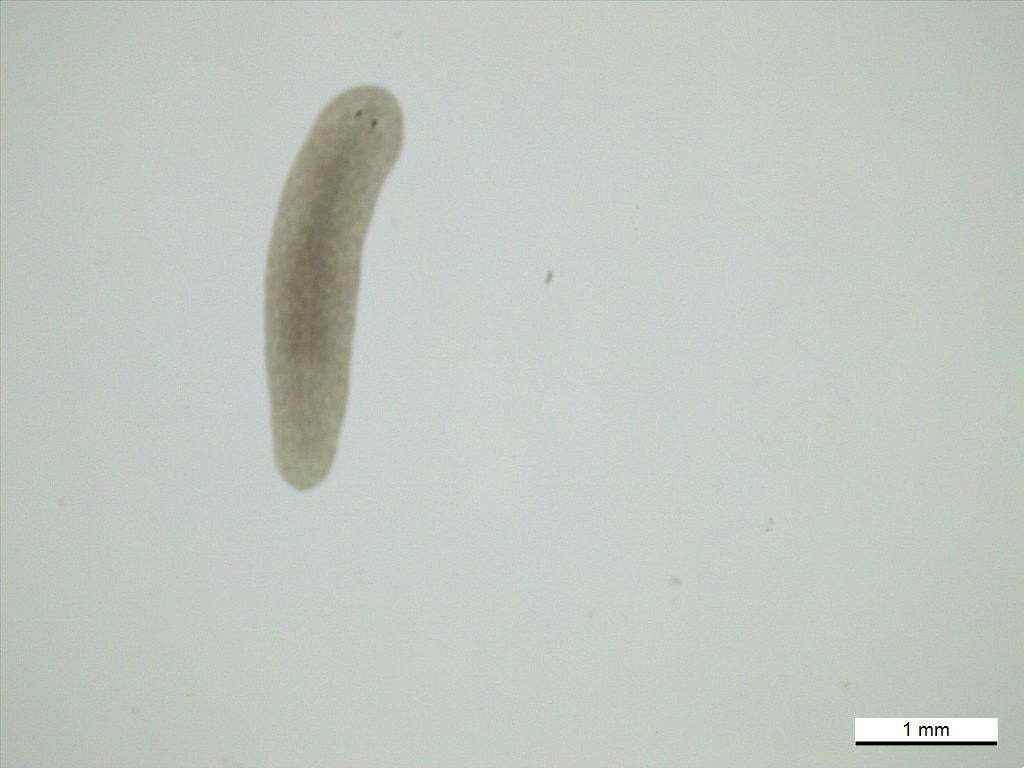

Supplement: Supplementary file 10 — Source data Fig. 3 [file 44318_2025_662_MOESM10_ESM.zip › Figure 3/3C-D/All_ythdf_RNAi_After_10_RNAi_feedings/All_ythdf_RNAi_After_10_RNAi_feedings_1.jpg]

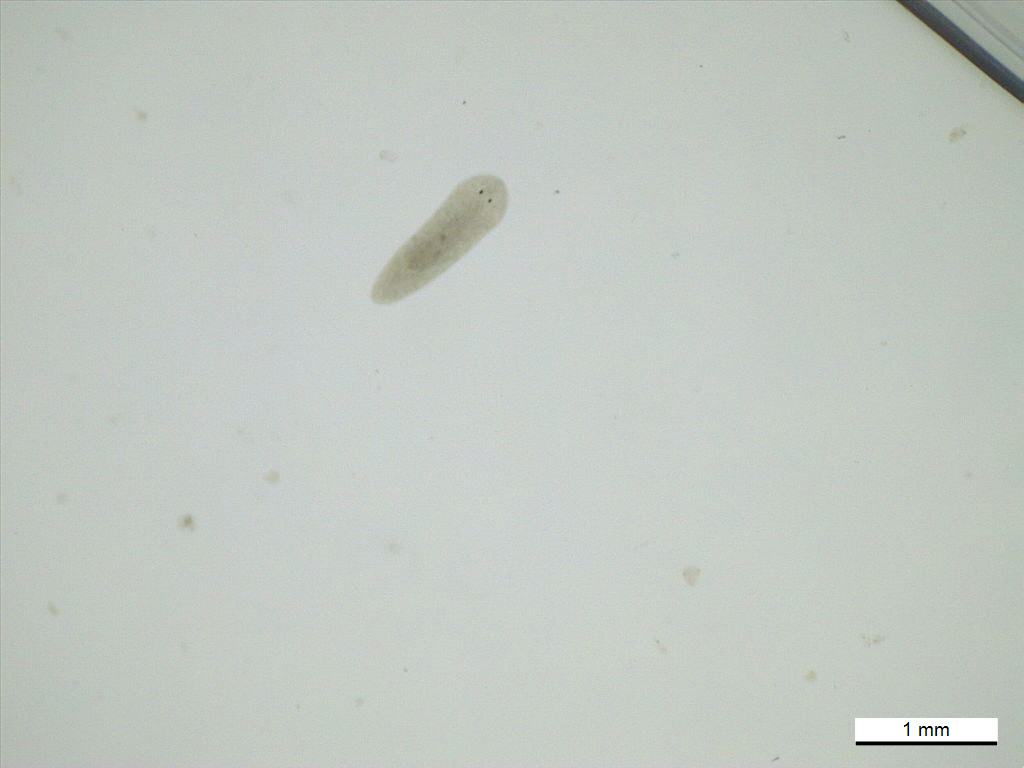

Supplement: Supplementary file 10 — Source data Fig. 3 [file 44318_2025_662_MOESM10_ESM.zip › Figure 3/3C-D/All_ythdf_RNAi_After_10_RNAi_feedings/All_ythdf_RNAi_After_10_RNAi_feedings_10.jpg]

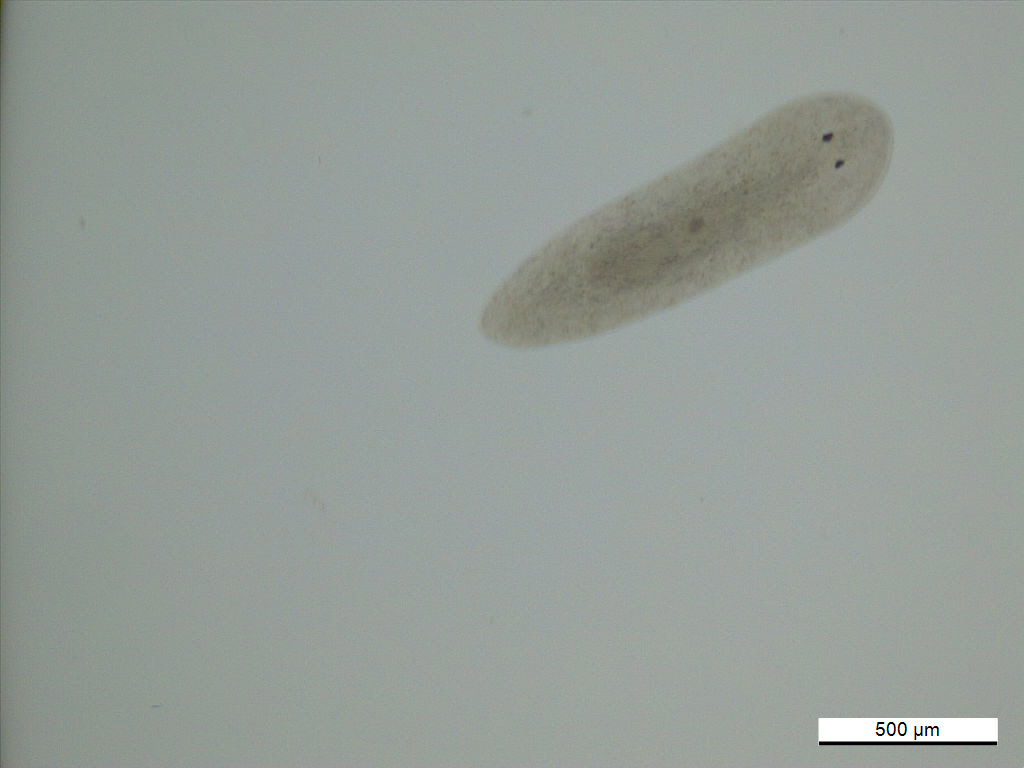

Supplement: Supplementary file 10 — Source data Fig. 3 [file 44318_2025_662_MOESM10_ESM.zip › Figure 3/3C-D/All_ythdf_RNAi_After_10_RNAi_feedings/All_ythdf_RNAi_After_10_RNAi_feedings_11.jpg]

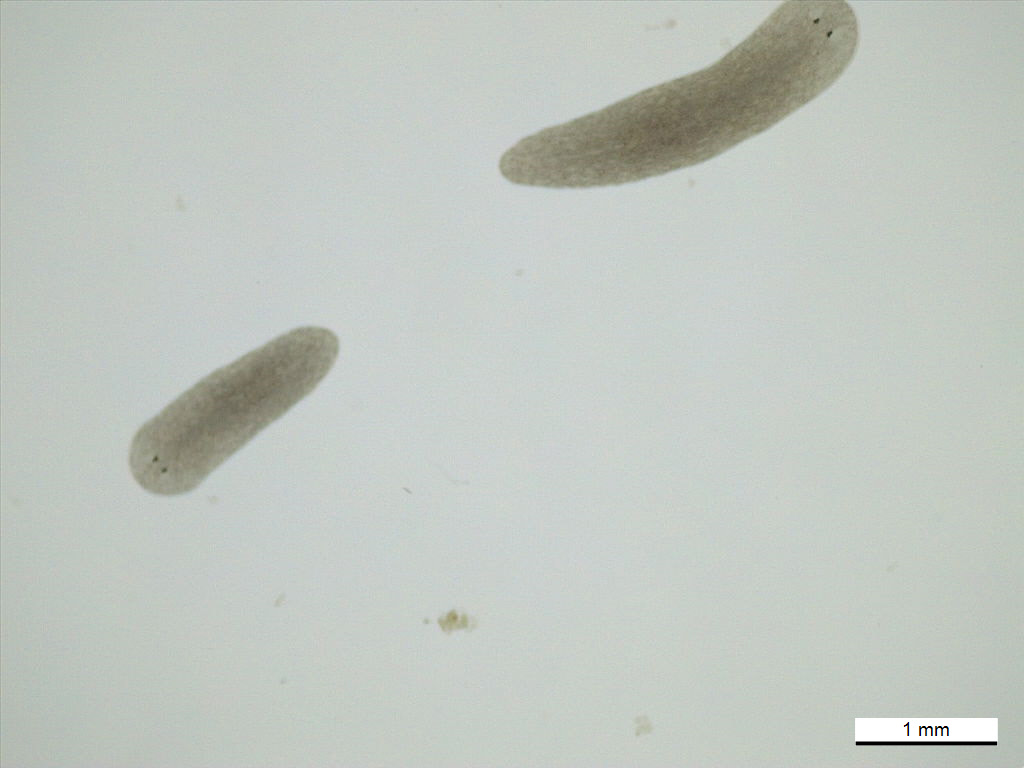

Supplement: Supplementary file 10 — Source data Fig. 3 [file 44318_2025_662_MOESM10_ESM.zip › Figure 3/3C-D/All_ythdf_RNAi_After_10_RNAi_feedings/All_ythdf_RNAi_After_10_RNAi_feedings_2-3.jpg]

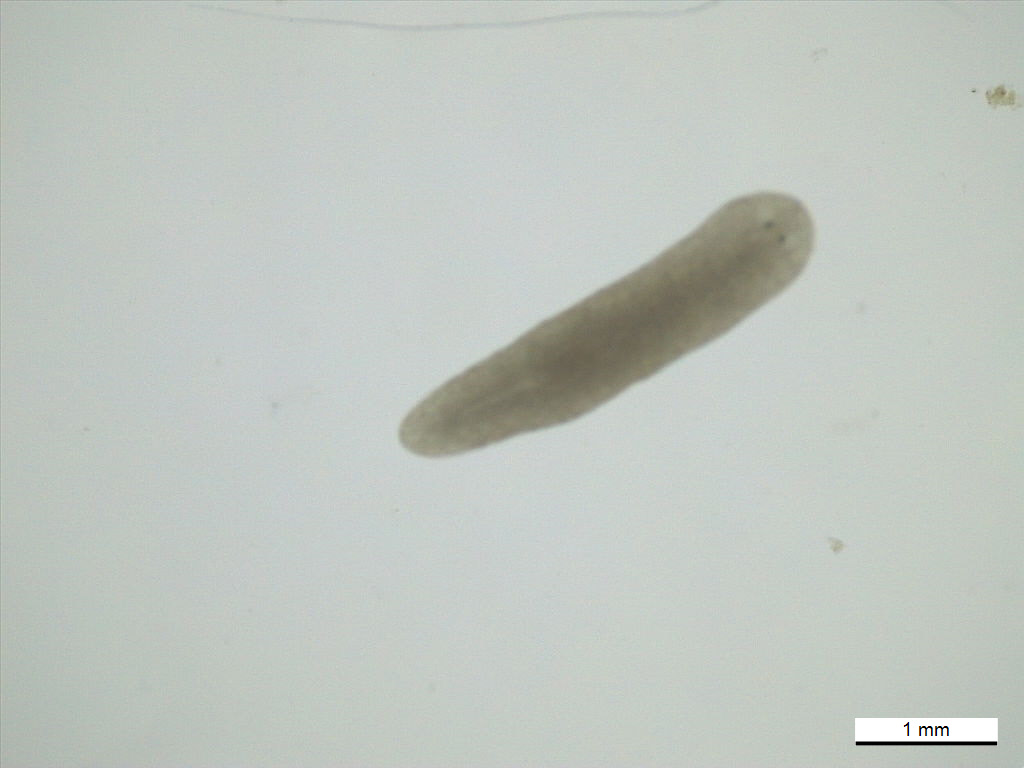

Supplement: Supplementary file 10 — Source data Fig. 3 [file 44318_2025_662_MOESM10_ESM.zip › Figure 3/3C-D/All_ythdf_RNAi_After_10_RNAi_feedings/All_ythdf_RNAi_After_10_RNAi_feedings_4.jpg]

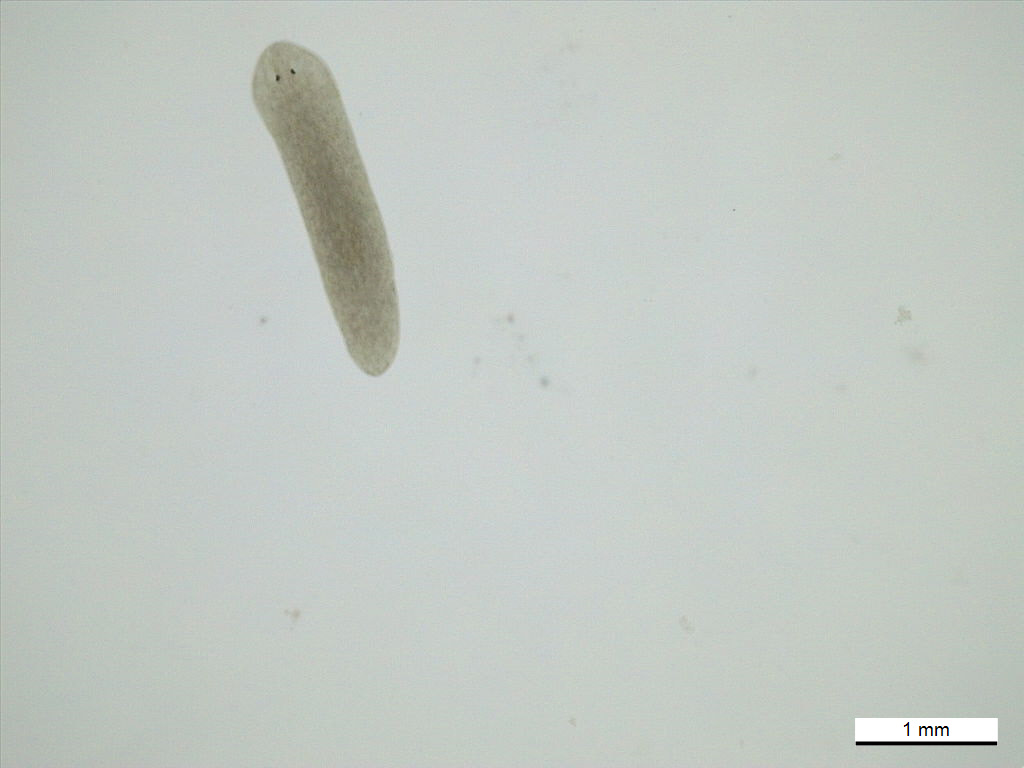

Supplement: Supplementary file 10 — Source data Fig. 3 [file 44318_2025_662_MOESM10_ESM.zip › Figure 3/3C-D/All_ythdf_RNAi_After_10_RNAi_feedings/All_ythdf_RNAi_After_10_RNAi_feedings_5.jpg]

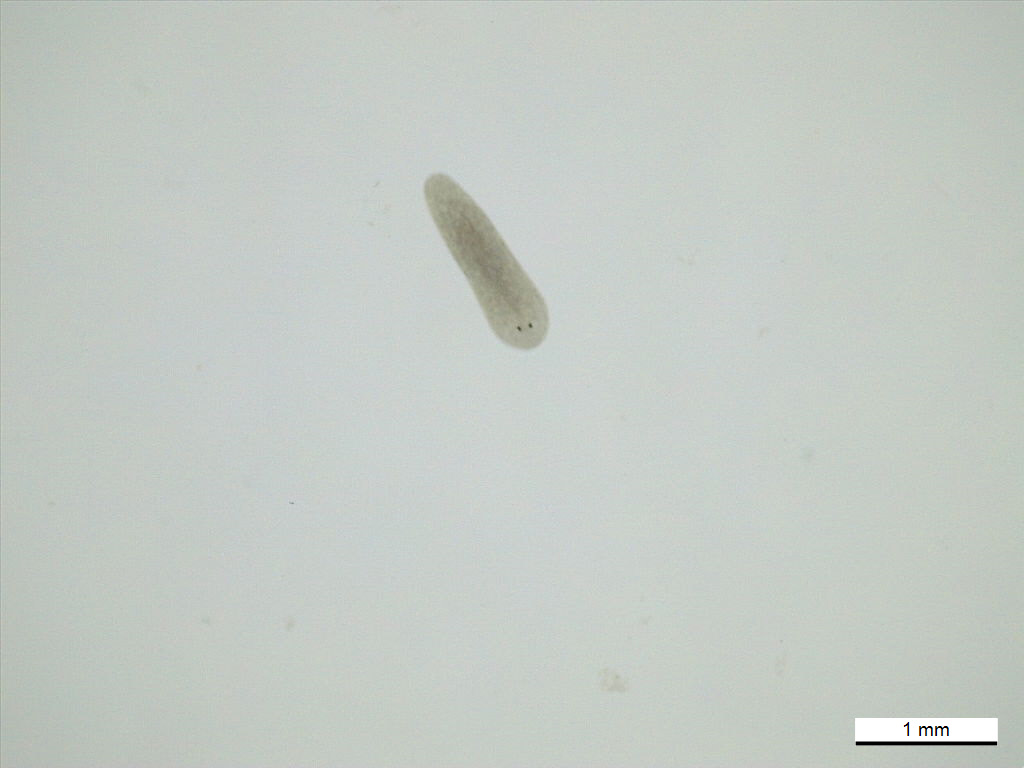

Supplement: Supplementary file 10 — Source data Fig. 3 [file 44318_2025_662_MOESM10_ESM.zip › Figure 3/3C-D/All_ythdf_RNAi_After_10_RNAi_feedings/All_ythdf_RNAi_After_10_RNAi_feedings_6.jpg]

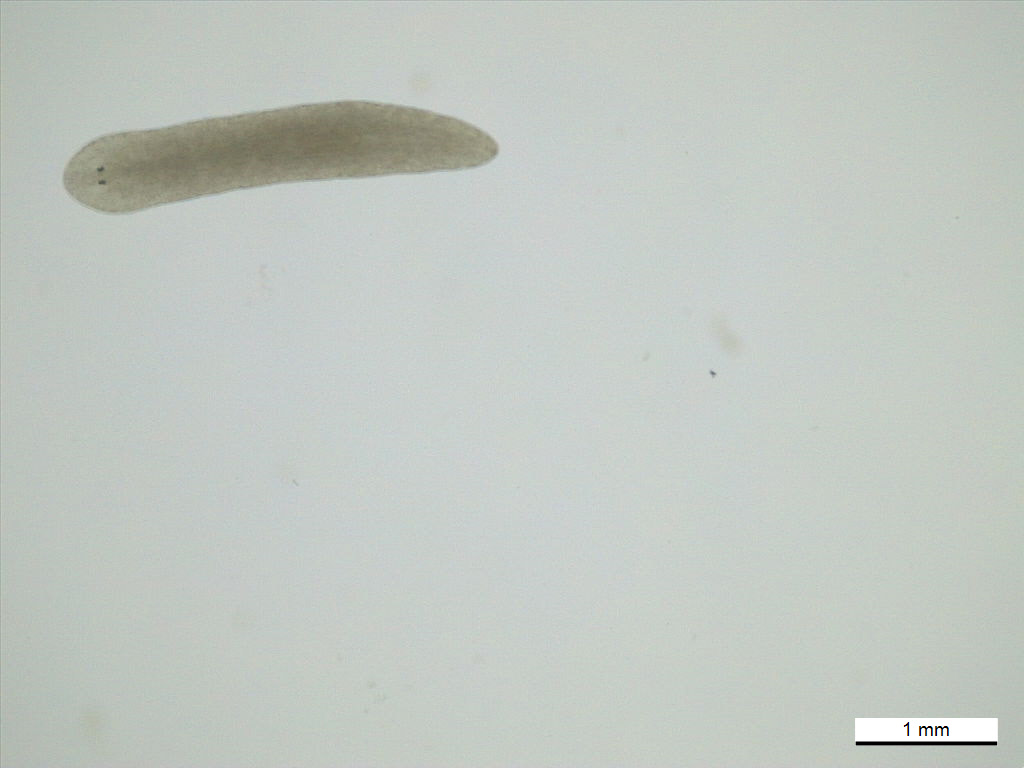

Supplement: Supplementary file 10 — Source data Fig. 3 [file 44318_2025_662_MOESM10_ESM.zip › Figure 3/3C-D/All_ythdf_RNAi_After_10_RNAi_feedings/All_ythdf_RNAi_After_10_RNAi_feedings_7.jpg]

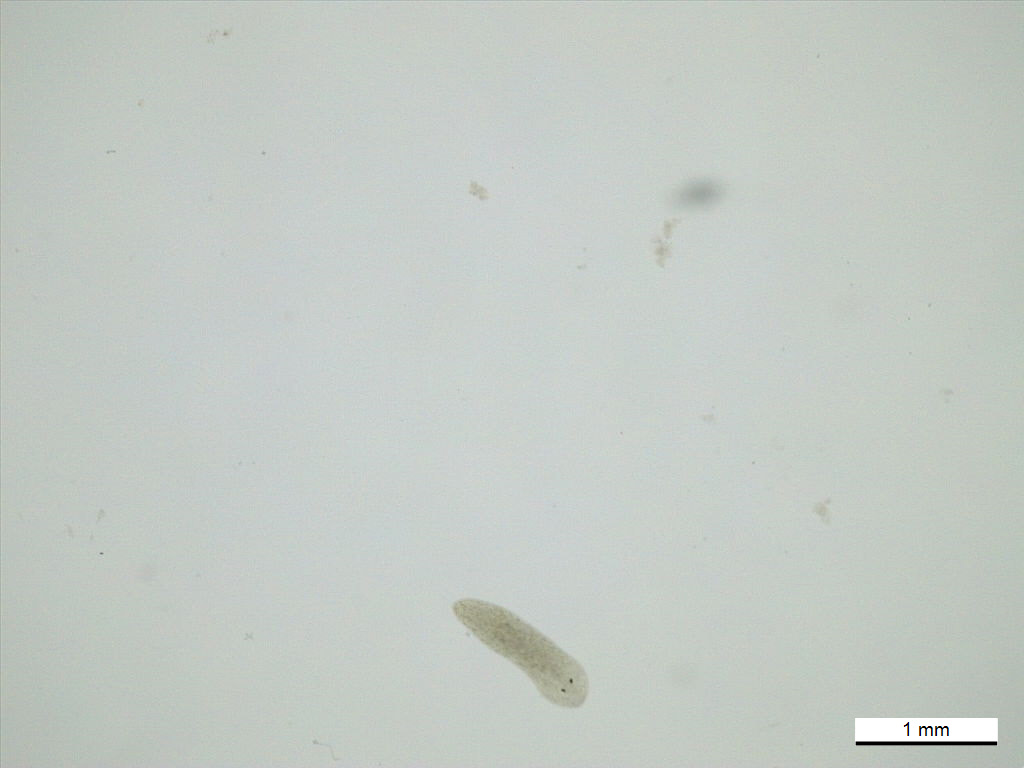

Supplement: Supplementary file 10 — Source data Fig. 3 [file 44318_2025_662_MOESM10_ESM.zip › Figure 3/3C-D/All_ythdf_RNAi_After_10_RNAi_feedings/All_ythdf_RNAi_After_10_RNAi_feedings_8.jpg]

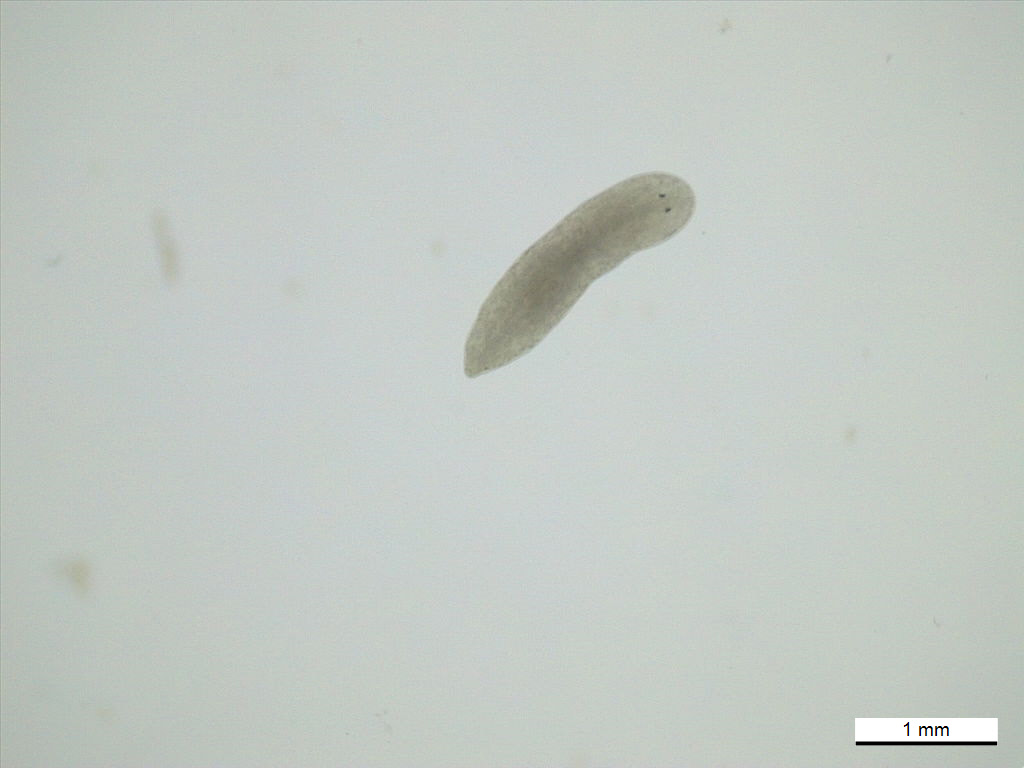

Supplement: Supplementary file 10 — Source data Fig. 3 [file 44318_2025_662_MOESM10_ESM.zip › Figure 3/3C-D/All_ythdf_RNAi_After_10_RNAi_feedings/All_ythdf_RNAi_After_10_RNAi_feedings_9.jpg]

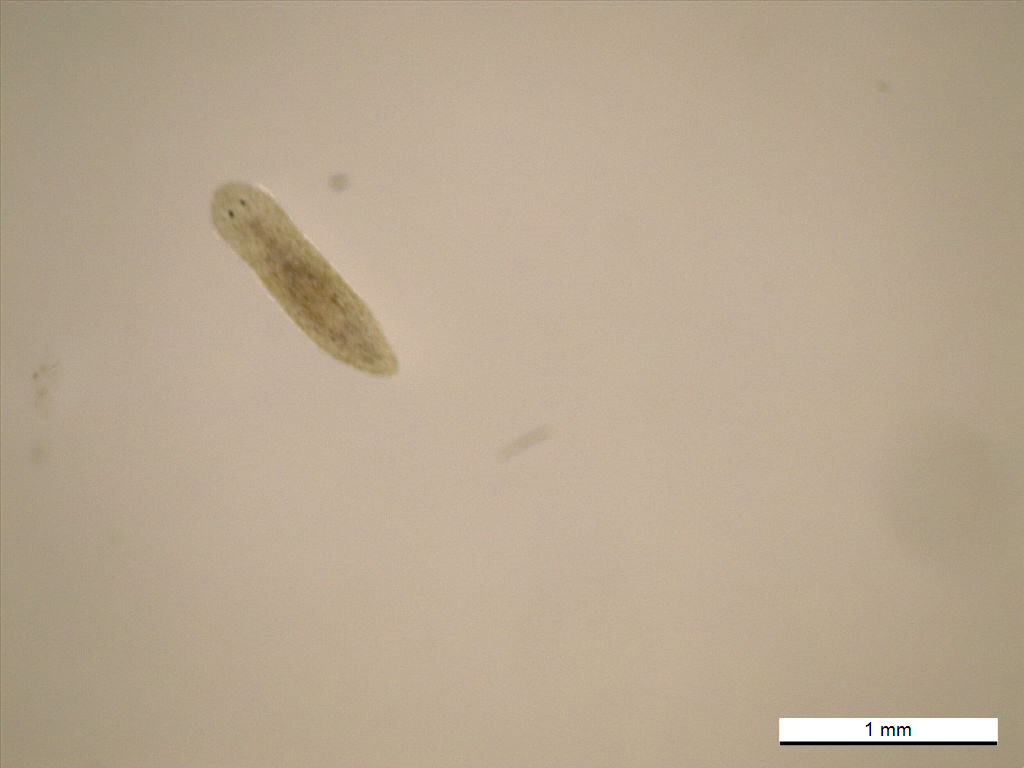

Supplement: Supplementary file 10 — Source data Fig. 3 [file 44318_2025_662_MOESM10_ESM.zip › Figure 3/3C-D/All_ythdf_RNAi_Before_RNAi_feedings/All_ythdf_RNAi_Before_RNAi_feedings_1.jpg]

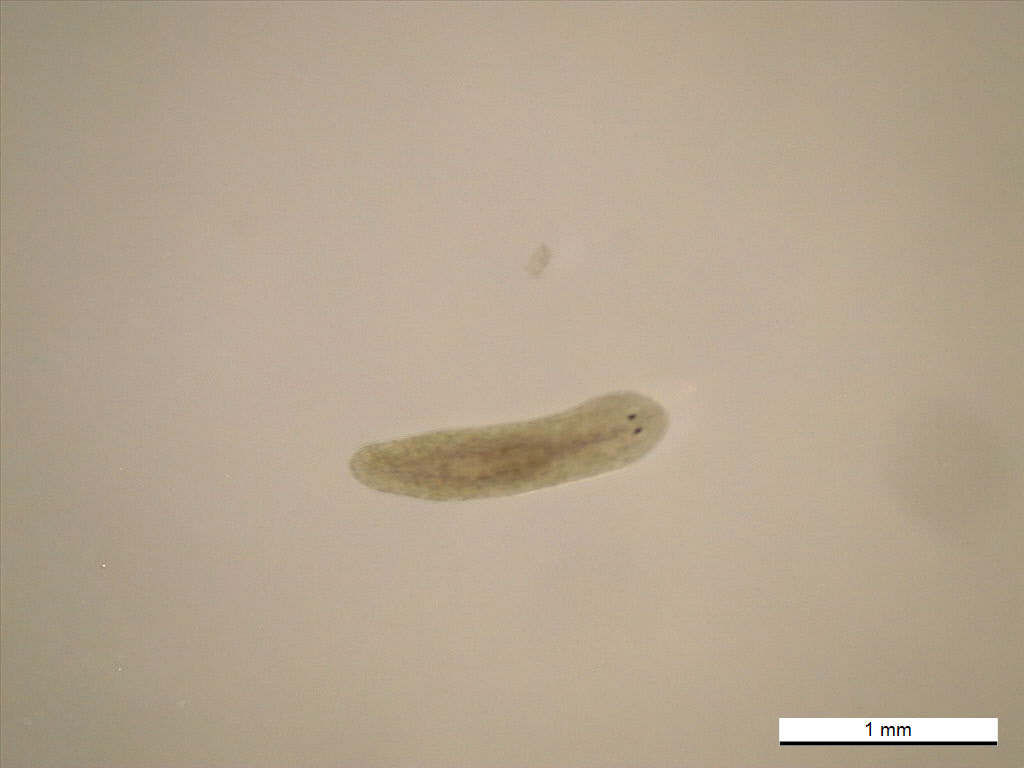

Supplement: Supplementary file 10 — Source data Fig. 3 [file 44318_2025_662_MOESM10_ESM.zip › Figure 3/3C-D/All_ythdf_RNAi_Before_RNAi_feedings/All_ythdf_RNAi_Before_RNAi_feedings_10.jpg]

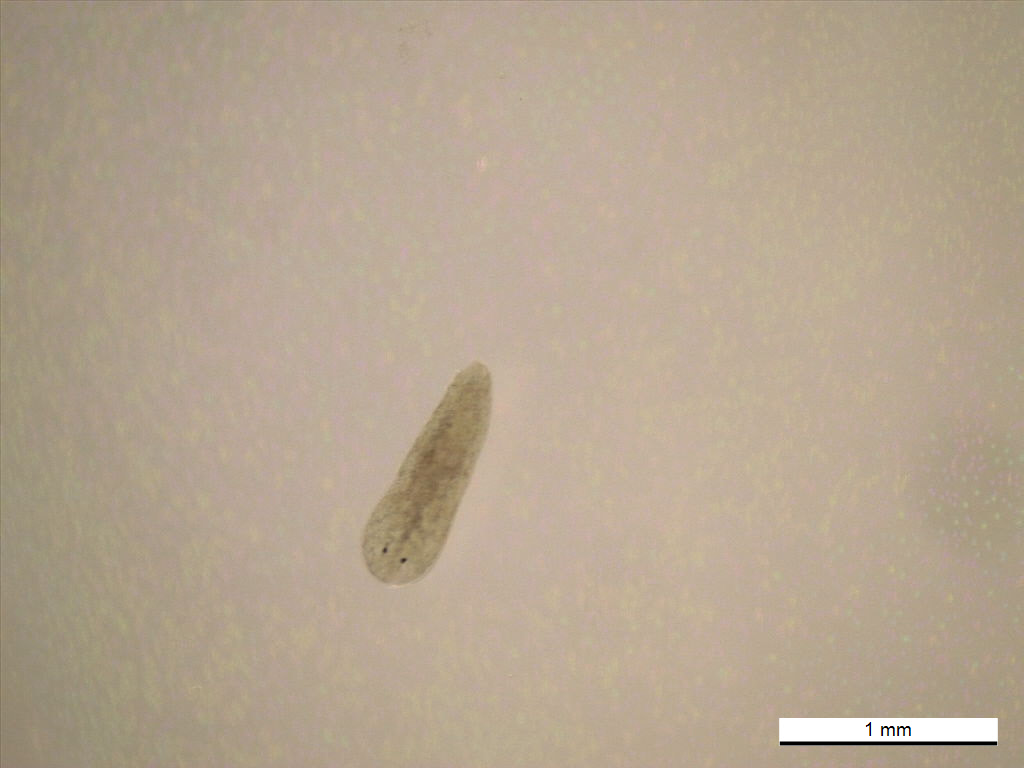

Supplement: Supplementary file 10 — Source data Fig. 3 [file 44318_2025_662_MOESM10_ESM.zip › Figure 3/3C-D/All_ythdf_RNAi_Before_RNAi_feedings/All_ythdf_RNAi_Before_RNAi_feedings_11.jpg]

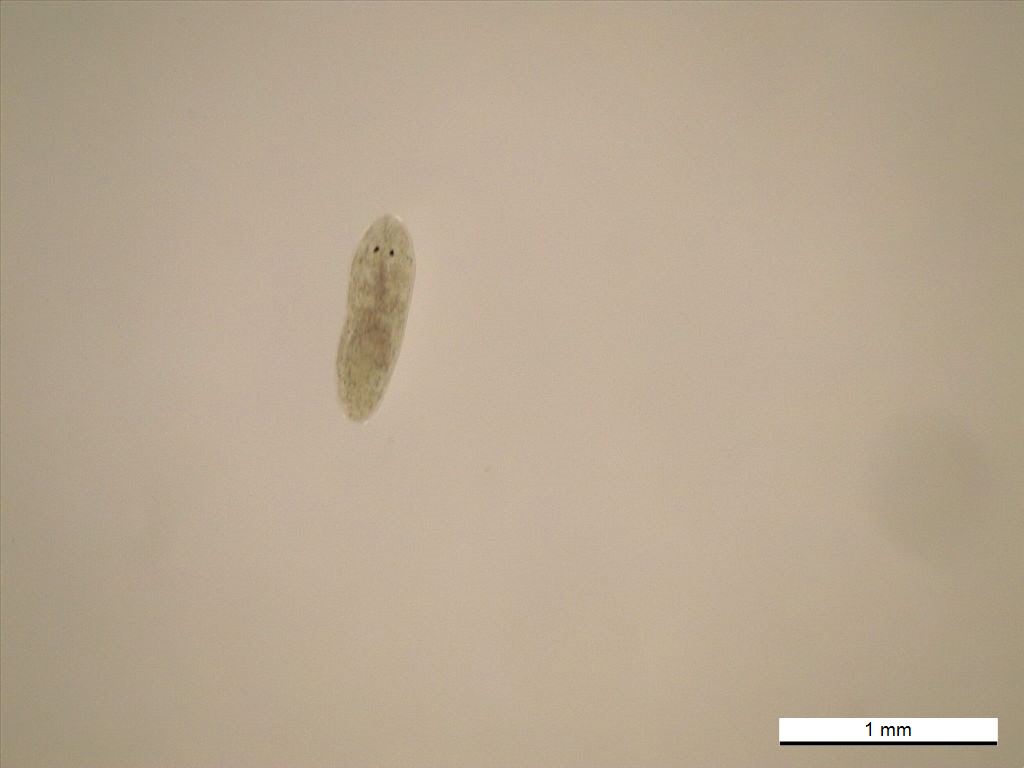

Supplement: Supplementary file 10 — Source data Fig. 3 [file 44318_2025_662_MOESM10_ESM.zip › Figure 3/3C-D/All_ythdf_RNAi_Before_RNAi_feedings/All_ythdf_RNAi_Before_RNAi_feedings_12.jpg]

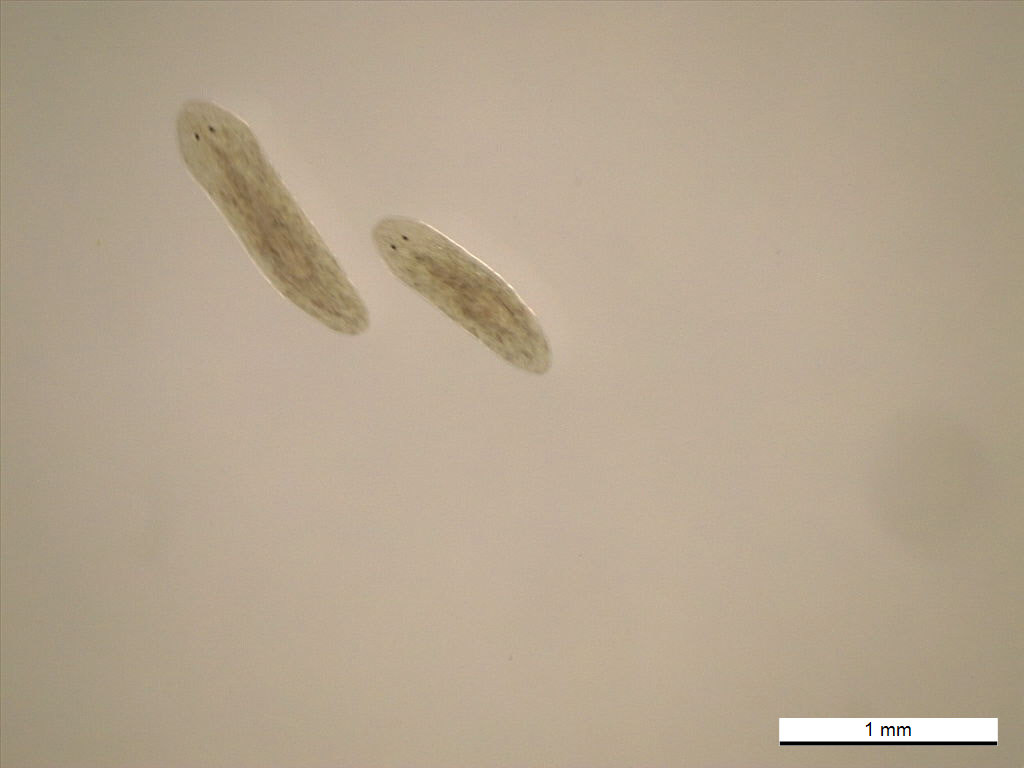

Supplement: Supplementary file 10 — Source data Fig. 3 [file 44318_2025_662_MOESM10_ESM.zip › Figure 3/3C-D/All_ythdf_RNAi_Before_RNAi_feedings/All_ythdf_RNAi_Before_RNAi_feedings_13.jpg]

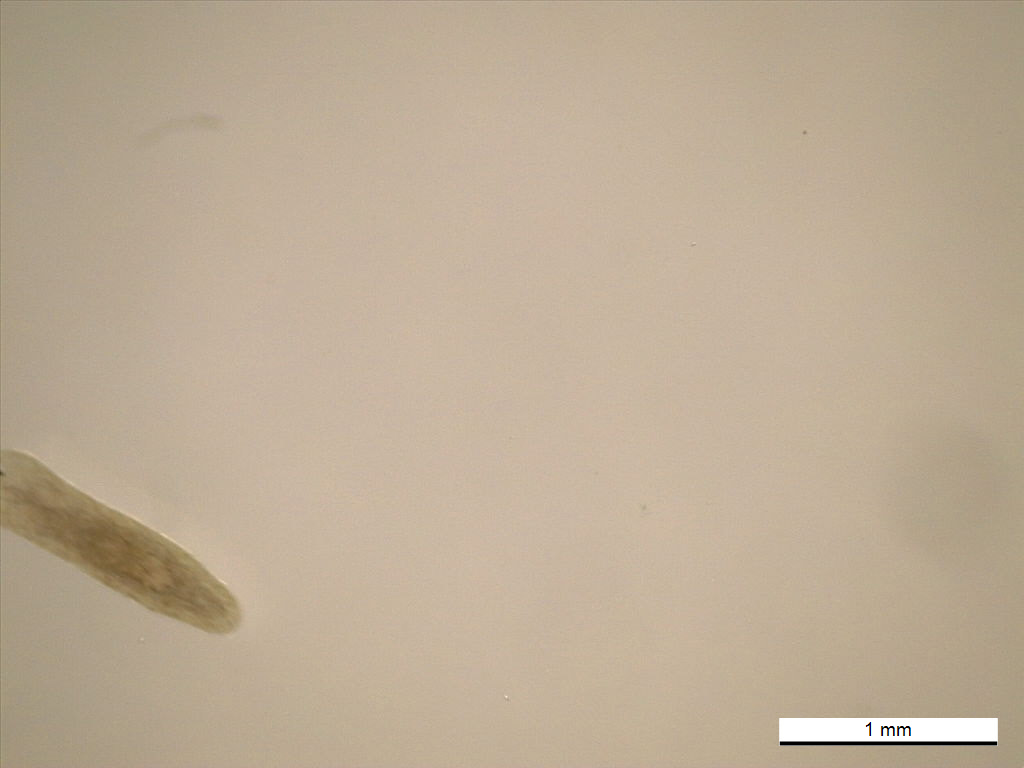

Supplement: Supplementary file 10 — Source data Fig. 3 [file 44318_2025_662_MOESM10_ESM.zip › Figure 3/3C-D/All_ythdf_RNAi_Before_RNAi_feedings/All_ythdf_RNAi_Before_RNAi_feedings_14.jpg]

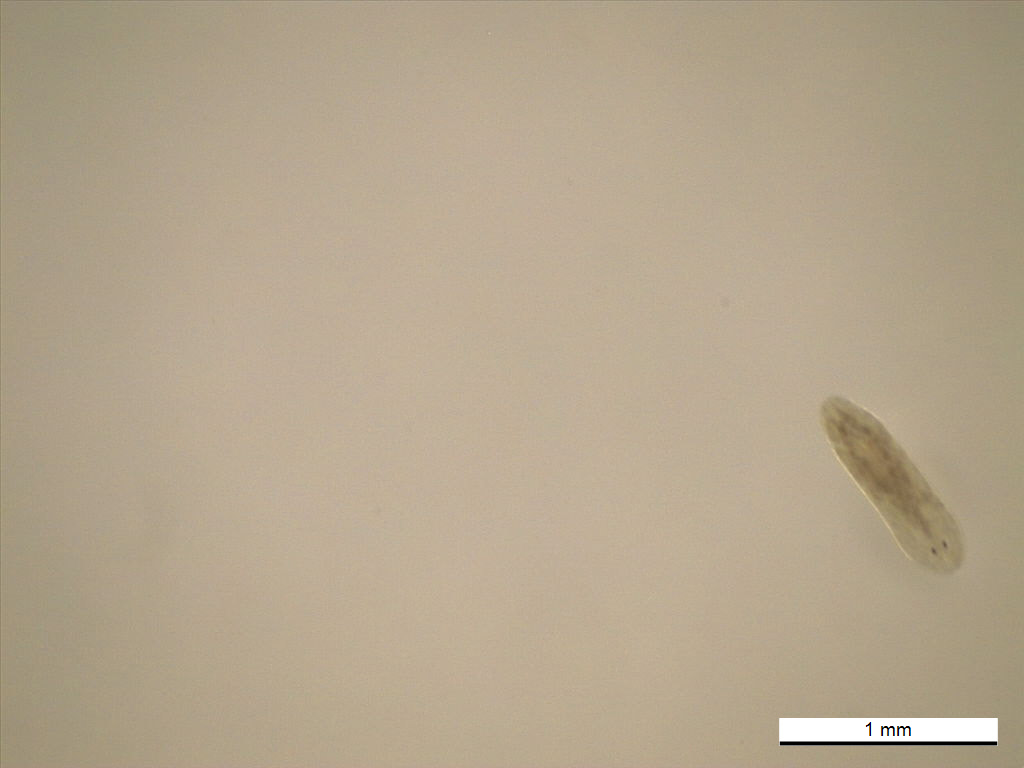

Supplement: Supplementary file 10 — Source data Fig. 3 [file 44318_2025_662_MOESM10_ESM.zip › Figure 3/3C-D/All_ythdf_RNAi_Before_RNAi_feedings/All_ythdf_RNAi_Before_RNAi_feedings_15.jpg]

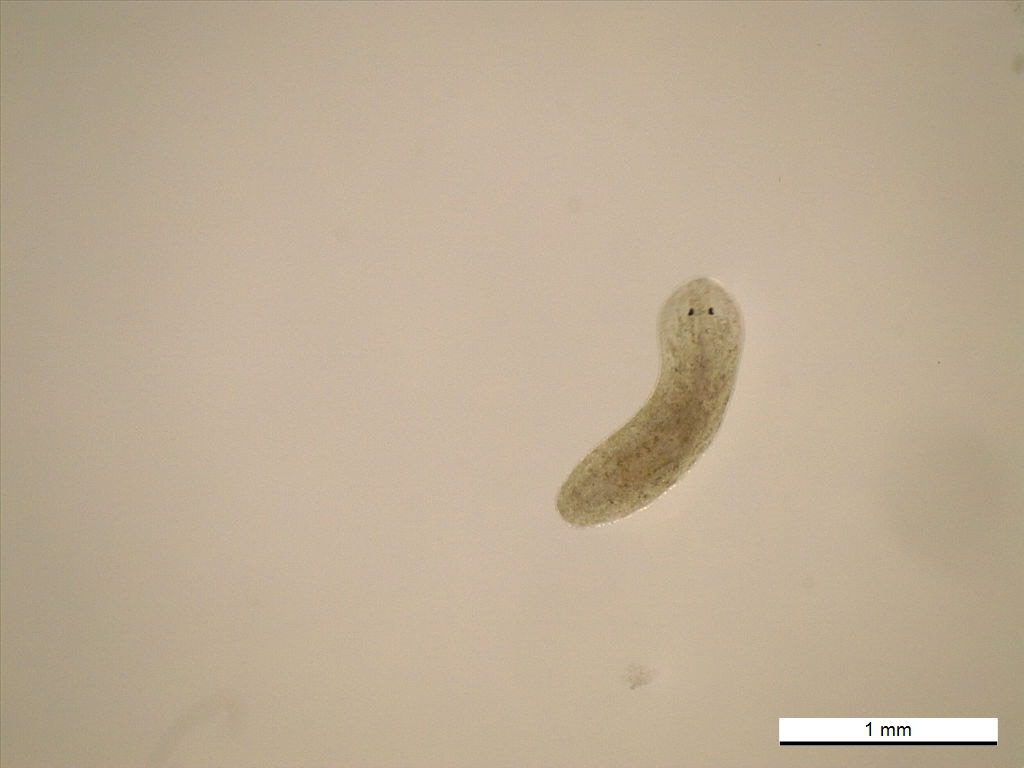

Supplement: Supplementary file 10 — Source data Fig. 3 [file 44318_2025_662_MOESM10_ESM.zip › Figure 3/3C-D/All_ythdf_RNAi_Before_RNAi_feedings/All_ythdf_RNAi_Before_RNAi_feedings_2.jpg]

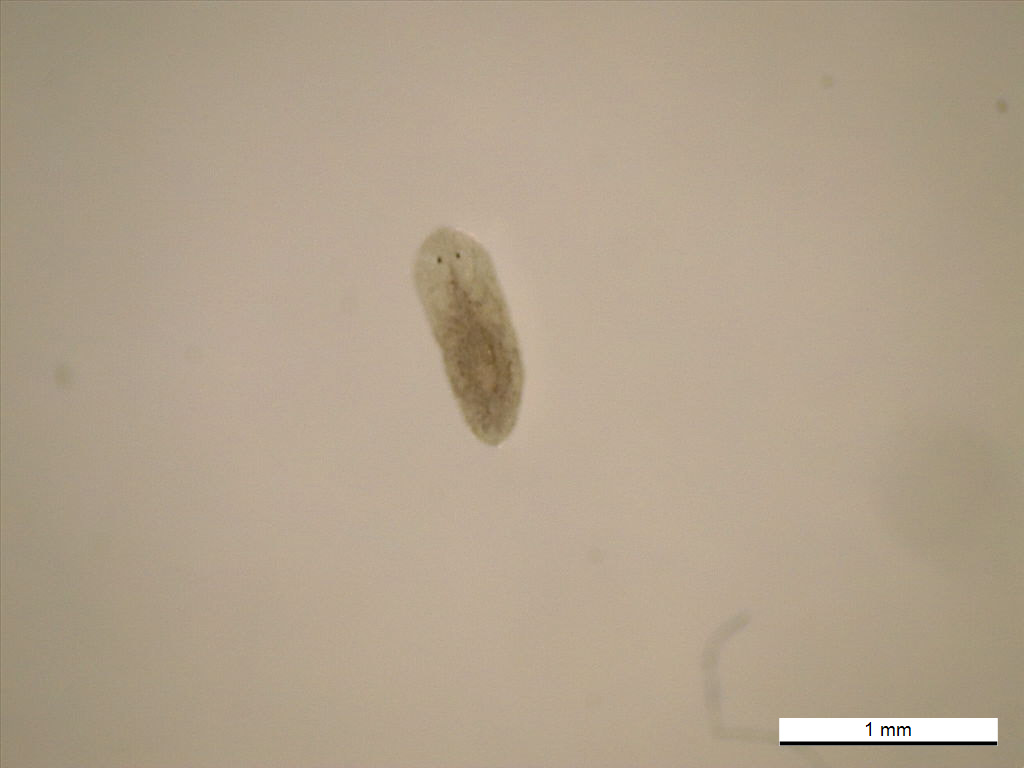

Supplement: Supplementary file 10 — Source data Fig. 3 [file 44318_2025_662_MOESM10_ESM.zip › Figure 3/3C-D/All_ythdf_RNAi_Before_RNAi_feedings/All_ythdf_RNAi_Before_RNAi_feedings_3.jpg]

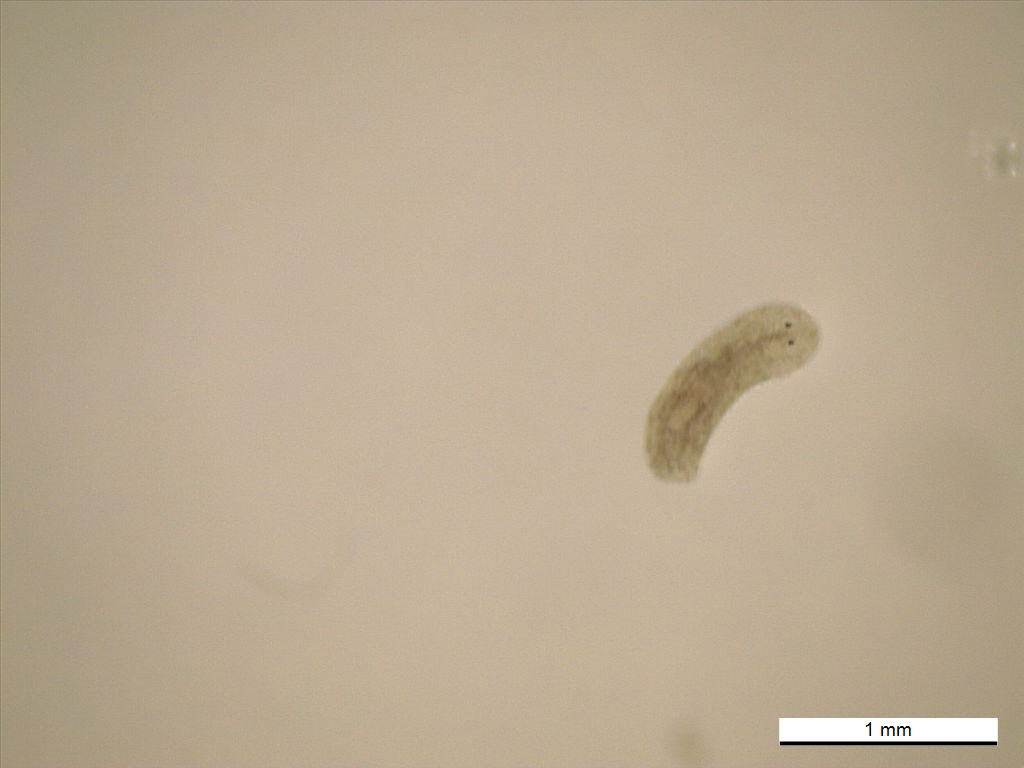

Supplement: Supplementary file 10 — Source data Fig. 3 [file 44318_2025_662_MOESM10_ESM.zip › Figure 3/3C-D/All_ythdf_RNAi_Before_RNAi_feedings/All_ythdf_RNAi_Before_RNAi_feedings_4.jpg]

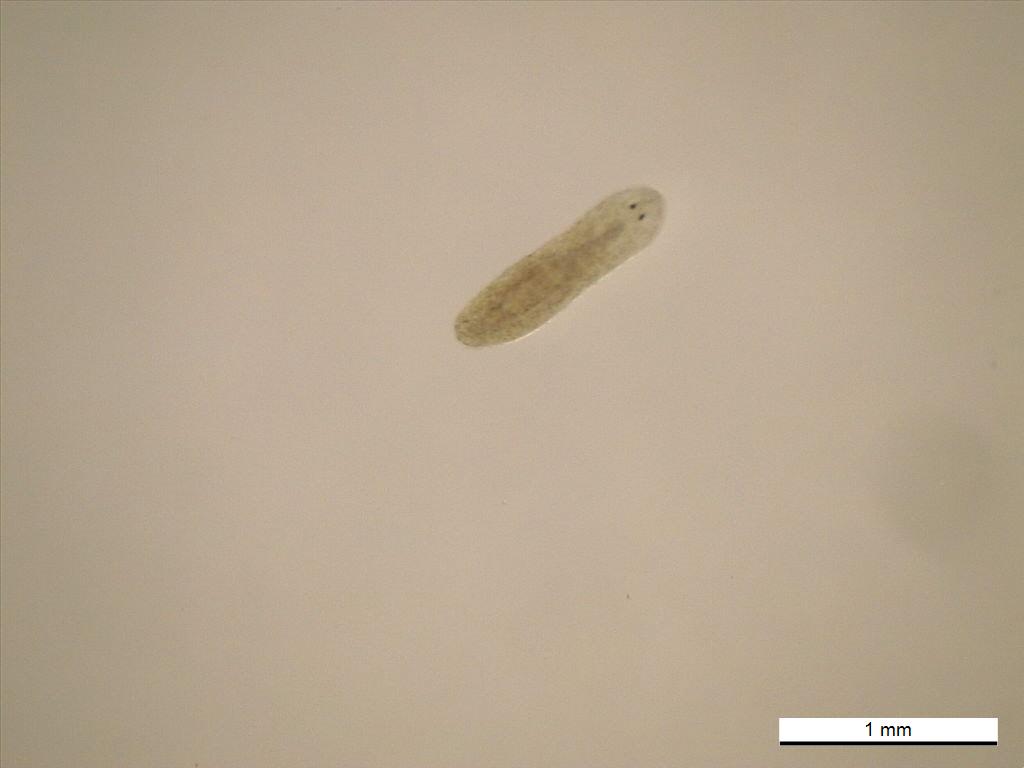

Supplement: Supplementary file 10 — Source data Fig. 3 [file 44318_2025_662_MOESM10_ESM.zip › Figure 3/3C-D/All_ythdf_RNAi_Before_RNAi_feedings/All_ythdf_RNAi_Before_RNAi_feedings_5.jpg]

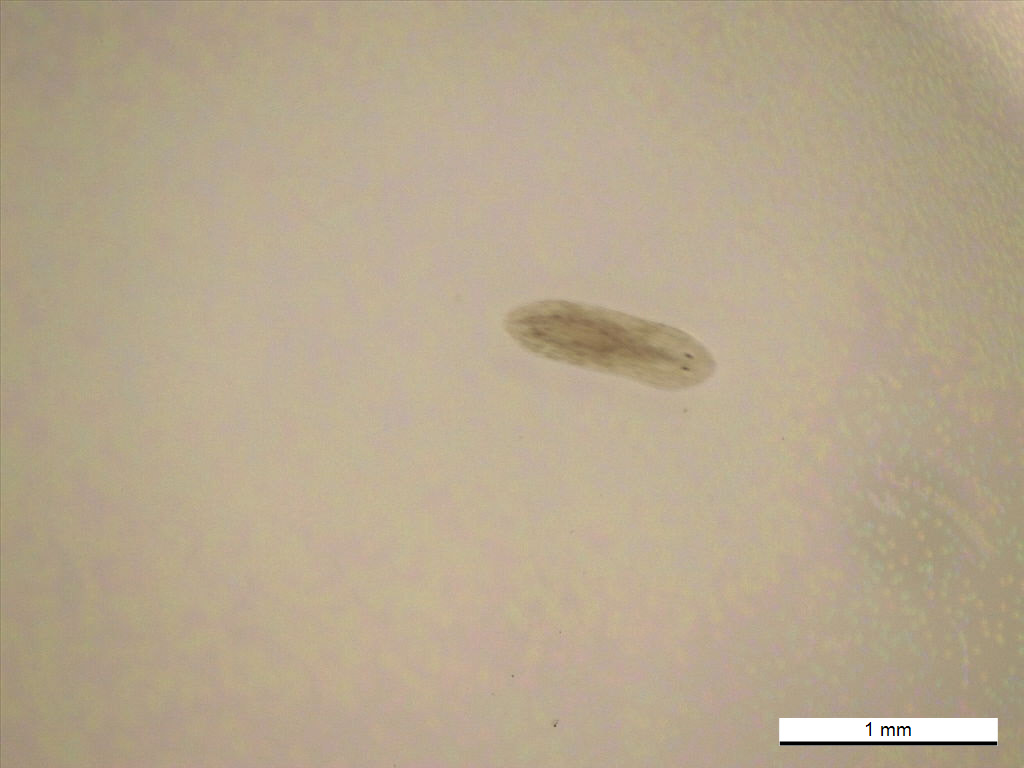

Supplement: Supplementary file 10 — Source data Fig. 3 [file 44318_2025_662_MOESM10_ESM.zip › Figure 3/3C-D/All_ythdf_RNAi_Before_RNAi_feedings/All_ythdf_RNAi_Before_RNAi_feedings_6.jpg]

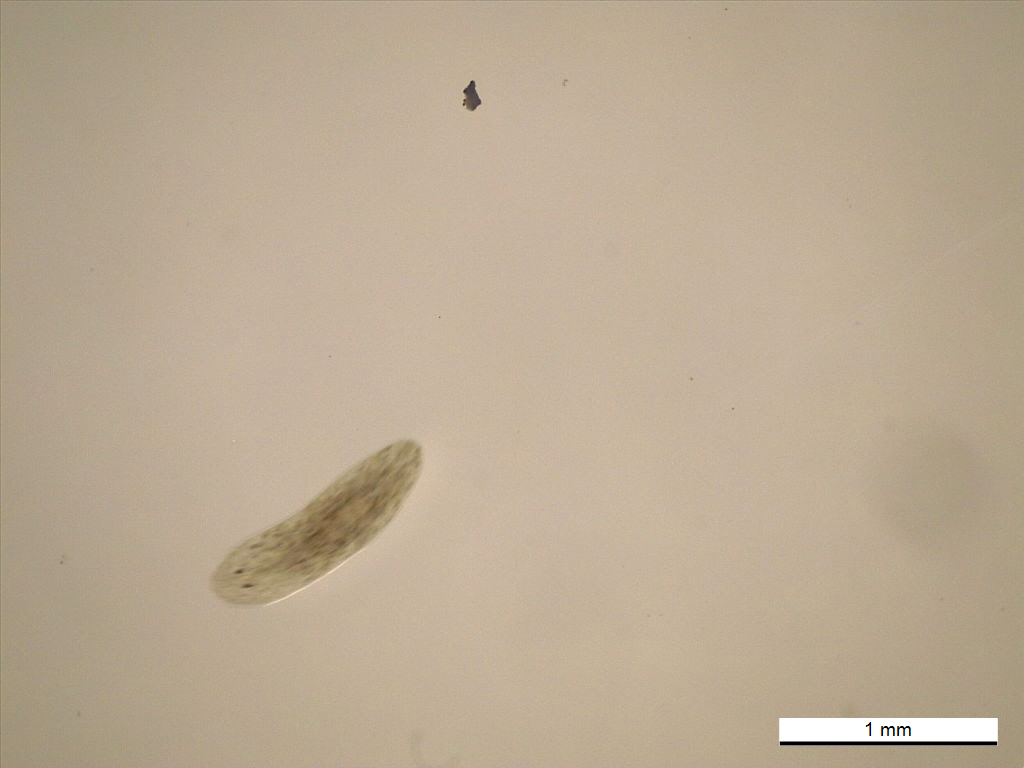

Supplement: Supplementary file 10 — Source data Fig. 3 [file 44318_2025_662_MOESM10_ESM.zip › Figure 3/3C-D/All_ythdf_RNAi_Before_RNAi_feedings/All_ythdf_RNAi_Before_RNAi_feedings_7.jpg]

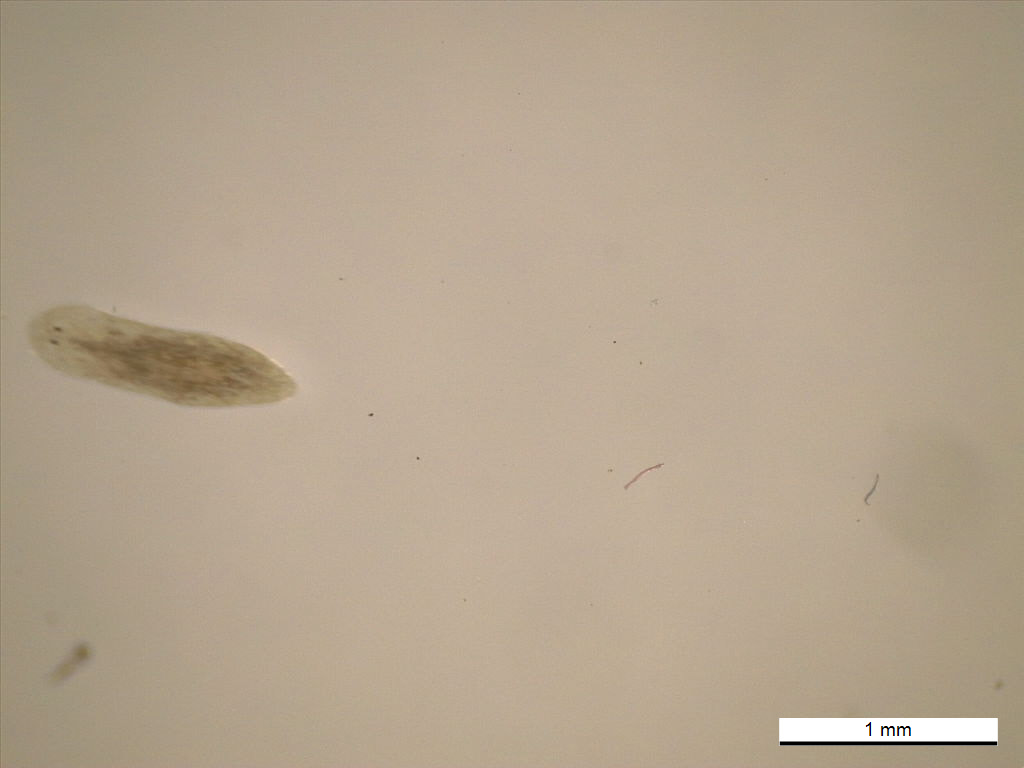

Supplement: Supplementary file 10 — Source data Fig. 3 [file 44318_2025_662_MOESM10_ESM.zip › Figure 3/3C-D/All_ythdf_RNAi_Before_RNAi_feedings/All_ythdf_RNAi_Before_RNAi_feedings_8.jpg]

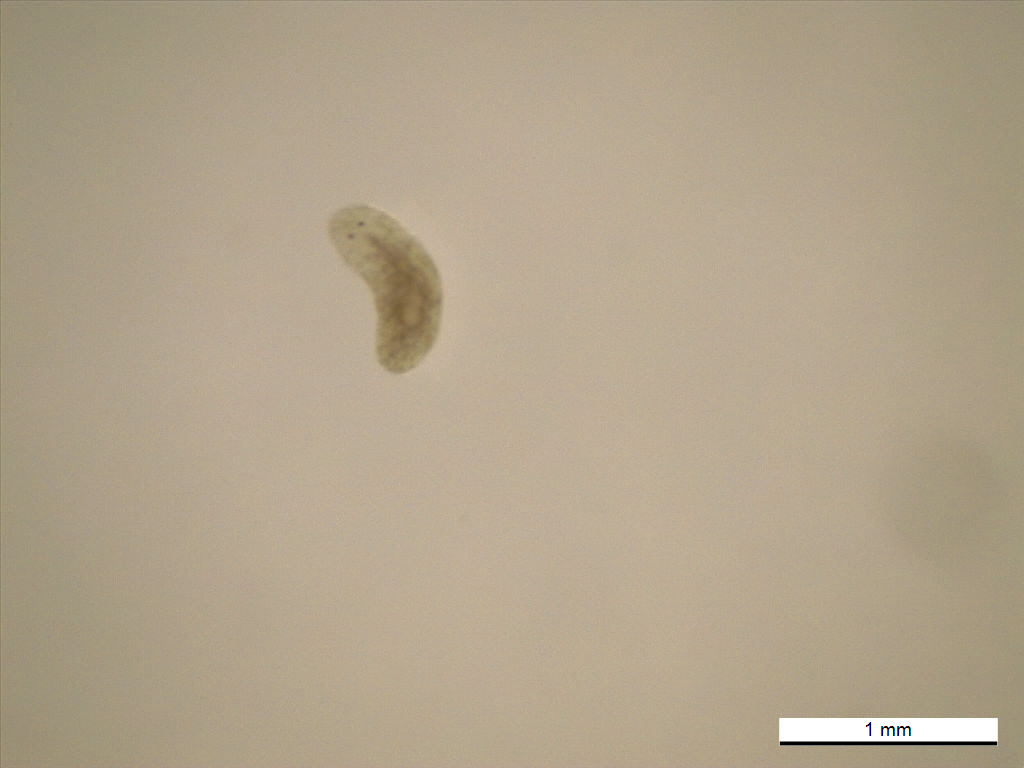

Supplement: Supplementary file 10 — Source data Fig. 3 [file 44318_2025_662_MOESM10_ESM.zip › Figure 3/3C-D/All_ythdf_RNAi_Before_RNAi_feedings/All_ythdf_RNAi_Before_RNAi_feedings_9.jpg]

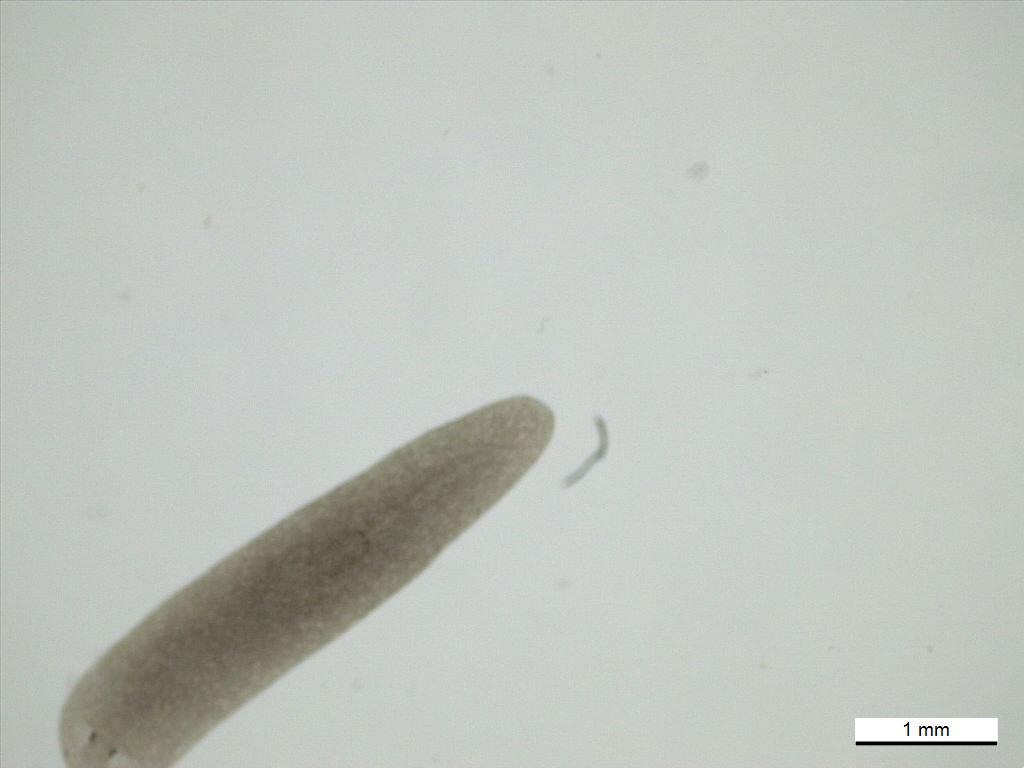

Supplement: Supplementary file 10 — Source data Fig. 3 [file 44318_2025_662_MOESM10_ESM.zip › Figure 3/3C-D/Control_After_10_RNAi_feedings/Control_After_10_RNAi_feedings_01.jpg]

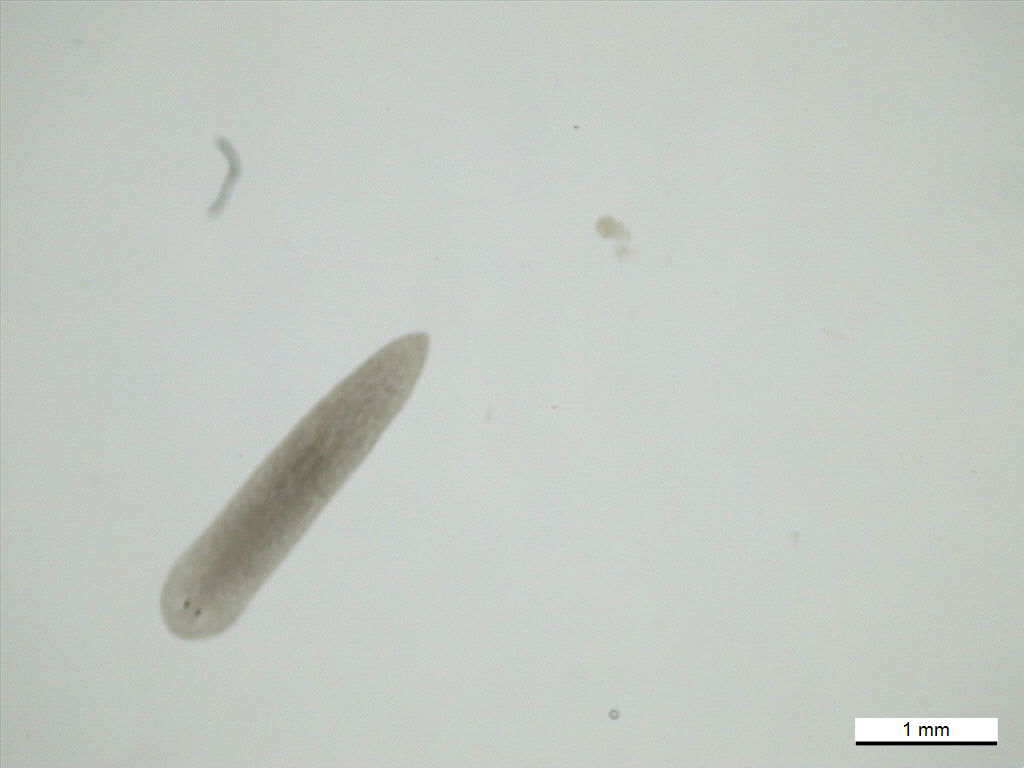

Supplement: Supplementary file 10 — Source data Fig. 3 [file 44318_2025_662_MOESM10_ESM.zip › Figure 3/3C-D/Control_After_10_RNAi_feedings/Control_After_10_RNAi_feedings_02.jpg]

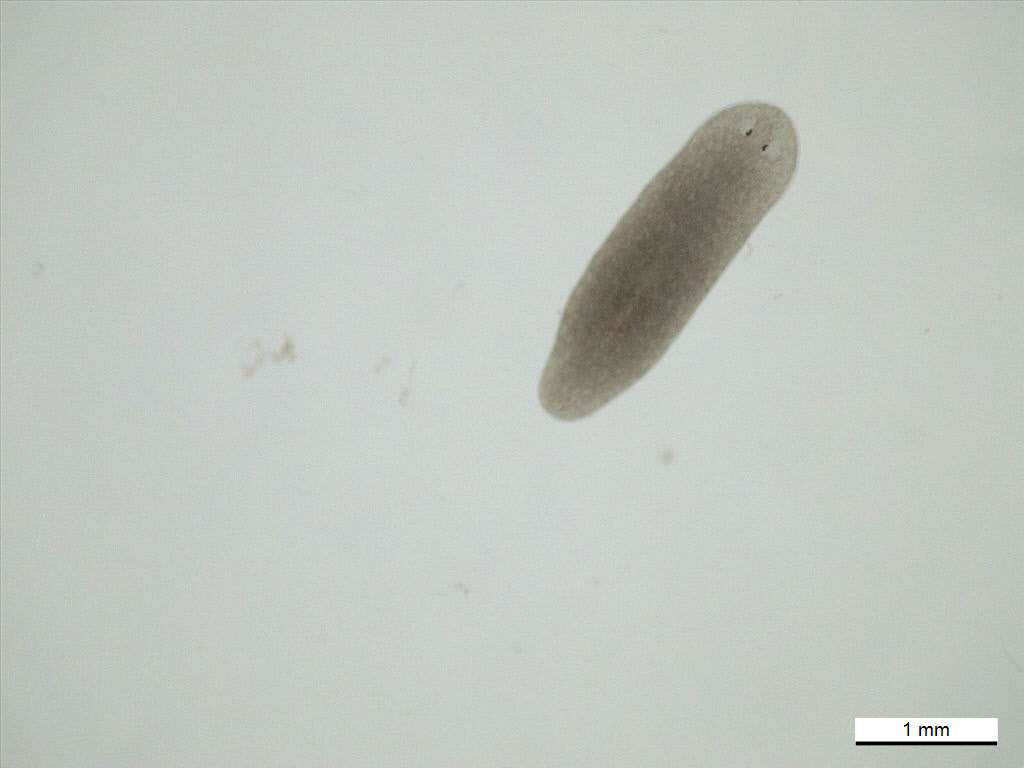

Supplement: Supplementary file 10 — Source data Fig. 3 [file 44318_2025_662_MOESM10_ESM.zip › Figure 3/3C-D/Control_After_10_RNAi_feedings/Control_After_10_RNAi_feedings_03.jpg]

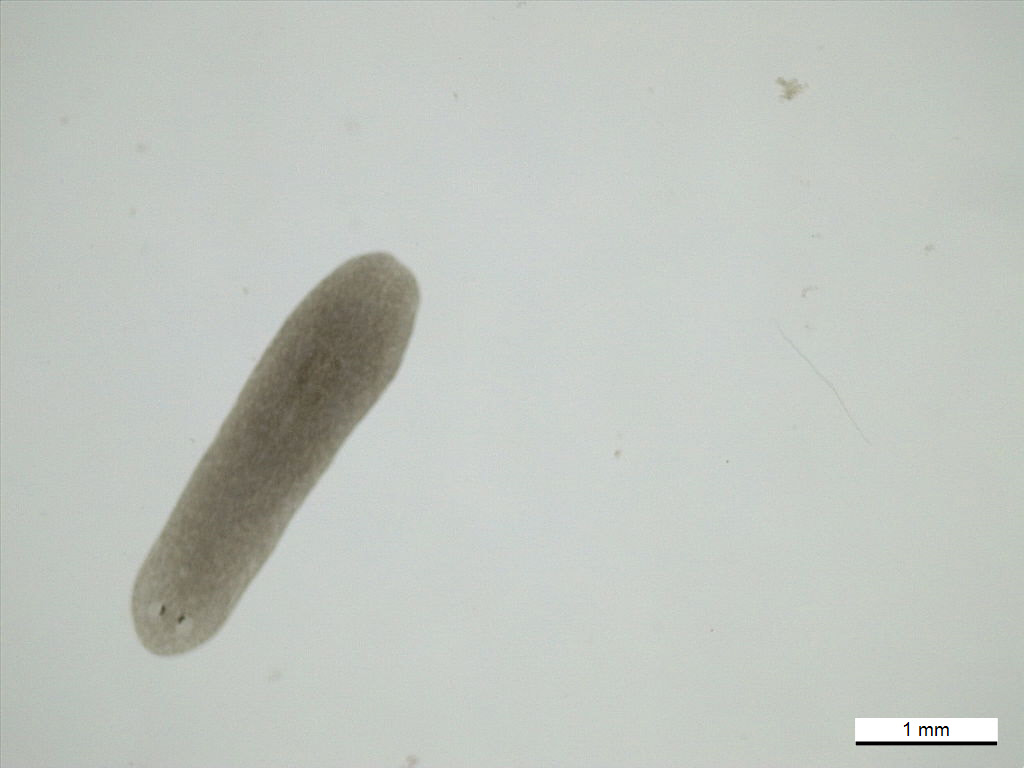

Supplement: Supplementary file 10 — Source data Fig. 3 [file 44318_2025_662_MOESM10_ESM.zip › Figure 3/3C-D/Control_After_10_RNAi_feedings/Control_After_10_RNAi_feedings_04.jpg]

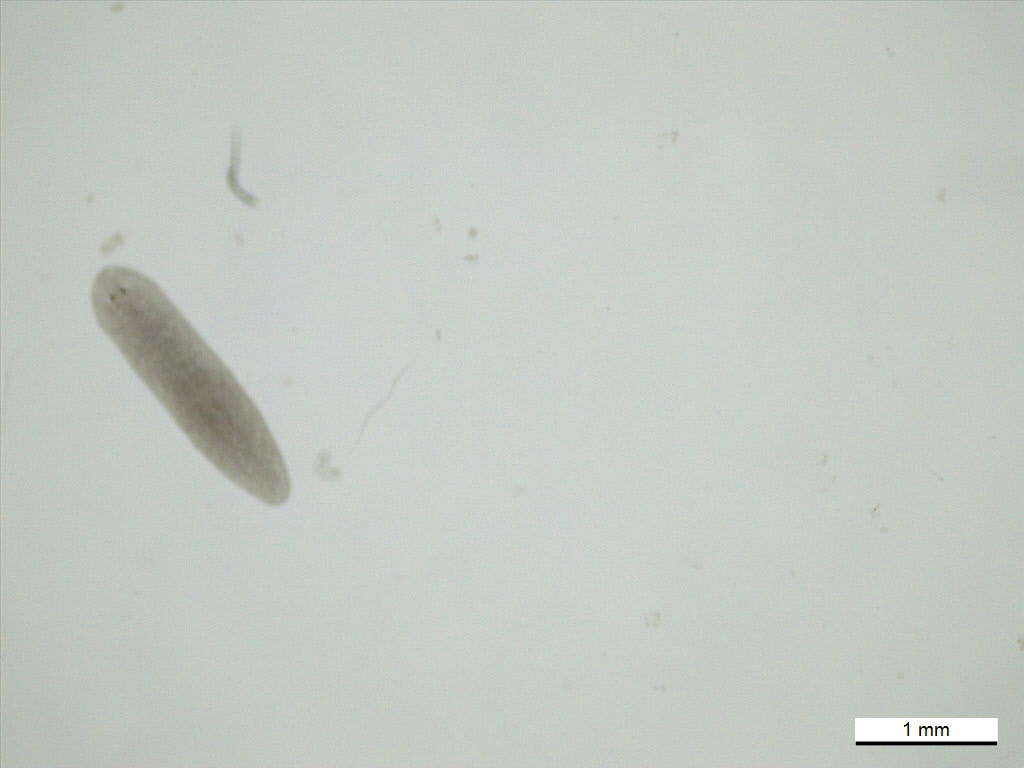

Supplement: Supplementary file 10 — Source data Fig. 3 [file 44318_2025_662_MOESM10_ESM.zip › Figure 3/3C-D/Control_After_10_RNAi_feedings/Control_After_10_RNAi_feedings_05.jpg]

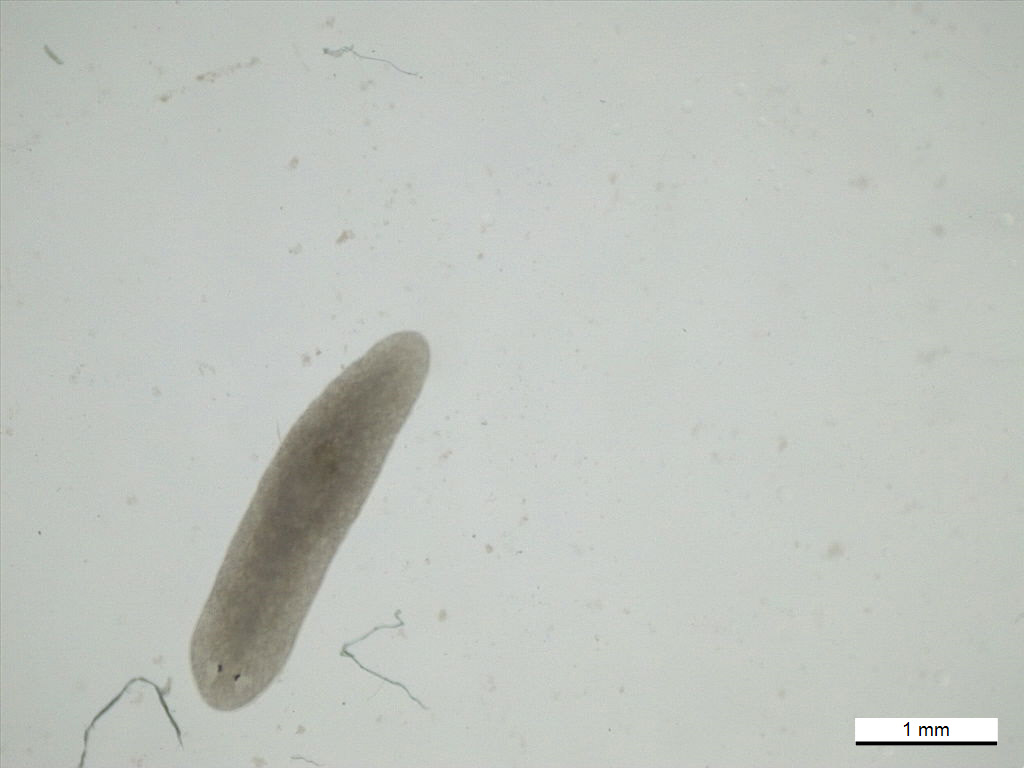

Supplement: Supplementary file 10 — Source data Fig. 3 [file 44318_2025_662_MOESM10_ESM.zip › Figure 3/3C-D/Control_After_10_RNAi_feedings/Control_After_10_RNAi_feedings_06.jpg]

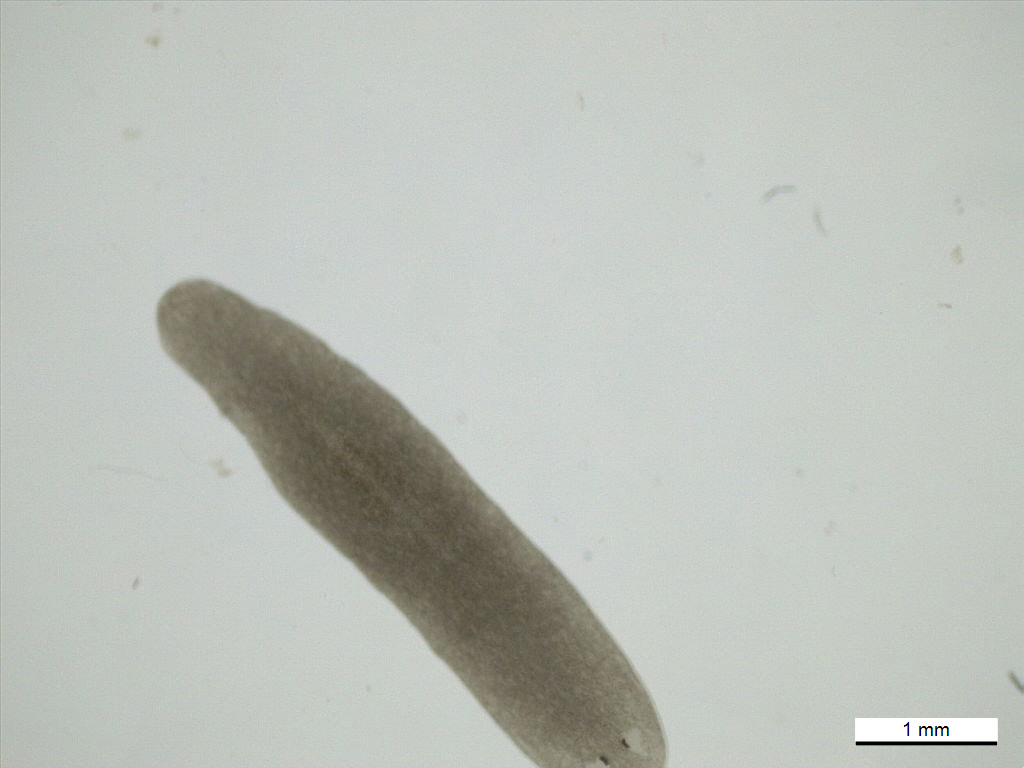

Supplement: Supplementary file 10 — Source data Fig. 3 [file 44318_2025_662_MOESM10_ESM.zip › Figure 3/3C-D/Control_After_10_RNAi_feedings/Control_After_10_RNAi_feedings_07.jpg]

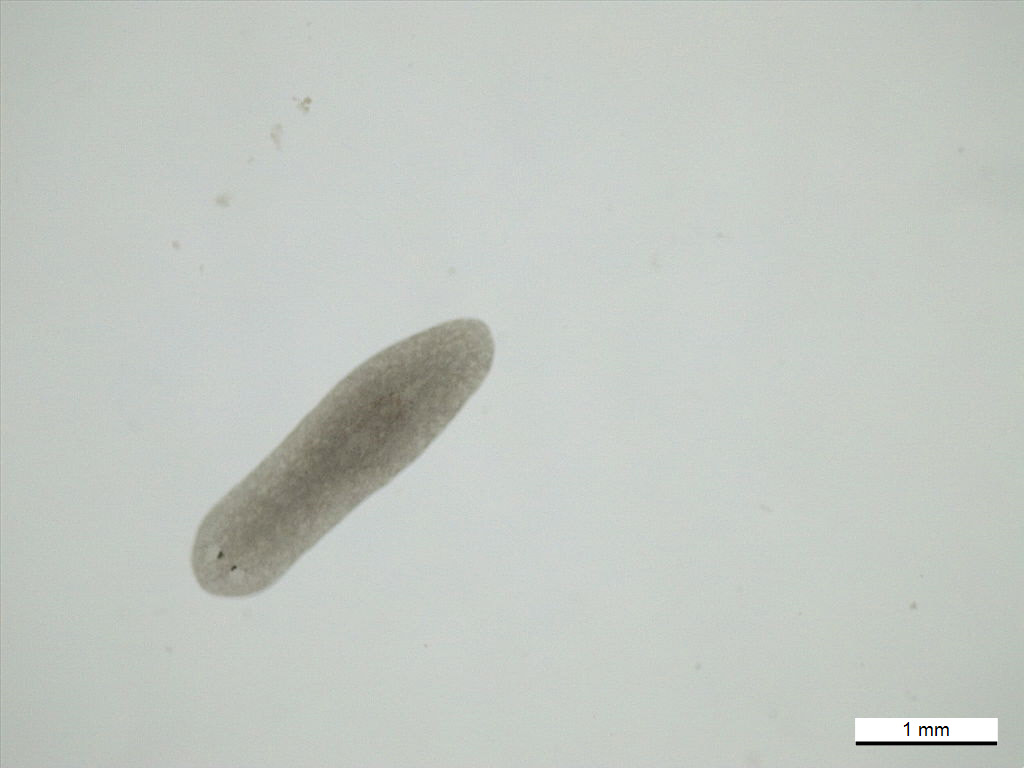

Supplement: Supplementary file 10 — Source data Fig. 3 [file 44318_2025_662_MOESM10_ESM.zip › Figure 3/3C-D/Control_After_10_RNAi_feedings/Control_After_10_RNAi_feedings_08.jpg]

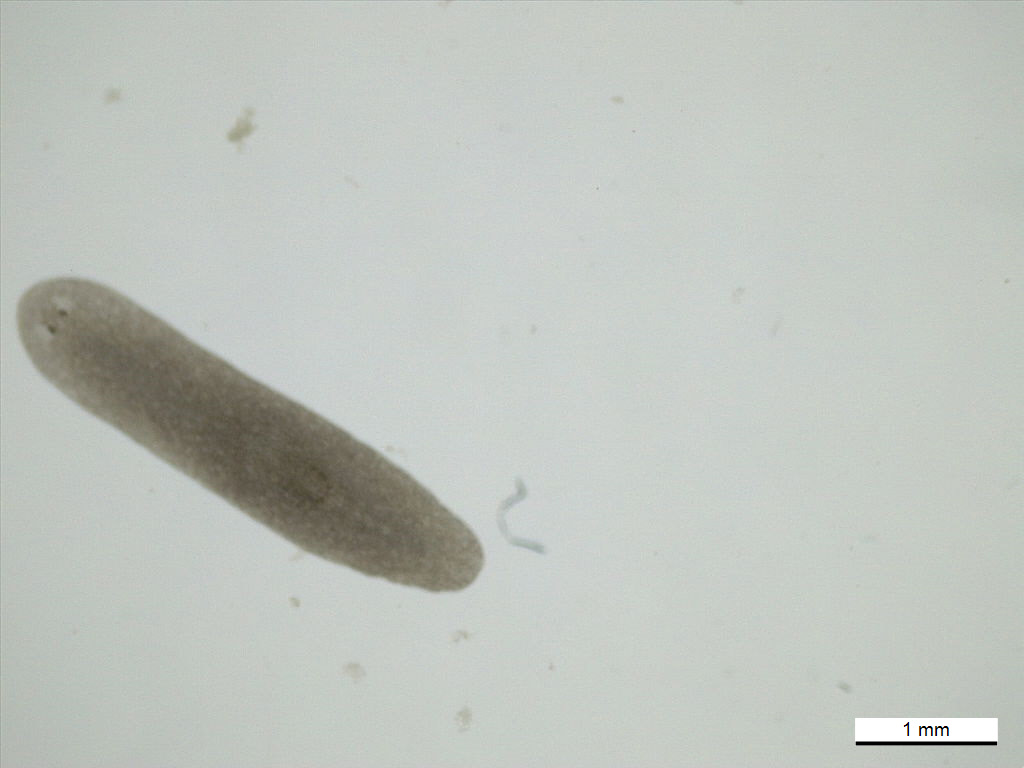

Supplement: Supplementary file 10 — Source data Fig. 3 [file 44318_2025_662_MOESM10_ESM.zip › Figure 3/3C-D/Control_After_10_RNAi_feedings/Control_After_10_RNAi_feedings_09.jpg]

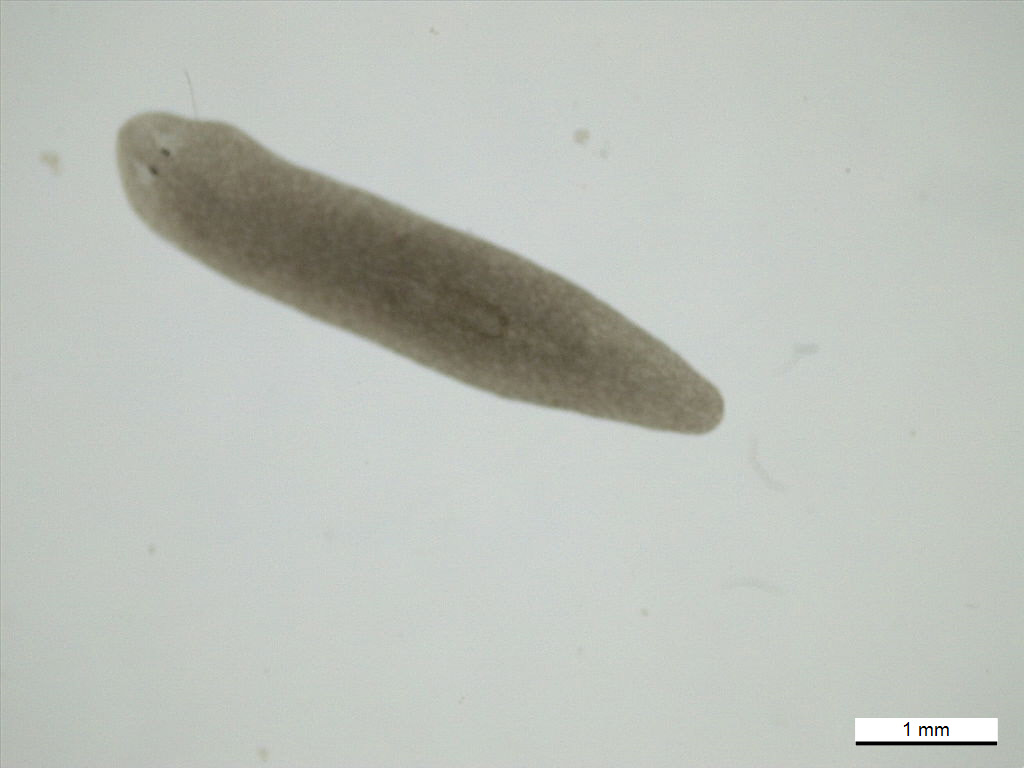

Supplement: Supplementary file 10 — Source data Fig. 3 [file 44318_2025_662_MOESM10_ESM.zip › Figure 3/3C-D/Control_After_10_RNAi_feedings/Control_After_10_RNAi_feedings_10.jpg]

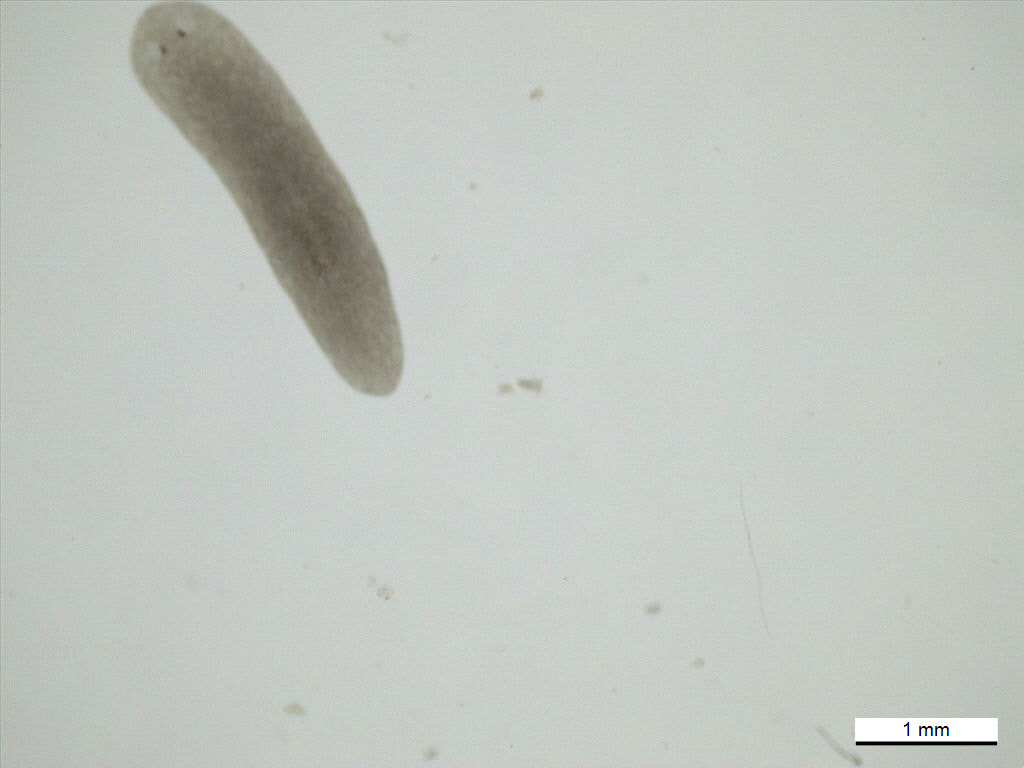

Supplement: Supplementary file 10 — Source data Fig. 3 [file 44318_2025_662_MOESM10_ESM.zip › Figure 3/3C-D/Control_After_10_RNAi_feedings/Control_After_10_RNAi_feedings_11.jpg]

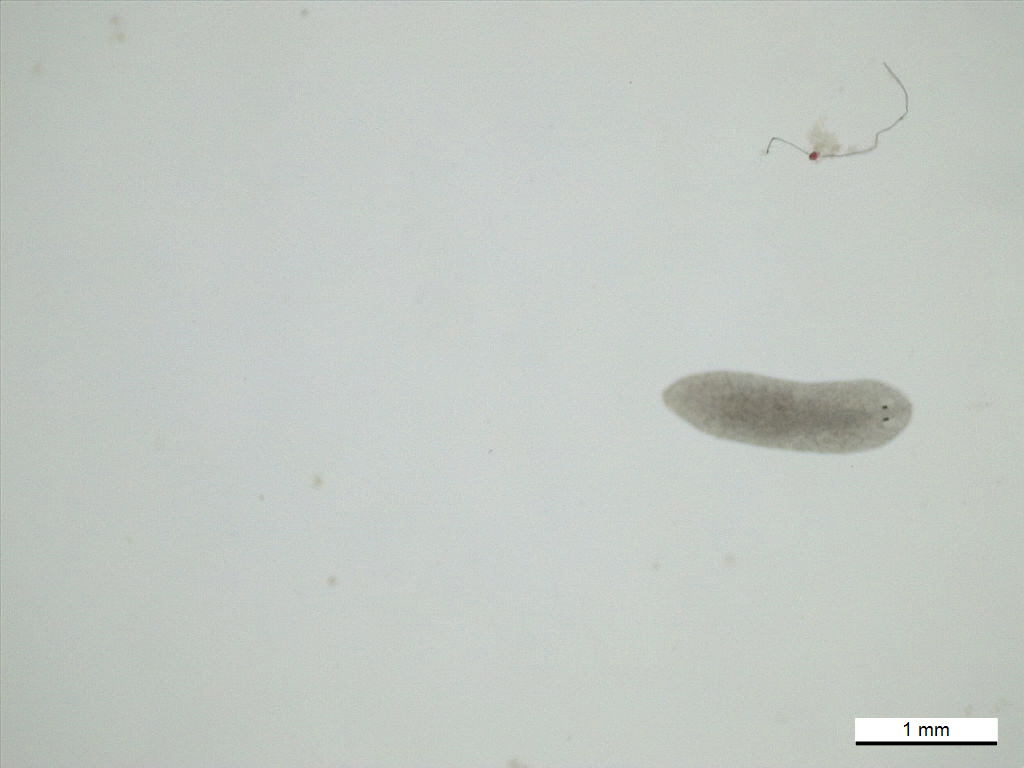

Supplement: Supplementary file 10 — Source data Fig. 3 [file 44318_2025_662_MOESM10_ESM.zip › Figure 3/3C-D/Control_After_10_RNAi_feedings/Control_After_10_RNAi_feedings_12.jpg]

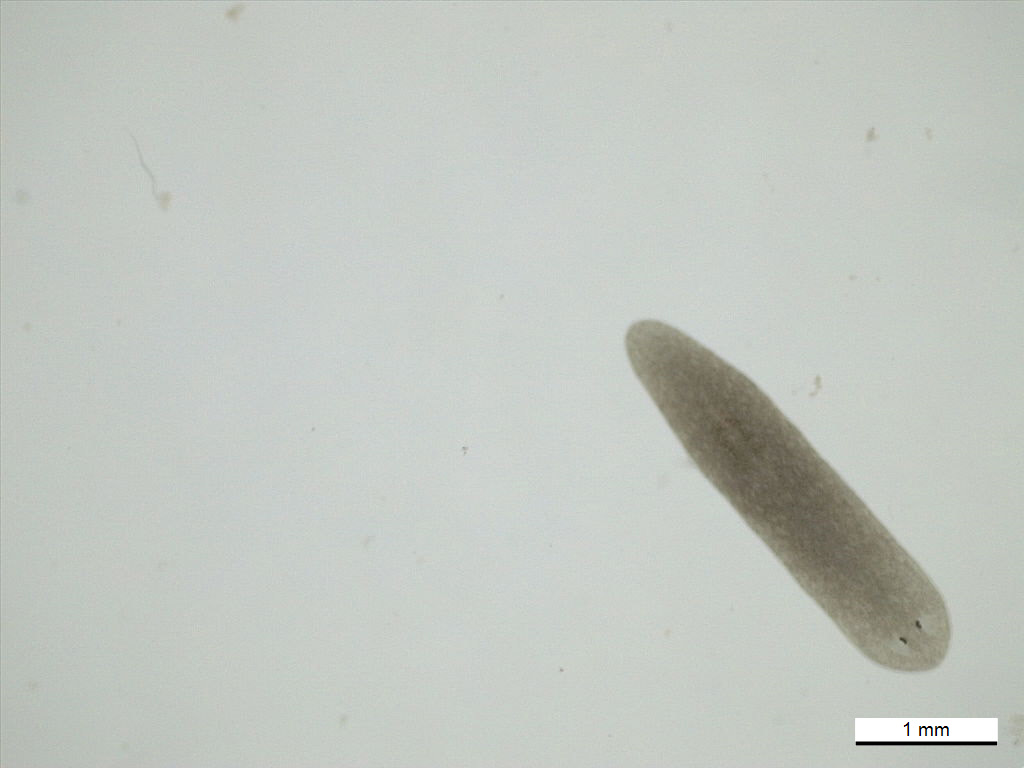

Supplement: Supplementary file 10 — Source data Fig. 3 [file 44318_2025_662_MOESM10_ESM.zip › Figure 3/3C-D/Control_After_10_RNAi_feedings/Control_After_10_RNAi_feedings_13.jpg]

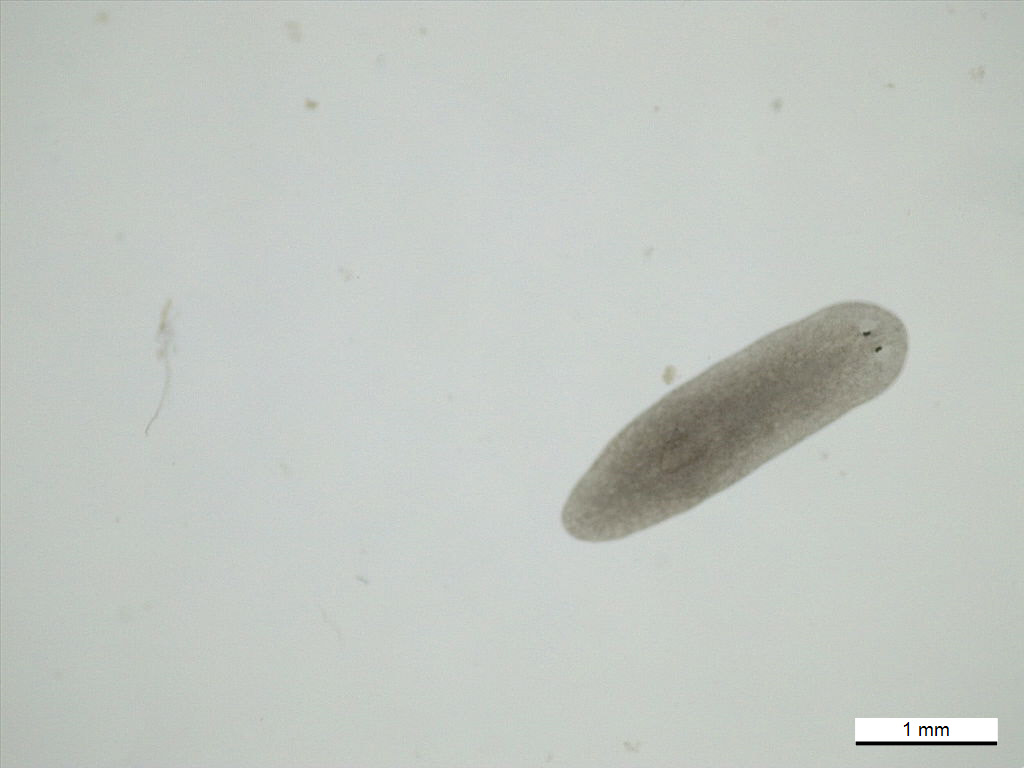

Supplement: Supplementary file 10 — Source data Fig. 3 [file 44318_2025_662_MOESM10_ESM.zip › Figure 3/3C-D/Control_After_10_RNAi_feedings/Control_After_10_RNAi_feedings_14.jpg]

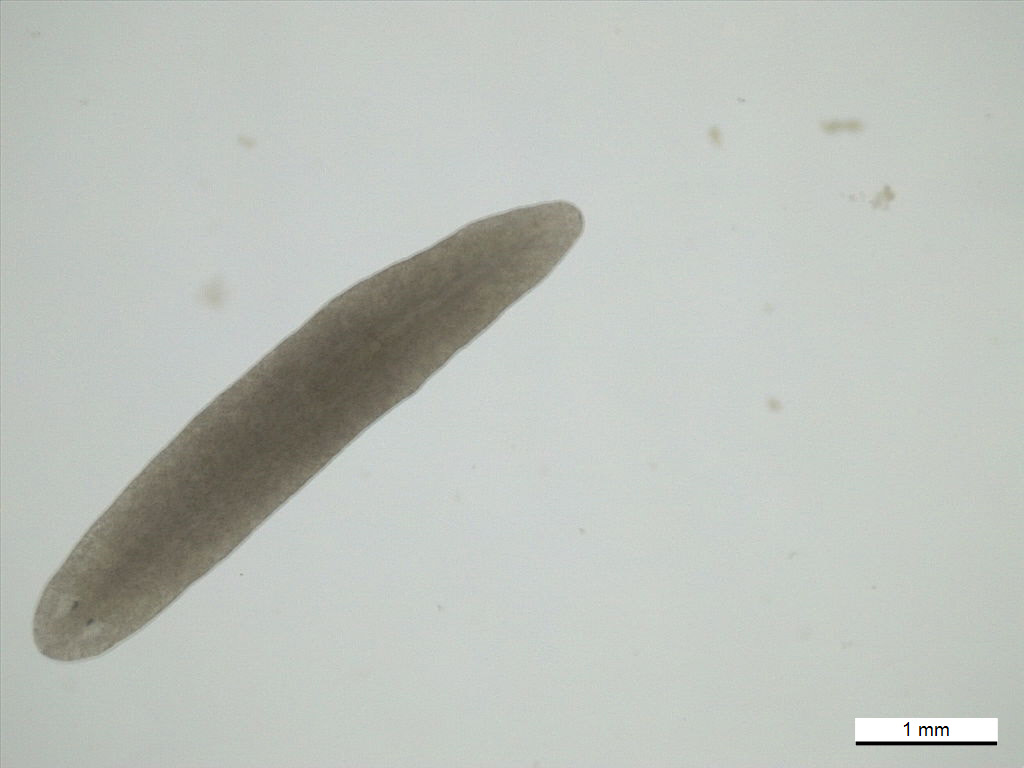

Supplement: Supplementary file 10 — Source data Fig. 3 [file 44318_2025_662_MOESM10_ESM.zip › Figure 3/3C-D/Control_After_10_RNAi_feedings/Control_After_10_RNAi_feedings_15.jpg]

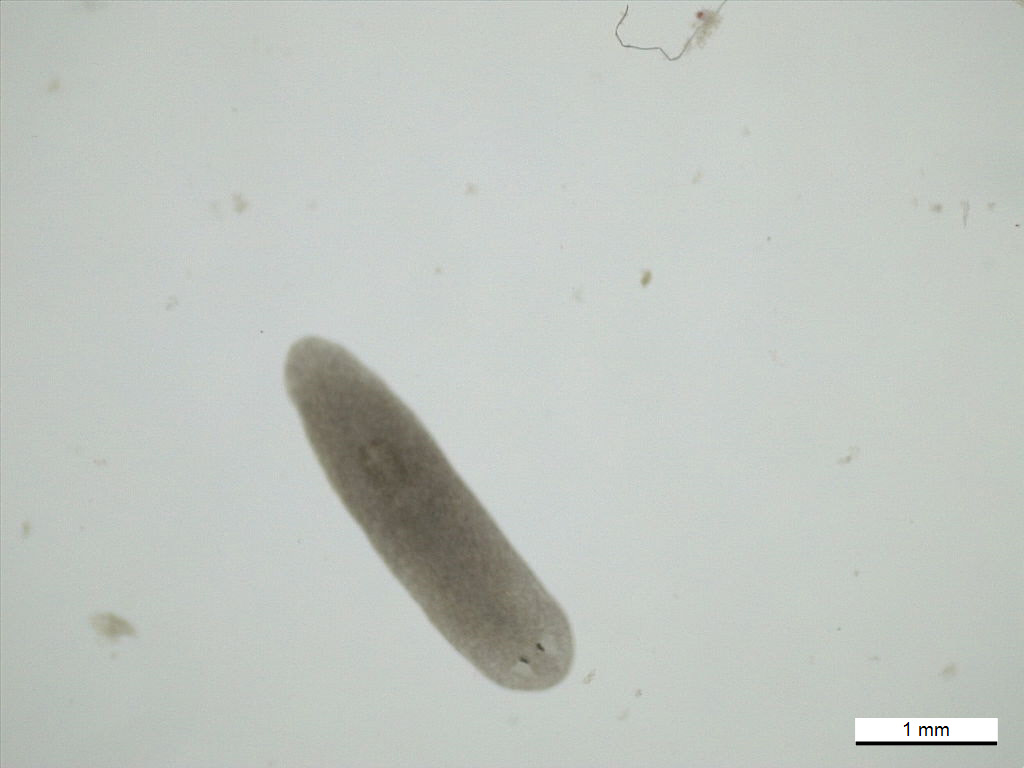

Supplement: Supplementary file 10 — Source data Fig. 3 [file 44318_2025_662_MOESM10_ESM.zip › Figure 3/3C-D/Control_After_10_RNAi_feedings/Control_After_10_RNAi_feedings_16.jpg]

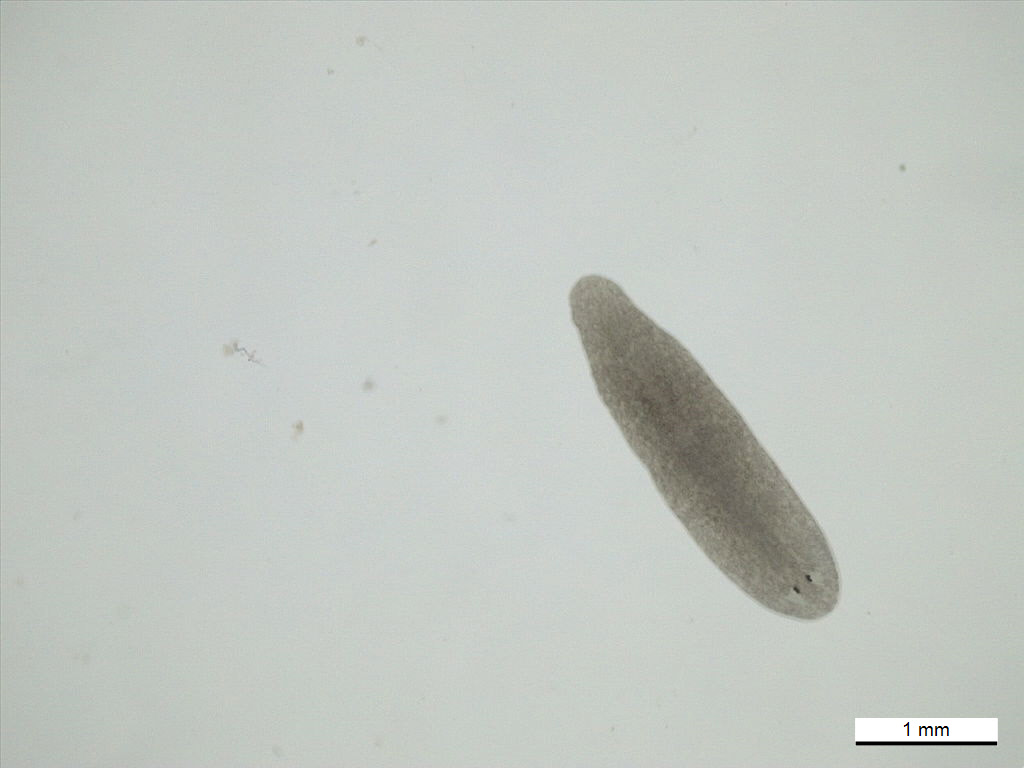

Supplement: Supplementary file 10 — Source data Fig. 3 [file 44318_2025_662_MOESM10_ESM.zip › Figure 3/3C-D/Control_After_10_RNAi_feedings/Control_After_10_RNAi_feedings_17.jpg]

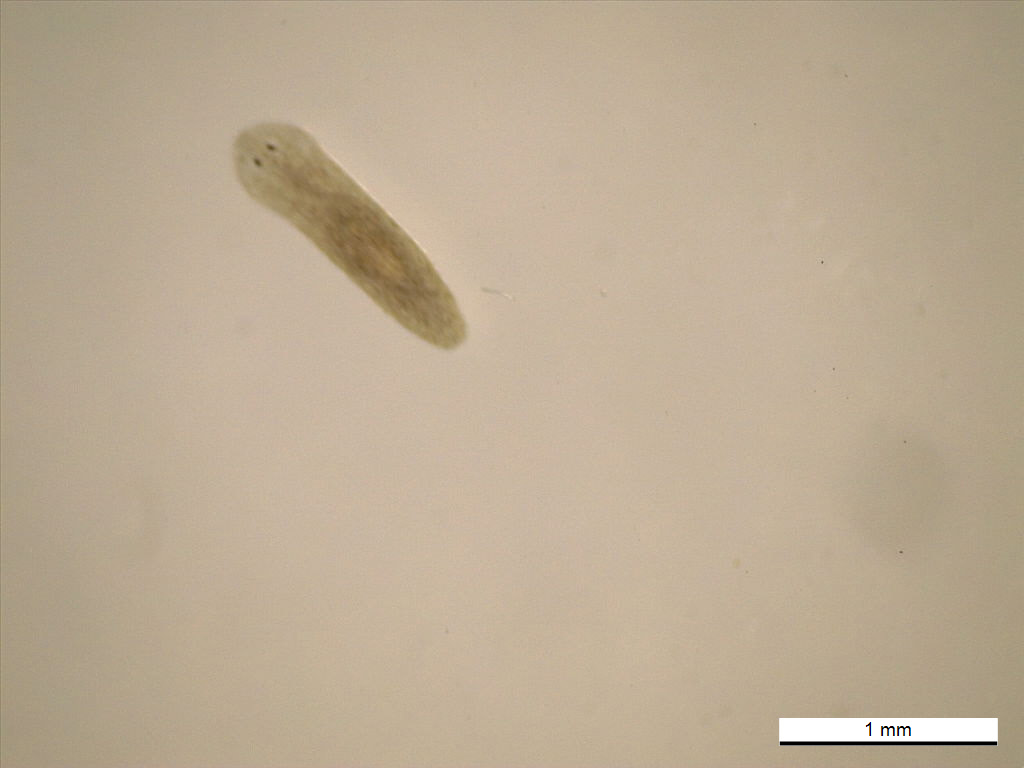

Supplement: Supplementary file 10 — Source data Fig. 3 [file 44318_2025_662_MOESM10_ESM.zip › Figure 3/3C-D/Control_Before_RNAi_feedings/Control_Before_Feedings_1.jpg]

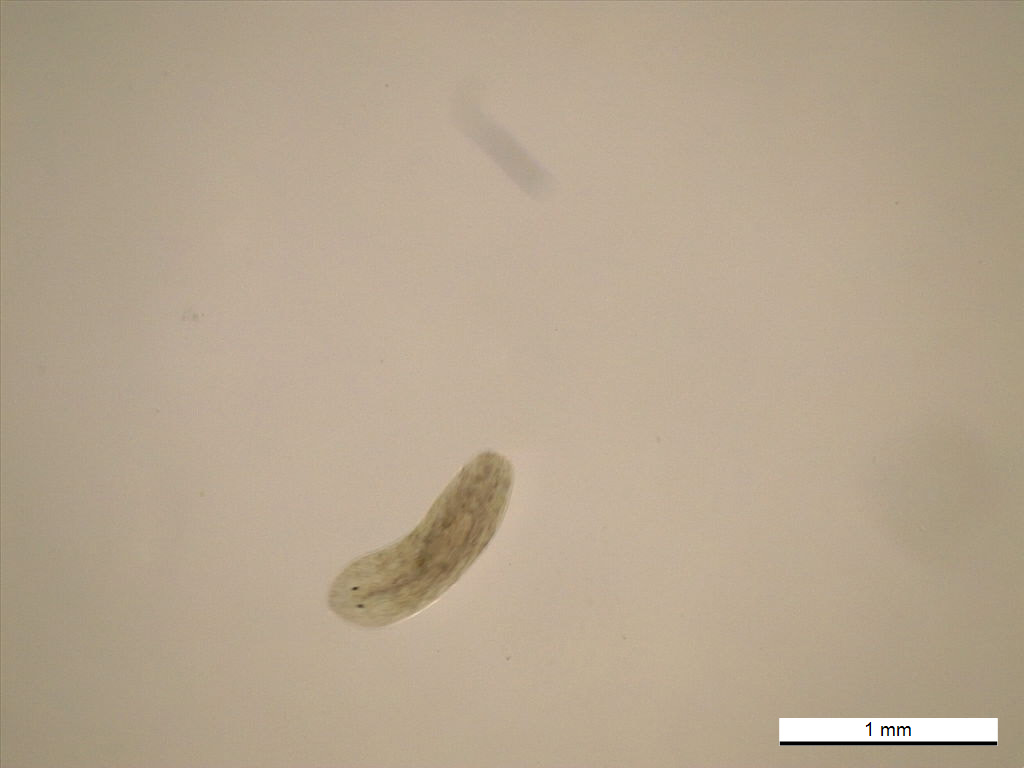

Supplement: Supplementary file 10 — Source data Fig. 3 [file 44318_2025_662_MOESM10_ESM.zip › Figure 3/3C-D/Control_Before_RNAi_feedings/Control_Before_Feedings_10.jpg]

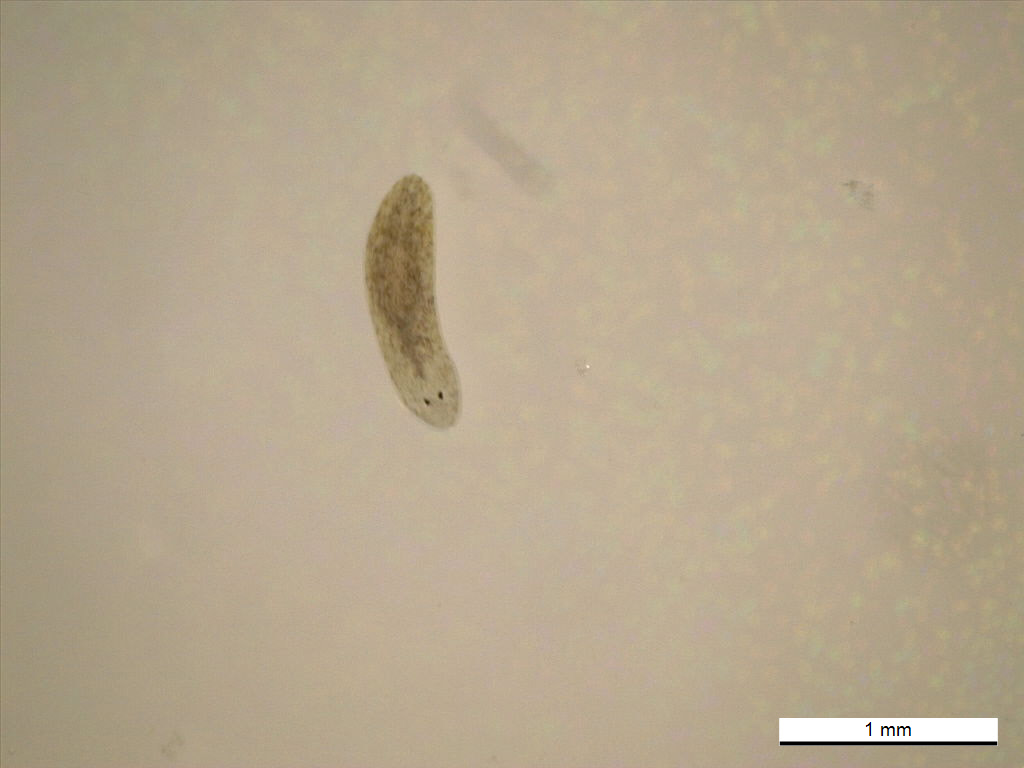

Supplement: Supplementary file 10 — Source data Fig. 3 [file 44318_2025_662_MOESM10_ESM.zip › Figure 3/3C-D/Control_Before_RNAi_feedings/Control_Before_Feedings_11.jpg]

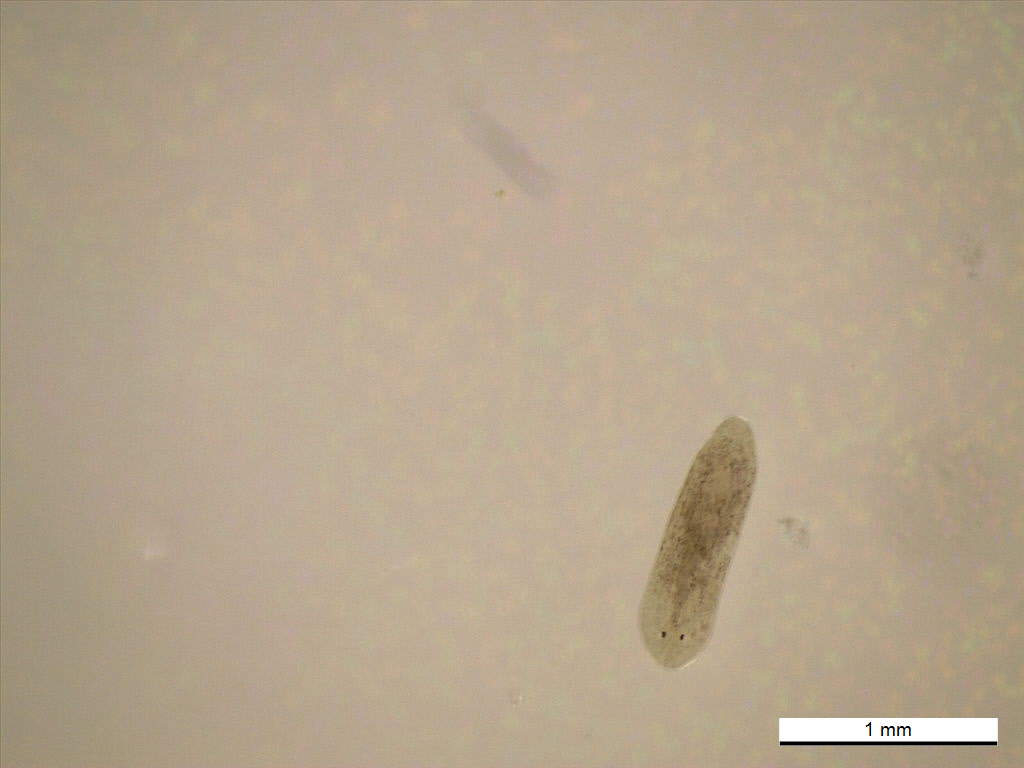

Supplement: Supplementary file 10 — Source data Fig. 3 [file 44318_2025_662_MOESM10_ESM.zip › Figure 3/3C-D/Control_Before_RNAi_feedings/Control_Before_Feedings_12.jpg]

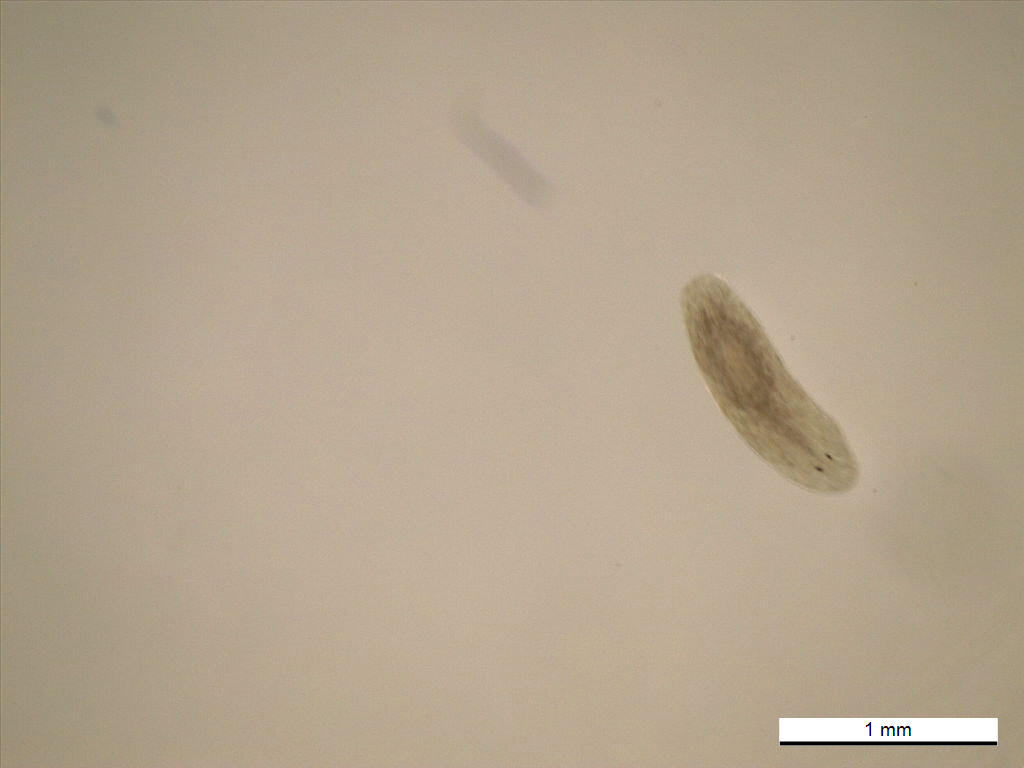

Supplement: Supplementary file 10 — Source data Fig. 3 [file 44318_2025_662_MOESM10_ESM.zip › Figure 3/3C-D/Control_Before_RNAi_feedings/Control_Before_Feedings_13.jpg]

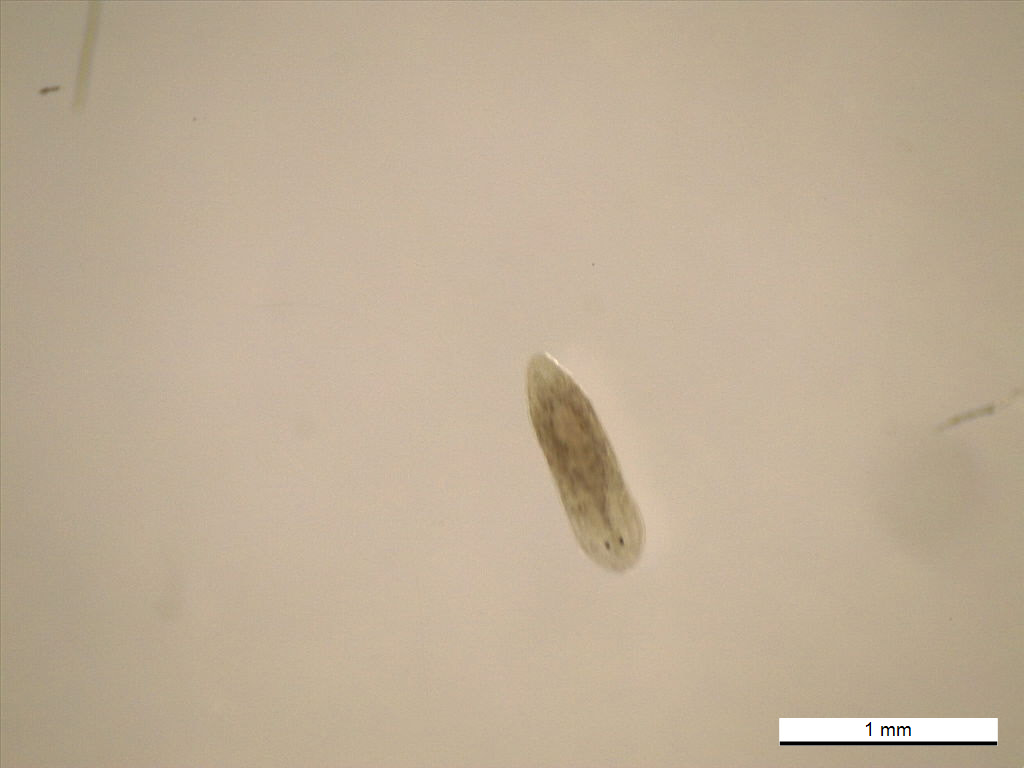

Supplement: Supplementary file 10 — Source data Fig. 3 [file 44318_2025_662_MOESM10_ESM.zip › Figure 3/3C-D/Control_Before_RNAi_feedings/Control_Before_Feedings_14.jpg]

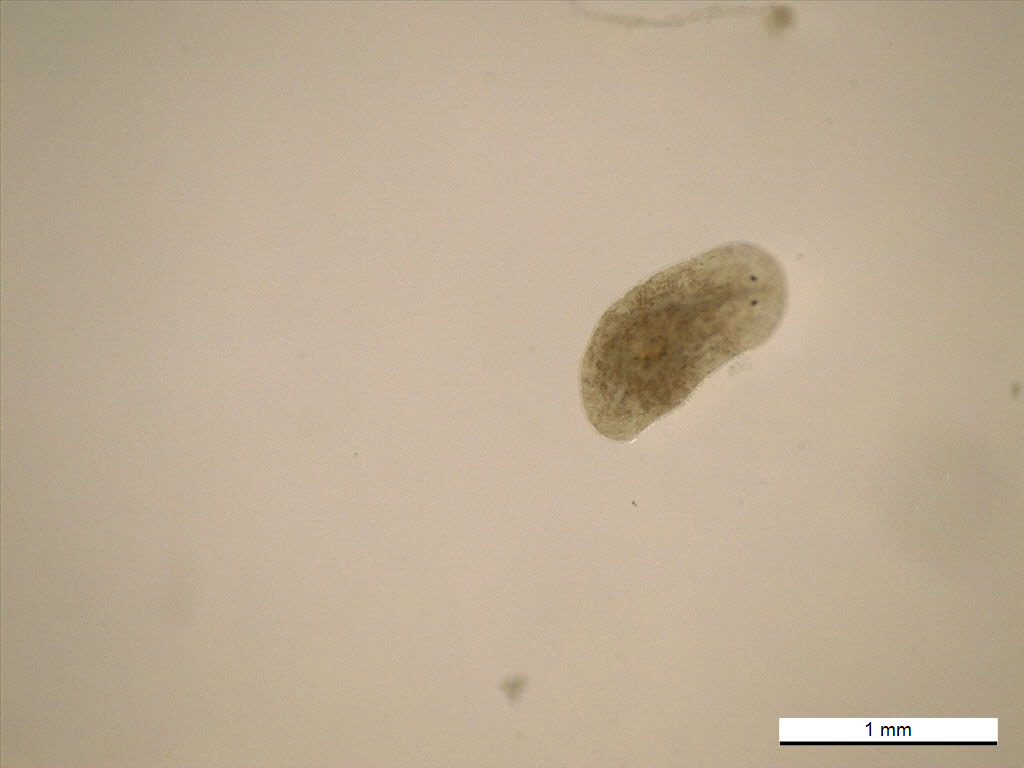

Supplement: Supplementary file 10 — Source data Fig. 3 [file 44318_2025_662_MOESM10_ESM.zip › Figure 3/3C-D/Control_Before_RNAi_feedings/Control_Before_Feedings_15.jpg]

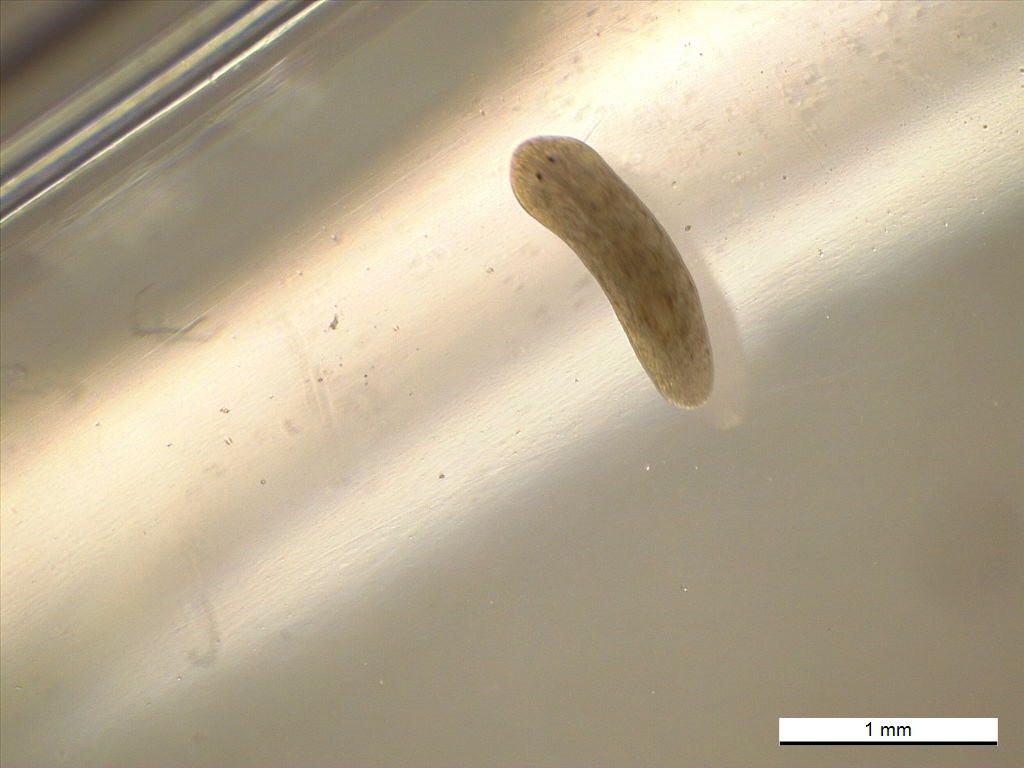

Supplement: Supplementary file 10 — Source data Fig. 3 [file 44318_2025_662_MOESM10_ESM.zip › Figure 3/3C-D/Control_Before_RNAi_feedings/Control_Before_Feedings_16.jpg]

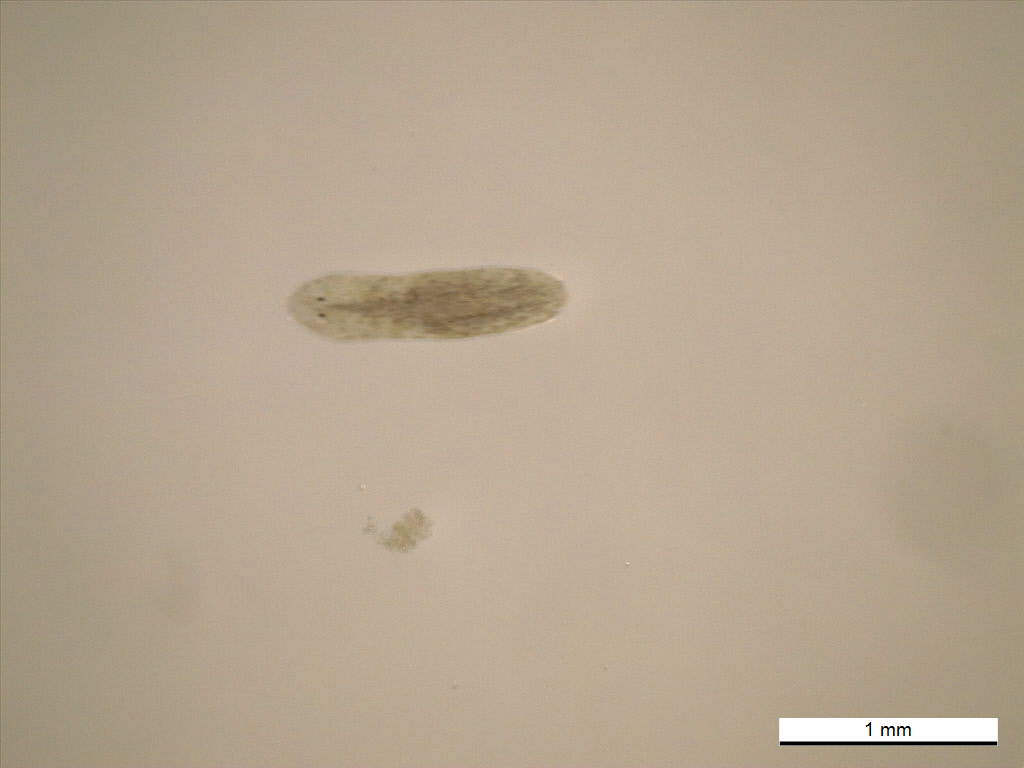

Supplement: Supplementary file 10 — Source data Fig. 3 [file 44318_2025_662_MOESM10_ESM.zip › Figure 3/3C-D/Control_Before_RNAi_feedings/Control_Before_Feedings_17.jpg]

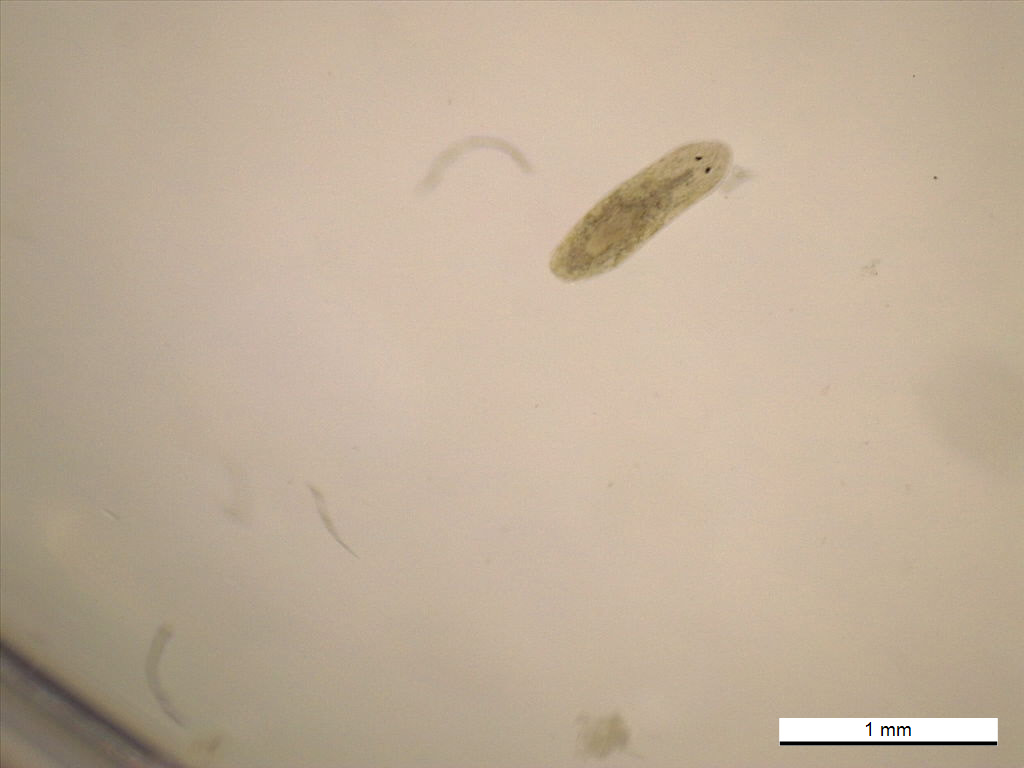

Supplement: Supplementary file 10 — Source data Fig. 3 [file 44318_2025_662_MOESM10_ESM.zip › Figure 3/3C-D/Control_Before_RNAi_feedings/Control_Before_Feedings_18.jpg]

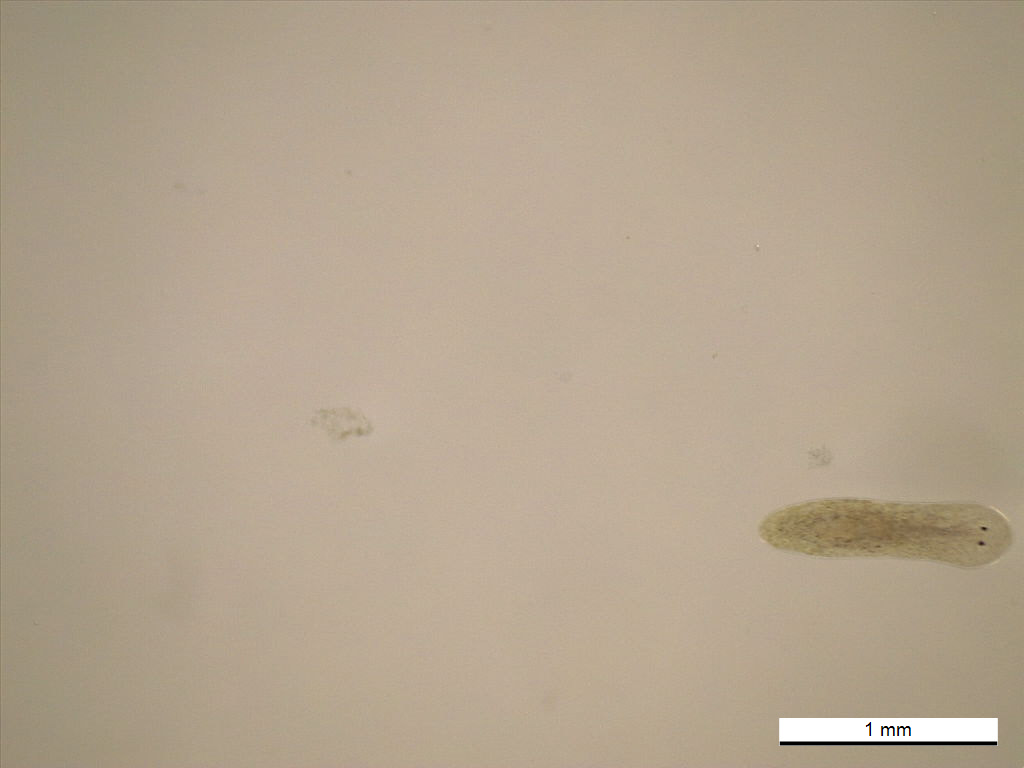

Supplement: Supplementary file 10 — Source data Fig. 3 [file 44318_2025_662_MOESM10_ESM.zip › Figure 3/3C-D/Control_Before_RNAi_feedings/Control_Before_Feedings_19.jpg]

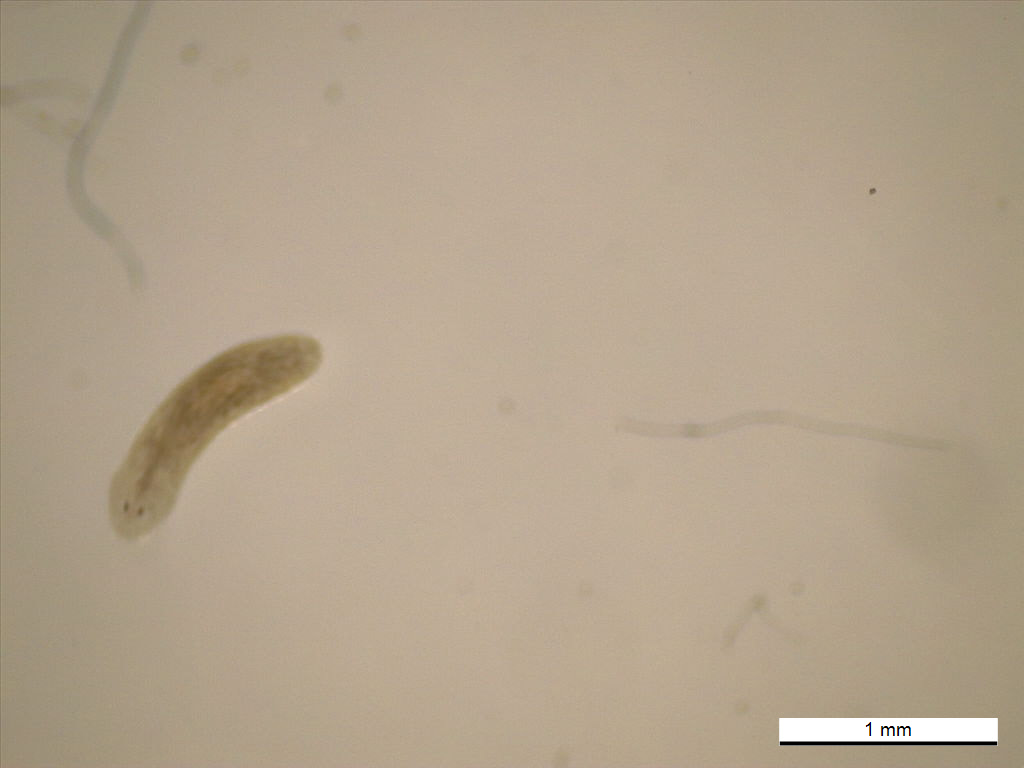

Supplement: Supplementary file 10 — Source data Fig. 3 [file 44318_2025_662_MOESM10_ESM.zip › Figure 3/3C-D/Control_Before_RNAi_feedings/Control_Before_Feedings_2.jpg]

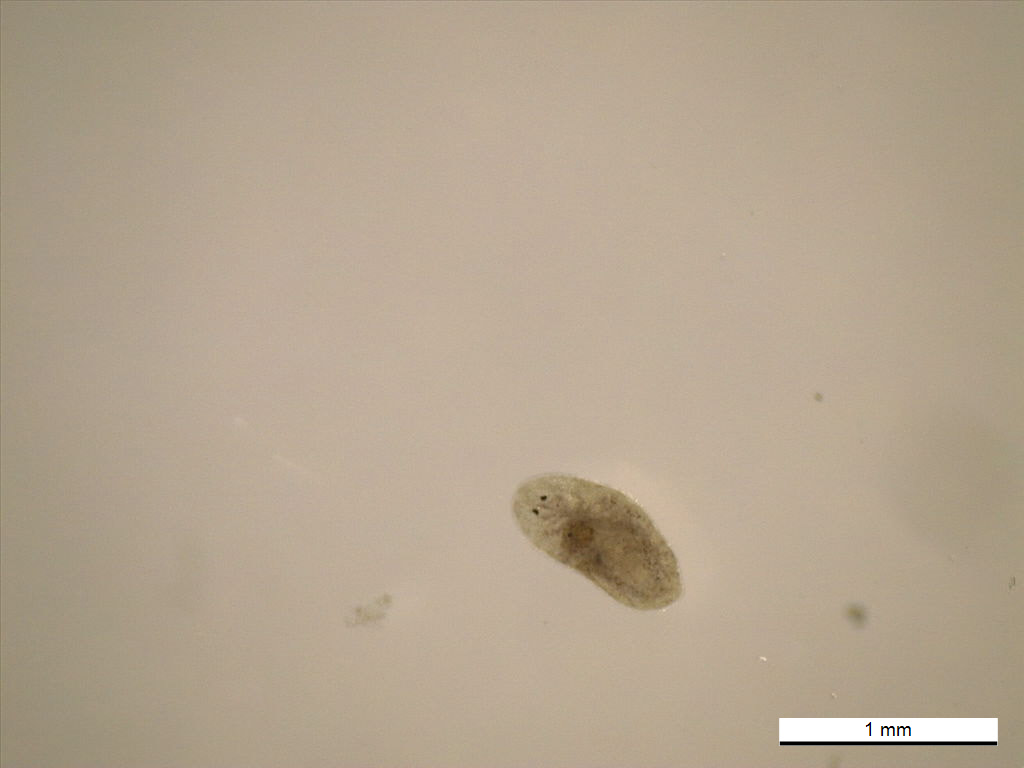

Supplement: Supplementary file 10 — Source data Fig. 3 [file 44318_2025_662_MOESM10_ESM.zip › Figure 3/3C-D/Control_Before_RNAi_feedings/Control_Before_Feedings_20.jpg]

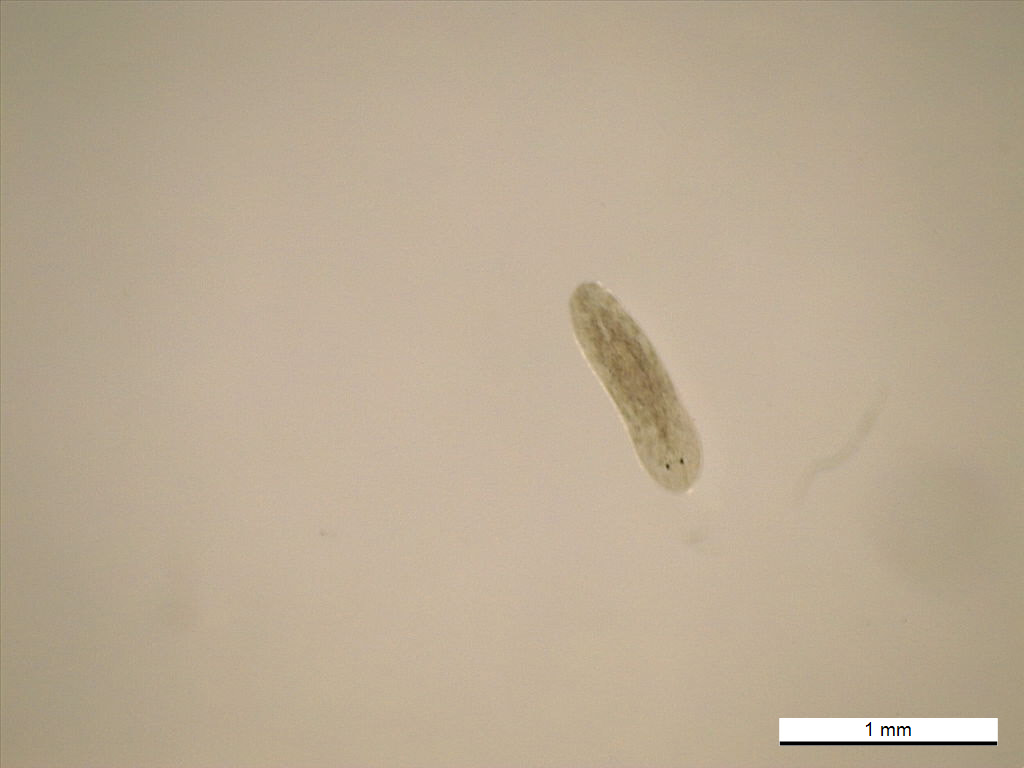

Supplement: Supplementary file 10 — Source data Fig. 3 [file 44318_2025_662_MOESM10_ESM.zip › Figure 3/3C-D/Control_Before_RNAi_feedings/Control_Before_Feedings_21.jpg]

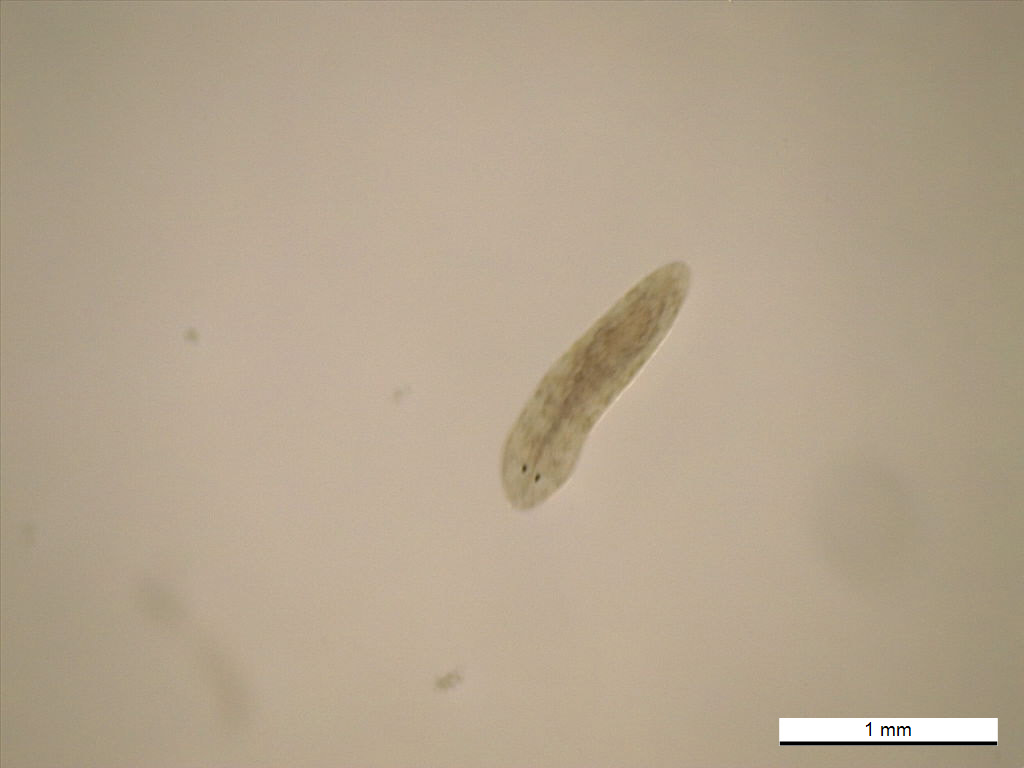

Supplement: Supplementary file 10 — Source data Fig. 3 [file 44318_2025_662_MOESM10_ESM.zip › Figure 3/3C-D/Control_Before_RNAi_feedings/Control_Before_Feedings_22.jpg]

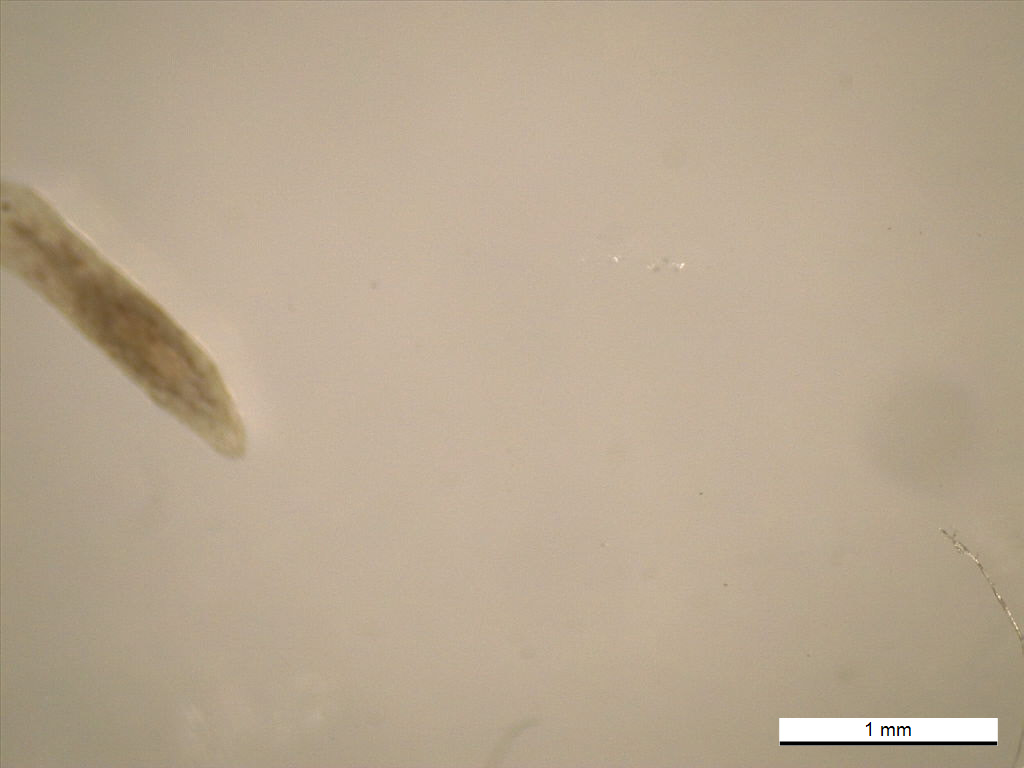

Supplement: Supplementary file 10 — Source data Fig. 3 [file 44318_2025_662_MOESM10_ESM.zip › Figure 3/3C-D/Control_Before_RNAi_feedings/Control_Before_Feedings_3.jpg]

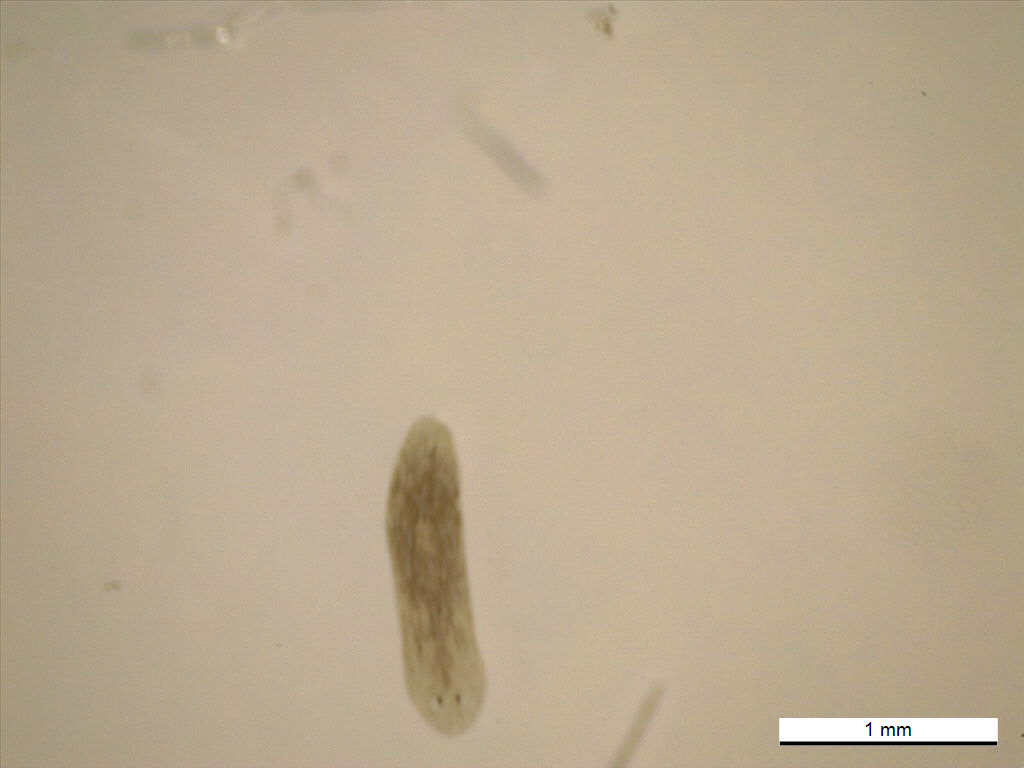

Supplement: Supplementary file 10 — Source data Fig. 3 [file 44318_2025_662_MOESM10_ESM.zip › Figure 3/3C-D/Control_Before_RNAi_feedings/Control_Before_Feedings_4.jpg]

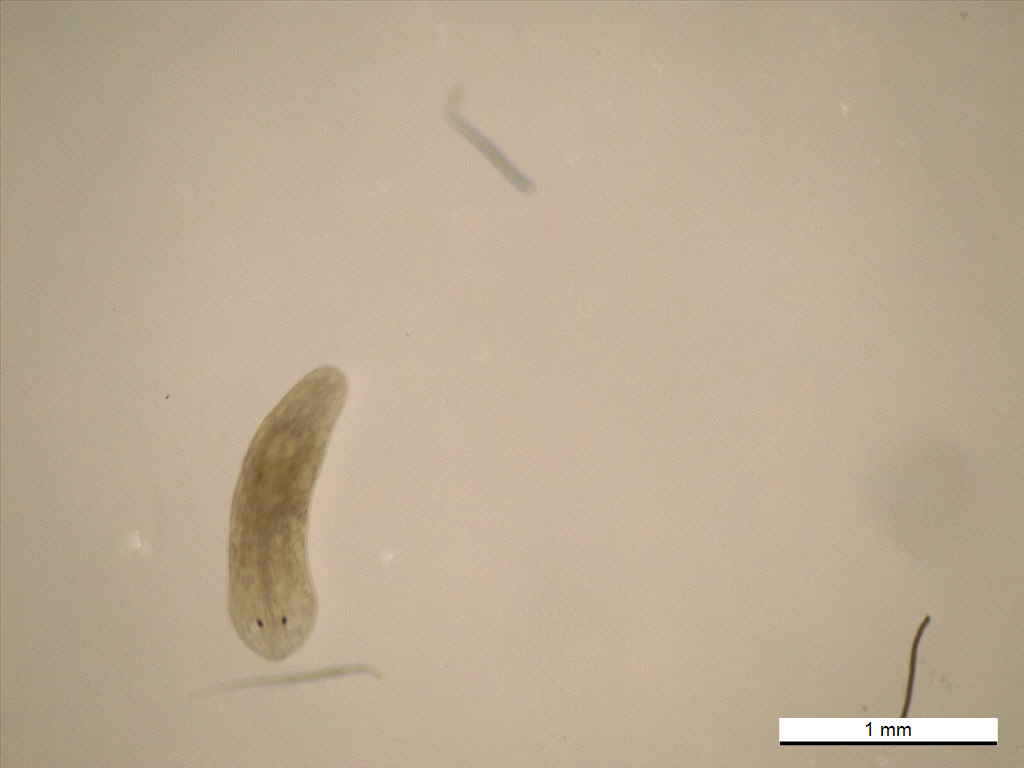

Supplement: Supplementary file 10 — Source data Fig. 3 [file 44318_2025_662_MOESM10_ESM.zip › Figure 3/3C-D/Control_Before_RNAi_feedings/Control_Before_Feedings_5.jpg]

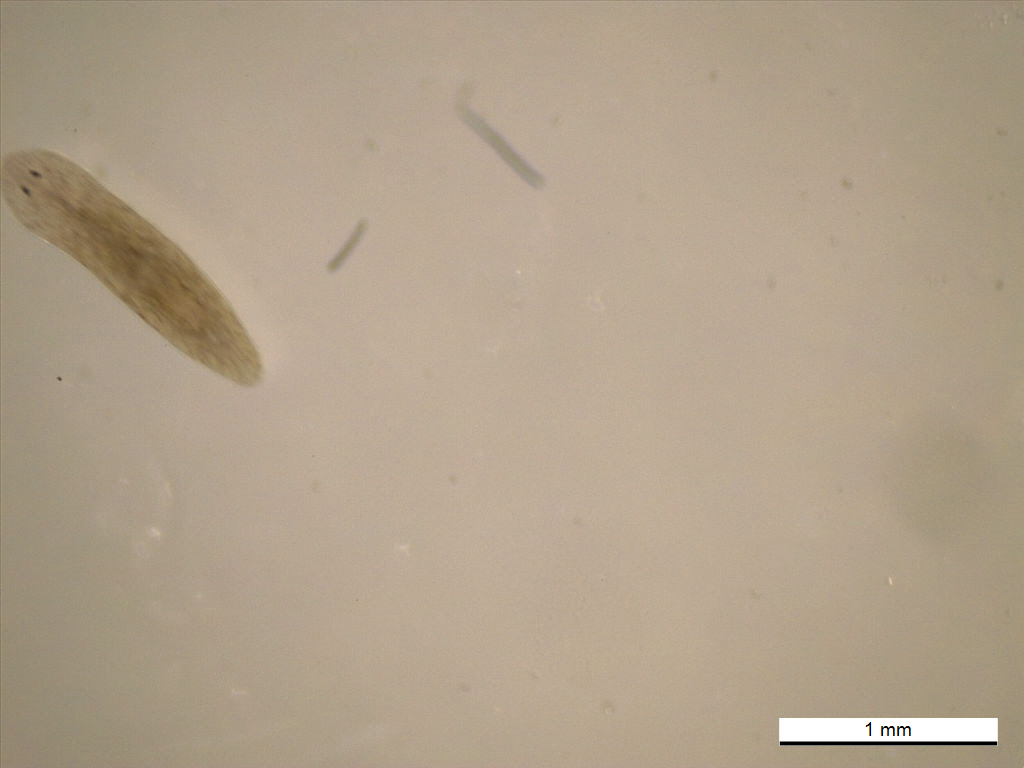

Supplement: Supplementary file 10 — Source data Fig. 3 [file 44318_2025_662_MOESM10_ESM.zip › Figure 3/3C-D/Control_Before_RNAi_feedings/Control_Before_Feedings_6.jpg]

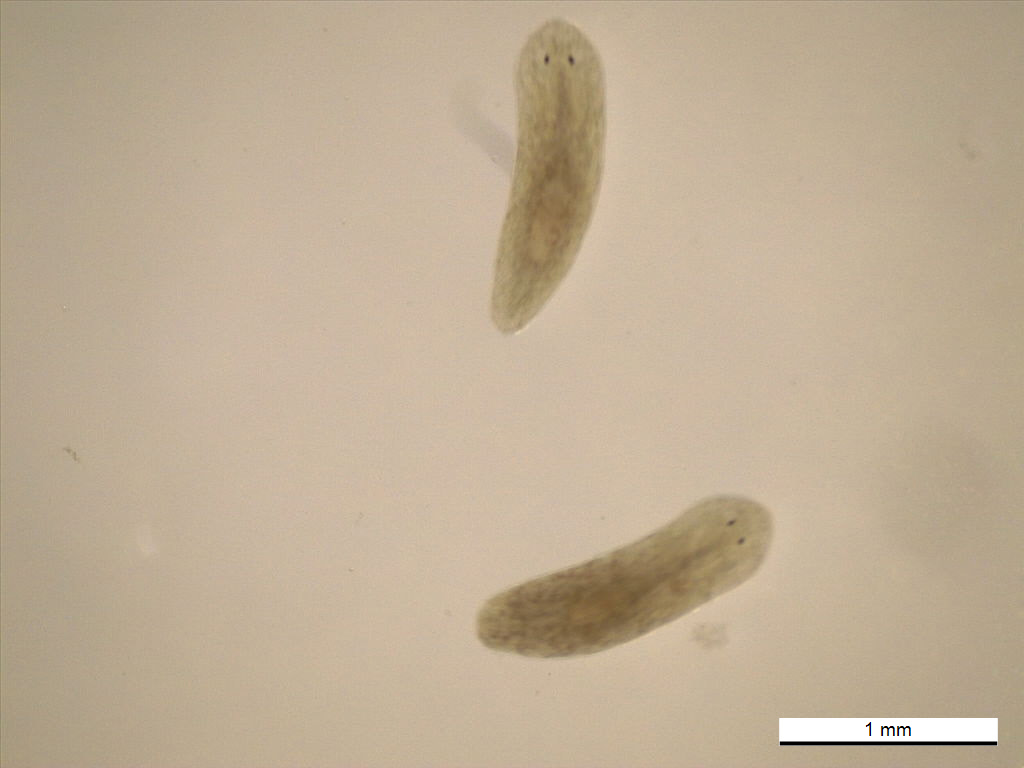

Supplement: Supplementary file 10 — Source data Fig. 3 [file 44318_2025_662_MOESM10_ESM.zip › Figure 3/3C-D/Control_Before_RNAi_feedings/Control_Before_Feedings_7.jpg]

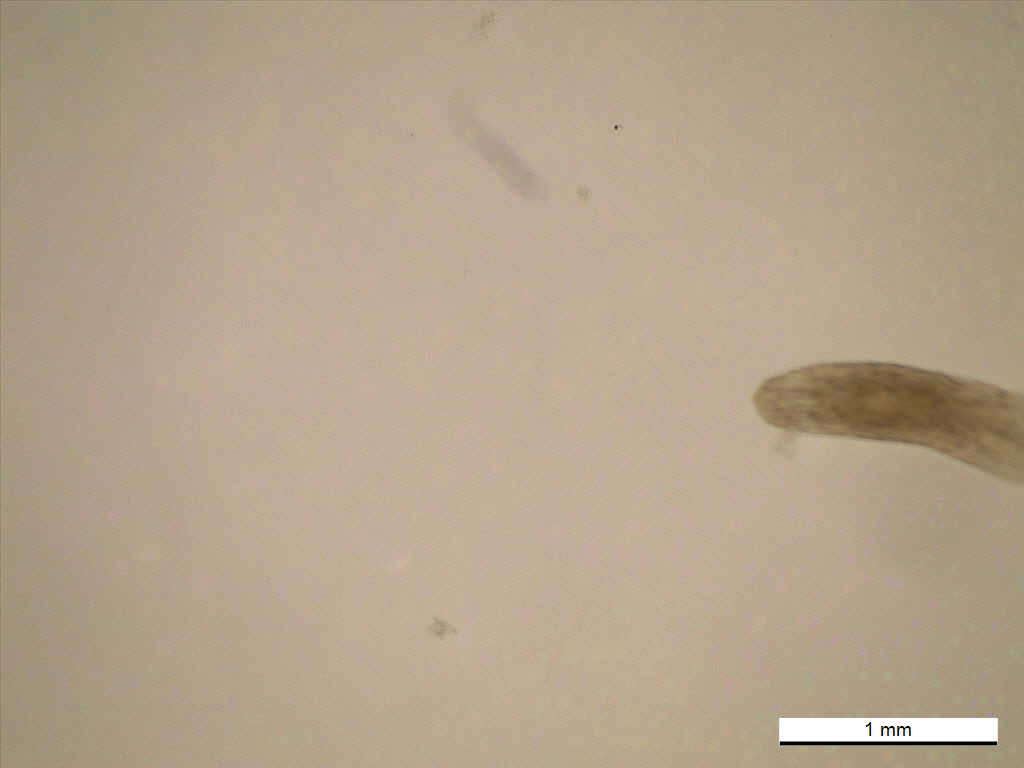

Supplement: Supplementary file 10 — Source data Fig. 3 [file 44318_2025_662_MOESM10_ESM.zip › Figure 3/3C-D/Control_Before_RNAi_feedings/Control_Before_Feedings_8.jpg]

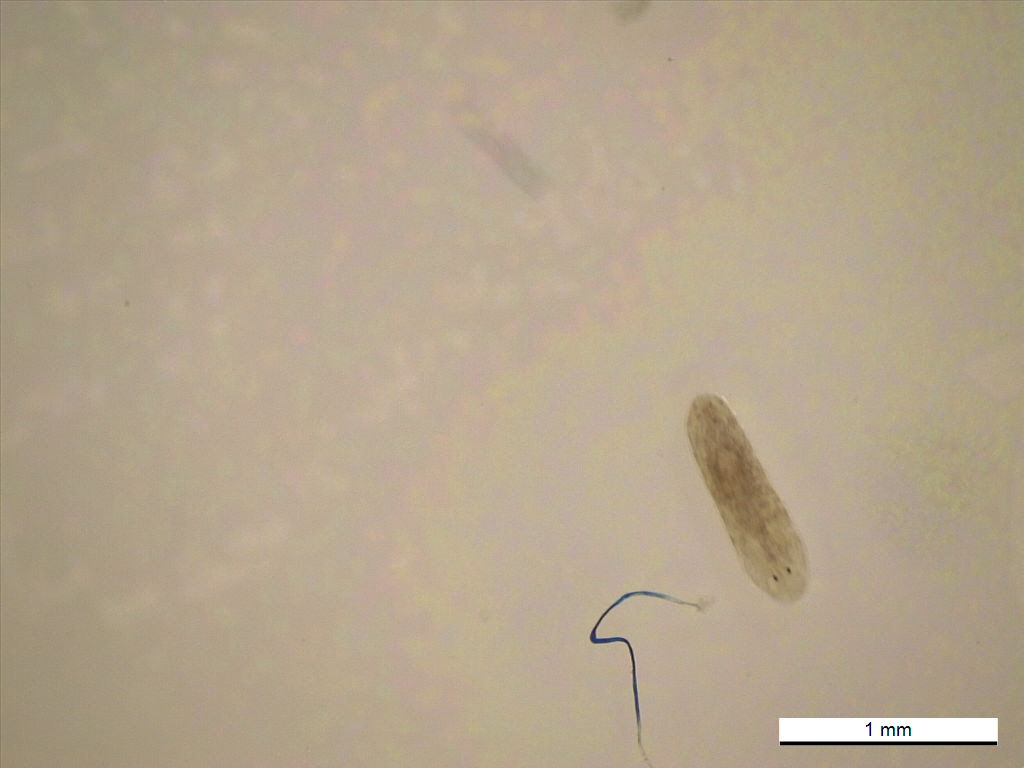

Supplement: Supplementary file 10 — Source data Fig. 3 [file 44318_2025_662_MOESM10_ESM.zip › Figure 3/3C-D/Control_Before_RNAi_feedings/Control_Before_Feedings_9.jpg]

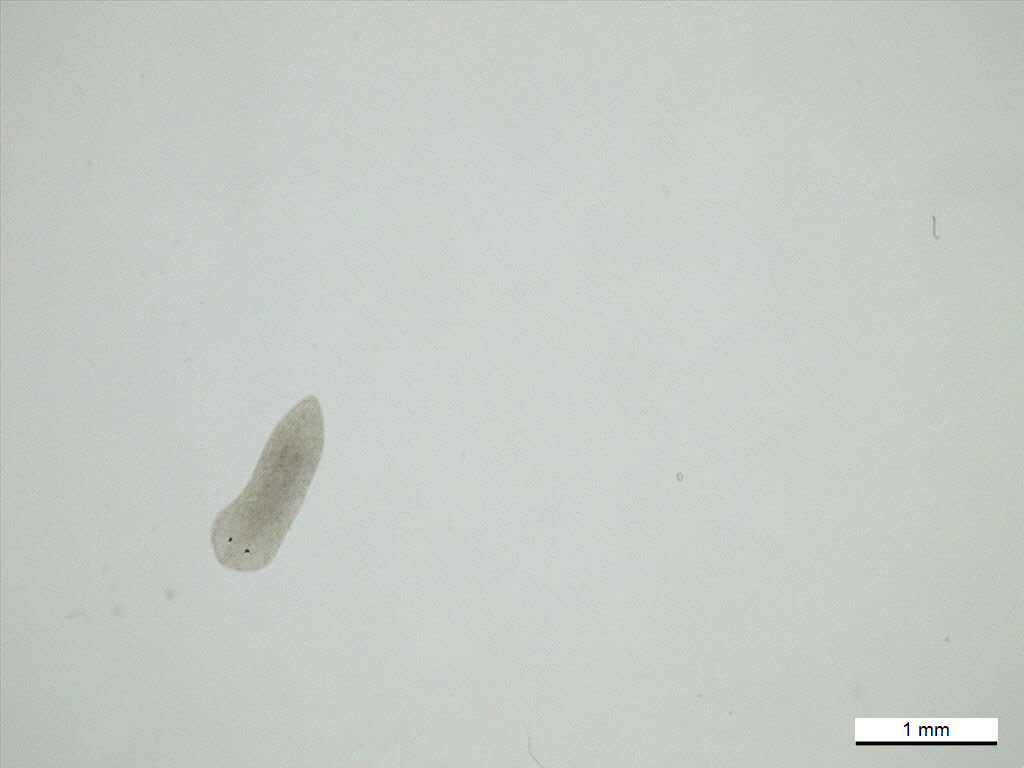

Supplement: Supplementary file 10 — Source data Fig. 3 [file 44318_2025_662_MOESM10_ESM.zip › Figure 3/3C-D/Triple_ythdf_RNAi_After_10_RNAi_feedings/Triple_ythdf_RNAi_After_10_RNAi_feedings_01.jpg]

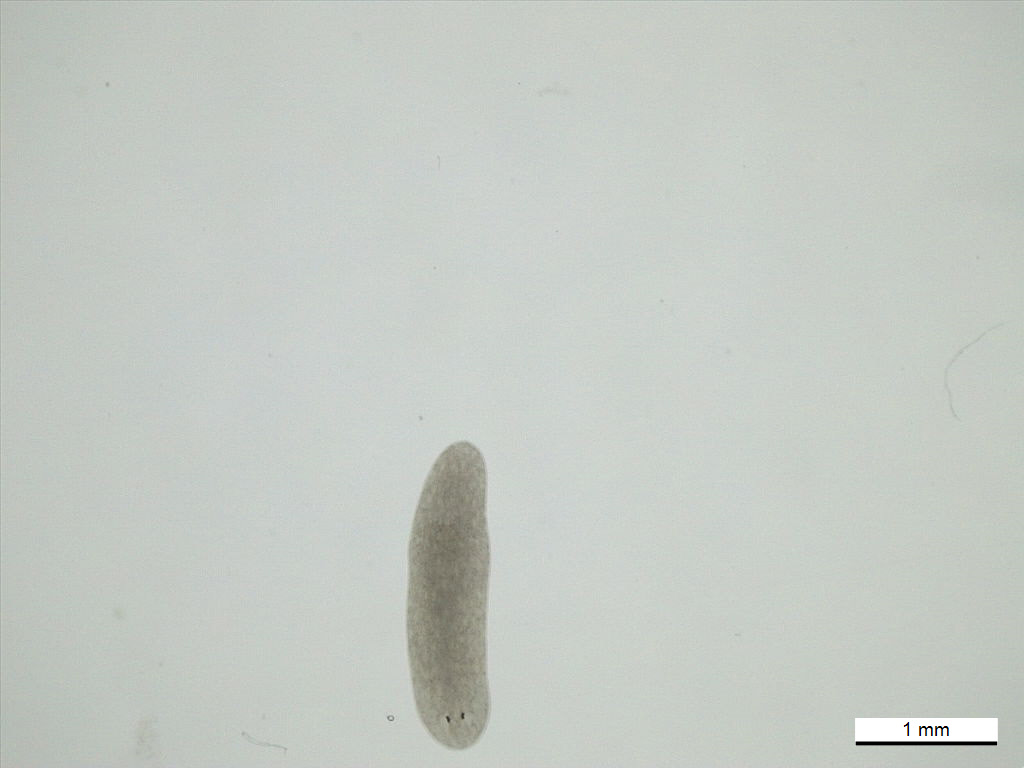

Supplement: Supplementary file 10 — Source data Fig. 3 [file 44318_2025_662_MOESM10_ESM.zip › Figure 3/3C-D/Triple_ythdf_RNAi_After_10_RNAi_feedings/Triple_ythdf_RNAi_After_10_RNAi_feedings_02.jpg]

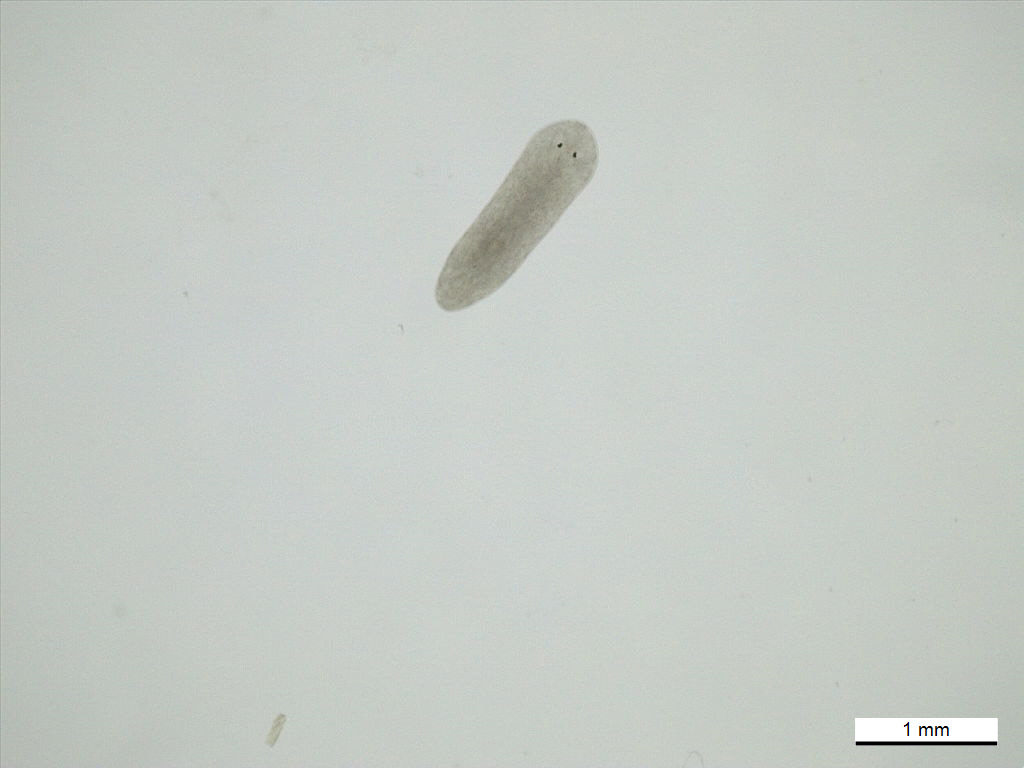

Supplement: Supplementary file 10 — Source data Fig. 3 [file 44318_2025_662_MOESM10_ESM.zip › Figure 3/3C-D/Triple_ythdf_RNAi_After_10_RNAi_feedings/Triple_ythdf_RNAi_After_10_RNAi_feedings_03.jpg]

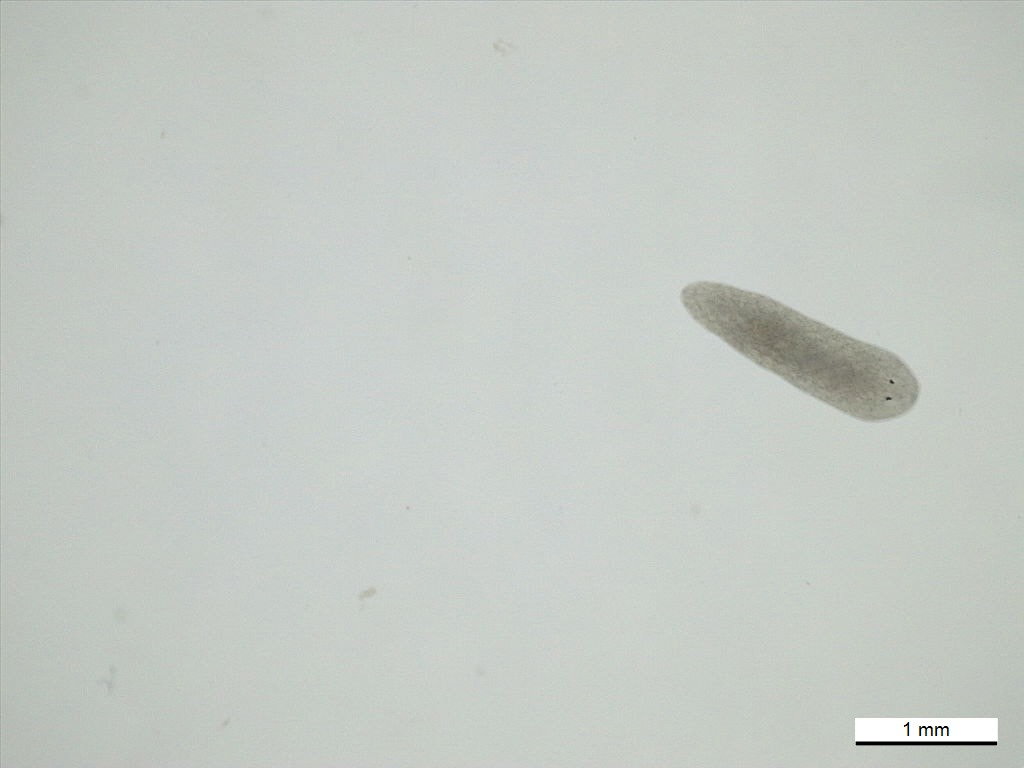

Supplement: Supplementary file 10 — Source data Fig. 3 [file 44318_2025_662_MOESM10_ESM.zip › Figure 3/3C-D/Triple_ythdf_RNAi_After_10_RNAi_feedings/Triple_ythdf_RNAi_After_10_RNAi_feedings_04.jpg]

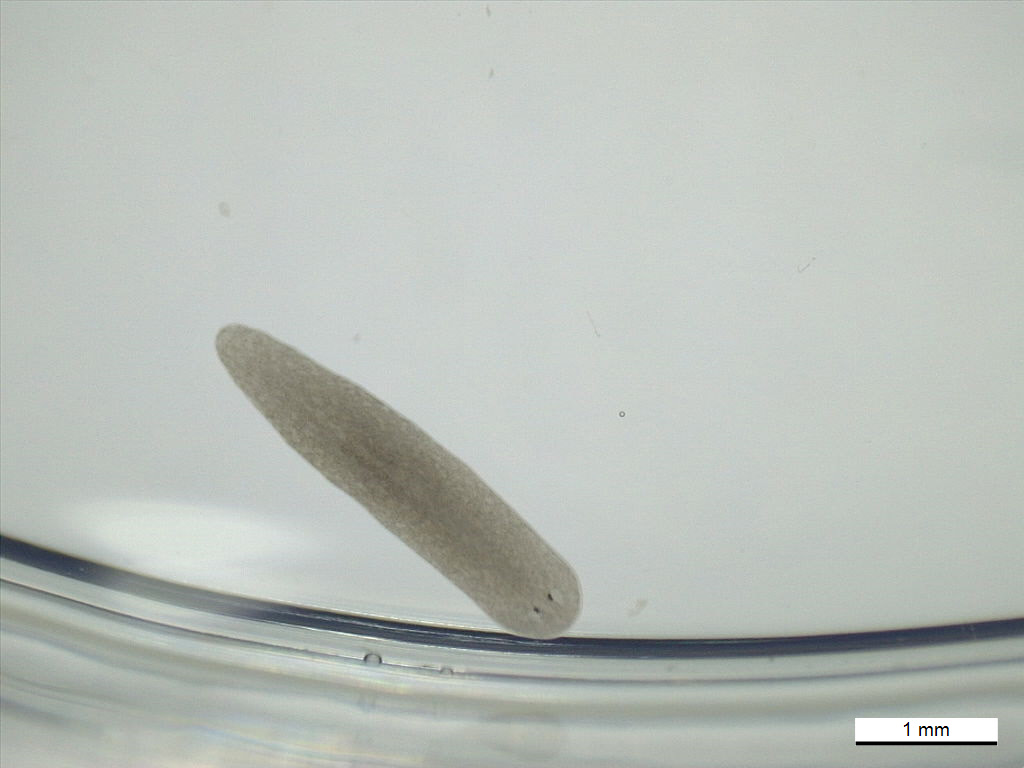

Supplement: Supplementary file 10 — Source data Fig. 3 [file 44318_2025_662_MOESM10_ESM.zip › Figure 3/3C-D/Triple_ythdf_RNAi_After_10_RNAi_feedings/Triple_ythdf_RNAi_After_10_RNAi_feedings_05.jpg]

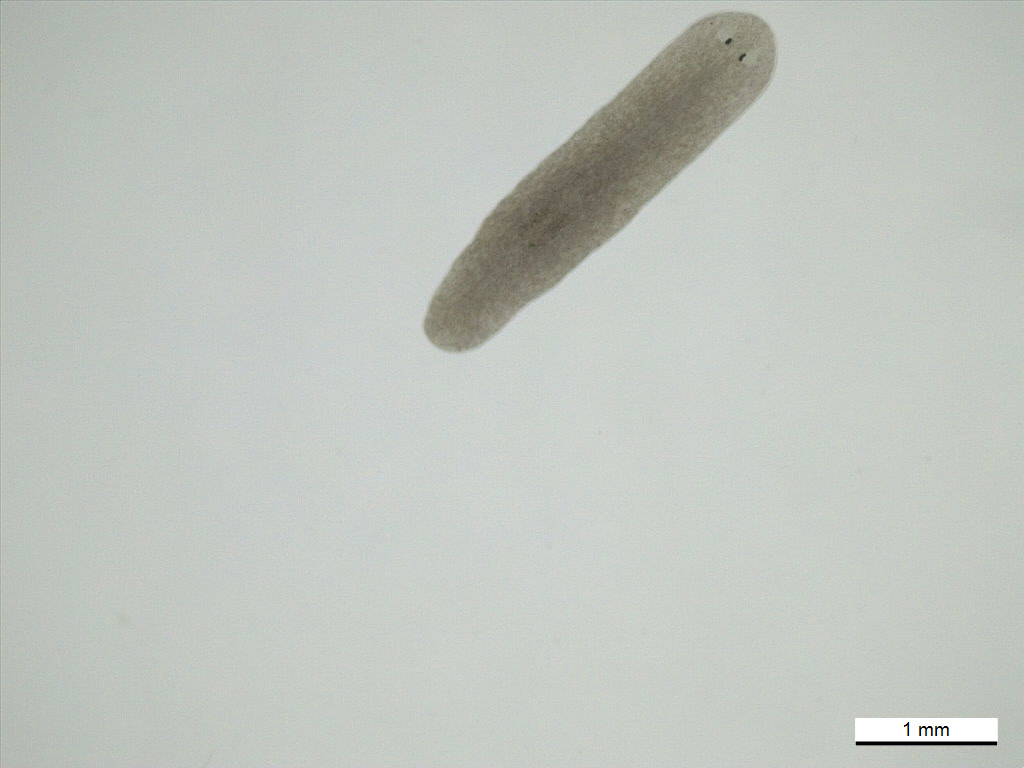

Supplement: Supplementary file 10 — Source data Fig. 3 [file 44318_2025_662_MOESM10_ESM.zip › Figure 3/3C-D/Triple_ythdf_RNAi_After_10_RNAi_feedings/Triple_ythdf_RNAi_After_10_RNAi_feedings_06.jpg]

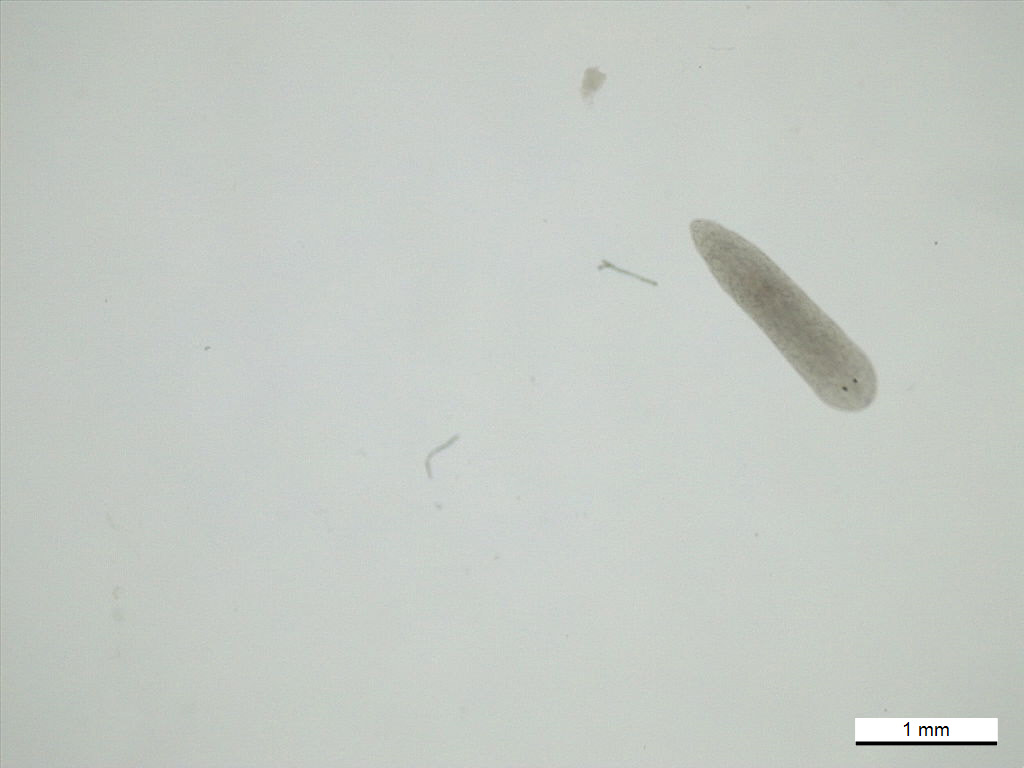

Supplement: Supplementary file 10 — Source data Fig. 3 [file 44318_2025_662_MOESM10_ESM.zip › Figure 3/3C-D/Triple_ythdf_RNAi_After_10_RNAi_feedings/Triple_ythdf_RNAi_After_10_RNAi_feedings_07.jpg]

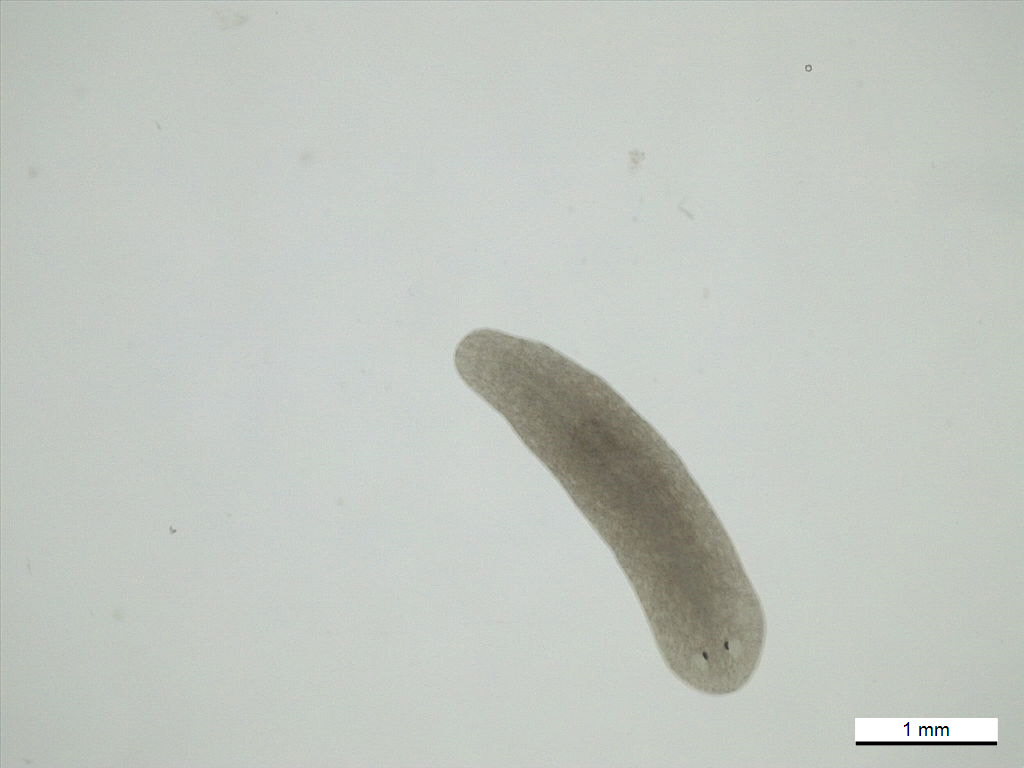

Supplement: Supplementary file 10 — Source data Fig. 3 [file 44318_2025_662_MOESM10_ESM.zip › Figure 3/3C-D/Triple_ythdf_RNAi_After_10_RNAi_feedings/Triple_ythdf_RNAi_After_10_RNAi_feedings_08.jpg]

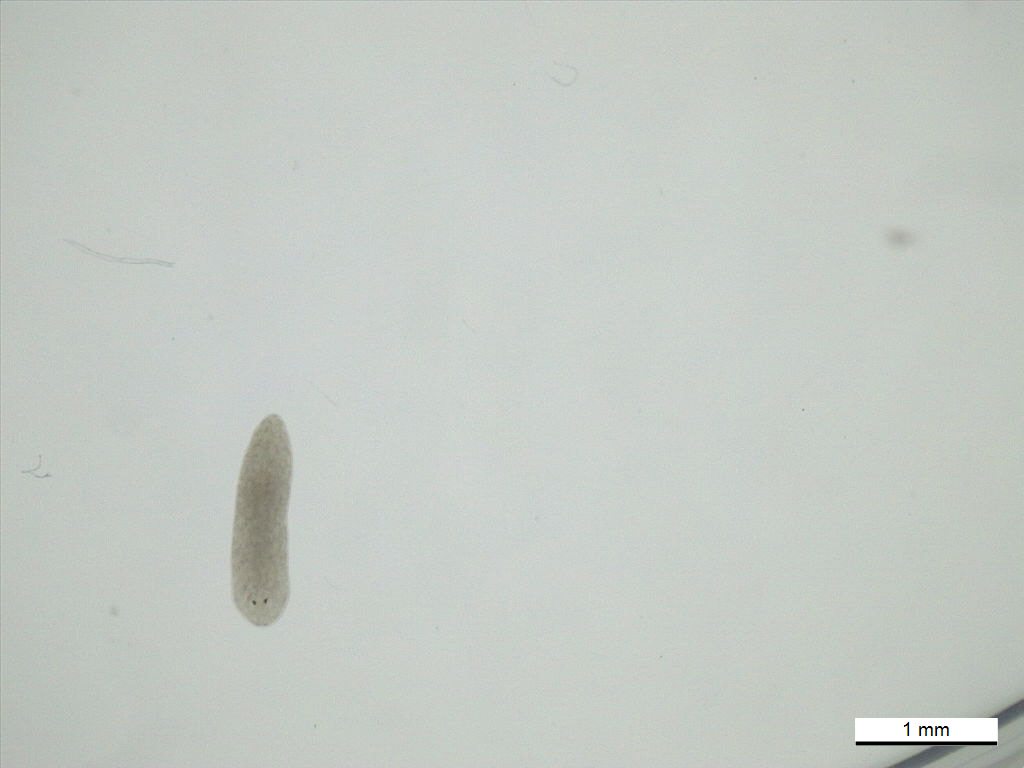

Supplement: Supplementary file 10 — Source data Fig. 3 [file 44318_2025_662_MOESM10_ESM.zip › Figure 3/3C-D/Triple_ythdf_RNAi_After_10_RNAi_feedings/Triple_ythdf_RNAi_After_10_RNAi_feedings_09.jpg]

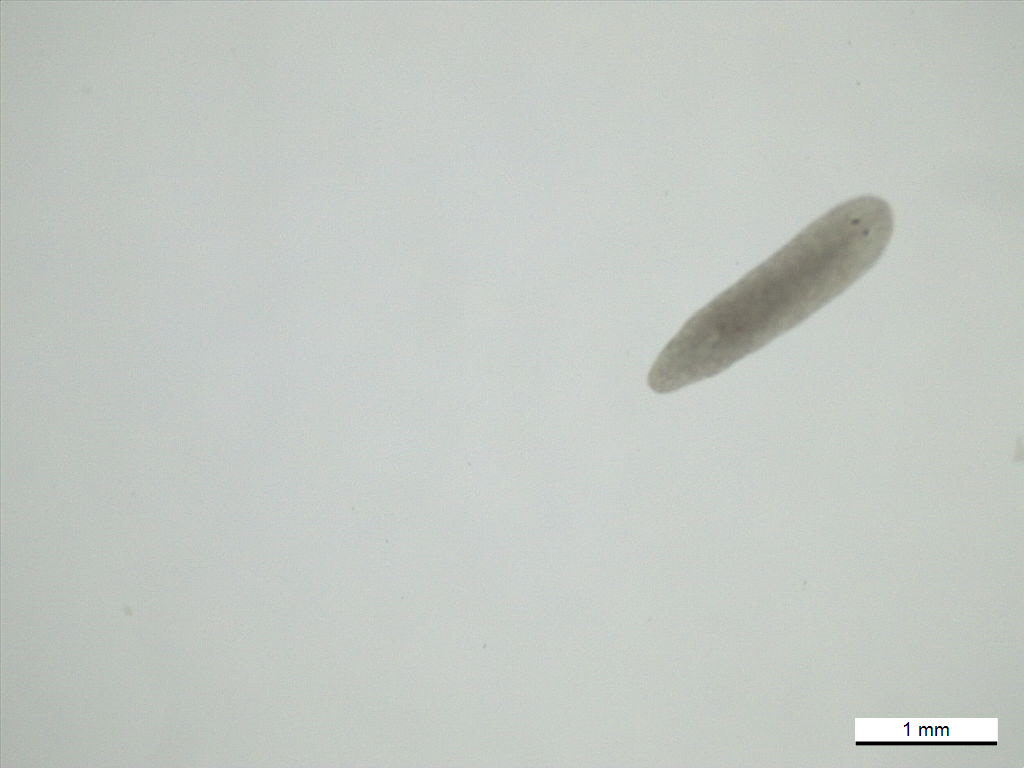

Supplement: Supplementary file 10 — Source data Fig. 3 [file 44318_2025_662_MOESM10_ESM.zip › Figure 3/3C-D/Triple_ythdf_RNAi_After_10_RNAi_feedings/Triple_ythdf_RNAi_After_10_RNAi_feedings_10.jpg]

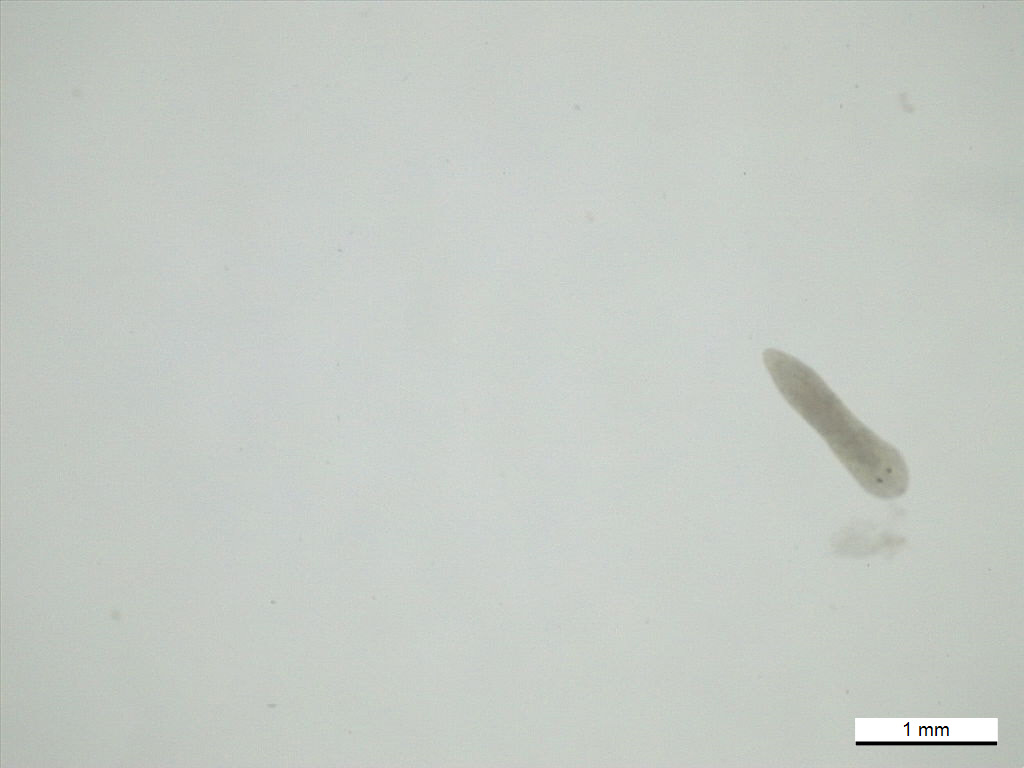

Supplement: Supplementary file 10 — Source data Fig. 3 [file 44318_2025_662_MOESM10_ESM.zip › Figure 3/3C-D/Triple_ythdf_RNAi_After_10_RNAi_feedings/Triple_ythdf_RNAi_After_10_RNAi_feedings_11.jpg]

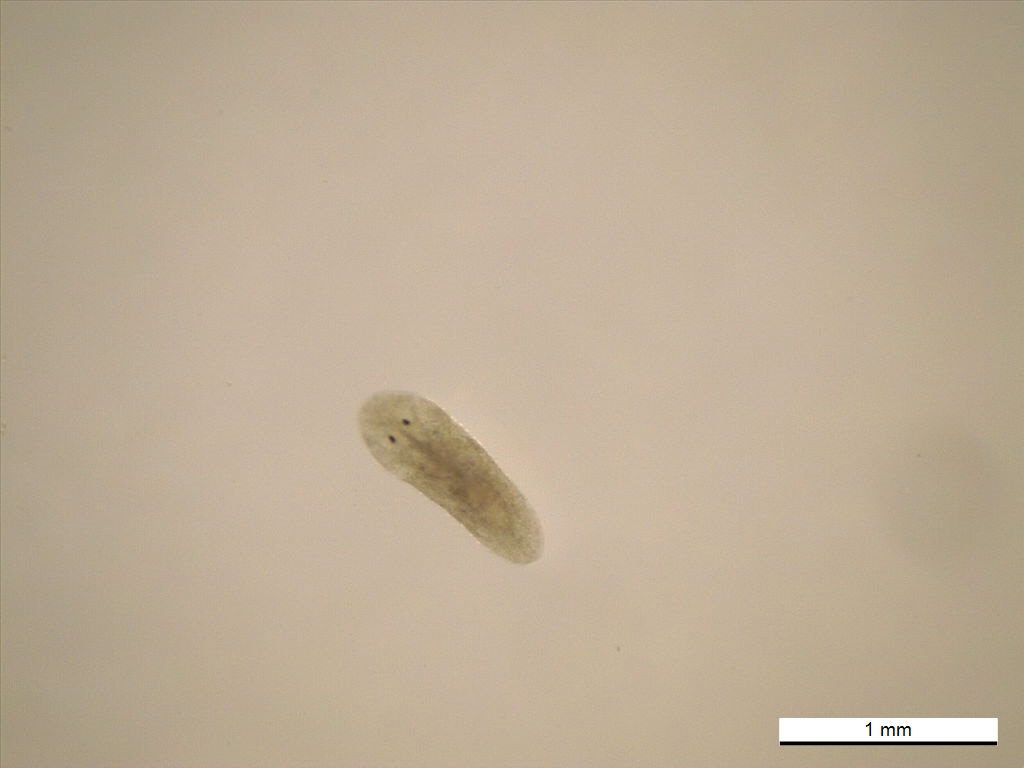

Supplement: Supplementary file 10 — Source data Fig. 3 [file 44318_2025_662_MOESM10_ESM.zip › Figure 3/3C-D/Triple_ythdf_RNAi_Before_RNAi_feedings/Triple_ythdf_RNAi_Before_RNAi_feedings_1.jpg]

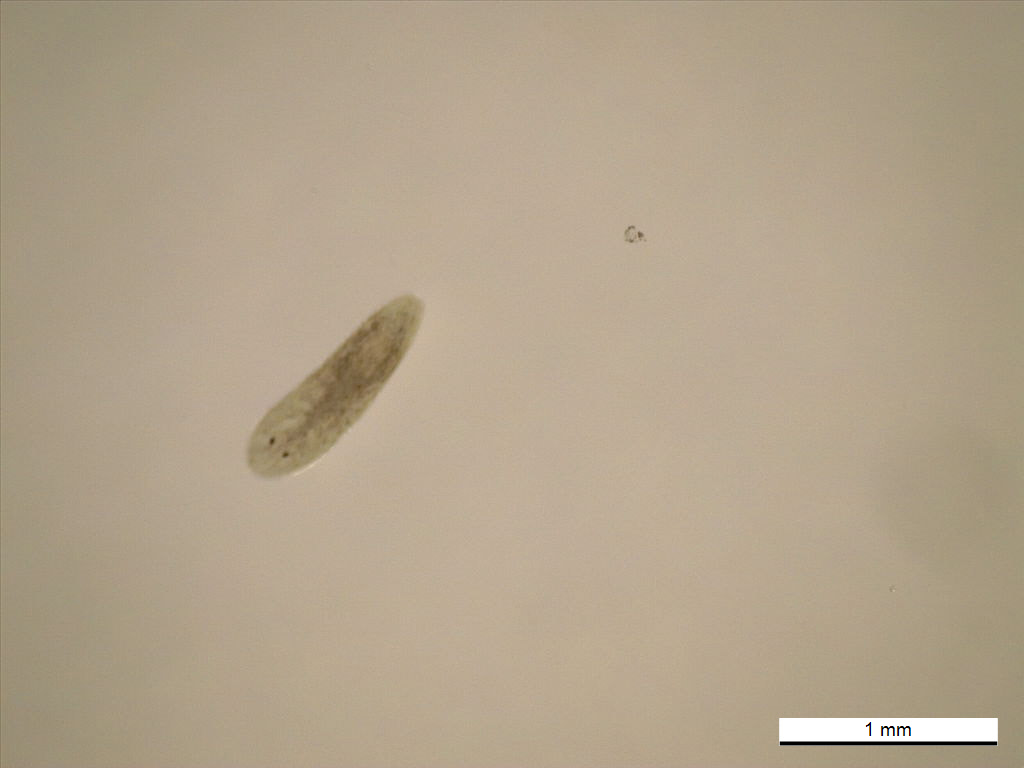

Supplement: Supplementary file 10 — Source data Fig. 3 [file 44318_2025_662_MOESM10_ESM.zip › Figure 3/3C-D/Triple_ythdf_RNAi_Before_RNAi_feedings/Triple_ythdf_RNAi_Before_RNAi_feedings_10.jpg]

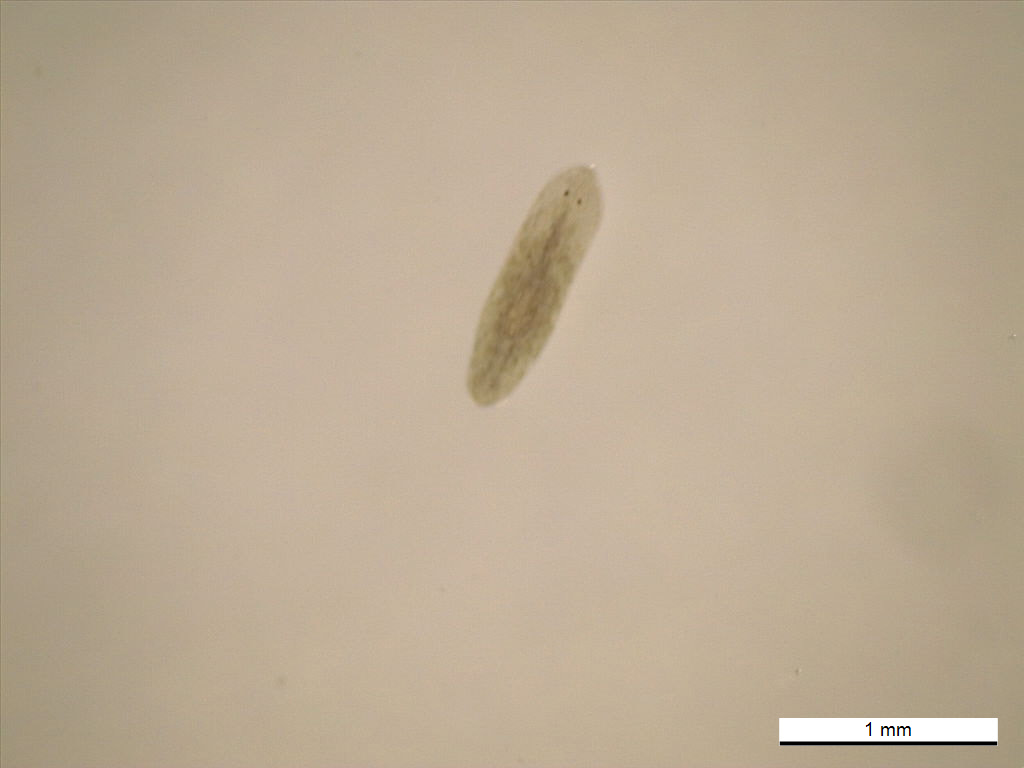

Supplement: Supplementary file 10 — Source data Fig. 3 [file 44318_2025_662_MOESM10_ESM.zip › Figure 3/3C-D/Triple_ythdf_RNAi_Before_RNAi_feedings/Triple_ythdf_RNAi_Before_RNAi_feedings_11.jpg]

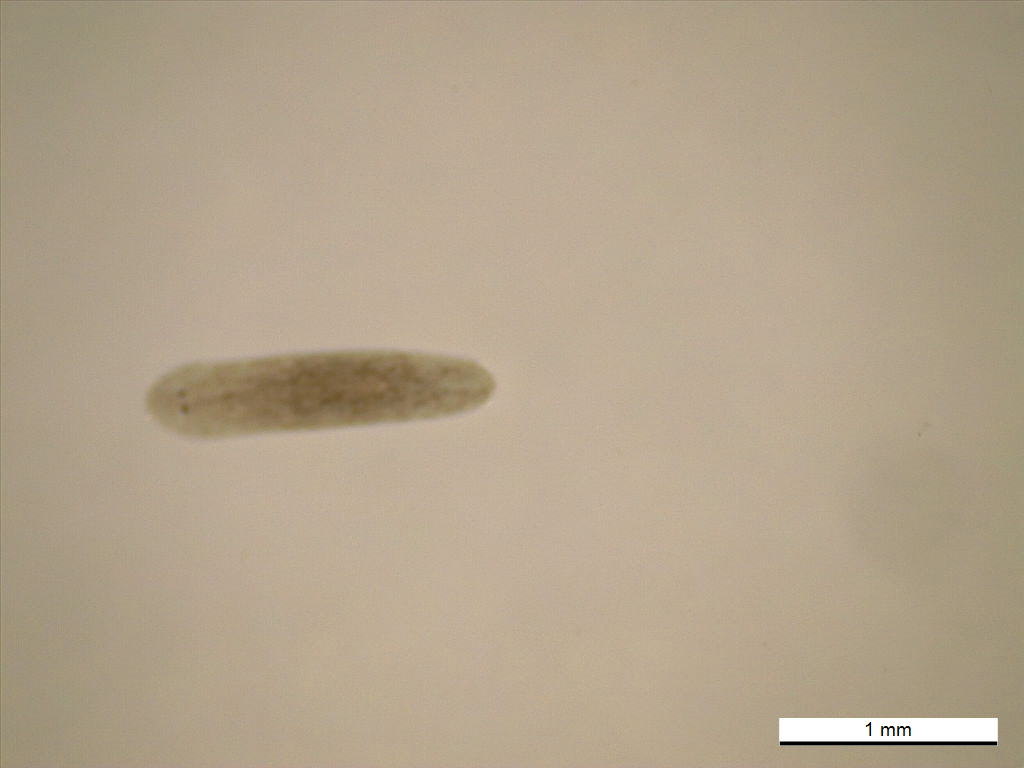

Supplement: Supplementary file 10 — Source data Fig. 3 [file 44318_2025_662_MOESM10_ESM.zip › Figure 3/3C-D/Triple_ythdf_RNAi_Before_RNAi_feedings/Triple_ythdf_RNAi_Before_RNAi_feedings_12.jpg]

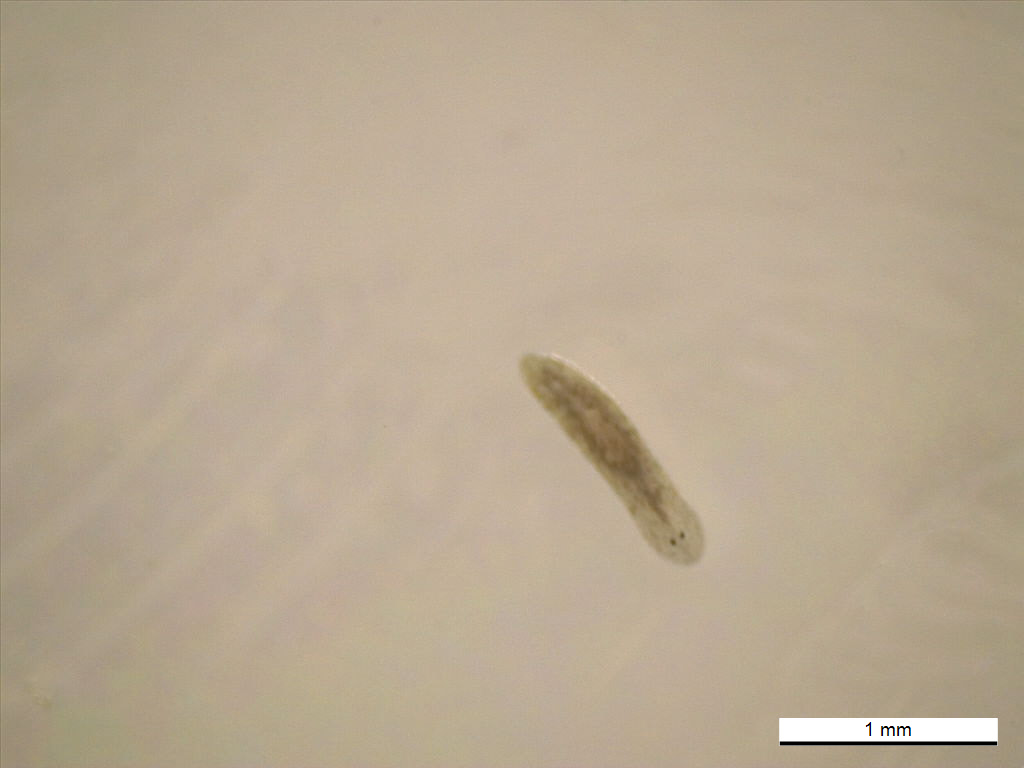

Supplement: Supplementary file 10 — Source data Fig. 3 [file 44318_2025_662_MOESM10_ESM.zip › Figure 3/3C-D/Triple_ythdf_RNAi_Before_RNAi_feedings/Triple_ythdf_RNAi_Before_RNAi_feedings_13.jpg]

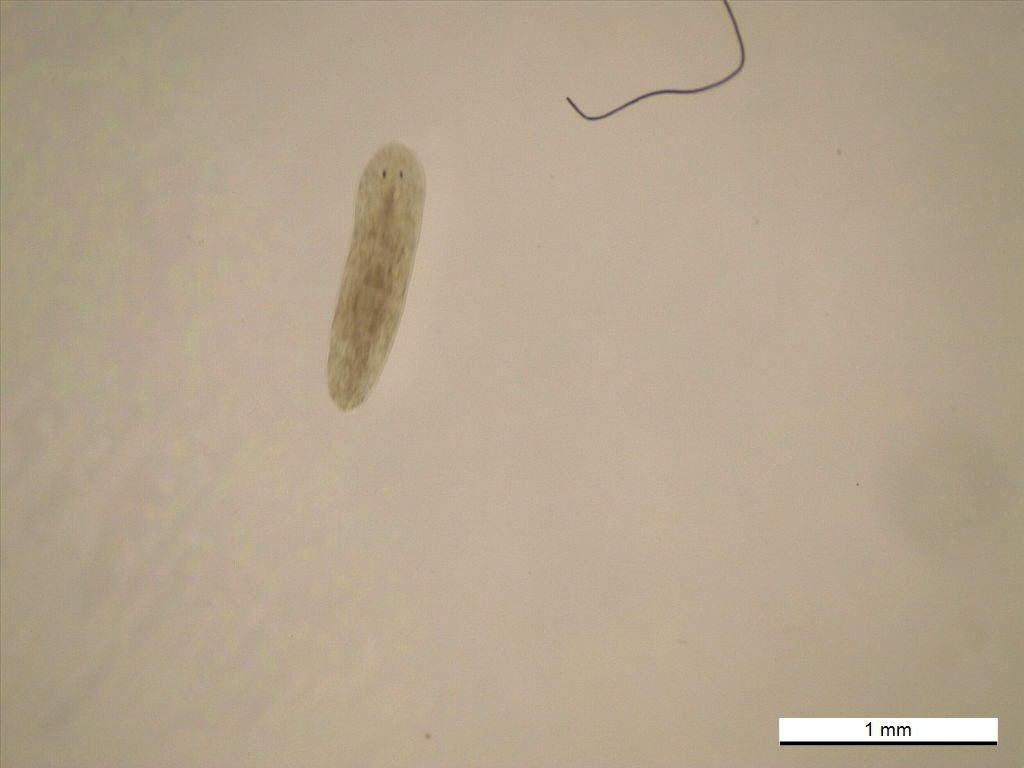

Supplement: Supplementary file 10 — Source data Fig. 3 [file 44318_2025_662_MOESM10_ESM.zip › Figure 3/3C-D/Triple_ythdf_RNAi_Before_RNAi_feedings/Triple_ythdf_RNAi_Before_RNAi_feedings_14.jpg]

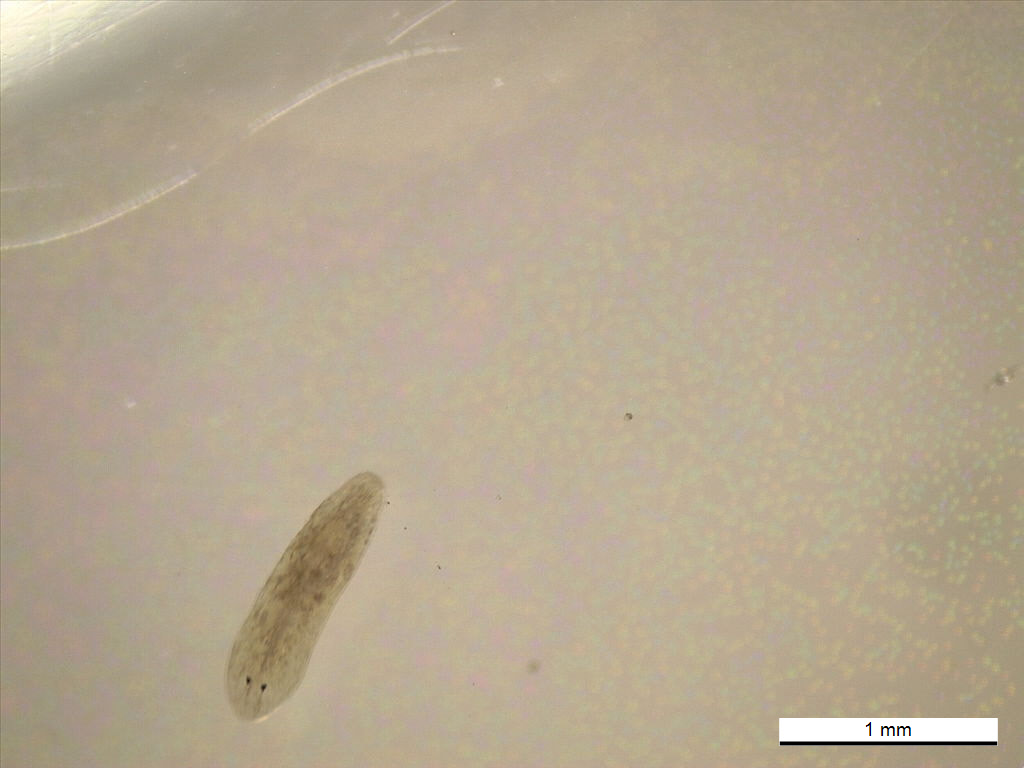

Supplement: Supplementary file 10 — Source data Fig. 3 [file 44318_2025_662_MOESM10_ESM.zip › Figure 3/3C-D/Triple_ythdf_RNAi_Before_RNAi_feedings/Triple_ythdf_RNAi_Before_RNAi_feedings_15.jpg]

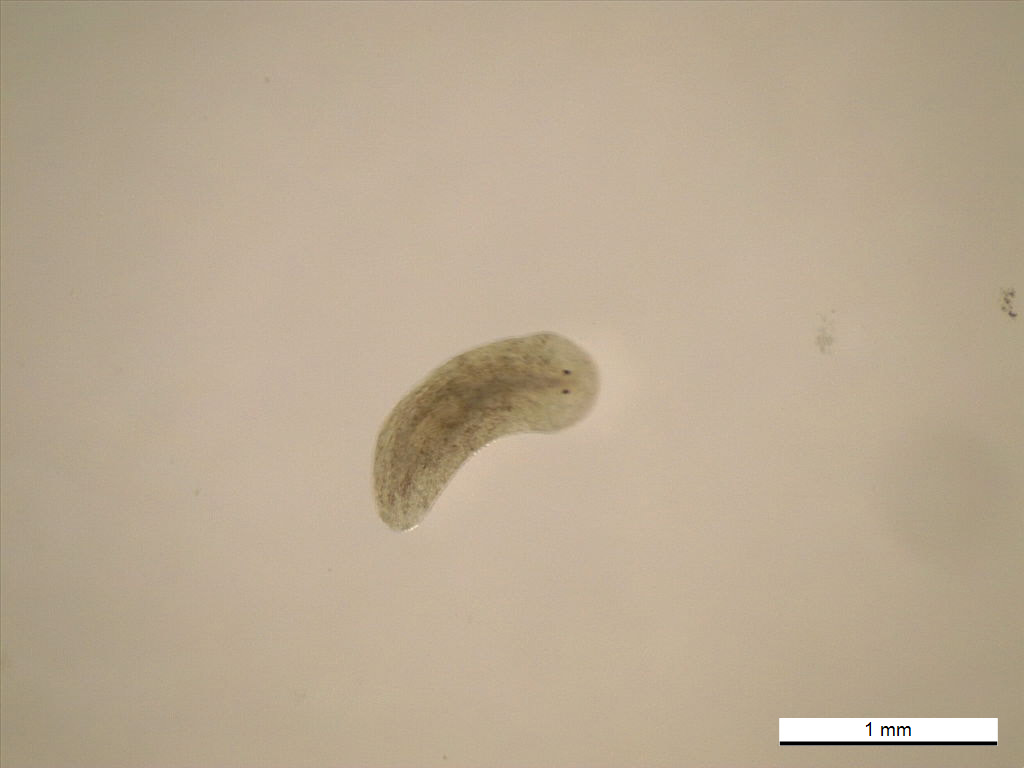

Supplement: Supplementary file 10 — Source data Fig. 3 [file 44318_2025_662_MOESM10_ESM.zip › Figure 3/3C-D/Triple_ythdf_RNAi_Before_RNAi_feedings/Triple_ythdf_RNAi_Before_RNAi_feedings_2.jpg]

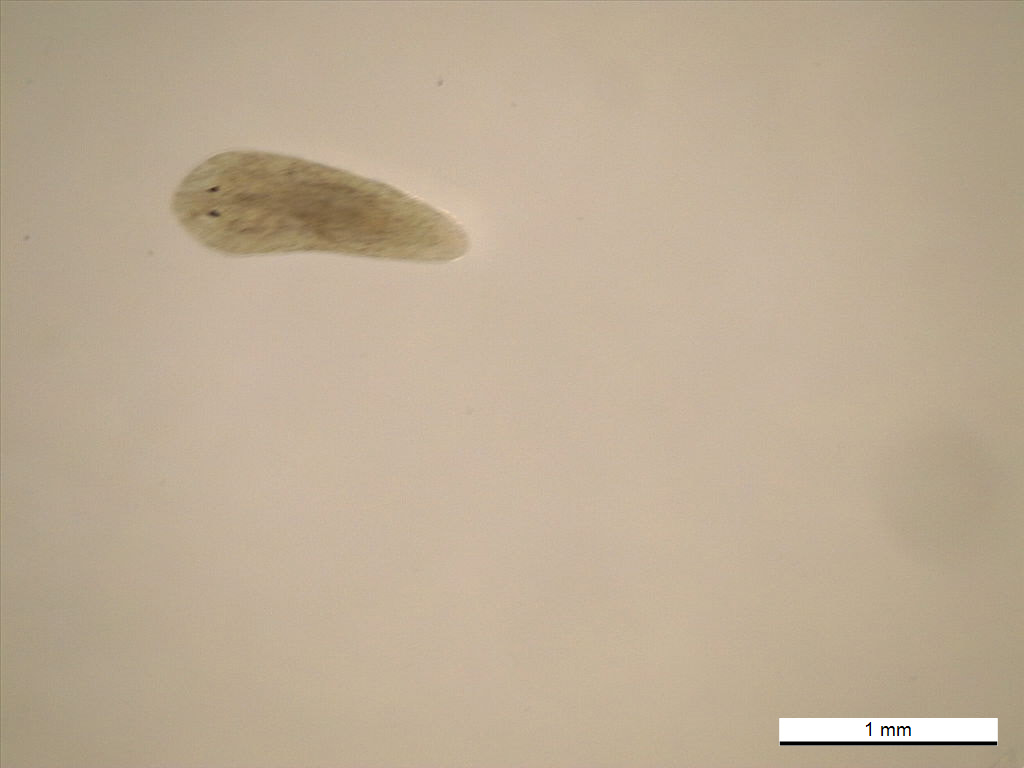

Supplement: Supplementary file 10 — Source data Fig. 3 [file 44318_2025_662_MOESM10_ESM.zip › Figure 3/3C-D/Triple_ythdf_RNAi_Before_RNAi_feedings/Triple_ythdf_RNAi_Before_RNAi_feedings_3.jpg]

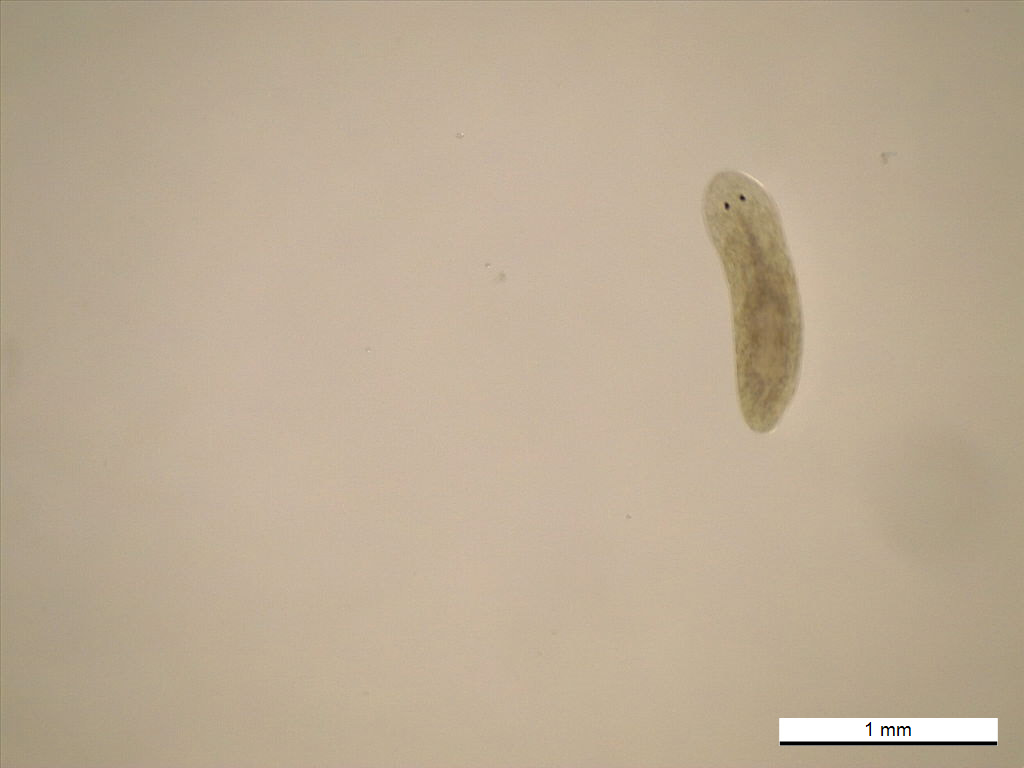

Supplement: Supplementary file 10 — Source data Fig. 3 [file 44318_2025_662_MOESM10_ESM.zip › Figure 3/3C-D/Triple_ythdf_RNAi_Before_RNAi_feedings/Triple_ythdf_RNAi_Before_RNAi_feedings_4.jpg]

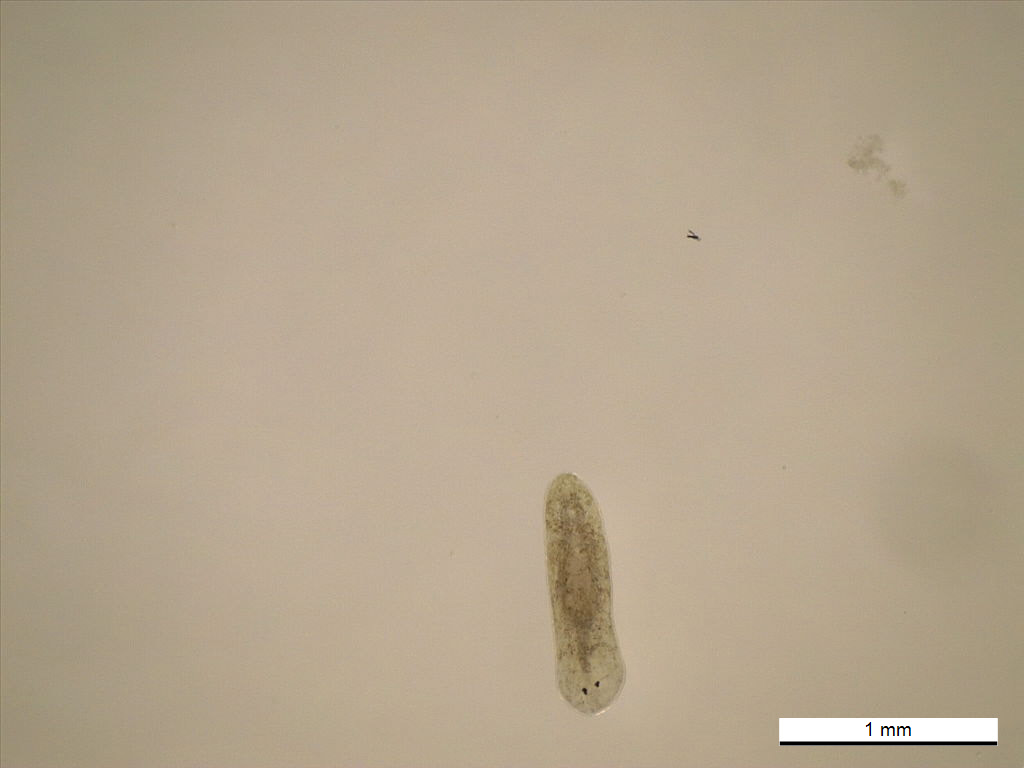

Supplement: Supplementary file 10 — Source data Fig. 3 [file 44318_2025_662_MOESM10_ESM.zip › Figure 3/3C-D/Triple_ythdf_RNAi_Before_RNAi_feedings/Triple_ythdf_RNAi_Before_RNAi_feedings_5.jpg]

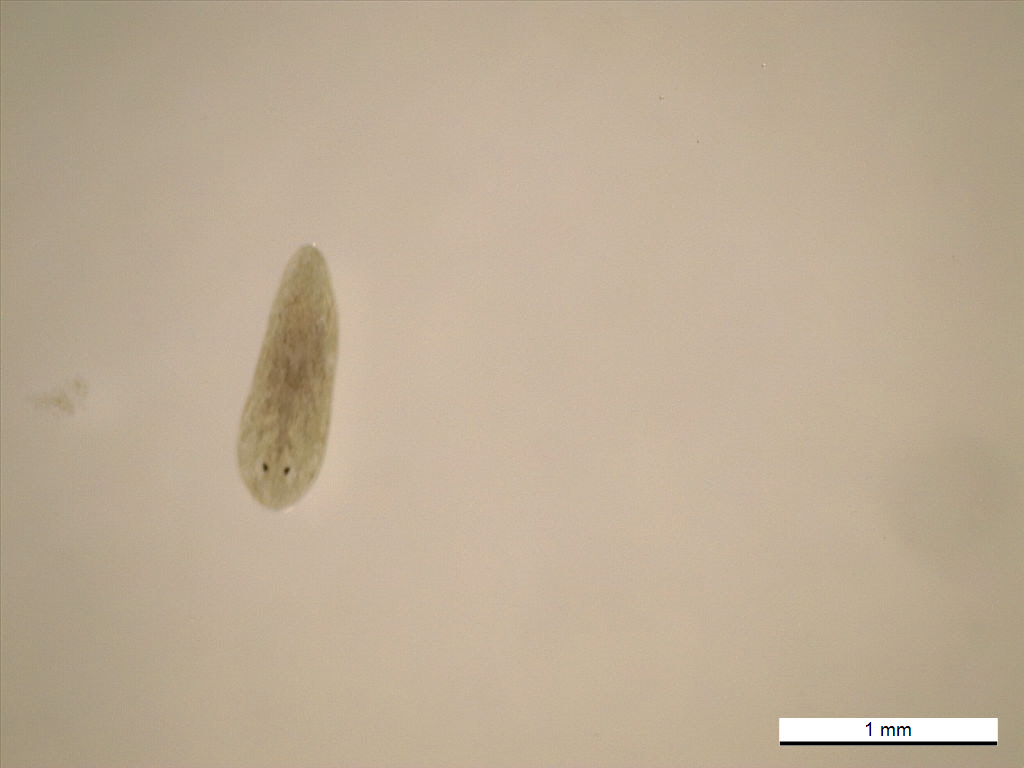

Supplement: Supplementary file 10 — Source data Fig. 3 [file 44318_2025_662_MOESM10_ESM.zip › Figure 3/3C-D/Triple_ythdf_RNAi_Before_RNAi_feedings/Triple_ythdf_RNAi_Before_RNAi_feedings_6.jpg]

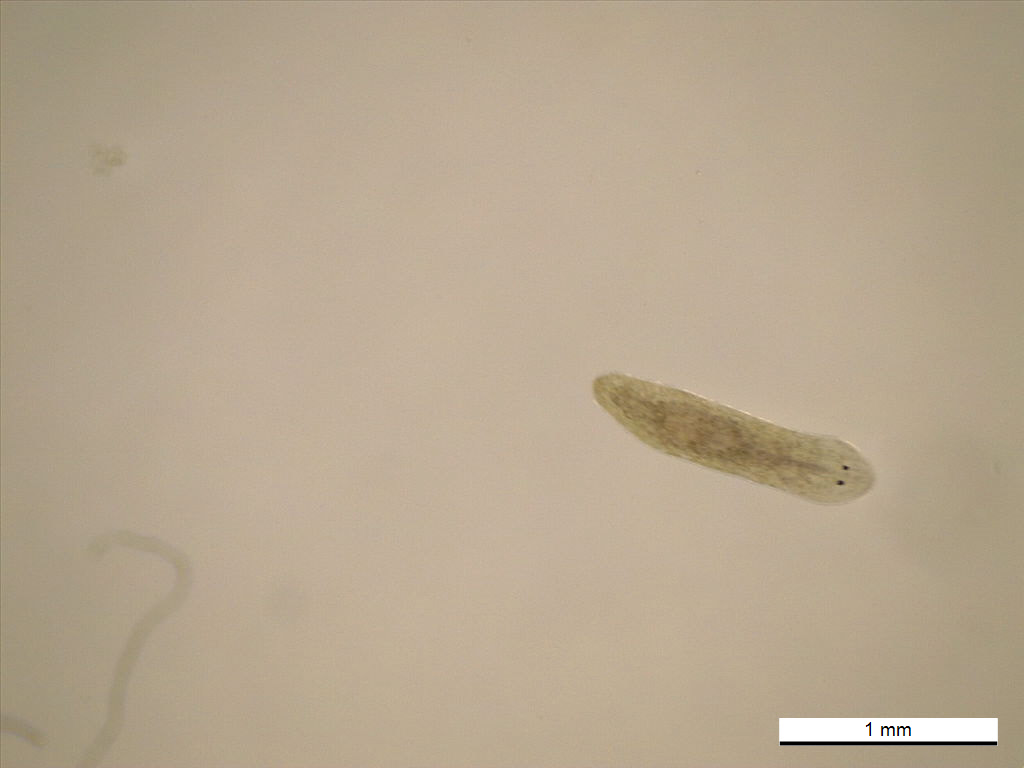

Supplement: Supplementary file 10 — Source data Fig. 3 [file 44318_2025_662_MOESM10_ESM.zip › Figure 3/3C-D/Triple_ythdf_RNAi_Before_RNAi_feedings/Triple_ythdf_RNAi_Before_RNAi_feedings_7.jpg]

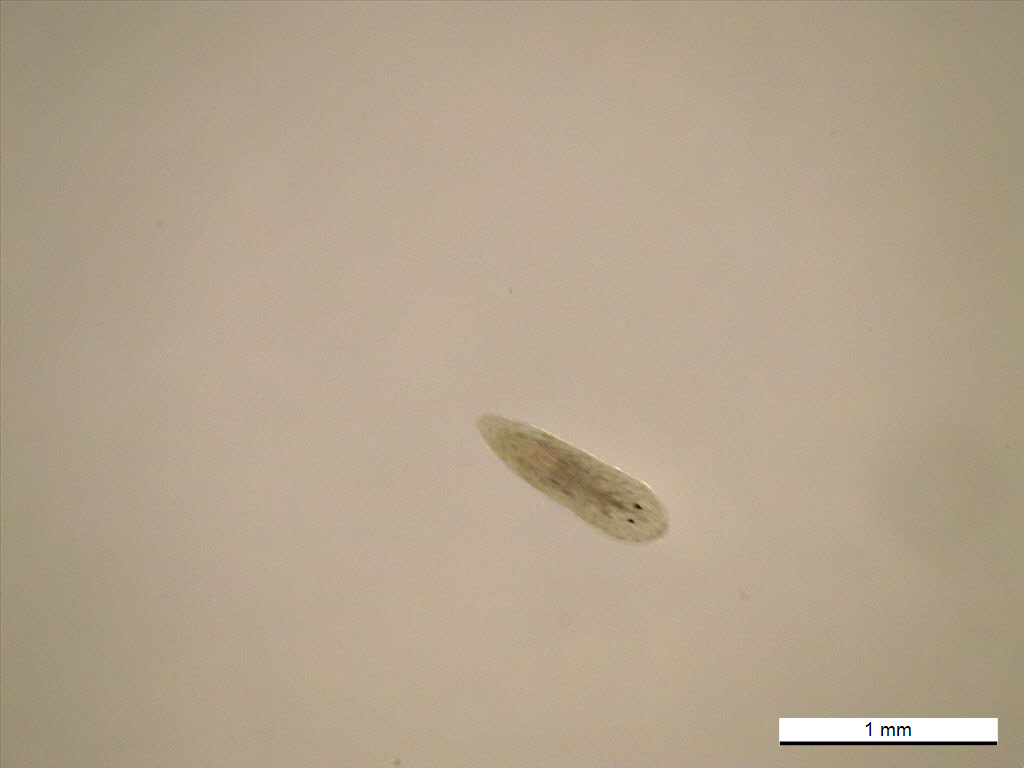

Supplement: Supplementary file 10 — Source data Fig. 3 [file 44318_2025_662_MOESM10_ESM.zip › Figure 3/3C-D/Triple_ythdf_RNAi_Before_RNAi_feedings/Triple_ythdf_RNAi_Before_RNAi_feedings_8.jpg]

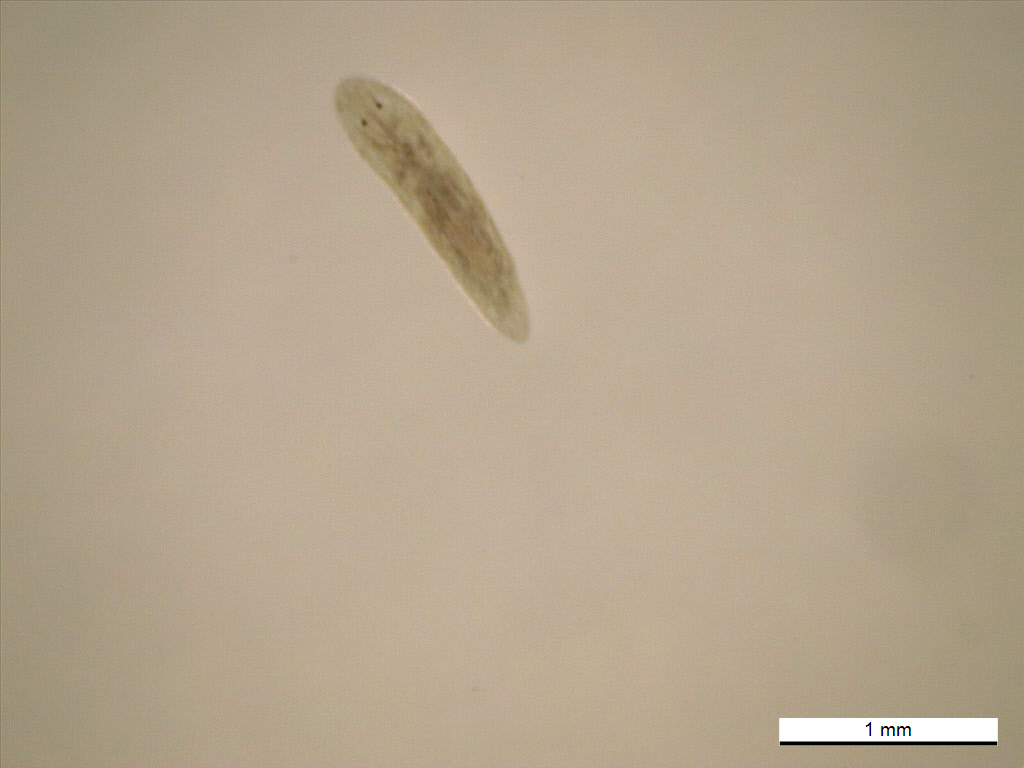

Supplement: Supplementary file 10 — Source data Fig. 3 [file 44318_2025_662_MOESM10_ESM.zip › Figure 3/3C-D/Triple_ythdf_RNAi_Before_RNAi_feedings/Triple_ythdf_RNAi_Before_RNAi_feedings_9.jpg]

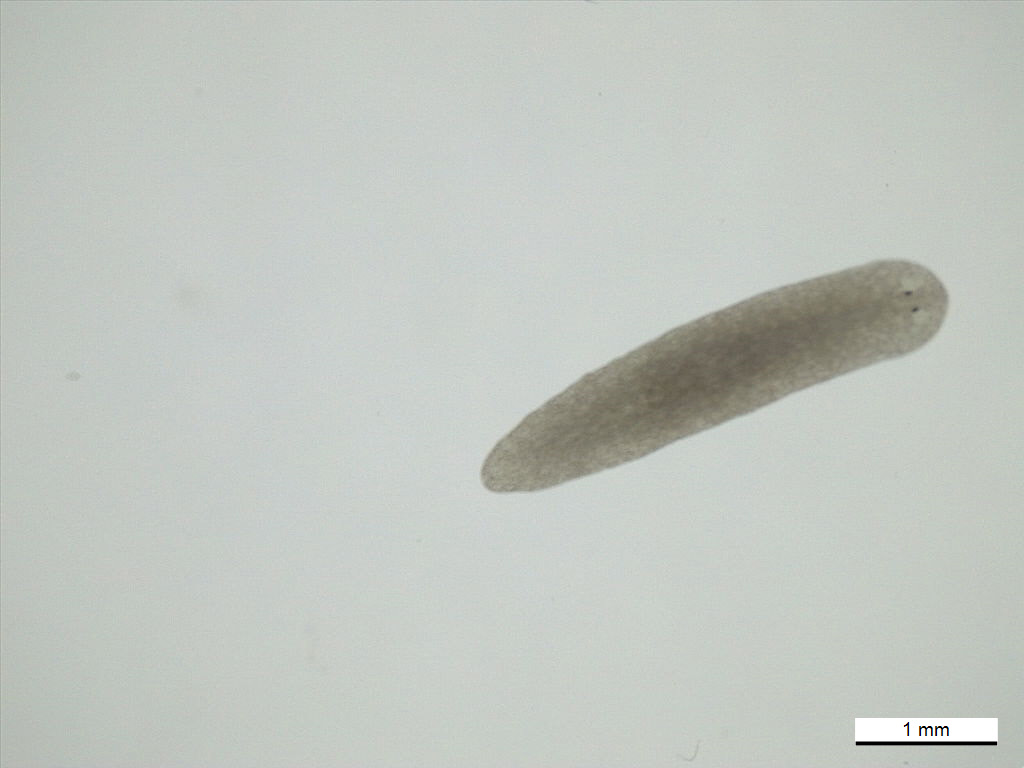

Supplement: Supplementary file 10 — Source data Fig. 3 [file 44318_2025_662_MOESM10_ESM.zip › Figure 3/3C-D/ythdf-a-b_RNAi_After_10_RNAi_feedings/ythdf_a-b_RNAi_After_10_RNAi_feedings_01.jpg]

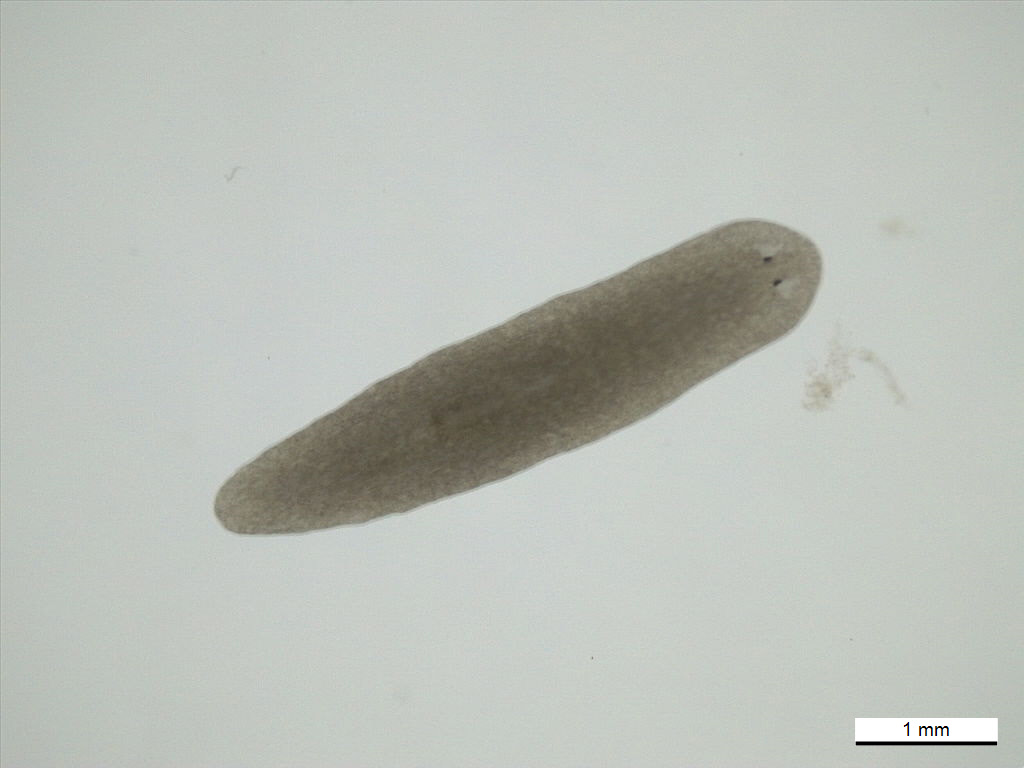

Supplement: Supplementary file 10 — Source data Fig. 3 [file 44318_2025_662_MOESM10_ESM.zip › Figure 3/3C-D/ythdf-a-b_RNAi_After_10_RNAi_feedings/ythdf_a-b_RNAi_After_10_RNAi_feedings_02.jpg]

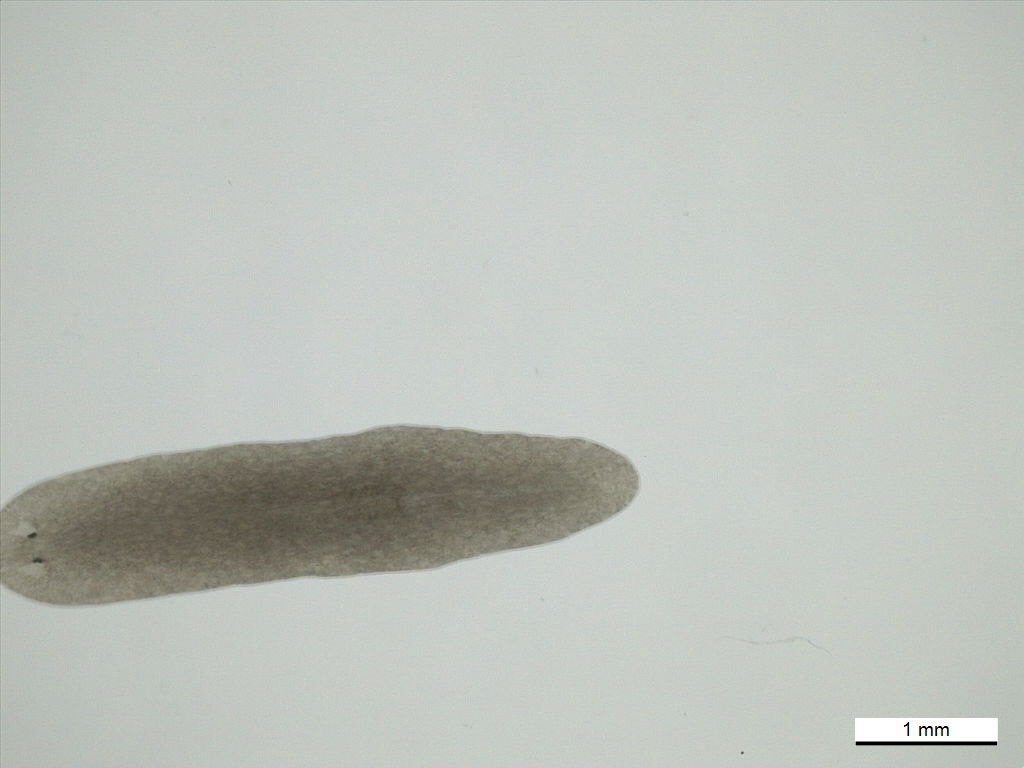

Supplement: Supplementary file 10 — Source data Fig. 3 [file 44318_2025_662_MOESM10_ESM.zip › Figure 3/3C-D/ythdf-a-b_RNAi_After_10_RNAi_feedings/ythdf_a-b_RNAi_After_10_RNAi_feedings_03.jpg]

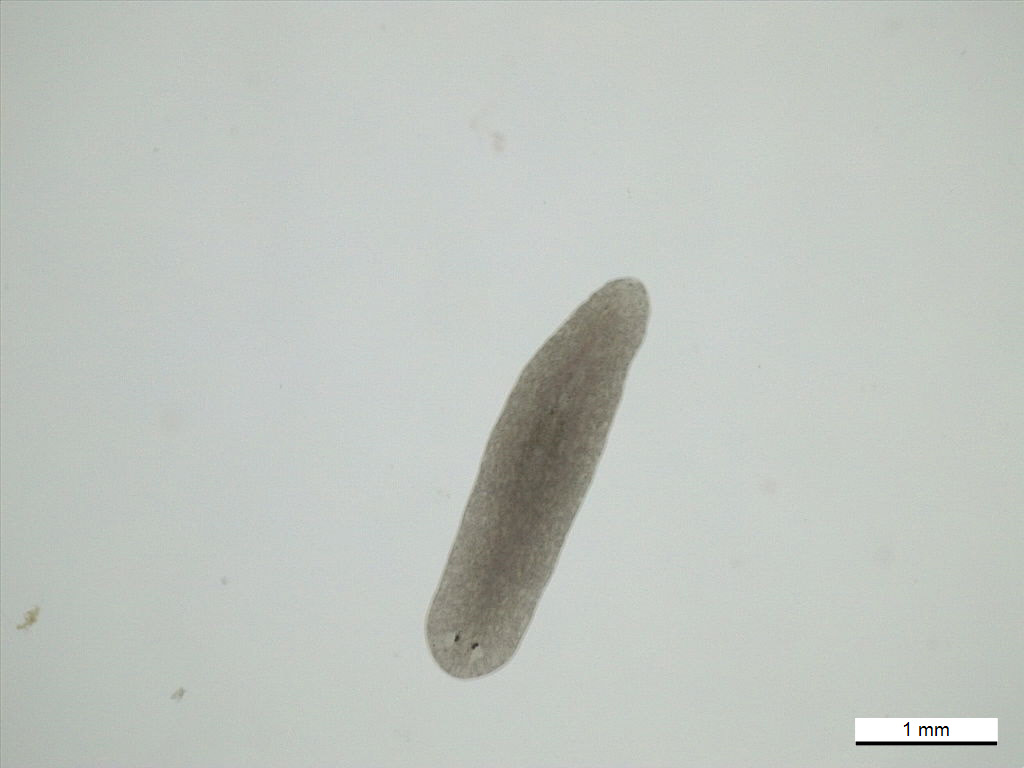

Supplement: Supplementary file 10 — Source data Fig. 3 [file 44318_2025_662_MOESM10_ESM.zip › Figure 3/3C-D/ythdf-a-b_RNAi_After_10_RNAi_feedings/ythdf_a-b_RNAi_After_10_RNAi_feedings_04.jpg]

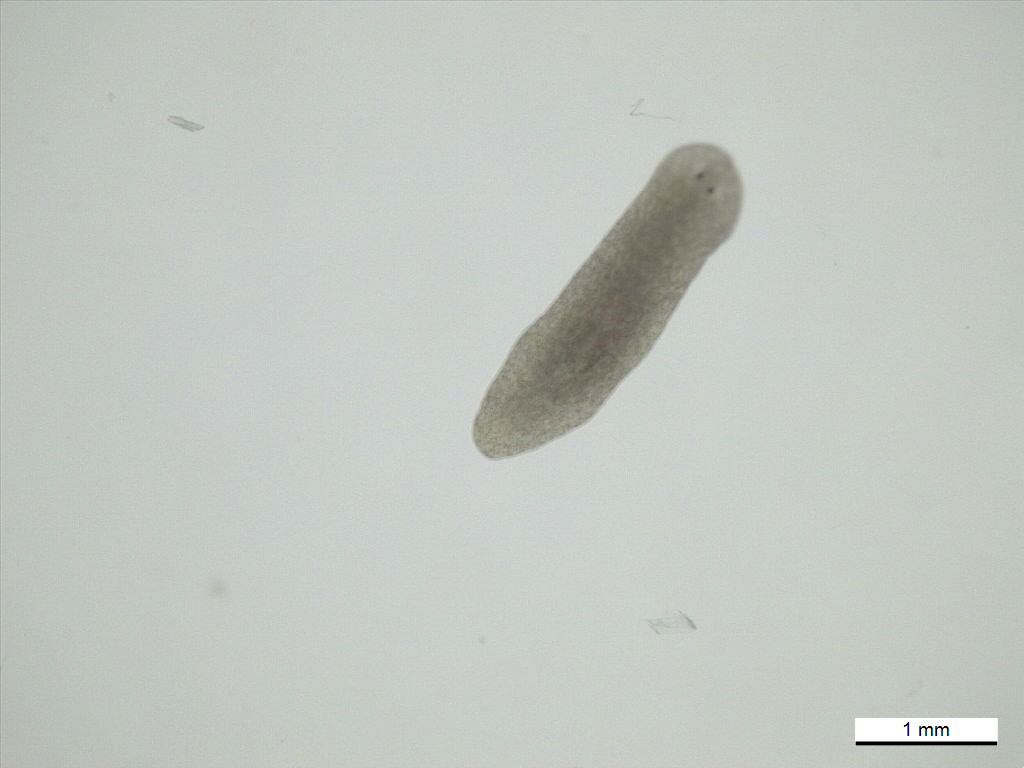

Supplement: Supplementary file 10 — Source data Fig. 3 [file 44318_2025_662_MOESM10_ESM.zip › Figure 3/3C-D/ythdf-a-b_RNAi_After_10_RNAi_feedings/ythdf_a-b_RNAi_After_10_RNAi_feedings_05.jpg]

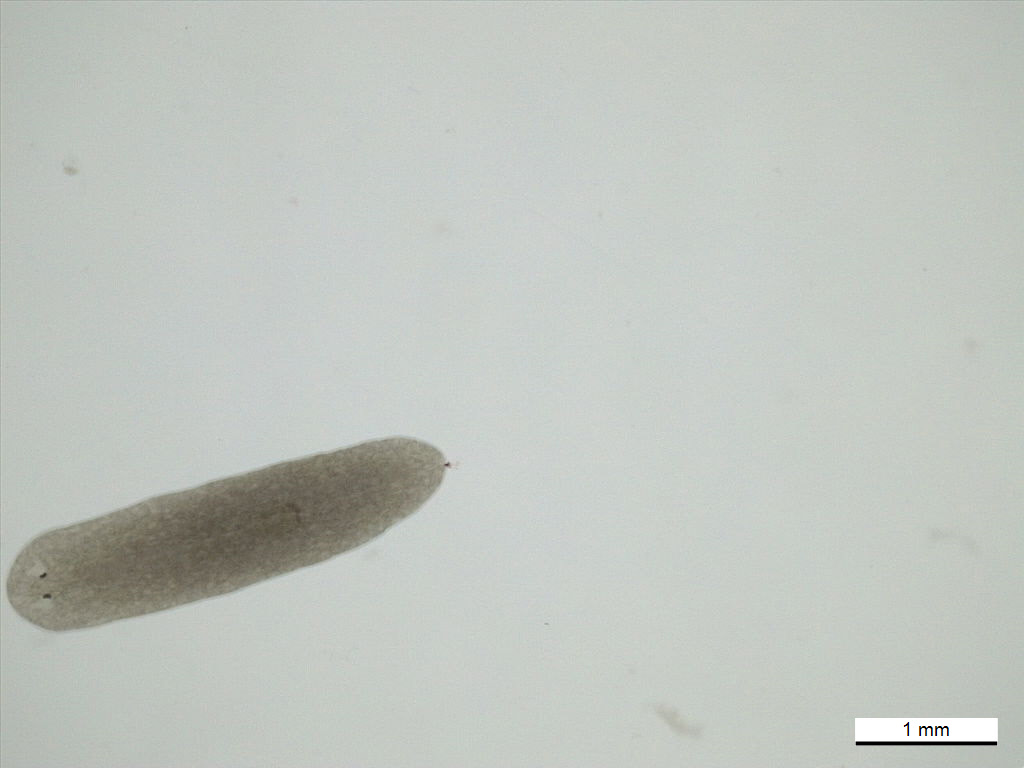

Supplement: Supplementary file 10 — Source data Fig. 3 [file 44318_2025_662_MOESM10_ESM.zip › Figure 3/3C-D/ythdf-a-b_RNAi_After_10_RNAi_feedings/ythdf_a-b_RNAi_After_10_RNAi_feedings_06.jpg]

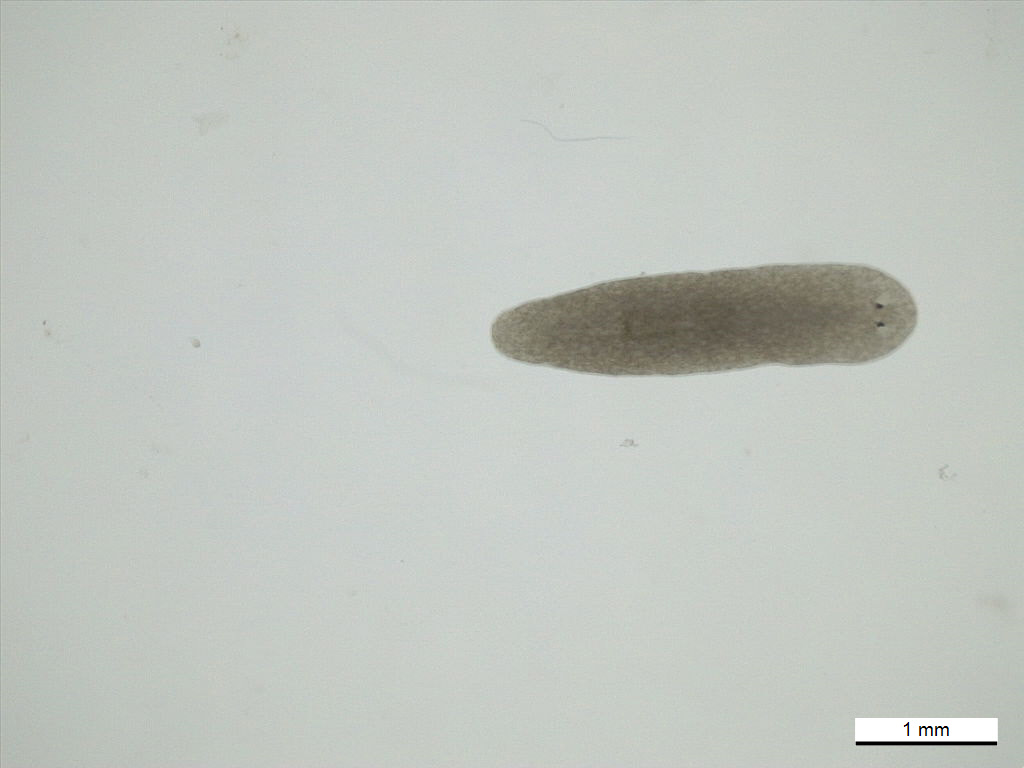

Supplement: Supplementary file 10 — Source data Fig. 3 [file 44318_2025_662_MOESM10_ESM.zip › Figure 3/3C-D/ythdf-a-b_RNAi_After_10_RNAi_feedings/ythdf_a-b_RNAi_After_10_RNAi_feedings_07.jpg]

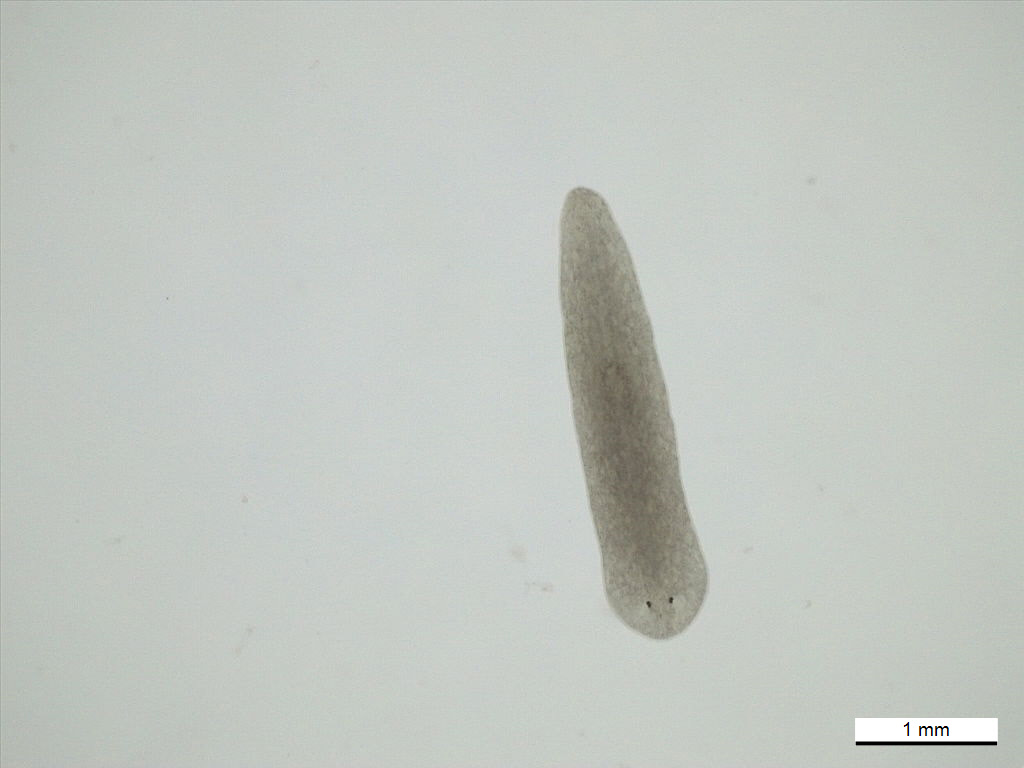

Supplement: Supplementary file 10 — Source data Fig. 3 [file 44318_2025_662_MOESM10_ESM.zip › Figure 3/3C-D/ythdf-a-b_RNAi_After_10_RNAi_feedings/ythdf_a-b_RNAi_After_10_RNAi_feedings_08.jpg]

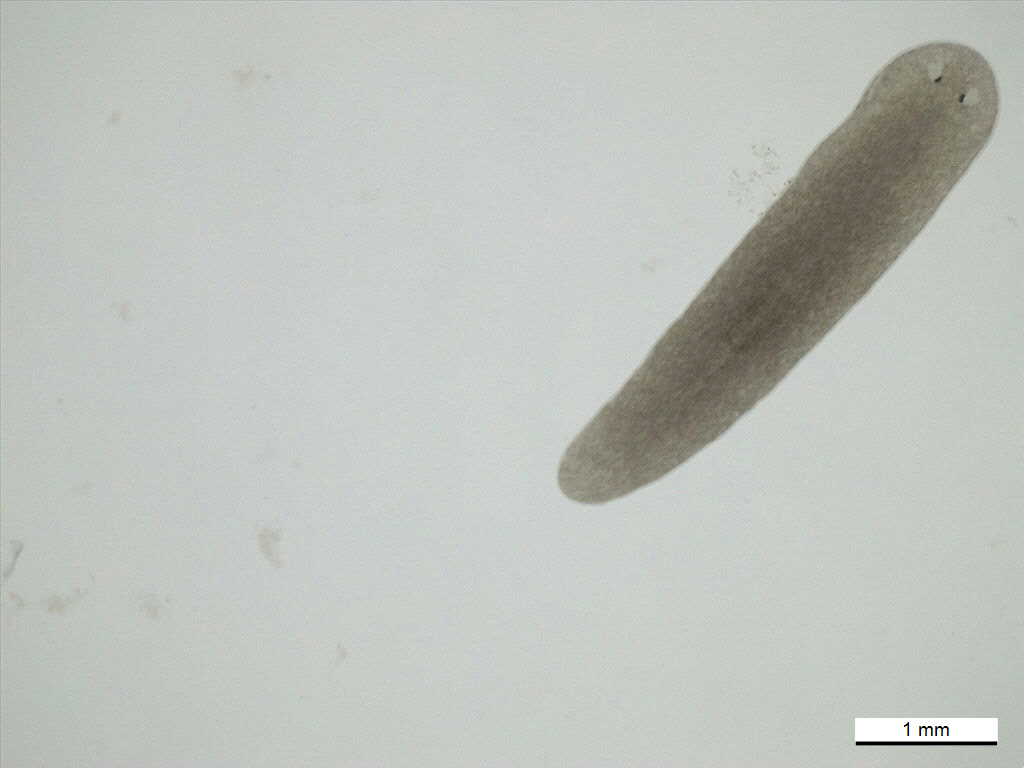

Supplement: Supplementary file 10 — Source data Fig. 3 [file 44318_2025_662_MOESM10_ESM.zip › Figure 3/3C-D/ythdf-a-b_RNAi_After_10_RNAi_feedings/ythdf_a-b_RNAi_After_10_RNAi_feedings_09.jpg]

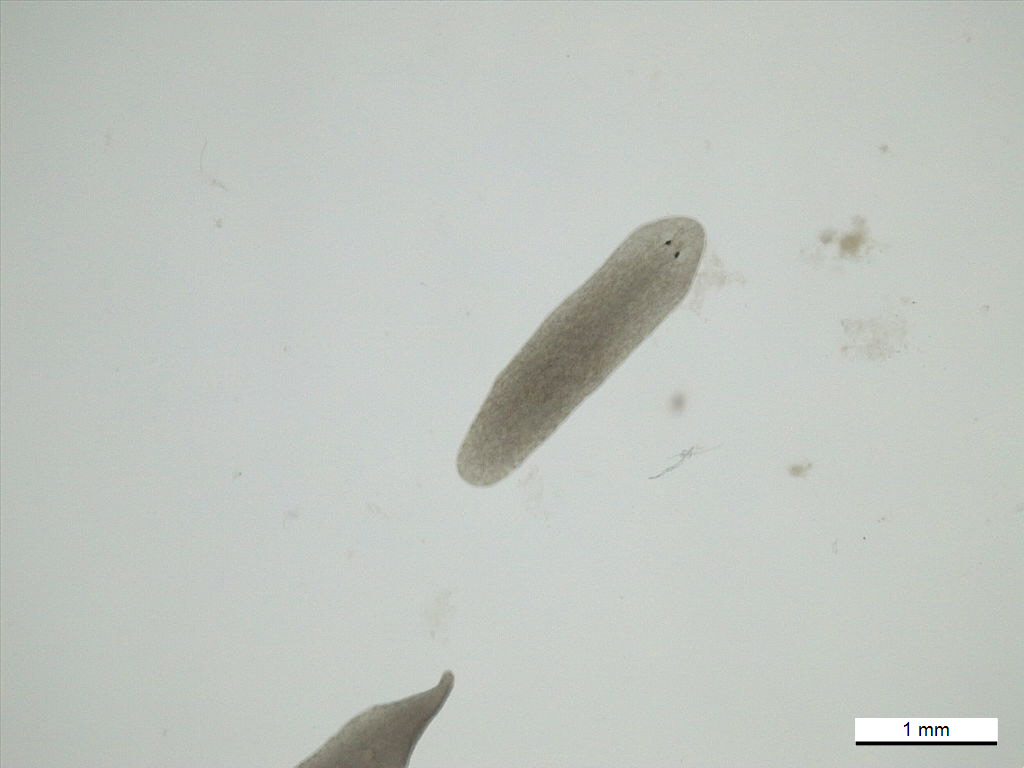

Supplement: Supplementary file 10 — Source data Fig. 3 [file 44318_2025_662_MOESM10_ESM.zip › Figure 3/3C-D/ythdf-a-b_RNAi_After_10_RNAi_feedings/ythdf_a-b_RNAi_After_10_RNAi_feedings_10.jpg]
