# Supplementary figures and images for: Single-nucleotide m⁶A mapping uncovers redundant YTHDF function in planarian progenitor fate selection (part 3 of 6)
Source: EMBO J. 2026 Jan 3;45(3):749–88. doi: 10.1038/s44318-025-00662-3 (PMC12864844; doi:10.1038/s44318-025-00662-3)

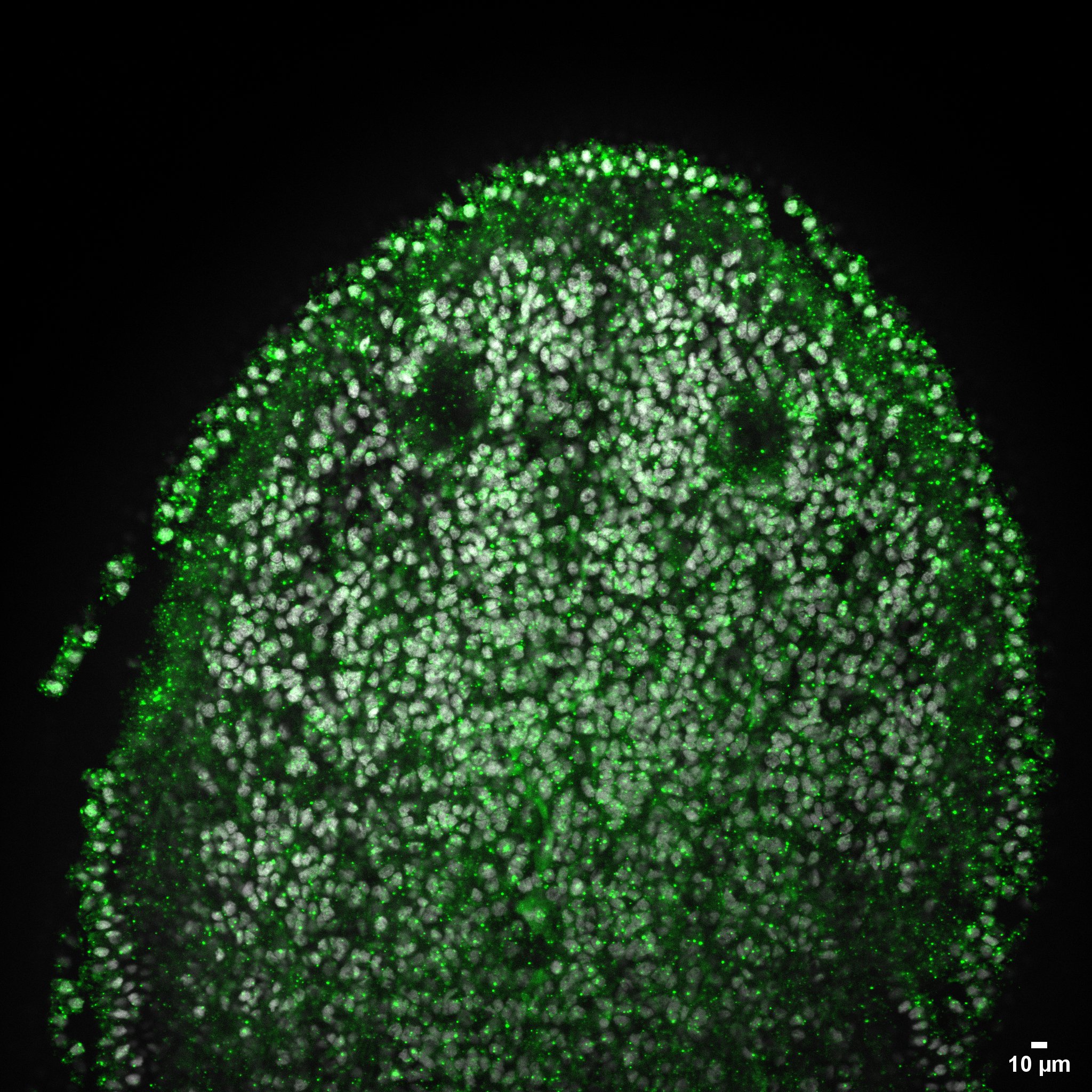

Supplement: Supplementary file 11 — Source data Fig. 4 [file 44318_2025_662_MOESM11_ESM.zip › Figure 4/4A/wildtype_ythdf-b_FITC-green_ythdf-c_Rhod-magenta_20x_Epidermis_FITC_channel.jpg]

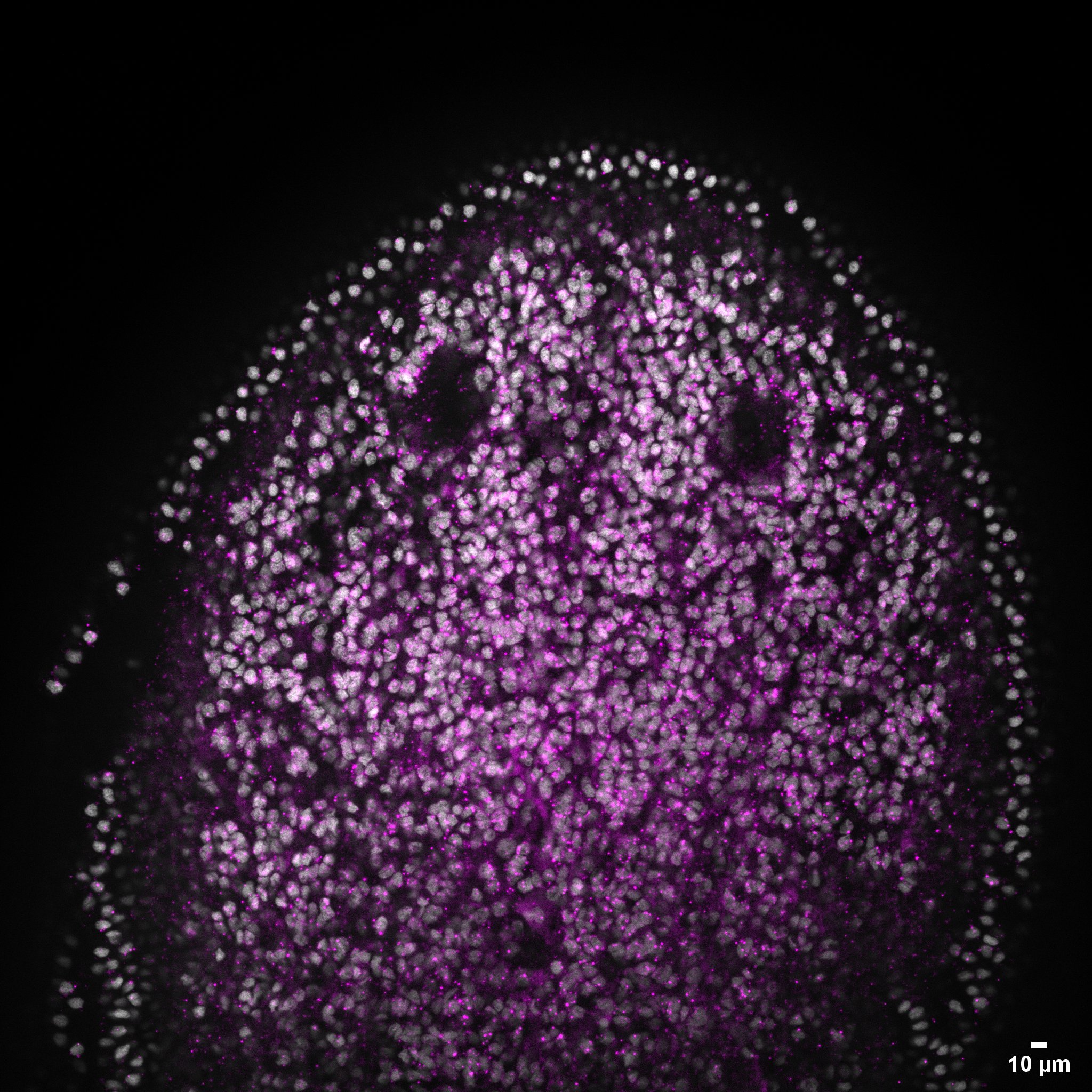

Supplement: Supplementary file 11 — Source data Fig. 4 [file 44318_2025_662_MOESM11_ESM.zip › Figure 4/4A/wildtype_ythdf-b_FITC-green_ythdf-c_Rhod-magenta_20x_Epidermis_Magenta_channel.jpg]

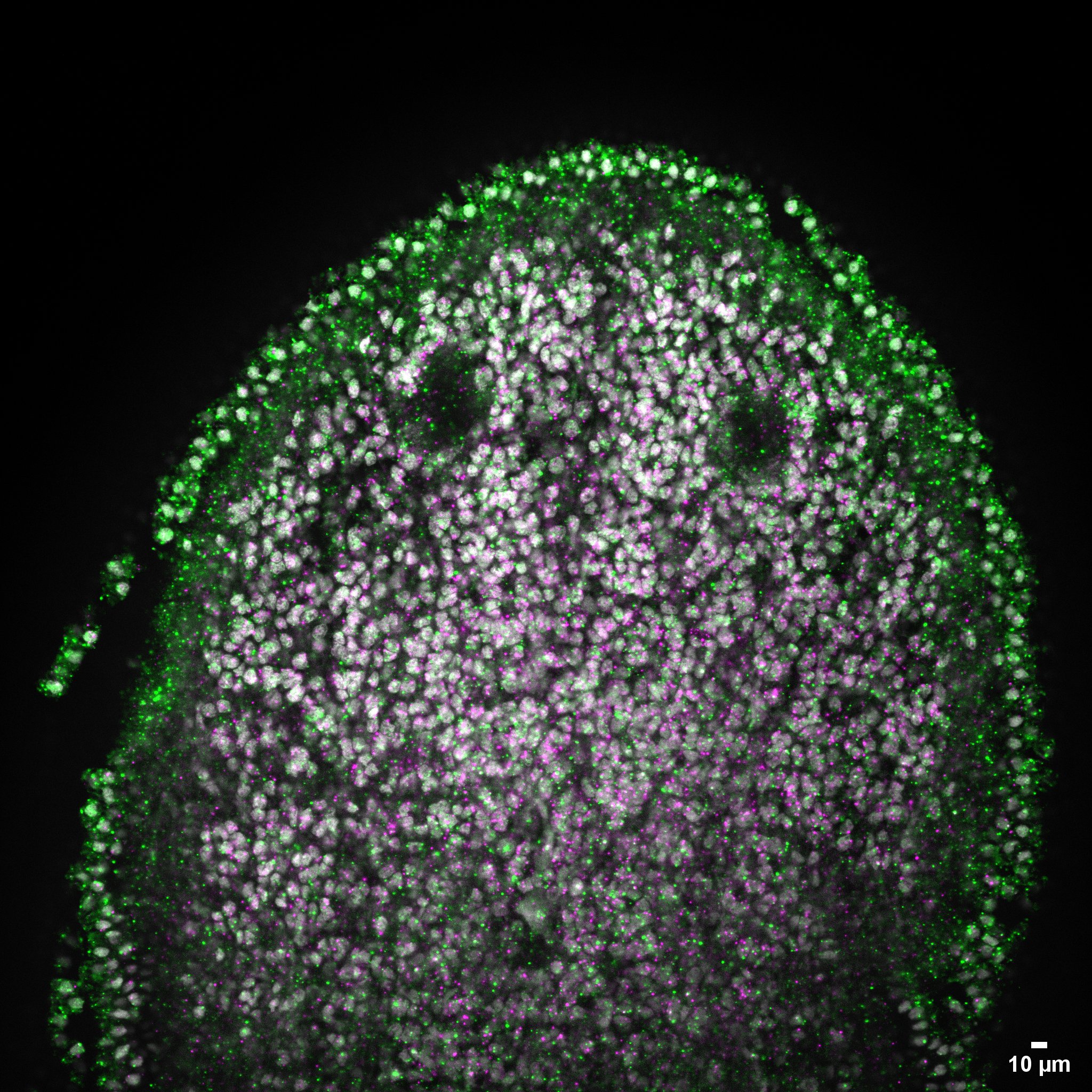

Supplement: Supplementary file 11 — Source data Fig. 4 [file 44318_2025_662_MOESM11_ESM.zip › Figure 4/4A/wildtype_ythdf-b_FITC-green_ythdf-c_Rhod-magenta_20x_Epidermis_Merged.jpg]

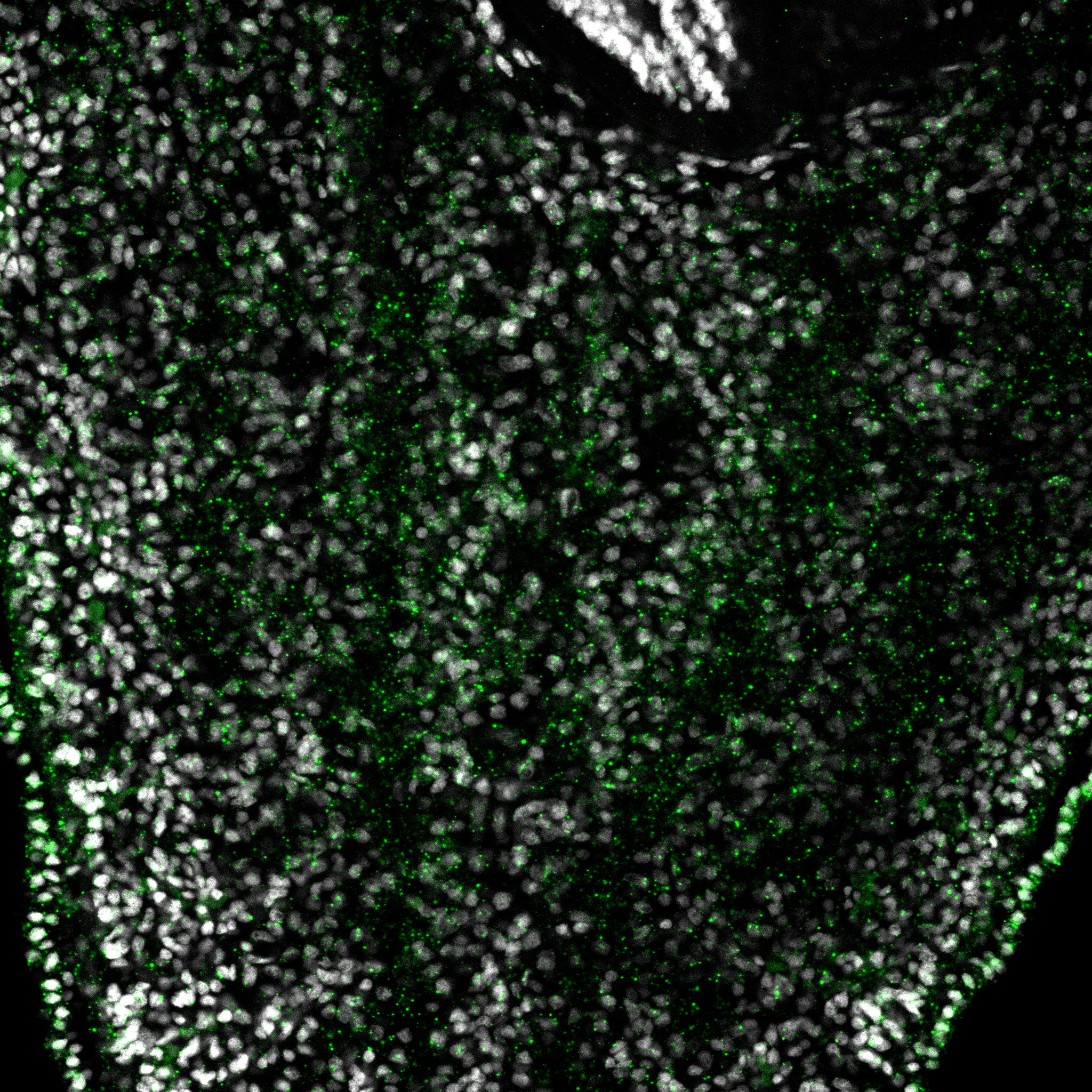

Supplement: Supplementary file 11 — Source data Fig. 4 [file 44318_2025_662_MOESM11_ESM.zip › Figure 4/4A/wildtype_ythdf-b_FITC-green_ythdf-c_Rhod-magenta_20x_intestine_FITC_channel.jpg]

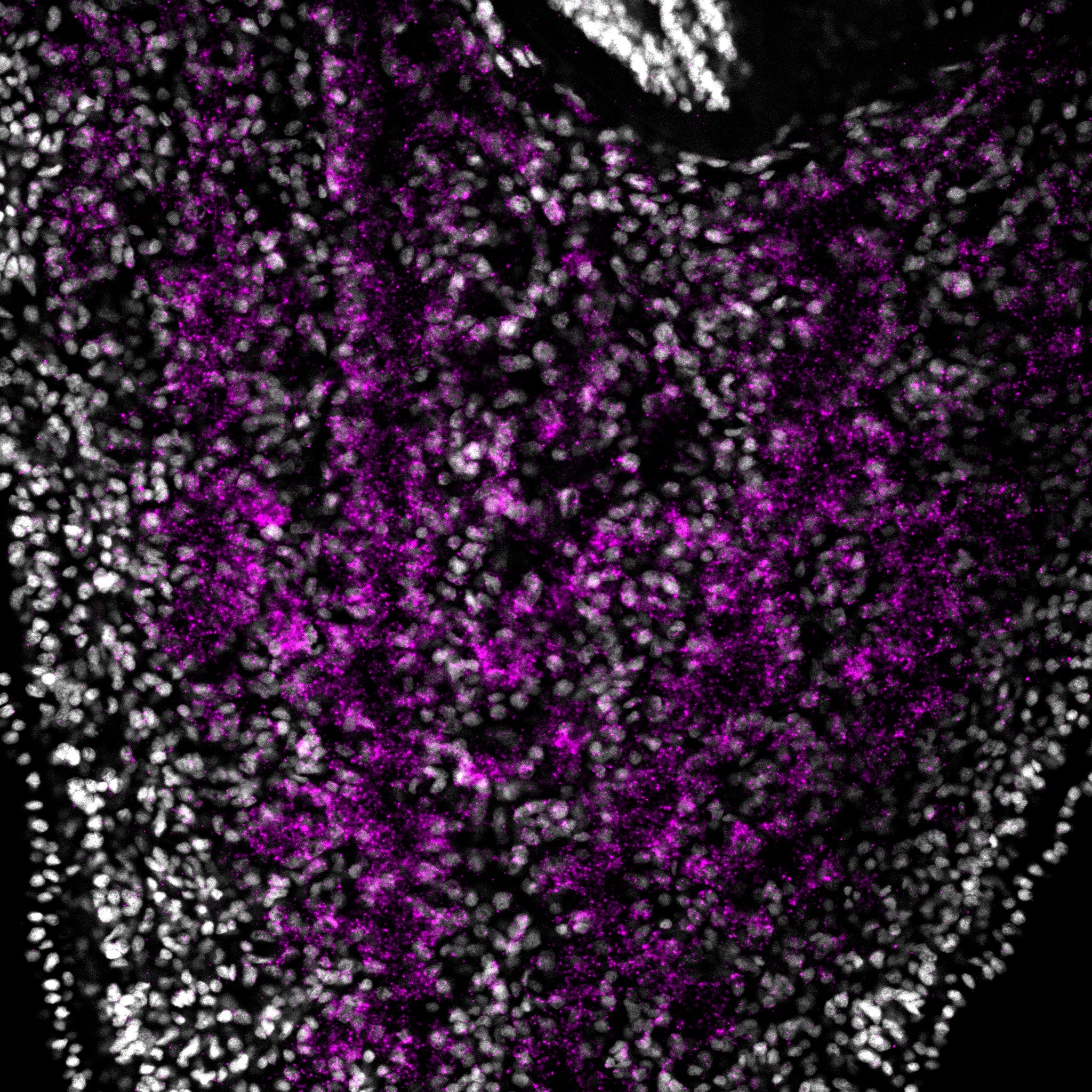

Supplement: Supplementary file 11 — Source data Fig. 4 [file 44318_2025_662_MOESM11_ESM.zip › Figure 4/4A/wildtype_ythdf-b_FITC-green_ythdf-c_Rhod-magenta_20x_intestine_Magenta_channel.jpg]

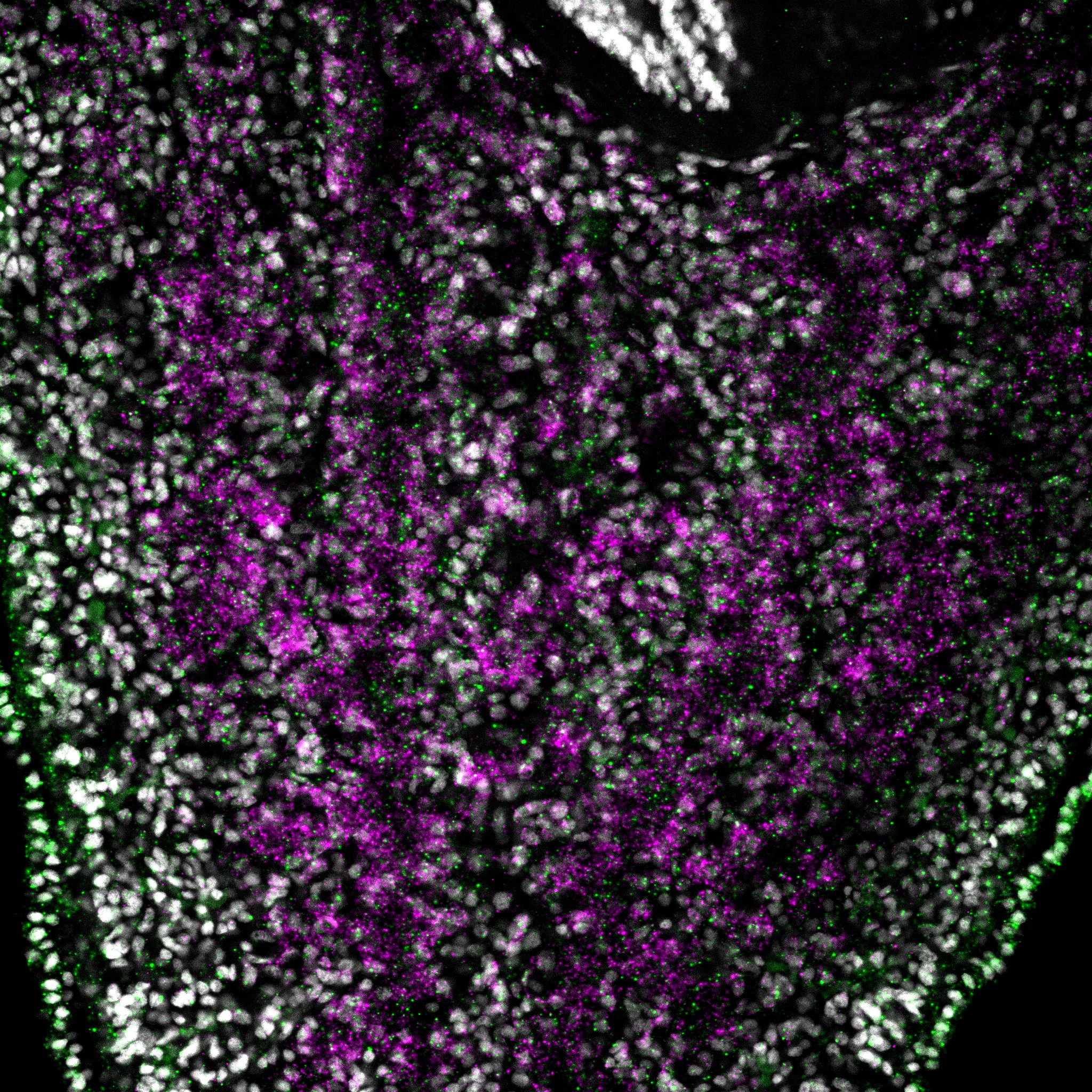

Supplement: Supplementary file 11 — Source data Fig. 4 [file 44318_2025_662_MOESM11_ESM.zip › Figure 4/4A/wildtype_ythdf-b_FITC-green_ythdf-c_Rhod-magenta_20x_intestine_Merged.jpg]

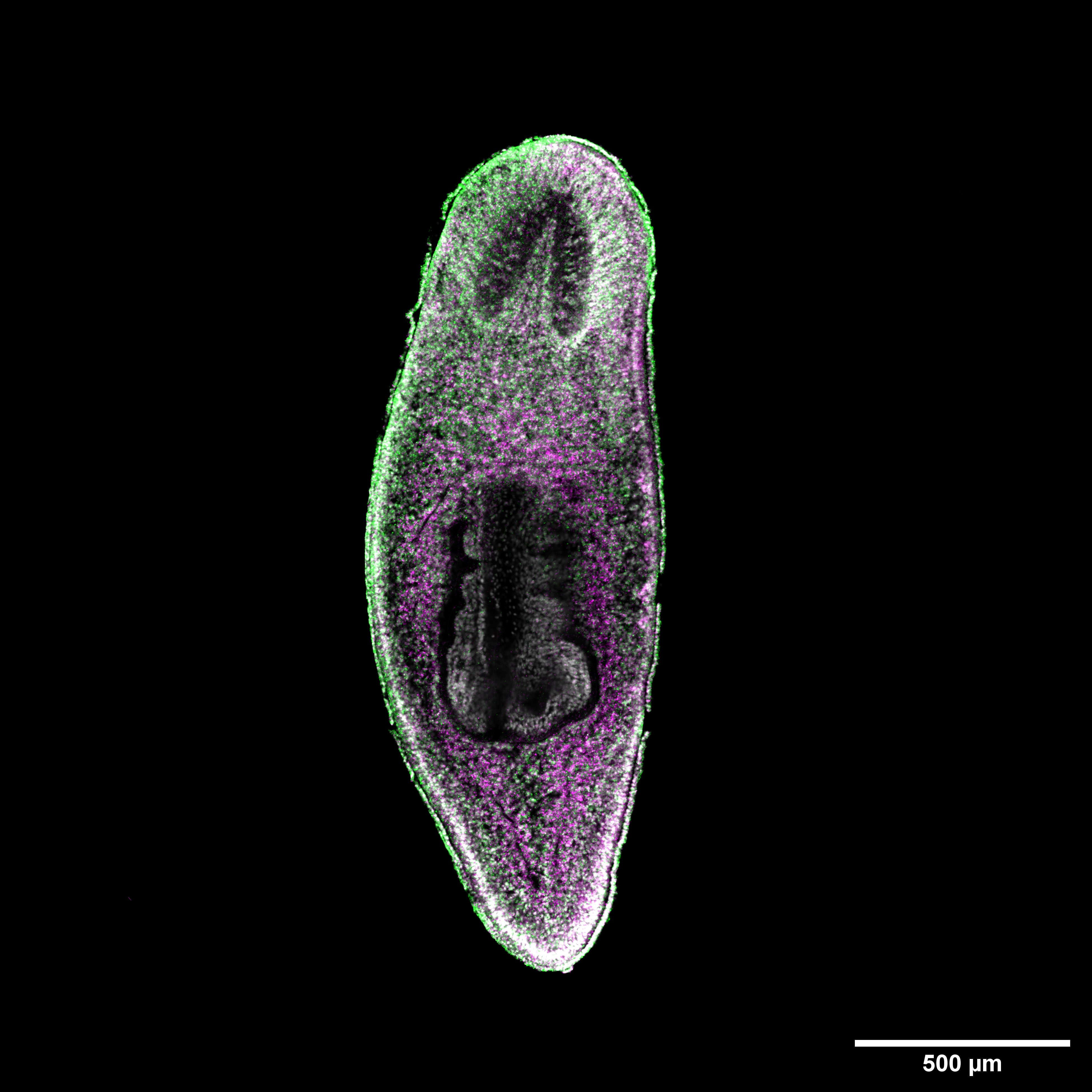

Supplement: Supplementary file 11 — Source data Fig. 4 [file 44318_2025_662_MOESM11_ESM.zip › Figure 4/4A/wildtype_ythdf-c_FITC-green_ythdf-a_Rhod-magenta_10x_stitched.jpg]

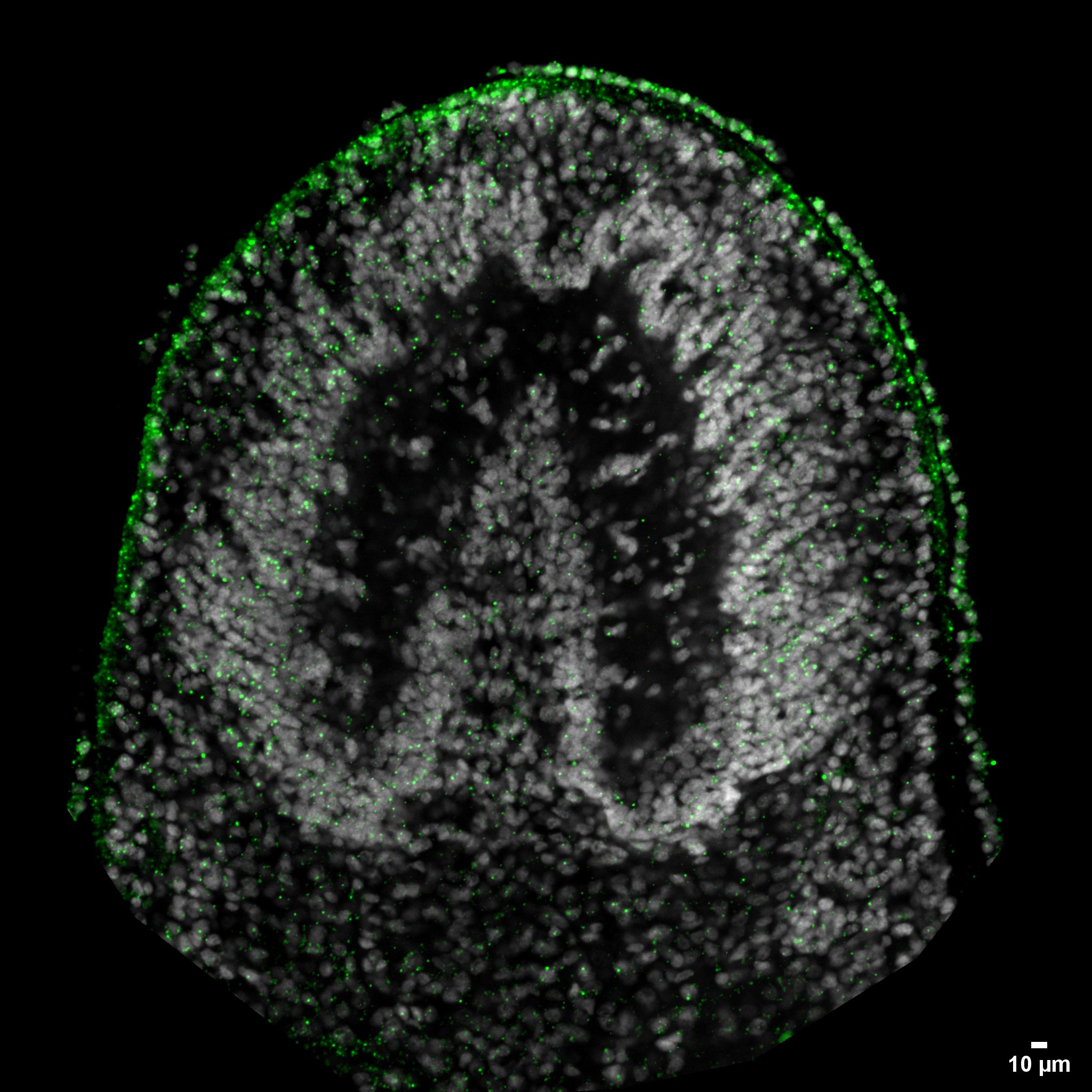

Supplement: Supplementary file 11 — Source data Fig. 4 [file 44318_2025_662_MOESM11_ESM.zip › Figure 4/4A/wildtype_ythdf-c_FITC-green_ythdf-a_Rhod-magenta_20x_Brain_FITC_channel.jpg]

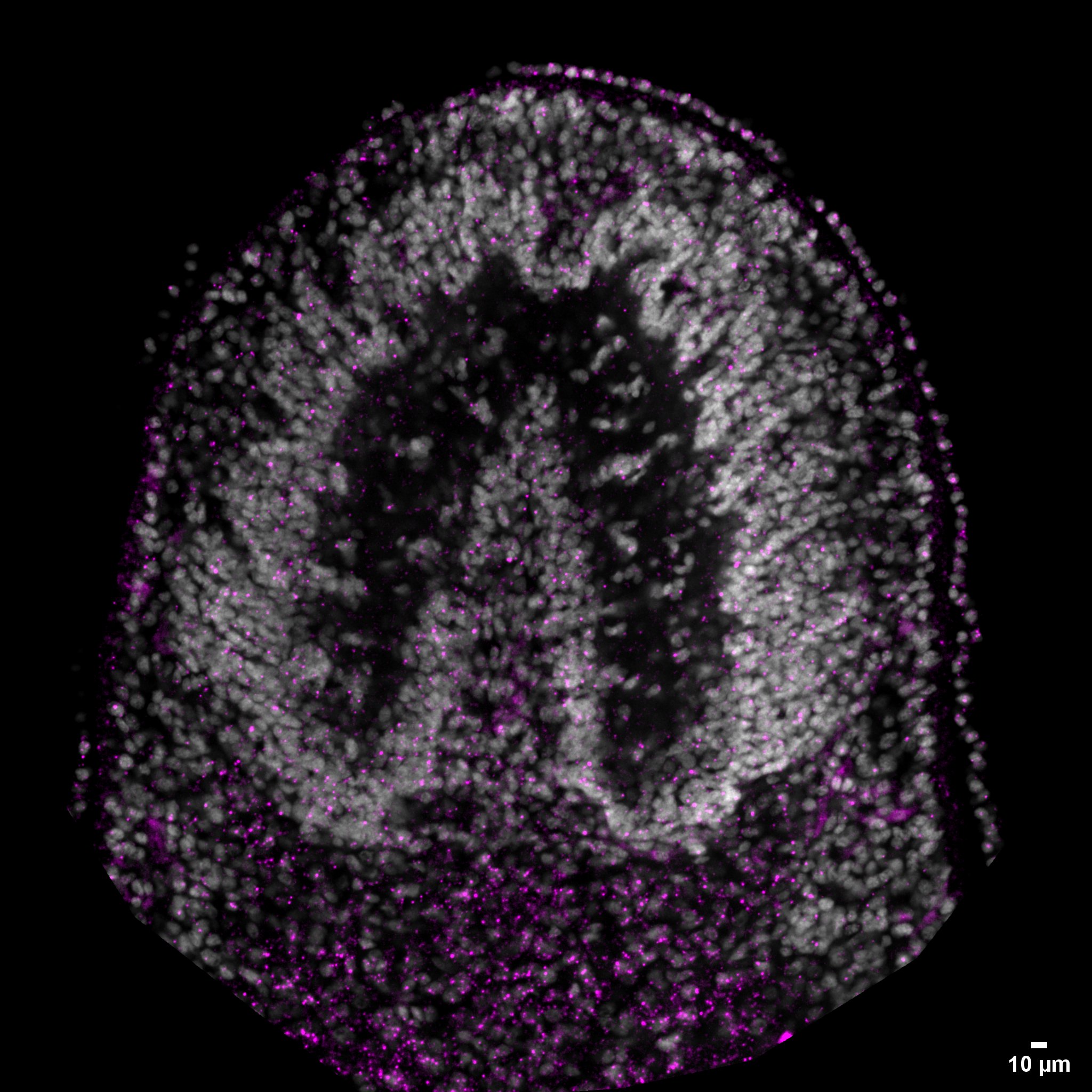

Supplement: Supplementary file 11 — Source data Fig. 4 [file 44318_2025_662_MOESM11_ESM.zip › Figure 4/4A/wildtype_ythdf-c_FITC-green_ythdf-a_Rhod-magenta_20x_Brain_Magenta_channel.jpg]

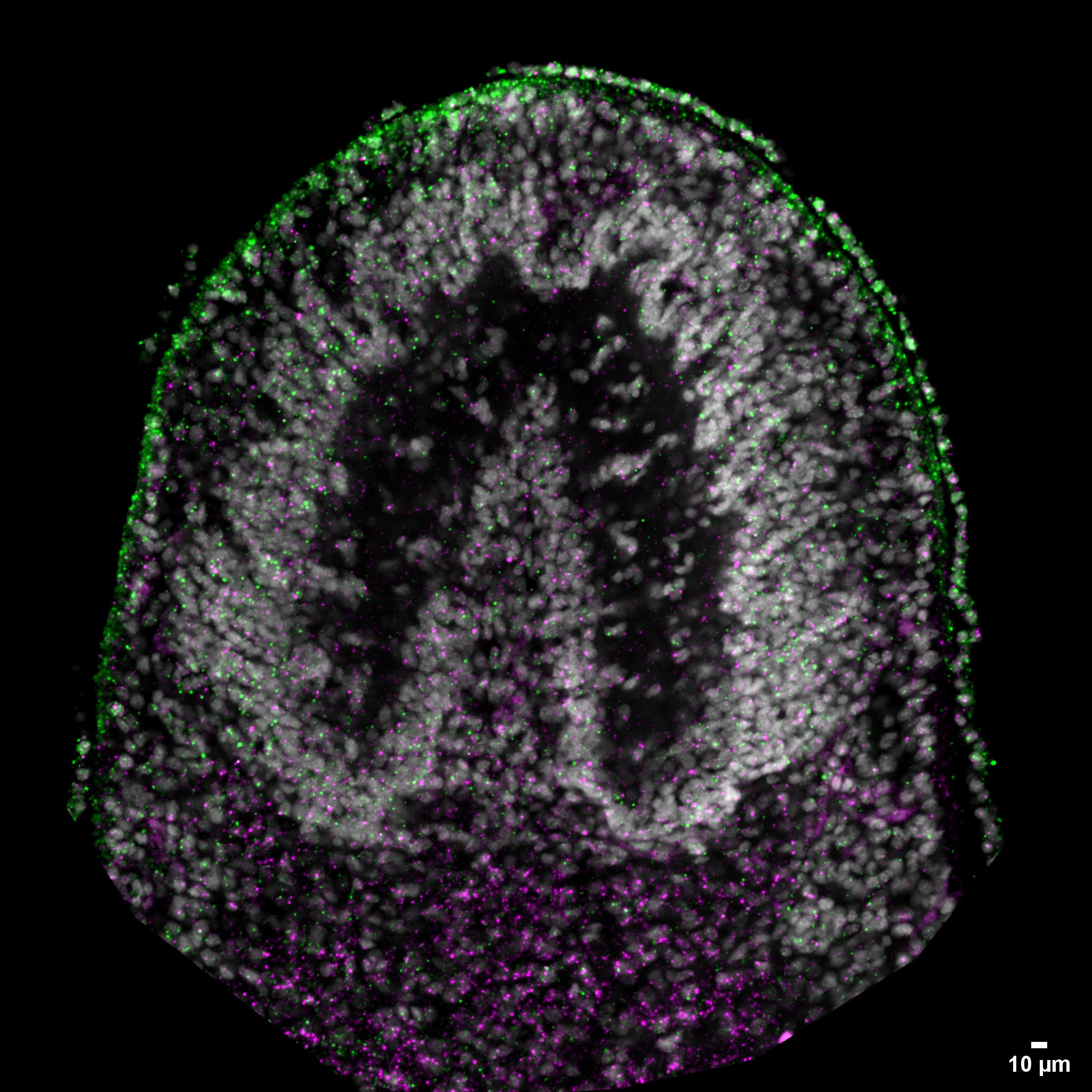

Supplement: Supplementary file 11 — Source data Fig. 4 [file 44318_2025_662_MOESM11_ESM.zip › Figure 4/4A/wildtype_ythdf-c_FITC-green_ythdf-a_Rhod-magenta_20x_Brain_Merged.jpg]

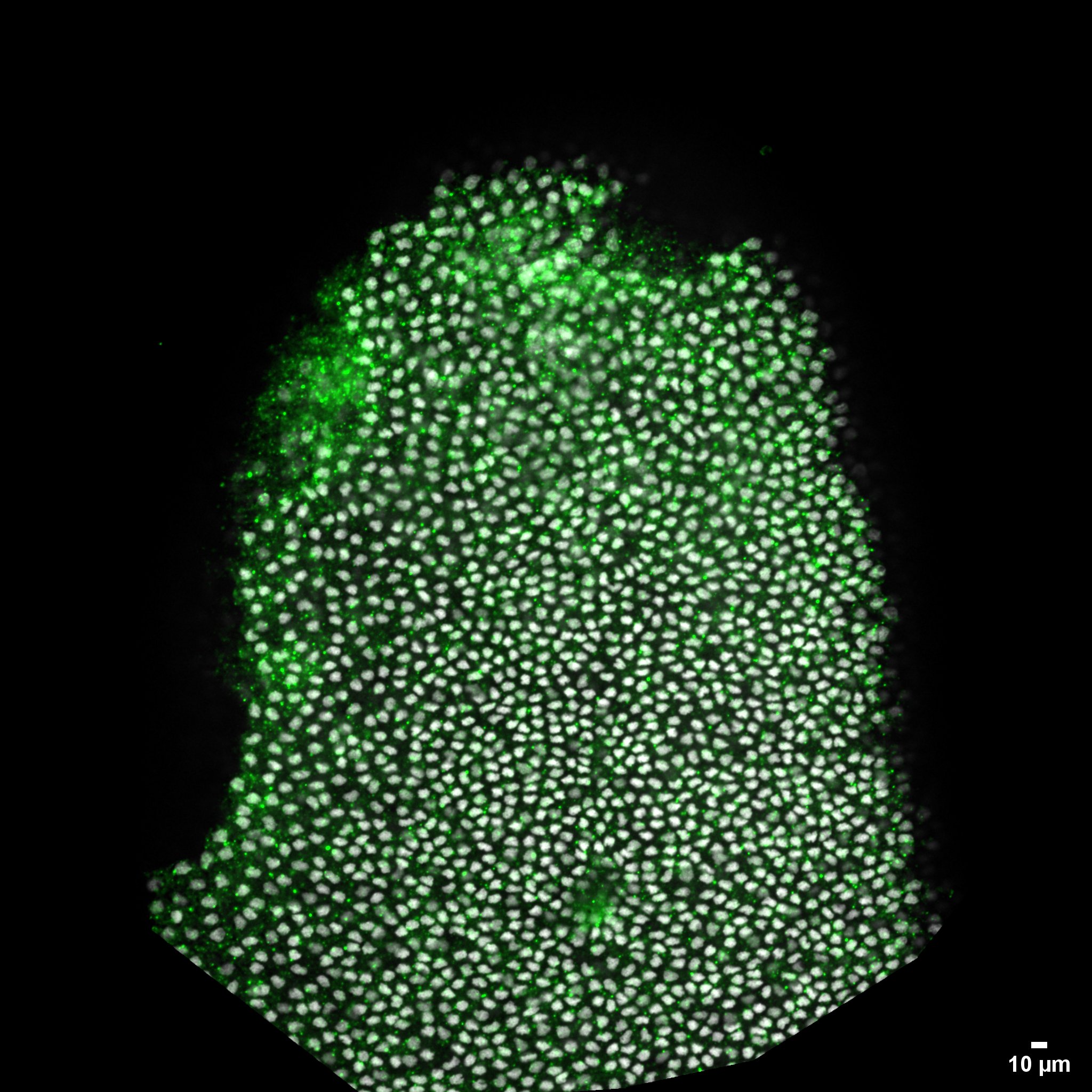

Supplement: Supplementary file 11 — Source data Fig. 4 [file 44318_2025_662_MOESM11_ESM.zip › Figure 4/4A/wildtype_ythdf-c_FITC-green_ythdf-a_Rhod-magenta_20x_Epidermis_FITC_channel.jpg]

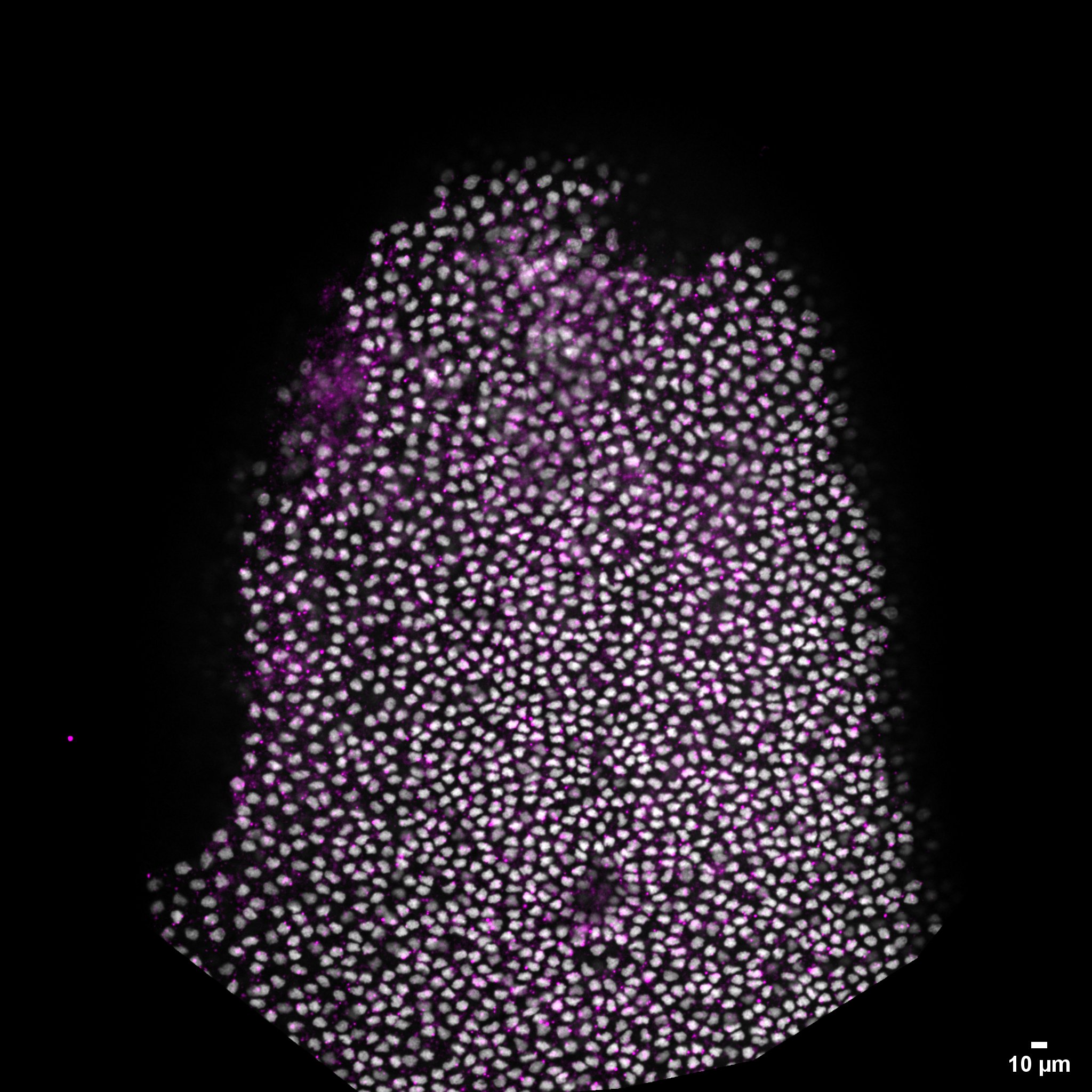

Supplement: Supplementary file 11 — Source data Fig. 4 [file 44318_2025_662_MOESM11_ESM.zip › Figure 4/4A/wildtype_ythdf-c_FITC-green_ythdf-a_Rhod-magenta_20x_Epidermis_Magenta_channel.jpg]

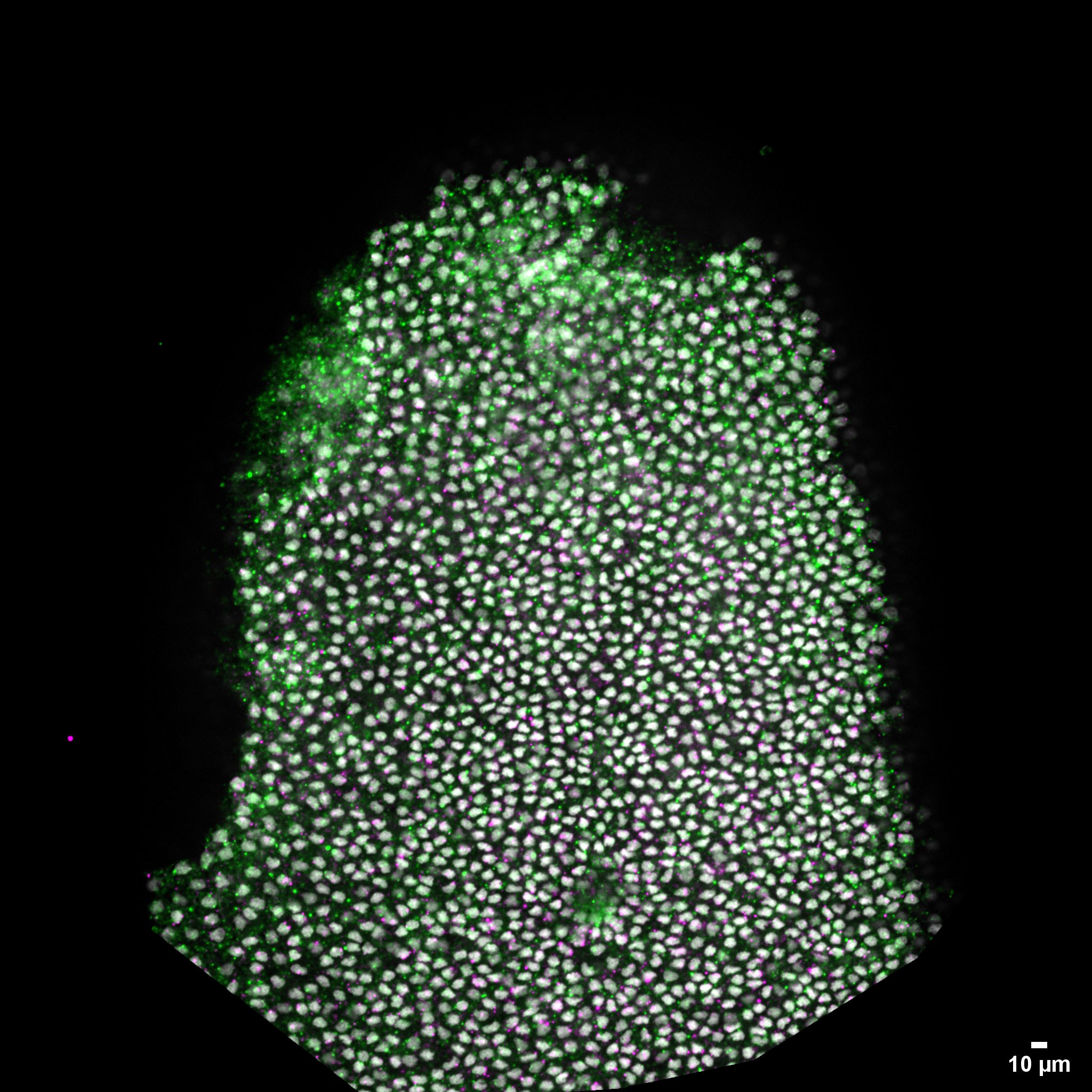

Supplement: Supplementary file 11 — Source data Fig. 4 [file 44318_2025_662_MOESM11_ESM.zip › Figure 4/4A/wildtype_ythdf-c_FITC-green_ythdf-a_Rhod-magenta_20x_Epidermis_Merged.jpg]

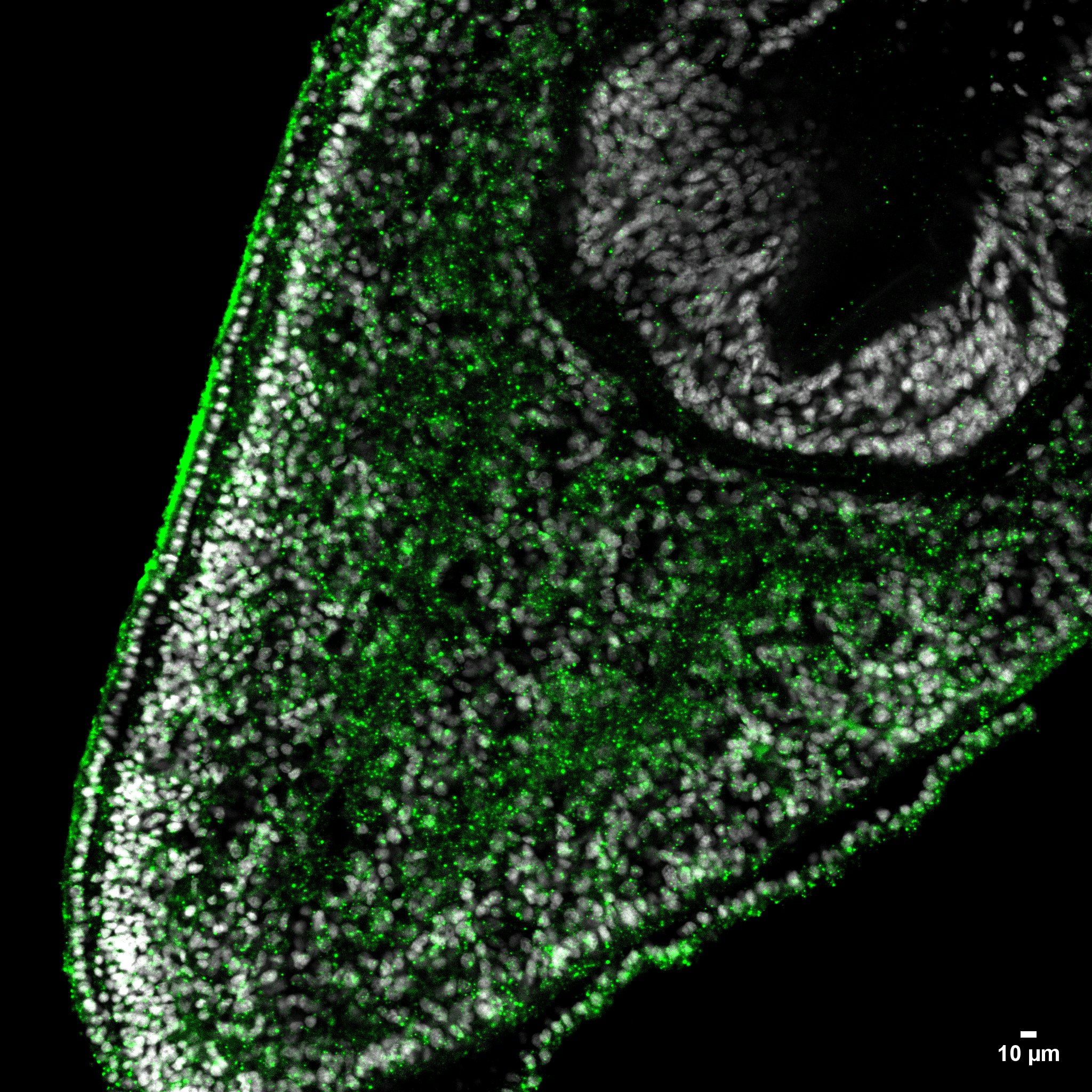

Supplement: Supplementary file 11 — Source data Fig. 4 [file 44318_2025_662_MOESM11_ESM.zip › Figure 4/4A/wildtype_ythdf-c_FITC-green_ythdf-a_Rhod-magenta_20x_Intestine_FITC_channel.jpg]

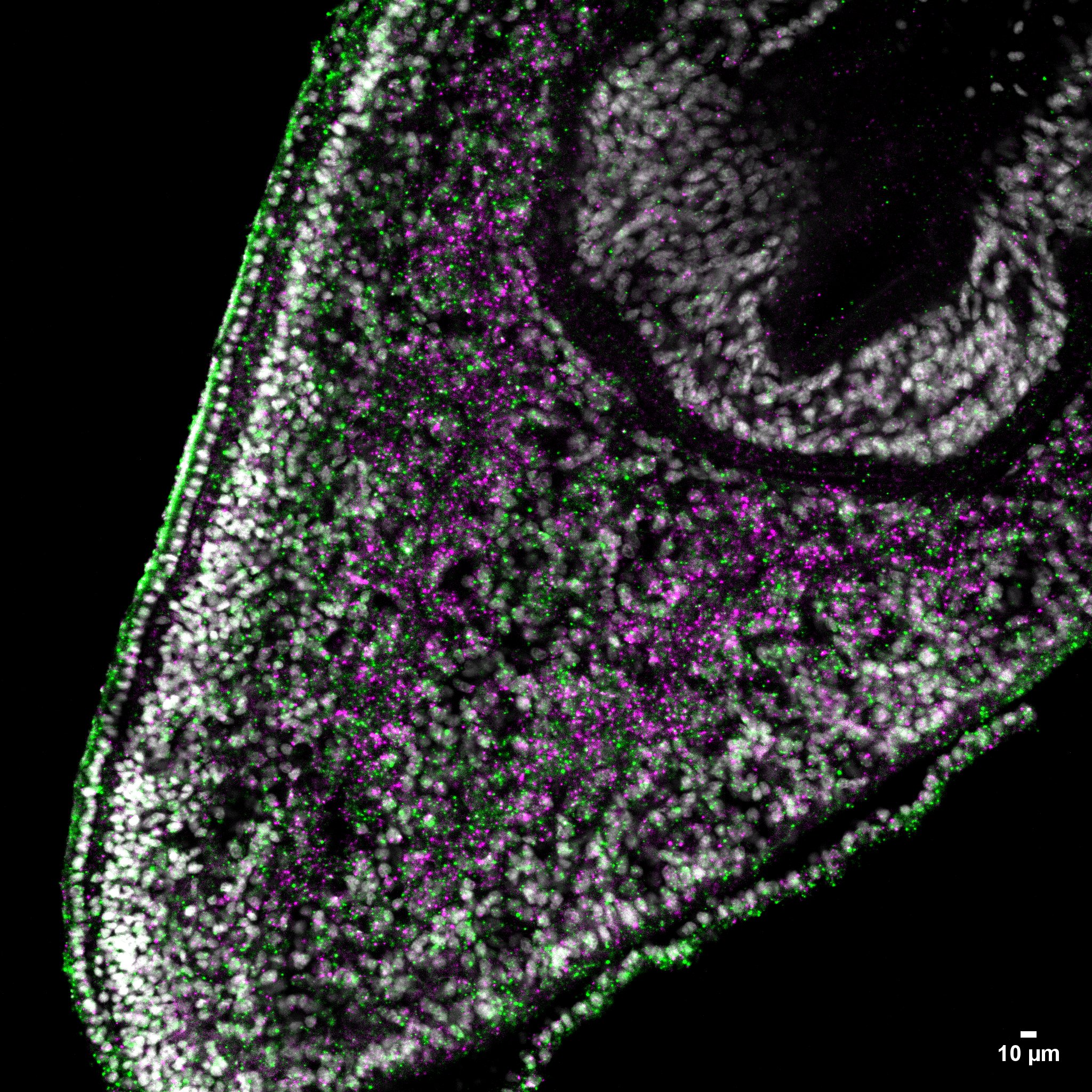

Supplement: Supplementary file 11 — Source data Fig. 4 [file 44318_2025_662_MOESM11_ESM.zip › Figure 4/4A/wildtype_ythdf-c_FITC-green_ythdf-a_Rhod-magenta_20x_Intestine_Magenta_channel.jpg]

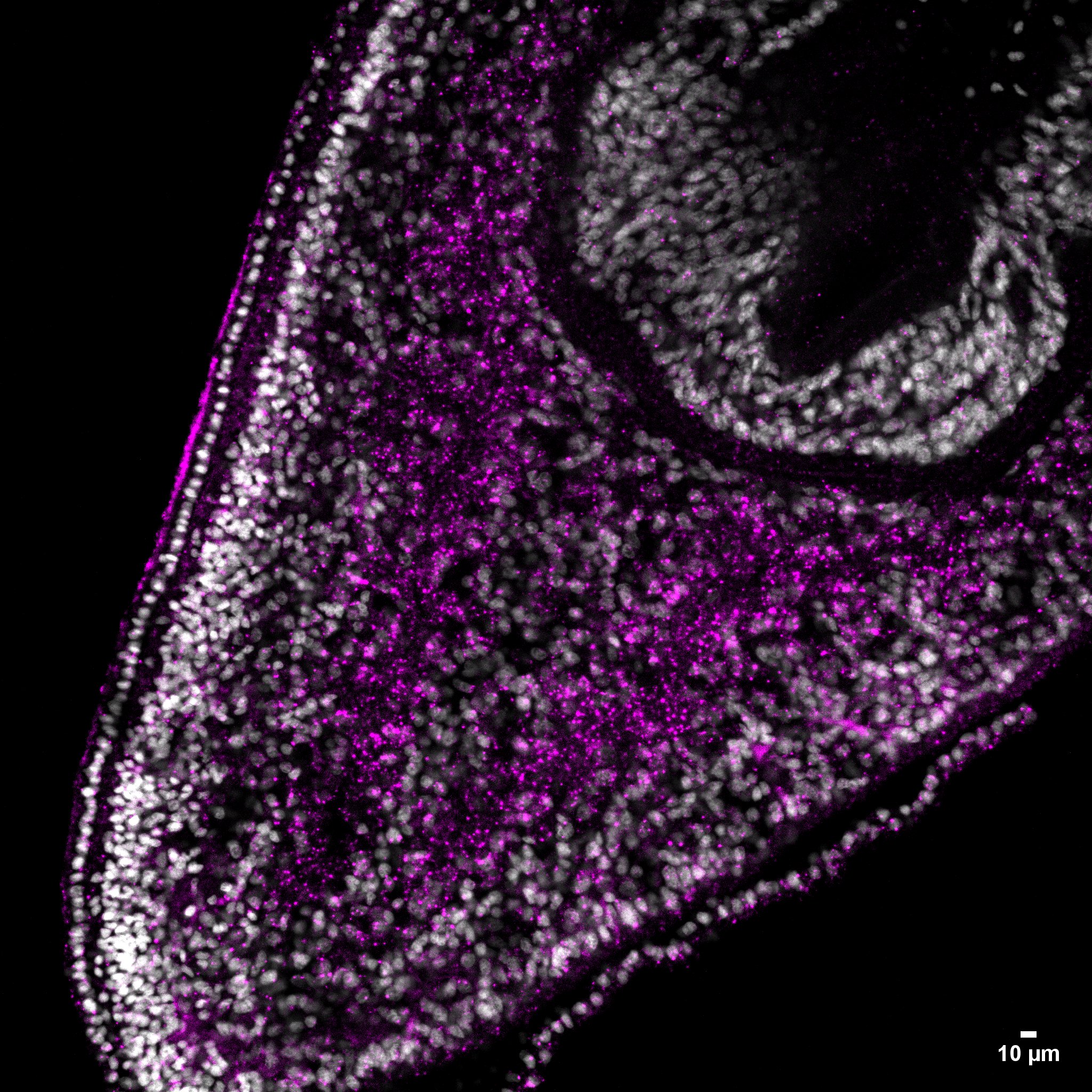

Supplement: Supplementary file 11 — Source data Fig. 4 [file 44318_2025_662_MOESM11_ESM.zip › Figure 4/4A/wildtype_ythdf-c_FITC-green_ythdf-a_Rhod-magenta_20x_Intestine_Merged.jpg]

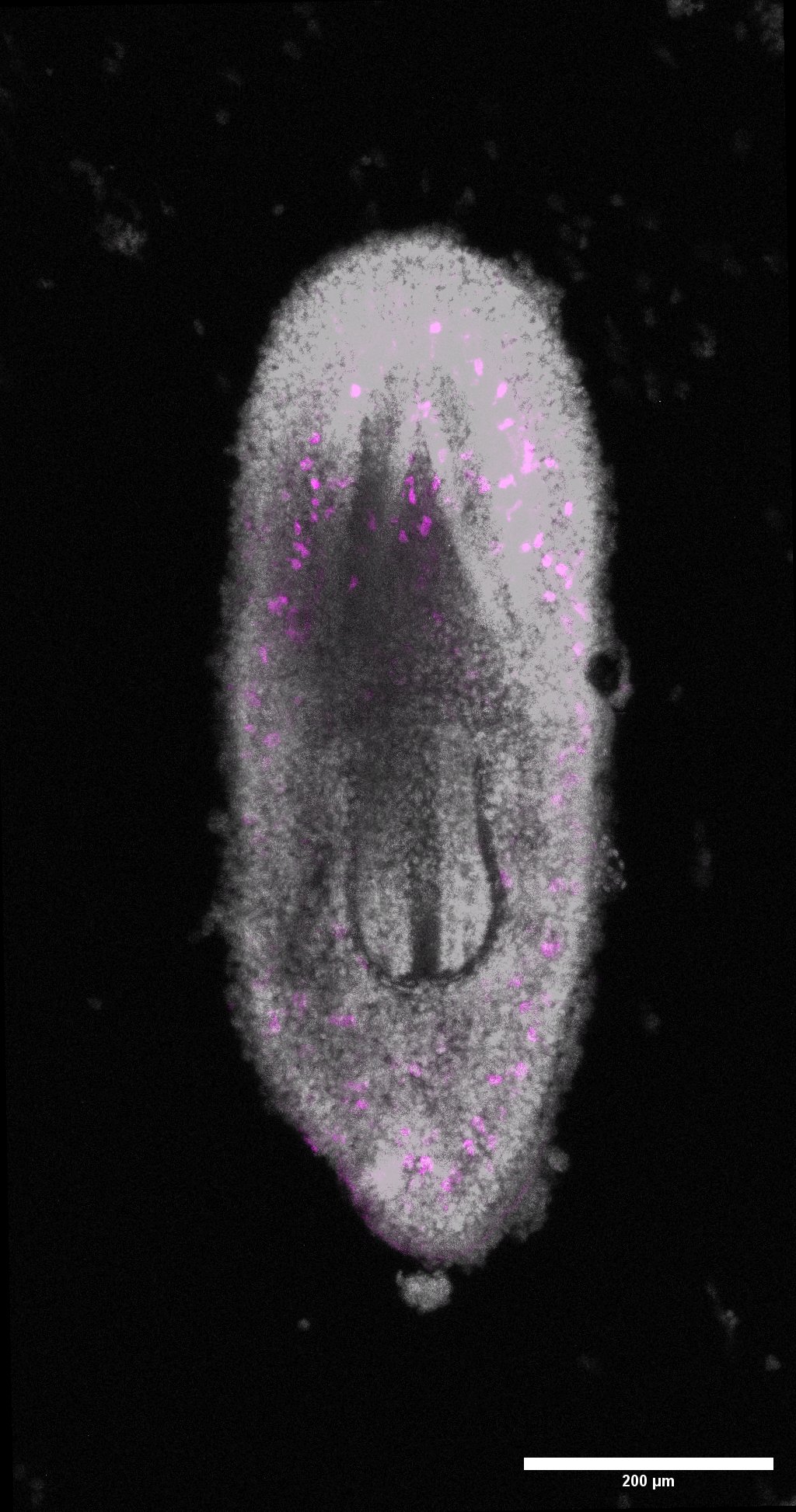

Supplement: Supplementary file 12 — Source data Fig. 5 [file 44318_2025_662_MOESM12_ESM.zip › Figure 5/5D/dd_3451/ID_10_Control_RNAi_Probe_dd3451_rhod_DAPI_10x.jpg]

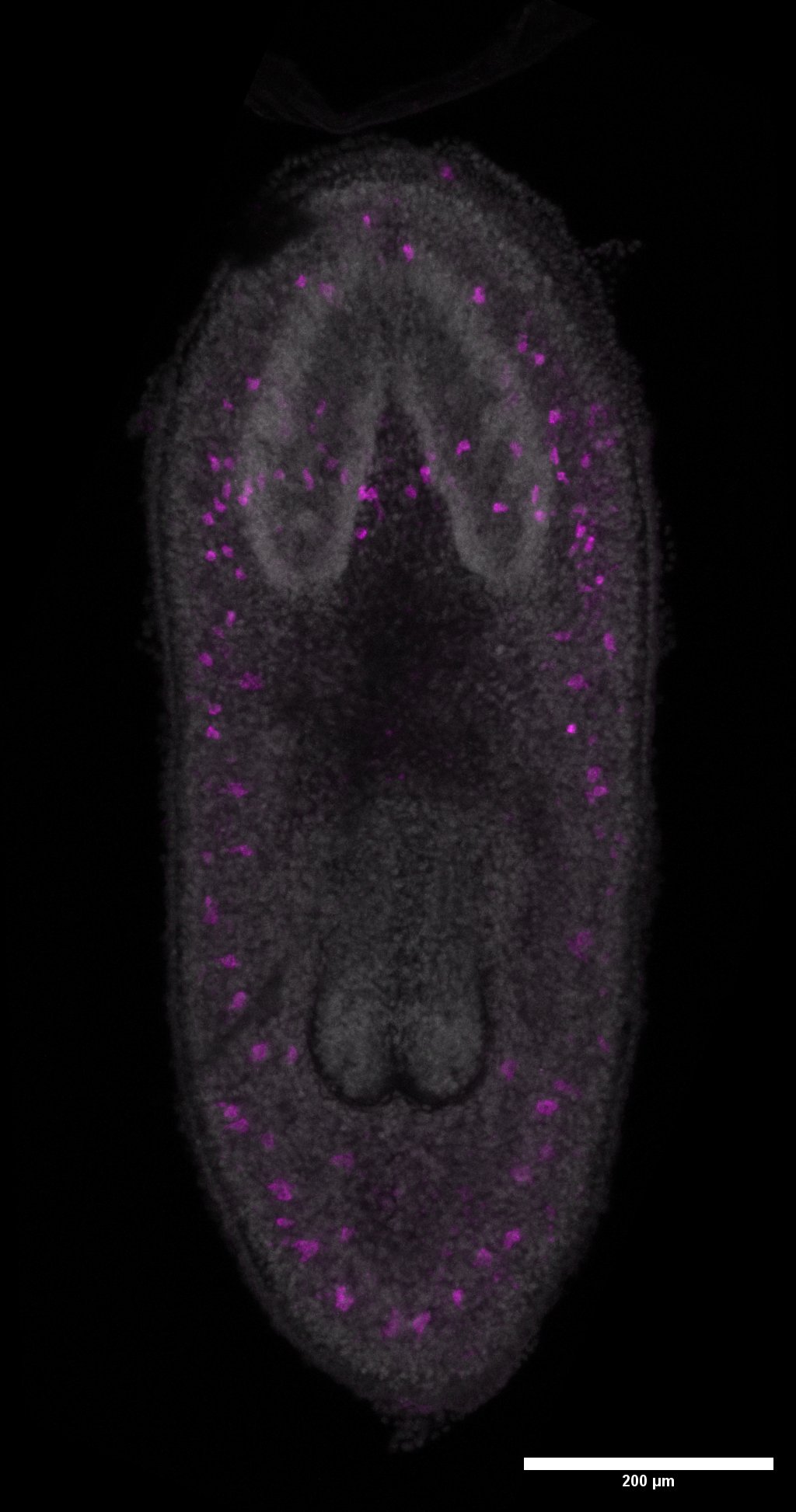

Supplement: Supplementary file 12 — Source data Fig. 5 [file 44318_2025_662_MOESM12_ESM.zip › Figure 5/5D/dd_3451/ID_10_Triple_RNAi_Probe_dd3451_rhod_DAPI_10x.jpg]

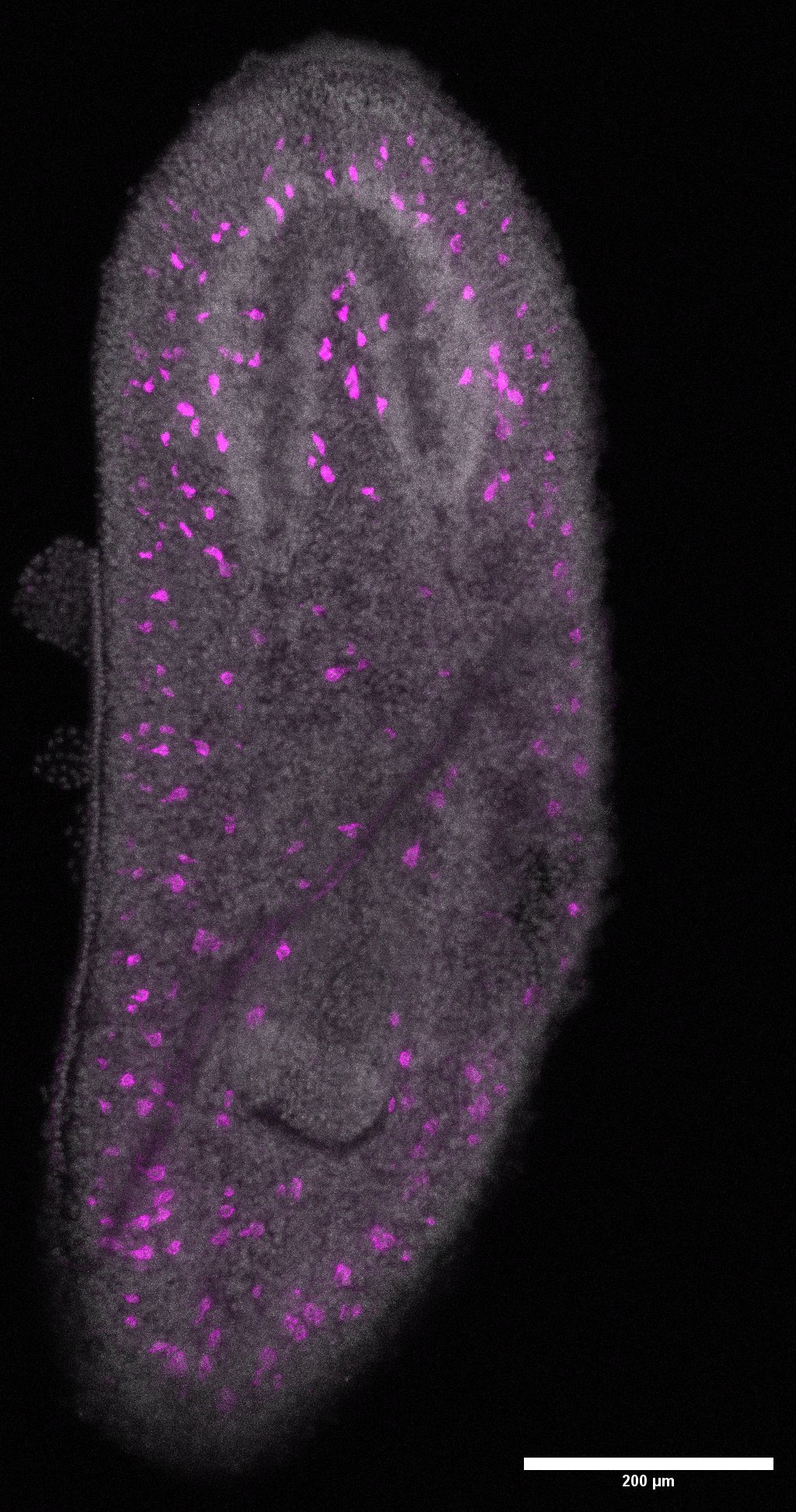

Supplement: Supplementary file 12 — Source data Fig. 5 [file 44318_2025_662_MOESM12_ESM.zip › Figure 5/5D/dd_3451/ID_10_ythdf-B_RNAi_Probe_dd3451_rhod_DAPI_10x.jpg]

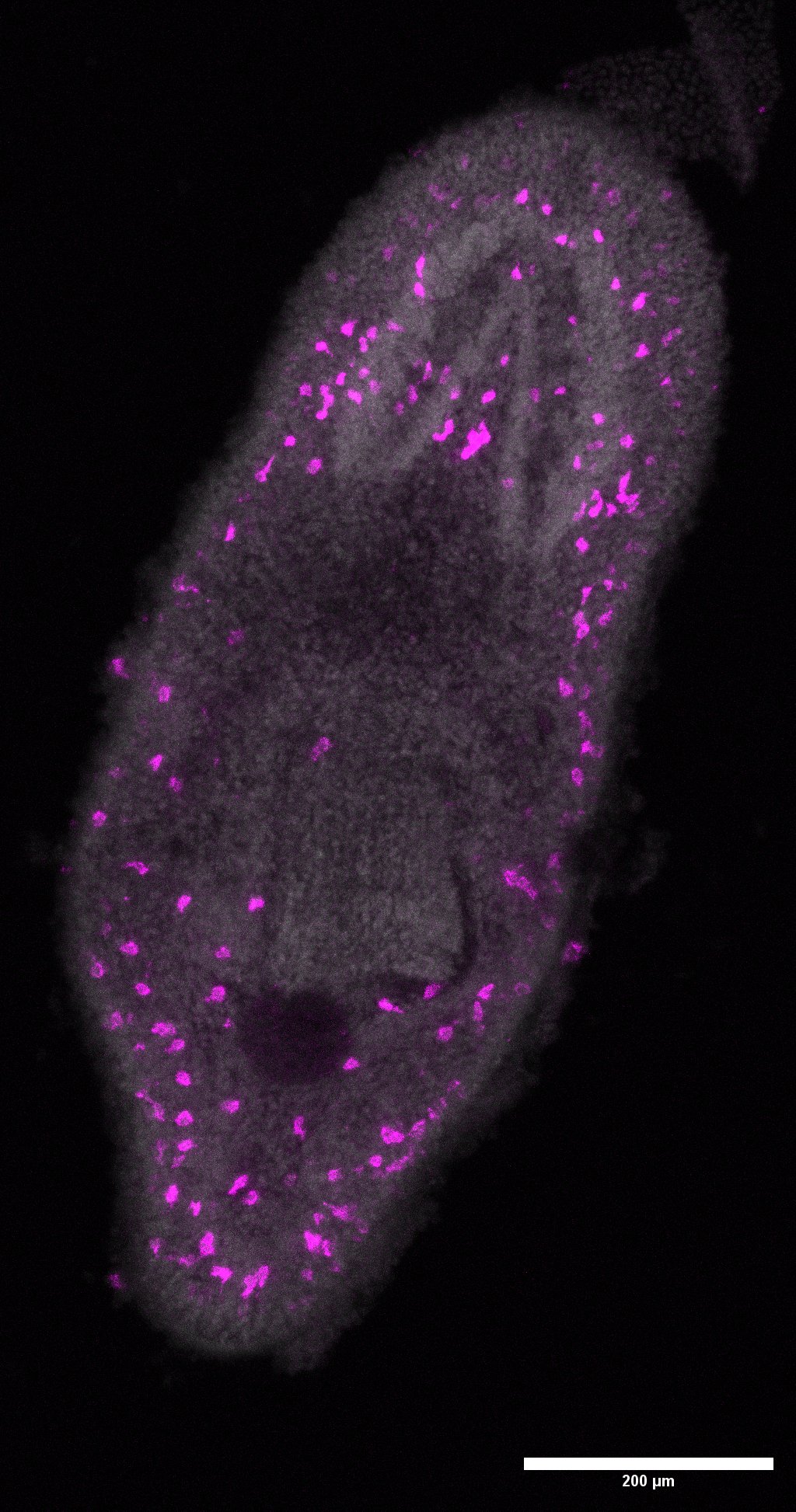

Supplement: Supplementary file 12 — Source data Fig. 5 [file 44318_2025_662_MOESM12_ESM.zip › Figure 5/5D/dd_3451/ID_10_ythdf-C_RNAi_Probe_dd3451_rhod_DAPI_10x.jpg]

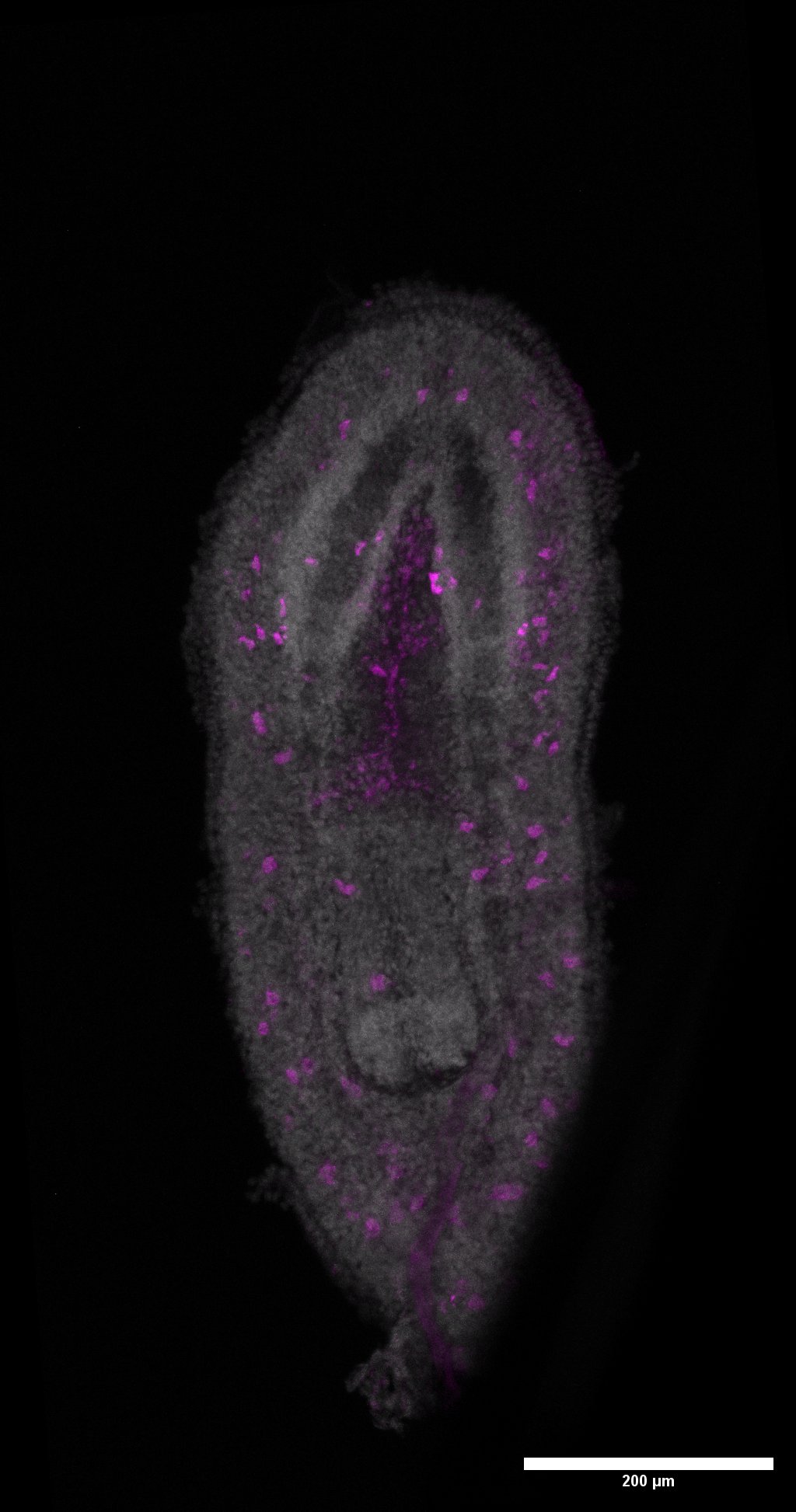

Supplement: Supplementary file 12 — Source data Fig. 5 [file 44318_2025_662_MOESM12_ESM.zip › Figure 5/5D/dd_3451/ID_11_Control_RNAi_Probe_dd3451_rhod_DAPI_10x.jpg]

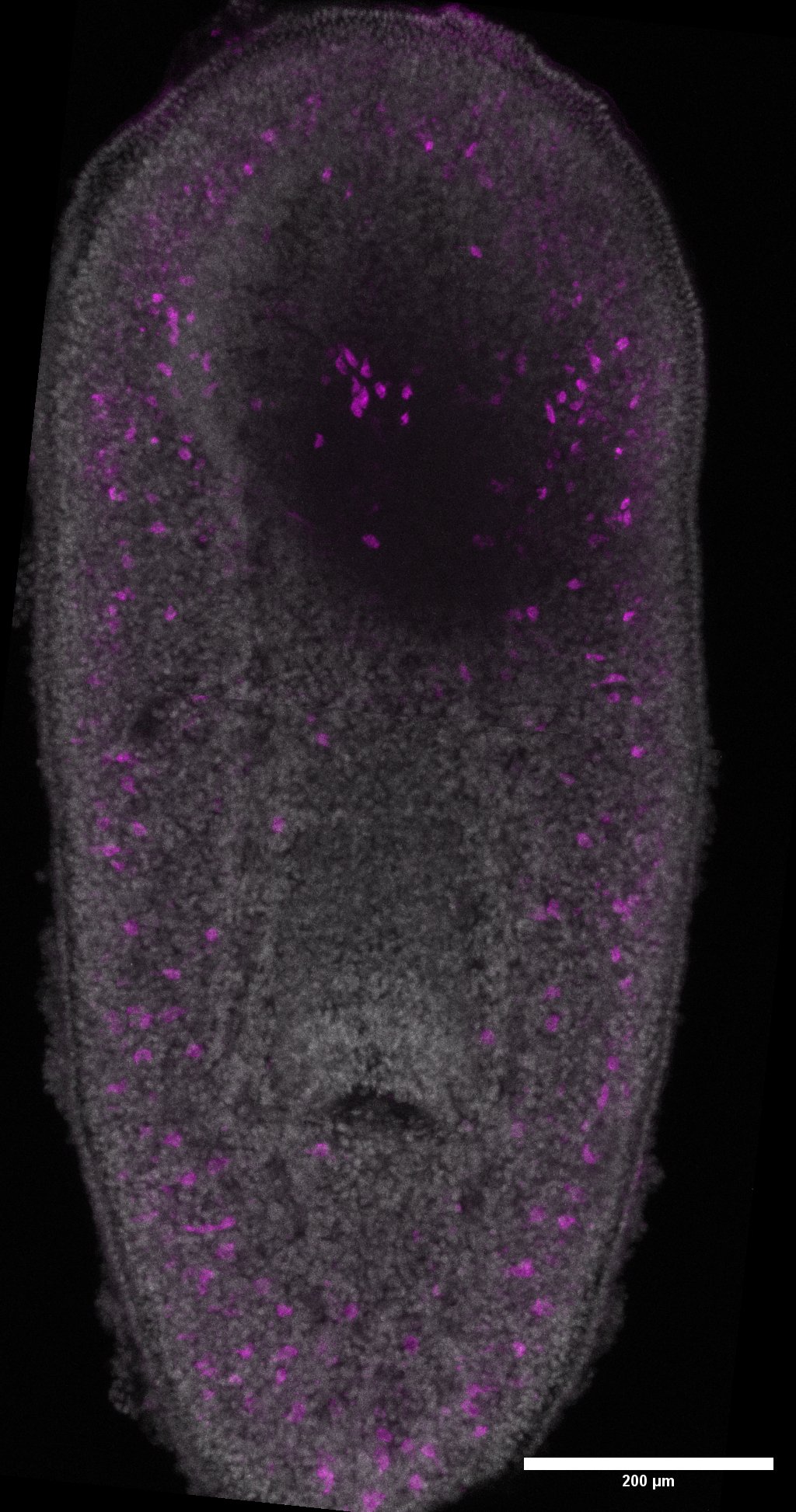

Supplement: Supplementary file 12 — Source data Fig. 5 [file 44318_2025_662_MOESM12_ESM.zip › Figure 5/5D/dd_3451/ID_11_Triple_RNAi_Probe_dd3451_rhod_DAPI_10x.jpg]

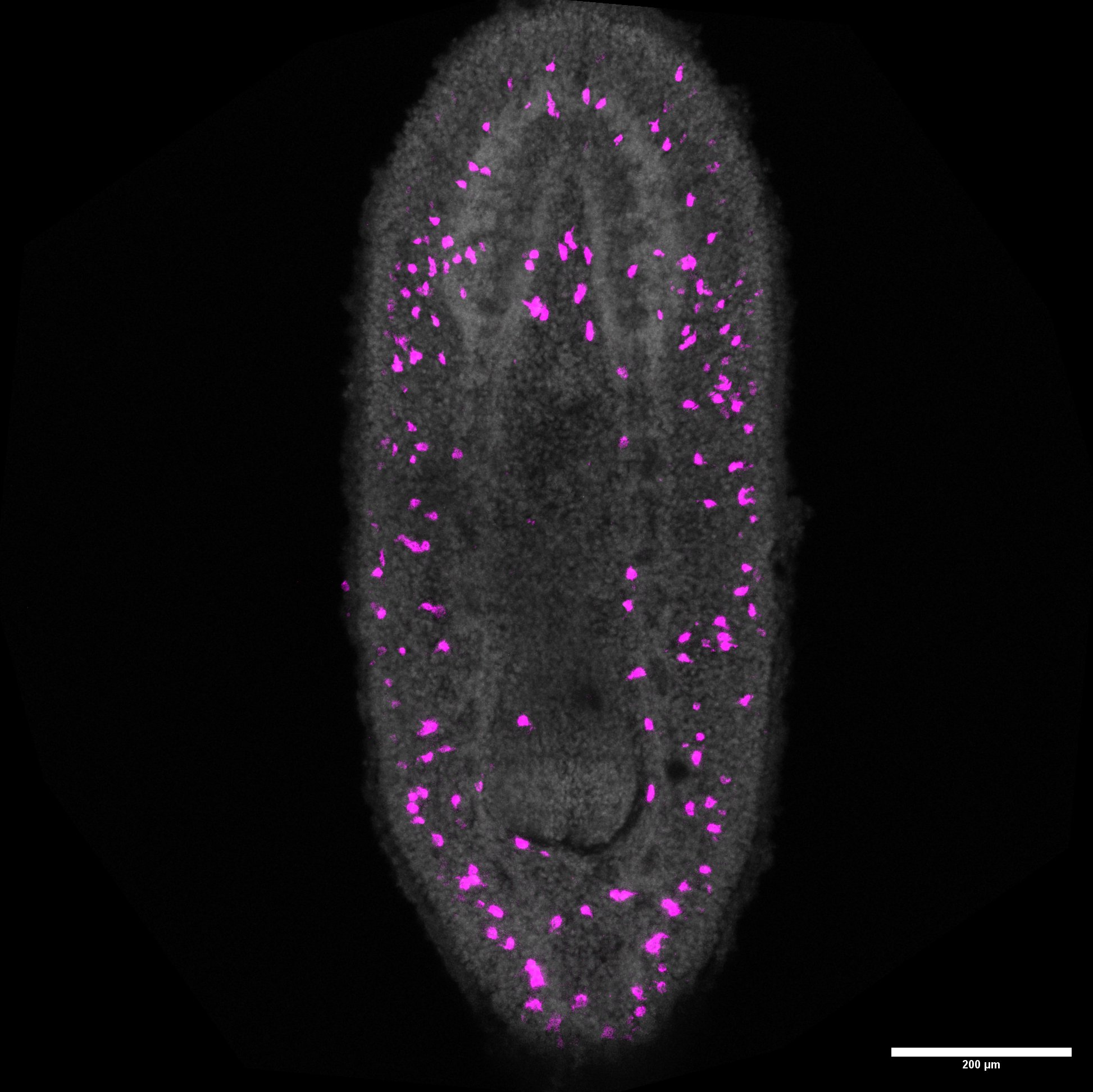

Supplement: Supplementary file 12 — Source data Fig. 5 [file 44318_2025_662_MOESM12_ESM.zip › Figure 5/5D/dd_3451/ID_11_ythdf-C_RNAi_Probe_dd3451_rhod_DAPI_10x.jpg]

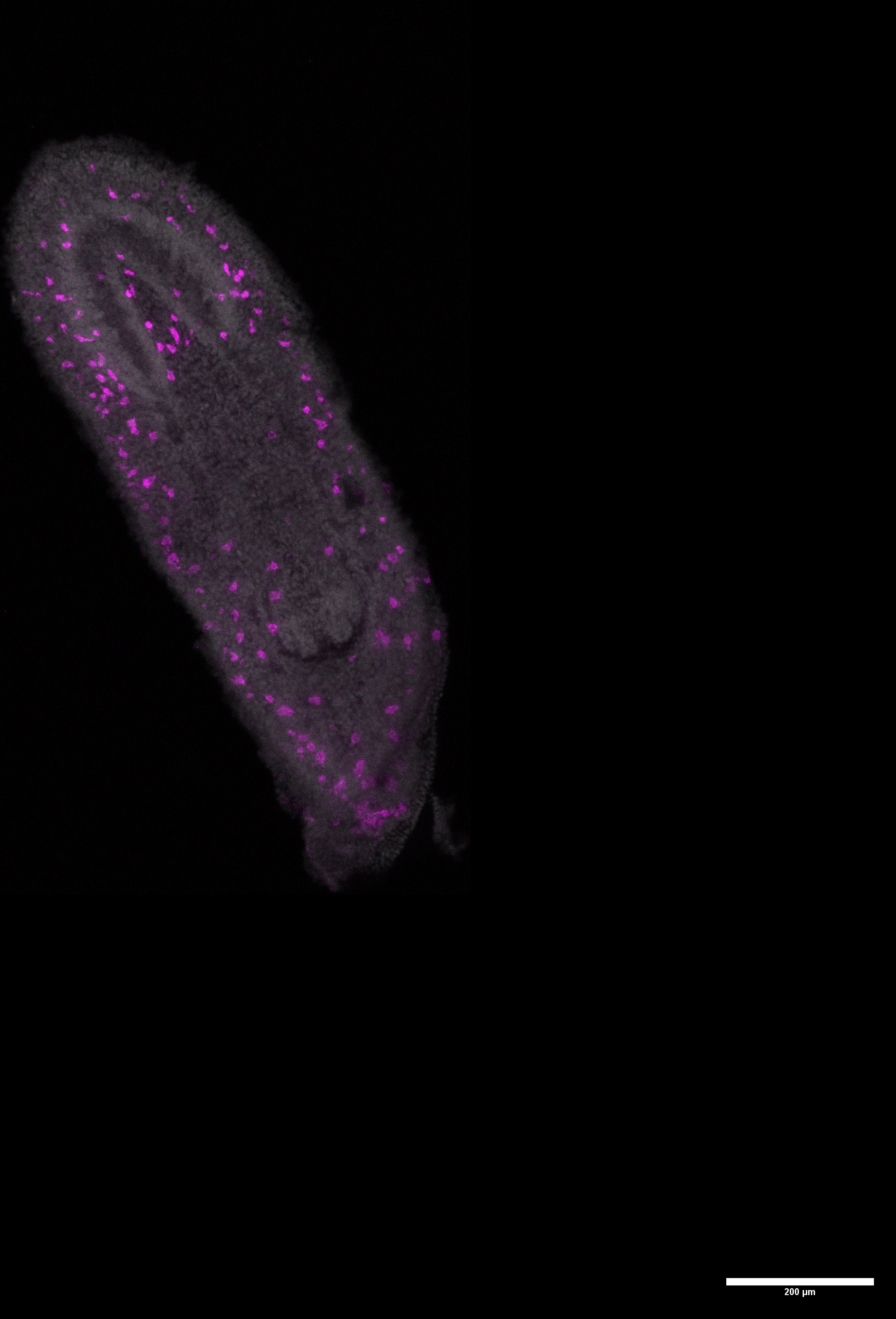

Supplement: Supplementary file 12 — Source data Fig. 5 [file 44318_2025_662_MOESM12_ESM.zip › Figure 5/5D/dd_3451/ID_12_ythdf-C_RNAi_Probe_dd3451_rhod_DAPI_10x.jpg]

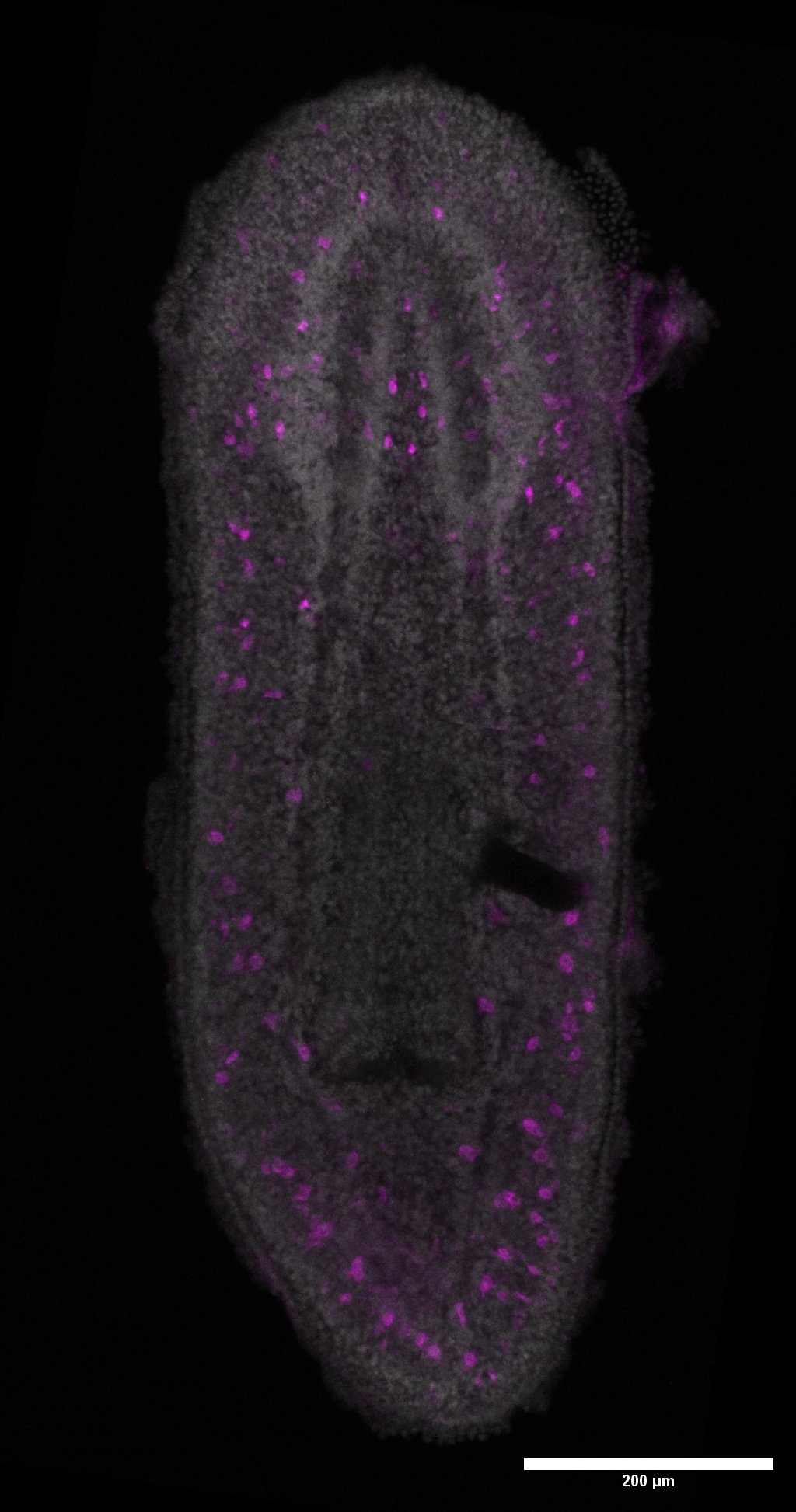

Supplement: Supplementary file 12 — Source data Fig. 5 [file 44318_2025_662_MOESM12_ESM.zip › Figure 5/5D/dd_3451/ID_13_Triple_RNAi_Probe_dd3451_rhod_DAPI_10x.jpg]

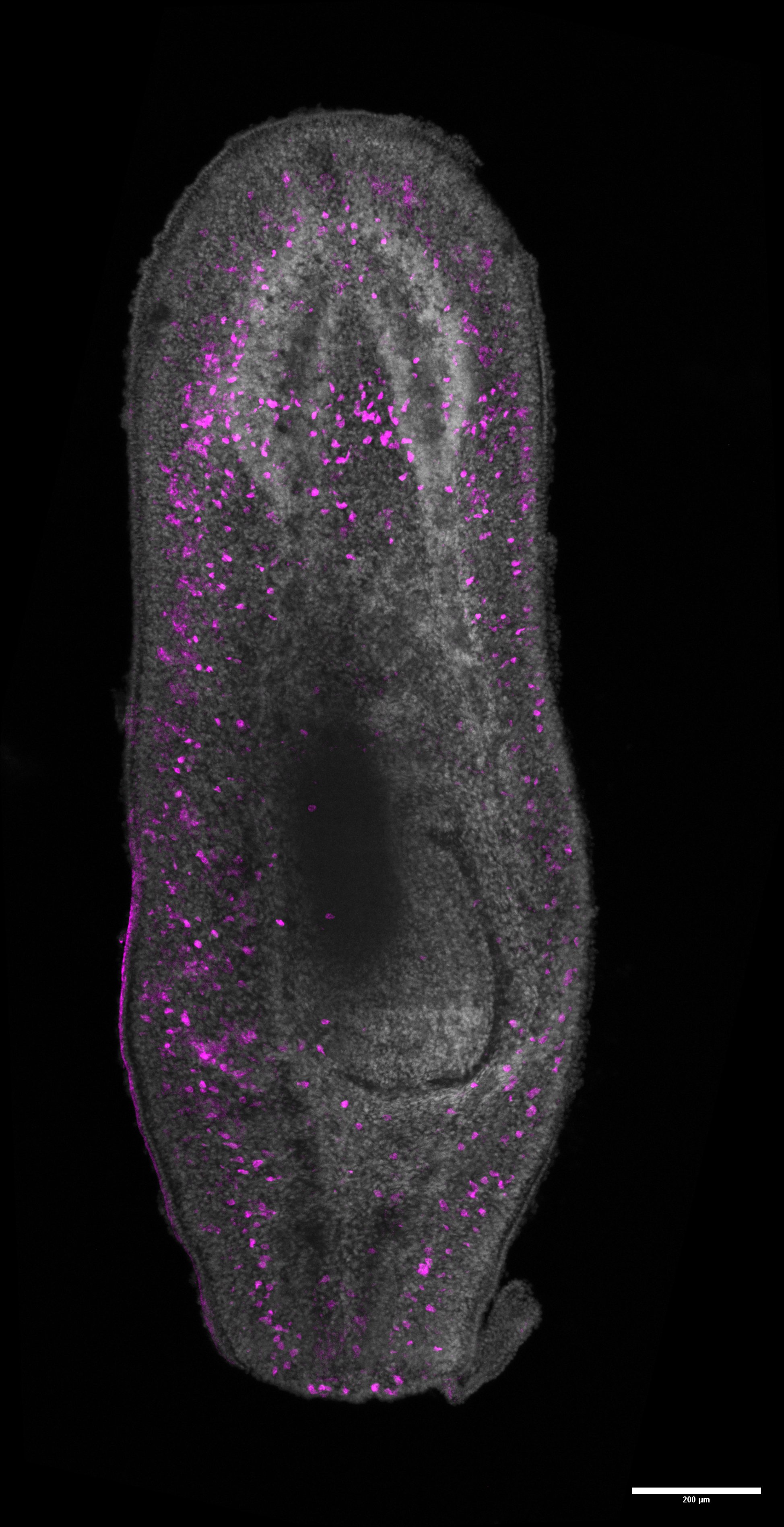

Supplement: Supplementary file 12 — Source data Fig. 5 [file 44318_2025_662_MOESM12_ESM.zip › Figure 5/5D/dd_3451/ID_1_Control_RNAi_Probe_dd3451_rhod_DAPI_10x.jpg]

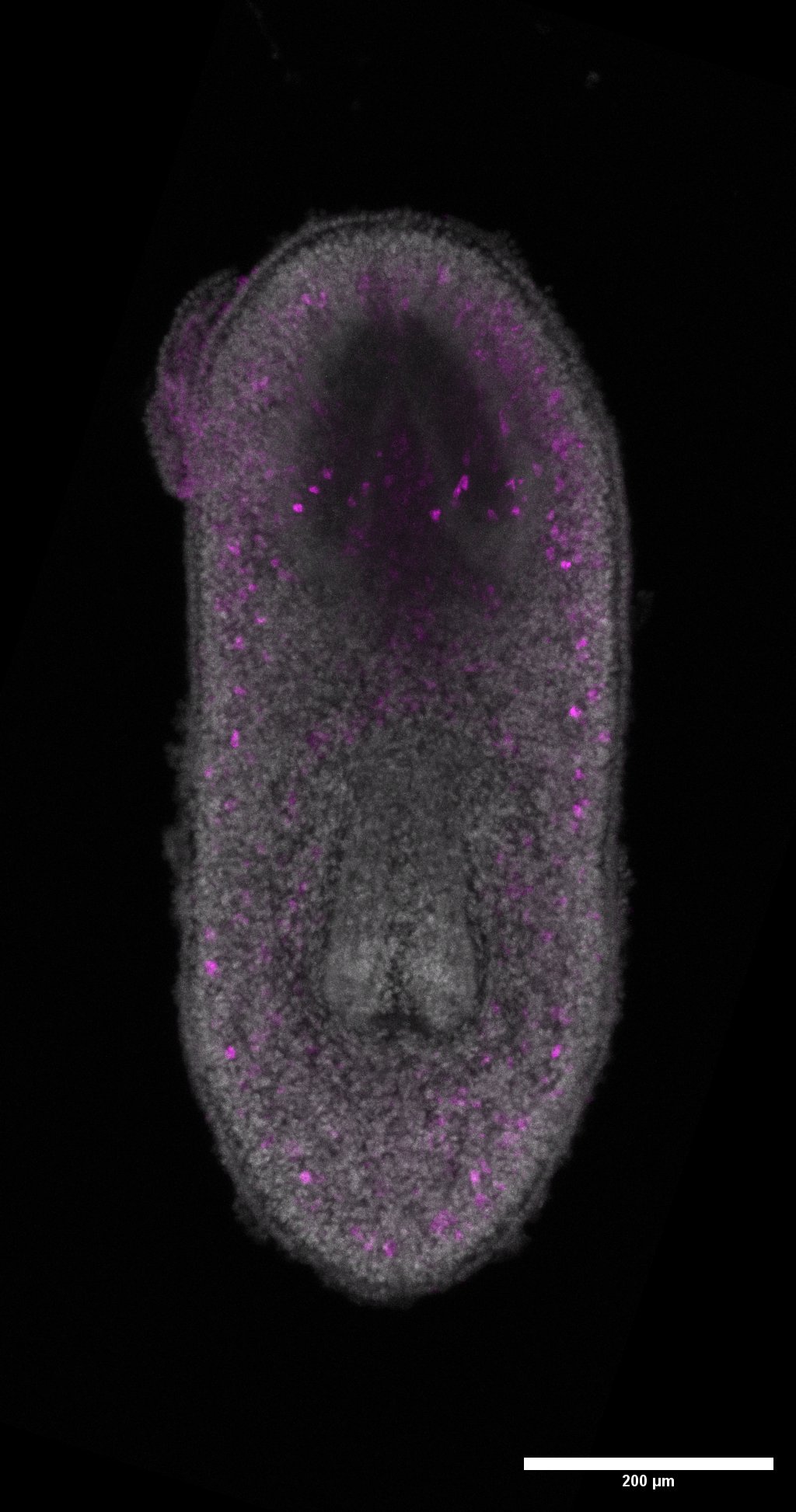

Supplement: Supplementary file 12 — Source data Fig. 5 [file 44318_2025_662_MOESM12_ESM.zip › Figure 5/5D/dd_3451/ID_1_Triple_RNAi_Probe_dd3451_rhod_DAPI_10x.jpg]

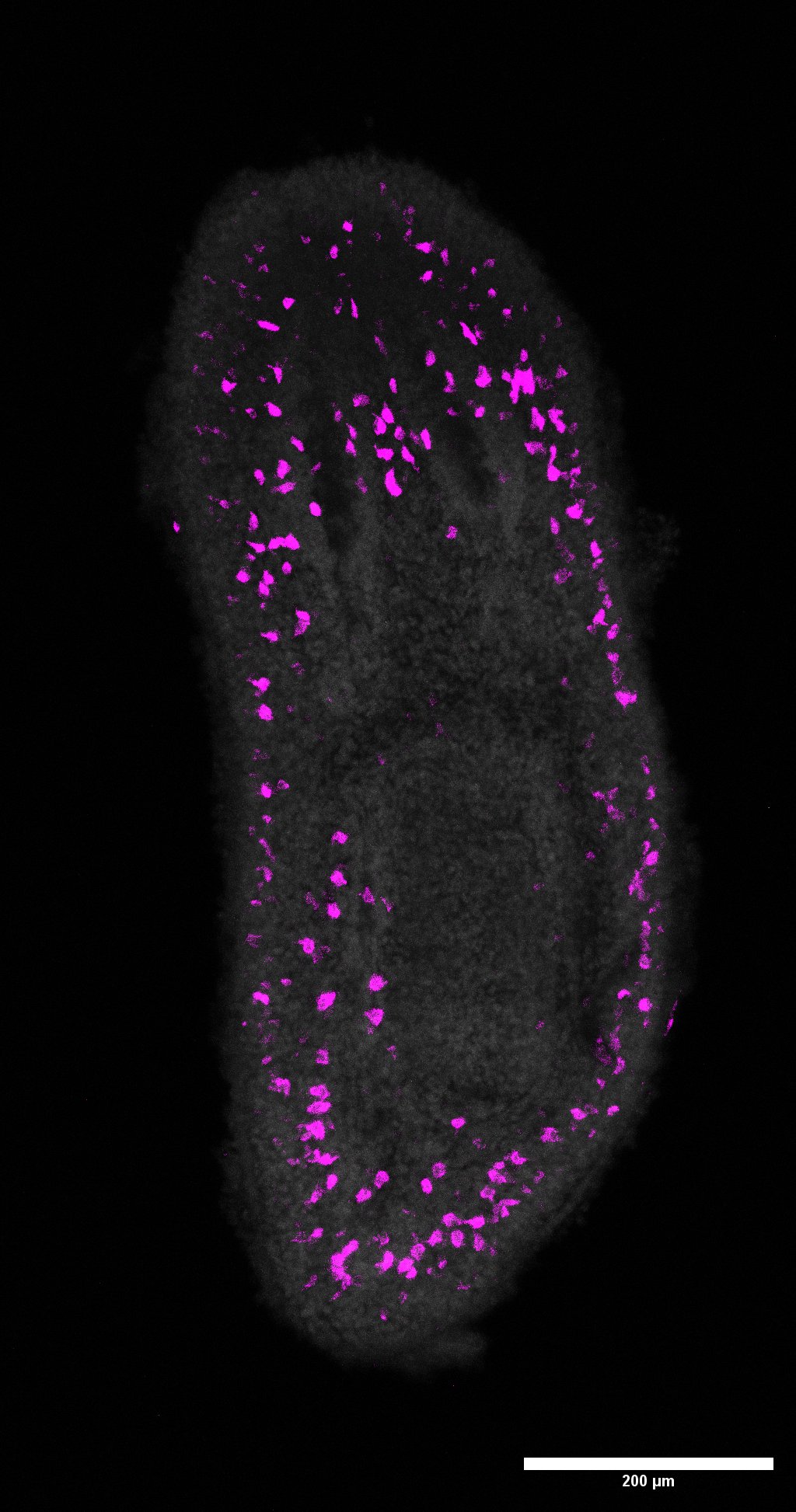

Supplement: Supplementary file 12 — Source data Fig. 5 [file 44318_2025_662_MOESM12_ESM.zip › Figure 5/5D/dd_3451/ID_1_ythdf-A_RNAi_Probe_dd3451_rhod_DAPI_10x.jpg]

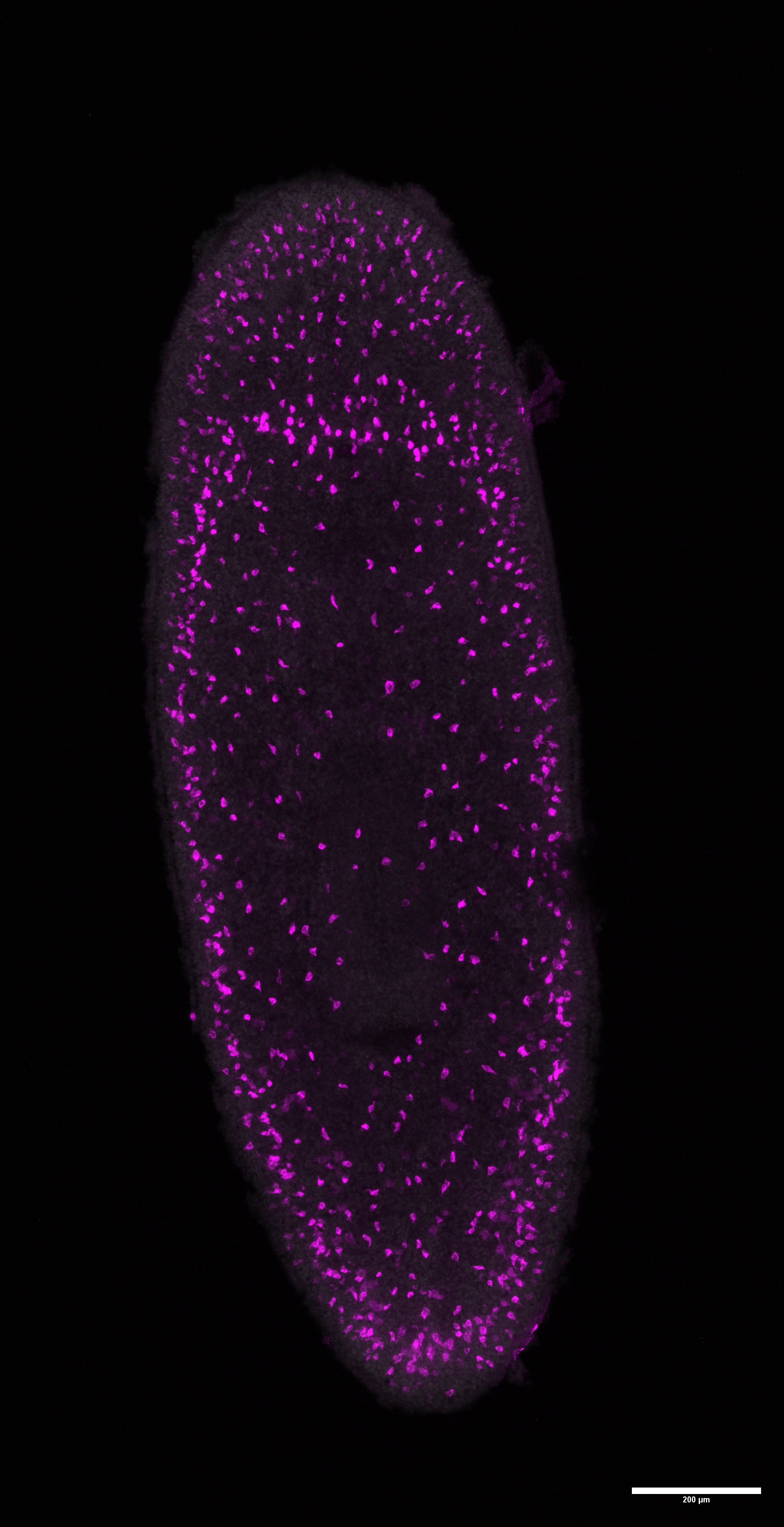

Supplement: Supplementary file 12 — Source data Fig. 5 [file 44318_2025_662_MOESM12_ESM.zip › Figure 5/5D/dd_3451/ID_1_ythdf-B_RNAi_Probe_dd3451_rhod_DAPI_10x.jpg]

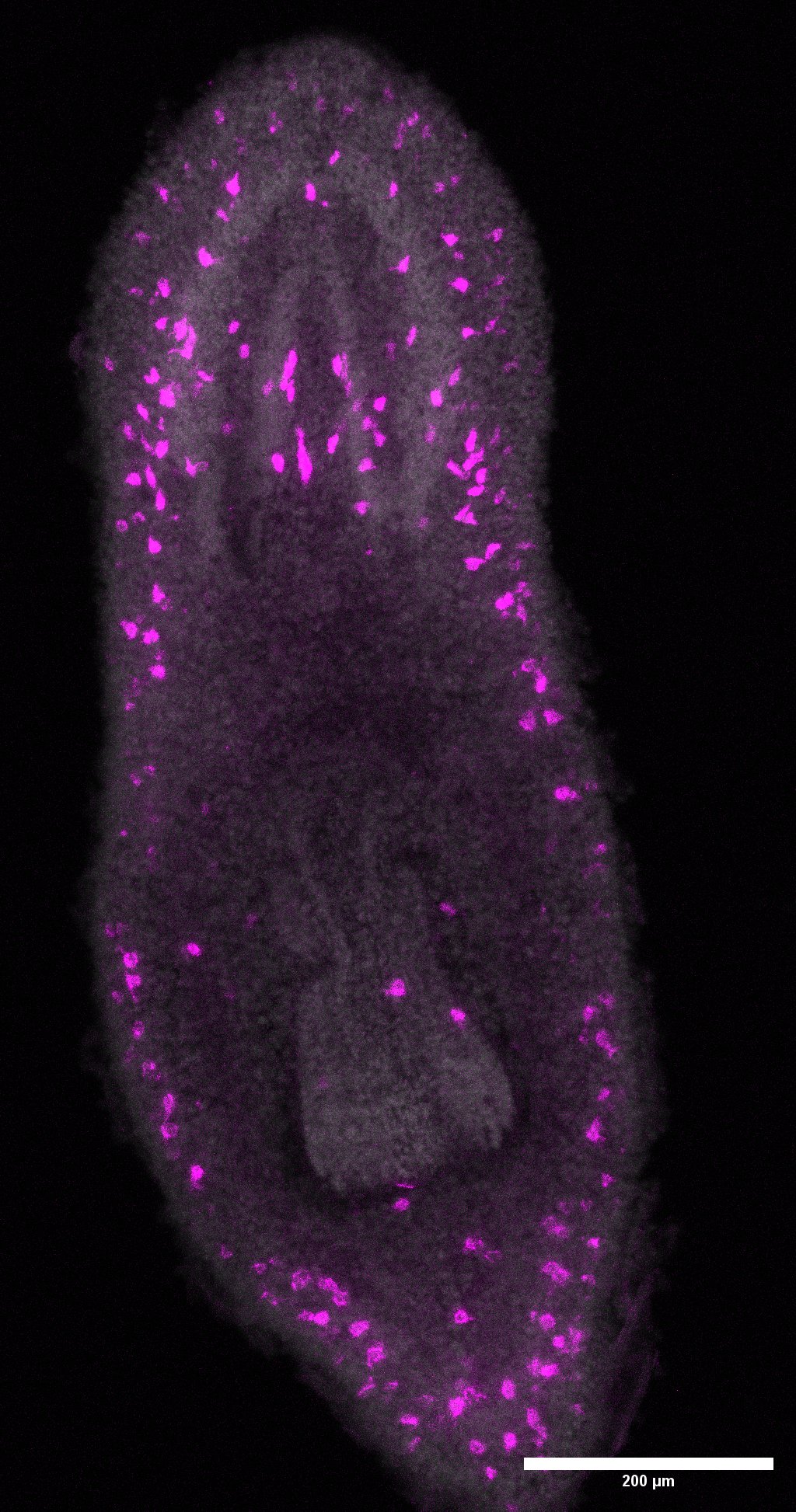

Supplement: Supplementary file 12 — Source data Fig. 5 [file 44318_2025_662_MOESM12_ESM.zip › Figure 5/5D/dd_3451/ID_1_ythdf-C_RNAi_Probe_dd3451_rhod_DAPI_10x.jpg]

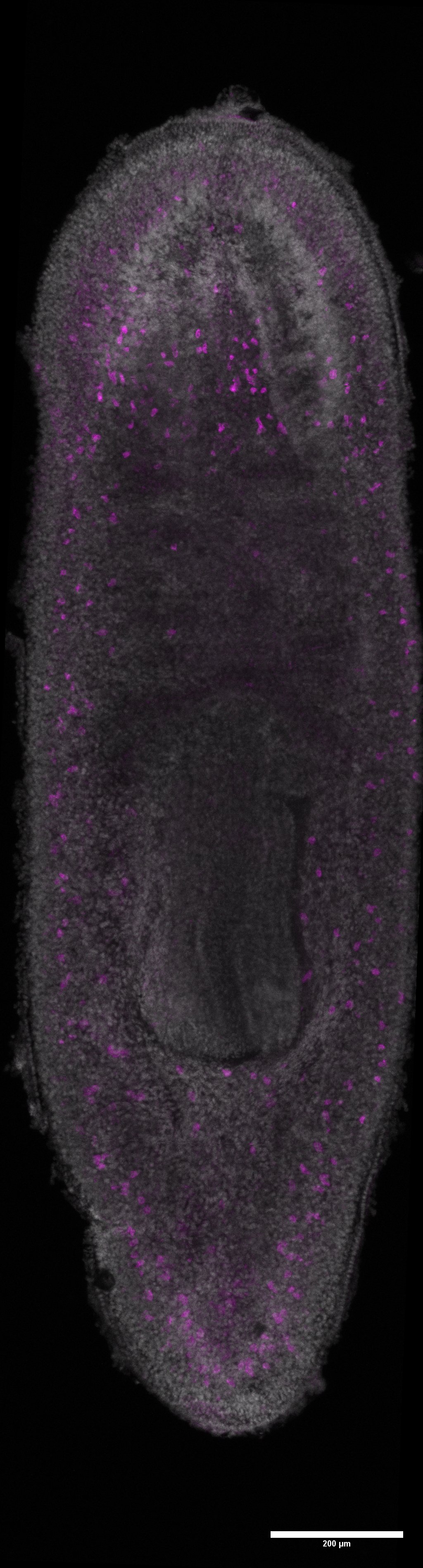

Supplement: Supplementary file 12 — Source data Fig. 5 [file 44318_2025_662_MOESM12_ESM.zip › Figure 5/5D/dd_3451/ID_2_Control_RNAi_Probe_dd3451_rhod_DAPI_10x.jpg]

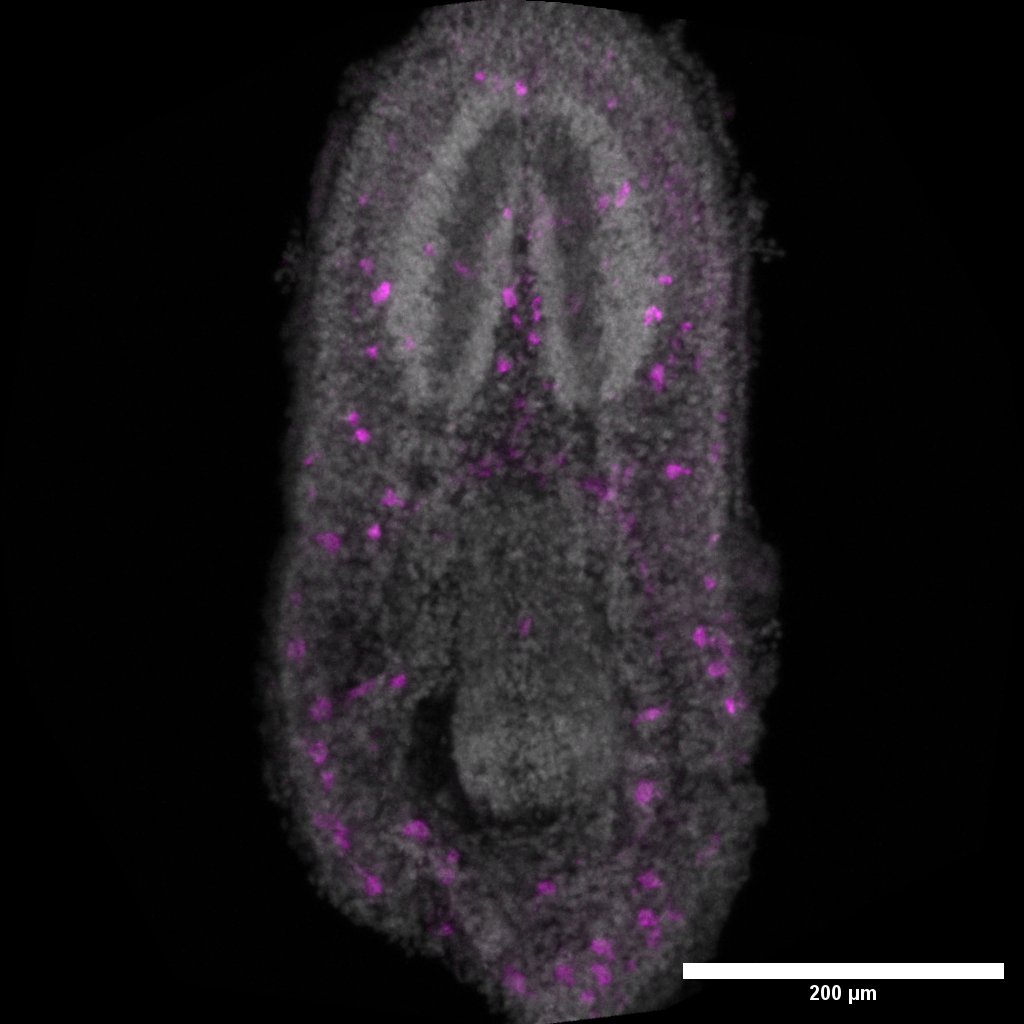

Supplement: Supplementary file 12 — Source data Fig. 5 [file 44318_2025_662_MOESM12_ESM.zip › Figure 5/5D/dd_3451/ID_2_Triple_RNAi_Probe_dd3451_rhod_DAPI_10x.jpg]

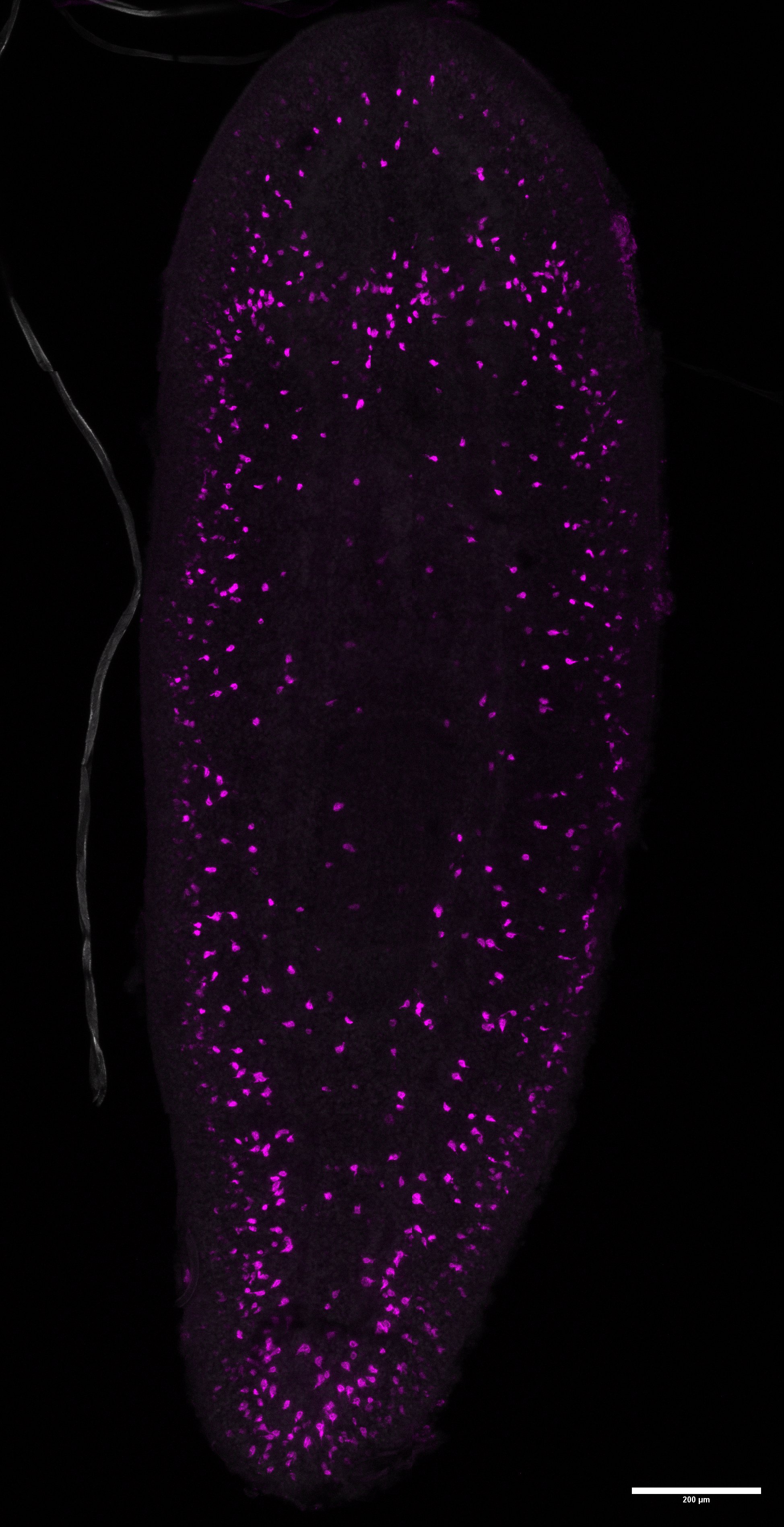

Supplement: Supplementary file 12 — Source data Fig. 5 [file 44318_2025_662_MOESM12_ESM.zip › Figure 5/5D/dd_3451/ID_2_ythdf-A_RNAi_Probe_dd3451_rhod_DAPI_10x.jpg]

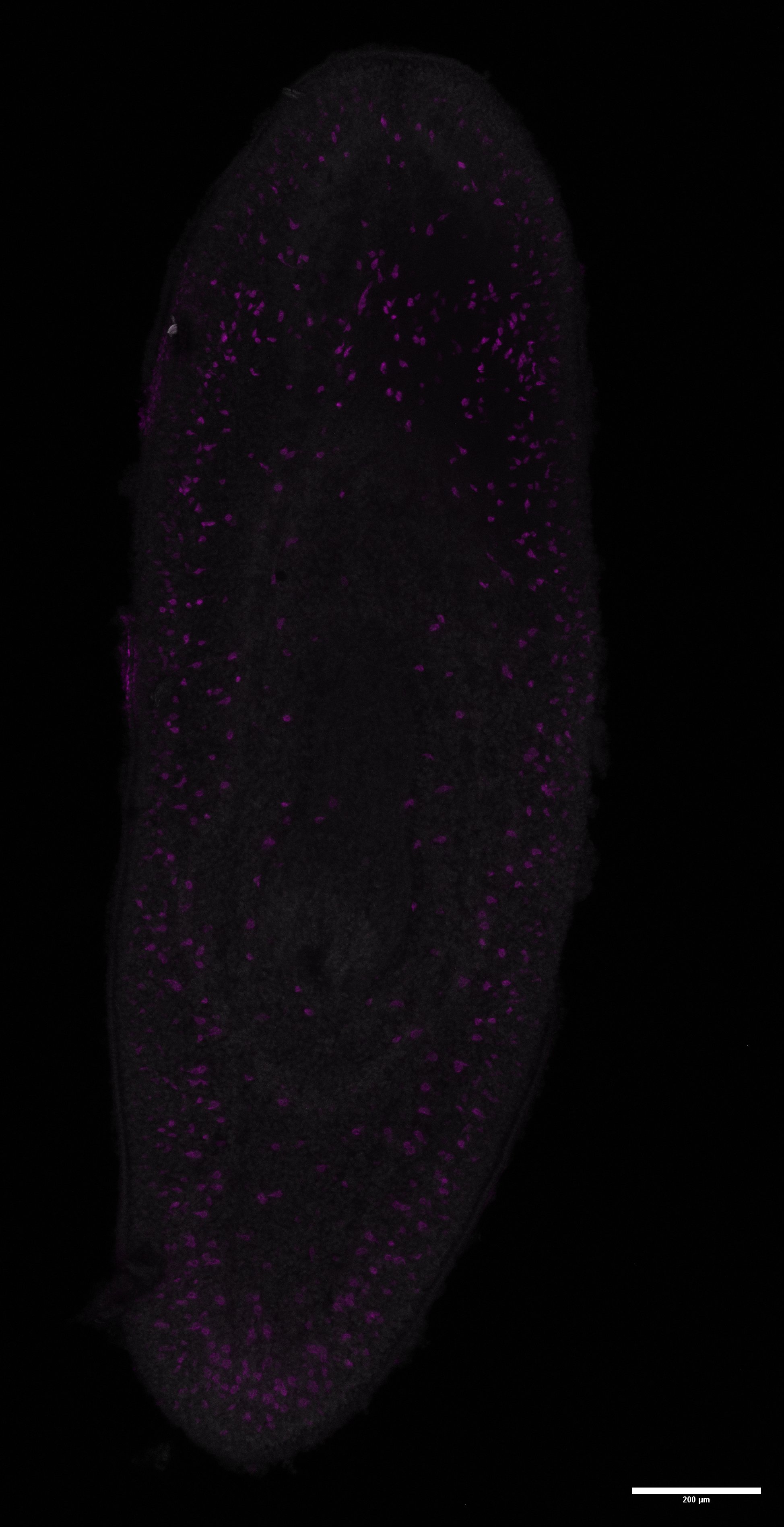

Supplement: Supplementary file 12 — Source data Fig. 5 [file 44318_2025_662_MOESM12_ESM.zip › Figure 5/5D/dd_3451/ID_2_ythdf-B_RNAi_Probe_dd3451_rhod_DAPI_10x.jpg]

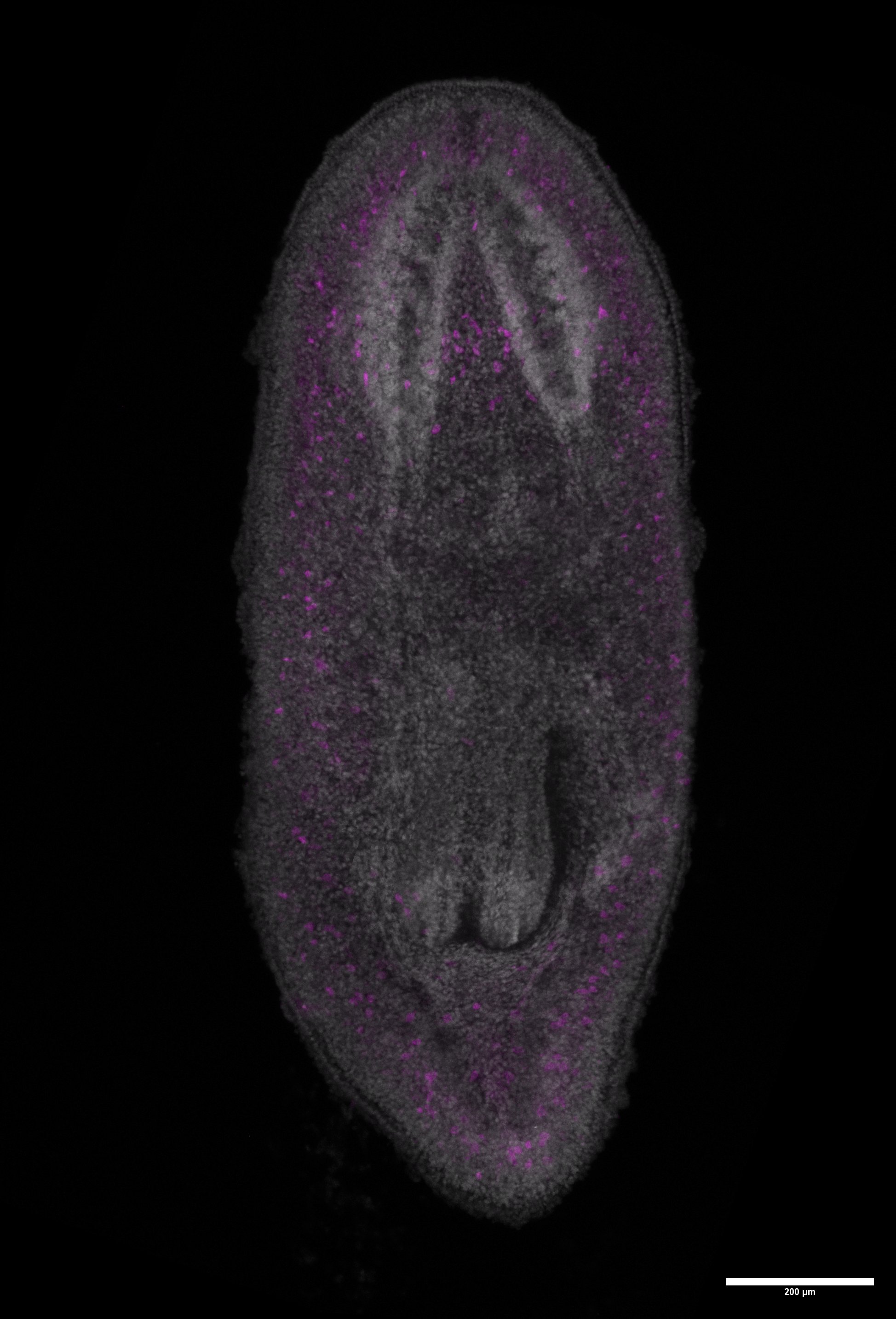

Supplement: Supplementary file 12 — Source data Fig. 5 [file 44318_2025_662_MOESM12_ESM.zip › Figure 5/5D/dd_3451/ID_3_Control_RNAi_Probe_dd3451_rhod_DAPI_10x.jpg]

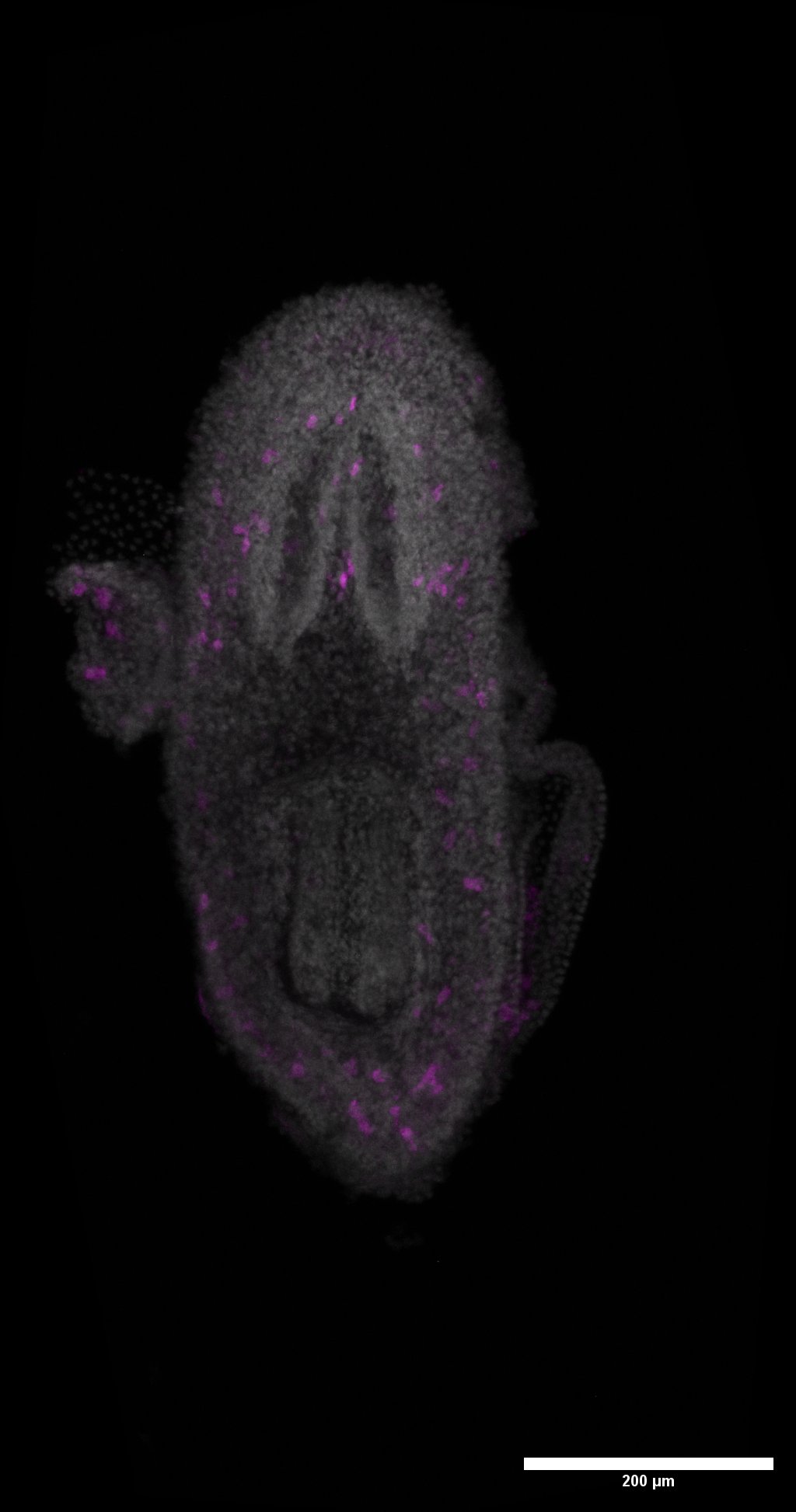

Supplement: Supplementary file 12 — Source data Fig. 5 [file 44318_2025_662_MOESM12_ESM.zip › Figure 5/5D/dd_3451/ID_3_Triple_RNAi_Probe_dd3451_rhod_DAPI_10x.jpg]

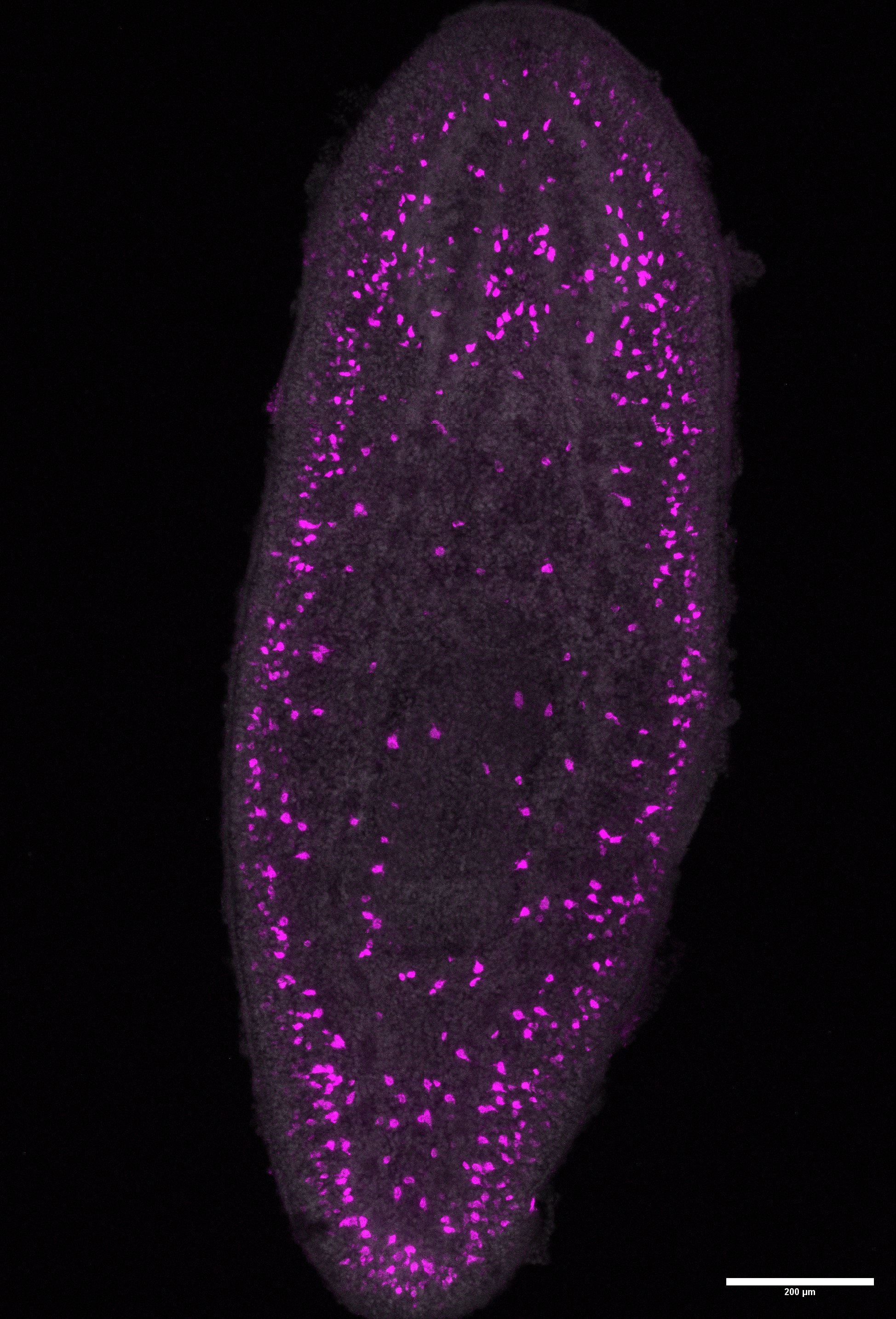

Supplement: Supplementary file 12 — Source data Fig. 5 [file 44318_2025_662_MOESM12_ESM.zip › Figure 5/5D/dd_3451/ID_3_ythdf-A_RNAi_Probe_dd3451_rhod_DAPI_10x.jpg]

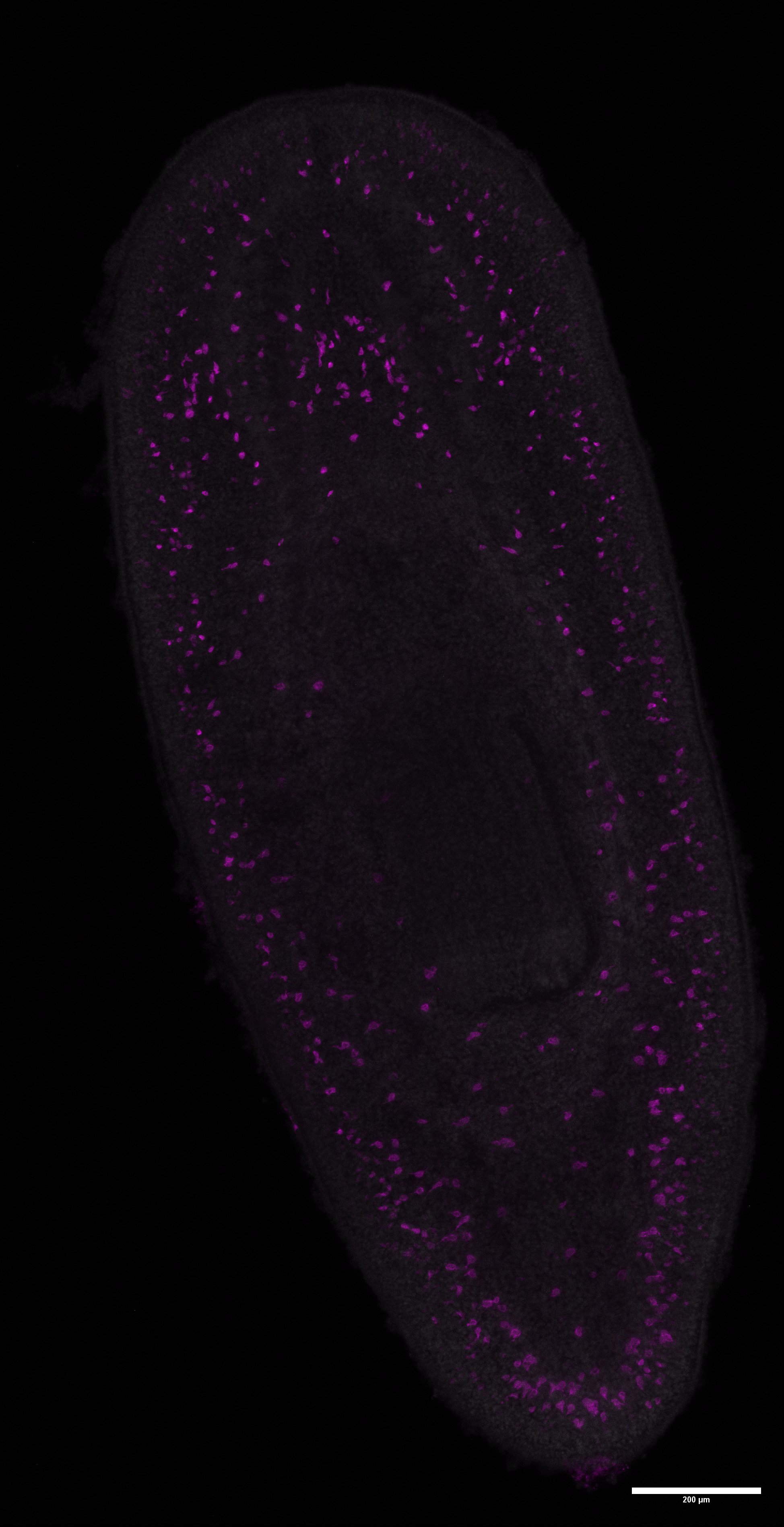

Supplement: Supplementary file 12 — Source data Fig. 5 [file 44318_2025_662_MOESM12_ESM.zip › Figure 5/5D/dd_3451/ID_3_ythdf-B_RNAi_Probe_dd3451_rhod_DAPI_10x.jpg]

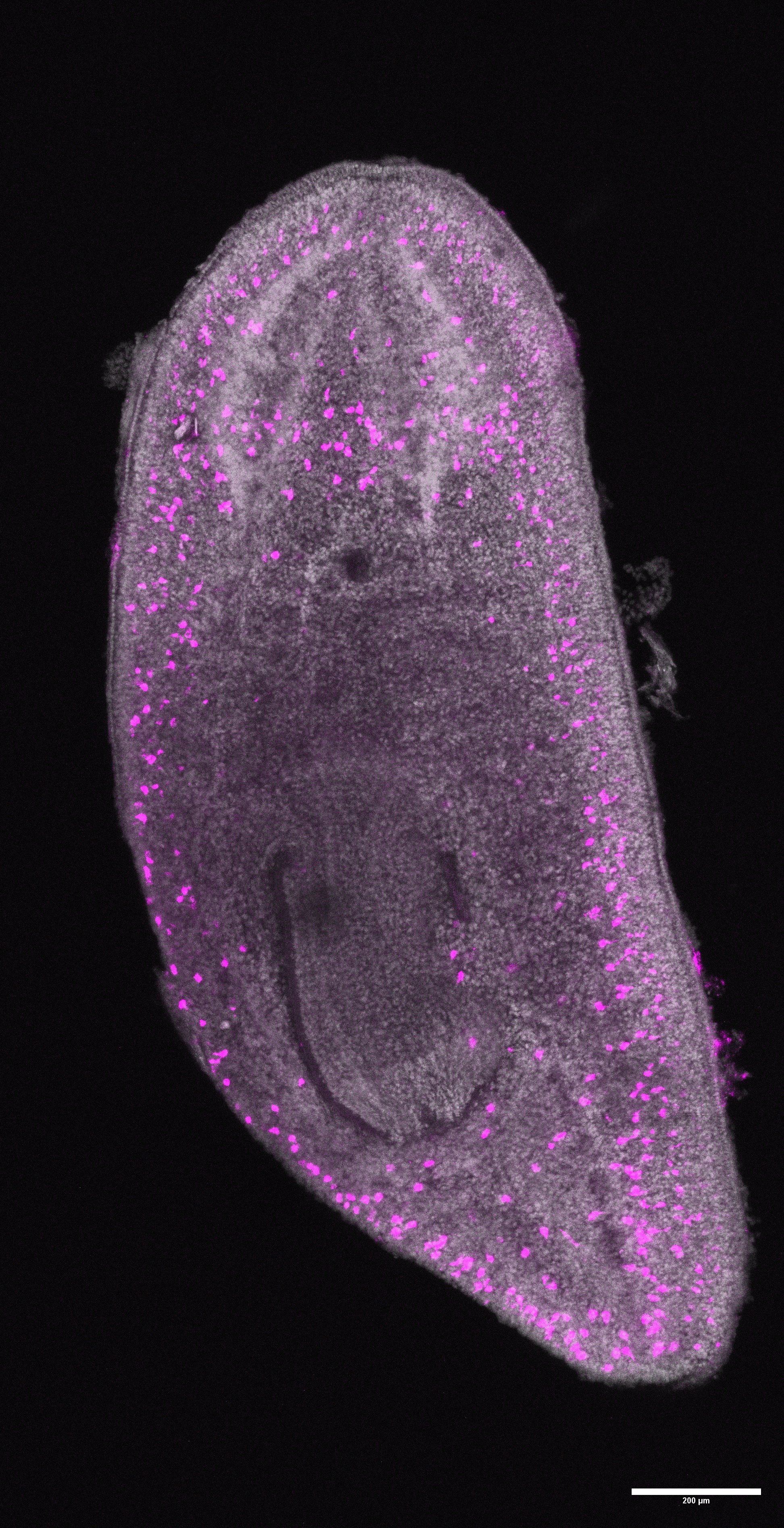

Supplement: Supplementary file 12 — Source data Fig. 5 [file 44318_2025_662_MOESM12_ESM.zip › Figure 5/5D/dd_3451/ID_3_ythdf-C_RNAi_Probe_dd3451_rhod_DAPI_10x.jpg]

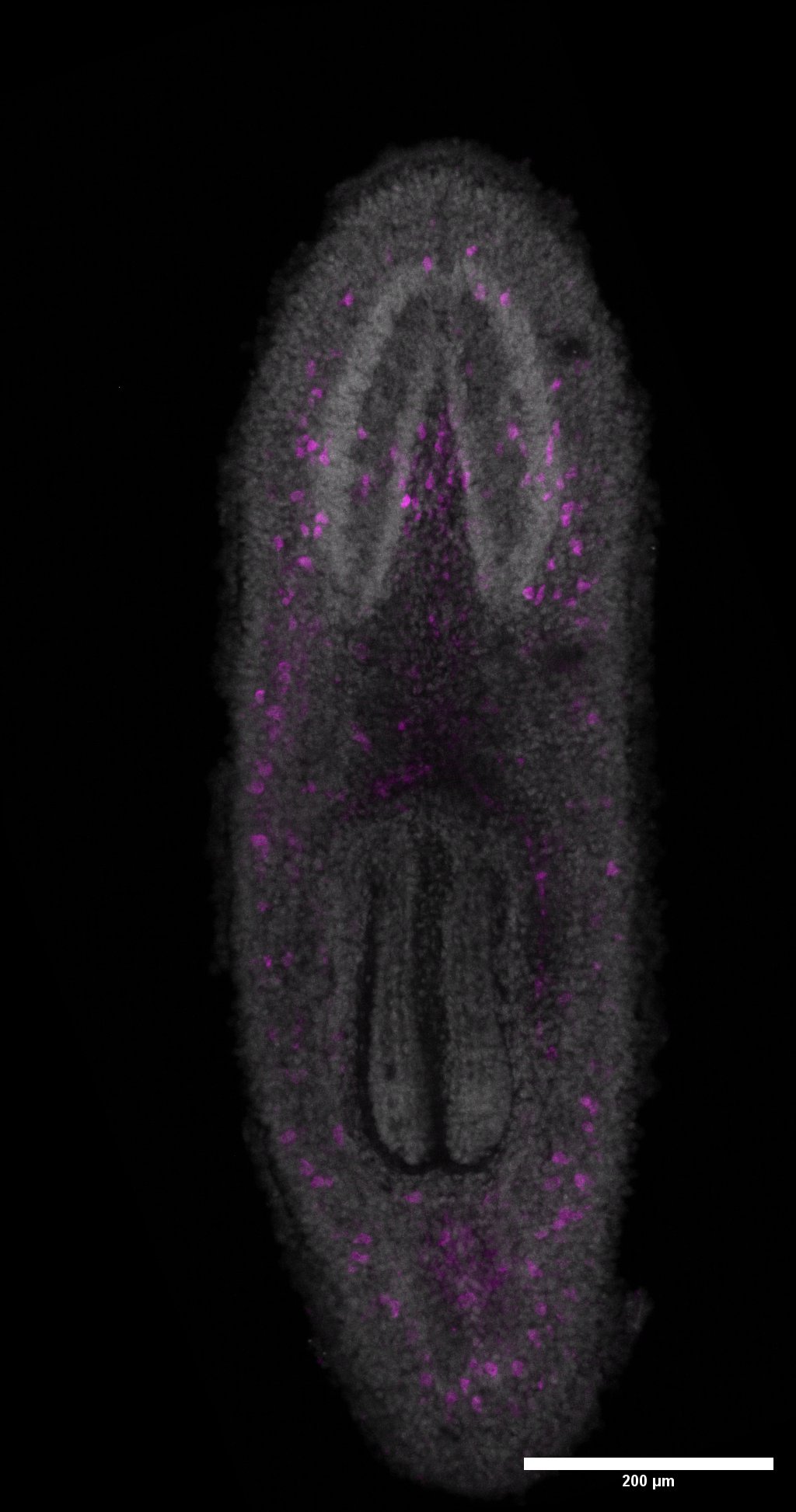

Supplement: Supplementary file 12 — Source data Fig. 5 [file 44318_2025_662_MOESM12_ESM.zip › Figure 5/5D/dd_3451/ID_4_Control_RNAi_Probe_dd3451_rhod_DAPI_10x.jpg]

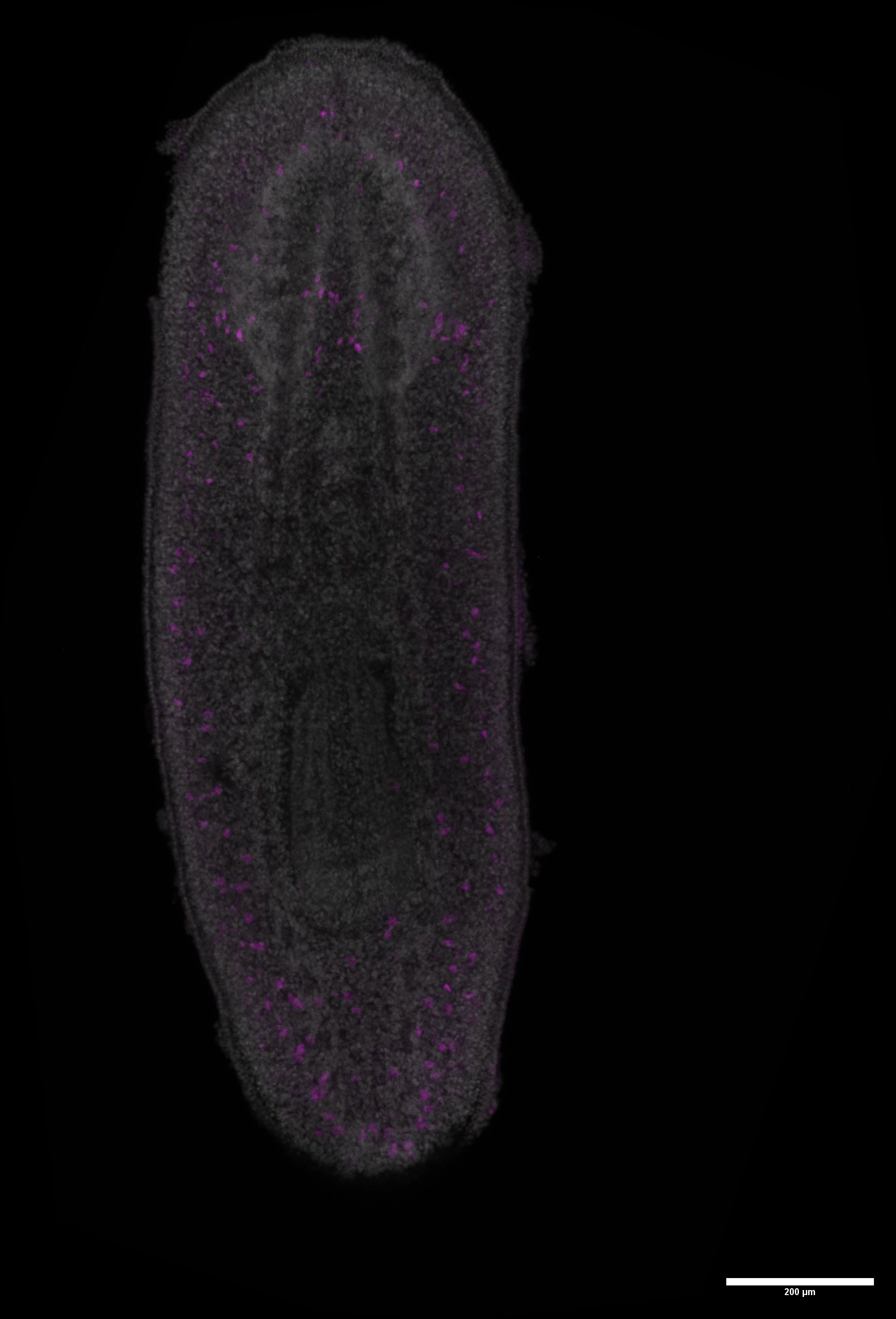

Supplement: Supplementary file 12 — Source data Fig. 5 [file 44318_2025_662_MOESM12_ESM.zip › Figure 5/5D/dd_3451/ID_4_Triple_RNAi_Probe_dd3451_rhod_DAPI_10x.jpg]

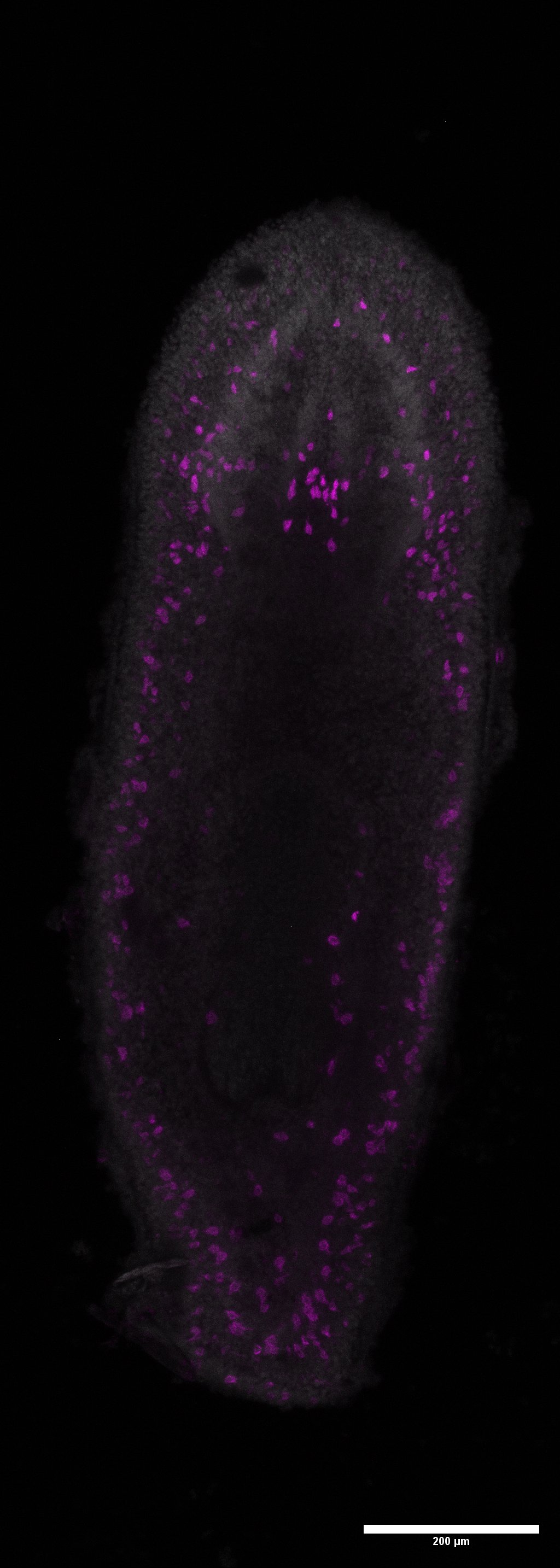

Supplement: Supplementary file 12 — Source data Fig. 5 [file 44318_2025_662_MOESM12_ESM.zip › Figure 5/5D/dd_3451/ID_4_ythdf-A_RNAi_Probe_dd3451_rhod_DAPI_10x.jpg]

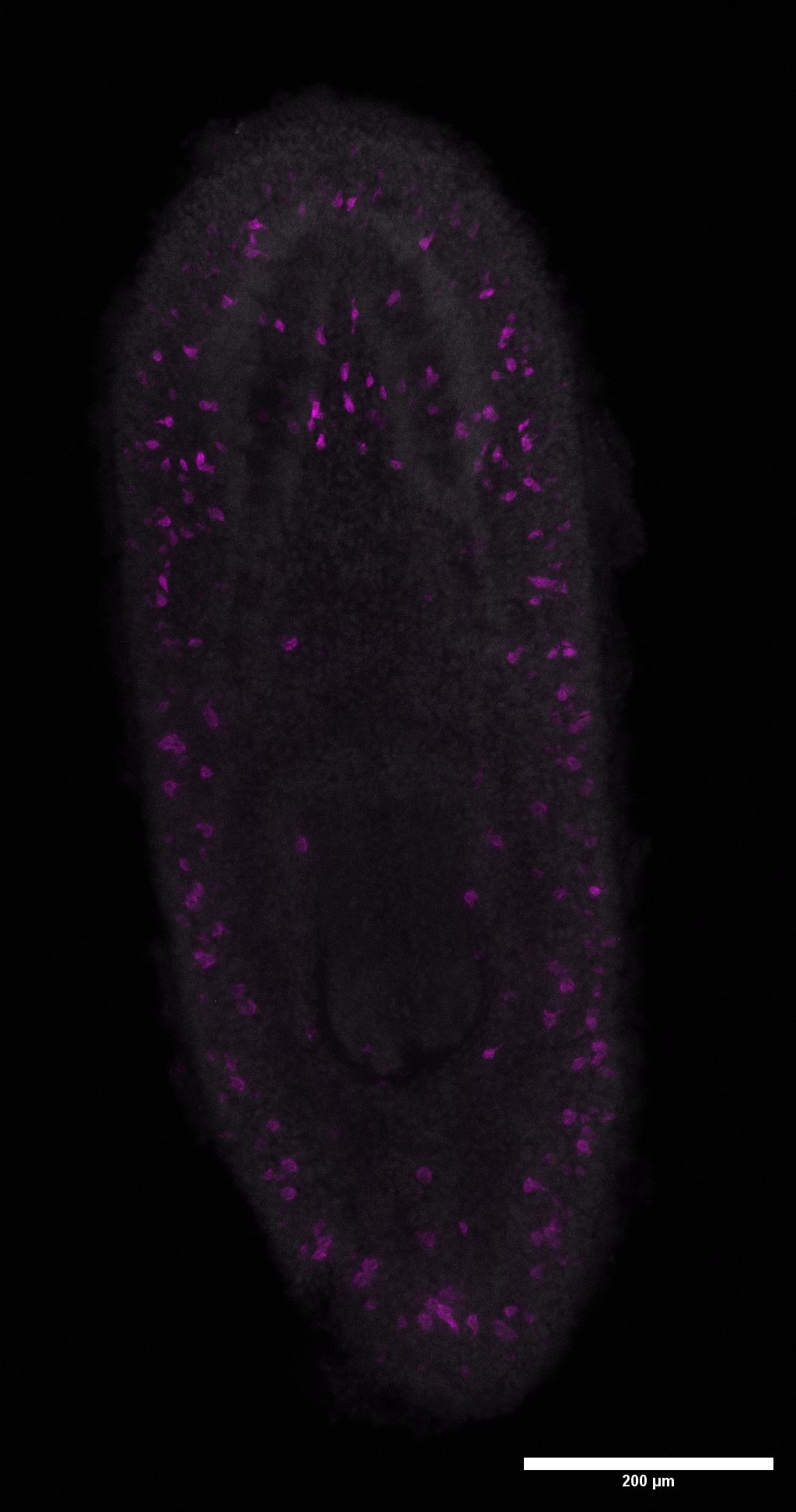

Supplement: Supplementary file 12 — Source data Fig. 5 [file 44318_2025_662_MOESM12_ESM.zip › Figure 5/5D/dd_3451/ID_4_ythdf-B_RNAi_Probe_dd3451_rhod_DAPI_10x.jpg]

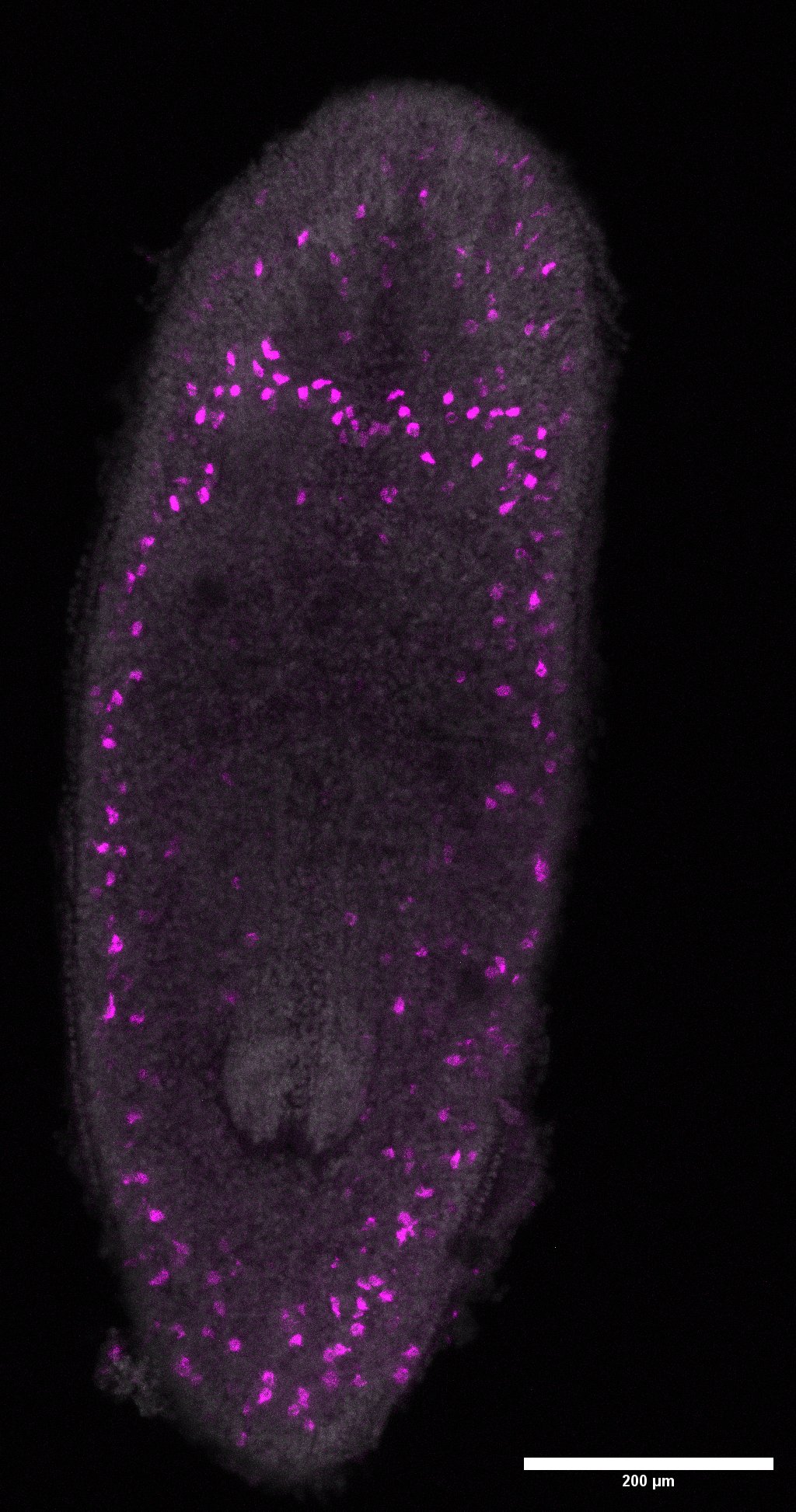

Supplement: Supplementary file 12 — Source data Fig. 5 [file 44318_2025_662_MOESM12_ESM.zip › Figure 5/5D/dd_3451/ID_4_ythdf-C_RNAi_Probe_dd3451_rhod_DAPI_10x.jpg]

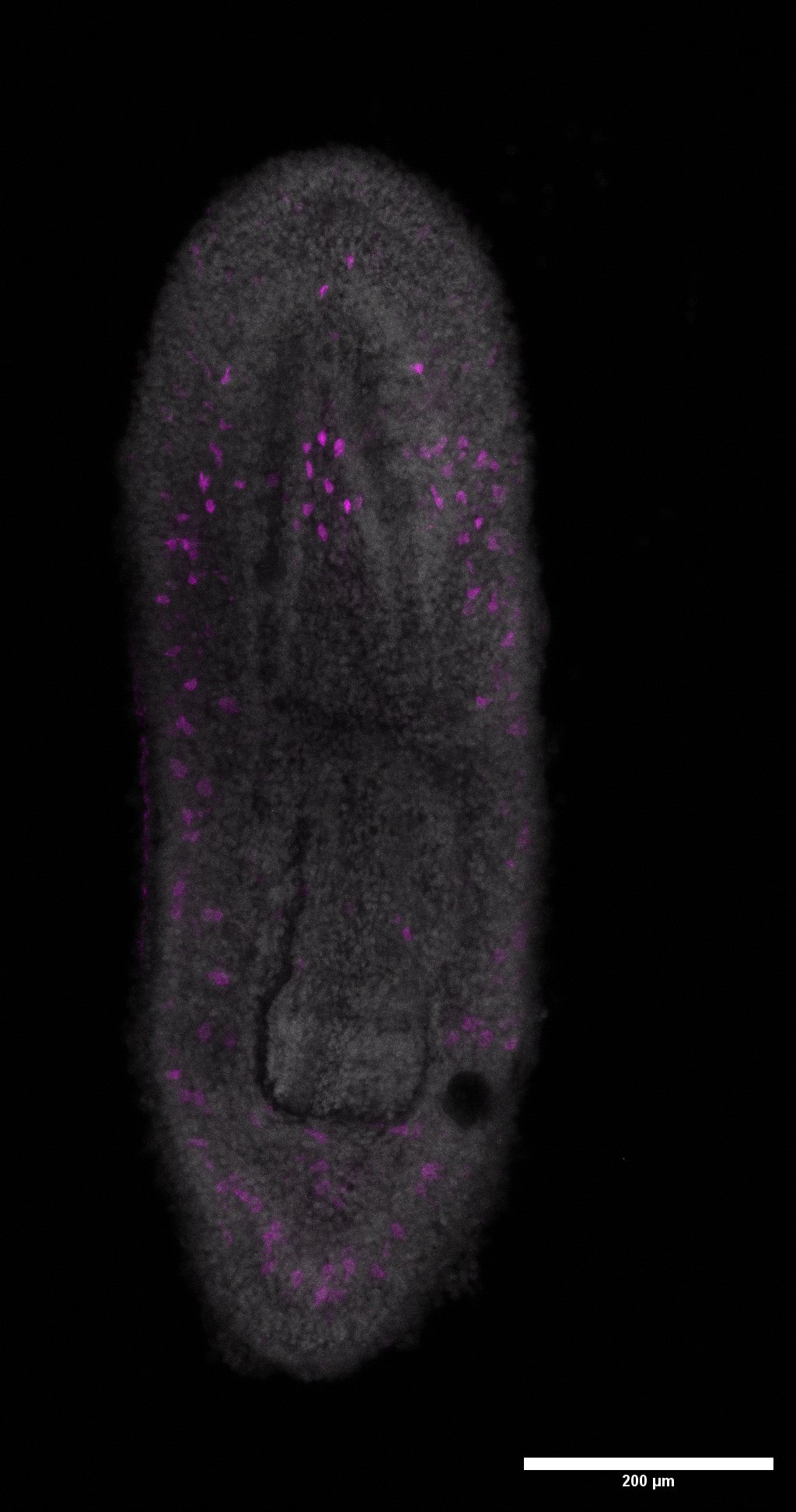

Supplement: Supplementary file 12 — Source data Fig. 5 [file 44318_2025_662_MOESM12_ESM.zip › Figure 5/5D/dd_3451/ID_5_Control_RNAi_Probe_dd3451_rhod_DAPI_10x.jpg]

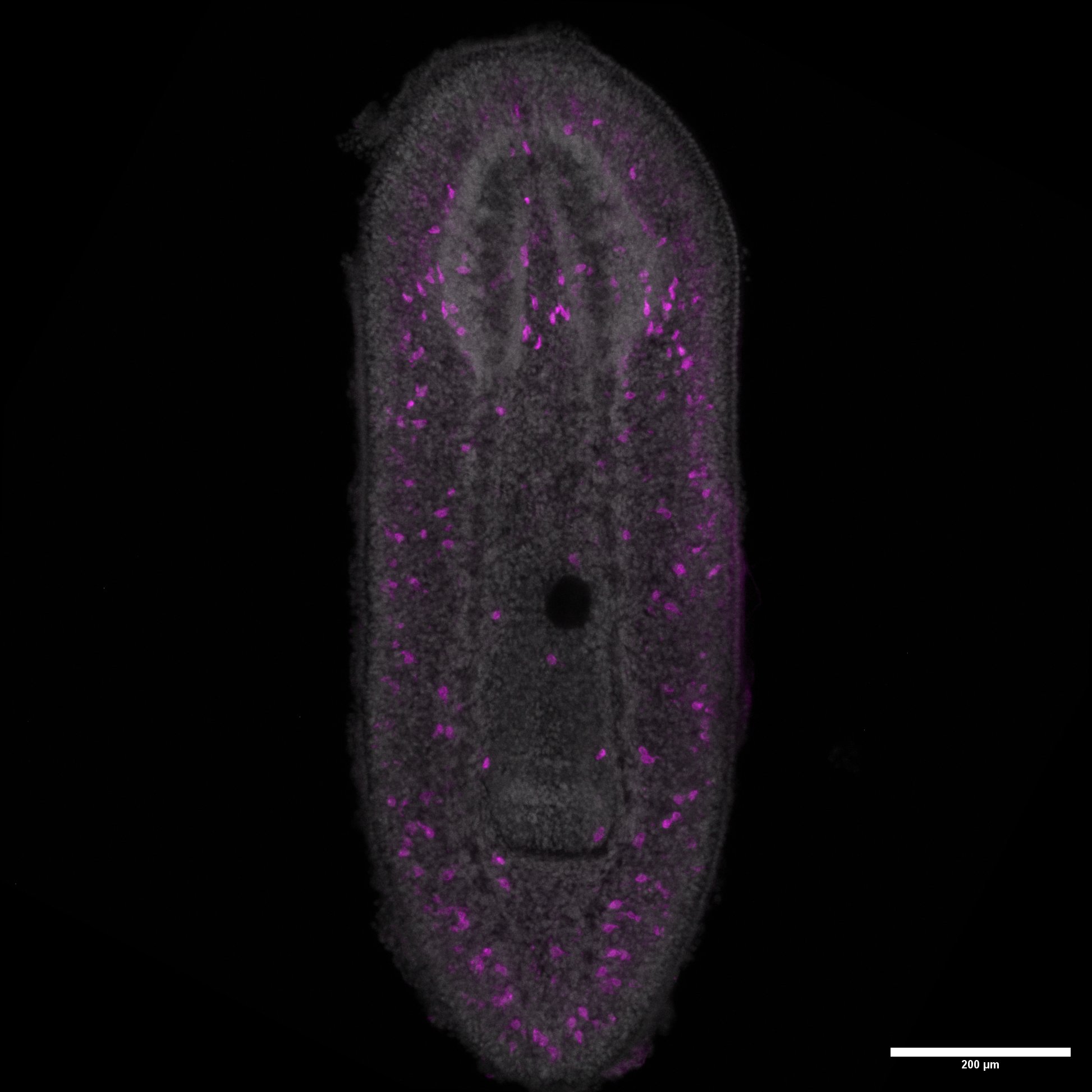

Supplement: Supplementary file 12 — Source data Fig. 5 [file 44318_2025_662_MOESM12_ESM.zip › Figure 5/5D/dd_3451/ID_5_Triple_RNAi_Probe_dd3451_rhod_DAPI_10x.jpg]

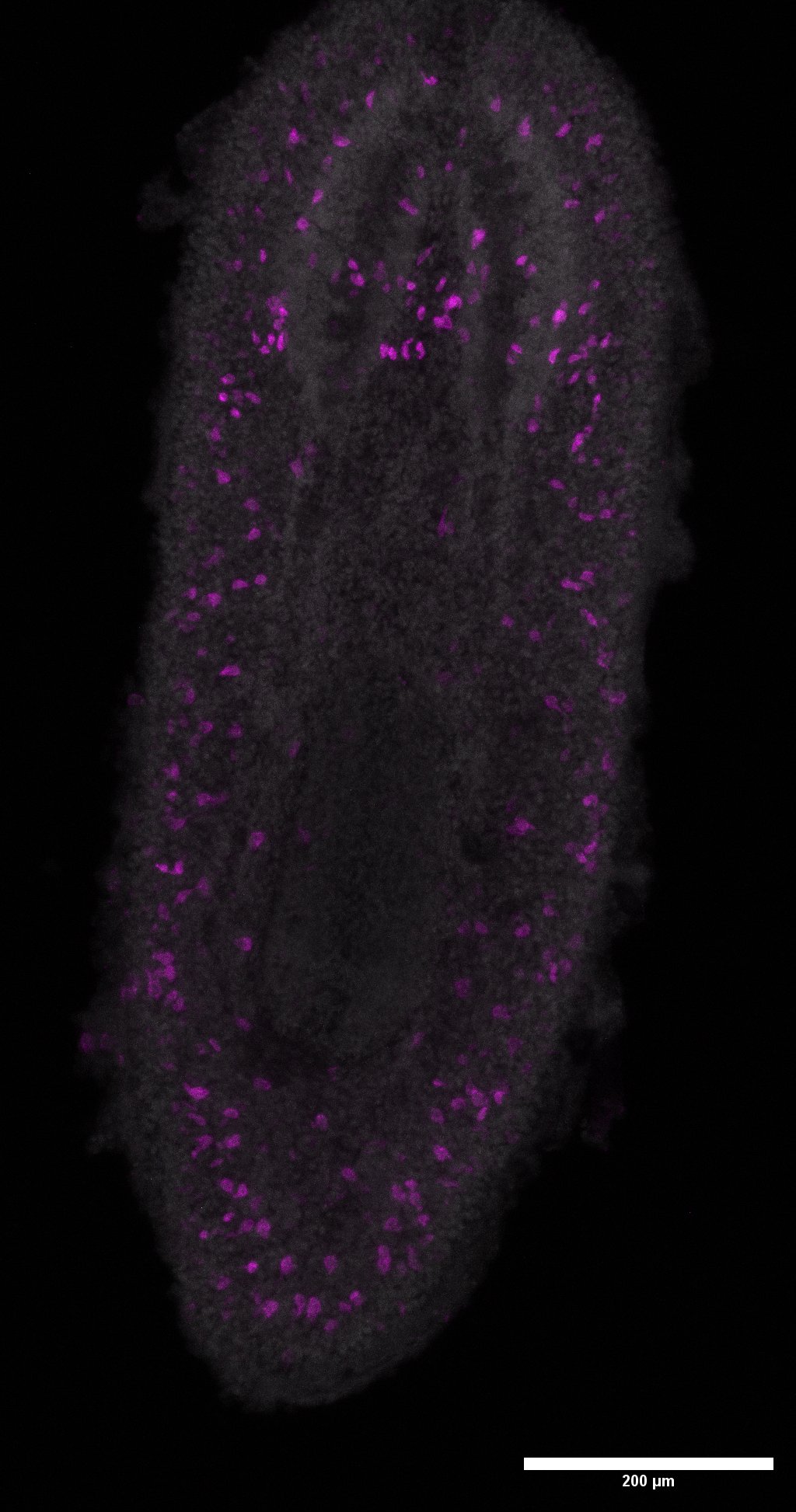

Supplement: Supplementary file 12 — Source data Fig. 5 [file 44318_2025_662_MOESM12_ESM.zip › Figure 5/5D/dd_3451/ID_5_ythdf-A_RNAi_Probe_dd3451_rhod_DAPI_10x.jpg]

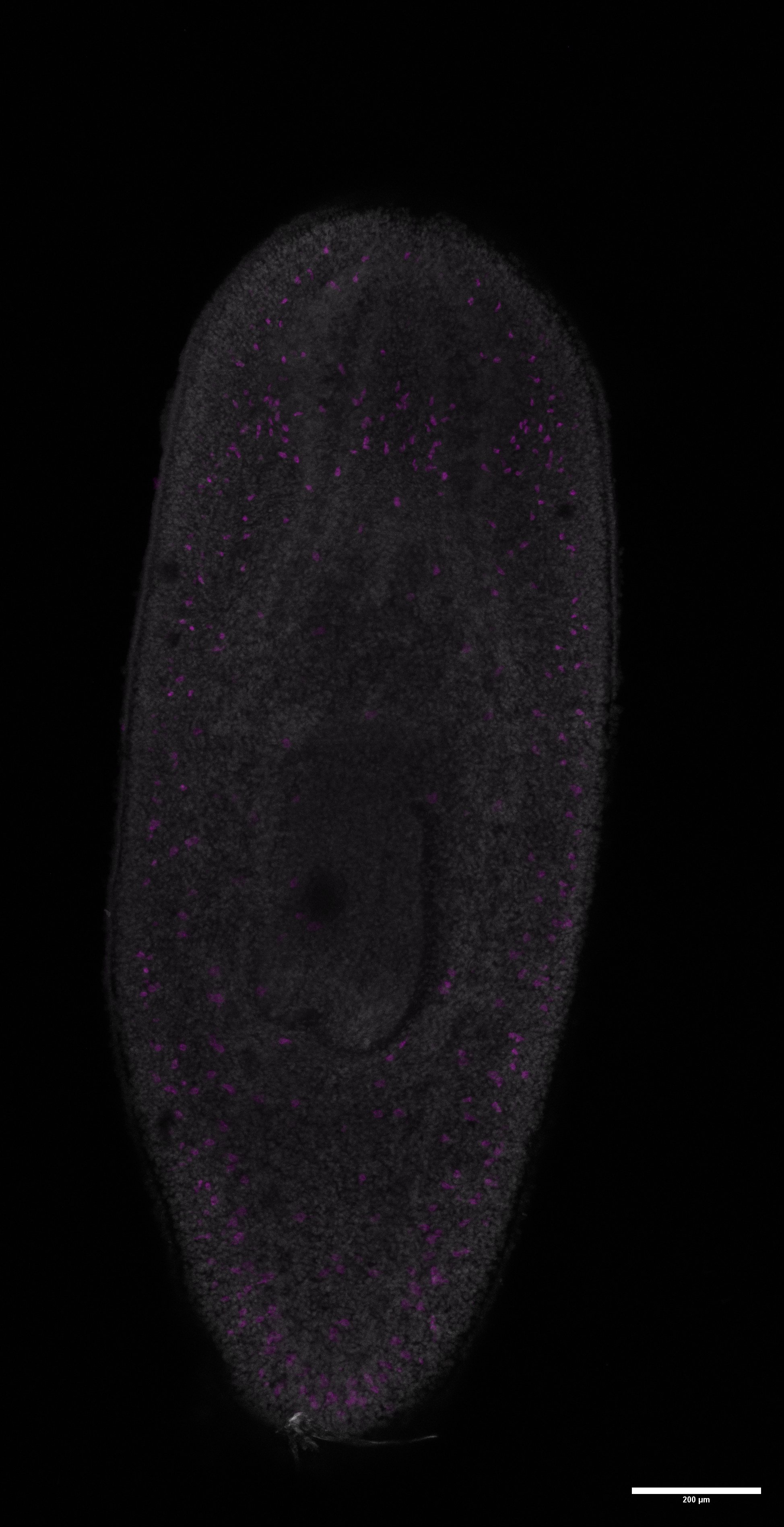

Supplement: Supplementary file 12 — Source data Fig. 5 [file 44318_2025_662_MOESM12_ESM.zip › Figure 5/5D/dd_3451/ID_5_ythdf-B_RNAi_Probe_dd3451_rhod_DAPI_10x.jpg]

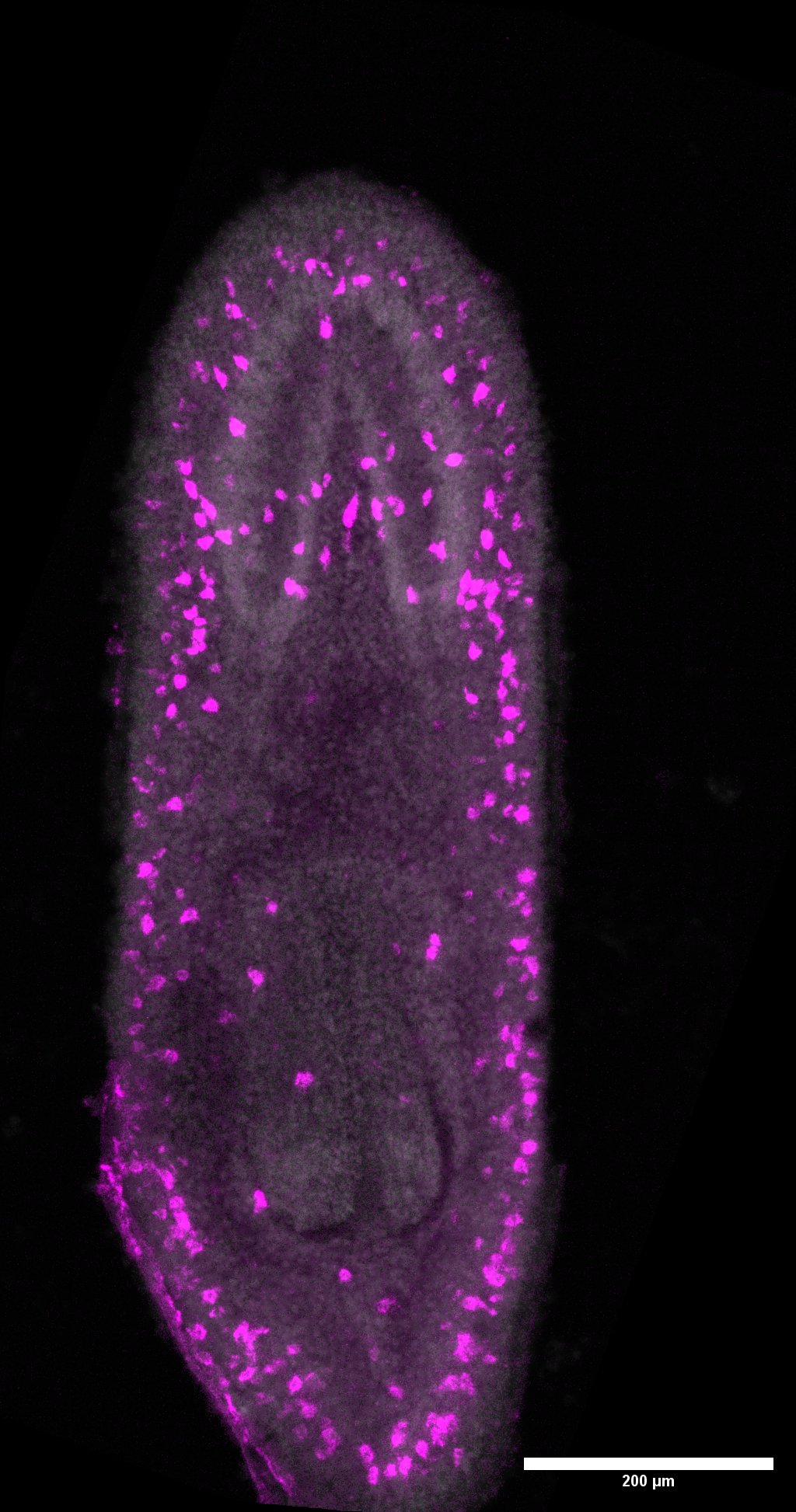

Supplement: Supplementary file 12 — Source data Fig. 5 [file 44318_2025_662_MOESM12_ESM.zip › Figure 5/5D/dd_3451/ID_5_ythdf-C_RNAi_Probe_dd3451_rhod_DAPI_10x.jpg]

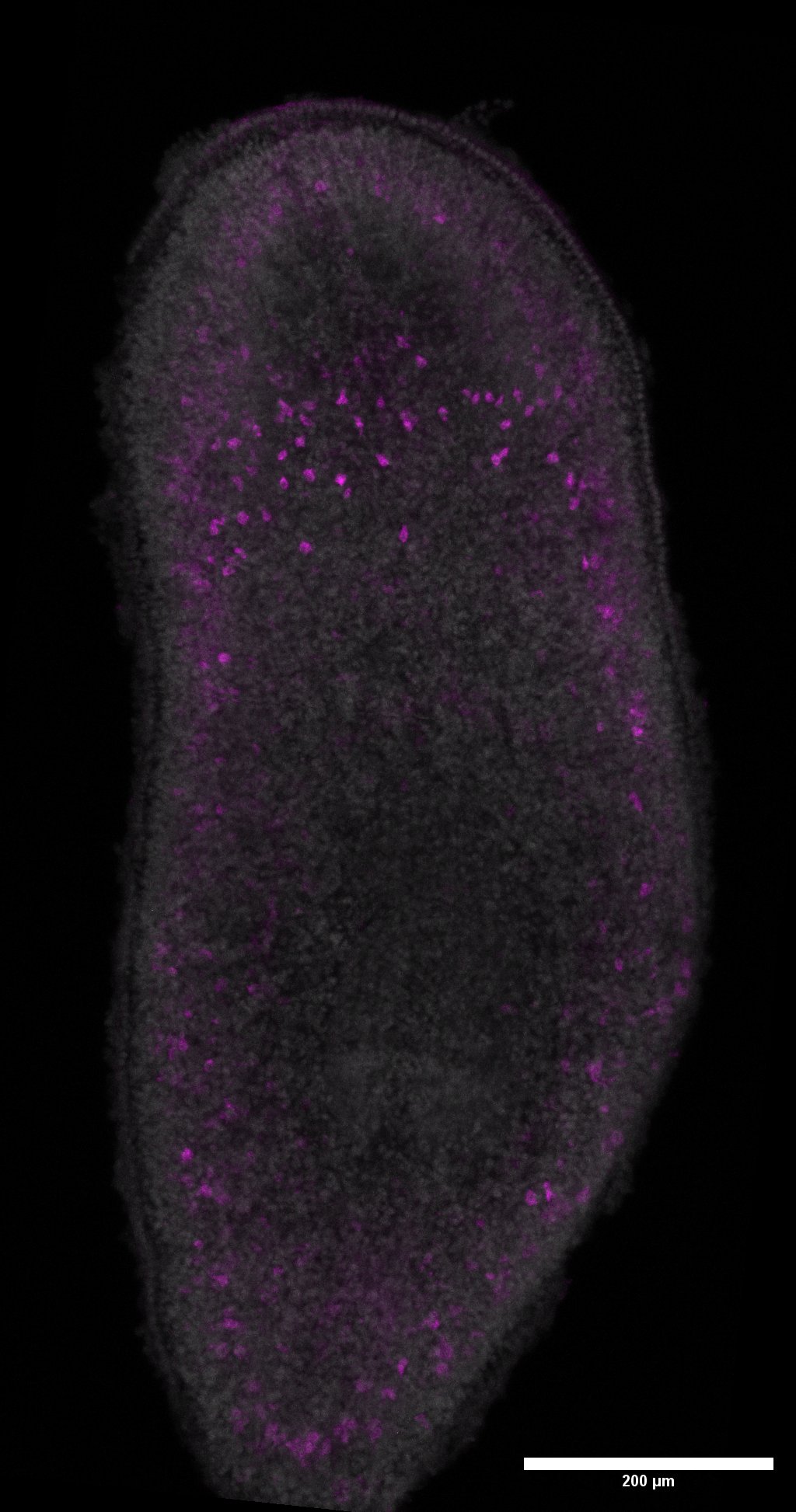

Supplement: Supplementary file 12 — Source data Fig. 5 [file 44318_2025_662_MOESM12_ESM.zip › Figure 5/5D/dd_3451/ID_6_Control_RNAi_Probe_dd3451_rhod_DAPI_10x.jpg]

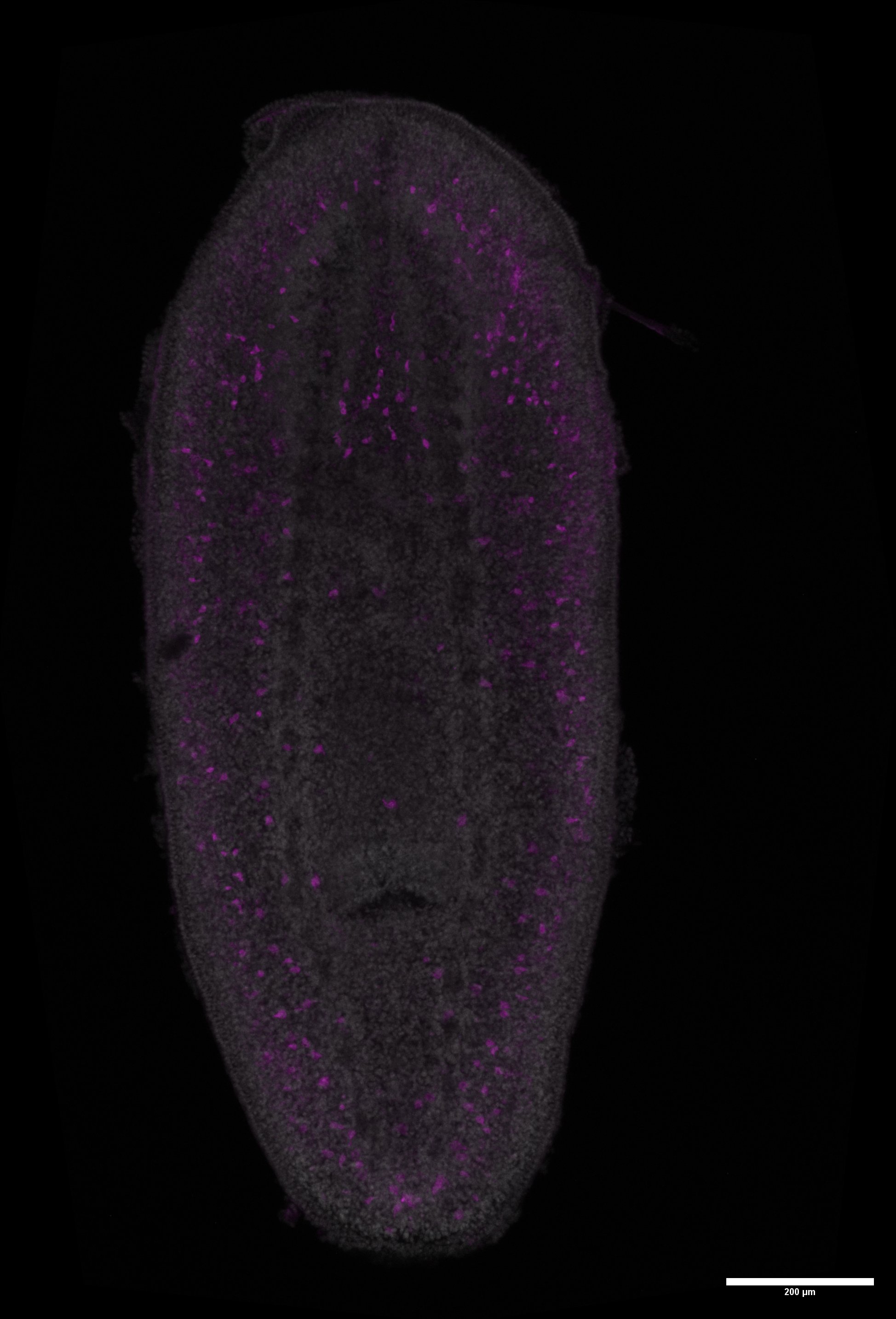

Supplement: Supplementary file 12 — Source data Fig. 5 [file 44318_2025_662_MOESM12_ESM.zip › Figure 5/5D/dd_3451/ID_6_Triple_RNAi_Probe_dd3451_rhod_DAPI_10x.jpg]

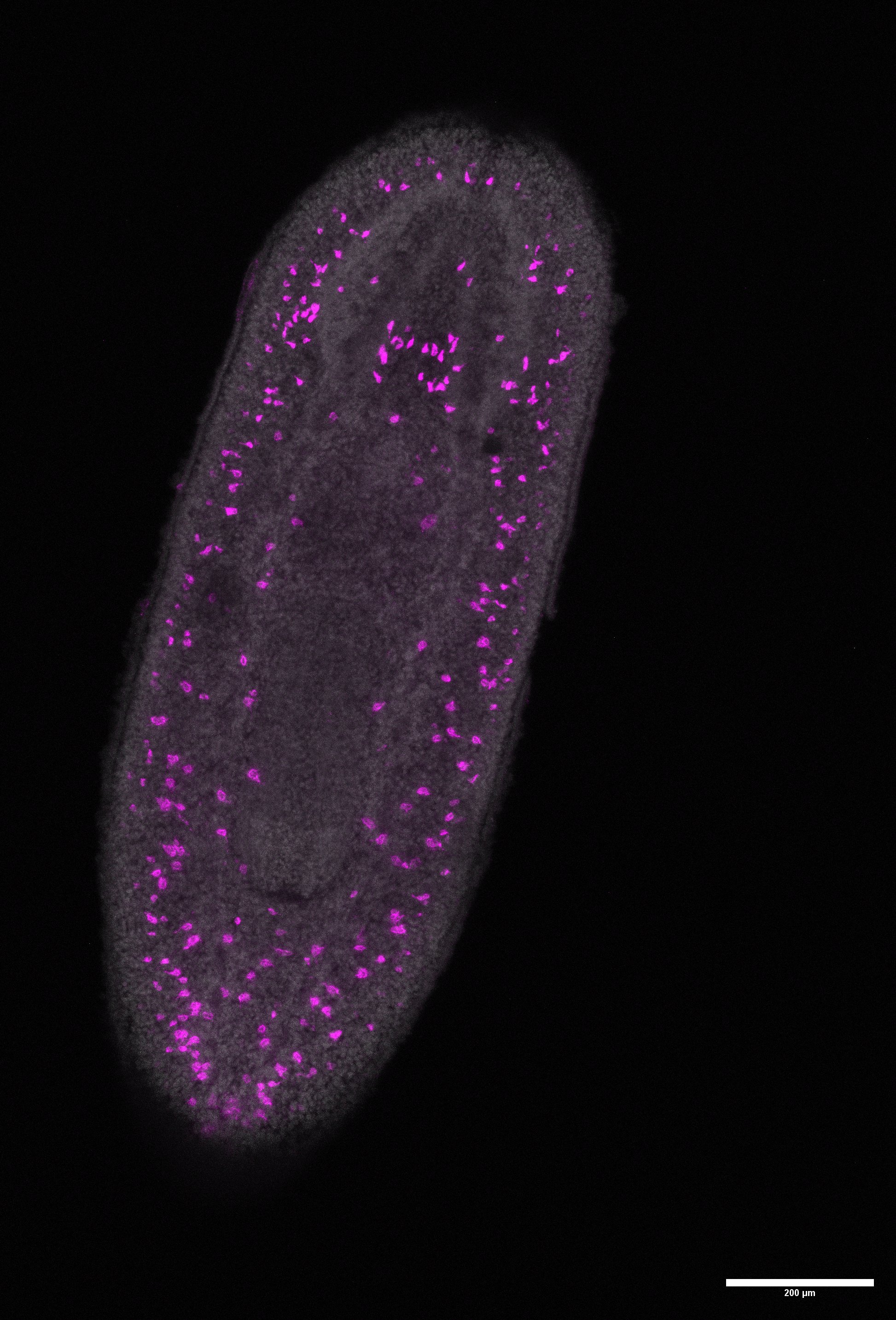

Supplement: Supplementary file 12 — Source data Fig. 5 [file 44318_2025_662_MOESM12_ESM.zip › Figure 5/5D/dd_3451/ID_6_ythdf-B_RNAi_Probe_dd3451_rhod_DAPI_10x.jpg]

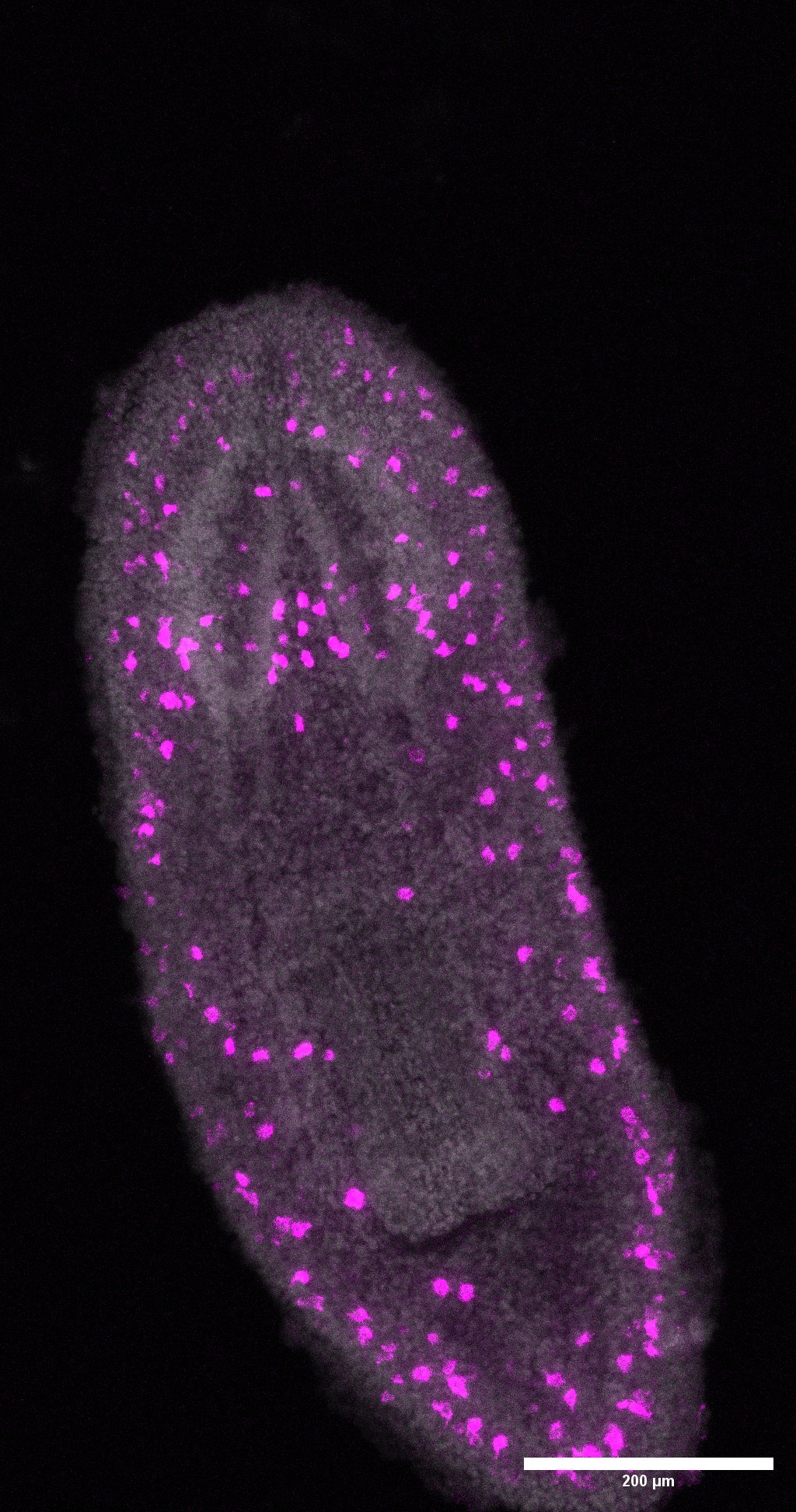

Supplement: Supplementary file 12 — Source data Fig. 5 [file 44318_2025_662_MOESM12_ESM.zip › Figure 5/5D/dd_3451/ID_6_ythdf-C_RNAi_Probe_dd3451_rhod_DAPI_10x.jpg]

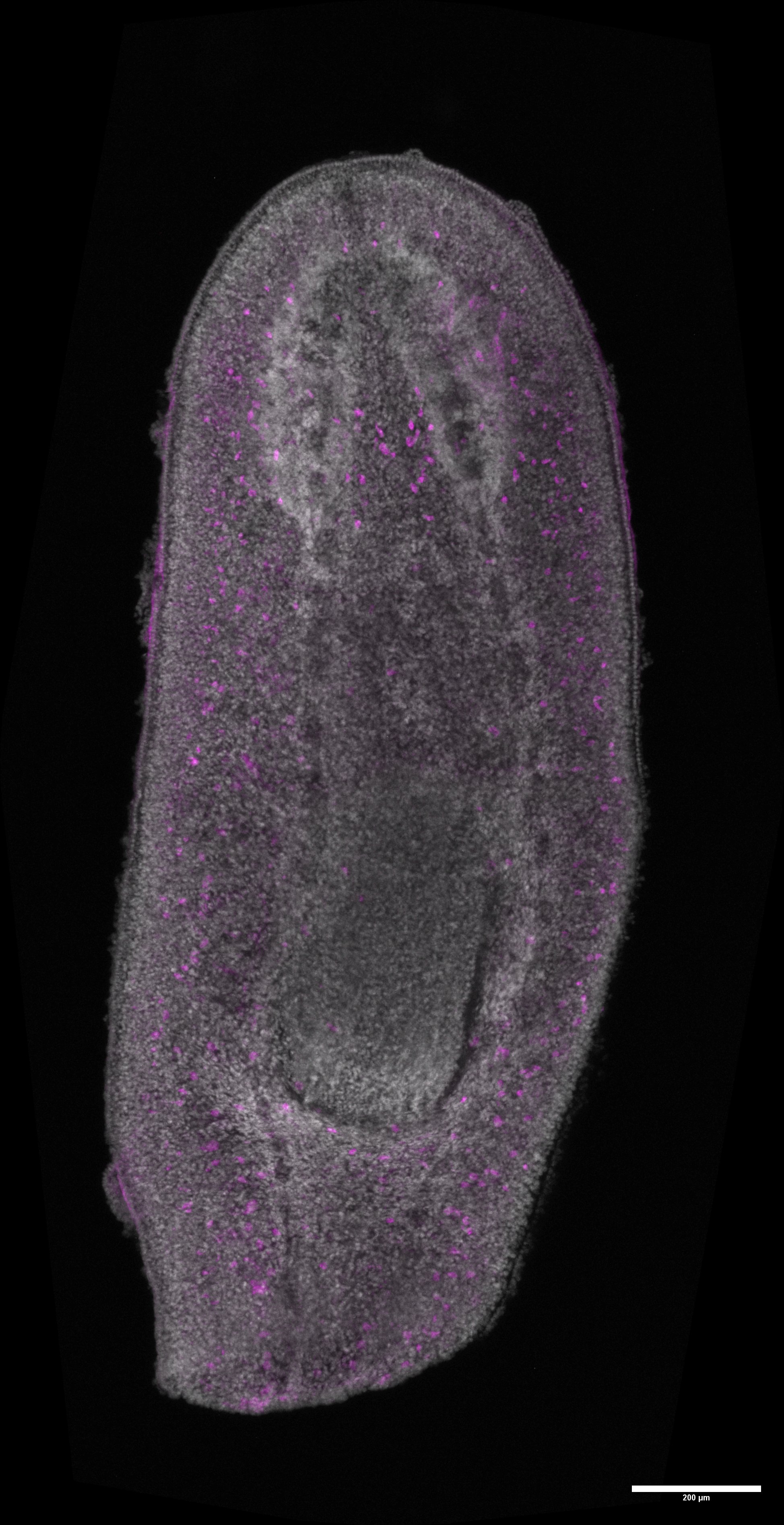

Supplement: Supplementary file 12 — Source data Fig. 5 [file 44318_2025_662_MOESM12_ESM.zip › Figure 5/5D/dd_3451/ID_7_Control_RNAi_Probe_dd3451_rhod_DAPI_10x.jpg]

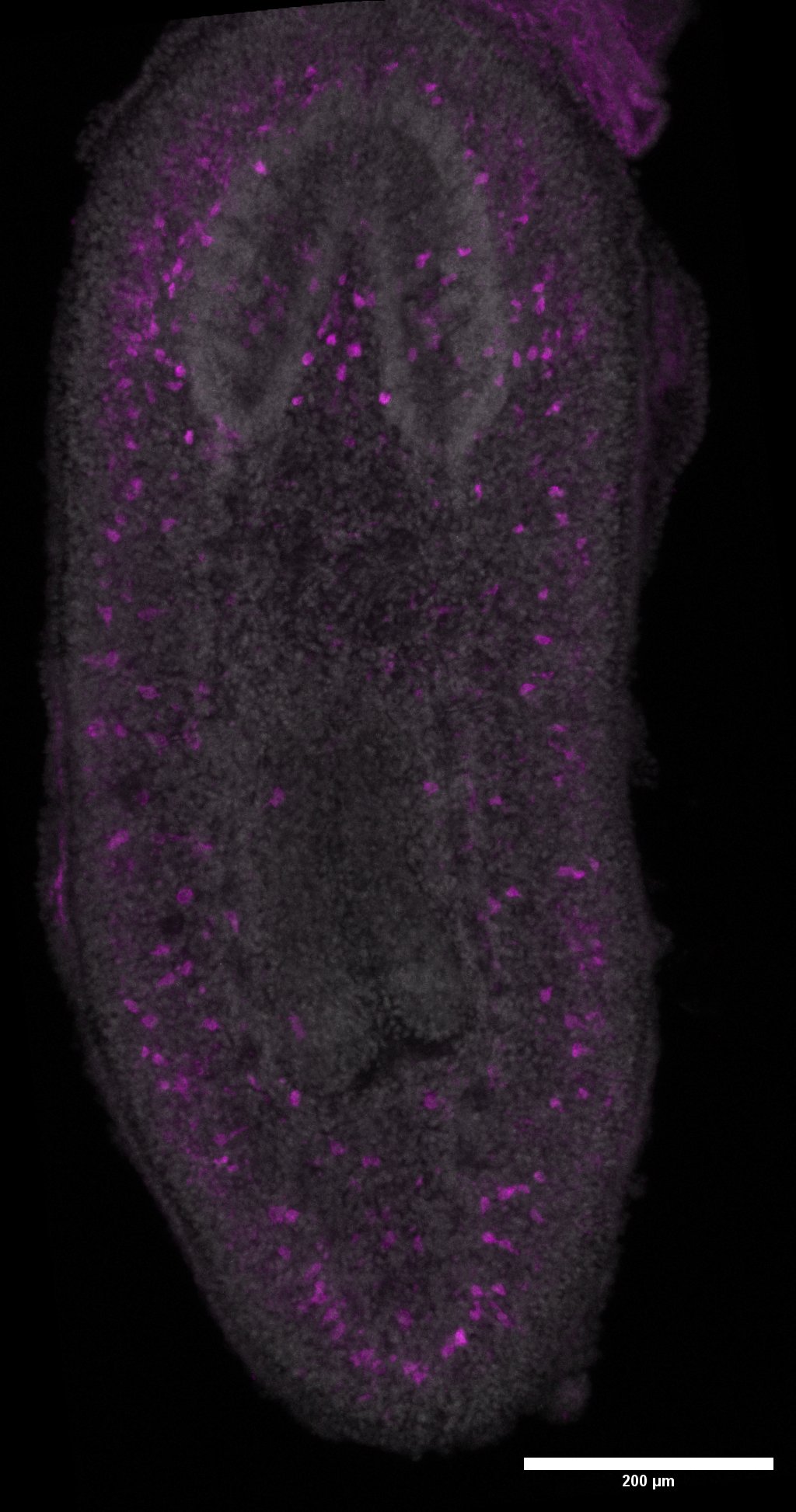

Supplement: Supplementary file 12 — Source data Fig. 5 [file 44318_2025_662_MOESM12_ESM.zip › Figure 5/5D/dd_3451/ID_7_Triple_RNAi_Probe_dd3451_rhod_DAPI_10x.jpg]

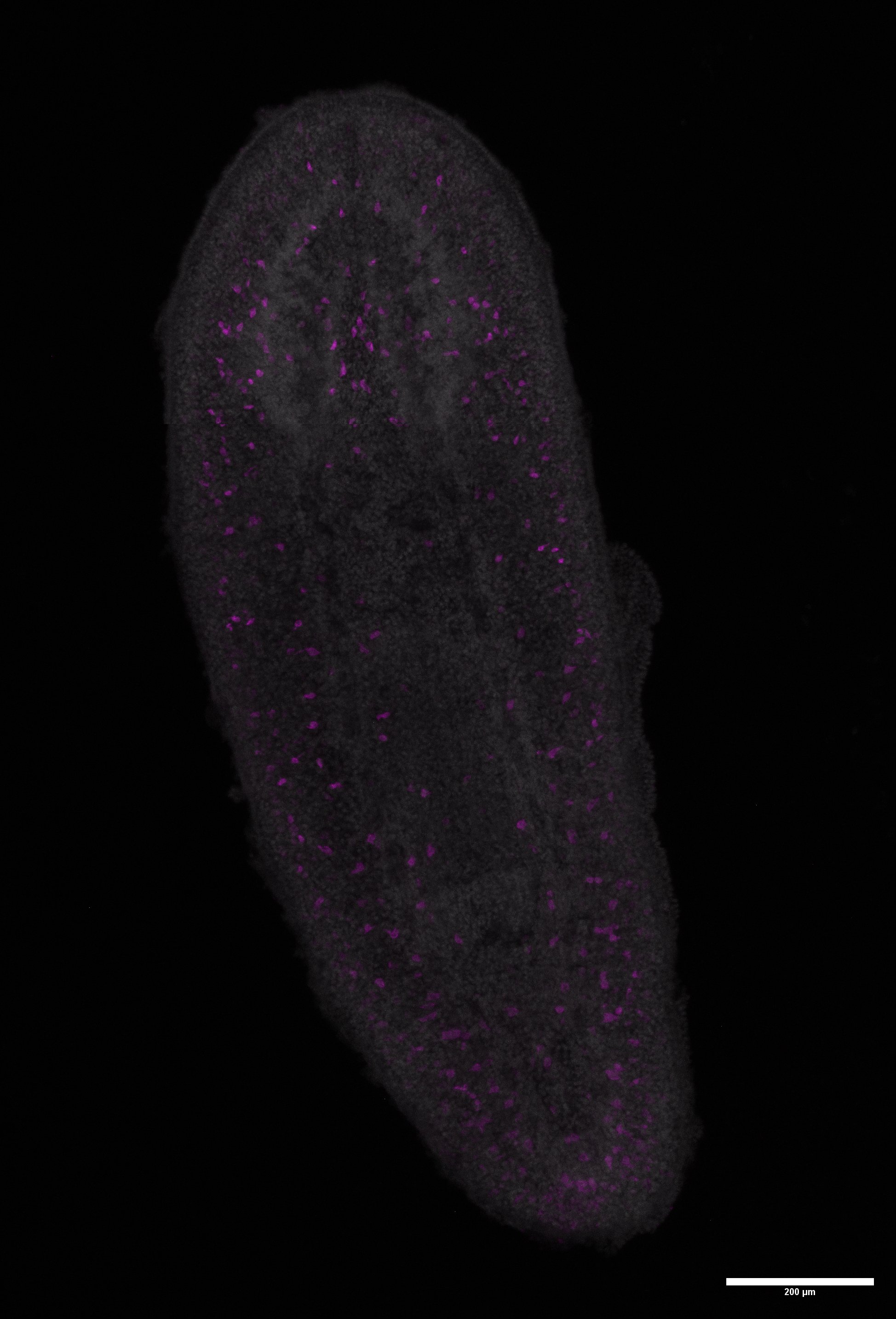

Supplement: Supplementary file 12 — Source data Fig. 5 [file 44318_2025_662_MOESM12_ESM.zip › Figure 5/5D/dd_3451/ID_7_ythdf-A_RNAi_Probe_dd3451_rhod_DAPI_10x.jpg]

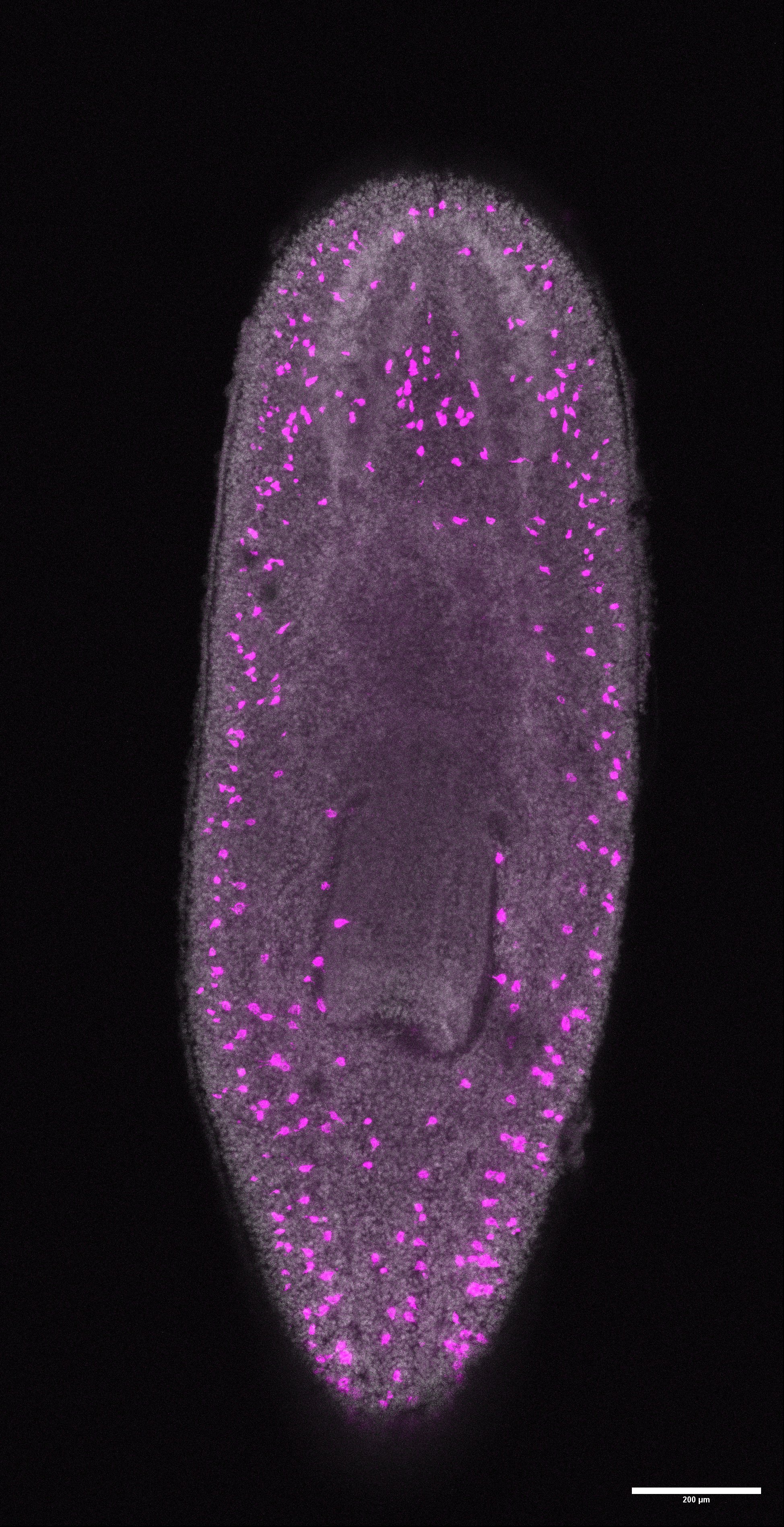

Supplement: Supplementary file 12 — Source data Fig. 5 [file 44318_2025_662_MOESM12_ESM.zip › Figure 5/5D/dd_3451/ID_7_ythdf-B_RNAi_Probe_dd3451_rhod_DAPI_10x.jpg]

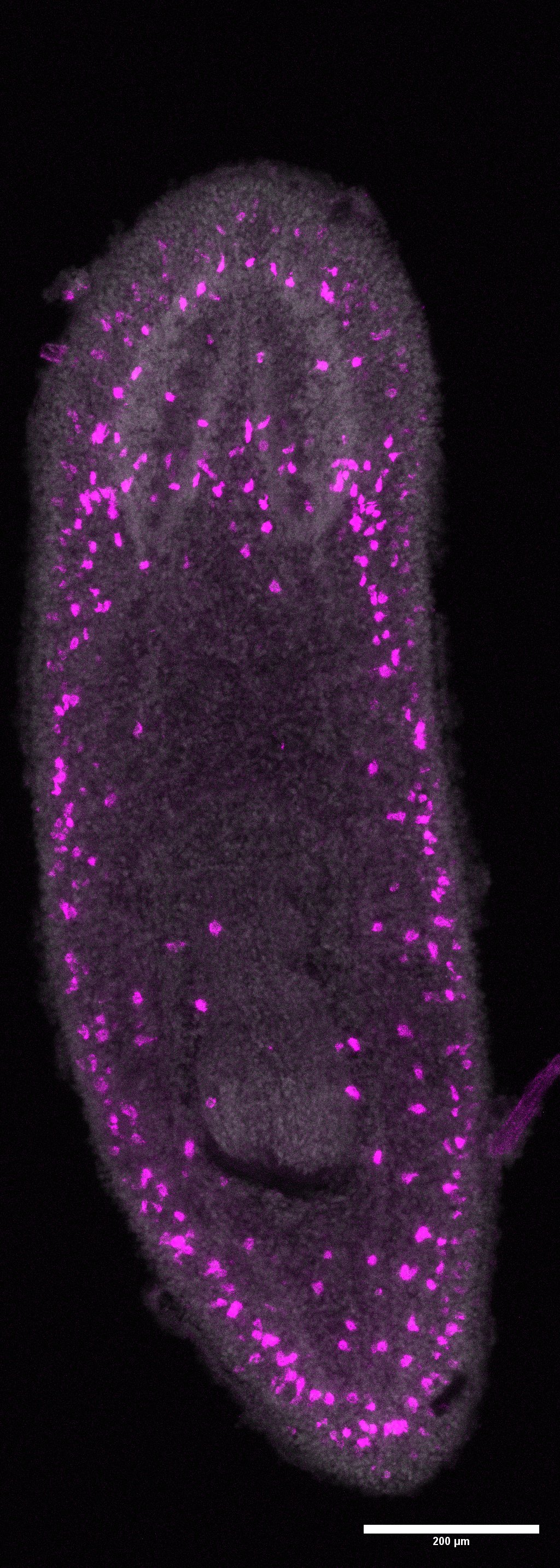

Supplement: Supplementary file 12 — Source data Fig. 5 [file 44318_2025_662_MOESM12_ESM.zip › Figure 5/5D/dd_3451/ID_7_ythdf-C_RNAi_Probe_dd3451_rhod_DAPI_10x.jpg]

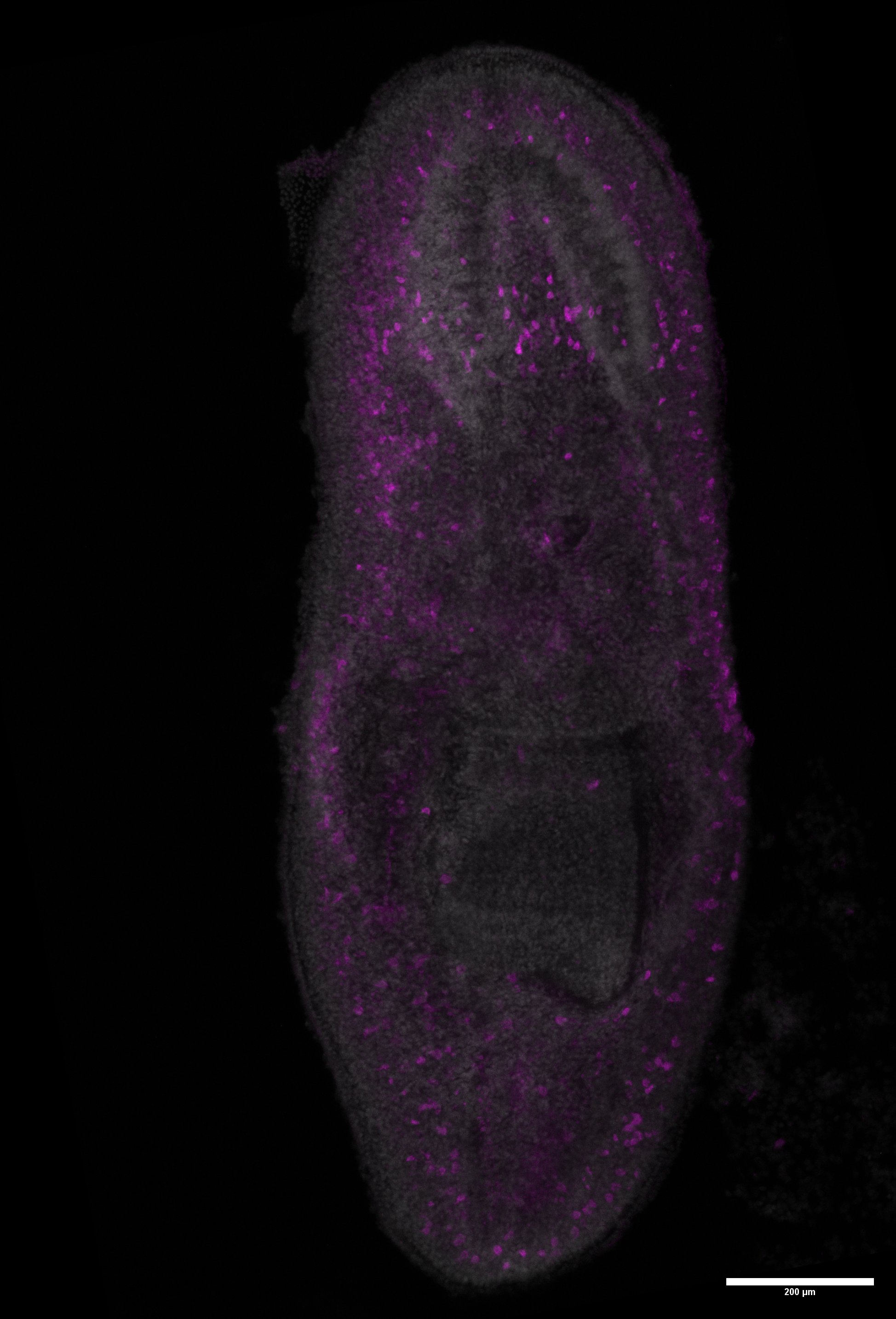

Supplement: Supplementary file 12 — Source data Fig. 5 [file 44318_2025_662_MOESM12_ESM.zip › Figure 5/5D/dd_3451/ID_8_Control_RNAi_Probe_dd3451_rhod_DAPI_10x.jpg]

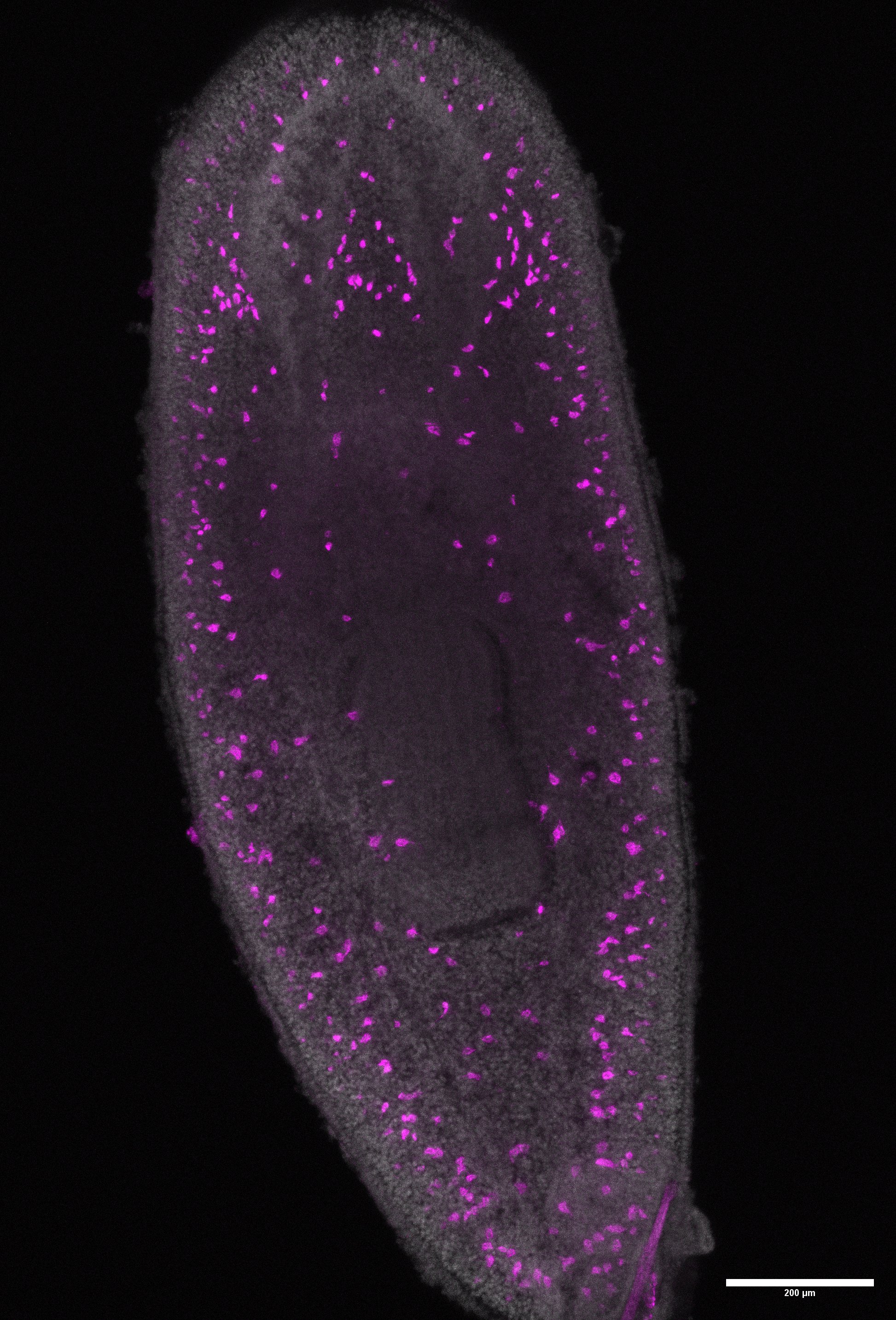

Supplement: Supplementary file 12 — Source data Fig. 5 [file 44318_2025_662_MOESM12_ESM.zip › Figure 5/5D/dd_3451/ID_8_X_ythdf-B_RNAi_Probe_dd3451_rhod_DAPI_10x.jpg]

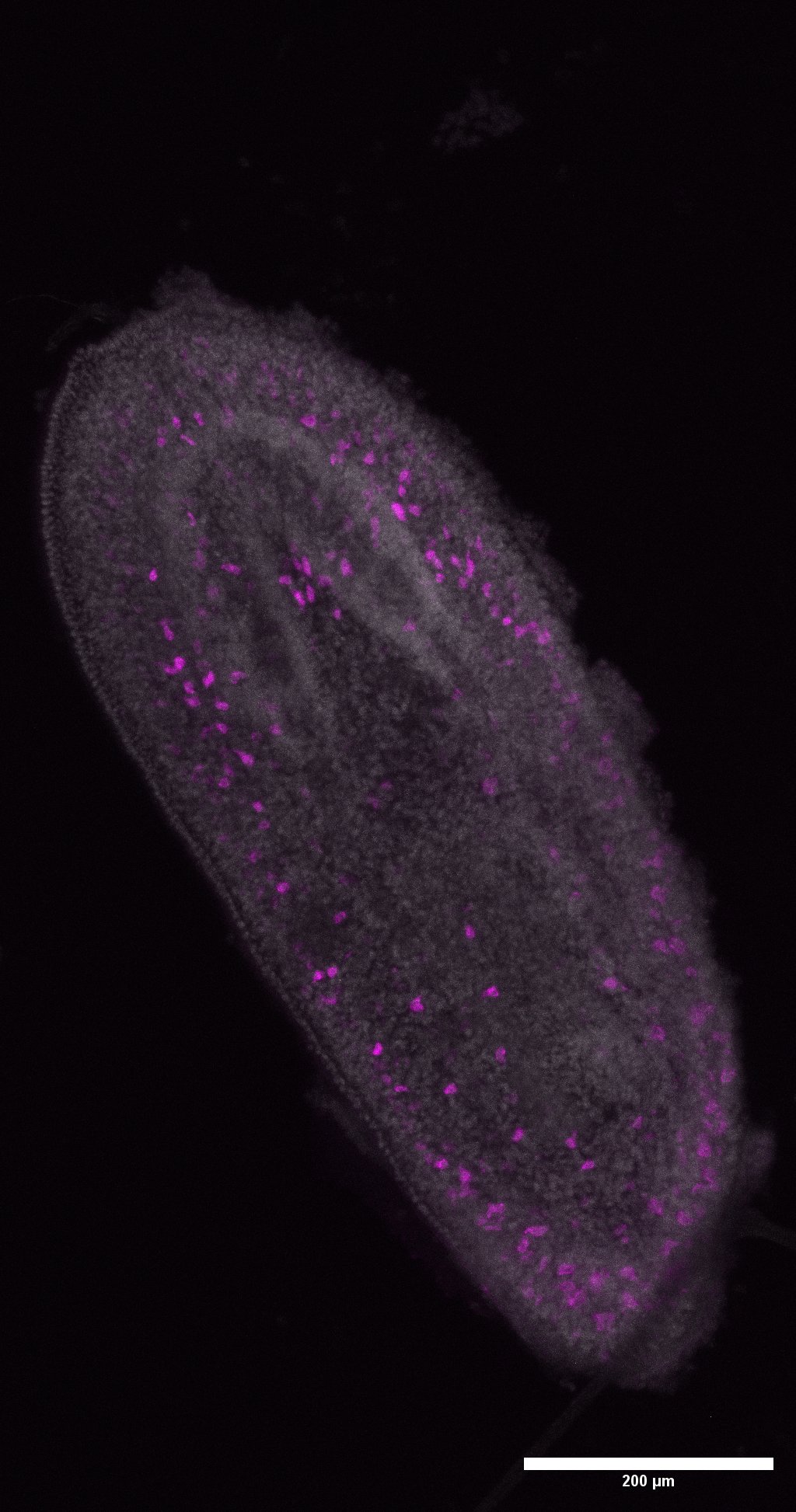

Supplement: Supplementary file 12 — Source data Fig. 5 [file 44318_2025_662_MOESM12_ESM.zip › Figure 5/5D/dd_3451/ID_8_ythdf-A_RNAi_Probe_dd3451_rhod_DAPI_10x.jpg]

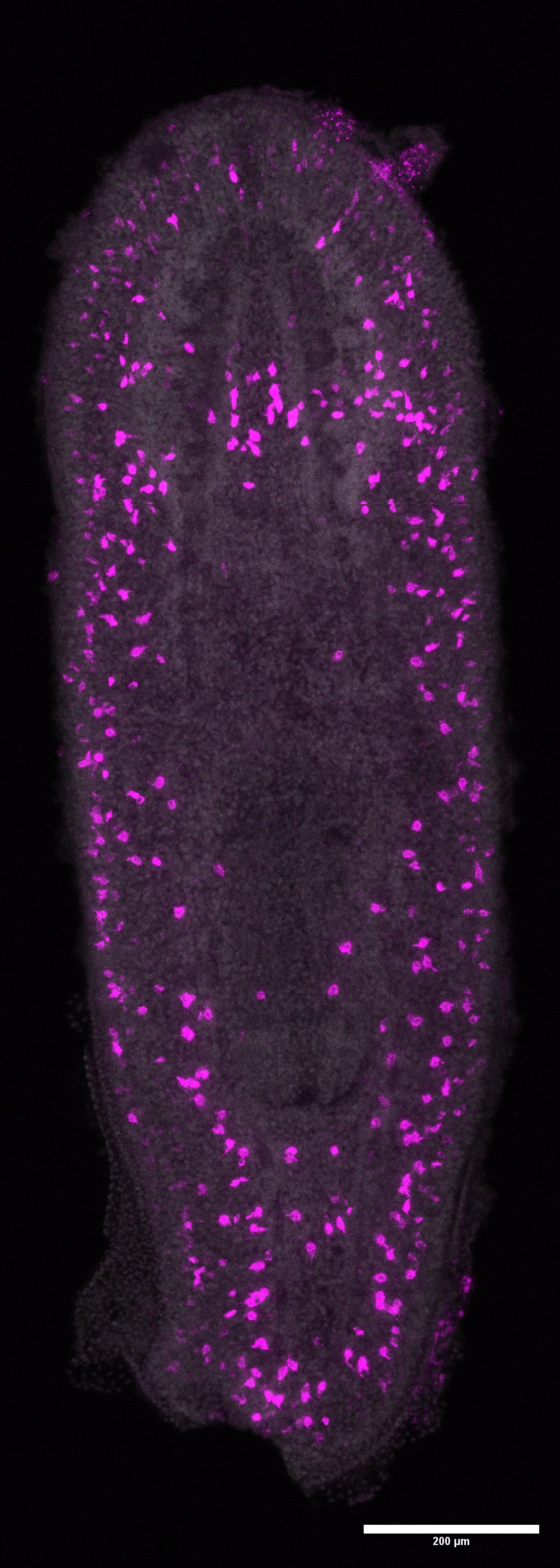

Supplement: Supplementary file 12 — Source data Fig. 5 [file 44318_2025_662_MOESM12_ESM.zip › Figure 5/5D/dd_3451/ID_8_ythdf-C_RNAi_Probe_dd3451_rhod_DAPI_10x.jpg]

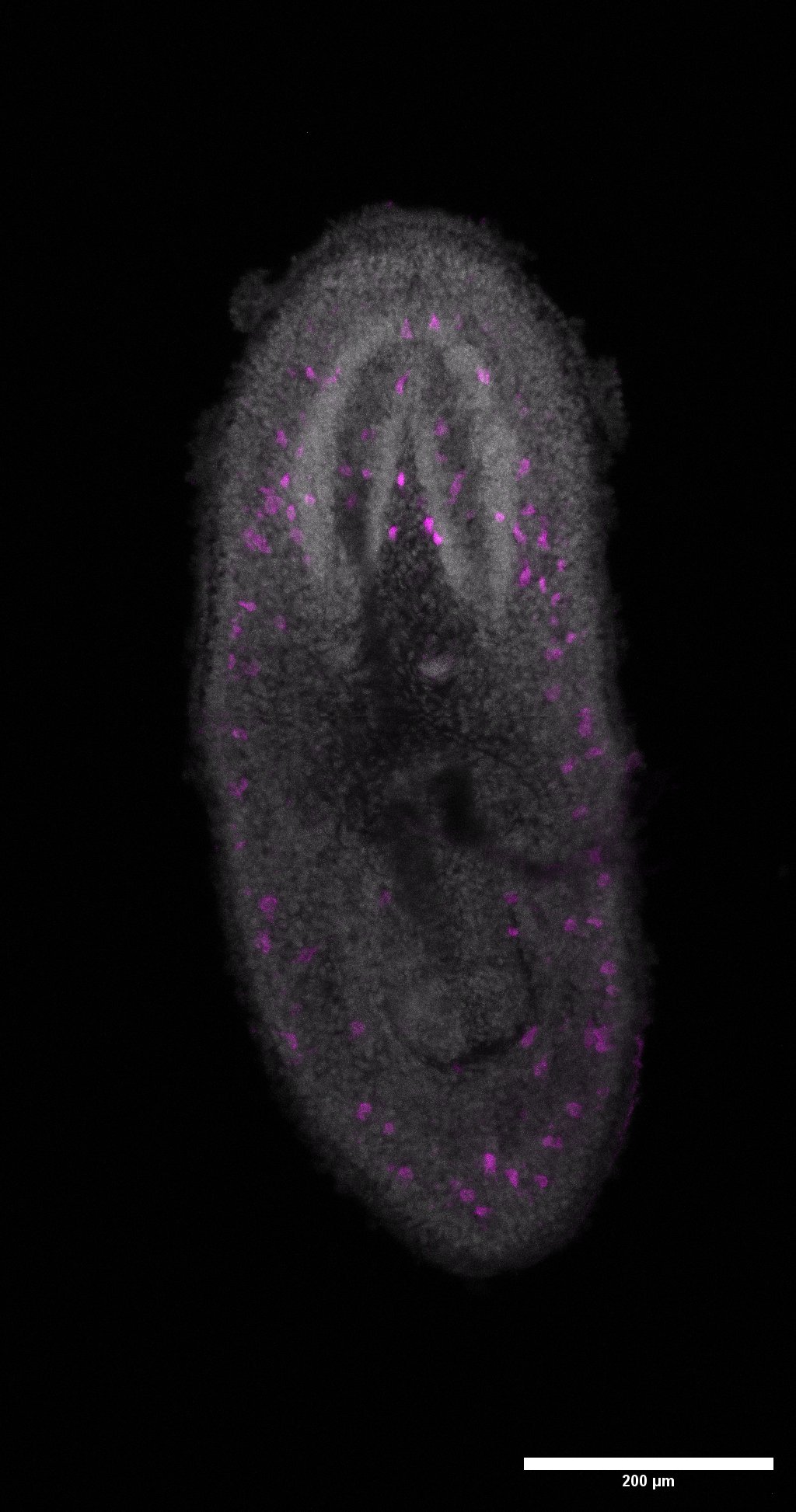

Supplement: Supplementary file 12 — Source data Fig. 5 [file 44318_2025_662_MOESM12_ESM.zip › Figure 5/5D/dd_3451/ID_9_Control_RNAi_Probe_dd3451_rhod_DAPI_10x.jpg]

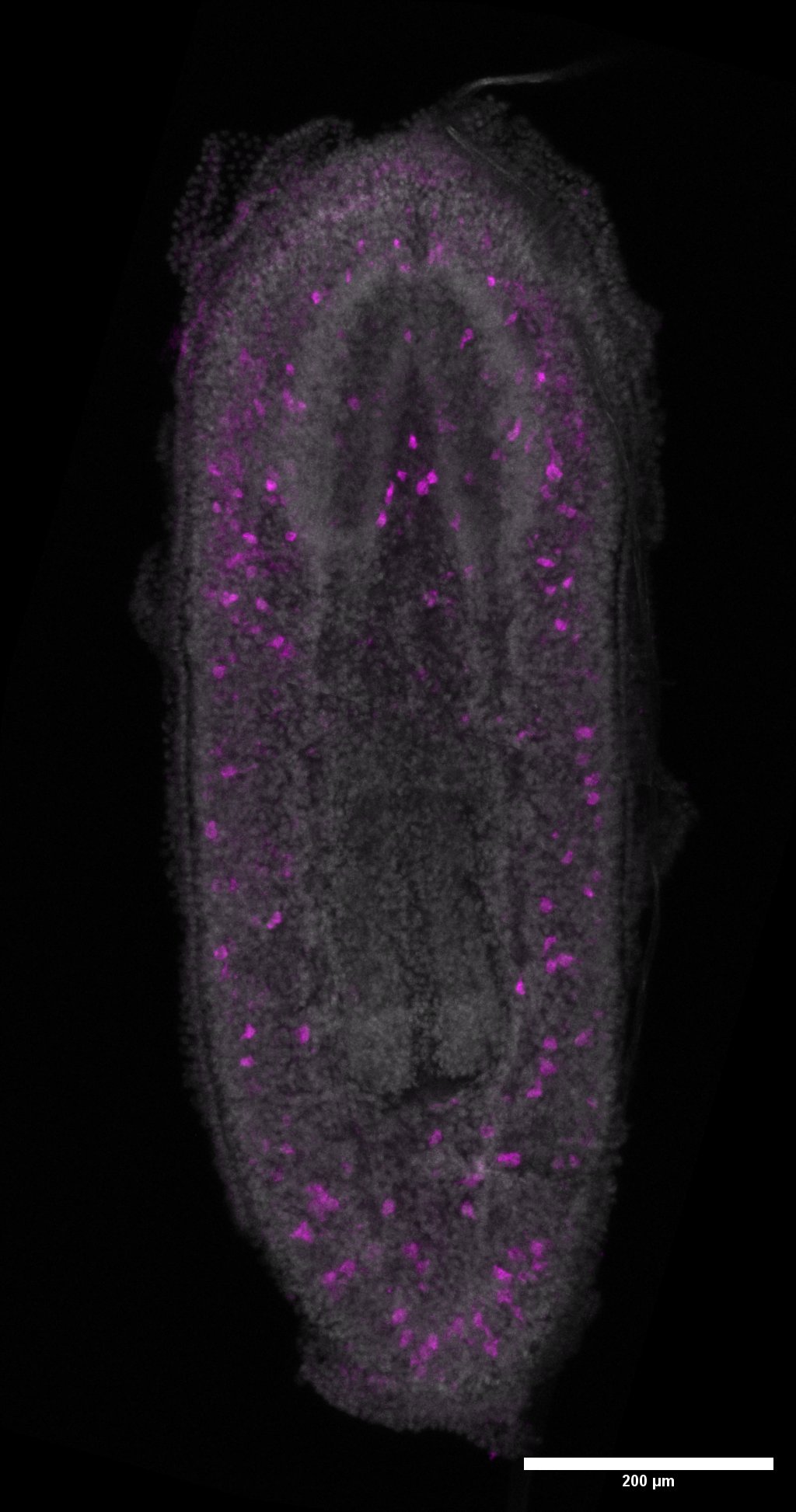

Supplement: Supplementary file 12 — Source data Fig. 5 [file 44318_2025_662_MOESM12_ESM.zip › Figure 5/5D/dd_3451/ID_9_Triple_RNAi_Probe_dd3451_rhod_DAPI_10x.jpg]

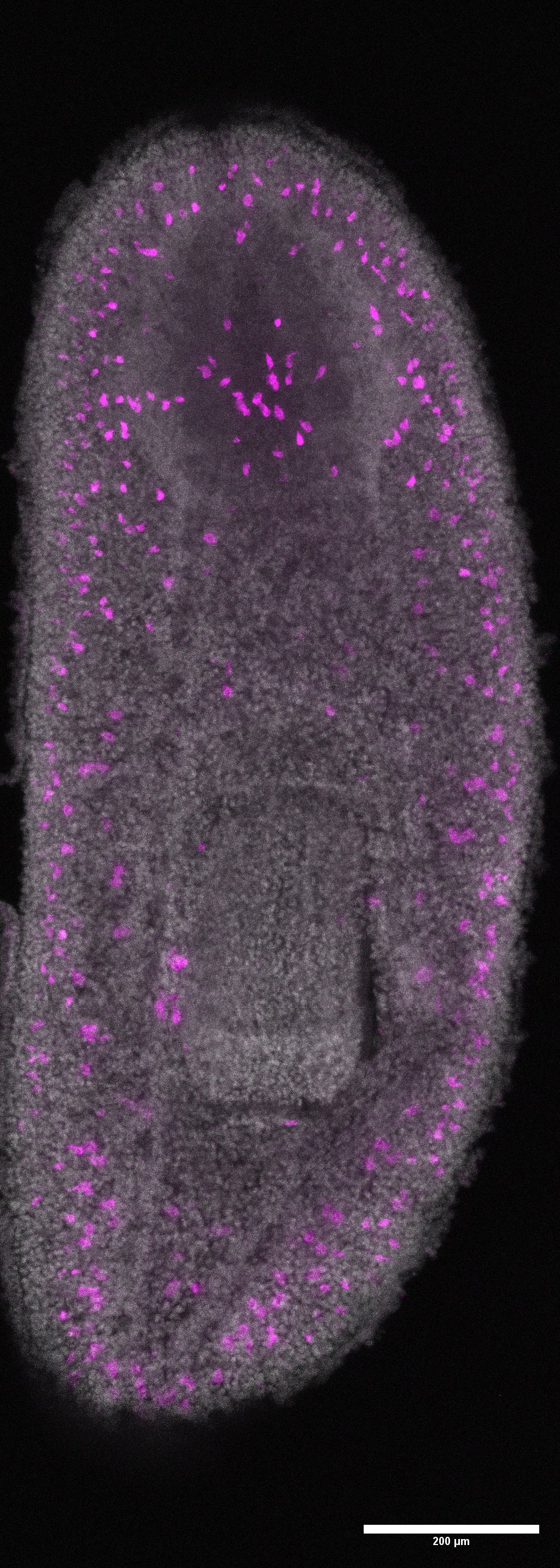

Supplement: Supplementary file 12 — Source data Fig. 5 [file 44318_2025_662_MOESM12_ESM.zip › Figure 5/5D/dd_3451/ID_9_X_ythdf-B_RNAi_Probe_dd3451_rhod_DAPI_10x.jpg]

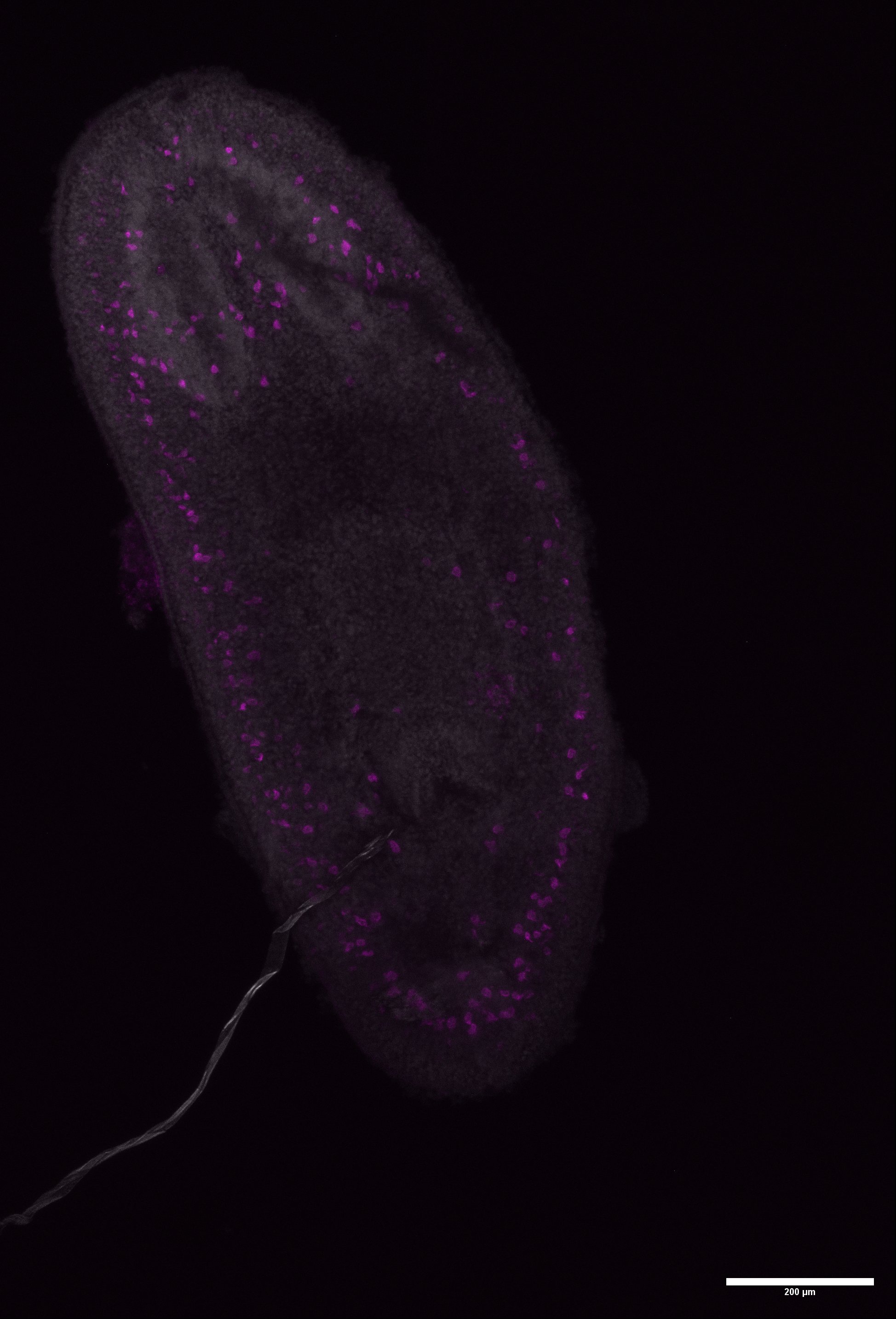

Supplement: Supplementary file 12 — Source data Fig. 5 [file 44318_2025_662_MOESM12_ESM.zip › Figure 5/5D/dd_3451/ID_9_ythdf-A_RNAi_Probe_dd3451_rhod_DAPI_10x.jpg]

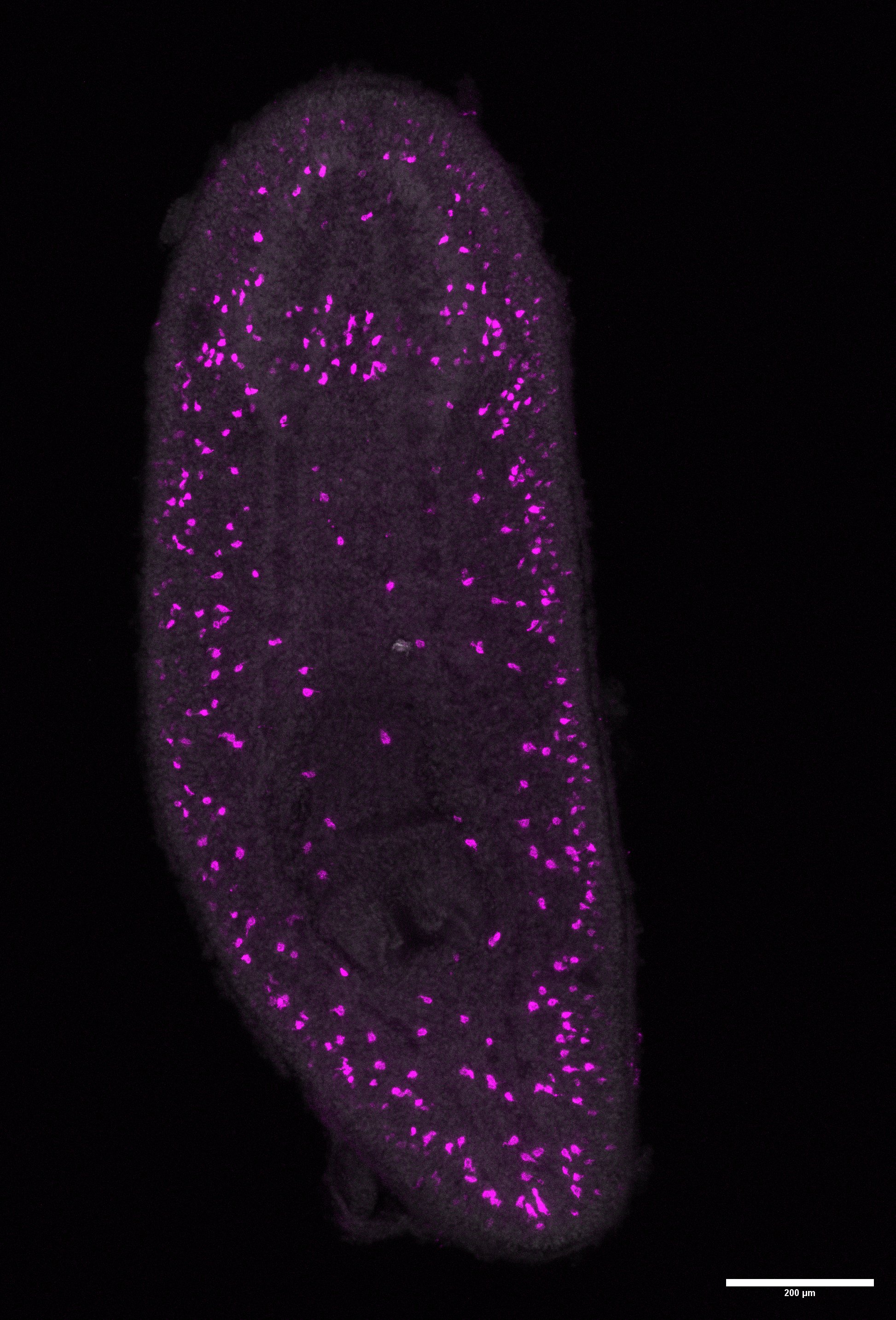

Supplement: Supplementary file 12 — Source data Fig. 5 [file 44318_2025_662_MOESM12_ESM.zip › Figure 5/5D/dd_3451/ID_9_ythdf-C_RNAi_Probe_dd3451_rhod_DAPI_10x.jpg]

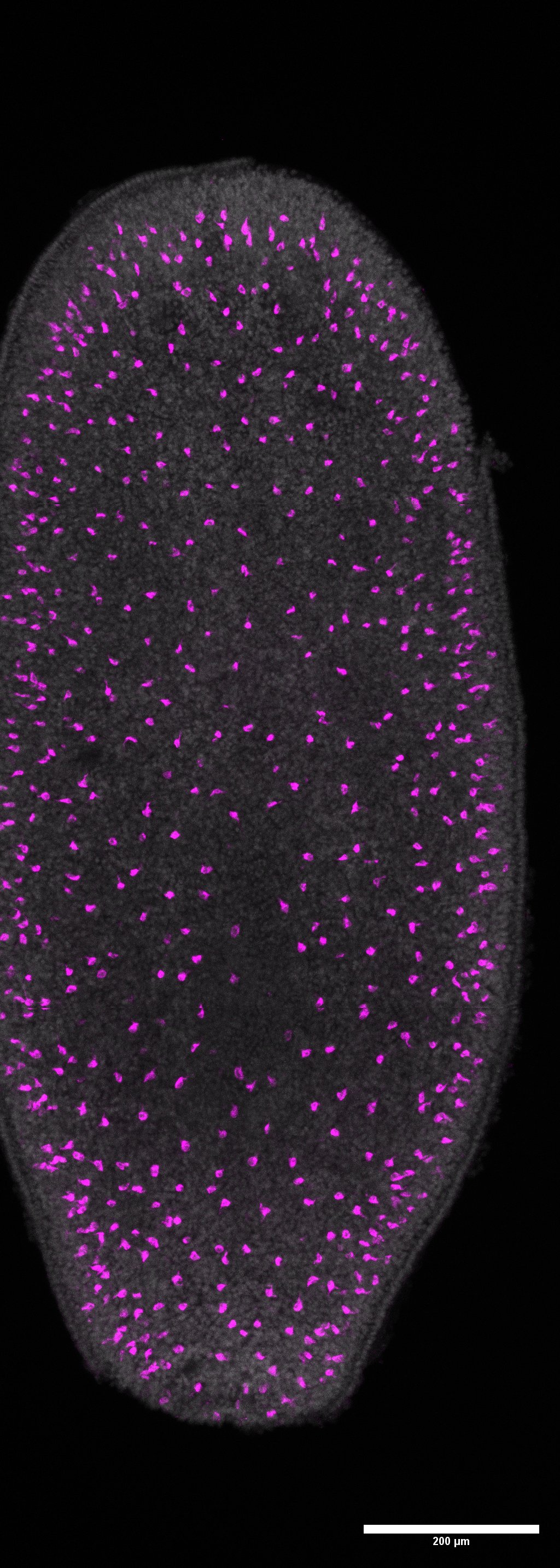

Supplement: Supplementary file 12 — Source data Fig. 5 [file 44318_2025_662_MOESM12_ESM.zip › Figure 5/5D/dd_356/ID_10_Control_RNAi_Probe_dd_356_rhod_DAPI_10x.jpg]

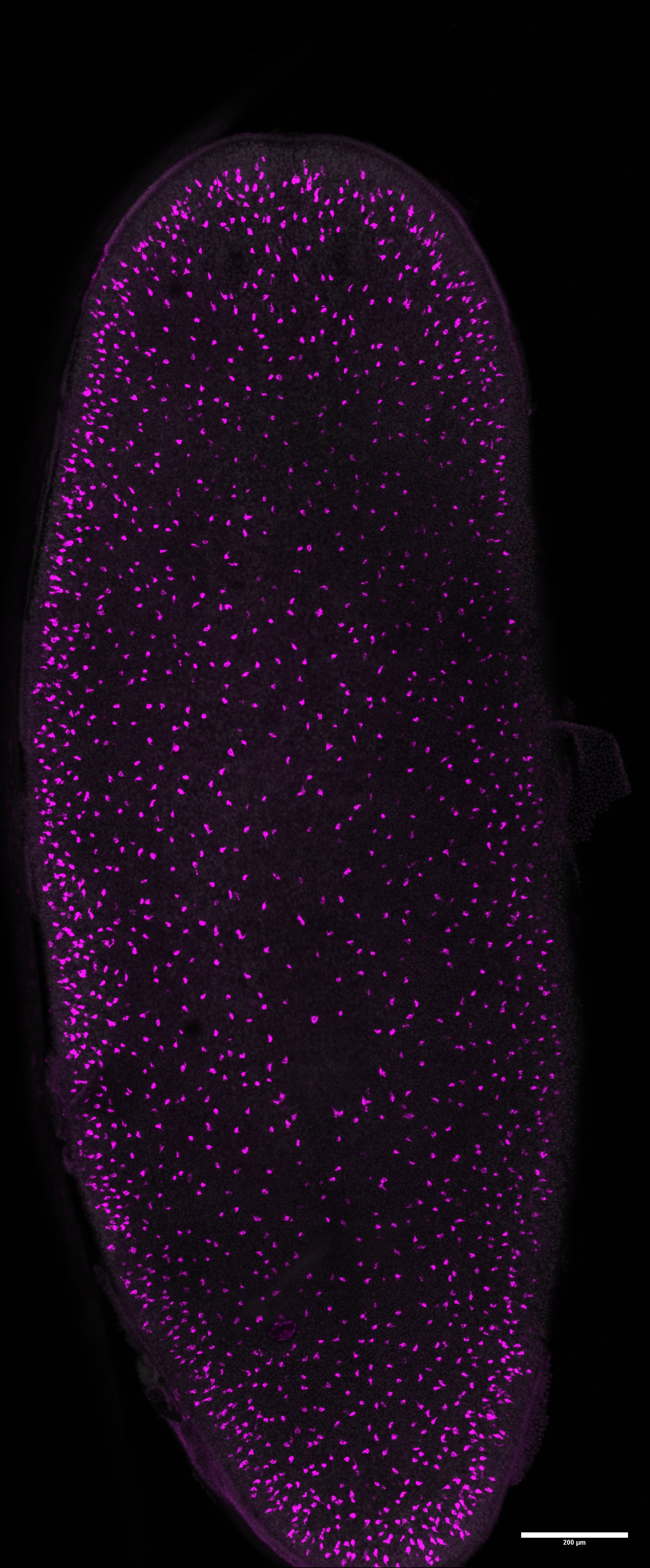

Supplement: Supplementary file 12 — Source data Fig. 5 [file 44318_2025_662_MOESM12_ESM.zip › Figure 5/5D/dd_356/ID_10_ythdf-C_RNAi_Probe_dd_356_rhod_DAPI_10x.jpg]

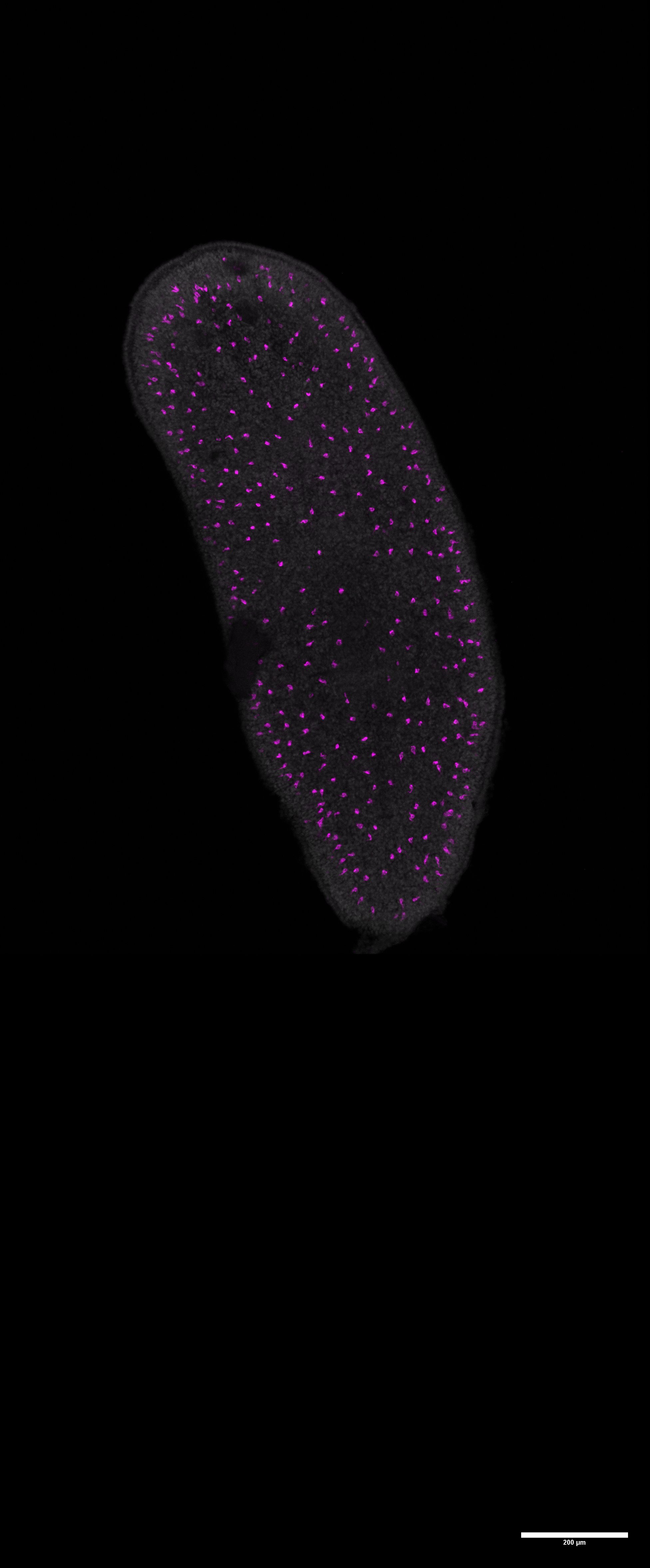

Supplement: Supplementary file 12 — Source data Fig. 5 [file 44318_2025_662_MOESM12_ESM.zip › Figure 5/5D/dd_356/ID_11_Control_RNAi_Probe_dd_356_rhod_DAPI_10x.jpg]

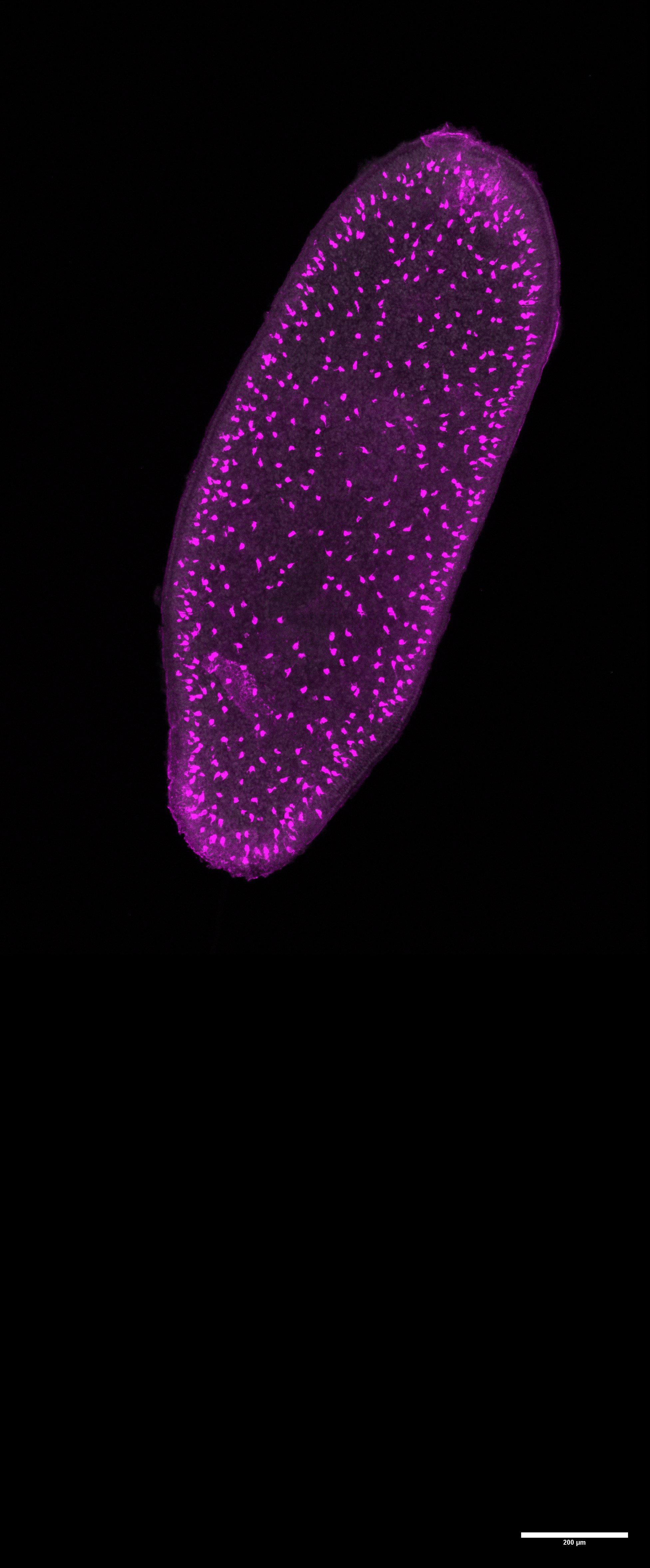

Supplement: Supplementary file 12 — Source data Fig. 5 [file 44318_2025_662_MOESM12_ESM.zip › Figure 5/5D/dd_356/ID_11_ythdf-A_RNAi_Probe_dd_356_rhod_DAPI_10x.jpg]

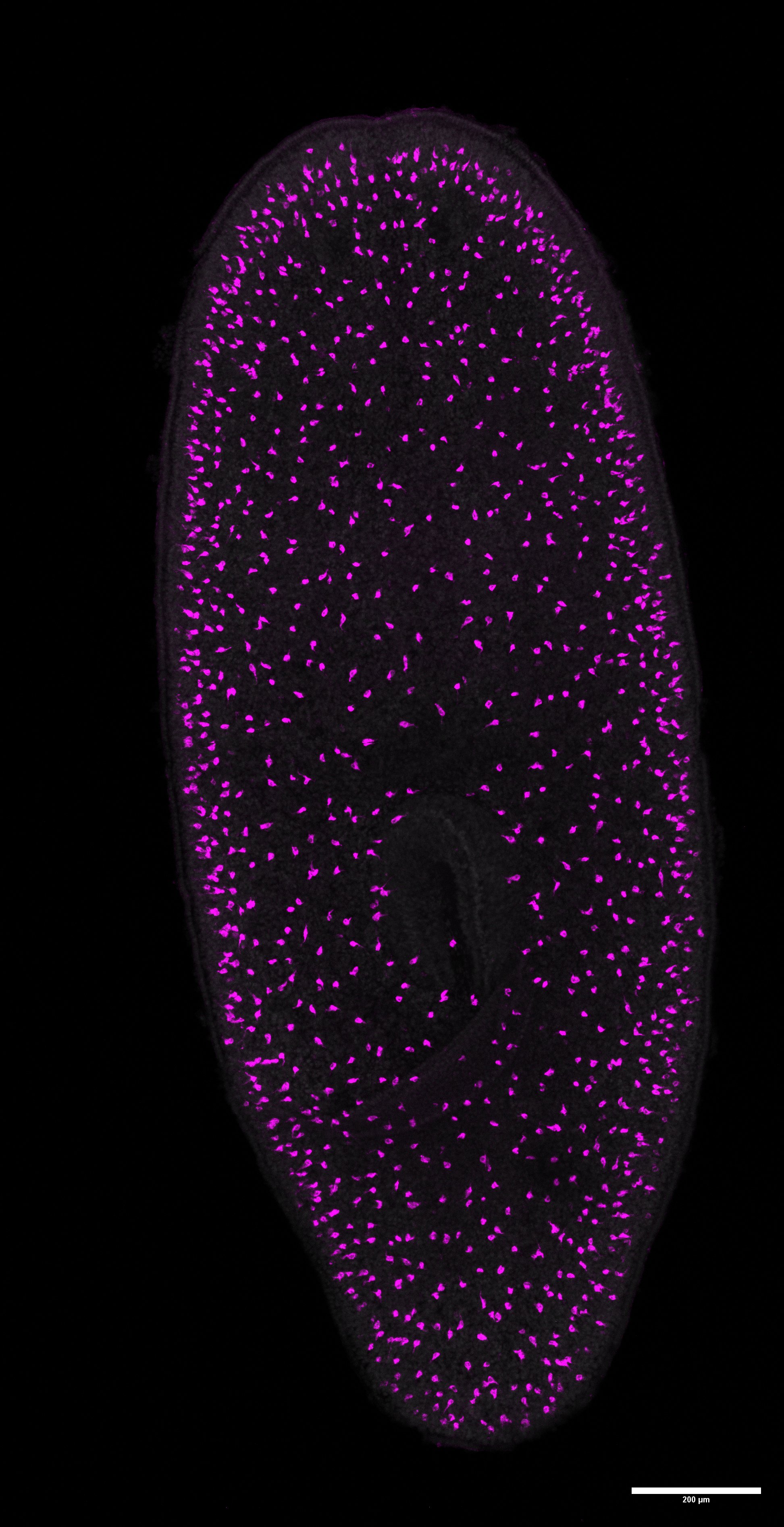

Supplement: Supplementary file 12 — Source data Fig. 5 [file 44318_2025_662_MOESM12_ESM.zip › Figure 5/5D/dd_356/ID_1_Control_RNAi_Probe_dd_356_rhod_DAPI_10x.jpg]

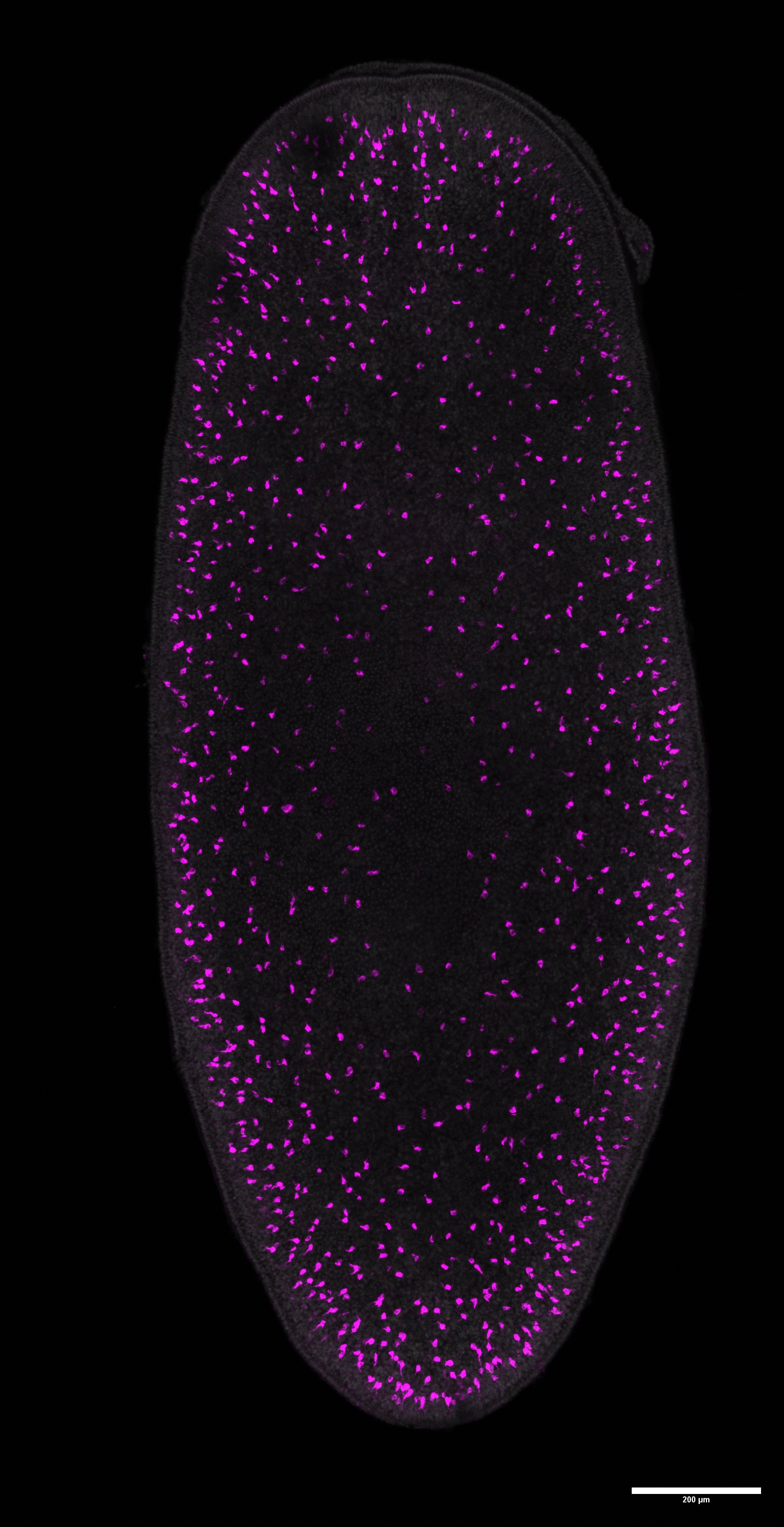

Supplement: Supplementary file 12 — Source data Fig. 5 [file 44318_2025_662_MOESM12_ESM.zip › Figure 5/5D/dd_356/ID_1_Triple_RNAi_Probe_dd_356_rhod_DAPI_10x.jpg]

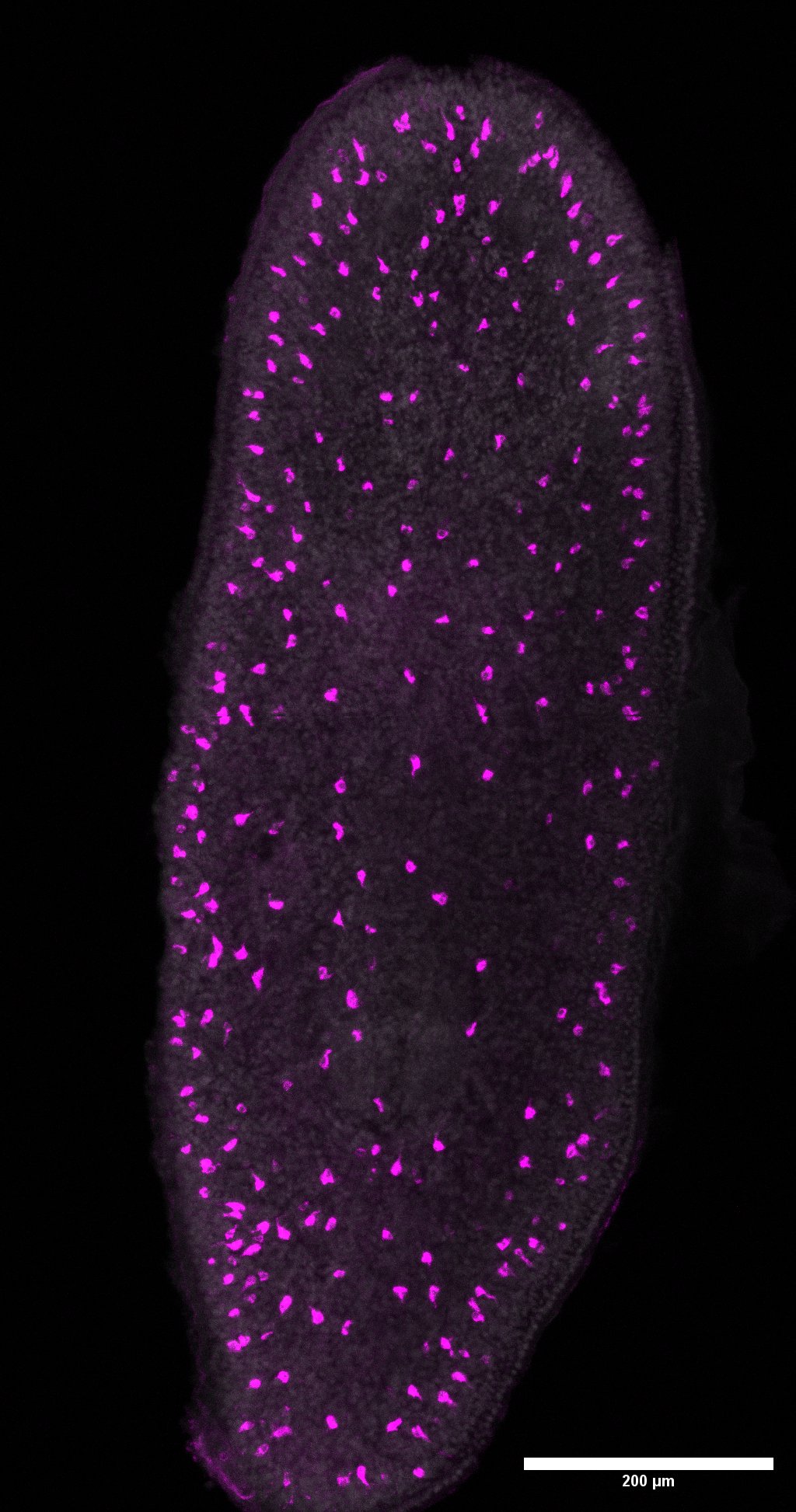

Supplement: Supplementary file 12 — Source data Fig. 5 [file 44318_2025_662_MOESM12_ESM.zip › Figure 5/5D/dd_356/ID_1_ythdf-A_RNAi_Probe_dd_356_rhod_DAPI_10x.jpg]

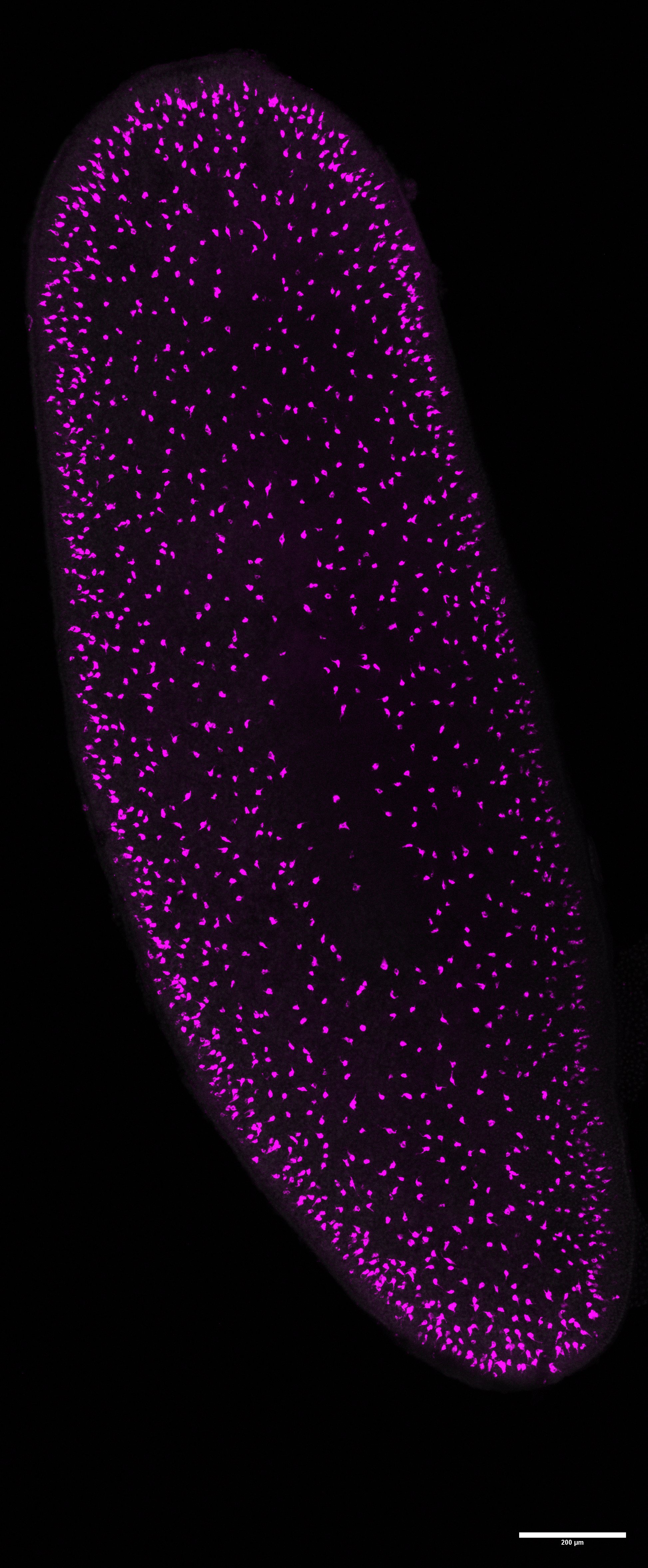

Supplement: Supplementary file 12 — Source data Fig. 5 [file 44318_2025_662_MOESM12_ESM.zip › Figure 5/5D/dd_356/ID_1_ythdf-B_RNAi_Probe_dd_356_rhod_DAPI_10x.jpg]

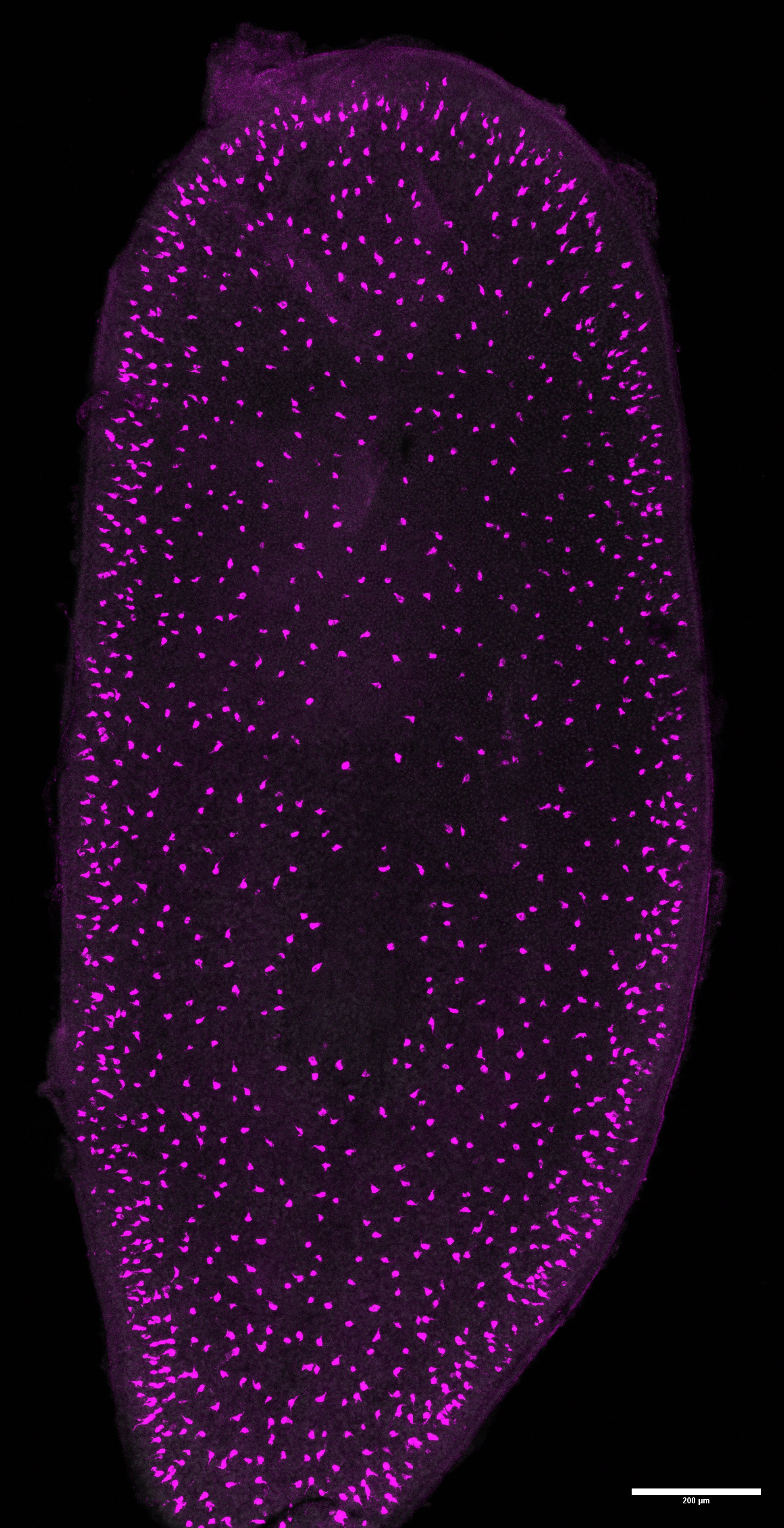

Supplement: Supplementary file 12 — Source data Fig. 5 [file 44318_2025_662_MOESM12_ESM.zip › Figure 5/5D/dd_356/ID_1_ythdf-C_RNAi_Probe_dd_356_rhod_DAPI_10x.jpg]

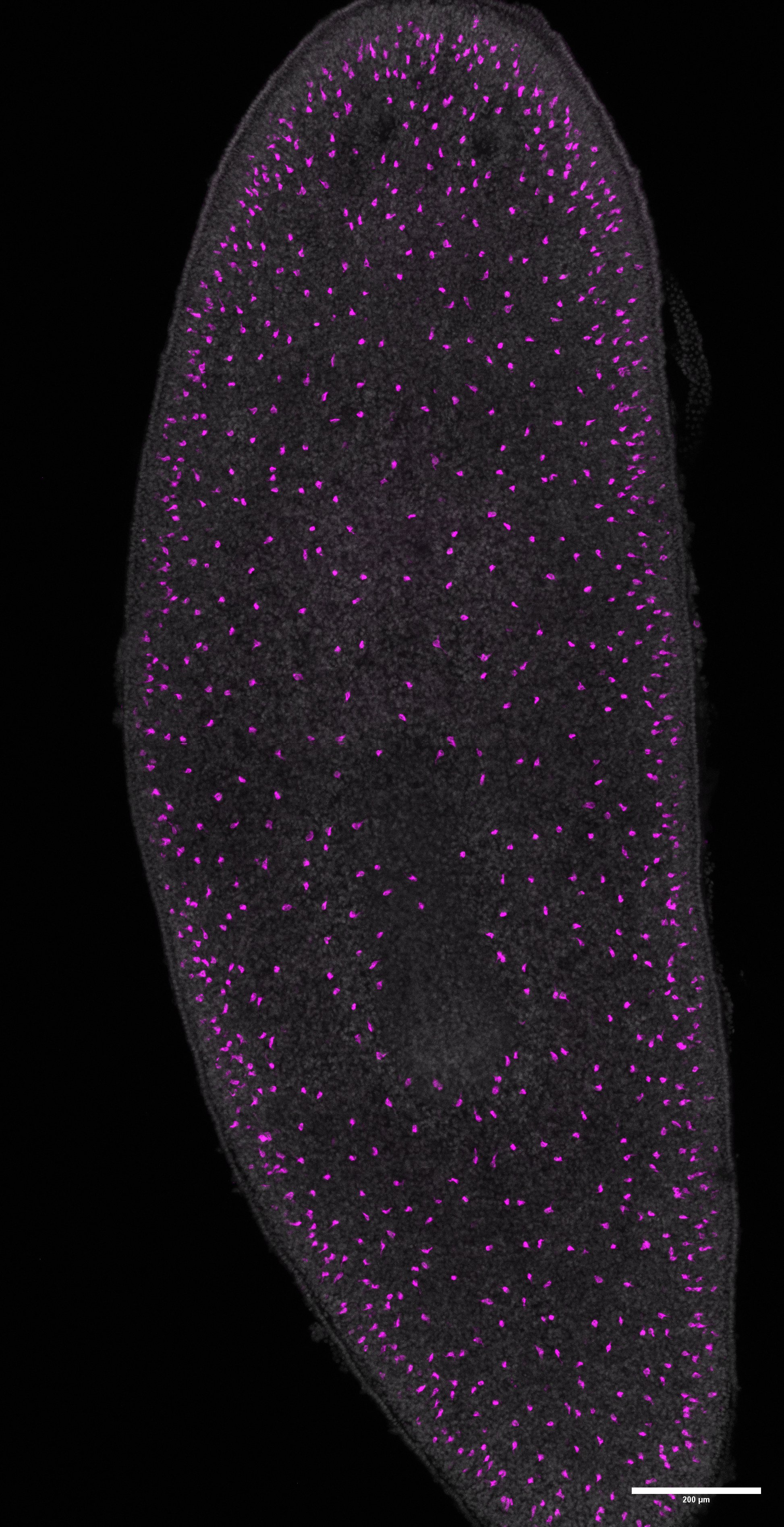

Supplement: Supplementary file 12 — Source data Fig. 5 [file 44318_2025_662_MOESM12_ESM.zip › Figure 5/5D/dd_356/ID_2_Control_RNAi_Probe_dd_356_rhod_DAPI_10x.jpg]

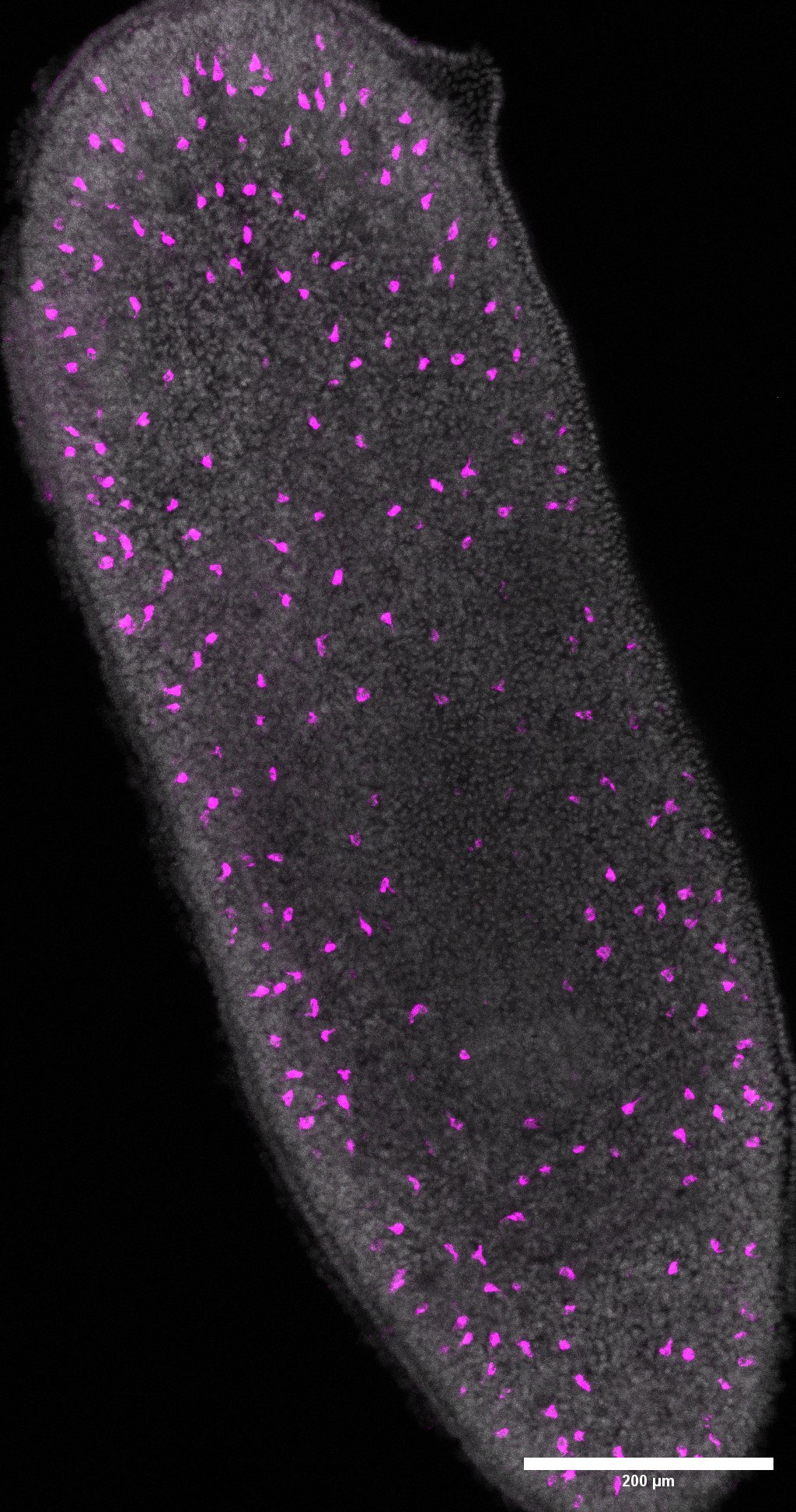

Supplement: Supplementary file 12 — Source data Fig. 5 [file 44318_2025_662_MOESM12_ESM.zip › Figure 5/5D/dd_356/ID_2_Triple_RNAi_Probe_dd_356_rhod_DAPI_10x.jpg]

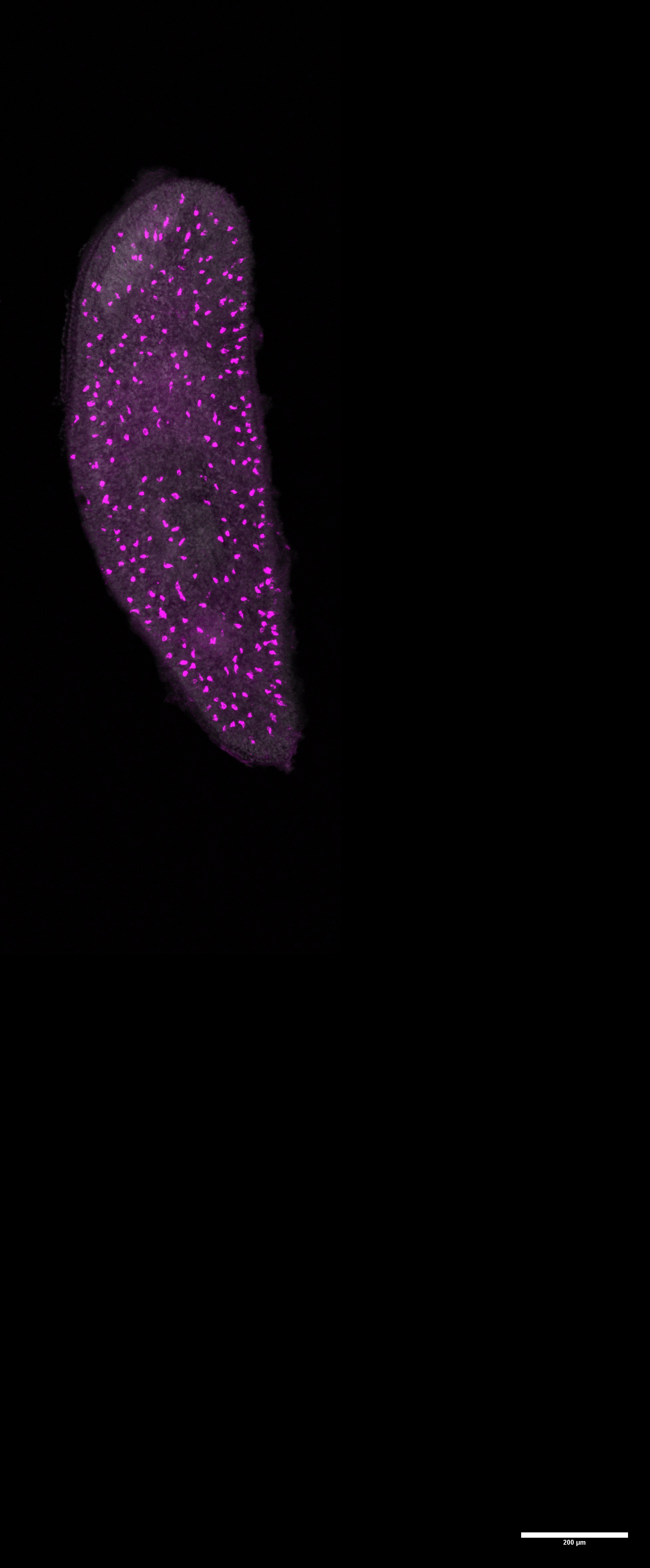

Supplement: Supplementary file 12 — Source data Fig. 5 [file 44318_2025_662_MOESM12_ESM.zip › Figure 5/5D/dd_356/ID_2_ythdf-A_RNAi_Probe_dd_356_rhod_DAPI_10x.jpg]

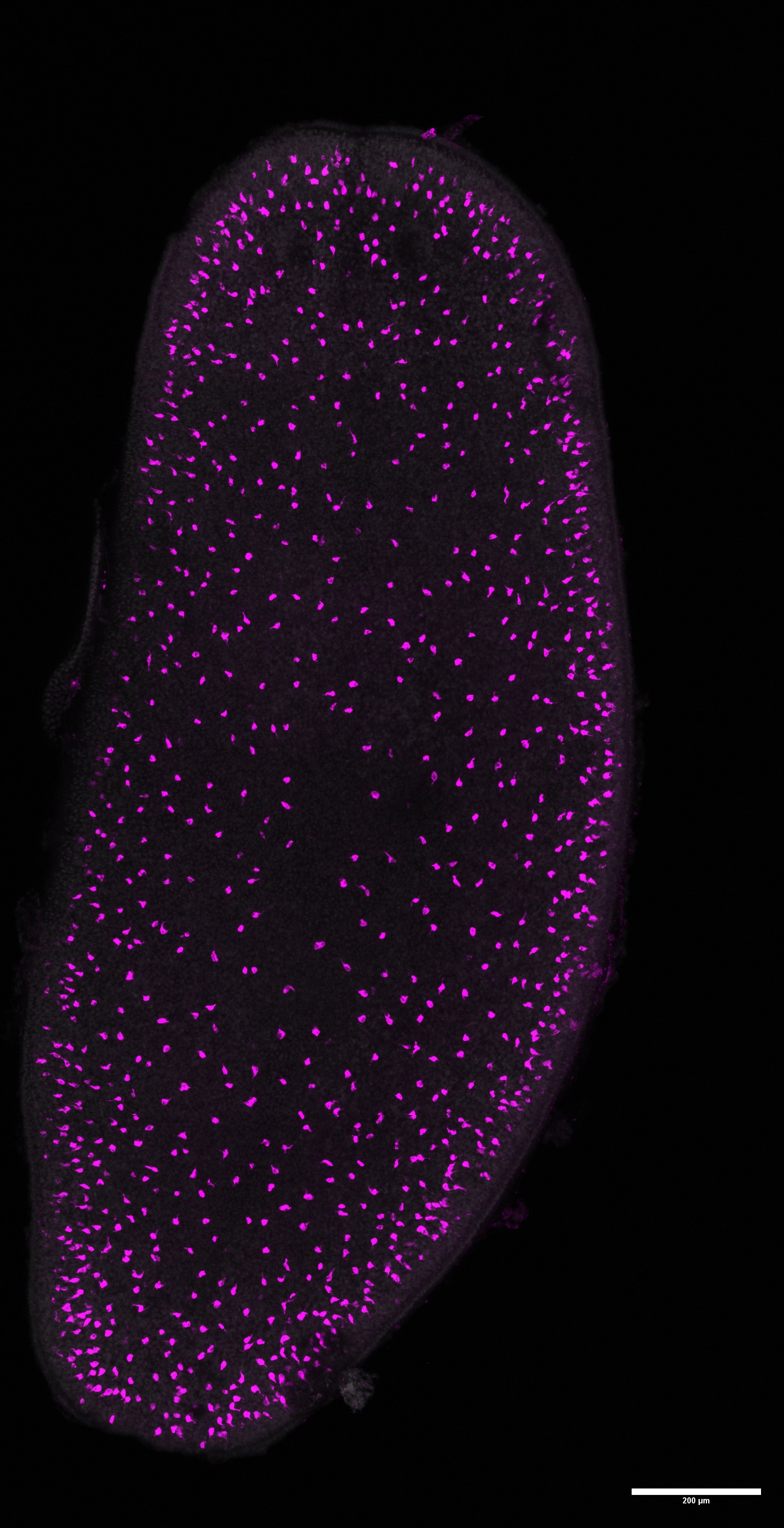

Supplement: Supplementary file 12 — Source data Fig. 5 [file 44318_2025_662_MOESM12_ESM.zip › Figure 5/5D/dd_356/ID_2_ythdf-B_RNAi_Probe_dd_356_rhod_DAPI_10x.jpg]

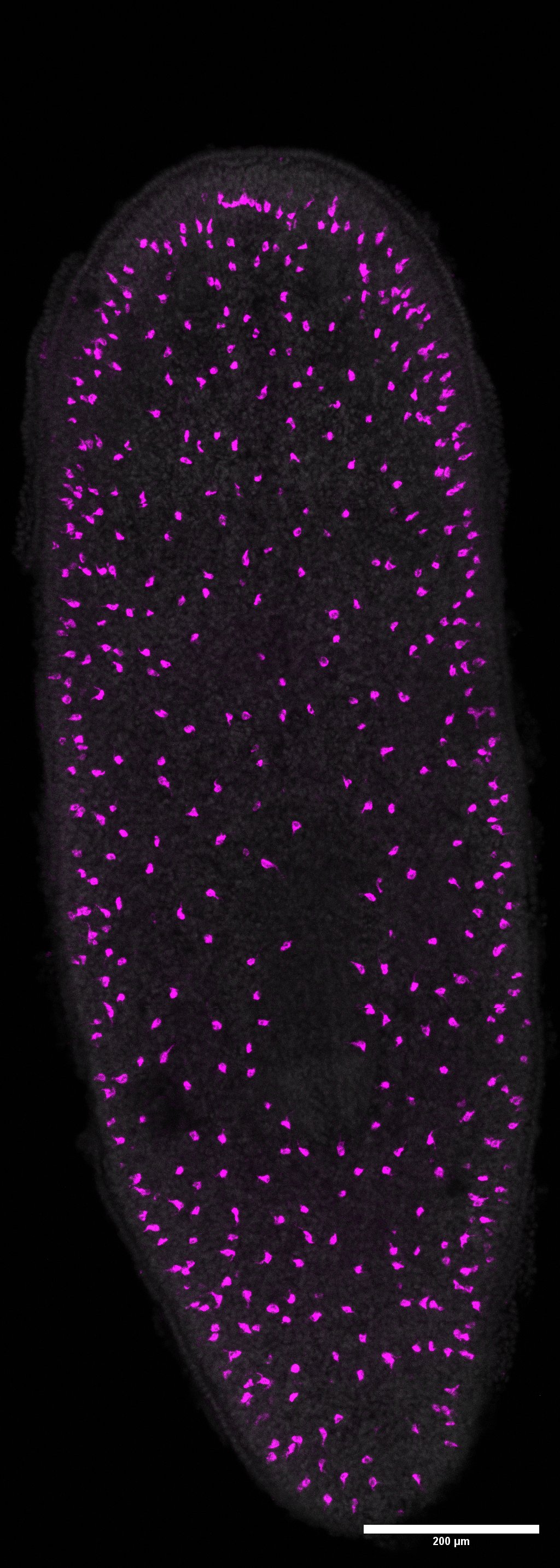

Supplement: Supplementary file 12 — Source data Fig. 5 [file 44318_2025_662_MOESM12_ESM.zip › Figure 5/5D/dd_356/ID_3_Control_RNAi_Probe_dd_356_rhod_DAPI_10x.jpg]

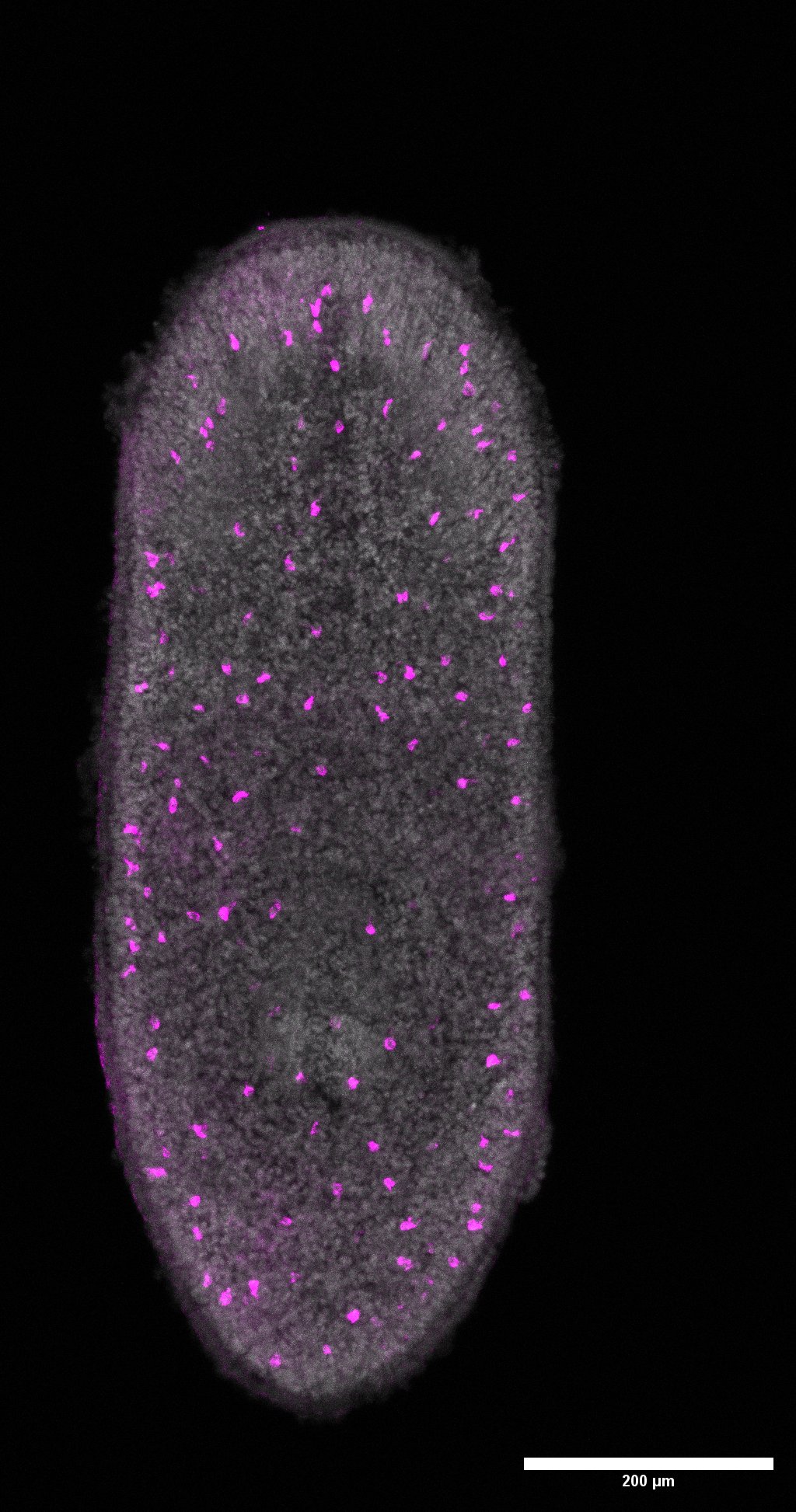

Supplement: Supplementary file 12 — Source data Fig. 5 [file 44318_2025_662_MOESM12_ESM.zip › Figure 5/5D/dd_356/ID_3_Triple_RNAi_Probe_dd_356_rhod_DAPI_10x.jpg]

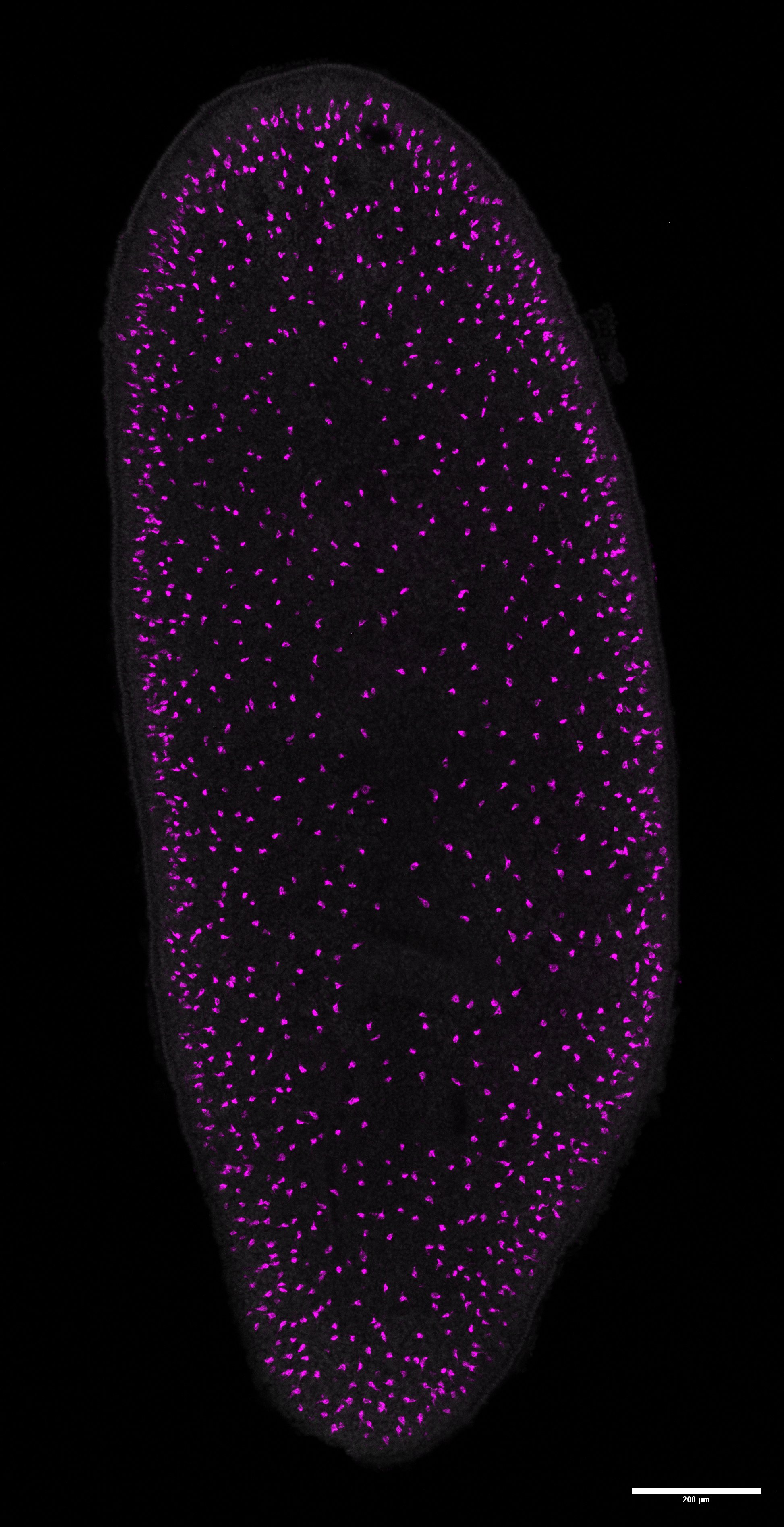

Supplement: Supplementary file 12 — Source data Fig. 5 [file 44318_2025_662_MOESM12_ESM.zip › Figure 5/5D/dd_356/ID_4_Control_RNAi_Probe_dd_356_rhod_DAPI_10x.jpg]

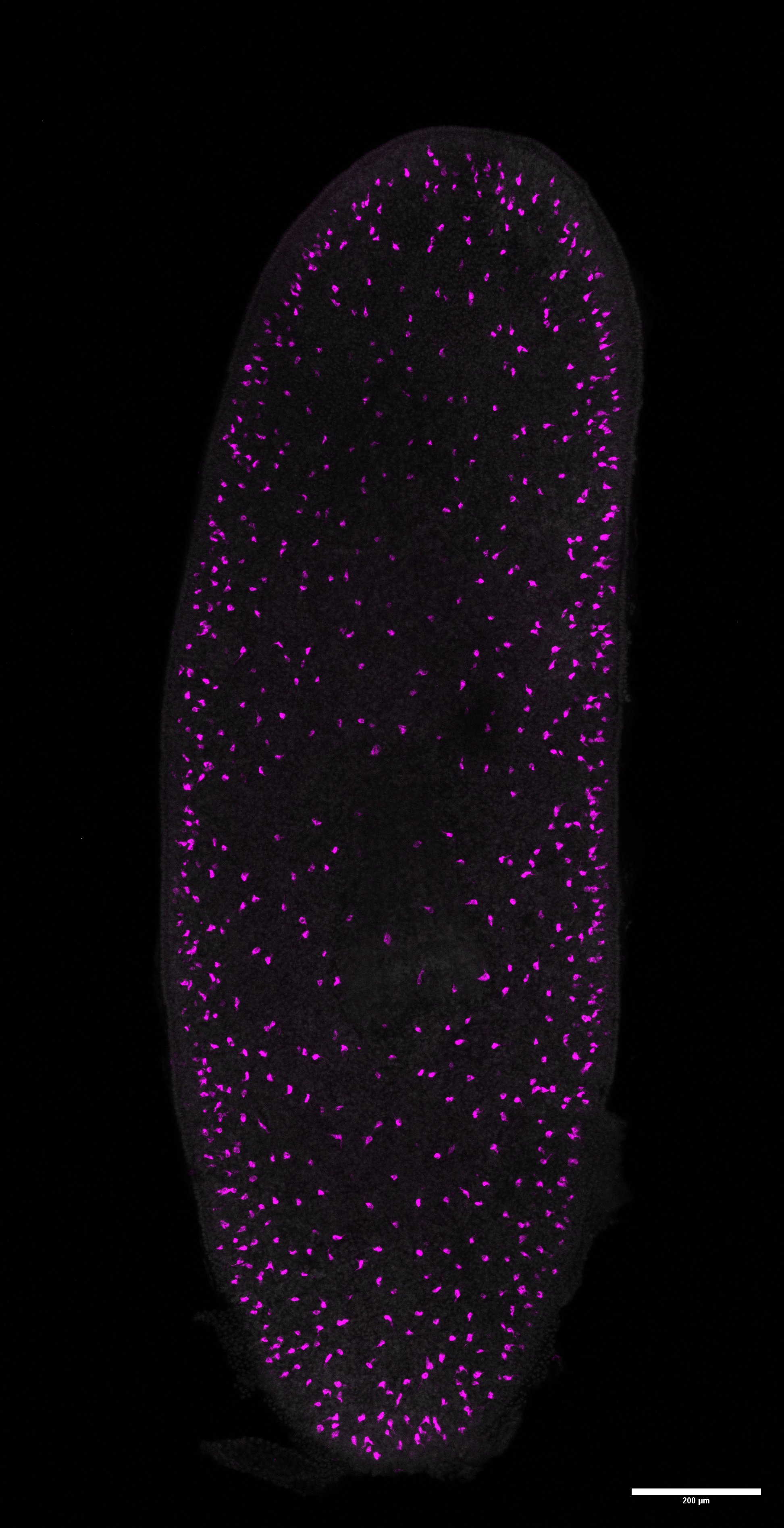

Supplement: Supplementary file 12 — Source data Fig. 5 [file 44318_2025_662_MOESM12_ESM.zip › Figure 5/5D/dd_356/ID_4_Triple_RNAi_Probe_dd_356_rhod_DAPI_10x.jpg]

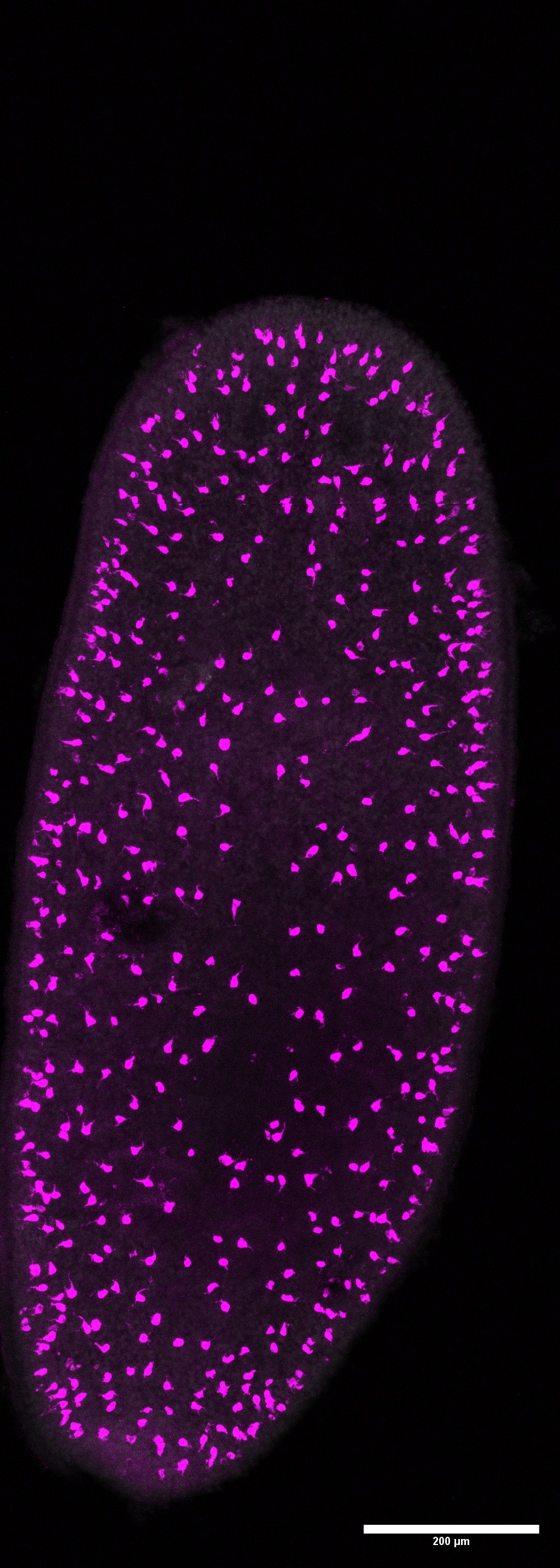

Supplement: Supplementary file 12 — Source data Fig. 5 [file 44318_2025_662_MOESM12_ESM.zip › Figure 5/5D/dd_356/ID_4_ythdf-B_RNAi_Probe_dd_356_rhod_DAPI_10x.jpg]

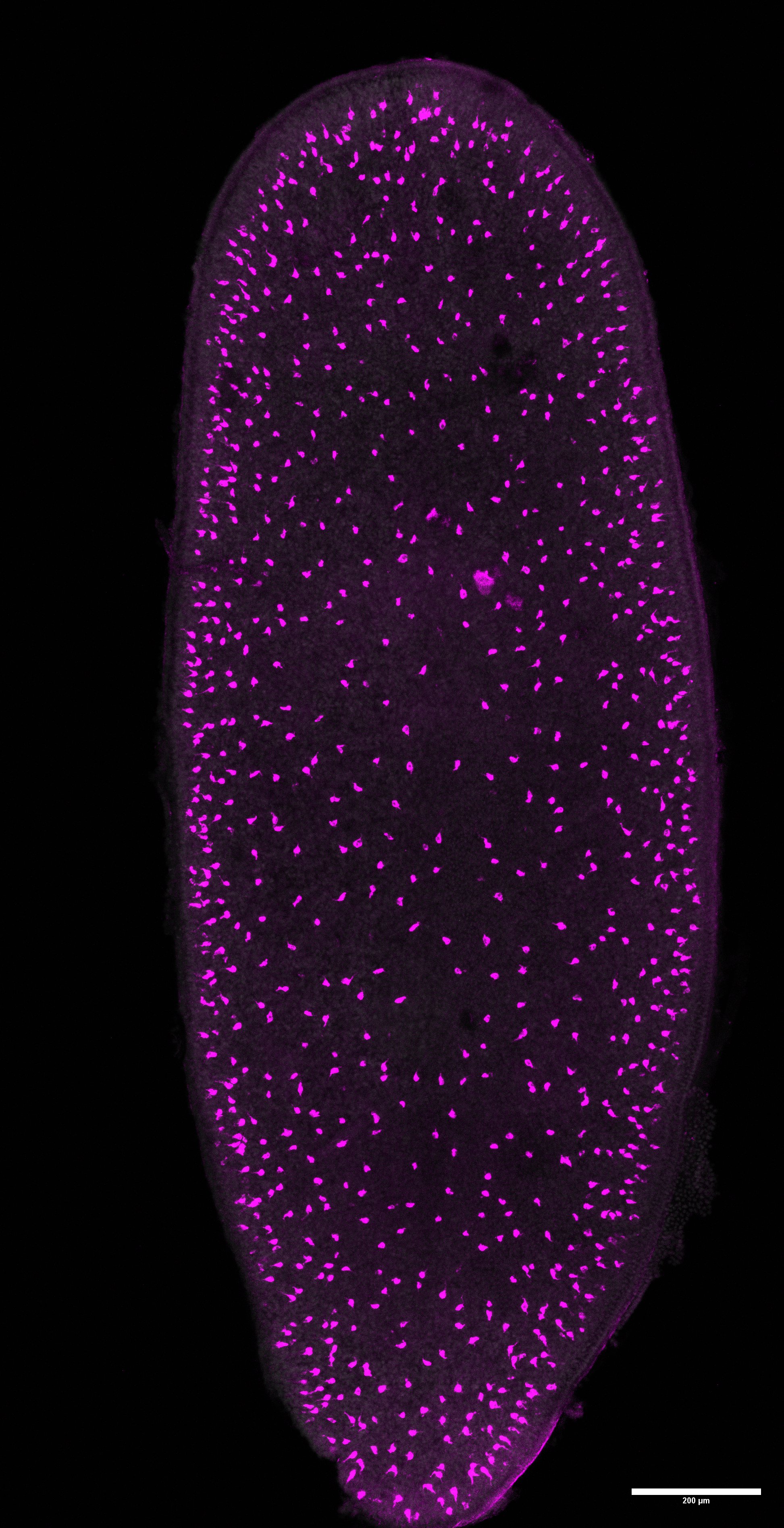

Supplement: Supplementary file 12 — Source data Fig. 5 [file 44318_2025_662_MOESM12_ESM.zip › Figure 5/5D/dd_356/ID_4_ythdf-C_RNAi_Probe_dd_356_rhod_DAPI_10x.jpg]

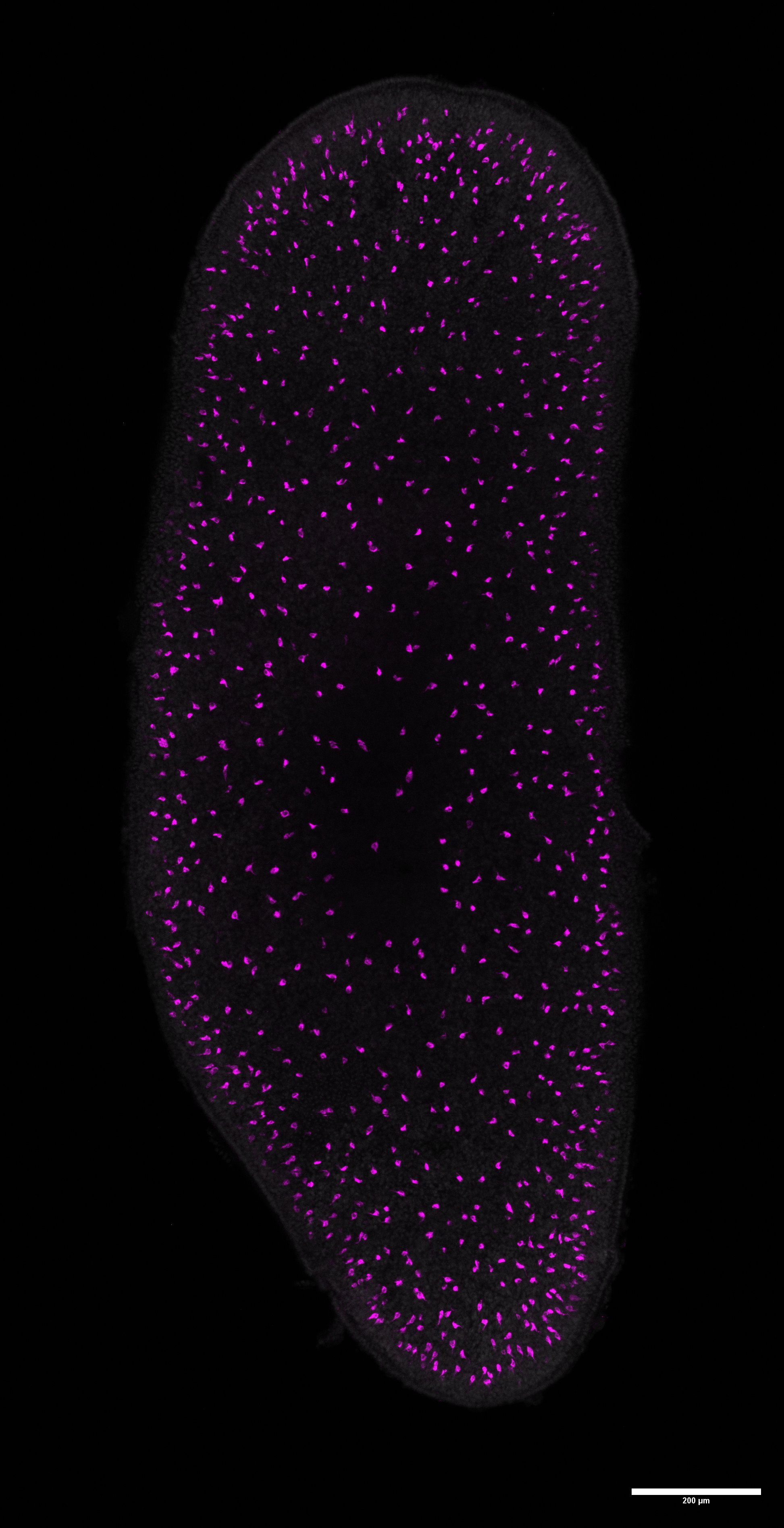

Supplement: Supplementary file 12 — Source data Fig. 5 [file 44318_2025_662_MOESM12_ESM.zip › Figure 5/5D/dd_356/ID_5_Control_RNAi_Probe_dd_356_rhod_DAPI_10x.jpg]

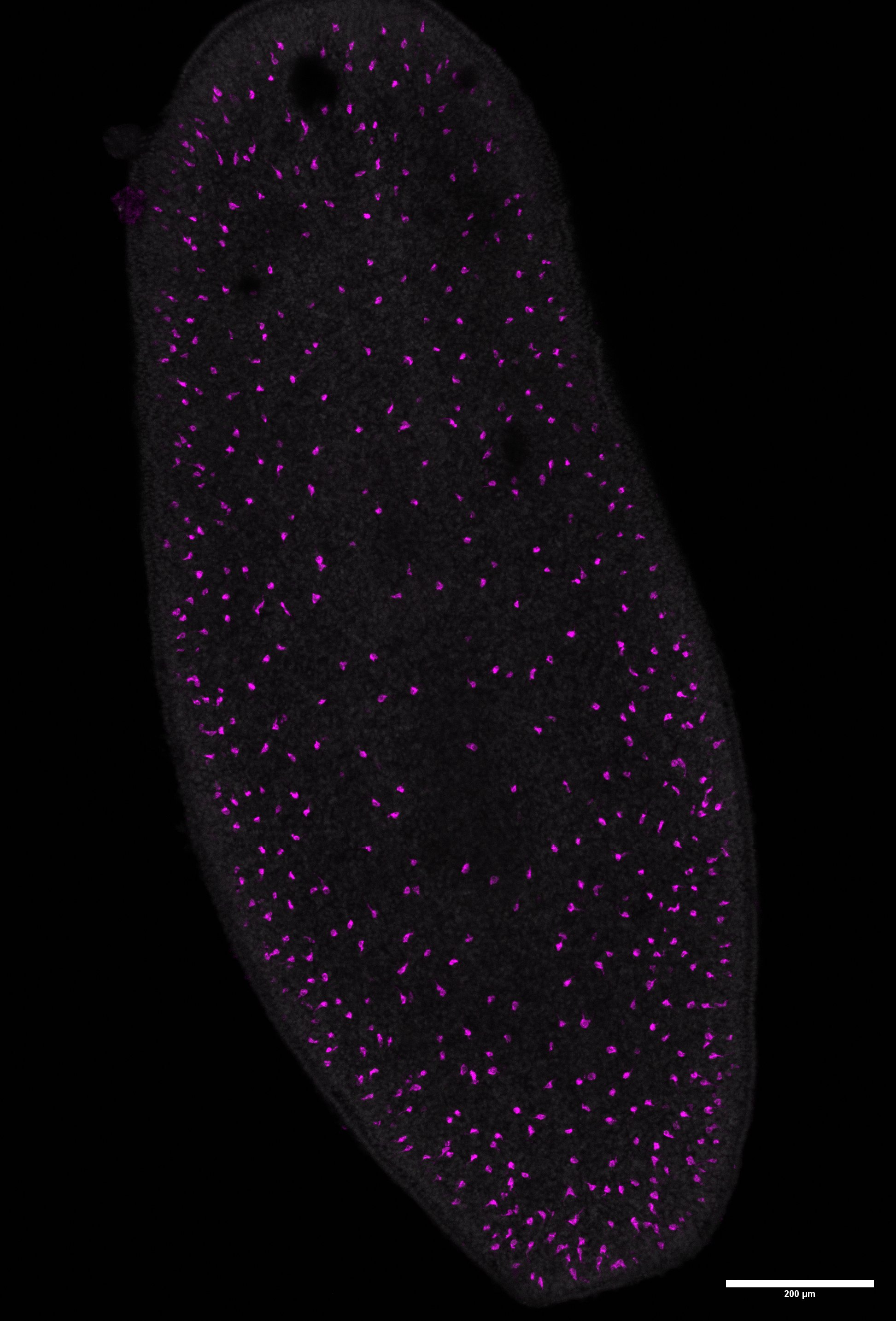

Supplement: Supplementary file 12 — Source data Fig. 5 [file 44318_2025_662_MOESM12_ESM.zip › Figure 5/5D/dd_356/ID_5_Triple_RNAi_Probe_dd_356_rhod_DAPI_10x.jpg]

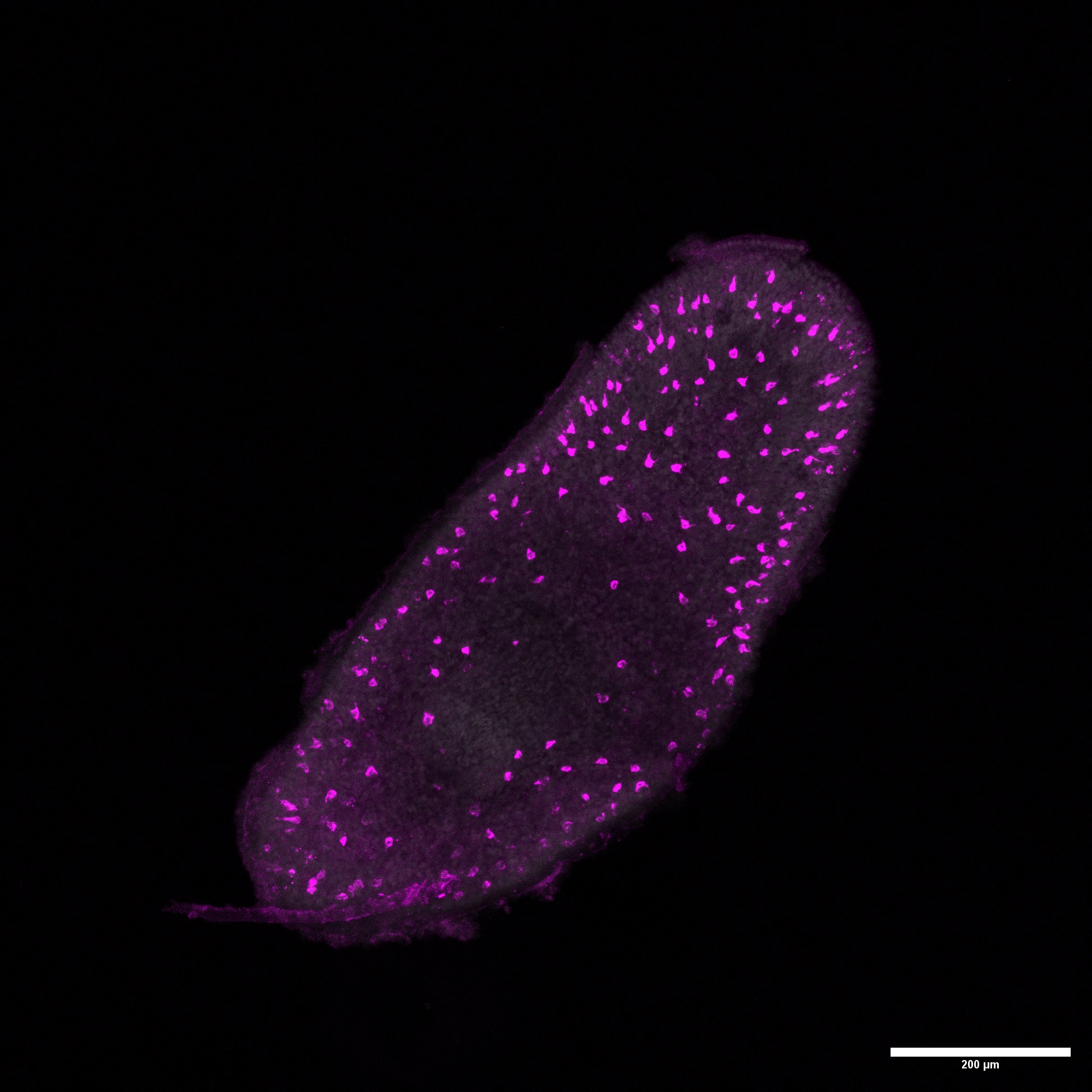

Supplement: Supplementary file 12 — Source data Fig. 5 [file 44318_2025_662_MOESM12_ESM.zip › Figure 5/5D/dd_356/ID_5_ythdf-A_RNAi_Probe_dd_356_rhod_DAPI_10x.jpg]

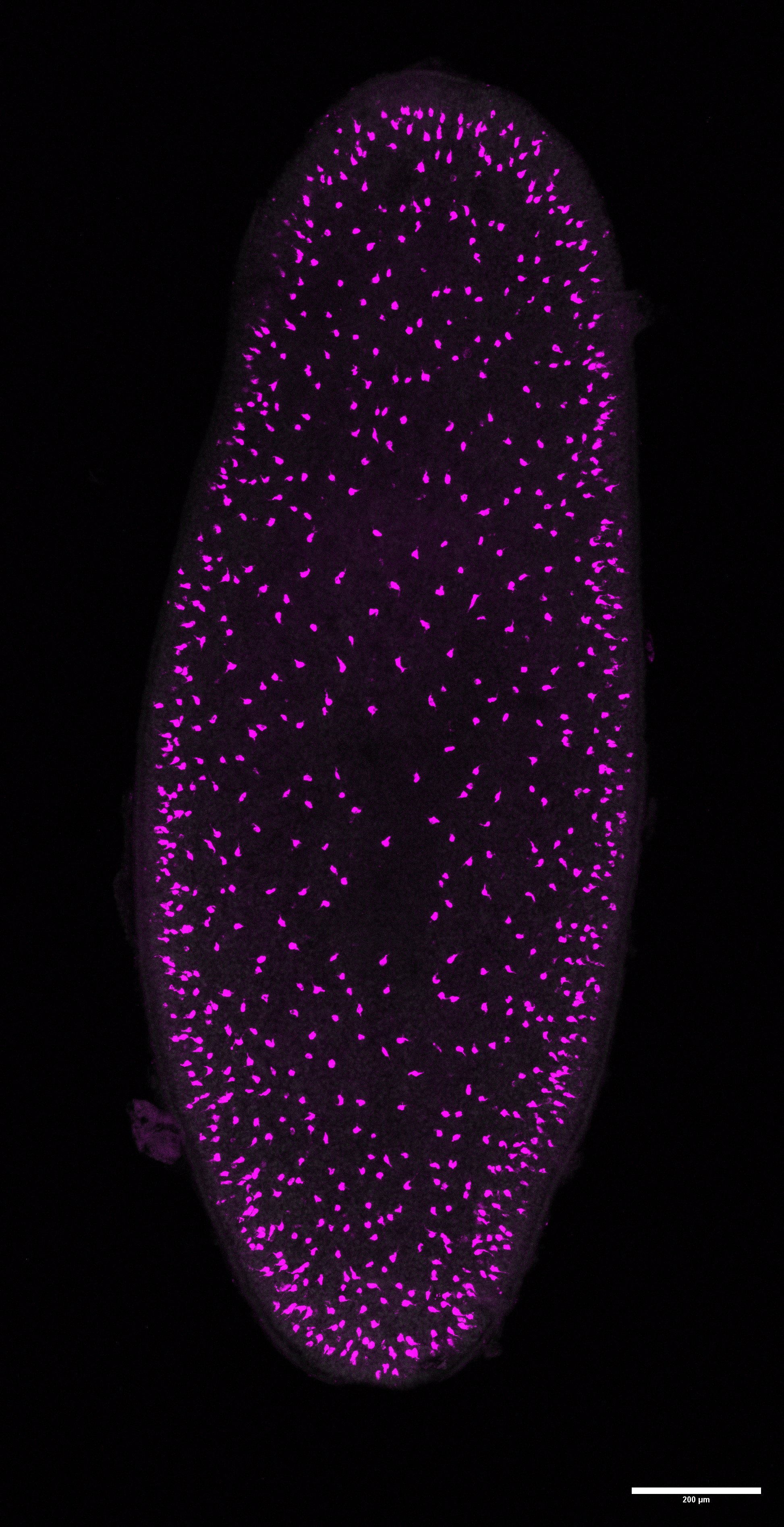

Supplement: Supplementary file 12 — Source data Fig. 5 [file 44318_2025_662_MOESM12_ESM.zip › Figure 5/5D/dd_356/ID_5_ythdf-B_RNAi_Probe_dd_356_rhod_DAPI_10x.jpg]

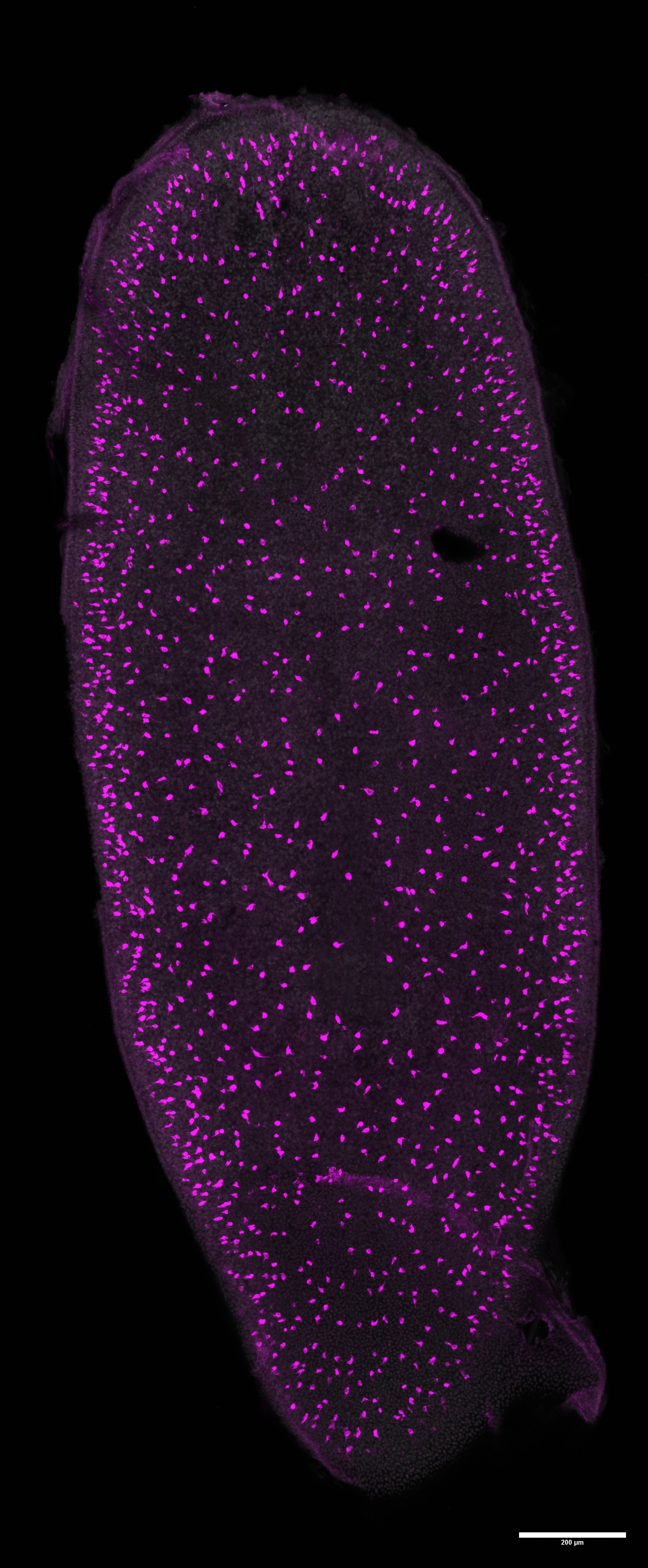

Supplement: Supplementary file 12 — Source data Fig. 5 [file 44318_2025_662_MOESM12_ESM.zip › Figure 5/5D/dd_356/ID_5_ythdf-C_RNAi_Probe_dd_356_rhod_DAPI_10x.jpg]

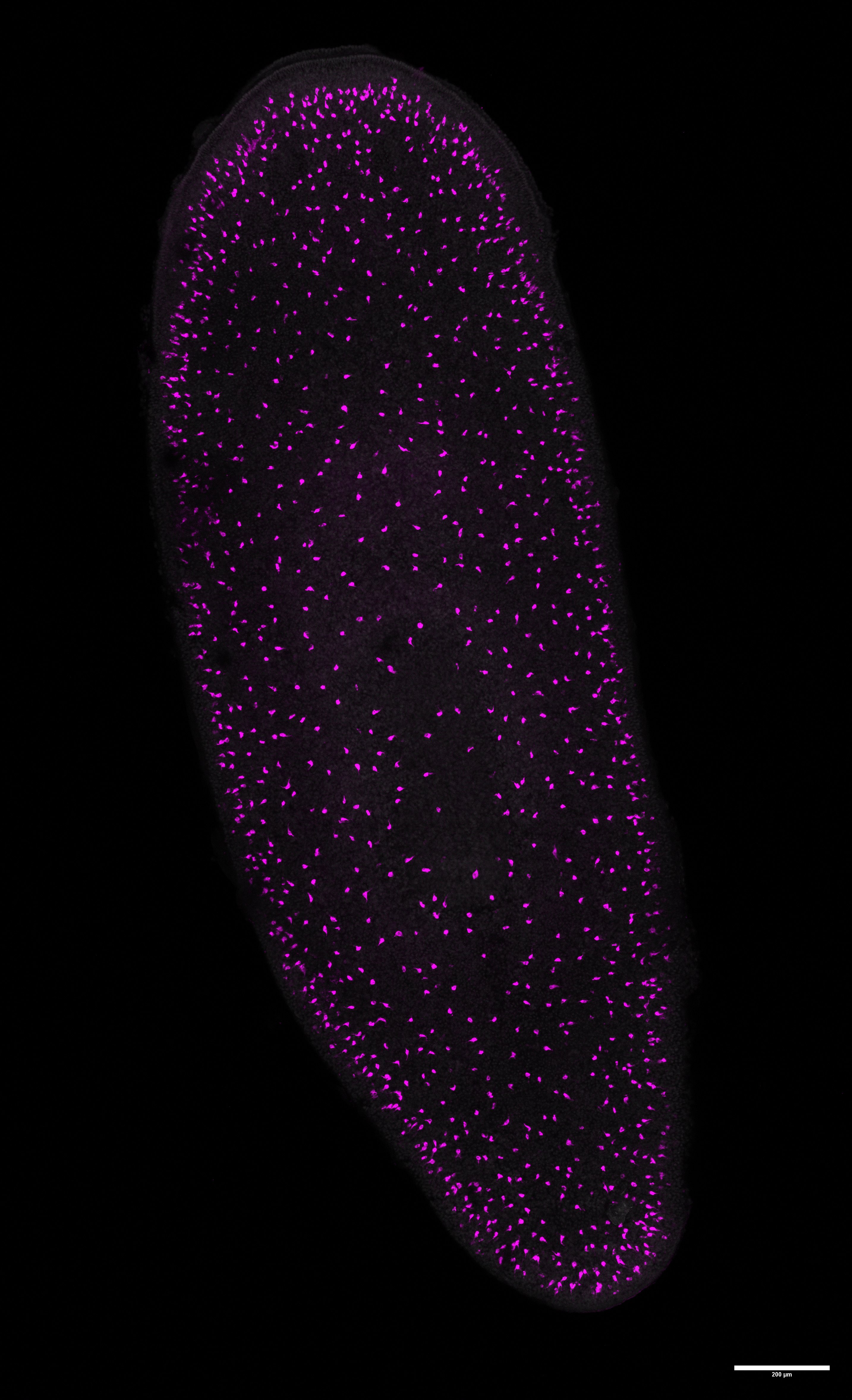

Supplement: Supplementary file 12 — Source data Fig. 5 [file 44318_2025_662_MOESM12_ESM.zip › Figure 5/5D/dd_356/ID_6_Control_RNAi_Probe_dd_356_rhod_DAPI_10x.jpg]

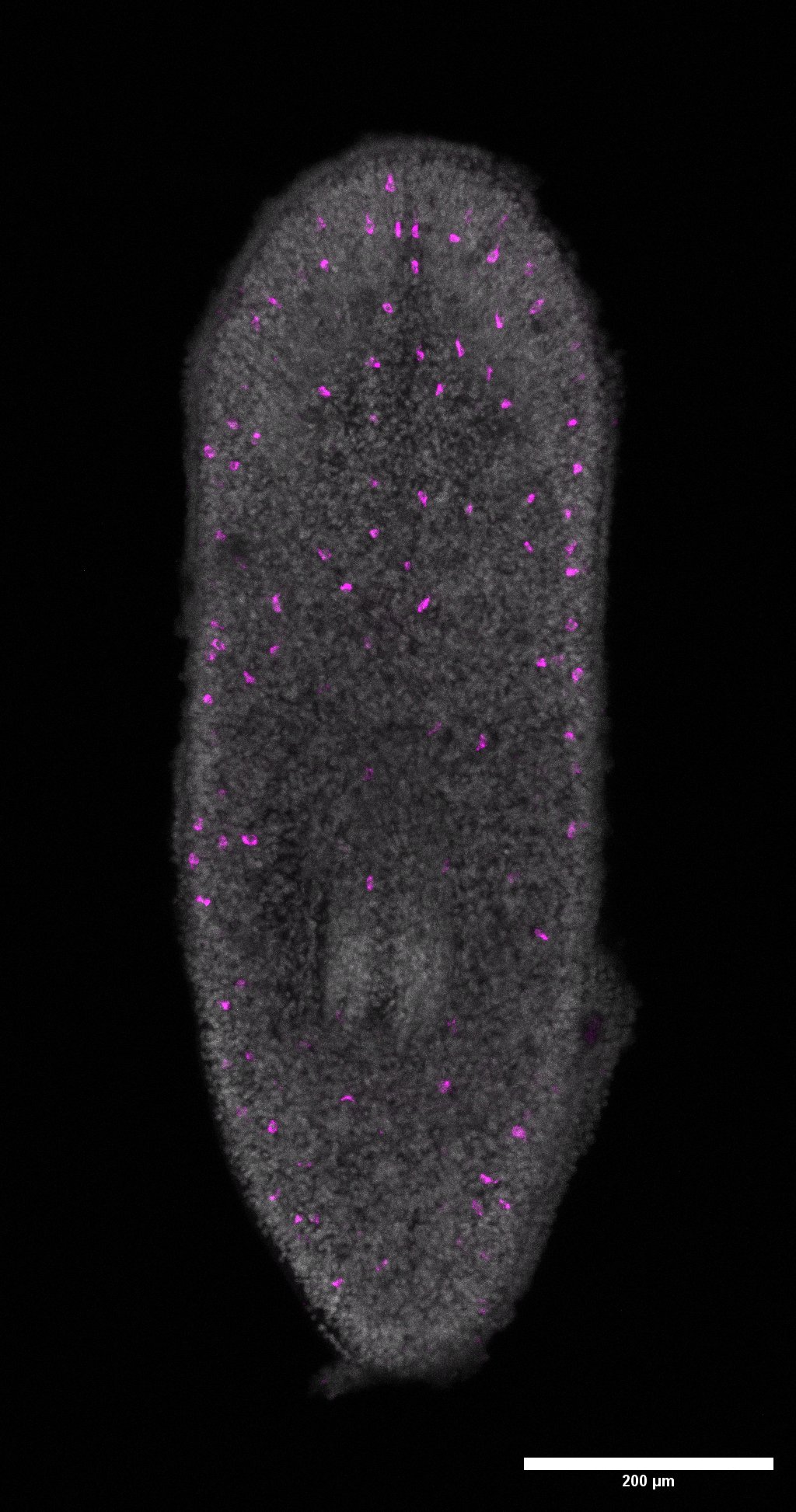

Supplement: Supplementary file 12 — Source data Fig. 5 [file 44318_2025_662_MOESM12_ESM.zip › Figure 5/5D/dd_356/ID_6_Triple_RNAi_Probe_dd_356_rhod_DAPI_10x.jpg]

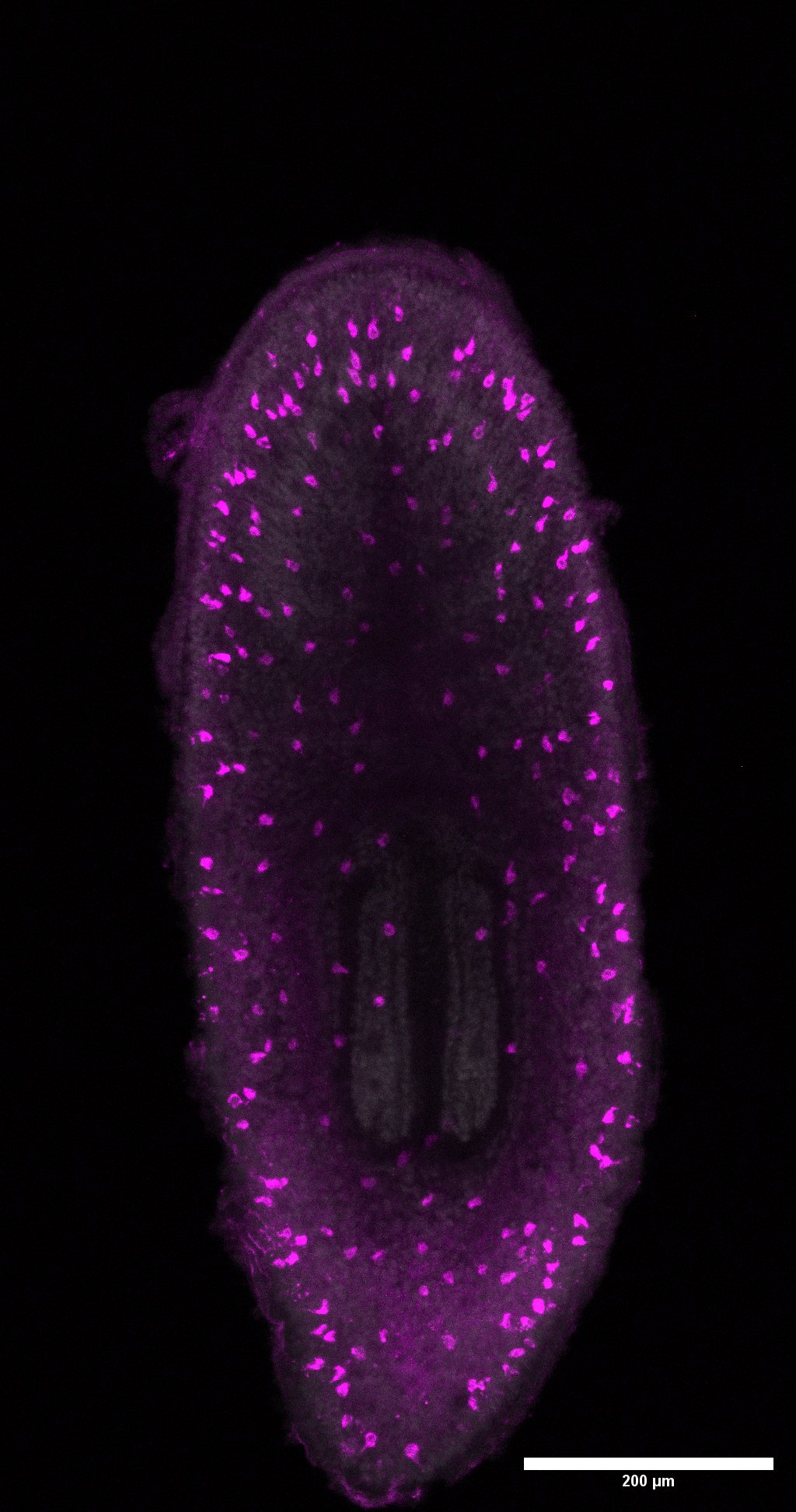

Supplement: Supplementary file 12 — Source data Fig. 5 [file 44318_2025_662_MOESM12_ESM.zip › Figure 5/5D/dd_356/ID_6_ythdf-A_RNAi_Probe_dd_356_rhod_DAPI_10x.jpg]

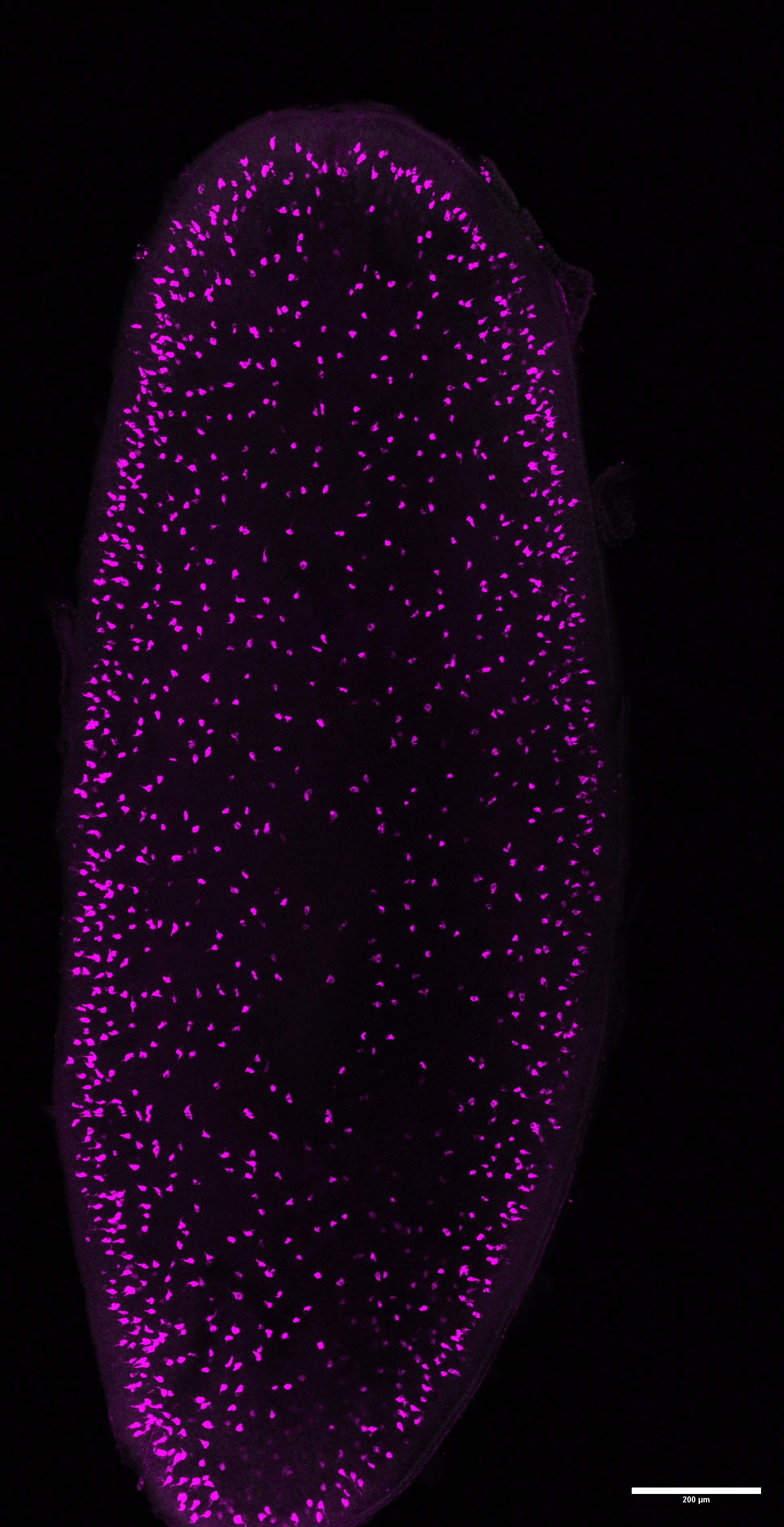

Supplement: Supplementary file 12 — Source data Fig. 5 [file 44318_2025_662_MOESM12_ESM.zip › Figure 5/5D/dd_356/ID_6_ythdf-B_RNAi_Probe_dd_356_rhod_DAPI_10x.jpg]

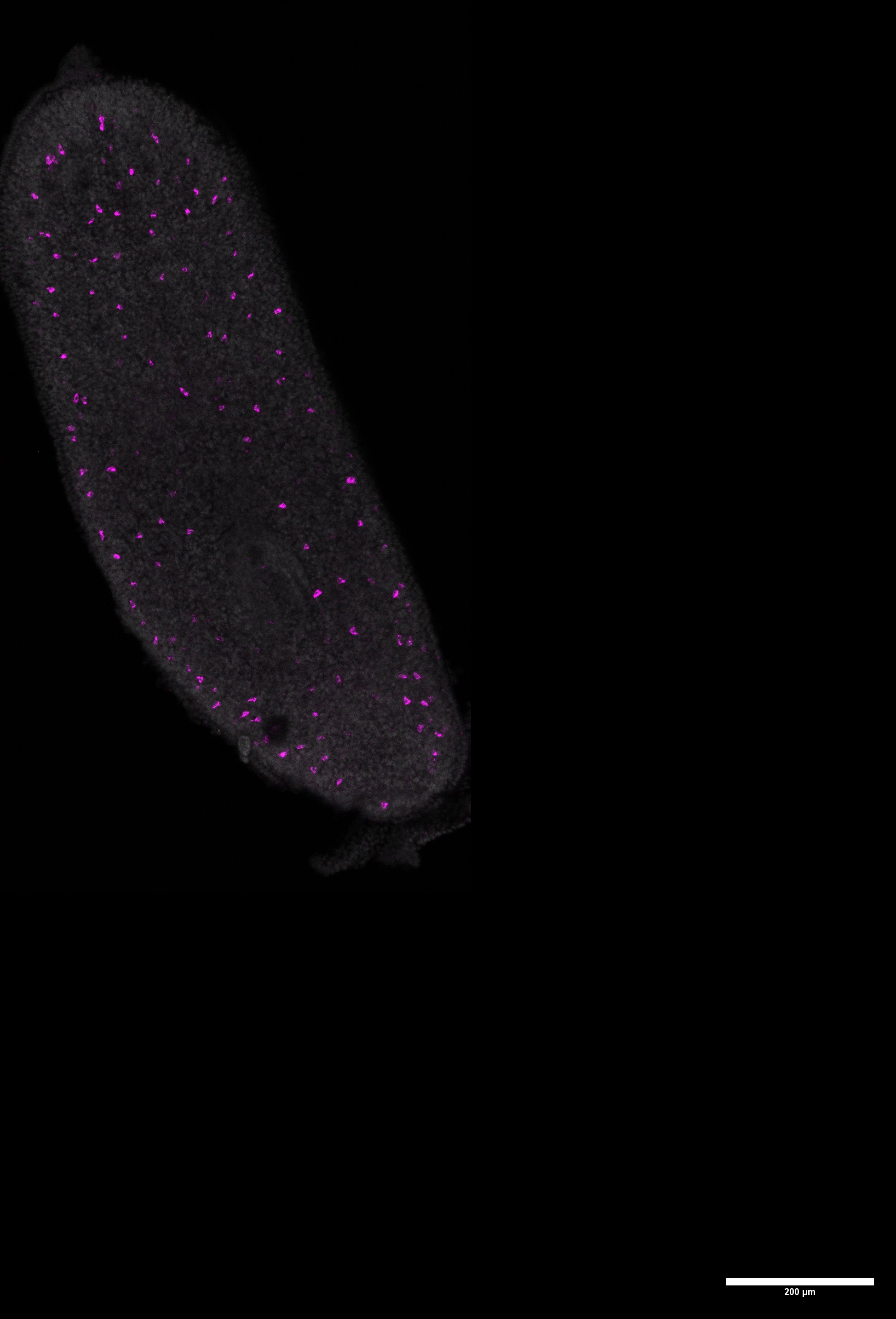

Supplement: Supplementary file 12 — Source data Fig. 5 [file 44318_2025_662_MOESM12_ESM.zip › Figure 5/5D/dd_356/ID_7_Triple_RNAi_Probe_dd_356_rhod_DAPI_10x.jpg]

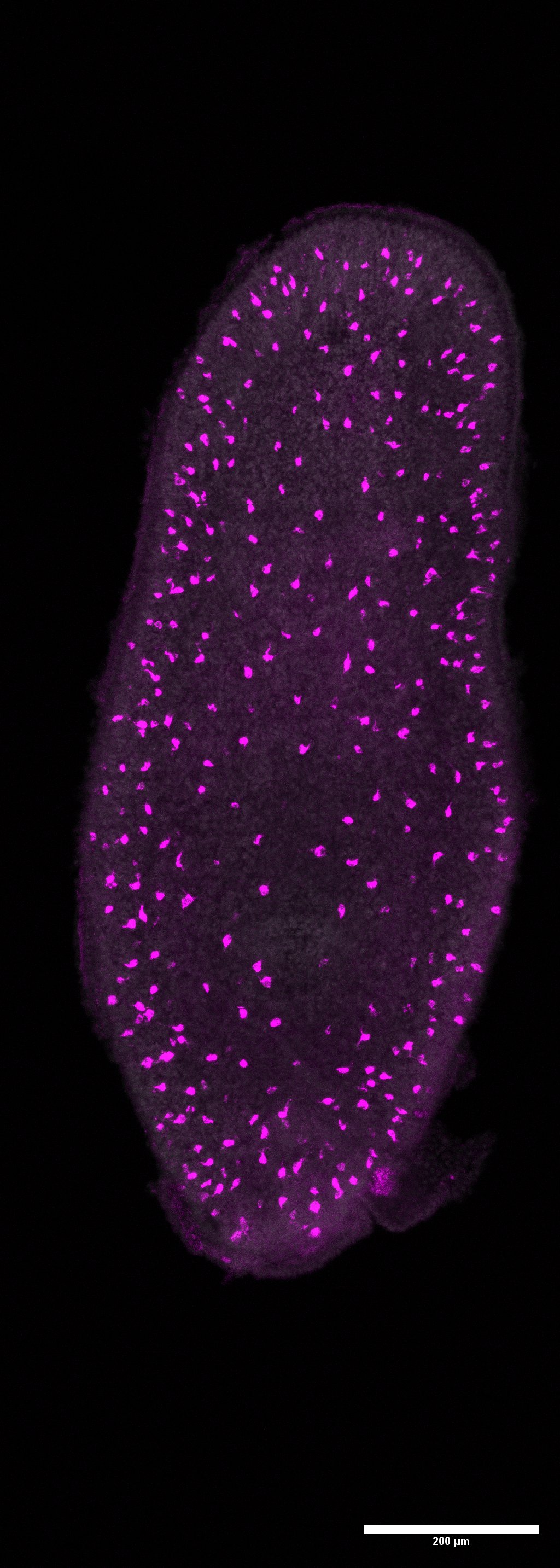

Supplement: Supplementary file 12 — Source data Fig. 5 [file 44318_2025_662_MOESM12_ESM.zip › Figure 5/5D/dd_356/ID_7_ythdf-A_RNAi_Probe_dd_356_rhod_DAPI_10x.jpg]

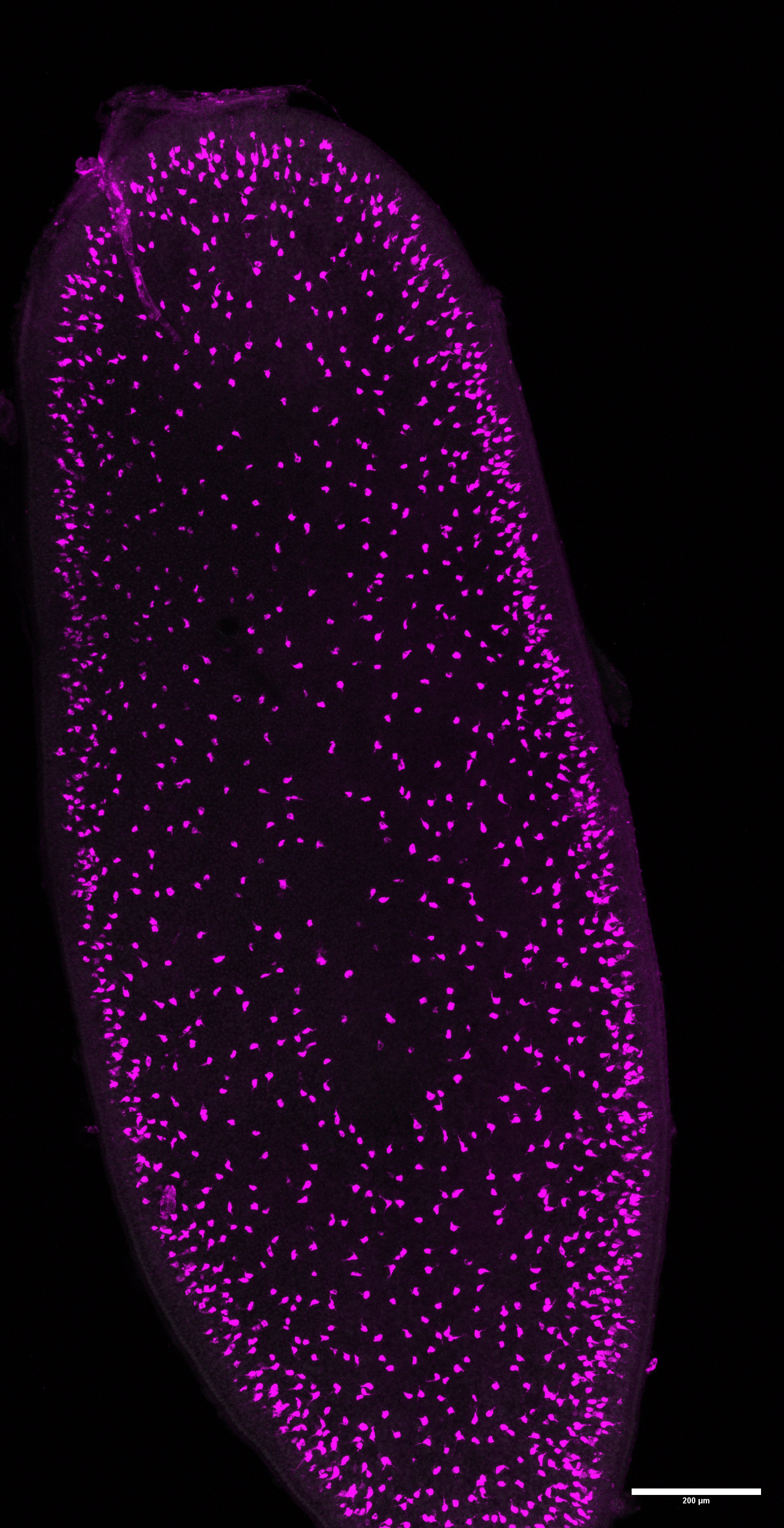

Supplement: Supplementary file 12 — Source data Fig. 5 [file 44318_2025_662_MOESM12_ESM.zip › Figure 5/5D/dd_356/ID_7_ythdf-B_RNAi_Probe_dd_356_rhod_DAPI_10x.jpg]

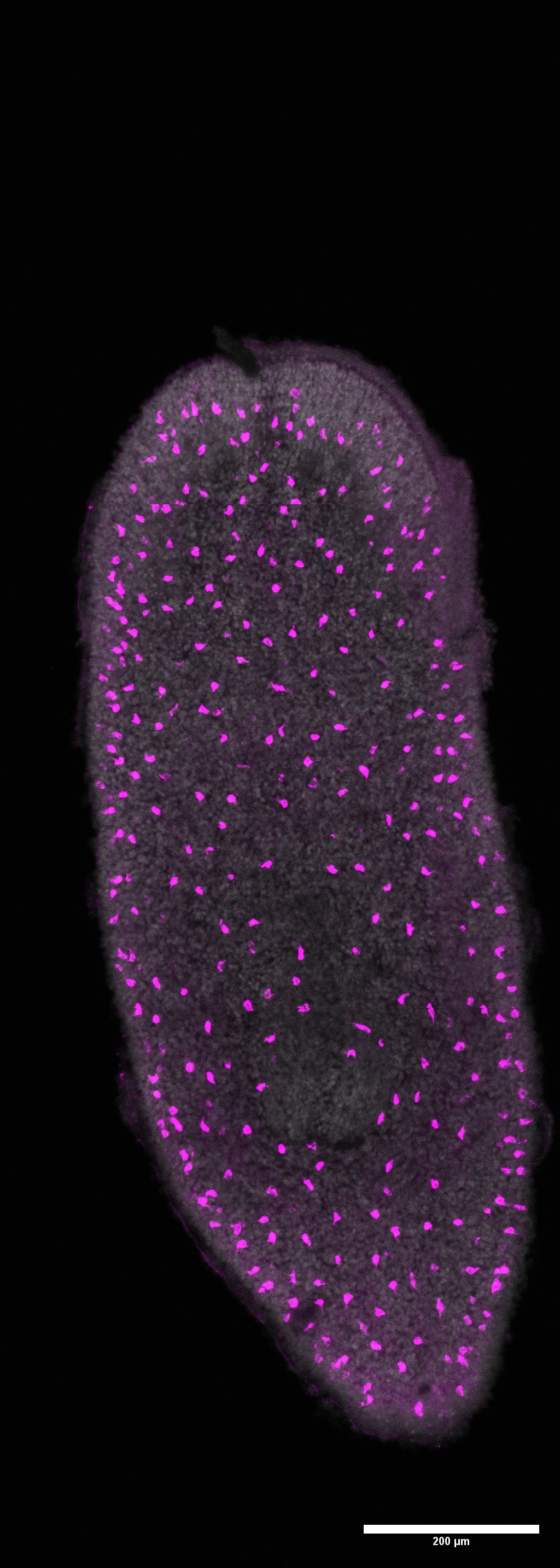

Supplement: Supplementary file 12 — Source data Fig. 5 [file 44318_2025_662_MOESM12_ESM.zip › Figure 5/5D/dd_356/ID_7_ythdf-C_RNAi_Probe_dd_356_rhod_DAPI_10x.jpg]

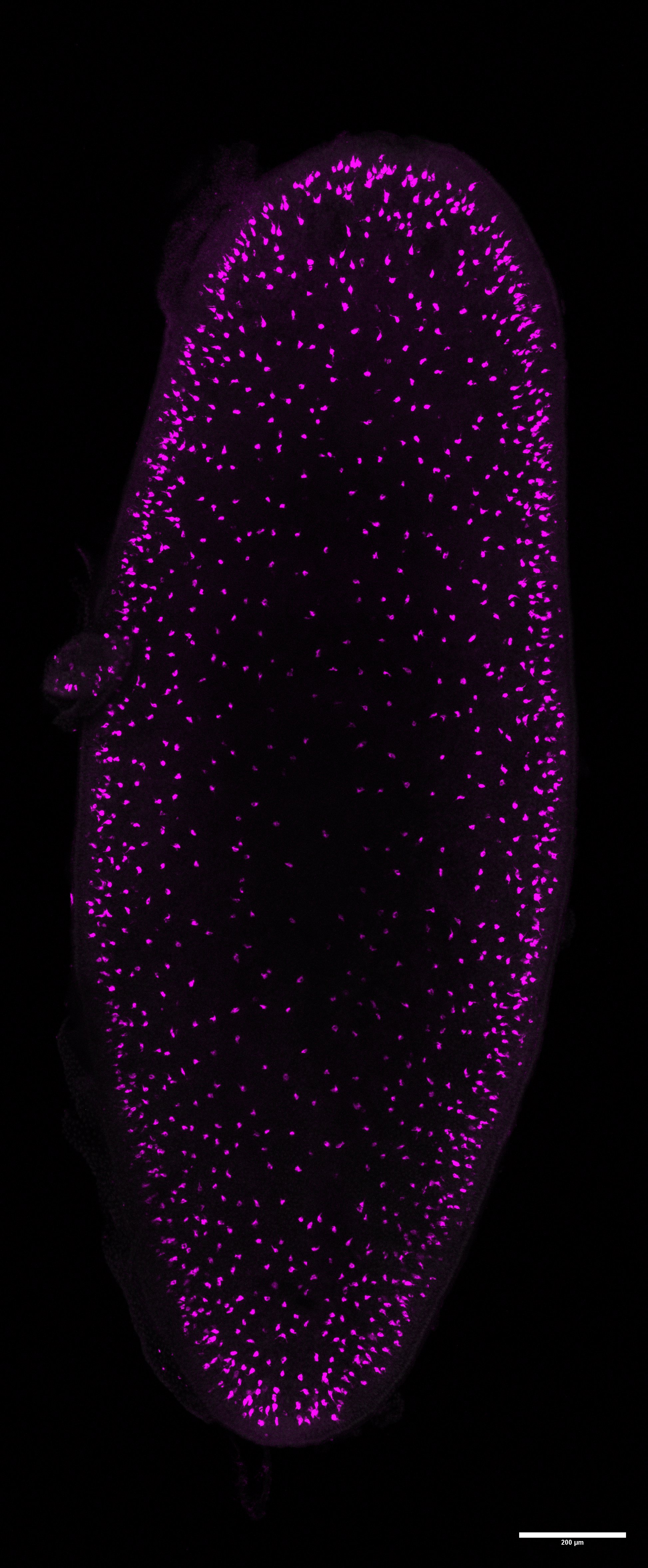

Supplement: Supplementary file 12 — Source data Fig. 5 [file 44318_2025_662_MOESM12_ESM.zip › Figure 5/5D/dd_356/ID_8_ythdf-B_RNAi_Probe_dd_356_rhod_DAPI_10x.jpg]
