# Supplementary figures and images for: Single-nucleotide m⁶A mapping uncovers redundant YTHDF function in planarian progenitor fate selection (part 4 of 6)
Source: EMBO J. 2026 Jan 3;45(3):749–88. doi: 10.1038/s44318-025-00662-3 (PMC12864844; doi:10.1038/s44318-025-00662-3)

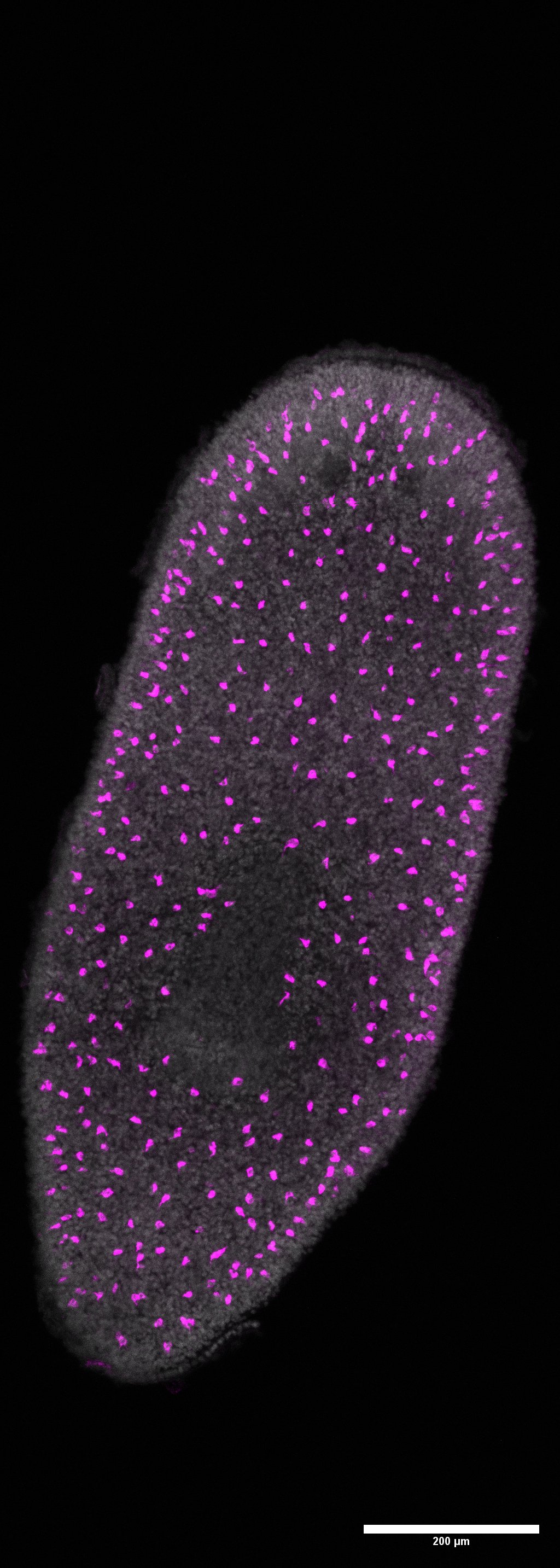

Supplement: Supplementary file 12 — Source data Fig. 5 [file 44318_2025_662_MOESM12_ESM.zip › Figure 5/5D/dd_356/ID_8_ythdf-C_RNAi_Probe_dd_356_rhod_DAPI_10x.jpg]

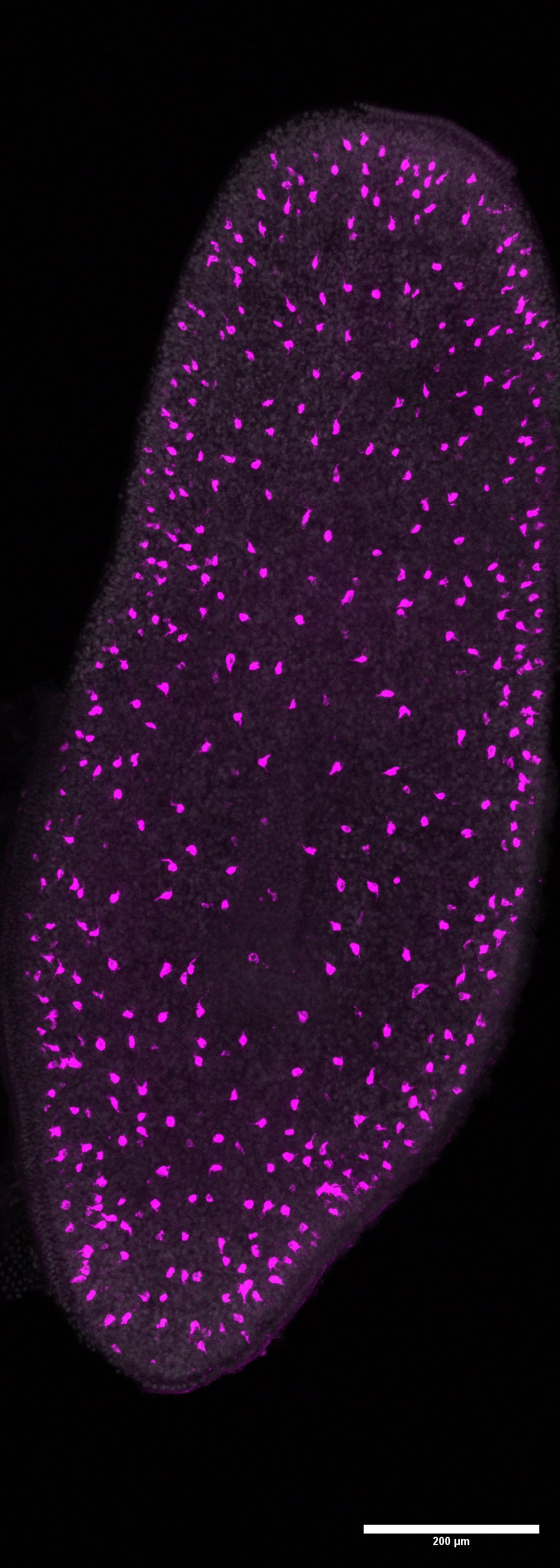

Supplement: Supplementary file 12 — Source data Fig. 5 [file 44318_2025_662_MOESM12_ESM.zip › Figure 5/5D/dd_356/ID_9_ythdf-A_RNAi_Probe_dd_356_rhod_DAPI_10x.jpg]

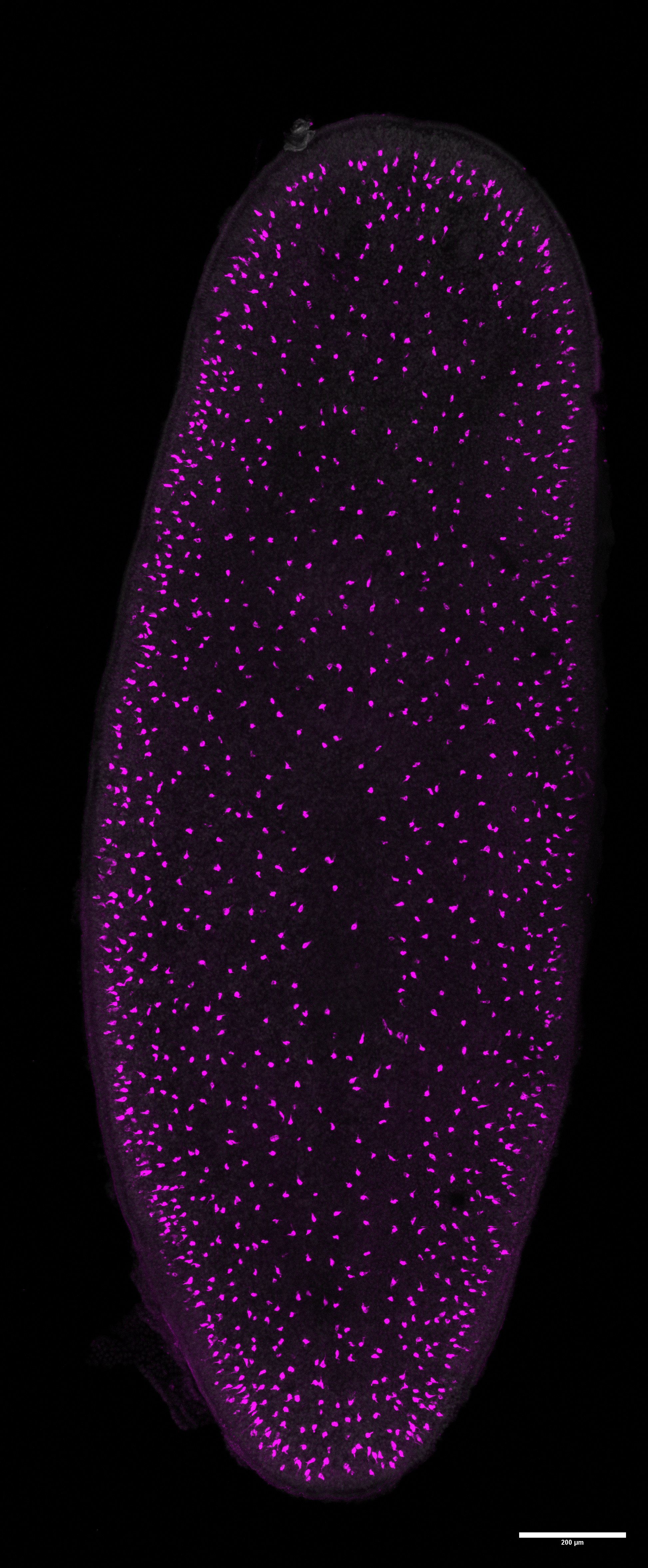

Supplement: Supplementary file 12 — Source data Fig. 5 [file 44318_2025_662_MOESM12_ESM.zip › Figure 5/5D/dd_356/ID_9_ythdf-C_RNAi_Probe_dd_356_rhod_DAPI_10x.jpg]

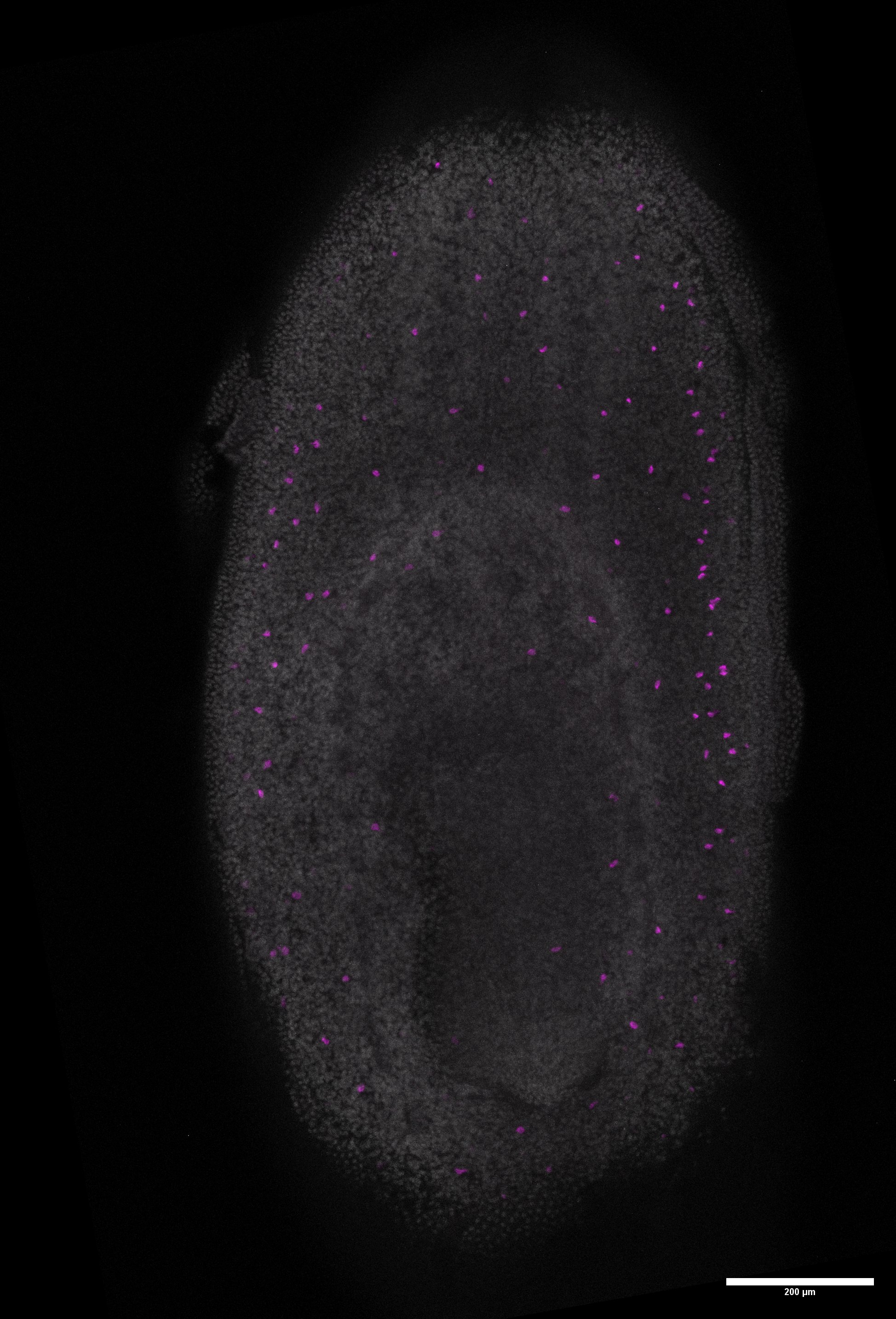

Supplement: Supplementary file 12 — Source data Fig. 5 [file 44318_2025_662_MOESM12_ESM.zip › Figure 5/5D/dd_924/ID_10_Control_RNAi_Probe_dd924_rhod_DAPI_10x.jpg]

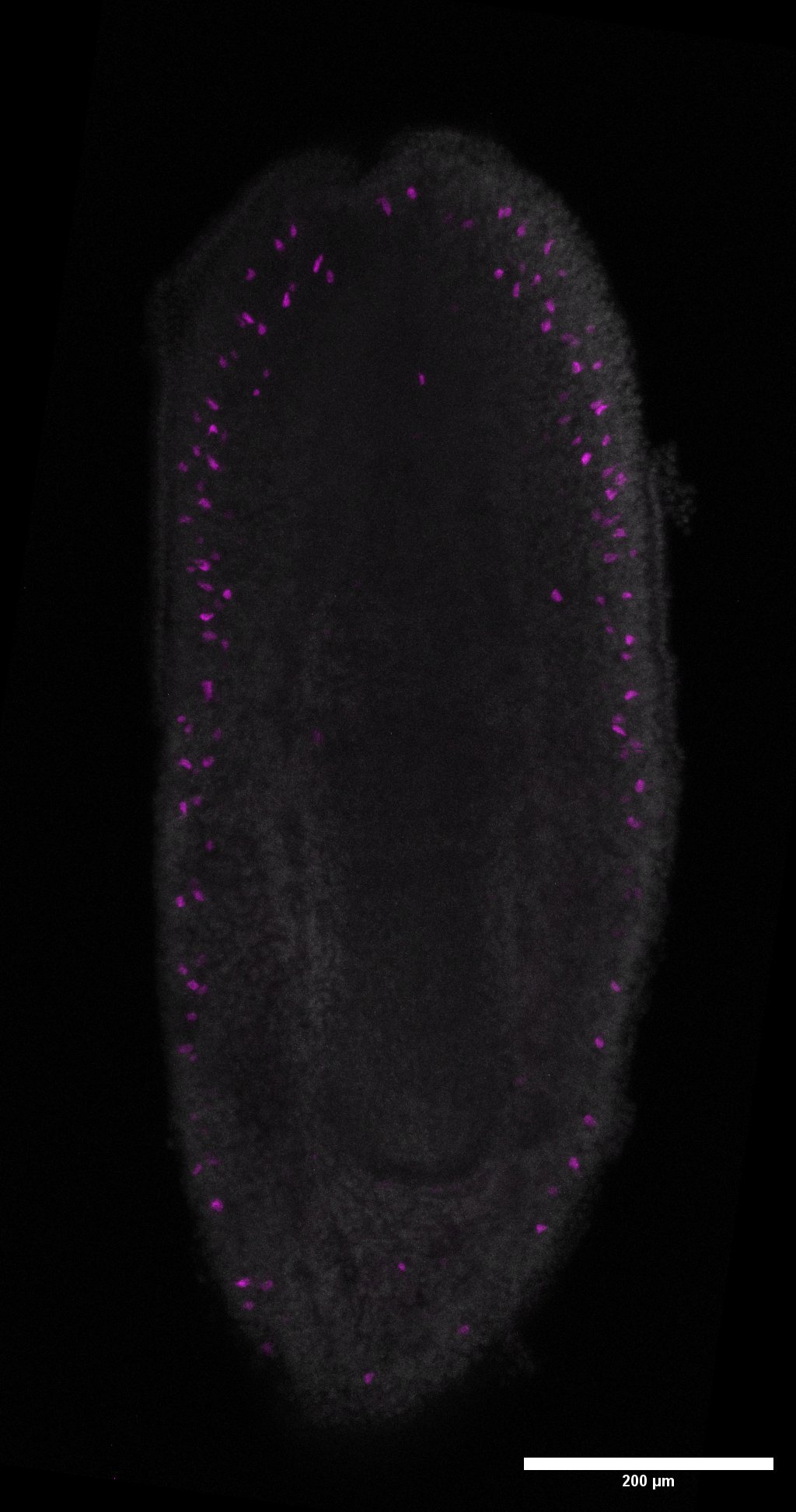

Supplement: Supplementary file 12 — Source data Fig. 5 [file 44318_2025_662_MOESM12_ESM.zip › Figure 5/5D/dd_924/ID_1_Control_RNAi_Probe_dd924_rhod_DAPI_10x.jpg]

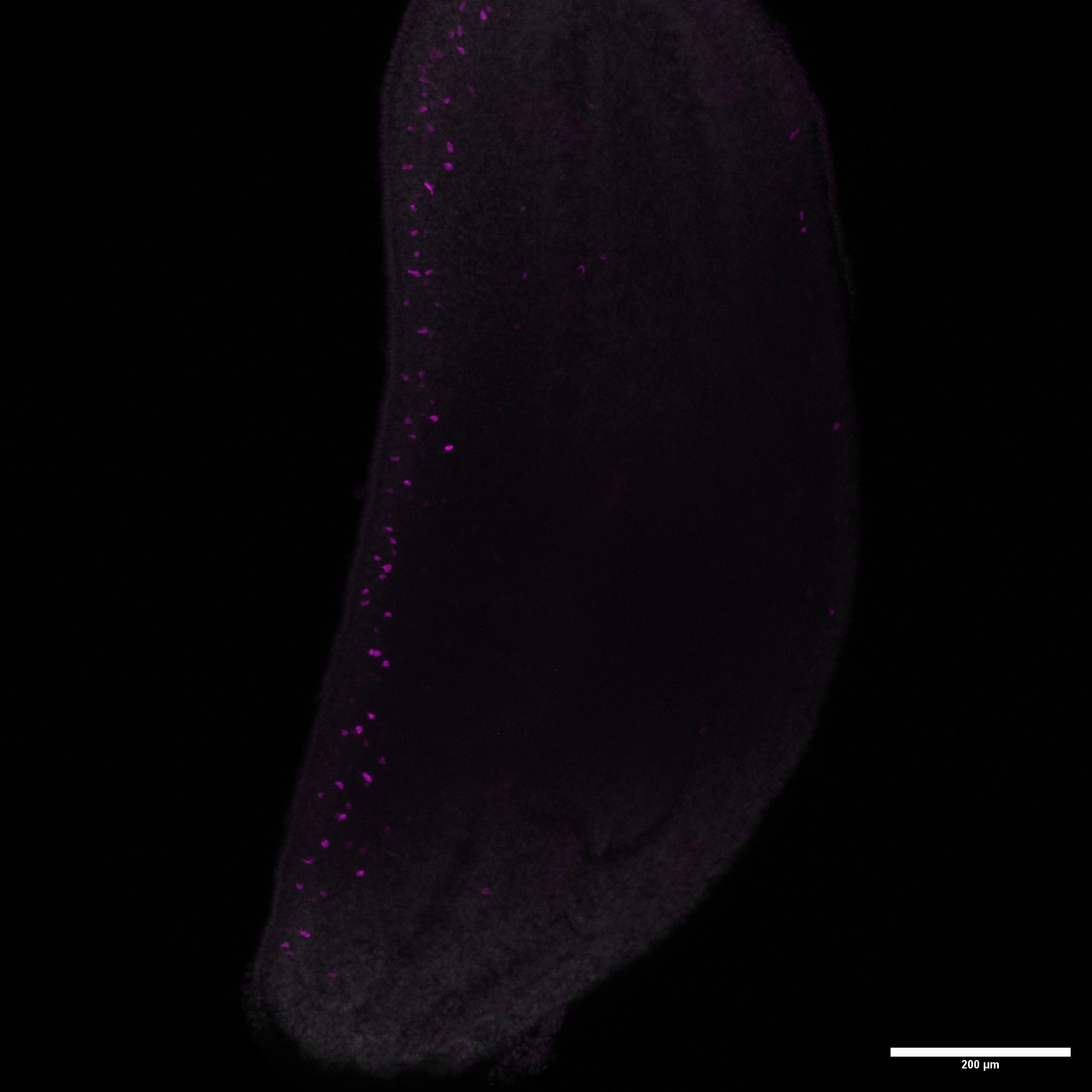

Supplement: Supplementary file 12 — Source data Fig. 5 [file 44318_2025_662_MOESM12_ESM.zip › Figure 5/5D/dd_924/ID_1_Triple_RNAi_Probe_dd924_rhod_DAPI_10x.jpg]

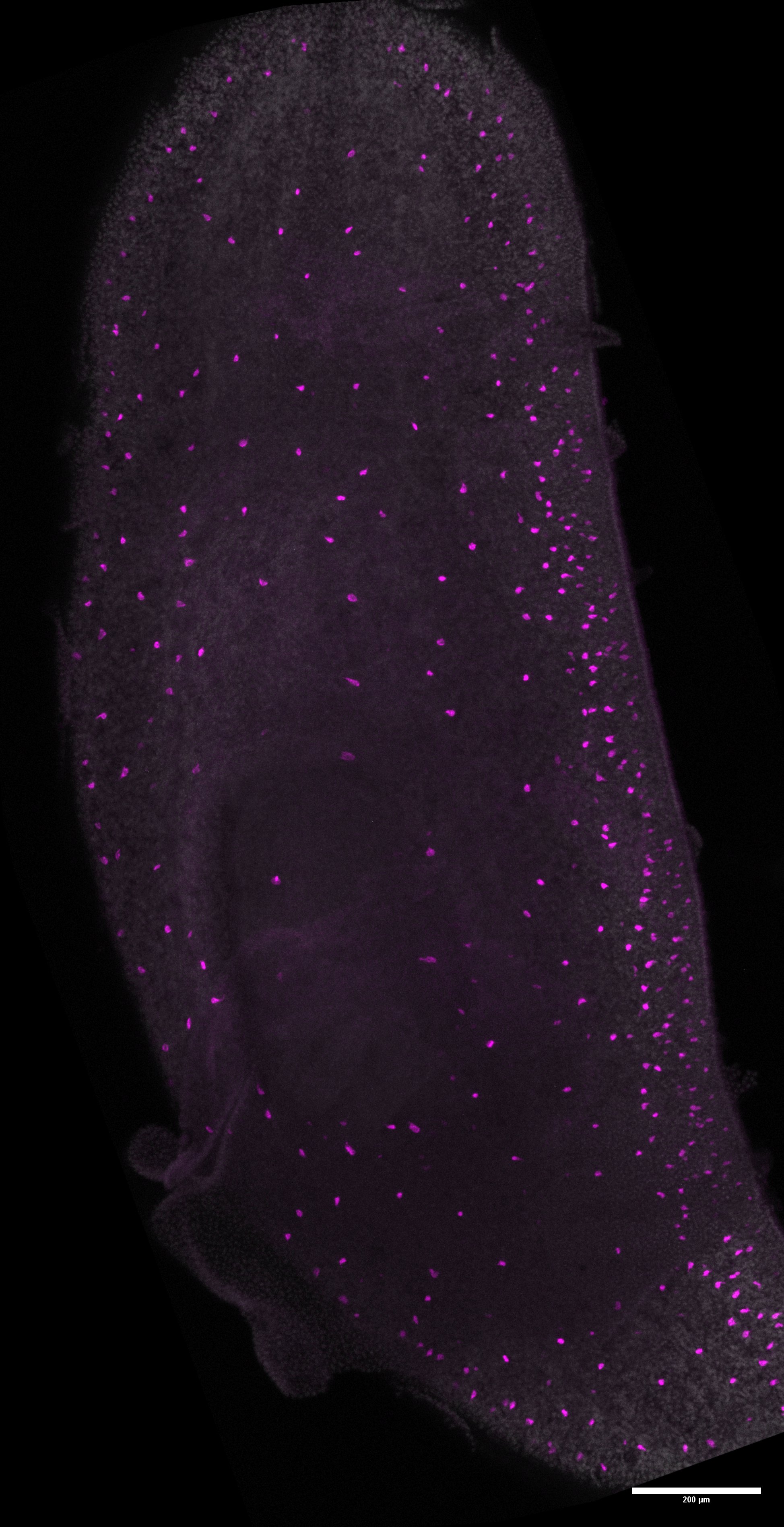

Supplement: Supplementary file 12 — Source data Fig. 5 [file 44318_2025_662_MOESM12_ESM.zip › Figure 5/5D/dd_924/ID_1_ythdf-A_RNAi_Probe_dd924_rhod_DAPI_10x.jpg]

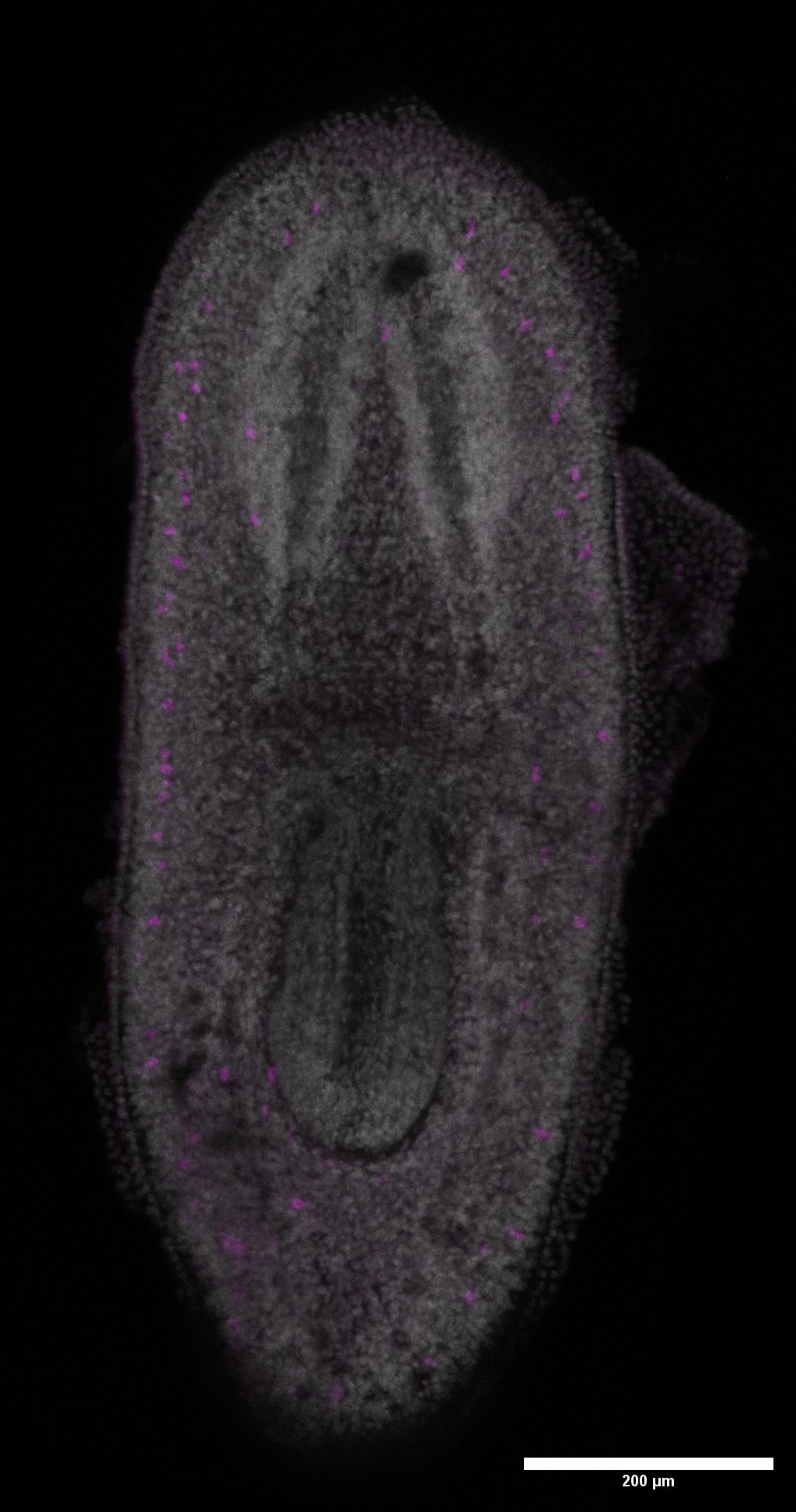

Supplement: Supplementary file 12 — Source data Fig. 5 [file 44318_2025_662_MOESM12_ESM.zip › Figure 5/5D/dd_924/ID_1_ythdf-B_RNAi_Probe_dd924_rhod_DAPI_10x.jpg]

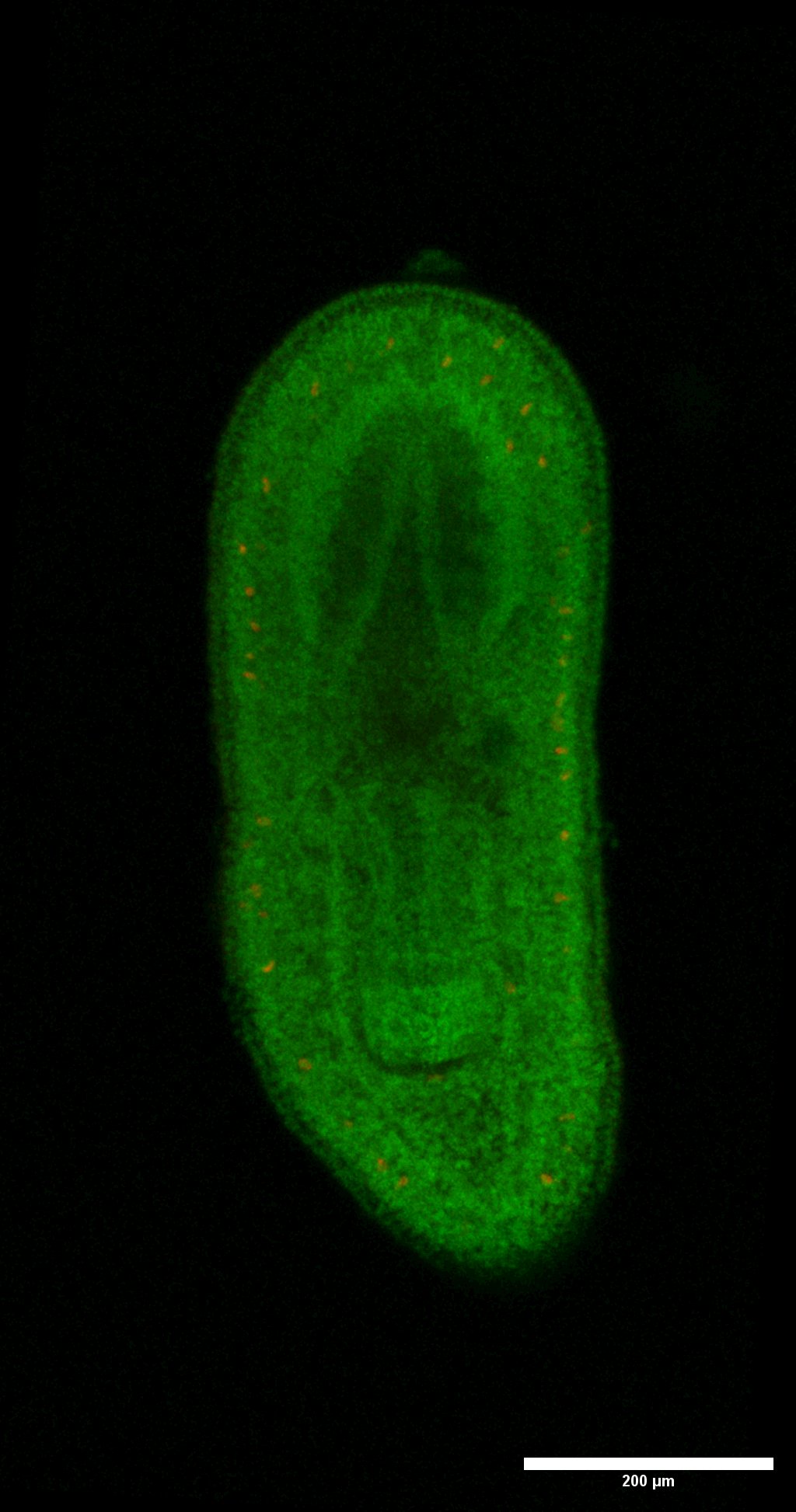

Supplement: Supplementary file 12 — Source data Fig. 5 [file 44318_2025_662_MOESM12_ESM.zip › Figure 5/5D/dd_924/ID_1_ythdf-C_RNAi_Probe_dd924_rhod_DAPI_10x.jpg]

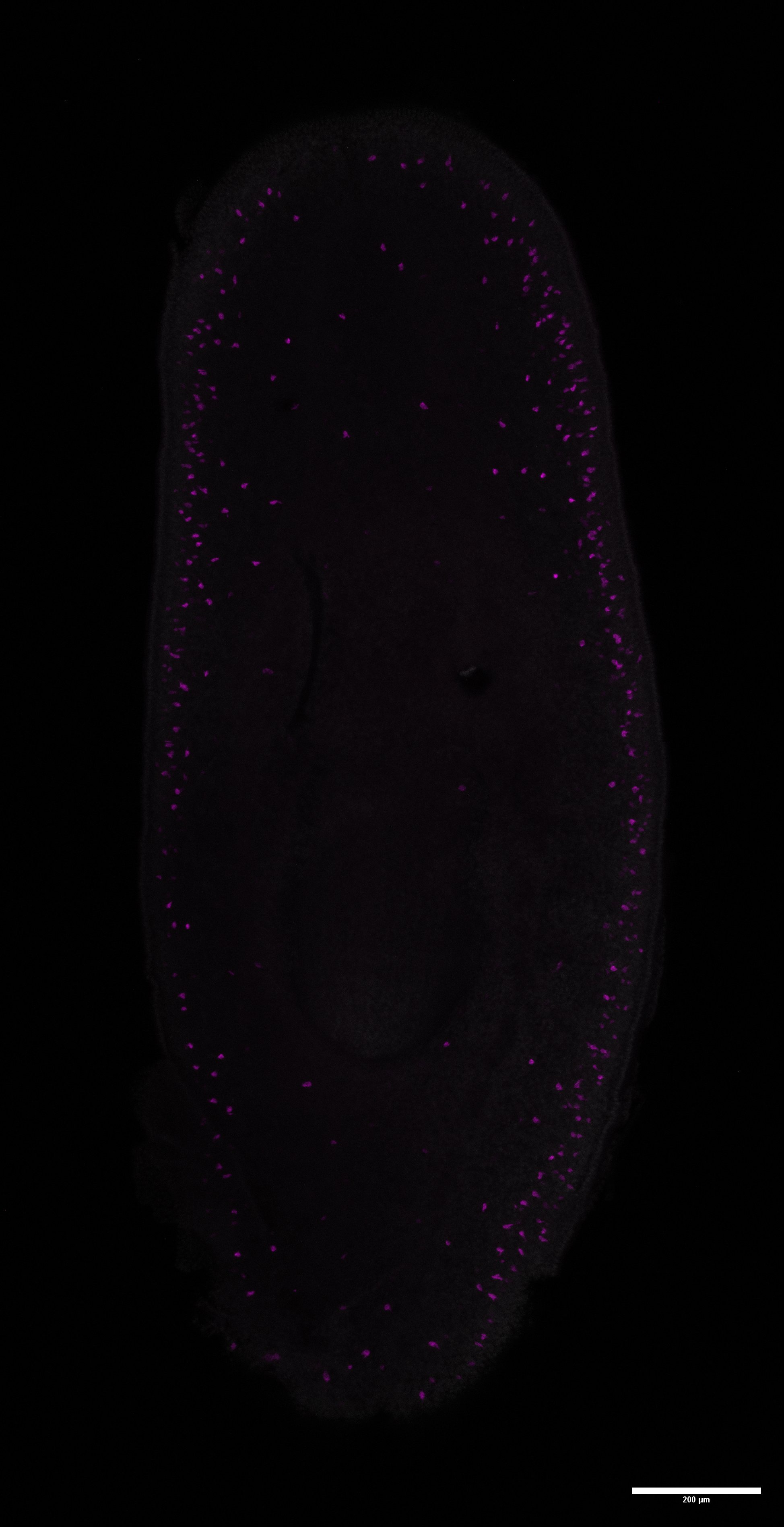

Supplement: Supplementary file 12 — Source data Fig. 5 [file 44318_2025_662_MOESM12_ESM.zip › Figure 5/5D/dd_924/ID_2_Control_RNAi_Probe_dd924_rhod_DAPI_10x.jpg]

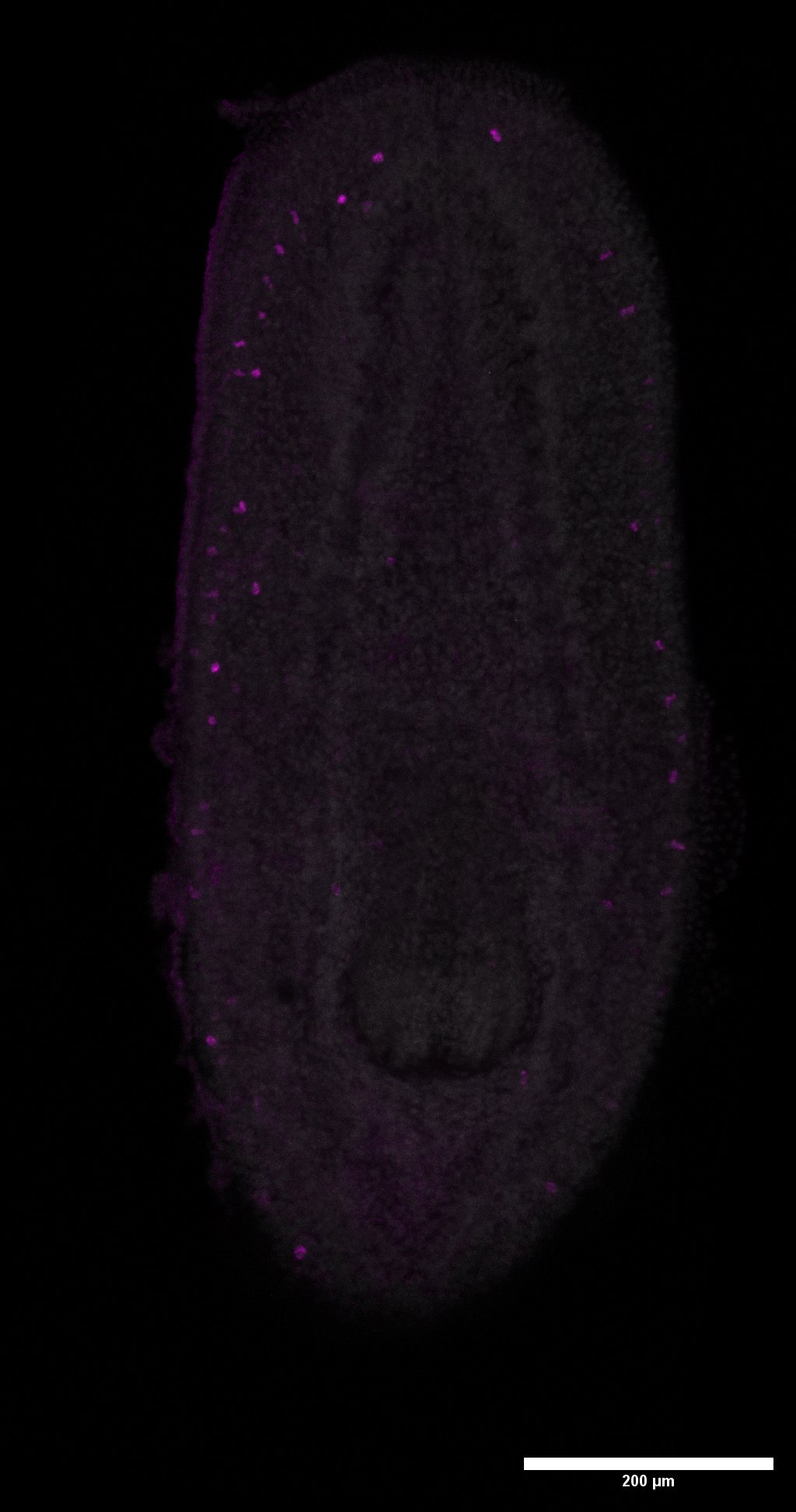

Supplement: Supplementary file 12 — Source data Fig. 5 [file 44318_2025_662_MOESM12_ESM.zip › Figure 5/5D/dd_924/ID_2_Triple_RNAi_Probe_dd924_rhod_DAPI_10x.jpg]

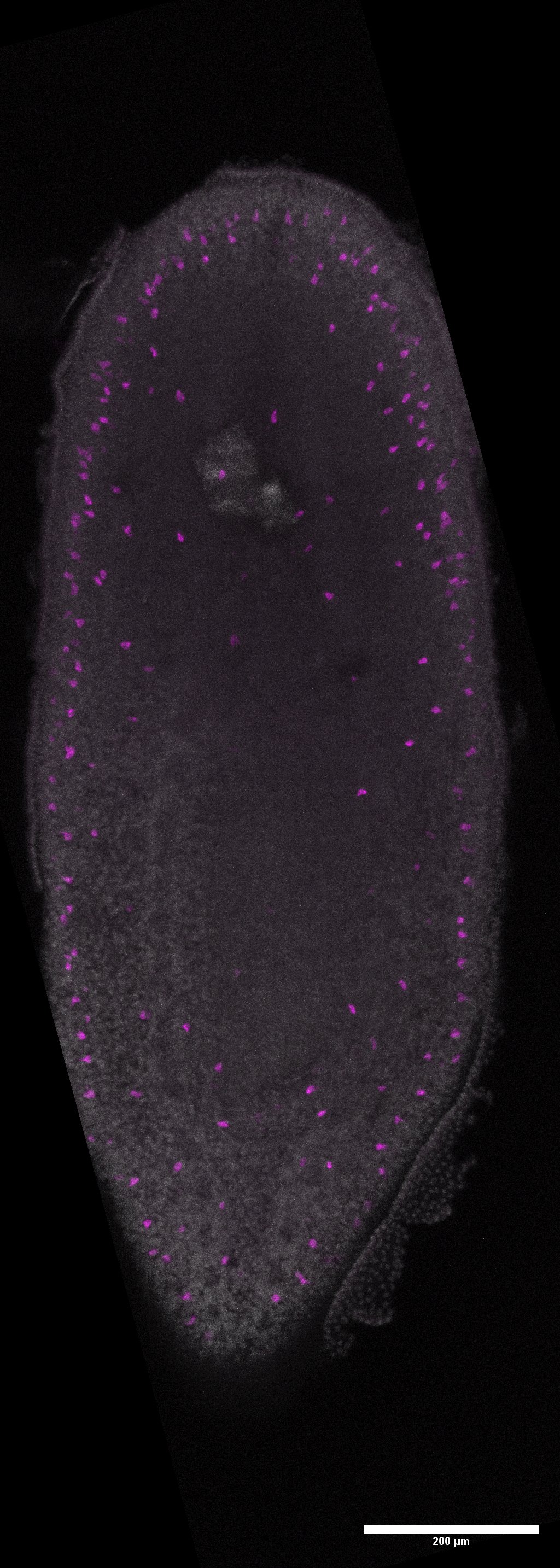

Supplement: Supplementary file 12 — Source data Fig. 5 [file 44318_2025_662_MOESM12_ESM.zip › Figure 5/5D/dd_924/ID_2_ythdf-A_RNAi_Probe_dd924_rhod_DAPI_10x.jpg]

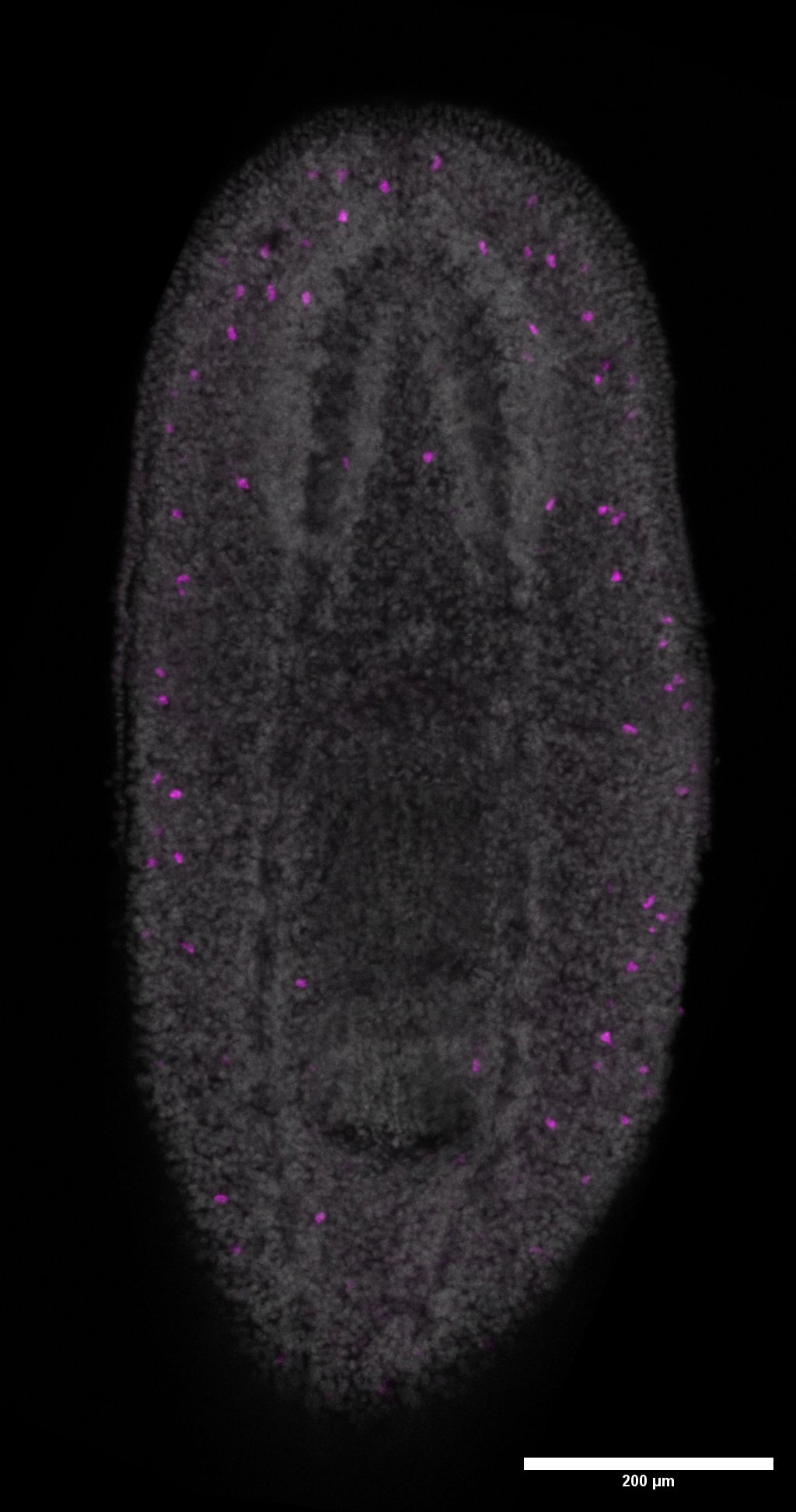

Supplement: Supplementary file 12 — Source data Fig. 5 [file 44318_2025_662_MOESM12_ESM.zip › Figure 5/5D/dd_924/ID_2_ythdf-B_RNAi_Probe_dd924_rhod_DAPI_10x.jpg]

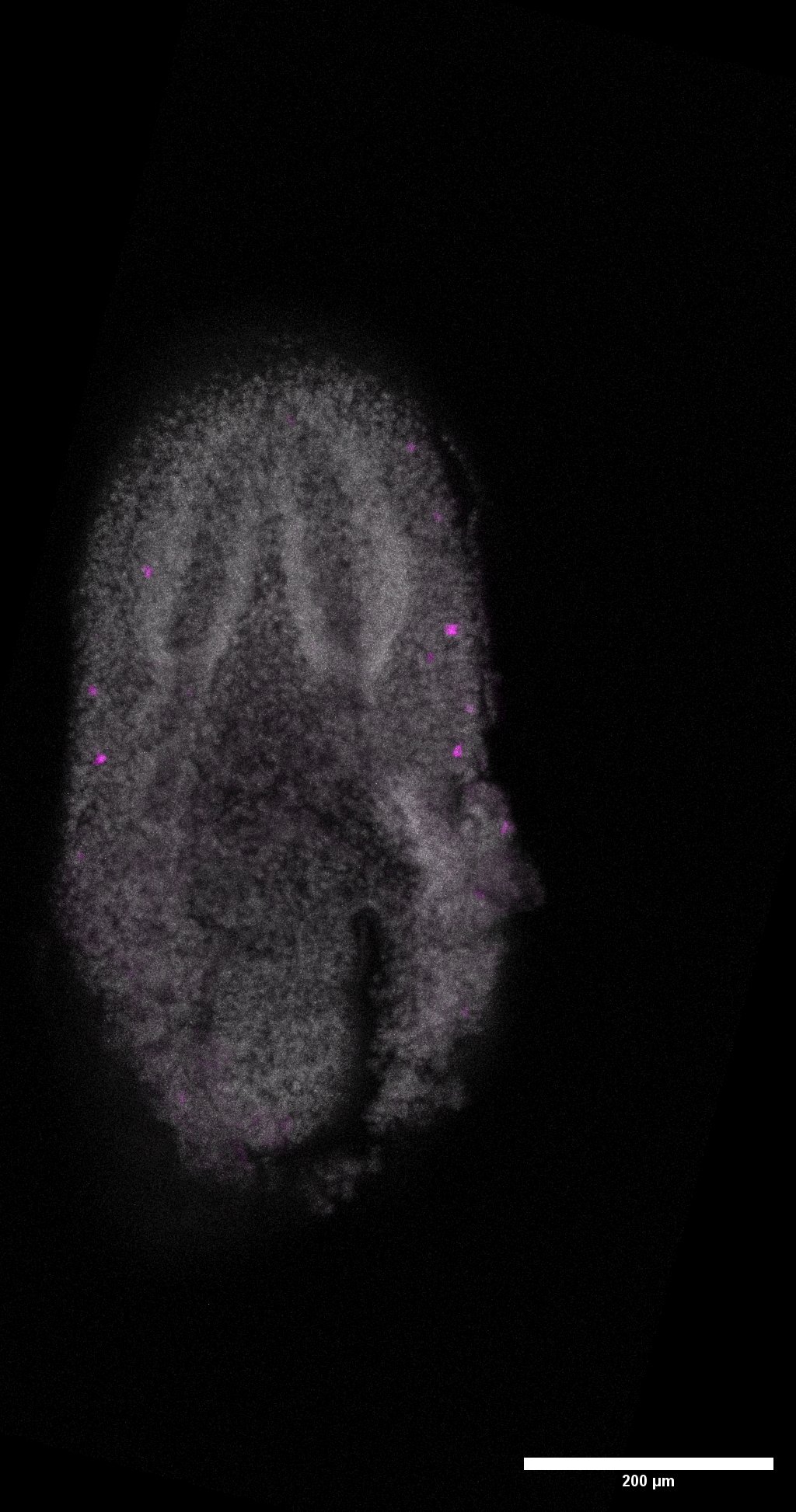

Supplement: Supplementary file 12 — Source data Fig. 5 [file 44318_2025_662_MOESM12_ESM.zip › Figure 5/5D/dd_924/ID_2_ythdf-C_RNAi_Probe_dd924_rhod_DAPI_10x.jpg]

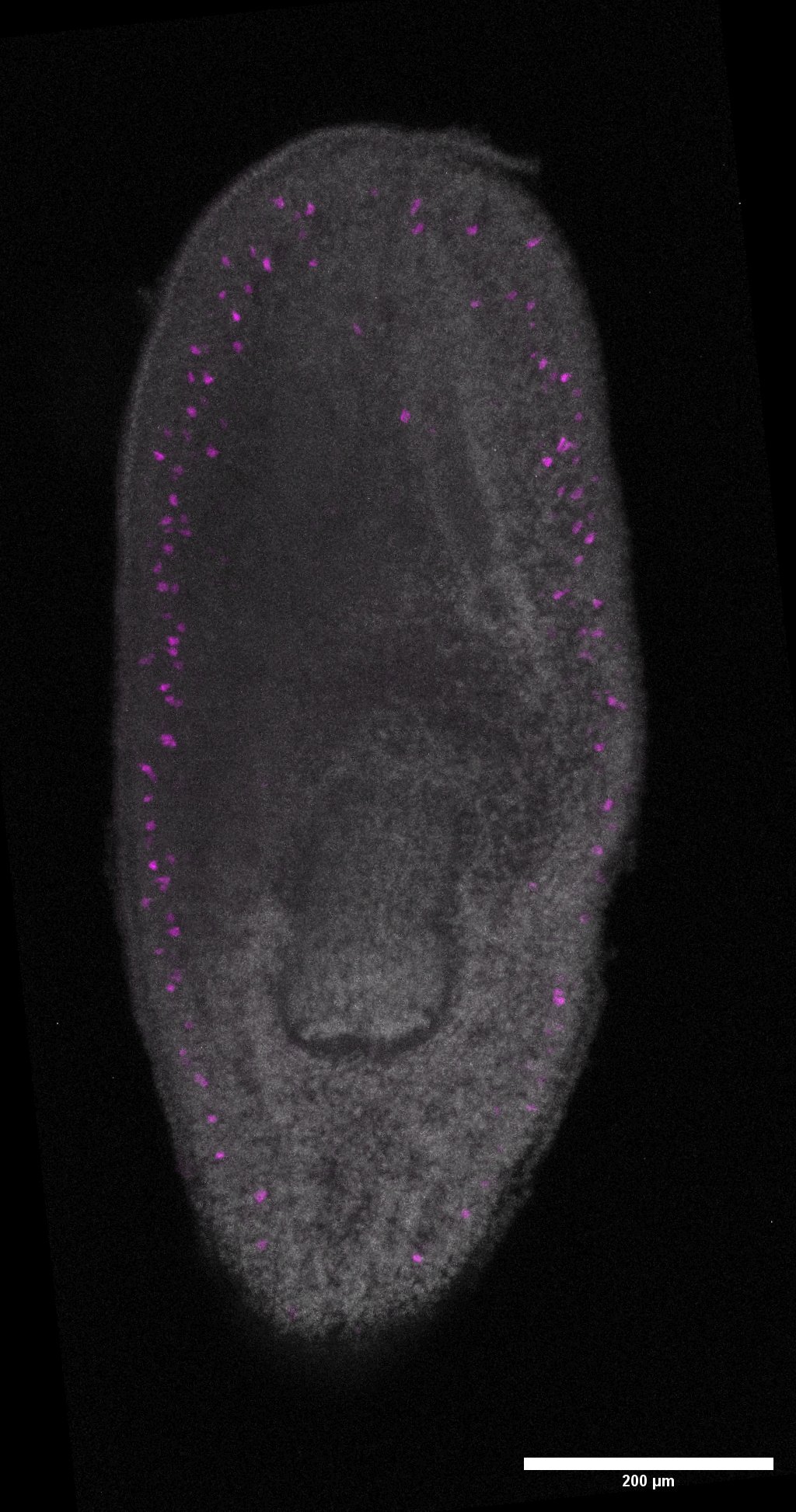

Supplement: Supplementary file 12 — Source data Fig. 5 [file 44318_2025_662_MOESM12_ESM.zip › Figure 5/5D/dd_924/ID_3_Control_RNAi_Probe_dd924_rhod_DAPI_10x.jpg]

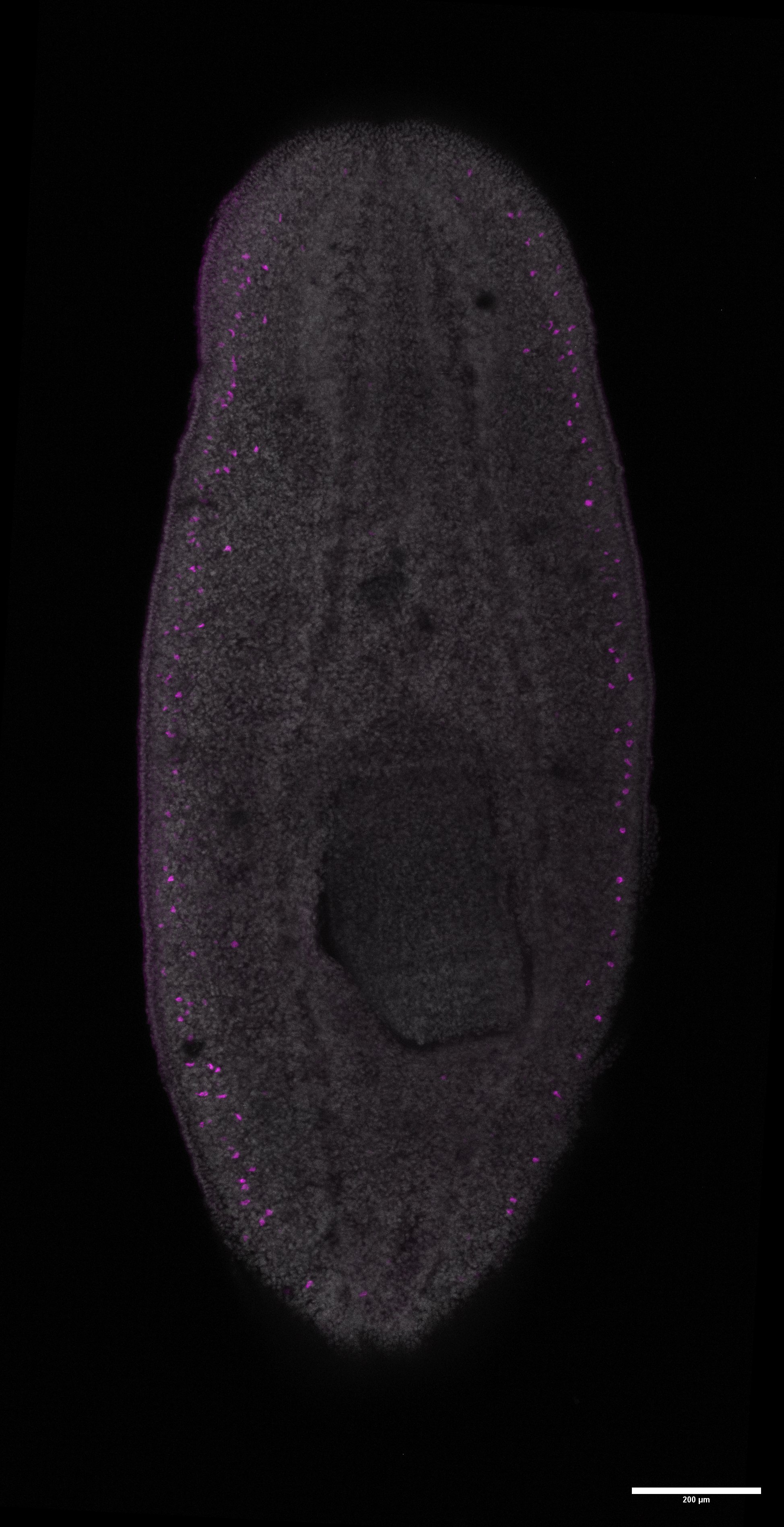

Supplement: Supplementary file 12 — Source data Fig. 5 [file 44318_2025_662_MOESM12_ESM.zip › Figure 5/5D/dd_924/ID_3_Triple_RNAi_Probe_dd924_rhod_DAPI_10x.jpg]

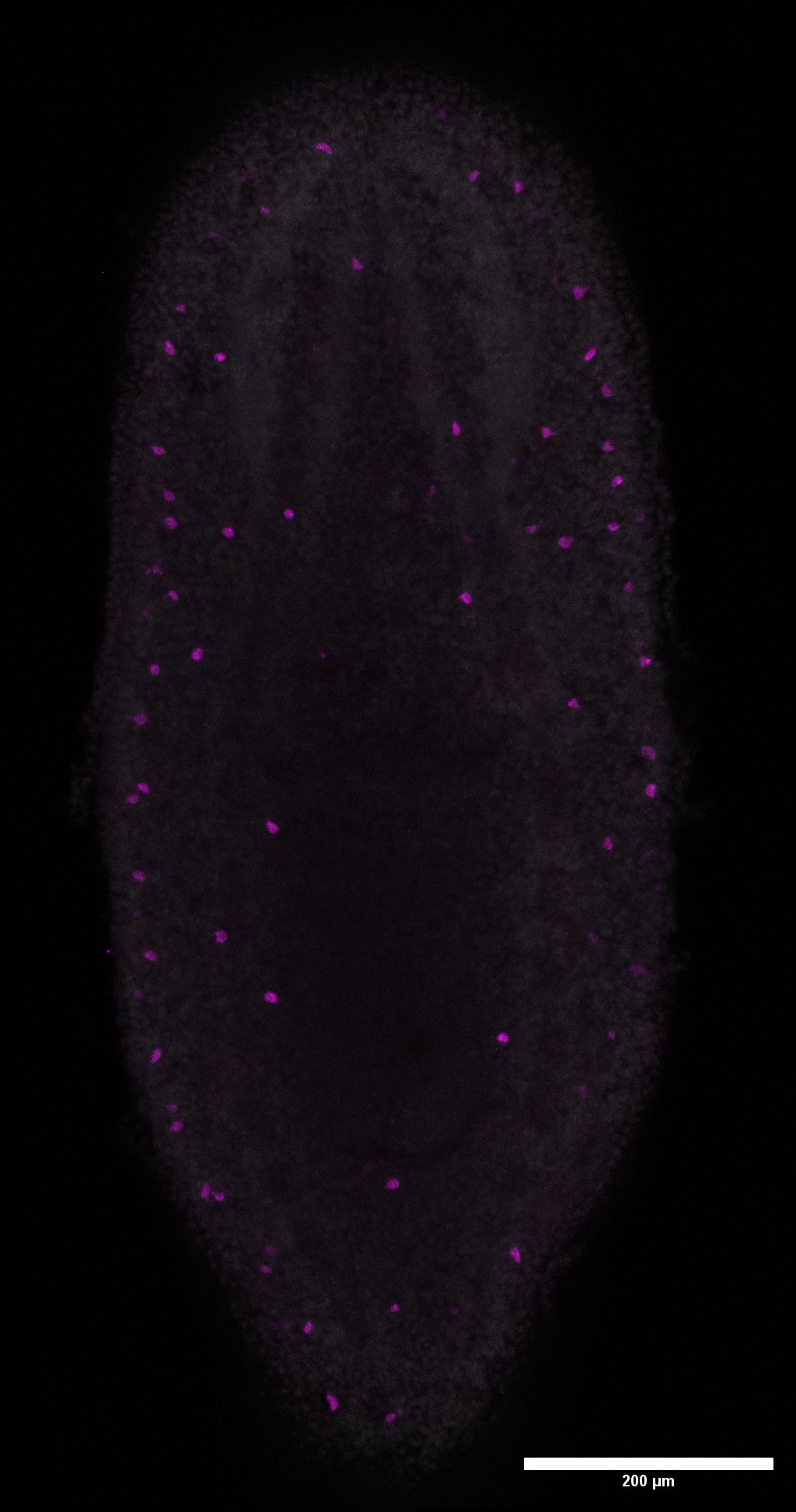

Supplement: Supplementary file 12 — Source data Fig. 5 [file 44318_2025_662_MOESM12_ESM.zip › Figure 5/5D/dd_924/ID_3_ythdf-A_RNAi_Probe_dd924_rhod_DAPI_10x.jpg]

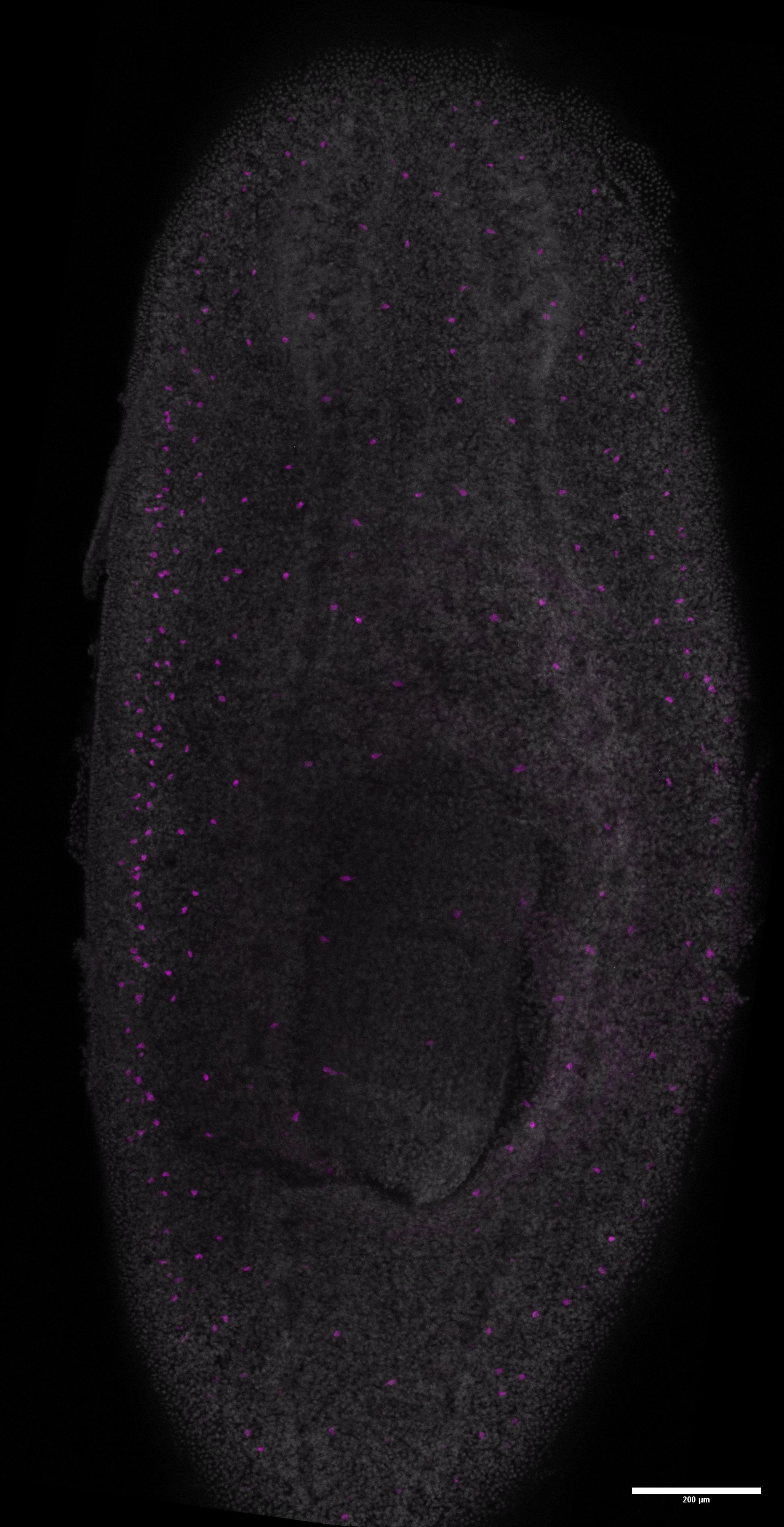

Supplement: Supplementary file 12 — Source data Fig. 5 [file 44318_2025_662_MOESM12_ESM.zip › Figure 5/5D/dd_924/ID_3_ythdf-B_RNAi_Probe_dd924_rhod_DAPI_10x.jpg]

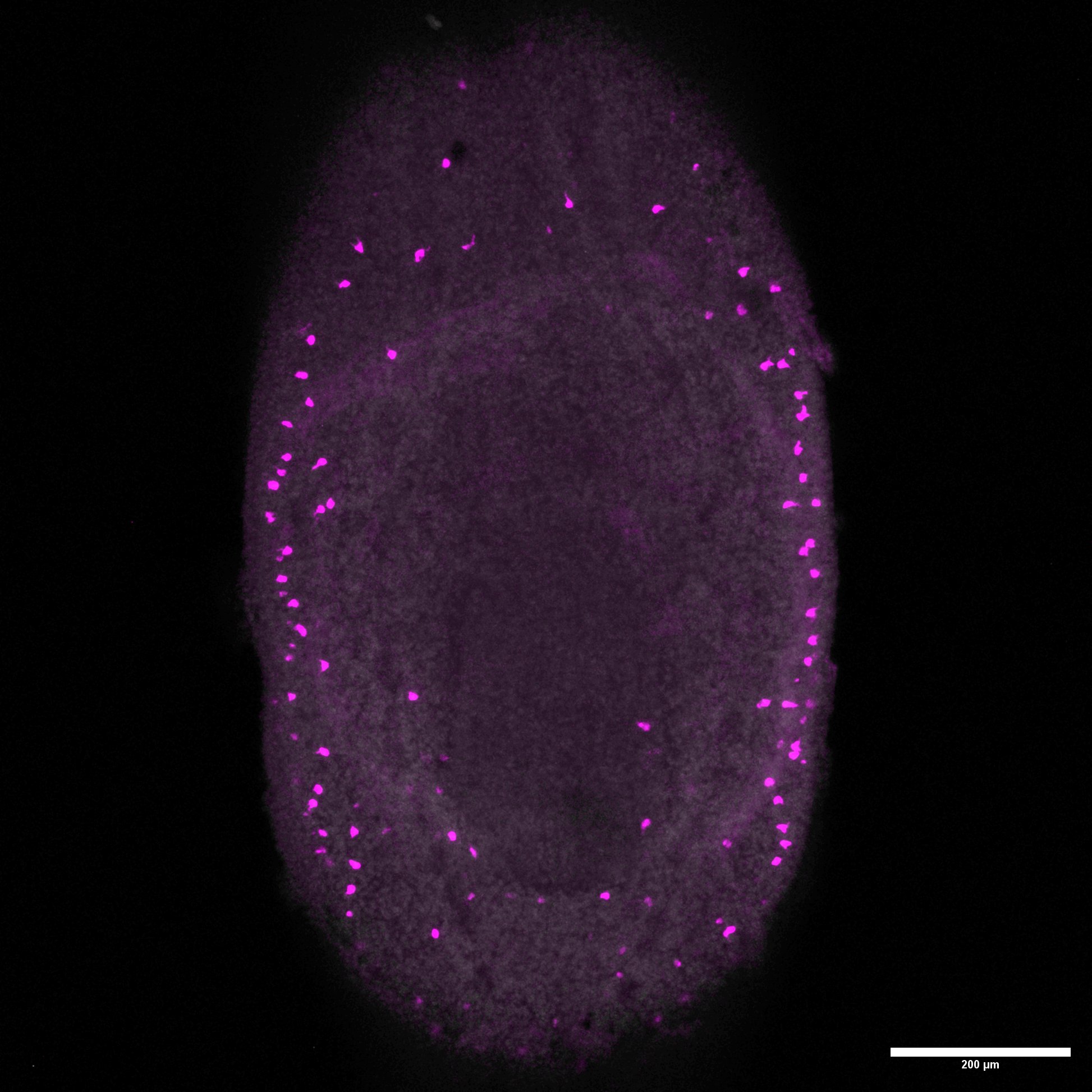

Supplement: Supplementary file 12 — Source data Fig. 5 [file 44318_2025_662_MOESM12_ESM.zip › Figure 5/5D/dd_924/ID_3_ythdf-C_RNAi_Probe_dd924_rhod_DAPI_10x.jpg]

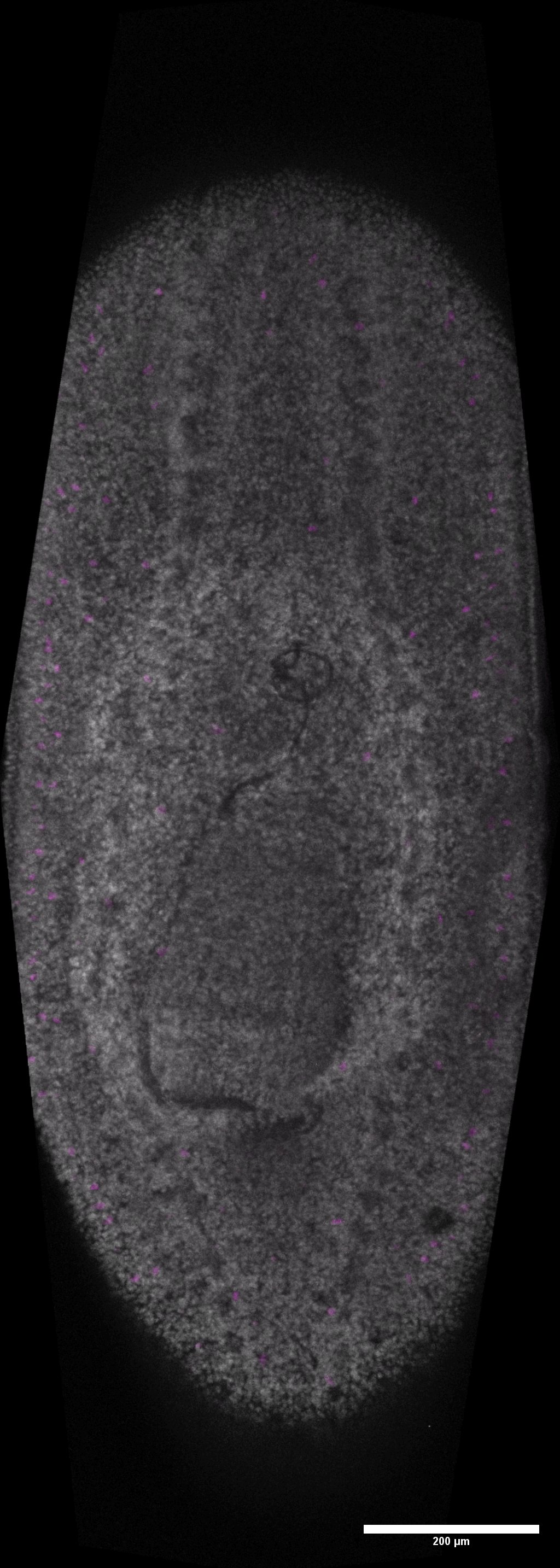

Supplement: Supplementary file 12 — Source data Fig. 5 [file 44318_2025_662_MOESM12_ESM.zip › Figure 5/5D/dd_924/ID_4_Control_RNAi_Probe_dd924_rhod_DAPI_10x.jpg]

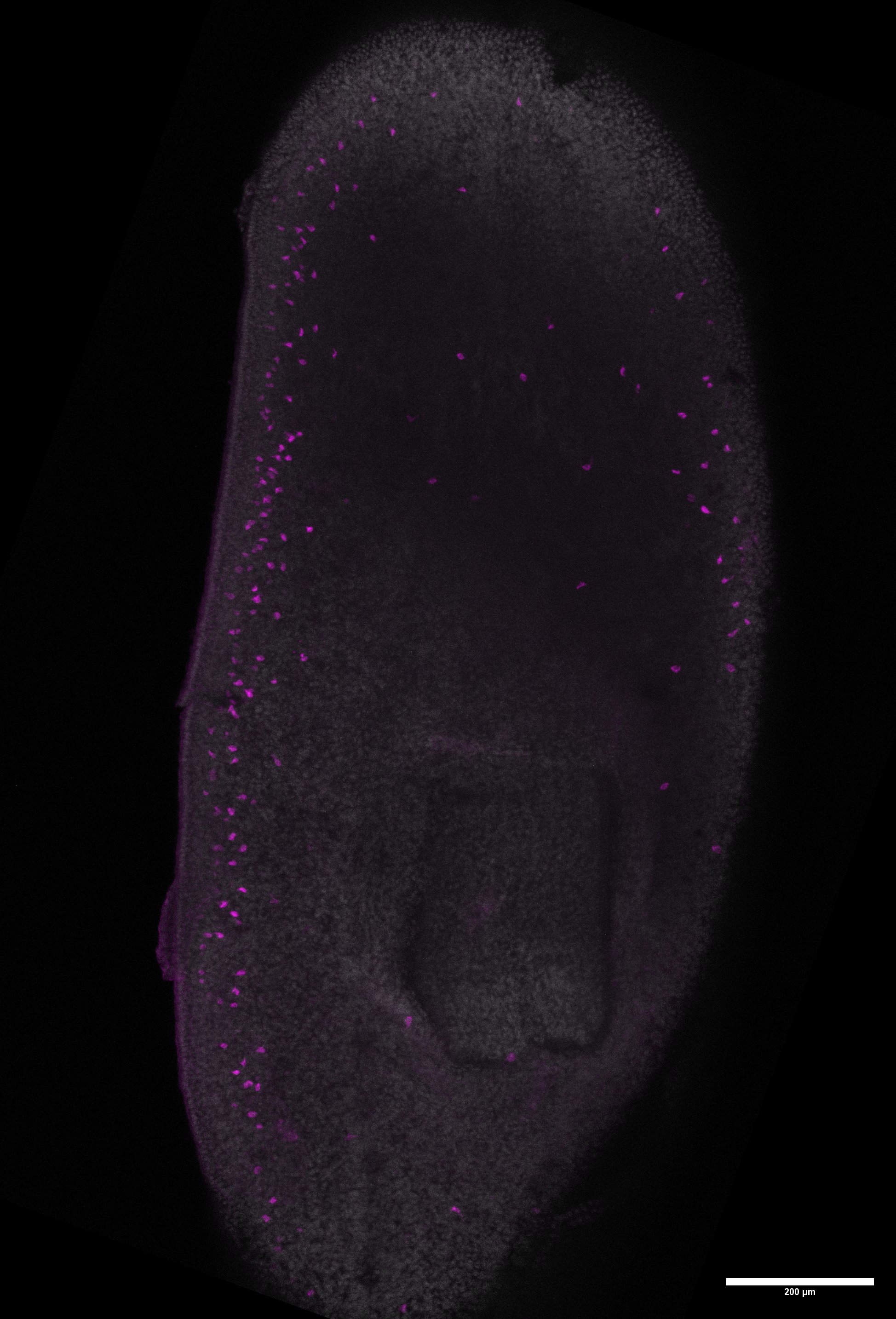

Supplement: Supplementary file 12 — Source data Fig. 5 [file 44318_2025_662_MOESM12_ESM.zip › Figure 5/5D/dd_924/ID_4_Triple_RNAi_Probe_dd924_rhod_DAPI_10x.jpg]

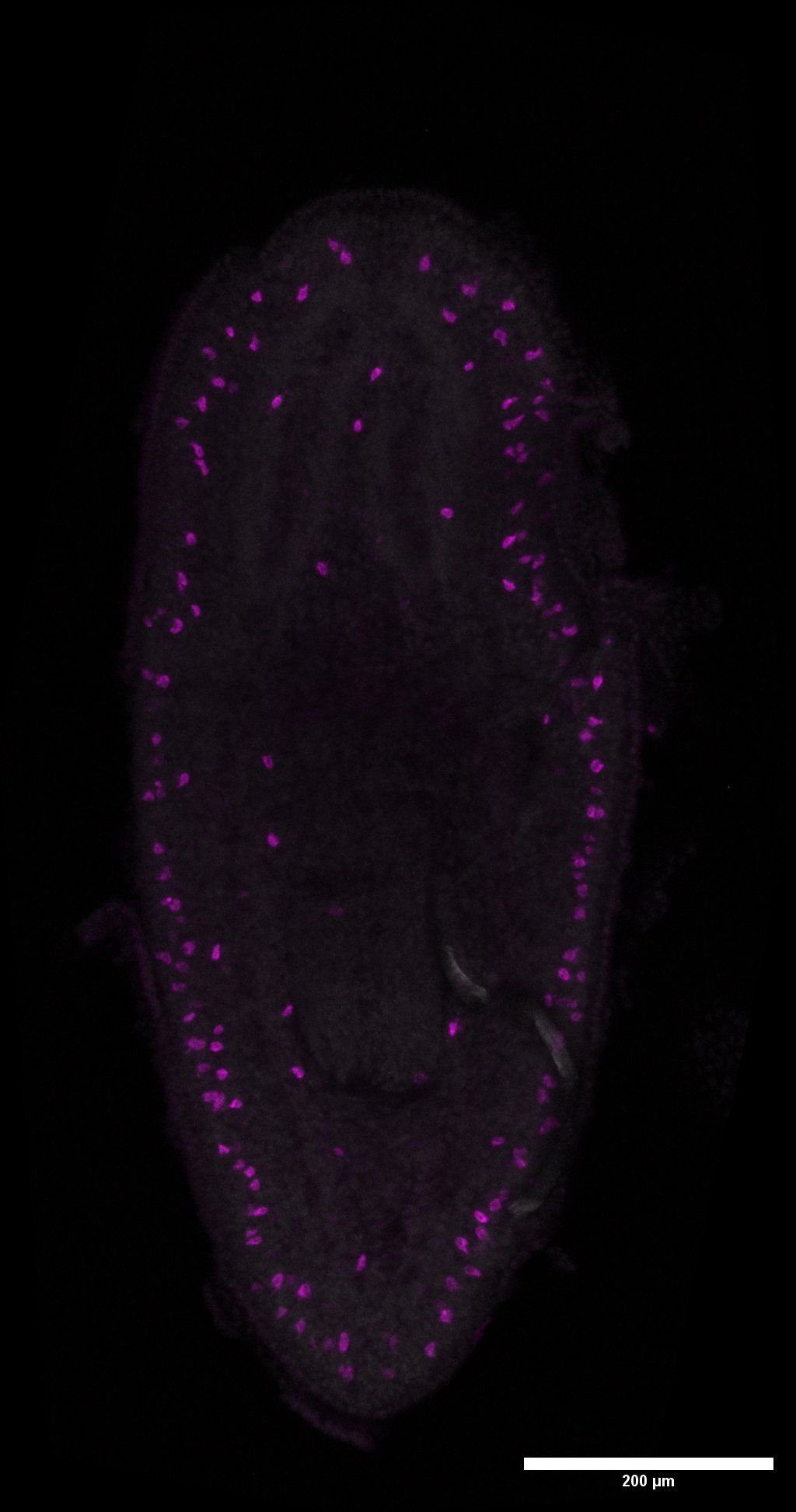

Supplement: Supplementary file 12 — Source data Fig. 5 [file 44318_2025_662_MOESM12_ESM.zip › Figure 5/5D/dd_924/ID_4_ythdf-A_RNAi_Probe_dd924_rhod_DAPI_10x.jpg]

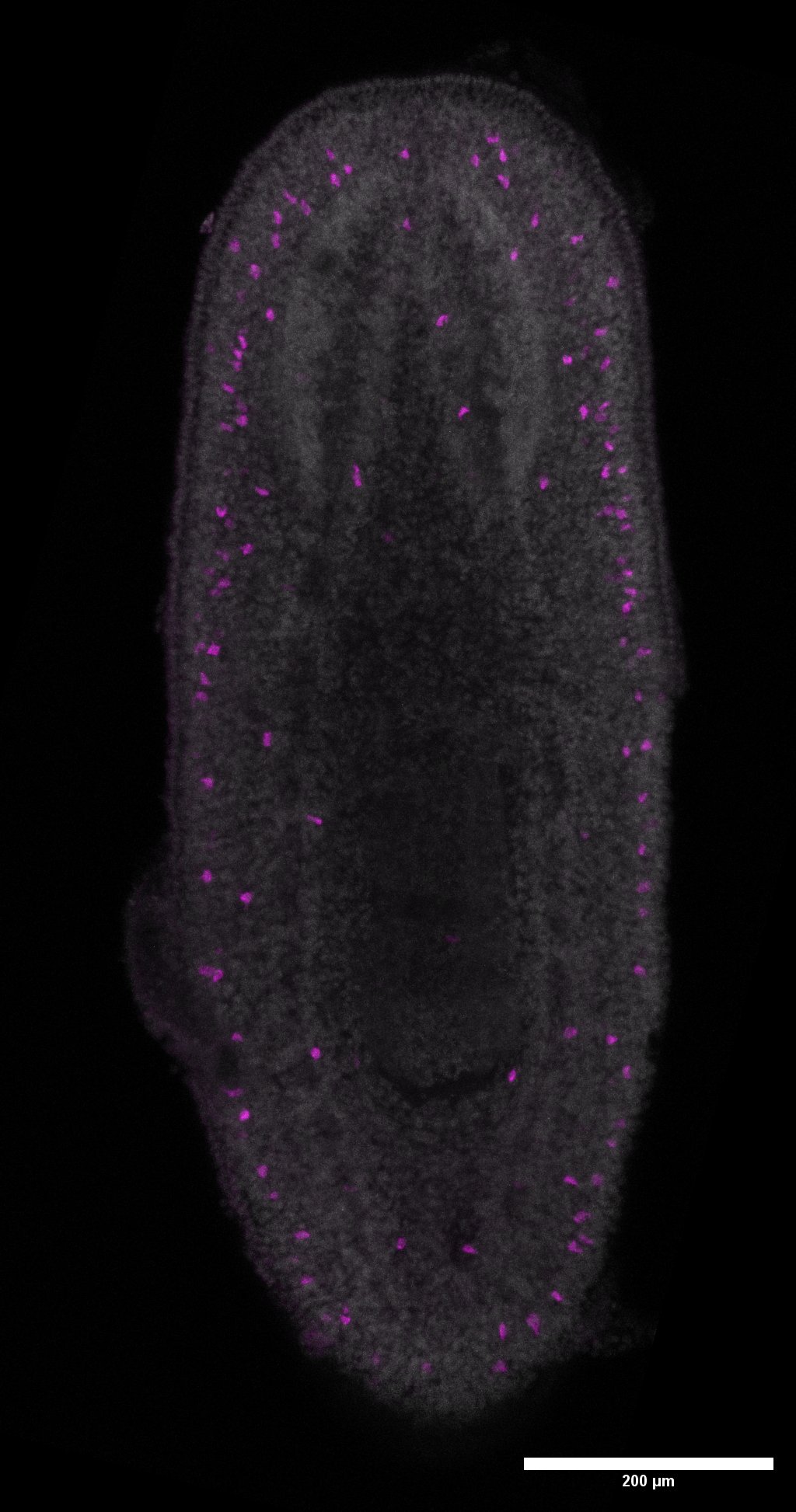

Supplement: Supplementary file 12 — Source data Fig. 5 [file 44318_2025_662_MOESM12_ESM.zip › Figure 5/5D/dd_924/ID_4_ythdf-B_RNAi_Probe_dd924_rhod_DAPI_10x.jpg]

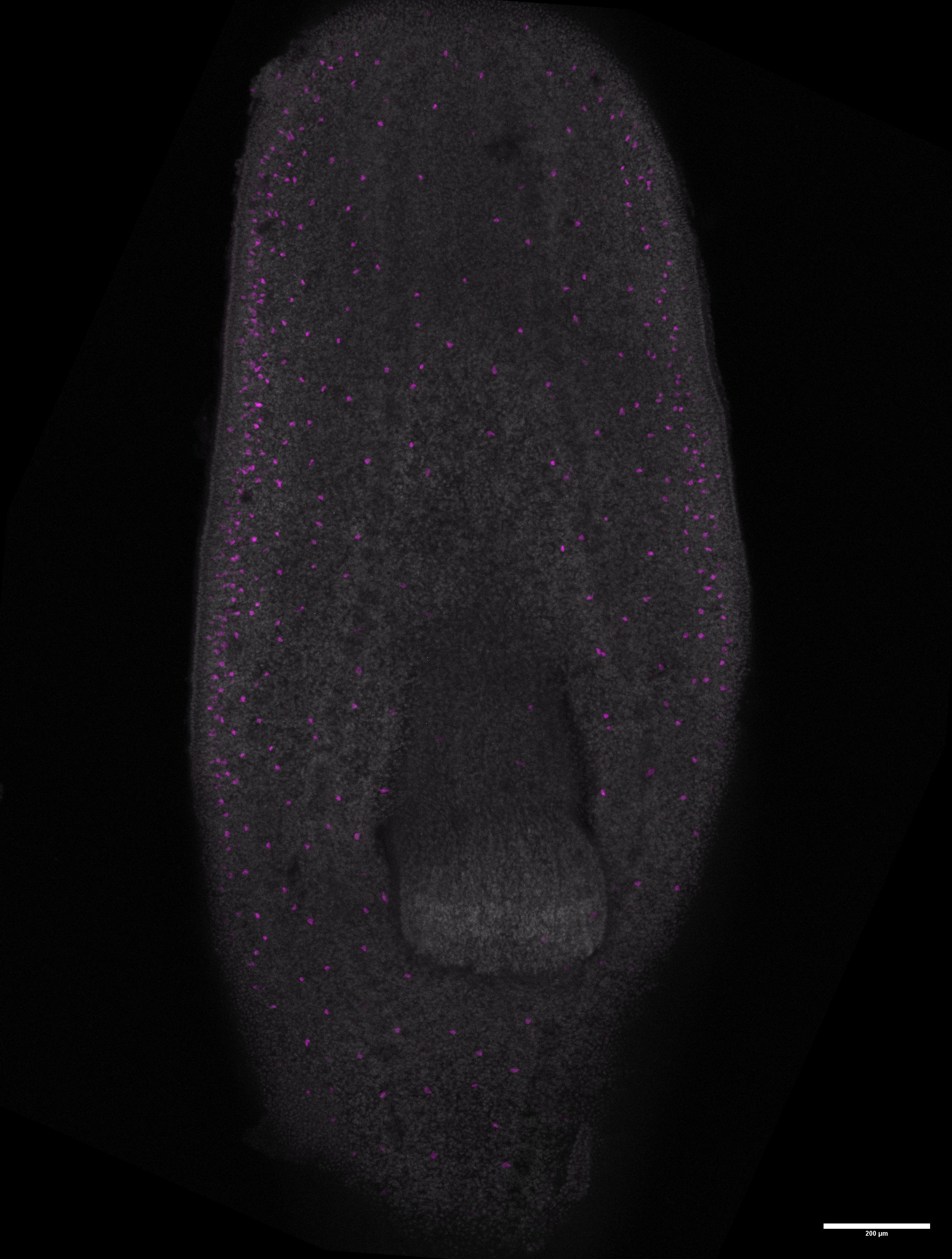

Supplement: Supplementary file 12 — Source data Fig. 5 [file 44318_2025_662_MOESM12_ESM.zip › Figure 5/5D/dd_924/ID_4_ythdf-C_RNAi_Probe_dd924_rhod_DAPI_10x.jpg]

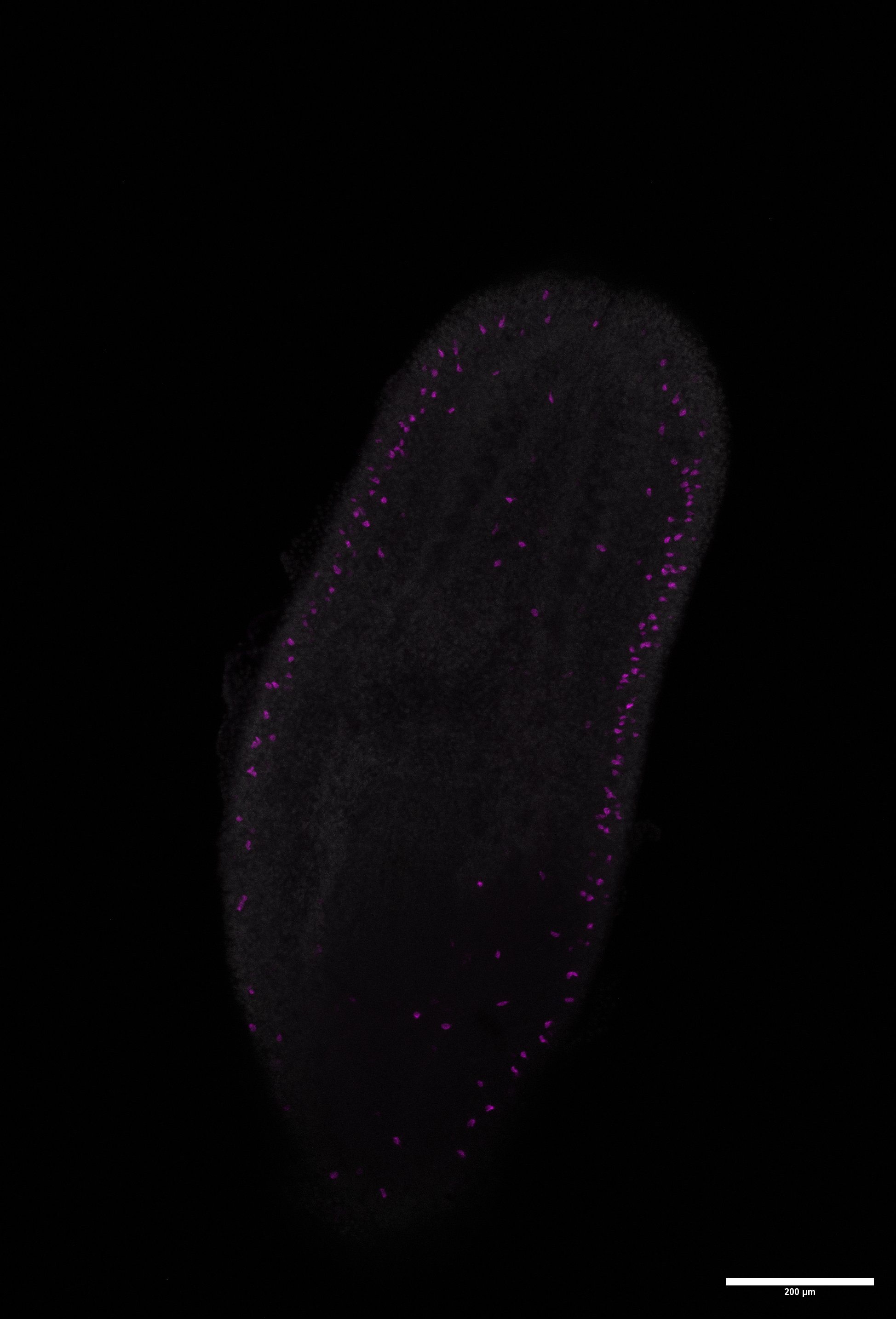

Supplement: Supplementary file 12 — Source data Fig. 5 [file 44318_2025_662_MOESM12_ESM.zip › Figure 5/5D/dd_924/ID_5_Control_RNAi_Probe_dd924_rhod_DAPI_10x.jpg]

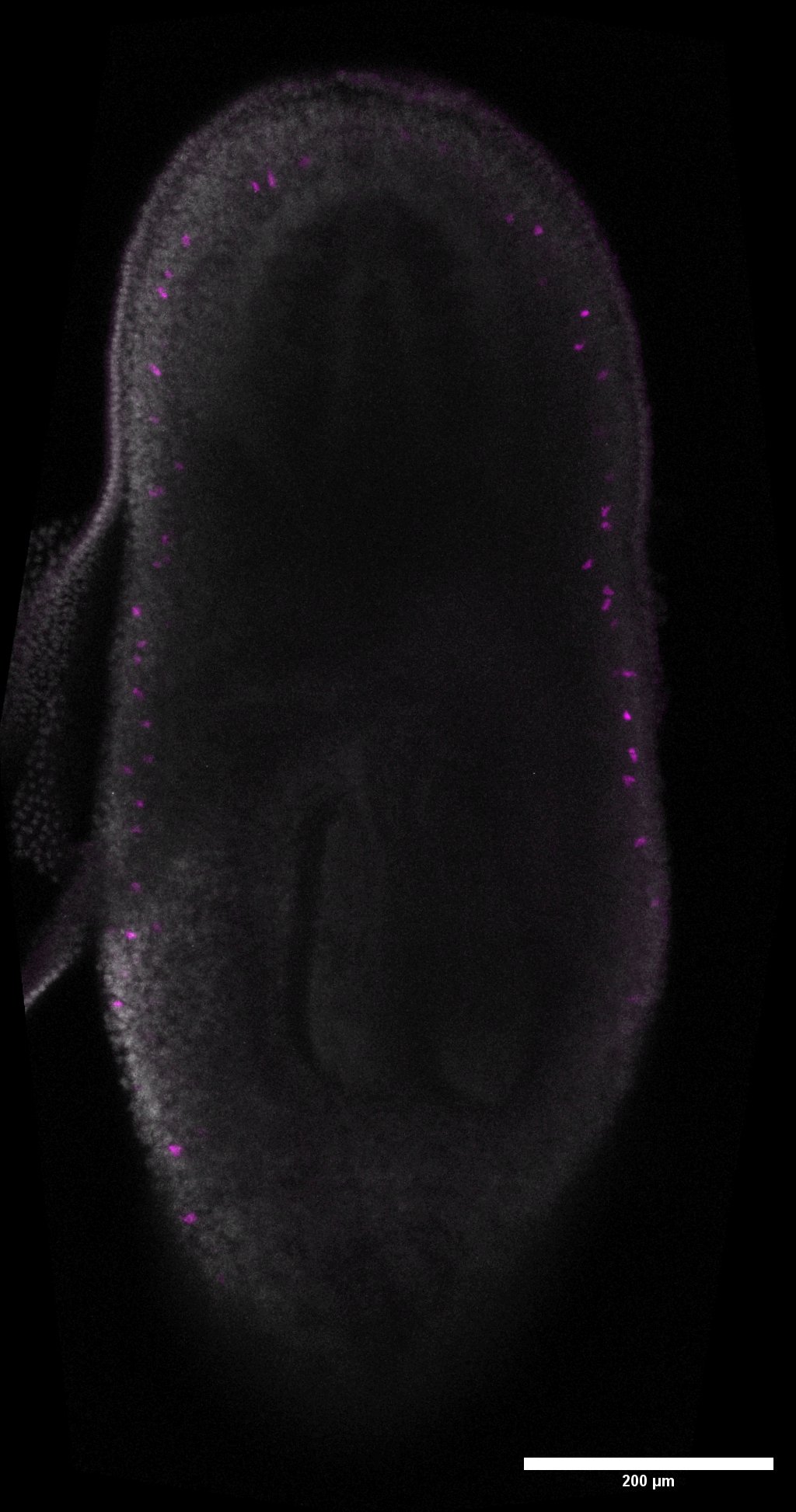

Supplement: Supplementary file 12 — Source data Fig. 5 [file 44318_2025_662_MOESM12_ESM.zip › Figure 5/5D/dd_924/ID_5_Triple_RNAi_Probe_dd924_rhod_DAPI_10x.jpg]

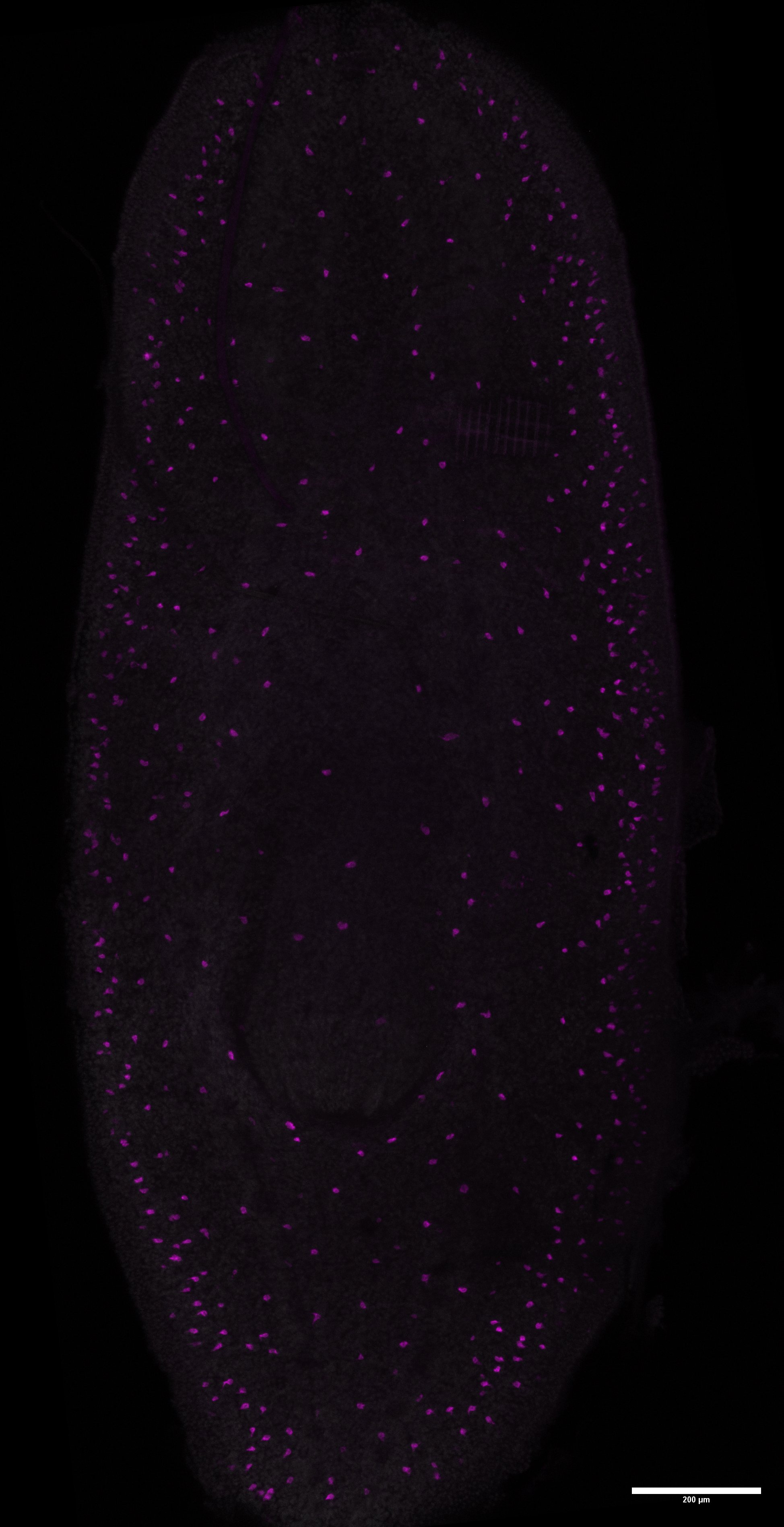

Supplement: Supplementary file 12 — Source data Fig. 5 [file 44318_2025_662_MOESM12_ESM.zip › Figure 5/5D/dd_924/ID_5_ythdf-A_RNAi_Probe_dd924_rhod_DAPI_10x.jpg]

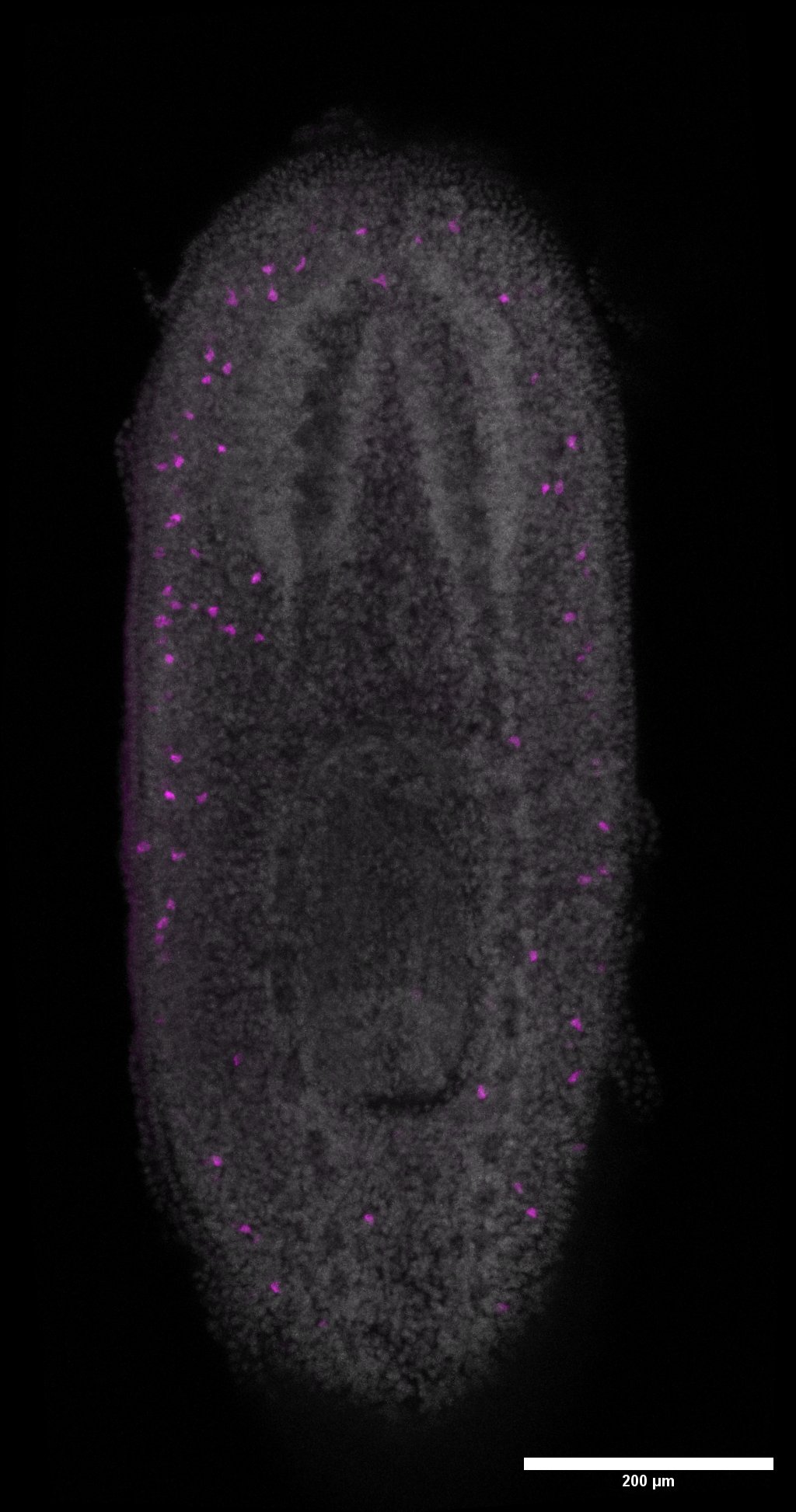

Supplement: Supplementary file 12 — Source data Fig. 5 [file 44318_2025_662_MOESM12_ESM.zip › Figure 5/5D/dd_924/ID_5_ythdf-B_RNAi_Probe_dd924_rhod_DAPI_10x.jpg]

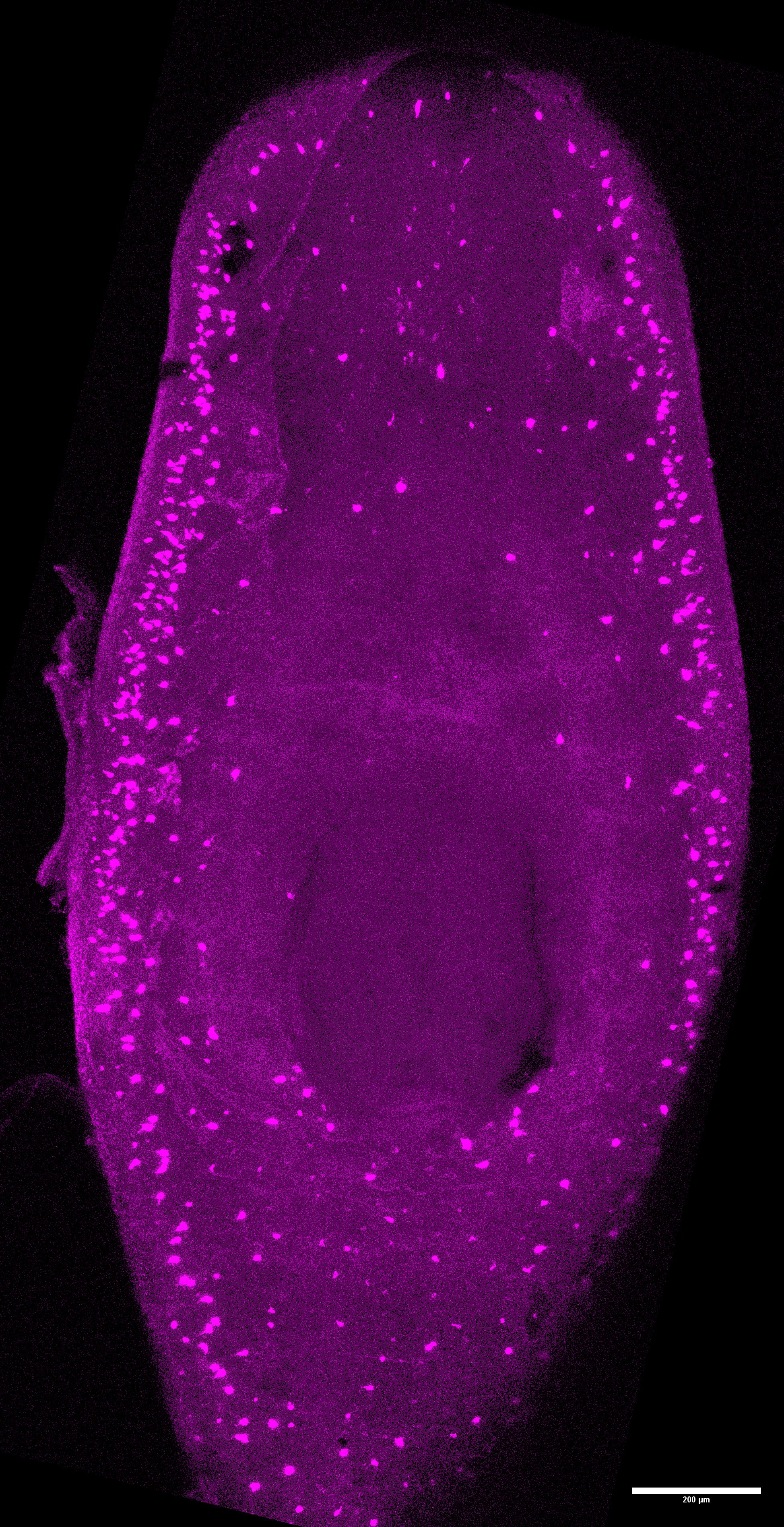

Supplement: Supplementary file 12 — Source data Fig. 5 [file 44318_2025_662_MOESM12_ESM.zip › Figure 5/5D/dd_924/ID_5_ythdf-C_RNAi_Probe_dd924_rhod_DAPI_10x.jpg]

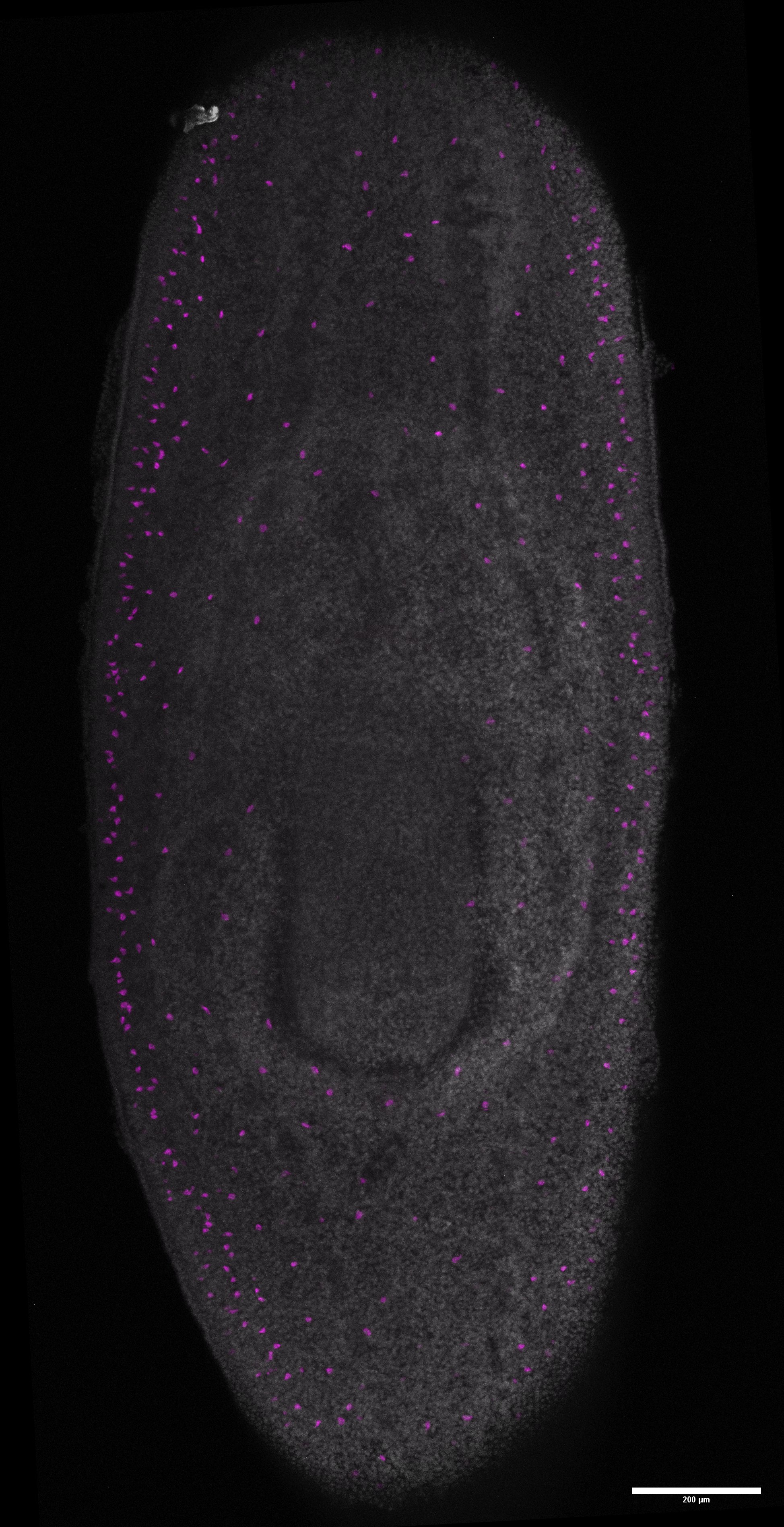

Supplement: Supplementary file 12 — Source data Fig. 5 [file 44318_2025_662_MOESM12_ESM.zip › Figure 5/5D/dd_924/ID_6_Control_RNAi_Probe_dd924_rhod_DAPI_10x.jpg]

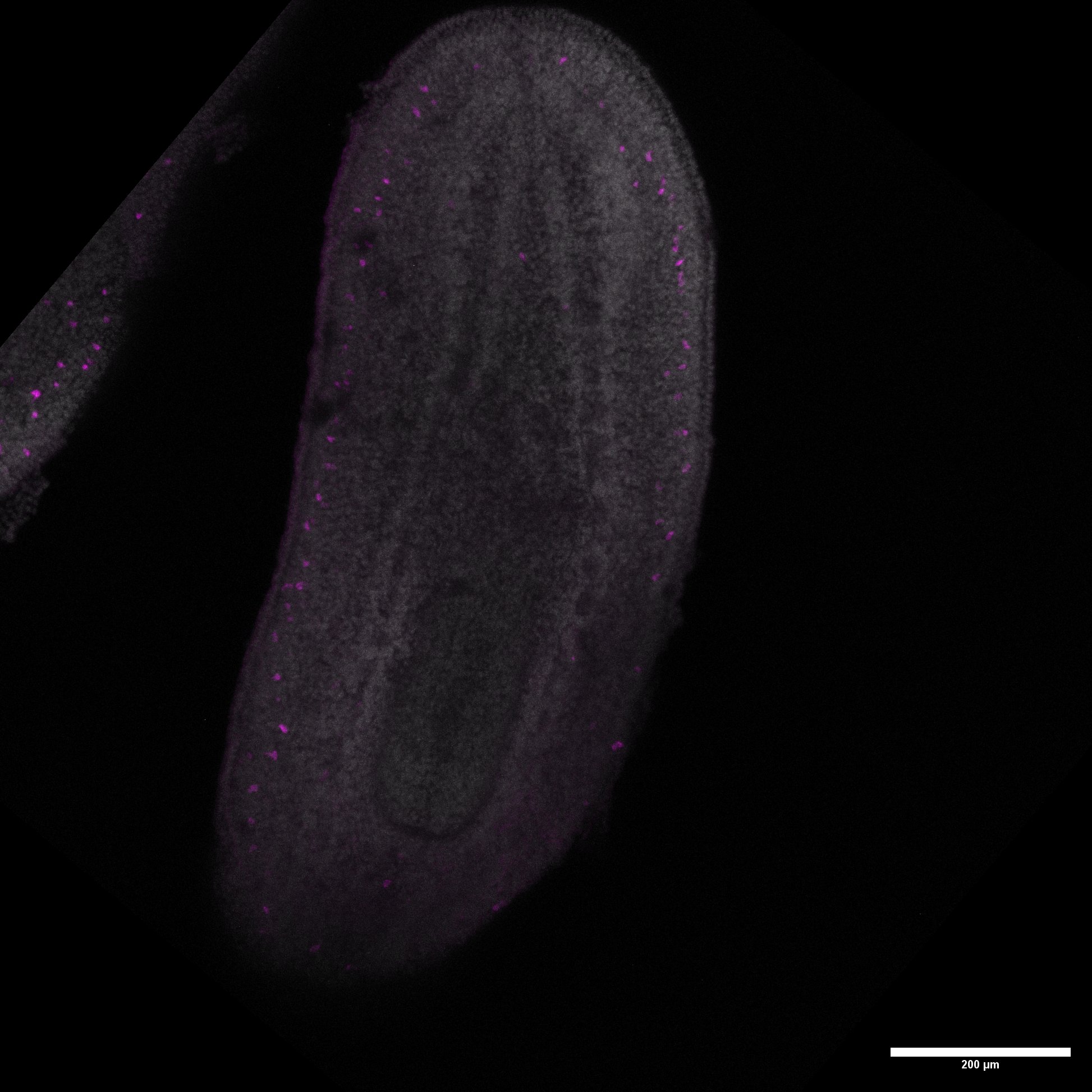

Supplement: Supplementary file 12 — Source data Fig. 5 [file 44318_2025_662_MOESM12_ESM.zip › Figure 5/5D/dd_924/ID_6_Triple_RNAi_Probe_dd924_rhod_DAPI_10x.jpg]

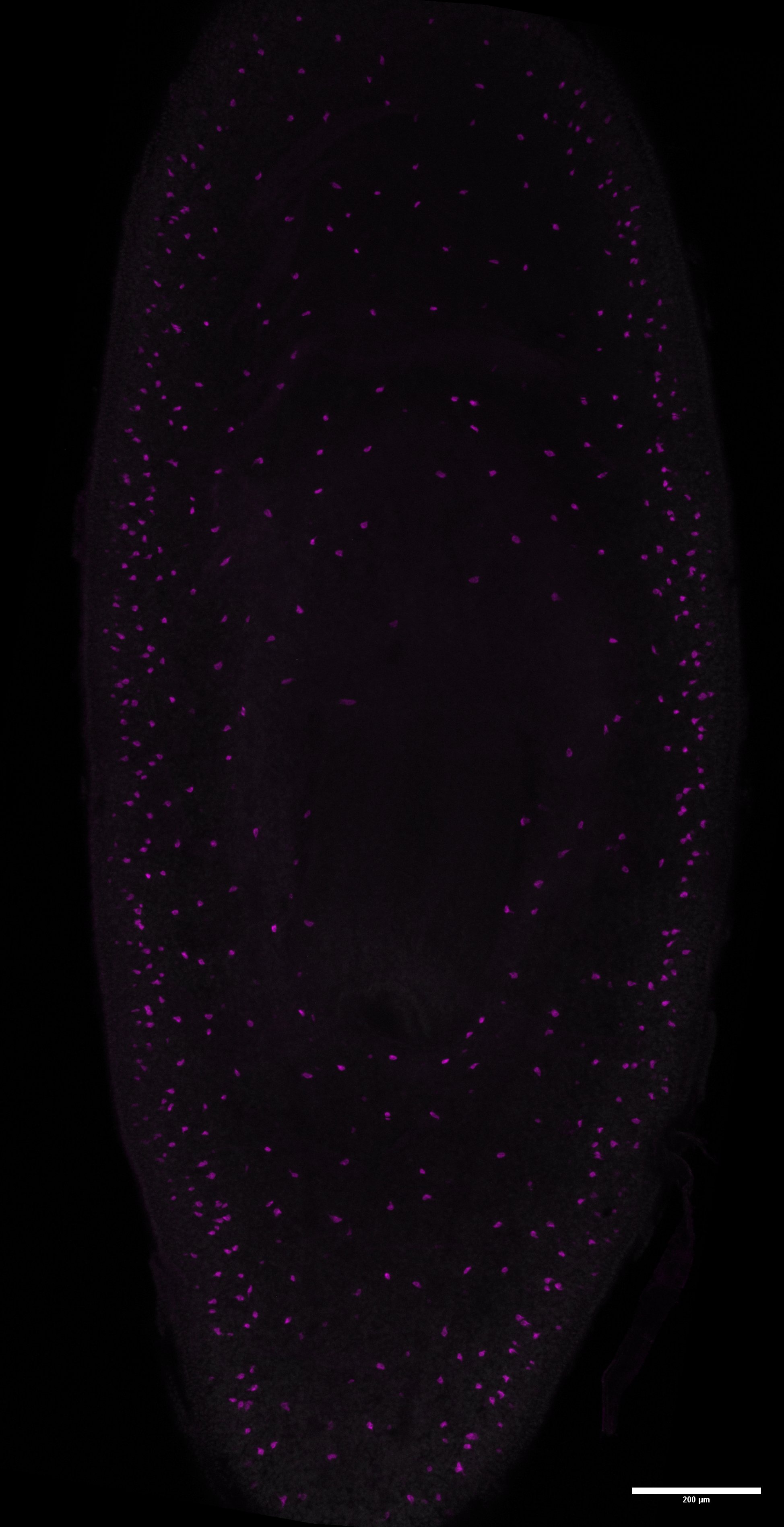

Supplement: Supplementary file 12 — Source data Fig. 5 [file 44318_2025_662_MOESM12_ESM.zip › Figure 5/5D/dd_924/ID_6_ythdf-A_RNAi_Probe_dd924_rhod_DAPI_10x.jpg]

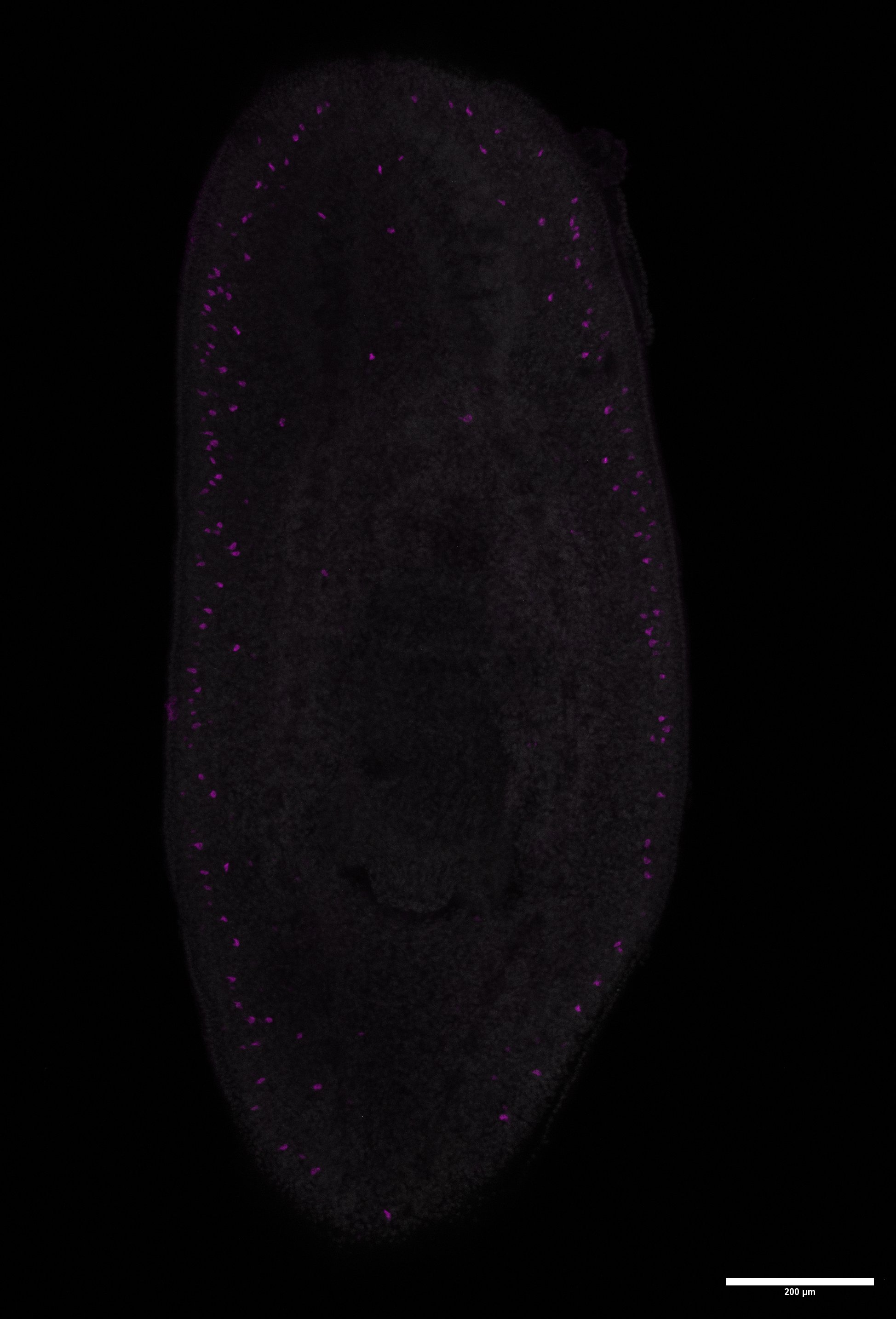

Supplement: Supplementary file 12 — Source data Fig. 5 [file 44318_2025_662_MOESM12_ESM.zip › Figure 5/5D/dd_924/ID_6_ythdf-B_RNAi_Probe_dd924_rhod_DAPI_10x.jpg]

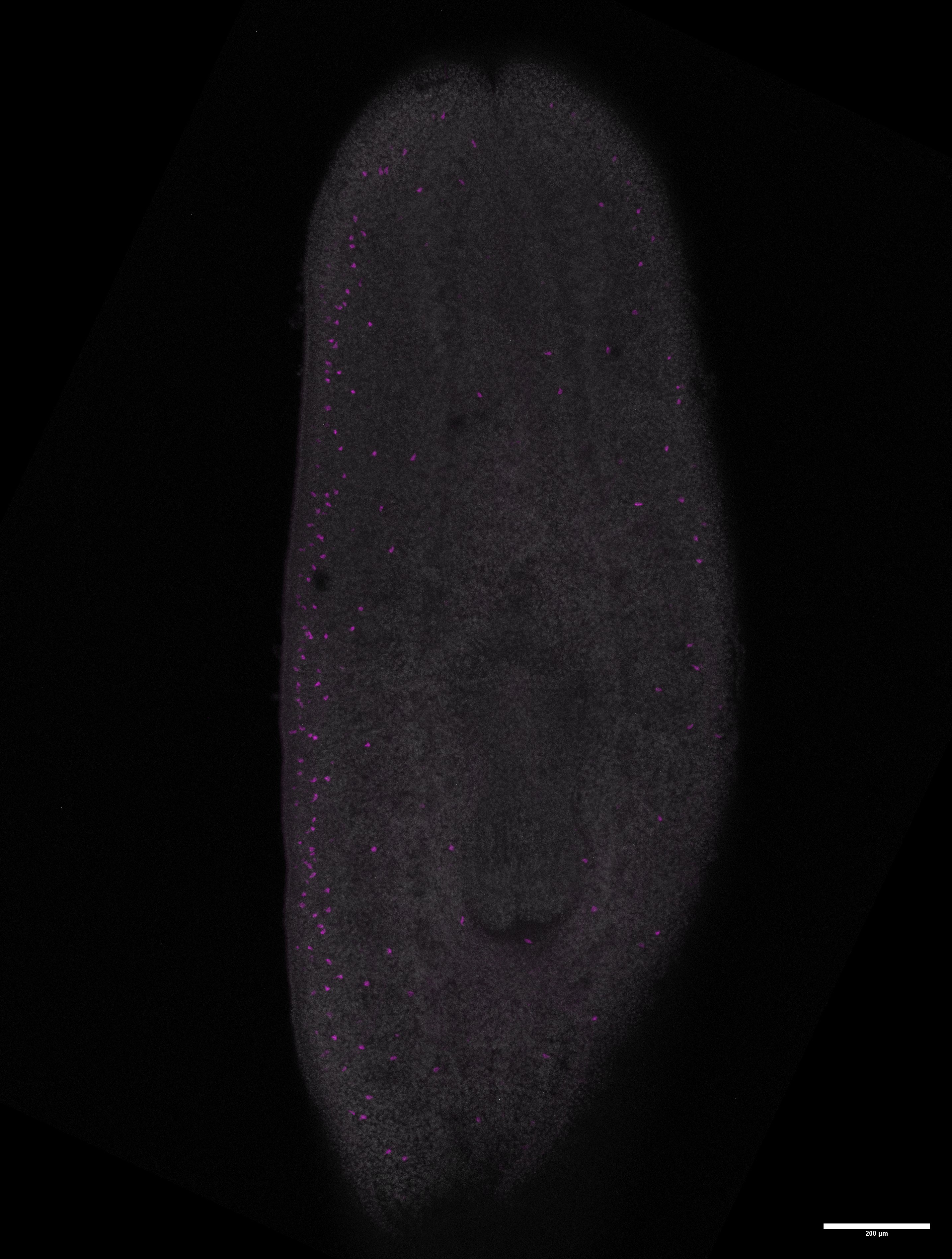

Supplement: Supplementary file 12 — Source data Fig. 5 [file 44318_2025_662_MOESM12_ESM.zip › Figure 5/5D/dd_924/ID_6_ythdf-C_RNAi_Probe_dd924_rhod_DAPI_10x.jpg]

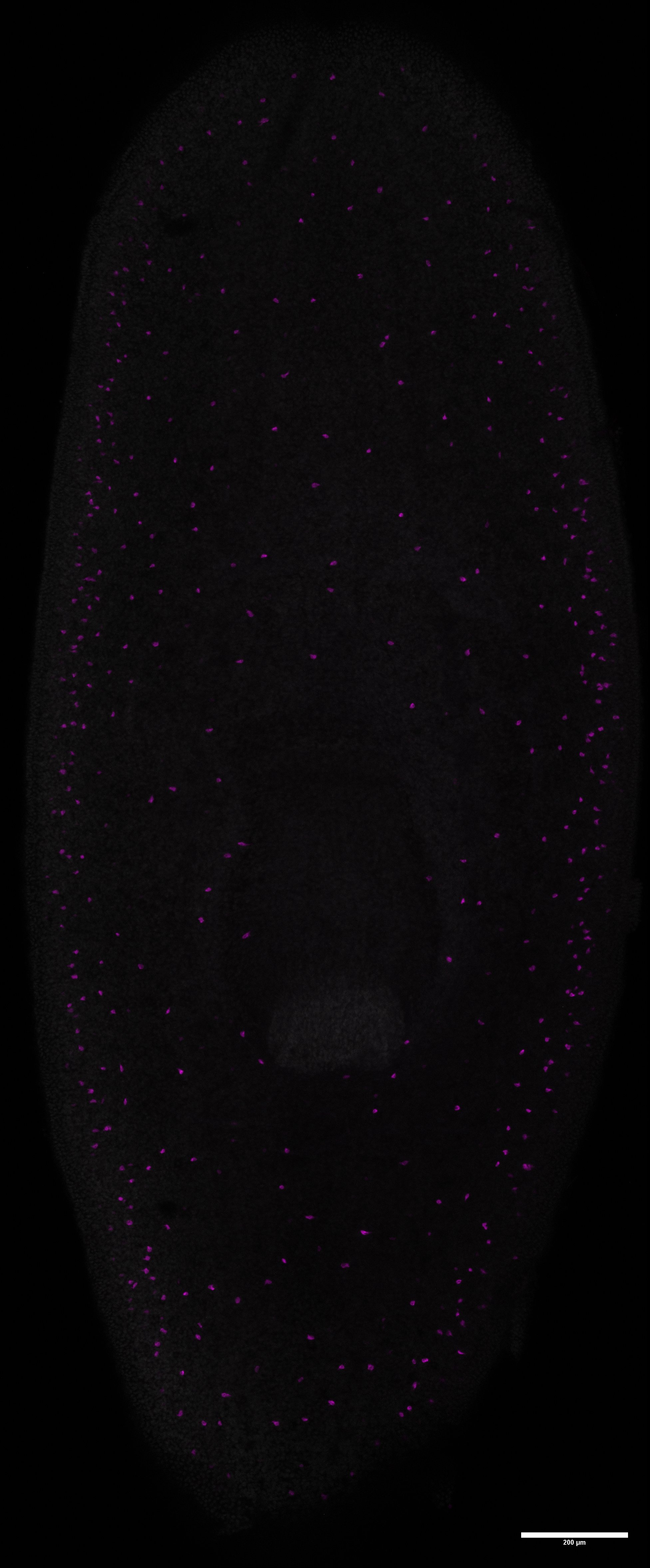

Supplement: Supplementary file 12 — Source data Fig. 5 [file 44318_2025_662_MOESM12_ESM.zip › Figure 5/5D/dd_924/ID_7_Control_RNAi_Probe_dd924_rhod_DAPI_10x.jpg]

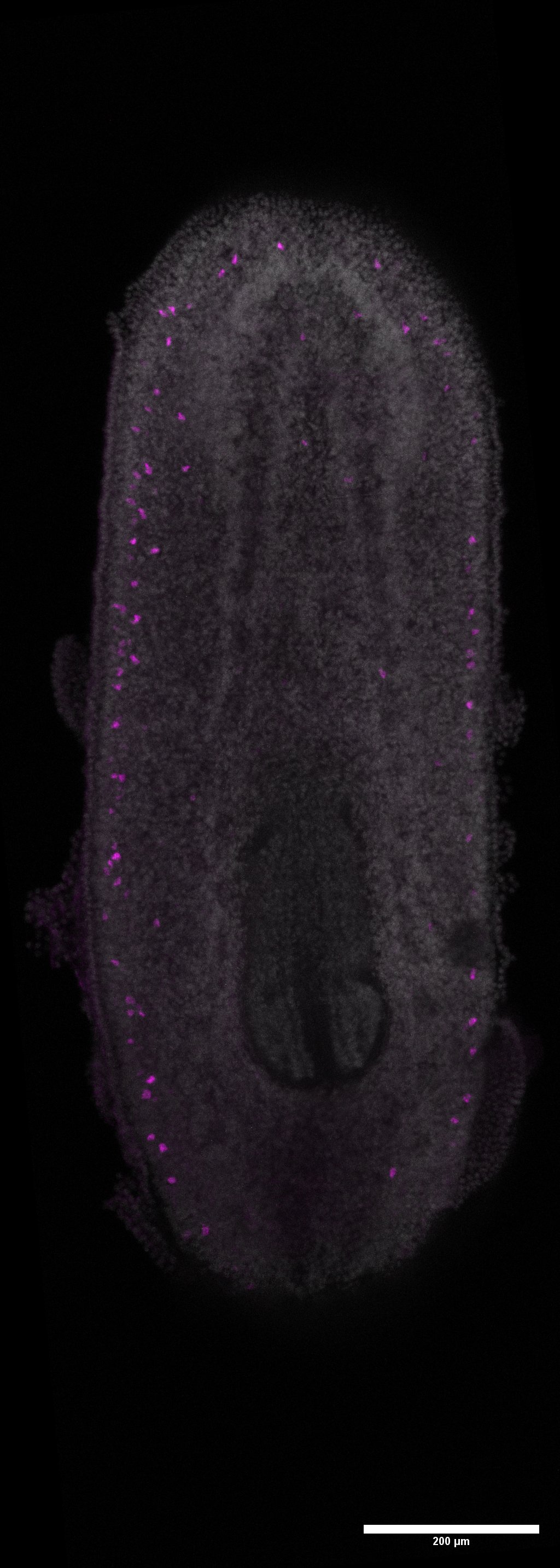

Supplement: Supplementary file 12 — Source data Fig. 5 [file 44318_2025_662_MOESM12_ESM.zip › Figure 5/5D/dd_924/ID_7_Triple_RNAi_Probe_dd924_rhod_DAPI_10x.jpg]

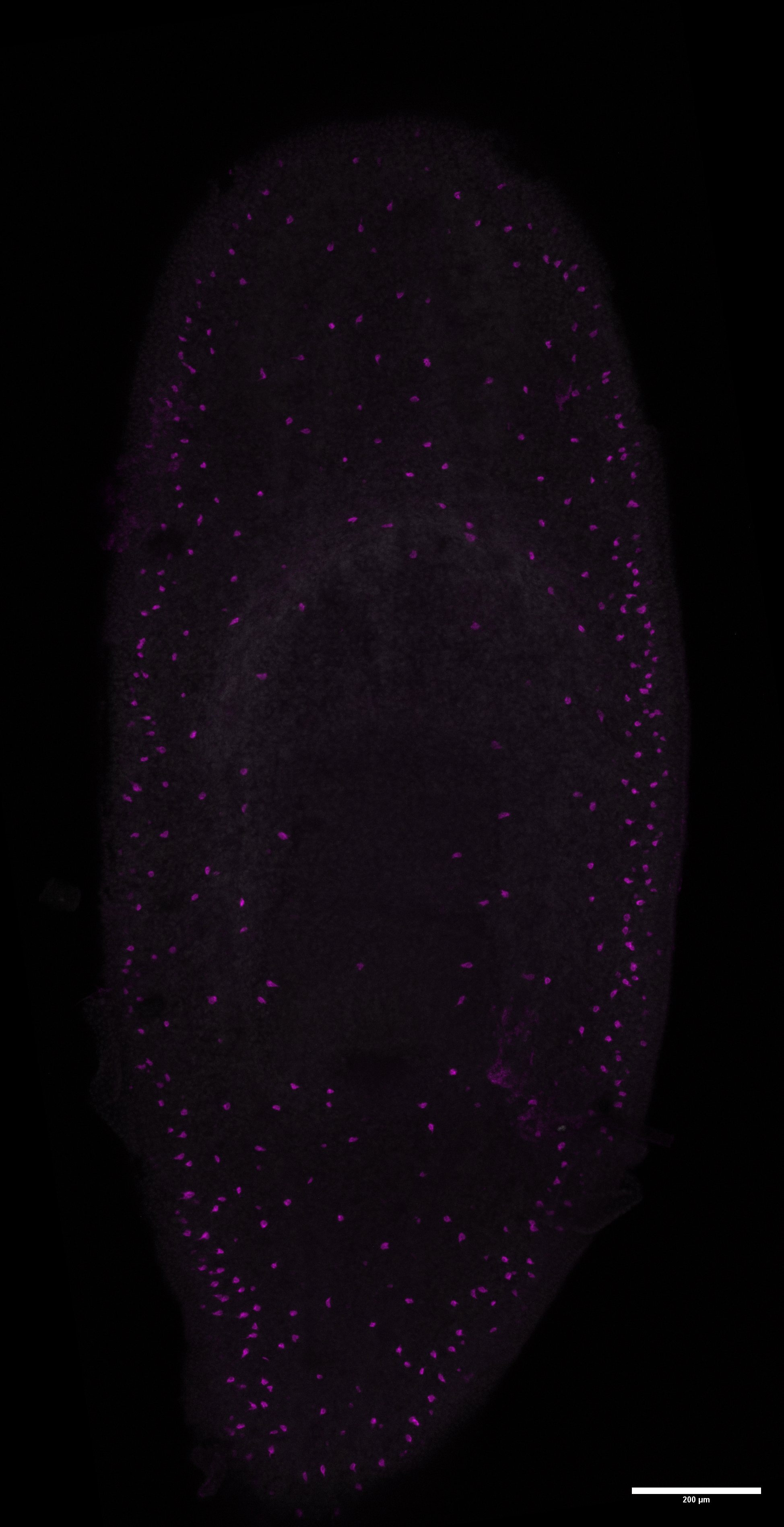

Supplement: Supplementary file 12 — Source data Fig. 5 [file 44318_2025_662_MOESM12_ESM.zip › Figure 5/5D/dd_924/ID_7_ythdf-A_RNAi_Probe_dd924_rhod_DAPI_10x.jpg]

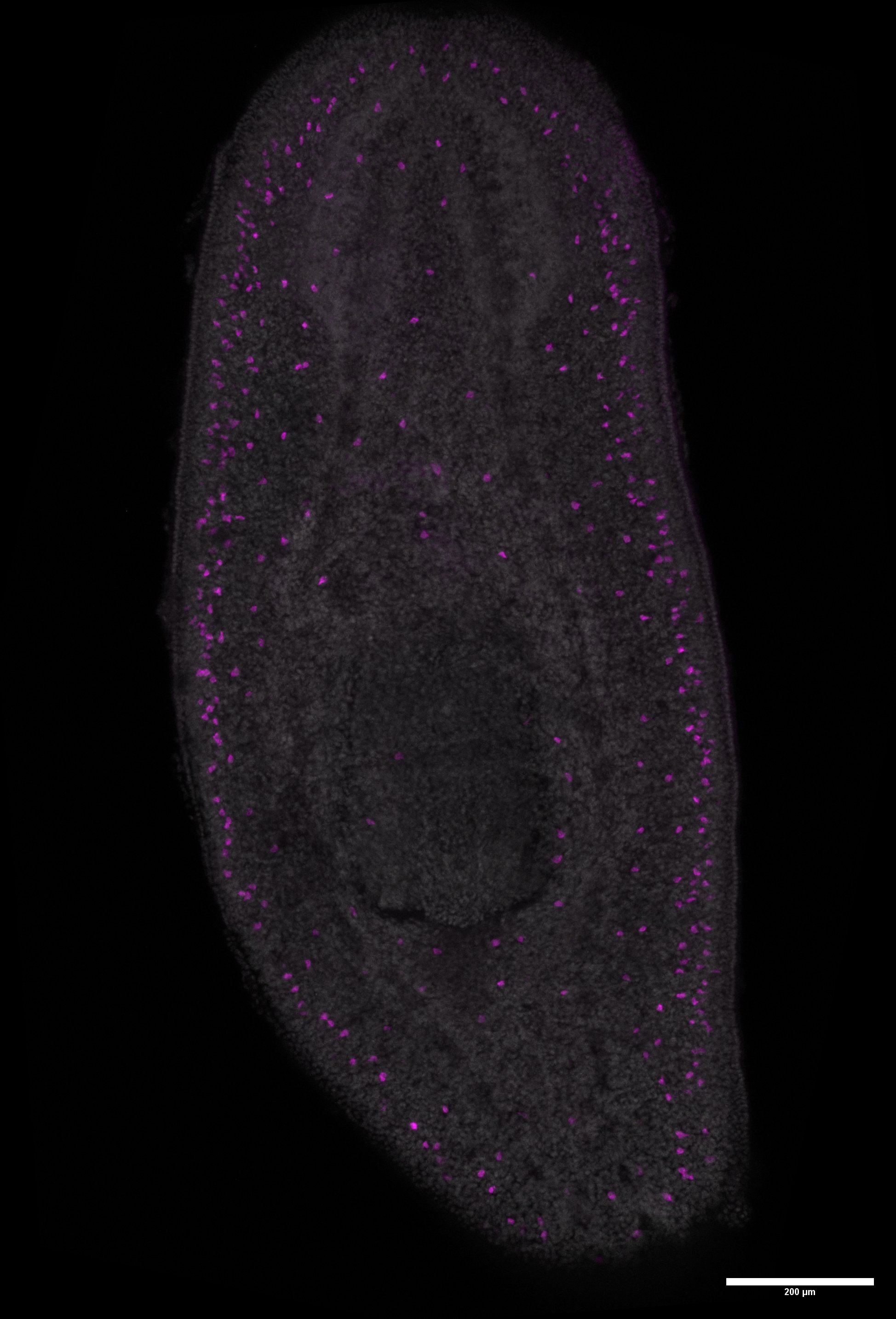

Supplement: Supplementary file 12 — Source data Fig. 5 [file 44318_2025_662_MOESM12_ESM.zip › Figure 5/5D/dd_924/ID_7_ythdf-B_RNAi_Probe_dd924_rhod_DAPI_10x.jpg]

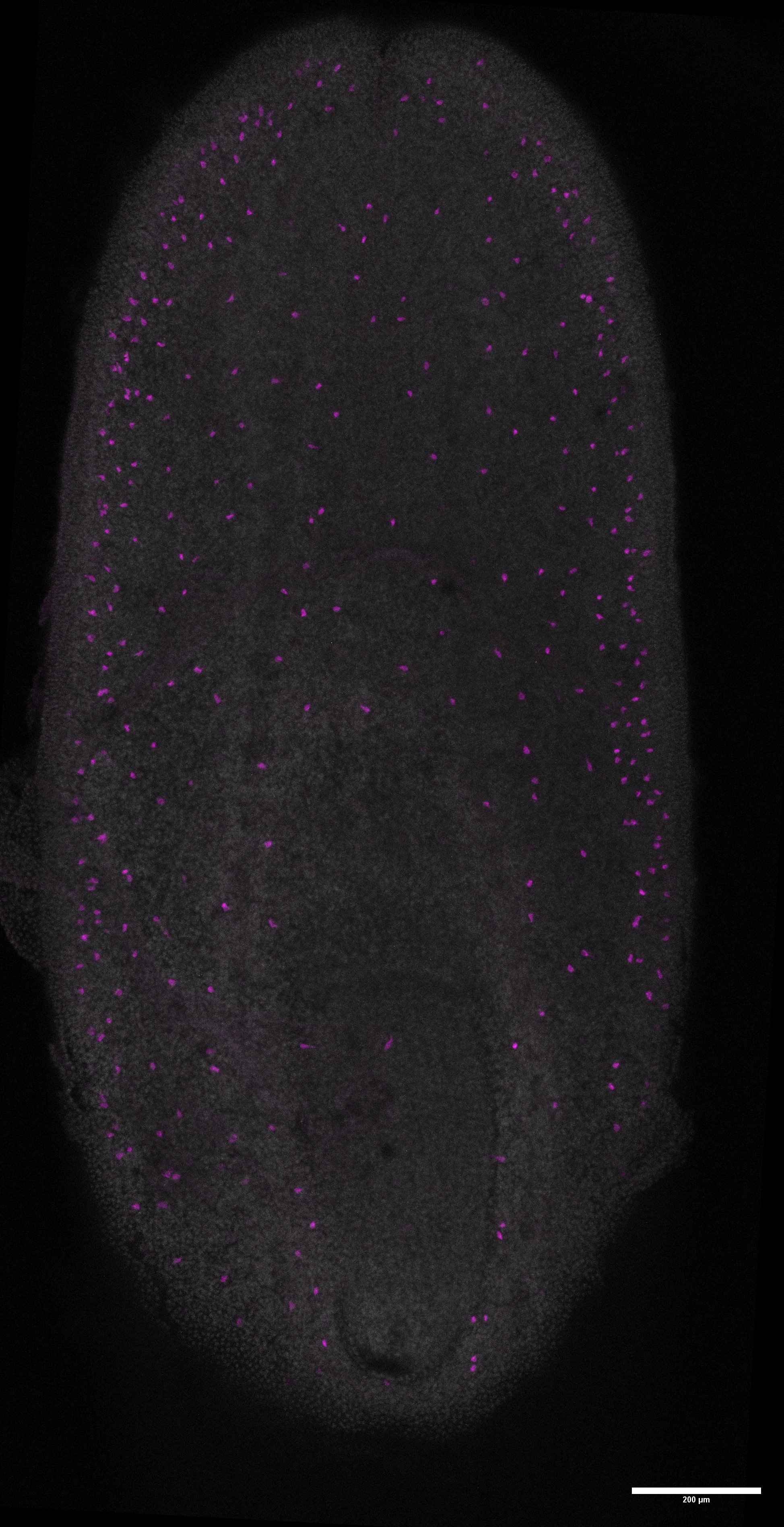

Supplement: Supplementary file 12 — Source data Fig. 5 [file 44318_2025_662_MOESM12_ESM.zip › Figure 5/5D/dd_924/ID_7_ythdf-C_RNAi_Probe_dd924_rhod_DAPI_10x.jpg]

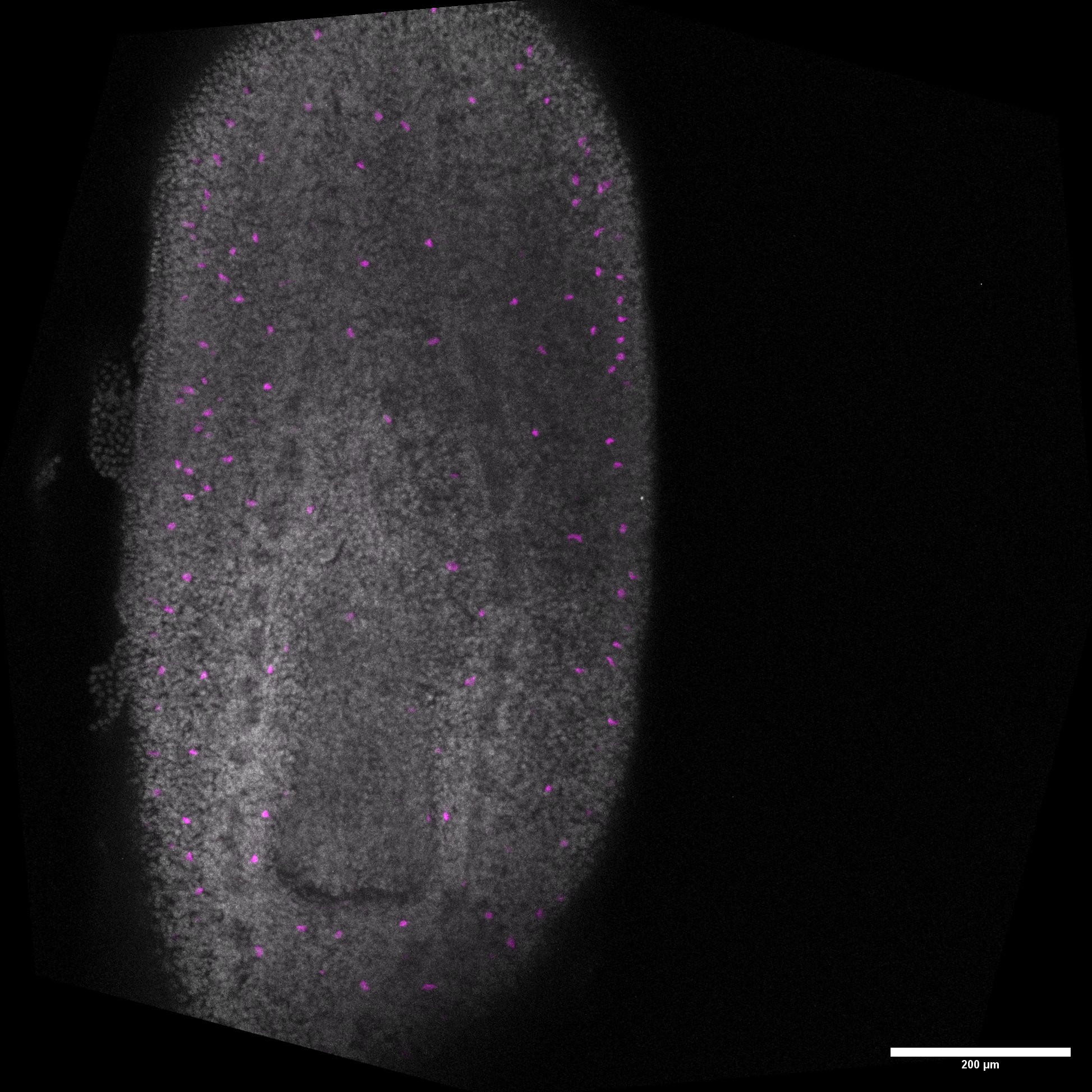

Supplement: Supplementary file 12 — Source data Fig. 5 [file 44318_2025_662_MOESM12_ESM.zip › Figure 5/5D/dd_924/ID_8_Control_RNAi_Probe_dd924_rhod_DAPI_10x.jpg]

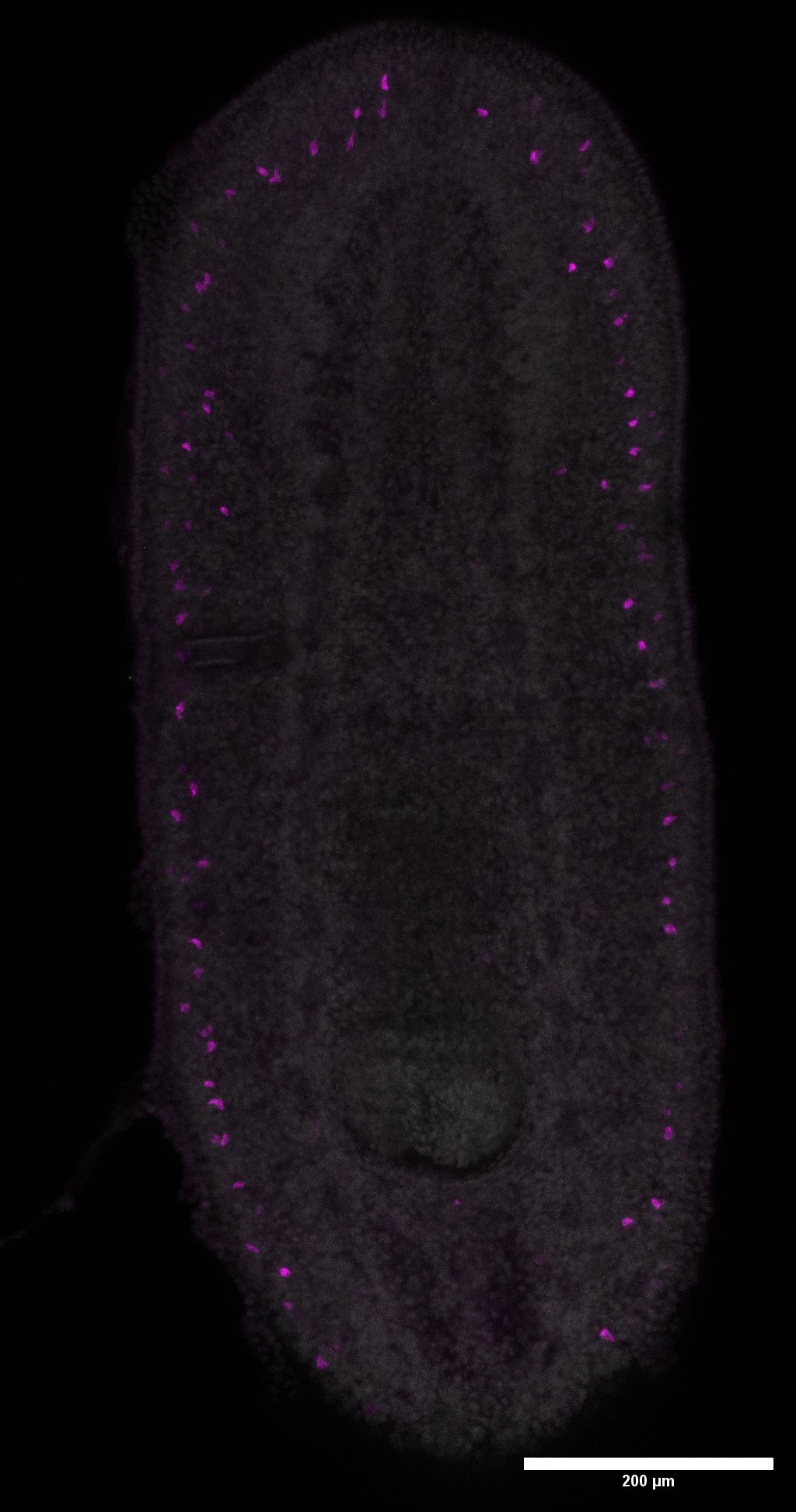

Supplement: Supplementary file 12 — Source data Fig. 5 [file 44318_2025_662_MOESM12_ESM.zip › Figure 5/5D/dd_924/ID_8_Triple_RNAi_Probe_dd924_rhod_DAPI_10x.jpg]

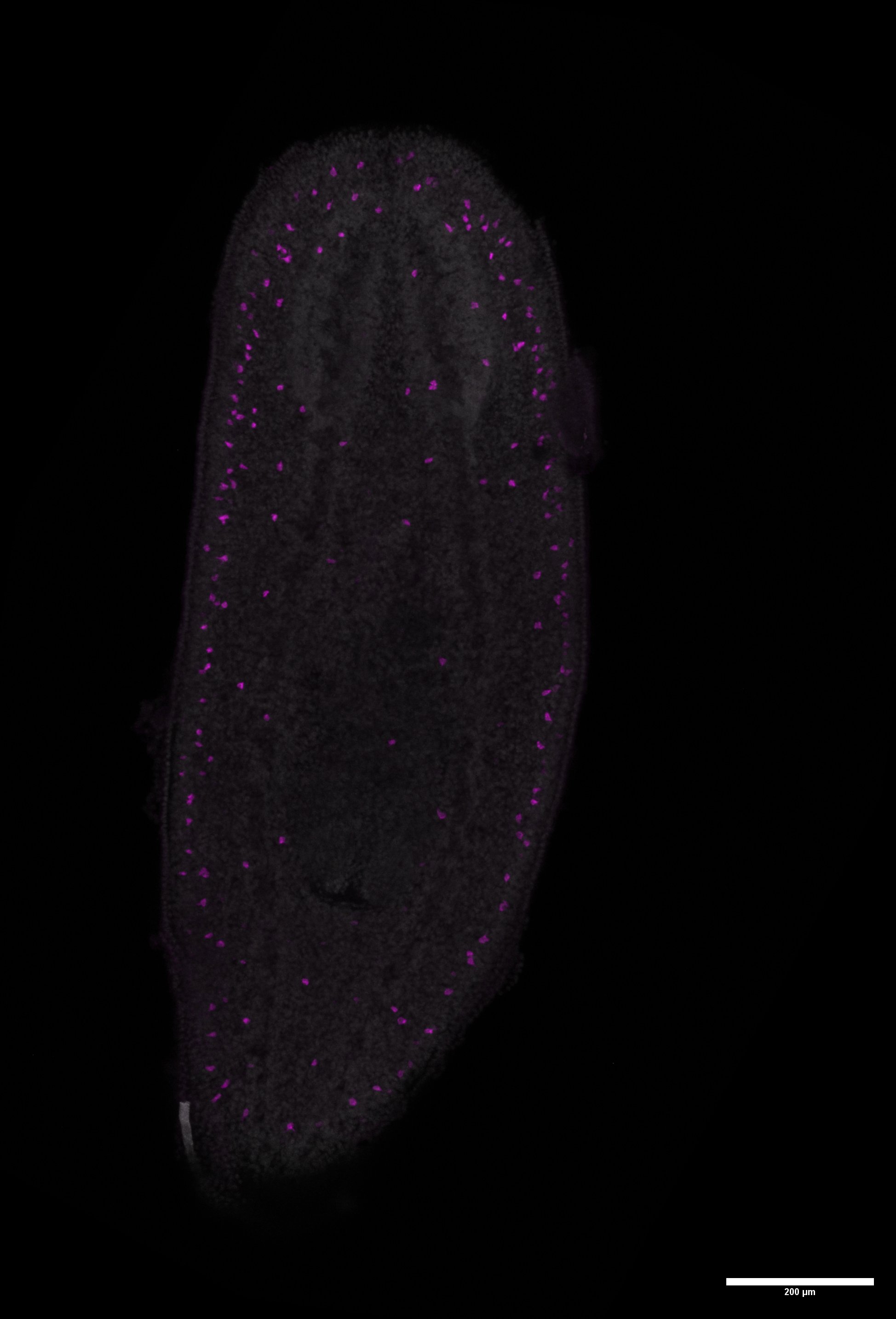

Supplement: Supplementary file 12 — Source data Fig. 5 [file 44318_2025_662_MOESM12_ESM.zip › Figure 5/5D/dd_924/ID_8_ythdf-B_RNAi_Probe_dd924_rhod_DAPI_10x.jpg]

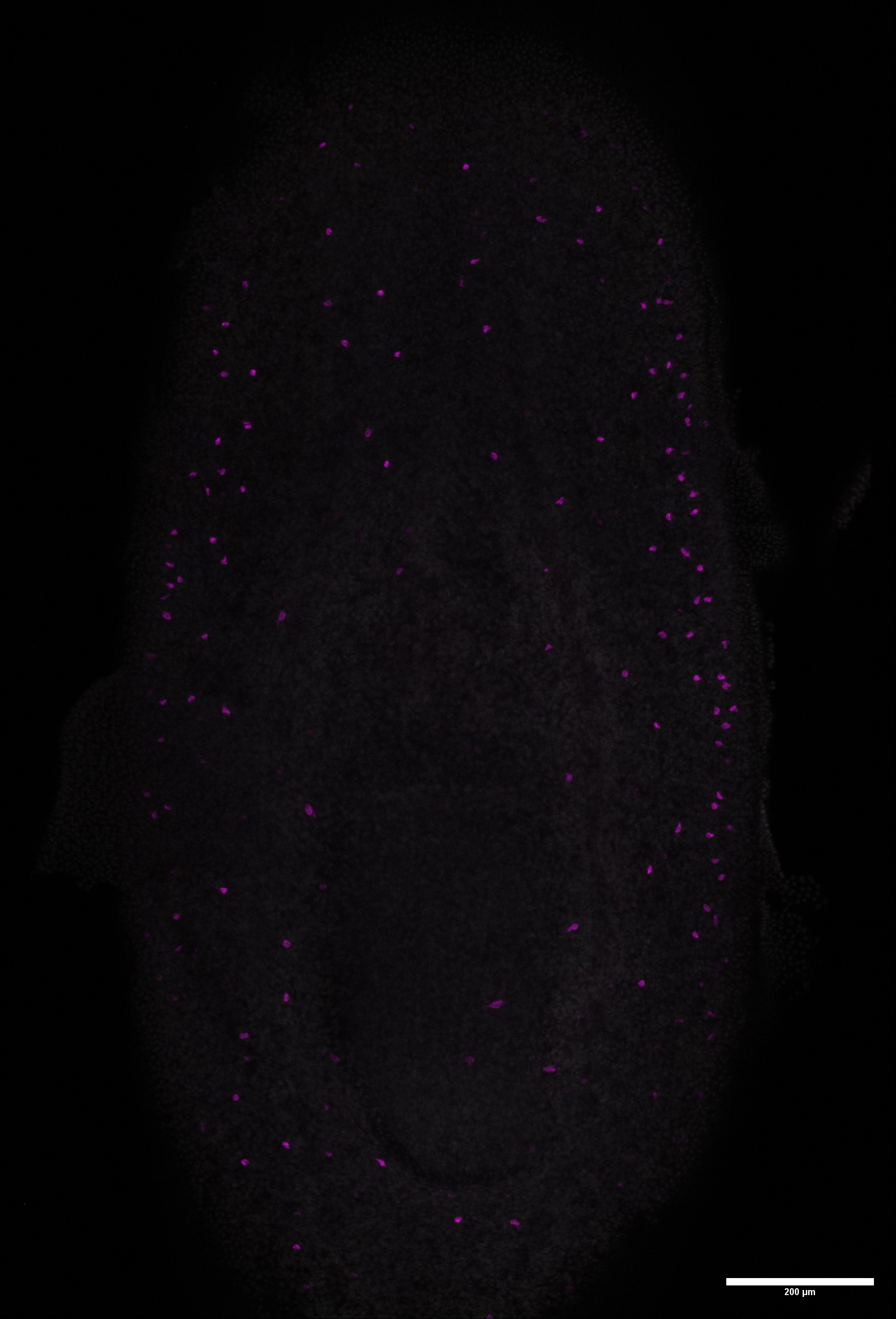

Supplement: Supplementary file 12 — Source data Fig. 5 [file 44318_2025_662_MOESM12_ESM.zip › Figure 5/5D/dd_924/ID_9_Control_RNAi_Probe_dd924_rhod_DAPI_10x.jpg]

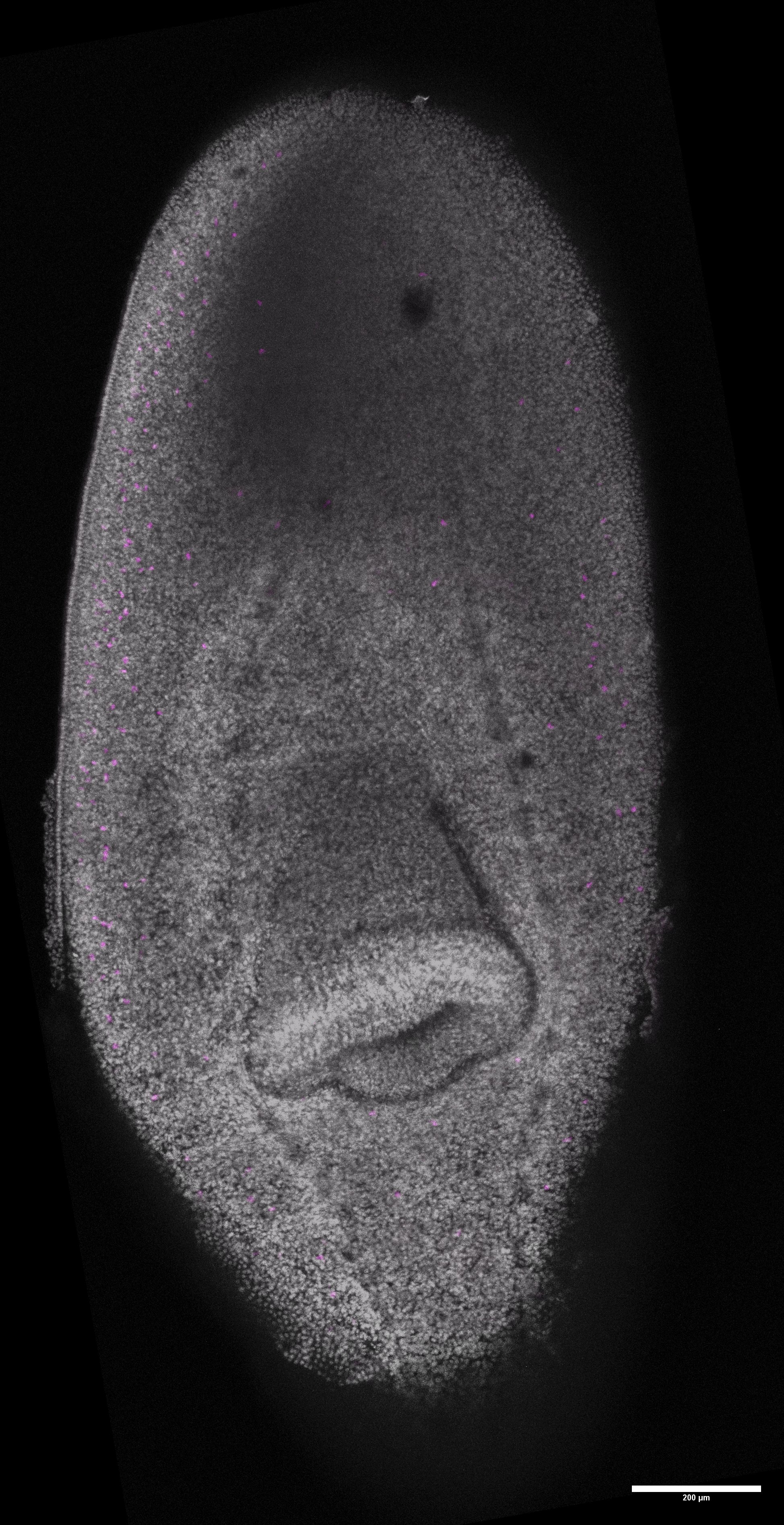

Supplement: Supplementary file 12 — Source data Fig. 5 [file 44318_2025_662_MOESM12_ESM.zip › Figure 5/5D/dd_924/ID_9_Triple_RNAi_Probe_dd924_rhod_DAPI_10x.jpg]

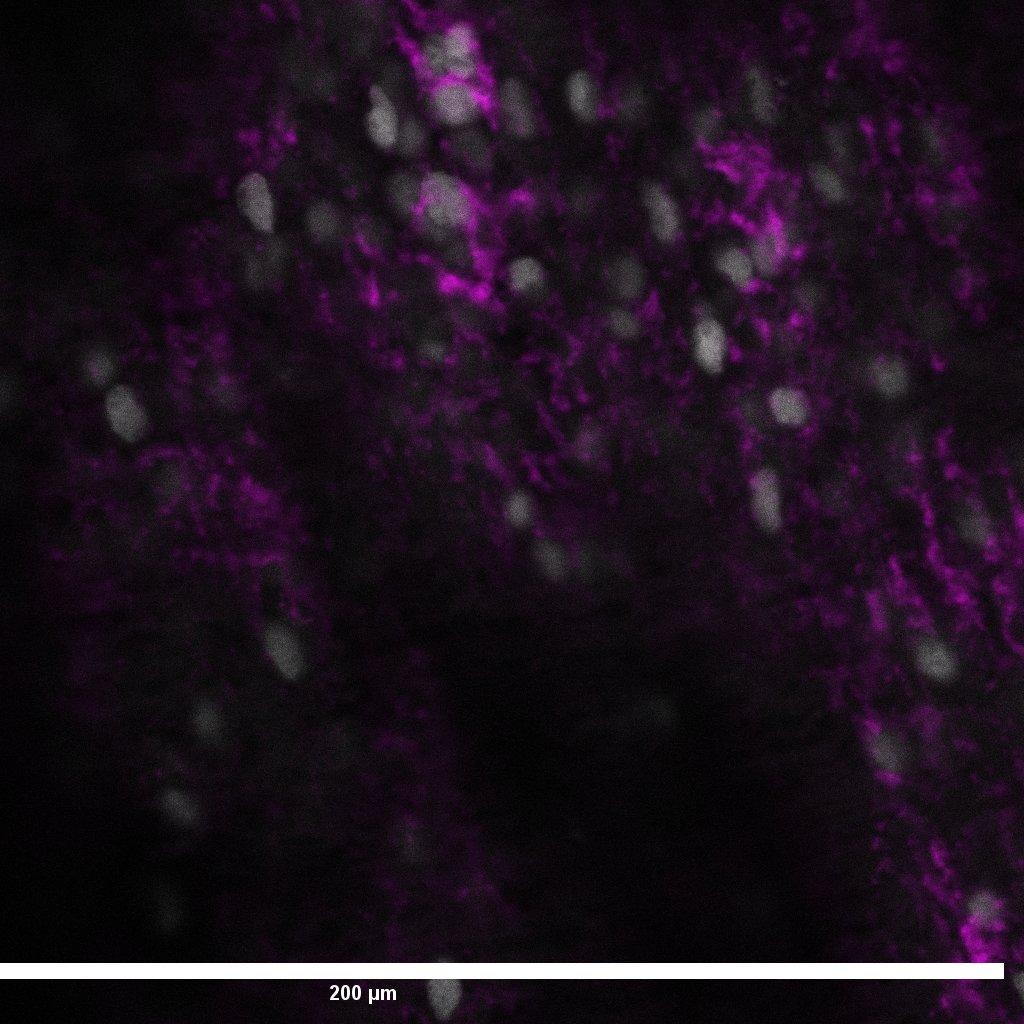

Supplement: Supplementary file 12 — Source data Fig. 5 [file 44318_2025_662_MOESM12_ESM.zip › Figure 5/5E/ID_1_Control_RNAi_Probe_dd_626_rhod_DAPI_10x.jpg]

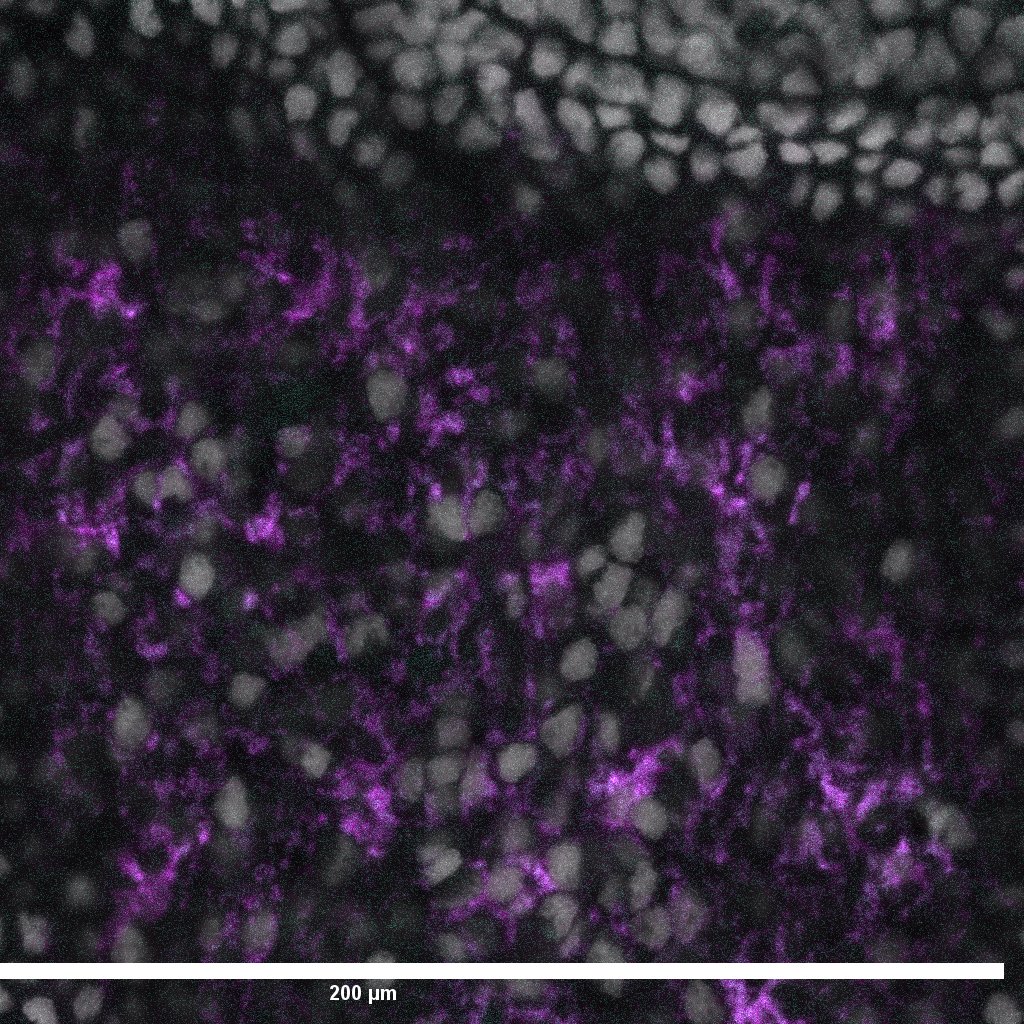

Supplement: Supplementary file 12 — Source data Fig. 5 [file 44318_2025_662_MOESM12_ESM.zip › Figure 5/5E/ID_1_Triple_RNAi_Probe_dd_626_rhod_DAPI_20x.jpg]

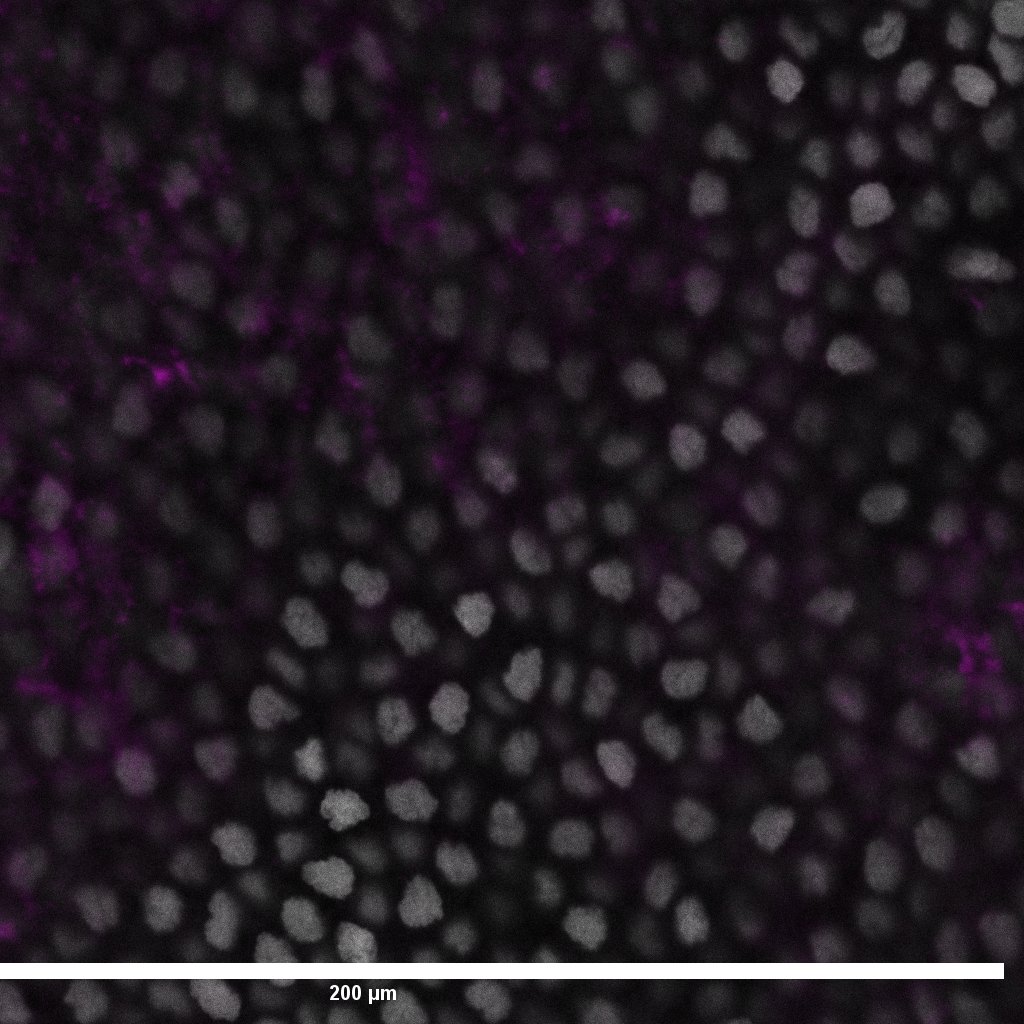

Supplement: Supplementary file 12 — Source data Fig. 5 [file 44318_2025_662_MOESM12_ESM.zip › Figure 5/5E/ID_2_Control_RNAi_Probe_dd_626_rhod_DAPI_10x.jpg]

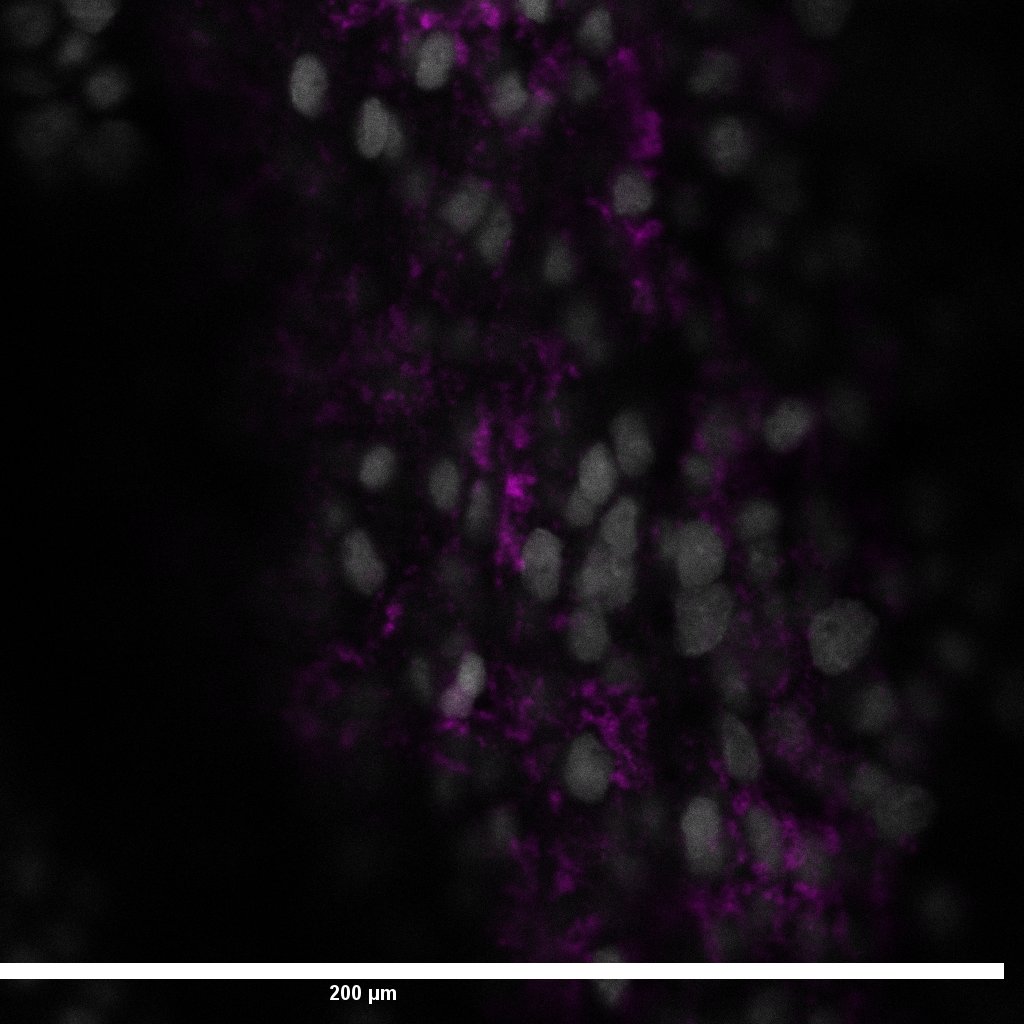

Supplement: Supplementary file 12 — Source data Fig. 5 [file 44318_2025_662_MOESM12_ESM.zip › Figure 5/5E/ID_2_Triple_RNAi_Probe_dd_626_rhod_DAPI_20x.jpg]

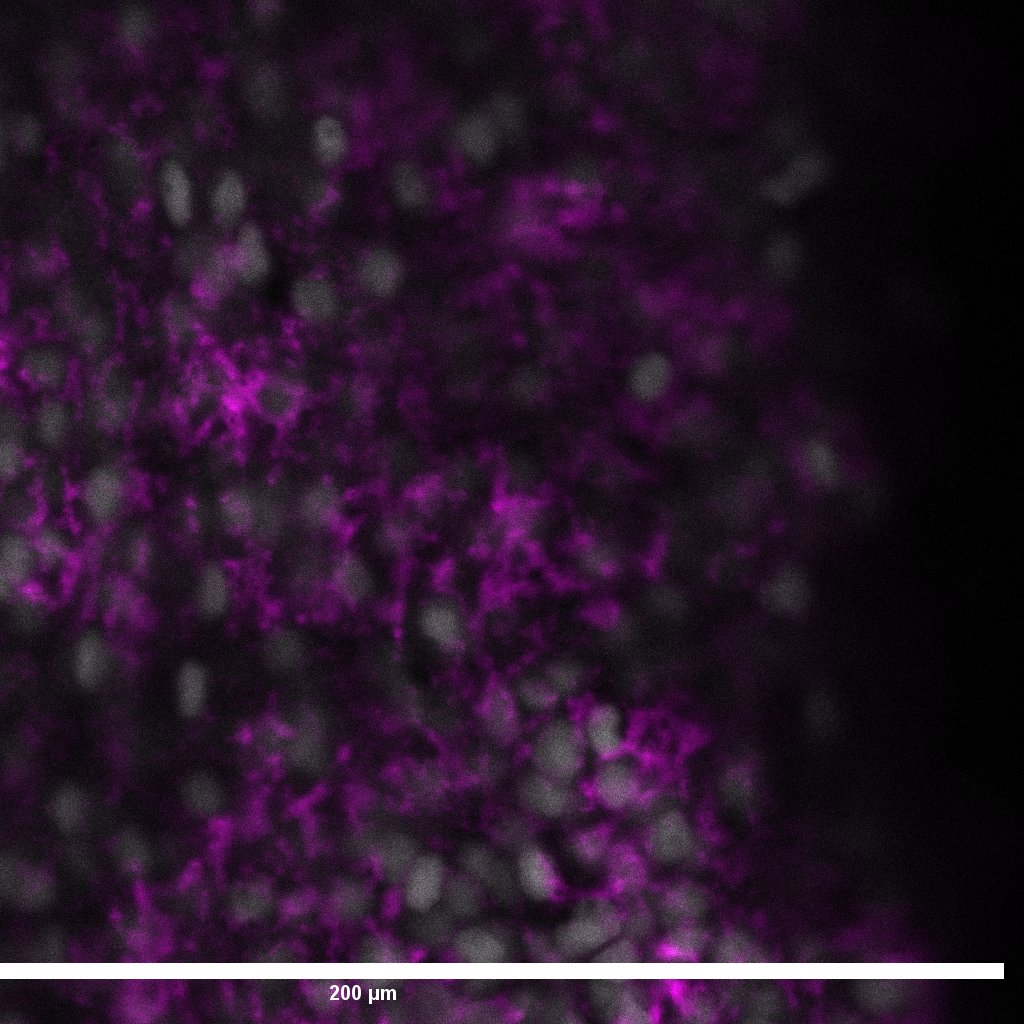

Supplement: Supplementary file 12 — Source data Fig. 5 [file 44318_2025_662_MOESM12_ESM.zip › Figure 5/5E/ID_3_Control_RNAi_Probe_dd_626_rhod_DAPI_10x.jpg]

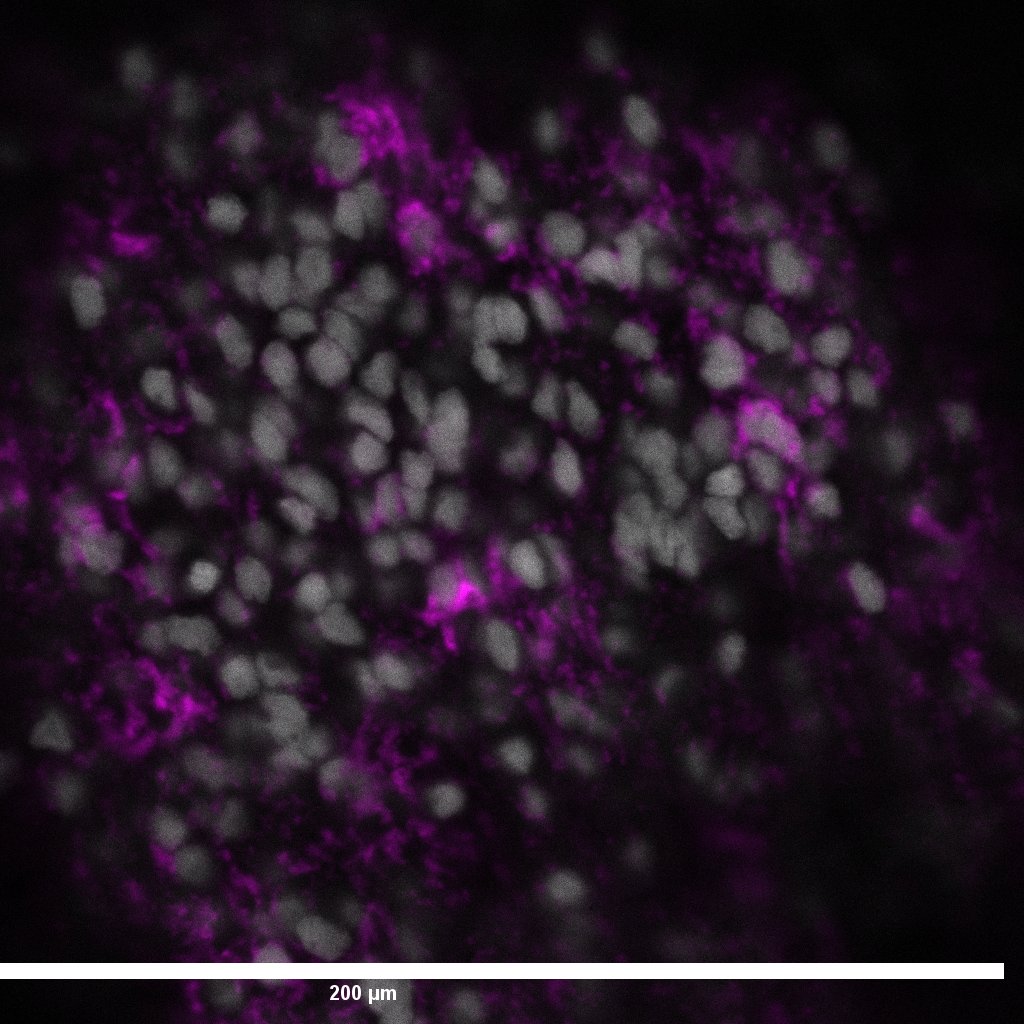

Supplement: Supplementary file 12 — Source data Fig. 5 [file 44318_2025_662_MOESM12_ESM.zip › Figure 5/5E/ID_3_Triple_RNAi_Probe_dd_626_rhod_DAPI_20x.jpg]

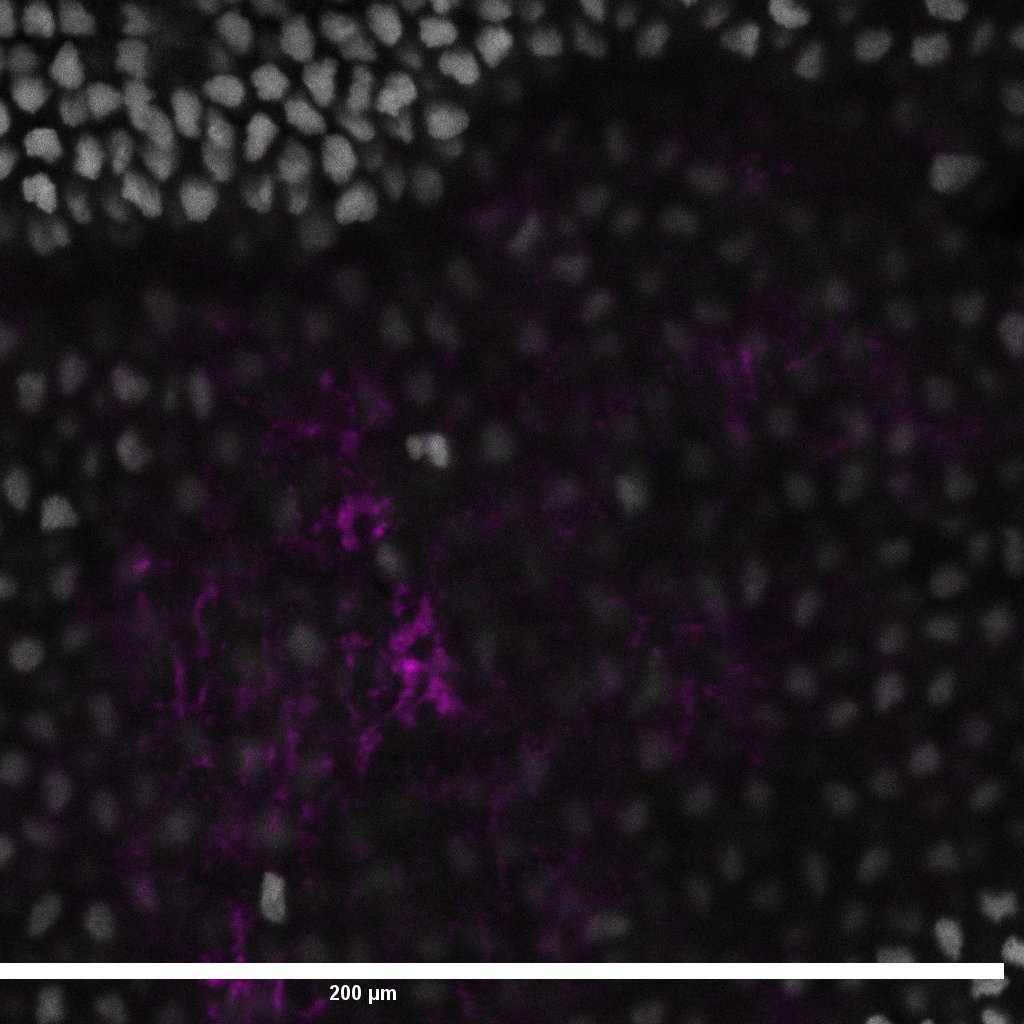

Supplement: Supplementary file 12 — Source data Fig. 5 [file 44318_2025_662_MOESM12_ESM.zip › Figure 5/5E/ID_4_Control_RNAi_Probe_dd_626_rhod_DAPI_10x.jpg]

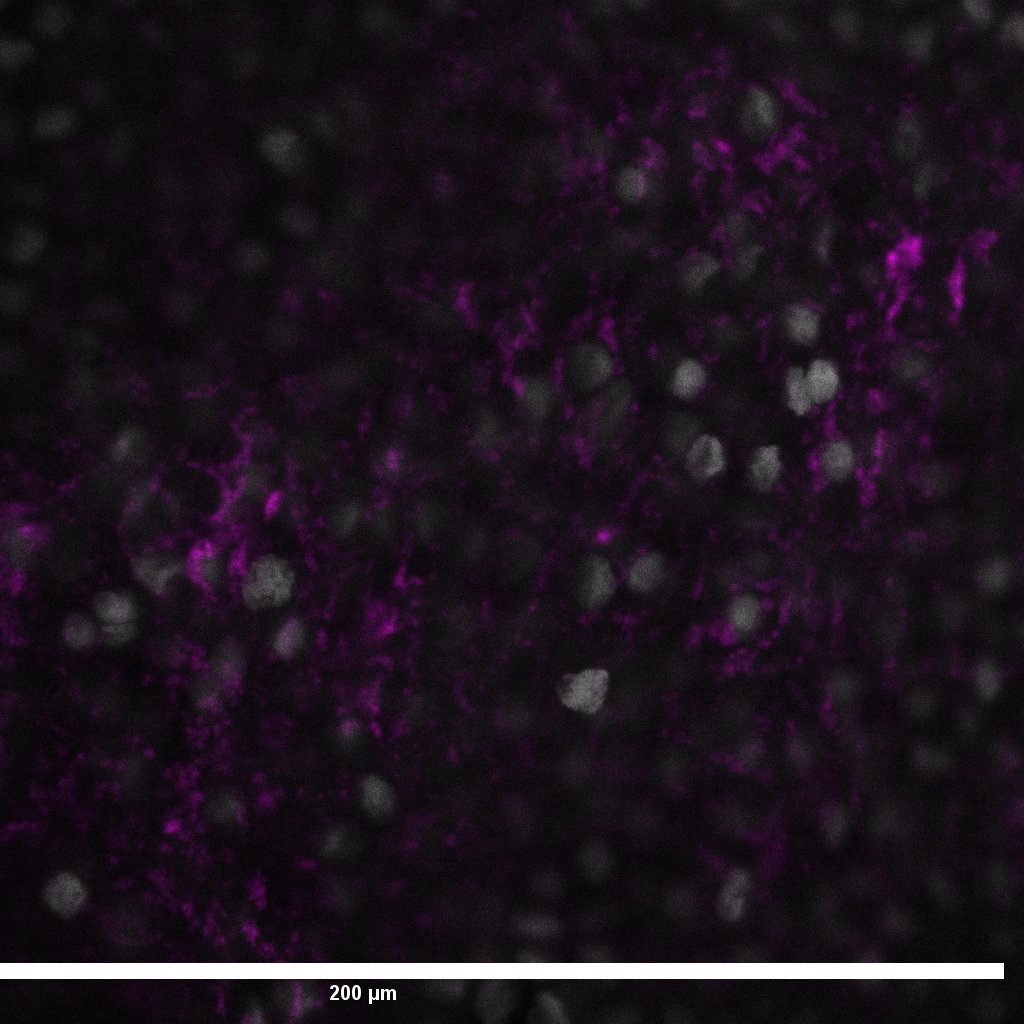

Supplement: Supplementary file 12 — Source data Fig. 5 [file 44318_2025_662_MOESM12_ESM.zip › Figure 5/5E/ID_4_Triple_RNAi_Probe_dd_626_rhod_DAPI_20x.jpg]

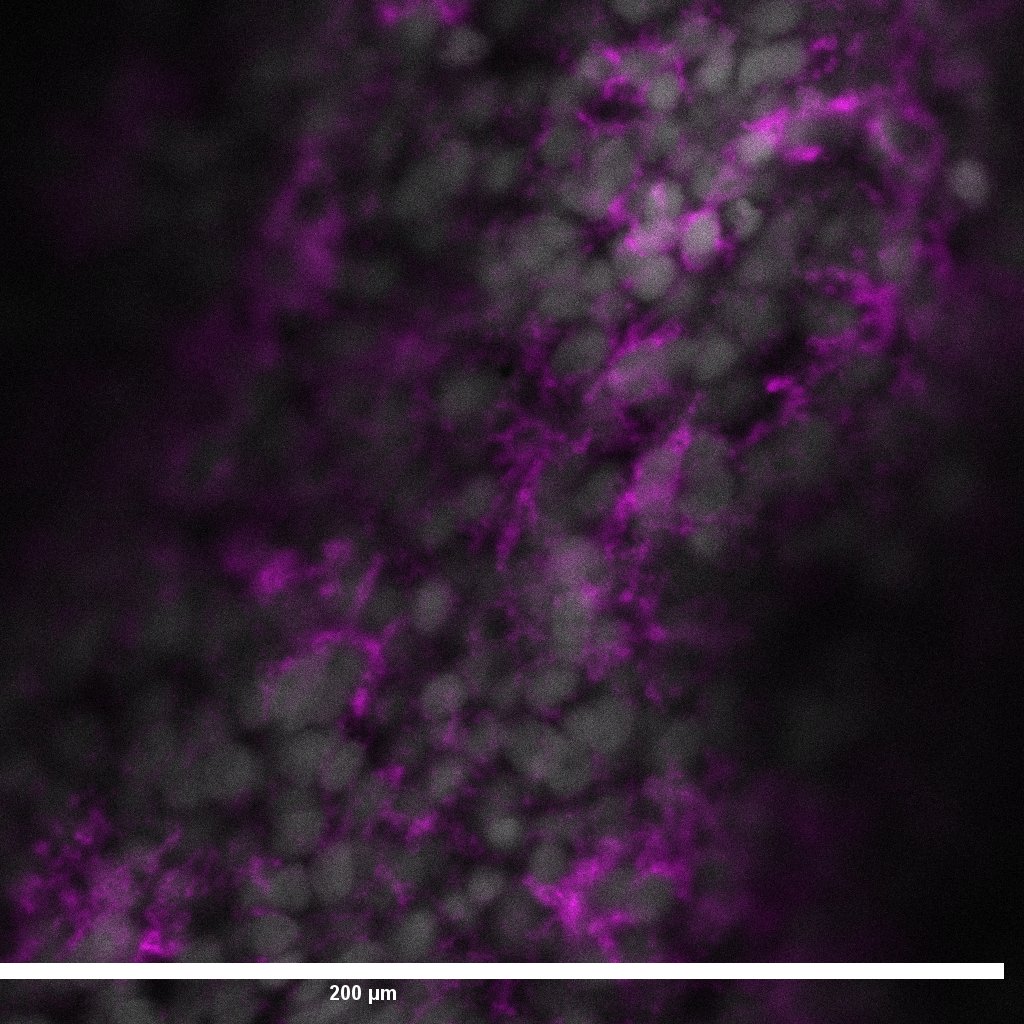

Supplement: Supplementary file 12 — Source data Fig. 5 [file 44318_2025_662_MOESM12_ESM.zip › Figure 5/5E/ID_5_Control_RNAi_Probe_dd_626_rhod_DAPI_10x.jpg]

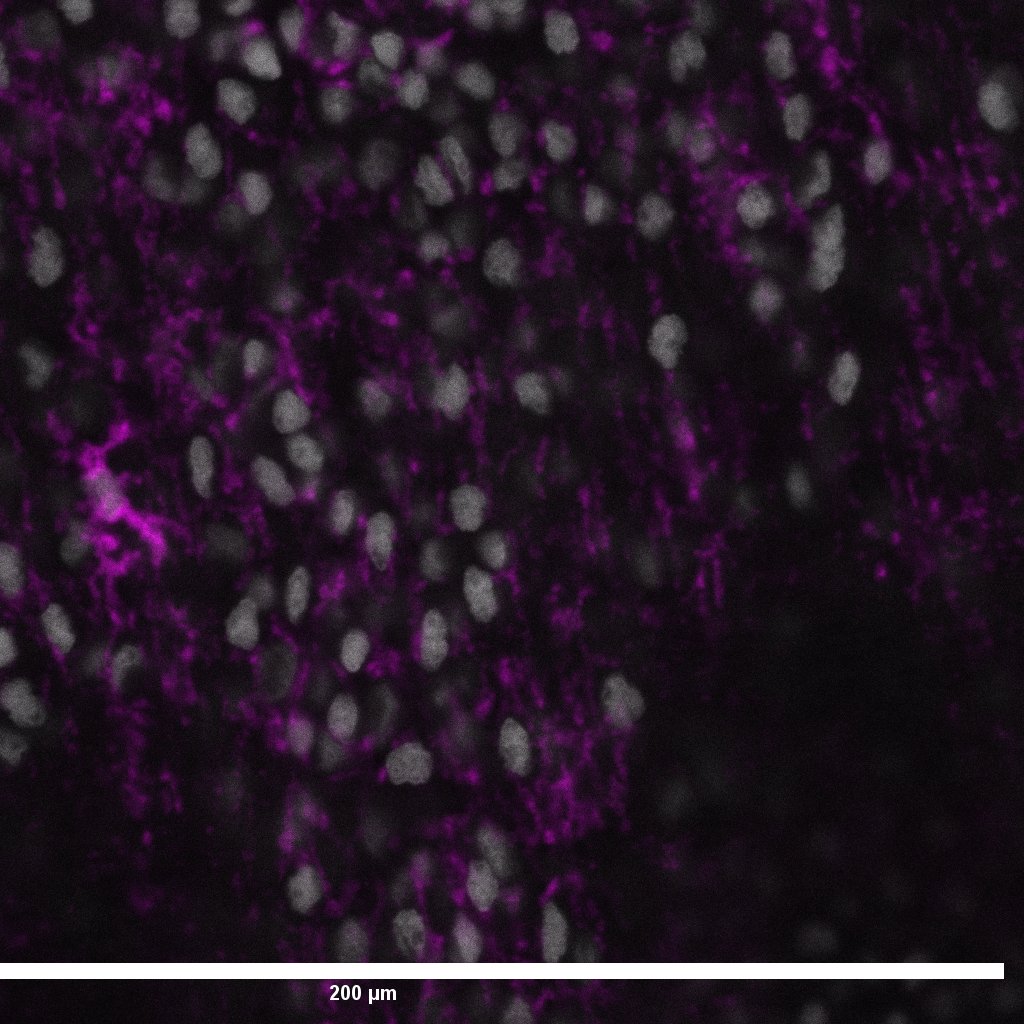

Supplement: Supplementary file 12 — Source data Fig. 5 [file 44318_2025_662_MOESM12_ESM.zip › Figure 5/5E/ID_5_Triple_RNAi_Probe_dd_626_rhod_DAPI_20x.jpg]

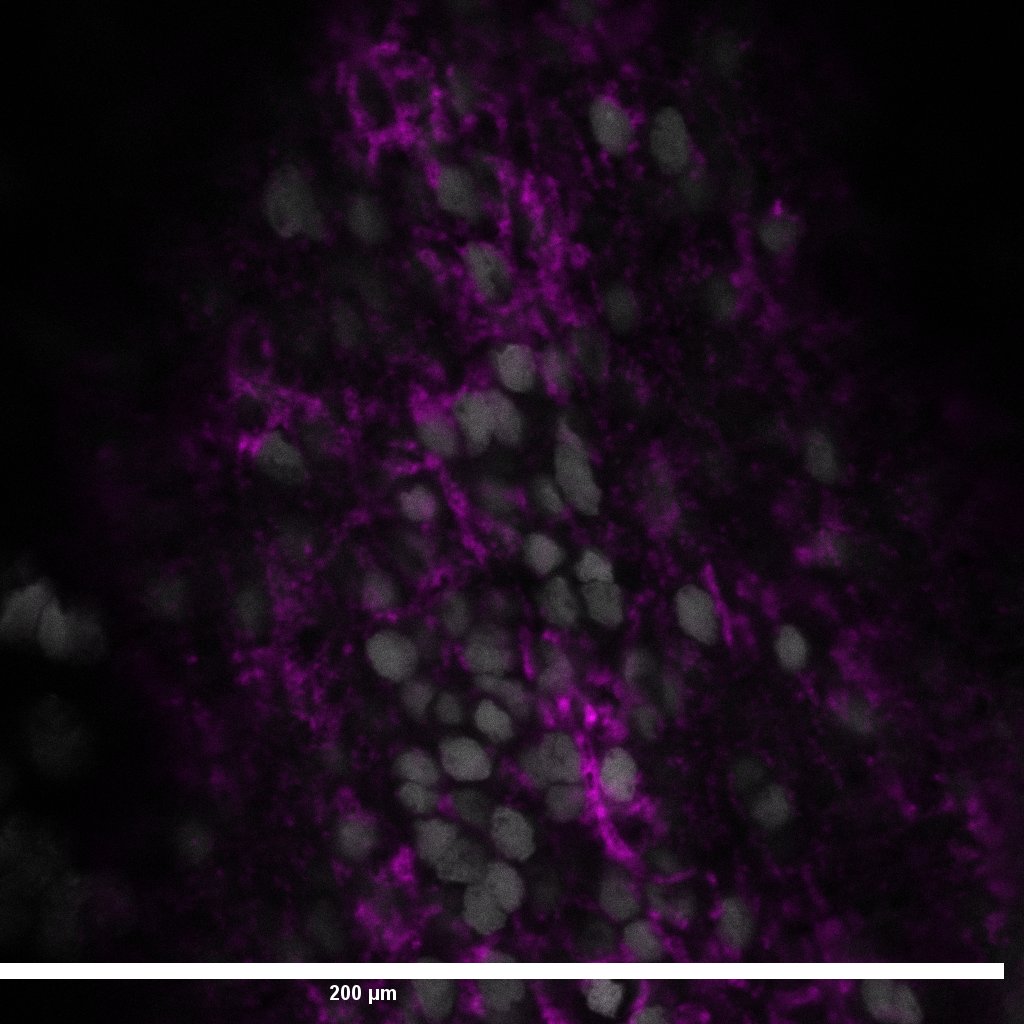

Supplement: Supplementary file 12 — Source data Fig. 5 [file 44318_2025_662_MOESM12_ESM.zip › Figure 5/5E/ID_6_Control_RNAi_Probe_dd_626_rhod_DAPI_10x.jpg]

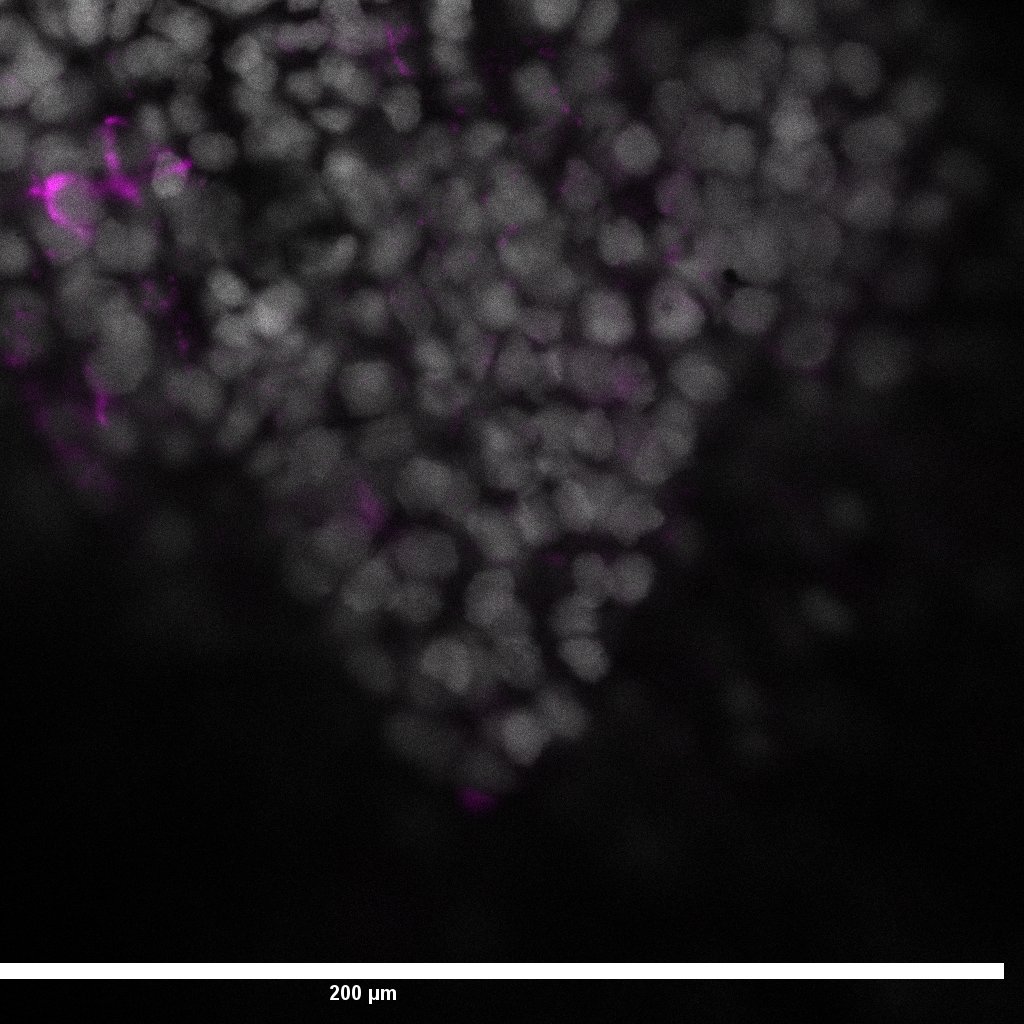

Supplement: Supplementary file 12 — Source data Fig. 5 [file 44318_2025_662_MOESM12_ESM.zip › Figure 5/5E/ID_6_Triple_RNAi_Probe_dd_626_rhod_DAPI_20x.jpg]

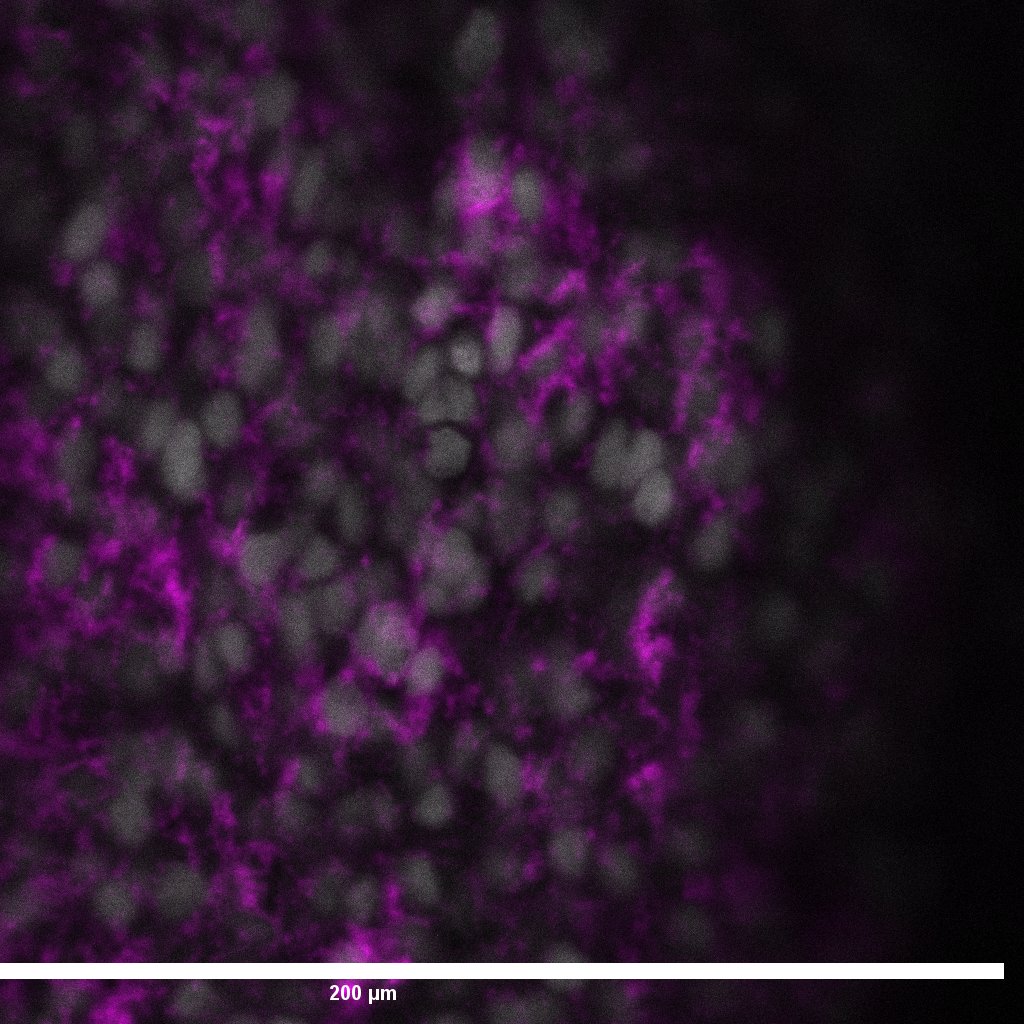

Supplement: Supplementary file 12 — Source data Fig. 5 [file 44318_2025_662_MOESM12_ESM.zip › Figure 5/5E/ID_7_Control_RNAi_Probe_dd_626_rhod_DAPI_10x.jpg]

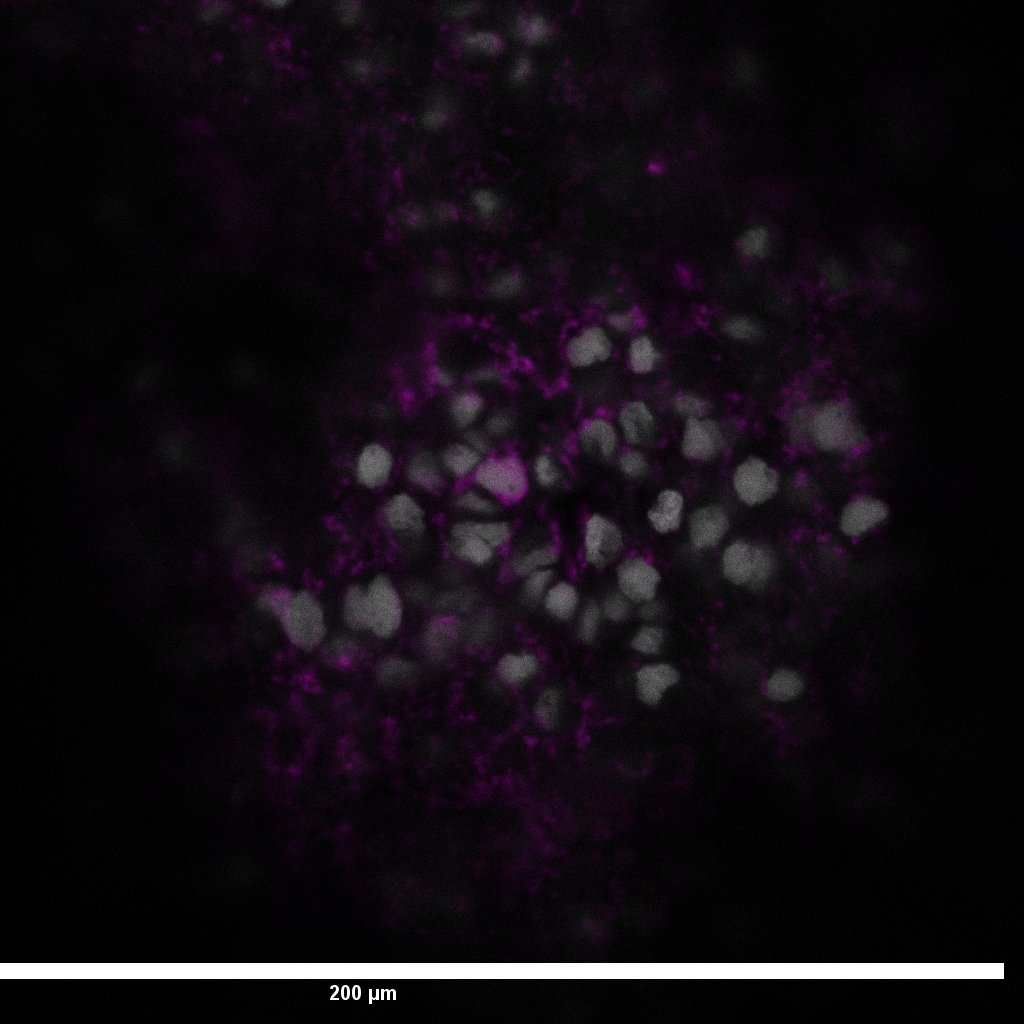

Supplement: Supplementary file 12 — Source data Fig. 5 [file 44318_2025_662_MOESM12_ESM.zip › Figure 5/5E/ID_7_Triple_RNAi_Probe_dd_626_rhod_DAPI_20x.jpg]

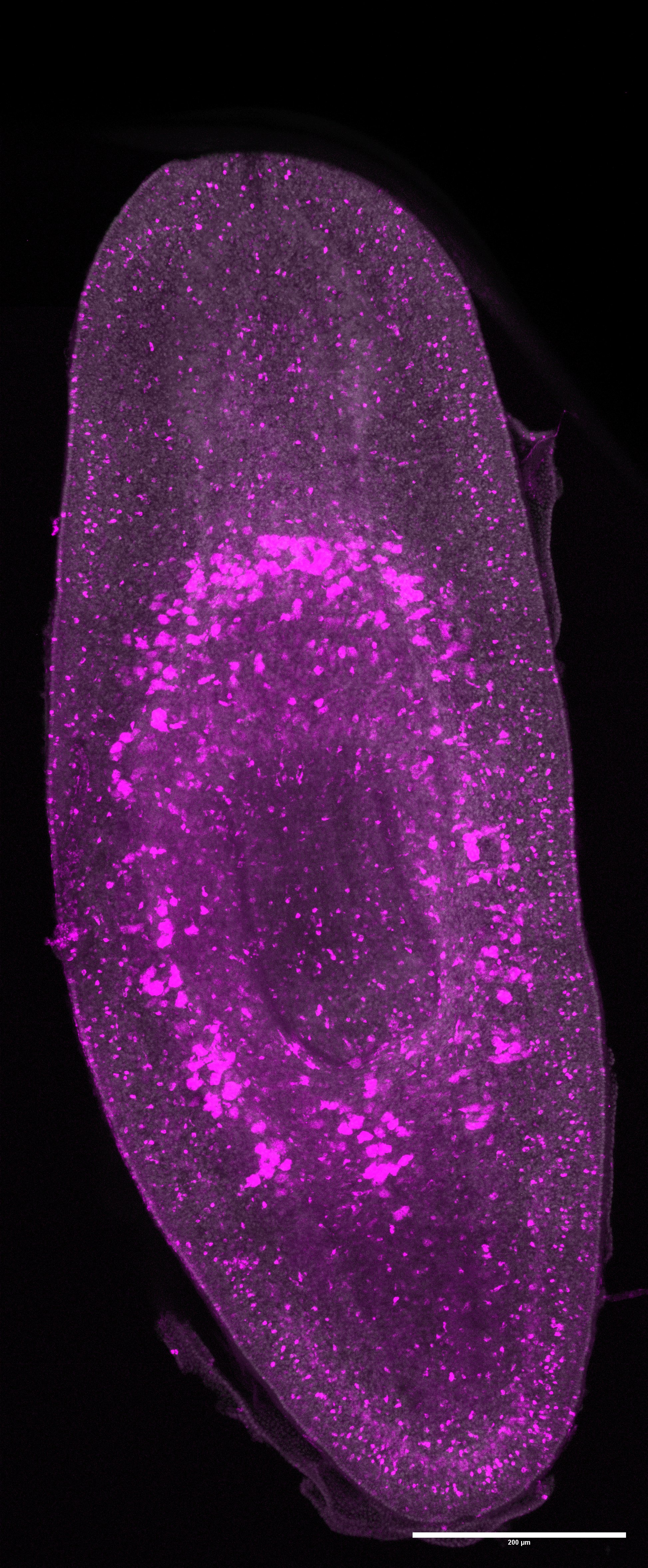

Supplement: Supplementary file 13 — Source data Fig. 6 [file 44318_2025_662_MOESM13_ESM.zip › Figure 6/6B/ID_1_Triple_RNAi_Probe_dd1837_rhod_DAPI_10x.jpg]

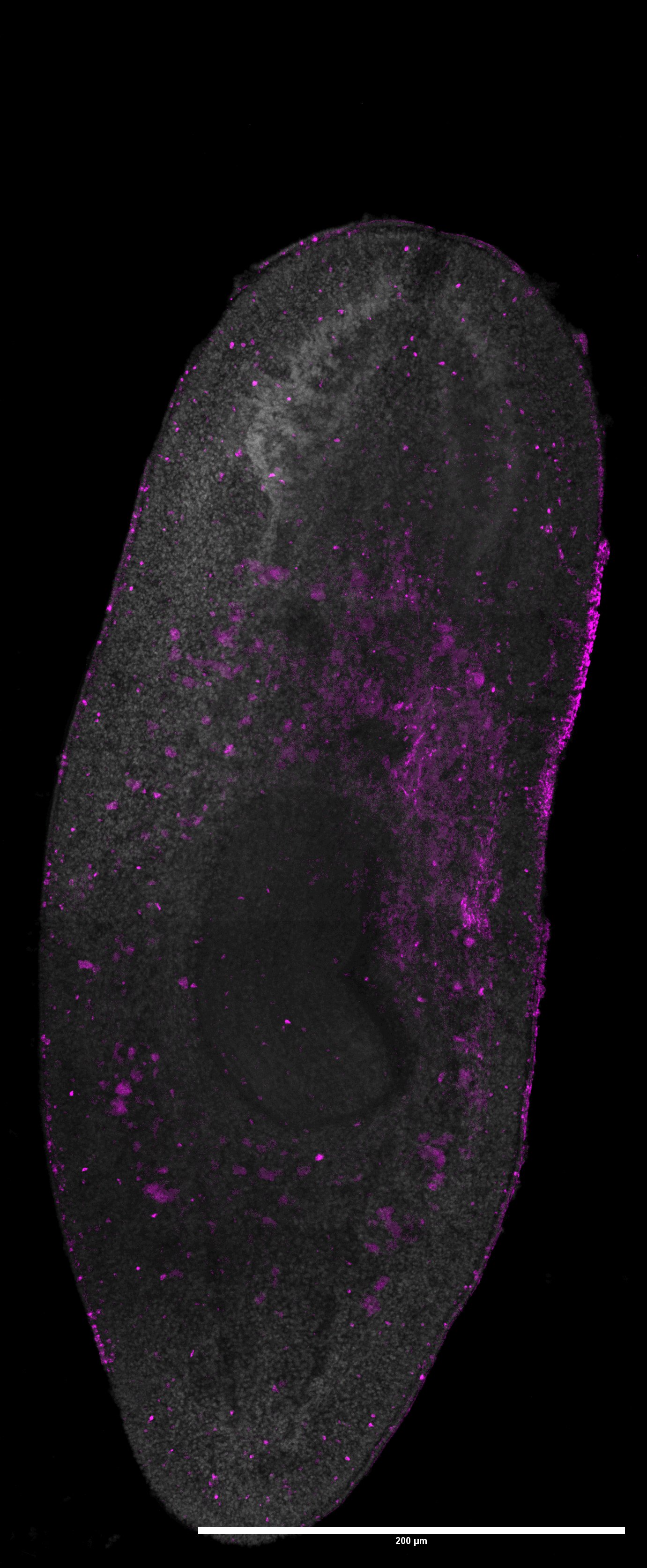

Supplement: Supplementary file 13 — Source data Fig. 6 [file 44318_2025_662_MOESM13_ESM.zip › Figure 6/6B/ID_2_Control_RNAi_Probe_dd1837_rhod_DAPI_10x.jpg]

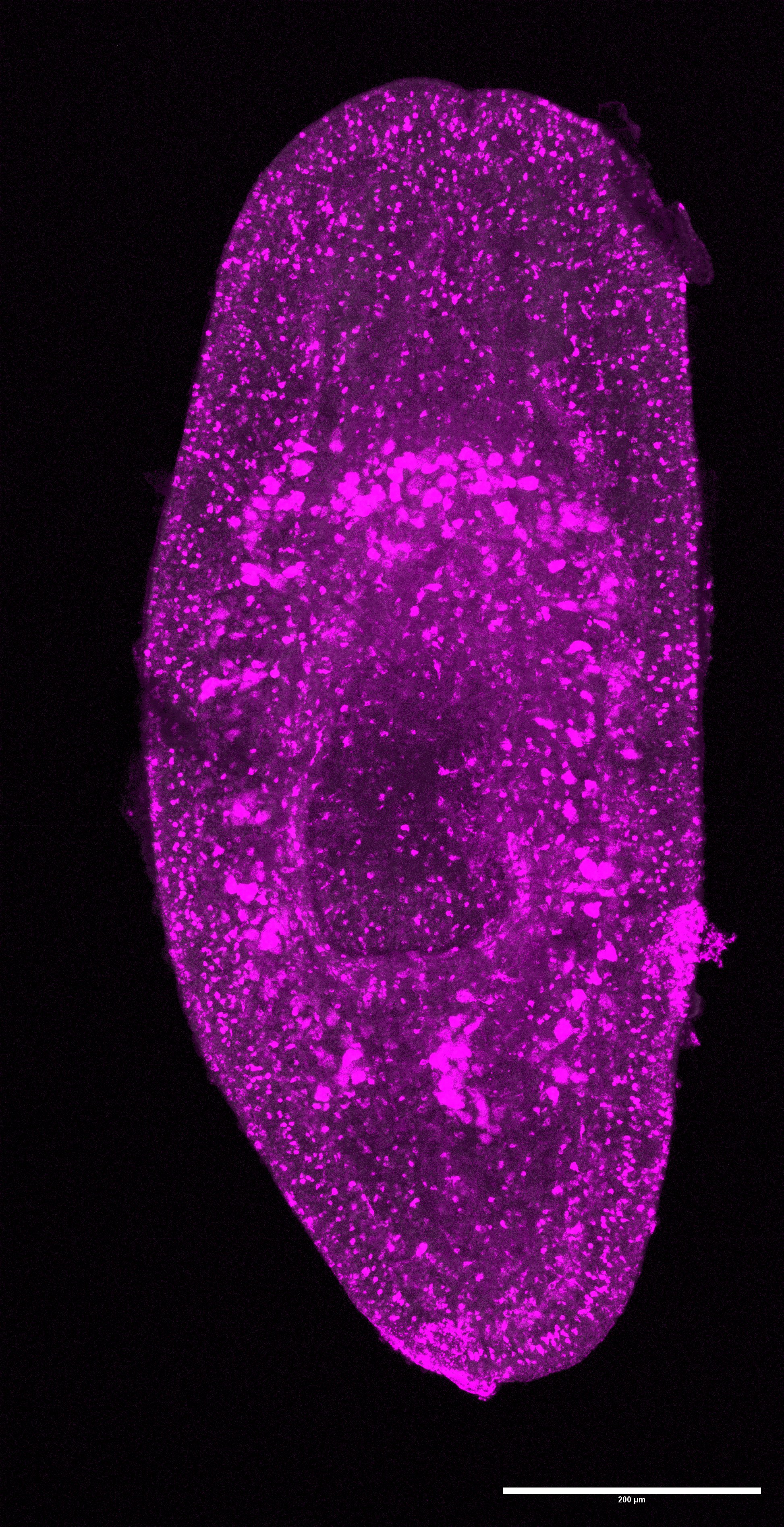

Supplement: Supplementary file 13 — Source data Fig. 6 [file 44318_2025_662_MOESM13_ESM.zip › Figure 6/6B/ID_2_Triple_RNAi_Probe_dd1837_rhod_DAPI_10x.jpg]

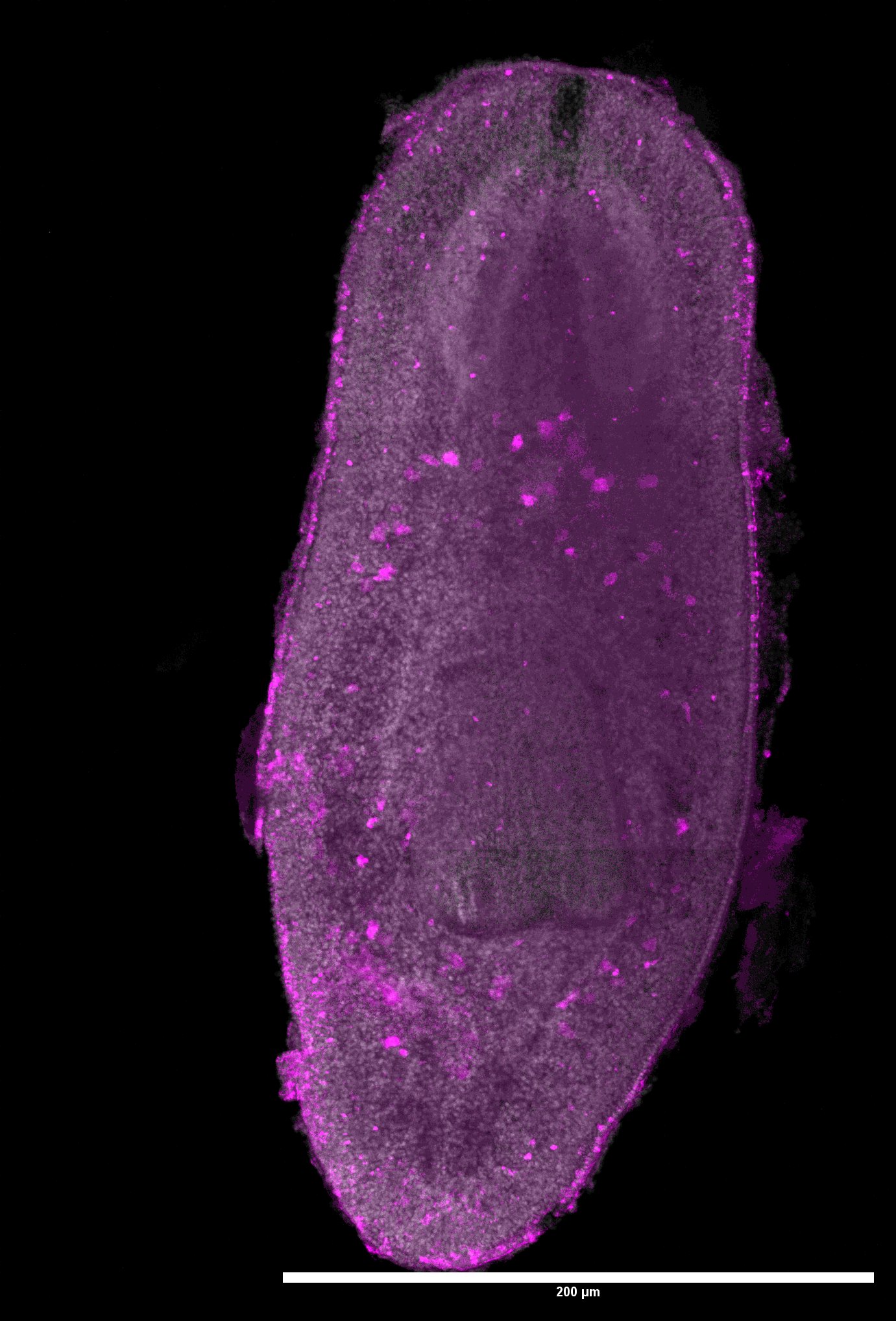

Supplement: Supplementary file 13 — Source data Fig. 6 [file 44318_2025_662_MOESM13_ESM.zip › Figure 6/6B/ID_3_Control_RNAi_Probe_dd1837_rhod_DAPI_10x.jpg]

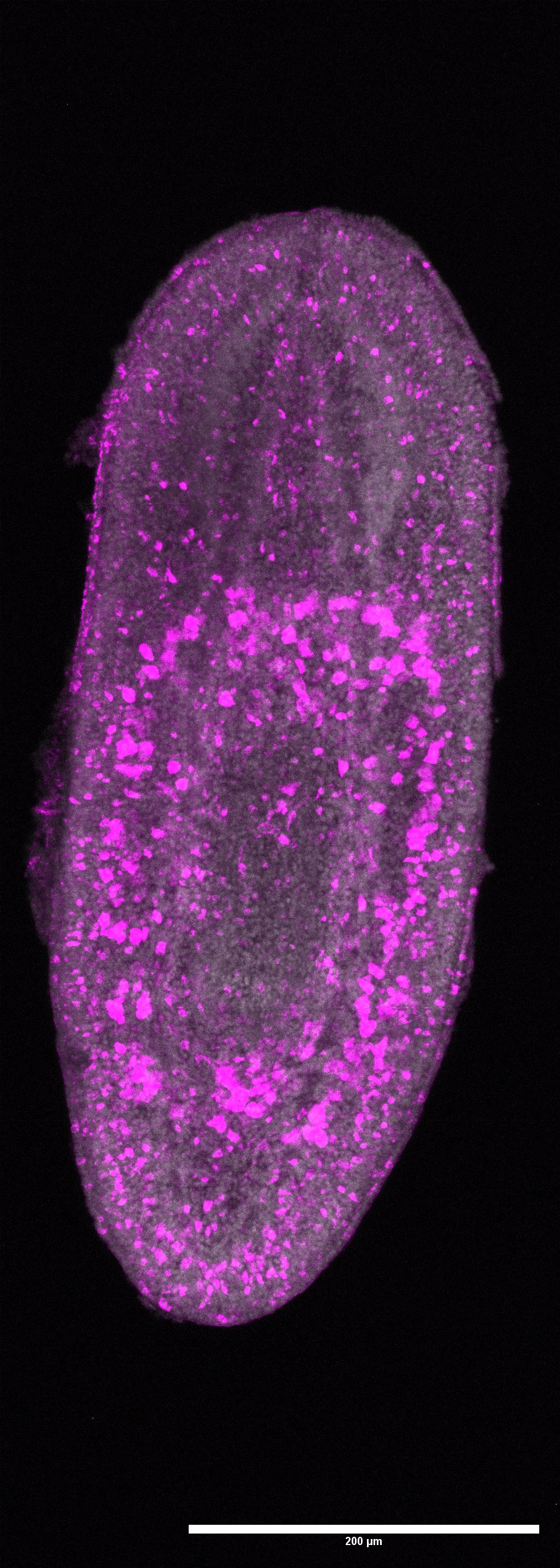

Supplement: Supplementary file 13 — Source data Fig. 6 [file 44318_2025_662_MOESM13_ESM.zip › Figure 6/6B/ID_3_Triple_RNAi_Probe_dd1837_rhod_DAPI_10x.jpg]

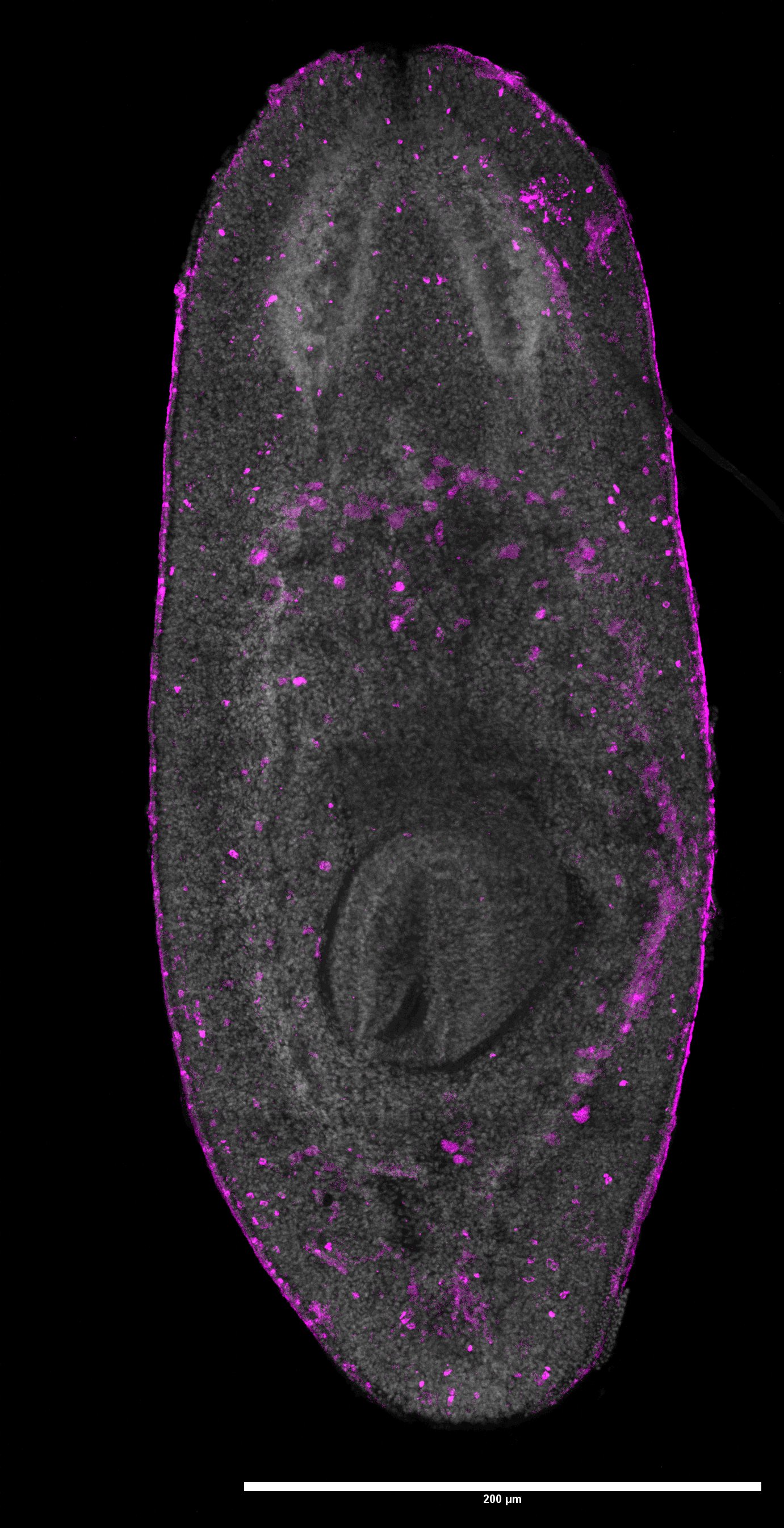

Supplement: Supplementary file 13 — Source data Fig. 6 [file 44318_2025_662_MOESM13_ESM.zip › Figure 6/6B/ID_4_Control_RNAi_Probe_dd1837_rhod_DAPI_10x.jpg]

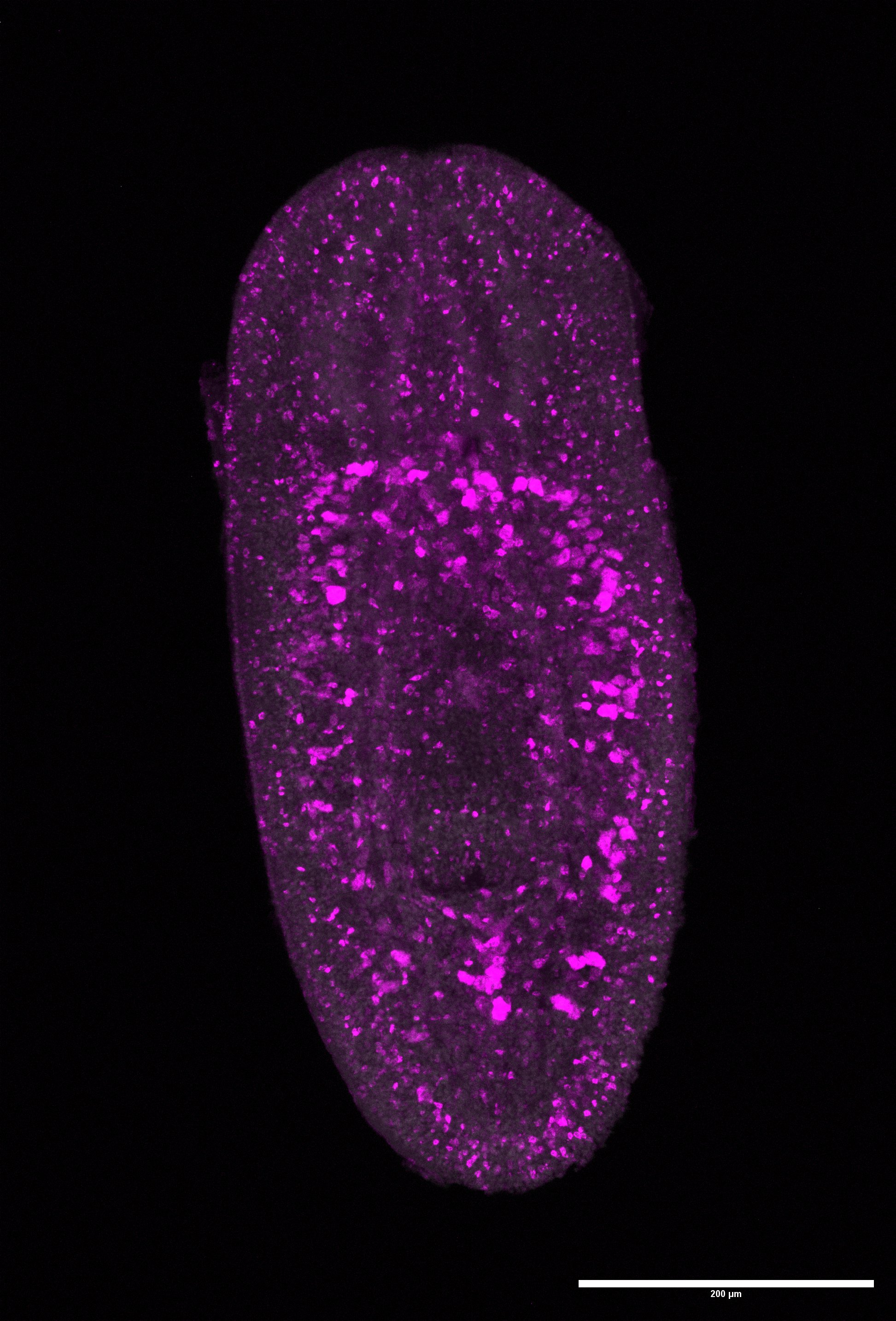

Supplement: Supplementary file 13 — Source data Fig. 6 [file 44318_2025_662_MOESM13_ESM.zip › Figure 6/6B/ID_4_Triple_RNAi_Probe_dd1837_rhod_DAPI_10x.jpg]

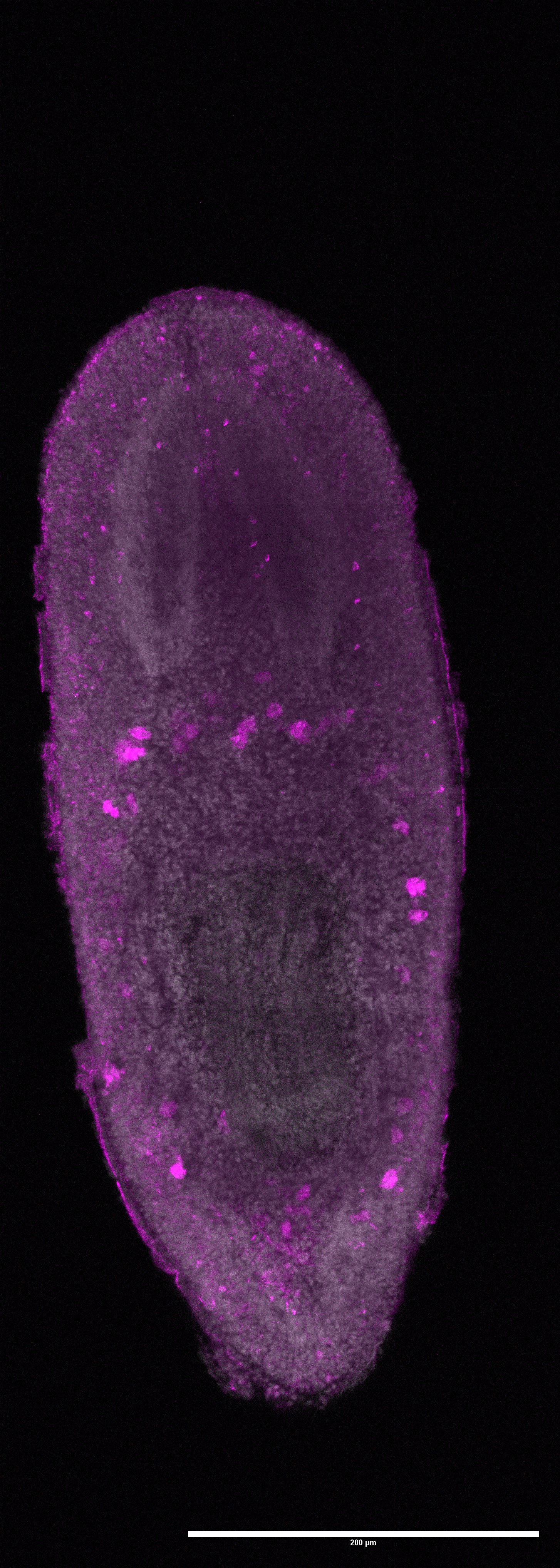

Supplement: Supplementary file 13 — Source data Fig. 6 [file 44318_2025_662_MOESM13_ESM.zip › Figure 6/6B/ID_5_Control_RNAi_Probe_dd1837_rhod_DAPI_10x.jpg]

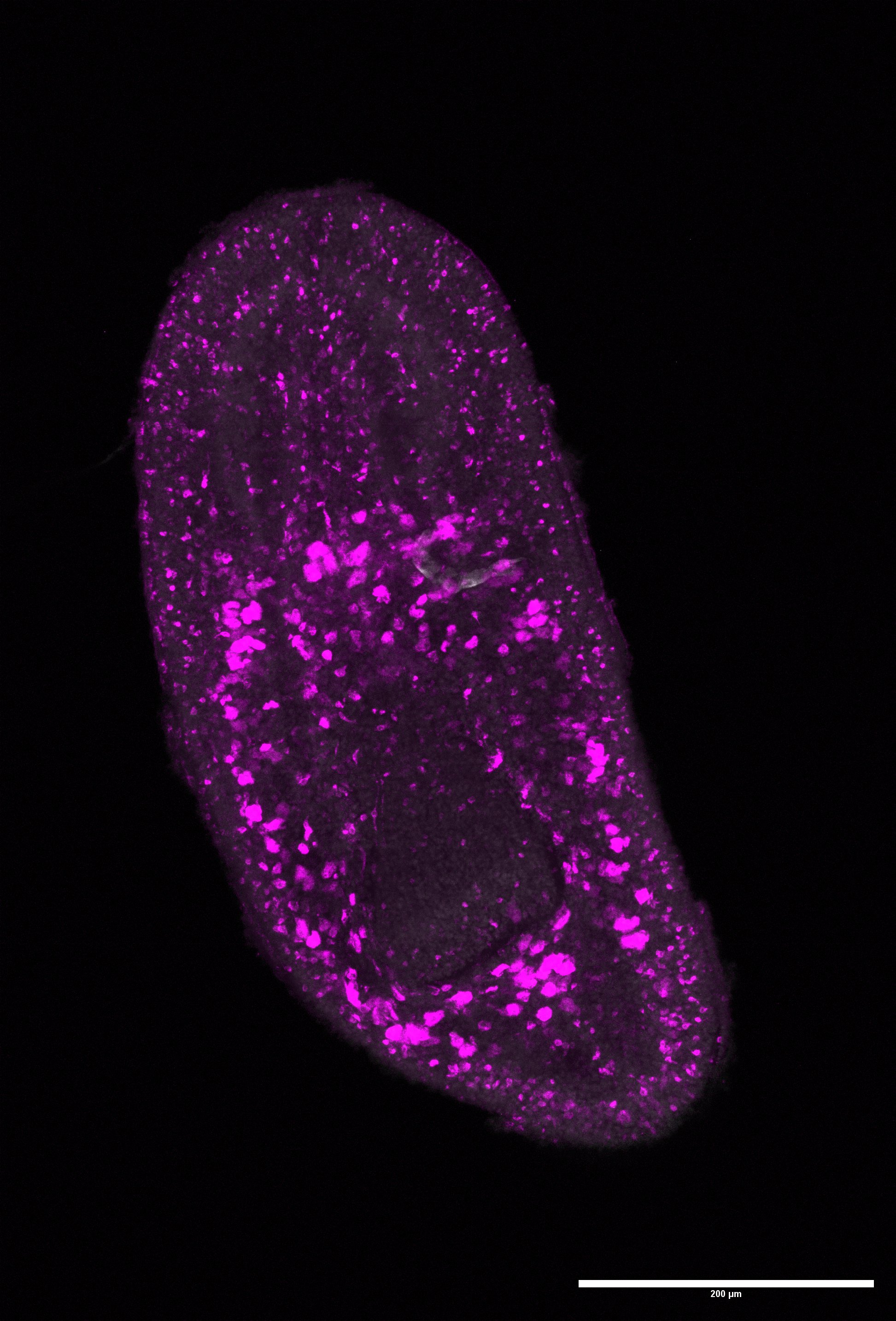

Supplement: Supplementary file 13 — Source data Fig. 6 [file 44318_2025_662_MOESM13_ESM.zip › Figure 6/6B/ID_5_Triple_RNAi_Probe_dd1837_rhod_DAPI_10x.jpg]

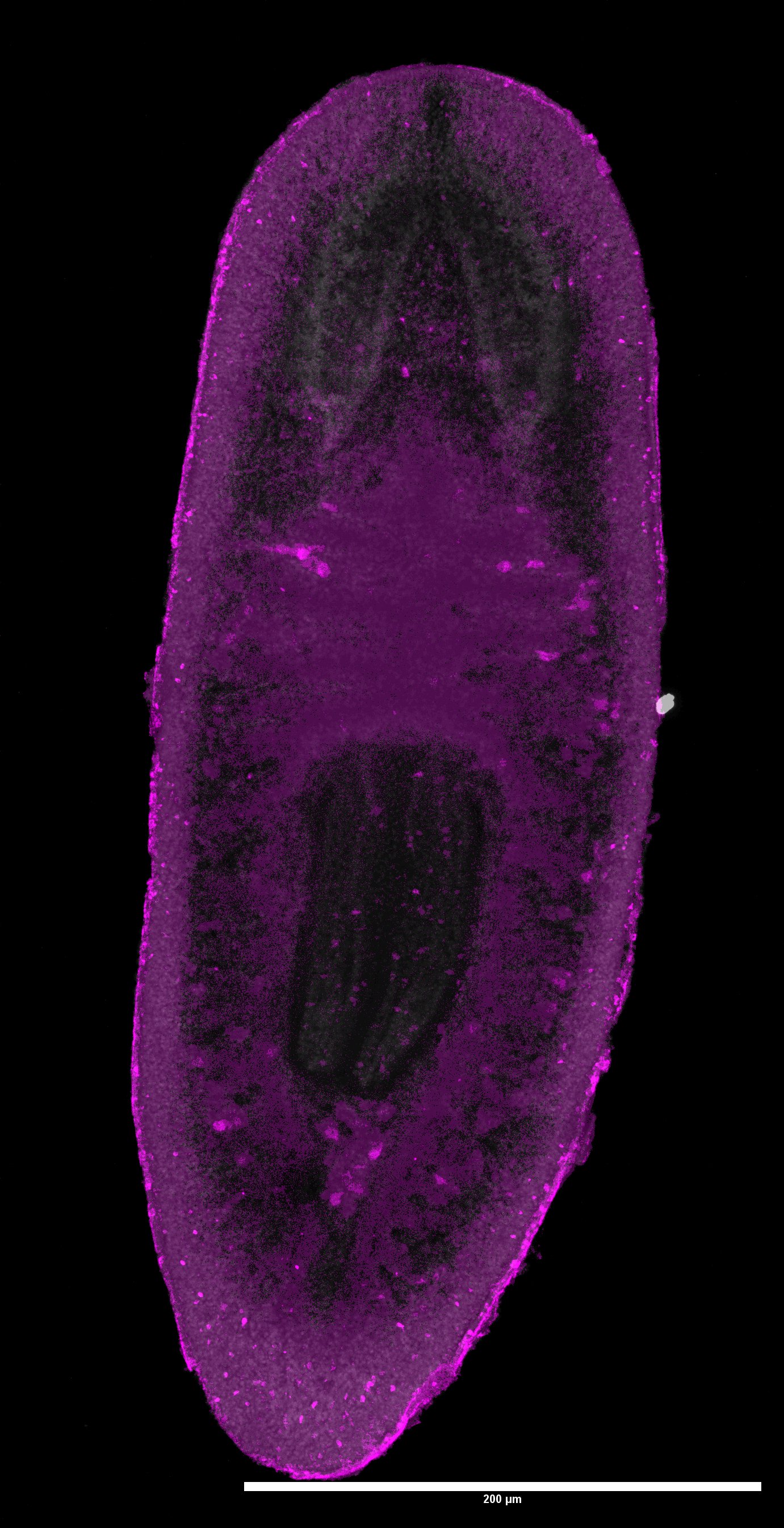

Supplement: Supplementary file 13 — Source data Fig. 6 [file 44318_2025_662_MOESM13_ESM.zip › Figure 6/6B/ID_6_Control_RNAi_Probe_dd1837_rhod_DAPI_10x.jpg]

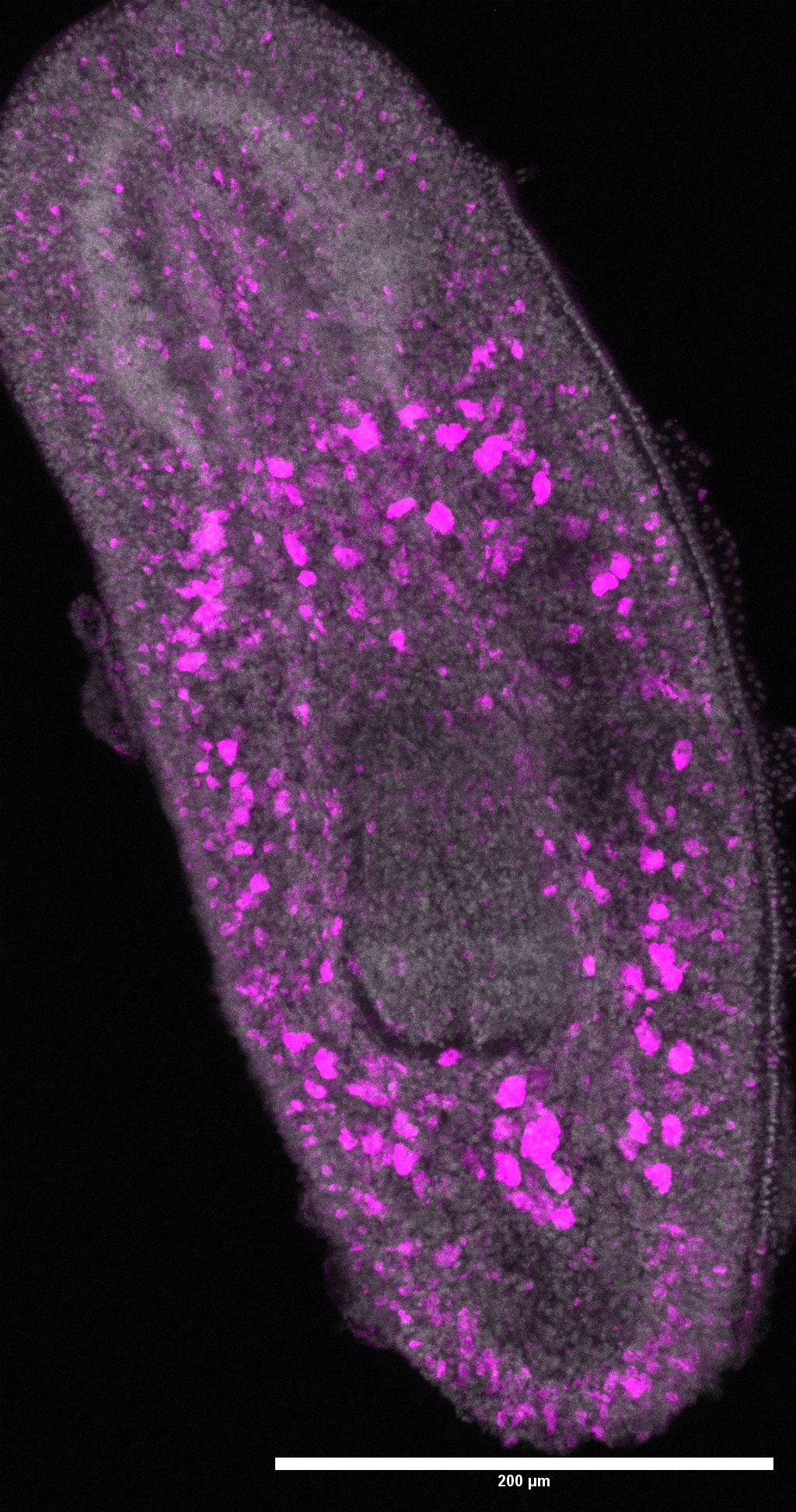

Supplement: Supplementary file 13 — Source data Fig. 6 [file 44318_2025_662_MOESM13_ESM.zip › Figure 6/6B/ID_6_Triple_RNAi_Probe_dd1837_rhod_DAPI_10x.jpg]

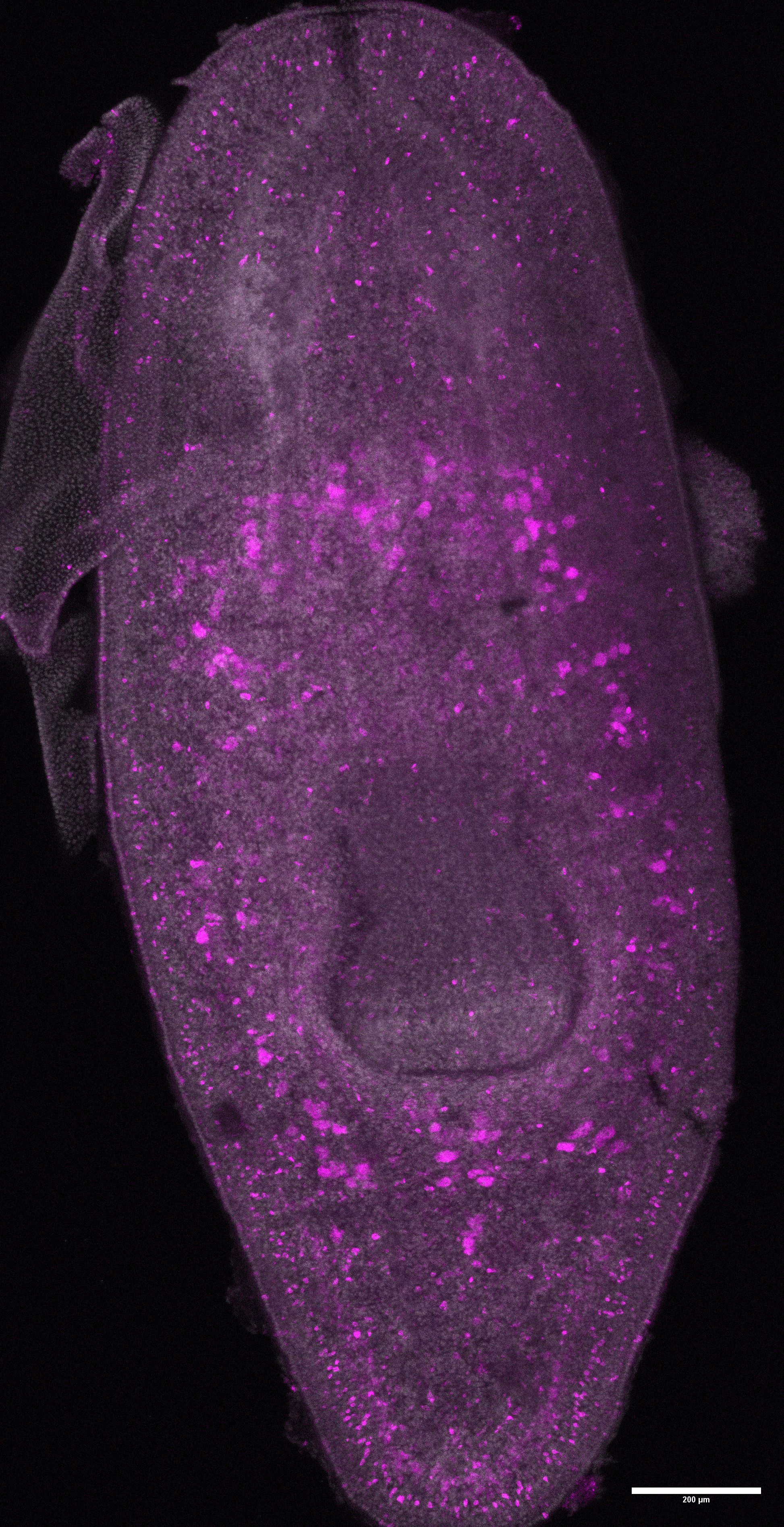

Supplement: Supplementary file 13 — Source data Fig. 6 [file 44318_2025_662_MOESM13_ESM.zip › Figure 6/6B/ID_7 _Triple_RNAi_Probe_dd1837_rhod_DAPI_10x.jpg]

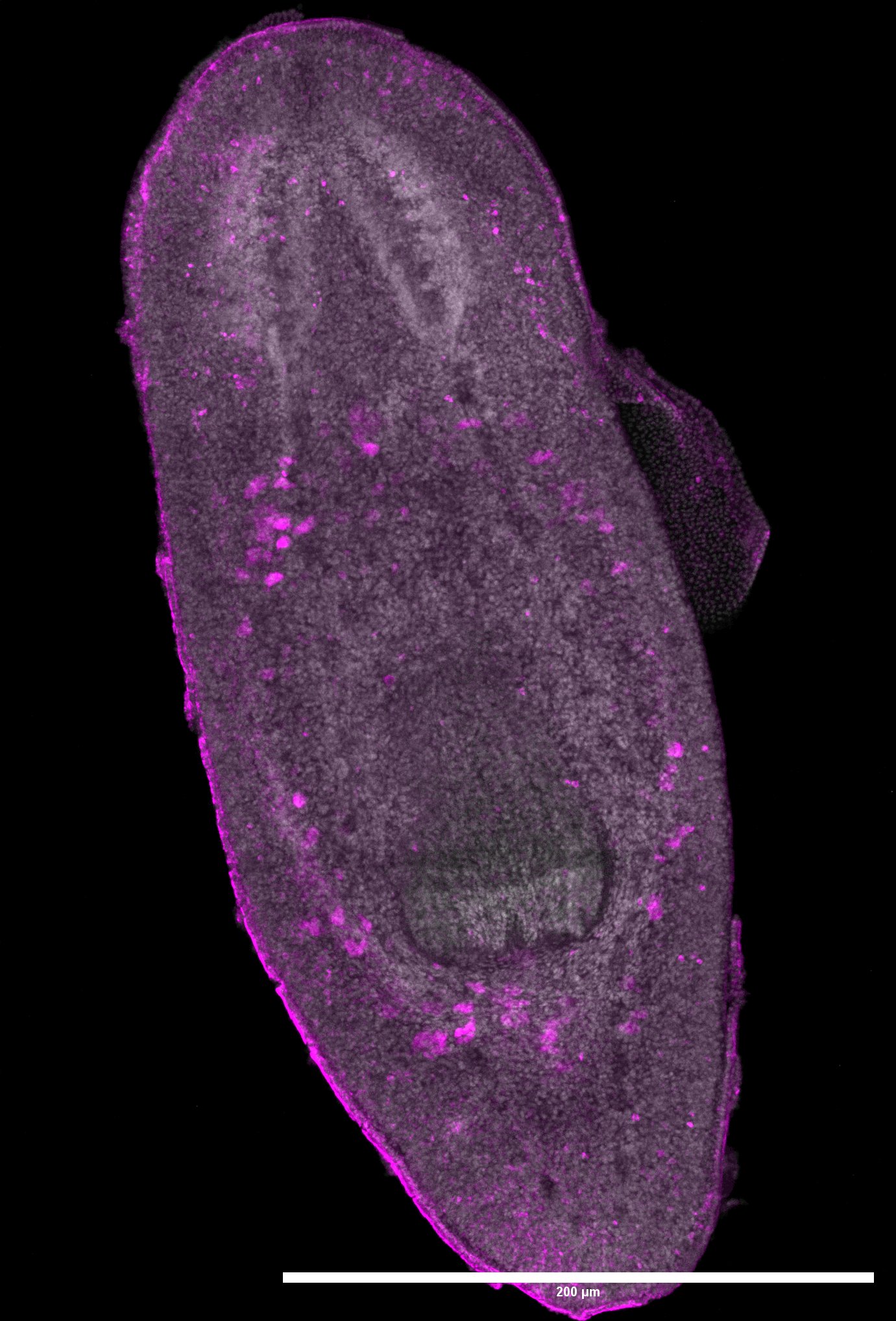

Supplement: Supplementary file 13 — Source data Fig. 6 [file 44318_2025_662_MOESM13_ESM.zip › Figure 6/6B/ID_7_Control_RNAi_Probe_dd1837_rhod_DAPI_10x.jpg]

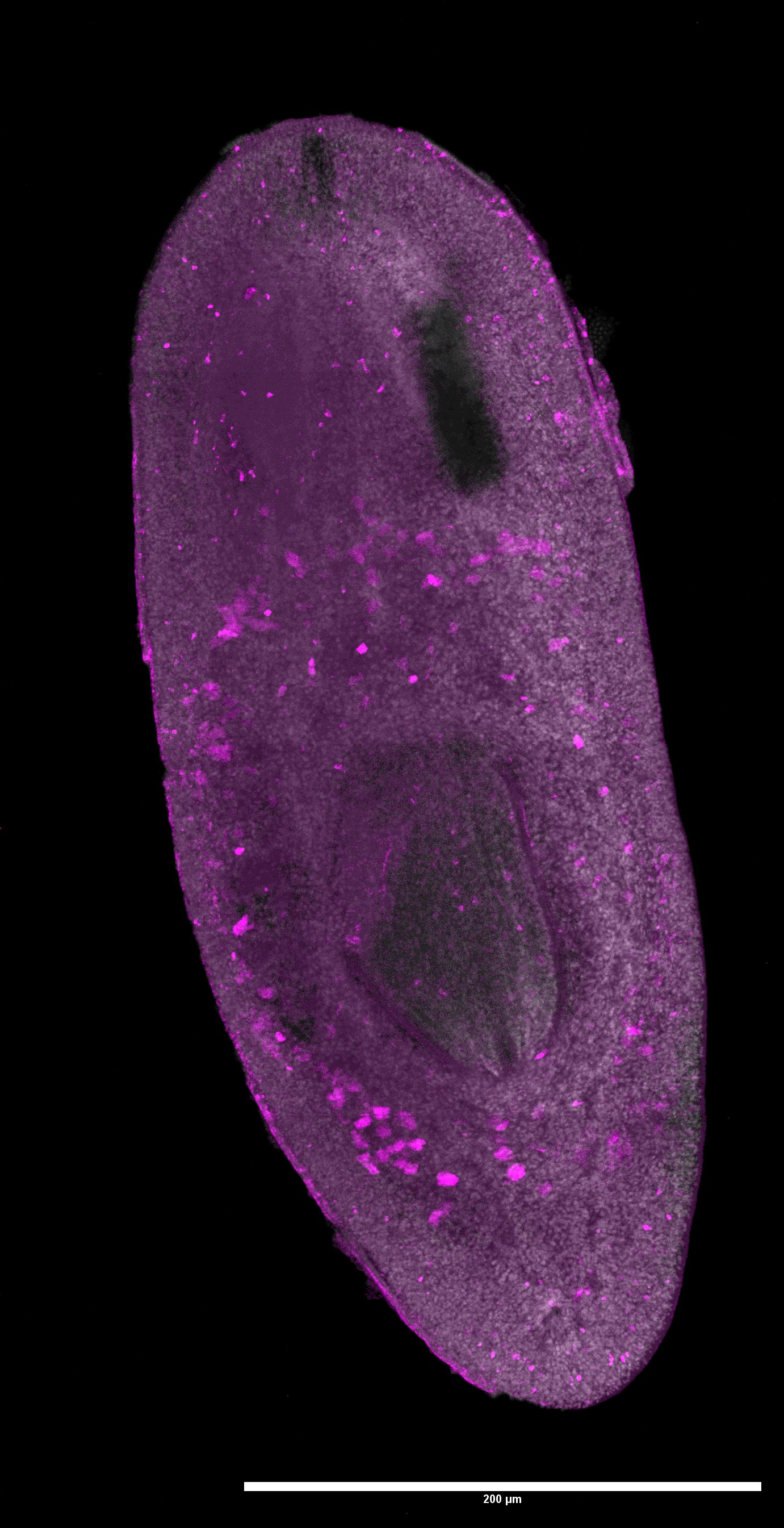

Supplement: Supplementary file 13 — Source data Fig. 6 [file 44318_2025_662_MOESM13_ESM.zip › Figure 6/6B/ID_8_Control_RNAi_Probe_dd1837_rhod_DAPI_10x.jpg]

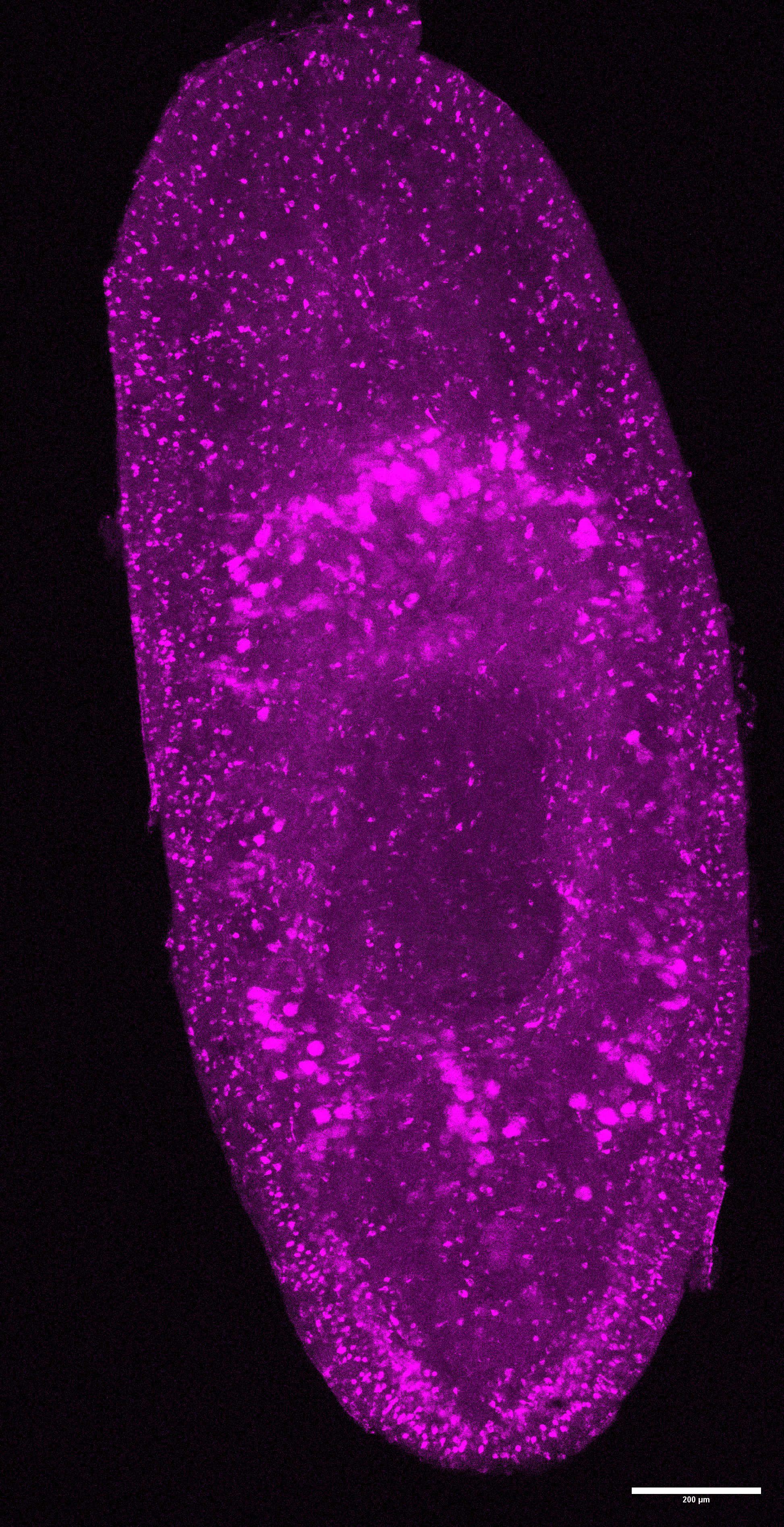

Supplement: Supplementary file 13 — Source data Fig. 6 [file 44318_2025_662_MOESM13_ESM.zip › Figure 6/6B/ID_8_Triple_RNAi_Probe_dd1837_rhod_DAPI_10x.jpg]

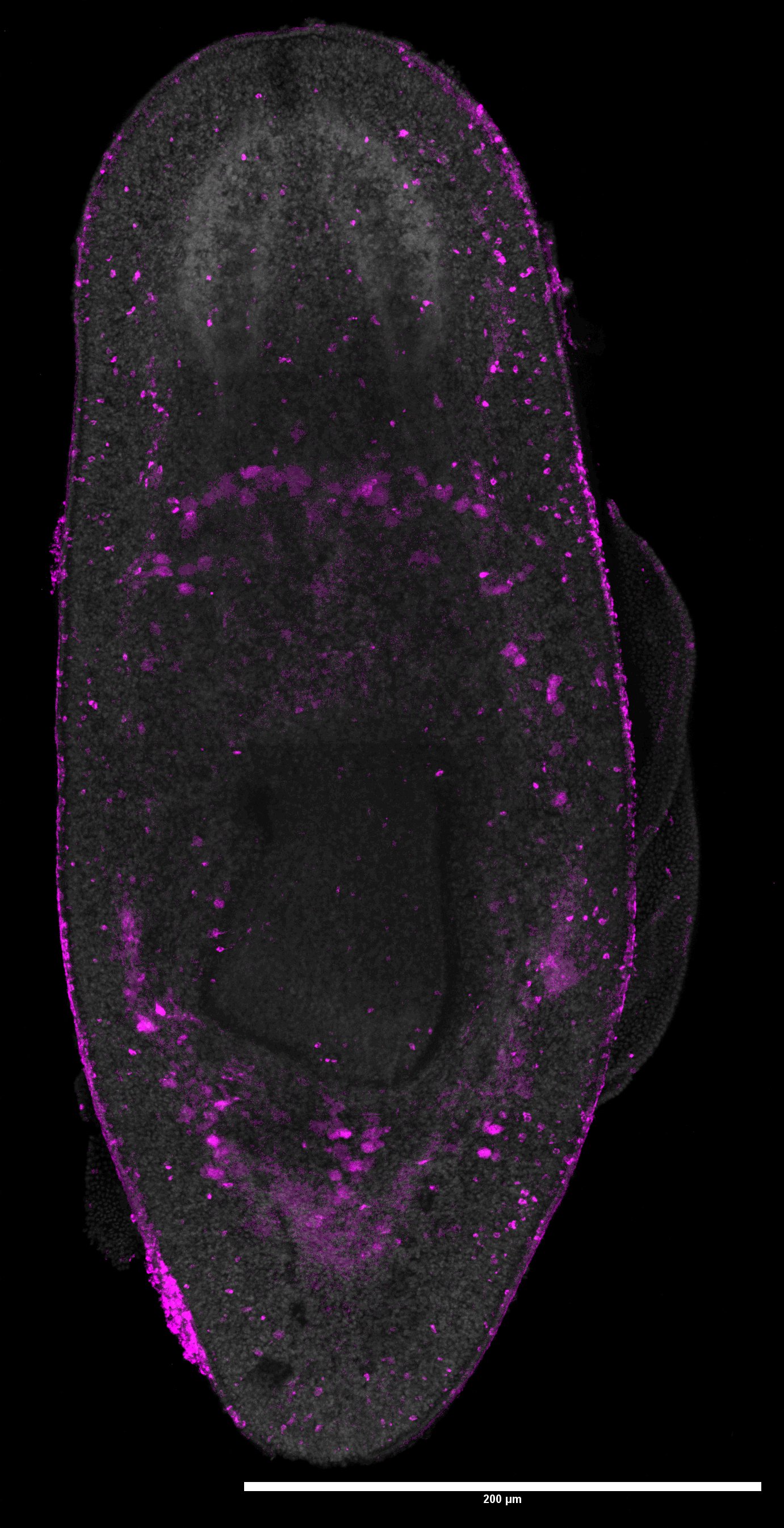

Supplement: Supplementary file 13 — Source data Fig. 6 [file 44318_2025_662_MOESM13_ESM.zip › Figure 6/6B/ID_9_Control_RNAi_Probe_dd1837_rhod_DAPI_10x.jpg]

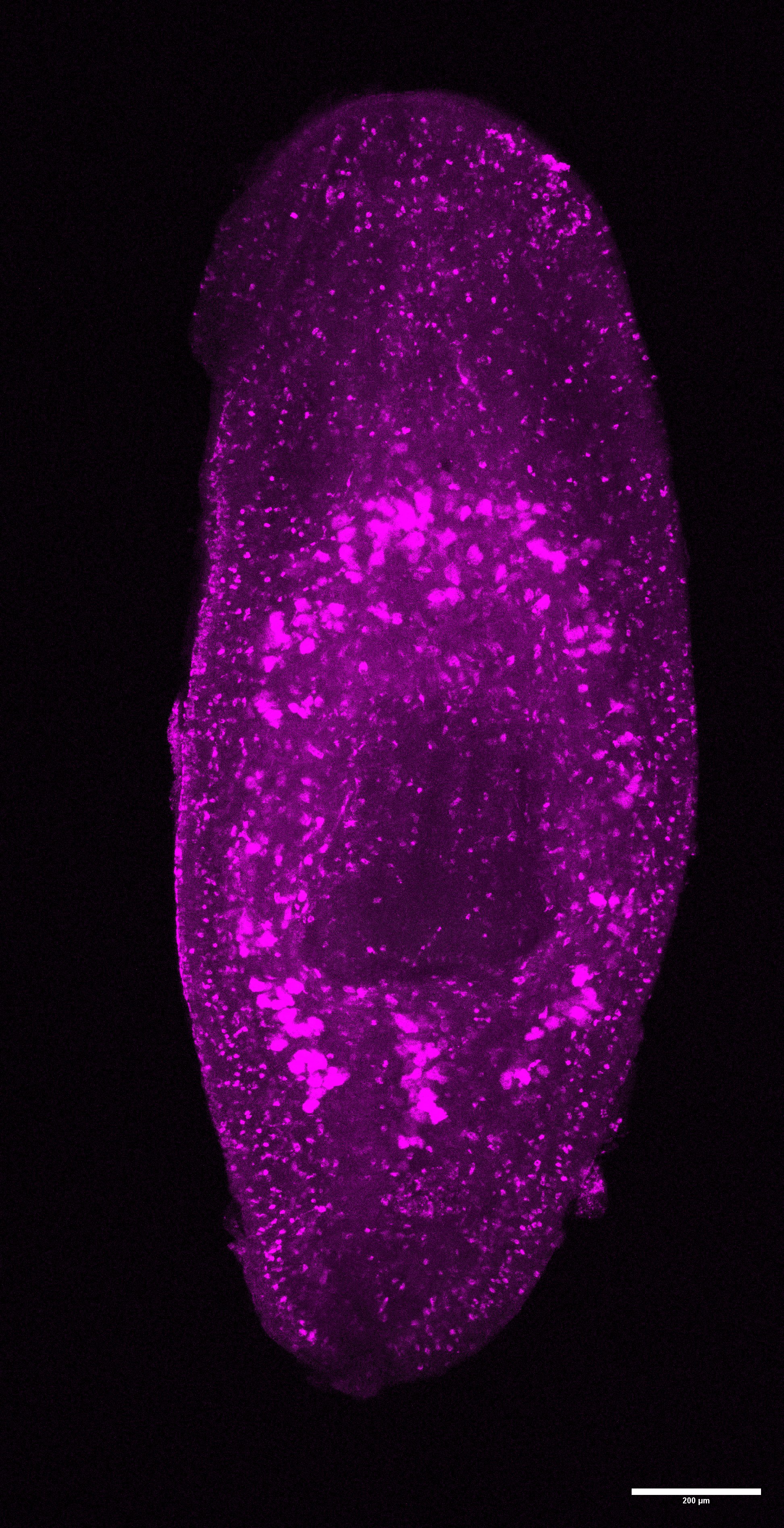

Supplement: Supplementary file 13 — Source data Fig. 6 [file 44318_2025_662_MOESM13_ESM.zip › Figure 6/6B/ID_9_Triple_RNAi_Probe_dd1837_rhod_DAPI_10x.jpg]

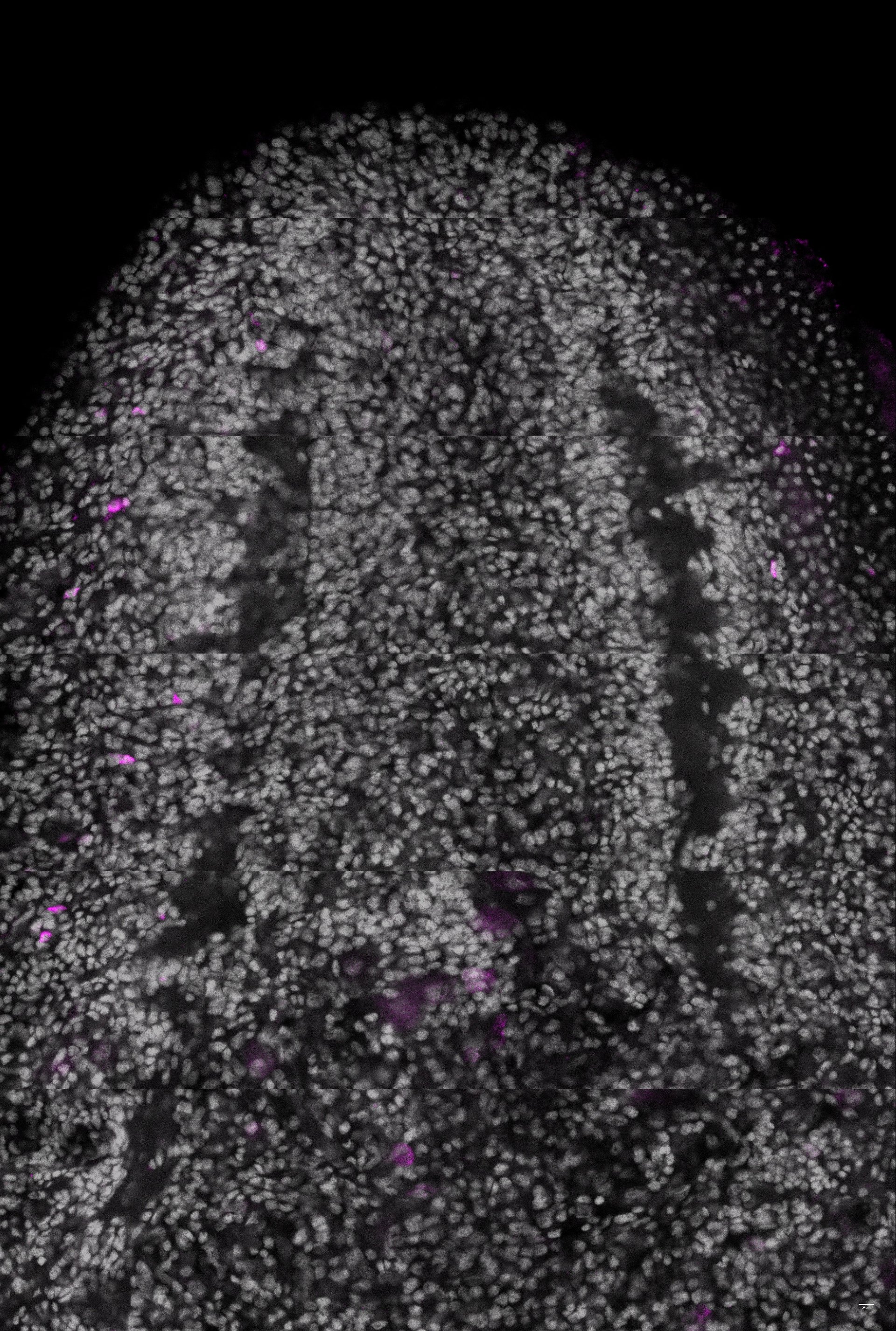

Supplement: Supplementary file 13 — Source data Fig. 6 [file 44318_2025_662_MOESM13_ESM.zip › Figure 6/6C/ID_Control_3_RNAi_Probe_1837_rhod_DAPI_20x_z2_Neural.jpg]

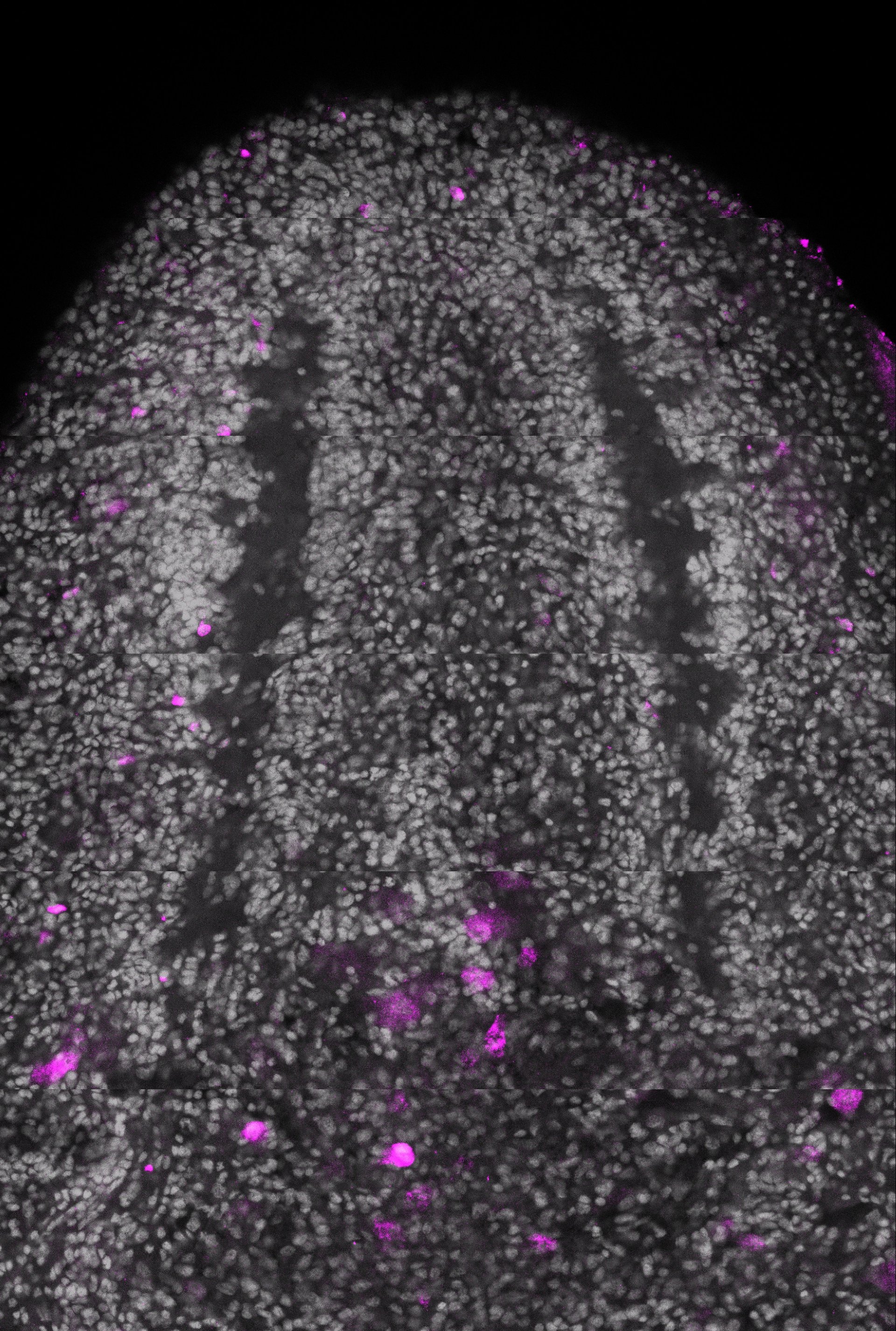

Supplement: Supplementary file 13 — Source data Fig. 6 [file 44318_2025_662_MOESM13_ESM.zip › Figure 6/6C/ID_Control_3_RNAi_Probe_1837_rhod_DAPI_20x_z2_Parenchyma.jpg]

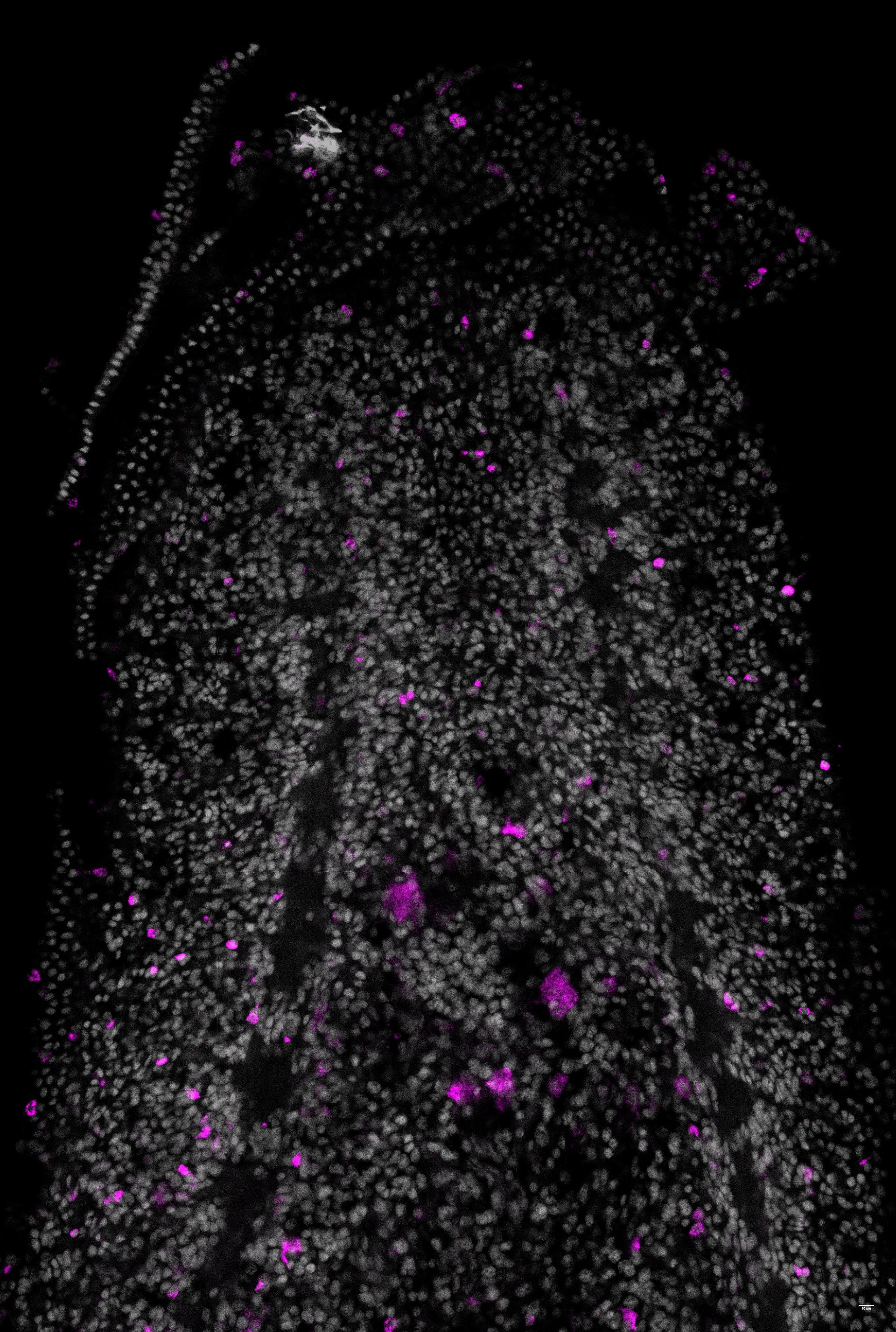

Supplement: Supplementary file 13 — Source data Fig. 6 [file 44318_2025_662_MOESM13_ESM.zip › Figure 6/6C/ID_Triple_2_RNAi_Probe_1837_rhod_DAPI_20x_z2_Neural.jpg]

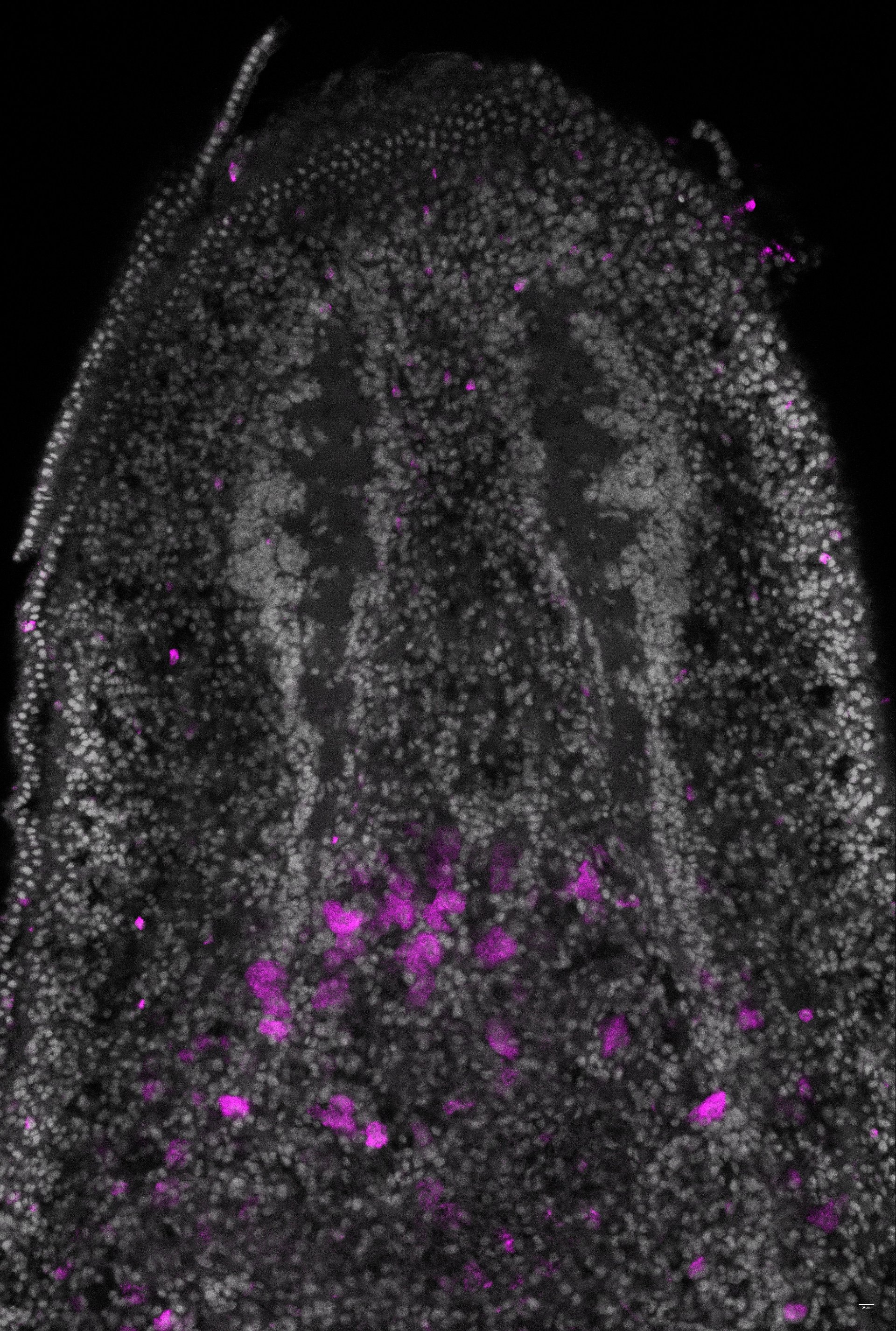

Supplement: Supplementary file 13 — Source data Fig. 6 [file 44318_2025_662_MOESM13_ESM.zip › Figure 6/6C/ID_Triple_2_RNAi_Probe_1837_rhod_DAPI_20x_z2_Parenchyma.jpg]

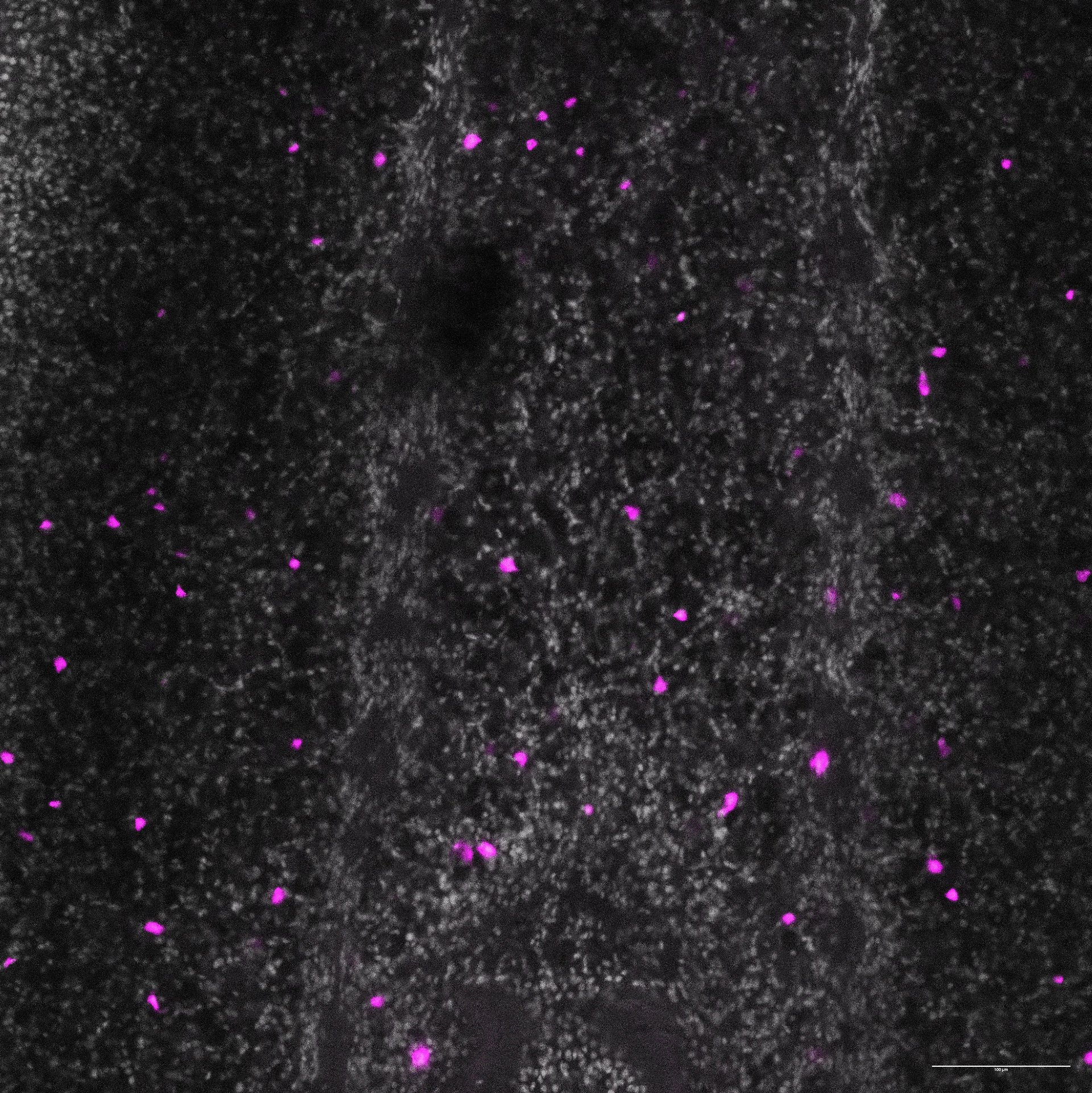

Supplement: Supplementary file 14 — Source data Fig. 7 [file 44318_2025_662_MOESM14_ESM.zip › Figure 7/7B/ID_10_Control_RNAi_H3P_rhod_DAPI_20x.jpg]

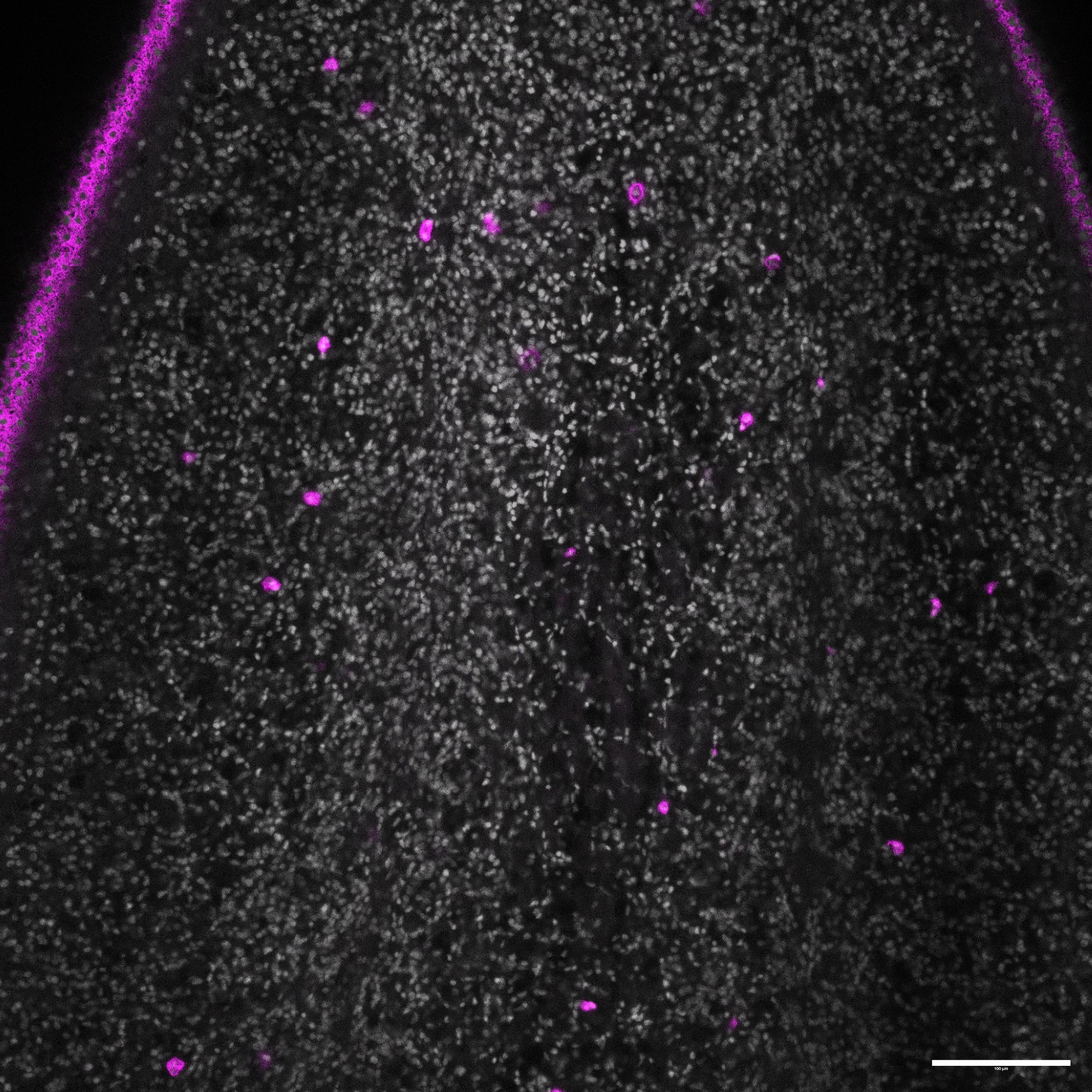

Supplement: Supplementary file 14 — Source data Fig. 7 [file 44318_2025_662_MOESM14_ESM.zip › Figure 7/7B/ID_10_Triple_RNAi_H3P_rhod_DAPI_20x.jpg]

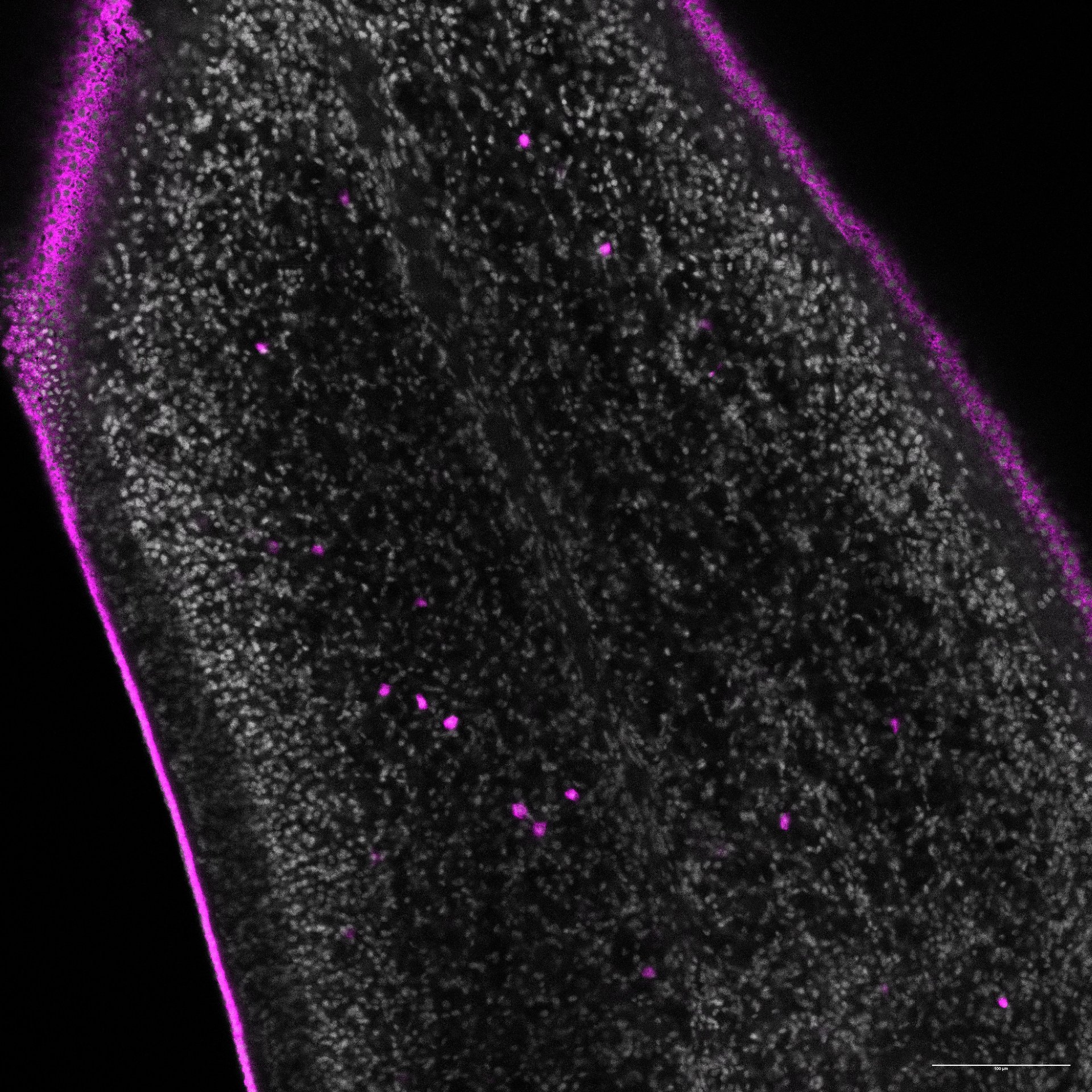

Supplement: Supplementary file 14 — Source data Fig. 7 [file 44318_2025_662_MOESM14_ESM.zip › Figure 7/7B/ID_11_Control_RNAi_H3P_rhod_DAPI_20x.jpg]

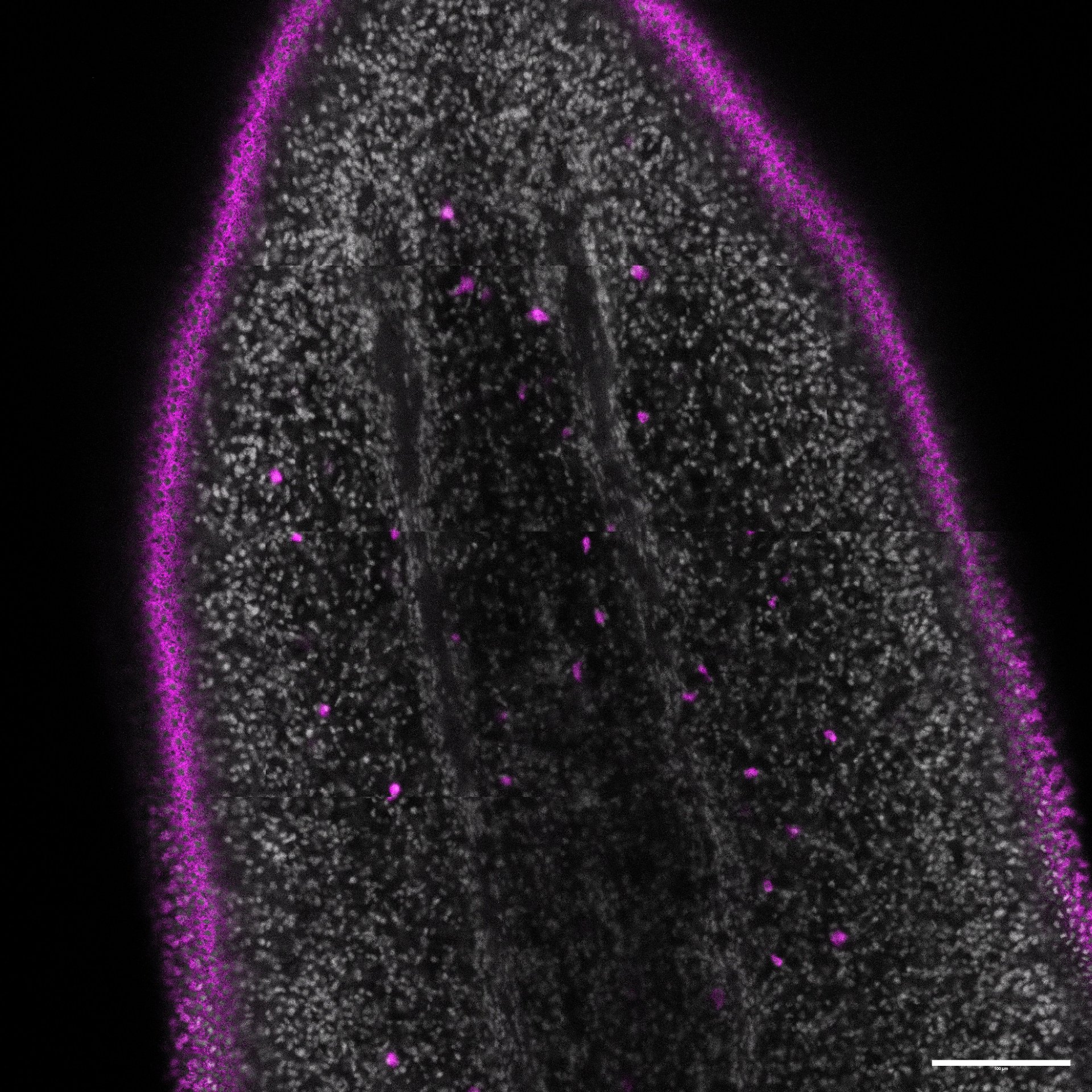

Supplement: Supplementary file 14 — Source data Fig. 7 [file 44318_2025_662_MOESM14_ESM.zip › Figure 7/7B/ID_11_Triple_RNAi_H3P_rhod_DAPI_20x.jpg]

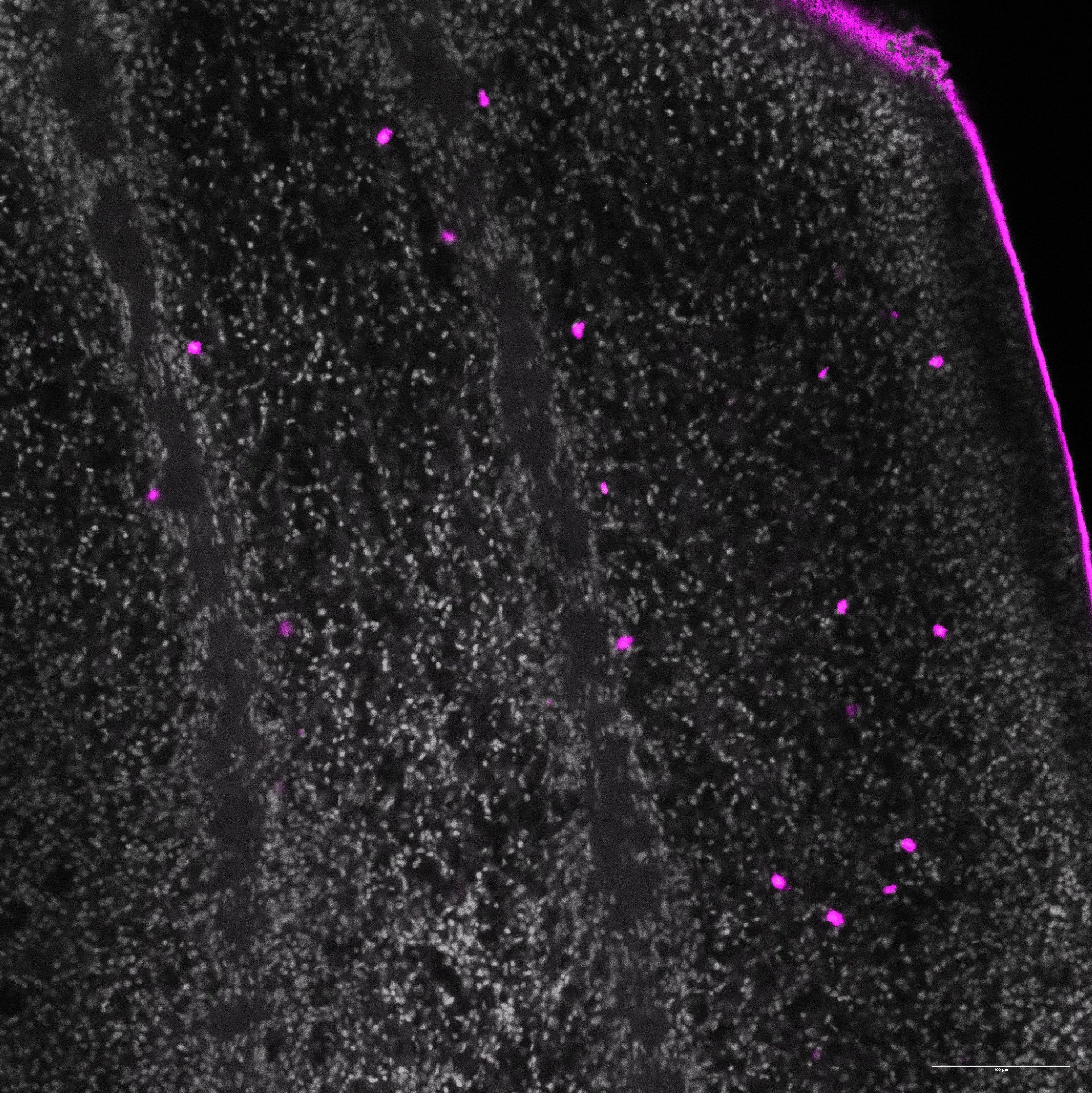

Supplement: Supplementary file 14 — Source data Fig. 7 [file 44318_2025_662_MOESM14_ESM.zip › Figure 7/7B/ID_12_Control_RNAi_H3P_rhod_DAPI_20x.jpg]

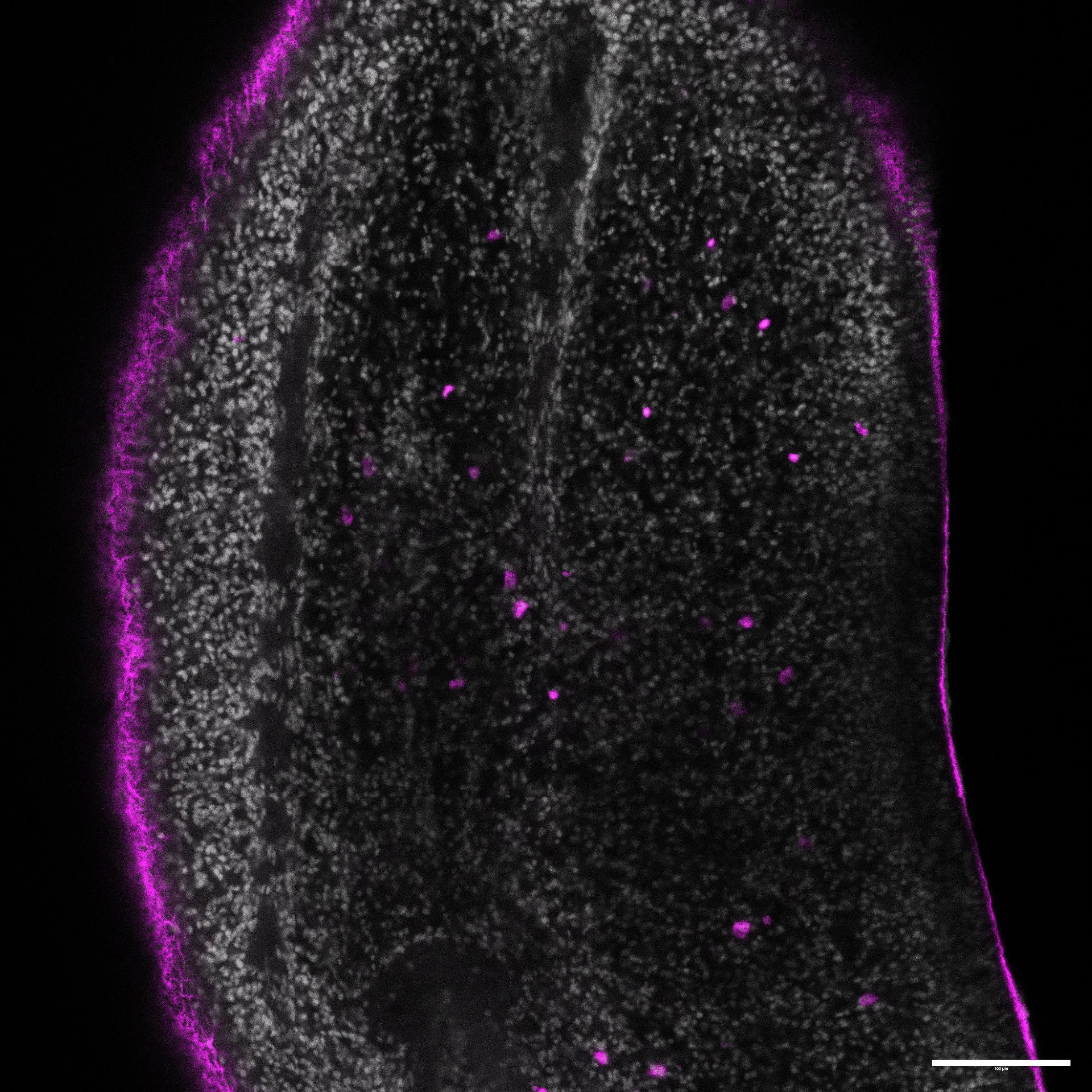

Supplement: Supplementary file 14 — Source data Fig. 7 [file 44318_2025_662_MOESM14_ESM.zip › Figure 7/7B/ID_12_Triple_RNAi_H3P_rhod_DAPI_20x.jpg]

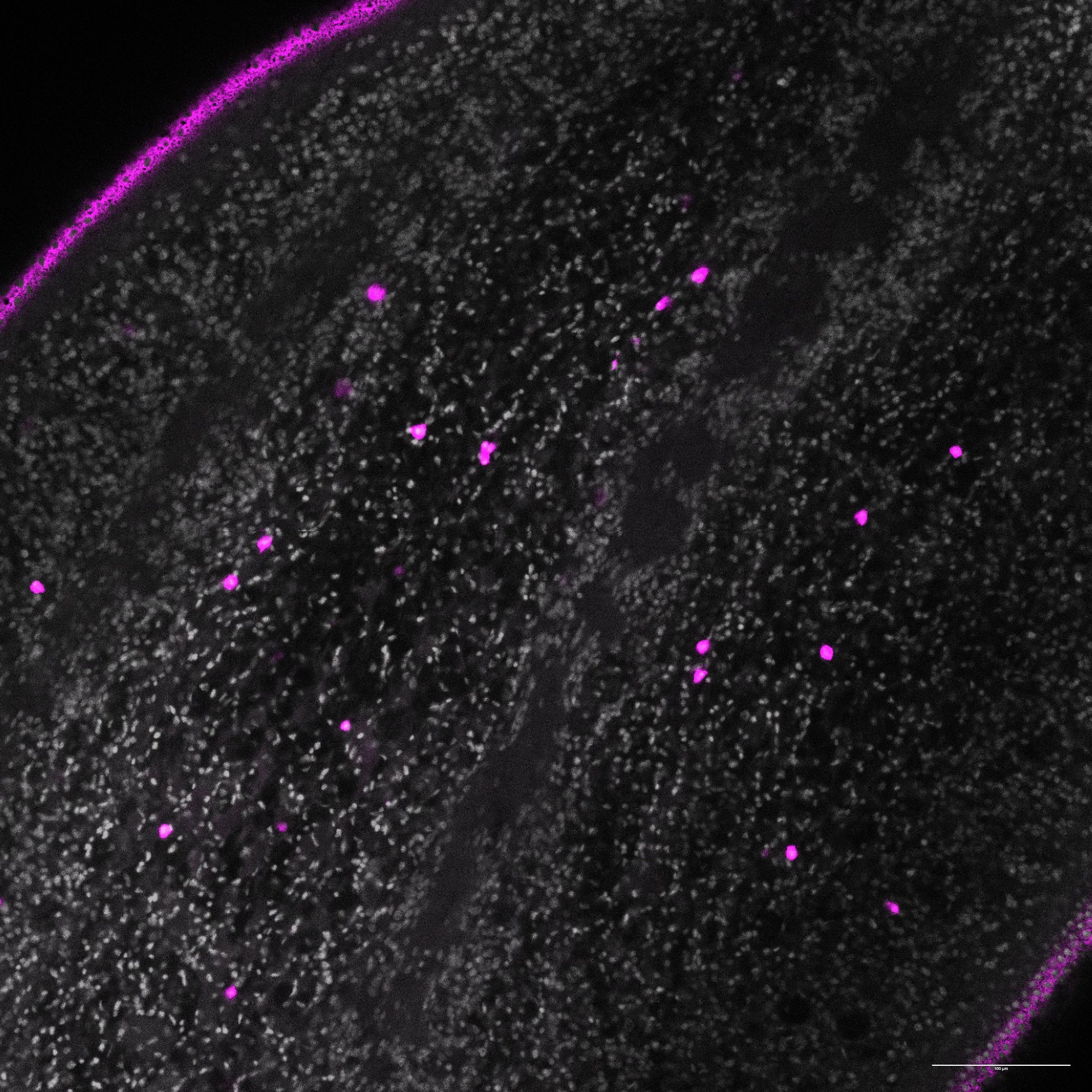

Supplement: Supplementary file 14 — Source data Fig. 7 [file 44318_2025_662_MOESM14_ESM.zip › Figure 7/7B/ID_13_Control_RNAi_H3P_rhod_DAPI_20x.jpg]

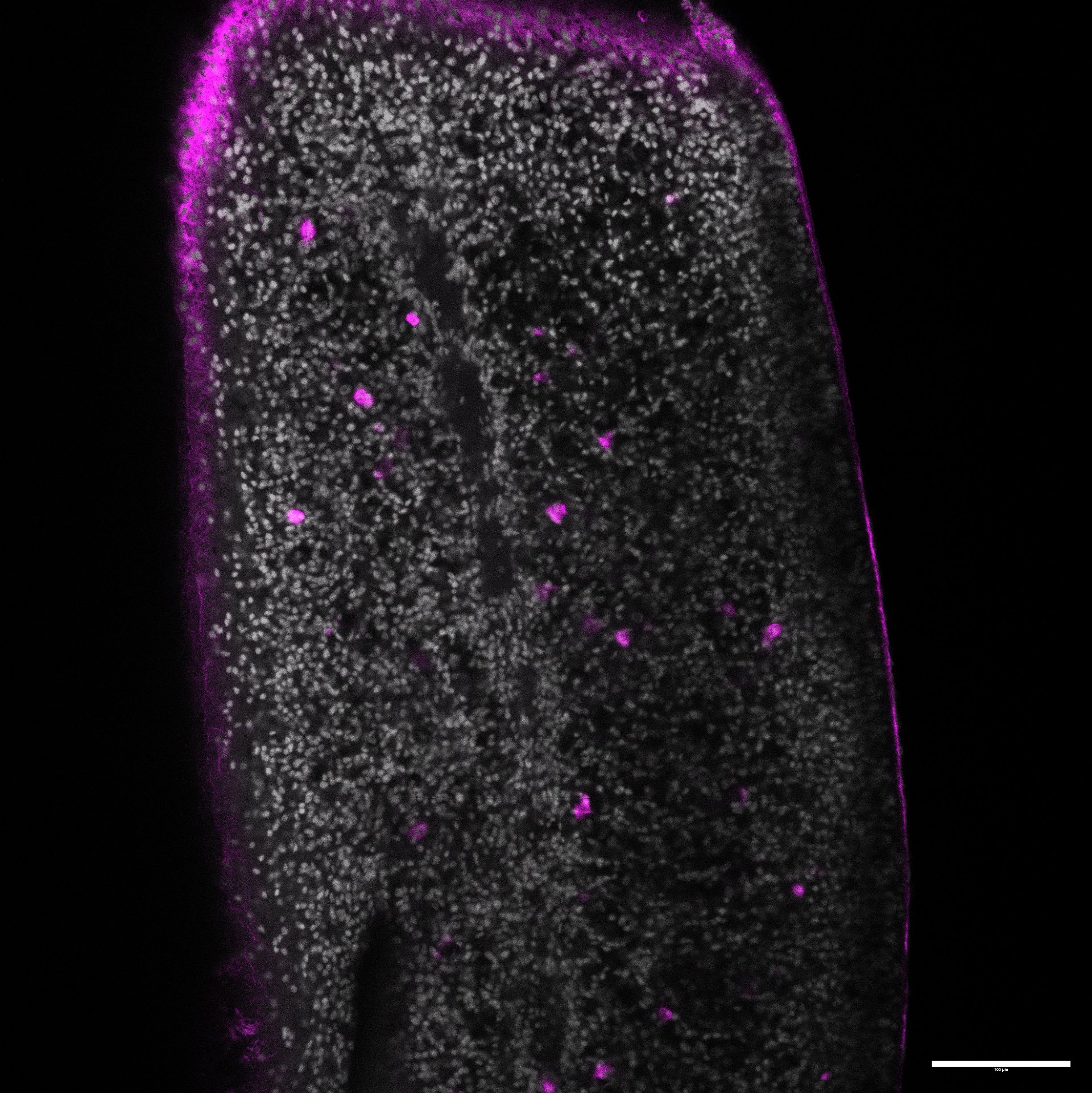

Supplement: Supplementary file 14 — Source data Fig. 7 [file 44318_2025_662_MOESM14_ESM.zip › Figure 7/7B/ID_13_Triple_RNAi_H3P_rhod_DAPI_20x.jpg]

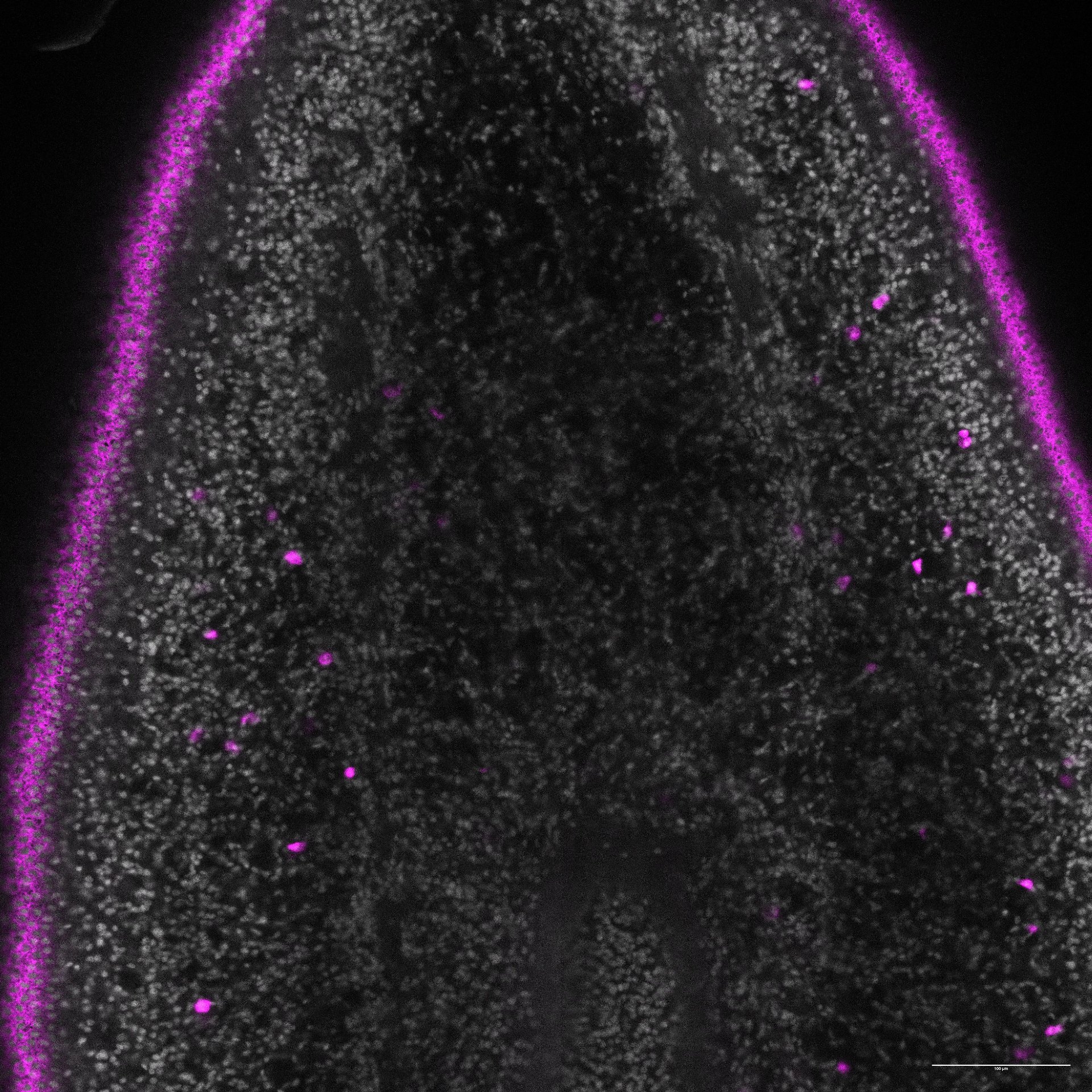

Supplement: Supplementary file 14 — Source data Fig. 7 [file 44318_2025_662_MOESM14_ESM.zip › Figure 7/7B/ID_14_Control_RNAi_H3P_rhod_DAPI_20x.jpg]

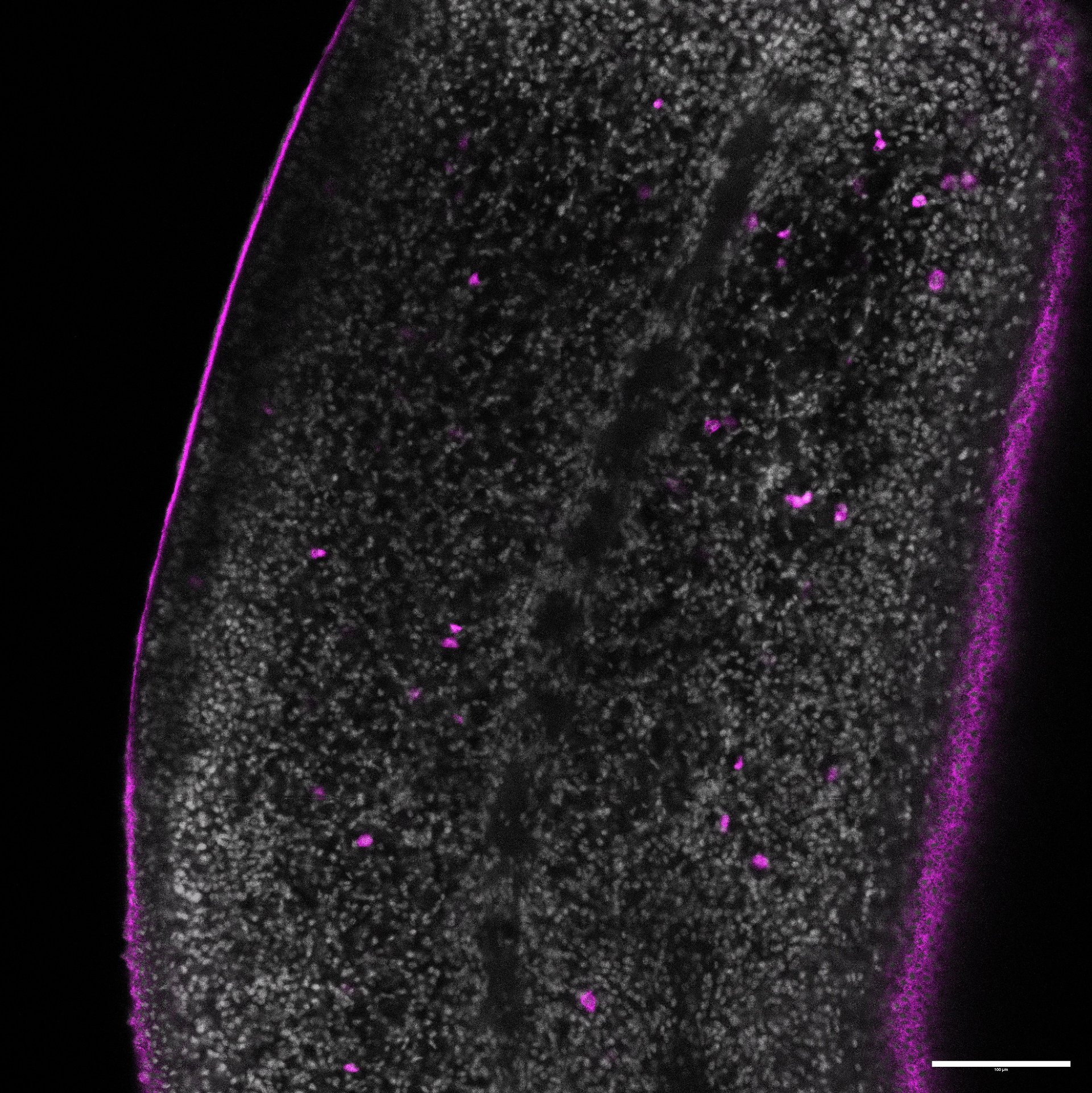

Supplement: Supplementary file 14 — Source data Fig. 7 [file 44318_2025_662_MOESM14_ESM.zip › Figure 7/7B/ID_14_Triple_RNAi_H3P_rhod_DAPI_20x.jpg]

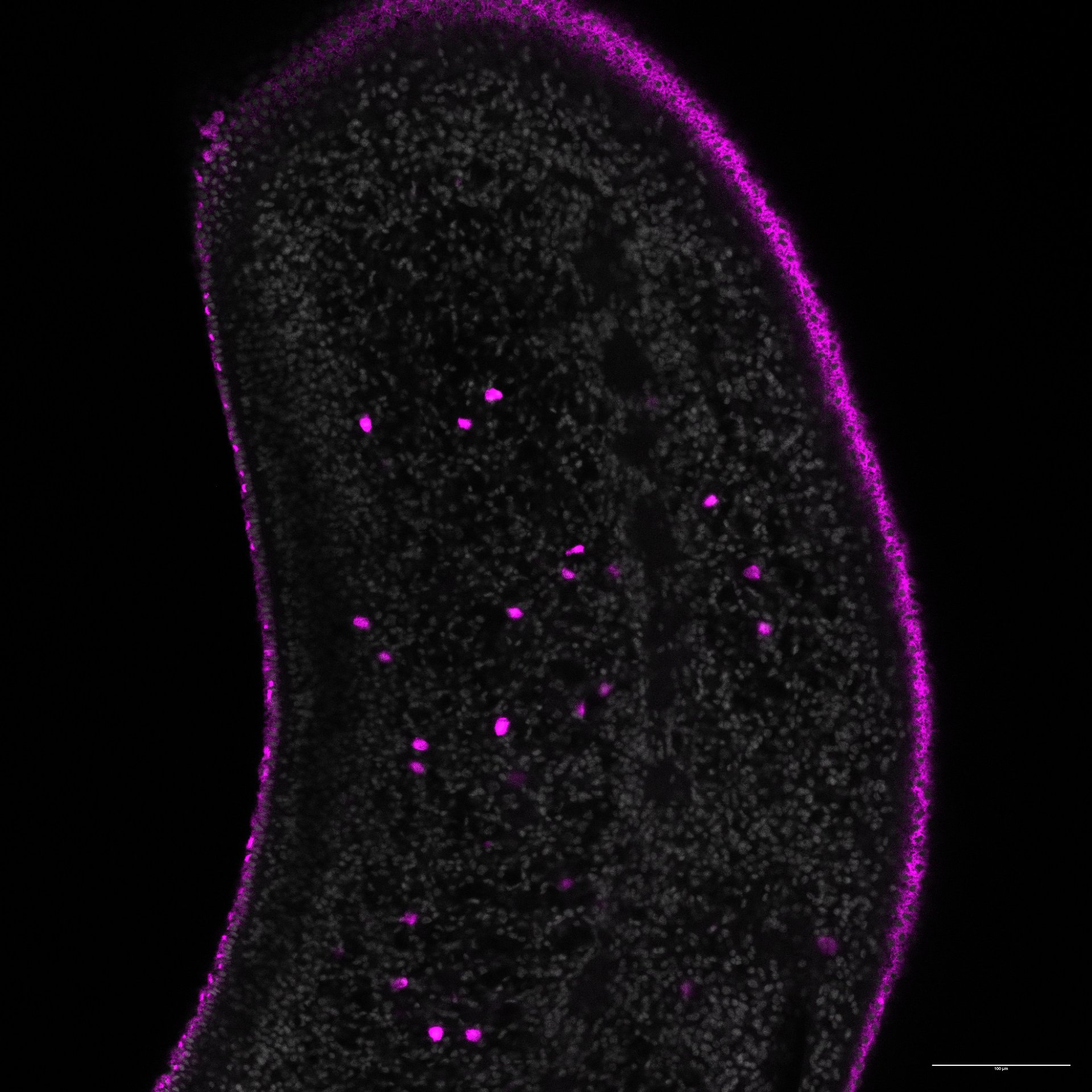

Supplement: Supplementary file 14 — Source data Fig. 7 [file 44318_2025_662_MOESM14_ESM.zip › Figure 7/7B/ID_15_Control_RNAi_H3P_rhod_DAPI_20x.jpg]

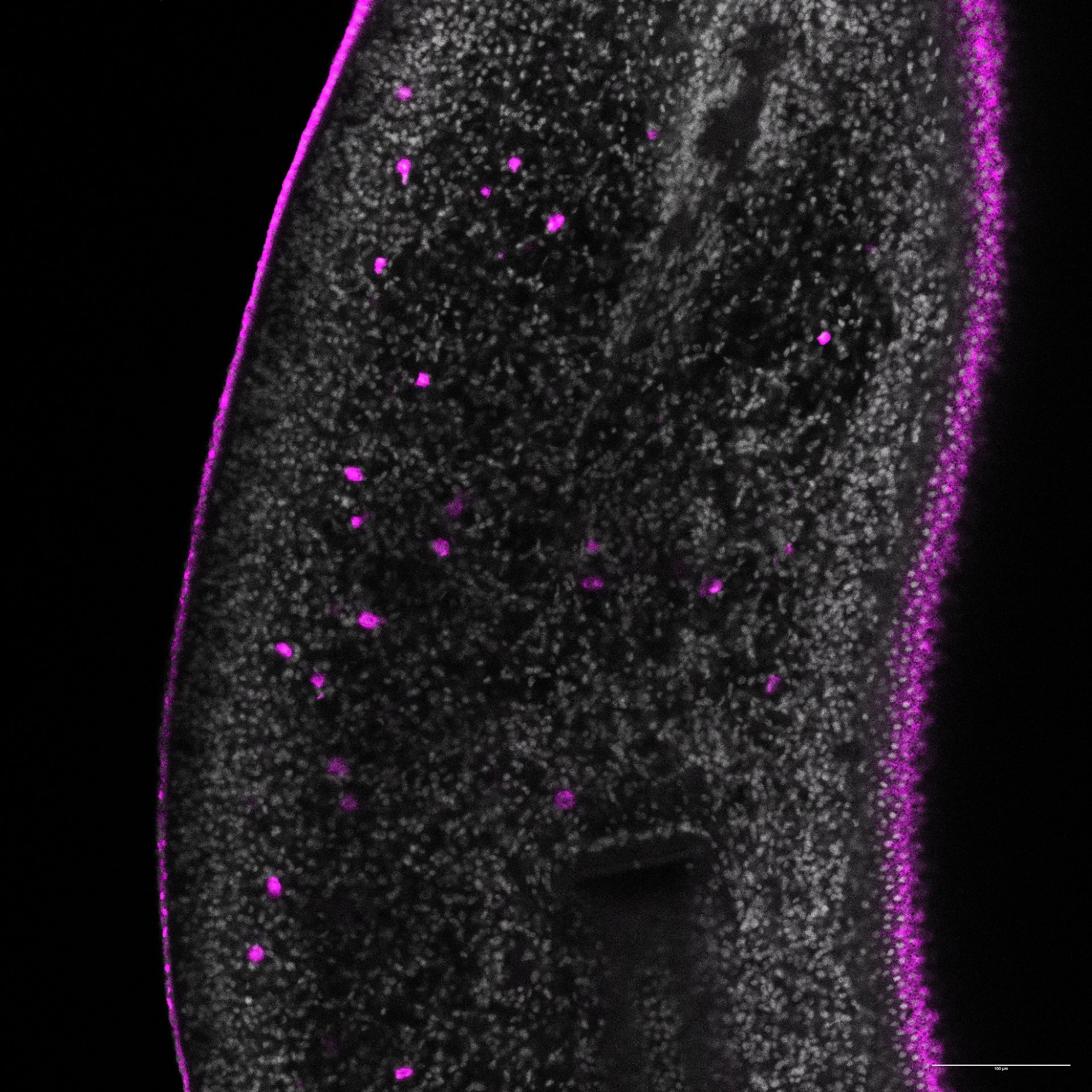

Supplement: Supplementary file 14 — Source data Fig. 7 [file 44318_2025_662_MOESM14_ESM.zip › Figure 7/7B/ID_16_Control_RNAi_H3P_rhod_DAPI_20x.jpg]

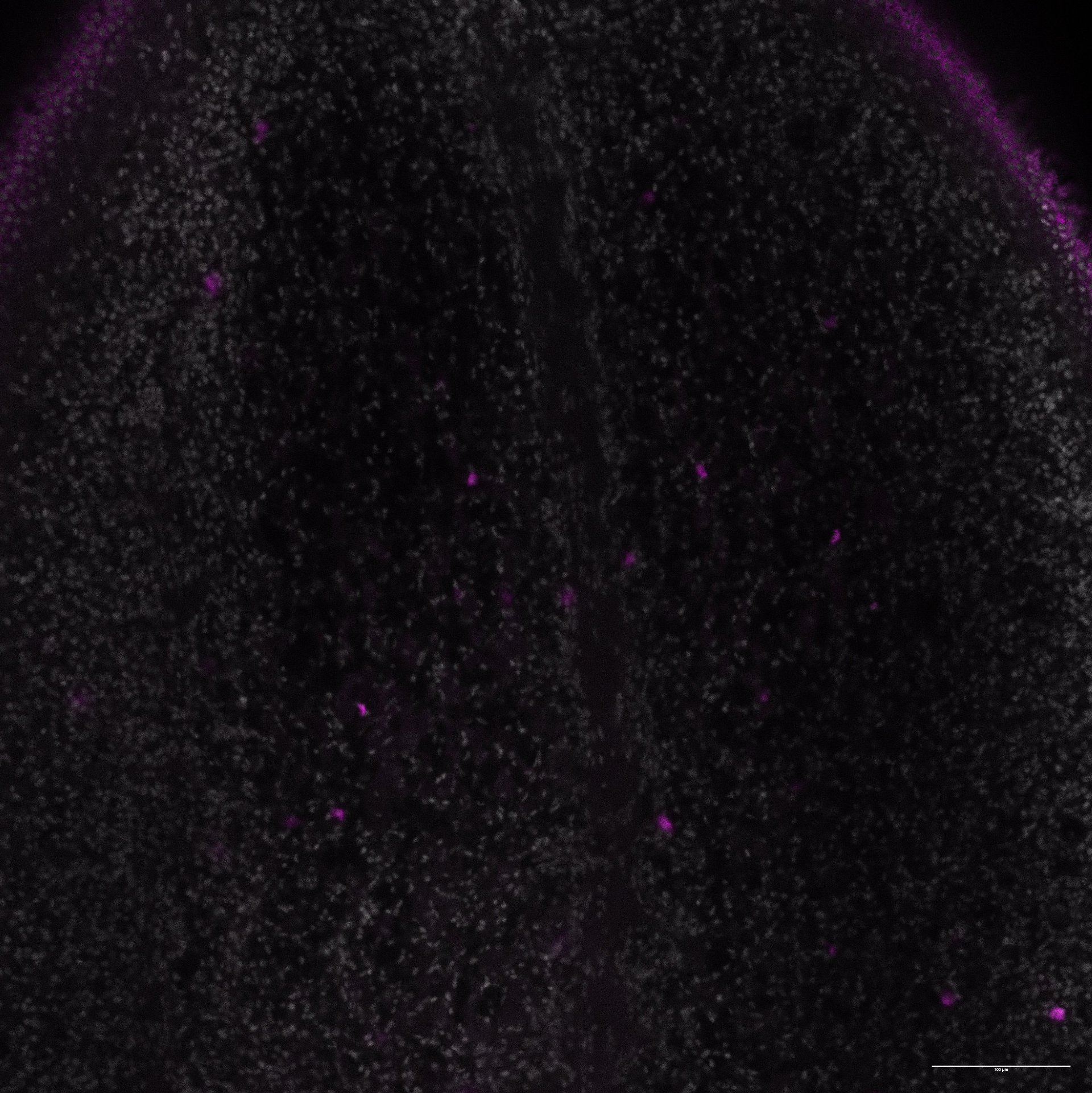

Supplement: Supplementary file 14 — Source data Fig. 7 [file 44318_2025_662_MOESM14_ESM.zip › Figure 7/7B/ID_1_Control_RNAi_H3P_rhod_DAPI_20x.jpg]

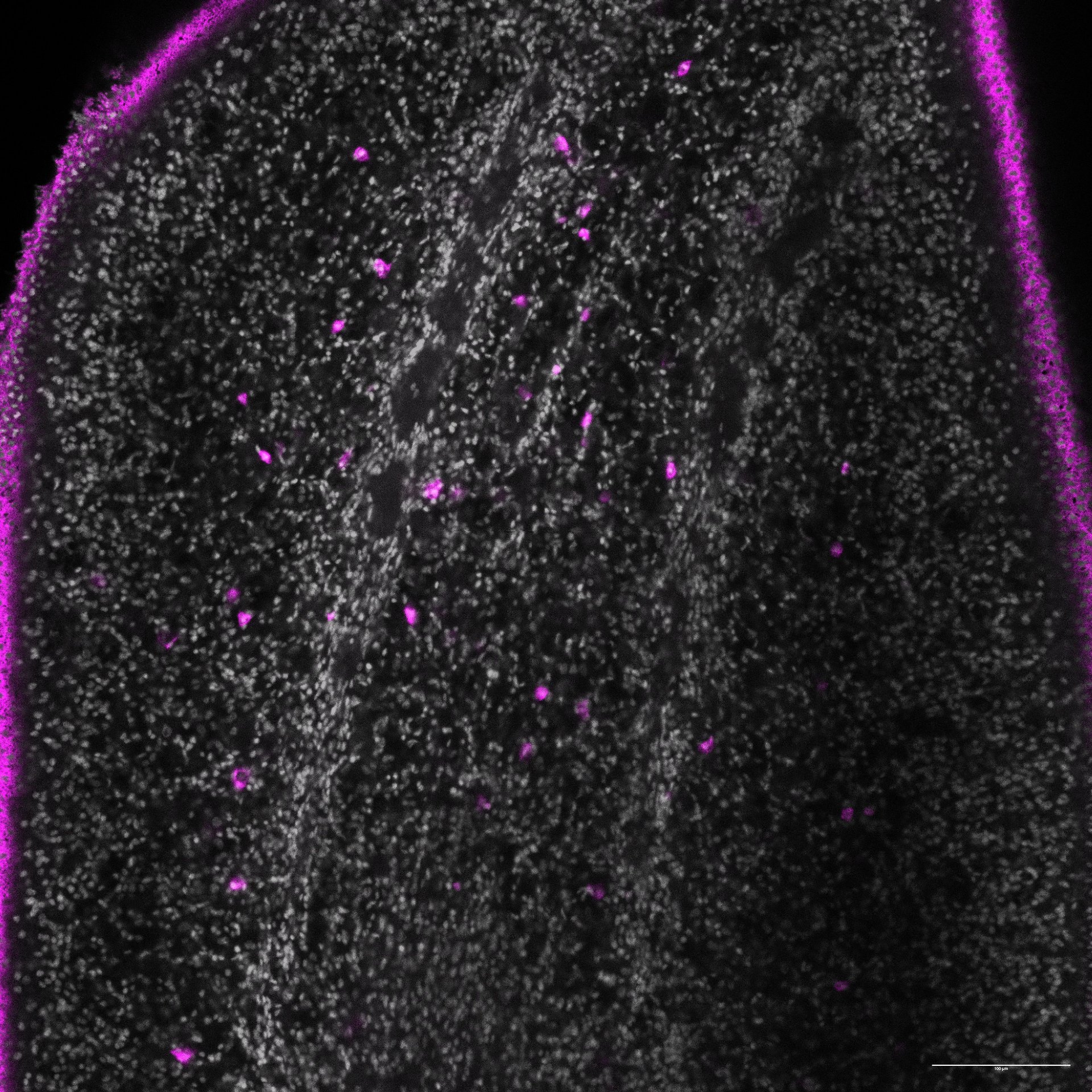

Supplement: Supplementary file 14 — Source data Fig. 7 [file 44318_2025_662_MOESM14_ESM.zip › Figure 7/7B/ID_1_Triple_RNAi_H3P_rhod_DAPI_20x.jpg]

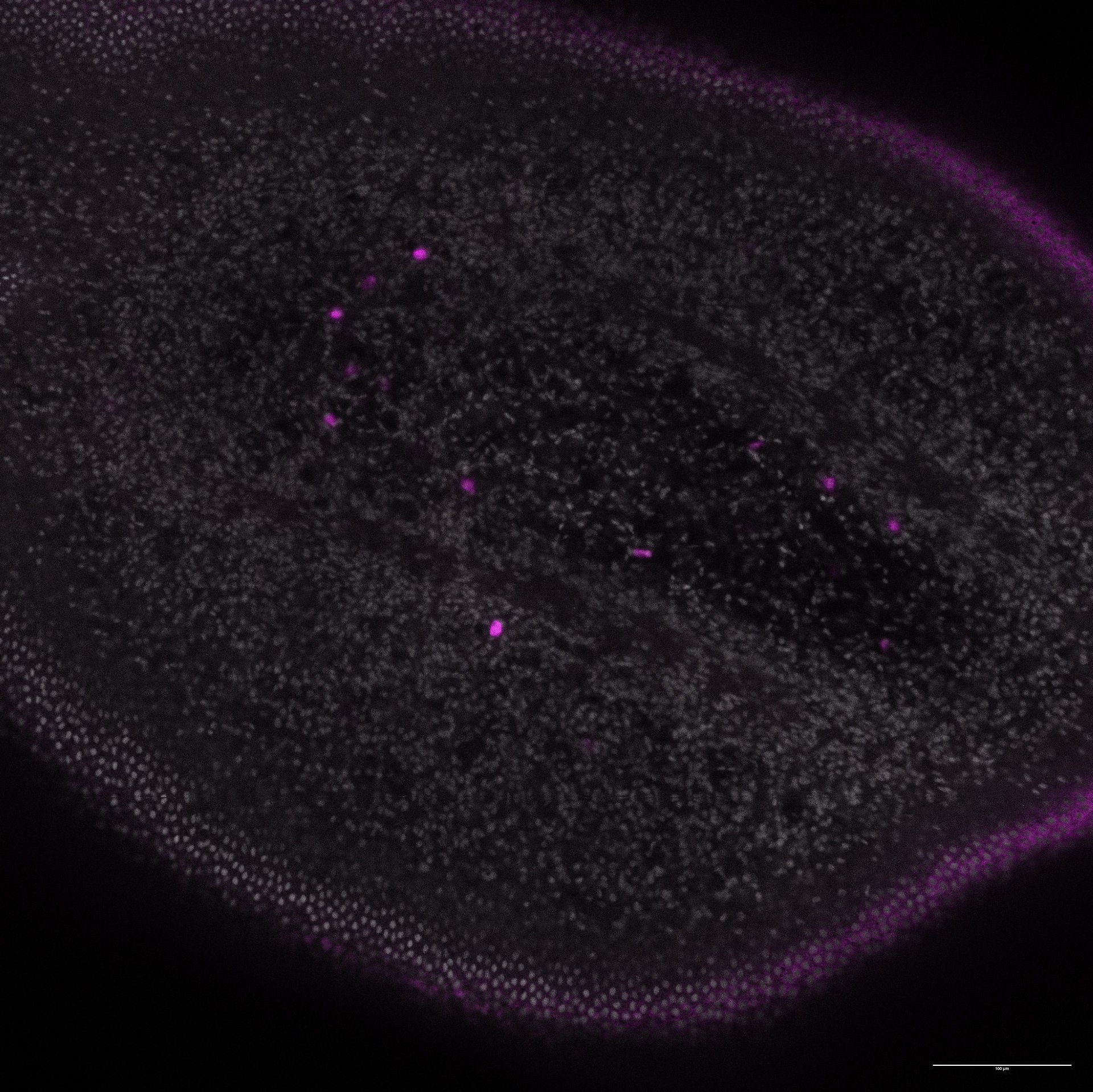

Supplement: Supplementary file 14 — Source data Fig. 7 [file 44318_2025_662_MOESM14_ESM.zip › Figure 7/7B/ID_2_Control_RNAi_H3P_rhod_DAPI_20x.jpg]

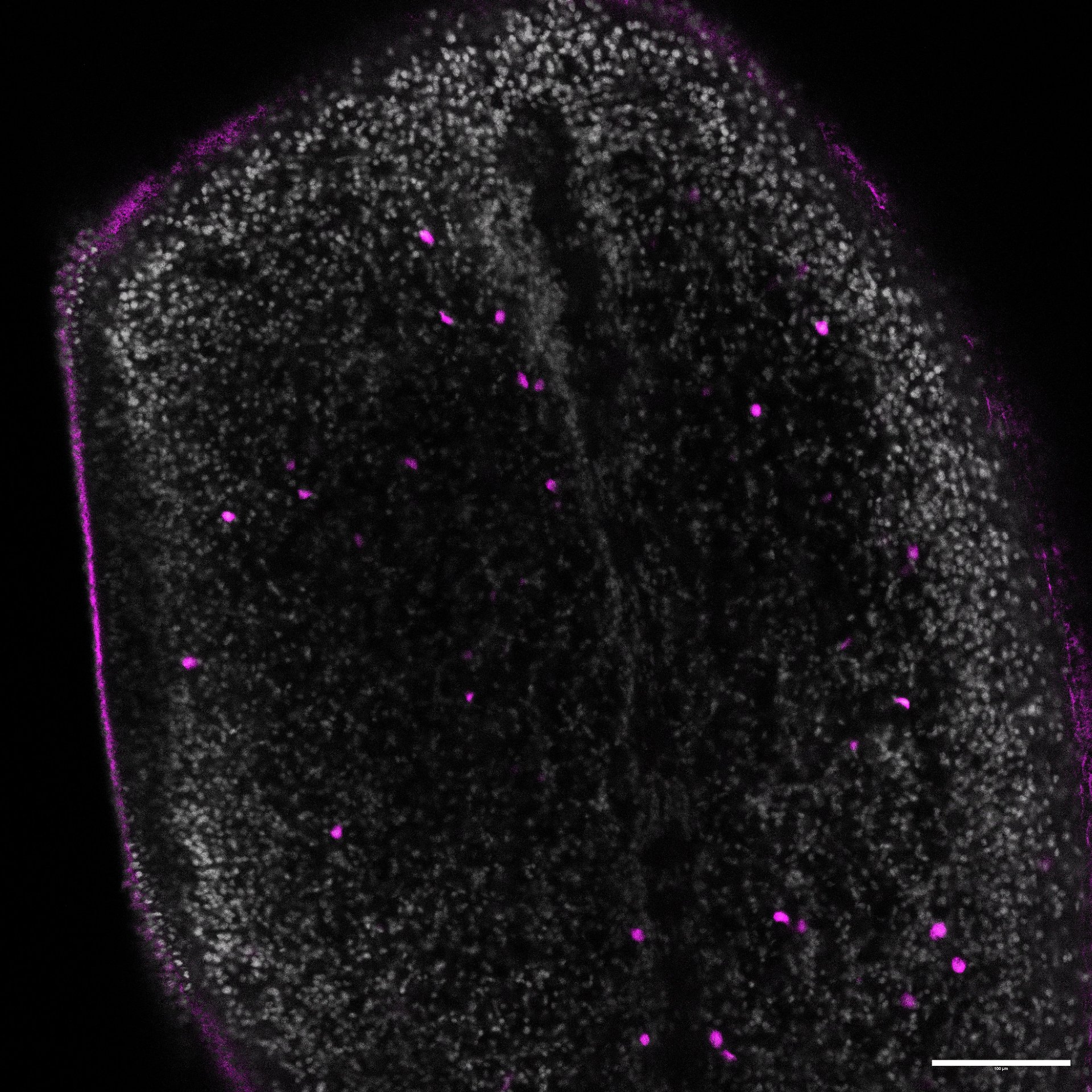

Supplement: Supplementary file 14 — Source data Fig. 7 [file 44318_2025_662_MOESM14_ESM.zip › Figure 7/7B/ID_2_Triple_RNAi_H3P_rhod_DAPI_20x.jpg]

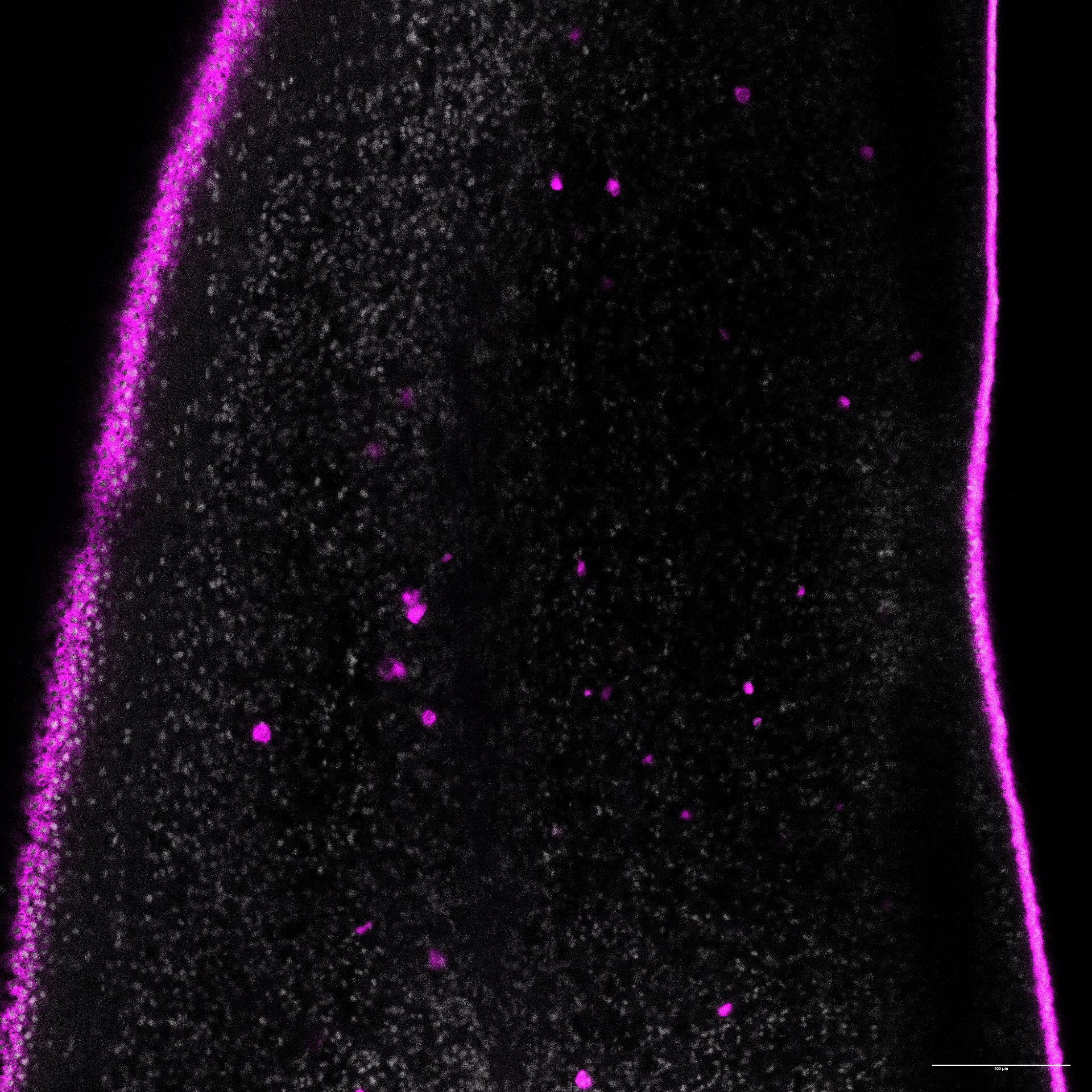

Supplement: Supplementary file 14 — Source data Fig. 7 [file 44318_2025_662_MOESM14_ESM.zip › Figure 7/7B/ID_3_Control_RNAi_H3P_rhod_DAPI_20x.jpg]

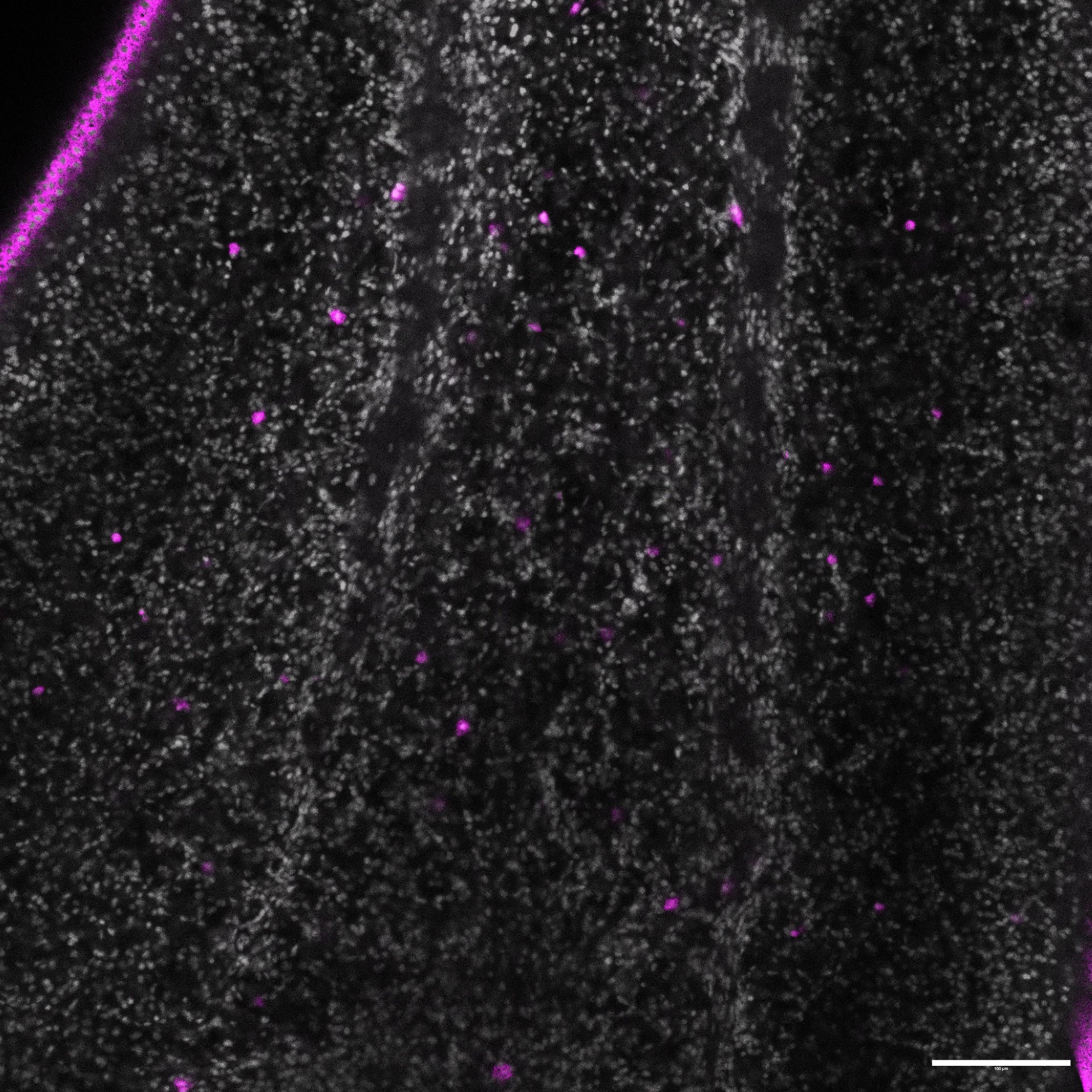

Supplement: Supplementary file 14 — Source data Fig. 7 [file 44318_2025_662_MOESM14_ESM.zip › Figure 7/7B/ID_3_Triple_RNAi_H3P_rhod_DAPI_20x.jpg]

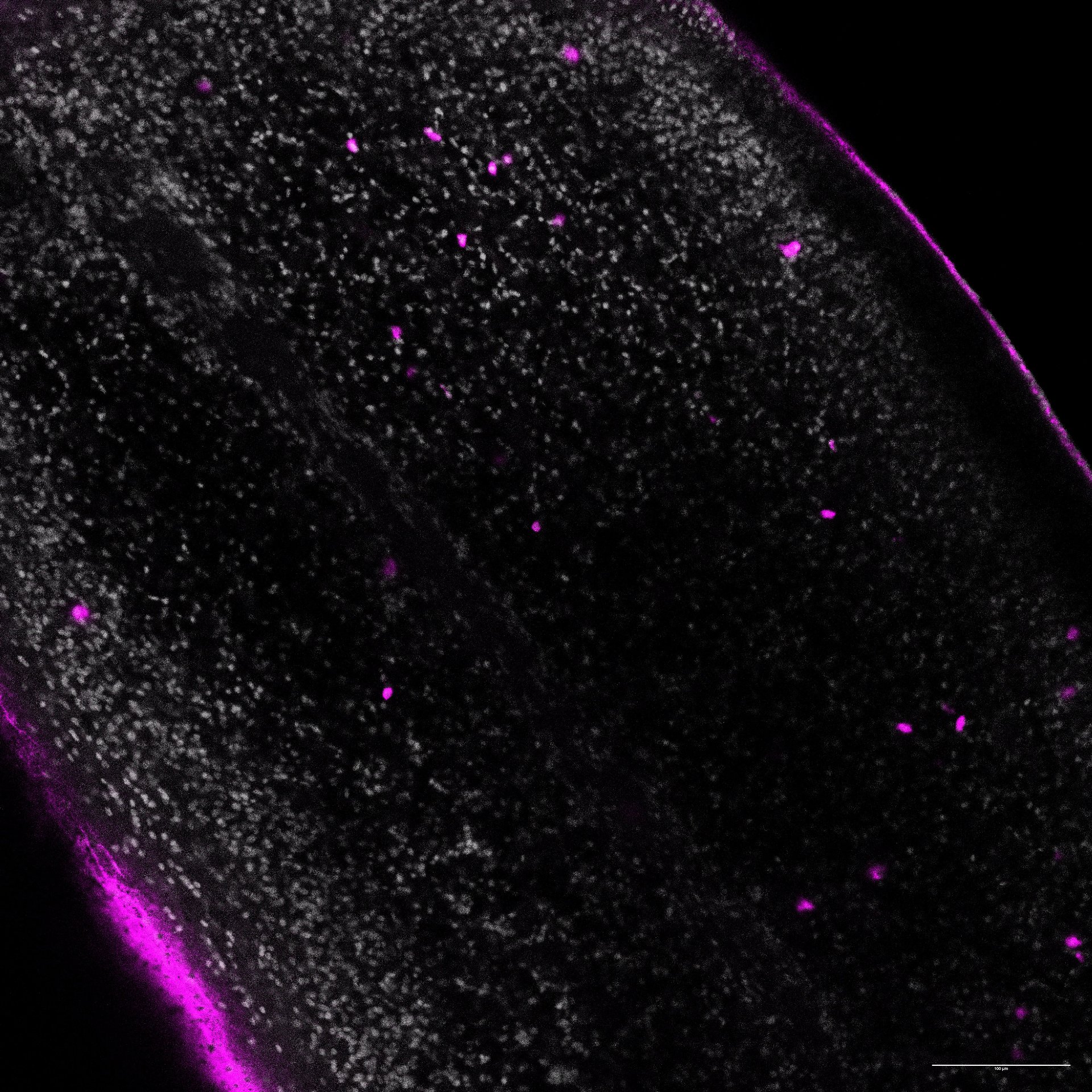

Supplement: Supplementary file 14 — Source data Fig. 7 [file 44318_2025_662_MOESM14_ESM.zip › Figure 7/7B/ID_4_Control_RNAi_H3P_rhod_DAPI_20x.jpg]

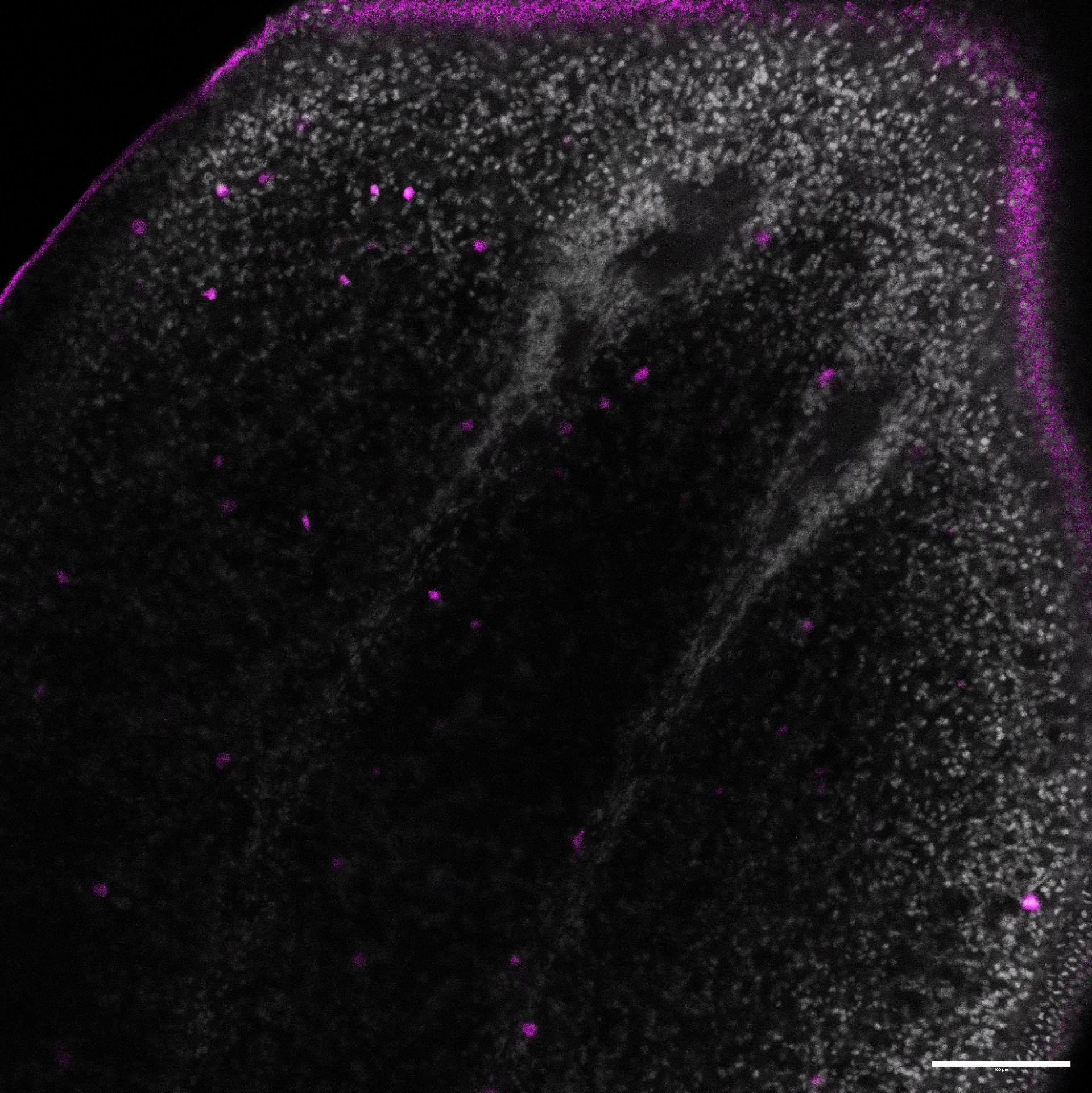

Supplement: Supplementary file 14 — Source data Fig. 7 [file 44318_2025_662_MOESM14_ESM.zip › Figure 7/7B/ID_4_Triple_RNAi_H3P_rhod_DAPI_20x.jpg]

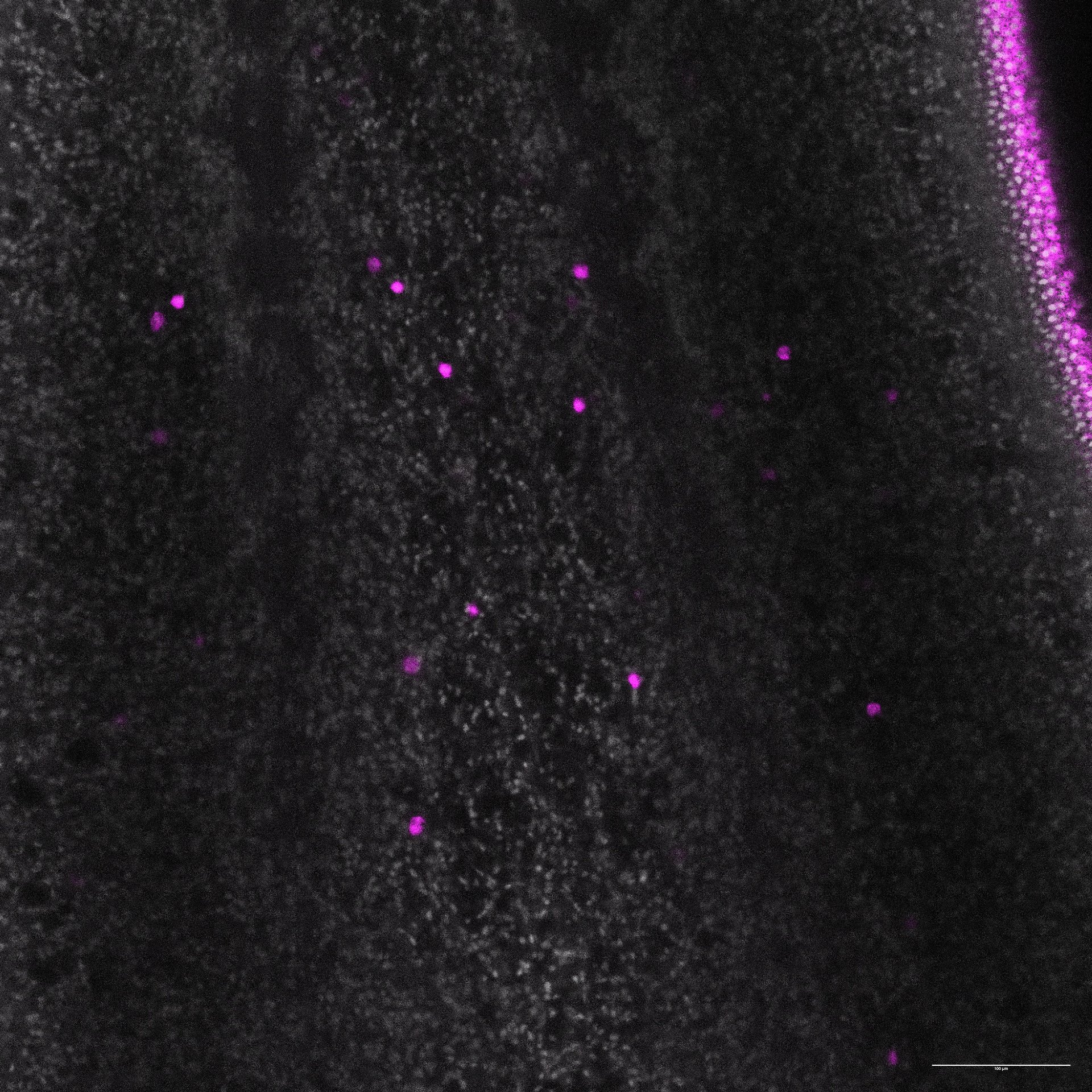

Supplement: Supplementary file 14 — Source data Fig. 7 [file 44318_2025_662_MOESM14_ESM.zip › Figure 7/7B/ID_5_Control_RNAi_H3P_rhod_DAPI_20x.jpg]
