# Supplementary figures and images for: Single-nucleotide m⁶A mapping uncovers redundant YTHDF function in planarian progenitor fate selection (part 6 of 6)
Source: EMBO J. 2026 Jan 3;45(3):749–88. doi: 10.1038/s44318-025-00662-3 (PMC12864844; doi:10.1038/s44318-025-00662-3)

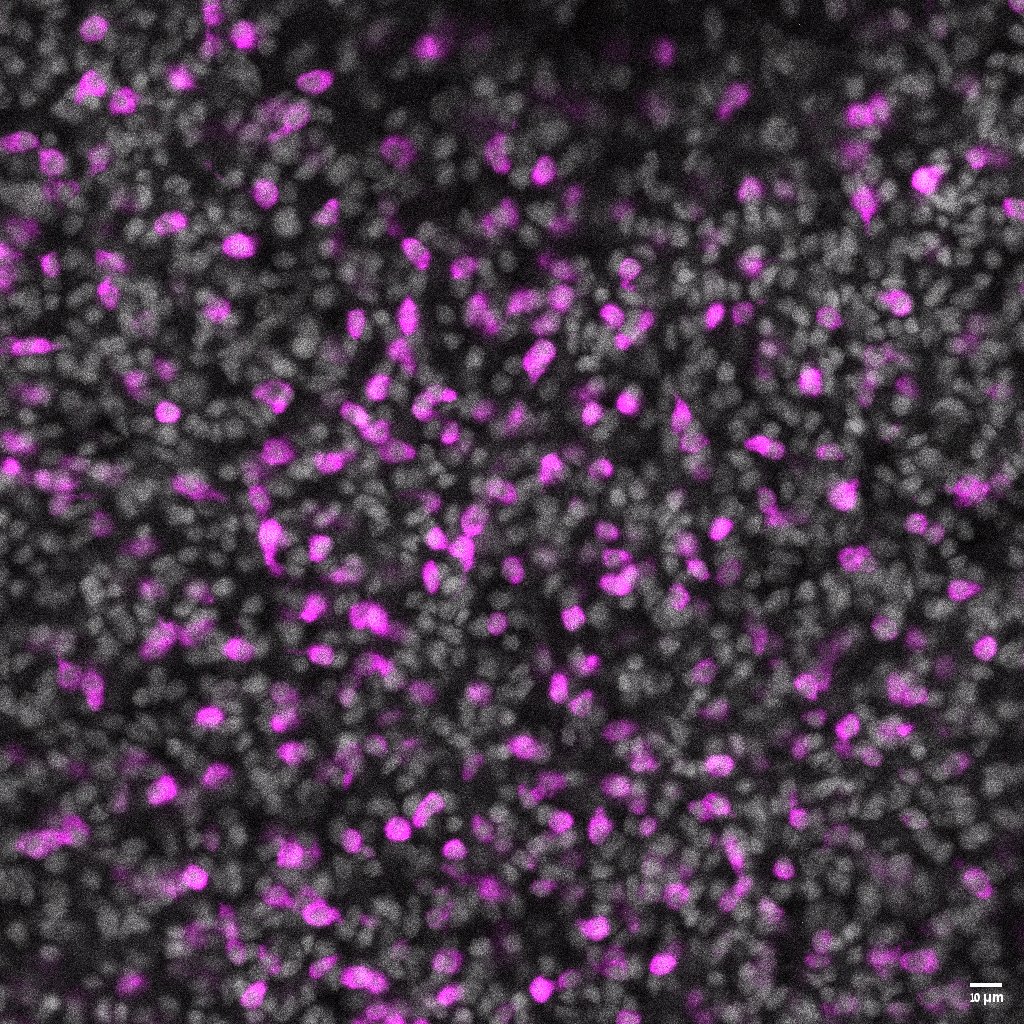

Supplement: Supplementary file 14 — Source data Fig. 7 [file 44318_2025_662_MOESM14_ESM.zip › Figure 7/7F/Main_figure_panel_Triple_RNAi_Probe_prog2_rhod_DAPI_20x_z1_Merged.jpg]

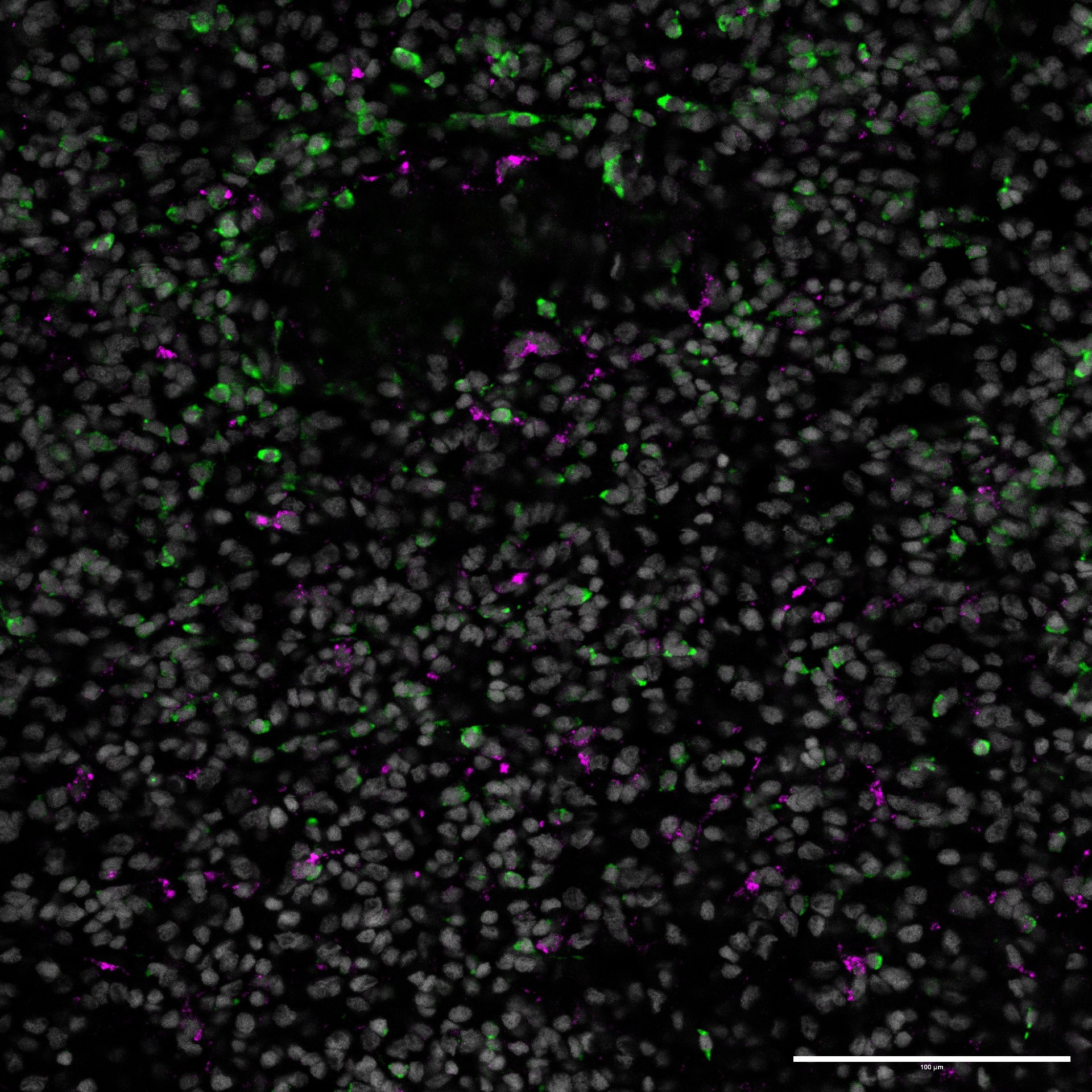

Supplement: Supplementary file 14 — Source data Fig. 7 [file 44318_2025_662_MOESM14_ESM.zip › Figure 7/7G/ID_10_Region_1_Control_RNAi_Probe_dd234_rhod_SMEDWI1_FITC_DAPI_20x_z3.jpg]

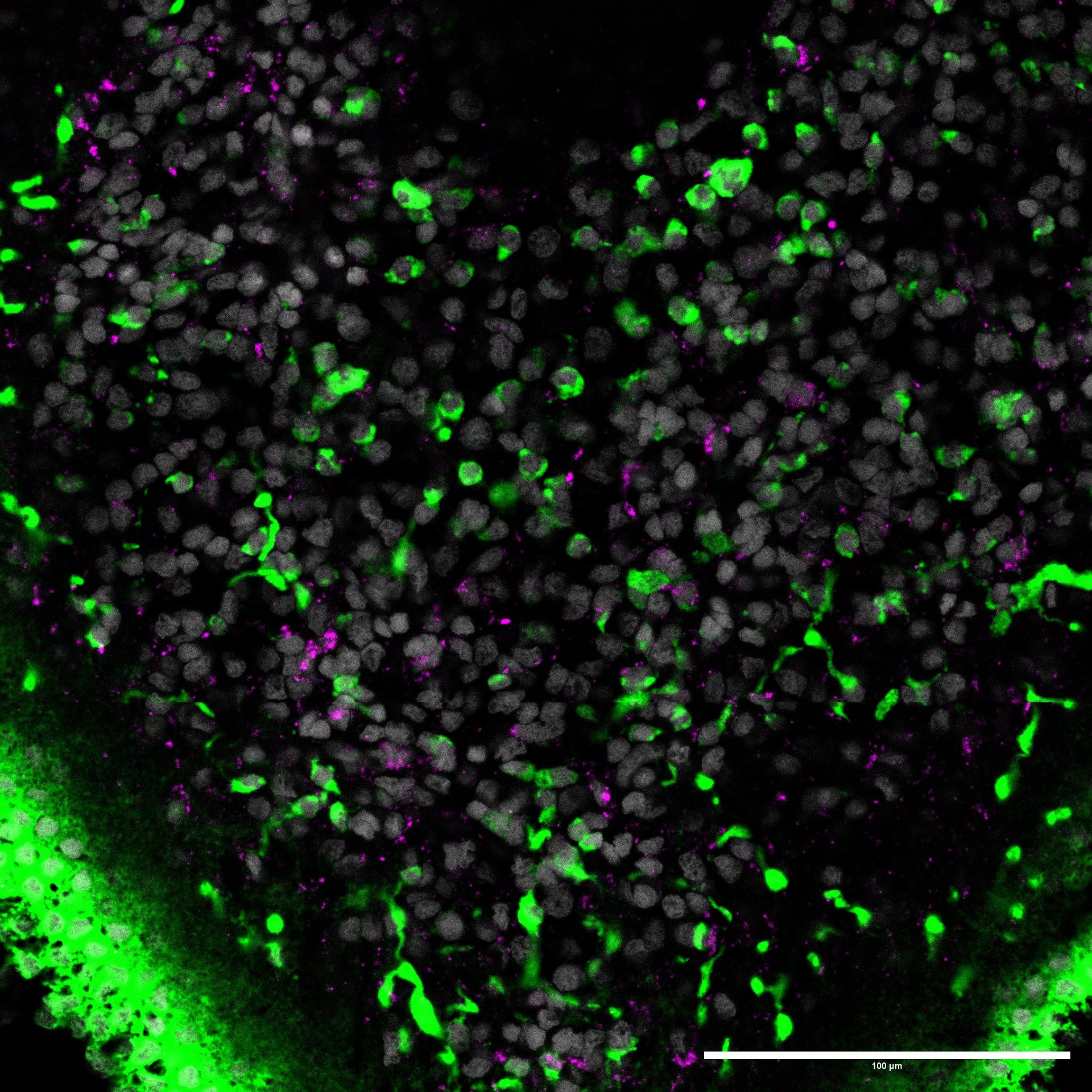

Supplement: Supplementary file 14 — Source data Fig. 7 [file 44318_2025_662_MOESM14_ESM.zip › Figure 7/7G/ID_10_Region_1_Triple_RNAi_Probe_dd234_rhod_SMEDWI1_FITC_DAPI_20x_z3.jpg]

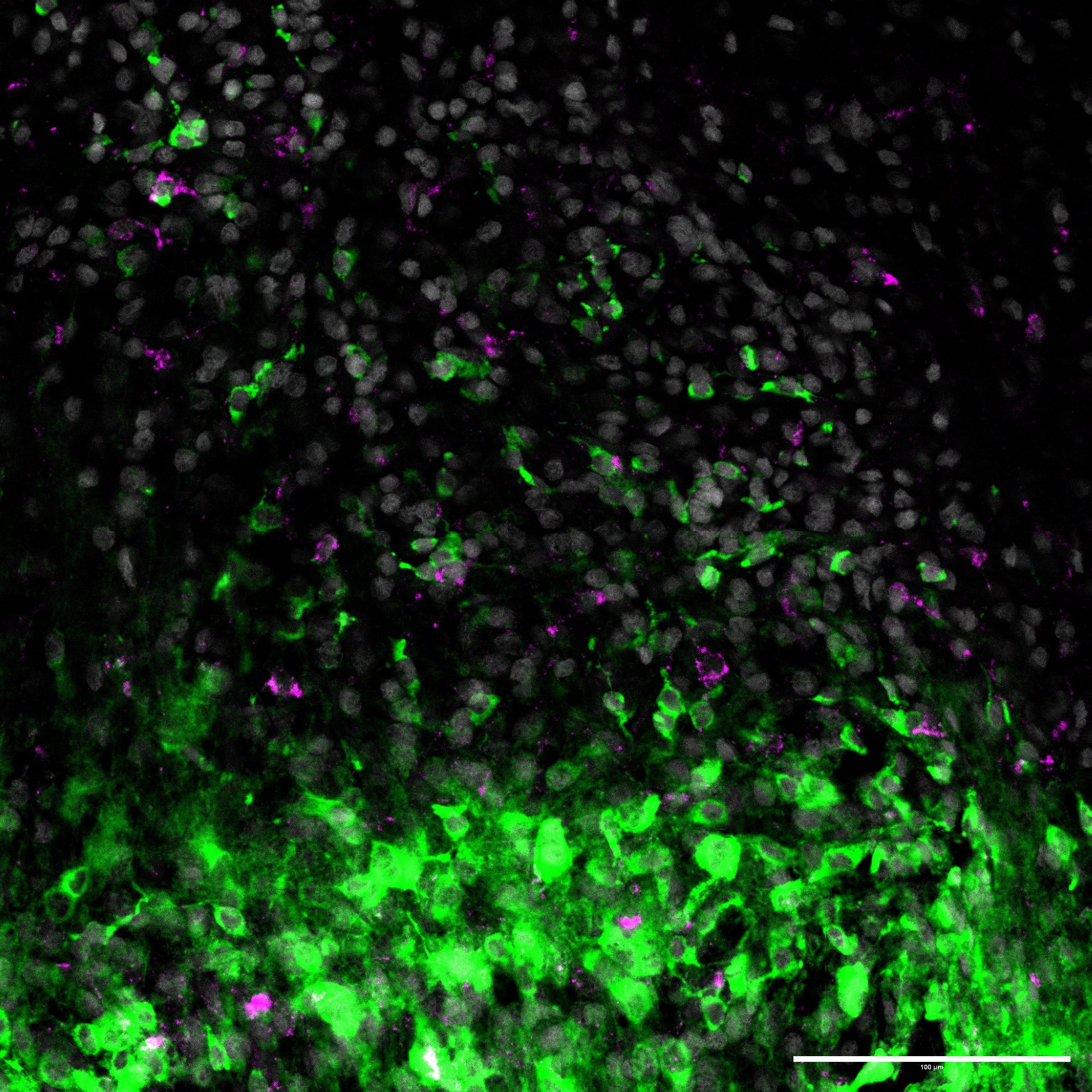

Supplement: Supplementary file 14 — Source data Fig. 7 [file 44318_2025_662_MOESM14_ESM.zip › Figure 7/7G/ID_10_Region_2_Control_RNAi_Probe_dd234_rhod_SMEDWI1_FITC_DAPI_20x_z3.jpg]

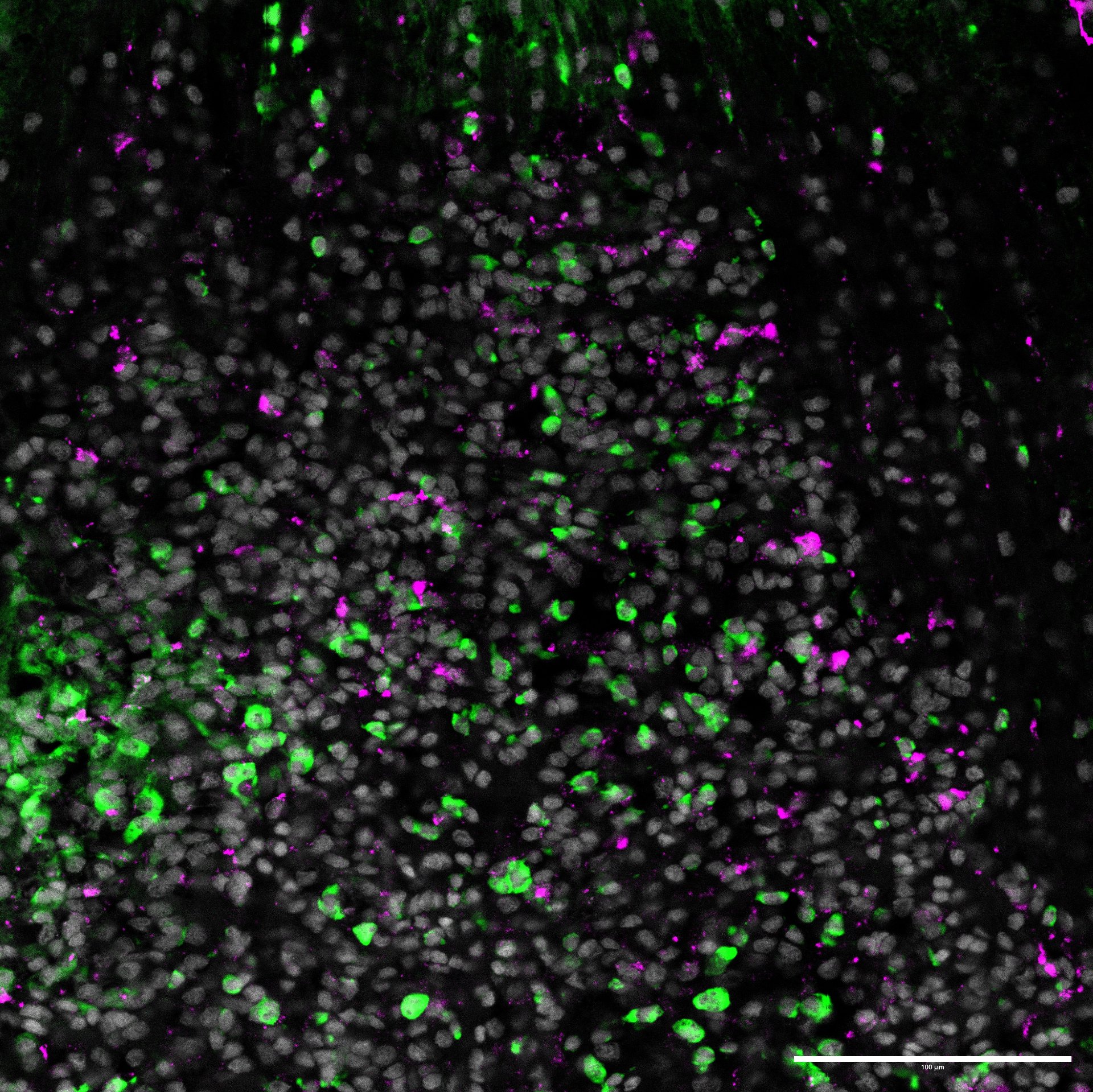

Supplement: Supplementary file 14 — Source data Fig. 7 [file 44318_2025_662_MOESM14_ESM.zip › Figure 7/7G/ID_11_Region_1_Control_RNAi_Probe_dd234_rhod_SMEDWI1_FITC_DAPI_20x_z3.jpg]

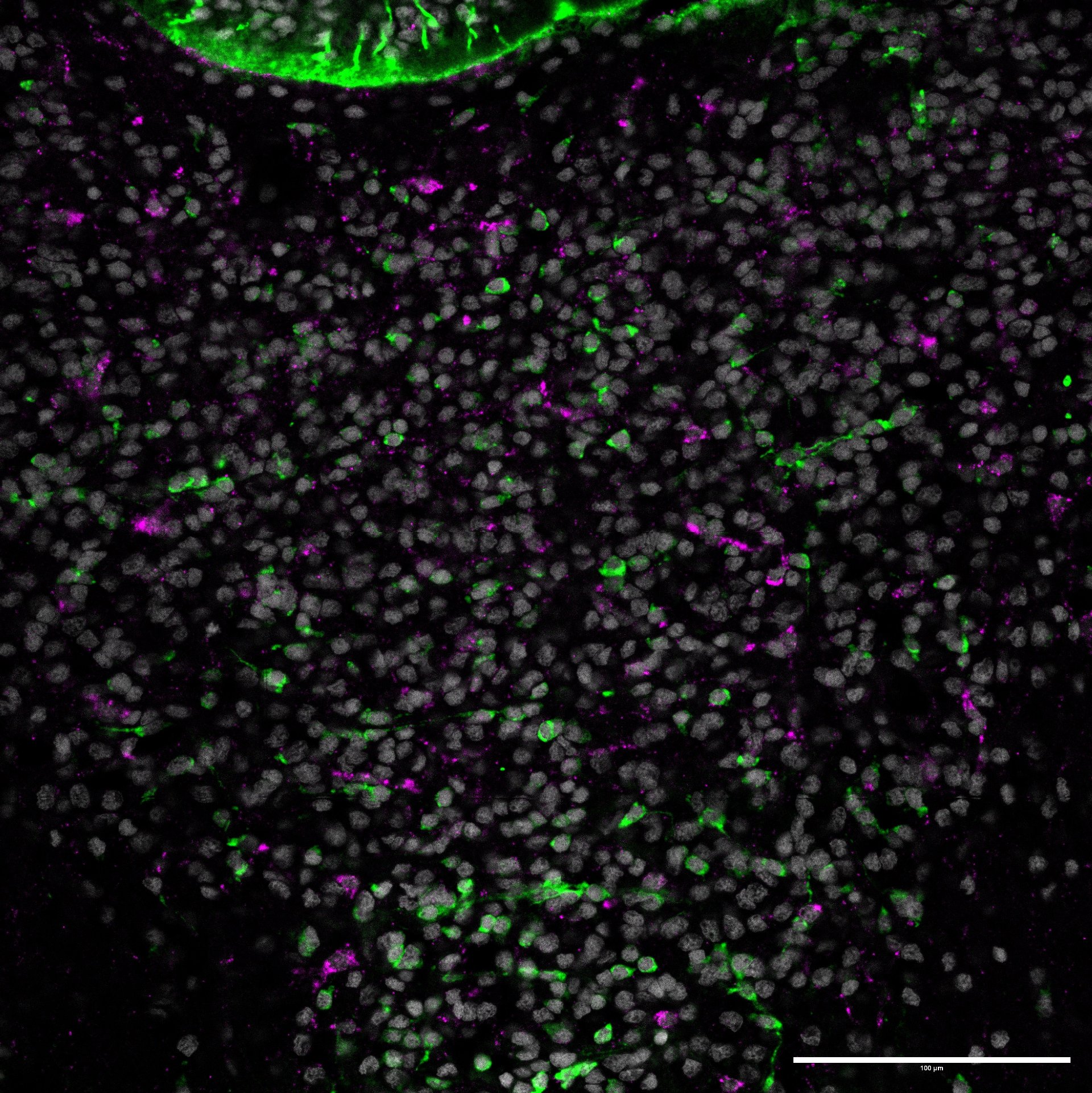

Supplement: Supplementary file 14 — Source data Fig. 7 [file 44318_2025_662_MOESM14_ESM.zip › Figure 7/7G/ID_11_Region_1_Triple_RNAi_Probe_dd234_rhod_SMEDWI1_FITC_DAPI_20x_z3.jpg]

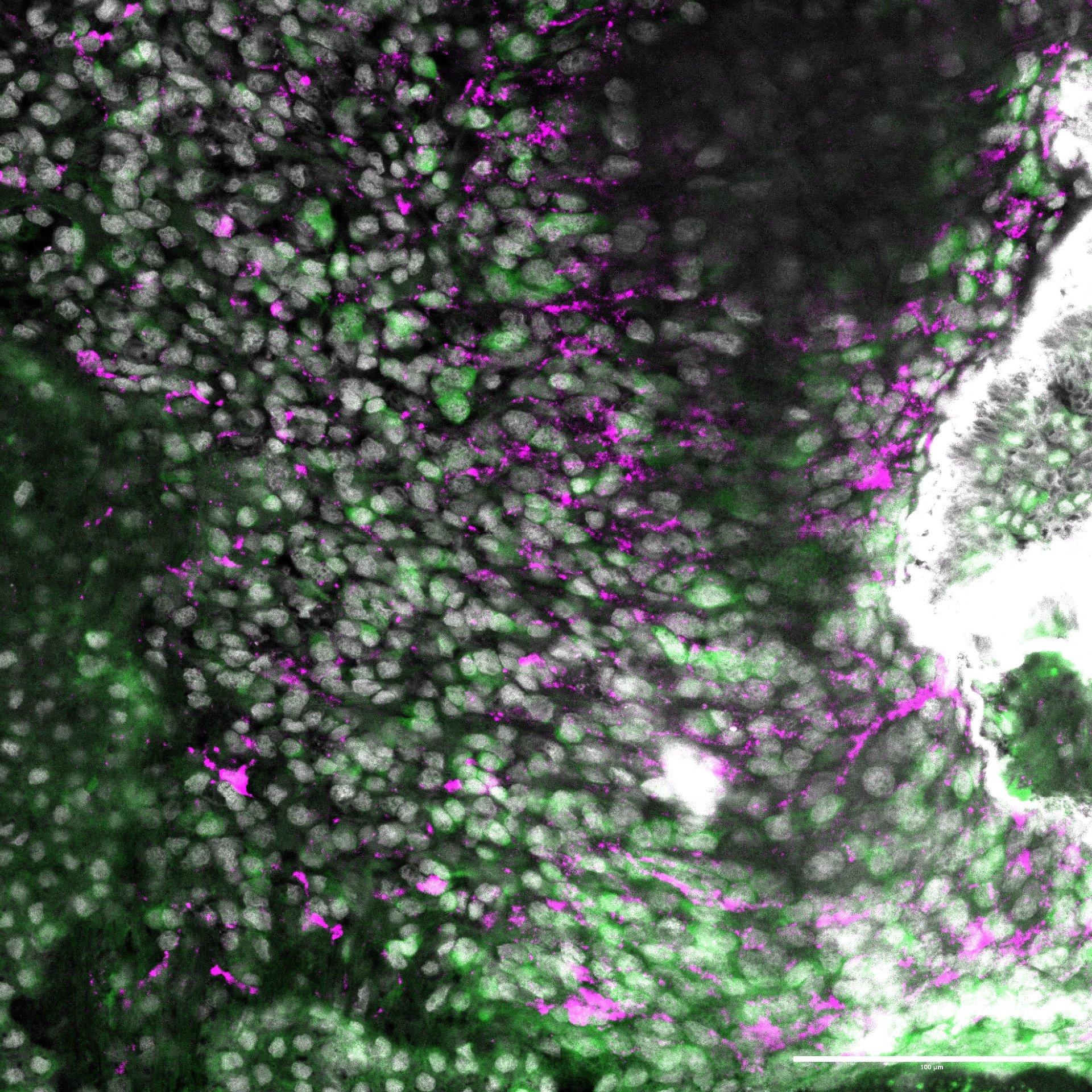

Supplement: Supplementary file 14 — Source data Fig. 7 [file 44318_2025_662_MOESM14_ESM.zip › Figure 7/7G/ID_11_Region_2_Control_RNAi_Probe_dd234_rhod_SMEDWI1_FITC_DAPI_20x_z3.jpg]

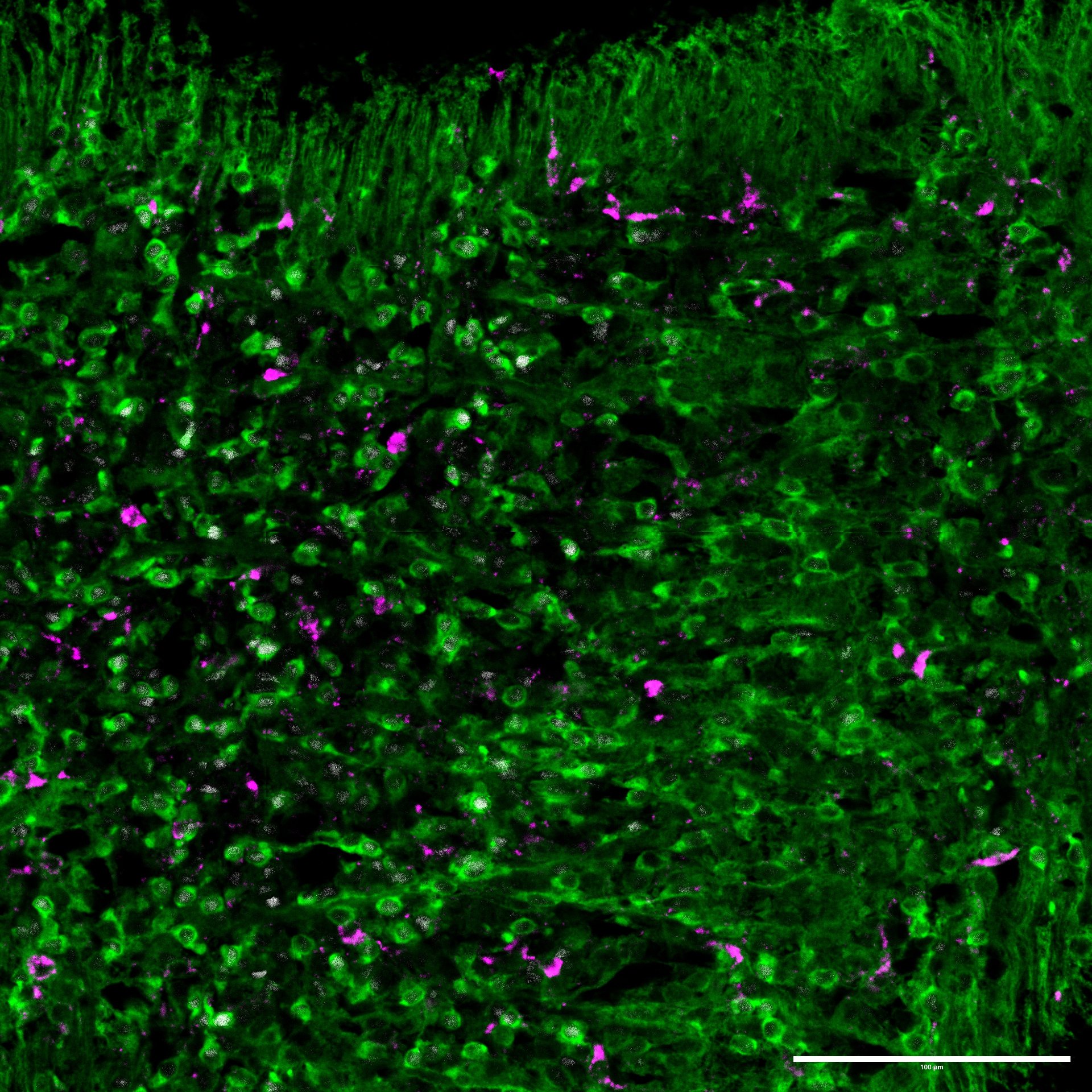

Supplement: Supplementary file 14 — Source data Fig. 7 [file 44318_2025_662_MOESM14_ESM.zip › Figure 7/7G/ID_1_Region_1_Control_RNAi_Probe_dd234_rhod_SMEDWI1_FITC_DAPI_20x_z3.jpg]

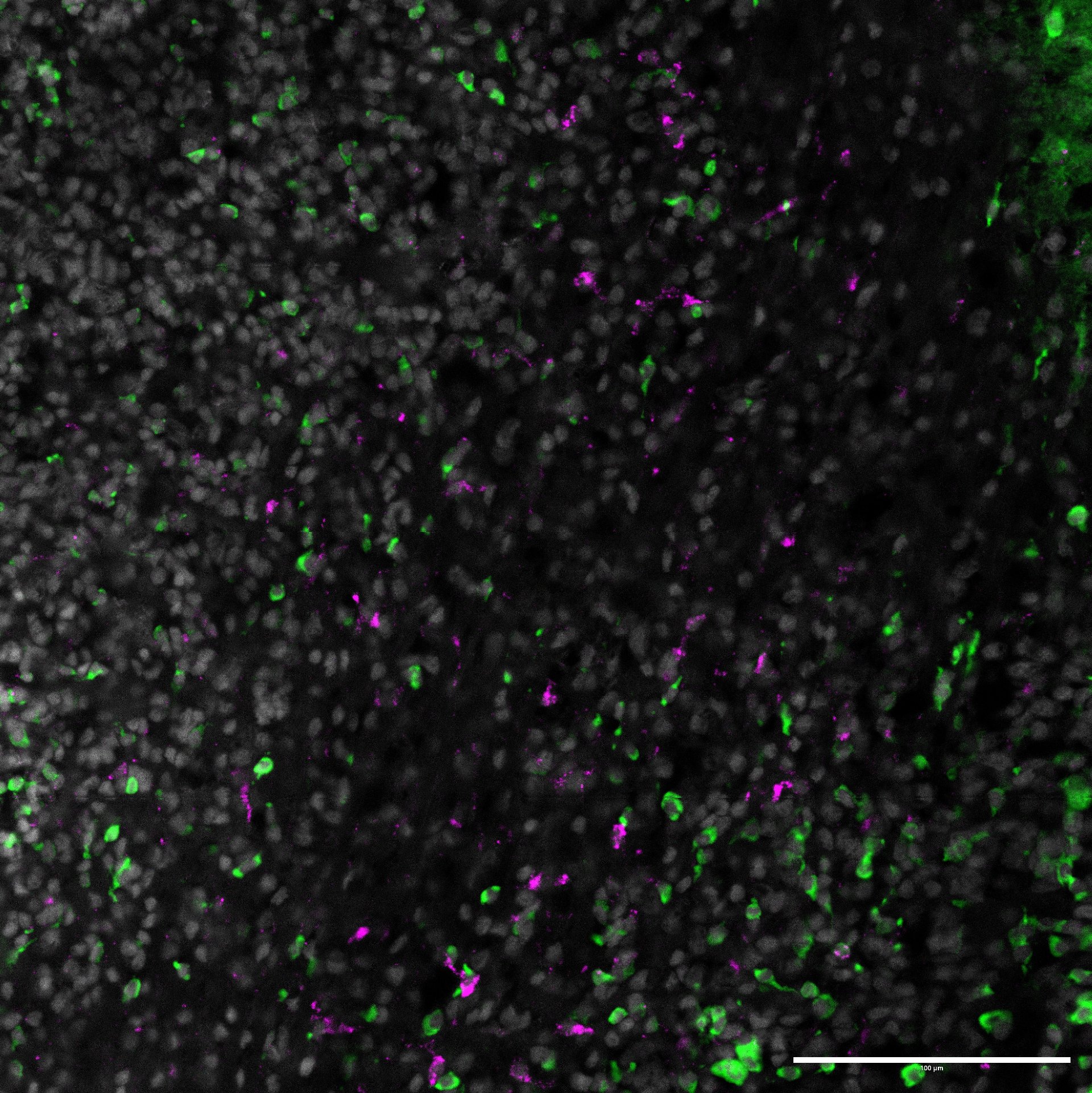

Supplement: Supplementary file 14 — Source data Fig. 7 [file 44318_2025_662_MOESM14_ESM.zip › Figure 7/7G/ID_1_Region_2_Control_RNAi_Probe_dd234_rhod_SMEDWI1_FITC_DAPI_20x_z3.jpg]

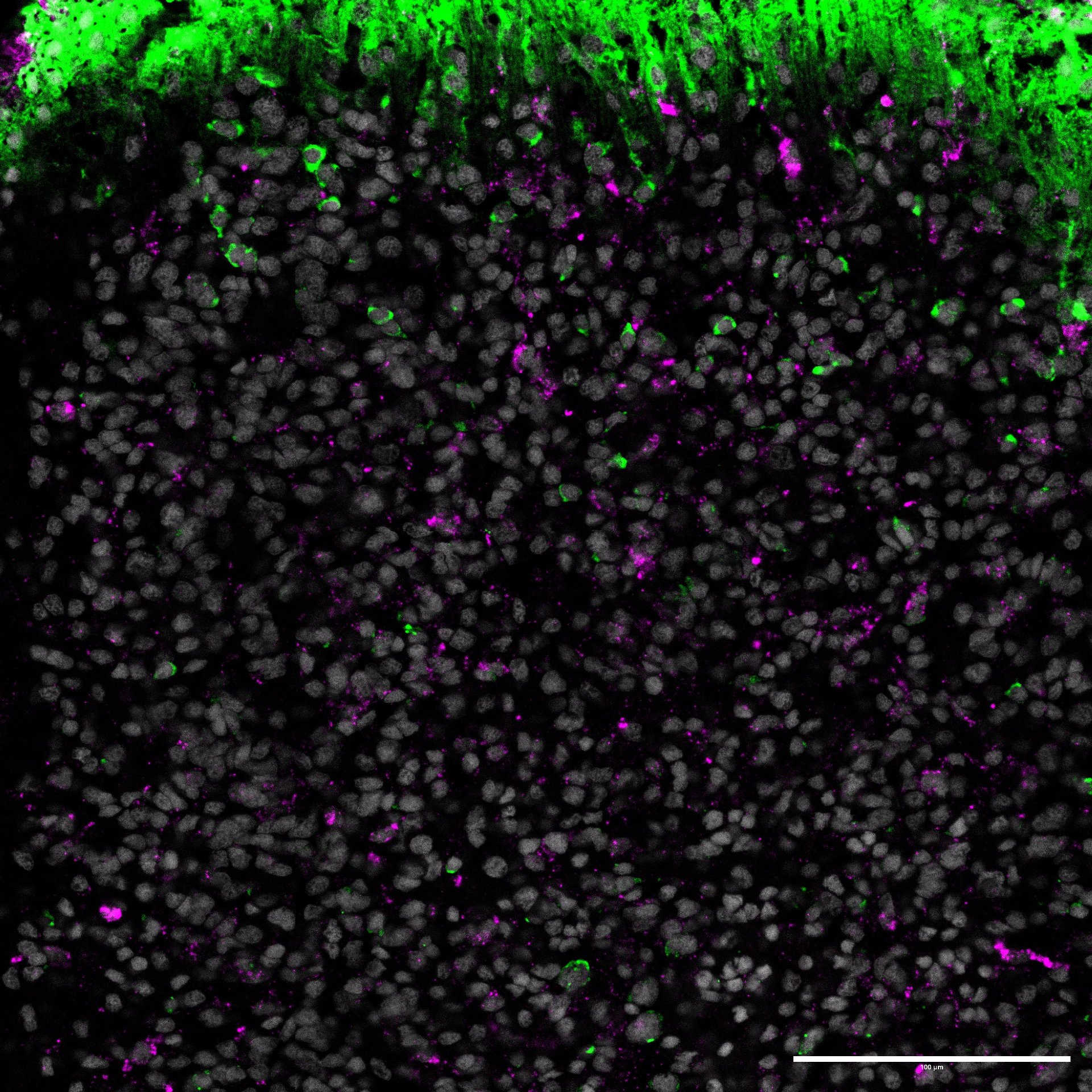

Supplement: Supplementary file 14 — Source data Fig. 7 [file 44318_2025_662_MOESM14_ESM.zip › Figure 7/7G/ID_1_Region_2_Triple_RNAi_Probe_dd234_rhod_SMEDWI1_FITC_DAPI_20x_z3.jpg]

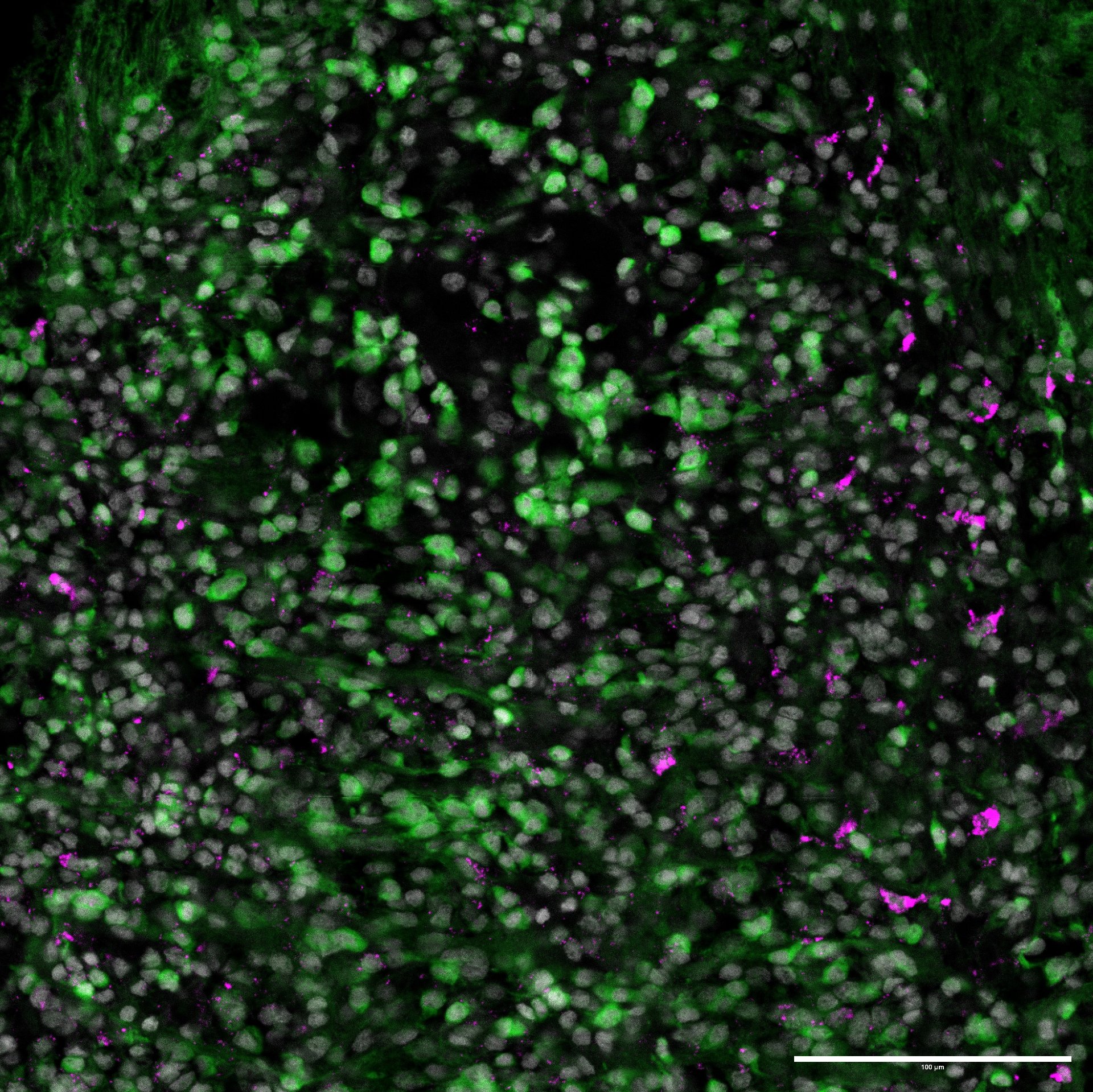

Supplement: Supplementary file 14 — Source data Fig. 7 [file 44318_2025_662_MOESM14_ESM.zip › Figure 7/7G/ID_2_Region_1_Control_RNAi_Probe_dd234_rhod_SMEDWI1_FITC_DAPI_20x_z3.jpg]

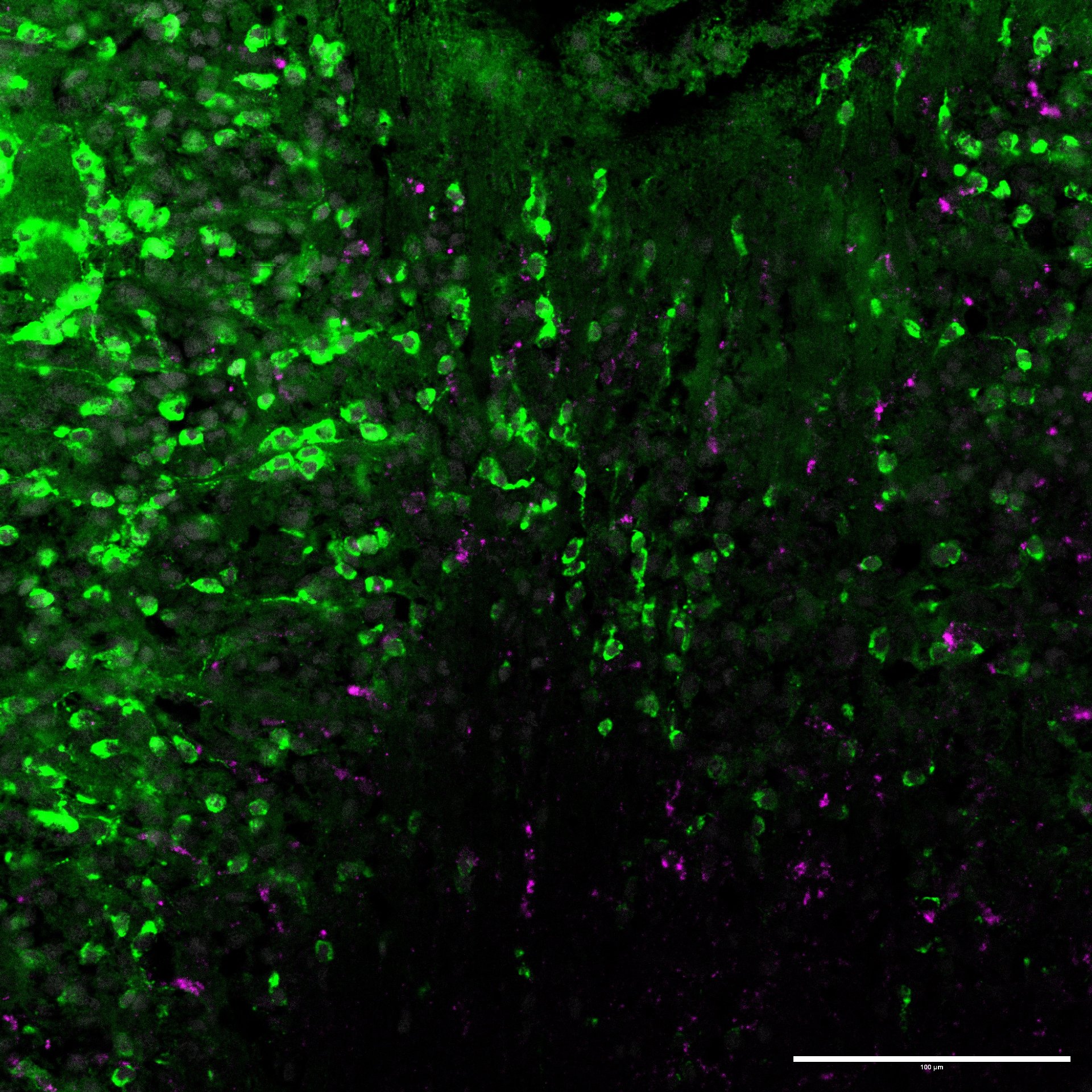

Supplement: Supplementary file 14 — Source data Fig. 7 [file 44318_2025_662_MOESM14_ESM.zip › Figure 7/7G/ID_2_Region_1_Triple_RNAi_Probe_dd234_rhod_SMEDWI1_FITC_DAPI_20x_z3.jpg]

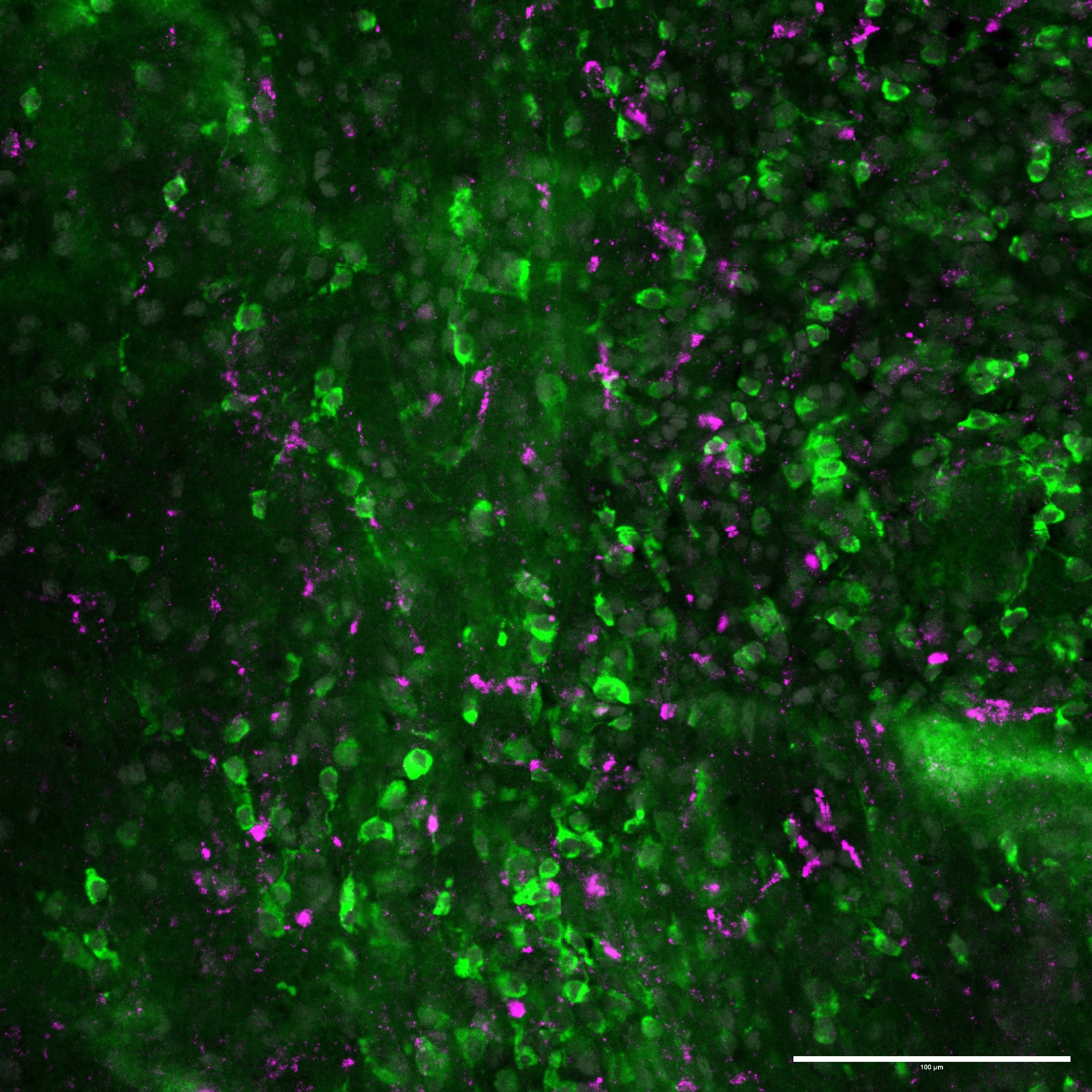

Supplement: Supplementary file 14 — Source data Fig. 7 [file 44318_2025_662_MOESM14_ESM.zip › Figure 7/7G/ID_2_Region_2_Triple_RNAi_Probe_dd234_rhod_SMEDWI1_FITC_DAPI_20x_z3.jpg]

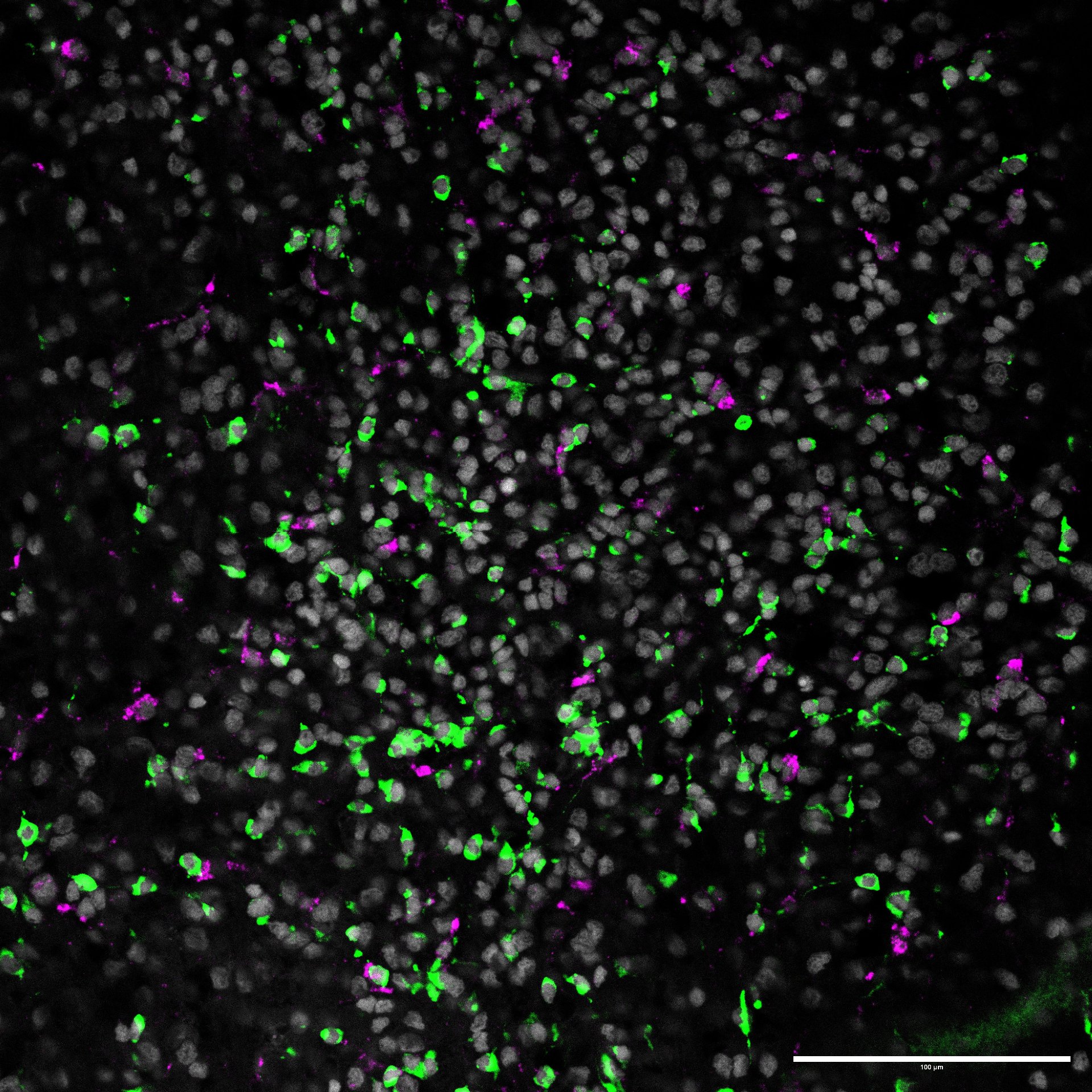

Supplement: Supplementary file 14 — Source data Fig. 7 [file 44318_2025_662_MOESM14_ESM.zip › Figure 7/7G/ID_3_Region_1_Control_RNAi_Probe_dd234_rhod_SMEDWI1_FITC_DAPI_20x_z3.jpg]

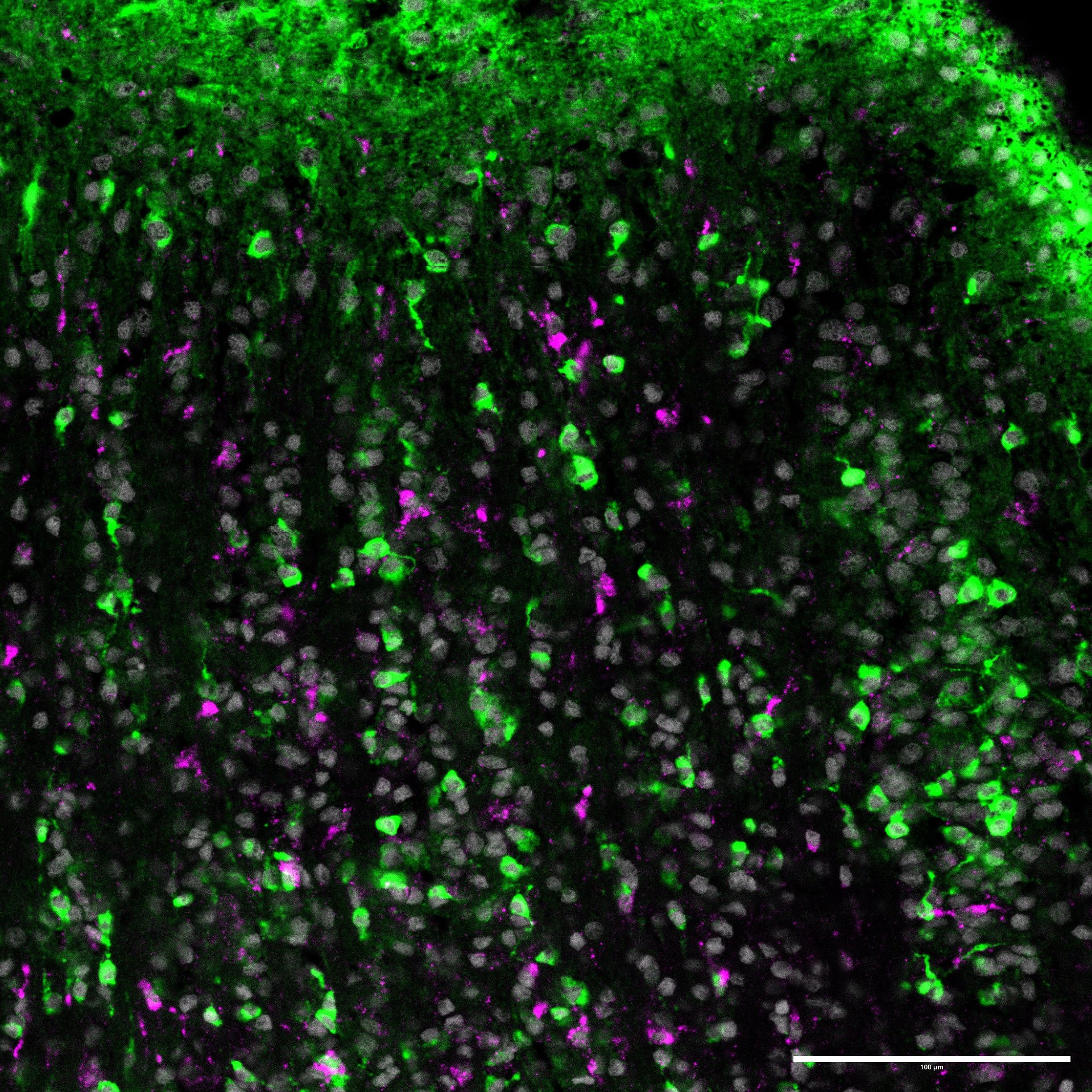

Supplement: Supplementary file 14 — Source data Fig. 7 [file 44318_2025_662_MOESM14_ESM.zip › Figure 7/7G/ID_3_Region_1_Triple_RNAi_Probe_dd234_rhod_SMEDWI1_FITC_DAPI_20x_z3.jpg]

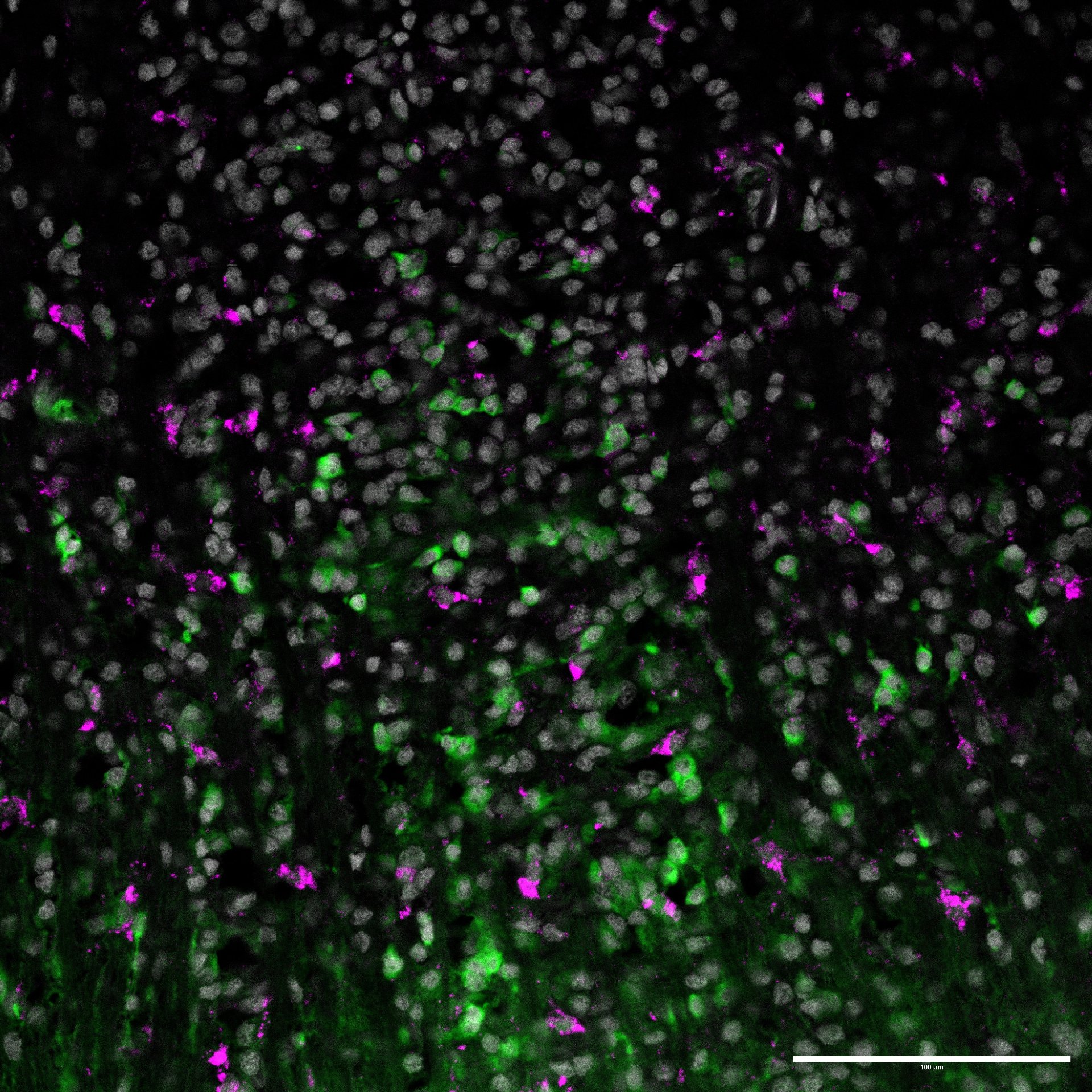

Supplement: Supplementary file 14 — Source data Fig. 7 [file 44318_2025_662_MOESM14_ESM.zip › Figure 7/7G/ID_3_Region_2_Control_RNAi_Probe_dd234_rhod_SMEDWI1_FITC_DAPI_20x_z3.jpg]

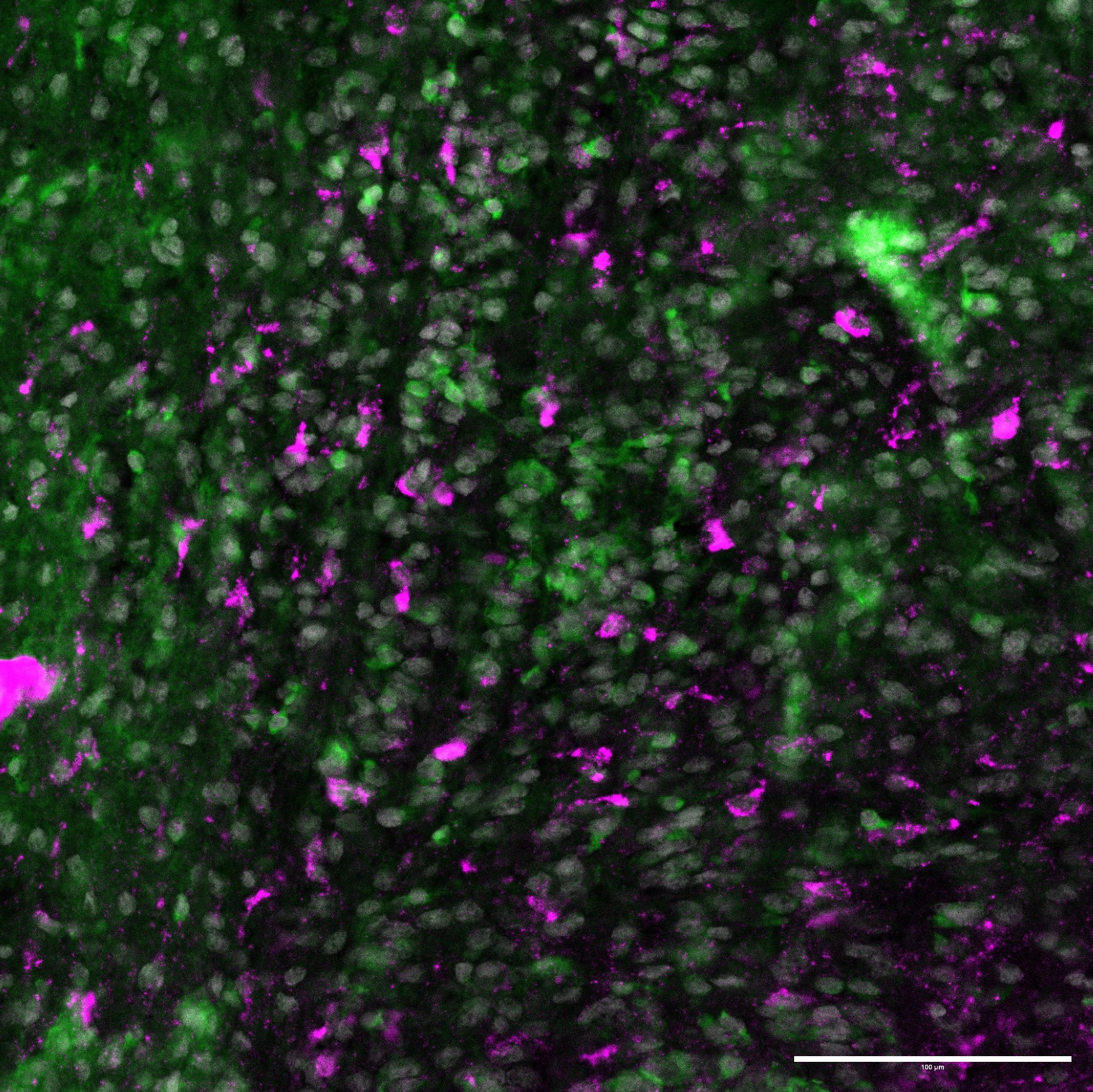

Supplement: Supplementary file 14 — Source data Fig. 7 [file 44318_2025_662_MOESM14_ESM.zip › Figure 7/7G/ID_3_Region_2_Triple_RNAi_Probe_dd234_rhod_SMEDWI1_FITC_DAPI_20x_z3.jpg]

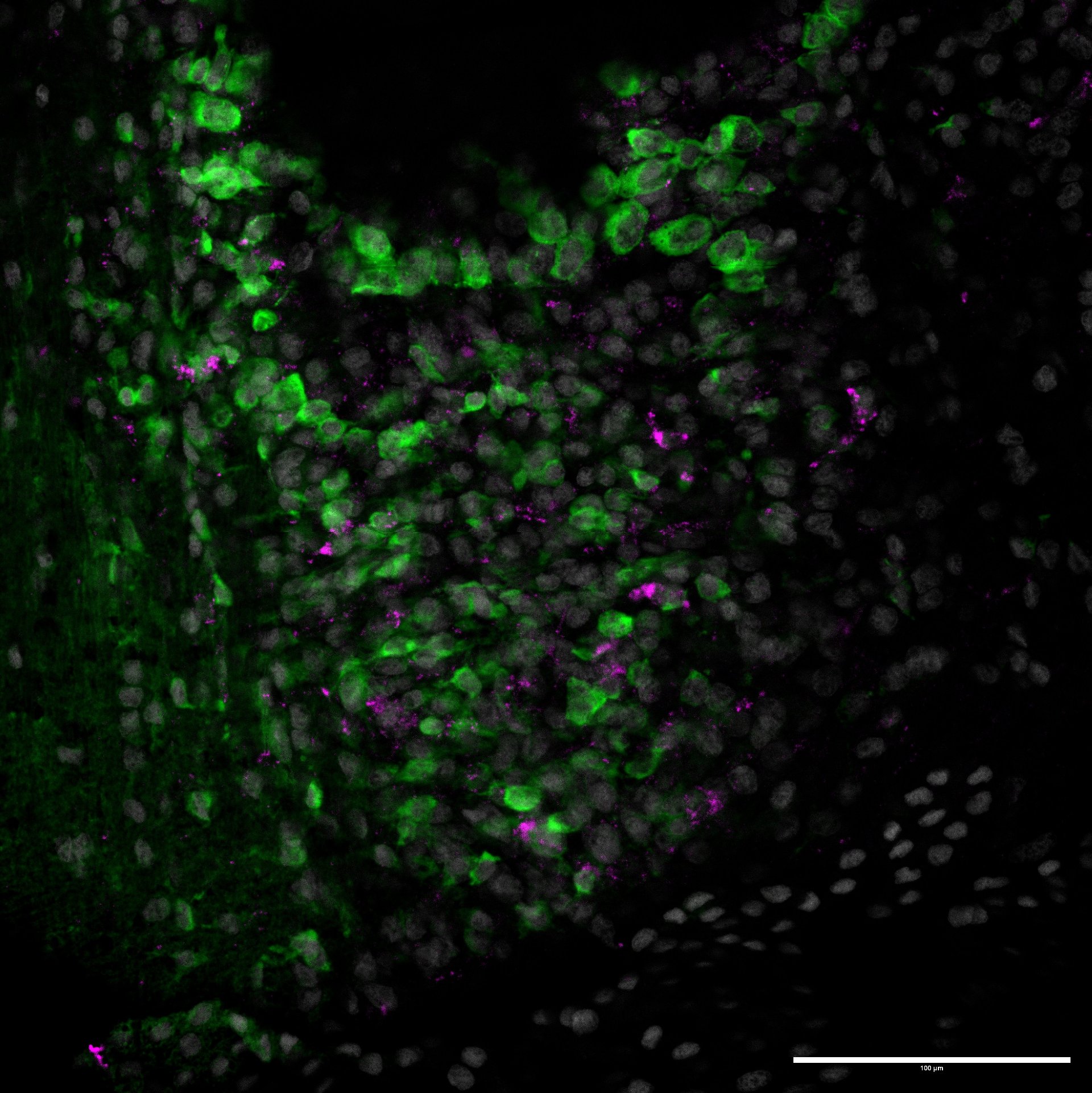

Supplement: Supplementary file 14 — Source data Fig. 7 [file 44318_2025_662_MOESM14_ESM.zip › Figure 7/7G/ID_4_Region_1_Control_RNAi_Probe_dd234_rhod_SMEDWI1_FITC_DAPI_20x_z3.jpg]

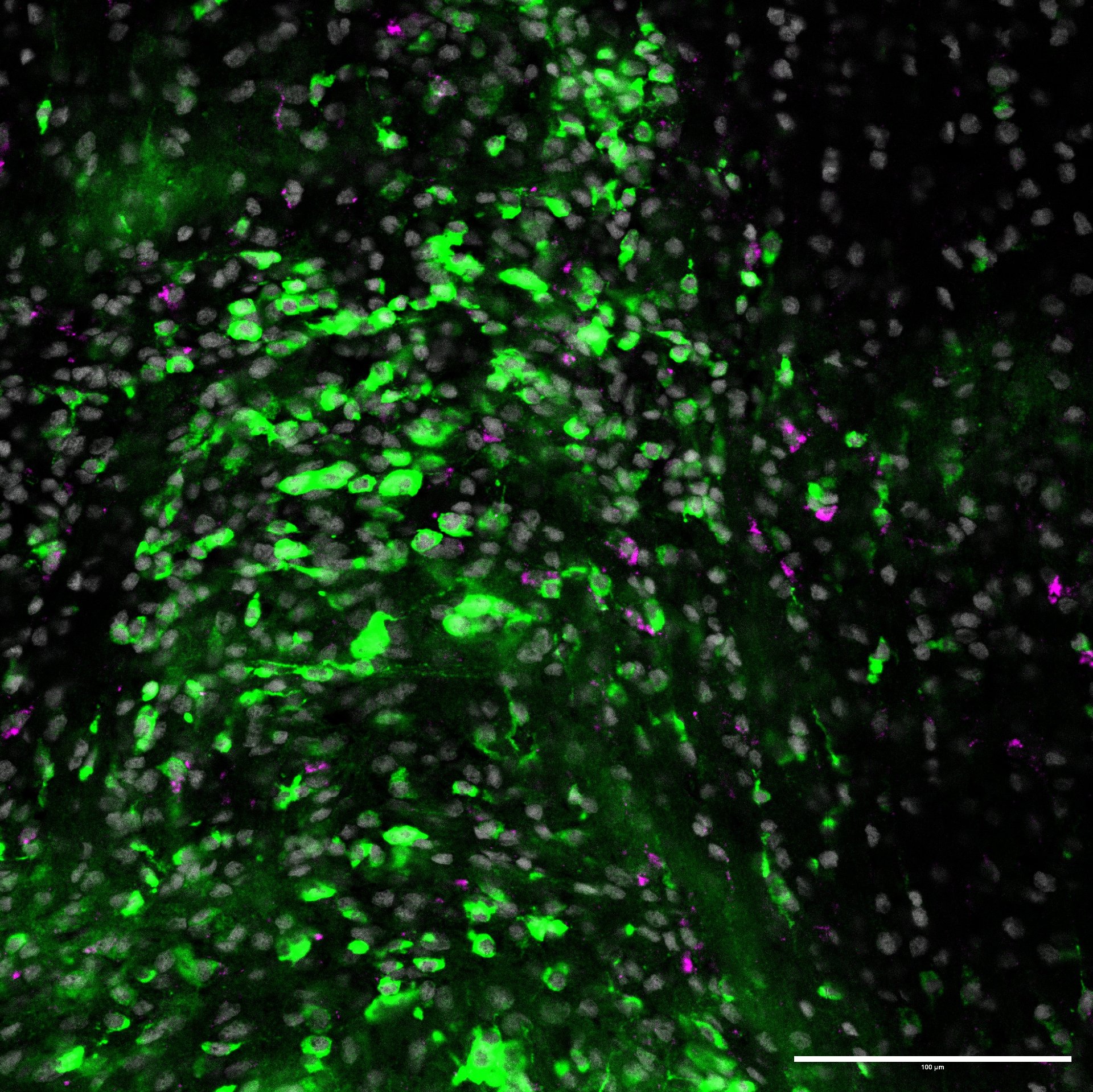

Supplement: Supplementary file 14 — Source data Fig. 7 [file 44318_2025_662_MOESM14_ESM.zip › Figure 7/7G/ID_4_Region_1_Triple_RNAi_Probe_dd234_rhod_SMEDWI1_FITC_DAPI_20x_z3.jpg]

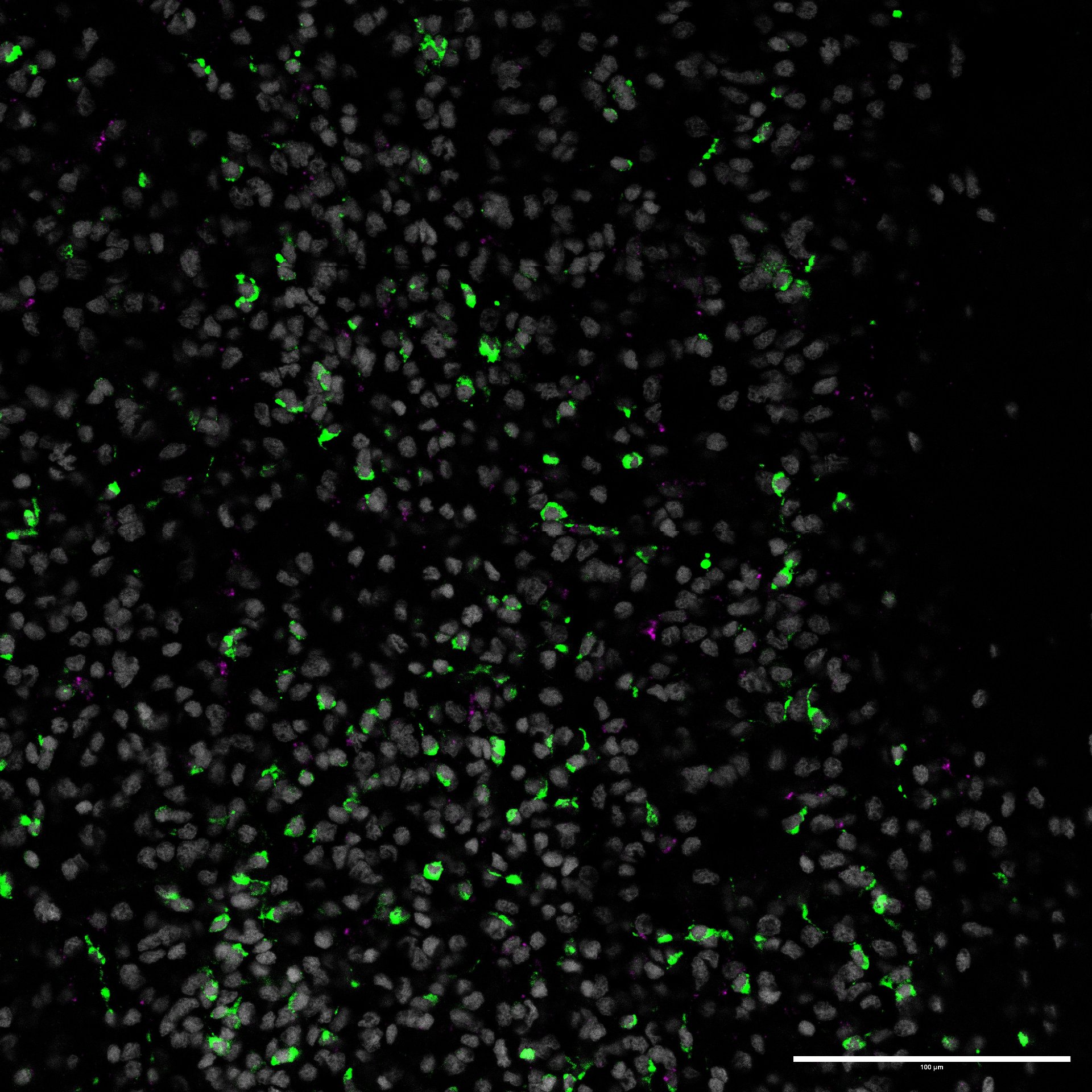

Supplement: Supplementary file 14 — Source data Fig. 7 [file 44318_2025_662_MOESM14_ESM.zip › Figure 7/7G/ID_4_Region_2_Triple_RNAi_Probe_dd234_rhod_SMEDWI1_FITC_DAPI_20x_z3.jpg]

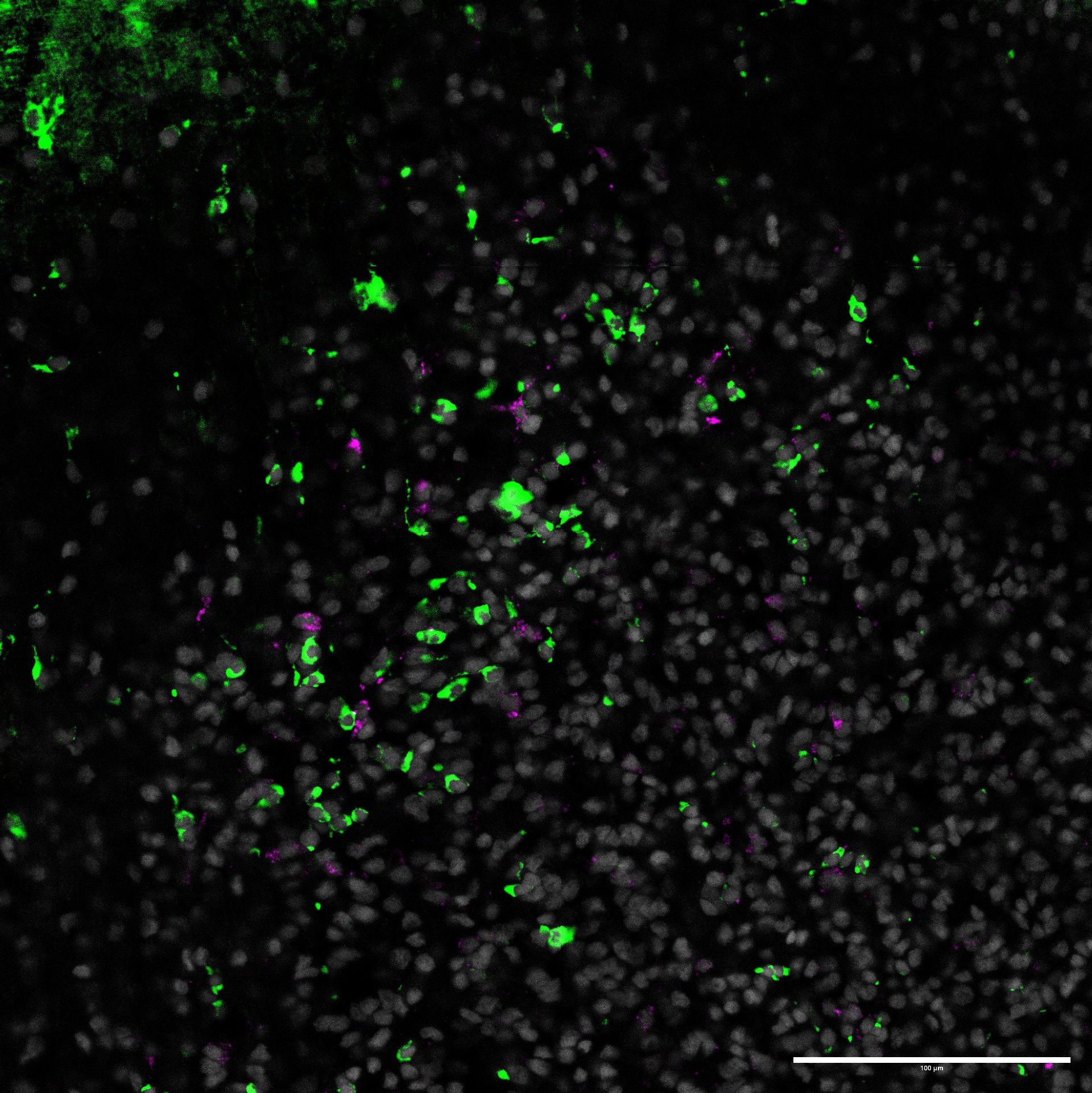

Supplement: Supplementary file 14 — Source data Fig. 7 [file 44318_2025_662_MOESM14_ESM.zip › Figure 7/7G/ID_5_Region_1_Control_RNAi_Probe_dd234_rhod_SMEDWI1_FITC_DAPI_20x_z3.jpg]

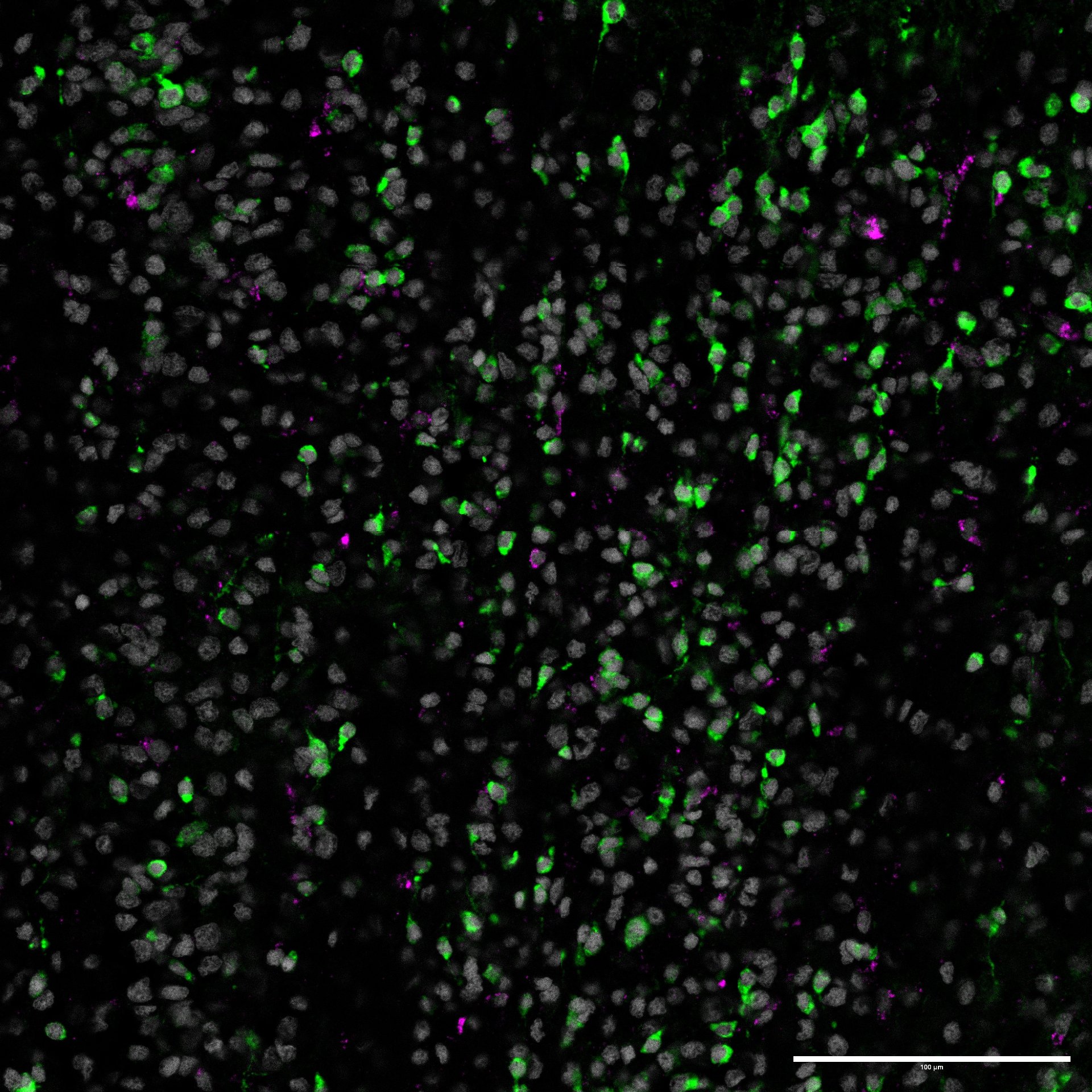

Supplement: Supplementary file 14 — Source data Fig. 7 [file 44318_2025_662_MOESM14_ESM.zip › Figure 7/7G/ID_5_Region_1_Triple_RNAi_Probe_dd234_rhod_SMEDWI1_FITC_DAPI_20x_z3.jpg]

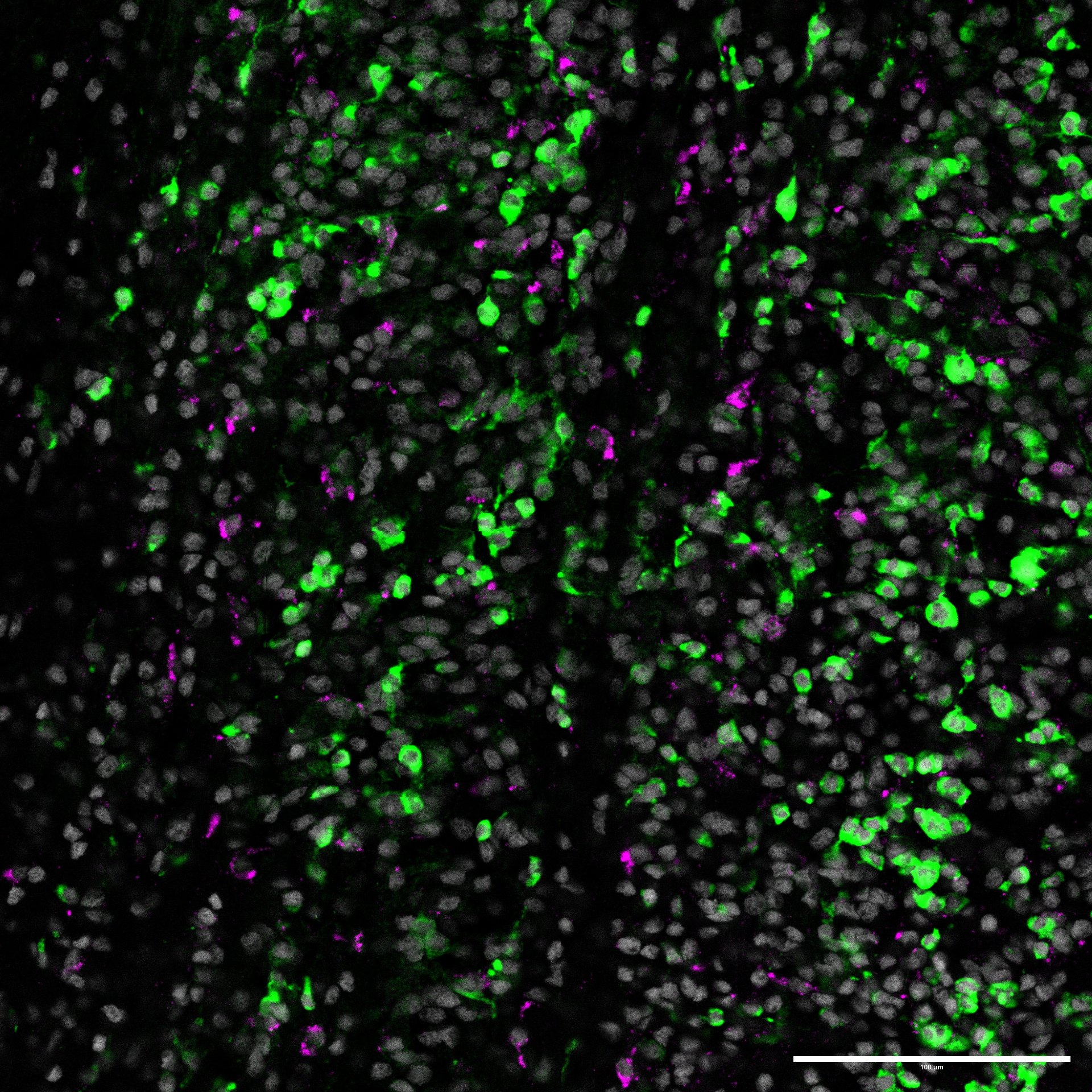

Supplement: Supplementary file 14 — Source data Fig. 7 [file 44318_2025_662_MOESM14_ESM.zip › Figure 7/7G/ID_5_Region_2_Triple_RNAi_Probe_dd234_rhod_SMEDWI1_FITC_DAPI_20x_z3.jpg]

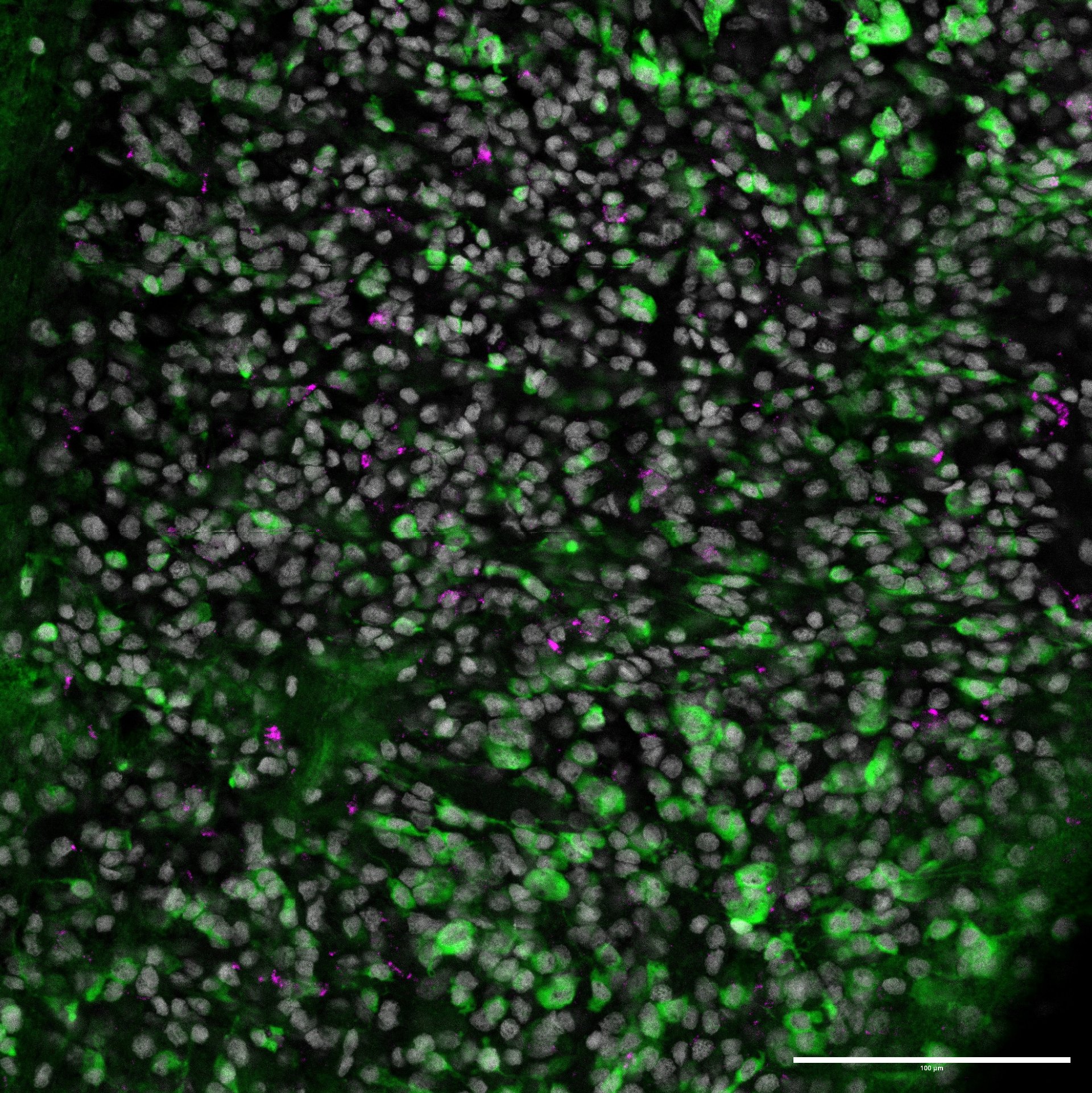

Supplement: Supplementary file 14 — Source data Fig. 7 [file 44318_2025_662_MOESM14_ESM.zip › Figure 7/7G/ID_6_Region_1_Control_RNAi_Probe_dd234_rhod_SMEDWI1_FITC_DAPI_20x_z3.jpg]

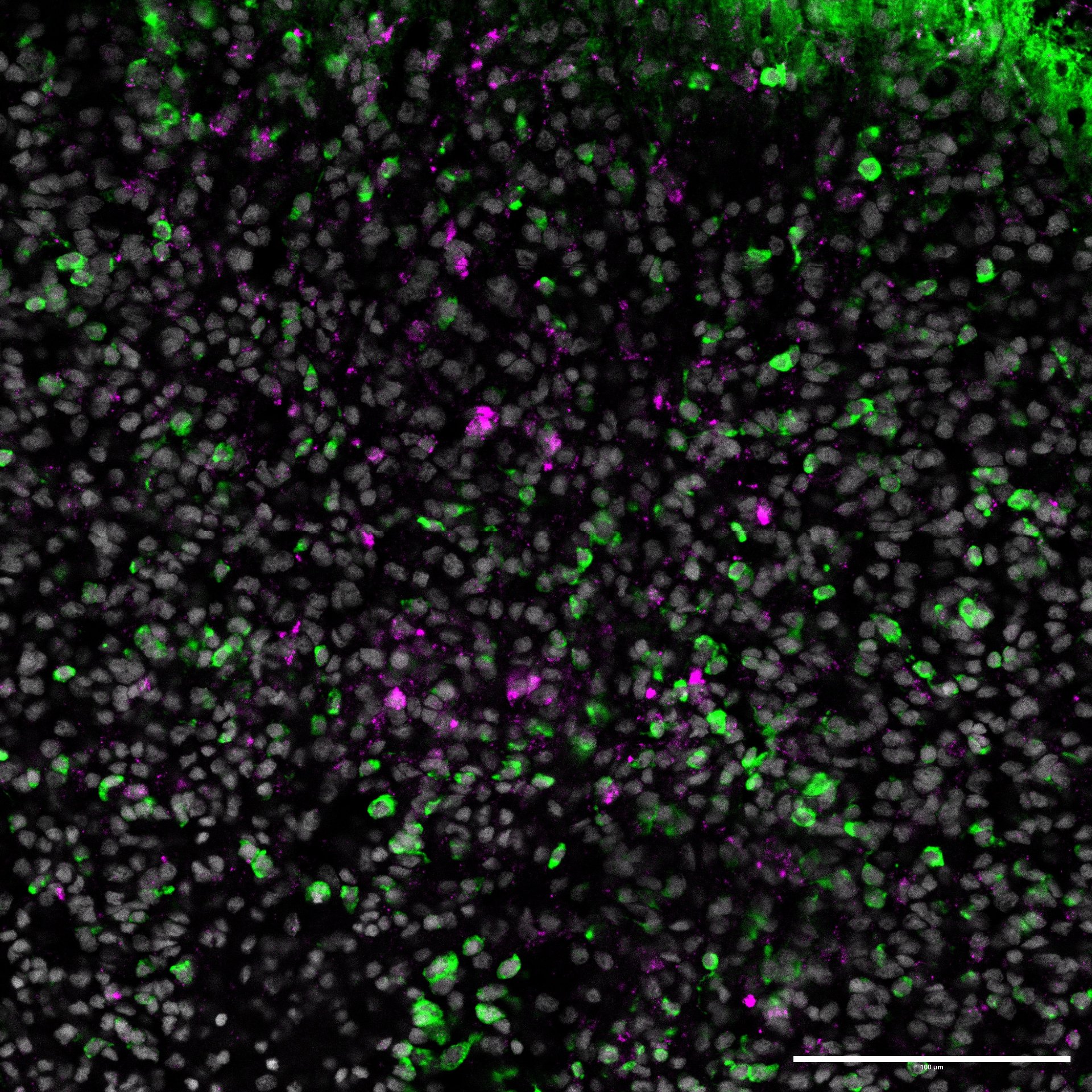

Supplement: Supplementary file 14 — Source data Fig. 7 [file 44318_2025_662_MOESM14_ESM.zip › Figure 7/7G/ID_6_Region_1_Triple_RNAi_Probe_dd234_rhod_SMEDWI1_FITC_DAPI_20x_z3.jpg]

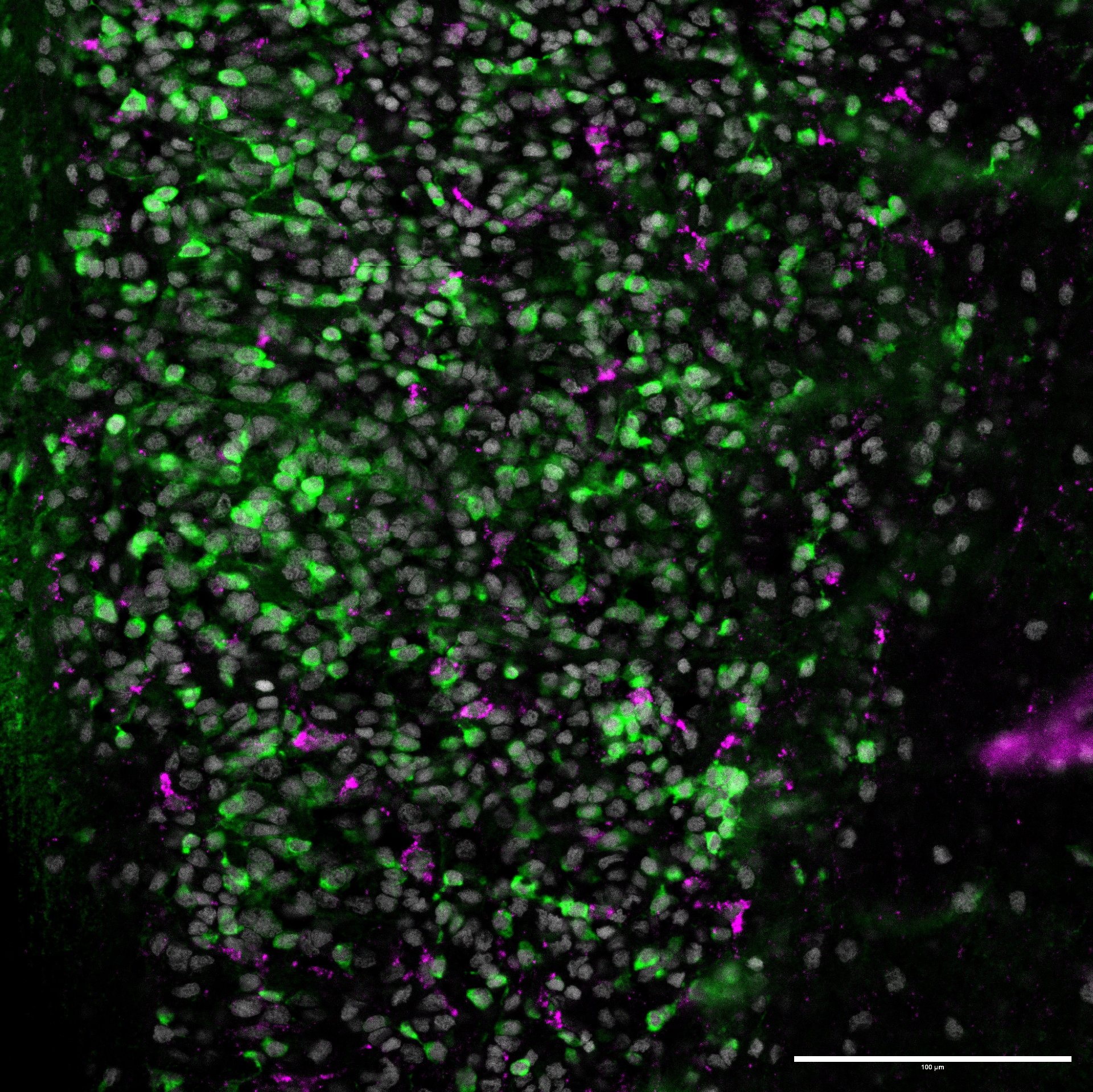

Supplement: Supplementary file 14 — Source data Fig. 7 [file 44318_2025_662_MOESM14_ESM.zip › Figure 7/7G/ID_6_Region_2_Triple_RNAi_Probe_dd234_rhod_SMEDWI1_FITC_DAPI_20x_z3.jpg]

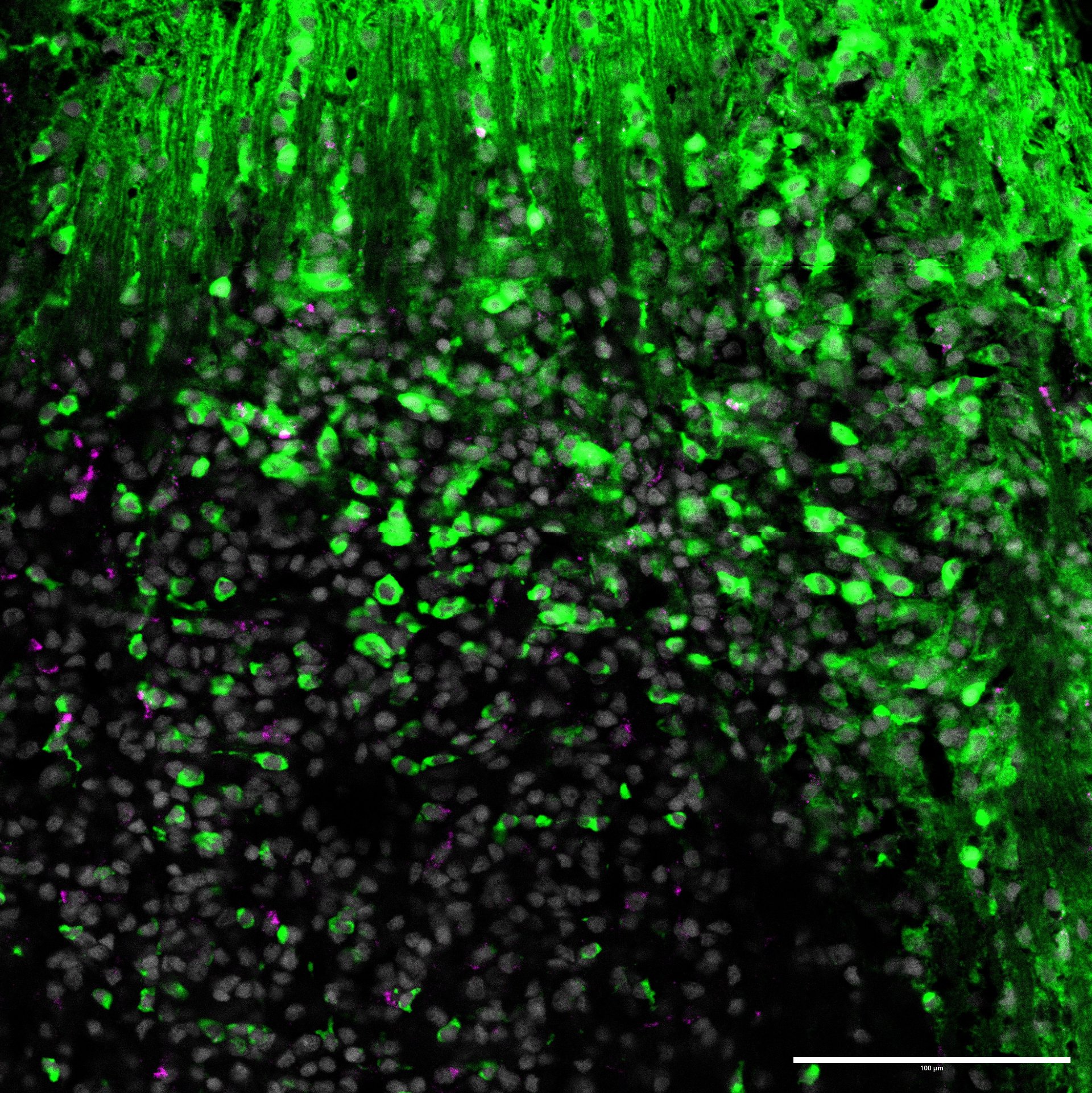

Supplement: Supplementary file 14 — Source data Fig. 7 [file 44318_2025_662_MOESM14_ESM.zip › Figure 7/7G/ID_7_Region_1_Control_RNAi_Probe_dd234_rhod_SMEDWI1_FITC_DAPI_20x_z3.jpg]

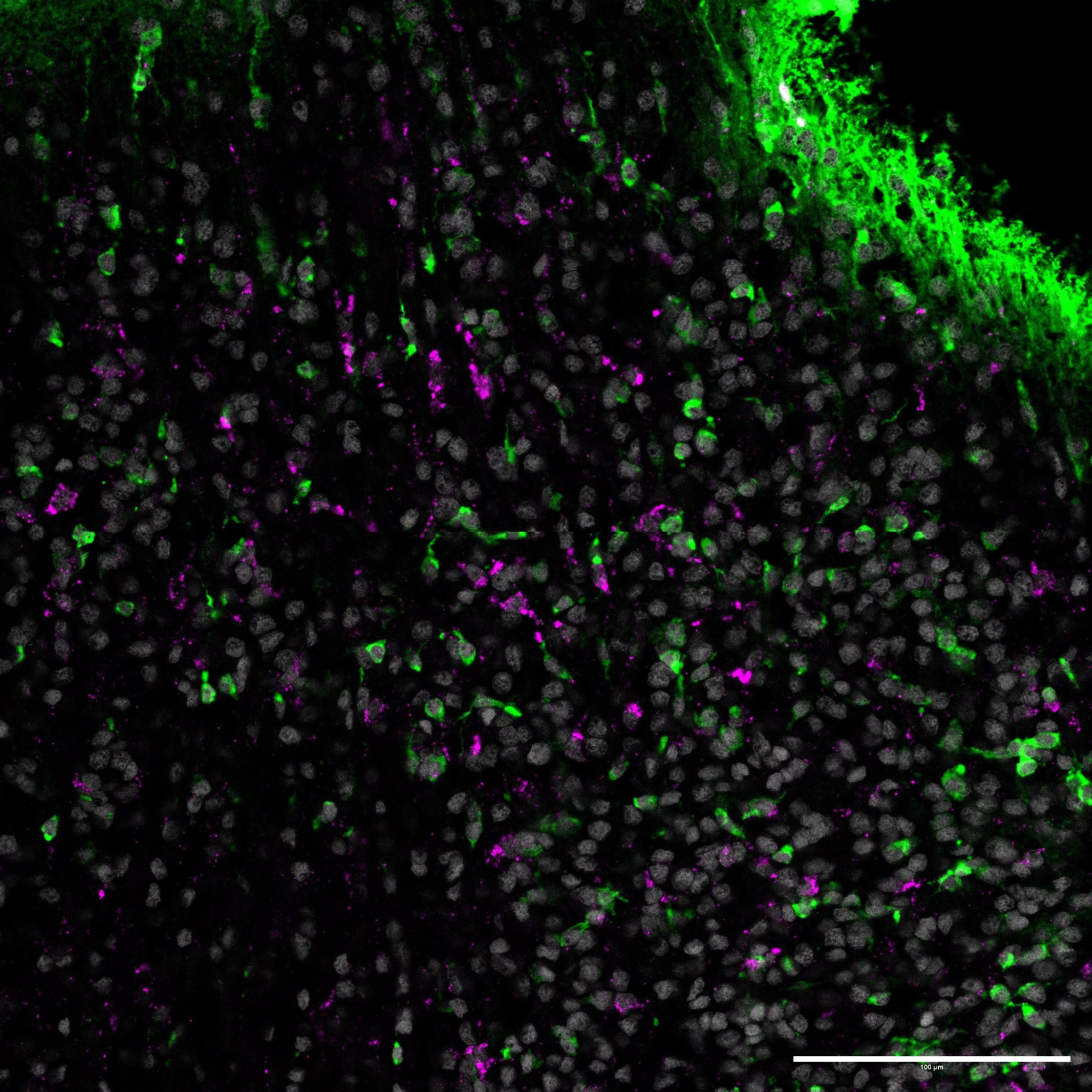

Supplement: Supplementary file 14 — Source data Fig. 7 [file 44318_2025_662_MOESM14_ESM.zip › Figure 7/7G/ID_7_Region_1_Triple_RNAi_Probe_dd234_rhod_SMEDWI1_FITC_DAPI_20x_z3.jpg]

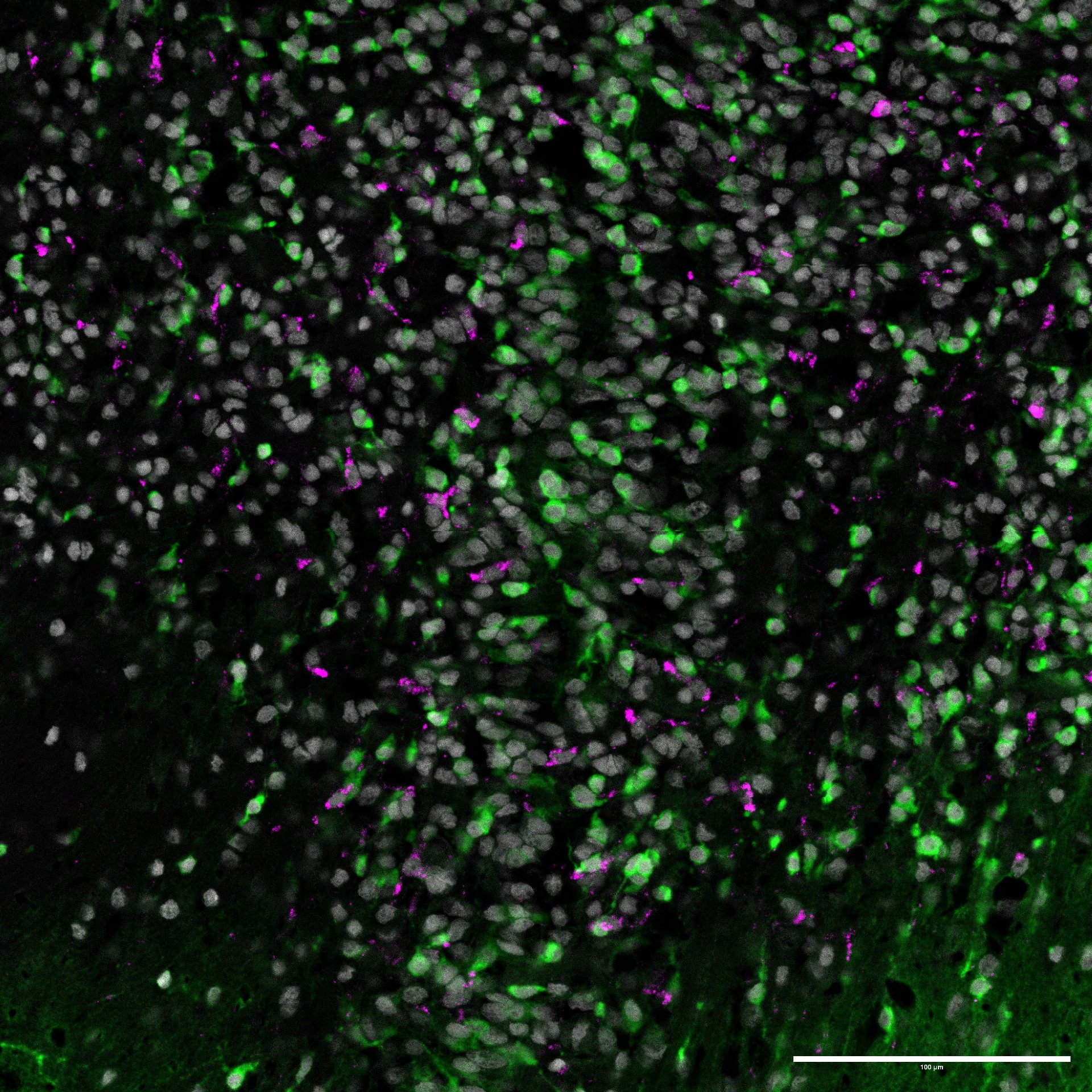

Supplement: Supplementary file 14 — Source data Fig. 7 [file 44318_2025_662_MOESM14_ESM.zip › Figure 7/7G/ID_7_Region_2_Control_RNAi_Probe_dd234_rhod_SMEDWI1_FITC_DAPI_20x_z3.jpg]

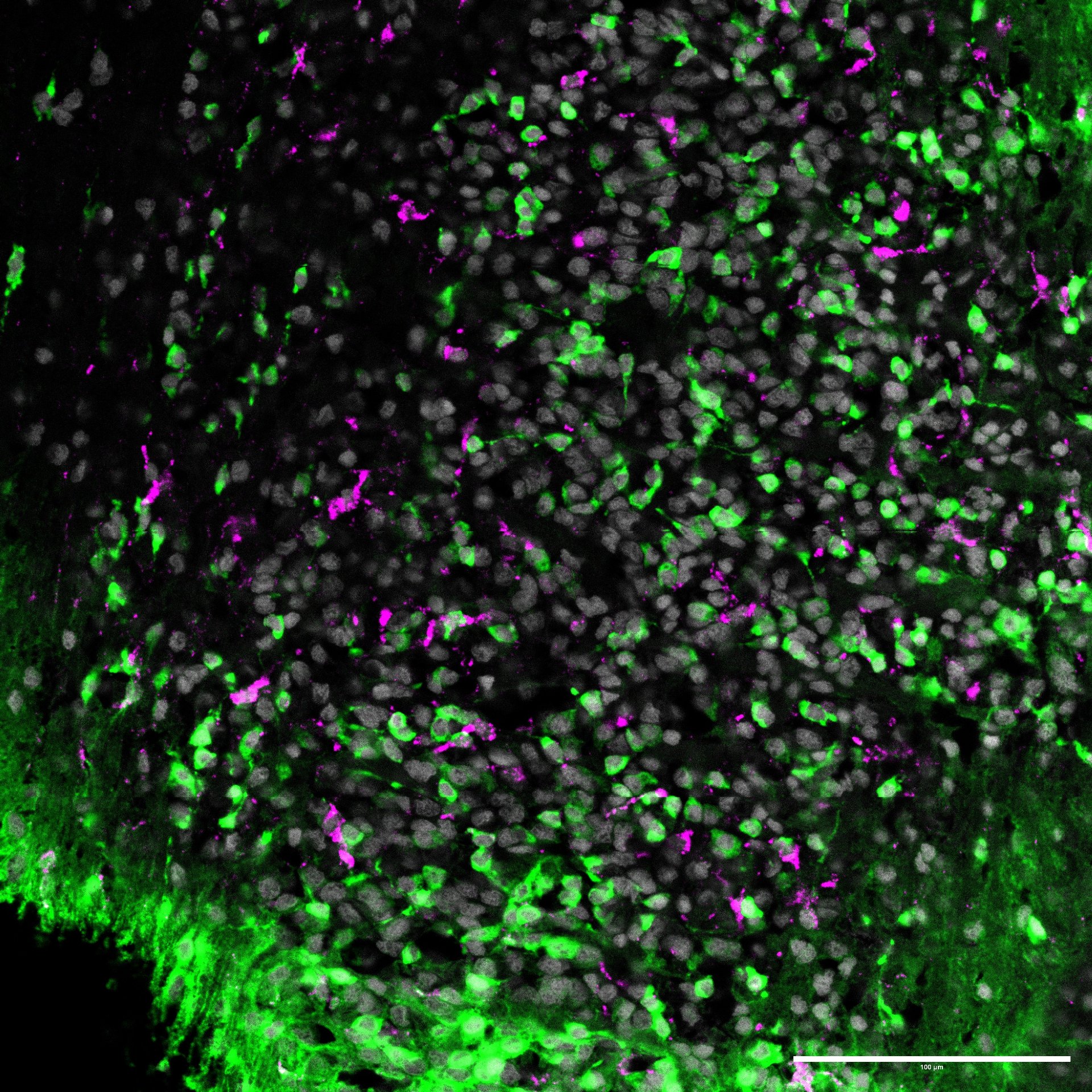

Supplement: Supplementary file 14 — Source data Fig. 7 [file 44318_2025_662_MOESM14_ESM.zip › Figure 7/7G/ID_8_Region_1_Control_RNAi_Probe_dd234_rhod_SMEDWI1_FITC_DAPI_20x_z3.jpg]

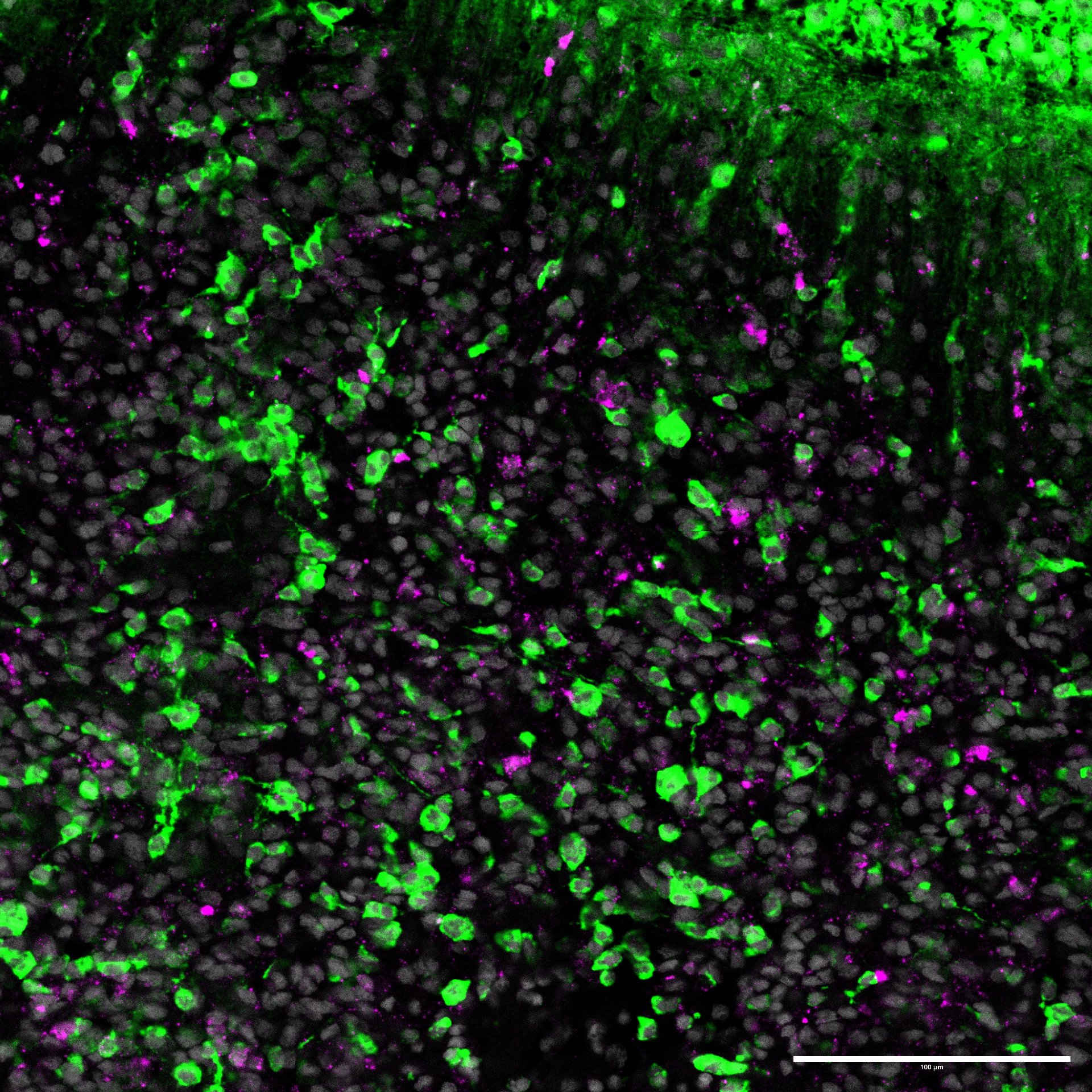

Supplement: Supplementary file 14 — Source data Fig. 7 [file 44318_2025_662_MOESM14_ESM.zip › Figure 7/7G/ID_8_Region_1_Triple_RNAi_Probe_dd234_rhod_SMEDWI1_FITC_DAPI_20x_z3.jpg]

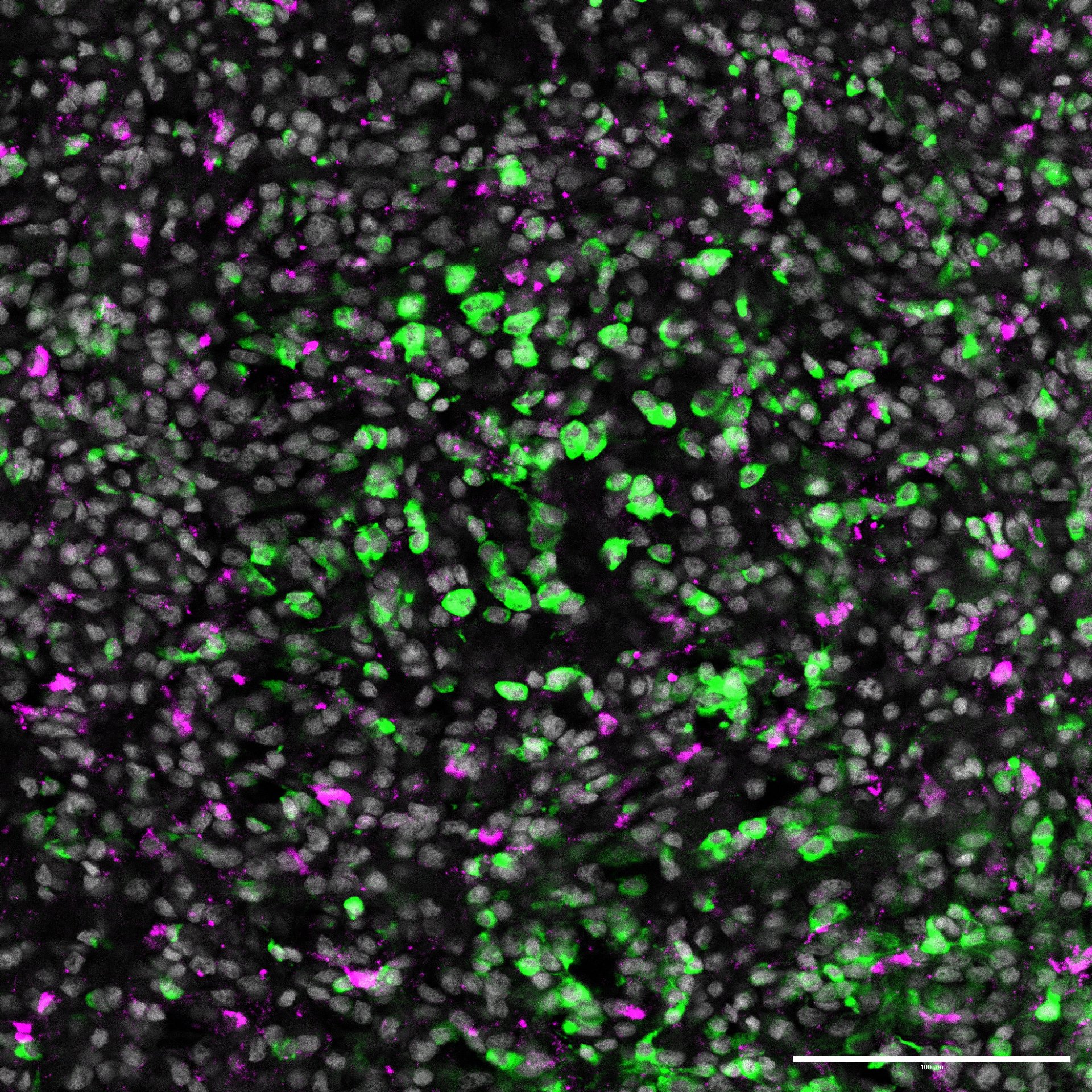

Supplement: Supplementary file 14 — Source data Fig. 7 [file 44318_2025_662_MOESM14_ESM.zip › Figure 7/7G/ID_8_Region_2_Control_RNAi_Probe_dd234_rhod_SMEDWI1_FITC_DAPI_20x_z3.jpg]

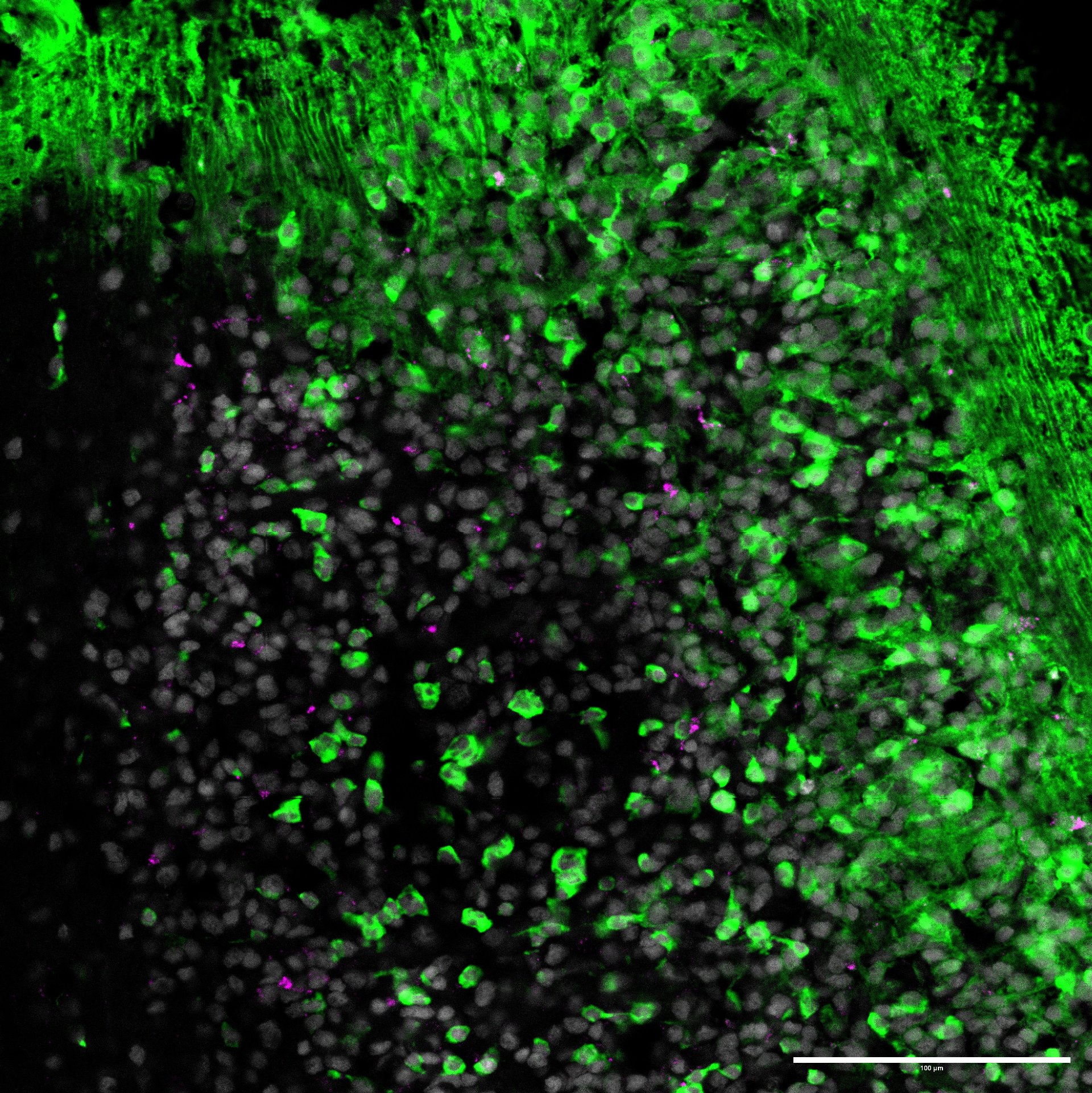

Supplement: Supplementary file 14 — Source data Fig. 7 [file 44318_2025_662_MOESM14_ESM.zip › Figure 7/7G/ID_9_Region_1_Control_RNAi_Probe_dd234_rhod_SMEDWI1_FITC_DAPI_20x_z3.jpg]

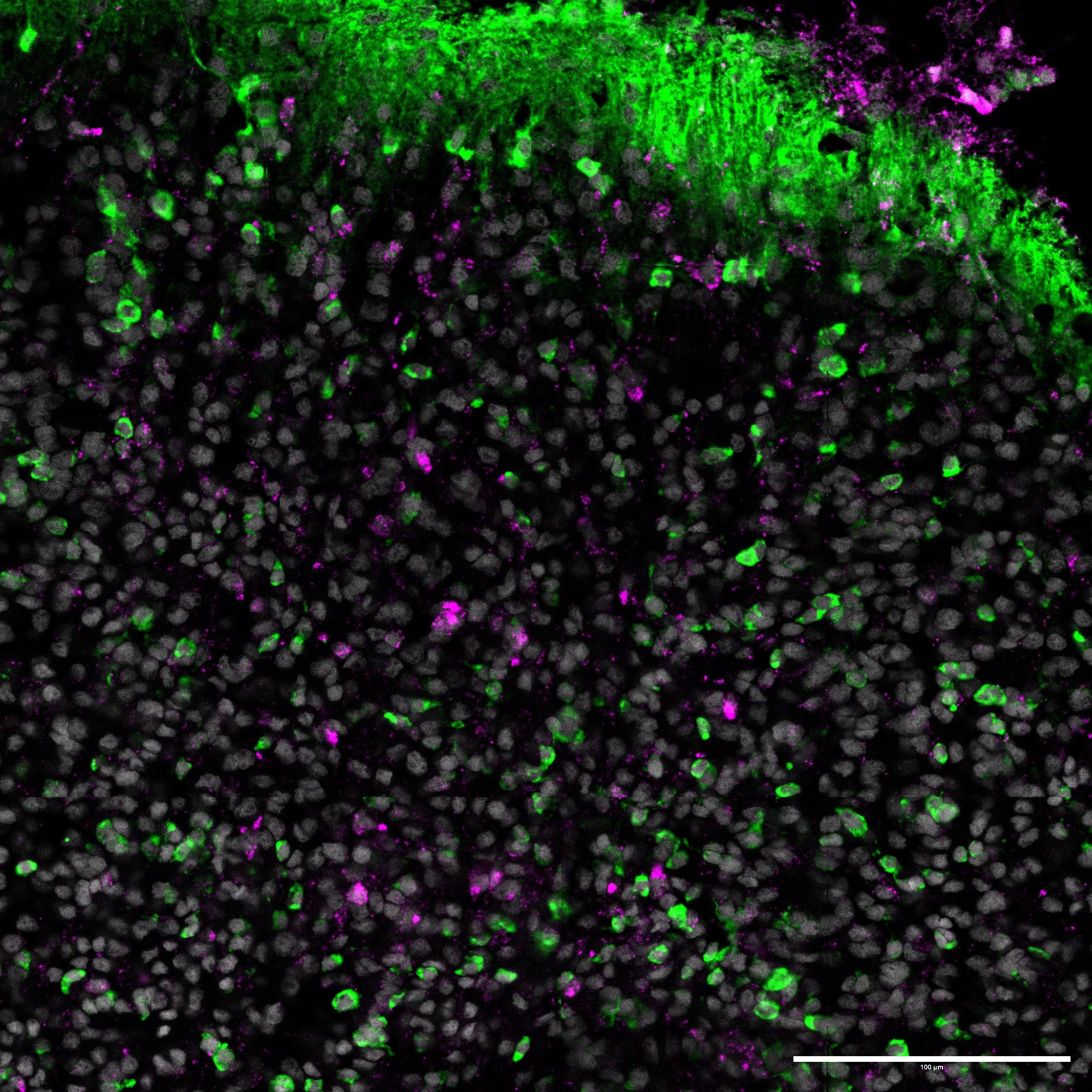

Supplement: Supplementary file 14 — Source data Fig. 7 [file 44318_2025_662_MOESM14_ESM.zip › Figure 7/7G/ID_9_Region_1_Triple_RNAi_Probe_dd234_rhod_SMEDWI1_FITC_DAPI_20x_z3.jpg]

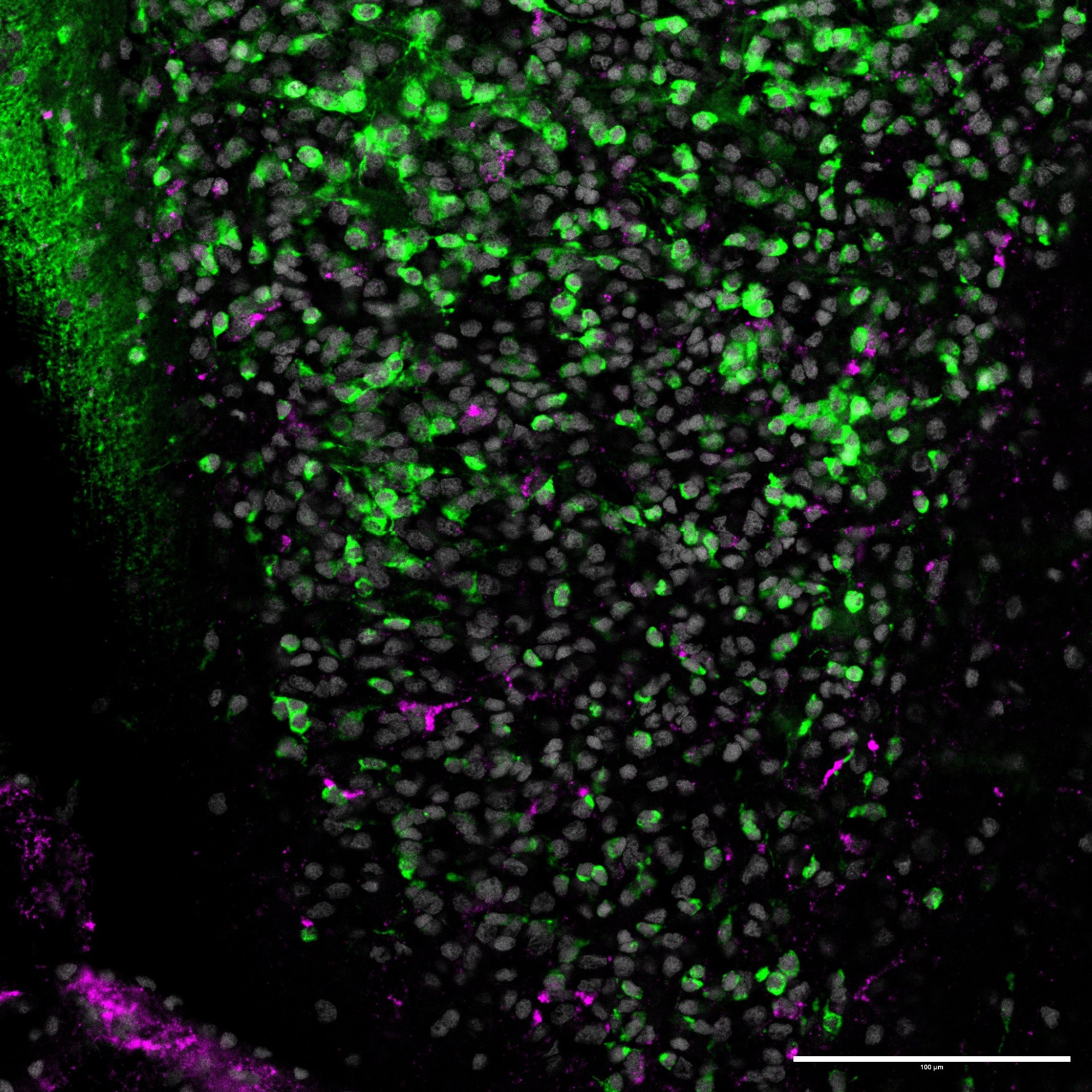

Supplement: Supplementary file 14 — Source data Fig. 7 [file 44318_2025_662_MOESM14_ESM.zip › Figure 7/7G/ID_9_Region_2_Triple_RNAi_Probe_dd234_rhod_SMEDWI1_FITC_DAPI_20x_z3.jpg]

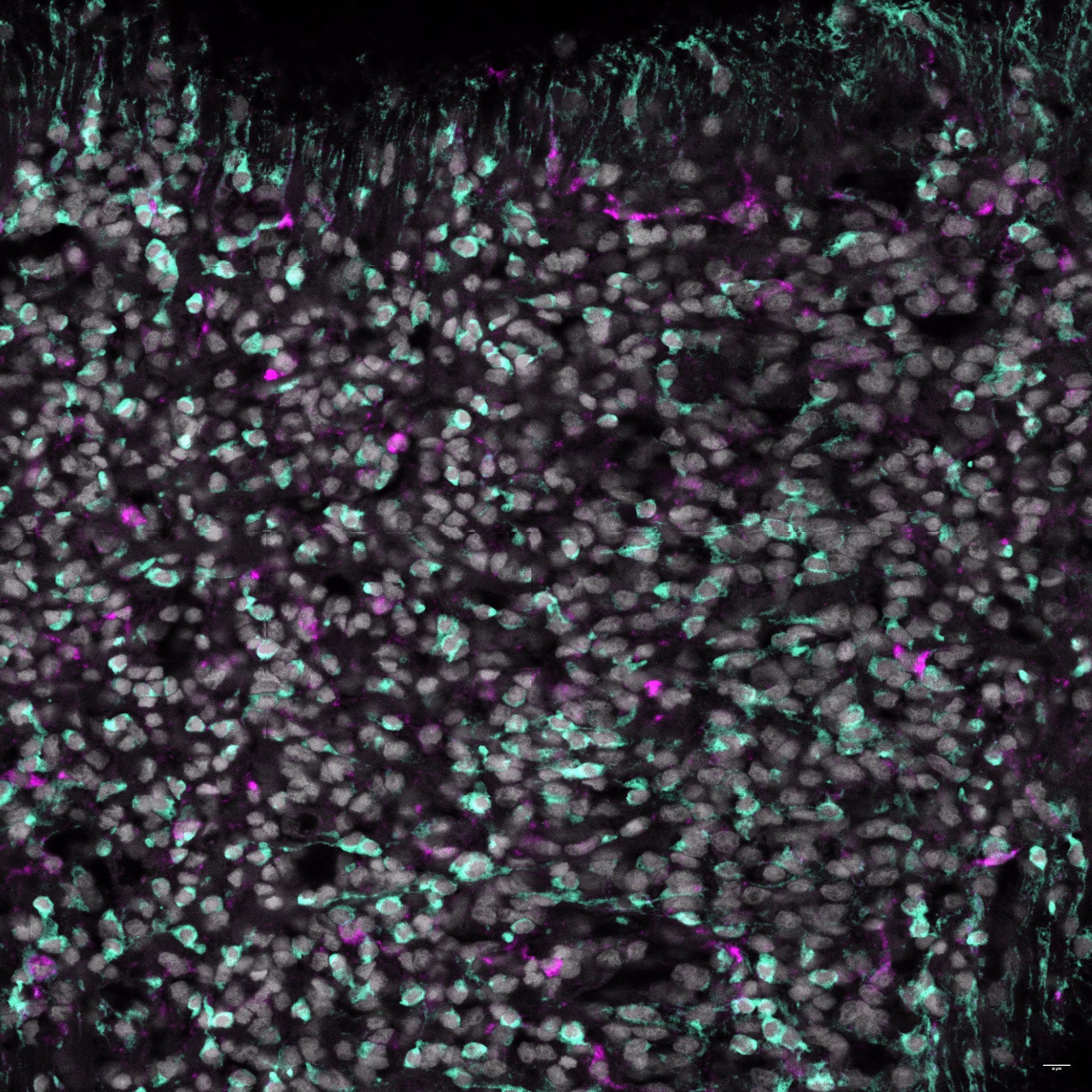

Supplement: Supplementary file 14 — Source data Fig. 7 [file 44318_2025_662_MOESM14_ESM.zip › Figure 7/7G/Main_figure_panel_Control_RNAi_Probe_dd234_rhod_SMEDWI1_FITC_DAPI_20x_z3._Merged.jpg]

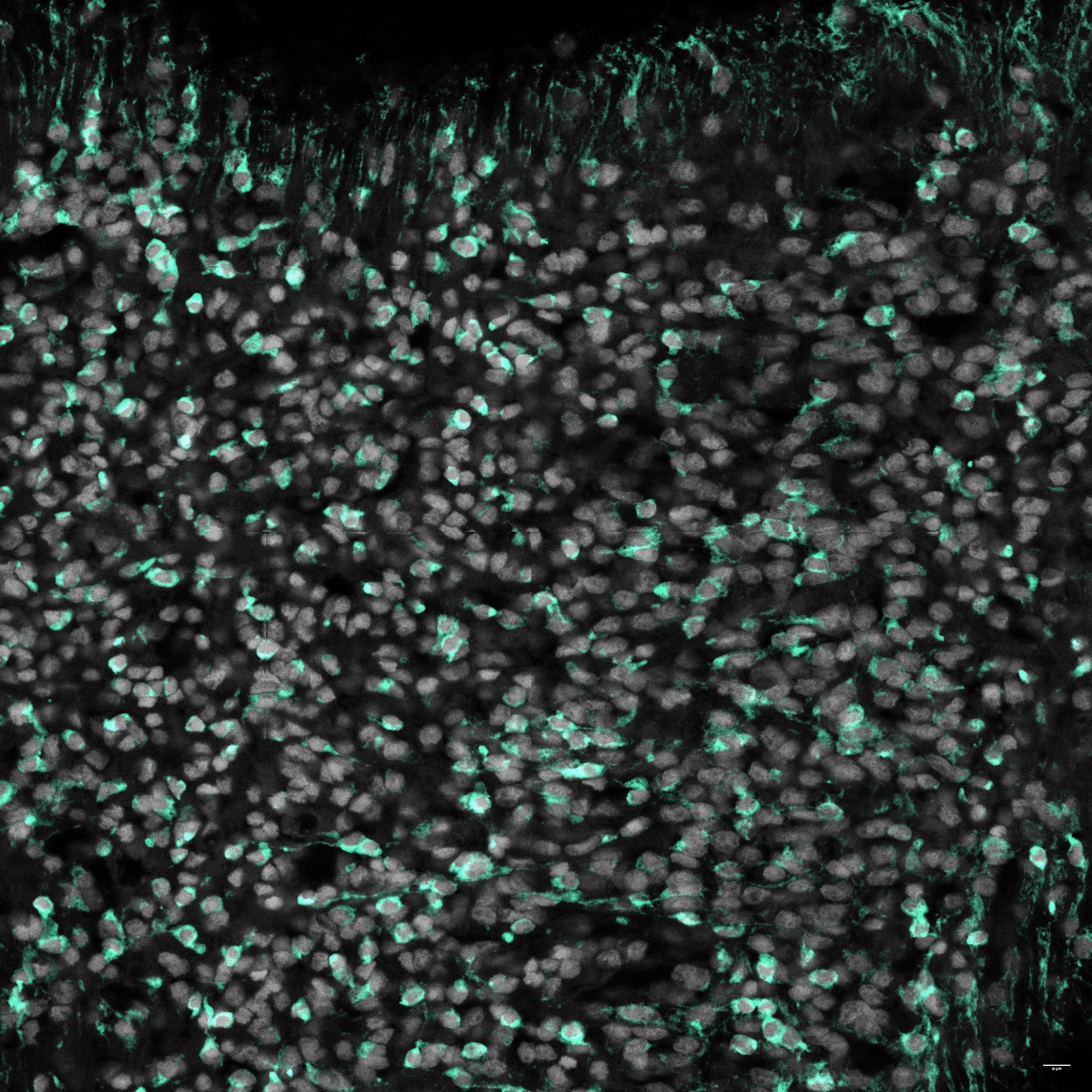

Supplement: Supplementary file 14 — Source data Fig. 7 [file 44318_2025_662_MOESM14_ESM.zip › Figure 7/7G/Main_figure_panel_Control_RNAi_Probe_dd234_rhod_SMEDWI1_FITC_DAPI_20x_z3_FITC_channel.jpg]

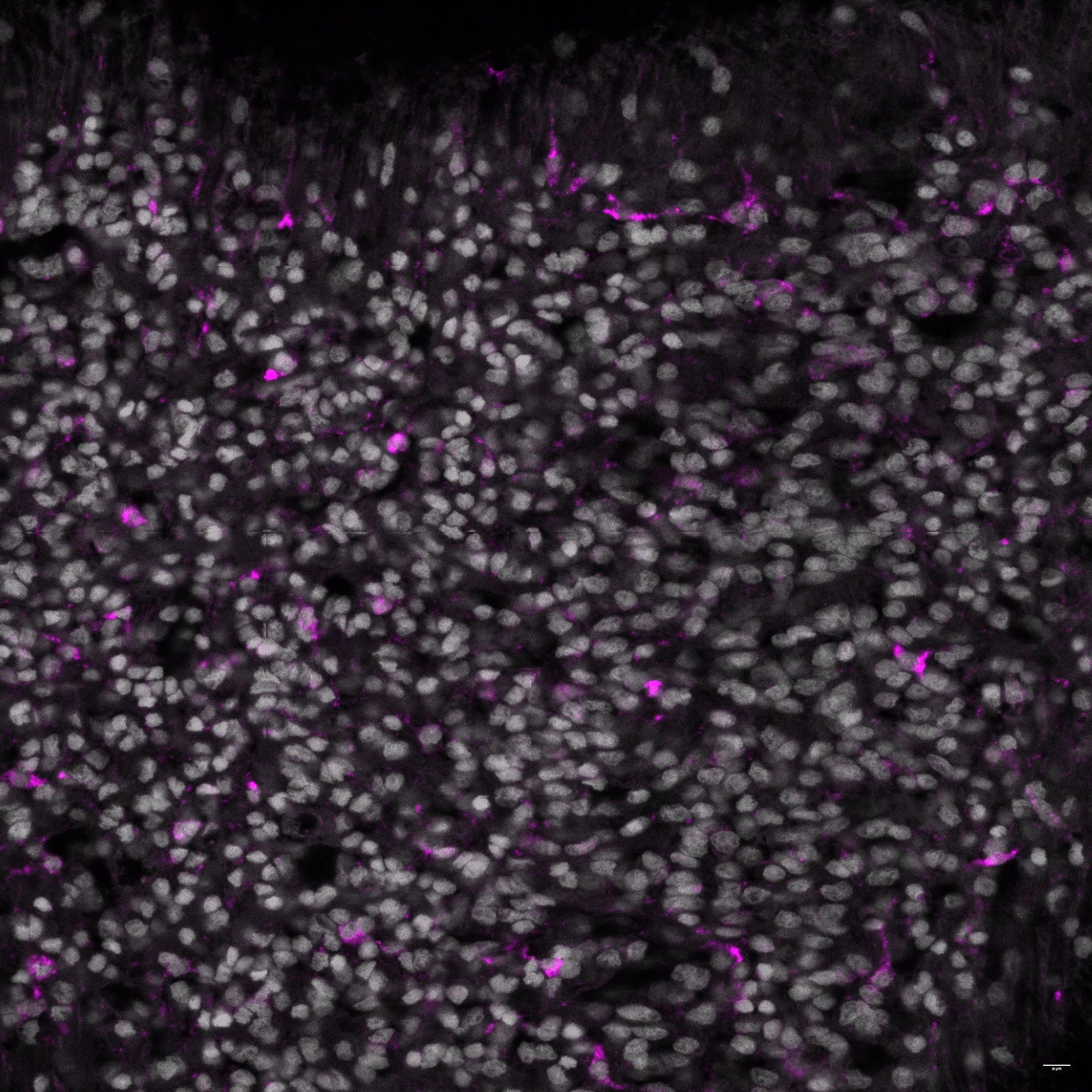

Supplement: Supplementary file 14 — Source data Fig. 7 [file 44318_2025_662_MOESM14_ESM.zip › Figure 7/7G/Main_figure_panel_Control_RNAi_Probe_dd234_rhod_SMEDWI1_FITC_DAPI_20x_z3_Magenta_channel.jpg]

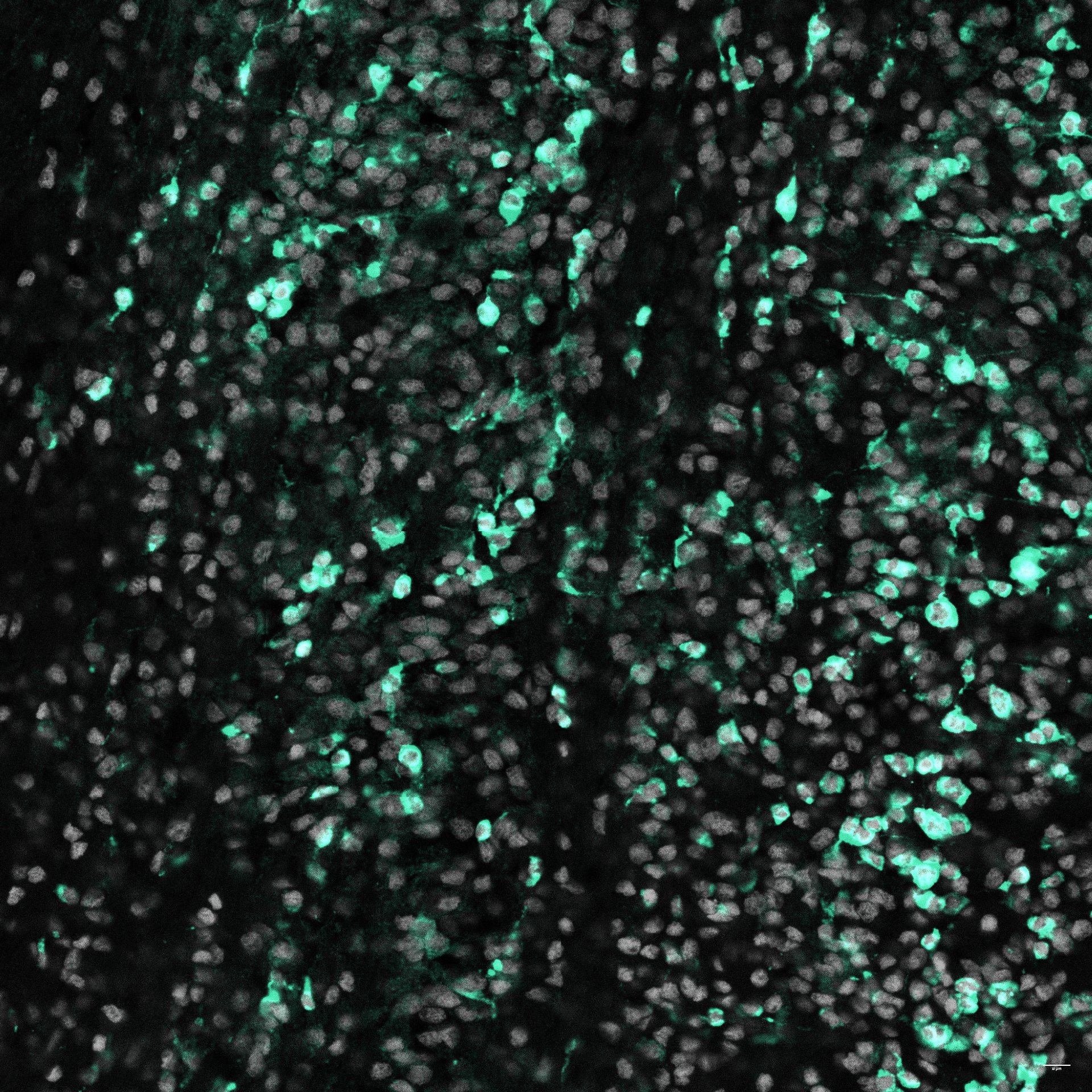

Supplement: Supplementary file 14 — Source data Fig. 7 [file 44318_2025_662_MOESM14_ESM.zip › Figure 7/7G/Main_figure_panel_Triple_RNAi_Probe_dd234_rhod_SMEDWI1_FITC_DAPI_20x_z3_FITC_channel.jpg]

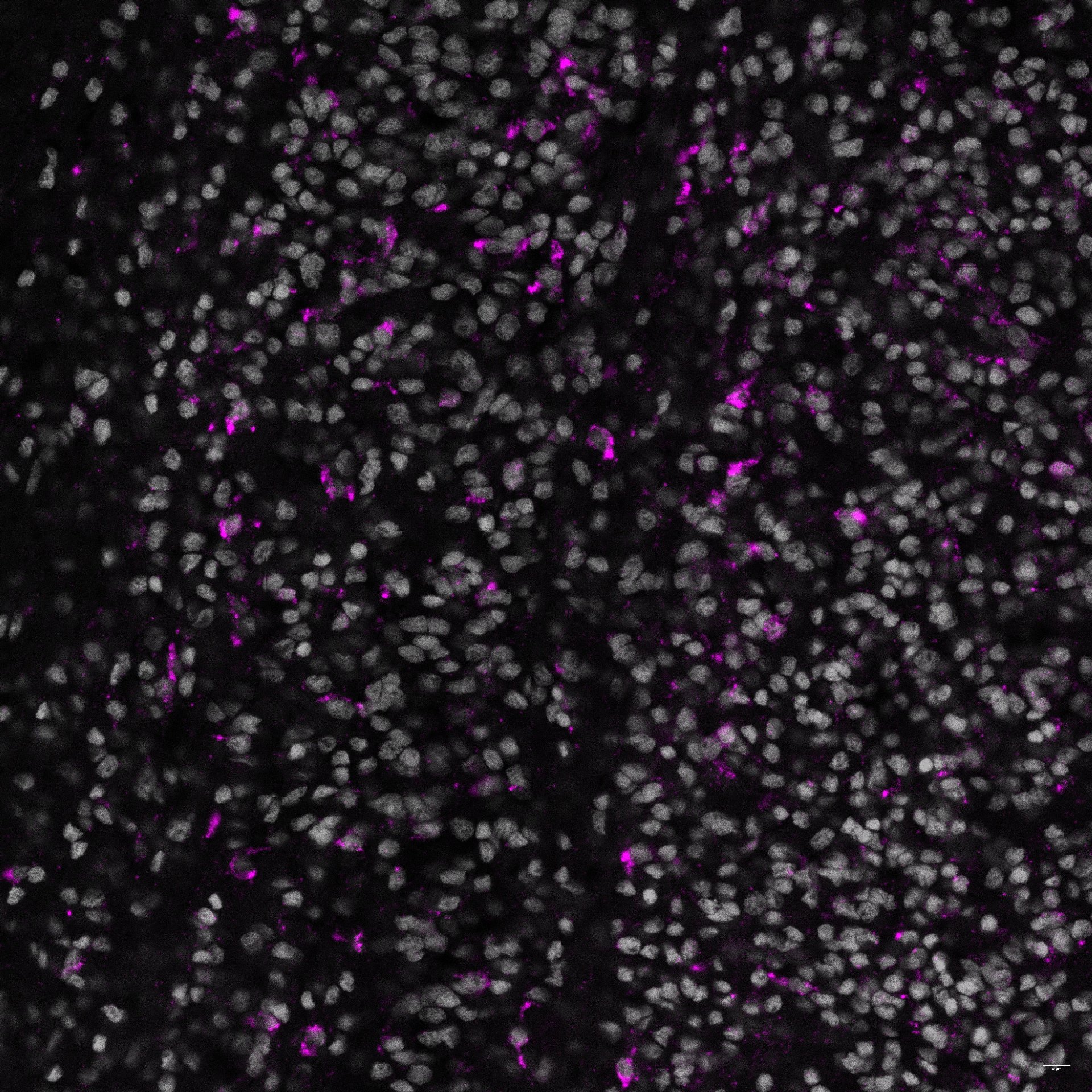

Supplement: Supplementary file 14 — Source data Fig. 7 [file 44318_2025_662_MOESM14_ESM.zip › Figure 7/7G/Main_figure_panel_Triple_RNAi_Probe_dd234_rhod_SMEDWI1_FITC_DAPI_20x_z3_Magenta_channel.jpg]

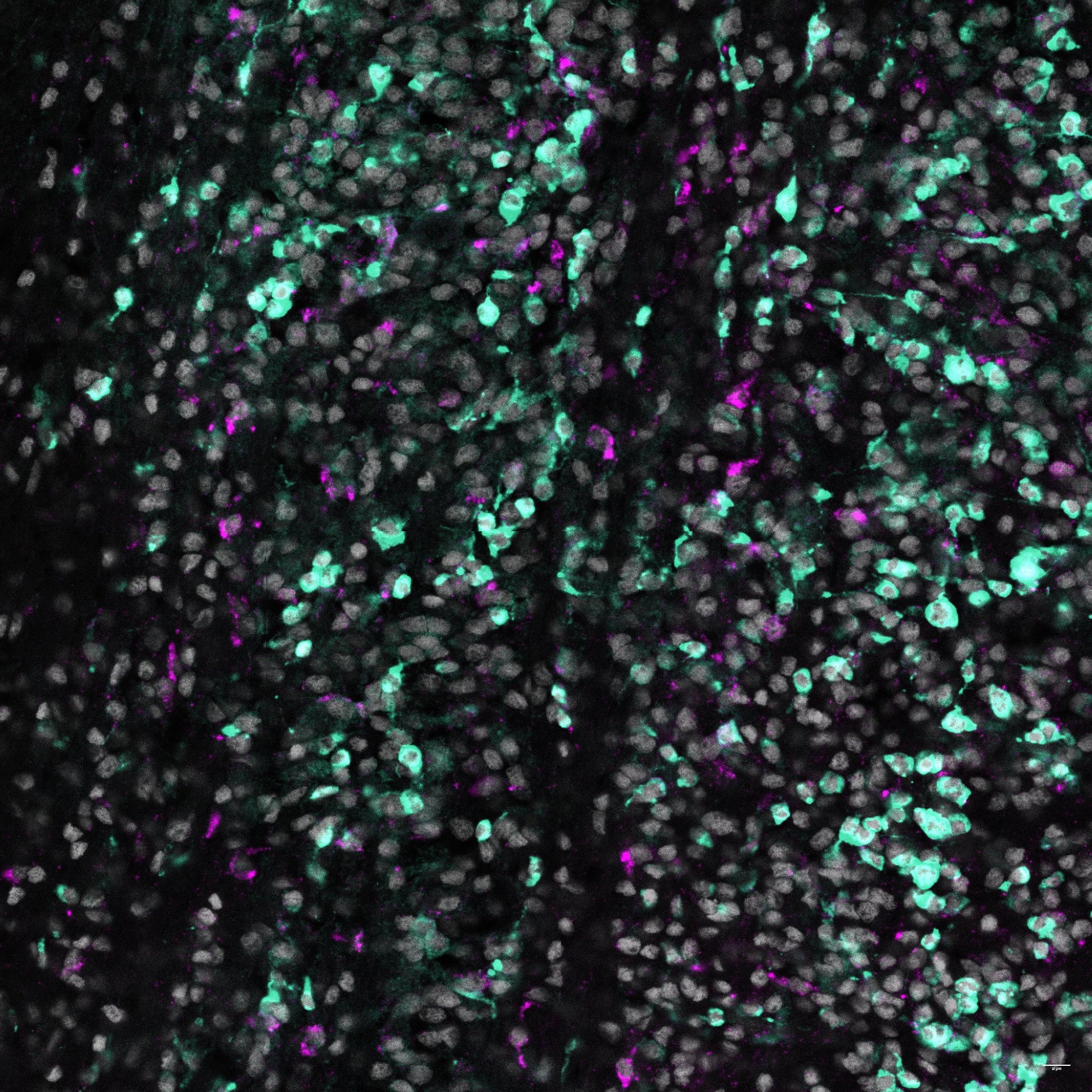

Supplement: Supplementary file 14 — Source data Fig. 7 [file 44318_2025_662_MOESM14_ESM.zip › Figure 7/7G/Main_figure_panel_Triple_RNAi_Probe_dd234_rhod_SMEDWI1_FITC_DAPI_20x_z3_Merged.jpg]
