# Supplementary figures and images for: Single-nucleotide m⁶A mapping uncovers redundant YTHDF function in planarian progenitor fate selection (part 5 of 6)
Source: EMBO J. 2026 Jan 3;45(3):749–88. doi: 10.1038/s44318-025-00662-3 (PMC12864844; doi:10.1038/s44318-025-00662-3)

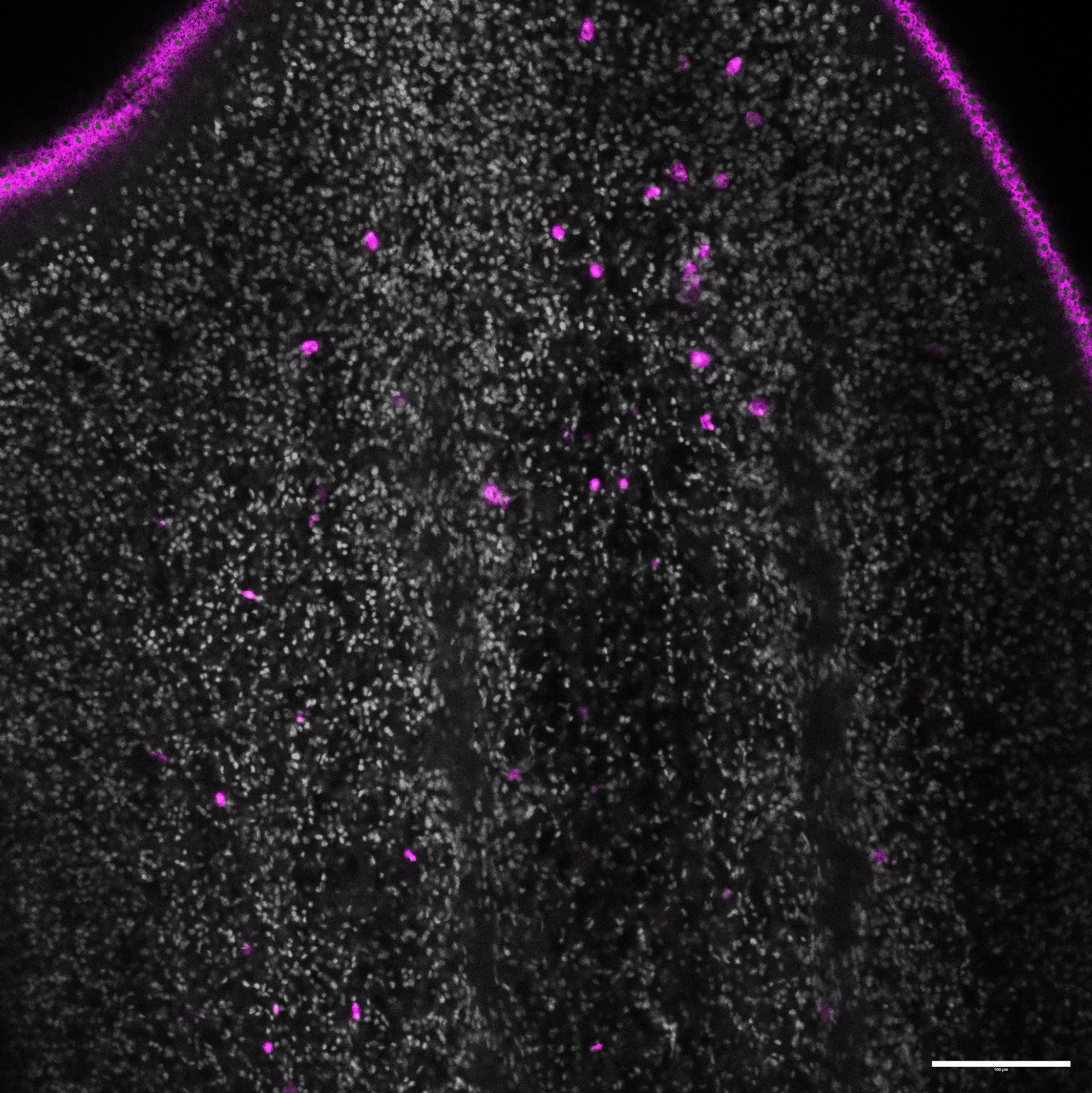

Supplement: Supplementary file 14 — Source data Fig. 7 [file 44318_2025_662_MOESM14_ESM.zip › Figure 7/7B/ID_5_Triple_RNAi_H3P_rhod_DAPI_20x.jpg]

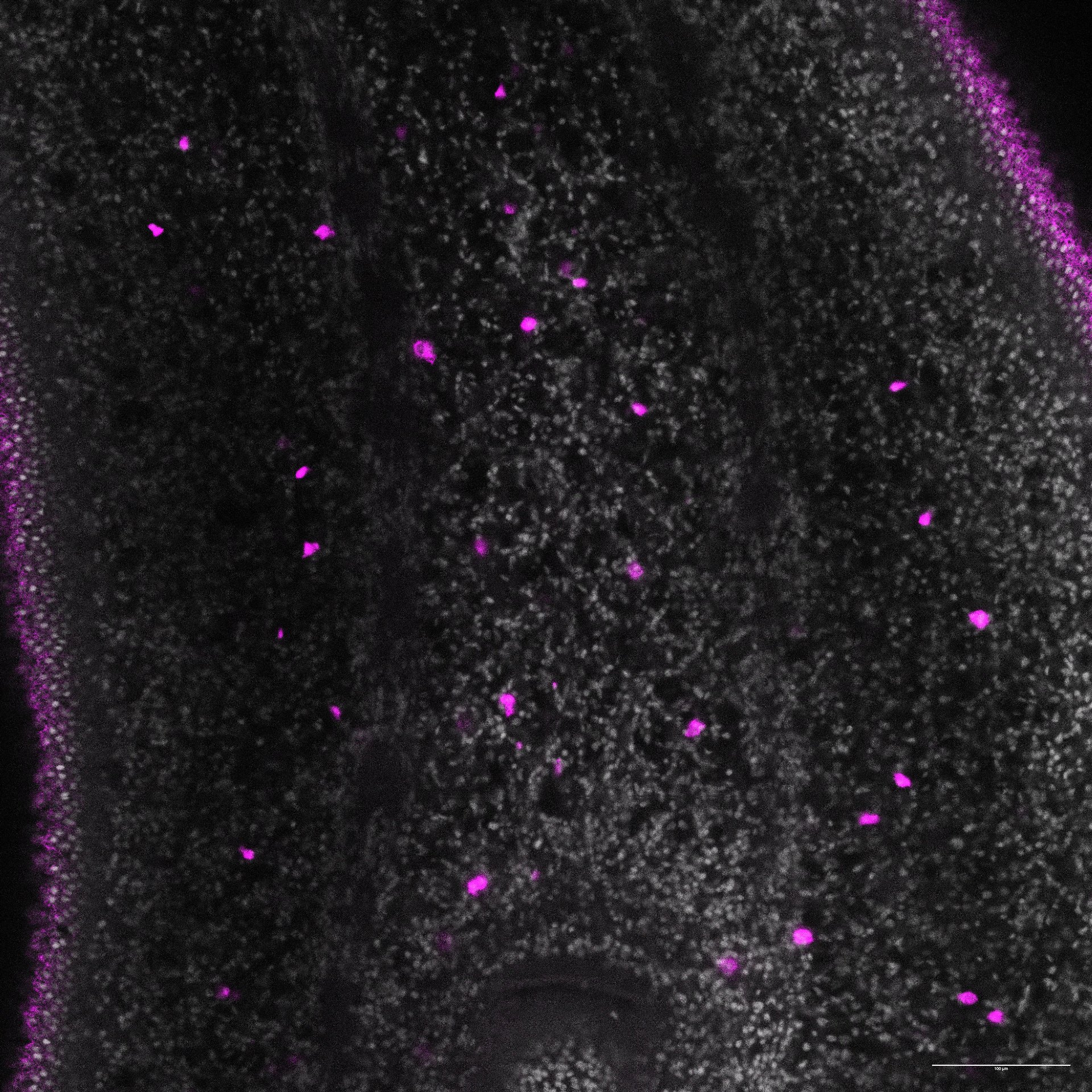

Supplement: Supplementary file 14 — Source data Fig. 7 [file 44318_2025_662_MOESM14_ESM.zip › Figure 7/7B/ID_6_Control_RNAi_H3P_rhod_DAPI_20x.jpg]

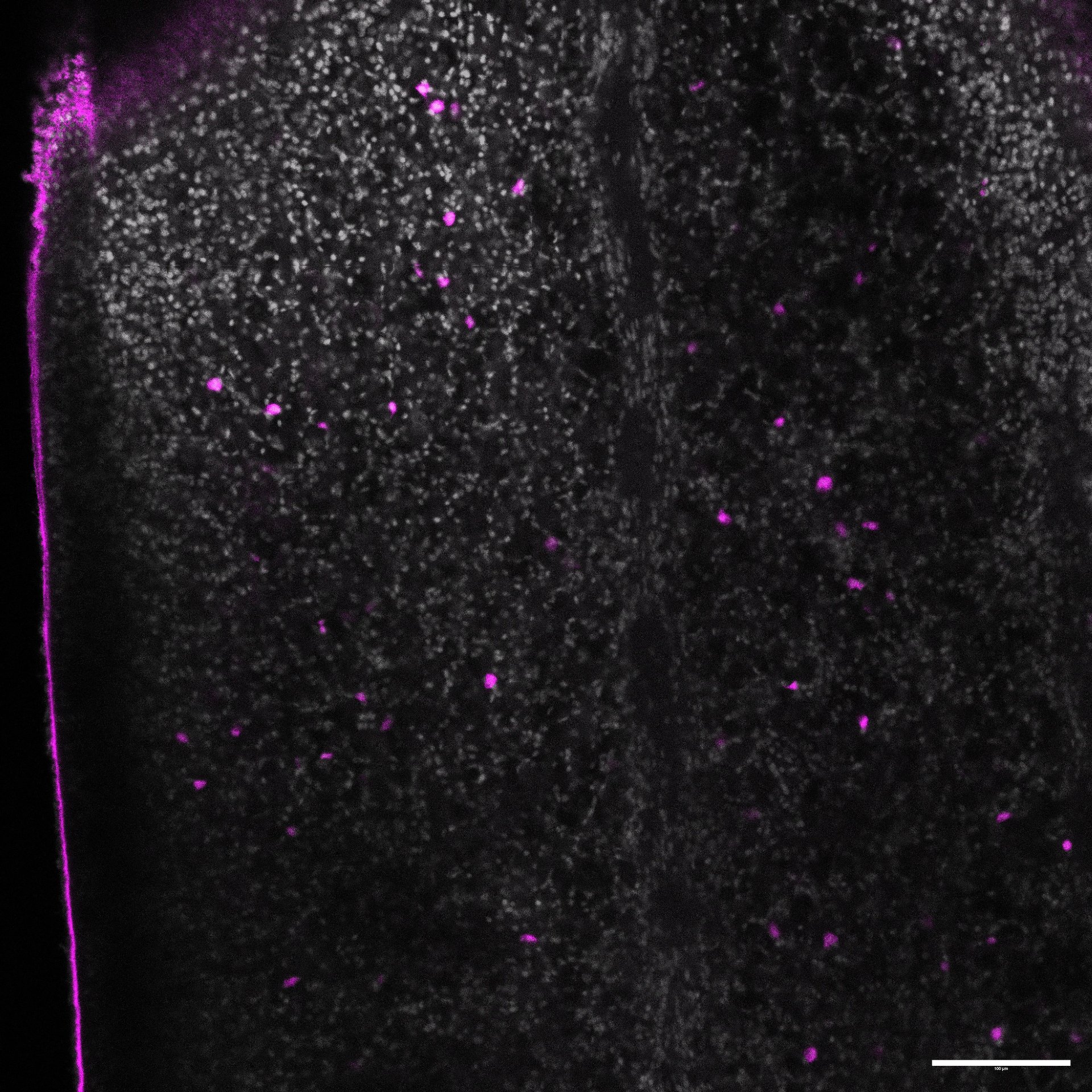

Supplement: Supplementary file 14 — Source data Fig. 7 [file 44318_2025_662_MOESM14_ESM.zip › Figure 7/7B/ID_6_Triple_RNAi_H3P_rhod_DAPI_20x.jpg]

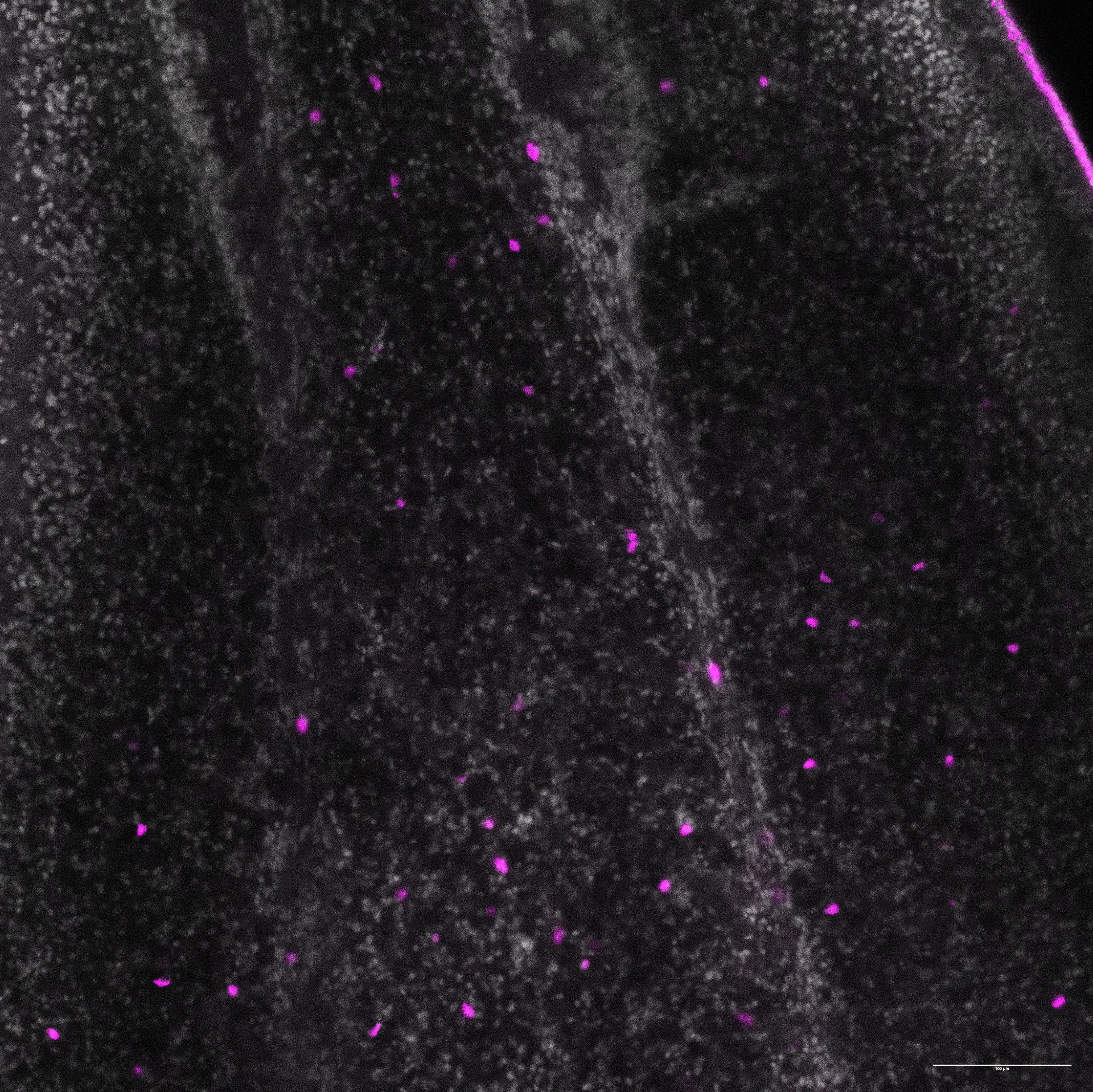

Supplement: Supplementary file 14 — Source data Fig. 7 [file 44318_2025_662_MOESM14_ESM.zip › Figure 7/7B/ID_7_Control_RNAi_H3P_rhod_DAPI_20x.jpg]

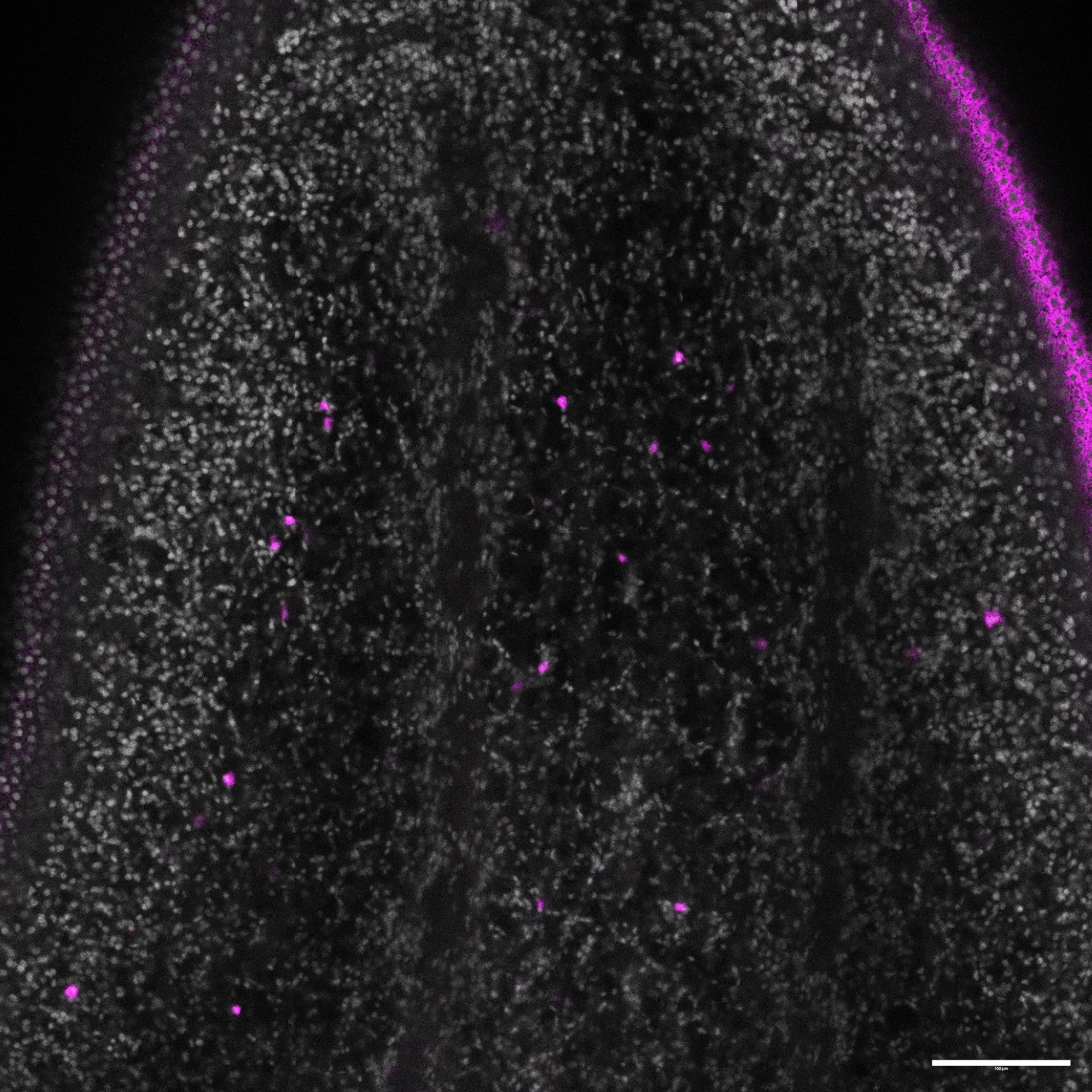

Supplement: Supplementary file 14 — Source data Fig. 7 [file 44318_2025_662_MOESM14_ESM.zip › Figure 7/7B/ID_7_Triple_RNAi_H3P_rhod_DAPI_20x.jpg]

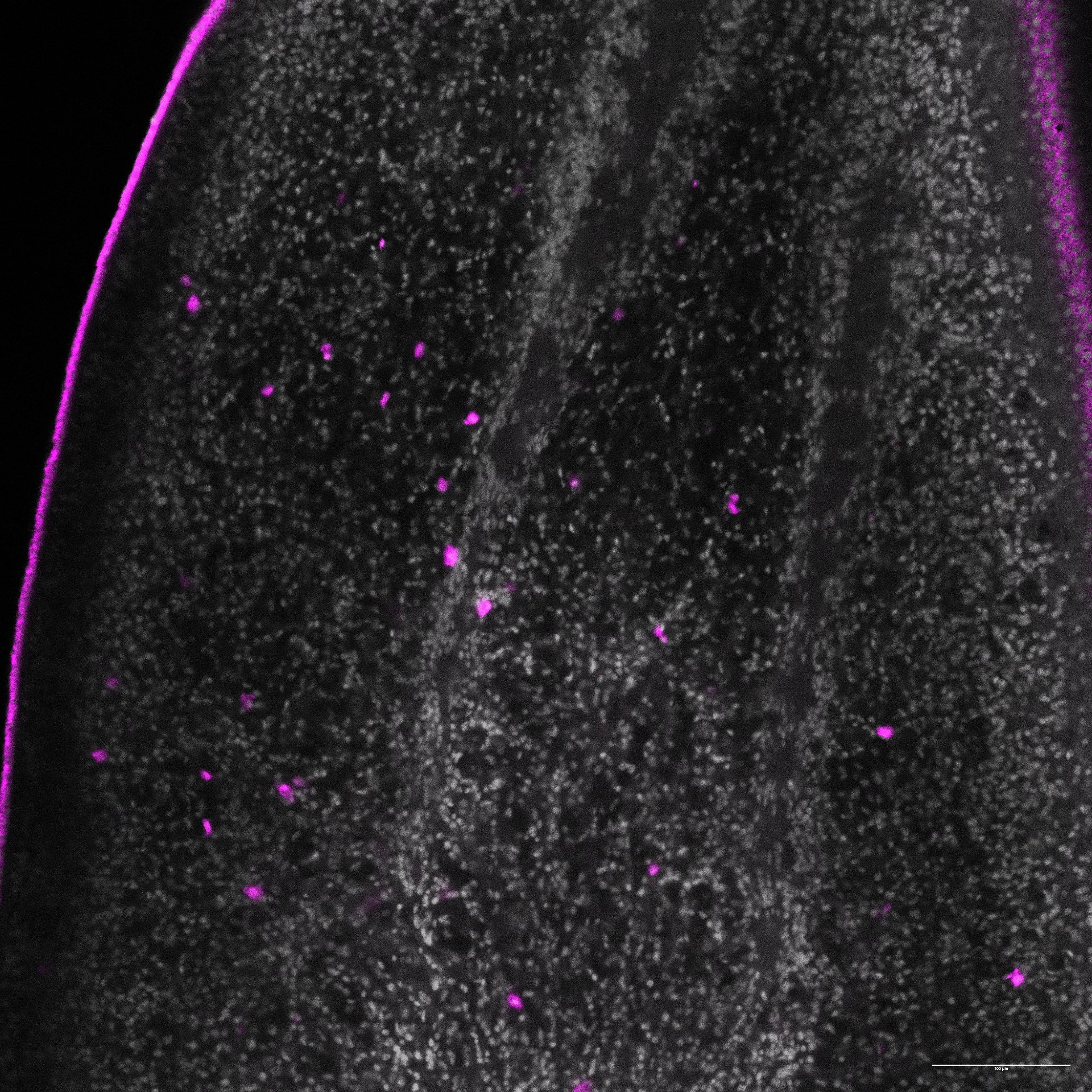

Supplement: Supplementary file 14 — Source data Fig. 7 [file 44318_2025_662_MOESM14_ESM.zip › Figure 7/7B/ID_8_Control_RNAi_H3P_rhod_DAPI_20x.jpg]

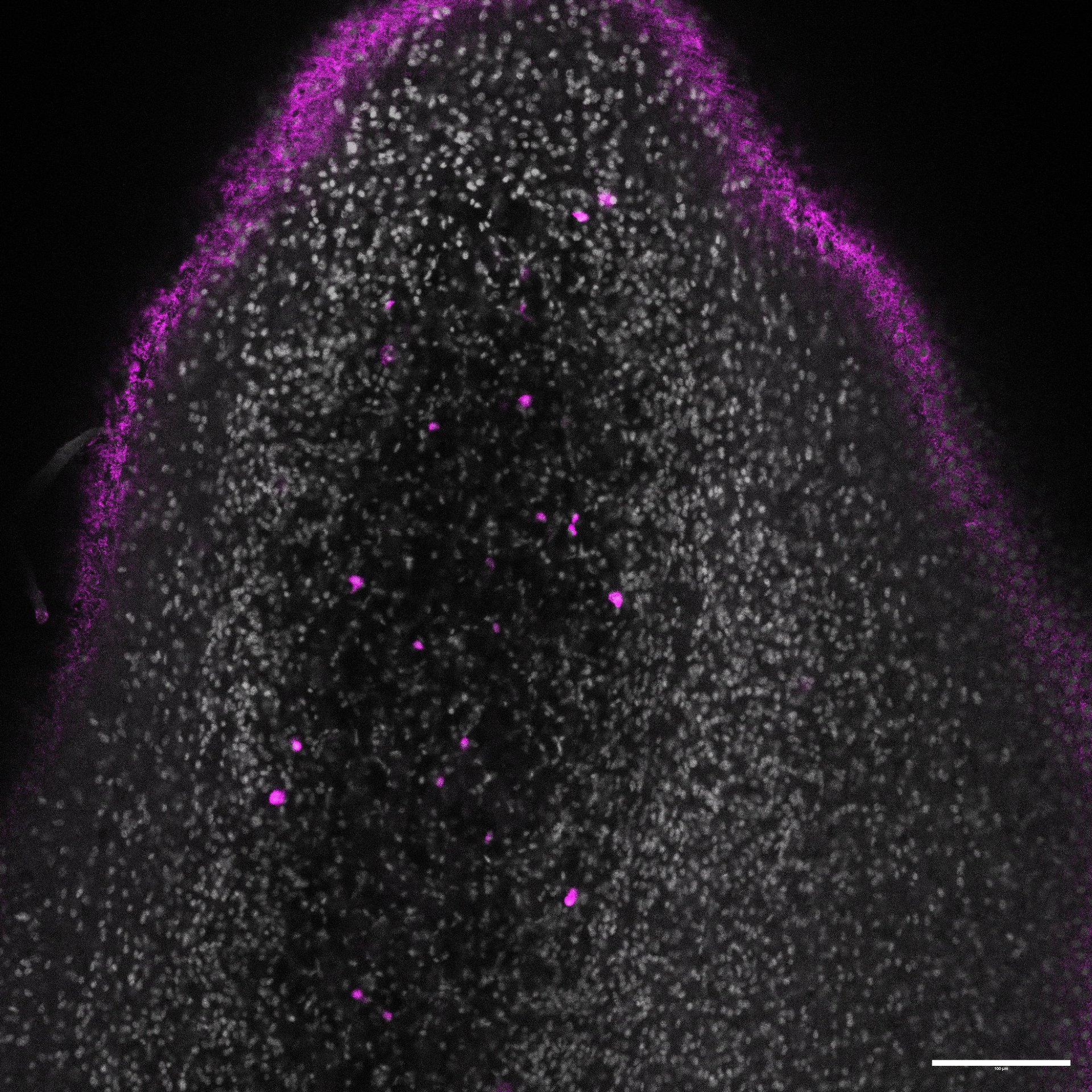

Supplement: Supplementary file 14 — Source data Fig. 7 [file 44318_2025_662_MOESM14_ESM.zip › Figure 7/7B/ID_8_Triple_RNAi_H3P_rhod_DAPI_20x.jpg]

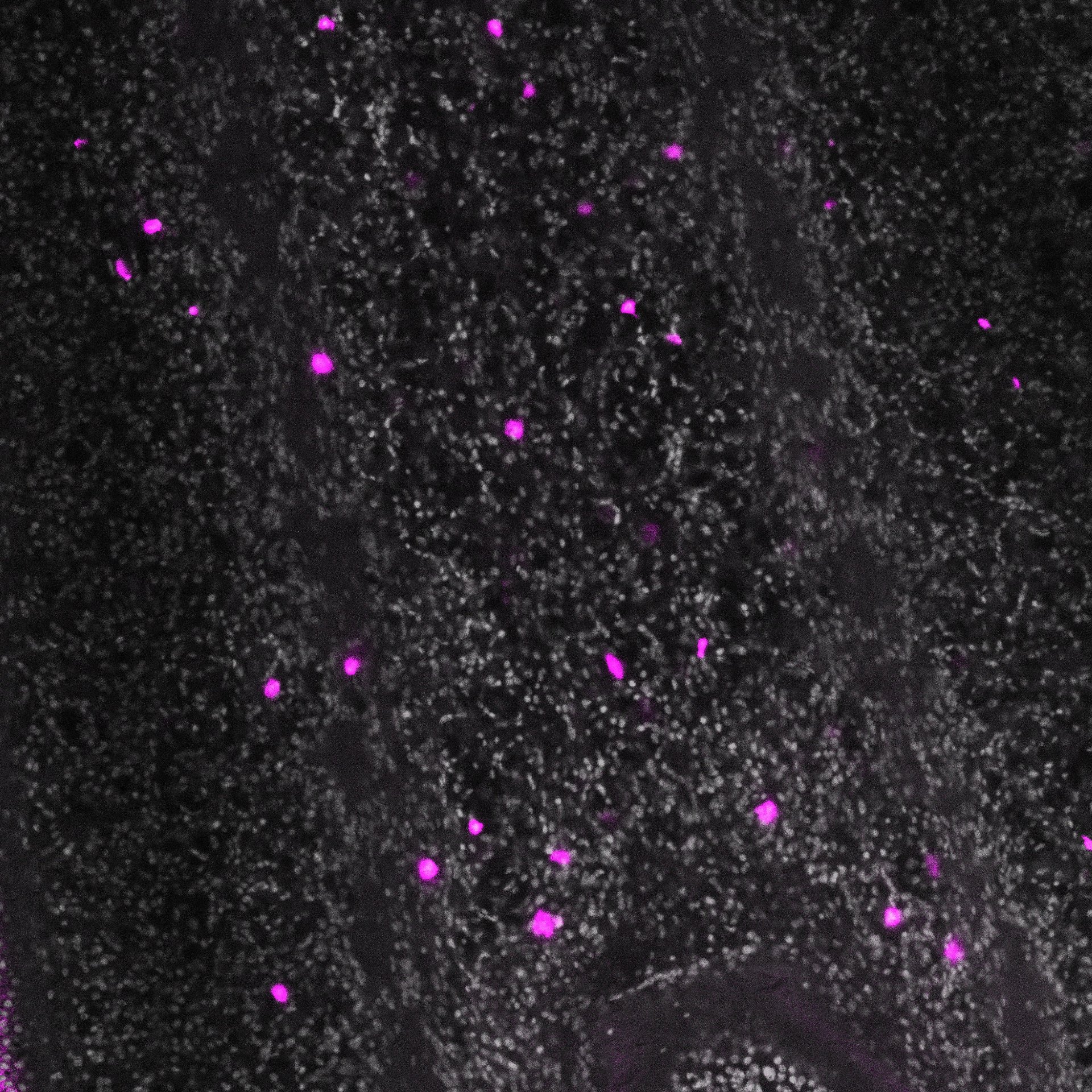

Supplement: Supplementary file 14 — Source data Fig. 7 [file 44318_2025_662_MOESM14_ESM.zip › Figure 7/7B/ID_9_Control_RNAi_H3P_rhod_DAPI_20x.jpg]

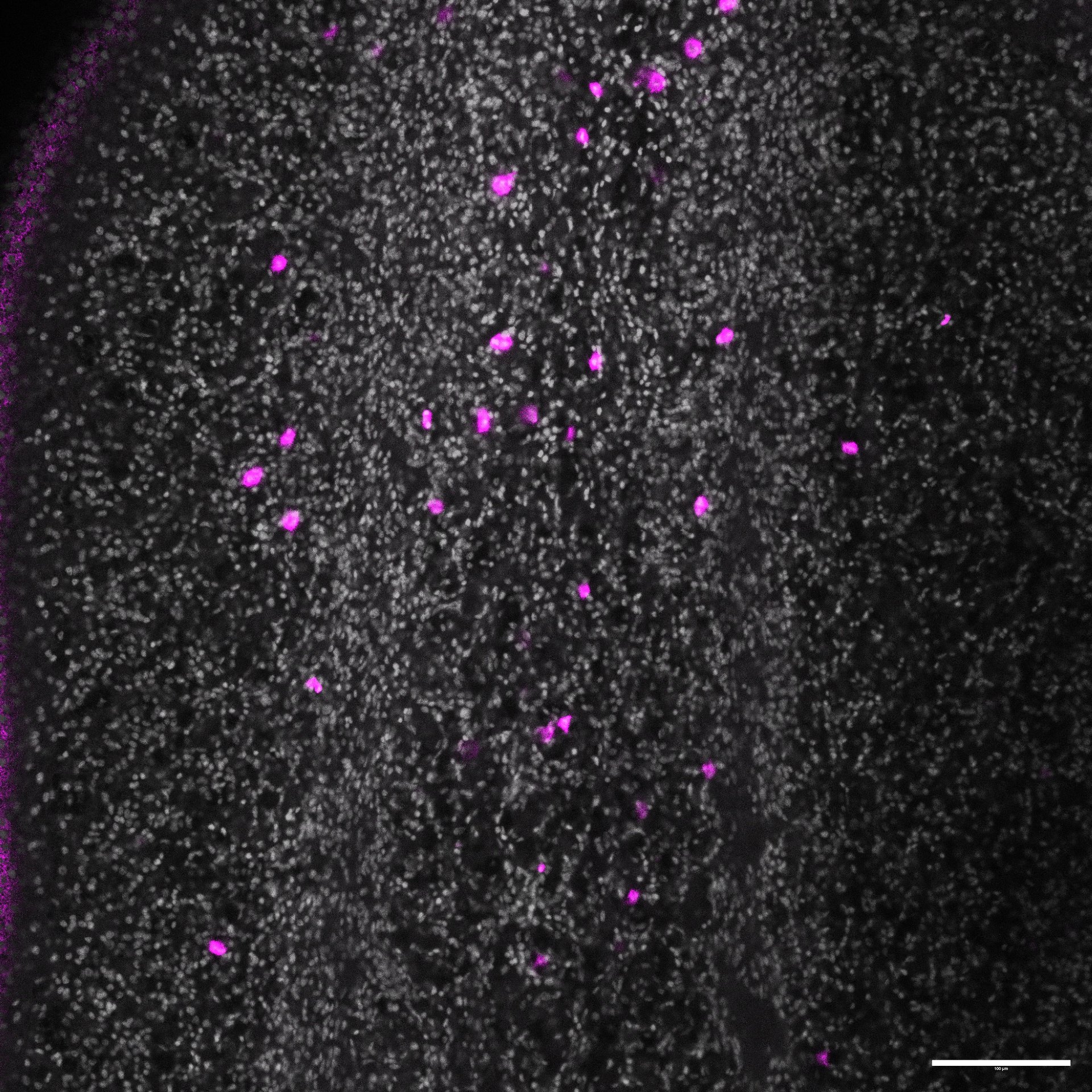

Supplement: Supplementary file 14 — Source data Fig. 7 [file 44318_2025_662_MOESM14_ESM.zip › Figure 7/7B/ID_9_Triple_RNAi_H3P_rhod_DAPI_20x.jpg]

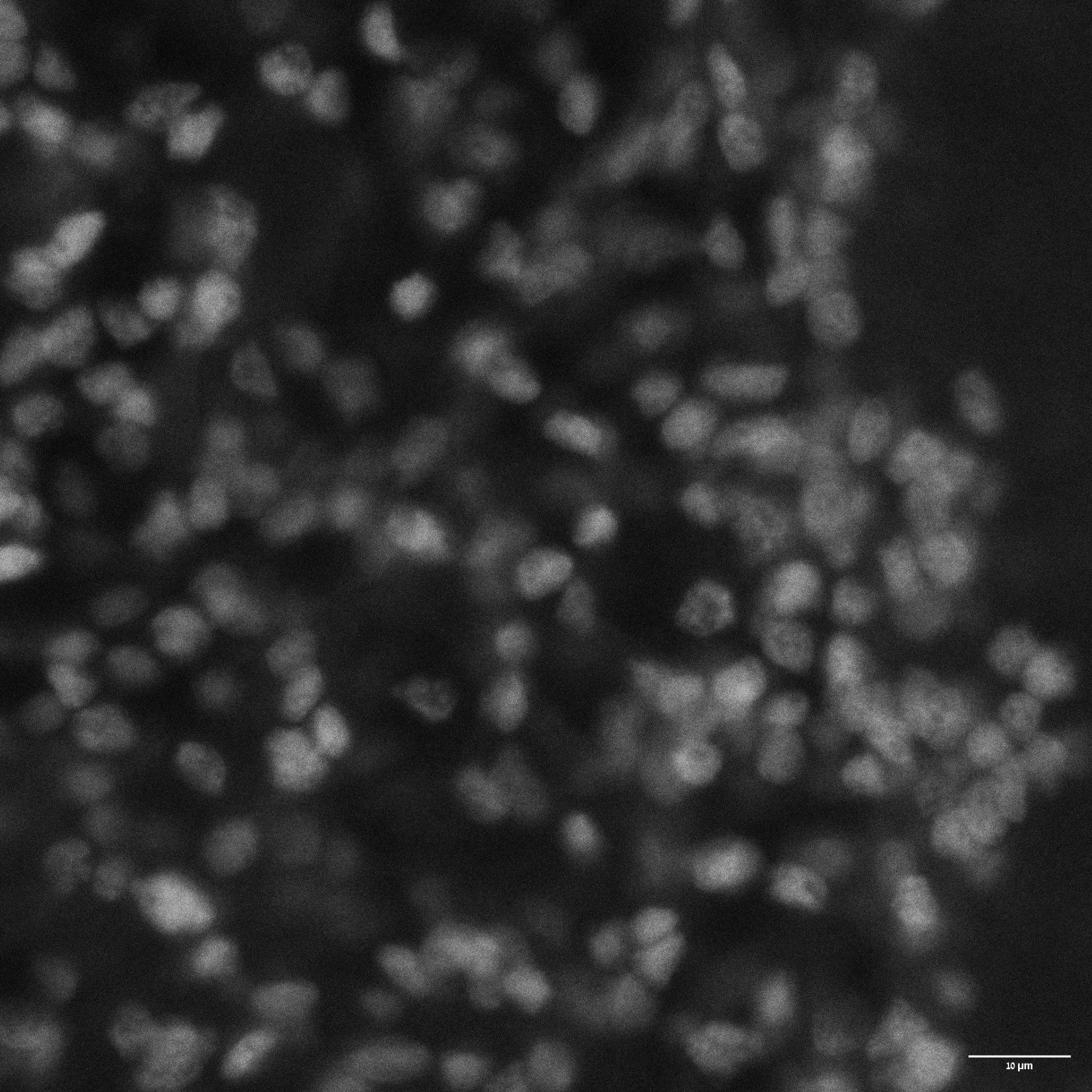

Supplement: Supplementary file 14 — Source data Fig. 7 [file 44318_2025_662_MOESM14_ESM.zip › Figure 7/7B/Main_figure_panel_Control_RNAi_H3P_DAPI.jpg]

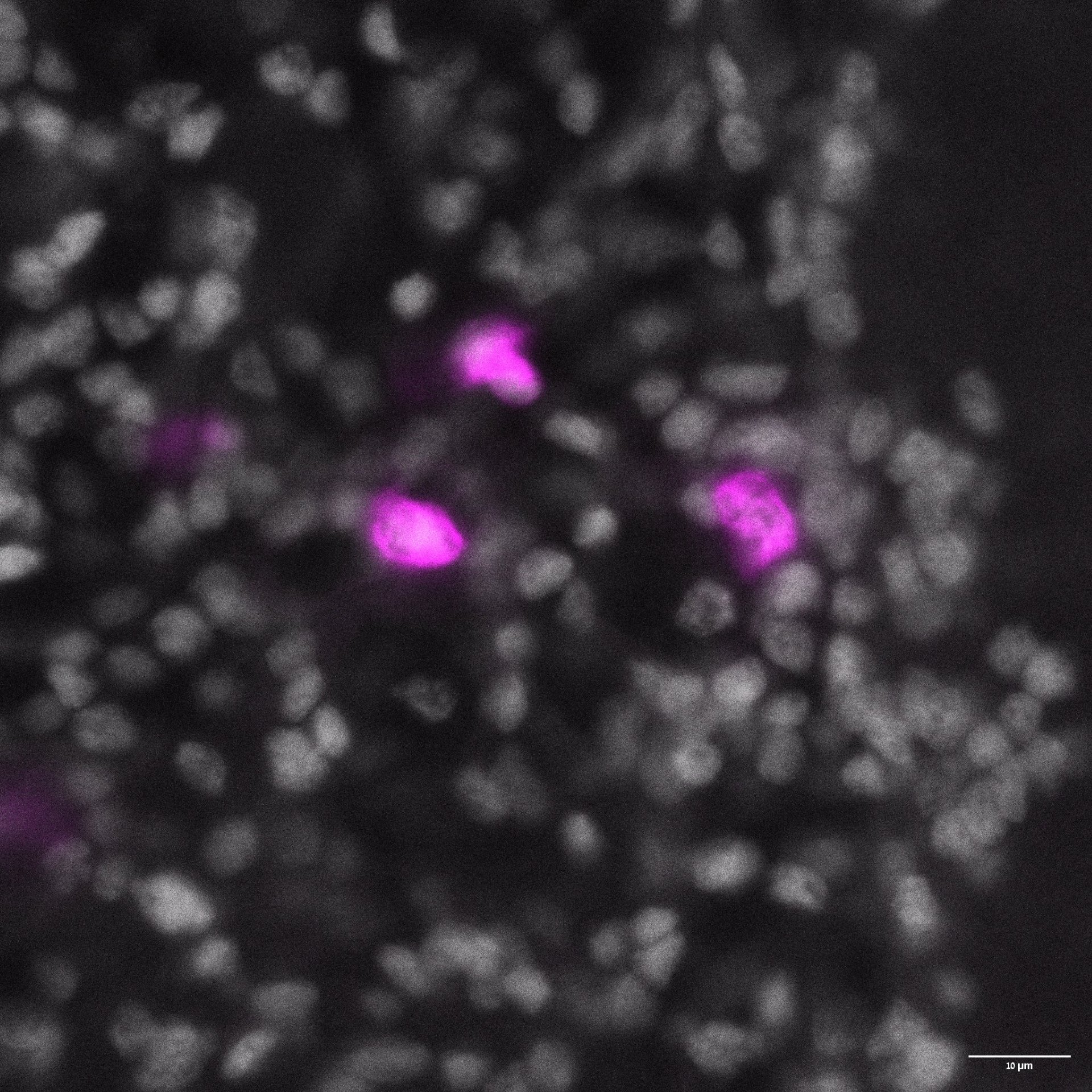

Supplement: Supplementary file 14 — Source data Fig. 7 [file 44318_2025_662_MOESM14_ESM.zip › Figure 7/7B/Main_figure_panel_Control_RNAi_H3P_Merged.jpg]

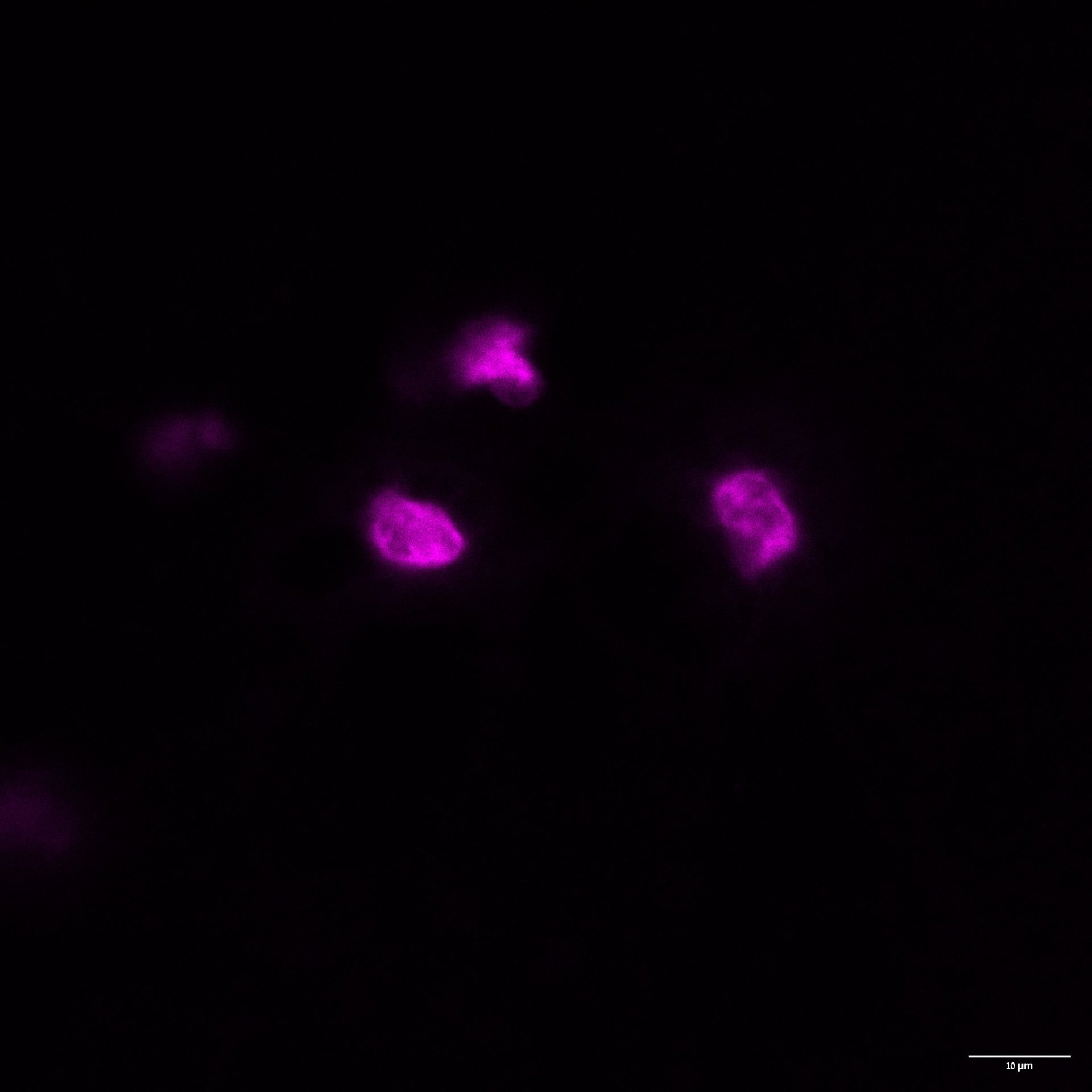

Supplement: Supplementary file 14 — Source data Fig. 7 [file 44318_2025_662_MOESM14_ESM.zip › Figure 7/7B/Main_figure_panel_Control_RNAi_H3P_Rhod.jpg]

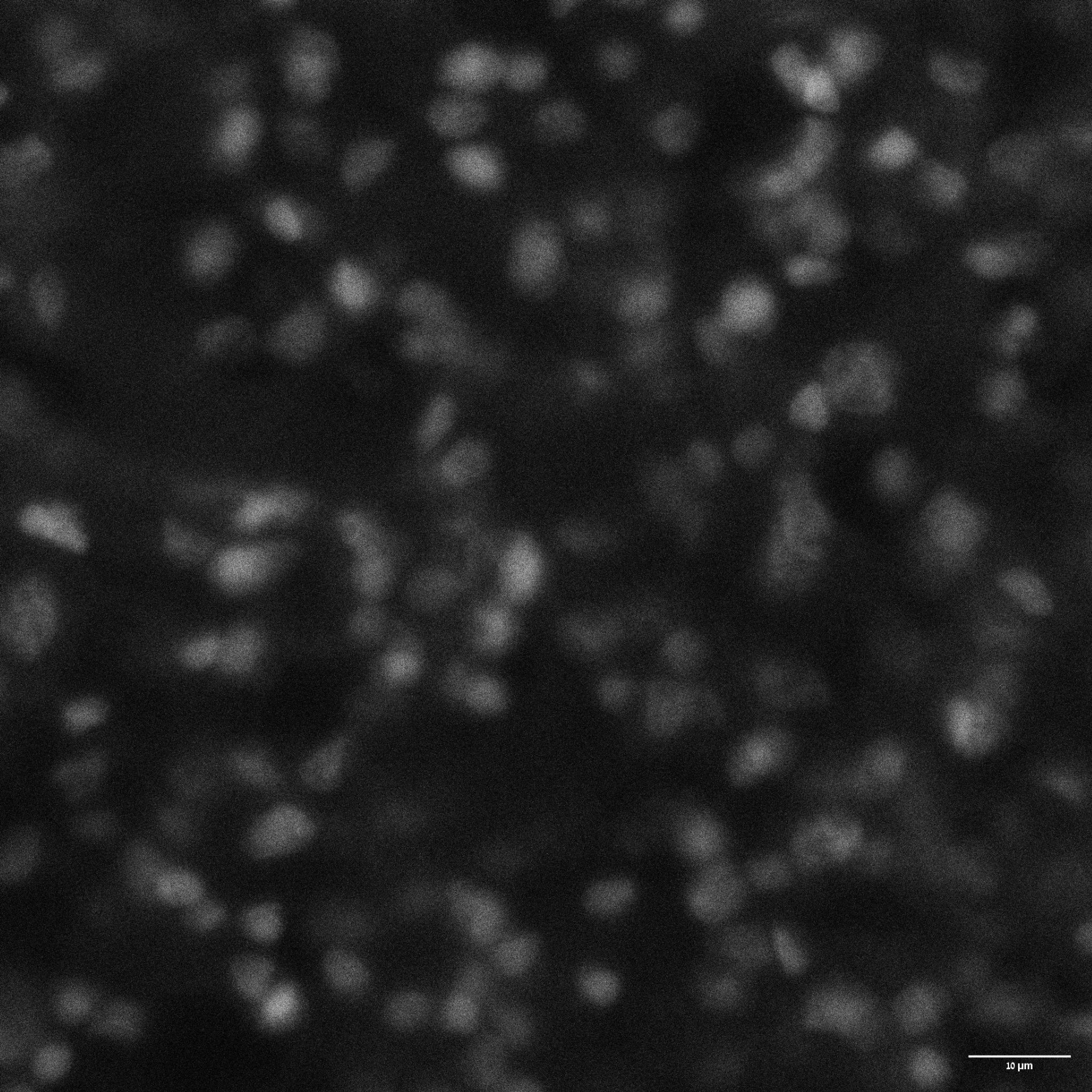

Supplement: Supplementary file 14 — Source data Fig. 7 [file 44318_2025_662_MOESM14_ESM.zip › Figure 7/7B/Main_figure_panel_Triple_RNAi_H3P_DAPI.jpg]

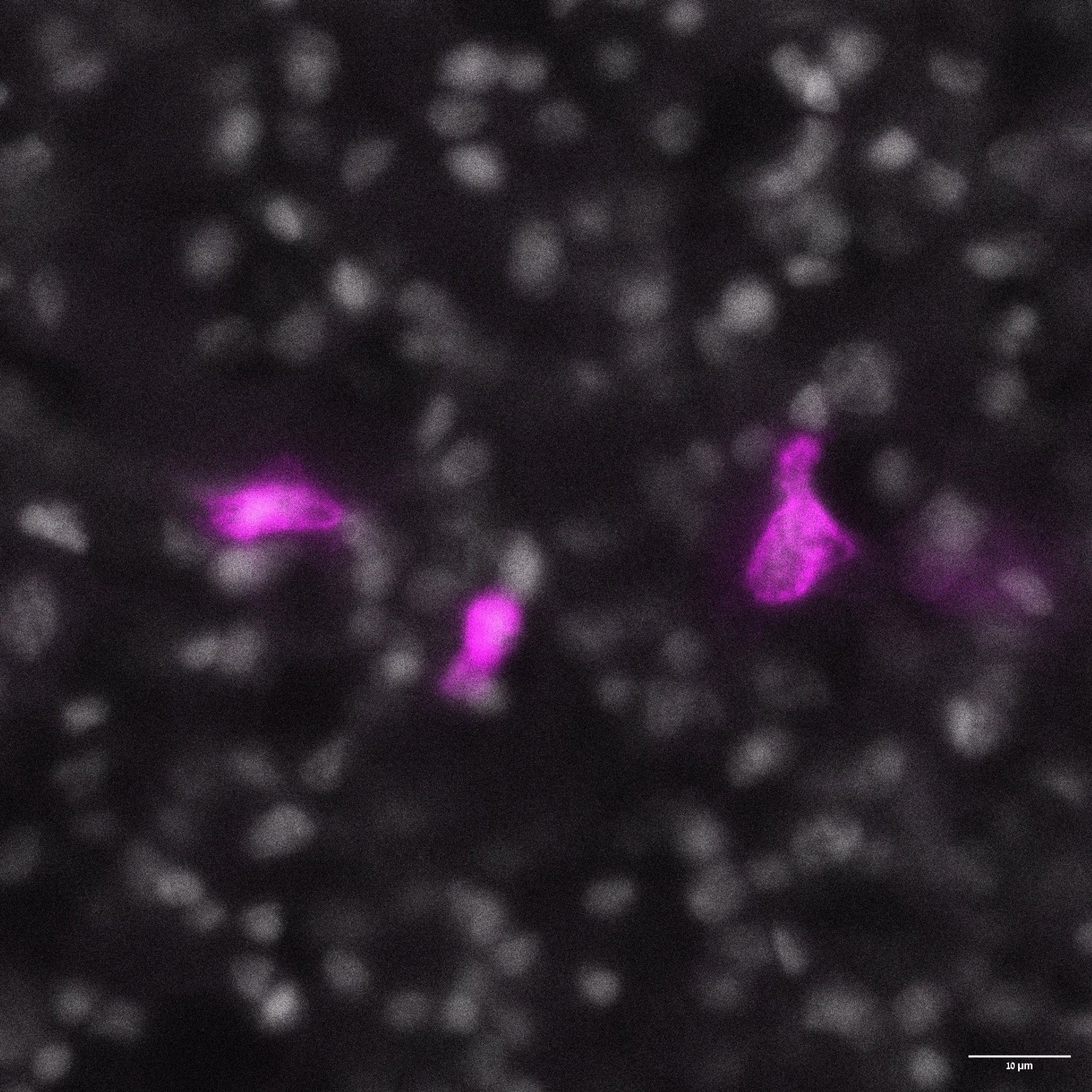

Supplement: Supplementary file 14 — Source data Fig. 7 [file 44318_2025_662_MOESM14_ESM.zip › Figure 7/7B/Main_figure_panel_Triple_RNAi_H3P_Merged.jpg]

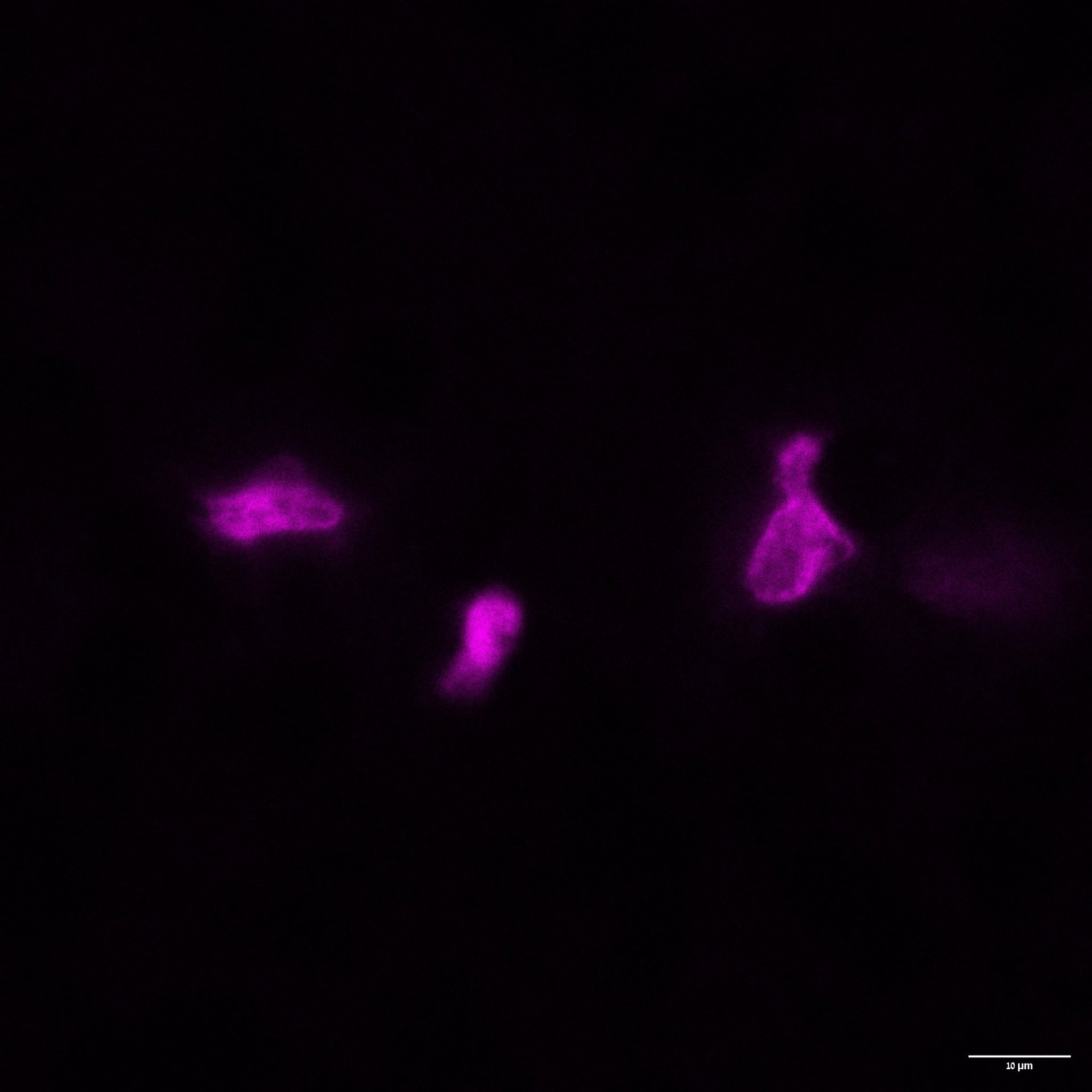

Supplement: Supplementary file 14 — Source data Fig. 7 [file 44318_2025_662_MOESM14_ESM.zip › Figure 7/7B/Main_figure_panel_Triple_RNAi_H3P_Rhod.jpg]

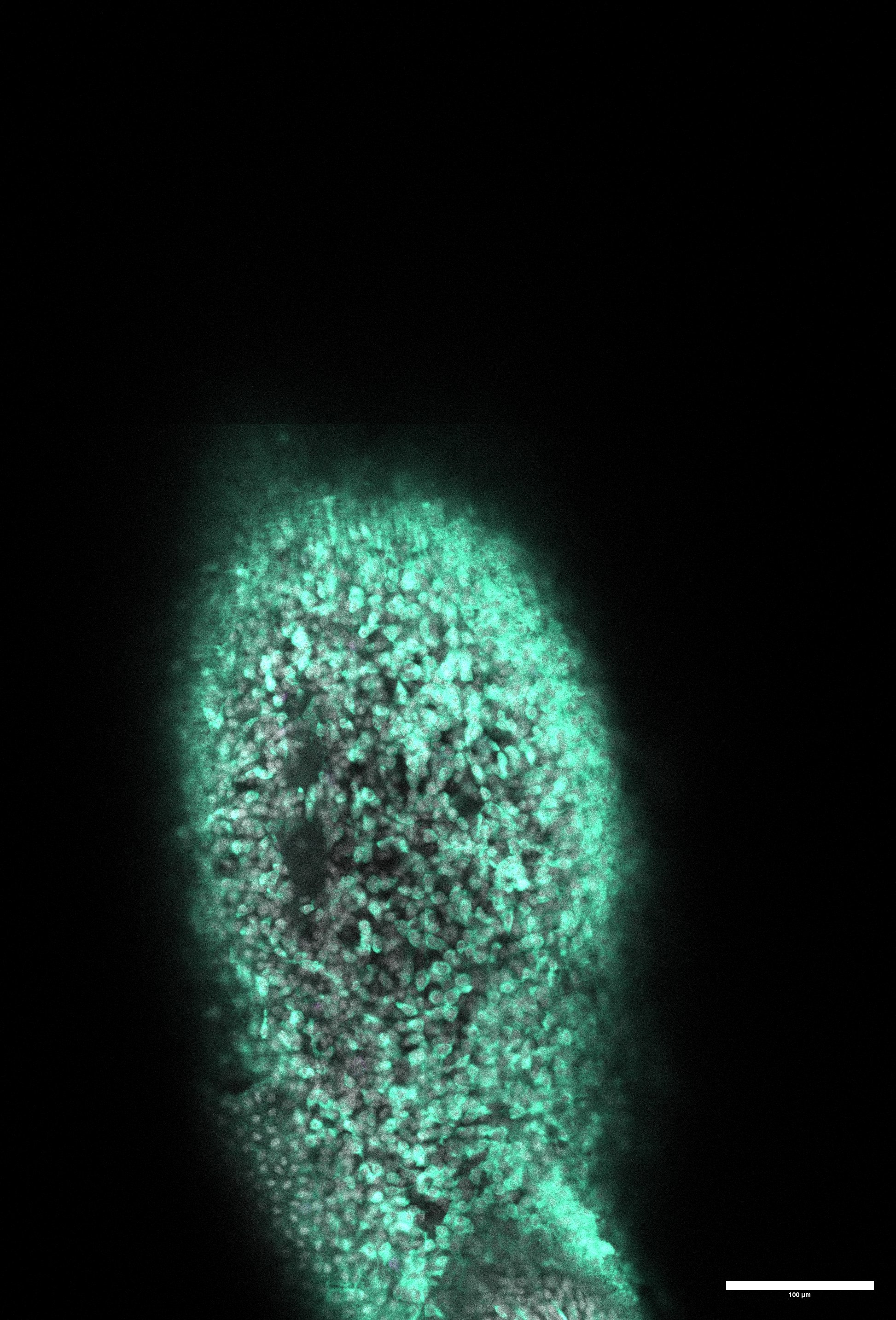

Supplement: Supplementary file 14 — Source data Fig. 7 [file 44318_2025_662_MOESM14_ESM.zip › Figure 7/7C/Representative_plane_1_Control_RNAi_probe_SMEDWI_FITC_DAPI_20x_z1.jpg]

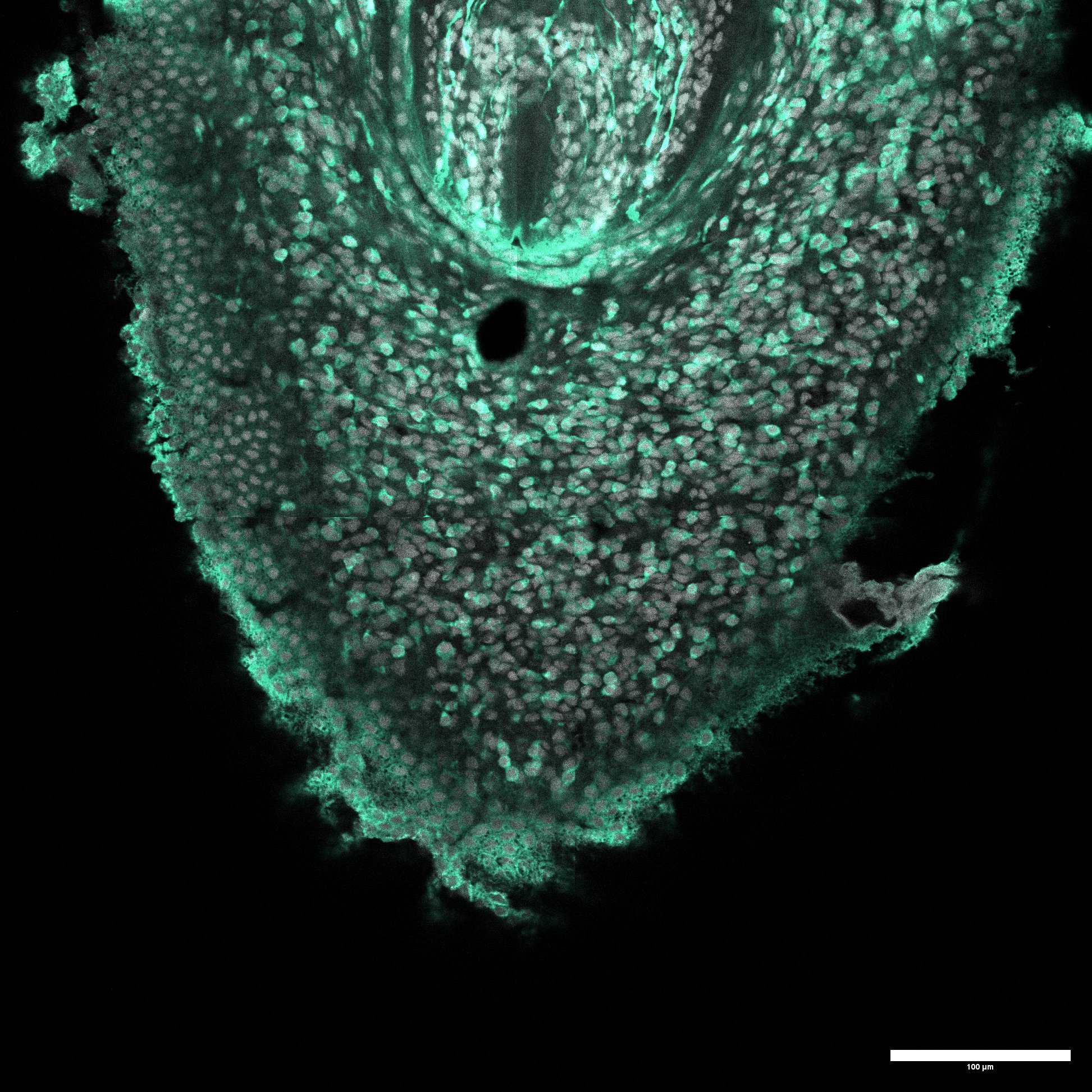

Supplement: Supplementary file 14 — Source data Fig. 7 [file 44318_2025_662_MOESM14_ESM.zip › Figure 7/7C/Representative_plane_1_Triple_RNAi_probe_SMEDWI_FITC_DAPI_20x_z1.jpg]

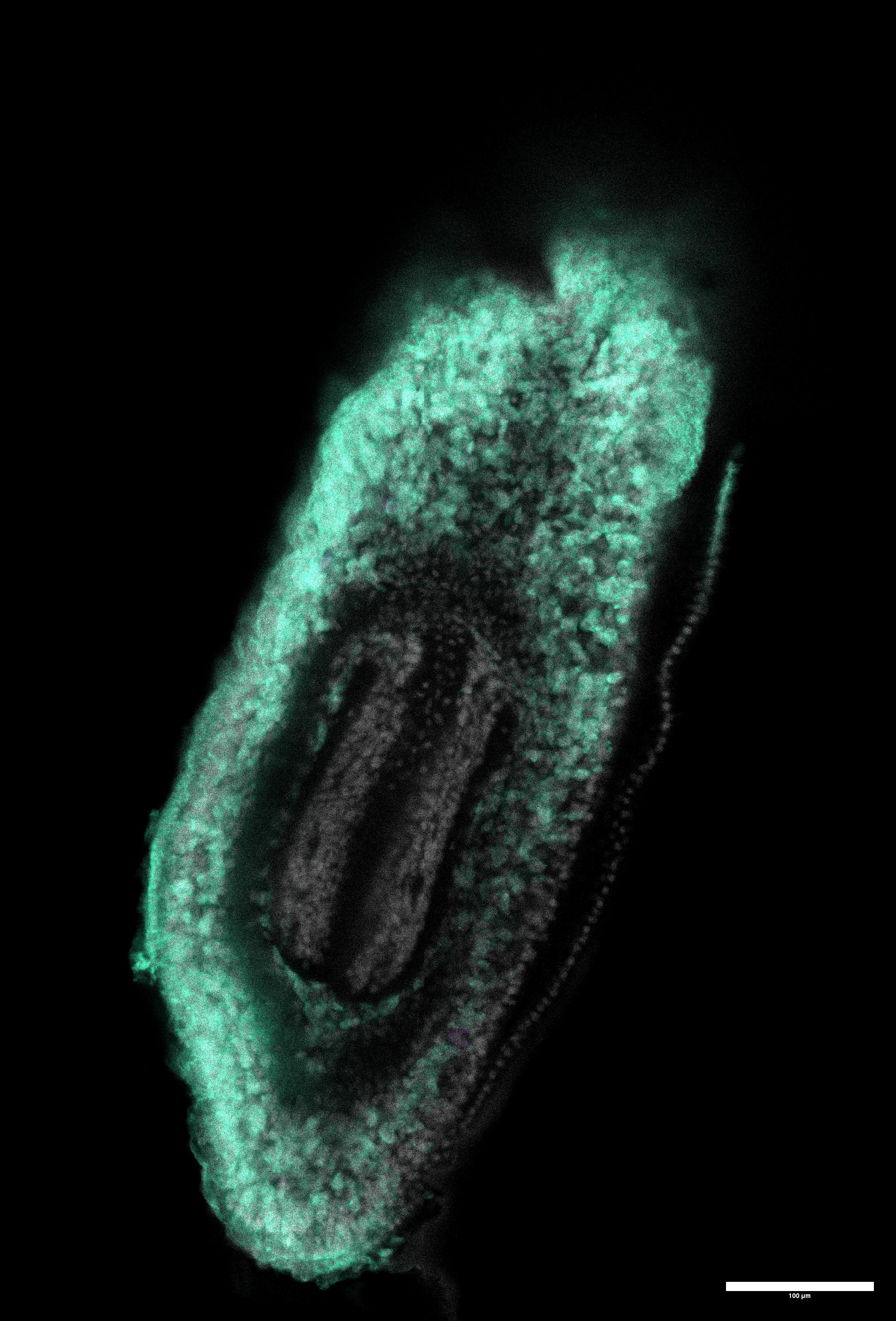

Supplement: Supplementary file 14 — Source data Fig. 7 [file 44318_2025_662_MOESM14_ESM.zip › Figure 7/7C/Representative_plane_2_Control_RNAi_probe_SMEDWI_FITC_DAPI_20x_z1.jpg]

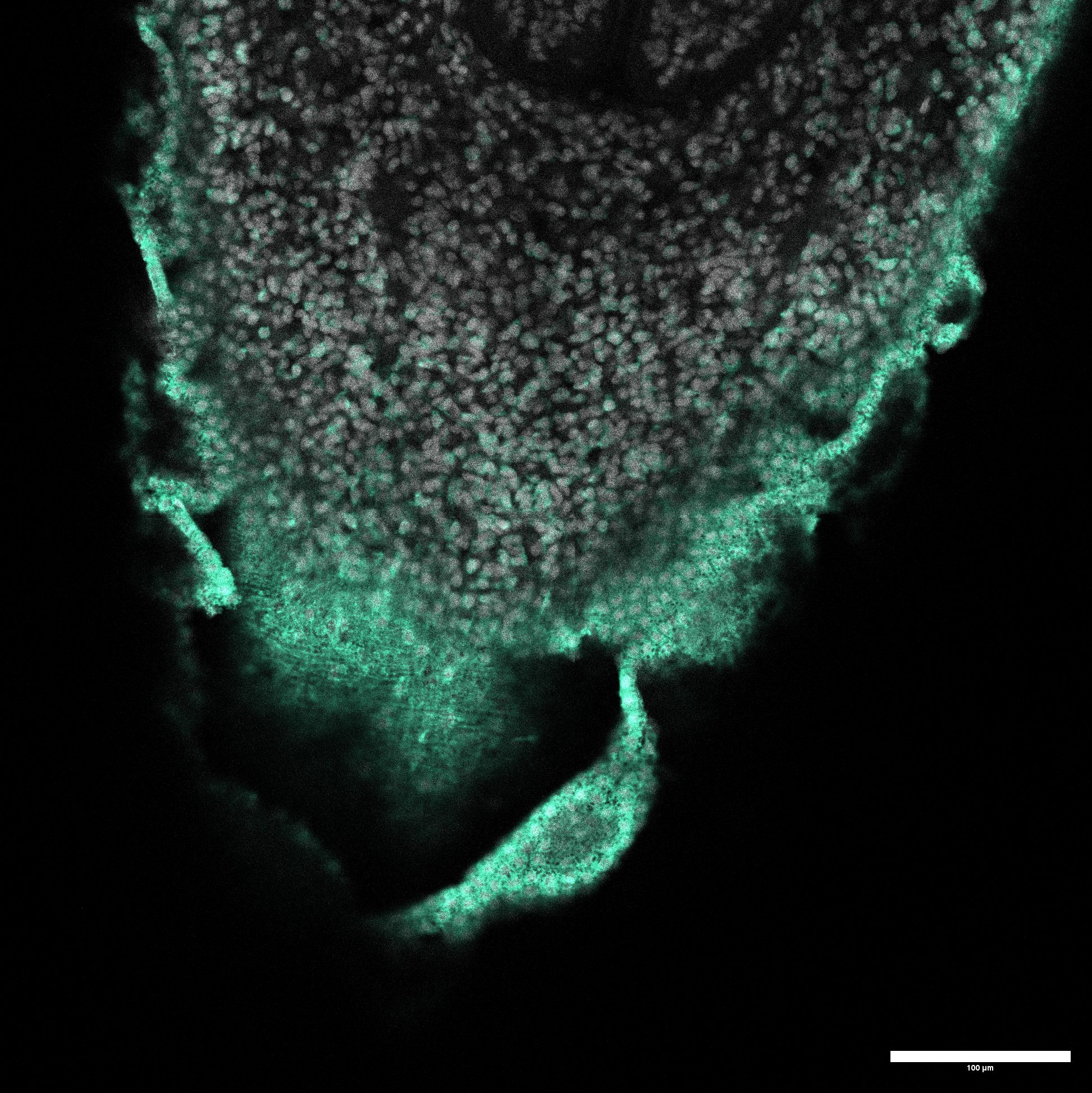

Supplement: Supplementary file 14 — Source data Fig. 7 [file 44318_2025_662_MOESM14_ESM.zip › Figure 7/7C/Representative_plane_2_Triple_RNAi_probe_SMEDWI_FITC_DAPI_20x_z1.jpg]

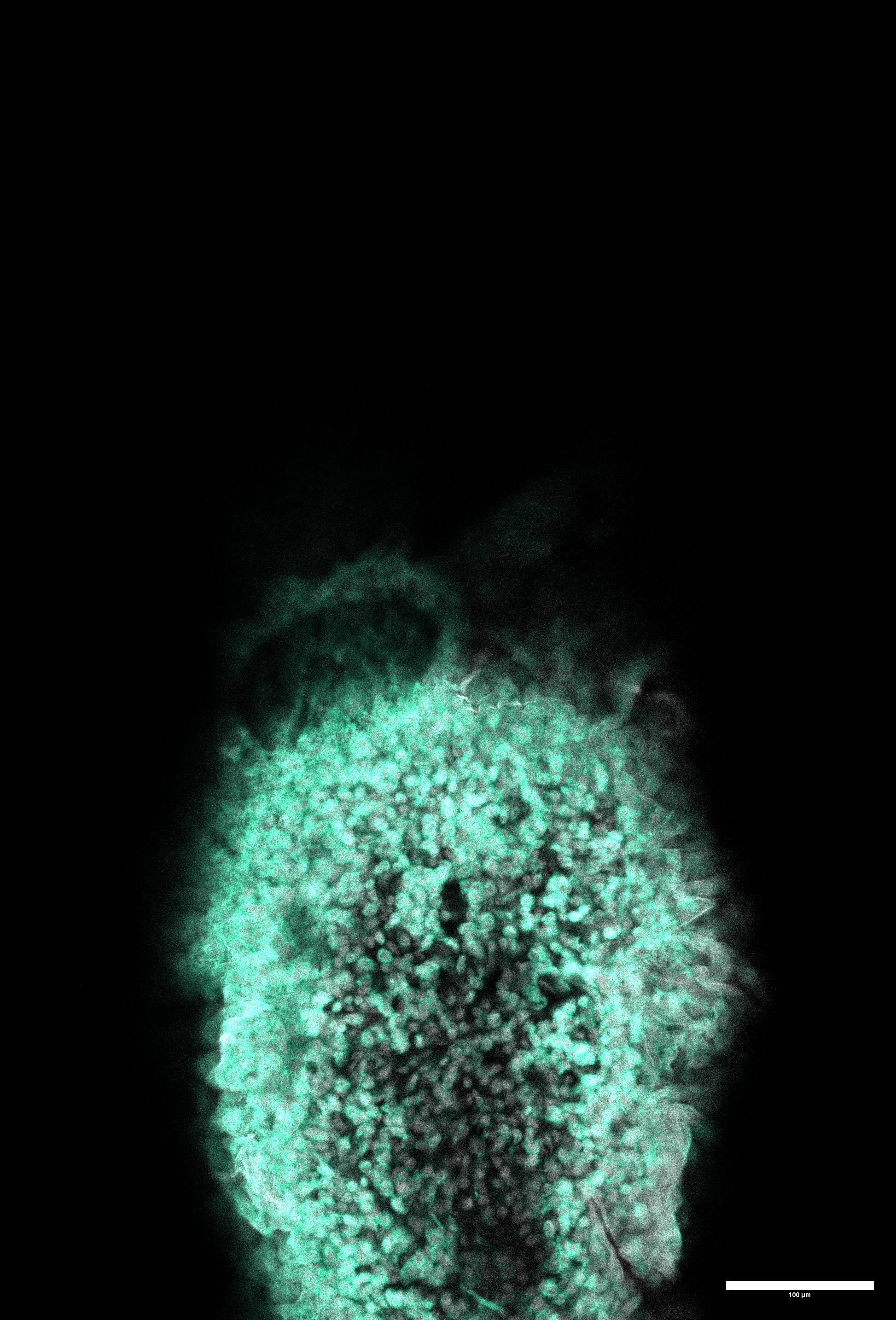

Supplement: Supplementary file 14 — Source data Fig. 7 [file 44318_2025_662_MOESM14_ESM.zip › Figure 7/7C/Representative_plane_3_Control_RNAi_probe_SMEDWI_FITC_DAPI_20x_z1.jpg]

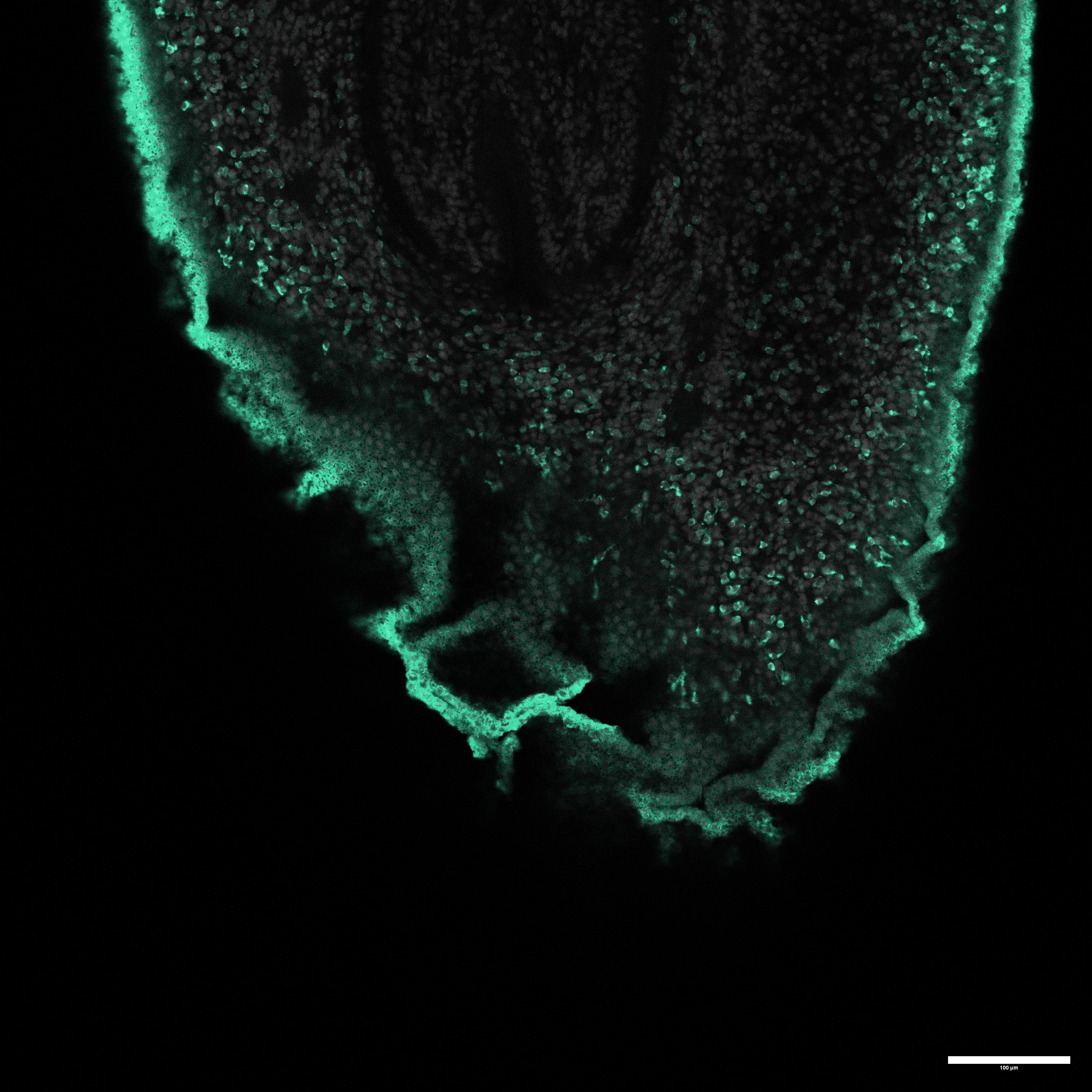

Supplement: Supplementary file 14 — Source data Fig. 7 [file 44318_2025_662_MOESM14_ESM.zip › Figure 7/7C/Representative_plane_3_Triple_RNAi_probe_SMEDWI_FITC_DAPI_20x_z1.jpg]

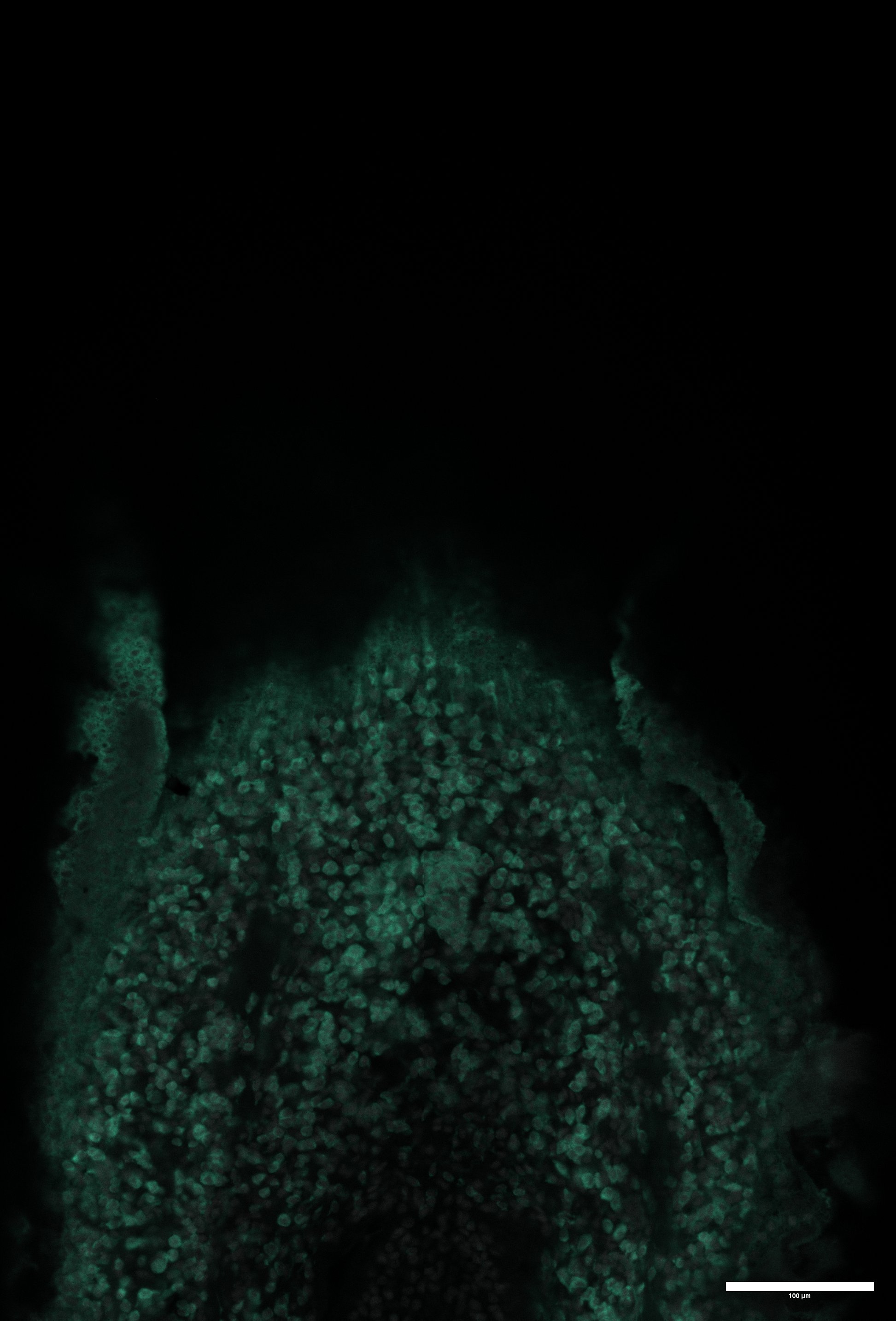

Supplement: Supplementary file 14 — Source data Fig. 7 [file 44318_2025_662_MOESM14_ESM.zip › Figure 7/7C/Representative_plane_4_Control_RNAi_probe_SMEDWI_FITC_DAPI_20x_z1.jpg]

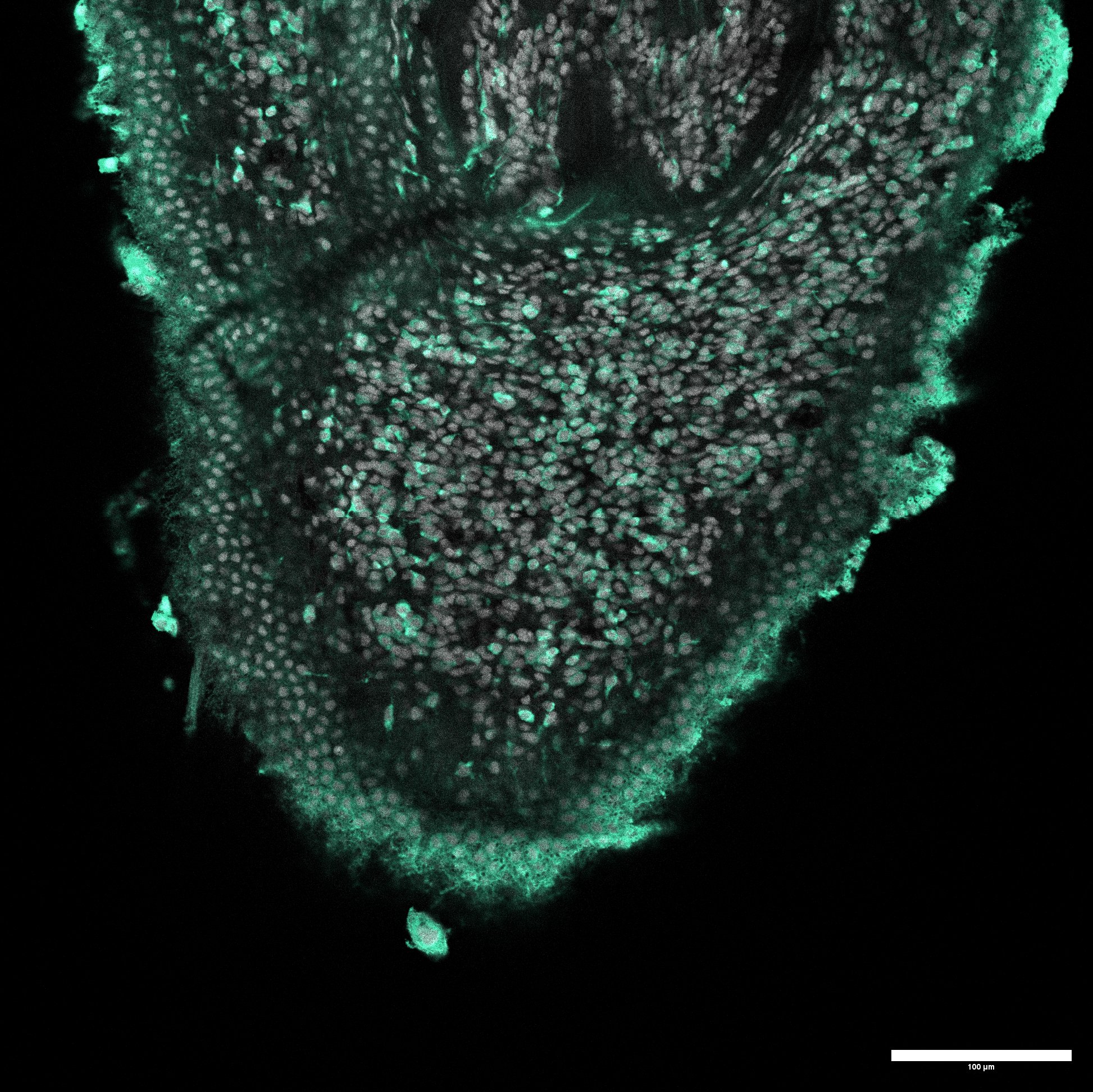

Supplement: Supplementary file 14 — Source data Fig. 7 [file 44318_2025_662_MOESM14_ESM.zip › Figure 7/7C/Representative_plane_4_Triple_RNAi_probe_SMEDWI_FITC_DAPI_20x_z1.jpg]

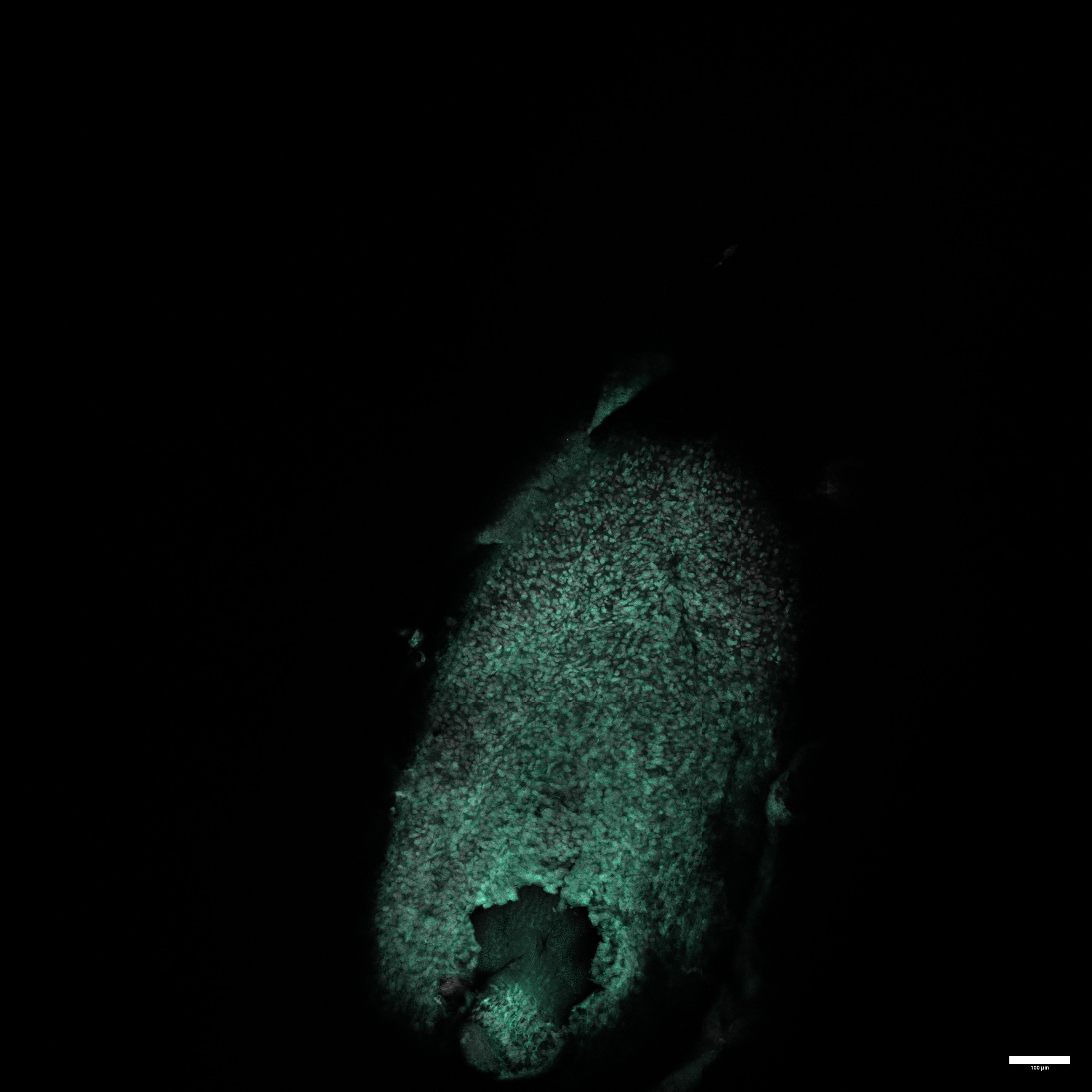

Supplement: Supplementary file 14 — Source data Fig. 7 [file 44318_2025_662_MOESM14_ESM.zip › Figure 7/7C/Representative_plane_5_Control_RNAi_probe_SMEDWI_FITC_DAPI_20x_z1.jpg]

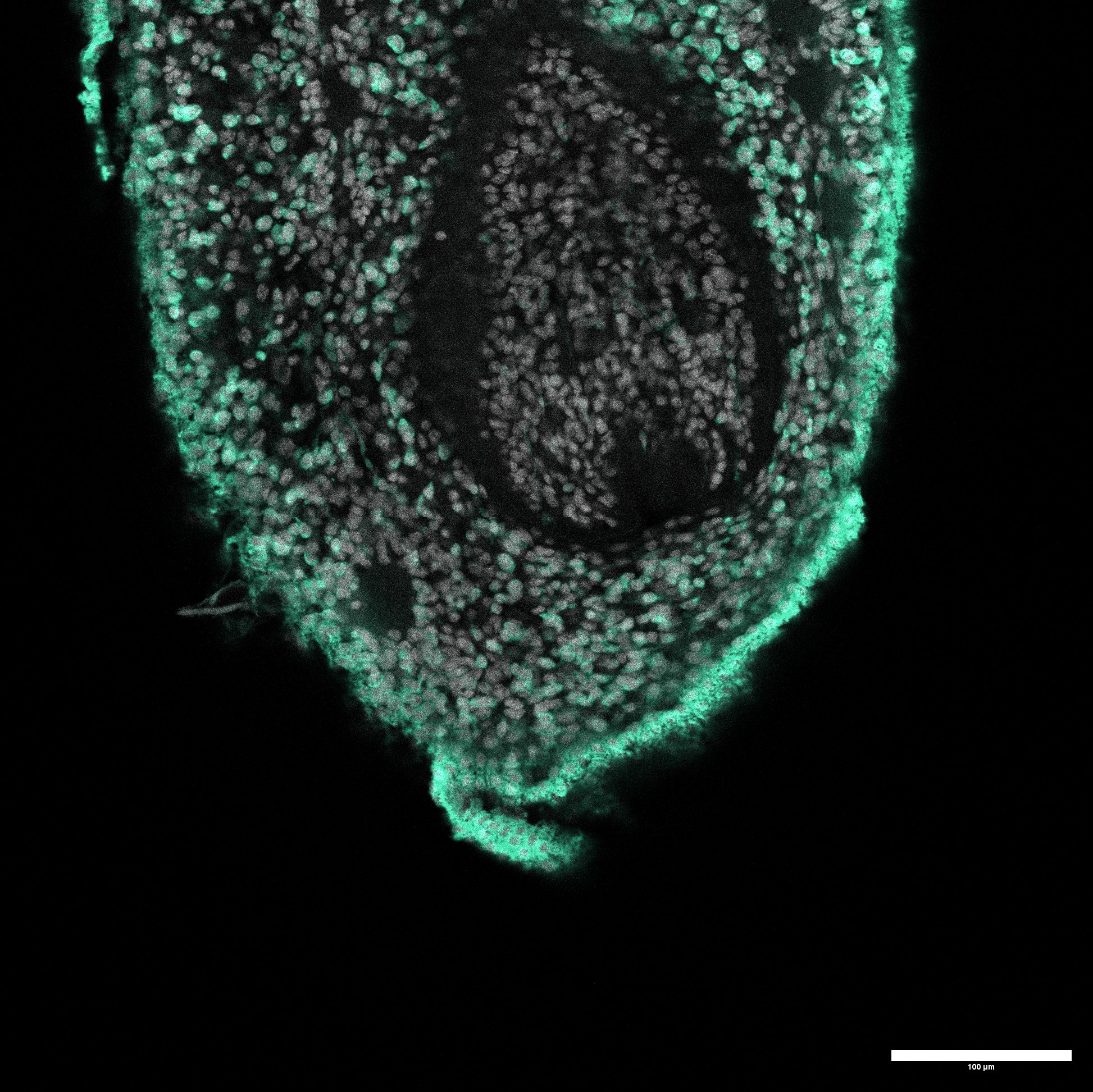

Supplement: Supplementary file 14 — Source data Fig. 7 [file 44318_2025_662_MOESM14_ESM.zip › Figure 7/7C/Representative_plane_5_Triple_RNAi_probe_SMEDWI_FITC_DAPI_20x_z1.jpg]

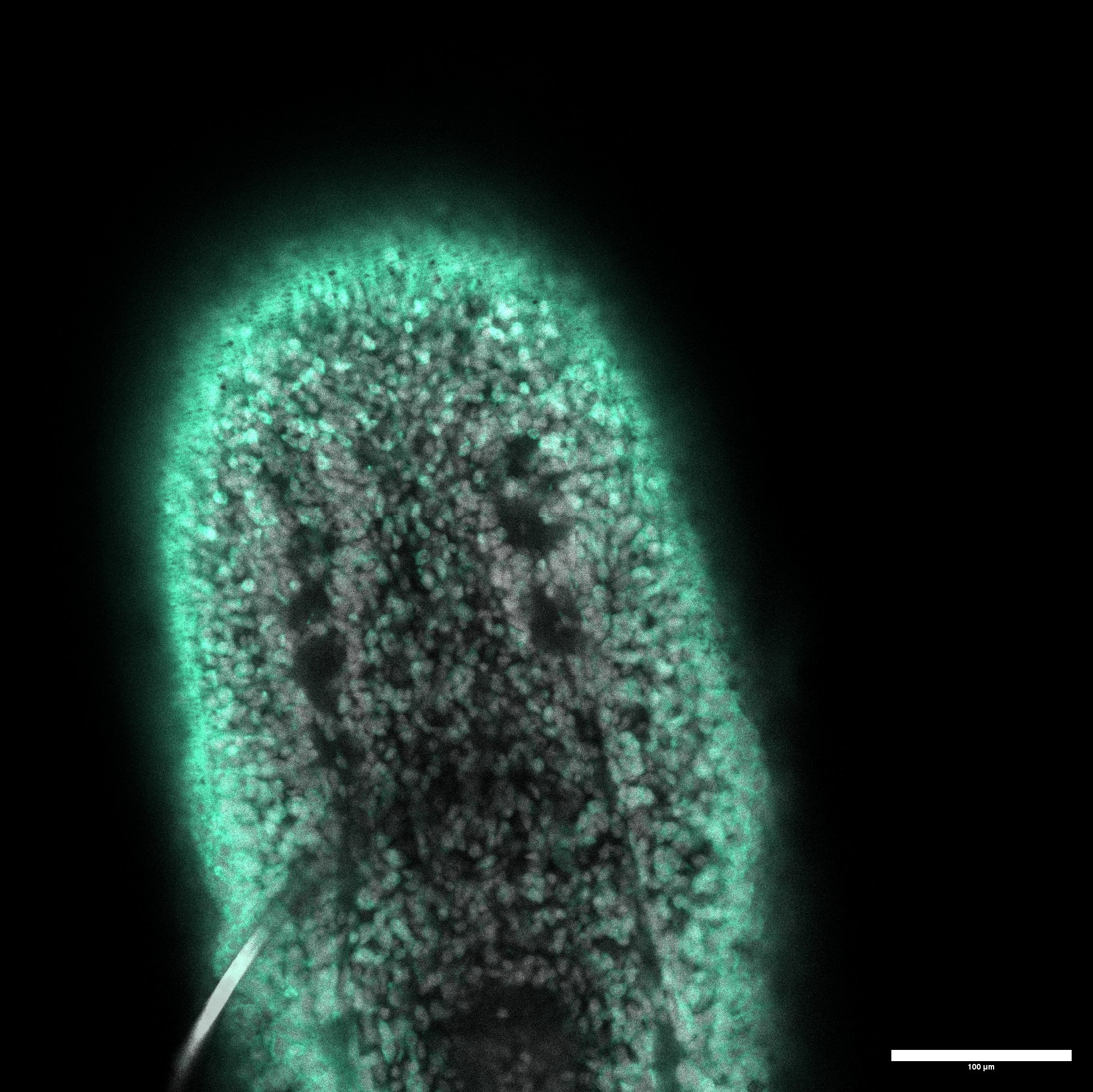

Supplement: Supplementary file 14 — Source data Fig. 7 [file 44318_2025_662_MOESM14_ESM.zip › Figure 7/7C/Representative_plane_6_Control_RNAi_probe_SMEDWI_FITC_DAPI_20x_z1.jpg]

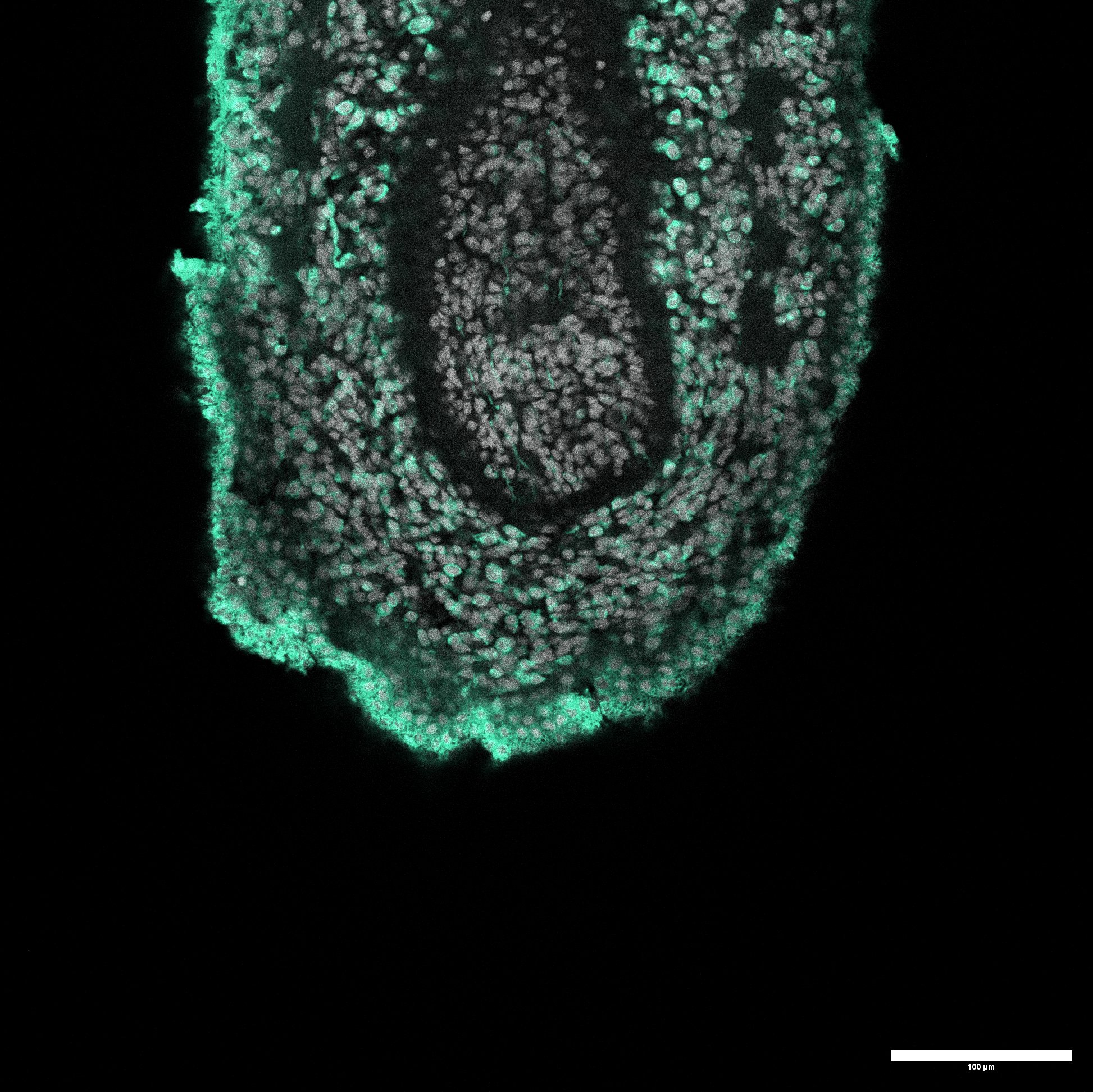

Supplement: Supplementary file 14 — Source data Fig. 7 [file 44318_2025_662_MOESM14_ESM.zip › Figure 7/7C/Representative_plane_6_Triple_RNAi_probe_SMEDWI_FITC_DAPI_20x_z1.jpg]

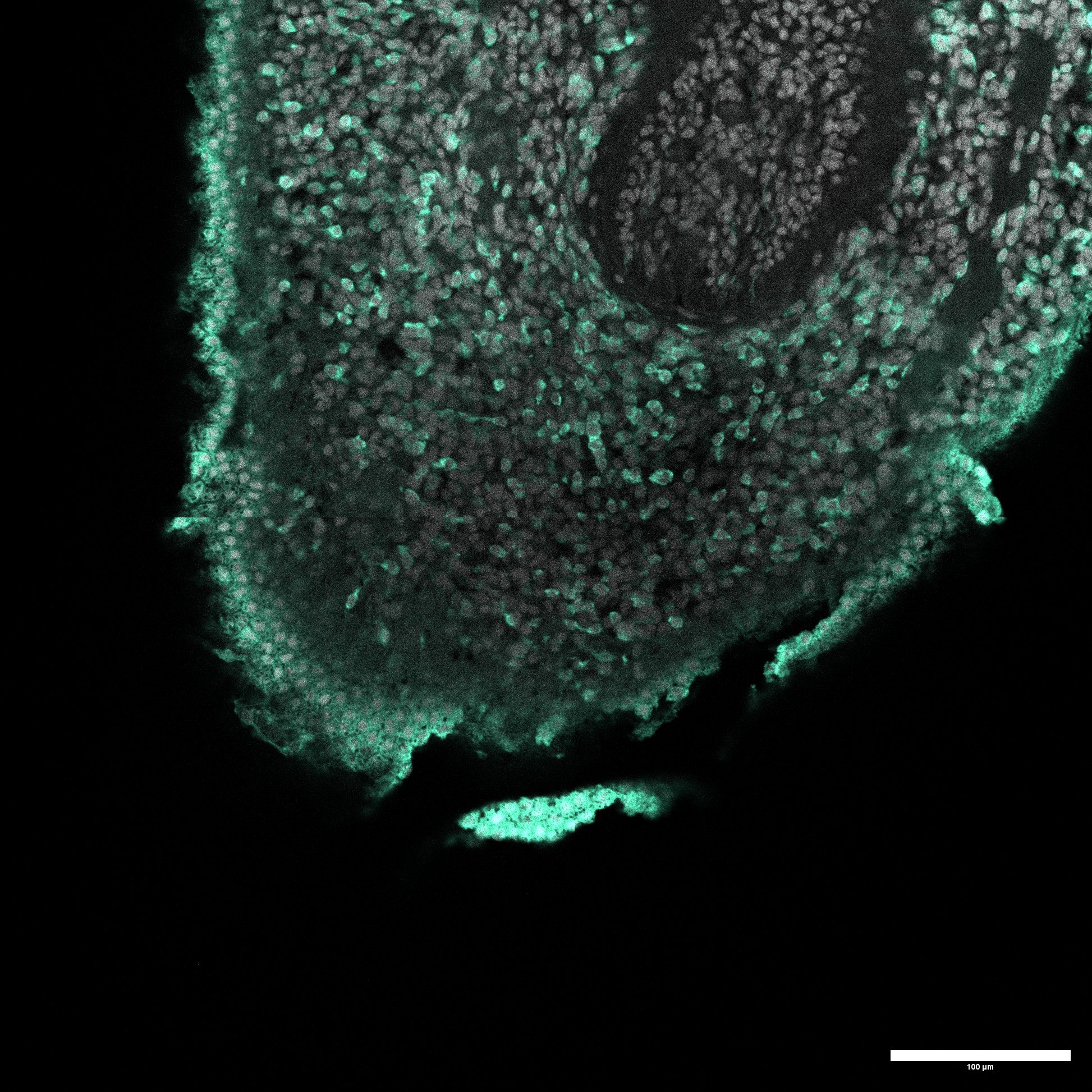

Supplement: Supplementary file 14 — Source data Fig. 7 [file 44318_2025_662_MOESM14_ESM.zip › Figure 7/7C/Representative_plane_7_Control_RNAi_probe_dd940_rhod_SMEDWI_FITC_DAPI_20x_z1.jpg]

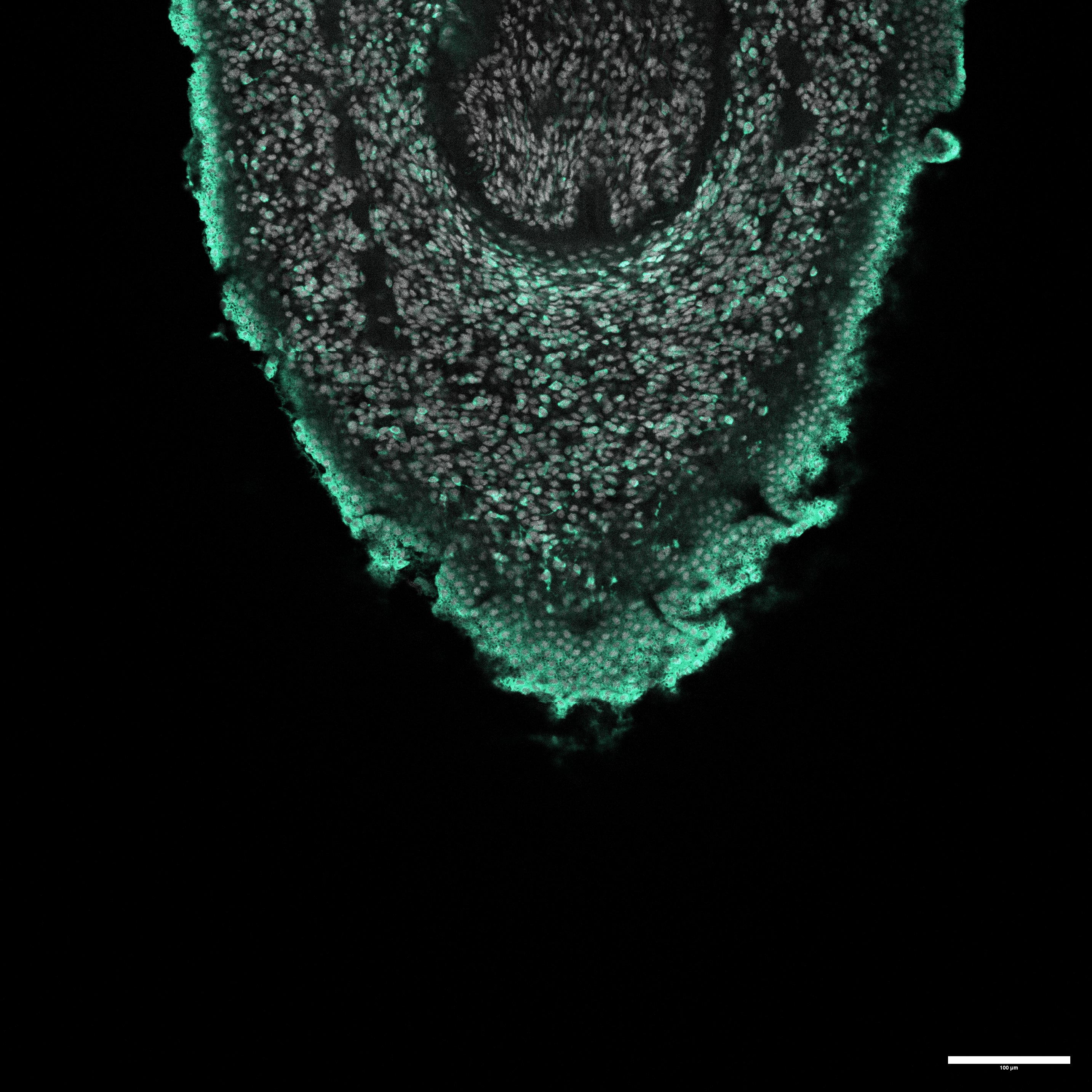

Supplement: Supplementary file 14 — Source data Fig. 7 [file 44318_2025_662_MOESM14_ESM.zip › Figure 7/7C/Representative_plane_7_Triple_RNAi_probe_SMEDWI_FITC_DAPI_20x_z1.jpg]

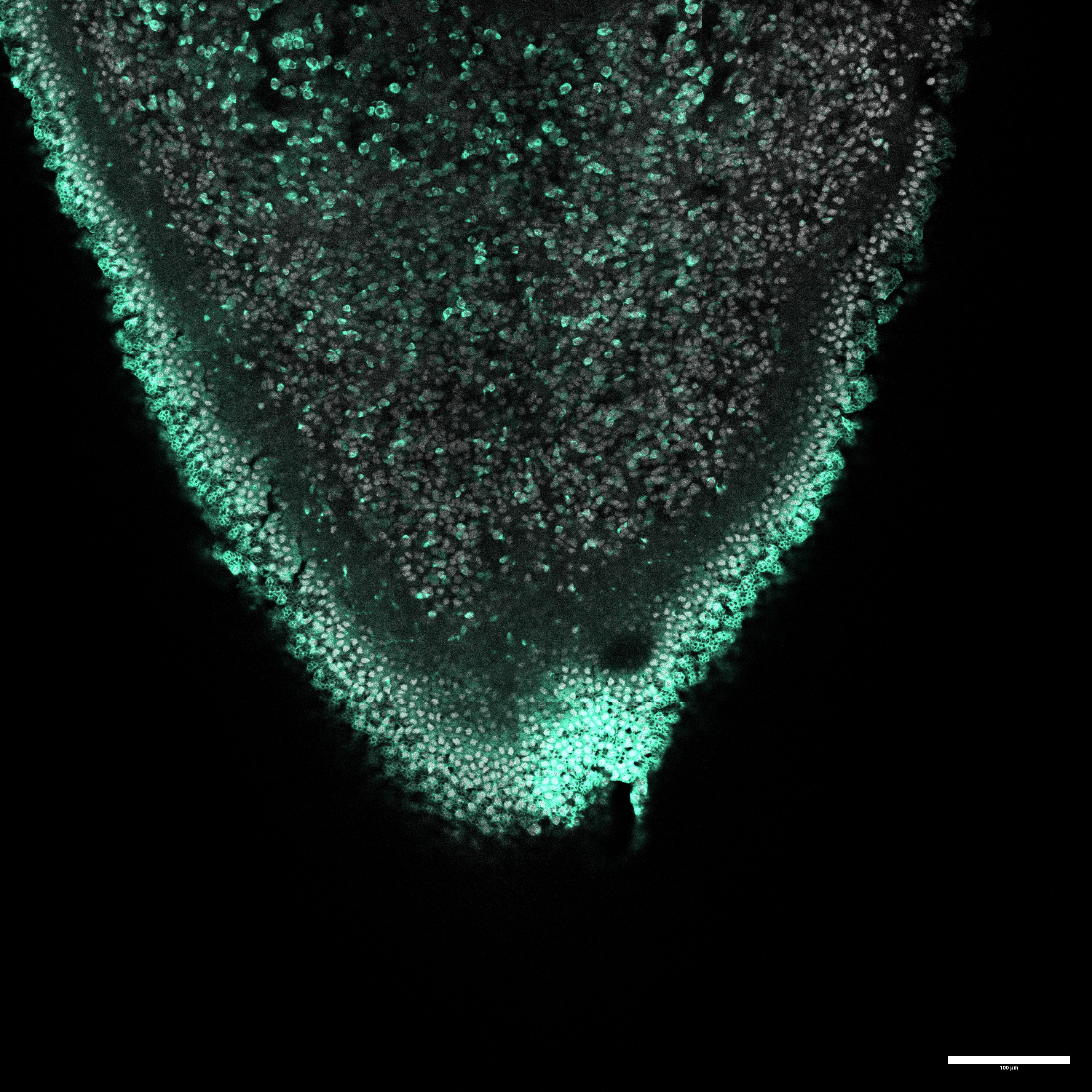

Supplement: Supplementary file 14 — Source data Fig. 7 [file 44318_2025_662_MOESM14_ESM.zip › Figure 7/7C/Representative_plane_8_Control_RNAi_probe_SMEDWI_FITC_DAPI_20x_z1.jpg]

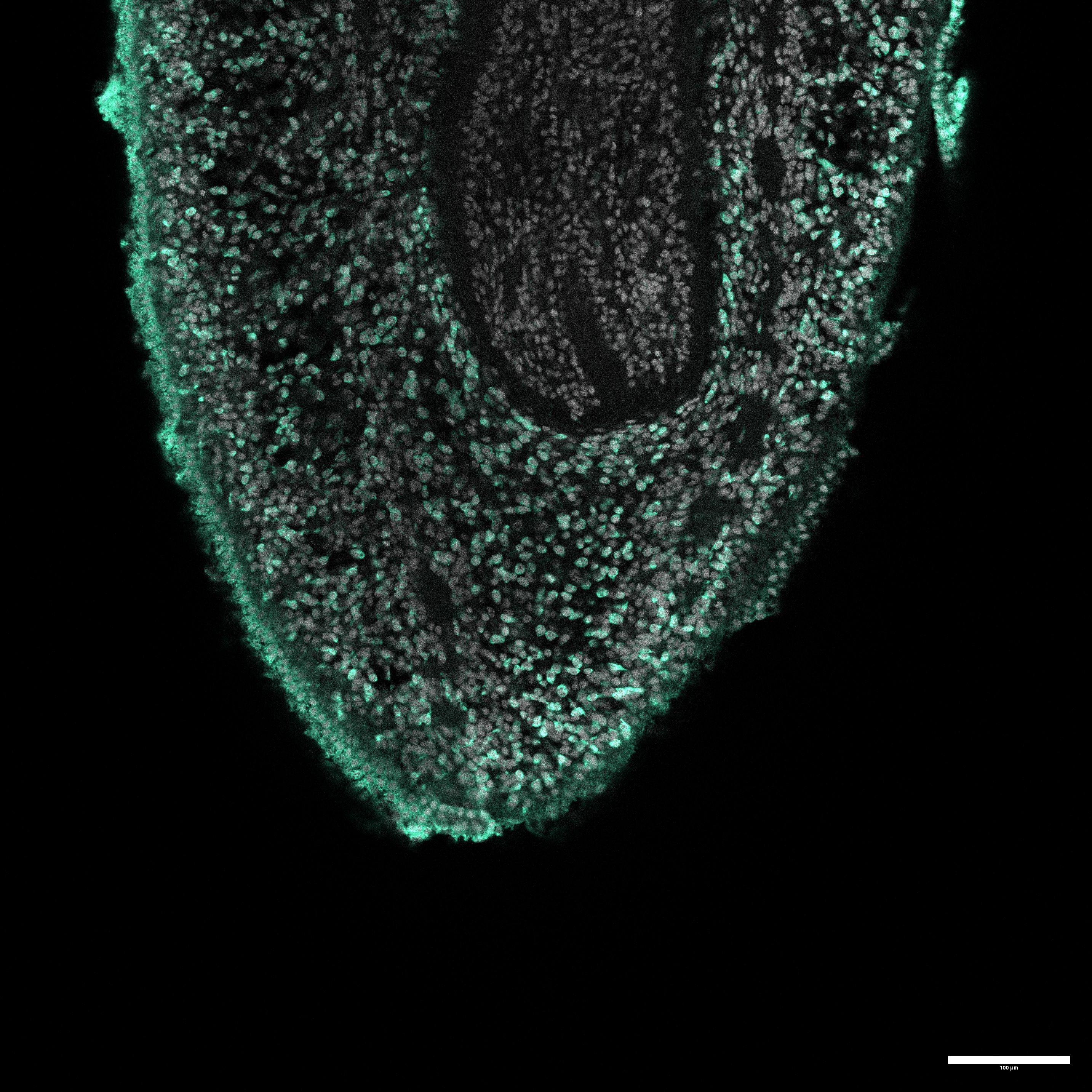

Supplement: Supplementary file 14 — Source data Fig. 7 [file 44318_2025_662_MOESM14_ESM.zip › Figure 7/7C/Representative_plane_9_Control_RNAi_probe_SMEDWI_FITC_DAPI_20x_z1.jpg]

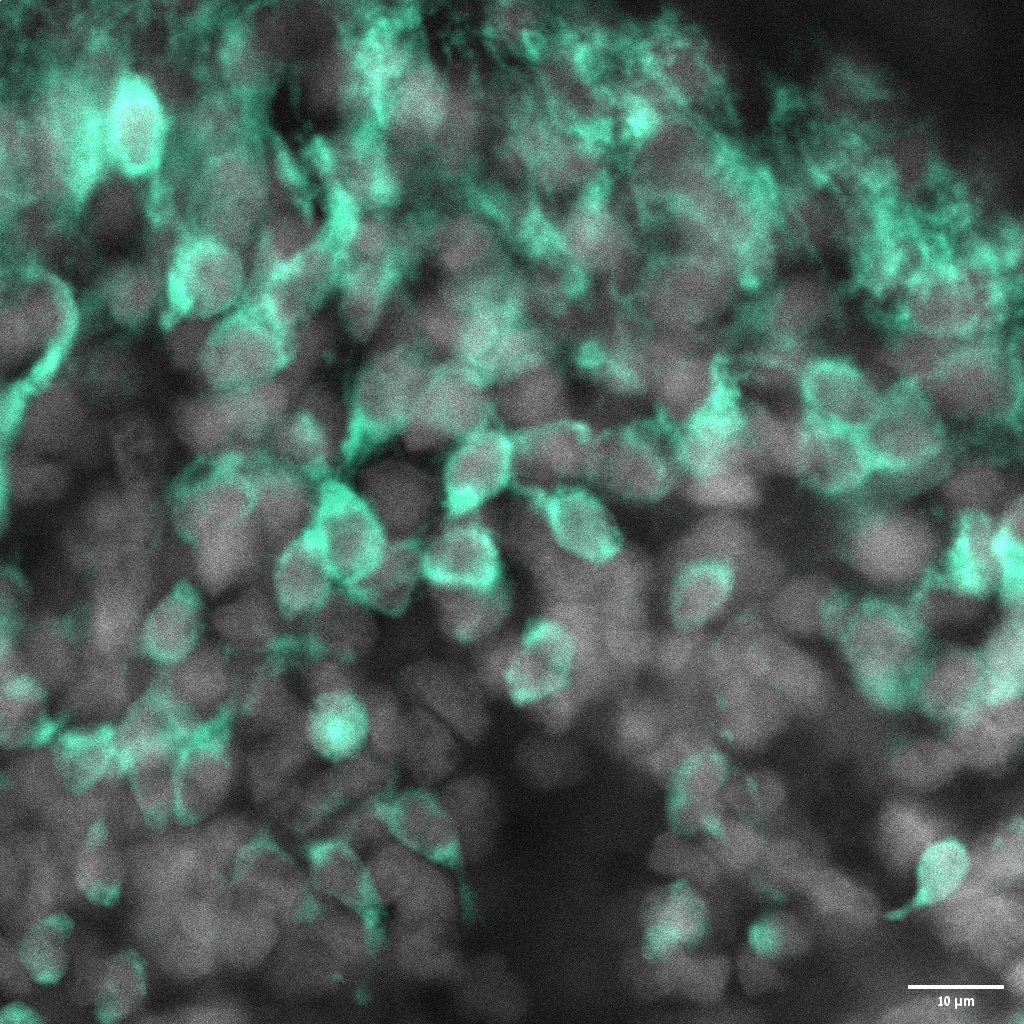

Supplement: Supplementary file 14 — Source data Fig. 7 [file 44318_2025_662_MOESM14_ESM.zip › Figure 7/7D/Main_figure_panel_Control_RNAi_Probe_dd1837_rhod_SMEDWI_FITC_DAPI_20x_FITC_channel.jpg]

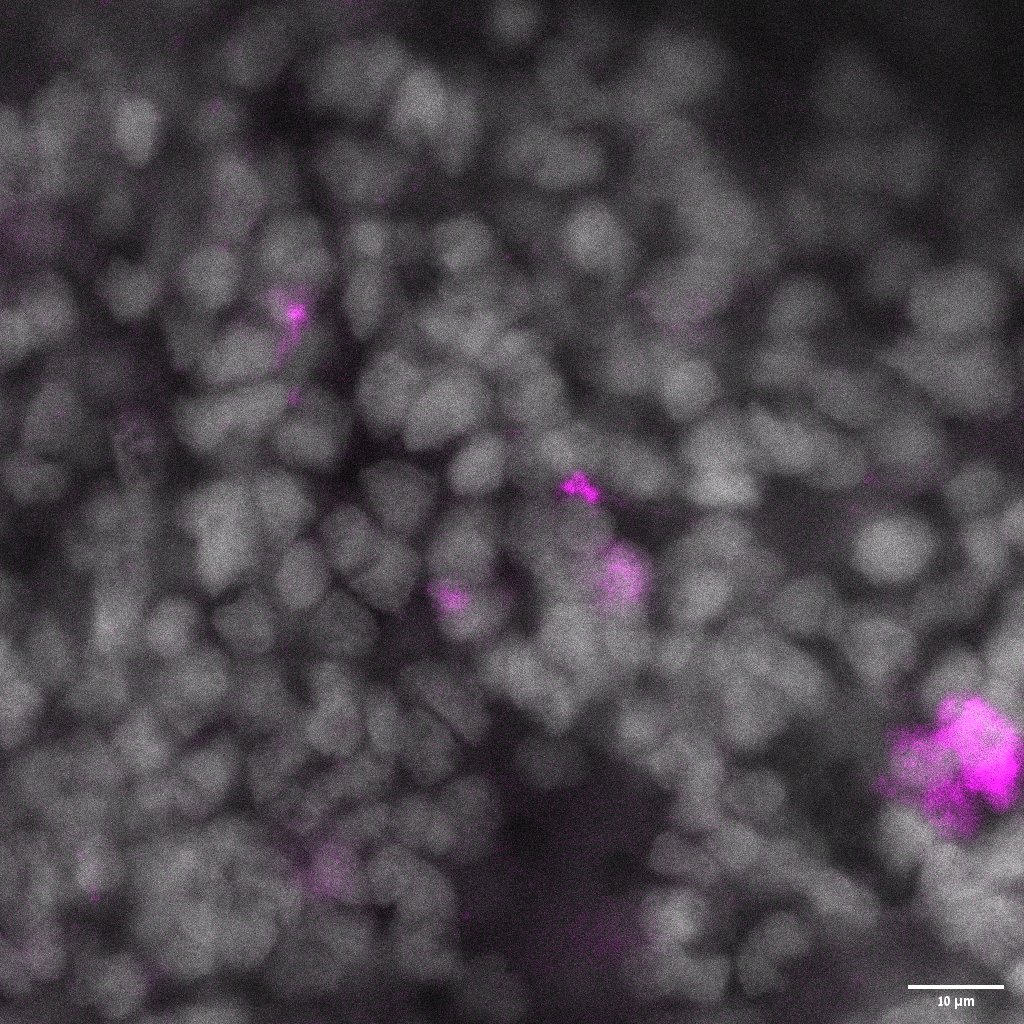

Supplement: Supplementary file 14 — Source data Fig. 7 [file 44318_2025_662_MOESM14_ESM.zip › Figure 7/7D/Main_figure_panel_Control_RNAi_Probe_dd1837_rhod_SMEDWI_FITC_DAPI_20x_Magenta_channel.jpg]

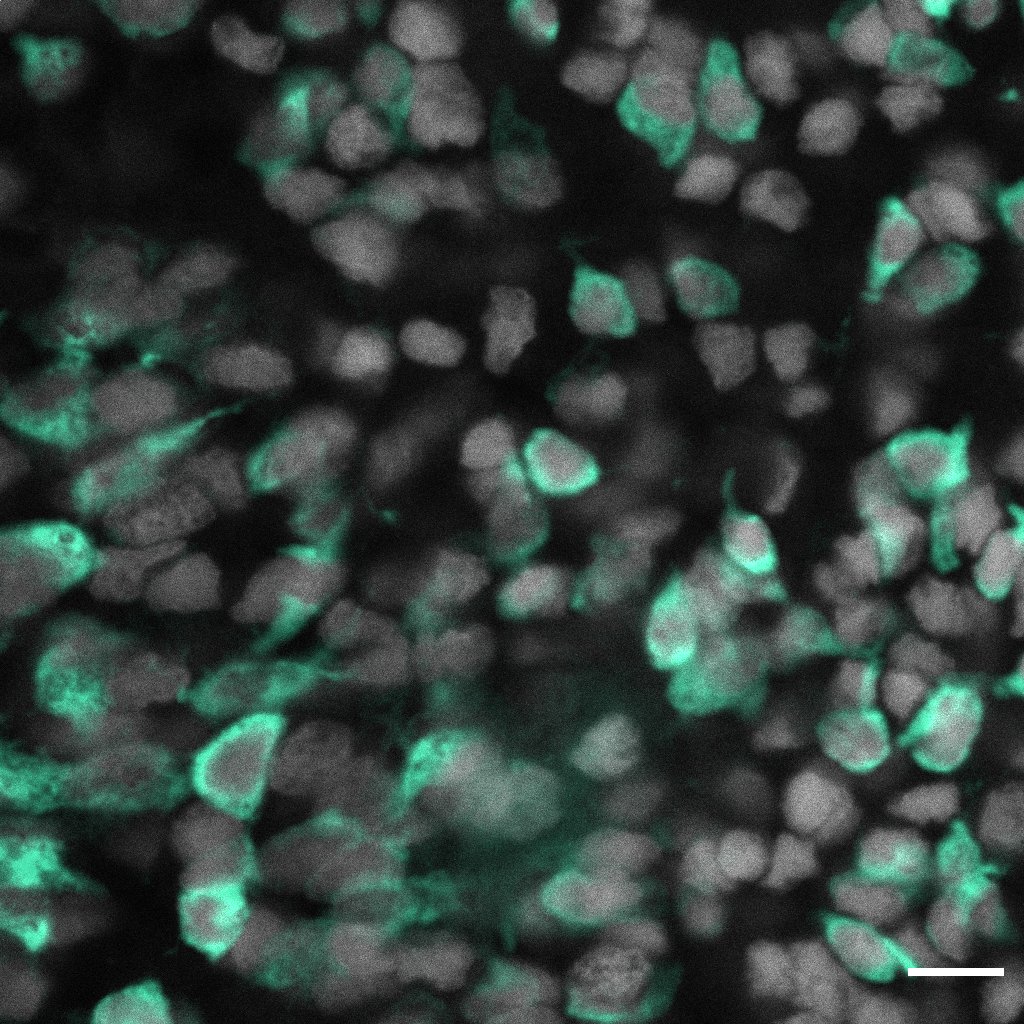

Supplement: Supplementary file 14 — Source data Fig. 7 [file 44318_2025_662_MOESM14_ESM.zip › Figure 7/7D/Main_figure_panel_Triple_RNAi_Probe_dd1837_rhod_SMEDWI_FITC_DAPI_20x_FITC_channel.jpg]

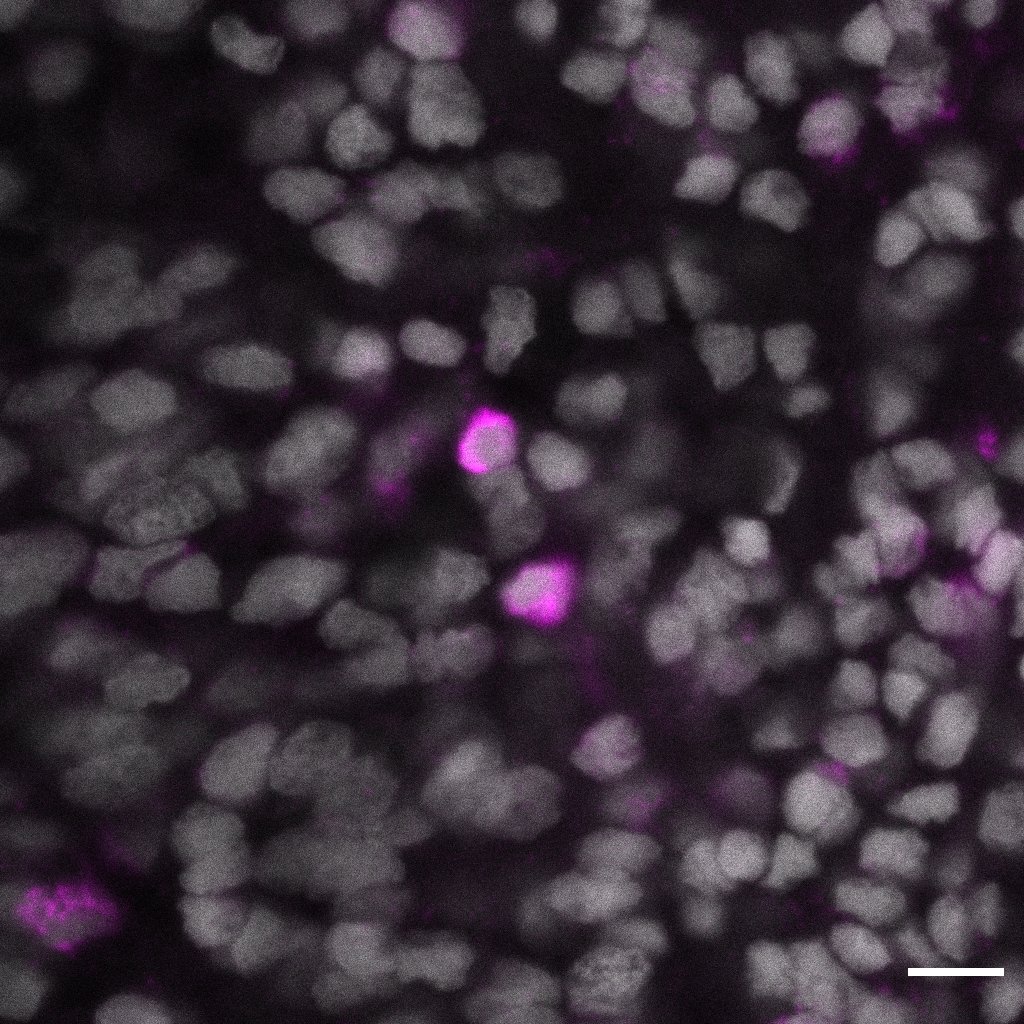

Supplement: Supplementary file 14 — Source data Fig. 7 [file 44318_2025_662_MOESM14_ESM.zip › Figure 7/7D/Main_figure_panel_Triple_RNAi_Probe_dd1837_rhod_SMEDWI_FITC_DAPI_20x_Magenta_channel.jpg]

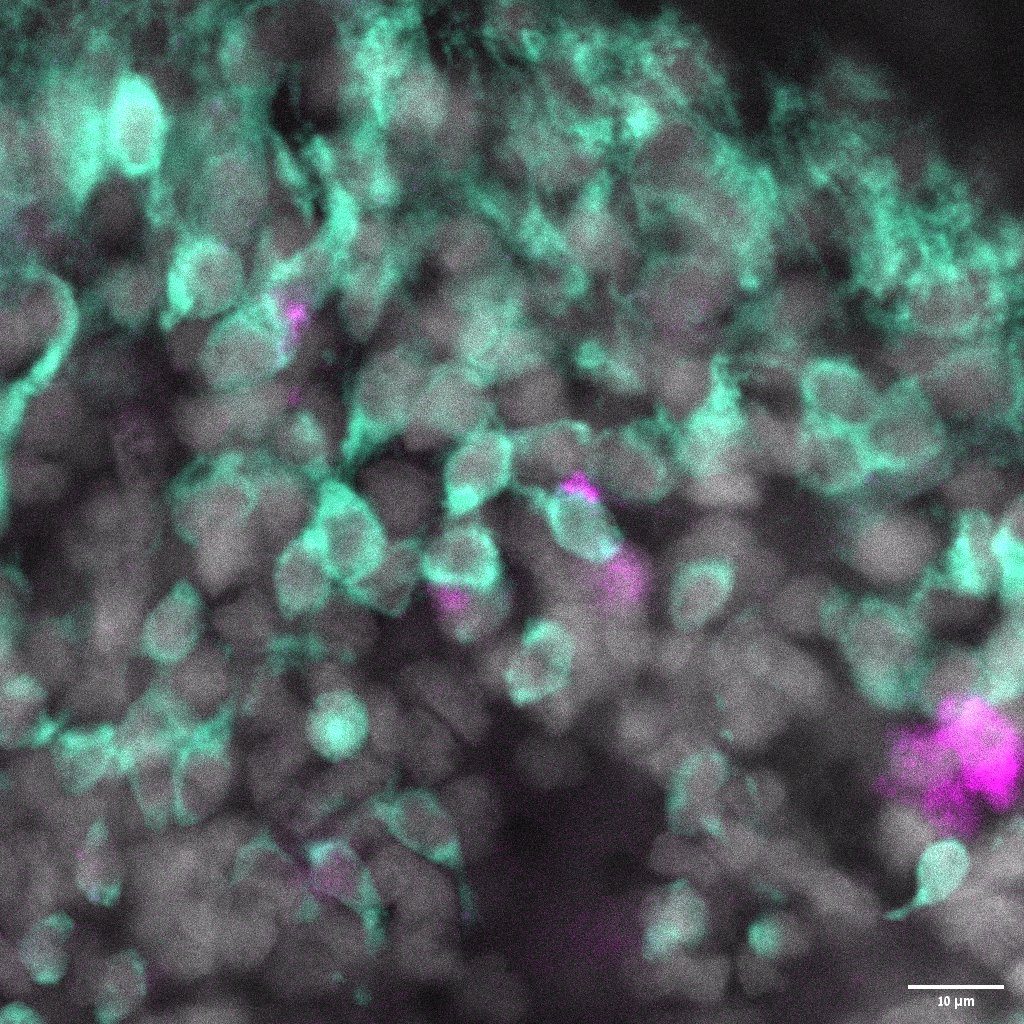

Supplement: Supplementary file 14 — Source data Fig. 7 [file 44318_2025_662_MOESM14_ESM.zip › Figure 7/7D/Main_figure_panel__Control_RNAi_Probe_dd1837_rhod_SMEDWI_FITC_DAPI_20x_Merged.jpg]

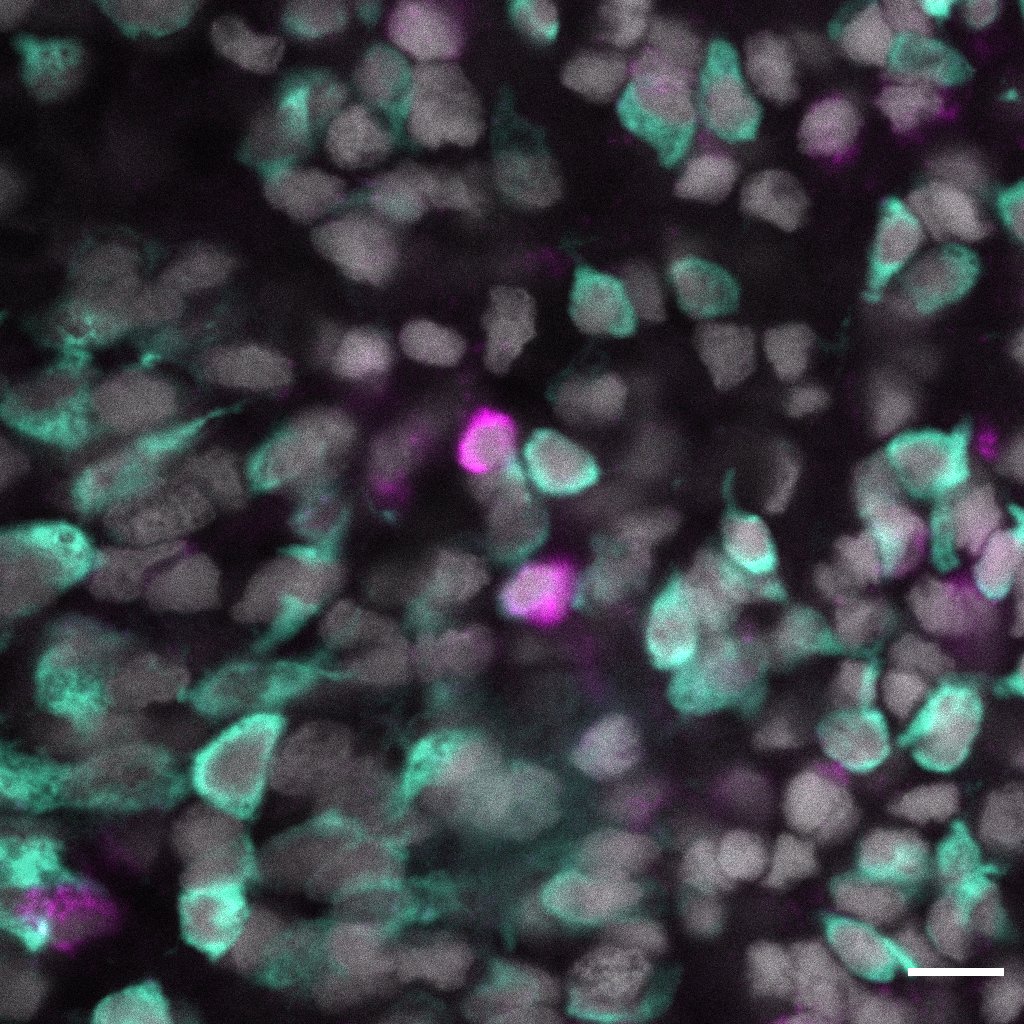

Supplement: Supplementary file 14 — Source data Fig. 7 [file 44318_2025_662_MOESM14_ESM.zip › Figure 7/7D/Main_figure_panel__Triple_RNAi_Probe_dd1837_rhod_SMEDWI_FITC_DAPI_20x_Merged.jpg]

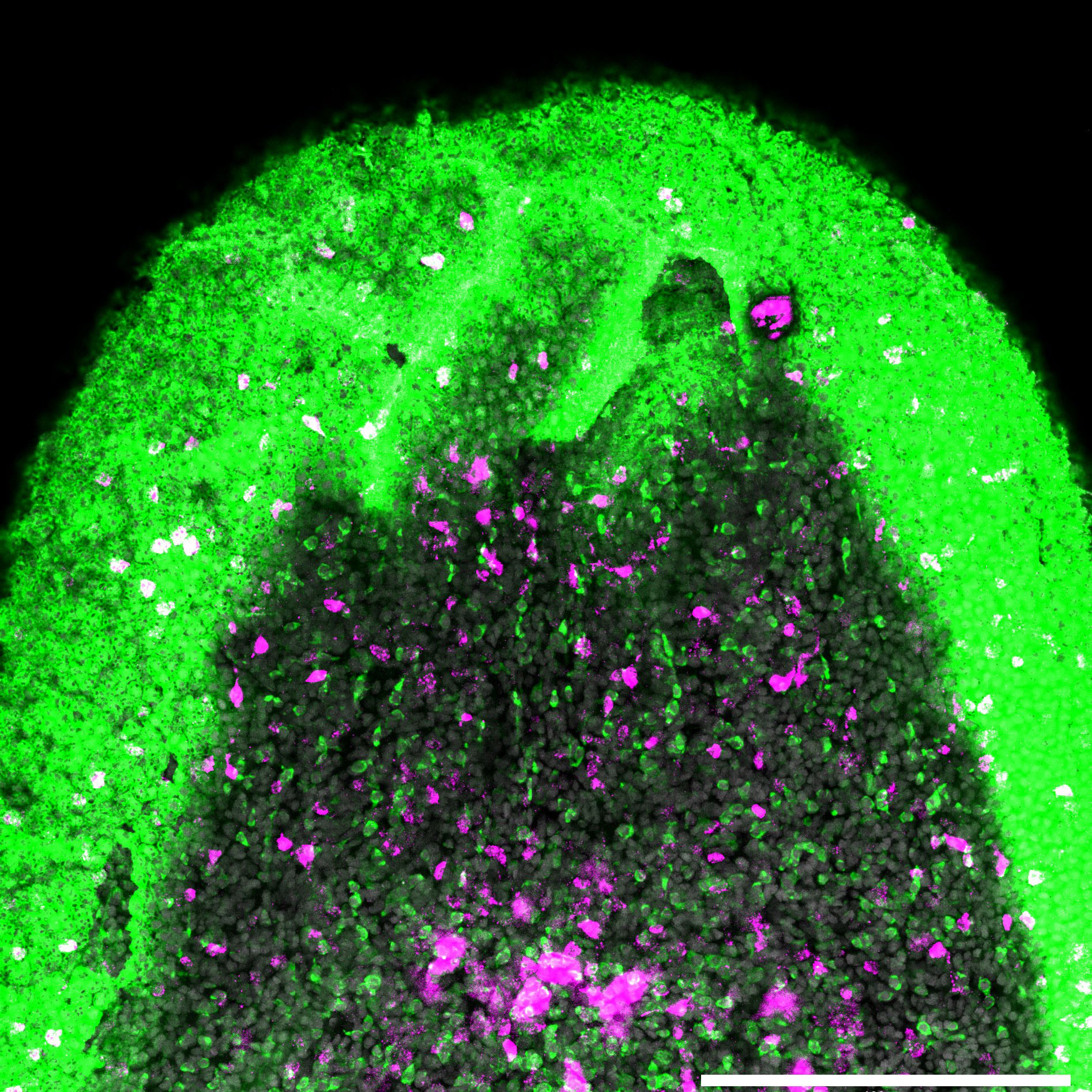

Supplement: Supplementary file 14 — Source data Fig. 7 [file 44318_2025_662_MOESM14_ESM.zip › Figure 7/7D/Max_projection_10_Triple_RNAi_probe_dd1837_rhod_SMEDWI_FITC_DAPI_20x_z2.jpg]

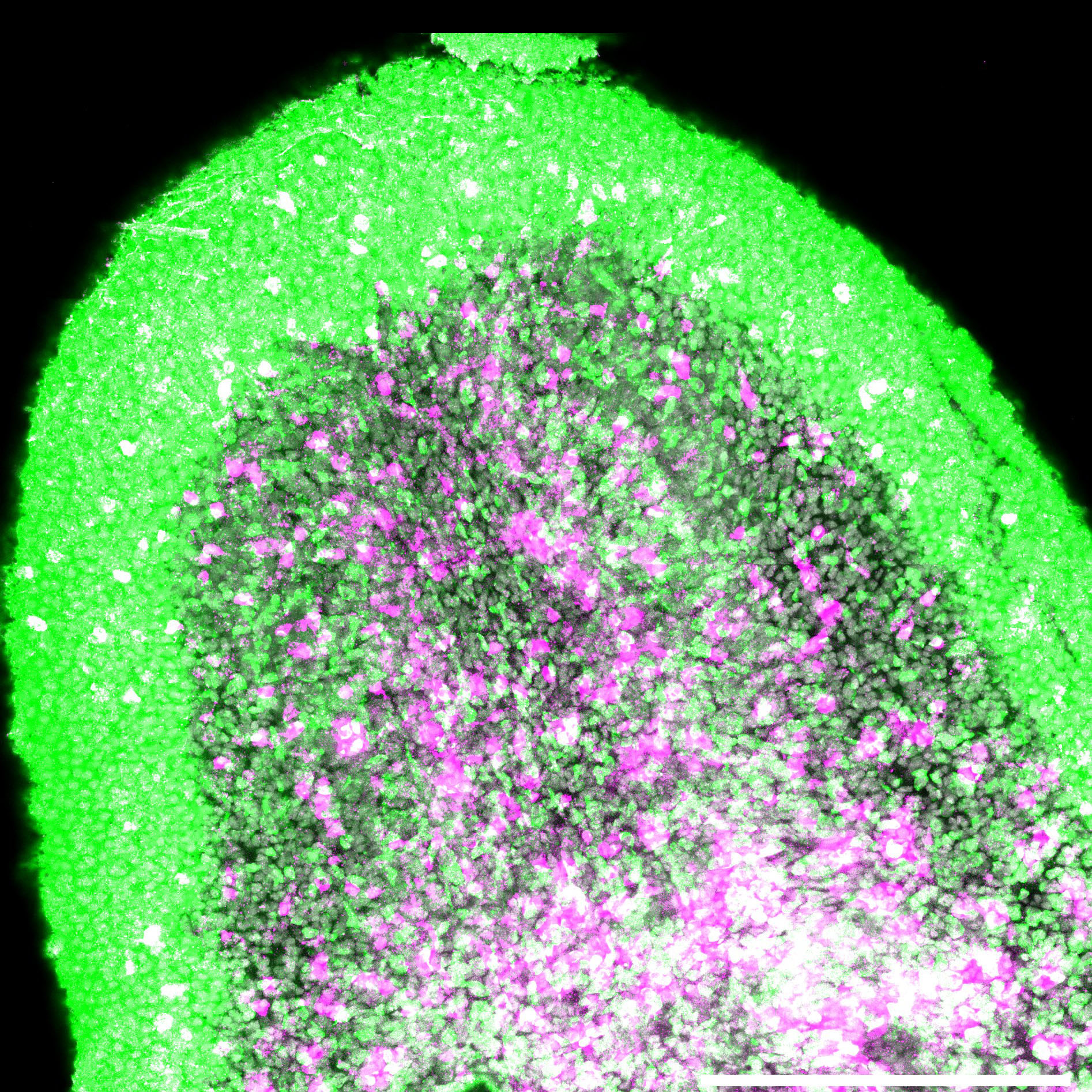

Supplement: Supplementary file 14 — Source data Fig. 7 [file 44318_2025_662_MOESM14_ESM.zip › Figure 7/7D/Max_projection_11_Triple_RNAi_probe_dd1837_rhod_SMEDWI_FITC_DAPI_20x_z2.jpg]

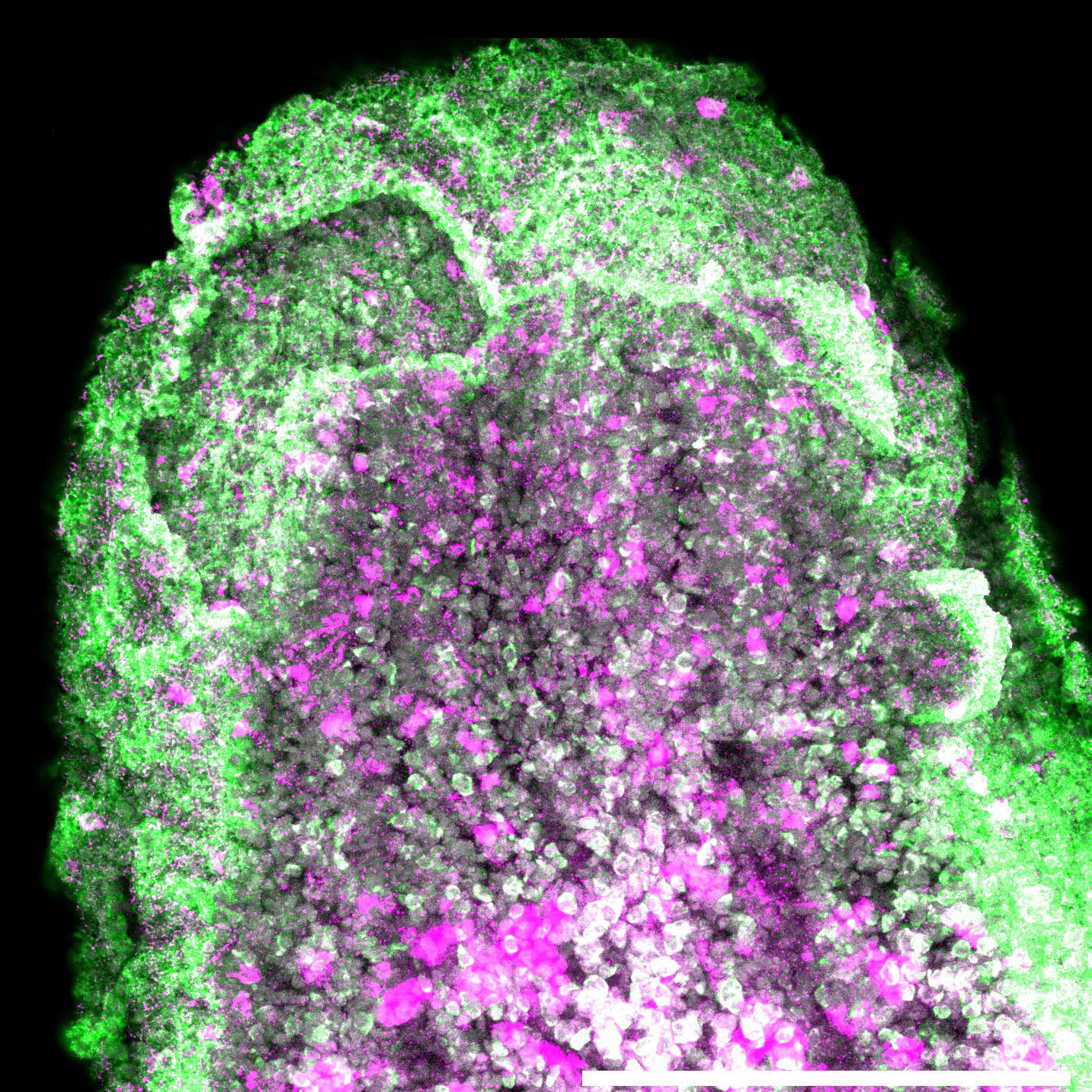

Supplement: Supplementary file 14 — Source data Fig. 7 [file 44318_2025_662_MOESM14_ESM.zip › Figure 7/7D/Max_projection_12_Triple_RNAi_probe_dd1837_rhod_SMEDWI_FITC_DAPI_20x_z2.jpg]

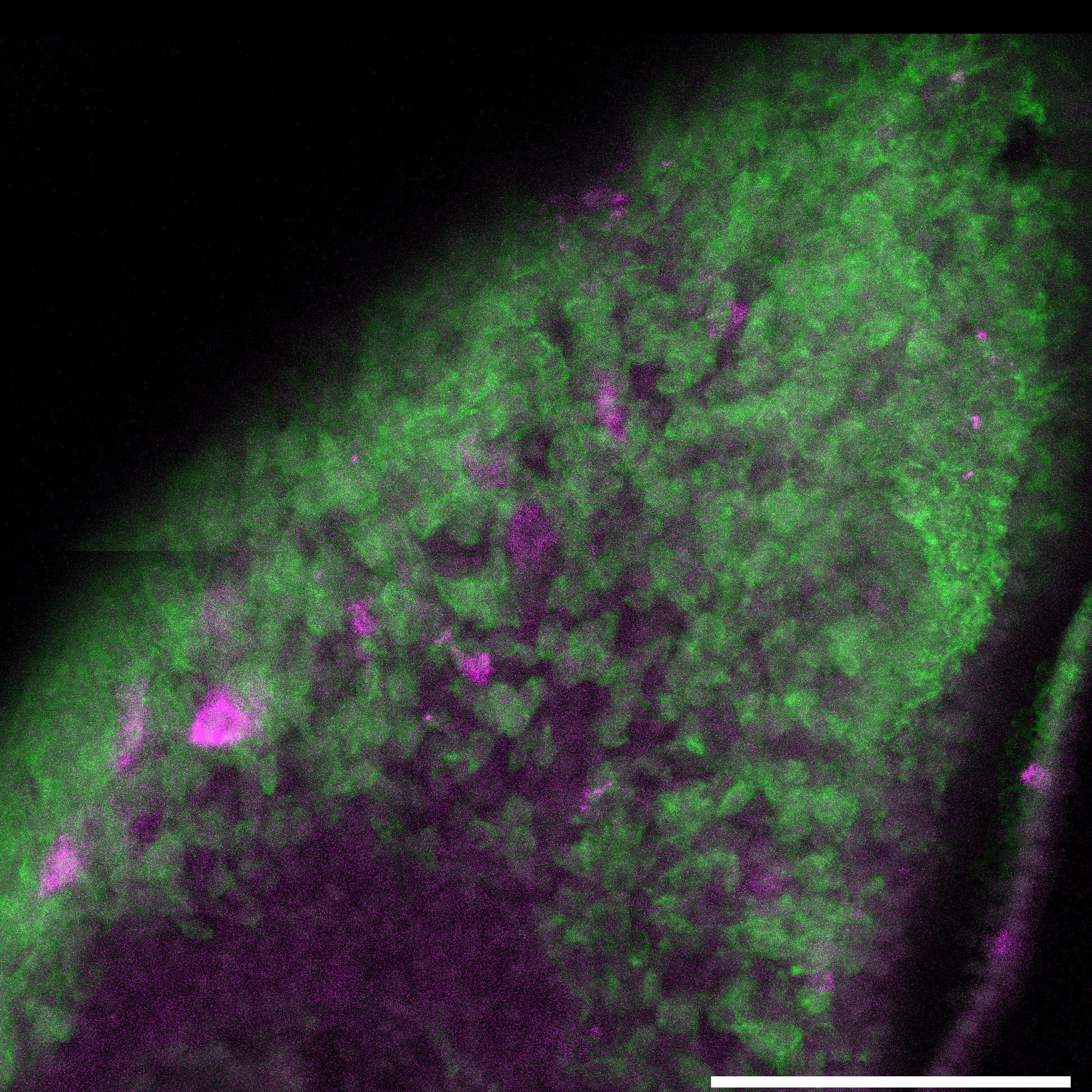

Supplement: Supplementary file 14 — Source data Fig. 7 [file 44318_2025_662_MOESM14_ESM.zip › Figure 7/7D/Max_projection_1_Control_RNAi_probe_dd1837_rhod_SMEDWI_FITC_DAPI_20x_z2.jpg]

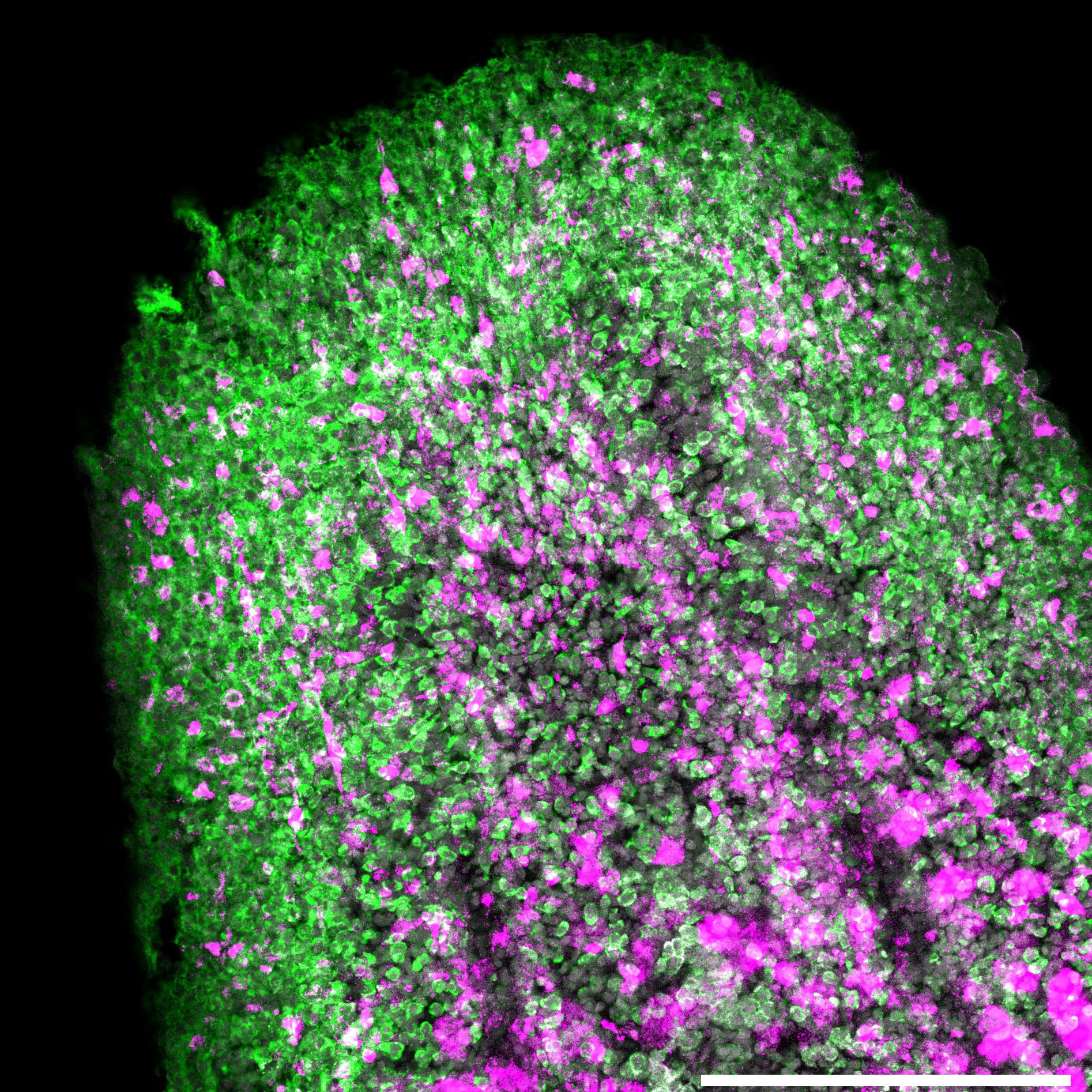

Supplement: Supplementary file 14 — Source data Fig. 7 [file 44318_2025_662_MOESM14_ESM.zip › Figure 7/7D/Max_projection_1_Triple_RNAi_probe_dd1837_rhod_SMEDWI_FITC_DAPI_20x_z2.jpg]

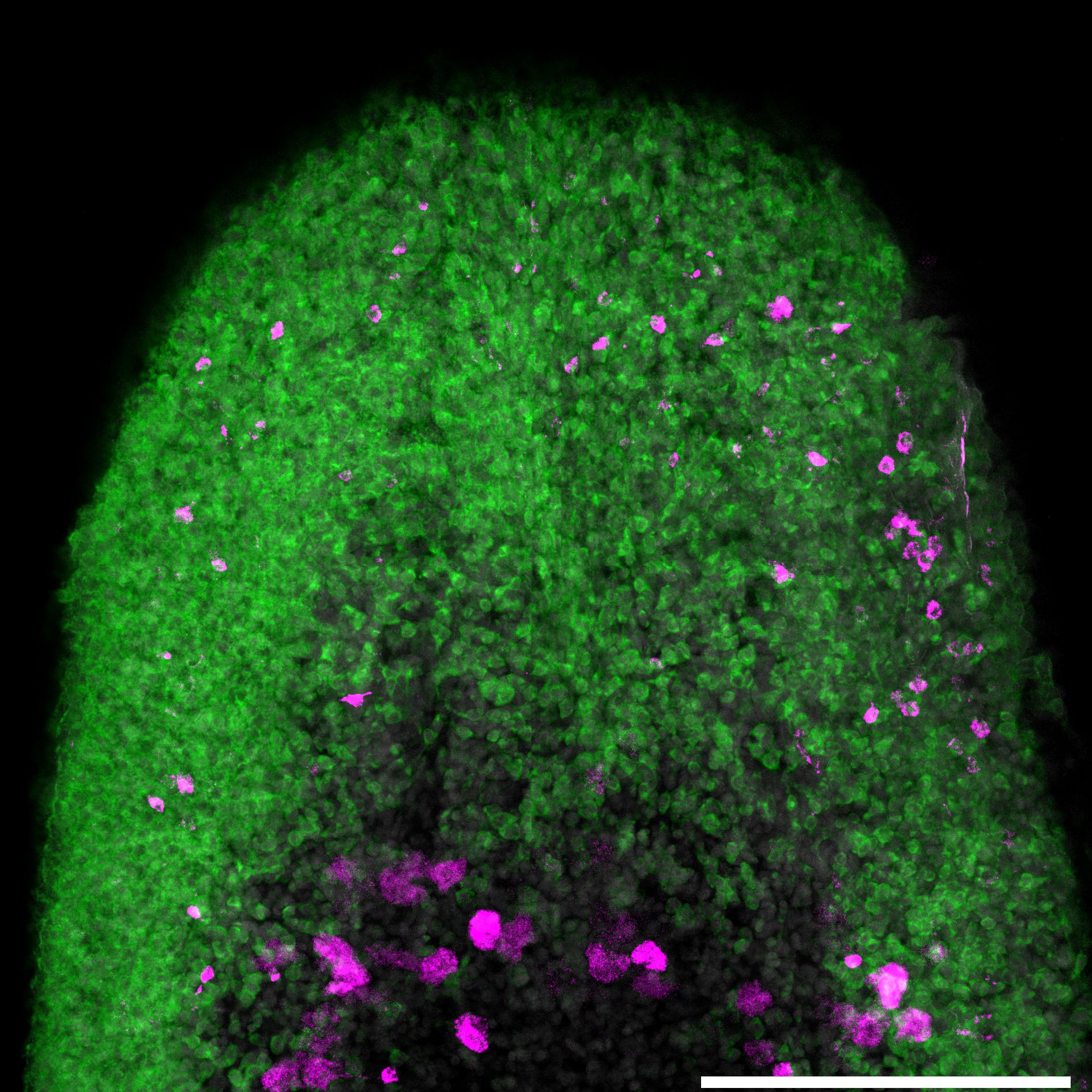

Supplement: Supplementary file 14 — Source data Fig. 7 [file 44318_2025_662_MOESM14_ESM.zip › Figure 7/7D/Max_projection_2_Control_RNAi_probe_dd1837_rhod_SMEDWI_FITC_DAPI_20x_z2.jpg]

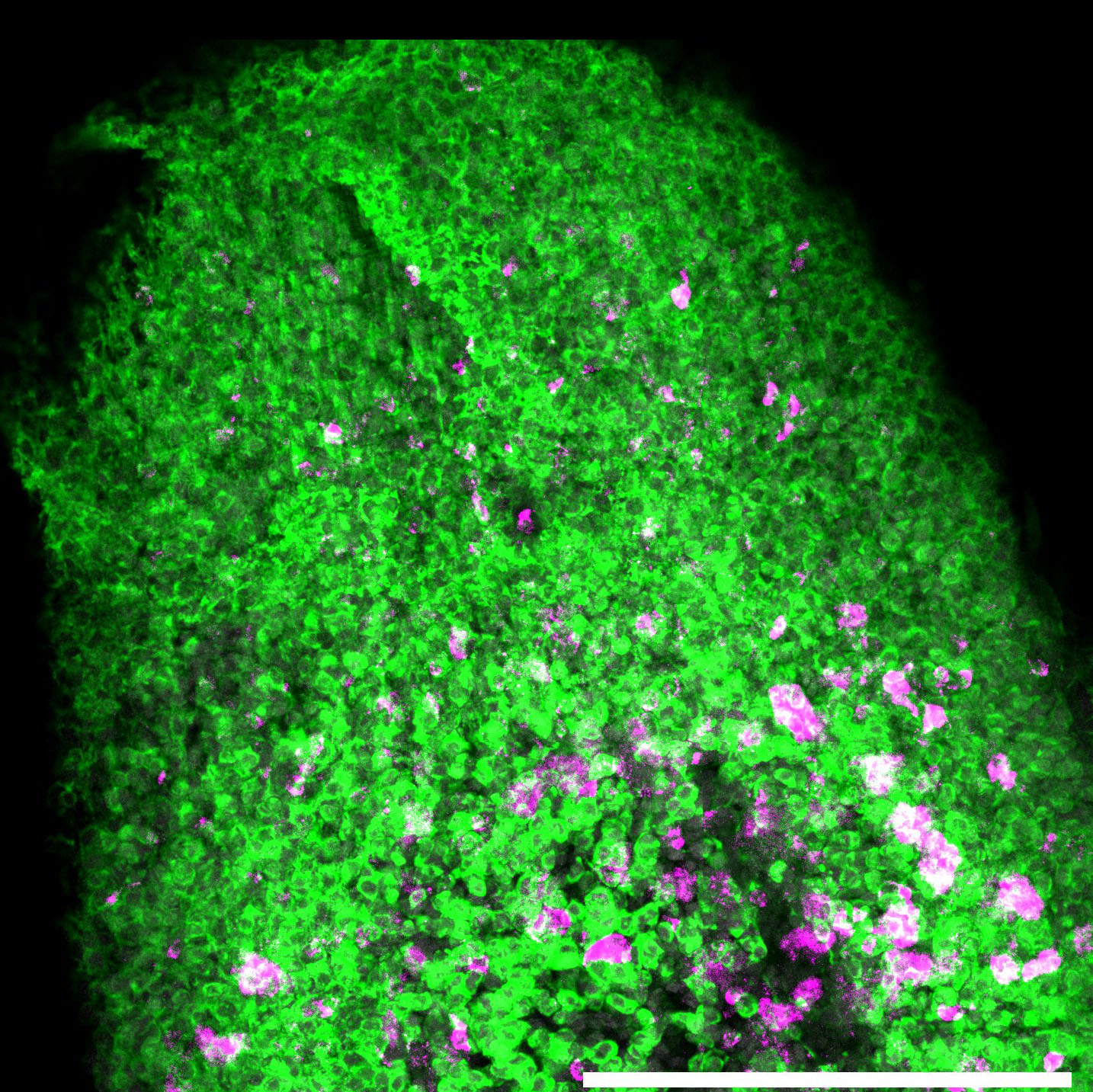

Supplement: Supplementary file 14 — Source data Fig. 7 [file 44318_2025_662_MOESM14_ESM.zip › Figure 7/7D/Max_projection_2_Triple_RNAi_probe_dd1837_rhod_SMEDWI_FITC_DAPI_20x_z2.jpg]

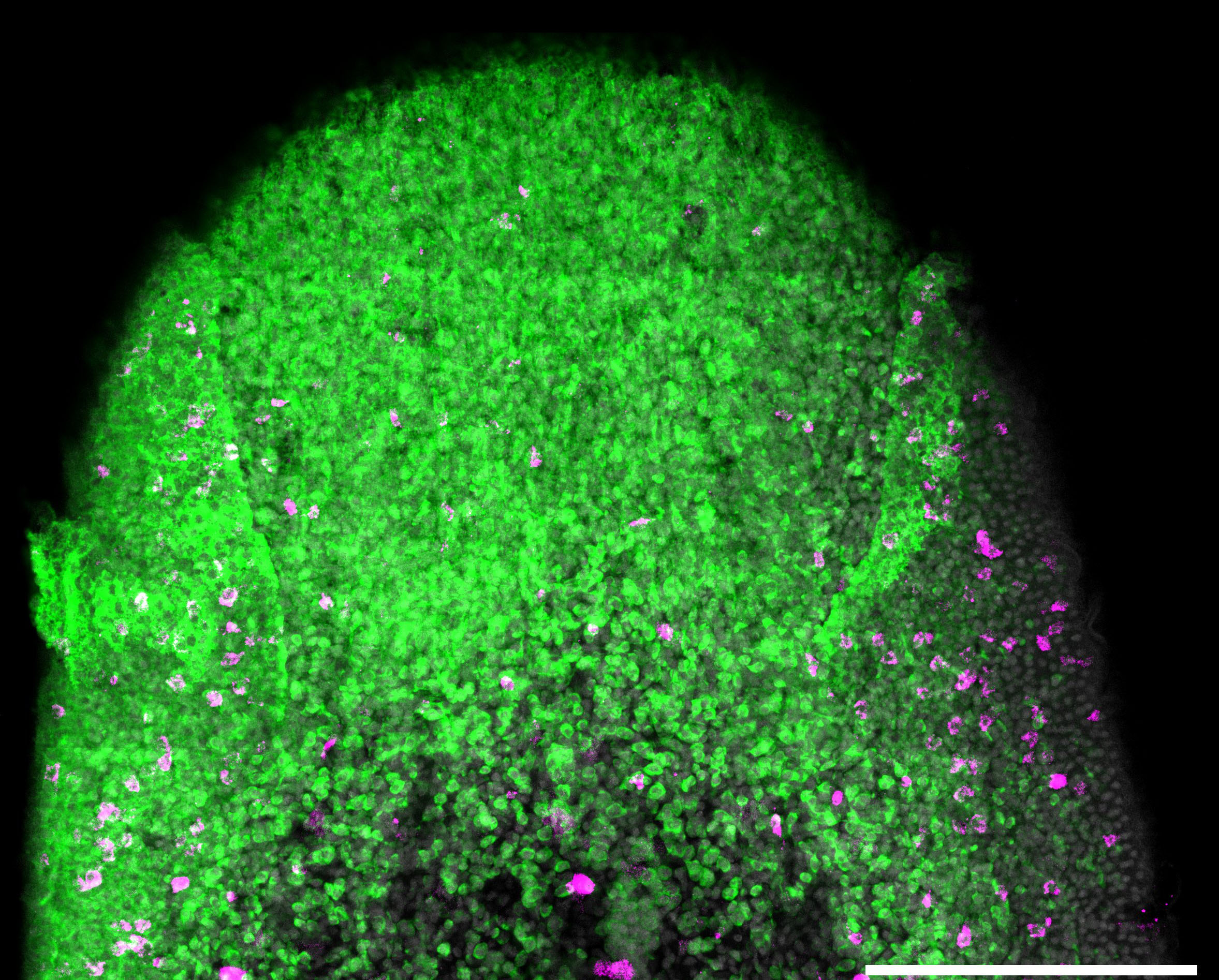

Supplement: Supplementary file 14 — Source data Fig. 7 [file 44318_2025_662_MOESM14_ESM.zip › Figure 7/7D/Max_projection_3_Control_RNAi_probe_dd1837_rhod_SMEDWI_FITC_DAPI_20x_z2.jpg]

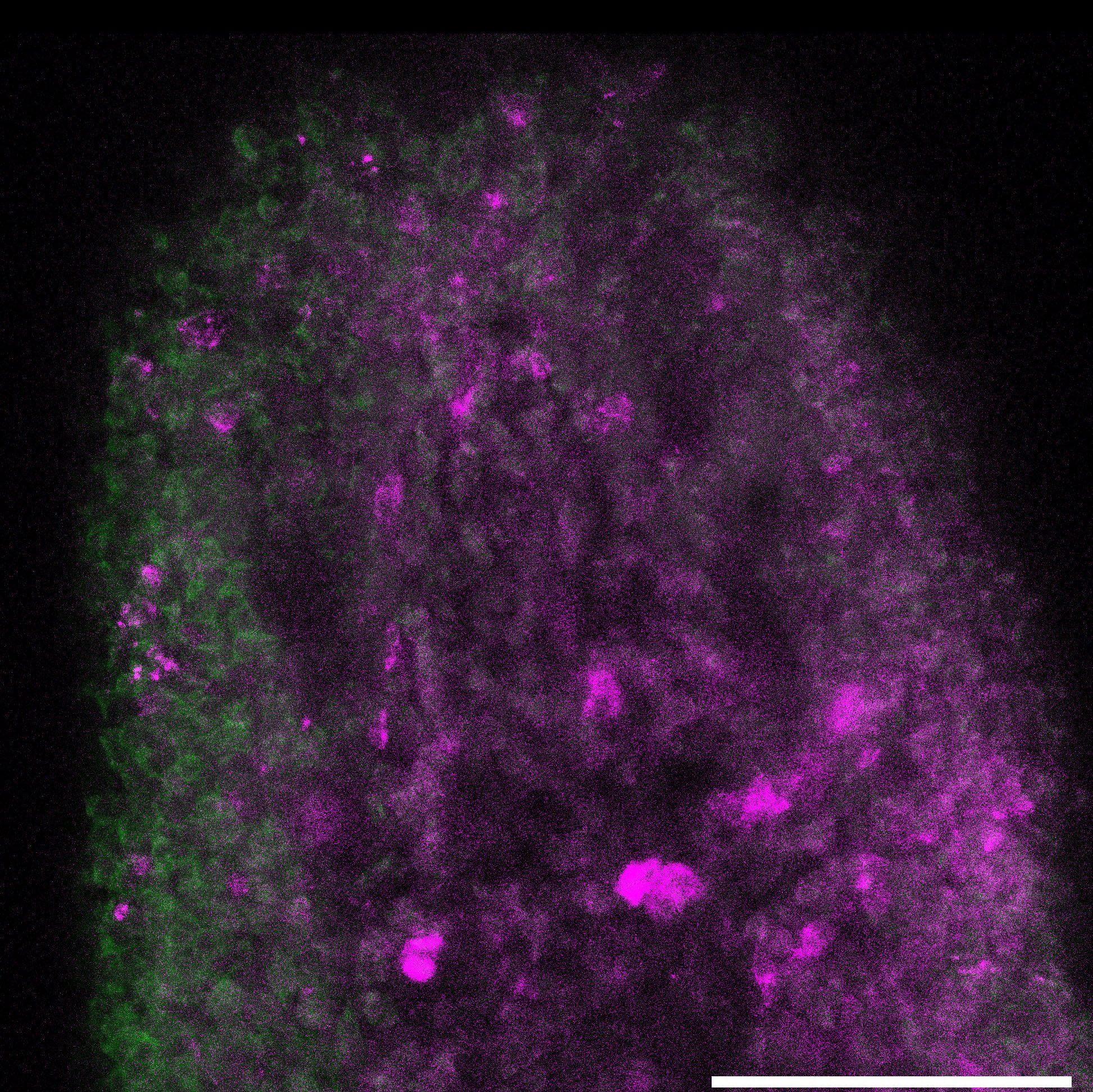

Supplement: Supplementary file 14 — Source data Fig. 7 [file 44318_2025_662_MOESM14_ESM.zip › Figure 7/7D/Max_projection_3_Triple_RNAi_probe_dd1837_rhod_SMEDWI_FITC_DAPI_20x_z2.jpg]

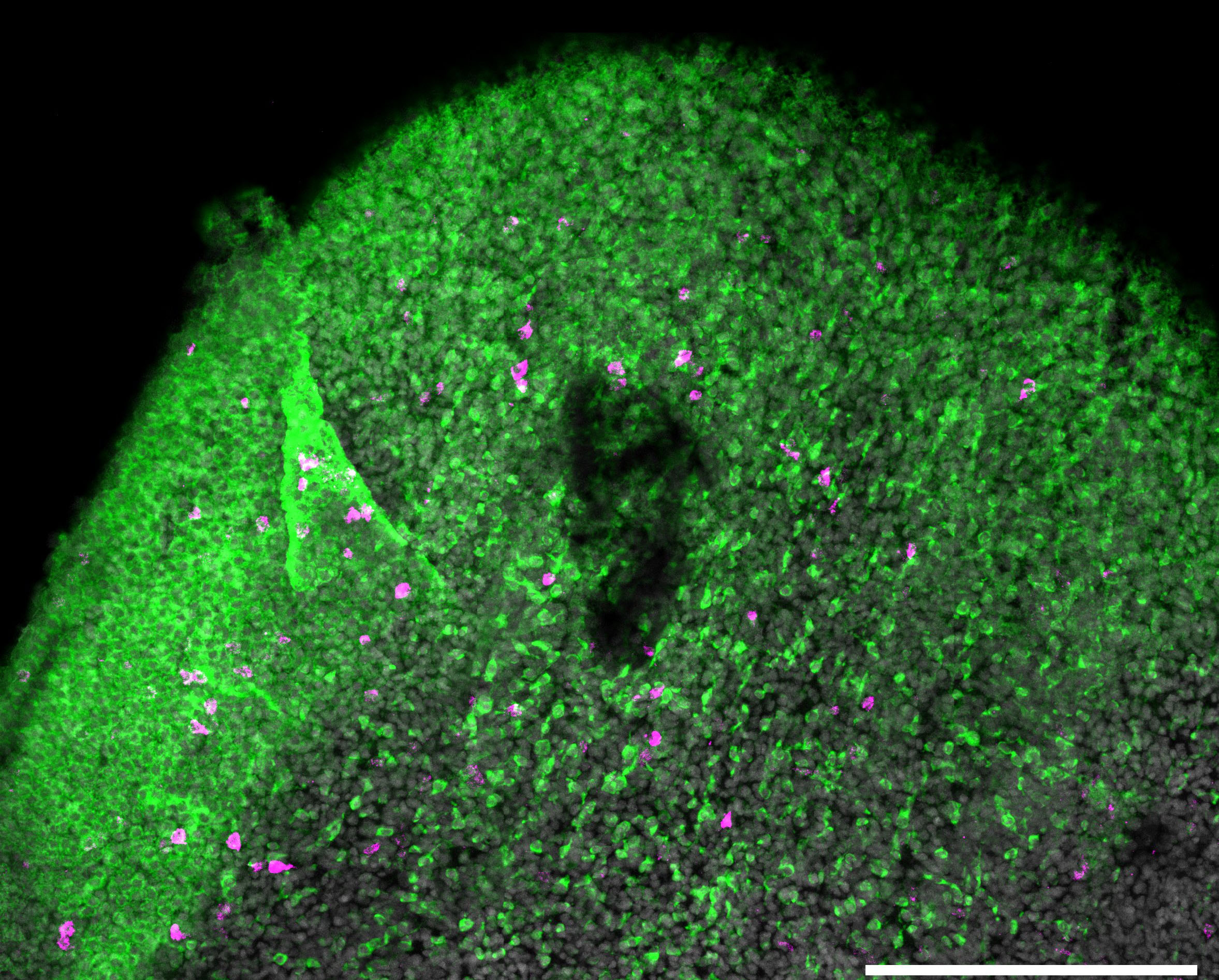

Supplement: Supplementary file 14 — Source data Fig. 7 [file 44318_2025_662_MOESM14_ESM.zip › Figure 7/7D/Max_projection_4_Control_RNAi_probe_dd1837_rhod_SMEDWI_FITC_DAPI_20x_z2.jpg]

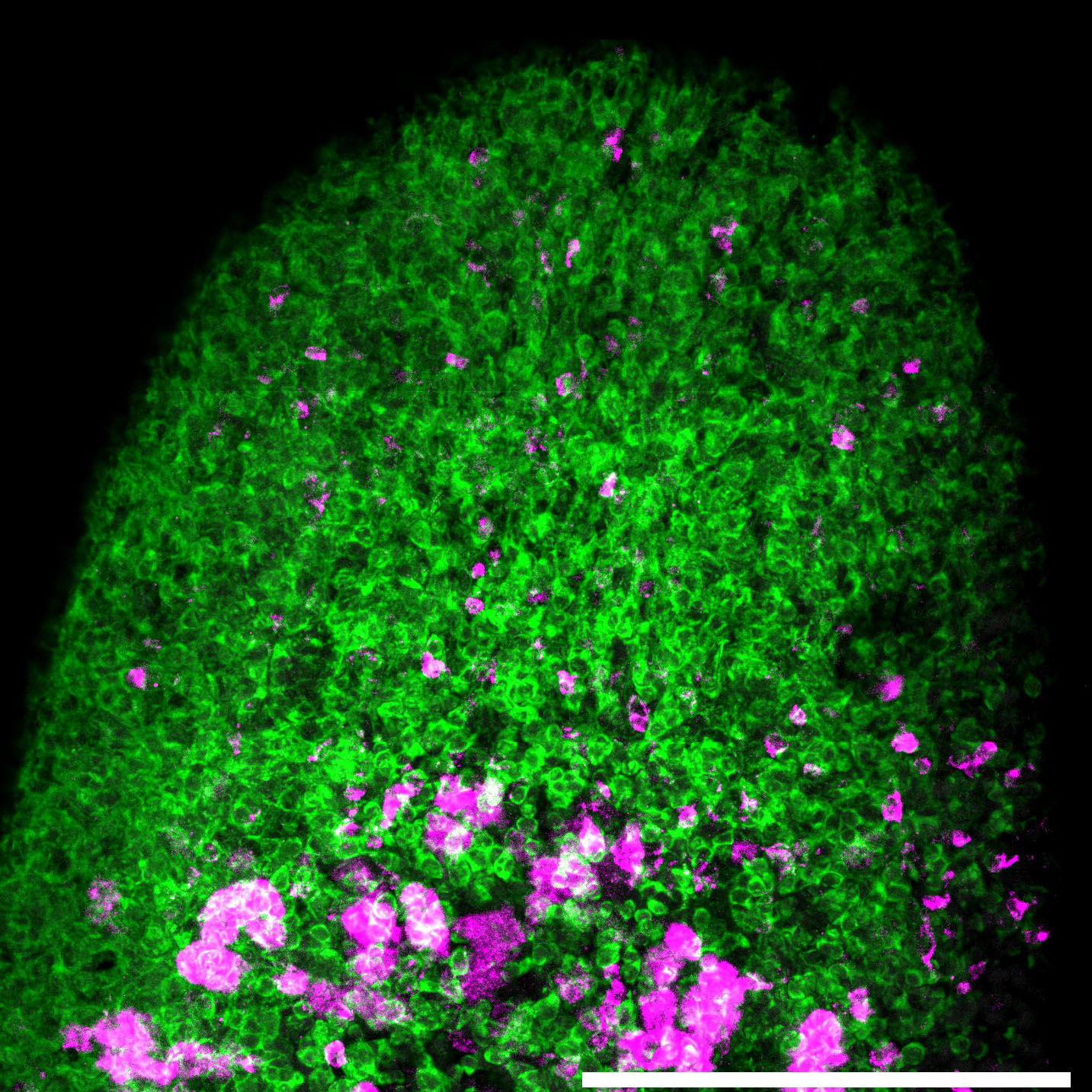

Supplement: Supplementary file 14 — Source data Fig. 7 [file 44318_2025_662_MOESM14_ESM.zip › Figure 7/7D/Max_projection_4_Triple_RNAi_probe_dd1837_rhod_SMEDWI_FITC_DAPI_20x_z2.jpg]

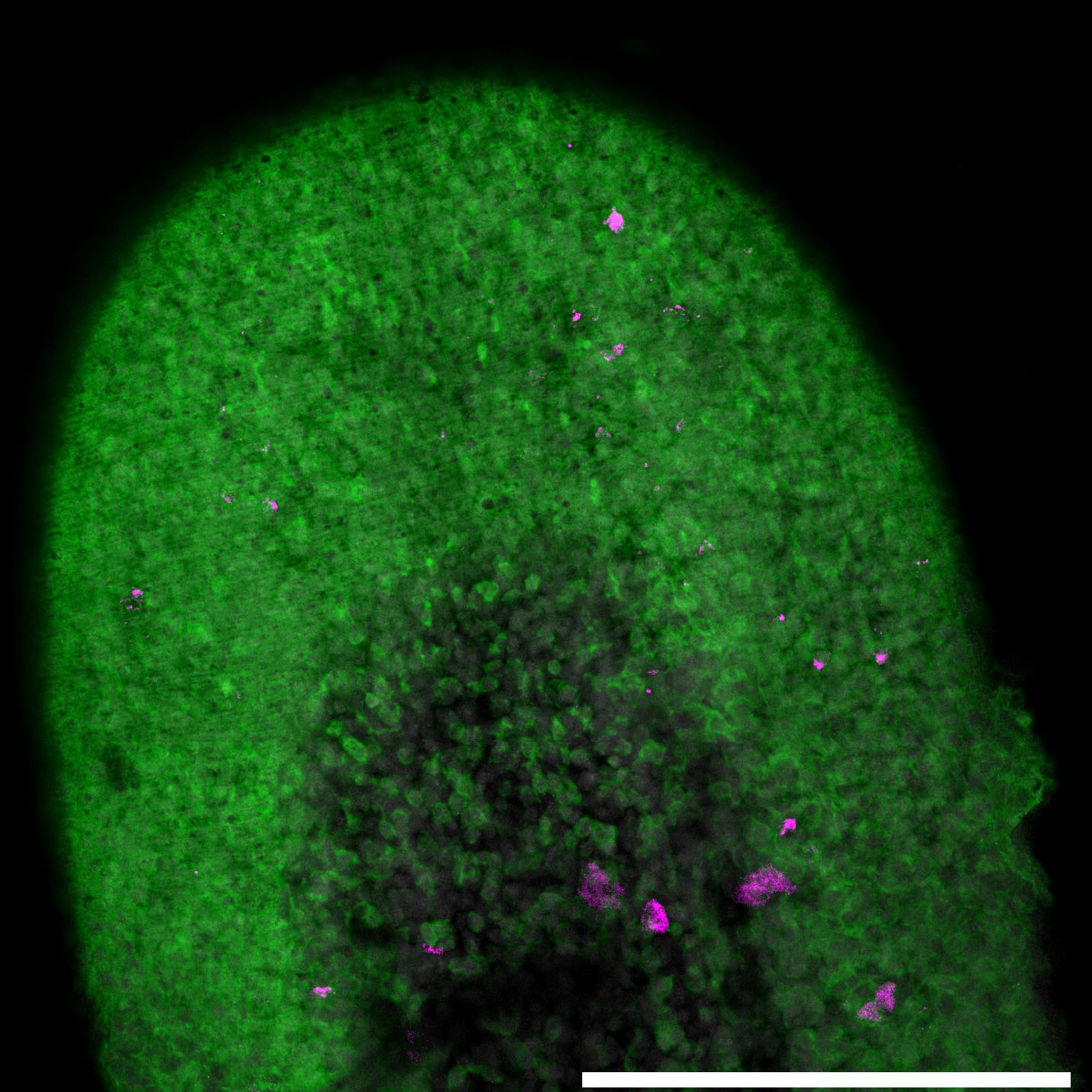

Supplement: Supplementary file 14 — Source data Fig. 7 [file 44318_2025_662_MOESM14_ESM.zip › Figure 7/7D/Max_projection_5_Control_RNAi_probe_dd1837_rhod_SMEDWI_FITC_DAPI_20x_z2.jpg]

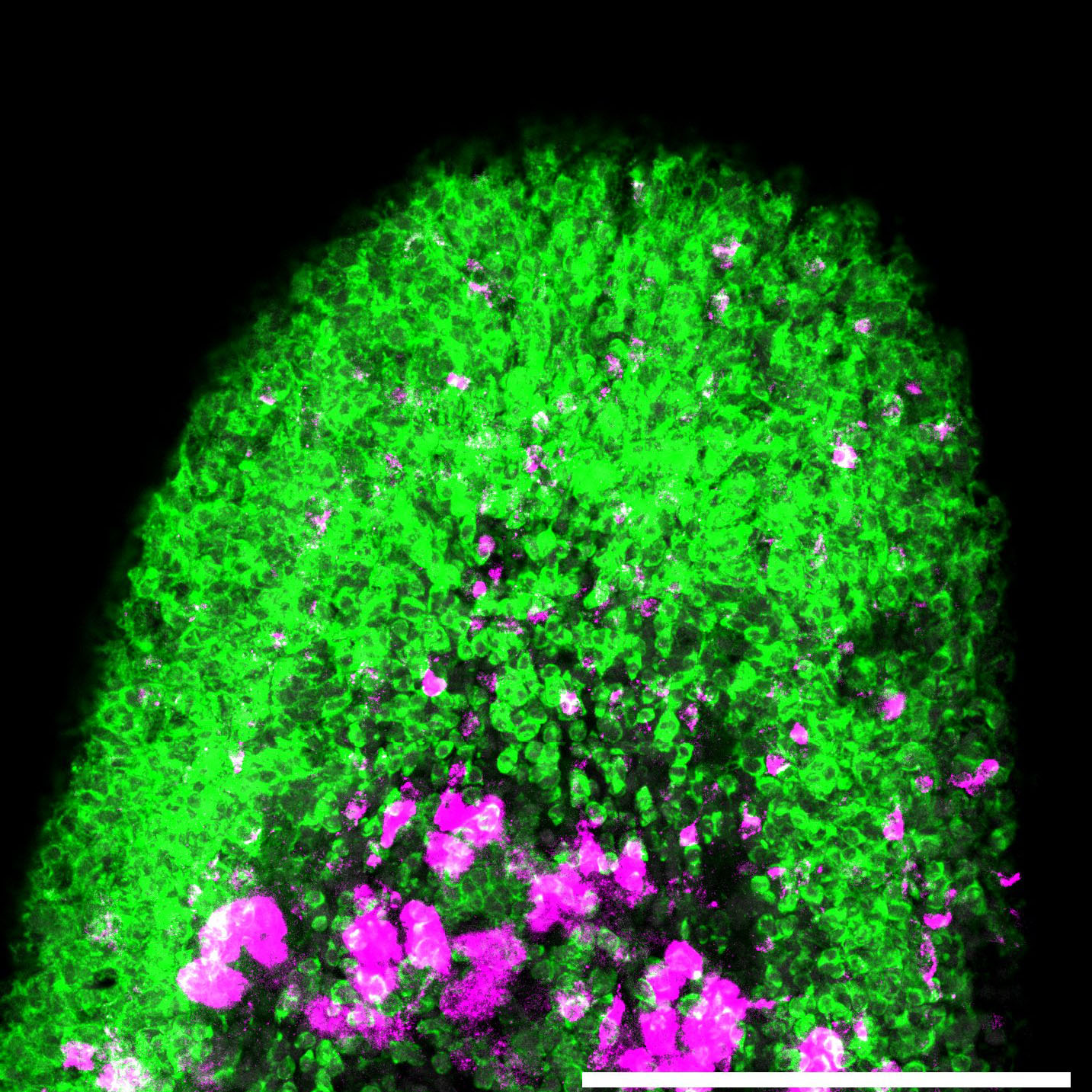

Supplement: Supplementary file 14 — Source data Fig. 7 [file 44318_2025_662_MOESM14_ESM.zip › Figure 7/7D/Max_projection_5_Triple_RNAi_probe_dd1837_rhod_SMEDWI_FITC_DAPI_20x_z2.jpg]

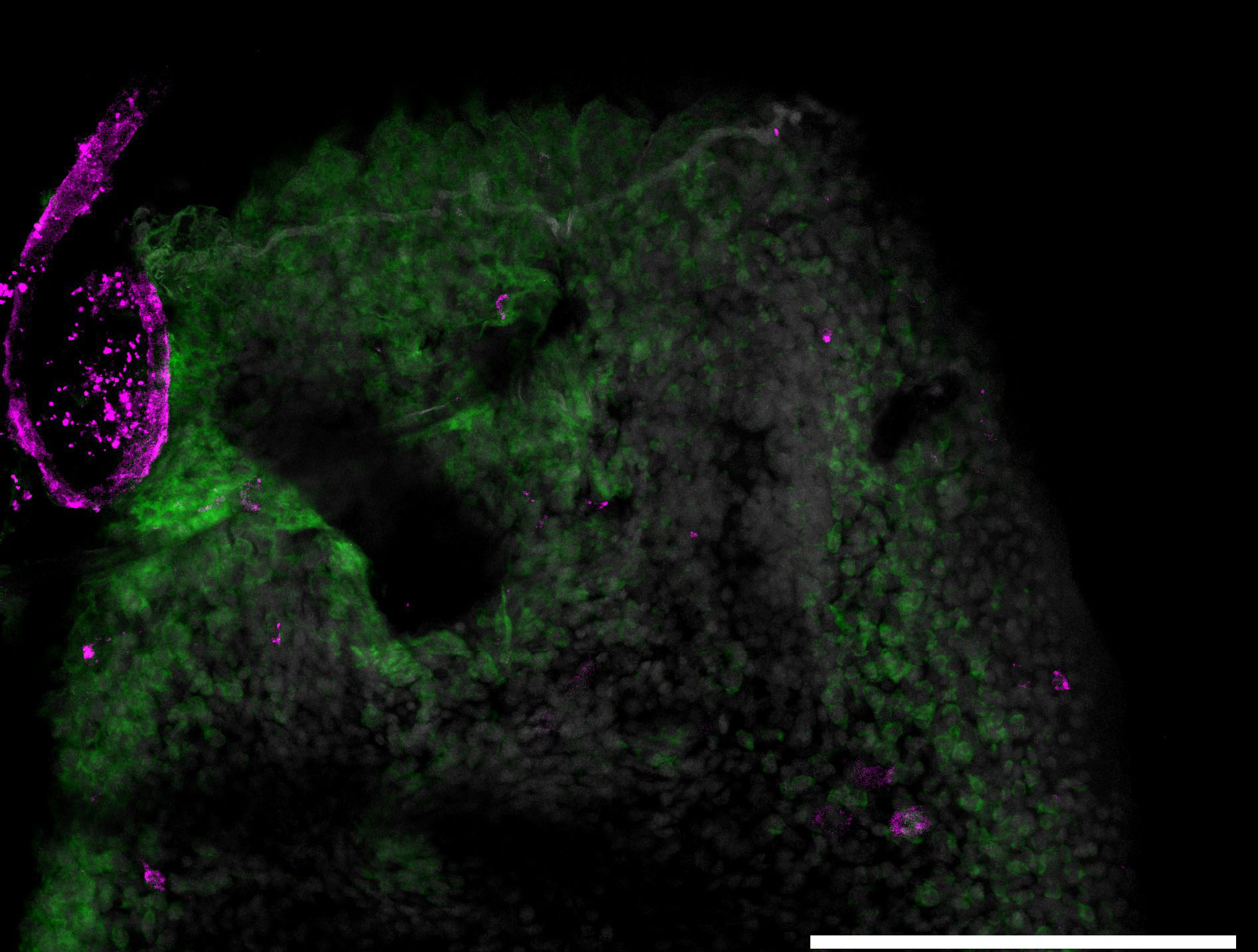

Supplement: Supplementary file 14 — Source data Fig. 7 [file 44318_2025_662_MOESM14_ESM.zip › Figure 7/7D/Max_projection_6_Control_RNAi_probe_dd1837_rhod_SMEDWI_FITC_DAPI_20x_z2.jpg]

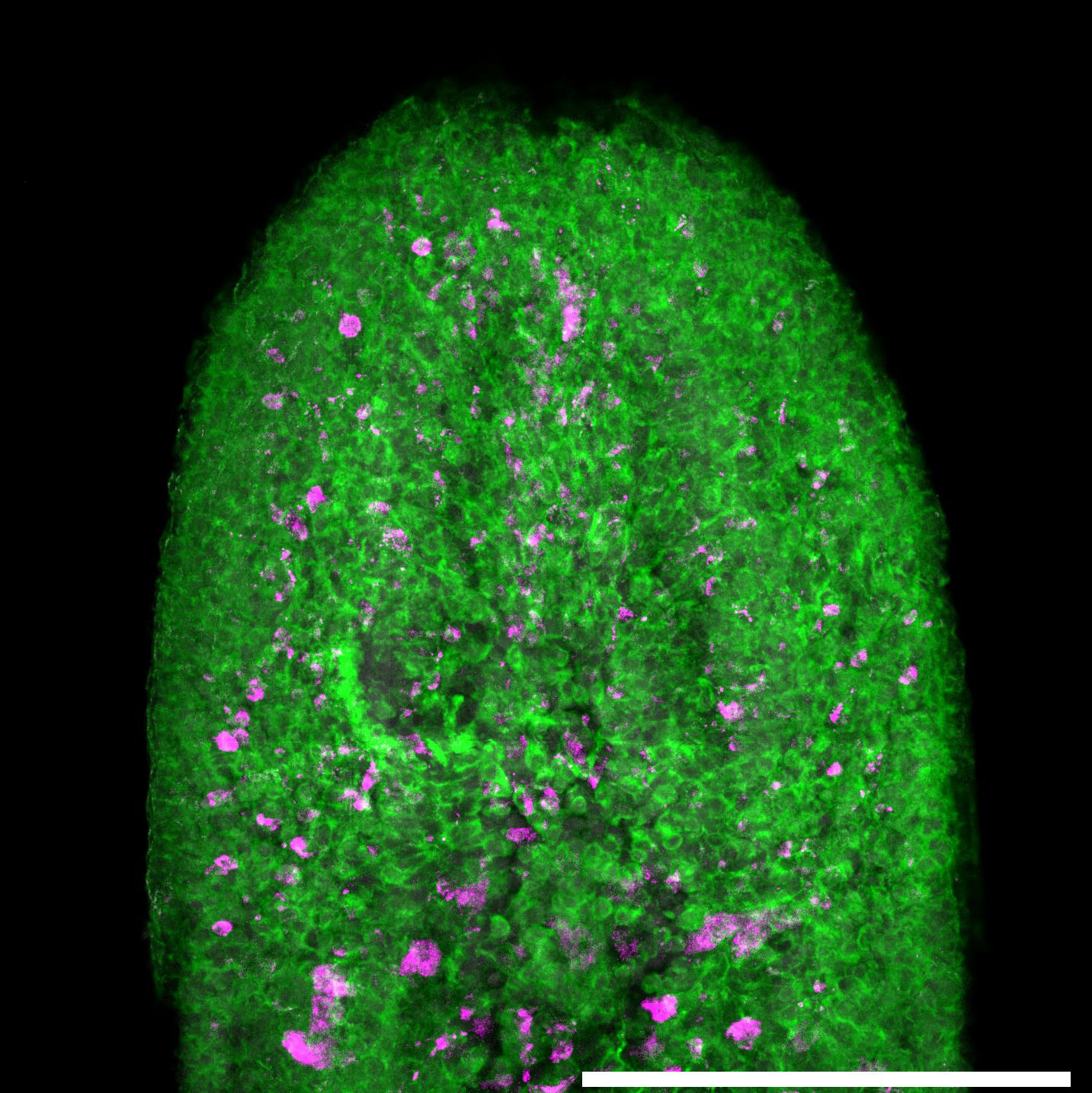

Supplement: Supplementary file 14 — Source data Fig. 7 [file 44318_2025_662_MOESM14_ESM.zip › Figure 7/7D/Max_projection_6_Triple_RNAi_probe_dd1837_rhod_SMEDWI_FITC_DAPI_20x_z2.jpg]

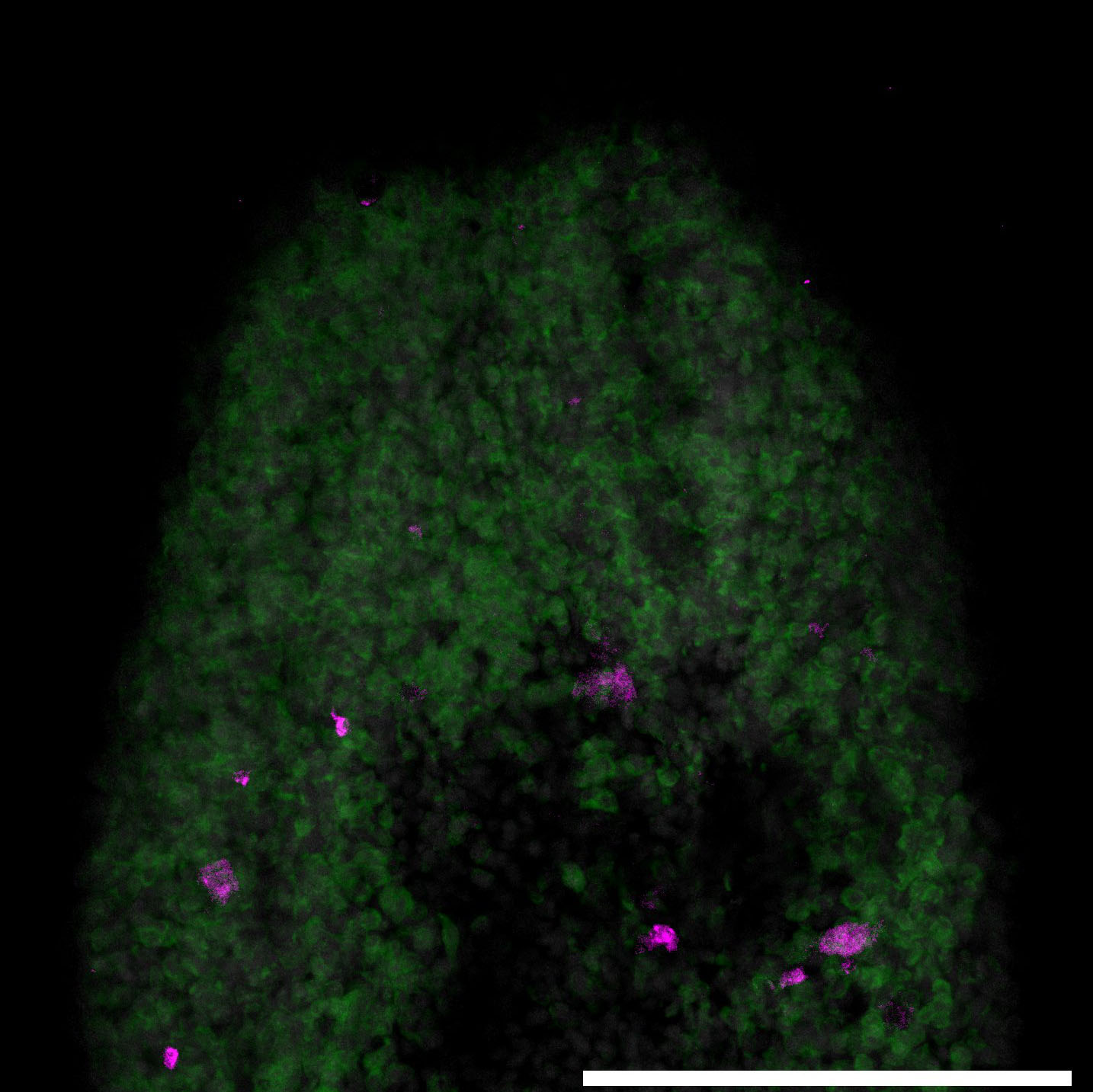

Supplement: Supplementary file 14 — Source data Fig. 7 [file 44318_2025_662_MOESM14_ESM.zip › Figure 7/7D/Max_projection_7_Control_RNAi_probe_dd1837_rhod_SMEDWI_FITC_DAPI_20x_z2.jpg]

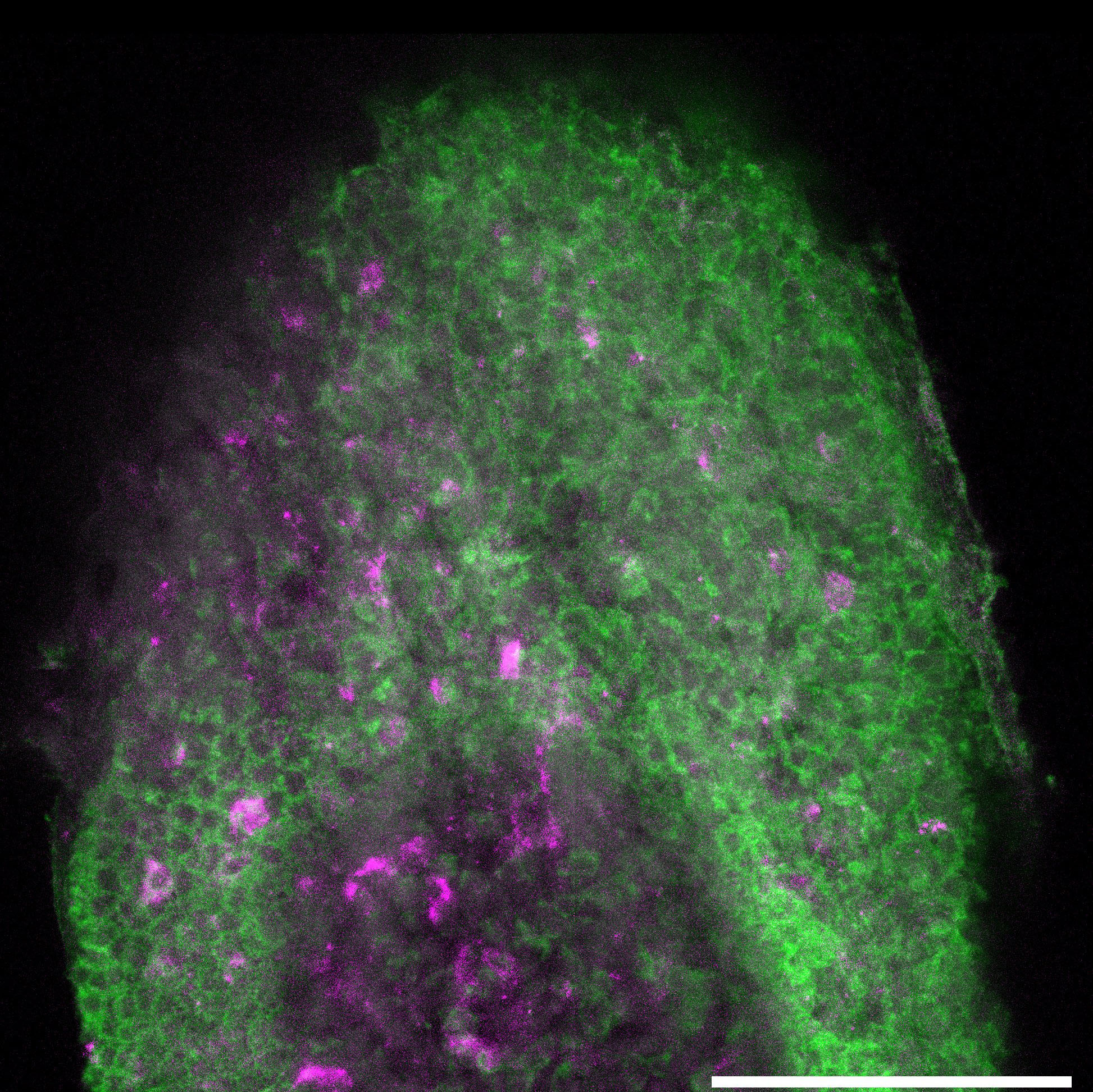

Supplement: Supplementary file 14 — Source data Fig. 7 [file 44318_2025_662_MOESM14_ESM.zip › Figure 7/7D/Max_projection_7_Triple_RNAi_probe_dd1837_rhod_SMEDWI_FITC_DAPI_20x_z2.jpg]

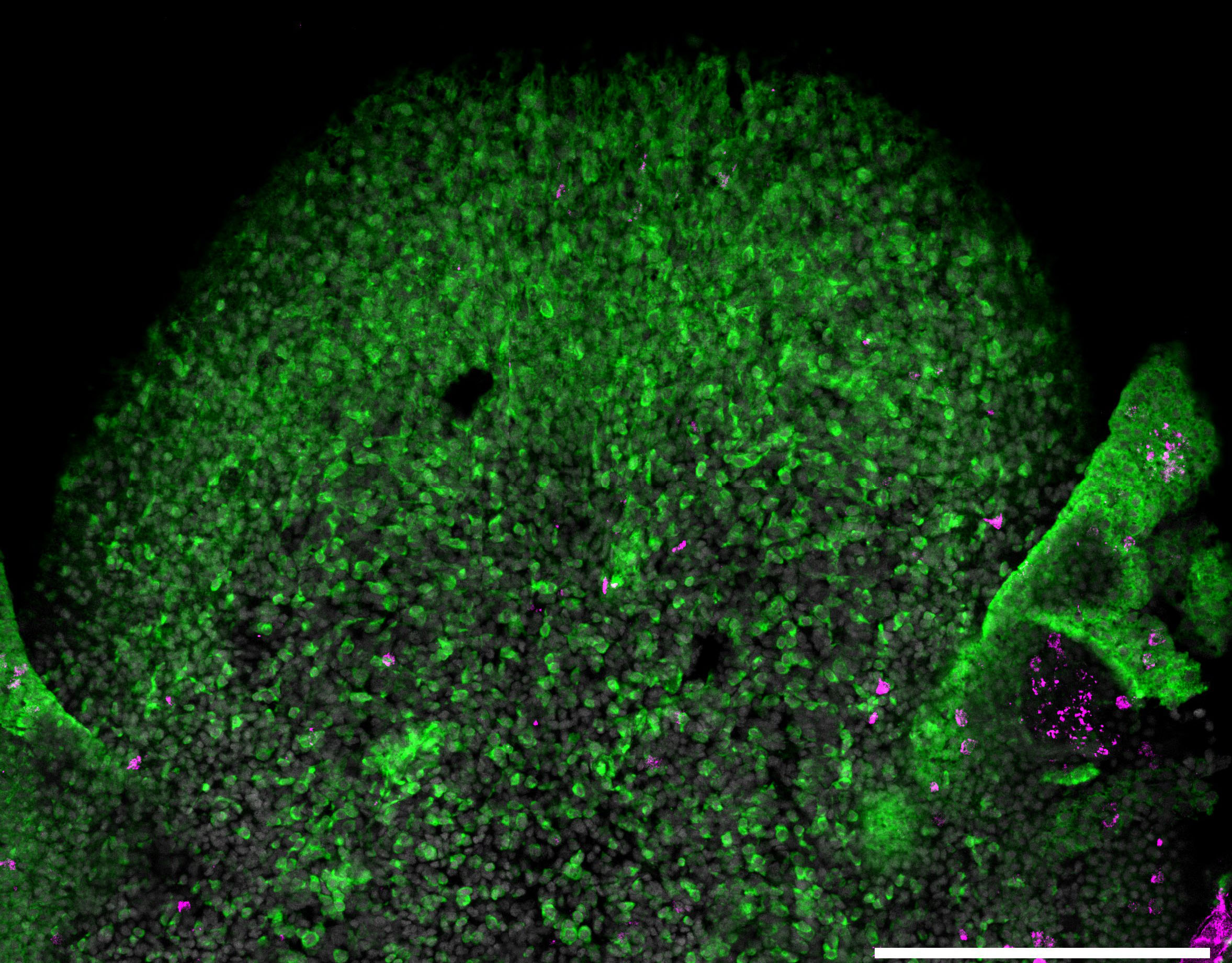

Supplement: Supplementary file 14 — Source data Fig. 7 [file 44318_2025_662_MOESM14_ESM.zip › Figure 7/7D/Max_projection_8_Control_RNAi_probe_dd1837_rhod_SMEDWI_FITC_DAPI_20x_z2.jpg]

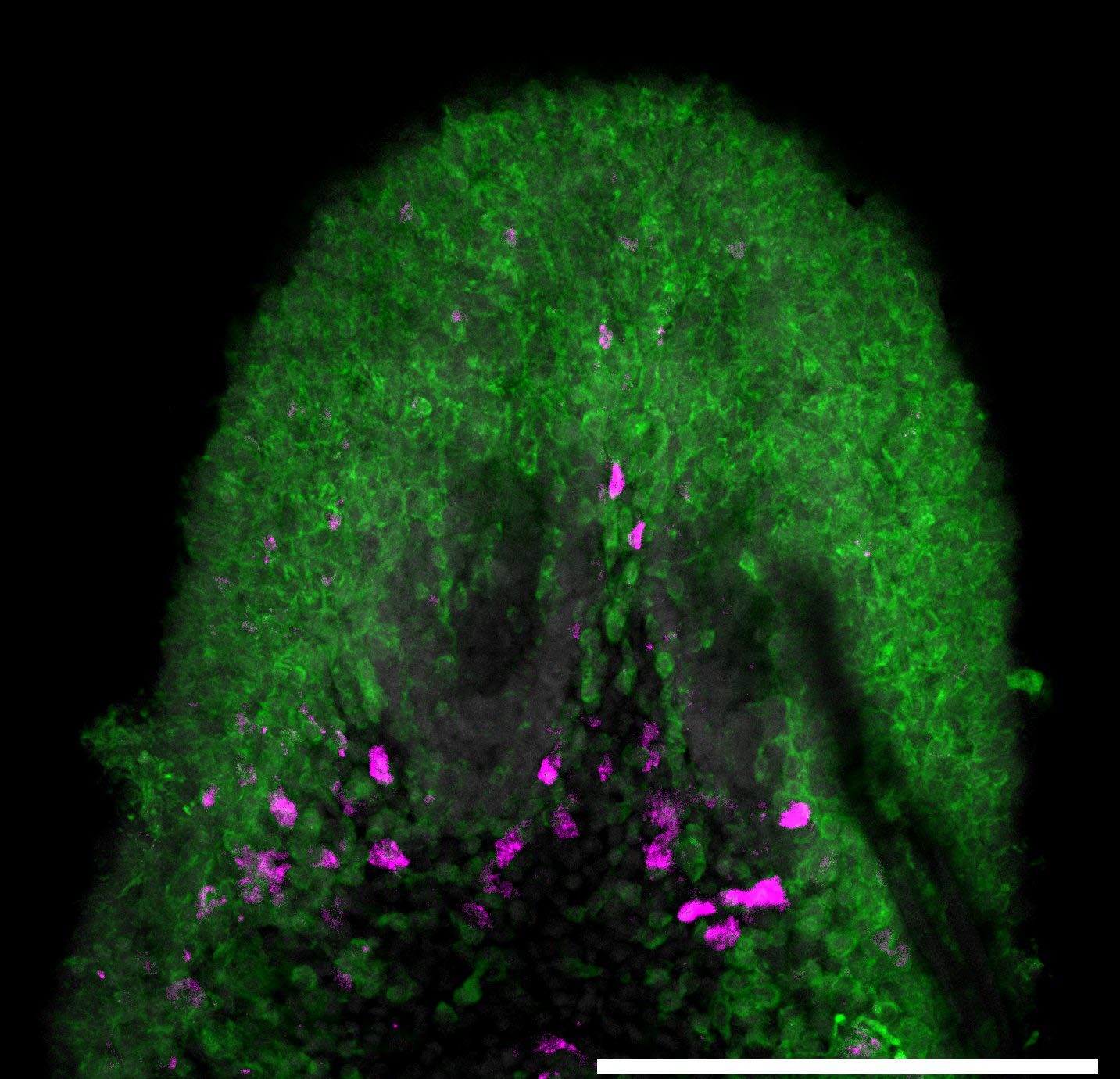

Supplement: Supplementary file 14 — Source data Fig. 7 [file 44318_2025_662_MOESM14_ESM.zip › Figure 7/7D/Max_projection_8_Triple_RNAi_probe_dd1837_rhod_SMEDWI_FITC_DAPI_20x_z2.jpg]

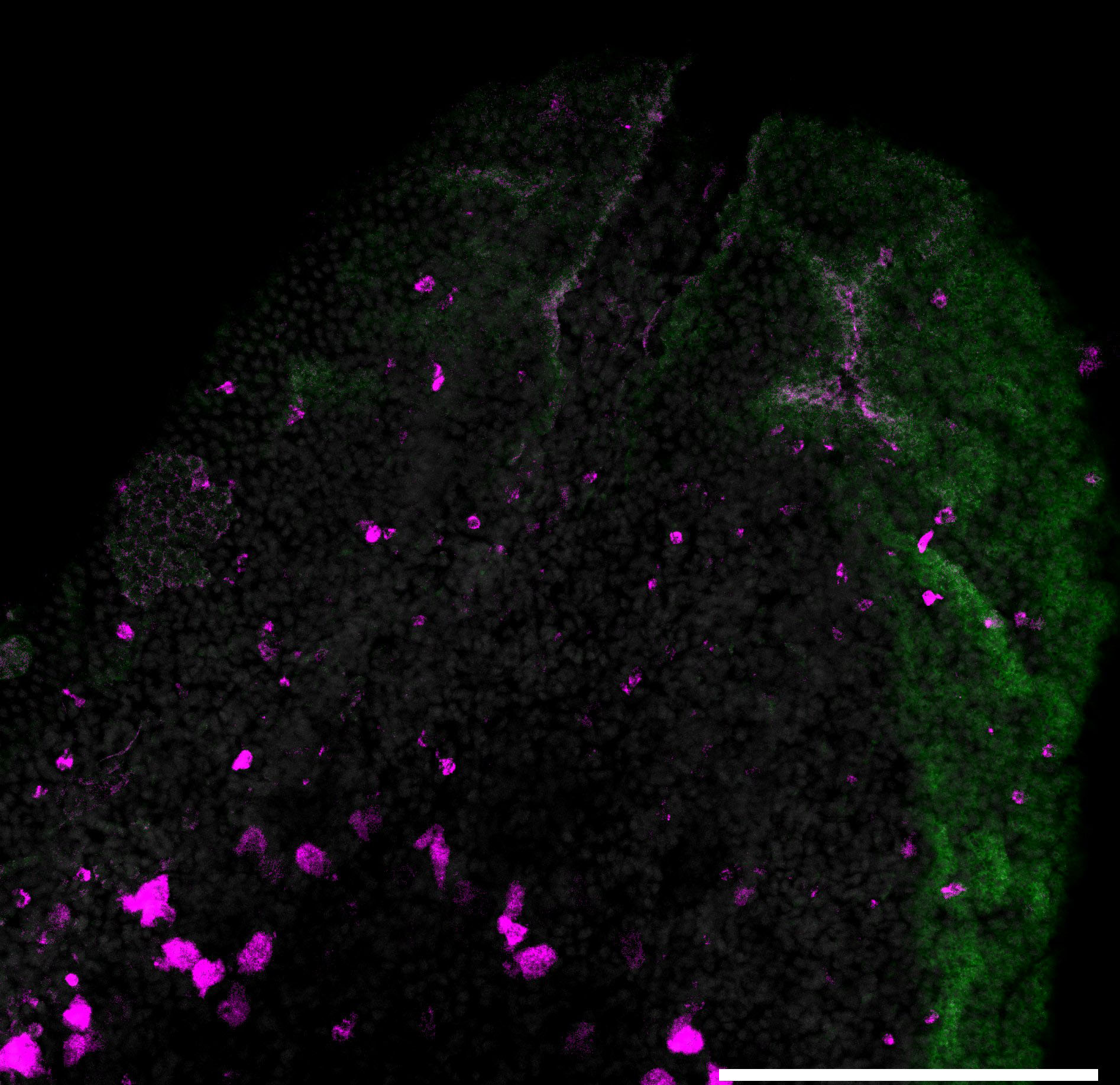

Supplement: Supplementary file 14 — Source data Fig. 7 [file 44318_2025_662_MOESM14_ESM.zip › Figure 7/7D/Max_projection_9_Control_RNAi_probe_dd1837_rhod_SMEDWI_FITC_DAPI_20x_z2.jpg]

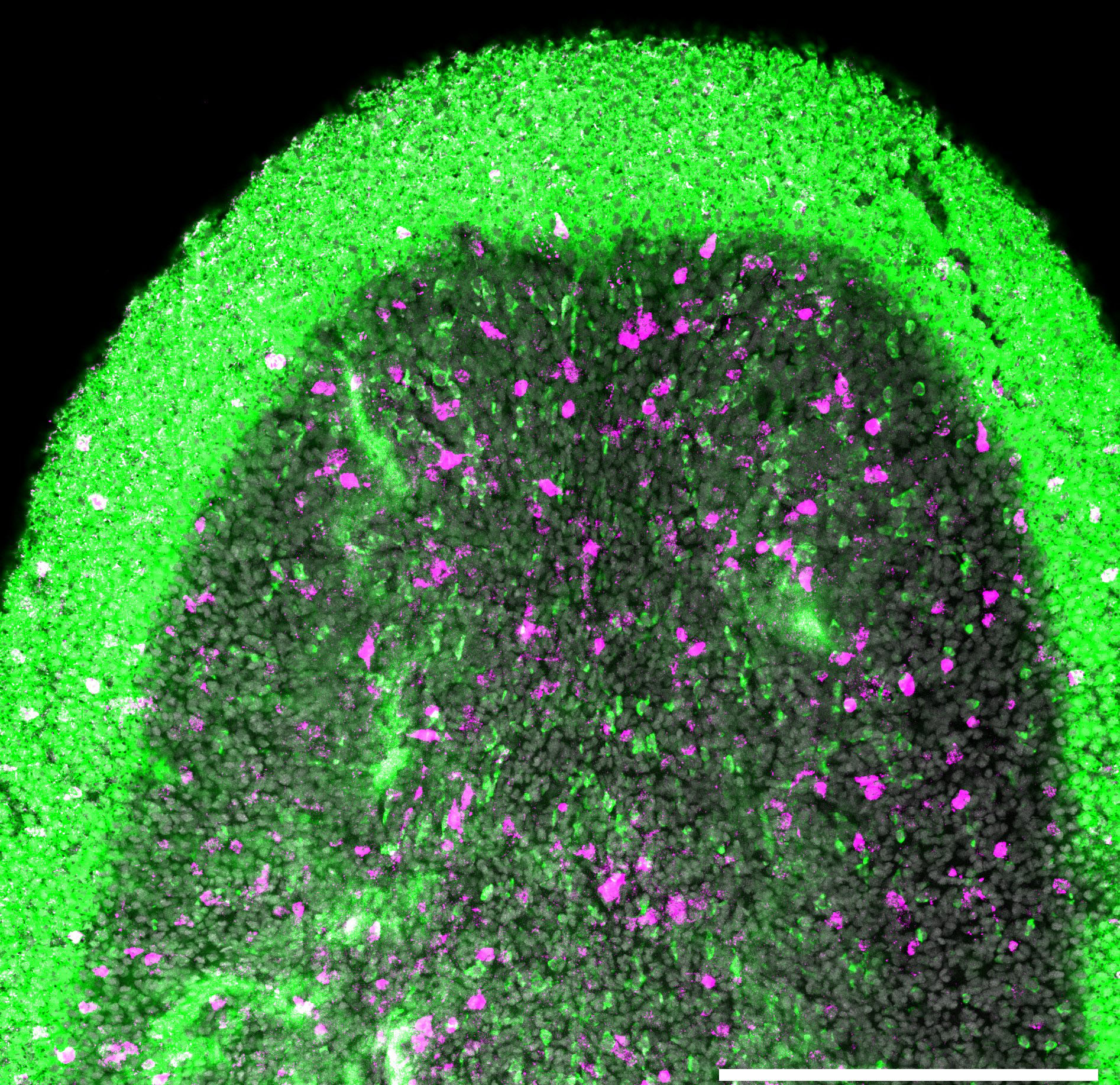

Supplement: Supplementary file 14 — Source data Fig. 7 [file 44318_2025_662_MOESM14_ESM.zip › Figure 7/7D/Max_projection_9_Triple_RNAi_probe_dd1837_rhod_SMEDWI_FITC_DAPI_20x_z2.jpg]

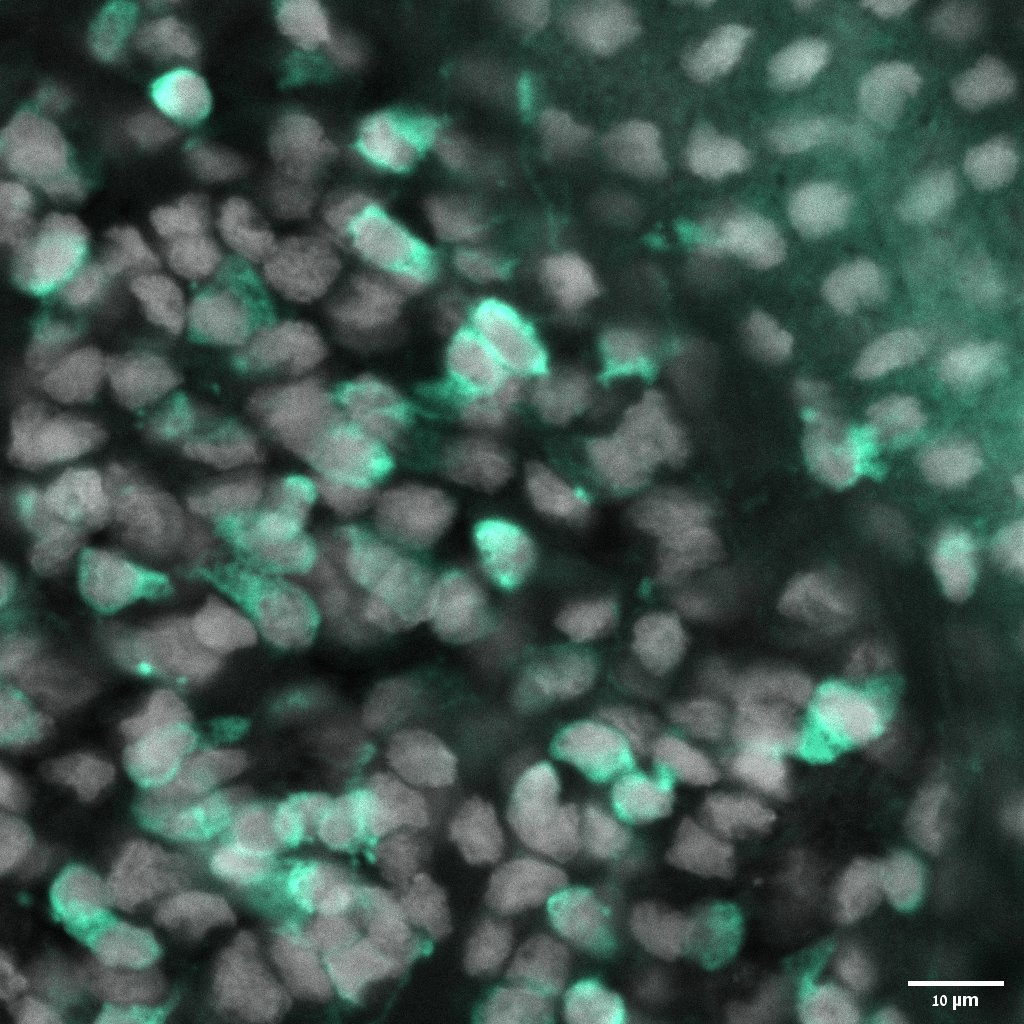

Supplement: Supplementary file 14 — Source data Fig. 7 [file 44318_2025_662_MOESM14_ESM.zip › Figure 7/7E/Main_figure_panel_Triple_RNAi_dd940_rhod_SMEDWI_FITC_DAPI_20x_z3_FITC_channel.jpg]

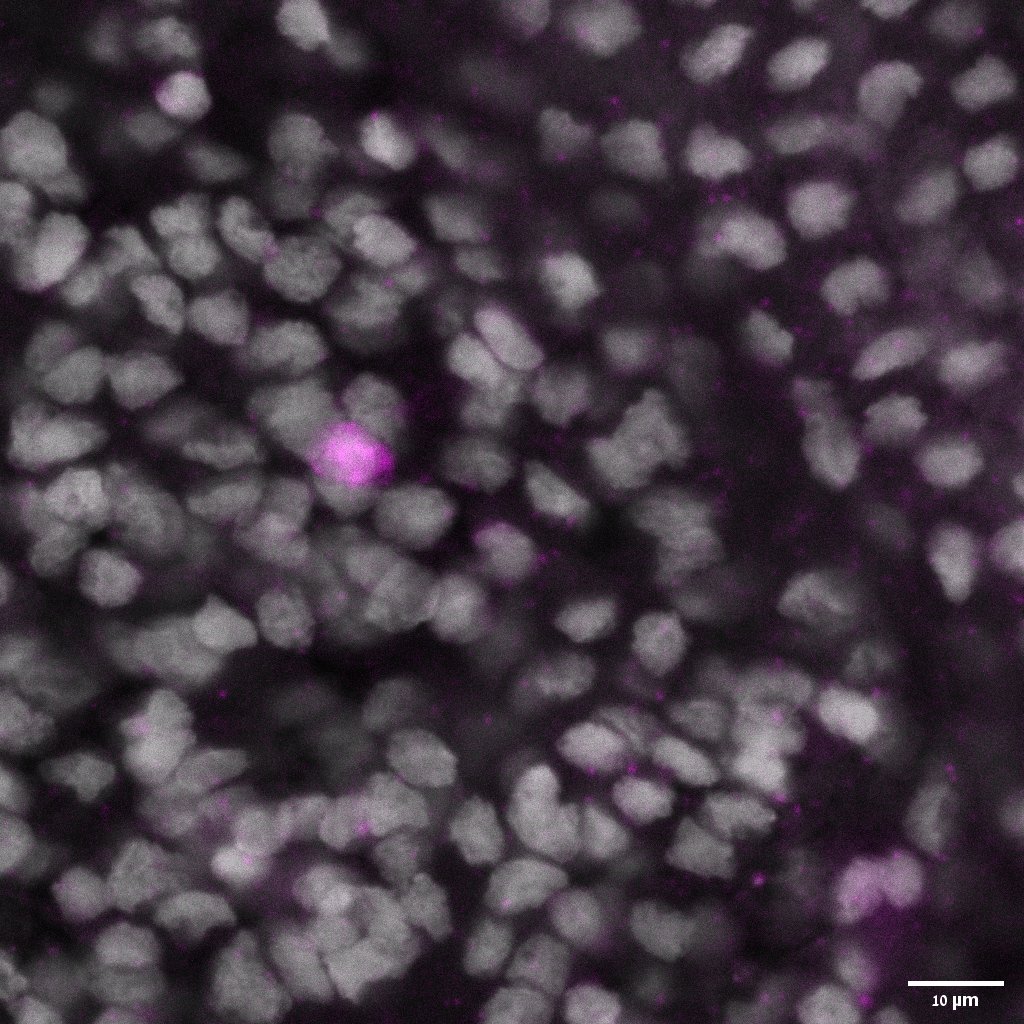

Supplement: Supplementary file 14 — Source data Fig. 7 [file 44318_2025_662_MOESM14_ESM.zip › Figure 7/7E/Main_figure_panel_Triple_RNAi_dd940_rhod_SMEDWI_FITC_DAPI_20x_z3_Magenta_channel.jpg]

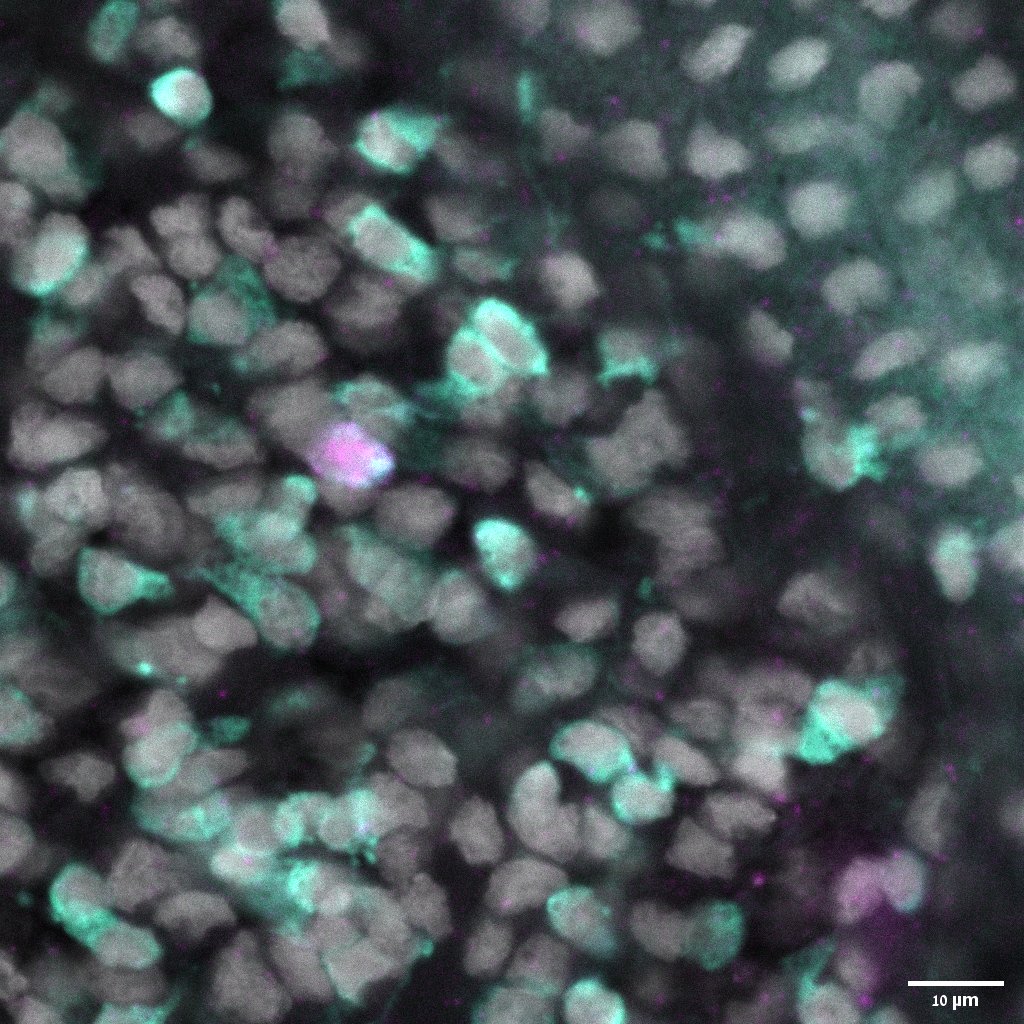

Supplement: Supplementary file 14 — Source data Fig. 7 [file 44318_2025_662_MOESM14_ESM.zip › Figure 7/7E/Main_figure_panel_Triple_RNAi_dd940_rhod_SMEDWI_FITC_DAPI_20x_z3_Merged.jpg]

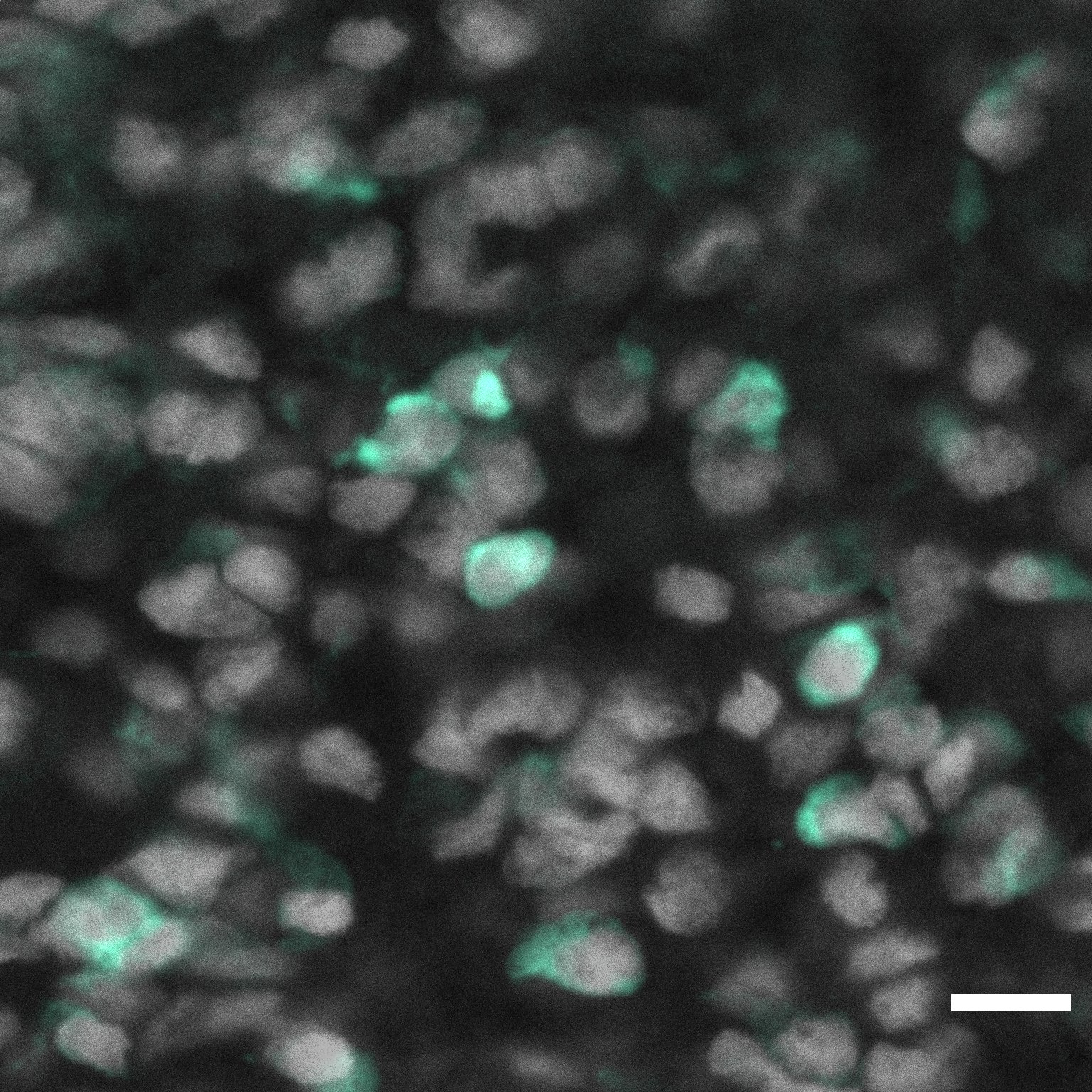

Supplement: Supplementary file 14 — Source data Fig. 7 [file 44318_2025_662_MOESM14_ESM.zip › Figure 7/7E/Main_figure_panel_Unc22_RNAi_dd940_rhod_SMEDWI_FITC_DAPI_20x_z3_FITC_channel.jpg]

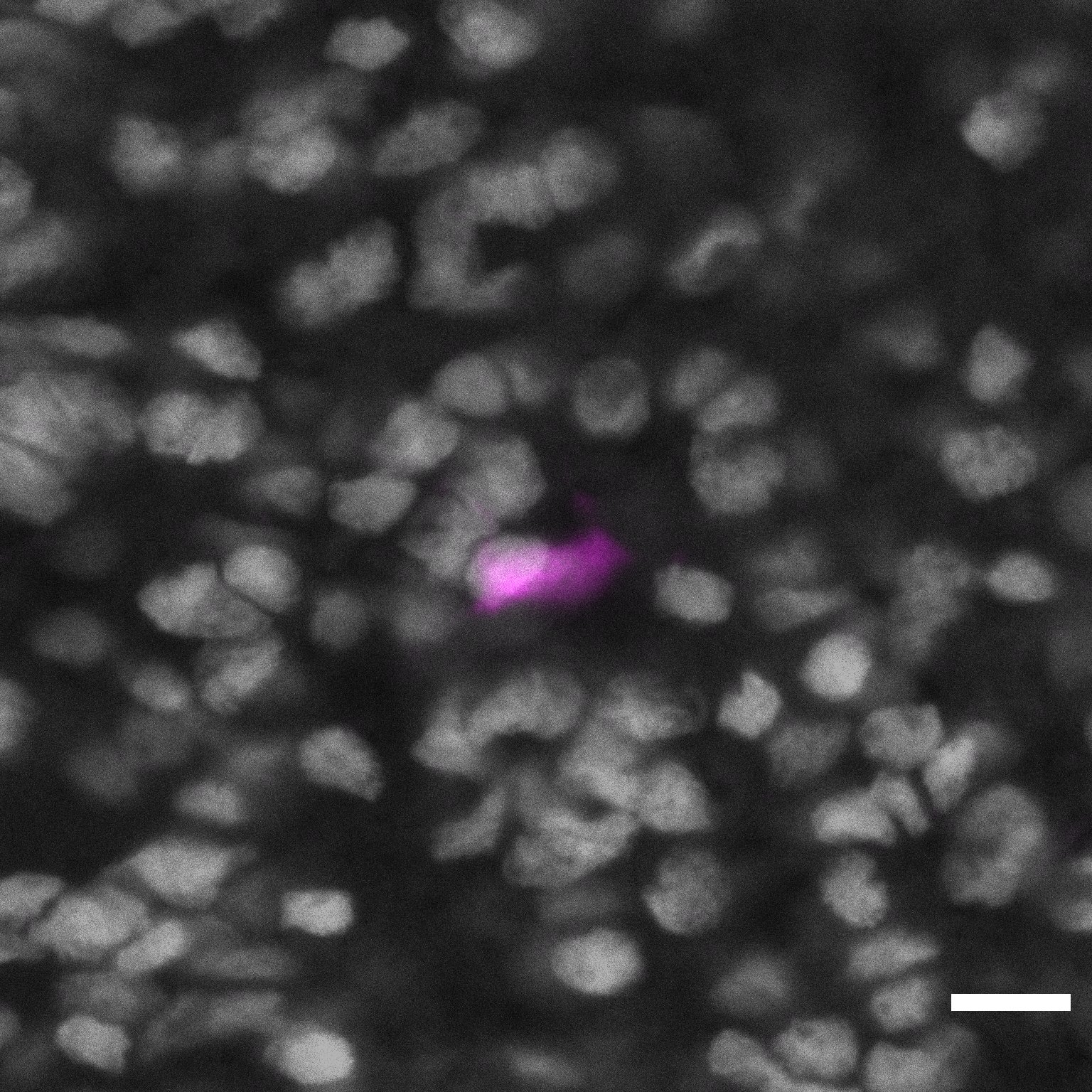

Supplement: Supplementary file 14 — Source data Fig. 7 [file 44318_2025_662_MOESM14_ESM.zip › Figure 7/7E/Main_figure_panel_Unc22_RNAi_dd940_rhod_SMEDWI_FITC_DAPI_20x_z3_Magenta_channel.jpg]

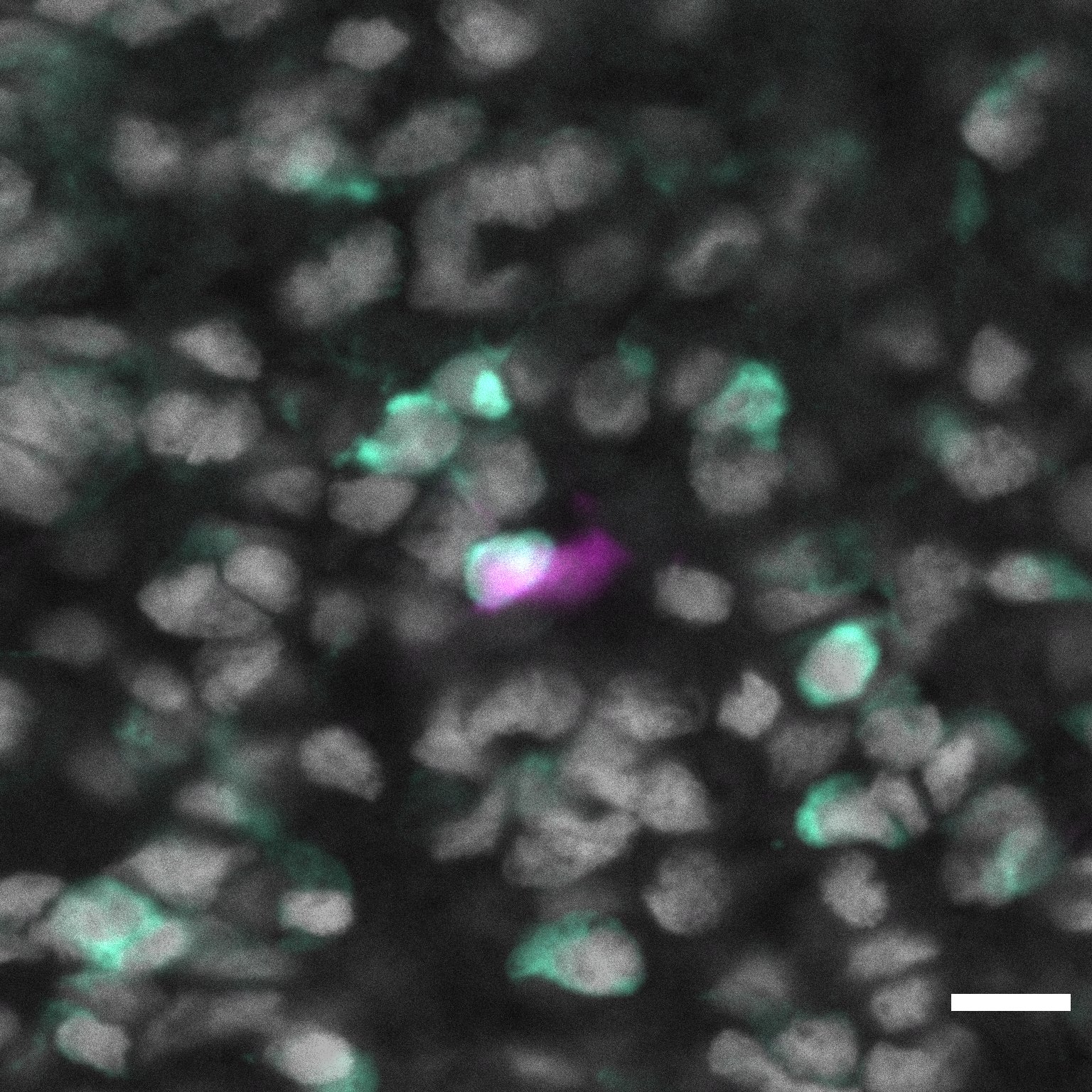

Supplement: Supplementary file 14 — Source data Fig. 7 [file 44318_2025_662_MOESM14_ESM.zip › Figure 7/7E/Main_figure_panel_Unc22_RNAi_dd940_rhod_SMEDWI_FITC_DAPI_20x_z3_Merged.jpg]

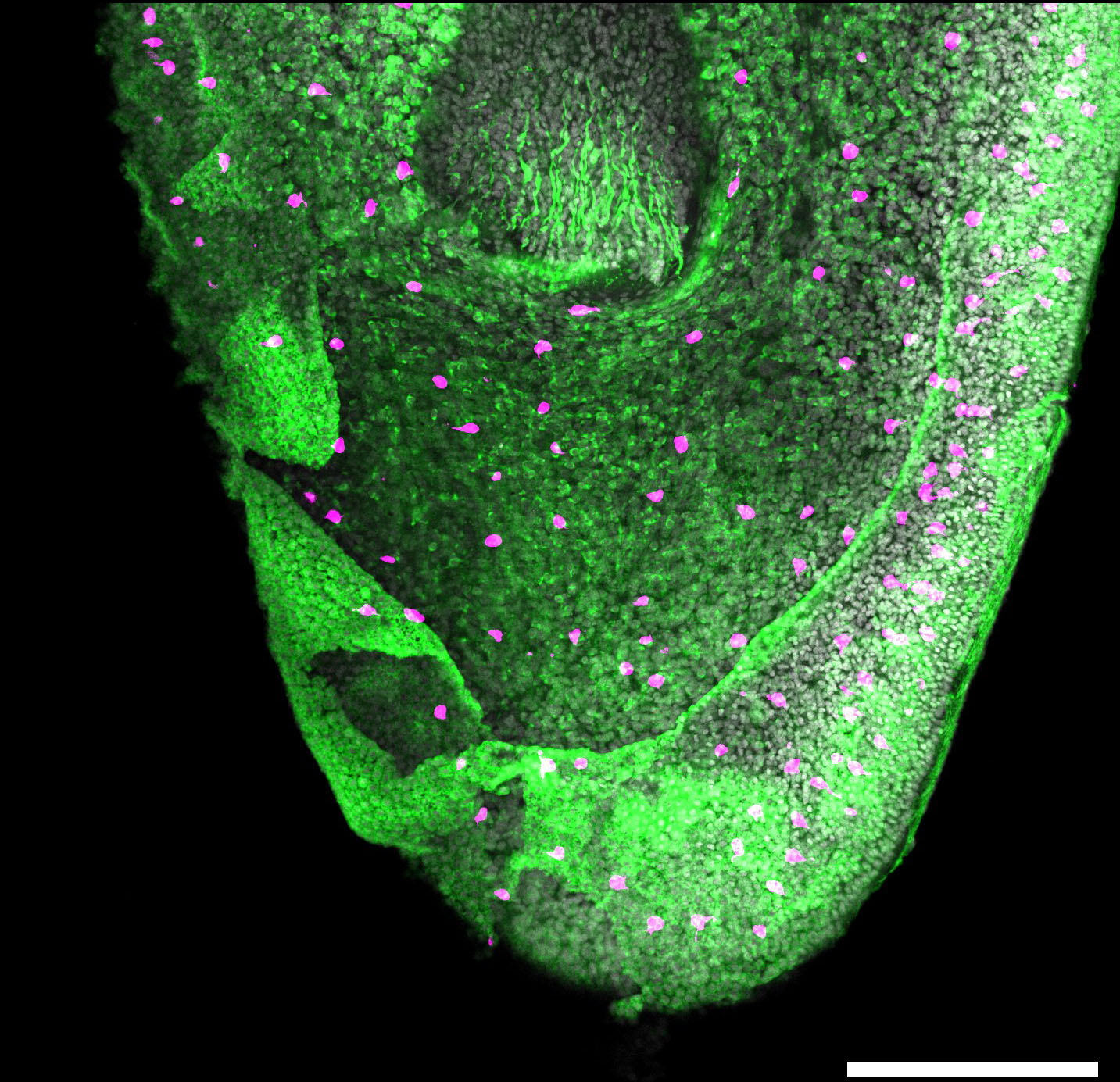

Supplement: Supplementary file 14 — Source data Fig. 7 [file 44318_2025_662_MOESM14_ESM.zip › Figure 7/7E/Max_projection_1_Control_RNAi_probe_dd940_rhod_SMEDWI_FITC_DAPI_20x_z1.jpg]

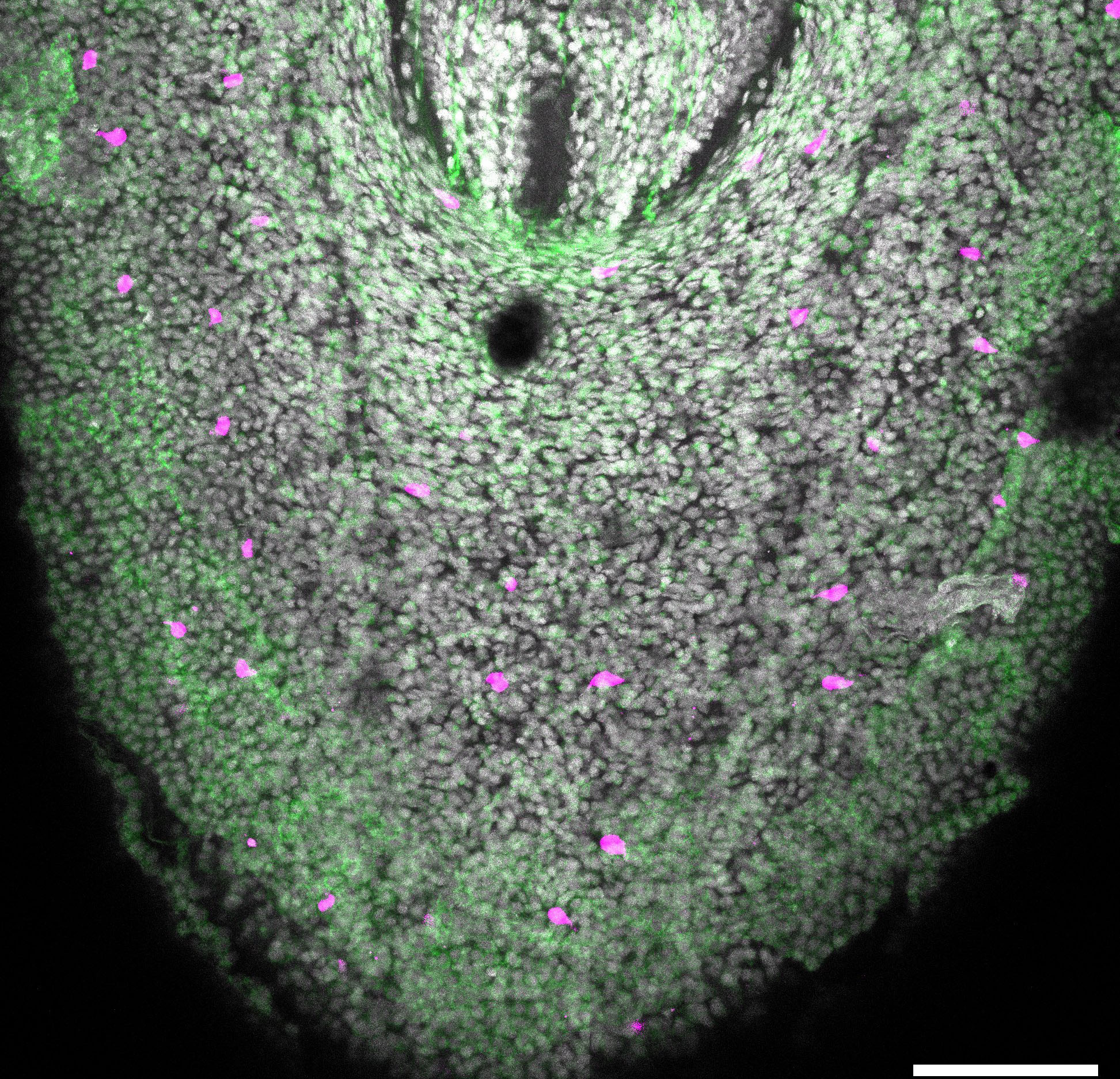

Supplement: Supplementary file 14 — Source data Fig. 7 [file 44318_2025_662_MOESM14_ESM.zip › Figure 7/7E/Max_projection_1_Triple_RNAi_probe_dd940_rhod_SMEDWI_FITC_DAPI_20x_z1.jpg]

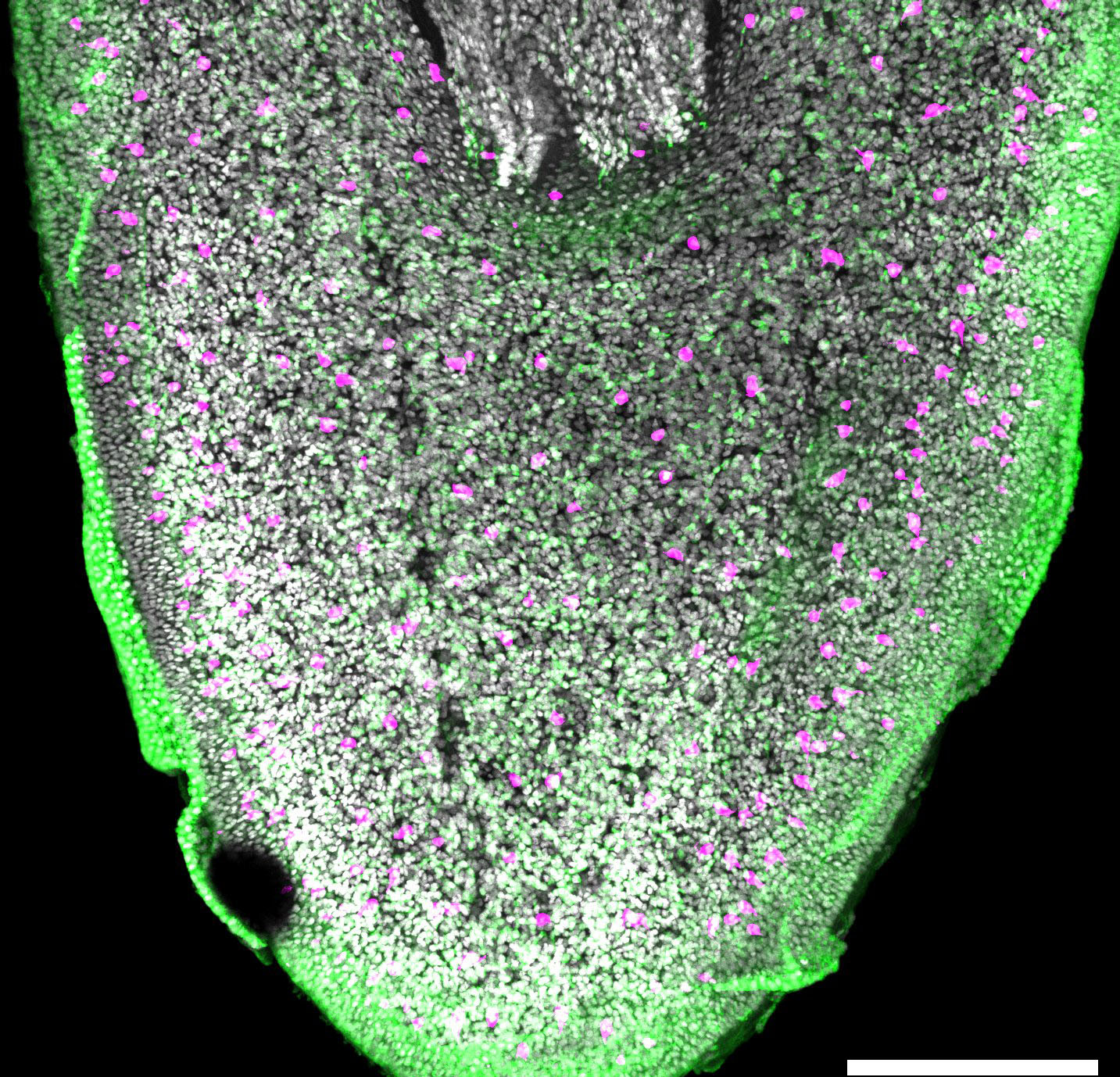

Supplement: Supplementary file 14 — Source data Fig. 7 [file 44318_2025_662_MOESM14_ESM.zip › Figure 7/7E/Max_projection_2_Control_RNAi_probe_dd940_rhod_SMEDWI_FITC_DAPI_20x_z1.jpg]

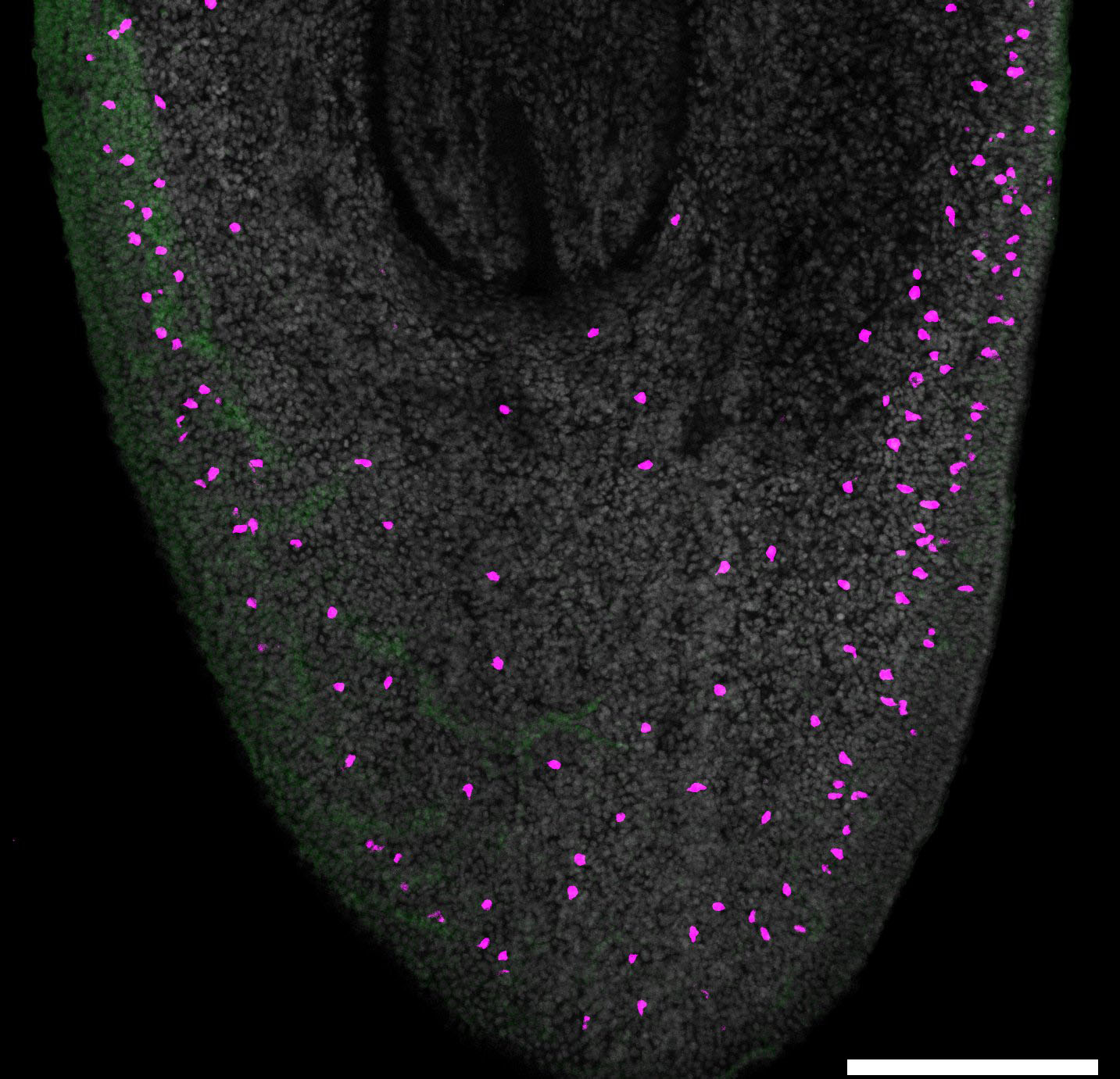

Supplement: Supplementary file 14 — Source data Fig. 7 [file 44318_2025_662_MOESM14_ESM.zip › Figure 7/7E/Max_projection_2_Triple_RNAi_probe_dd940_rhod_SMEDWI_FITC_DAPI_20x_z1.jpg]

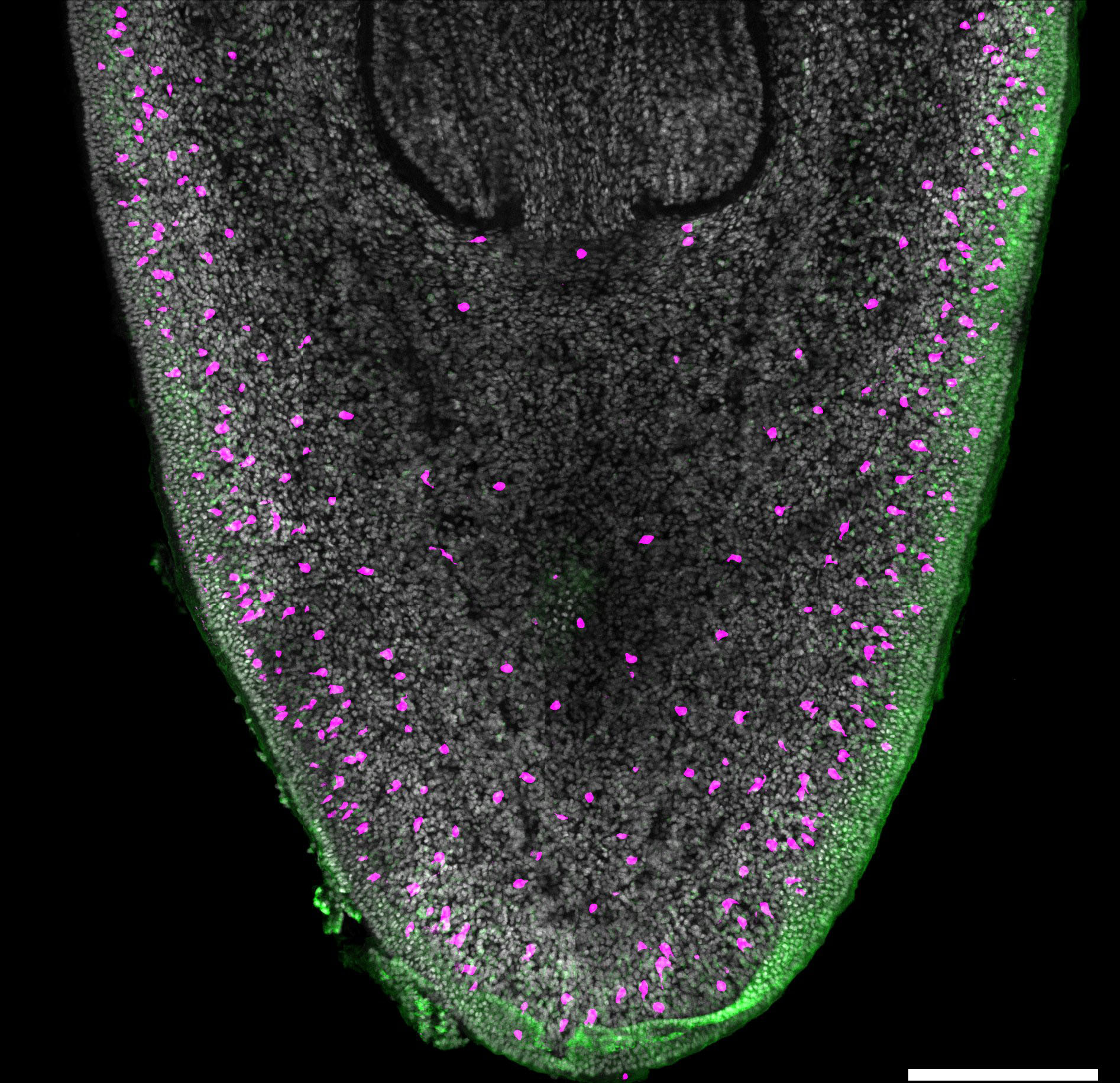

Supplement: Supplementary file 14 — Source data Fig. 7 [file 44318_2025_662_MOESM14_ESM.zip › Figure 7/7E/Max_projection_3_Control_RNAi_probe_dd940_rhod_SMEDWI_FITC_DAPI_20x_z1.jpg]

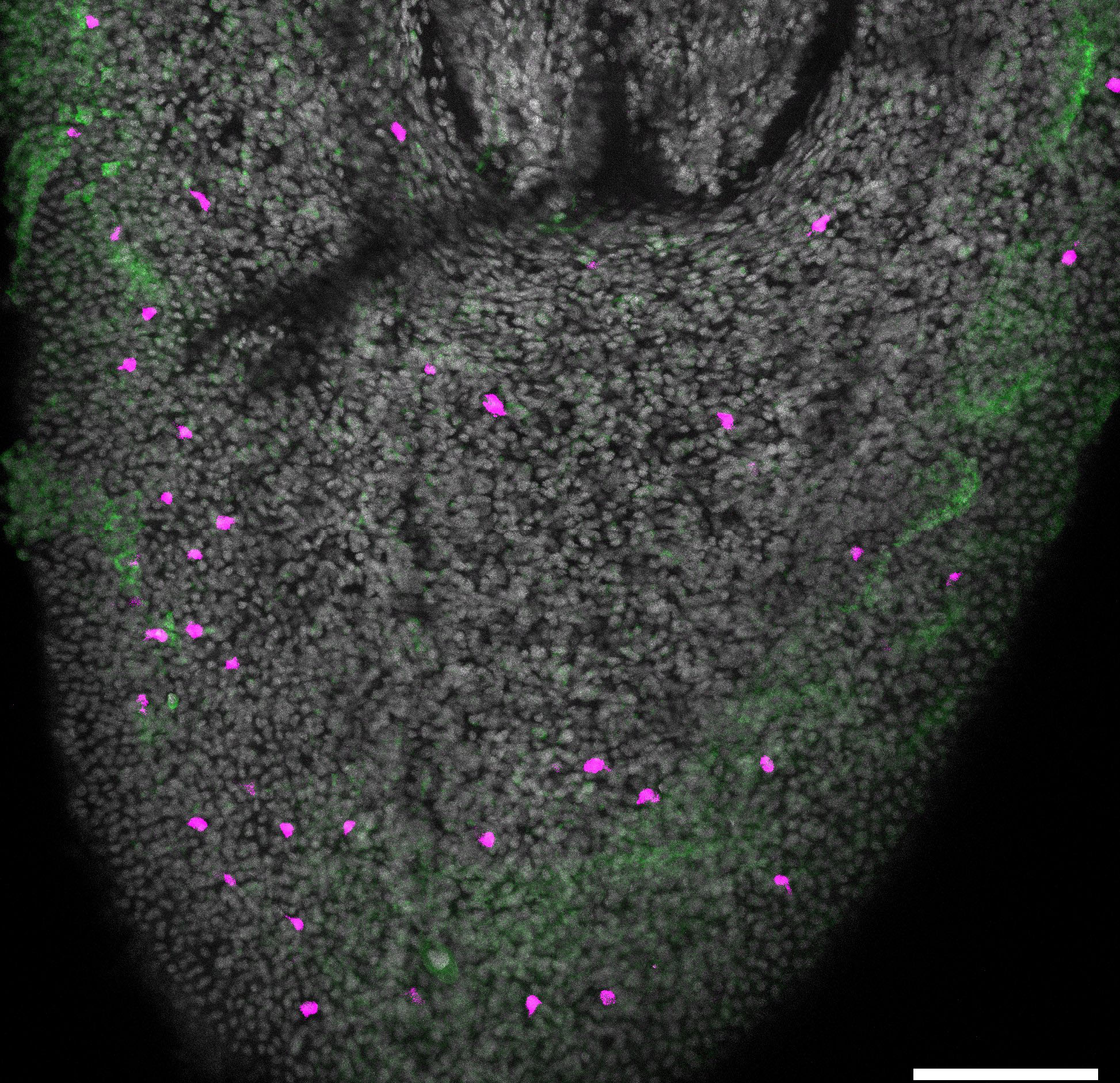

Supplement: Supplementary file 14 — Source data Fig. 7 [file 44318_2025_662_MOESM14_ESM.zip › Figure 7/7E/Max_projection_3_Triple_RNAi_probe_dd940_rhod_SMEDWI_FITC_DAPI_20x_z1.jpg]

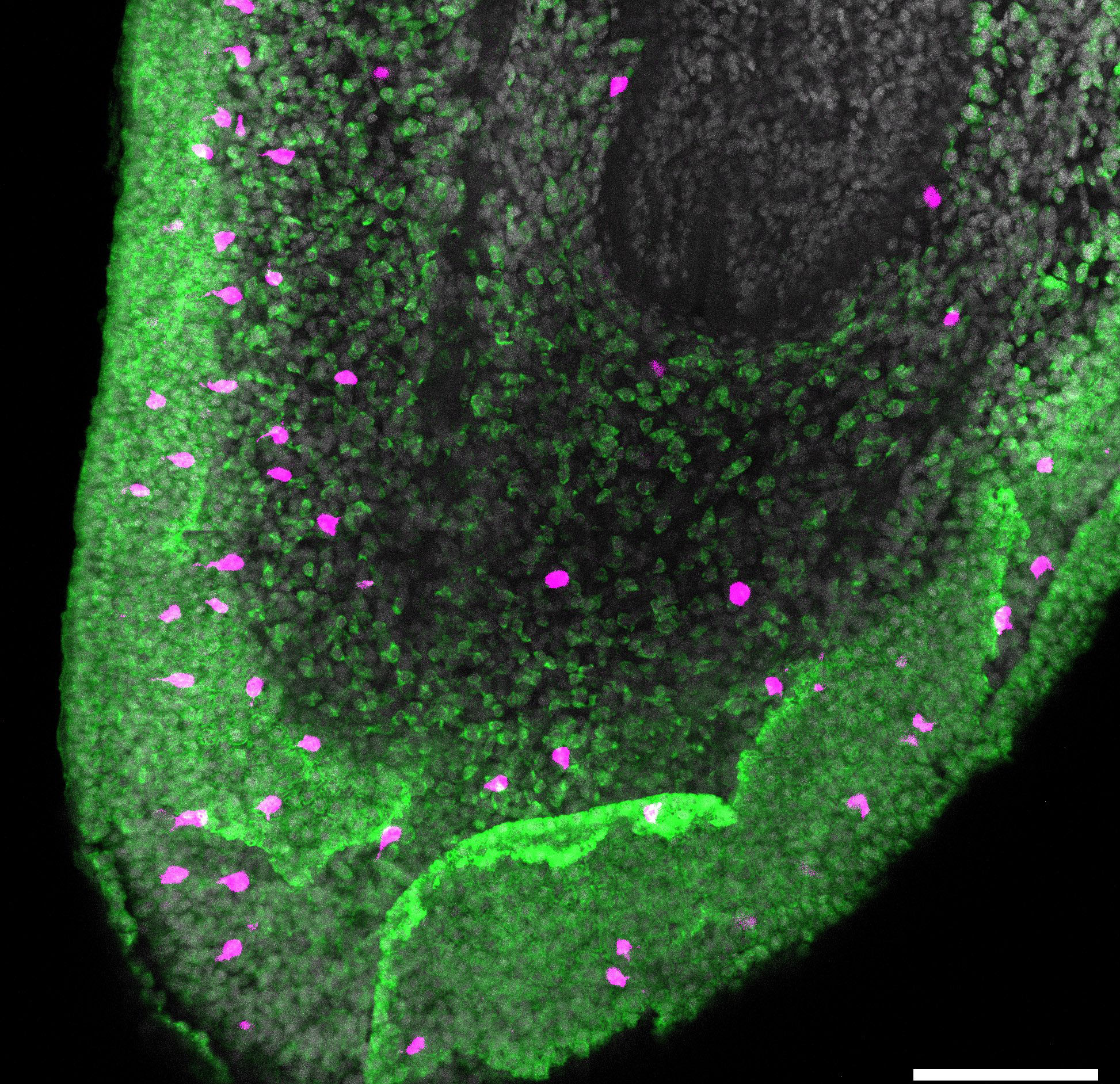

Supplement: Supplementary file 14 — Source data Fig. 7 [file 44318_2025_662_MOESM14_ESM.zip › Figure 7/7E/Max_projection_4_Control_RNAi_probe_dd940_rhod_SMEDWI_FITC_DAPI_20x_z1.jpg]

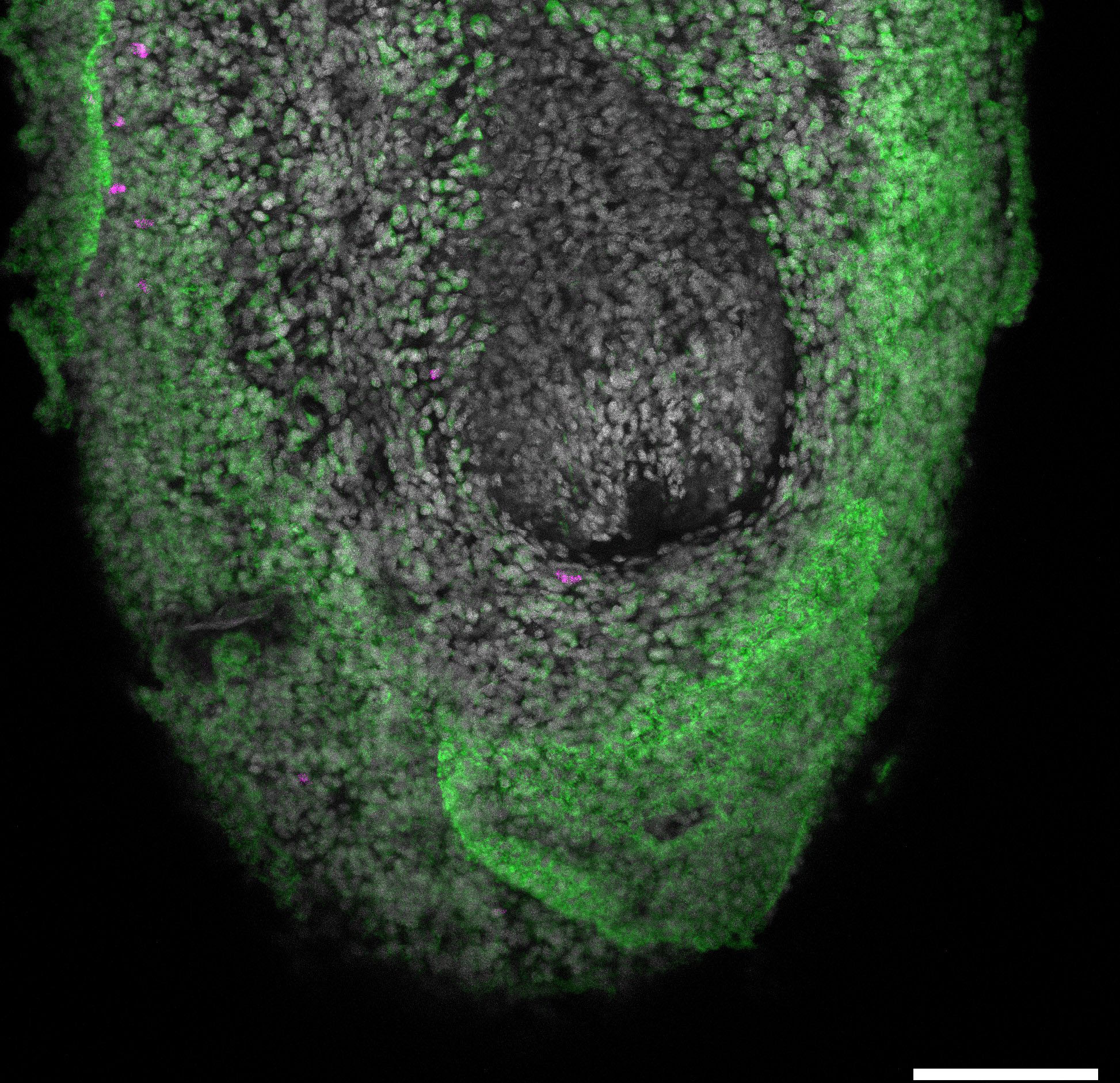

Supplement: Supplementary file 14 — Source data Fig. 7 [file 44318_2025_662_MOESM14_ESM.zip › Figure 7/7E/Max_projection_4_Triple_RNAi_probe_dd940_rhod_SMEDWI_FITC_DAPI_20x_z1.jpg]

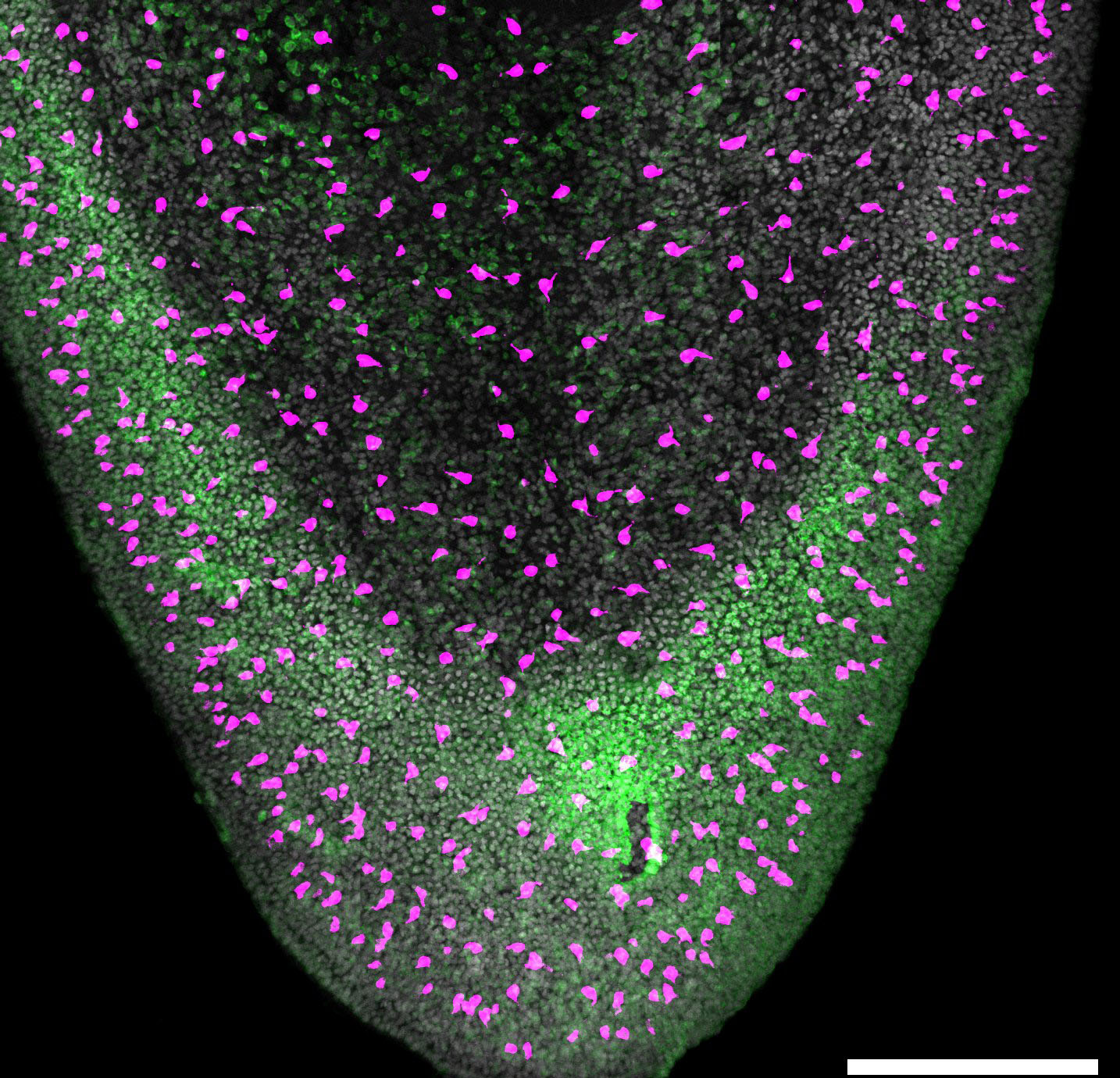

Supplement: Supplementary file 14 — Source data Fig. 7 [file 44318_2025_662_MOESM14_ESM.zip › Figure 7/7E/Max_projection_5_Control_RNAi_probe_dd940_rhod_SMEDWI_FITC_DAPI_20x_z1.jpg]

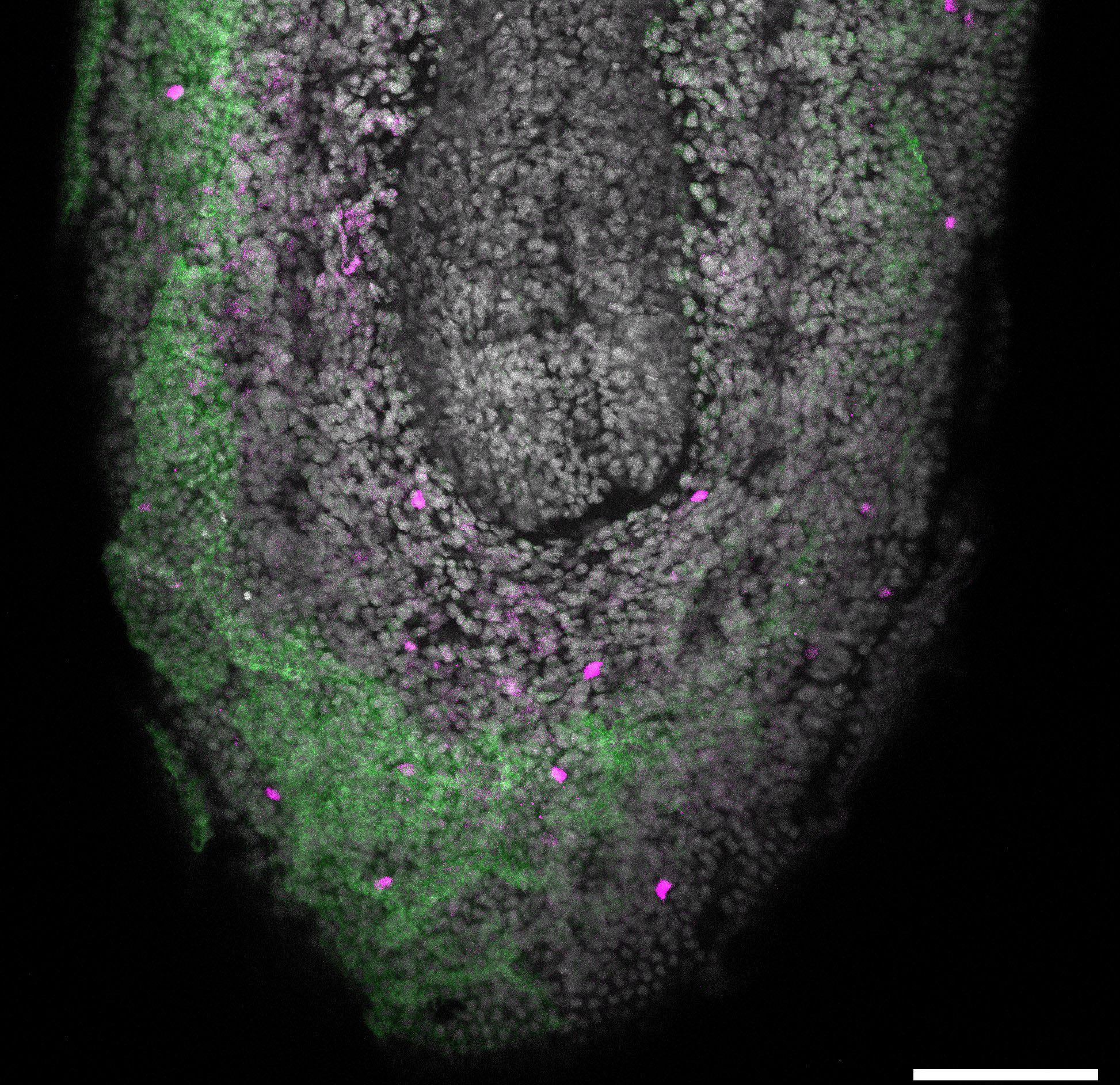

Supplement: Supplementary file 14 — Source data Fig. 7 [file 44318_2025_662_MOESM14_ESM.zip › Figure 7/7E/Max_projection_5_Triple_RNAi_probe_dd940_rhod_SMEDWI_FITC_DAPI_20x_z1.jpg]

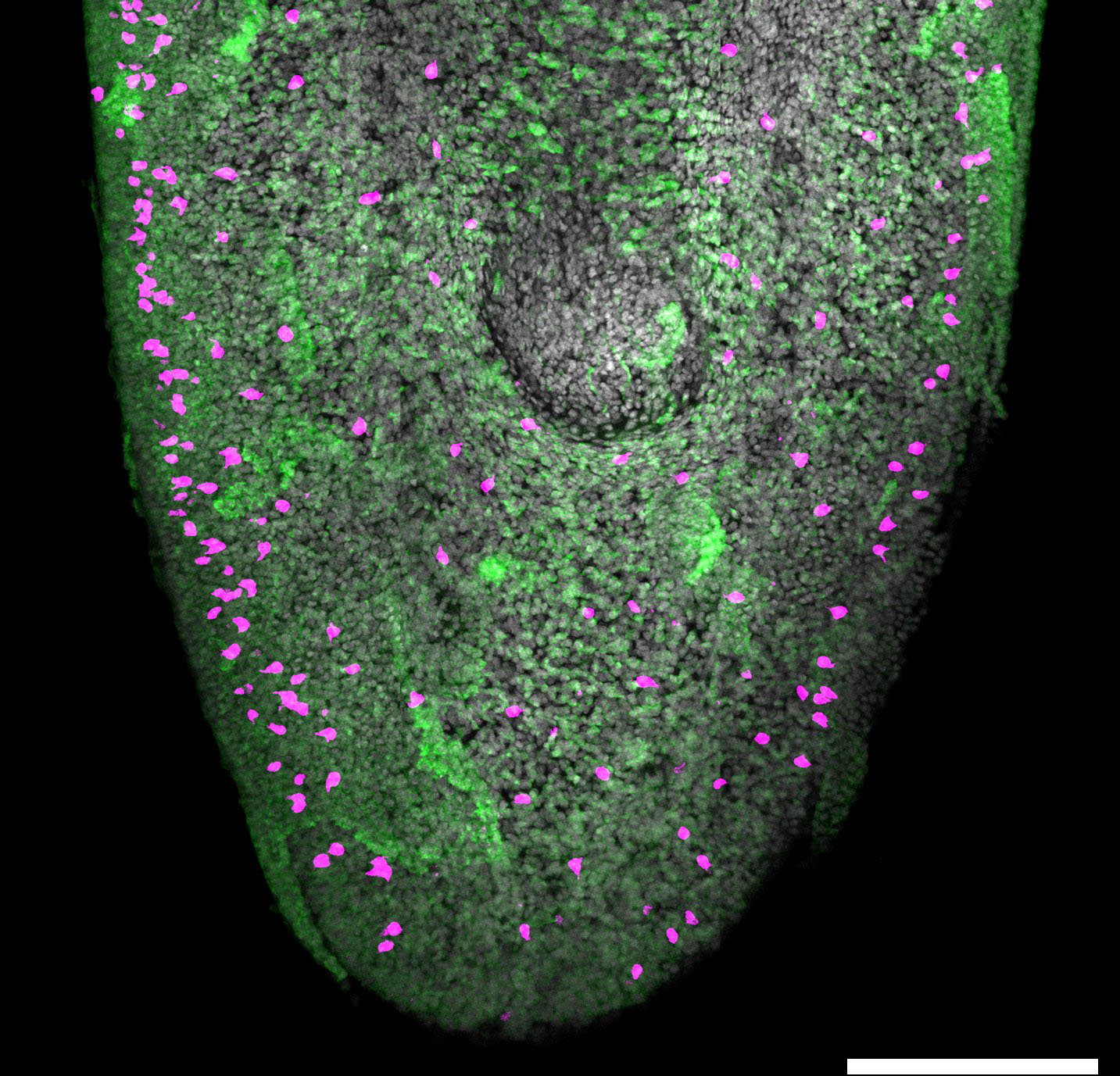

Supplement: Supplementary file 14 — Source data Fig. 7 [file 44318_2025_662_MOESM14_ESM.zip › Figure 7/7E/Max_projection_6_Control_RNAi_probe_dd940_rhod_SMEDWI_FITC_DAPI_20x_z1.jpg]

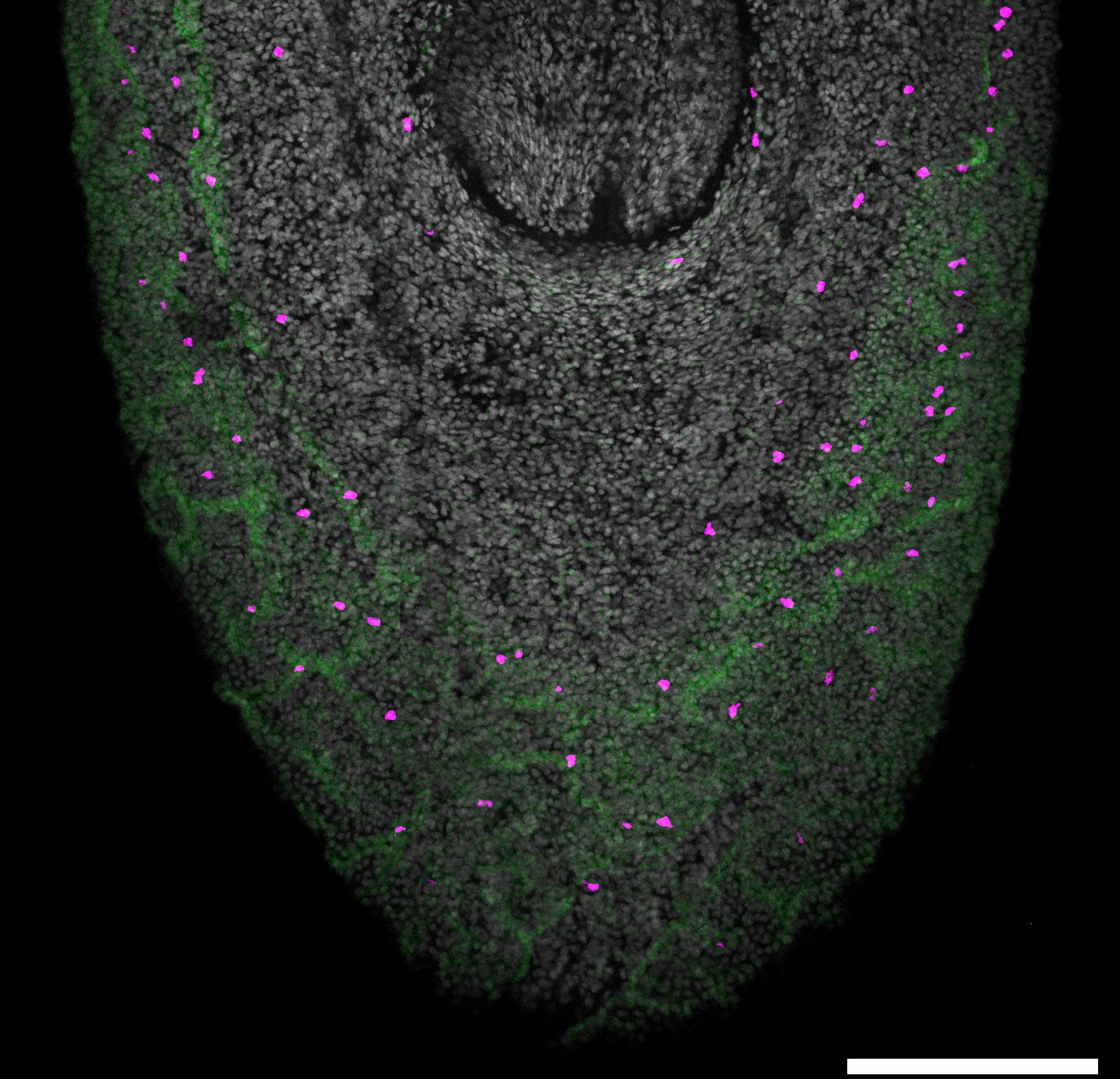

Supplement: Supplementary file 14 — Source data Fig. 7 [file 44318_2025_662_MOESM14_ESM.zip › Figure 7/7E/Max_projection_6_Triple_RNAi_probe_dd940_rhod_SMEDWI_FITC_DAPI_20x_z1.jpg]

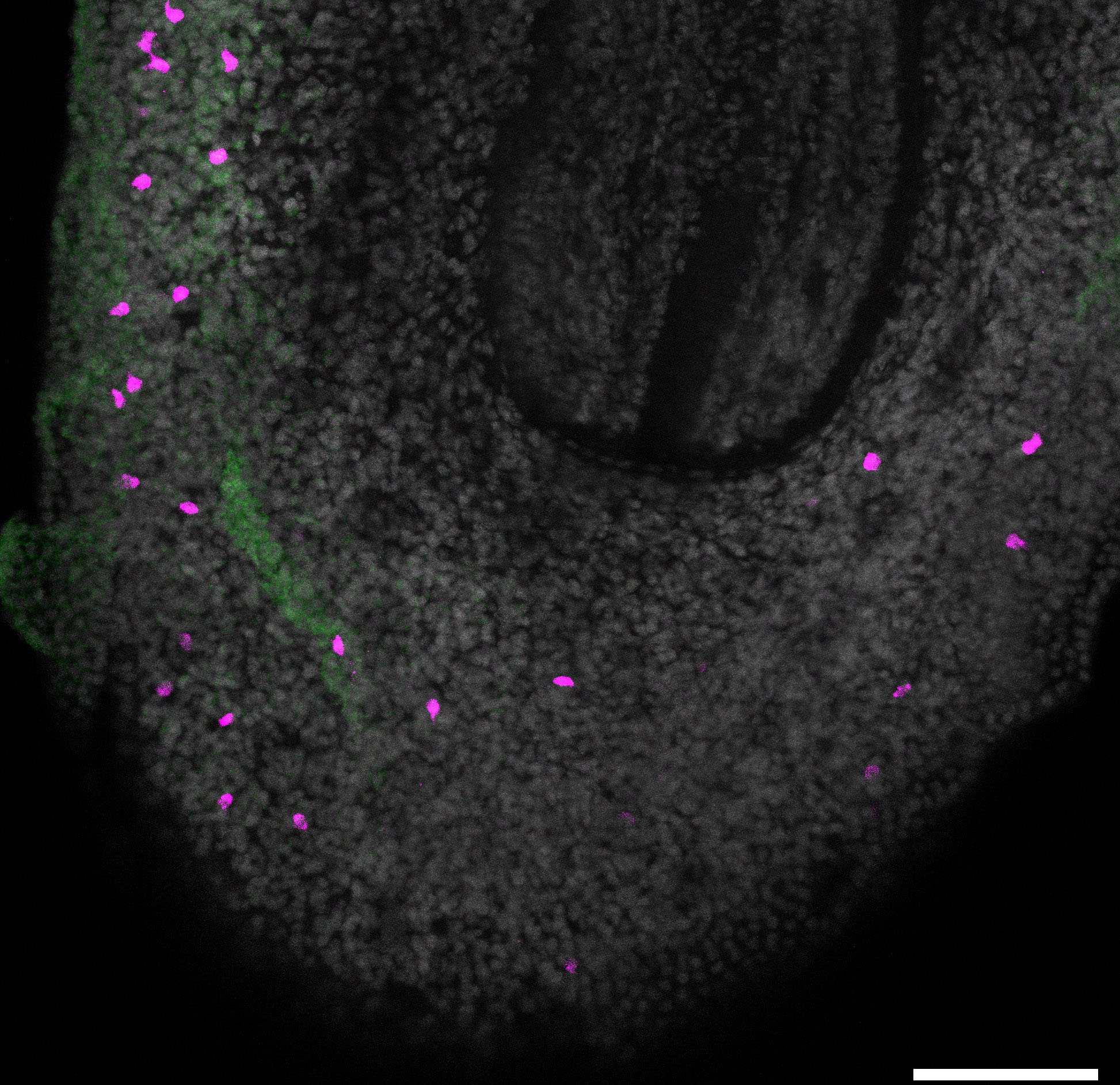

Supplement: Supplementary file 14 — Source data Fig. 7 [file 44318_2025_662_MOESM14_ESM.zip › Figure 7/7E/Max_projection_7_Triple_RNAi_probe_dd940_rhod_SMEDWI_FITC_DAPI_20x_z1.jpg]

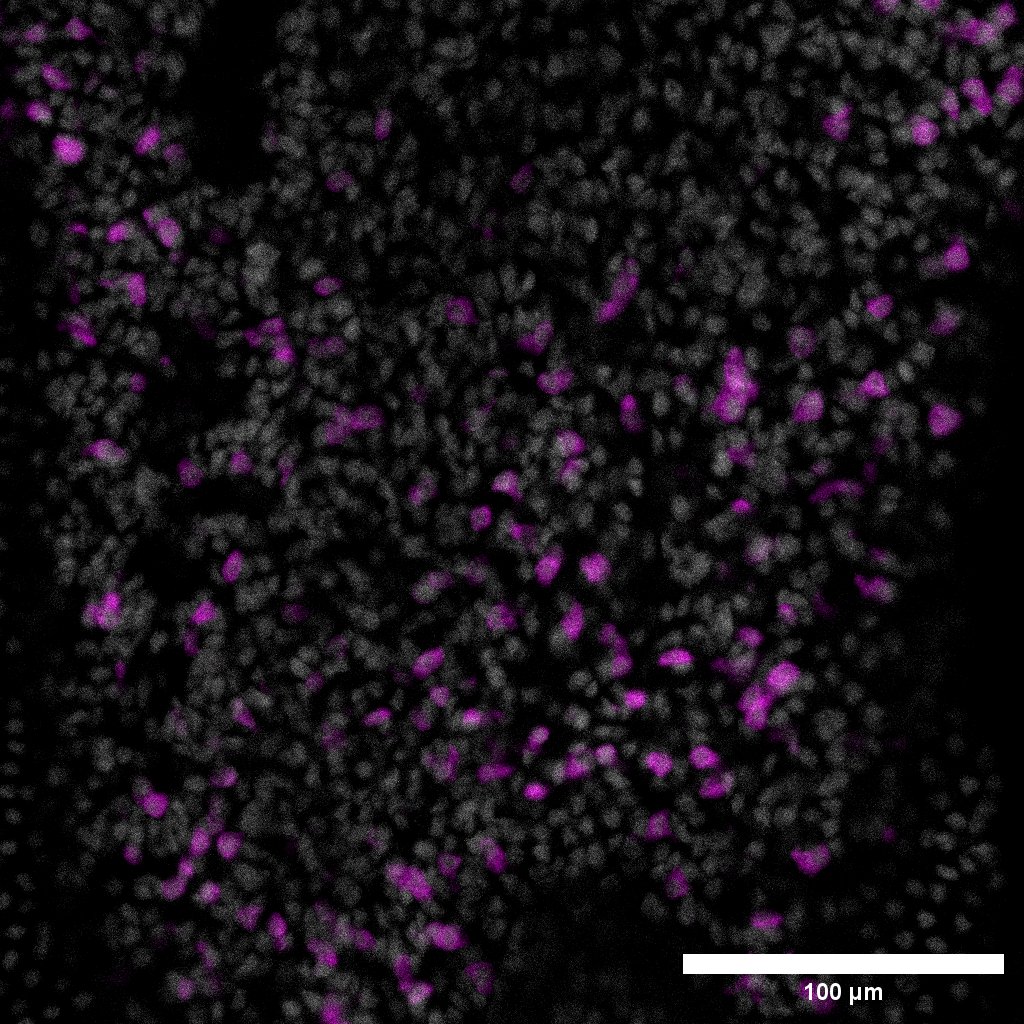

Supplement: Supplementary file 14 — Source data Fig. 7 [file 44318_2025_662_MOESM14_ESM.zip › Figure 7/7F/ID_10_Control_RNAi_Probe_prog2_rhod_DAPI_20x_z1.jpg]

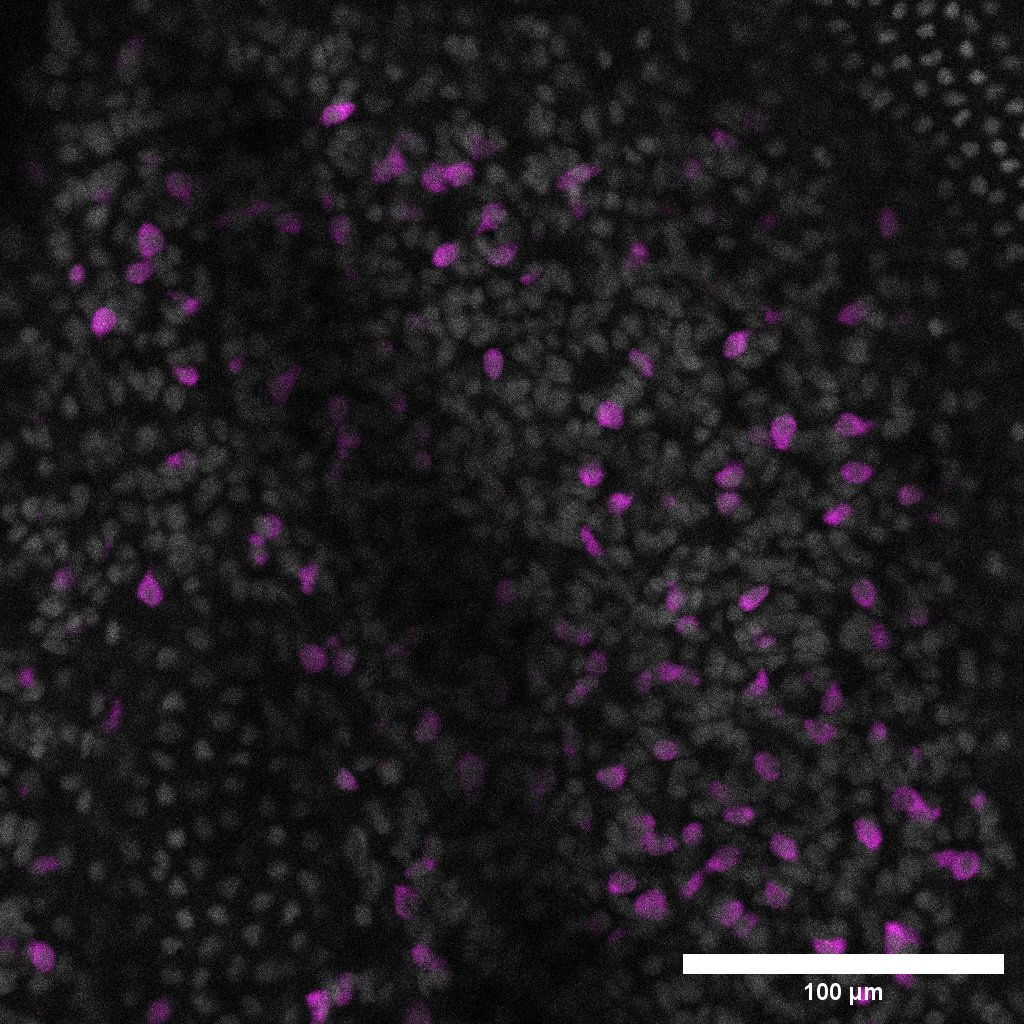

Supplement: Supplementary file 14 — Source data Fig. 7 [file 44318_2025_662_MOESM14_ESM.zip › Figure 7/7F/ID_10_Triple_RNAi_Probe_prog2_rhod_DAPI_20x_z1.jpg]

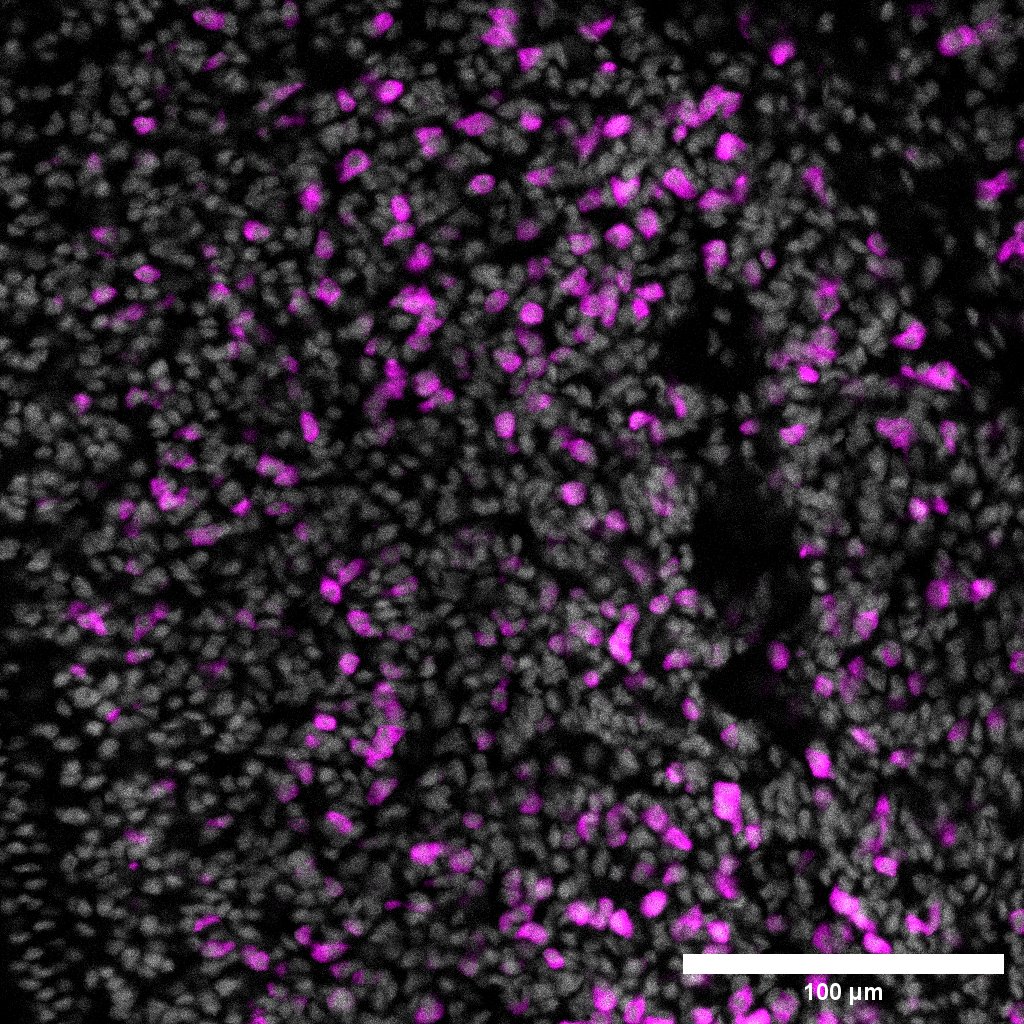

Supplement: Supplementary file 14 — Source data Fig. 7 [file 44318_2025_662_MOESM14_ESM.zip › Figure 7/7F/ID_1_Control_RNAi_Probe_prog2_rhod_DAPI_20x_z1.jpg]

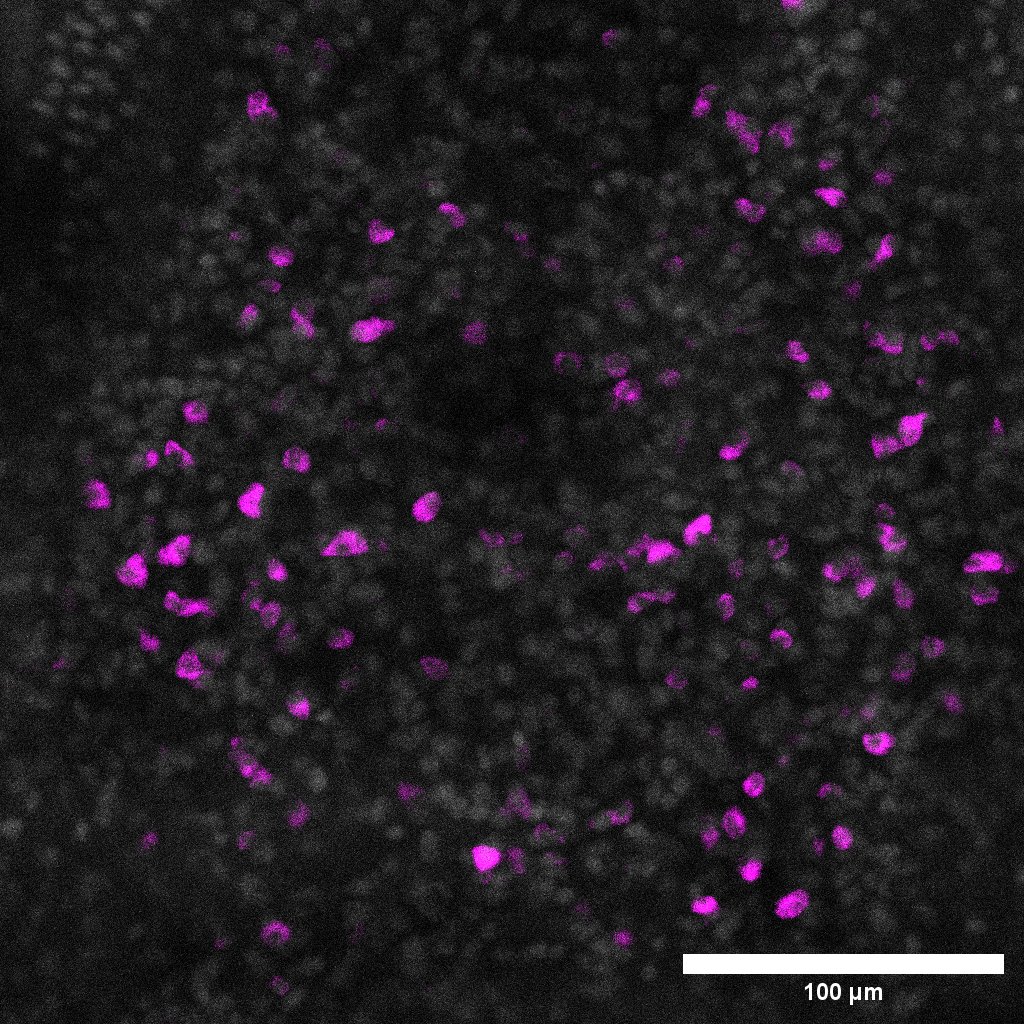

Supplement: Supplementary file 14 — Source data Fig. 7 [file 44318_2025_662_MOESM14_ESM.zip › Figure 7/7F/ID_1_Triple_RNAi_Probe_prog2_rhod_DAPI_20x_z1.jpg]

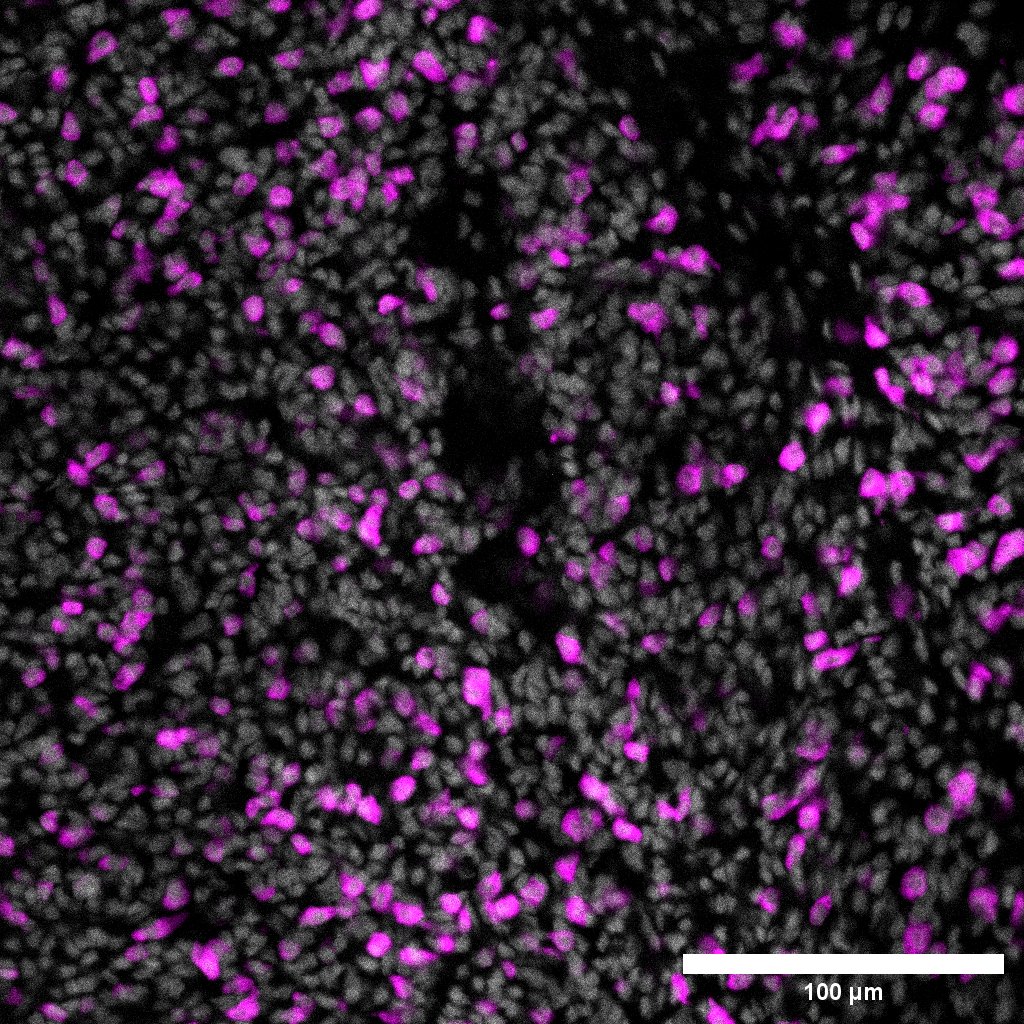

Supplement: Supplementary file 14 — Source data Fig. 7 [file 44318_2025_662_MOESM14_ESM.zip › Figure 7/7F/ID_2_Control_RNAi_Probe_prog2_rhod_DAPI_20x_z1.jpg]

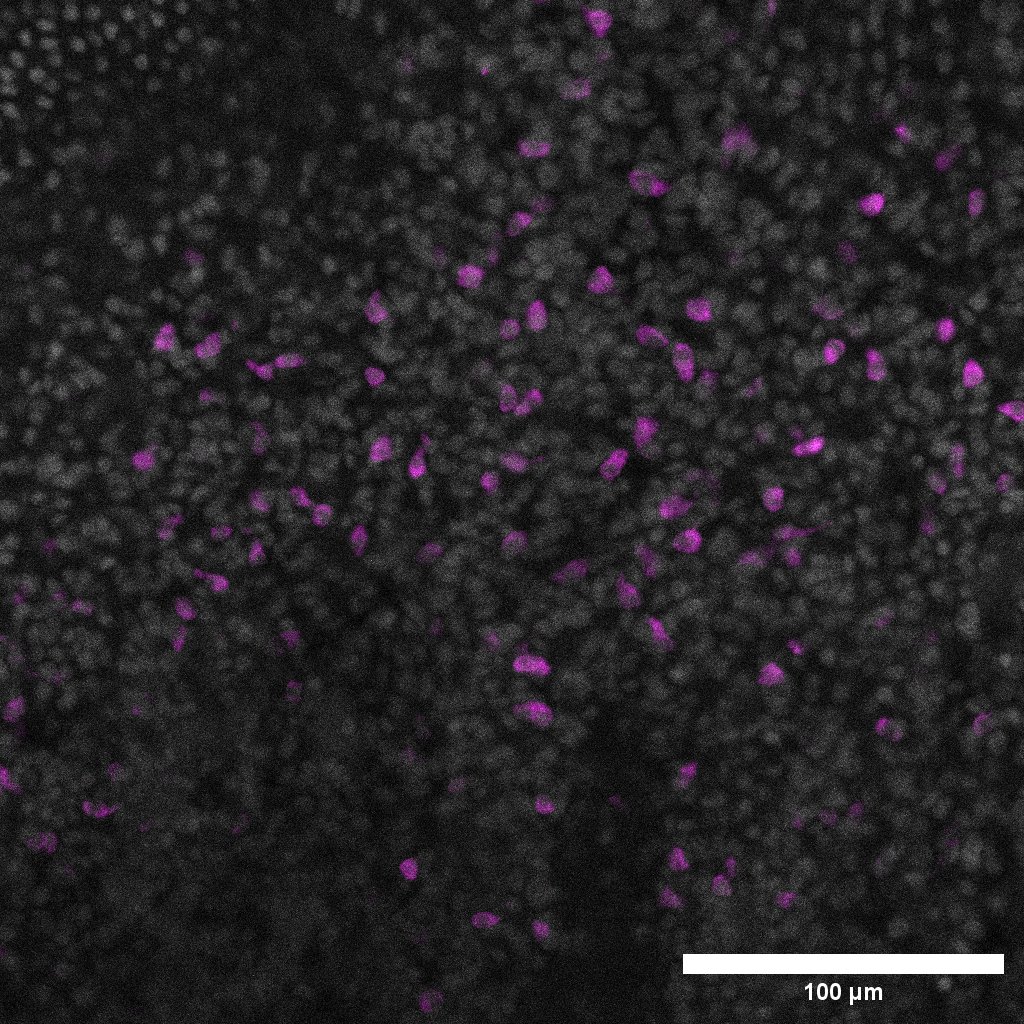

Supplement: Supplementary file 14 — Source data Fig. 7 [file 44318_2025_662_MOESM14_ESM.zip › Figure 7/7F/ID_2_Triple_RNAi_Probe_prog2_rhod_DAPI_20x_z1.jpg]

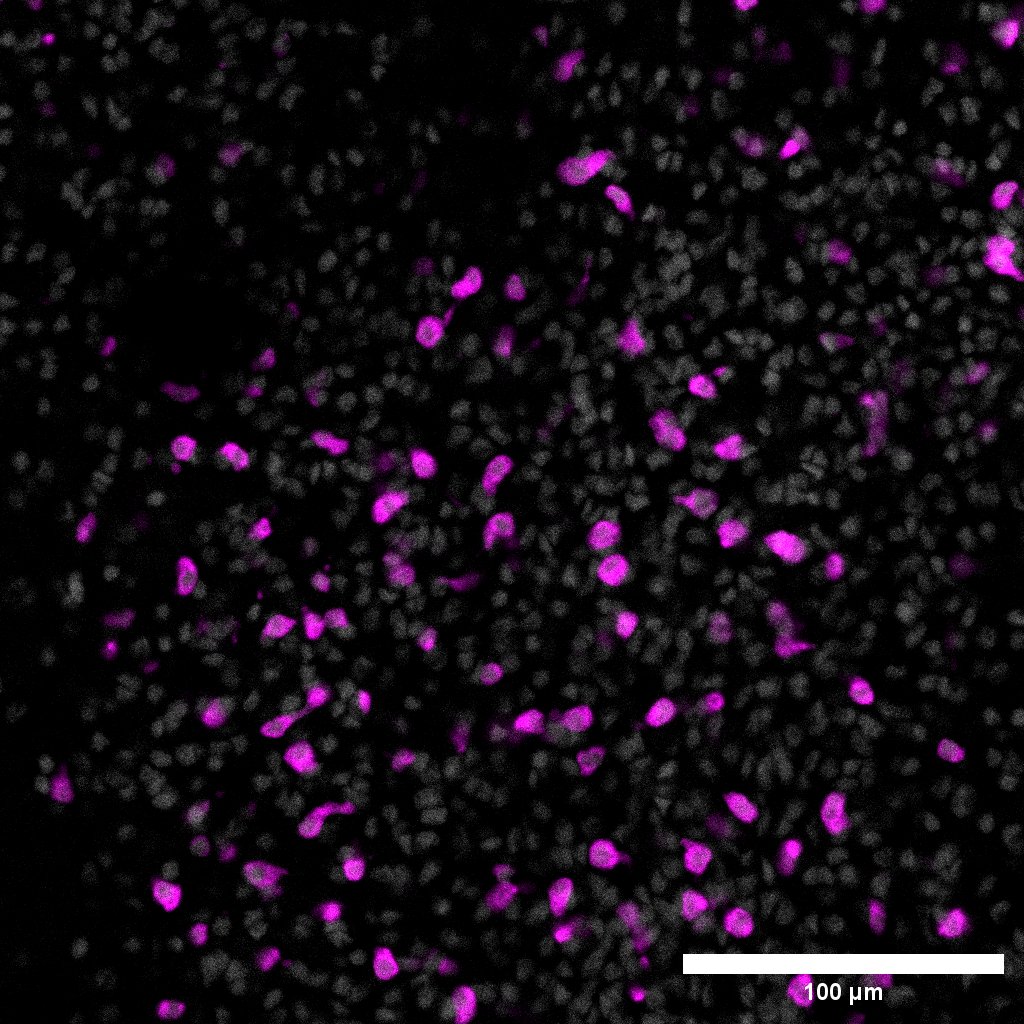

Supplement: Supplementary file 14 — Source data Fig. 7 [file 44318_2025_662_MOESM14_ESM.zip › Figure 7/7F/ID_3_Control_RNAi_Probe_prog2_rhod_DAPI_20x_z1.jpg]

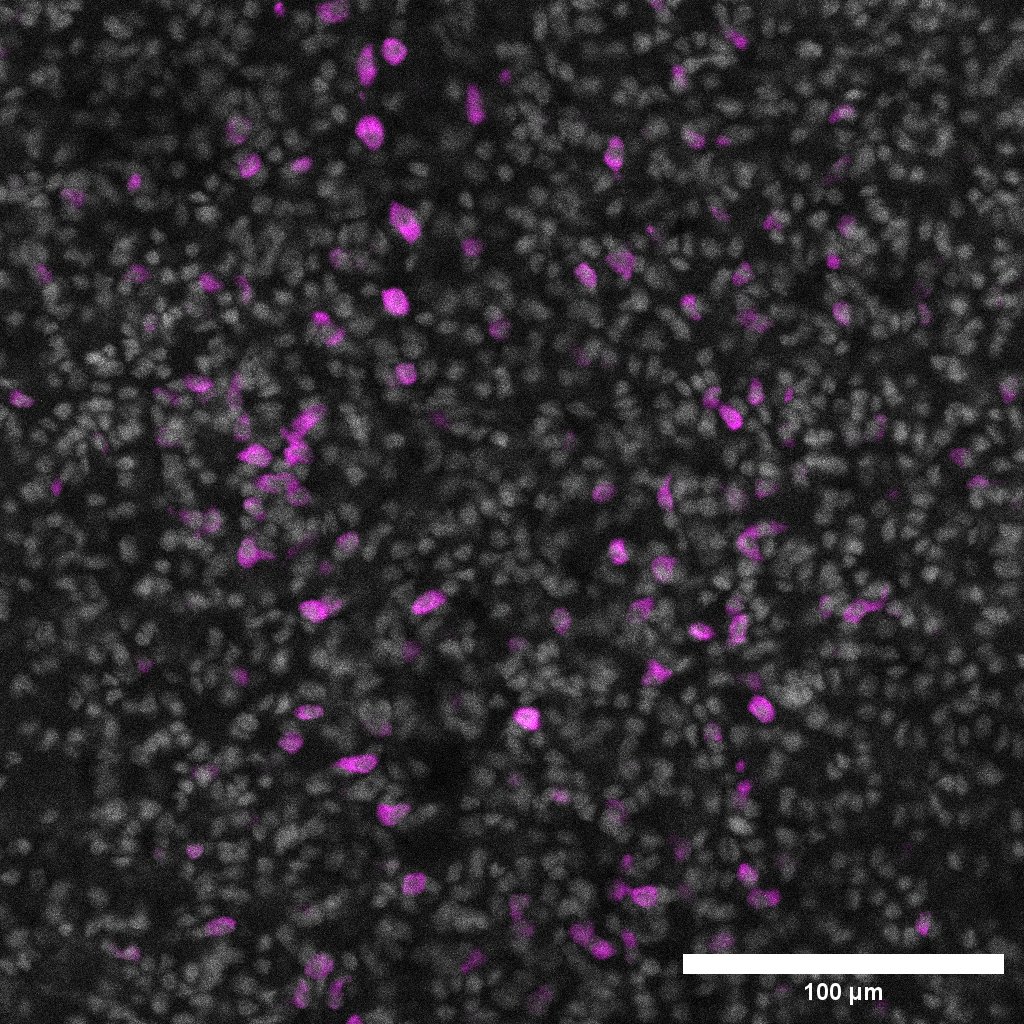

Supplement: Supplementary file 14 — Source data Fig. 7 [file 44318_2025_662_MOESM14_ESM.zip › Figure 7/7F/ID_3_Triple_RNAi_Probe_prog2_rhod_DAPI_20x_z1.jpg]

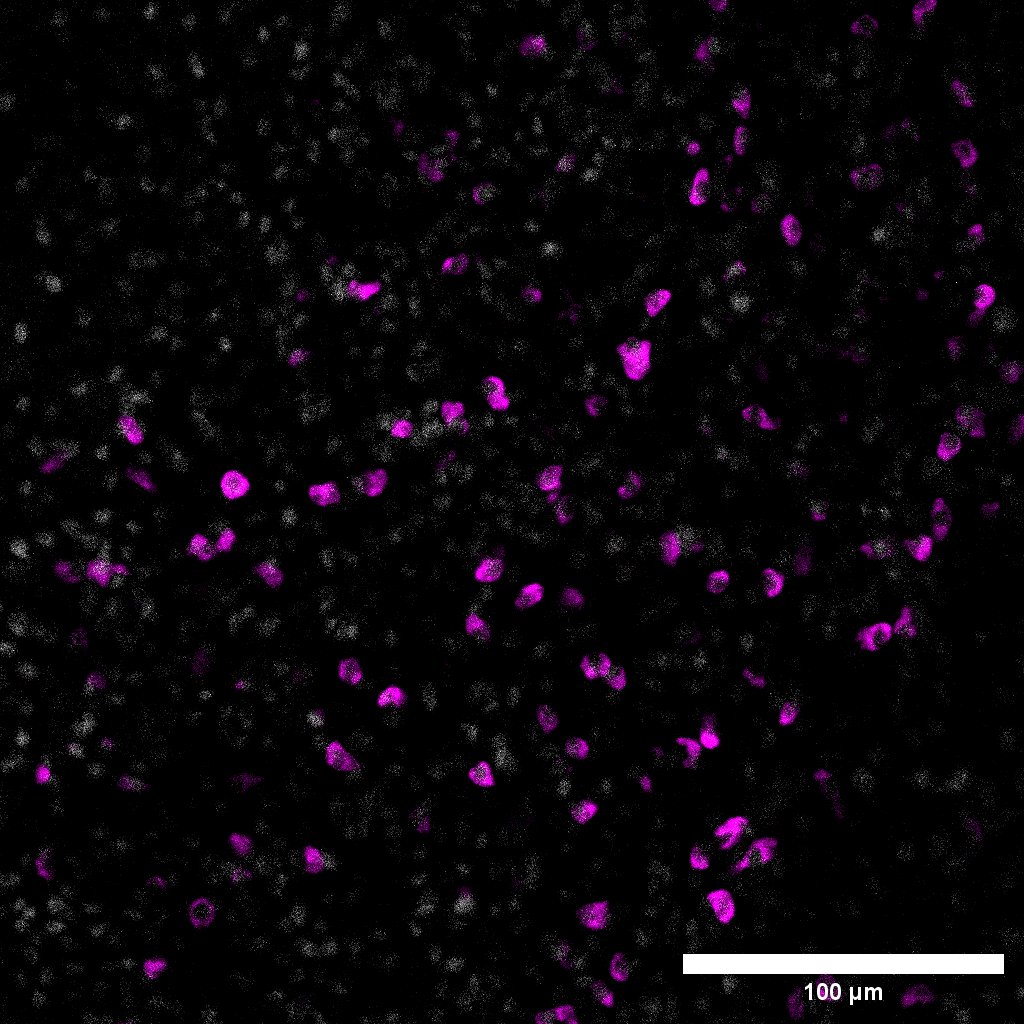

Supplement: Supplementary file 14 — Source data Fig. 7 [file 44318_2025_662_MOESM14_ESM.zip › Figure 7/7F/ID_4_Control_RNAi_Probe_prog2_rhod_DAPI_20x_z1.jpg]

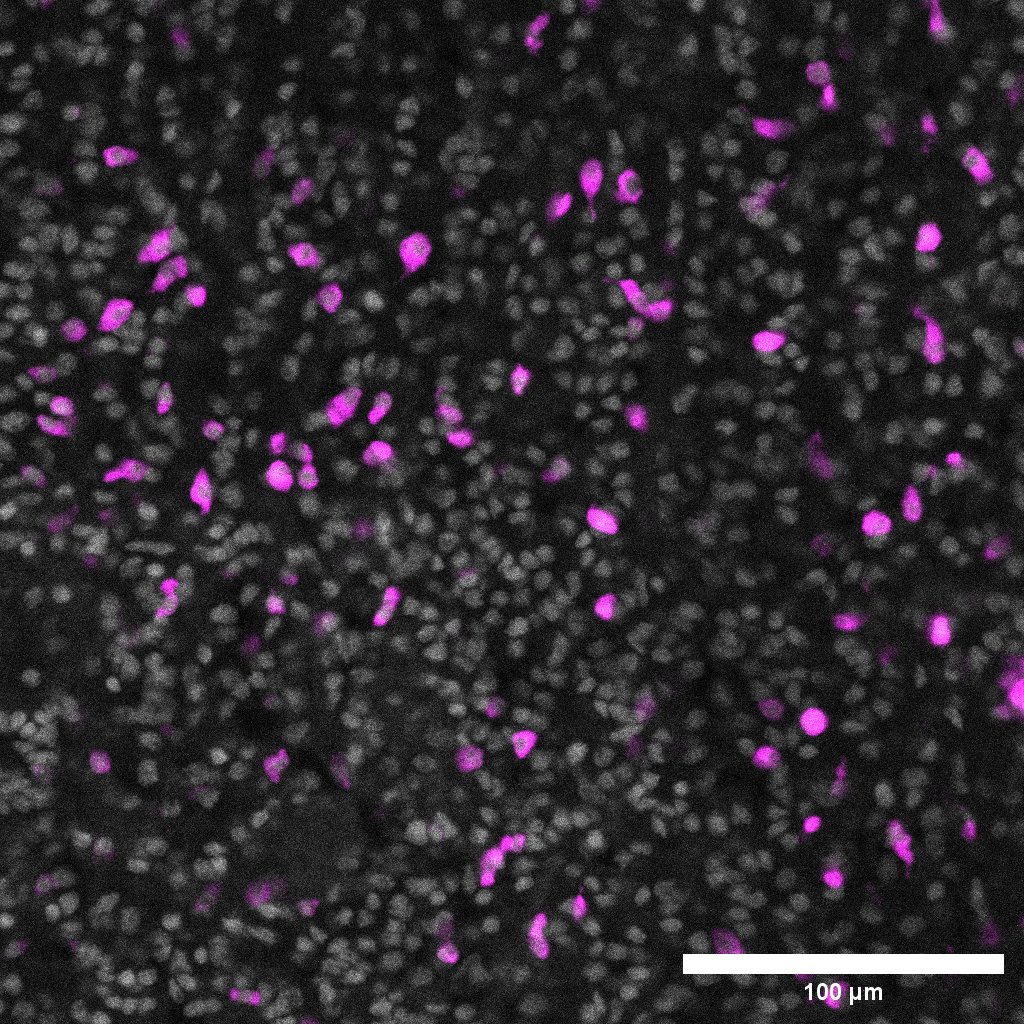

Supplement: Supplementary file 14 — Source data Fig. 7 [file 44318_2025_662_MOESM14_ESM.zip › Figure 7/7F/ID_4_Triple_RNAi_Probe_prog2_rhod_DAPI_20x_z1.jpg]

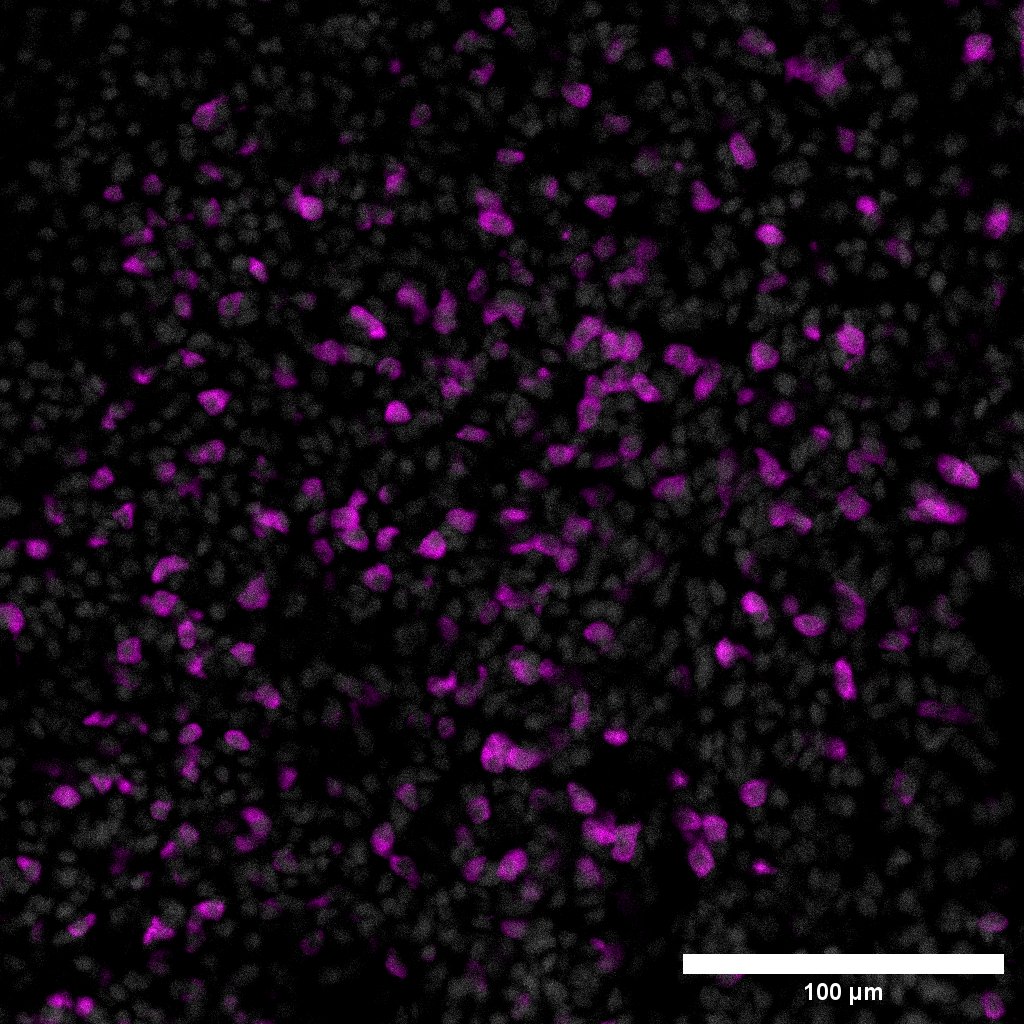

Supplement: Supplementary file 14 — Source data Fig. 7 [file 44318_2025_662_MOESM14_ESM.zip › Figure 7/7F/ID_5_Control_RNAi_Probe_prog2_rhod_DAPI_20x_z1.jpg]

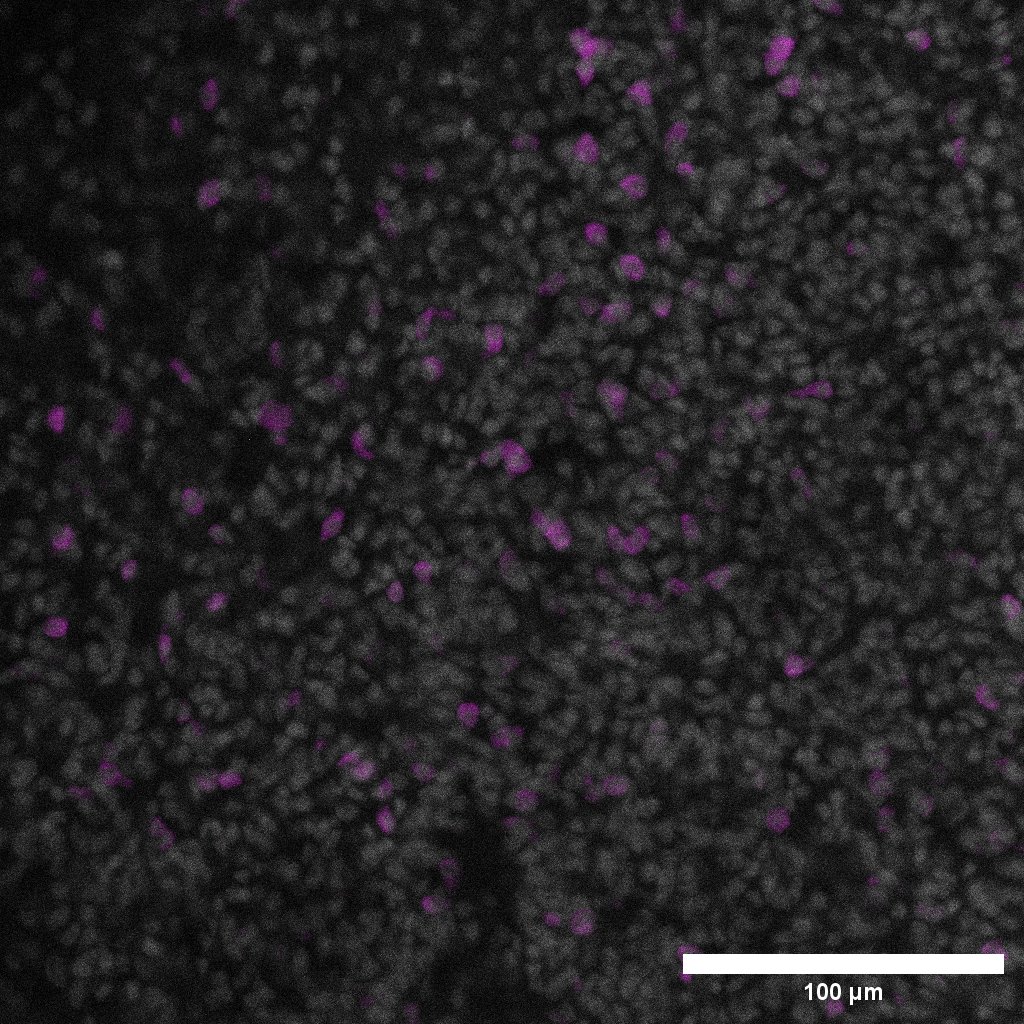

Supplement: Supplementary file 14 — Source data Fig. 7 [file 44318_2025_662_MOESM14_ESM.zip › Figure 7/7F/ID_5_Triple_RNAi_Probe_prog2_rhod_DAPI_20x_z1.jpg]

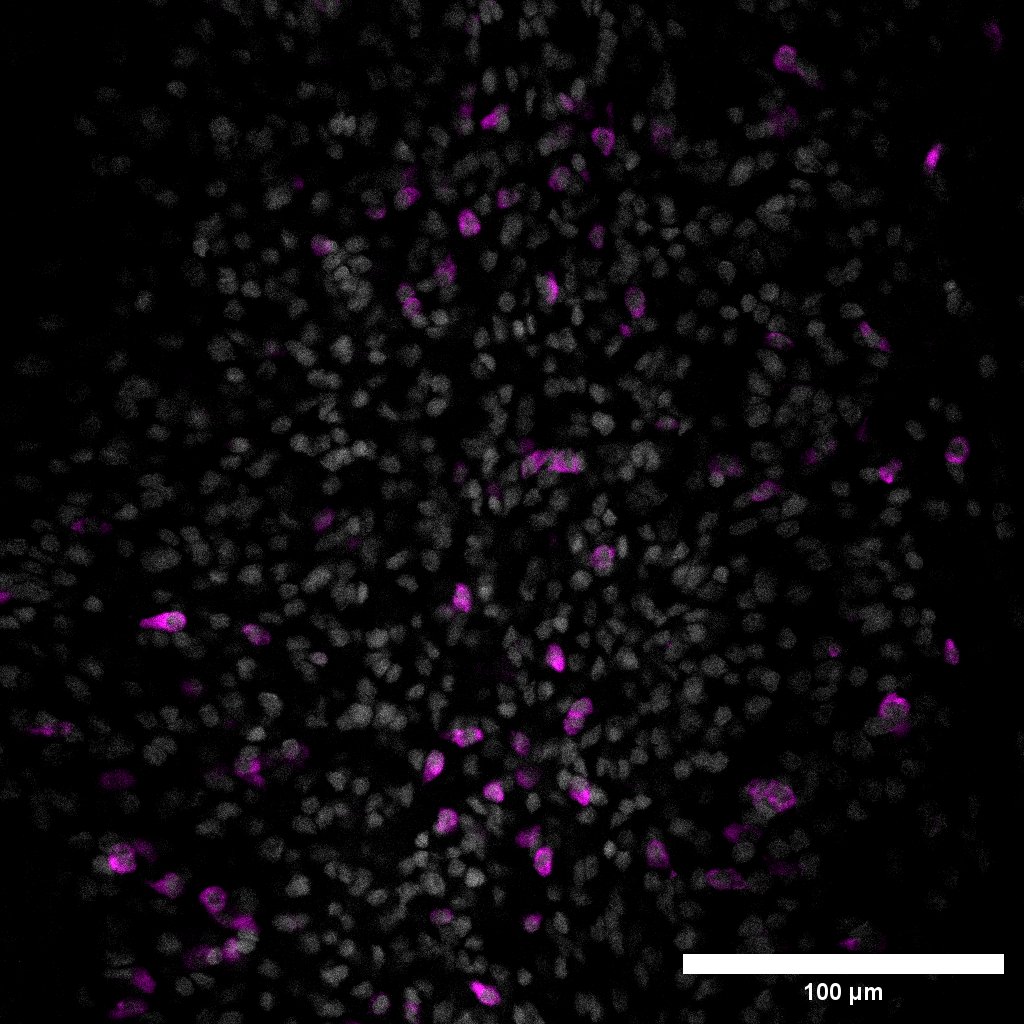

Supplement: Supplementary file 14 — Source data Fig. 7 [file 44318_2025_662_MOESM14_ESM.zip › Figure 7/7F/ID_6_Control_RNAi_Probe_prog2_rhod_DAPI_20x_z1.jpg]

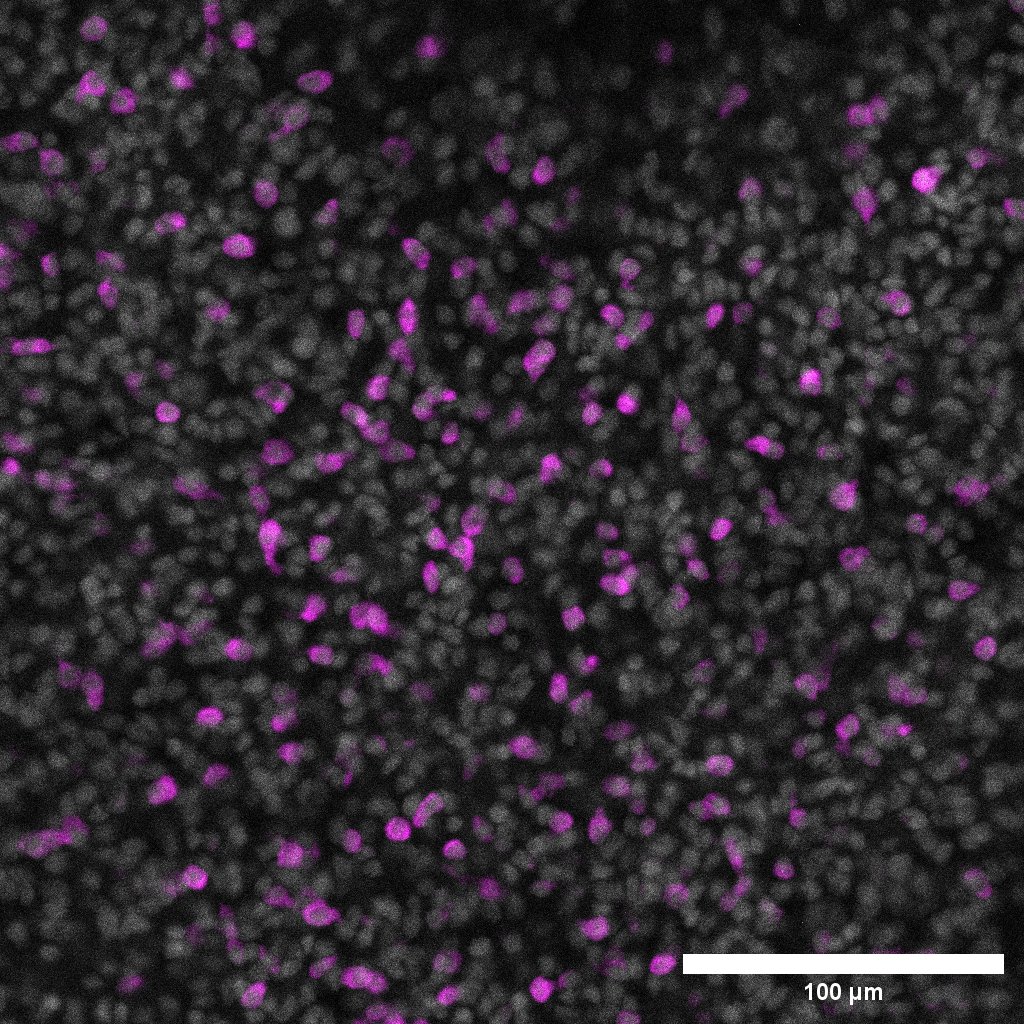

Supplement: Supplementary file 14 — Source data Fig. 7 [file 44318_2025_662_MOESM14_ESM.zip › Figure 7/7F/ID_6_Triple_RNAi_Probe_prog2_rhod_DAPI_20x_z1.jpg]

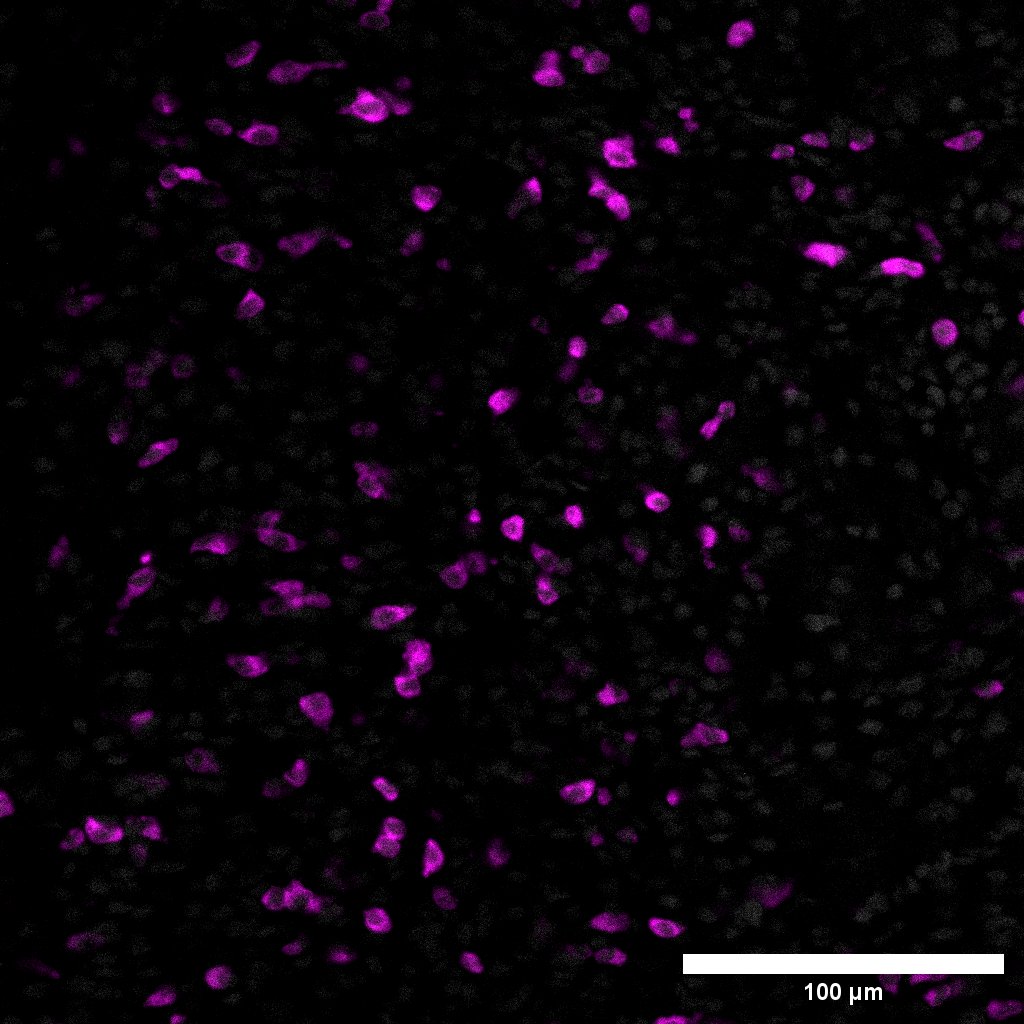

Supplement: Supplementary file 14 — Source data Fig. 7 [file 44318_2025_662_MOESM14_ESM.zip › Figure 7/7F/ID_7_Control_RNAi_Probe_prog2_rhod_DAPI_20x_z1.jpg]

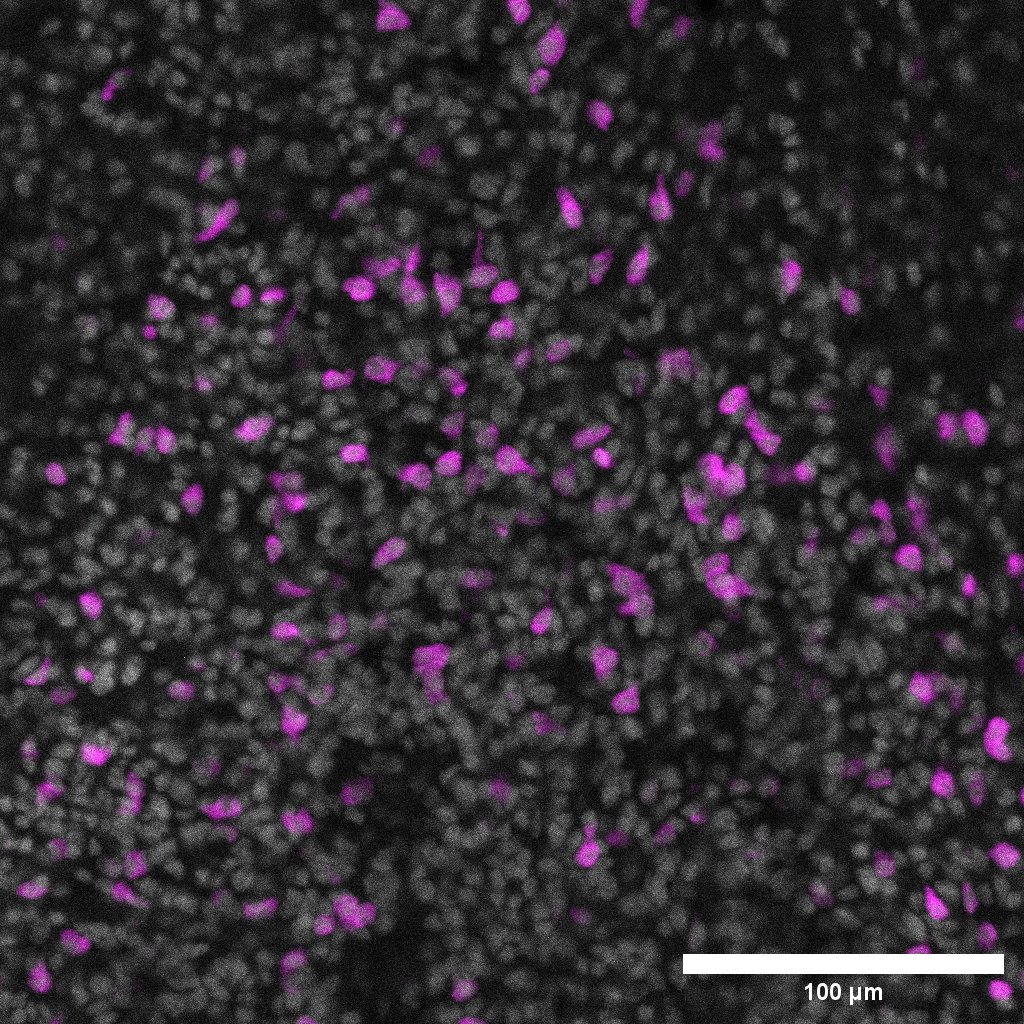

Supplement: Supplementary file 14 — Source data Fig. 7 [file 44318_2025_662_MOESM14_ESM.zip › Figure 7/7F/ID_7_Triple_RNAi_Probe_prog2_rhod_DAPI_20x_z1.jpg]

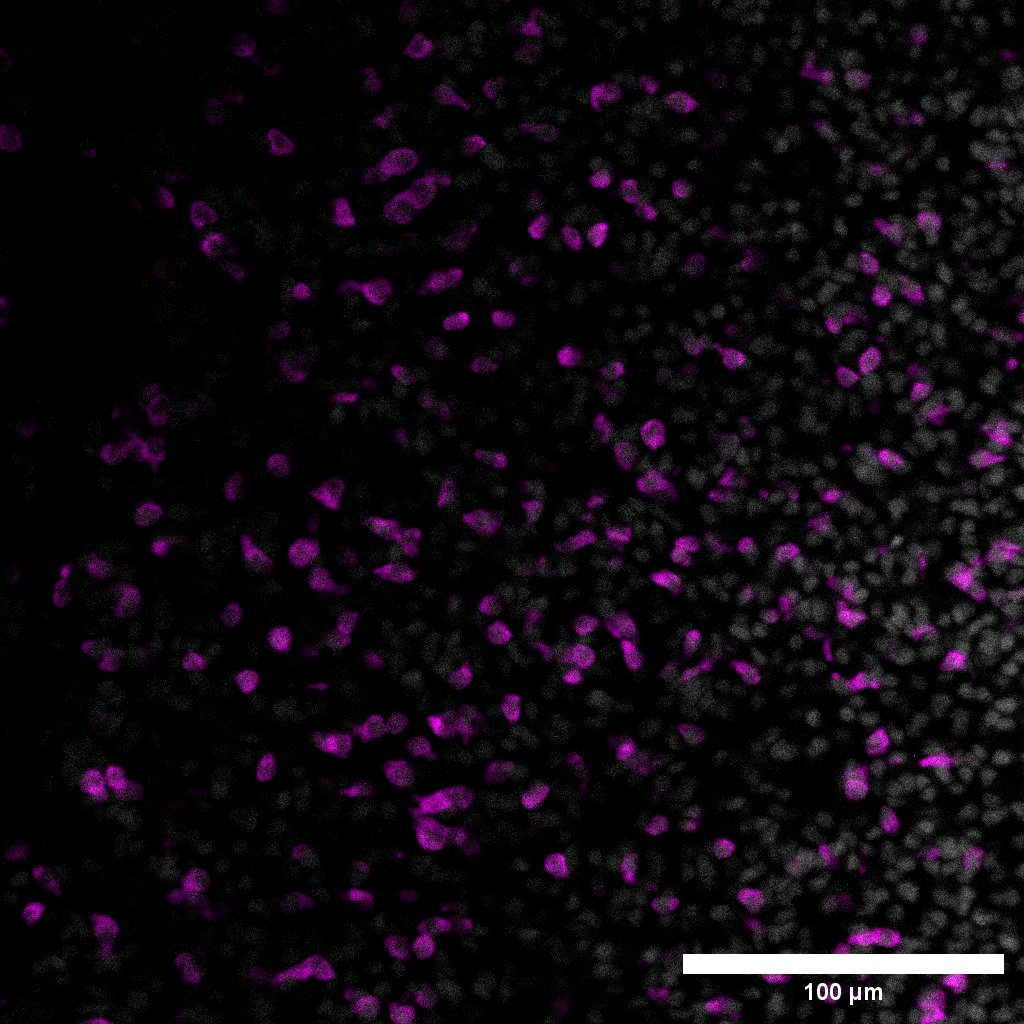

Supplement: Supplementary file 14 — Source data Fig. 7 [file 44318_2025_662_MOESM14_ESM.zip › Figure 7/7F/ID_8_Control_RNAi_Probe_prog2_rhod_DAPI_20x_z1.jpg]

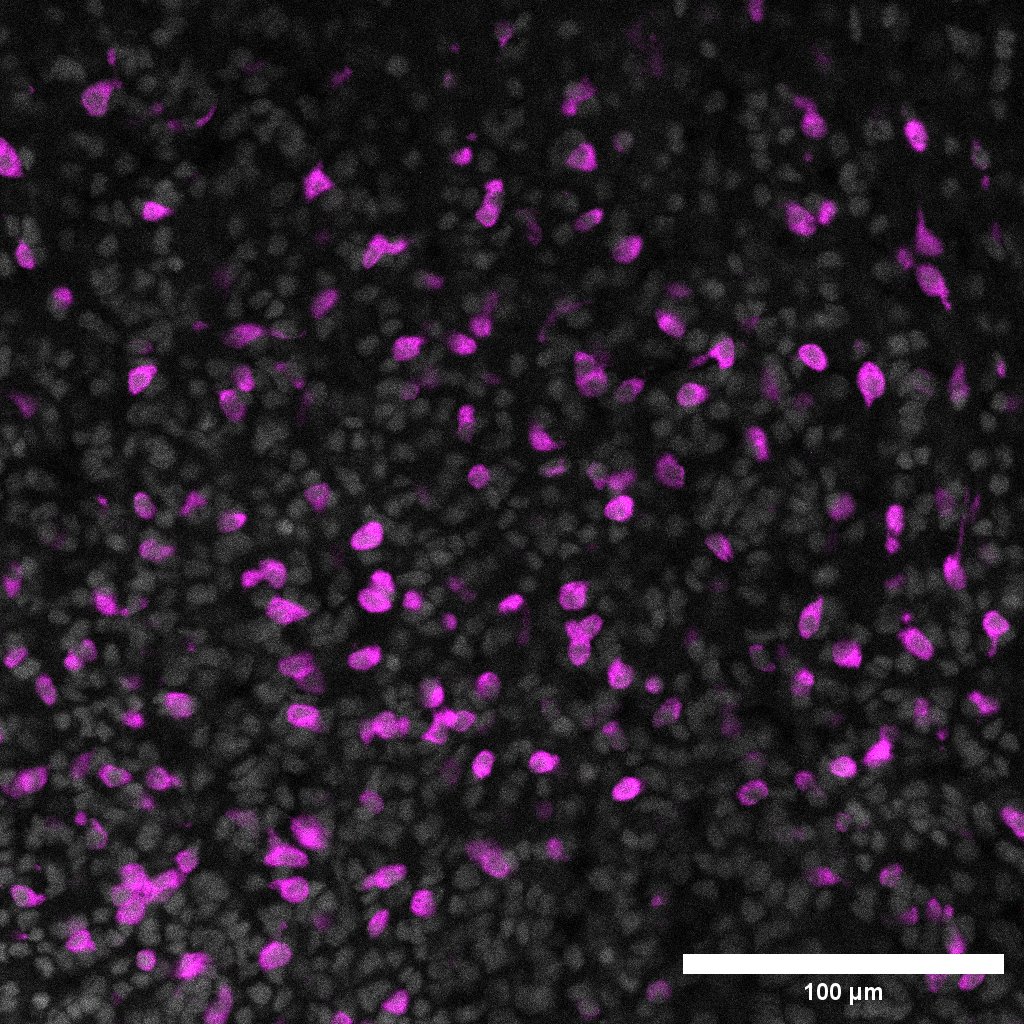

Supplement: Supplementary file 14 — Source data Fig. 7 [file 44318_2025_662_MOESM14_ESM.zip › Figure 7/7F/ID_8_Triple_RNAi_Probe_prog2_rhod_DAPI_20x_z1.jpg]

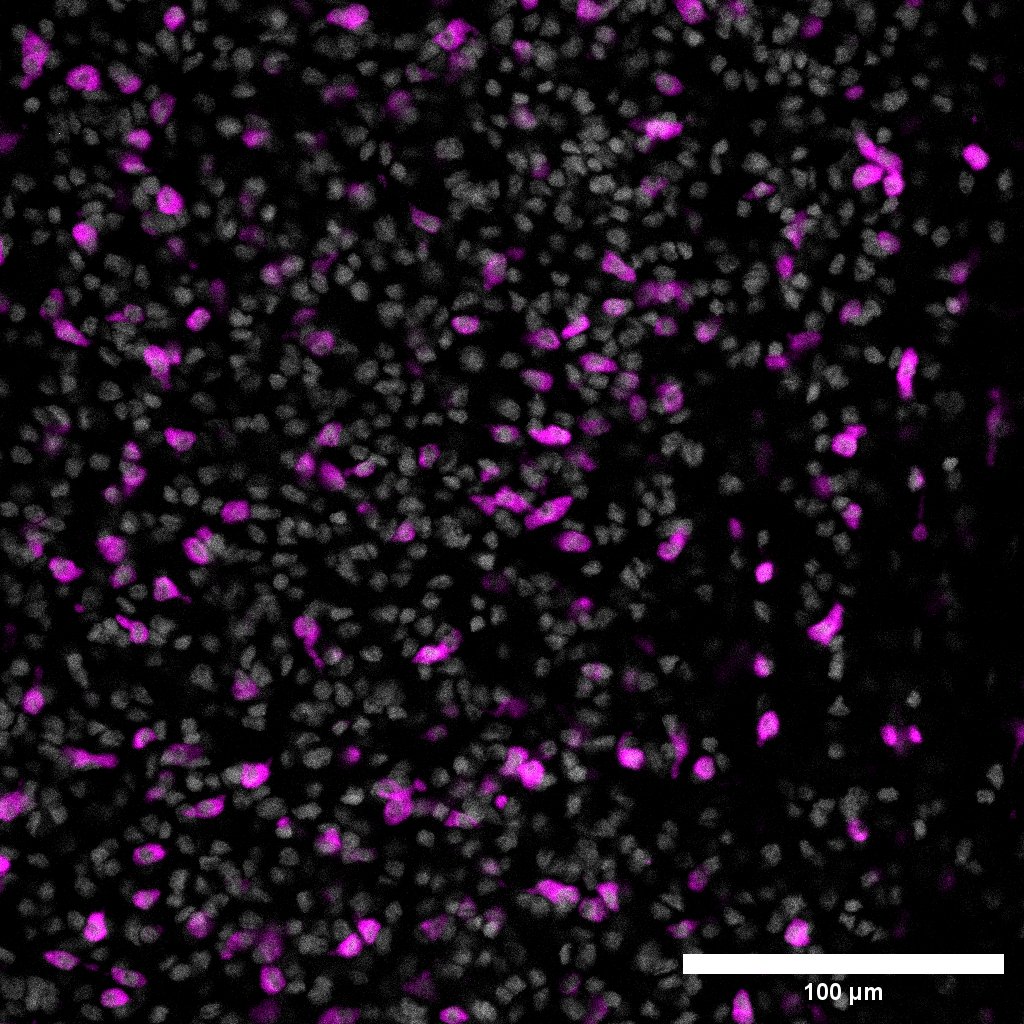

Supplement: Supplementary file 14 — Source data Fig. 7 [file 44318_2025_662_MOESM14_ESM.zip › Figure 7/7F/ID_9_Control_RNAi_Probe_prog2_rhod_DAPI_20x_z1.jpg]

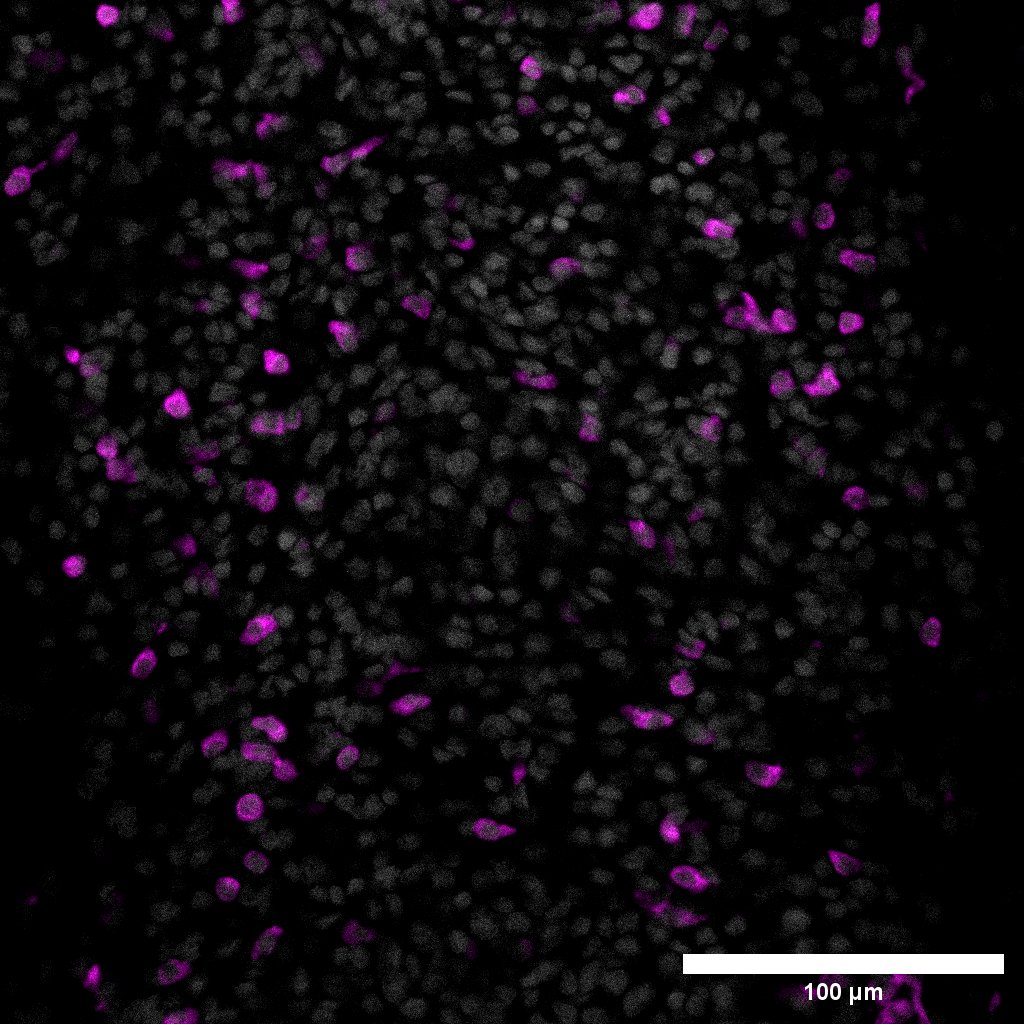

Supplement: Supplementary file 14 — Source data Fig. 7 [file 44318_2025_662_MOESM14_ESM.zip › Figure 7/7F/ID_9_Triple_RNAi_Probe_prog2_rhod_DAPI_20x_z1.jpg]

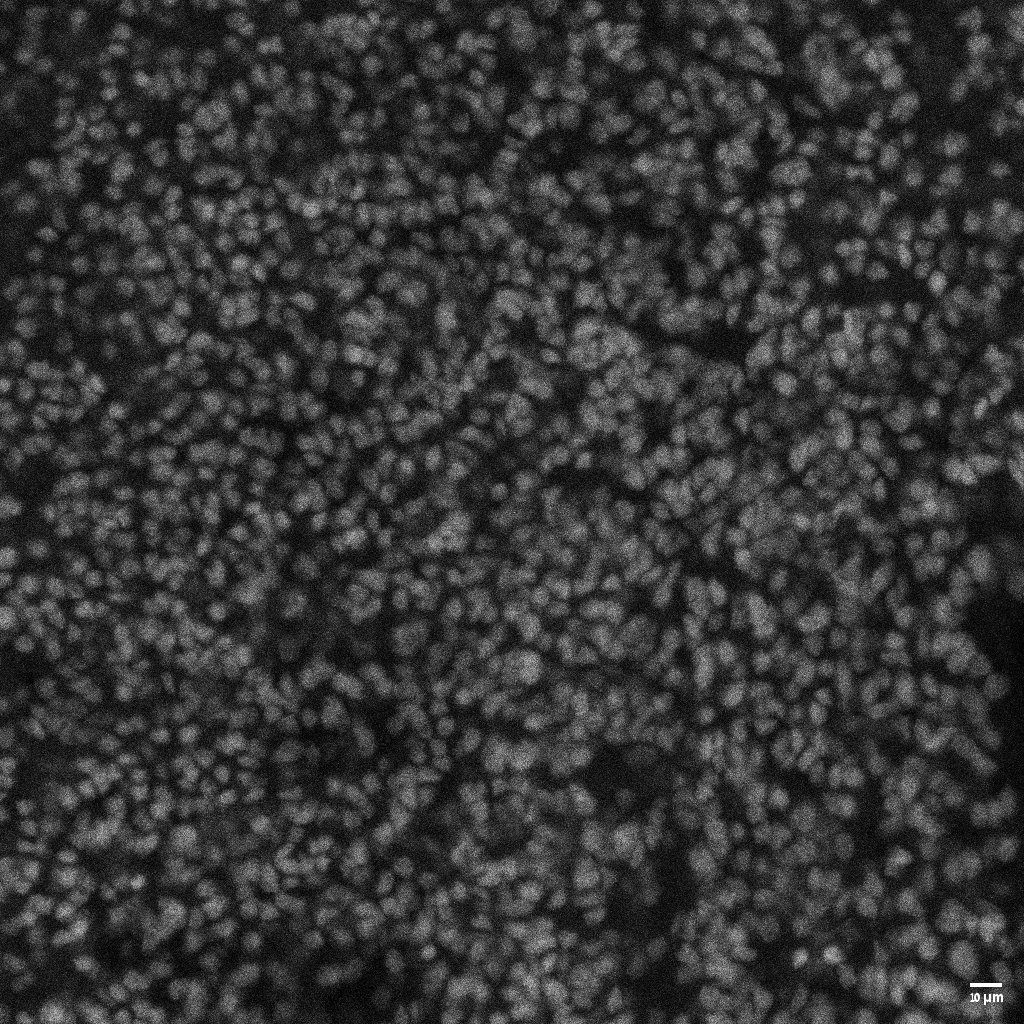

Supplement: Supplementary file 14 — Source data Fig. 7 [file 44318_2025_662_MOESM14_ESM.zip › Figure 7/7F/Main_figure_panel_Control_RNAi_Probe_prog2_rhod_DAPI_20x_z1_DAPI.jpg]

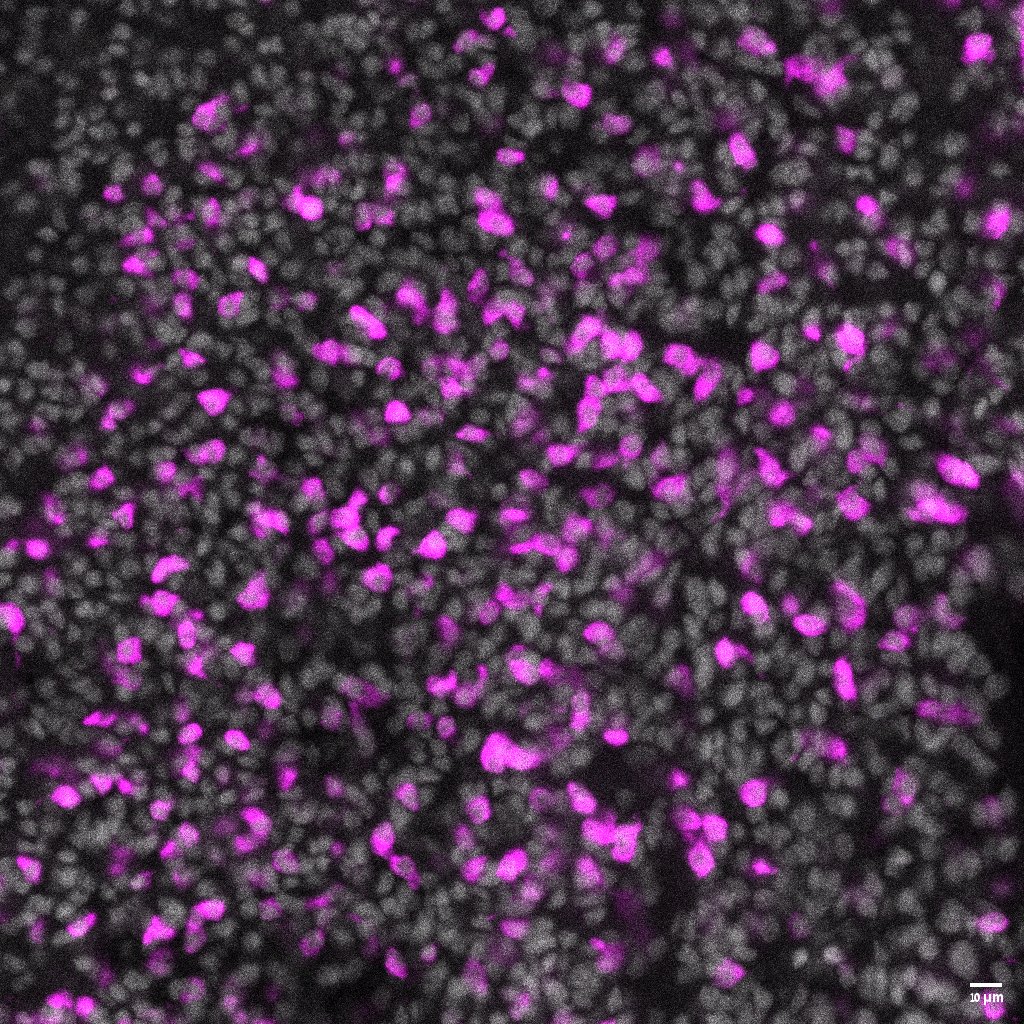

Supplement: Supplementary file 14 — Source data Fig. 7 [file 44318_2025_662_MOESM14_ESM.zip › Figure 7/7F/Main_figure_panel_Control_RNAi_Probe_prog2_rhod_DAPI_20x_z1_Merged.jpg]

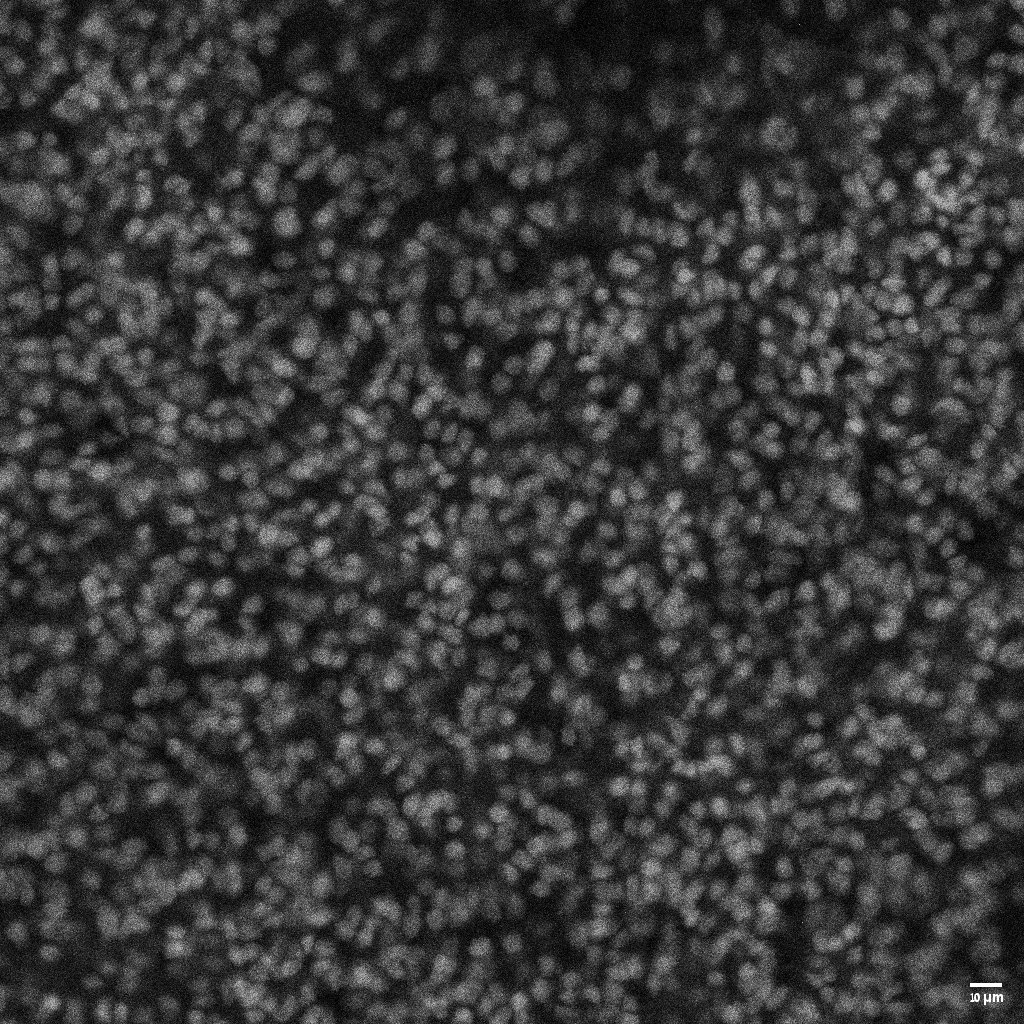

Supplement: Supplementary file 14 — Source data Fig. 7 [file 44318_2025_662_MOESM14_ESM.zip › Figure 7/7F/Main_figure_panel_Triple_RNAi_Probe_prog2_rhod_DAPI_20x_z1_DAPI.jpg]
