# Supplementary material for: Single-nucleotide m⁶A mapping uncovers redundant YTHDF function in planarian progenitor fate selection
Source: EMBO J. 2026 Jan 3;45(3):749–88. doi: 10.1038/s44318-025-00662-3 (PMC12864844; doi:10.1038/s44318-025-00662-3)
Supplement: Supplementary file 15 — Expanded View Figures [file 44318_2025_662_MOESM15_ESM.pdf]

# Expanded View Figures

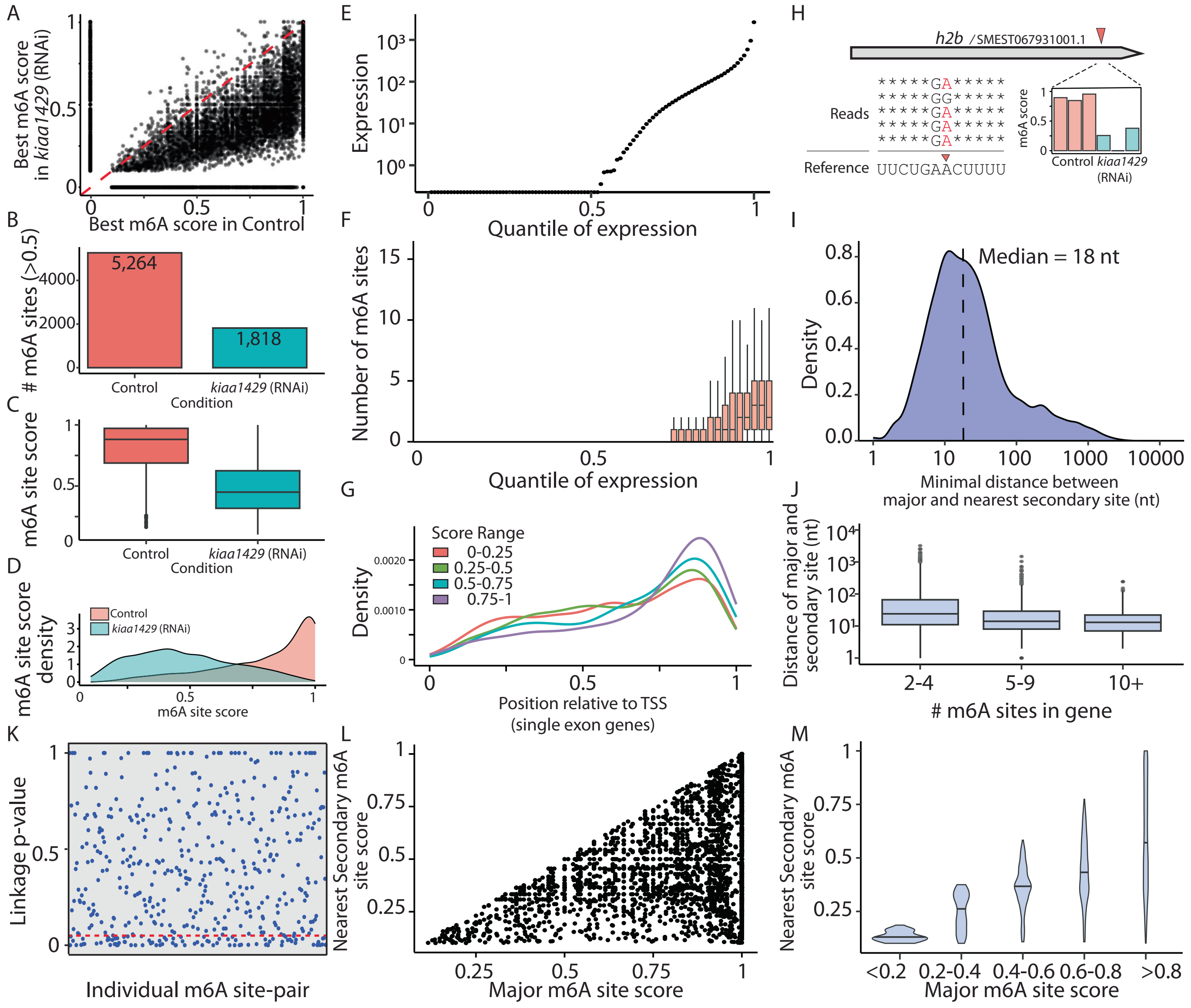


**Figure EV1. Properties of planarian m$^6$A sites.**

(**A**) The scatter plot displays m$^6$A site scores (black dots) in control samples, where m$^6$A levels are normal, and in *kiaa1429* (RNAi) samples, where m$^6$A levels are reduced. The red dashed line represents a slope of one. For each site, the score shown is the highest observed across any of the three replicates for each condition. (**B**) Number of m$^6$A sites with median score greater than the 0.5 in control and *kiaa1429* (RNAi) libraries demonstrates the reduction in overall methylation in *kiaa1429* (RNAi). (**C, D**) Shown is the distribution of median scores of m$^6$A sites in control and *kiaa1429* (RNAi) libraries as boxplot (**C**) and density (**D**) plots, for sites having coverage of at least 10 sequencing reads ($n = 2284$) in each of the three replicates used for the two conditions in this analysis. Boxplots show the median (center line), the IQR (box), and whiskers extending up to 1.5× the IQR range, with points beyond show outliers. (**E**) Shown is the median normalized expression (Love et al, 2014) of each gene in input libraries as a function of the quantile of expression. For example, the median expression of ~50% of the genes is 0. (**F**) The median number of m$^6$A sites is shown (median score in control libraries >0.1) as a function of quantile of gene expression, which was calculated based on the expression level of the gene in the input GLORI (i.e., untreated) libraries. (**G**) Metagene analysis of m$^6$A positions ($n = 2609$) of single gene exons shows a strong enrichment towards the 3′ end, indicating that 3′ end preference is not unlikely to be governed only by splicing specific factors. (**H**) Canonical histone transcripts are also targets for methylation. Shown is a canonical *h2b* (dd_2520) transcript with an m$^6$A site (red arrow) detected near the 3′ end. Read sequences that cover the m$^6$A site are shown (bottom left) with the methylated A highlighted in red. The m$^6$A score of the site in different replicates indicates that the m$^6$A installation is dependent on the MTC. (**I**) Shown is the minimal distance between the site having the highest m$^6$A score (i.e., major) with a secondary site (i.e., m$^6$A site having a lower score than the major site), in genes that have multiple m$^6$A sites. (**J**) Shown is a comparison of distance between the major and secondary site for genes with multiple m$^6$A sites. Boxplots show the median (center line), the IQR (box), and whiskers extending up to 1.5x the IQR range, with points beyond show outliers. Sizes of each category: 2–4 sites ($n = 3132$), 5–9 sites ($n = 1266$), and 10+ sites ($n = 379$). (**K**) The *P* value of linkage of m$^6$A installation is shown for m$^6$A site-pairs (Methods). For each site pair (blue dot) a *P* value was calculated (*y* axis). (**L, M**) Correlation of m$^6$A score between the major m$^6$A site and the nearest secondary site is shown.

 © The Author(s)

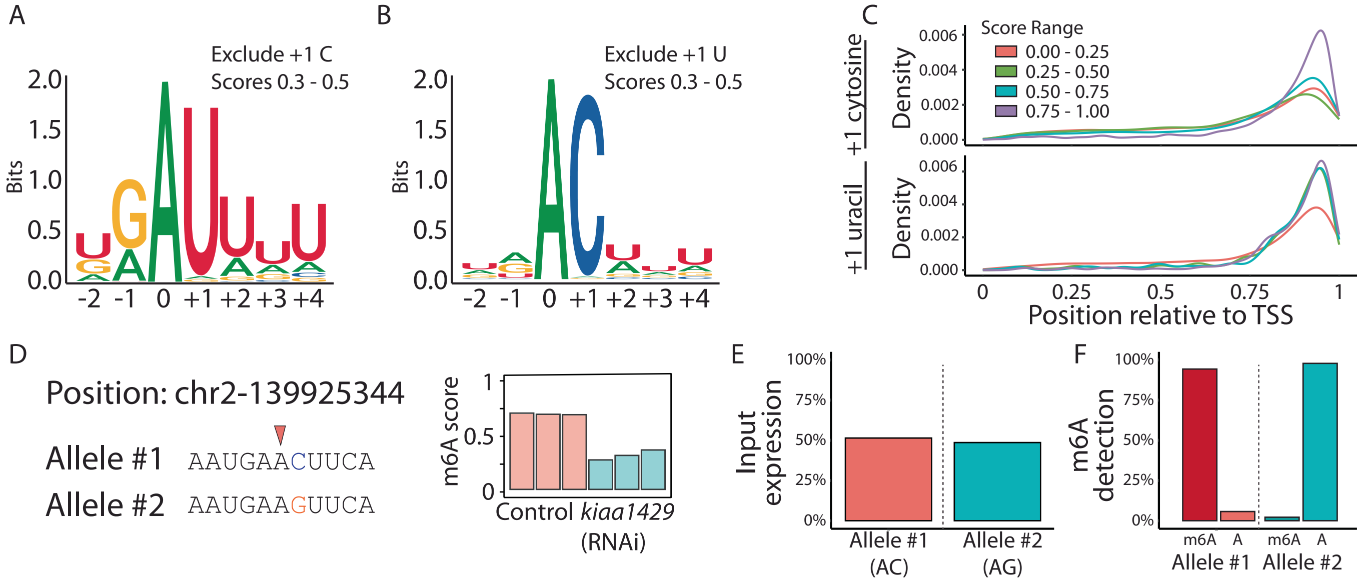


**Figure EV2. Sequence characteristics of m⁶A sites.**

(**A**) Shown is a sequence motif of m⁶A sites having low scores (0.3–0.5) and lacking a +1 C. The presence of a stretch of Us is observed as well as preference for a −1 G. (**B**) Shown is a sequence motif of m⁶A sites having low scores (0.3–0.5) and lacking a +1 U. There is no strong sequence preference for particular nucleotides other than a +1 C. (**C**) Metagene analysis of sites having either a +1 C (top) or +1 U (bottom) shows a similar distribution across the gene, indicating that the sites are installed similarly, despite the incompatibility of the +1 U with the known m⁶A installation consensus DRACH. (**D–F**) A rare example of an m⁶A site located at a sequence position that differs between alleles is shown. (**D**) The site (left, red arrow) is methylated in the allele with the AC sequence, whereas the other allele, which contains a +1 G, is refractory to m⁶A installation and remains unmethylated. The m⁶A site score (right) indicates methylation in the allele containing a +1 C. (**E**) The expression levels of both alleles were nearly identical, as determined by counting reads covering the sequence difference and distinguishing between them based on the nucleotide identity at the +1 position relative to the m⁶A site. (**F**) The fraction of methylated and unmethylated A is shown for both alleles, demonstrating that a +1 G is incompatible with m⁶A deposition.

© The Author(s)

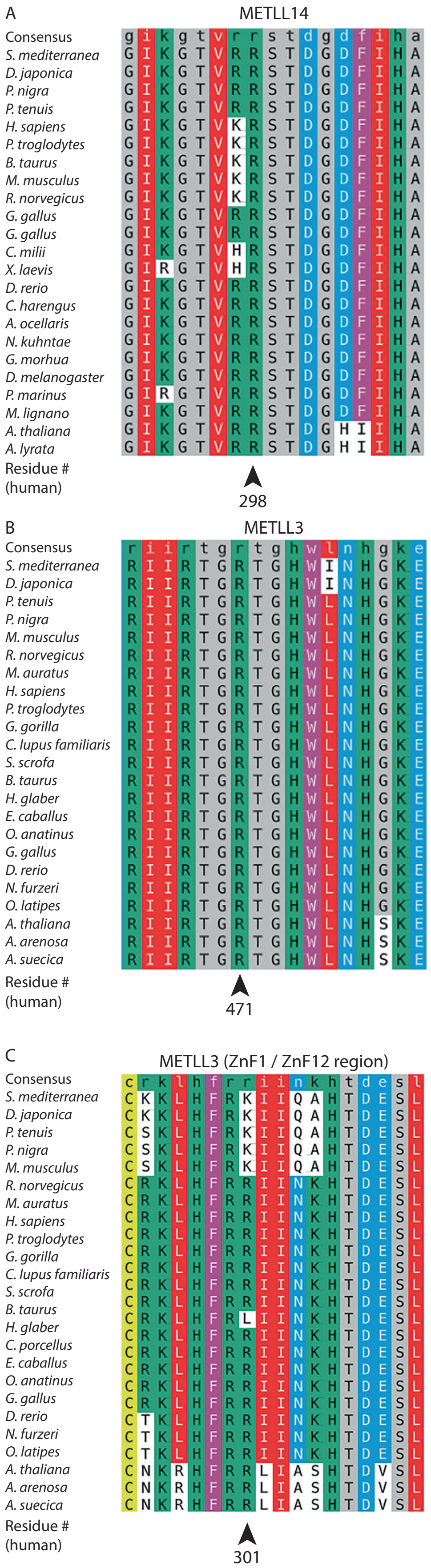
A
METLL14
Consensus
g i k g t v r r s t d g d f i h a
S. mediterranea
D. japonica
P. nigra
P. tenuis
H. sapiens
P. troglodytes
B. taurus
M. musculus
R. norvegicus
G. gallus
G. gallus
C. milii
X. laevis
D. rerio
C. harengus
A. ocellaris
N. kuhntae
G. morhua
D. melanogaster
P. marinus
M. lignano
A. thaliana
A. lyrata
Residue # (human)
298
B
METLL3
Consensus
r i i r t g r t g h w l n h g k e
S. mediterranea
D. japonica
P. tenuis
P. nigra
M. musculus
R. norvegicus
M. auratus
H. sapiens
P. troglodytes
G. gorilla
C. lupus familiaris
S. scrofa
B. taurus
H. glaber
E. caballus
O. anatinus
G. gallus
D. rerio
N. furzeri
O. latipes
A. thaliana
A. arenosa
A. suecica
Residue # (human)
471
C
METLL3 (ZnF1 / ZnF12 region)
Consensus
c r k l h f r r i i n k h t d e s l
S. mediterranea
D. japonica
P. tenuis
P. nigra
M. musculus
R. norvegicus
M. auratus
H. sapiens
P. troglodytes
G. gorilla
C. lupus familiaris
S. scrofa
B. taurus
H. glaber
C. porcellus
E. caballus
O. anatinus
G. gallus
D. rerio
N. furzeri
O. latipes
A. thaliana
A. arenosa
A. suecica
Residue # (human)
301

 © The Author(s)

**Figure EV3. Multiple alignment of key RNA recognition regions in the MTA.**

(**A–C**) Alignments ("Methods") include multiple planarian species, diverse metazoans, and representative *Arabidopsis* orthologs; residue numbering follows the human proteins. (**A**, **B**) The residues annotated, human cancer-related, change the RNA sequence binding preference of the human MTC from GGAC to GGAU (Zhang et al, 2024; Qi et al, 2024). (**C**) METTL3 ZnF1/2 region. The arrow marks the position homologous to human R301, a residue on the RNA-interaction surface of the MTC required for efficient MTC activity (Huang et al, 2019). In planarians, this position is substituted by lysine, preserving charge. We speculate that this change might have contributed to altered binding of the RNA residue.

© The Author(s)

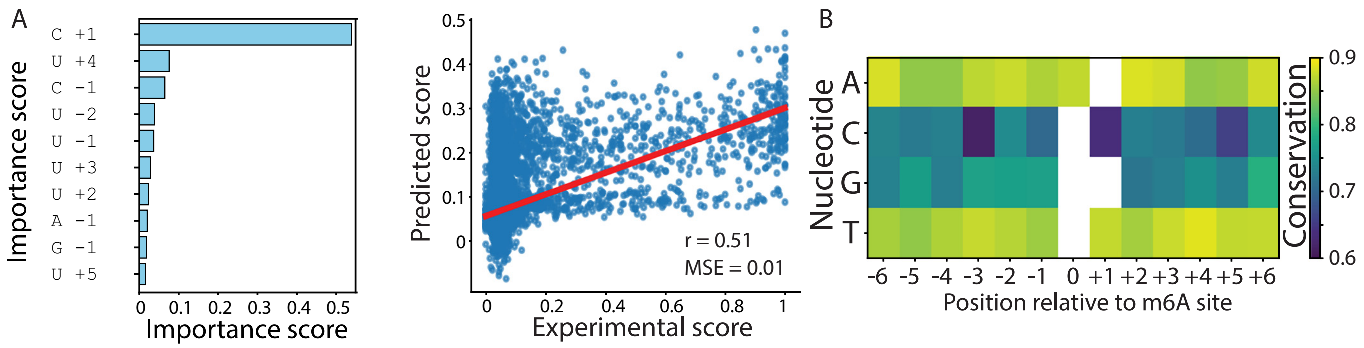


**Figure EV4. Sequence characteristics of m$^6$A sites.**

(**A**) The importance scores of sequence features near the m$^6$A site are shown. The strongest determinant of m$^6$A installation is the presence of a +1 C, while a + 4U is also associated with high methylation potential (left). A model incorporating only local sequence features performed better at identifying sequences that were poor methylation targets than at predicting methylation-compatible sequences (right), suggesting that factors beyond local sequence identity influence methylation levels. (**B**) Sequence conservation was calculated for individual m$^6$A sites between *Schmidtea mediterranea* and *Dugesia japonica*. The comparison revealed similar conservation patterns across sites when considering the nucleotide identity near the m$^6$A site. Overall, C and G were less conserved than A and T.

 © The Author(s)

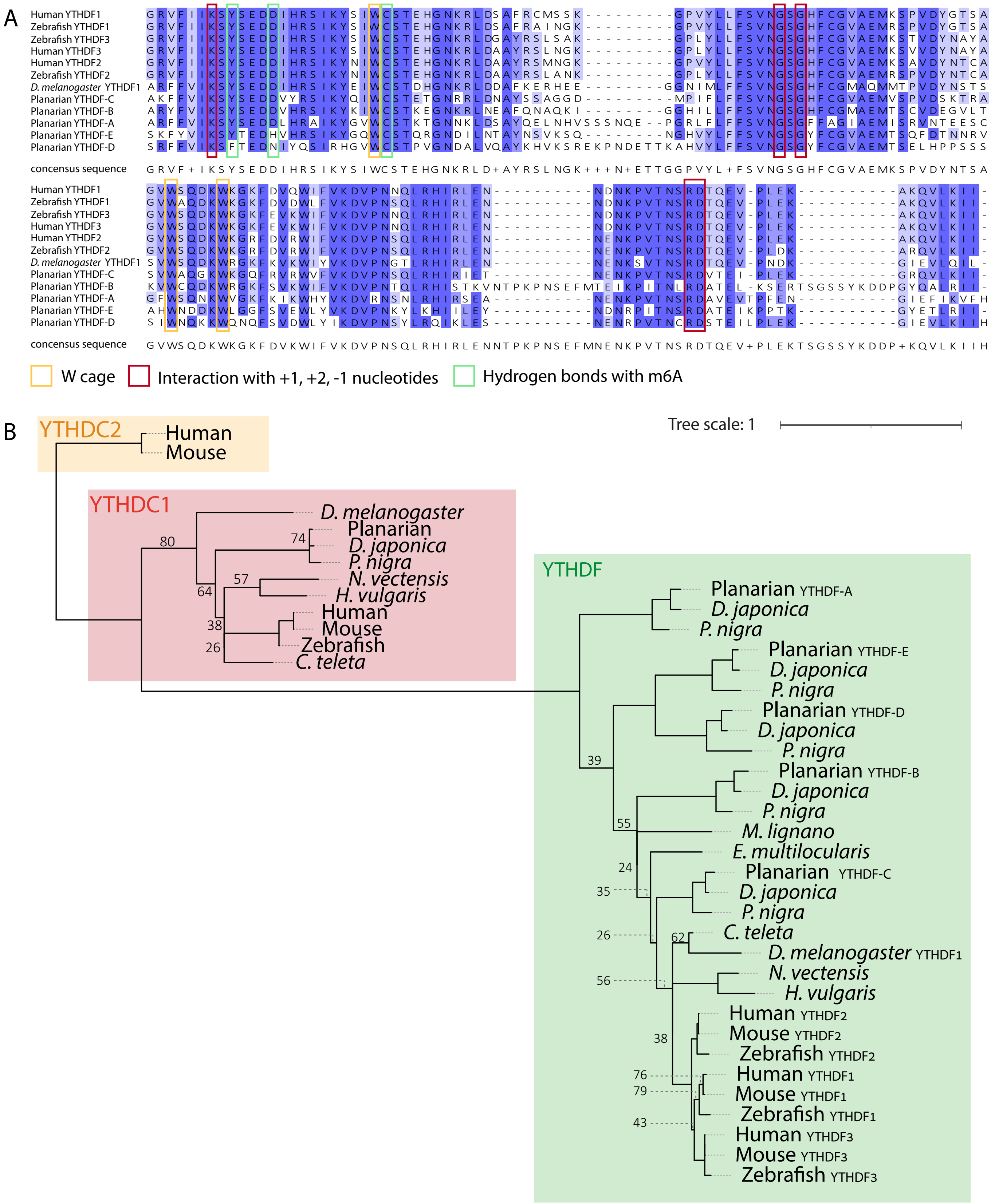
A
Human YTHDF1
Zebrafish YTHDF1
Zebrafish YTHDF3
Human YTHDF3
Human YTHDF2
Zebrafish YTHDF2
D. melanogaster YTHDF1
Planarian YTHDF-C
Planarian YTHDF-B
Planarian YTHDF-A
Planarian YTHDF-E
Planarian YTHDF-D
concensus sequence
G R V F + I K S Y S E D D I H R S I K Y S I W C S T E H G N K R L D + A Y R S L N G K + + + N + E T T G G P V Y L + F S V N G S G H F C G V A E M K S P V D Y N T S A
concensus sequence
G V W S Q D K W K G K F D V K W I F V K D V P N S Q L R H I R L E N N T P K P N S E F M N E N K P V T N S R D T Q E V + P L E K T S G S S Y K D D P + K Q V L K I I H
W cage
Interaction with +1, +2, -1 nucleotides
Hydrogen bonds with m6A
B
YTHDC2
Human
Mouse
Tree scale: 1
YTHDC1
D. melanogaster
Planarian
D. japonica
P. nigra
N. vectensis
H. vulgaris
Human
Mouse
Zebrafish
C. teleta
80
74
57
64
38
26
YTHDF
Planarian YTHDF-A
D. japonica
P. nigra
Planarian YTHDF-E
D. japonica
P. nigra
Planarian YTHDF-D
D. japonica
P. nigra
Planarian YTHDF-B
D. japonica
P. nigra
M. lignano
E. multilocularis
Planarian YTHDF-C
D. japonica
P. nigra
C. teleta
D. melanogaster YTHDF1
N. vectensis
H. vulgaris
Human YTHDF2
Mouse YTHDF2
Zebrafish YTHDF2
Human YTHDF1
Mouse YTHDF1
Zebrafish YTHDF1
Human YTHDF3
Mouse YTHDF3
Zebrafish YTHDF3
39
55
24
35
26
62
56
38
76
79
43

**Figure EV5. phylogenetic analysis of planarian YTHDF proteins.**

(**A**) Multiple sequence alignment of the conserved YTH-domain from YTHDF proteins in planarians, zebrafish, and *Drosophila*. Darker colors represent higher levels of amino acid conservation. (**B**) Phylogenetic tree illustrating the evolutionary relationships among various YTH-domain proteins across different species (Dataset EV3). Numbers indicate bootstrap values (1–100); bootstrap values > 85 were omitted for visual clarity. Planarian: *Schmidtea mediterranea*; *Spol*: *Schmidtea polychroa*; *D. japonica*: *Dugesia japonica*; *Pnig*: *polycelis nigra; C. teleta*: *Capitella teleta*; *M. lignano*: *Macrostomum lignano*; *Echinococcus multilocularis*; *H. vulgaris*: *Hydra vulgaris*; *N. vectensis*: *Nematostella vectensis*; *D. melanogaster*: *Drosophila melanogaster*.


© The Author(s)

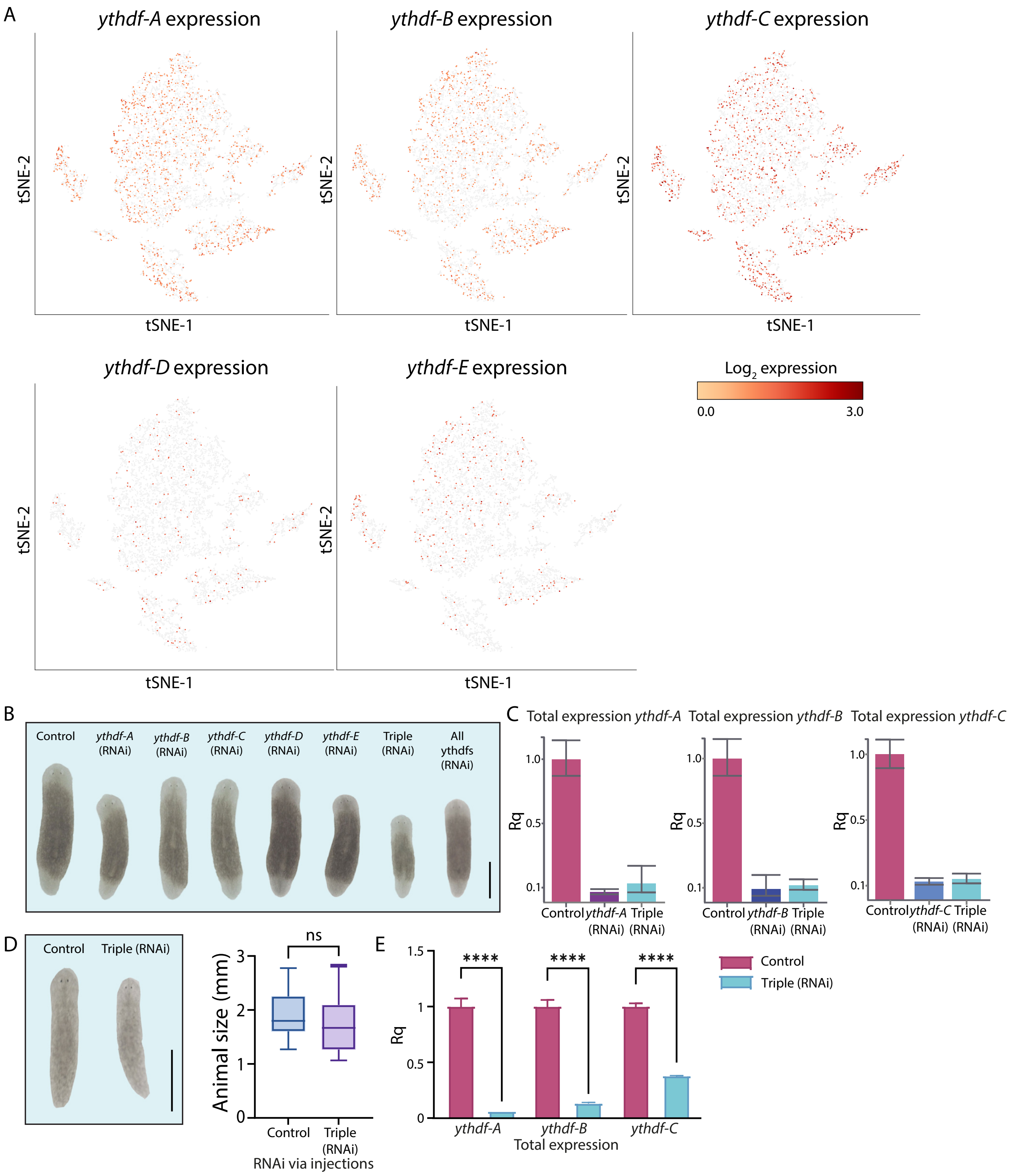
A
ythdf-A expression
ythdf-B expression
ythdf-C expression
ythdf-D expression
ythdf-E expression
tSNE-1
tSNE-2
Log2 expression
0.0
3.0
B
Control
ythdf-A (RNAi)
ythdf-B (RNAi)
ythdf-C (RNAi)
ythdf-D (RNAi)
ythdf-E (RNAi)
Triple (RNAi)
All ythdfs (RNAi)
C
Total expression ythdf-A
Total expression ythdf-B
Total expression ythdf-C
Rq
Control ythdf-A (RNAi) Triple (RNAi)
Control ythdf-B (RNAi) Triple (RNAi)
Control ythdf-C (RNAi) Triple (RNAi)
D
Control
Triple (RNAi)
ns
Animal size (mm)
RNAi via injections
E
****
Rq
ythdf-A
ythdf-B
ythdf-C
Total expression
Control
Triple (RNAi)

**Figure EV6. *ythdf* expression is not required for planarian regeneration.**

(**A**) *ythdf*-encoding genes exhibit broad expression across planarian cells based on scRNAseq data. Shown here is the expression of the five *ythdf* genes, extracted from a published dataset of G0 planarian cells (King et al, 2024). (**B**) *ythdf* (RNAi) animals successfully regenerated heads and tails following amputation (15/15). Scale bar = 1 mm. (**C**) qPCR analysis showing significant downregulation of *ythdf-A*, *ythdf-B*, and *ythdf-C* gene expression following RNAi in both single and triple inhibition conditions. Error bars indicate the 95% confidence interval. Data include two technical replicates and three biological replicates. *P* value for each condition compared to its control was as high as 0.0078. (**D**) Comparison of animal sizes following three weeks of co-*ythdf* dsRNA injections compared to control animals. Shown are representative images (left) and quantification. There was no significant difference in the animal sizes (Student's two-tailed *t* test; *P* value = 0.511; group size *n* = 18; Boxes represent the IQR, whiskers represent min to max, and central band represents the median) ("Methods"). Scale = 1 mm. (**E**) Shown is qPCR validation that the co-*ythdf* (RNAi) were efficiently silenced by the microinjections. **** Student's *t* test *P* value < 0.0001. Data includes three biological replicates, each measured by two technical replicates, error bars represent SD and the center is the mean.

 © The Author(s)

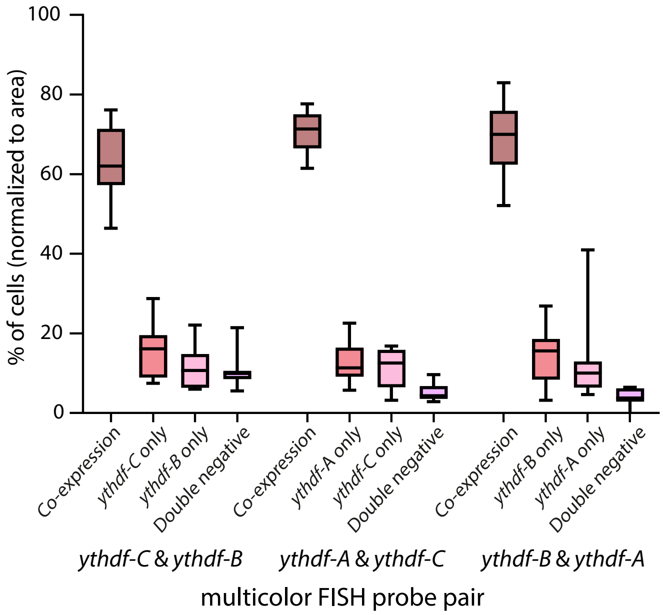


**Figure EV7. Quantification of *ythdf* expression in the planarian epidermis.**

Boxplots representing the percentage of epidermal cells expressing *ythdf* genes. Boxes represent the IQR, whiskers represent min to max, and central band represents the median. Multicolor fluorescence in situ hybridization (FISH) analysis was used to categorize cells into four groups: double-negative cells, single-positive cells (expressing only one of the two analyzed *ythdf*), and double-positive cells (co-expressing the two analyzed *ythdf* genes). Data were collected from 10 distinct epidermal regions from the top of the pharynx to the brain and normalized to the area of each region ("Methods").

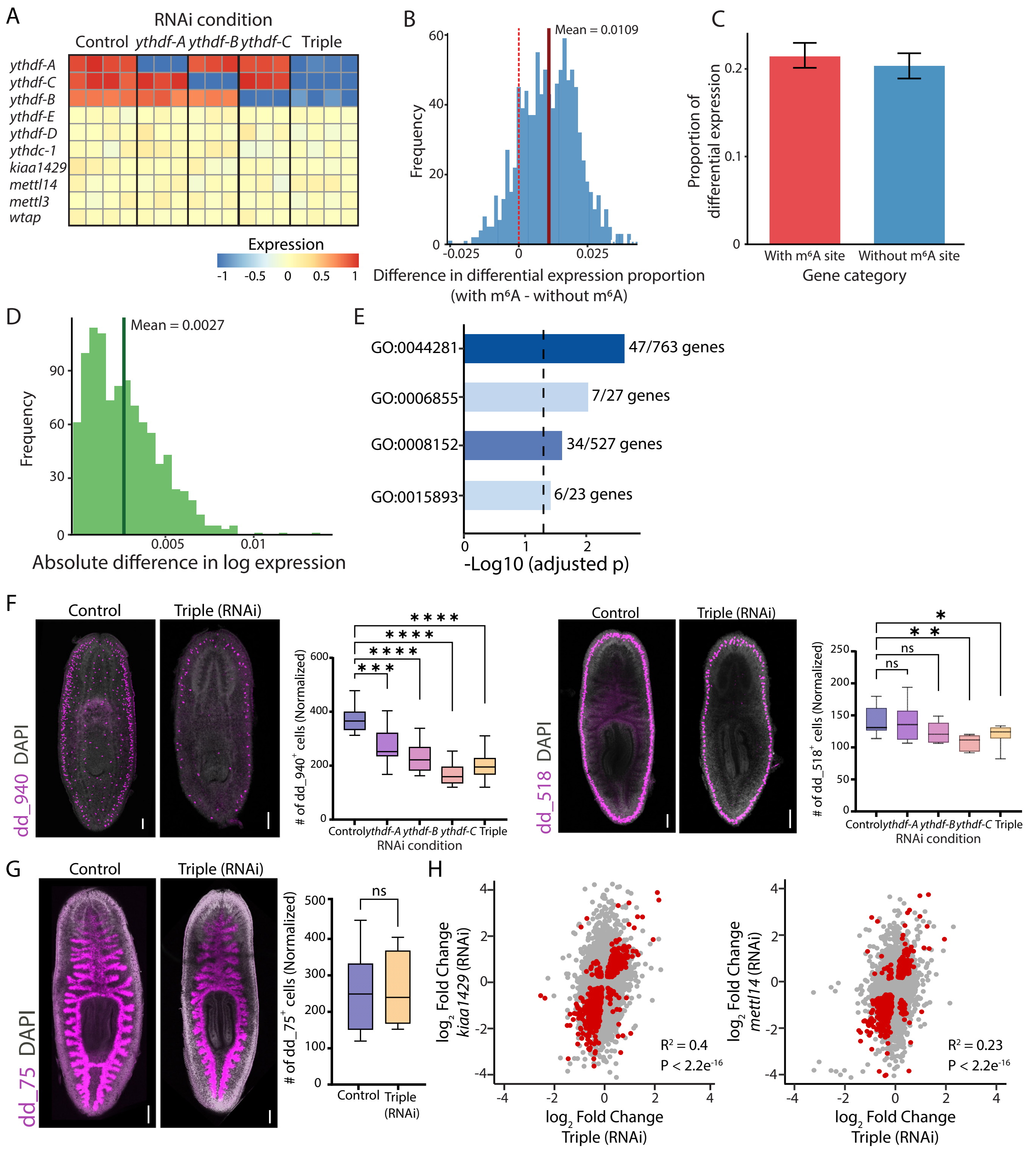
A
RNAi condition
Control ythdf-A ythdf-B ythdf-C Triple
ythdf-A
ythdf-C
ythdf-B
ythdf-E
ythdf-D
ythdc-1
kiaa1429
mettl14
mettl3
wtap
Expression
-1 -0.5 0 0.5 1
B
Mean = 0.0109
Frequency
Difference in differential expression proportion
(with m6A - without m6A)
C
Proportion of differential expression
With m6A site
Without m6A site
Gene category
D
Mean = 0.0027
Frequency
Absolute difference in log expression
E
GO:0044281
47/763 genes
GO:0006855
7/27 genes
GO:0008152
34/527 genes
GO:0015893
6/23 genes
-Log10 (adjusted p)
F
Control
Triple (RNAi)
dd_940 DAPI
# of dd_940+ cells (Normalized)
Control ythdf-A ythdf-B ythdf-C Triple
RNAi condition
dd_518 DAPI
# of dd_518+ cells (Normalized)
ns
G
dd_75 DAPI
# of dd_75+ cells (Normalized)
Control
Triple (RNAi)
H
log2 Fold Change kiaa1429 (RNAi)
R2 = 0.4
P < 2.2e-16
log2 Fold Change mettl14 (RNAi)
R2 = 0.23
P < 2.2e-16
log2 Fold Change Triple (RNAi)

 © The Author(s)

**Figure EV8. Inhibition of *ythdf* genes resulted in a reduction in parenchymal cells.**

(**A**) Heatmap of YTH-domain protein encoding genes and MTC encoding gene expression following inhibition of *ythdf* genes. Inhibition of *ythdf* genes did not affect the expression of other components of the m$^6$A pathway. Displayed are z-scores ranging from −1 to 1 (FDR < $1 \times 10^{-5}$). Each column represents a biological replicate.
(**B**) Distribution of the difference in the proportion of differentially expressed genes between expression-matched sets with versus without mapped m$^6$A sites across 1000 bootstrap iterations. The mean difference was calculated (red thick line). The red dashed line represents no difference. Empirical two-sided *P* value = 0.302, indicating no enrichment in the number of differentially expressed genes among m6A-containing genes after co-*ythdf* suppression. (**C**) Mean fraction of differentially expressed genes (adjusted $P < 0.05$) for genes having m$^6$A versus genes without m$^6$A sites across the same bootstraps; error bars show 95% confidence intervals. (**D**) Distribution of the absolute difference in baseline log expression between the matched gene sets (mean = 0.0027), confirming close expression matching. Lower value indicates a greater similarity in gene expression between the groups. (**E**) Shown are significantly enriched biological processes (gene ontology categories) in differentially expressed genes following the co-suppression of *ythdf*s ("Methods"). (**F**) FISH analysis showing changes in different parenchymal cell types (Fincher et al, 2018) following inhibition of *ythdf* genes using the markers dd_940 (top right) and dd_518 (bottom left). Cell counts were normalized to animal size ("Methods") in *ythdf-A* (RNAi), *ythdf-B* (RNAi), *ythdf-C* (RNAi), and triple (RNAi) animals and compared to control animals. Statistical significance was assessed using one-way ANOVA followed by Dunnett's correction; *P* values: dd_940 *ythdf-A* (RNAi) $P = 0.0007$, *ythdf-B* (RNAi) $P = 5.3 \times 10^{-6}$, *ythdf-C* (RNAi) $P = 3.0 \times 10^{-9}$, triple (RNAi) $P = 1.85 \times 10^{-8}$; dd_518 *ythdf-C* (RNAi) $P = 0.0039$, triple (RNAi) $P = 0.0451$; group size $n > 8$, Boxes represent the IQR, whiskers represent min to max, and central band represents the median). Scale bar = 100 µm. (**G**) FISH analysis detecting intestinal cells expressing dd_75, in triple (RNAi) and control animals. Cell count of dd_75$^+$ cells in the intestinal region spanning the pharynx was normalized to animal size, revealing no reduction in intestine cell number following *ythdf*s inhibition (Student's two-tailed *t* test, $P > 0.05$, group size $n > 8$). Scale bar = 100 µm. (**H**) Correlation of gene expression changes between *kiaa1429* (RNAi) and triple (RNAi) compared to their controls (left), and *mettl14* (RNAi) and Triple (RNAi) compared to their controls (right). Each colored dot represents a gene, red and black represents significant (FDR < 0.05 for both conditions) and nonsignificant change in gene expression compared to controls, respectively.

© The Author(s)

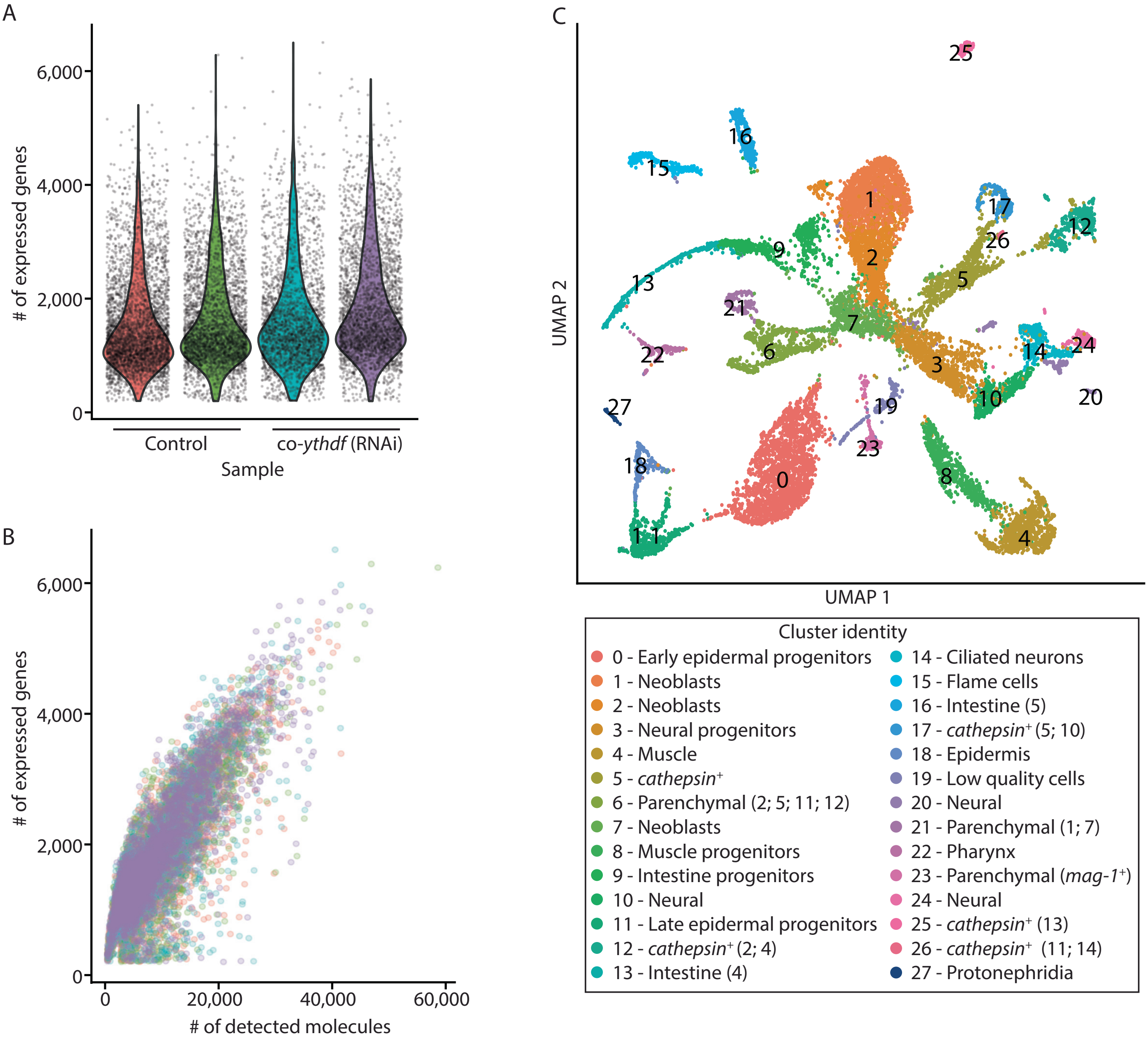


**Figure EV9. scRNAseq quality and clustering after co-*ythdf* suppression.**

(**A**) Unique transcripts detected per cell shown in two biological replicates of control and co-*ythdf* (RNAi) samples, showing comparable library complexity. Dots represent cells. (**B**) Genes detected as a function of detected molecules (UMIs) per cell, indicating similar saturation across conditions Dots represent cells. (**C**) UMAP of cells from the four sequenced samples, with clusters (0–27) annotated based on expression of gene markers ("Methods"). Numbers in parenthesis indicate specific sub-cluster identity based on the Planarian Cell Atlas annotation (Fincher et al, 2018).


© The Author(s)

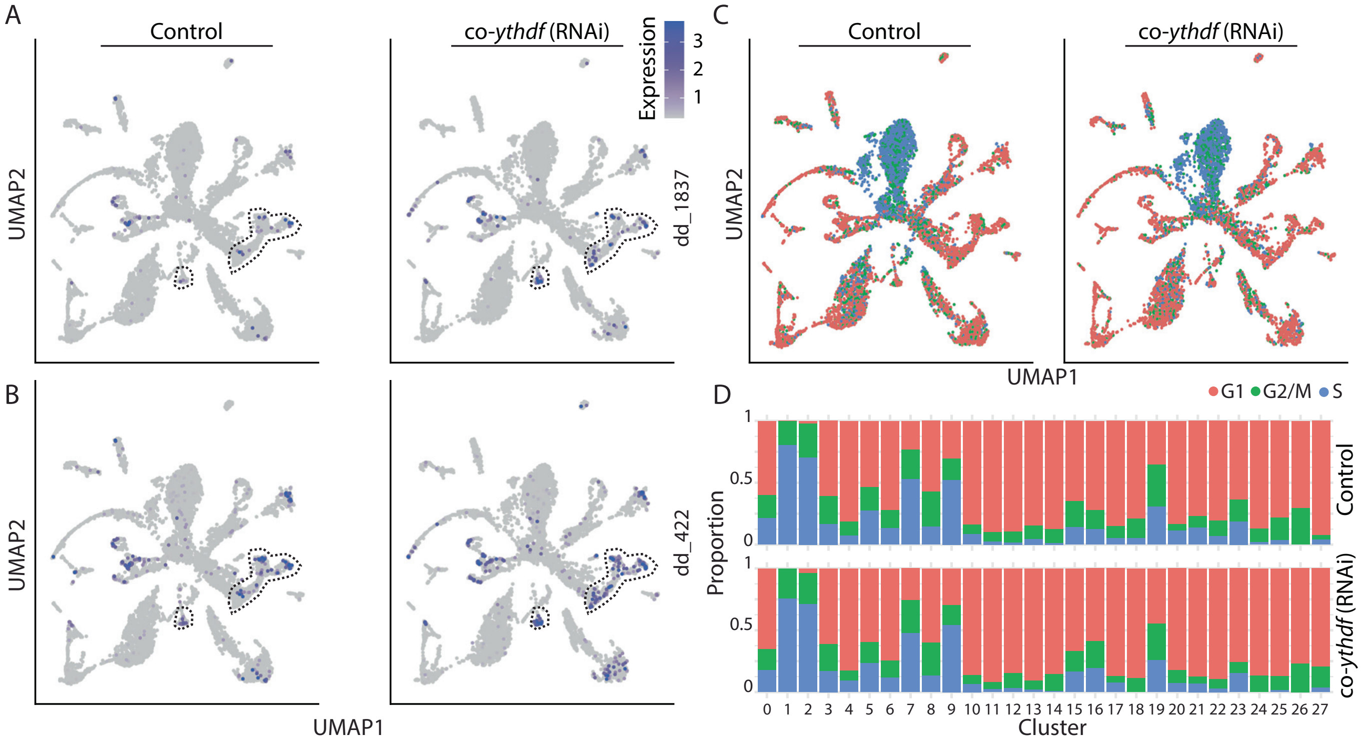


**Figure EV10. scRNAseq gene expression and cell cycle analysis.**

(**A**, **B**) UMAP plots showing the expression of dd_1837 (**A**) and dd_422 (**B**) in Control and co-*ythdf* (RNAi) conditions. Dashed outlines mark clusters with strongest induction. Gray to purple, low to high log-normalized expression, respectively. (**C**) UMAP plots colored by inferred cell-cycle phase (G1, red; G2/M, green; and S, blue) for Control and co-*ythdf* (RNAi) conditions ("Methods"). (**D**) Proportion of cells in G1, G2/M, and S phase cells per cluster in Control (top) and co-*ythdf* (RNAi) (bottom), showing broadly similar cell-cycle composition across conditions.

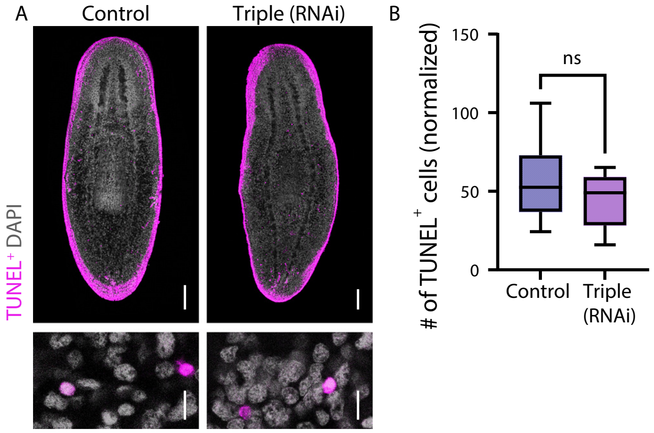


**Figure EV11. Analysis of cell death by whole-mount TUNEL.**

(**A**) TUNEL analysis of apoptotic cells in control and co-*ythdf* (RNAi) animals following six dsRNA injections ("Methods"). scale bar = 100 μm. (**B**) Counts of TUNEL$^+$ cells across the entire body were normalized to animal size, revealing no significant difference in the number of apoptotic cells following co-*ythdf* inhibition (right, Student's two-tailed $t$ test $P > 0.05$; group size $n > 10$).

 © The Author(s)
